# Identification of New Differentially Methylated Genes That Have Potential Functional Consequences in Prostate Cancer

**DOI:** 10.1371/journal.pone.0048455

**Published:** 2012-10-31

**Authors:** Jin W. Kim, Seong-Tae Kim, Aubrey R. Turner, Tracey Young, Shelly Smith, Wennuan Liu, Johan Lindberg, Lars Egevad, Henrik Gronberg, William B. Isaacs, Jianfeng Xu

**Affiliations:** 1 Center for Cancer Genomics, Wake Forest University, Winston-Salem, North Carolina, United States of America; 2 Center for Genomics and Personalized Medicine Research, Wake Forest University, Winston-Salem, North Carolina, United States of America; 3 Department of Cancer Biology, Wake Forest University, Winston-Salem, North Carolina, United States of America; 4 Department of Medical Epidemiology and Biostatistics, Karolinska Institutet, Stockholm, Sweden; 5 Department of Oncology-Pathology, Karolinska Institutet, Stockholm, Sweden; 6 Department of Urology, Johns Hopkins Medical Institutions, Baltimore, Maryland, United States of America; National Cancer Institute, National Institutes of Health, United States of America

## Abstract

Many differentially methylated genes have been identified in prostate cancer (PCa), primarily using candidate gene-based assays. Recently, several global DNA methylation profiles have been reported in PCa, however, each of these has weaknesses in terms of ability to observe global DNA methylation alterations in PCa. We hypothesize that there remains unidentified aberrant DNA methylation in PCa, which may be identified using higher resolution assay methods. We used the newly developed Illumina HumanMethylation450 BeadChip in PCa (*n* = 19) and adjacent normal tissues (*n* = 4) and combined these with gene expression data for identifying new DNA methylation that may have functional consequences in PCa development and progression. We also confirmed our methylation results in an independent data set. Two aberrant DNA methylation genes were validated among an additional 56 PCa samples and 55 adjacent normal tissues. A total 28,735 CpG sites showed significant differences in DNA methylation (FDR adjusted *P*<0.05), defined as a mean methylation difference of at least 20% between PCa and normal samples. Furthermore, a total of 122 genes had more than one differentially methylated CpG site in their promoter region and a gene expression pattern that was inverse to the direction of change in DNA methylation (e.g. decreased expression with increased methylation, and vice-versa). Aberrant DNA methylation of two genes, *AOX1* and *SPON2,* were confirmed via bisulfate sequencing, with most of the respective CpG sites showing significant differences between tumor samples and normal tissues. The *AOX1* promoter region showed hypermethylation in 92.6% of 54 tested PCa samples in contrast to only three out of 53 tested normal tissues. This study used a new BeadChip combined with gene expression data in PCa to identify novel differentially methylated CpG sites located within genes. The newly identified differentially methylated genes may be used as biomarkers for PCa diagnosis.

## Introduction

DNA methylation is a covalent addition of a methyl group at the 5 position carbon of a cytosine base and this epigenetic change is almost exclusively found in cytosine guanine dinucleotides (CpG) in differentiated human cells [1]. CpG sites are mainly found in repetitive sequences or CpG islands (CGI), which are CpG-rich regions that can be found throughout the human genome. In normal human brain tissue, less than 3% of CGIs located in promoter regions were found to be methylated, in contrast up to 34% of CGI located in intragenic regions were methylated [2]. However, CGI methylation in promoter regions is well documented in many studies due to association with gene silencing [3]–[5].

Aberrant DNA methylation between cancer samples and normal tissues has been widely observed in various cancers including PCa [6]–[9]. More than 67 differentially methylated genes were identified in PCa, primarily based on gene-specific assay methods [10]. Recent developments in state-of-the-art techniques such as microarray technology and next-generation sequencing have ushered in a new epigenomic era in DNA methylation studies [11]–[14]. Multiple studies have reported aberrant patterns of genome-wide DNA methylation in PCa tissue samples, using Agilent CpG island microarrays, Illumina HumanMethylation27 (HM27) BeadChips or next-generation sequencing methods [15]–[20]. Four papers have reported DNA methylation profiles using the HM27 BeadChip [17]–[20]. Kobayashi et al. reported more than 8,000 differentially methylated CpG sites (DMCs) in PCa samples compared with adjacent normal tissues using a relatively large number of samples [18]. Mahapatra et al. identified and confirmed many aberrantly methylated genes that can be used as diagnostic or prognostic biomarkers [20]. This HM27 BeadChip method provides DNA methylation data at single-nucleotide resolution in more than 27,000 CpG sites. However, this HM27 platform covers only 0.1% of all CpG sites in the human genome and all test CpG sites are located in promoter regions.

MethylPlex-next-generation sequencing (M-NGS) combines enzymatic enrichment of methylated CpG islands with a next-generation sequencing technique [16]. This method was used by Kim et al. to identify 2,481 cancer-specific differentially hypermethylated regions in promoter regions by comparing PCa samples, adjacent normal tissues, and disease-free prostate tissues. However, when using this method, Kim et al. did not report any results on hypomethylated regions in PCa. Furthermore, the size of differentially hypermethylated regions reported by Kim et al. was relatively large (3,000 base pairs). Using this method, it may difficult to identify core methylated regions that are critical for regulating gene expression; therefore an additional assay is required for precise assessment of DNA methylation at individual CpG sites [21]. Despite the weaknesses of each technique, these genome-wide DNA methylation studies have provided evidence that there are a lot of unidentified differentially methylated CpG sites (or regions, depending on the assay techniques), and these unidentified DMC may have important value as new drug targets and diagnostic or prognostic biomarkers.

Illumina HumanMethylation450 (HM450) is a newly developed BeadChip platform, which can test more than 480,000 individual CpG sites in the human genome [22], [23]. This coverage corresponds to around 1.7% of all CpG sites in the human genome, and represents a huge increase from the predecessor HM27 BeadChip (0.1% of all CpG sites). The high correlation between HM450 data and whole-genome bisulfite sequencing data indicates that this new BeadChip can provide reliable DNA methylation data for epigenomic profiling studies [23].

We conducted a pilot study using this new HM450 BeadChip to evaluate PCa samples and adjacent normal tissues. A set of genes that are deregulated by aberrant DNA methylation in PCa was identified and then combined with independent gene expression data. We then focused on two aberrant DNA methylation genes (*AOX1* and *SPON2*), with confirmation analyses using additional PCa and adjacent normal prostate tissues.

## Materials and Methods

### Study Subjects

All tissues specimens in this study were obtained from prostate cancer patients undergoing radical prostatectomy for treatment of clinically localized disease at the Karolinska Institute, Sweden (*n* = 23) and the Johns Hopkins Hospital (JHH; *n* = 111). All prostate samples used for this study were collected after appropriate human subjects approvals were obtained and written documentation of informed consent was provided. All study protocols were approved by the Karolinska Institute or the Johns Hopkins Hospital or the Wake Forest University School of Medicine Institutional Review Board. Subject samples were selected based on the ability to obtain genomic DNA of sufficient quantity and purity (>70% cancer cells for cancer specimens, no detectable cancer cells for normal samples) by macrodissection of matched adjacent non-malignant (hereafter referred to as normal) and cancer containing areas of prostate tissue as determined by histological evaluation of hematoxylin and eosin stained frozen sections of radical prostatectomy specimens. Genomic DNA was isolated from trimmed frozen tissues as described previously [24].

### DNA Methylation Assay

Genome-wide DNA methylation profiling was performed using the HM450 BeadChip (Illumina, San Diego, CA). Genomic DNAs were modified using the EZ-DNA methylation kit (Zymo Research, Orange, CA) following recommendations from Illumina. The Illumina Infinium assay was also conducted according to the manufacturer’s protocol.

DNA samples were modified in preparation for sequencing as described previously [25]. Bisulfite sequencing primers were designed by Methyl Primer Express software (Applied Biosystems, Foster City, CA) to amplify bisulfite modified DNA (Table S1). Hot Start polymerase chain reaction (PCR) (Qiagen, Valencia, CA) was performed using the following cycling program: 95°C for 15 minutes; 94°C for 30 seconds, 56°C for 30 seconds, and 72°C for 30 seconds for 50 cycles; and a final step at 72°C for 10 minutes. Amplified PCR products were subcloned using the TOPO TA cloning kit (Invitrogen, Carlsbad, CA). After transformation of *Escherichia coli*, we randomly selected approximately 10 to 20 individual *E. coli* colonies for each sample assessed. Plasmid DNA was directly amplified (Templiphi amplification kit; GE healthcare, Piscataway, NJ) and then sequenced using BigDye Terminator v1.1 Cycle Sequencing Kits (Applied Biosystems) with the M13 forward primer.

Each bisulfite sequencing primer set was evaluated using reconstituted control DNA sets that were mixtures in varying amounts (0, 20, 50, 80 and 100% methylation) of two control DNAs; *M.SssI*-treated DNA as methylated DNA (Millipore, Billerica, MA) and whole genome amplified DNA as unmethylated DNA [26], [27].

The bisulfite sequence data were compared with the UCSC genome reference sequence in order to assess the methylation status of each CpG site using BiQ Analyzer software [28]. Clones with a minimum of 95% bisulfite conversion rate were included in subsequent analyses. Each CpG site in our sequencing data was numbered from the 5′ to 3′ direction. CpG sites 2 and 34 in the *AOX1* promoter region were excluded from further analysis because of a frequent missing single nucleotide in long thymine homopolymeric stretches around each of these CpG sites.

### Published Methylation and Expression Data

Raw DNA methylation data in PCa using HM27 were downloaded from Gene Expression Omnibus, a public data repository [18]. These methylation data were generated using 86 adjacent normal prostate tissues and 92 primary PCa samples. Gene expression data in PCa that was based on the Affymetrix Exon 1.0 ST Array were downloaded from the data portal web site (http://cbio.mskcc.org/cancergenomics/prostate/data/) [29]. These expression data were generated using 29 adjacent normal tissues, 131 primary PCa samples and 19 metastatic PCa samples. Normalized log_2_ transformed gene-level (whole transcript signal intensity) data were used for statistical analysis.

### Statistical Analysis

Raw HM450 data were obtained using GenomeStudio software (Illumina) after scanning the BeadChips. Color balance adjustment, simple scaling normalization, and unsupervised hierarchical clustering analysis were conducted using the Bioconductor *lumi* package [30]. The β (methylation) value was calculated based on the suggested equation by Du et al. [31]. The β value is a continuous variable between 0 and 1, with β values approaching to 1 (or 0) indicating complete methylation (or no methylation, respectively) in each CpG site. CpG sites that had detection *P* values greater than 0.05, were excluded.

**Figure 1:**
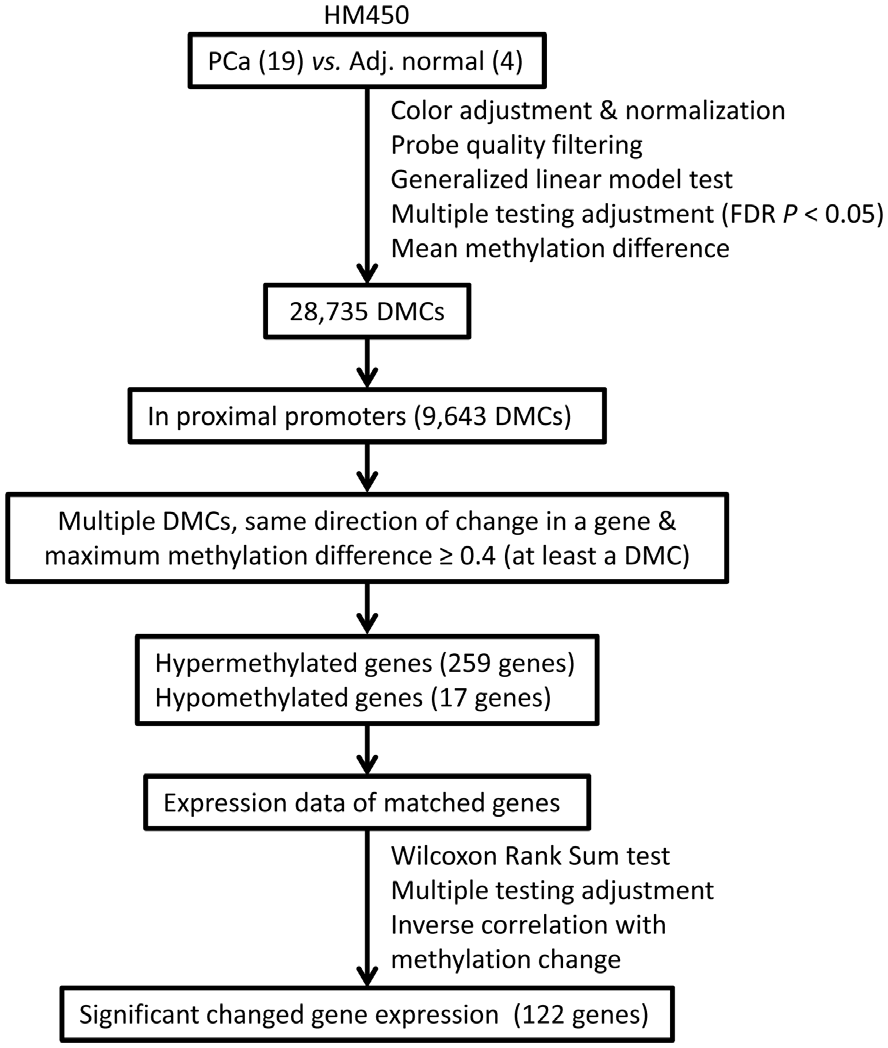
Schematic of selection procedure to identify differentially methylated CpG sites.

Association between each phenotype or clinicopathological parameter and DNA methylation at each CpG site was tested separately within the HM450 data. These statistical tests were performed with a generalized linear model using PROC GENMOD in SAS software (SAS Institute Inc, Cary, NC) [32]. The beta-distribution of β values was accounted for with a binomial logit link and a scale variable estimated with Pearson residuals. We determined whether each individual CpG site was statistically significant based on the false discovery rate (FDR) in order to correct possible false positives from multiple tests (α = 0.05). We also subsequently calculated a mean methylation (β value) of a CpG site in each group and selected CpG sites that had greater or equal to 0.2 mean methylation difference between two comparison groups [27], [33]. Therefore, a differentially methylated CpG (DMC) was defined as a CpG site that had a FDR adjusted *P* value <0.05 from a generalized linear model test and a mean methylation difference between two comparison groups greater or equal to 0.2 (Figure 1). The HM27 data were also processed using the same procedure.

We used Fisher’s exact test to test the associations between DMCs and genomic locations across the genome. The statistical test for gene expression was performed with Wilcoxon Rank Sum test in SAS software. We determined whether each individual gene was statistically significant based on the FDR adjusted *P* value (α = 0.05).

## Results

### Identification of Differentially Methylated CpG Sites in PCa

We conducted genome-wide DNA methylation profiling using the Illumina HM450 BeadChip. We evaluated four paired tumor samples and adjacent normal tissues, plus 15 additional unpaired tumor samples that were originally collected from Swedish subjects (Table S2A). After normalization, all 23 tissue samples showed very similar signal intensity distributions, while unsupervised hierarchical clustering on the entire DNA methylation data set showed the two main clusters were each composed by a phenotype; tumor or normal (Figure S1). This clear separation between PCa and normal samples indicated a different DNA methylation pattern between two phenotypes.

To identify aberrant DMCs in prostate cancer, we performed statistical analysis and narrowed down the selection of CpG sites based on DNA methylation differences (Figure 1). A total of 28,735 CpG sites showed statistical significance with FDR adjusted *P*<0.05 and at least 0.2 mean methylation difference between PCa and normal samples (Table S3). These 28,735 DMCs included 20,187 hypermethylated CpG sites (hyper-DMCs) and 8,548 hypomethylated CpG sites (hypo-DMCs) in tumors compared with normal tissues. Interestingly, 69.5% of hyper-DMCs (14,038 CpG sites) were located in CGI, CGI shores or CGI shelves, but only 37.0% of hypo-DMCs were found in these regions (Fisher exact two-tailed *p*<10^−44^; Table 1A). Furthermore, the distribution of identified hyper- and hypo-DMCs in tumors was statistically different between gene region categories provided by the manufacturer (Fisher exact two-tailed *p* = 2.0×10^−44^; Table 1B) [23]. Hyper-DMCs were more frequently identified in proximal promoter regions (including transcription start site [TSS]1500, TSS200, 5′untranslated region [UTR] and1st exon) than hypo-DMCs, however hypo-DMCs were more often found in non-promoter regions (including gene body and 3′UTR).

**Table 1:** Distribution of differentially methylated CpG sites in genomic regions.

A. Between CGI and non-CGI regions.			
	Hyper-DMC	Hypo-DMC	P value
CGI*	14038	3160	<10^−44^
Non-CGI	6149	5388	
**B. Between promoter and non-promoter regions**			
	**Hyper-DMC**	**Hypo-DMC**	**P value**
Promoter*	7361	2282	2.0×10^−44^
Non-promoter*	6686	3241	

* CGI: CGIs, CGI shores, and CGI shelves.

* promoter: TSS1500, TSS200, 5′UTR, and 1st exon.

* non-promoter: gene body and 3′UTR.

A total of 19,570 DMCs located in genic regions, corresponded to 7,031 unique genes. A total of 32 out of 67 genes previously reported in PCa to have promoter hypermethylation were identified in our list (Table S4) [10]. This number exceeds the number of common genes from the M-NGS method (20 common genes), which is somewhat surprising because M-NGS provides better practical coverage of genomic CpG (29.6%) sites compared with the HM450 BeadChip (1.7%) [16].

We compared our results with published data sets. Among 28,735 DMCs, a total 1,175 CpG sites were present in the HM27 BeadChips that were used in a previous PCa study [18]. Statistical analysis indicated that 1,157 (98.5%) out of 1,175 CpG sites showed significantly different DNA methylation between tumor and normal samples (FDR adjusted *P*<0.05) and all of these 1,157 CpG sites showed the same direction of methylation change in the two data sets (928 hyper-DMCs and 229 hypo-DMCs, Table S3).

### DMC Associations with Deregulated Gene Expression in PCa

To find aberrant DNA methylation that may cause gene expression change in PCa, we analyzed gene expression data among our list of DMC genes. To increase the likelihood of identifying true positives, we only tested genes that met three criteria. First, more than one DMC were identified in proximal promoter regions (TSS1500, TSS200, 5′UTR and 1st exon) of a gene; second, at least three quarters of these multiple DMCs in a gene promoter region had the same direction of methylation changes (hyper or hypomethylation in PCa); third, at least one DMC in a gene promoter region had at least 0.4 mean methylation difference between cancer and normal phenotypes. Based on these criteria, a total of 276 genes were selected from 9,643 DMCs that were located in proximal gene promoter regions and available 269 matched gene expression data were tested [29]. A total of 122 genes showed significant differences in gene expression between tumor and normal samples (FDR adjusted *P*<0.05 and inverse correlation between DNA methylation and expression change; Table 2 and Table S5). This set included seven known methylation genes in PCa, for example, *GSTP1, CAV1, and RARB*. A total of 65 out of 122 genes are confirmed to have differential methylation in the HM27 data [18]. These 65 confirmed genes in the HM27 data showed the same direction of methylation changes with our HM450 data. Furthermore, 55 out of 122 genes has been identified as cDMRs in next-generation sequencing methylation data (M-NGS) [16].

**Table 2:** Hypermethylated (top 10) and hypomethylated (all) genes in PCa*.

	Gene expression†		HM450		HM27†		M-NGS†	Known methylated gene†
Gene Symbol	FDR *P*	ΔLog Intensity‡	No. of DMC#	Δβ‡	FDR *P*	Δβ‡		
*AOX1*	1.5E-08	−1.07	11	0.52	2.8E-23	0.33	cDMR	
*GSTP1*	1.1E-09	−1.05	3	0.48	3.6E-28	0.31	Group3	Yes
*CAV1*	4.0E-09	−1.04	8	0.43	9.5E-26	0.24		Yes
*FABP3*	1.5E-08	−0.76	5	0.41				
*TIMP2*	5.0E-06	−0.73	3	0.57			cDMR	
*GSTM2*	2.4E-06	−0.69	6	0.44	1.8E-19	0.23	cDMR	
*ANXA2*	5.2E-07	−0.69	7	0.50			cDMR	
*EFEMP1*	7.6E-07	−0.68	5	0.47	1.2E-27	0.28	cDMR	
*ANXA6*	3.2E-07	−0.66	5	0.52	1.3E-30	0.29	cDMR	
*ACSF2*	7.8E-10	−0.64	3	0.49	4.0E-33	0.29	cDMR	
*MC5R*	3.9E-02	0.11	3	−0.46				
*KIAA0182*	1.2E-02	0.14	10	−0.43				
*LOC100130872*	4.1E-03	0.35	5	−0.50				
*ALDH1A3*	6.0E-07	0.61	8	−0.45	1.2E-20	−0.20		
*CLDN8*	2.4E-07	0.81	4	−0.43	1.9E-18	−0.29		
*SPON2*	3.1E-04	0.93	5	−0.50				
*GDF15*	6.0E-07	1.06	2	−0.46			cDMR	

† Expression [29], HM27 [18], M-NGS [16], Known methylated gene [10].

‡ Difference between PCa and normal (Tumor - Normal). Δβ (methylation difference) indicated the biggest difference among multiple DMCs.

# Only located in the proximal promoter.

* See Table S5 for the full list of 122 genes.

### Confirmation of the AOX1 Promoter Hypermethylation in Additional PCa Samples

Among the set of 122 aberrantly methylated genes, *AOX1*, which encodes aldehyde oxidase 1 and is located at chromosome 2q33.1, was the most significantly down-regulated gene in PCa samples, based on HM450 data. The changes in DNA methylation and gene expression of *AOX1* between PCa and normal tissues were very similar to *GSTP1* (Table 2). A total of 13 out of 19 tested CpG sites in the *AOX1* gene were identified as hyper-DMCs (range of FDR adjusted *P*: 7.4×10^−8^∼0.01 and range of mean methylation difference: 0.20∼0.52) and 11 of these 13 DMCs were located in proximal promoter regions (Table 2 and Figure S2A).

Two CpG sites in the *AOX1* promoter region were also confirmed as hyper-DMCs in the independent HM27 data set (Table S3 and Figure S2A) [18]. Mahapatra et al. also identified this gene as a differentially methylated gene using the same HM27 platform [20]. Kim et al. identified a differentially methylated region in *AOX1* that overlaps with our 13 hyper-DMC sites using M-NGS method in PCa samples [16]. Kim et al. also performed bisulfite sequencing of the *AOX1* promoter in two prostate cell lines, in which they confirmed evidence of differential methylation in *AOX1*. However methylation status in PCa tissue samples with single-nucleotide resolution has not been performed yet.

**Figure 2:**
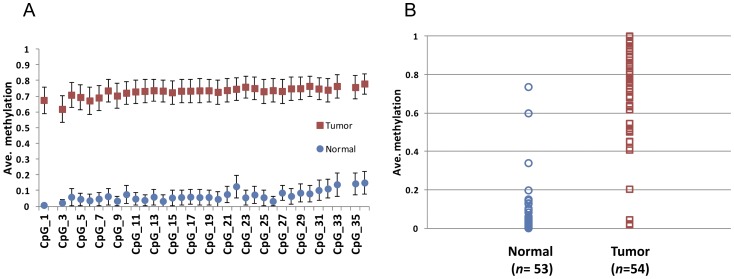
Differential *AOX1* promoter methylation in PCa samples and normal prostate tissues. A. Average methylation of 34 tested CpG sites in each phenotype. Error bars indicate 95% confidence intervals for each CpG site. B. Distribution of *AOX1* promoter methylation in each phenotype. Each circle or square represents the average methylation of 34 tested CpG sites in each tested tissue sample.

Therefore, we used bisulfite sequencing to confirm our finding in the *AOX1* gene among additional PCa and normal prostate tissue samples (Table S2B). We amplified the *AOX1* promoter region, which included 5 hyper-DMCs and overlapped with a CpG island, then sequenced after bacterial transformation (Figure S2A). Data from a series of control DNAs showed reliable results from this bisulfite sequencing primer set (Figure S2B). Among a total of 107 tissue samples from JHH including 54 PCa samples and 53 normal tissues (51 matched pairs), we observed the *AOX1* gene promoter to be heavily hypermethylated in PCa samples compared with normal tissues at all of the 34 tested CpG sites (mean methylation in all 34 CpG sites: PCa *vs*. normal = 0.73 *vs*. 0.07, Figure 2A and Table S6); these differences were statistically significant (FDR adjusted *P*<8.8×10^−11^). We plotted the mean methylation of all 34 CpG sites in each tumor sample and the matched normal samples (*n* = 51, Figure S2C). This plot indicated the almost all of the tumor samples had greater than the mean methylation value of 0.3 in the *AOX1* promoter, while paired adjacent normal tissue had relatively lower methylation (less than mean methylation value 0.3). After reviewing these data, we arbitrarily set a cut-off level of 0.3 for assigning *AOX1* promoter methylation to distinguish tumor from normal tissue (Figure 2B). The sensitivity of DNA methylation in the *AOX1* promoter for the detection of PCa was 92.6% (50 out of 54 tested PCa samples) and 94.3% of positive prediction value. We were not able to calculate specificity in this replication population, because the samples included adjacent normal tissues of PCa patients. However, 50 out of 53 (94.3%) of these normal samples showed negative results of *AOX1* promoter methylation.

### Confirmation of the SPON2 Promoter Hypomethylation in Additional PCa Samples

*SPON2*, which encodes spondin 2 and is located at chromosome 4p16.3, was the second most up-regulated gene among the set of 122 significantly deregulated genes in PCa, based on HM450 data. A total of five CpG sites in the *SPON2* promoter were identified as hypo-DMCs (range of FDR adjusted *P*: 2.1×10^−14^∼8.0×10^−8^ and range of mean methylation difference: –0.20∼–0.50) and these five DMCs were also located in the LOC100130872 promoter region (Table 2).

**Figure 3:**
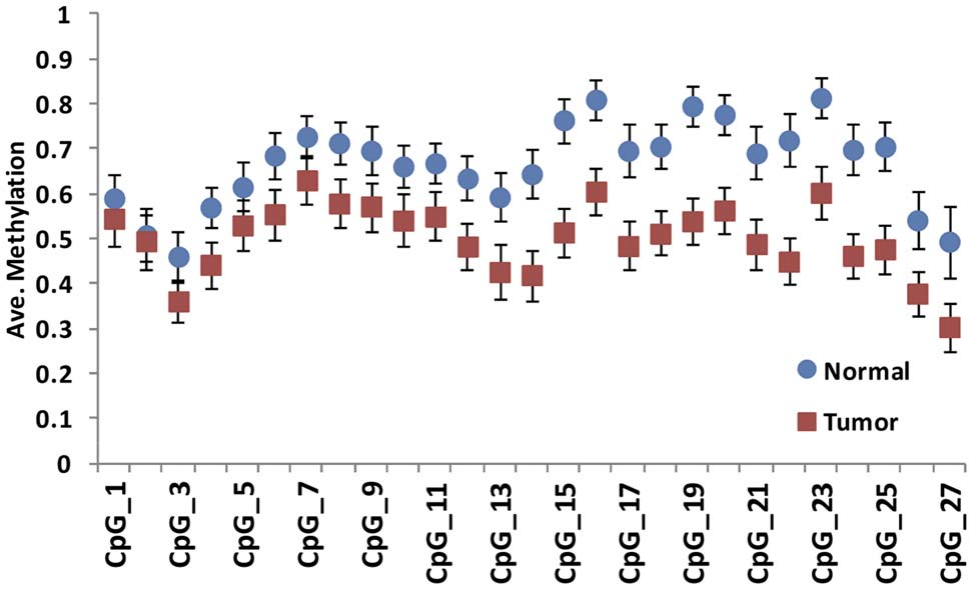
Differential *SPON2* promoter methylation in PCa samples and normal prostate tissues. Each circle or square represents the average methylation of 27 tested CpG sites in each phenotype. Error bars indicate 95% confidence intervals for each CpG site.

We also confirmed methylation of the *SPON2* promoter region, which includes five hypo-DMCs and is located in a CpG island shore, using the same bisulfite sequencing method described above (Figure S3A). Data from a series of control DNAs showed reliable results from this bisulfite sequencing primer set (Figure S3B). A total of 109 JHH tissue samples including 54 PCa samples and 55 normal tissues (54 matched pairs) were tested for *SPON2* promoter methylation. The result of bisulfite sequencing showed hypomethylation of the *SPON2* gene promoter in PCa samples compared with normal tissues, and these differences were statistically significant in 25 out of 27 tested CpG sites (mean methylation difference between PCa samples and normal tissues: –0.1 ∼ –0.27, Figure 3 and Table S7). However, methylation values in this set of 25 CpG sites showed more variation than what we observed for the *AOX1* promoter (Figure 2A). A total of 55 normal tissues showed mean DNA methylation between 0.46 and 0.81 in the 25 CpG sites, but PCa samples (*n* = 54) showed average DNA methylation between 0.30 and 0.63. DNA methylation of the *SPON2* promoter did not show any association with clinicopathological parameters including age at the time of surgery, Gleason score, and TNM stage in PCa samples and normal tissues (data not shown).

### DMCs Associated with Clinicopathological Properties

To identify CpG sites associated with clinicopathological properties, we compared the methylation data of PCa samples with Gleason score or status of biochemical recurrence (BCR). First, PCa samples were divided by Gleason score (lower Gleason 6 and 3+4 vs. higher Gleason 4+3, 9, 10). A total of 245 CpG sites were identified as Gleason score associated DMCs (FDR adjusted *P*<0.05), but only 40 CpG sites showed greater than 0.2 mean methylation difference between higher and lower Gleason PCa (Table S8). Gene expression data of 22 genes that had Gleason score associated DMCs in genic regions were tested for association with Gleason score in PCa samples. Among these 22 tested genes, only *PPARGC1A* showed significant differences of gene expression between tumors with lower Gleason versus higher Gleason PCa (FDR adjusted *P* = 0.022). Furthermore, methylation of *PPARGC1A* (cg12691631) was inversely correlated with gene expression (Figure S4).

Comparisons between BCR (biochemical recurrence; defined as detection of total PSA greater than 0.2 ng/ml after surgery) and non-BCR groups identified only two CpG sites as DMC (cg11385353 in *AGXT* and cg14218447 in an intergenic region) using FDR adjusted *P*<0.05 and greater than 0.2 mean methylation difference between two groups. These two CpG sites showed significant difference after adjustment for treatment information. However, gene expression of *AGXT* showed no difference between BCR and non-BCR primary PCa (data not shown).

## Discussion

We tested DNA methylation in more than 480,000 CpG sites using the HumanMethylation450 Beadchip in PCa samples and normal tissues. We successfully identified 28,735 differentially methylated CpG sites in PCa. Many of the identified DMCs were independent replications of previous findings of differential DNA methylation in PCa [16], [18]. In the first, 98.5% (1,157 out of 1,175) of common CpG sites that were present in our DMC list and the HM27 BeadChip showed significant methylation differences in the previous data set [18]. In the second, a total of 1,014 out of 2,481 (40.9%) cancer-specific methylated regions that were identified using the M-NGS method in PCa, overlapped with our DMCs [16]. When considering the huge differences in the practical CpG coverage of these two methods (M-NGS *vs*. HM450∶29.6% *vs*. 1.7%), the concordance rate of 40.9% can be considered very high.

The distribution of DMCs in the HM450 showed a similar pattern to the distribution of DMCs in a colon cancer cell line [22]. Sandoval et al. found that most hypermethylated CpG sites were located in CGI, CGI shore, and CGI shelf regions, while more than half of hypermethylated CpG sites were indentified in proximal promoter regions. Our data was consistent with that observation, showing that 69.5% of hyper-DMCs were located in CGI, CGI shore, and CGI shelf regions, while 52.4% of hyper-DMCs were indentified in proximal promoter regions.

We have noticed that a total of 6,907 CpG sites assayed by the HM450 BeadChip happen to be located within single nucleotide polymorphism (SNP) loci. A total of 336 CpG sites among the 28,735 DMCs that we identified were also located in SNP loci as indicated in Table S3. Therefore special attention is required to interpret any observed associations with these 336 DMCs.

We chose to limit our analysis to the proximal promoter region because this region is well characterized for its effects of DNA methylation on gene silencing. This approach also allowed us to evaluate associations with gene expression changes based on an independent PCa data set, based on the assumption that inverse correlations between promoter methylation and gene expression may plausibly indicate a functional result of differentially methylated genes identified in PCa. This approach identified a total of 122 genes including seven known DNA methylation genes in PCa. As positive confirmation of our approach, *GSTP1*, which is the most thoroughly studied hypermethylated gene in PCa was found to be hypermethylated in this analysis (Table 2). A total 7 out of 15 CpG sites in the HM450 BeadChip were identified as hyper-DMCs (range of mean methylation difference: 0.27 ∼ 0.48) in the *GSTP1* gene (Table S3). Messenger RNA of the *GSTP1* gene has been previously shown to be significantly down-regulated in primary PCa, and metastatic tumors show further down-regulation [29].

The aldehyde oxidase 1 (*AOX1*) gene is involved in various metabolic pathways, including drug metabolism and generation of reactive oxygen species [34]–[36]. Reduced AOX1 protein expression in chronic pancreatitis and an absence of AOX1 protein expression in pancreatic cancer have been reported [34]. In addition, decreased AOX1 protein expression was detected in hepatocellular carcinoma and this deregulation of AOX1 expression was associated with tumor stage and metastatic status [37]. Aberrant DNA hypermethylation of the *AOX1* promoter region was recently reported in colon cancer and PCa [16], [18], [20], [38], [39]. Within the context of these prior published data, our data further supports a role for aberrant DNA methylation in the *AOX1* promoter of PCa tumors, while also providing the most comprehensive coverage of DMCs in this gene to date.

The observed differences of DNA methylation in the *AOX1* promoter between PCa samples and normal tissues were remarkable. This promoter region showed a very consistent methylation pattern in 34 CpG sites spanning 400 base pairs in each phenotype. This genomic location is amenable to evaluation via other assays, such as Pyrosequencing or methylation-specific PCR.

The majority of PCa tumors (92.6%; sensitivity) were observed to have positive DNA methylation, with a mean methylation cut-off of 0.3 and 94.3% of normal tissues showing negative DNA methylation with the same cut-off. This sensitivity of *AOX1* methylation was similar to other well-known methylated genes including *GSTP1* and *RARB* that were tested using Pyrosequencing methods [40]. This high sensitivity of *AOX1* promoter methylation and huge differences of DNA methylation between PCa samples and normal tissues suggest the potential utility of this gene as a biomarker for PCa diagnosis. However, additional studies will be required before assessing the feasibility of this gene as a biomarker. Studies of *AOX1* promoter methylation status in control groups and/or using biofluid samples such as serum and urine may be needed.

Our analysis also associated promoter methylation with reduced *AOX1* gene expression in PCa samples compared with normal tissues and further decreased expression of this gene in metastatic tumor samples (Figure S5A). This *AOX1* gene expression was associated with Gleason score in PCa (*P* = 0.012). PCa samples with higher Gleason scores showed lower *AOX1* gene expression than samples with lower Gleason PCa (Figure S5A). The HM27 methylation data showed hypermethylation in samples with higher Gleason PCa than in samples with lower Gleason PCa (*P* = 0.039, Figure S5B). However, our bisulfite sequencing data on the *AOX1* promoter region did not show any association with clinicopathological parameters including age at the time of surgery, Gleason score, and TNM stage in PCa or normal samples (data not shown). The precise molecular mechanism of deregulated *AOX1* in human carcinogenesis remains unknown and may need to be explored in the future.

Interestingly, AOX1 converts 5-hydroxyindoleacetaldehyde to 5-hydroxyindoleacetate, which is a significantly down-regulated metabolite in metastatic prostate cancers compared to primary tumor samples (Figure S6) [41]. This metabolite 5-hydroxyindoleacetate is produced by two major enzymes, aldehyde dehydrogenase family proteins (*ALDH2* and *ALDH3A2* etc.) and aldehyde oxidase (*AOX1*) in tryptophan metabolic pathway [42]. The expression of *ALDH2* and *ALDH3A2* were significantly down-regulated in primary tumor samples and further down-regulated in metastatic tumors, similar to *AOX1* expression (Figure S7A, B, and C) [29]. Expression of *MAOB*, which catalyzes the conversion of serotonin to 5-hydroxyindoleacetaldehyde was also significantly down-regulated (Figure S7D). This data supports the hypothesis that some portion of the tryptophan metabolic pathway is deregulated in PCa development and progression. However, the biological effects of down-regulated *AOX1* or 5-hydroxyindoleacetate on PCa development may need to be further studied.

The *SPON2* gene encodes spondin2, which is an extracellular matrix protein. Spondin2 protein has multiple biological functions including neuronal development and innate immune response [43], [44]. Significantly increased expression of *SPON2* mRNA and protein has been previously reported in PCa [45], [46]. The biological function of the *SPON2* gene in human carcinogenesis is not yet elucidated, but it was used as the target of antibody-based radiotherapy in PCa [46]. This is the first time aberrant *SPON2* hypomethylation was identified and an inverse correlation was observed between *SPON2* methylation and *SPON2* gene expression.

Our comparison of DNA methylation in PCa samples and normal tissues identified more DMCs than did the other comparisons groups we examined such as the Gleason score PCa groups. Comparison of PCa progression groups showed relatively small changes in global DNA methylation and these relatively small changes were observed in three studies that used a BeadChip technique, including the current study [18], [20]. Such evaluations may require more precise detection methods with much larger CpG coverage to detect DNA methylation changes in PCa progression.

The HM450 BeadChip tested 18 different CpG sites in the *PPARGC1A* gene. A Gleason-associated DMC (cg12691631) was identified in a CpG site that is located within the promoter region of the *PPARGC1A* gene. Among 18 CpG sites on the HM450, eight additional CpG sites showed weak associations with Gleason score (range of raw *P* values: 5.13×10^−6^ ∼ 0.03) and all of these CpG sites showed higher methylation in higher Gleason PCa than lower Gleason PCa. The protein encoded by this *PPARGC1A* gene is a transcriptional coactivator that regulates the genes involved energy metabolism [47], [48]. This protein is known to interact with other nuclear receptors such as estrogen receptor α and androgen receptor [49], [50]. Furthermore, a genetic variant in this gene was associated with breast cancer [51]. One CpG site in the HM27 data also showed weak association with Gleason score (*P* values = 0.01 with higher methylation in higher Gleason PCa). This may need to be confirmed amongst a larger number of samples.

Even though DMCs from non-promoter regions were excluded from this study, future studies should not ignore the importance of these DMCs. DNA methylation in non-promoter regions may play functional roles [2], [52], [53]. DNA methylation of intragenic regions may regulate gene expression by functioning as alternative promoters [2], [53]. However, the correlation between DNA methylation of intragenic regions and gene expression is not clear because contradictory results have been obtained in different cells or tissues [2], [53], [54]. It is also not clear whether DNA methylation of intragenic regions is a consequence of other molecular mechanisms [55]. A total of 204 DMCs (81 genes) passed the same selection steps and it is possible that DMCs in non-promoter regions will have diagnostic potential as a biomarker. Therefore, many DMCs in these non-promoter regions need to be evaluated in the future.

In this study, we used only the gene-level expression data, without considering alternative promoters, in order to avoid over-complication of the analysis. However, it should be possible to perform exon-level analysis using higher coverage HM450 data and exon expression data. Such analysis may provide interesting results on the effect of DNA methylation on alternative splicing or alternative promoters within a gene.

In this study, we identified 122 differentially methylated genes that were functionally deregulated in prostate cancer. A quarter of the genes in this set (32 genes) were newly identified as aberrantly methylated genes in PCa. Some of these genes may have the potential to distinguish between tumor versus normal tissues, and thus could serve as a diagnostic biomarker.

## Supporting Information

Figure S1 **Unsupervised hierarchical clustering of the entire HM450 DNA methylation data.** A. Distribution of signal intensities of individual Swedish samples assayed via HM450 BeadChip after color balance adjustment and simple scaling normalization. B. A dendrogram of unsupervised hierarchical clustering using whole HM450 DNA methylation data after color balance adjustment and simple scaling normalization. These results were generated using the Bioconductor *lumi* package. SPCA or SPN in the sample number indicates tumor sample or normal tissue, respectively.
(PDF)

Figure S2 **Bisulfite sequencing of the** ***AOX1*** **promoter region.** A. DMCs in the *AOX1* promoter region has been identified by multiple PCa studies; the HM27 array (purple tick marks), the HM450 array (red tick marks) and the M-NGS data set (blue bar; partial). Black and green bars indicate the bisulfite-sequenced region and a CpG island, respectively. B. Results of bisulfite sequencing using control DNAs. Five different reconstituted (0, 20, 50, 80 and 100% methylation) control DNAs were tested. C. Pair-wise comparison of the average methylation of 34 tested CpG sites in tumor and matched normal tissue (*n* = 51).
(PDF)

Figure S3 **Bisulfite sequencing of the** ***SPON2*** **promoter region.** A. DMCs (red tick marks) in the *SPON2* promoter region as identified by the HM450 array. Dark blue bars and lines indicate RefSeq genes *SPON2* and *LOC100130872*, respectively. Black bars indicate a region evaluated with bisulfite sequencing. Green bars show a CpG island. B. Results of bisulfite sequencing using control DNAs. Five different reconstituted (0, 20, 50, 80 and 100% methylation) control DNAs were tested.
(PDF)

Figure S4 **Aberrant DNA methylation and gene expression of** ***PPARGC1A*** **associated with Gleason score in PCa.** A. Box plot of DNA methylation at the cg12691631 CpG site, in lower Gleason PCa and higher Gleason PCa samples. B. Box plot of *PPARGC1A* gene expression in PCa samples. The labels L-GS, H-GS, and Met indicate lower Gleason PCa, higher Gleason PCa, and metastatic cancer samples, respectively.
(PDF)

Figure S5 **Association of** ***AOX1*** **gene expression (A) or DNA methylation (B) with Gleason score in PCa.** A. Box plot of *AOX1* expression in PCa samples. The labels L-GS, H-GS, and Met indicate lower Gleason PCa, higher Gleason PCa, and metastatic cancer samples, respectively. B. Box plot of *AOX1* methylation (cg02144933 of the HM27 data set) in PCa samples.
(PDF)

Figure S6 **Schematic of enzyme reactions from serotonin to 5-hydroxyindoleacetate in the tryptophan metabolic pathway.** Significantly deregulated genes in PCa are highlighted with green color.
(PDF)

Figure S7 **Aberrant expression of tryptophan metabolic pathway genes in PCa.** Box plots of *ALDH2* (A), *ALDH3A2* (B), *AOX1* (C), and *MAOB* (D) gene expression in PCa samples. The labels normal, PCa, and Met indicate adjacent normal prostate tissues, primary prostate cancer samples and metastatic cancer samples, respectively. Comparisons between normal and PCa, PCa and Met in four genes were statistically significant (*P*<0.05).
(PDF)

Table S1 **Primer sequences for bisulfite sequencing assay.**
(PDF)

Table S2 **Clinicopathological information of tissue samples.** A. Inforamtion of the HM450 profiling samples. B. Inforamtion of the bisulfite sequencing samples.
(PDF)

Table S3 **List of DMCs from HM450 data.**
(PDF)

Table S4 **Comparison of differentially methylated genes between genome-wide profiles and 67 known methylated genes in PCa.**
(PDF)

Table S5 **Differentially methylated genes by combination of DNA methylation and gene expression data.**
(PDF)

Table S6 **Results of bisulfite sequencing in the AOX1 promoter region.**
(PDF)

Table S7 **Results of bisulfied sequencing in the SPON2 promoter region.**
(PDF)

Table S8 **List of Gleason score-associated DMCs in PCa.**
(PDF)
