# Supplementary figures and images for: Identification of New Differentially Methylated Genes That Have Potential Functional Consequences in Prostate Cancer
Source: PLoS One. 2012 Oct 31;7(10):e48455. doi: 10.1371/journal.pone.0048455 (PMC3485209; doi:10.1371/journal.pone.0048455)

Figure S1.

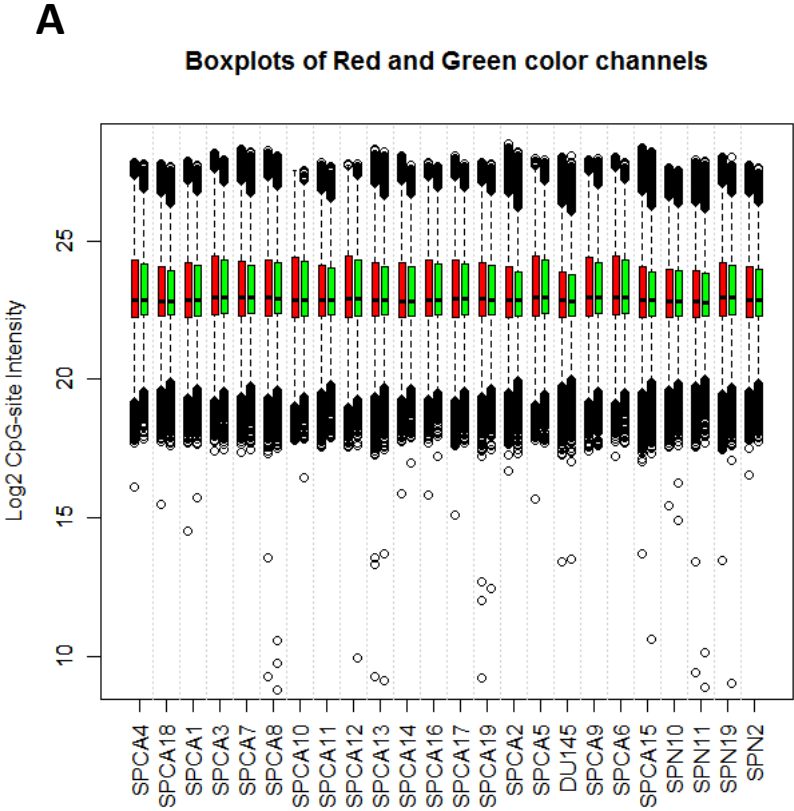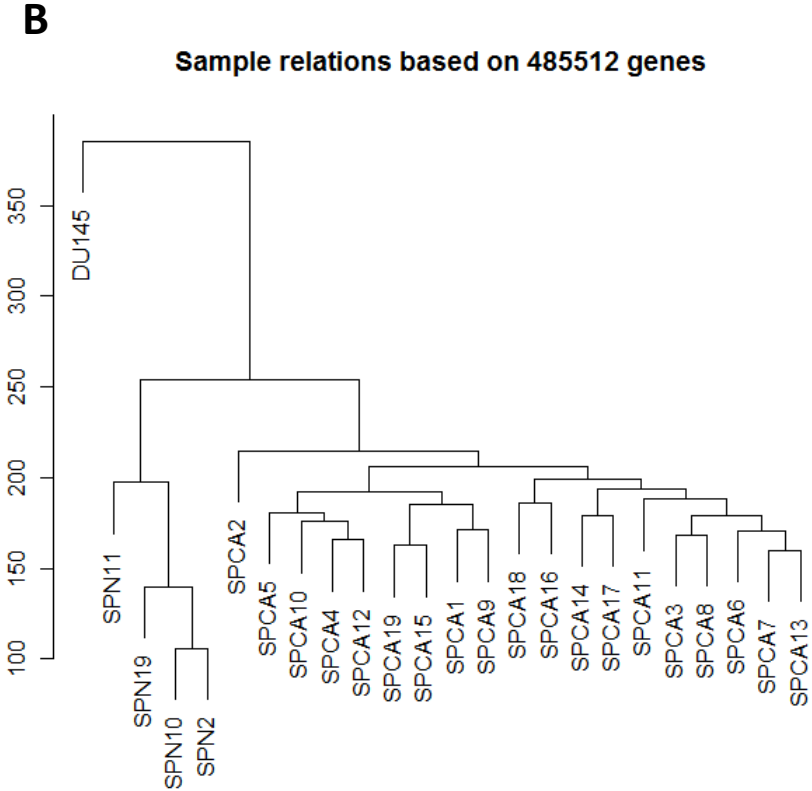

Supplement: Figure S1 — Unsupervised hierarchical clustering of the entire HM450 DNA methylation data. A. Distribution of signal intensities of individual Swedish samples assayed via HM450 BeadChip after color balance adjustment and simple scaling normalization. B. A dendrogram of unsupervised hierarchical clustering using whole HM450 DNA methylation data after color balance adjustment and simple scaling normalization. These results were generated using the Bioconductor lumi package. SPCA or SPN in the sample number indicates tumor sample or normal tissue, respectively. (PDF) [file pone.0048455.s001.pdf]

Figure S2.

A

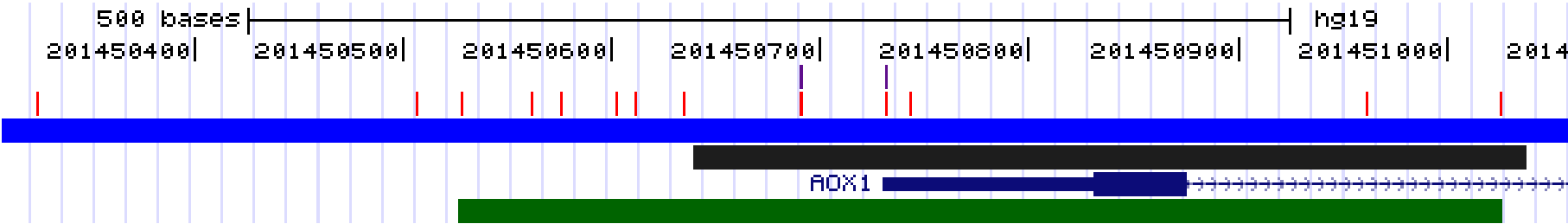

B

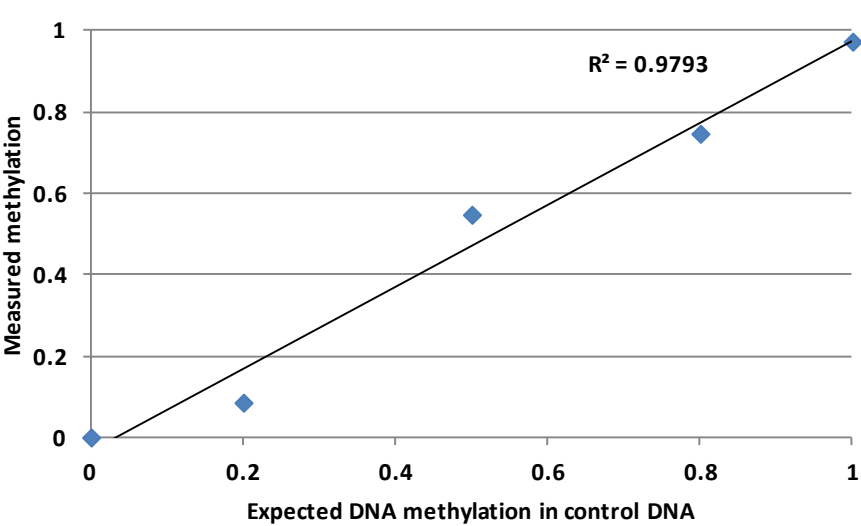

C

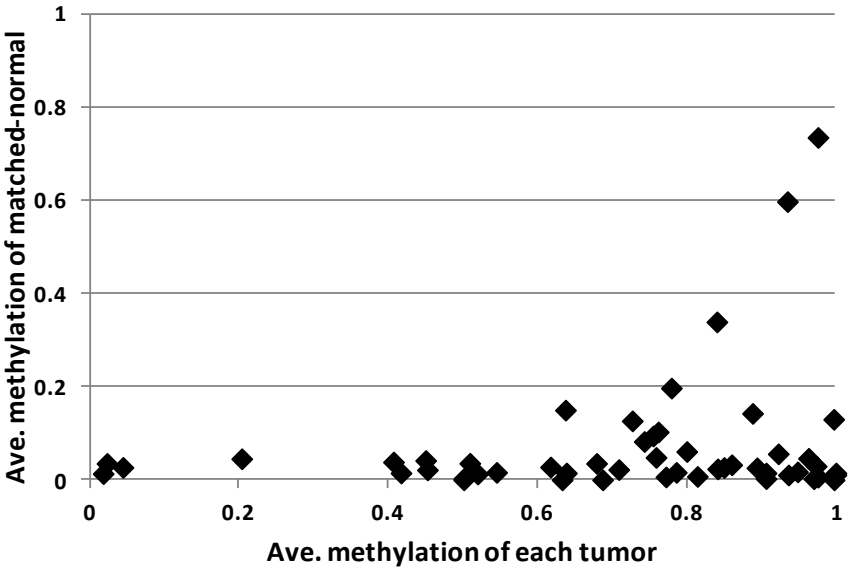

Supplement: Figure S2 — Bisulfite sequencing of the AOX1 promoter region. A. DMCs in the AOX1 promoter region has been identified by multiple PCa studies; the HM27 array (purple tick marks), the HM450 array (red tick marks) and the M-NGS data set (blue bar; partial). Black and green bars indicate the bisulfite-sequenced region and a CpG island, respectively. B. Results of bisulfite sequencing using control DNAs. Five different reconstituted (0, 20, 50, 80 and 100% methylation) control DNAs were tested. C. Pair-wise comparison of the average methylation of 34 tested CpG sites in tumor and matched normal tissue (n = 51). (PDF) [file pone.0048455.s002.pdf]

Figure S3.

A

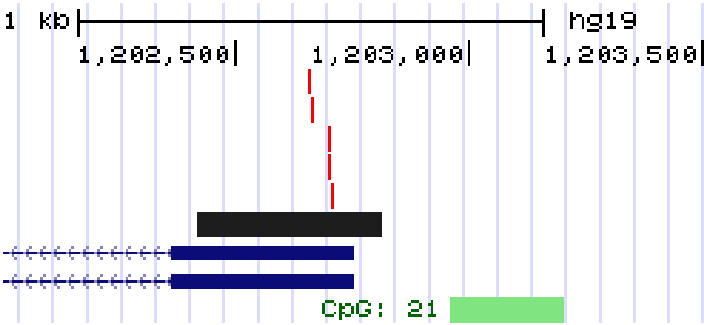

B

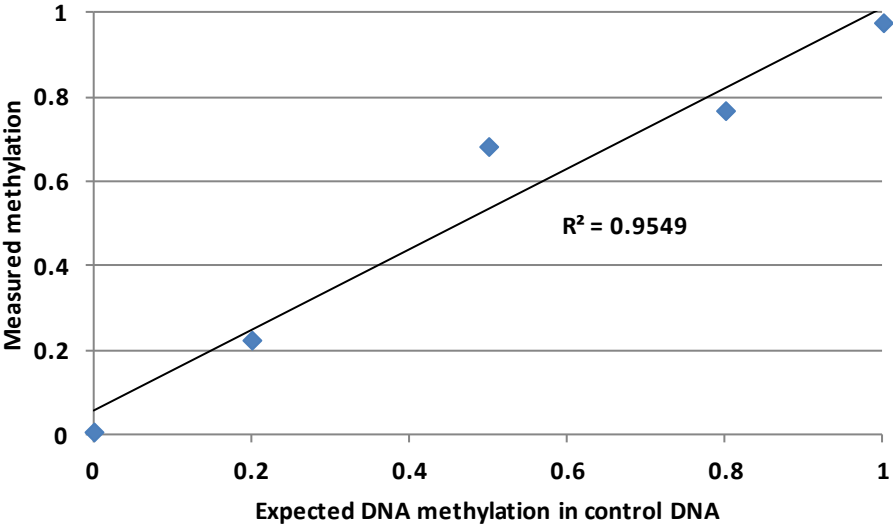

Supplement: Figure S3 — Bisulfite sequencing of the SPON2 promoter region. A. DMCs (red tick marks) in the SPON2 promoter region as identified by the HM450 array. Dark blue bars and lines indicate RefSeq genes SPON2 and LOC100130872, respectively. Black bars indicate a region evaluated with bisulfite sequencing. Green bars show a CpG island. B. Results of bisulfite sequencing using control DNAs. Five different reconstituted (0, 20, 50, 80 and 100% methylation) control DNAs were tested. (PDF) [file pone.0048455.s003.pdf]

Figure S4.

**A**

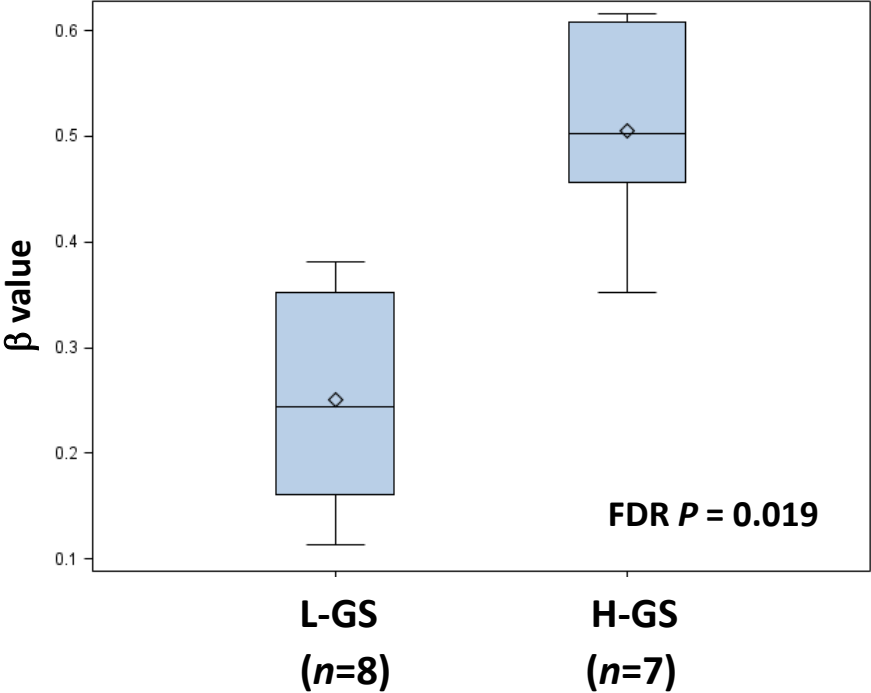

**B**

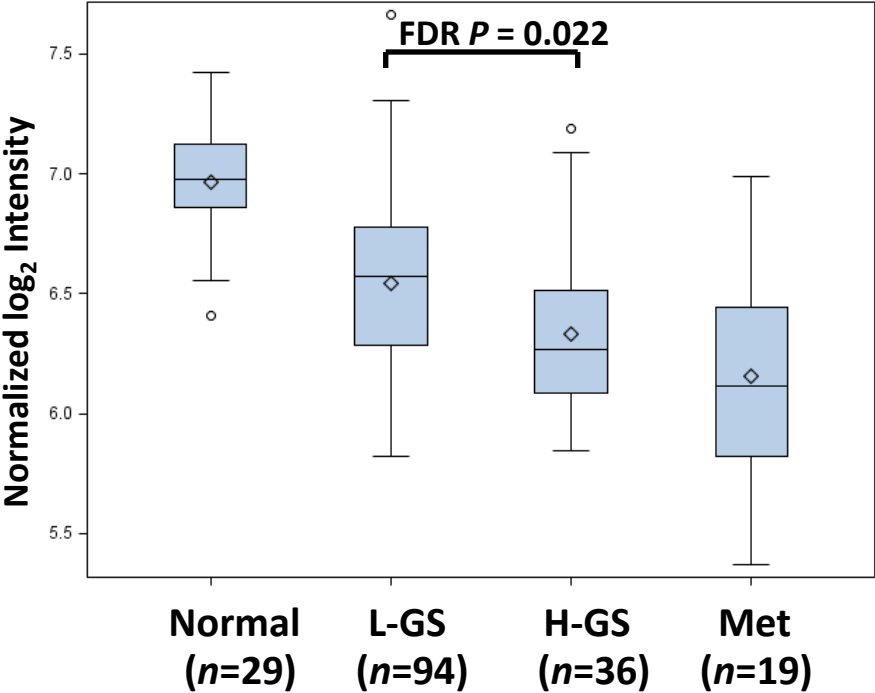

Supplement: Figure S4 — Aberrant DNA methylation and gene expression of PPARGC1A associated with Gleason score in PCa. A. Box plot of DNA methylation at the cg12691631 CpG site, in lower Gleason PCa and higher Gleason PCa samples. B. Box plot of PPARGC1A gene expression in PCa samples. The labels L-GS, H-GS, and Met indicate lower Gleason PCa, higher Gleason PCa, and metastatic cancer samples, respectively. (PDF) [file pone.0048455.s004.pdf]

Figure S5.

**A**

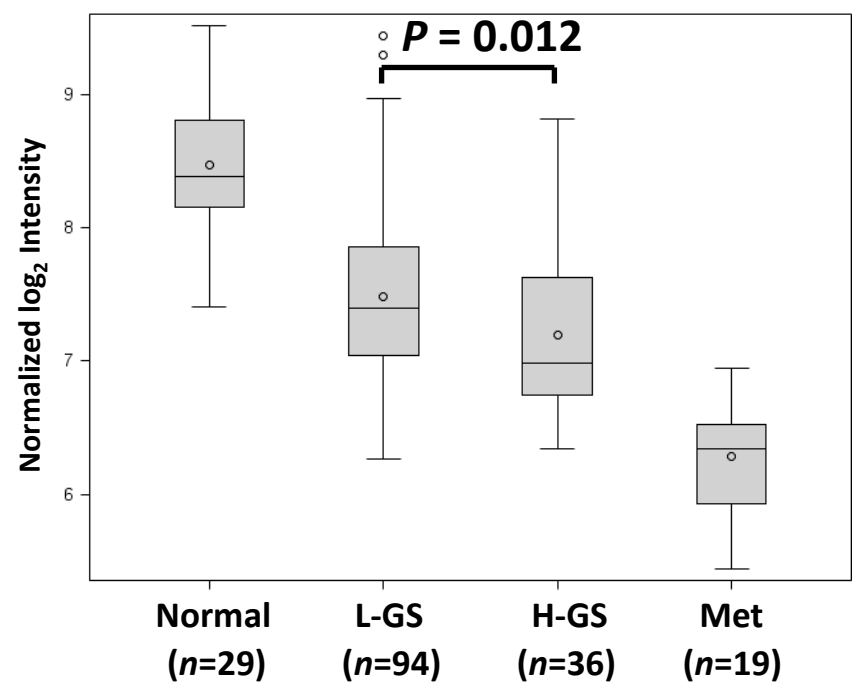

**B**

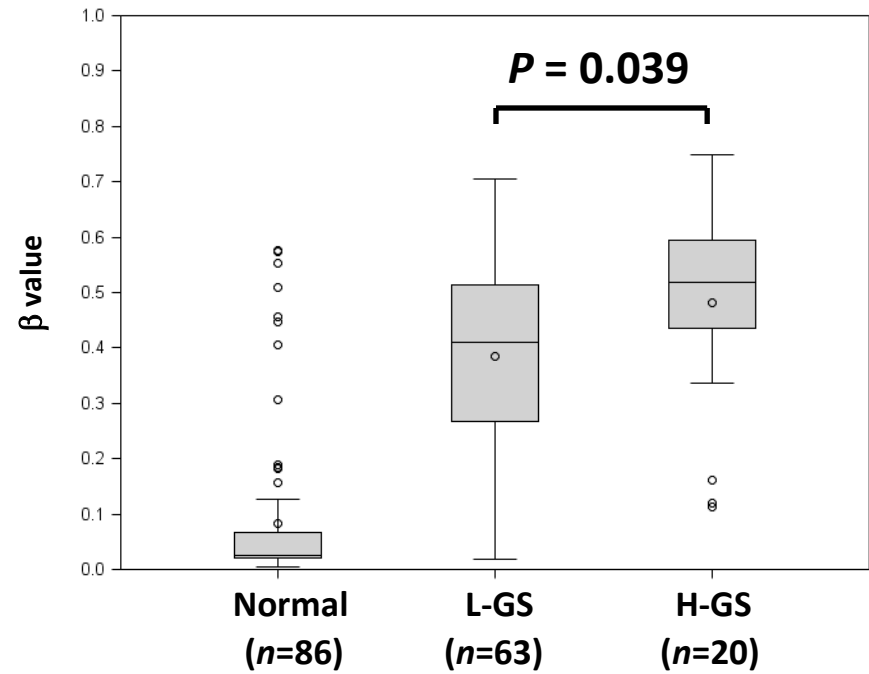

Supplement: Figure S5 — Association of AOX1 gene expression (A) or DNA methylation (B) with Gleason score in PCa. A. Box plot of AOX1 expression in PCa samples. The labels L-GS, H-GS, and Met indicate lower Gleason PCa, higher Gleason PCa, and metastatic cancer samples, respectively. B. Box plot of AOX1 methylation (cg02144933 of the HM27 data set) in PCa samples. (PDF) [file pone.0048455.s005.pdf]

Figure S6.

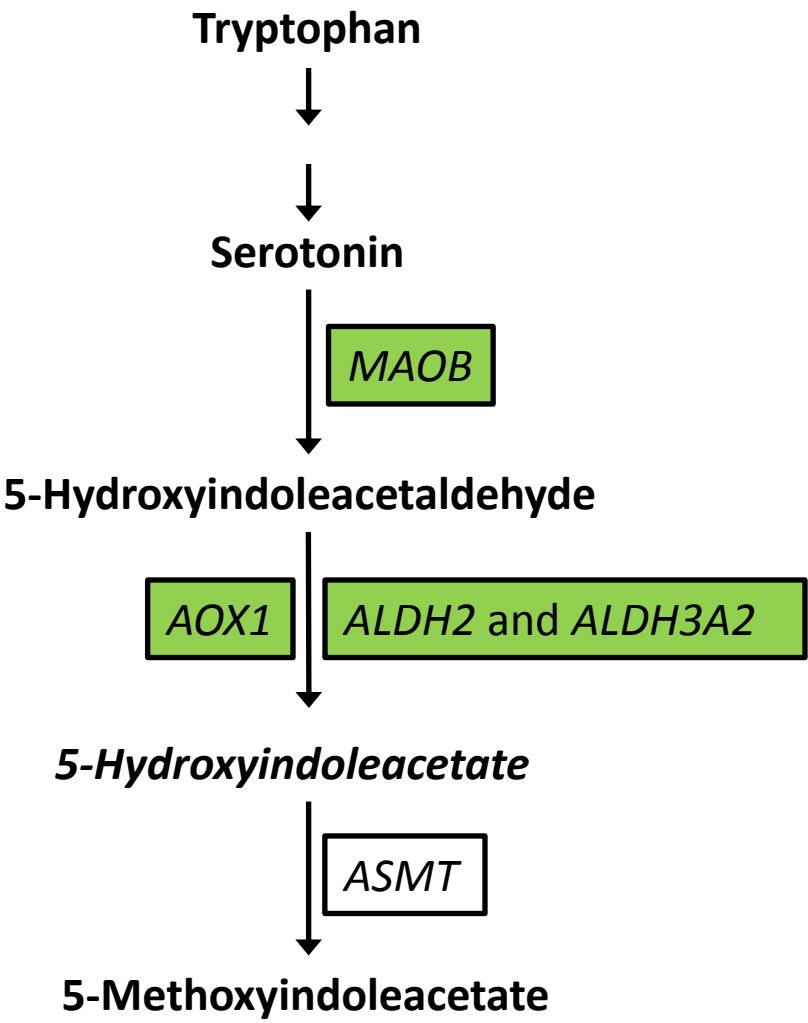

Supplement: Figure S6 — Schematic of enzyme reactions from serotonin to 5-hydroxyindoleacetate in the tryptophan metabolic pathway. Significantly deregulated genes in PCa are highlighted with green color. (PDF) [file pone.0048455.s006.pdf]

Figure S7.

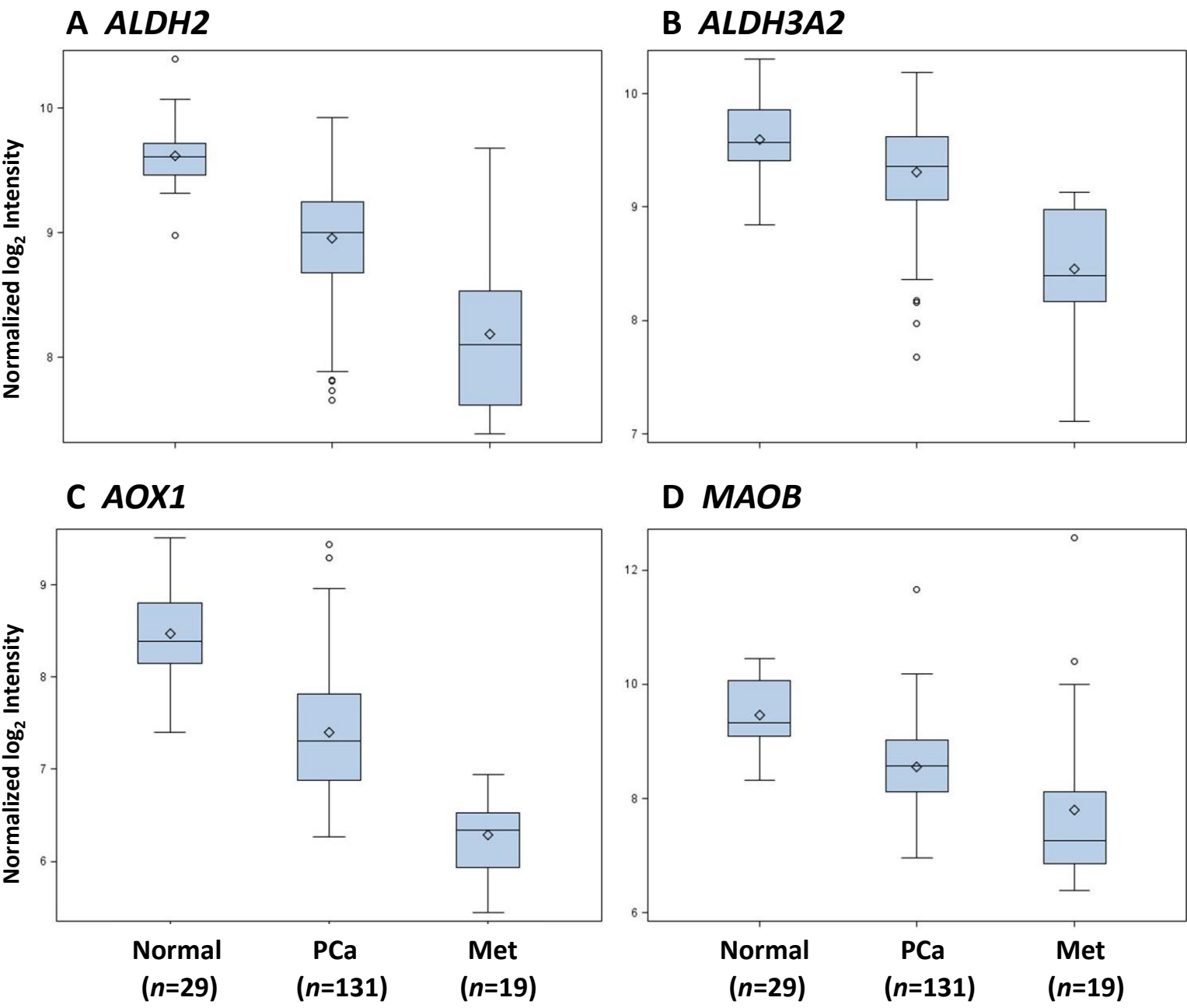

Supplement: Figure S7 — Aberrant expression of tryptophan metabolic pathway genes in PCa. Box plots of ALDH2 (A), ALDH3A2 (B), AOX1 (C), and MAOB (D) gene expression in PCa samples. The labels normal, PCa, and Met indicate adjacent normal prostate tissues, primary prostate cancer samples and metastatic cancer samples, respectively. Comparisons between normal and PCa, PCa and Met in four genes were statistically significant (P<0.05). (PDF) [file pone.0048455.s007.pdf]
