# Supplementary material for: Identification of New Differentially Methylated Genes That Have Potential Functional Consequences in Prostate Cancer
Source: PLoS One. 2012 Oct 31;7(10):e48455. doi: 10.1371/journal.pone.0048455 (PMC3485209; doi:10.1371/journal.pone.0048455)
Supplement: Table S1 — Primer sequences for bisulfite sequencing assay. (PDF) [file pone.0048455.s008.pdf]

Table S1. Primer sequences for bisulfite sequencing assay.

Reaction	Forward primer	Reverse primer	Size of amplicone	Size of sequence reading	Total number of sequenced CpG sites
AOX1	AGTTTTTGGTAAAGAGTTTAGGA	ACCCCTAATAAACAAAAATACTTT	567 bp	400 bp	36
SPON2	AGGTTTATGAATTTGGGTTTAT	CCTACCCAAAACCTAAAAAAT	455 bp	400 bp	27

All primer are 5' →3' oriented.
