# Supplementary material for: Identification of New Differentially Methylated Genes That Have Potential Functional Consequences in Prostate Cancer
Source: PLoS One. 2012 Oct 31;7(10):e48455. doi: 10.1371/journal.pone.0048455 (PMC3485209; doi:10.1371/journal.pone.0048455)
Supplement: Table S2 — Clinicopathological information of tissue samples. A. Inforamtion of the HM450 profiling samples. B. Inforamtion of the bisulfite sequencing samples. (PDF) [file pone.0048455.s009.pdf]

Table S2. Clinicopathological information of tissue samples.

A. Inforamtion of the HM450 profiling samples.

	PCa	Normal
Number of samples	19	4
Age (mean±S.D)	65.8±4.5	68.5±4.4
Gleason score		
6	3 (15.8)	-
3+4	5 (26.3)	-
4+3	3 (15.8)	-
9	3 (15.8)	-
10	1 (5.3)	-
n.a.	4 (21.1)	-
BCR		
Yes	10 (52.6)	-
No	7 (36.8)	-
n.a	2 (10.5)	-
TNM		
T2a	1 (5.3)	-
T2b	5 (26.3)	-
T2c	1 (5.3)	-
T3a	4 (21.1)	-
T3b	4 (21.1)	-
Tx	2 (10.5)	-
n.a.	2 (10.5)	-

Value in parentheses indicates the percentage of cases presenting the group.
n.a.: not available.
BCR: biochemical recurrence

B. Inforamtion of the bisulfite sequencing samples.

	PCa	Normal
Number of samples	56	55
Age (mean±S.D)	59.8±6.3	59.7±6.5
Race		
European American	49 (87.5)	47 (85.5)
African American	4 (7.1)	4 (7.3)
Other	-	1 (1.8)
n.a.	3 (5.4)	3 (5.5)
Gleason score		
5	1 (1.8)	-
6	9 (16.1)	-
3+4	18 (32.1)	-
4+3	7 (12.5)	-
8	5 (8.9)	-
9	14 (25.0)	-
10	1 (1.8)	-
n.a.	1 (1.8)	-
TNM		
T2	5 (8.9)	-
T3a	22 (39.3)	-
T3b	22 (39.3)	-
n.a.	7 (12.5)	-
N0	36 (64.3)	-
N1	13 (23.2)	-
n.a.	7 (12.5)	-

Value in parentheses indicates the percentage of cases presenting the group.
n.a.: not available.
