# Supplementary material for: Identification of New Differentially Methylated Genes That Have Potential Functional Consequences in Prostate Cancer
Source: PLoS One. 2012 Oct 31;7(10):e48455. doi: 10.1371/journal.pone.0048455 (PMC3485209; doi:10.1371/journal.pone.0048455)
Supplement: Table S3 — List of DMCs from HM450 data. (PDF) [file pone.0048455.s010.pdf]

Table S3. List of DMCs from HM450 data.

Taeget_ID	chr	locus	FDR *P*	Δβ∗	Δβ∗ of paired samples	HM27[#]_FDR *P*	HM27[#]_Δβ∗	SNP locus	Gene Symbol	Genomic region
cg12539796	5	7,850,203	4.82E-43	0.697	0.704				*C5orf49*	Body
cg14117138	19	46,800,085	1.11E-62	0.650	0.650				*HIF3A*	TSS1500
cg03596016	1	246,952,362	4.41E-71	0.635	0.611				*LOC149134*	TSS1500
cg17355294	19	51,416,098	4.84E-32	0.614	0.621					
cg26388816	12	570,155	6.49E-38	0.612	0.577				*B4GALNT3*	Body
cg16596604	16	21,294,947	2.22E-38	0.605	0.602				*CRYM*	5'UTR
cg22473620	19	46,379,929	9.64E-63	0.603	0.583					
cg00817367	12	52,401,214	5.25E-77	0.603	0.586				*GRASP*	Body
cg11863717	10	73,725,041	7.77E-16	0.598	0.615				*CHST3*	5'UTR
cg22260952	12	104,852,446	2.1E-36	0.597	0.565				*CHST11*	Body
cg24033558	15	45,479,755	1.06E-46	0.596	0.565				*SHF*	Body
cg26786980	3	25,469,872	2.72E-43	0.596	0.601				*RARB*	5'UTR
cg01030534	7	143,579,698	2.64E-72	0.594	0.597				*FAM115A*	5'UTR
cg08901662	2	131,595,321	1.12E-59	0.590	0.542					
cg04223671	1	201,509,316	3.32E-10	0.590	0.512					
cg03514404	7	143,579,665	8.21E-45	0.588	0.571				*FAM115A*	5'UTR
cg18525352	12	106,533,867	1.28E-52	0.585	0.573				*NUAK1*	TSS200
cg23855505	12	104,852,439	1.19E-65	0.585	0.556				*CHST11*	Body
cg10069493	10	45,914,688	4.86E-22	0.584	0.540				*ALOX5*	Body
cg00675569	10	45,914,840	1.91E-30	0.578	0.566				*ALOX5*	Body
cg13506653	4	54,965,863	5.48E-06	0.578	0.633				*GSX2*	TSS1500
cg05415131	11	58,940,866	5.79E-74	0.578	0.575				*DTX4*	Body
cg20601919	2	219,646,831	0.001593	0.577	0.407				*CYP27A1*	5'UTR
cg18856388	5	1,875,886	3.42E-17	0.576	0.538					
cg04497116	6	1,625,574	1.37E-13	0.575	0.546				*GMDS*	Body
cg05618150	Y	7,140,718	9.57E-24	0.575	0.569				*PRKY*	TSS1500
cg23241781	14	70,653,964	1.76E-41	0.574	0.578				*SLC8A3*	5'UTR
cg24396624	3	25,469,860	3.55E-58	0.573	0.565				*RARB*	5'UTR
cg20883831	7	150,652,948	3.61E-22	0.572	0.564				*KCNH2*	TSS200
cg25737323	2	29,338,100	1.13E-06	0.572	0.440				*CLIP4*	TSS1500
cg05064925	X	107,681,469	3.3E-46	0.572	0.566				*COL4A6*	1stExon

cg05306745	17	76,921,845	2.67E-35	0.571	0.539				*TIMP2*	TSS1500
cg03225210	7	143,579,951	3.57E-46	0.569	0.580				*FAM115A*	5'UTR
cg09296001	7	127,672,564	2.6E-115	0.569	0.561				*SND1*	Body
cg01710865	1	48,937,404	1.8E-07	0.568	0.440				*SPATA6*	Body
cg19897330	5	180,018,201	6.51E-25	0.568	0.575				*SCGB3A1*	Body
cg13132370	17	46,830,260	1.98E-60	0.567	0.573					
cg14931854	6	42,738,967	3.69E-19	0.567	0.533					
cg27151711	X	47,441,773	0.000218	0.566	0.526				*SYN1*	Body
cg11450947	X	106,750,342	1.54E-09	0.562	0.598					
cg26537639	16	88,717,374	1.79E-51	0.561	0.539	2.82E-33	0.357		*CYBA*	1stExon
cg12507655	7	100,465,051	1.18E-50	0.561	0.549				*TRIP6*	1stExon
cg05270634	17	41,177,445	1.48E-34	0.560	0.538	4.72E-28	0.379		*RND2*	1stExon
cg21914941	7	158,261,145	6.34E-26	0.559	0.565				*PTPRN2*	Body
cg24208826	12	54,389,891	3.96E-07	0.558	0.482					
cg15236541	X	73,642,618	1.29E-30	0.557	0.515				*SLC16A2*	Body
cg24452181	19	48,984,318	1.35E-28	0.554	0.572				*CYTH2*	3'UTR
cg16277128	1	146,556,479	4.3E-09	0.554	0.524					
cg04194674	17	36,715,953	7.75E-10	0.553	0.461				*SRCIN1*	Body
cg27493151	19	38,701,994	3.11E-12	0.553	0.607				*DPF1*	3'UTR
cg03787864	16	88,717,134	1.36E-08	0.552	0.489				*CYBA*	Body
cg14904662	8	41,655,673	1E-14	0.552	0.481				*ANK1*	TSS1500
cg05769889	12	89,744,701	1.4E-15	0.550	0.493				*DUSP6*	Body
cg14936968	3	57,198,533	7E-114	0.550	0.527				*IL17RD*	Body
cg08843517	16	88,717,464	8.38E-52	0.548	0.532				*CYBA*	TSS200
cg06484514	21	46,824,278	1E-23	0.547	0.475				*COL18A1*	TSS1500
cg18582992	15	45,479,642	4.6E-71	0.547	0.552				*SHF*	Body
cg05127821	10	94,822,908	5.39E-31	0.545	0.525				*CYP26C1*	Body
cg24180402	17	43,221,464	2.95E-08	0.542	0.426				*ACBD4*	3'UTR
cg11716106	19	46,380,134	3.08E-82	0.542	0.524					
cg18222083	17	41,364,007	1.97E-13	0.542	0.557				*TMEM106A*	5'UTR
cg04879832	16	88,717,456	1.03E-92	0.540	0.521				*CYBA*	1stExon
cg09329826	X	153,094,594	4.81E-29	0.538	0.487				*PDZD4*	Body
cg15931205	6	29,894,820	2.85E-08	0.538	0.544				*HCG4P6*	TSS1500
cg22237200	17	7,555,298	7.56E-25	0.538	0.514				*ATP1B2*	Body

cg07302069	7	27,196,286	1.44E-05	0.538	0.547				*HOXA7*	5'UTR
cg01934626	5	180,018,562	7.2E-117	0.538	0.534				*SCGB3A1*	TSS200
cg02245020	7	143,580,147	4.05E-38	0.538	0.537				*FAM115A*	5'UTR
cg06248767	8	56,852,290	2.45E-26	0.537	0.517				*LYN*	5'UTR
cg17498803	8	61,822,428	4.56E-17	0.536	0.539					
cg20678442	1	244,893,503	7.17E-06	0.536	0.482					
cg24922143	15	35,014,270	1.93E-59	0.536	0.505					
cg13257636	2	29,338,109	3.77E-08	0.535	0.413				*CLIP4*	TSS200
cg23428985	2	29,338,113	1.87E-06	0.534	0.408				*CLIP4*	TSS200
cg02027945	11	20,618,230	8.04E-05	0.534	0.480					
cg12222949	1	184,005,360	4.3E-24	0.533	0.458				*GLT25D2*	Body
cg00650640	X	13,587,678	7.55E-18	0.532	0.564				*EGFL6*	TSS200
cg22771999	X	64,888,437	1.5E-36	0.532	0.519				*MSN*	Body
cg24940138	17	41,363,741	1.65E-18	0.532	0.481				*TMEM106A*	TSS200
cg23697546	12	54,424,085	1.47E-06	0.531	0.434				*HOXC4*	5'UTR
cg19156875	17	43,974,446	1.51E-31	0.531	0.496				*MAPT*	5'UTR
cg24275475	X	107,681,397	1.22E-29	0.531	0.494				*COL4A6*	Body
cg16755500	17	27,918,338	2.7E-71	0.531	0.528					
cg21523564	15	75,251,491	1.15E-11	0.531	0.478					
cg22524657	1	47,999,163	0.000392	0.530	0.494					
cg03069204	7	75,535,491	9.34E-27	0.530	0.541					
cg16876647	2	43,451,842	1.12E-05	0.530	0.485				*ZFP36L2*	Body
cg09489281	5	43,604,149	0.001989	0.528	0.274				*NNT*	5'UTR
cg10162691	17	41,177,409	4.33E-20	0.528	0.493				*RND2*	1stExon
cg07519235	16	19,894,978	6.56E-34	0.528	0.497				*GPRC5B*	5'UTR
cg17397631	4	779,880	1.6E-102	0.527	0.525				*CPLX1*	3'UTR
cg25363807	1	44,703,923	1.61E-37	0.526	0.513				*ERI3*	Body
cg25726664	7	42,277,807	2.42E-14	0.526	0.471				*GLI3*	TSS1500
cg05923595	1	33,547,713	1.91E-44	0.526	0.506				*ADC*	5'UTR
cg23255835	2	29,338,121	4.45E-07	0.526	0.406				*CLIP4*	TSS200
cg06282270	11	61,062,910	6.88E-06	0.525	0.398				*VWCE*	TSS200
cg20771240	11	133,906,693	6.5E-84	0.525	0.524				*LOC100128239*	Body
cg18423852	7	150,653,001	1.22E-23	0.524	0.479				*KCNH2*	TSS200
cg08243728	19	45,905,890	4.37E-55	0.524	0.515				*PPP1R13L*	5'UTR

cg22859061	5	180,018,503	2.1E-131	0.524	0.518				*SCGB3A1*	TSS200
cg23179456	14	24,803,873	0.000836	0.524	0.557				*ADCY4*	TSS200
cg09827752	1	2,461,823	2.85E-39	0.524	0.512				*HES5*	TSS200
cg25170591	10	103,881,062	7.8E-26	0.523	0.524				*LDB1*	TSS1500
cg22747076	12	54,447,873	6.78E-09	0.523	0.501				*HOXC4*	1stExon
cg13555354	14	24,458,014	1.88E-26	0.522	0.516				*DHRS4L2*	TSS200
cg00002719	1	169,396,706	2.91E-06	0.522	0.476				*C1orf114*	TSS200
cg19505129	X	56,258,695	1.05E-10	0.522	0.476				*KLF8*	TSS200
cg16786640	4	3,485,263	1.07E-12	0.521	0.484				*DOK7*	Body
cg13875120	2	201,450,743	0.002612	0.520	0.494				*AOX1*	1stExon
cg03900851	8	67,351,768	1.01E-05	0.520	0.533				*ADHFE1*	Body
cg00800229	14	22,004,993	1.33E-32	0.520	0.501				*SALL2*	1stExon
cg06331595	7	141,211,956	1.1E-69	0.519	0.517					
cg20527270	8	49,468,688	1.18E-14	0.519	0.490					
cg01089498	4	11,428,985	4.48E-62	0.519	0.508				*HS3ST1*	5'UTR
cg08350814	2	191,045,309	1.13E-07	0.519	0.449				*C2orf88*	TSS1500
cg00100121	1	169,396,635	0.000593	0.518	0.483				*C1orf114*	1stExon
cg07330230	3	125,899,925	8.24E-07	0.518	0.453				*ALDH1L1*	TSS1500
cg08300419	11	47,208,959	1.2E-129	0.518	0.519				*PACSIN3*	TSS1500
cg22221554	X	129,245,238	8.8E-09	0.517	0.412				*ELF4*	TSS1500
cg21623671	5	150,536,851	6.35E-20	0.517	0.443	1.33E-30	0.289		*ANXA6*	5'UTR
cg09060908	1	223,743,126	6.81E-47	0.517	0.508				*CAPN8*	Body
cg09908110	11	63,828,713	1.75E-26	0.517	0.517				*MACROD1*	Body
cg03065447	8	49,292,486	0.000133	0.517	0.458					
cg11644815	17	4,891,687	4.26E-12	0.516	0.409				*INCA1*	3'UTR
cg06733794	10	123,922,970	8.73E-44	0.515	0.500				*TACC2*	Body
cg01451391	17	53,343,561	6.99E-21	0.513	0.492				*HLF*	Body
cg04262428	11	70,673,256	5.08E-36	0.513	0.487				*SHANK2*	Body
cg22352818	2	97,193,289	2.37E-68	0.512	0.488					
cg07902749	2	37,813,553	7.3E-17	0.512	0.536					
cg26676468	5	112,539,223	1.74E-09	0.512	0.485				*MCC*	Body
cg01643123	12	63,207,179	6.82E-13	0.511	0.498				*PPM1H*	Body
cg09994356	20	42,875,779	8.51E-06	0.511	0.361				*GDAP1L1*	TSS200
cg15680720	2	171,568,412	0.011376	0.509	0.351					

cg24651977	4	37,978,758	4.58E-49	0.509	0.500				*TBC1D1*	Body
cg19310908	19	46,800,064	1.37E-35	0.509	0.509				*HIF3A*	TSS1500
cg09696939	10	60,272,079	1.19E-06	0.509	0.406				*BICC1*	TSS1500
cg24331301	10	73,156,377	9.79E-45	0.509	0.479				*CDH23*	TSS1500
cg04034767	12	52,400,907	2.16E-09	0.508	0.462	1.35E-25	0.331		*GRASP*	1stExon
cg23108709	2	219,847,136	2.47E-71	0.508	0.510				*FEV*	Body
cg00857907	2	202,896,898	9.05E-16	0.507	0.498					
cg24046411	16	88,717,646	6.02E-32	0.505	0.475				*CYBA*	TSS200
cg00971050	17	40,573,764	2.18E-92	0.505	0.512				*PTRF*	Body
cg18991611	8	49,468,828	1.17E-05	0.504	0.484					
cg21330423	6	29,975,068	1.44E-08	0.504	0.496				*HLA-J*	Body
cg24830730	7	150,653,195	1.07E-36	0.504	0.499				*KCNH2*	Body
cg15313226	2	191,045,668	4.75E-09	0.503	0.434				*C2orf88*	5'UTR
cg00970396	16	9,107,199	3.2E-48	0.503	0.493					
cg22774088	19	2,253,398	1.56E-31	0.502	0.478				*JSRP1*	Body
cg23013309	12	65,218,841	1.01E-34	0.502	0.471					
cg23367107	12	115,136,287	1.9E-21	0.502	0.495					
cg06457357	X	56,259,243	2.55E-21	0.502	0.458	8.15E-24	0.284		*KLF8*	5'UTR
cg00349607	1	39,546,688	3.59E-06	0.501	0.377				*MACF1*	TSS1500
cg06966660	10	123,923,066	3.84E-19	0.501	0.502				*TACC2*	Body
cg07198194	10	3,109,053	1.81E-30	0.500	0.503				*PFKP*	TSS1500
cg14619259	5	141,705,539	1.06E-17	0.500	0.409				*SPRY4*	TSS1500
cg23095615	6	146,136,563	9.34E-08	0.500	0.373				*FBXO30*	TSS1500
cg04294888	22	28,839,249	1.32E-11	0.500	0.431				*TTC28*	Body
cg25274503	7	116,140,128	1.76E-24	0.500	0.493				*CAV2*	Body
cg21899596	4	57,522,493	2.76E-18	0.500	0.503				*HOPX*	1stExon
cg24605338	X	17,395,856	5.53E-78	0.499	0.496				*NHS*	Body
cg27062326	X	117,629,163	5.69E-20	0.499	0.507				*DOCK11*	TSS1500
cg22074576	11	3,181,726	2.44E-21	0.499	0.460				*OSBPL5*	5'UTR
cg00054525	16	88,717,587	7.35E-37	0.499	0.484				*CYBA*	TSS200
cg01272707	6	26,172,185	0.001258	0.499	0.567					
cg11022432	3	125,899,878	1.51E-63	0.499	0.460				*ALDH1L1*	TSS1500
cg18844382	14	23,834,977	4.07E-42	0.498	0.458				*EFS*	TSS200
cg20118643	15	60,690,852	4.52E-51	0.498	0.460				*ANXA2*	TSS1500

cg05304177	3	134,083,080	1.3E-23	0.498	0.528				*AMOTL2*	Body
cg02242344	2	85,640,943	8.35E-13	0.498	0.450					
cg02268650	X	68,348,465	3.89E-08	0.497	0.552					
cg22796860	11	63,828,346	3.39E-75	0.497	0.510				*MACROD1*	Body
cg17755964	1	2,461,834	1.52E-37	0.496	0.477				*HES5*	TSS200
cg02590401	19	48,984,071	2.89E-34	0.496	0.500				*CYTH2*	3'UTR
cg08535928	1	246,952,495	8.2E-156	0.496	0.474				*LOC149134*	TSS1500
cg01837657	15	90,039,822	5.18E-26	0.496	0.504				*RHCG*	TSS200
cg04528060	4	57,371,868	3.21E-07	0.495	0.513				*ARL9*	5'UTR
cg21883802	14	92,413,706	1.11E-09	0.495	0.511				*FBLN5*	5'UTR
cg18516619	17	78,806,891	5.92E-26	0.495	0.489				*RPTOR*	Body
cg04382470	17	43,974,975	2.67E-46	0.495	0.460				*MAPT*	5'UTR
cg16332610	15	74,658,547	9.95E-18	0.494	0.454				*CYP11A1*	1stExon
cg20430847	1	2,461,820	1.09E-22	0.494	0.474				*HES5*	TSS200
cg05269938	20	62,461,294	8.7E-37	0.494	0.504					
cg08193650	2	43,451,868	5.16E-08	0.493	0.478				*ZFP36L2*	Body
cg19548479	17	41,363,737	1.19E-18	0.493	0.468				*TMEM106A*	TSS200
cg09195491	X	56,258,900	8.29E-13	0.493	0.440				*KLF8*	5'UTR
cg19033654	6	30,711,138	5.31E-24	0.492	0.489				*IER3*	3'UTR
cg20172627	2	25,439,110	5.91E-11	0.492	0.541					
cg08825929	3	193,776,240	4.37E-08	0.492	0.434					
cg23674344	17	48,503,163	8.1E-44	0.492	0.446				*ACSF2*	TSS1500
cg21013866	14	23,834,985	6.06E-48	0.492	0.456				*EFS*	TSS200
cg13365334	X	153,598,742	3.18E-33	0.492	0.436				*FLNA*	Body
cg10246531	16	88,717,628	4.01E-41	0.491	0.475				*CYBA*	TSS200
cg13546240	19	46,379,991	3.46E-23	0.491	0.478					
cg14950169	12	54,145,841	1.02E-36	0.491	0.475					
cg25858132	17	1,808,671	8.73E-25	0.491	0.427					
cg17231999	4	41,882,564	6.81E-05	0.490	0.456					
cg11318054	7	157,361,692	1.85E-30	0.490	0.481				*PTPRN2*	Body
cg05693611	6	150,263,722	4.16E-10	0.490	0.498				*ULBP2*	Body
cg09974990	16	67,204,447	2.76E-05	0.490	0.534					
cg12106728	12	115,136,258	1.66E-13	0.490	0.486					
cg03764259	7	127,176,905	5.6E-05	0.490	0.451					

cg14018731	9	98,784,336	1.47E-05	0.490	0.316				*NCRNA00092*	TSS1500
cg15083233	9	112,810,402	4.52E-25	0.489	0.468	1.37E-17	0.197		*PALM2-AKAP2*	Body
cg08062049	2	121,427,380	3.85E-09	0.489	0.458					
cg22078451	5	58,335,681	6.89E-22	0.489	0.425				*PDE4D*	Body
cg00409000	X	17,395,816	5.99E-09	0.489	0.516				*NHS*	Body
cg08360726	19	40,871,823	0.000772	0.488	0.515				*PLD3*	5'UTR
cg00677574	17	43,221,327	1.9E-35	0.488	0.449				*ACBD4*	3'UTR
cg06817490	17	55,888,548	2.99E-25	0.488	0.505					
cg00389463	19	10,397,620	6.2E-14	0.488	0.526				*ICAM4*	TSS200
cg03306374	16	23,847,325	1.02E-11	0.488	0.471				*PRKCB*	1stExon
cg01364755	10	1,370,694	7.89E-12	0.488	0.439				*ADARB2*	Body
cg04194494	10	45,914,450	1.17E-17	0.487	0.443				*ALOX5*	Body
cg09038676	11	67,351,608	2.68E-30	0.487	0.476	3E-29	0.283		*GSTP1*	Body
cg22590258	17	61,615,739	1.1E-68	0.486	0.460			Yes	*KCNH6*	Body
cg06464594	18	59,560,191	1.37E-09	0.486	0.387				*RNF152*	1stExon
cg02423318	7	65,509,125	0.001965	0.486	0.433					
cg16647921	4	41,867,533	2.15E-05	0.486	0.481					
cg24029517	4	11,429,531	5.62E-68	0.486	0.502				*HS3ST1*	5'UTR
cg19023309	X	106,750,168	3.51E-31	0.486	0.503					
cg04715503	16	3,062,795	4.28E-15	0.486	0.485				*CLDN9*	1stExon
cg14993276	19	10,397,642	5.52E-27	0.486	0.502				*ICAM4*	TSS200
cg17629929	5	1,875,548	9.72E-24	0.485	0.452					
cg13123964	8	38,323,238	9.04E-11	0.485	0.471				*FGFR1*	5'UTR
cg00264441	11	57,250,469	2.15E-41	0.485	0.480					
cg27422872	22	28,839,137	3.18E-12	0.485	0.426				*TTC28*	Body
cg21981270	10	45,914,525	1.27E-16	0.485	0.440				*ALOX5*	Body
cg17172980	3	196,755,973	2.8E-15	0.485	0.438				*MFI2*	Body
cg08848774	17	43,047,733	0.000525	0.484	0.371					
cg21526205	1	76,540,574	1.78E-20	0.484	0.459				*ST6GALNAC3*	Body
cg12628196	7	127,672,458	9.2E-193	0.484	0.485				*SND1*	Body
cg06333058	2	29,338,077	2.26E-07	0.484	0.365				*CLIP4*	TSS1500
cg19513834	2	105,470,561	4.8E-06	0.484	0.472				*POU3F3*	TSS1500
cg12799885	10	74,020,976	2E-08	0.483	0.490					
cg08781325	2	26,521,777	1.07E-24	0.483	0.480					

cg01336390	6	29,895,059	9.85E-13	0.483	0.466					
cg14151783	20	44,098,409	3.53E-07	0.483	0.351				*WFDC2*	1stExon
cg06679087	10	99,473,206	8.78E-13	0.483	0.409				*MARVELD1*	TSS1500
cg16831085	10	45,914,645	1.37E-33	0.482	0.448				*ALOX5*	Body
cg21249376	9	27,528,432	3.03E-84	0.482	0.462				*MOBKL2B*	5'UTR
cg23997405	14	101,033,689	8.18E-15	0.482	0.463				*BEGAIN*	Body
cg06641285	17	76,921,829	3.4E-15	0.482	0.479				*TIMP2*	TSS1500
cg16771578	3	125,899,895	1.85E-26	0.482	0.433				*ALDH1L1*	TSS1500
cg14820199	4	11,429,415	6.13E-26	0.482	0.502				*HS3ST1*	5'UTR
cg08478709	3	193,776,092	3.54E-27	0.481	0.469					
cg20801007	11	830,320	6.76E-06	0.481	0.528				*EFCAB4A*	Body
cg22671410	6	29,895,031	2.55E-05	0.481	0.400					
cg03604011	5	400,201	9.16E-06	0.481	0.427				*AHRR*	Body
cg05098590	10	111,767,379	4.45E-05	0.480	0.370				*ADD3*	5'UTR
cg10292154	16	27,461,445	9.66E-20	0.480	0.476				*IL21R*	3'UTR
cg06385187	20	44,098,287	7.35E-05	0.480	0.317				*WFDC2*	TSS200
cg02131967	17	61,554,106	2.05E-30	0.480	0.444	3.73E-25	0.274		*ACE*	TSS1500
cg25032595	13	96,204,978	1.18E-19	0.480	0.471				*CLDN10*	5'UTR
cg04978107	14	24,458,000	4.3E-105	0.480	0.475				*DHRS4L2*	TSS200
cg00604840	5	154,230,173	1.61E-34	0.479	0.454				*C5orf4*	5'UTR
cg09486902	12	65,219,478	5.5E-99	0.479	0.470					
cg21229570	12	115,124,768	9.19E-10	0.479	0.440					
cg04920951	11	67,351,271	1.04E-23	0.479	0.493	3.61E-28	0.309		*GSTP1*	1stExon
cg19859290	11	20,618,399	6.1E-06	0.479	0.324					
cg02534163	8	120,650,994	0.000167	0.479	0.462				*ENPP2*	1stExon
cg05971966	5	40,681,137	9.3E-26	0.479	0.493				*PTGER4*	Body
cg21770622	11	46,383,066	5.73E-48	0.478	0.489				*DGKZ*	Body
cg20899354	3	25,469,392	7.08E-17	0.478	0.448				*RARB*	TSS1500
cg15026277	17	41,363,789	1.5E-12	0.478	0.455				*TMEM106A*	TSS200
cg00402172	16	68,118,754	1.91E-94	0.477	0.461				*NFATC3*	TSS1500
cg24691910	12	115,132,460	8.47E-14	0.477	0.484			Yes		
cg07671805	5	74,907,694	2.88E-21	0.477	0.420					
cg01740766	6	44,528,958	9.48E-33	0.477	0.414					
cg05559795	X	56,258,812	1.84E-08	0.476	0.400				*KLF8*	TSS200

cg22262168	9	27,528,999	2.03E-17	0.476	0.434	3.36E-34	0.373		*MOBKL2B*	5'UTR
cg25386707	10	131,748,428	2.03E-22	0.476	0.489				*EBF3*	Body
cg03942855	1	110,210,925	5.59E-38	0.476	0.424				*GSTM2*	Body
cg06766427	17	12,877,546	2.5E-06	0.476	0.319				*RICH2*	Body
cg18188717	5	154,230,223	1.57E-27	0.475	0.464				*C5orf4*	TSS200
cg25214789	5	7,850,070	6.75E-13	0.475	0.476				*C5orf49*	Body
cg05385513	2	56,150,478	3.59E-22	0.475	0.449				*EFEMP1*	TSS200
cg17643864	8	56,852,148	3.34E-46	0.475	0.461				*LYN*	5'UTR
cg03457729	15	89,953,295	7.85E-34	0.474	0.464					
cg05133205	6	32,121,249	1.06E-26	0.474	0.495				*PPT2*	TSS200
cg00040312	12	54,424,679	6.42E-08	0.474	0.415				*HOXC4*	5'UTR
cg13782615	11	4,079,556	1.86E-21	0.474	0.478				*STIM1*	Body
cg13670316	9	78,507,533	5.42E-19	0.474	0.472				*PCSK5*	Body
cg01532436	19	48,983,942	7.56E-20	0.474	0.482				*CYTH2*	3'UTR
cg09248054	1	969,257	6.45E-08	0.474	0.503				*AGRN*	Body
cg11348442	2	220,117,784	1.84E-35	0.473	0.494				*TUBA4B*	TSS200
cg25307318	10	123,923,081	1.9E-30	0.473	0.439				*TACC2*	Body
cg10593416	X	106,750,120	4.04E-25	0.473	0.454					
cg11332236	15	90,039,818	6.6E-21	0.473	0.490				*RHCG*	TSS200
cg22212414	21	34,775,032	0.010507	0.472	0.293				*IFNGR2*	TSS200
cg07188523	6	44,528,793	7.38E-30	0.472	0.453					
cg06361405	10	104,434,010	6.52E-31	0.472	0.456				*ARL3*	3'UTR
cg11916729	5	180,018,564	7.1E-109	0.472	0.469				*SCGB3A1*	TSS200
cg07153010	22	21,312,388	9.36E-14	0.472	0.371					
cg12564567	11	116,371,188	6.32E-11	0.472	0.451					
cg11424456	17	1,131,878	7.72E-17	0.472	0.446					
cg00397479	6	31,831,510	3.53E-47	0.472	0.447				*NEU1*	TSS1500
cg14621217	17	80,944,134	4.8E-26	0.471	0.422				*B3GNTL1*	Body
cg11280661	3	61,549,475	6.02E-47	0.471	0.451			Yes	*PTPRG*	Body
cg06998282	8	120,651,398	1.66E-15	0.471	0.466				*ENPP2*	TSS1500
cg10706230	1	25,944,514	6.5E-13	0.471	0.433				*MAN1C1*	1stExon
cg20449590	1	146,551,201	2.25E-20	0.471	0.483					
cg10747118	3	184,243,718	0.001302	0.470	0.312					
cg07635623	6	2,841,875	1E-08	0.470	0.400				*SERPINB1*	5'UTR

cg24008544	17	41,363,899	6.59E-30	0.470	0.446				*TMEM106A*	5'UTR
cg16493531	6	30,711,802	4.81E-15	0.470	0.512				*FLOT1*	TSS1500
cg01940855	12	104,852,355	4.32E-37	0.470	0.426				*CHST11*	Body
cg09691340	17	42,402,882	2.09E-05	0.470	0.432				*SLC25A39*	TSS1500
cg14564616	5	1,876,337	2.33E-12	0.470	0.406					
cg03610137	X	56,259,040	1.62E-11	0.470	0.446				*KLF8*	5'UTR
cg22502206	15	60,690,826	8.52E-43	0.470	0.430				*ANXA2*	TSS1500
cg03103218	11	63,828,672	5.88E-31	0.470	0.453				*MACROD1*	Body
cg19617897	15	71,818,628	7.51E-14	0.469	0.485				*THSD4*	Body
cg02373152	6	52,227,767	3E-22	0.469	0.443				*PAQR8*	5'UTR
cg21211480	17	41,363,774	2.33E-15	0.468	0.436				*TMEM106A*	TSS200
cg26375284	6	29,855,401	3.84E-07	0.468	0.435			Yes	*HLA-H*	Body
cg11146691	12	47,219,737	1.92E-13	0.468	0.438				*SLC38A4*	1stExon
cg08066035	19	16,999,614	5.68E-05	0.468	0.493				*F2RL3*	TSS1500
cg03576469	19	46,917,061	7.15E-23	0.468	0.434				*CCDC8*	TSS200
cg26452868	10	57,387,715	7.56E-07	0.468	0.525					
cg11610350	3	64,253,705	1.73E-55	0.468	0.471					
cg05829338	X	106,750,612	1.24E-18	0.468	0.474					
cg08812897	X	20,286,243	2.7E-101	0.467	0.452				*RPS6KA3*	TSS1500
cg26124016	3	25,469,402	6.81E-25	0.467	0.434	1.61E-27	0.331		*RARB*	TSS1500
cg25587069	5	151,066,484	5E-115	0.467	0.454				*SPARC*	1stExon
cg03004714	18	59,560,365	1.57E-06	0.467	0.361				*RNF152*	TSS200
cg05154698	1	26,202,441	9.59E-05	0.467	0.339					
cg21244846	9	27,529,339	0.000239	0.466	0.321				*MOBKL2B*	5'UTR
cg03125427	16	88,717,482	5.7E-05	0.466	0.493				*CYBA*	TSS200
cg15657686	4	37,978,666	2.72E-42	0.466	0.445				*TBC1D1*	Body
cg27519622	16	3,079,877	7.49E-49	0.466	0.471				*CCDC64B*	Body
cg12162377	2	232,261,007	4.26E-16	0.466	0.461				*B3GNT7*	Body
cg00498024	19	41,934,170	5.52E-16	0.466	0.499				*B3GNT8*	5'UTR
cg27232866	1	43,251,073	2.89E-15	0.466	0.434					
cg08177833	16	88,717,537	1.9E-35	0.466	0.477				*CYBA*	TSS200
cg00672333	17	36,728,456	1.06E-24	0.466	0.420				*SRCIN1*	Body
cg05063999	3	64,253,605	2.37E-36	0.466	0.463					
cg08104202	1	169,396,712	1.29E-08	0.466	0.431				*C1orf114*	TSS200

cg07589773	7	50,343,883	2.72E-43	0.465	0.477				*IKZF1*	TSS1500
cg10155432	7	1,293,933	6.74E-52	0.465	0.455					
cg27071152	7	39,649,443	8.37E-06	0.465	0.411				*LOC646999*	Body
cg18638914	4	779,568	6.57E-37	0.465	0.443				*CPLX1*	3'UTR
cg12080909	17	27,917,982	1.31E-42	0.465	0.461				*GIT1*	TSS1500
cg02383154	17	53,343,283	6.09E-69	0.465	0.437				*HLF*	Body
cg01367393	15	89,438,731	2.5E-10	0.464	0.437				*HAPLN3*	5'UTR
cg07937925	17	15,820,699	3.58E-19	0.464	0.404					
cg04986616	11	63,537,378	1.21E-30	0.464	0.440				*C11orf95*	TSS1500
cg22878441	3	159,756,648	1.7E-17	0.464	0.487					
cg05744675	16	88,717,723	1.42E-23	0.464	0.457				*CYBA*	TSS1500
cg13181974	15	37,389,489	0.000529	0.464	0.583				*MEIS2*	Body
cg25943872	X	73,642,385	9.69E-17	0.463	0.404				*SLC16A2*	Body
cg23818870	1	169,396,785	4.4E-14	0.463	0.442				*C1orf114*	TSS200
cg11026555	12	124,247,223	5.82E-05	0.463	0.246				*DNAH10*	Body
cg00113675	6	29,855,347	1.21E-24	0.463	0.426				*HLA-H*	TSS200
cg16836311	1	25,944,712	2.08E-30	0.463	0.425				*MAN1C1*	1stExon
cg17195771	9	71,789,541	3.53E-43	0.463	0.445				*TJP2*	Body
cg13390867	9	86,717,438	2.97E-13	0.463	0.453					
cg15071854	1	45,792,688	0.000168	0.463	0.462				*HPDL*	1stExon
cg25548414	X	130,192,160	0.000131	0.463	0.427				*FLJ30058*	TSS200
cg22538054	12	95,941,988	8.63E-11	0.462	0.399				*USP44*	5'UTR
cg14287235	14	24,804,339	3.91E-10	0.462	0.485				*ADCY4*	TSS1500
cg14142965	12	104,609,432	2.59E-12	0.462	0.439				*TXNRD1*	TSS200
cg09094393	6	146,136,749	9.12E-08	0.462	0.397				*FBXO30*	TSS1500
cg13387113	17	74,696,737	2.66E-32	0.462	0.430				*MXRA7*	Body
cg22806907	17	40,826,212	7.35E-12	0.462	0.453				*PLEKHH3*	Body
cg17633020	5	58,335,180	2.03E-53	0.462	0.435				*PDE4D*	Body
cg21919857	5	180,018,102	9.44E-11	0.462	0.424				*SCGB3A1*	Body
cg00400221	8	38,323,291	4.02E-10	0.462	0.446				*FGFR1*	5'UTR
cg26654286	17	38,465,510	2.96E-14	0.462	0.467				*RARA*	5'UTR
cg08348496	15	89,438,671	8.59E-05	0.462	0.439	4.68E-21	0.279		*HAPLN3*	5'UTR
cg11444704	5	159,738,916	4.66E-10	0.462	0.456				*CCNJL*	Body
cg04156293	7	100,488,942	4.28E-07	0.462	0.507				*ACHE*	Body

cg24117274	1	21,945,805	5.89E-31	0.462	0.421				*RAP1GAP*	Body
cg16955726	X	134,125,259	7.37E-08	0.462	0.457				*LOC644538*	1stExon
cg22027897	17	60,885,856	2.15E-26	0.462	0.400				*MARCH10*	TSS200
cg19241327	7	157,484,559	4.1E-70	0.462	0.469				*PTPRN2*	Body
cg25714826	16	85,784,062	3.36E-18	0.461	0.374				*C16orf74*	5'UTR
cg11606461	5	112,629,604	0.000305	0.461	0.368				*MCC*	Body
cg24808280	1	169,396,868	8.72E-31	0.461	0.441				*C1orf114*	TSS200
cg01514828	17	36,715,074	2.83E-10	0.461	0.385				*SRCIN1*	Body
cg09777525	17	74,965,708	3.03E-06	0.461	0.310					
cg23083315	11	35,639,656	0.000283	0.461	0.368				*FJX1*	TSS200
cg11739675	12	104,852,151	6.85E-12	0.461	0.400				*CHST11*	Body
cg12070159	8	54,789,325	2.45E-24	0.461	0.439				*RGS20*	Body
cg08498787	11	57,250,780	5.33E-33	0.461	0.458					
cg25247183	22	38,073,049	6.25E-08	0.461	0.463				*LGALS1*	Body
cg06839900	1	2,461,900	4.93E-74	0.461	0.444				*HES5*	TSS1500
cg20399616	12	25,055,967	0.00074	0.461	0.438				*BCAT1*	Body
cg18572413	2	233,741,639	5.85E-29	0.460	0.396					
cg11009590	6	149,082,648	1.57E-26	0.460	0.393				*UST*	Body
cg22758700	7	99,156,126	8.5E-05	0.460	0.334				*ZNF655*	TSS1500
cg08047907	1	169,396,858	1.01E-18	0.460	0.441	3.89E-24	0.247		*C1orf114*	TSS200
cg20384898	6	1,625,111	5.02E-21	0.460	0.444				*GMDS*	Body
cg08763705	5	139,525,657	0.00035	0.460	0.364					
cg16556145	13	96,205,184	1.14E-35	0.460	0.446				*CLDN10*	1stExon
cg04178787	2	123,418,787	3.43E-37	0.460	0.453					
cg05019905	6	29,894,831	9.13E-11	0.460	0.450				*HCG4P6*	TSS1500
cg17395064	12	104,609,476	1.02E-11	0.460	0.437				*TXNRD1*	TSS200
cg25464921	1	169,429,698	7.58E-11	0.460	0.457					
cg07980518	18	59,560,394	1.36E-10	0.460	0.361	1.43E-22	0.201		*RNF152*	TSS200
cg23146663	5	151,066,662	8.76E-27	0.460	0.452				*SPARC*	TSS200
cg00073010	17	17,627,194	9.99E-35	0.459	0.462				*RAI1*	5'UTR
cg04203238	4	16,085,702	1.5E-37	0.459	0.449				*PROM1*	TSS200
cg04573276	11	35,639,632	2.12E-09	0.459	0.374				*FJX1*	TSS200
cg21026663	6	26,189,234	8.22E-05	0.458	0.444	1.46E-19	0.243		*HIST1H4D*	1stExon
cg01834022	4	5,710,411	1.52E-05	0.458	0.360				*EVC2*	5'UTR

cg08640923	2	220,117,945	4.33E-08	0.458	0.486				*TUBA4B*	TSS200
cg18960629	17	36,585,452	2.33E-35	0.458	0.451					
cg12178578	1	45,792,714	2.98E-05	0.458	0.442				*HPDL*	1stExon
cg16215203	14	24,803,917	3.34E-05	0.458	0.468				*ADCY4*	TSS200
cg07434284	4	779,691	2.36E-26	0.458	0.438				*CPLX1*	3'UTR
cg02499249	3	25,469,720	1.49E-41	0.458	0.444	8.17E-28	0.271		*RARB*	TSS200
cg03217795	16	23,847,556	5.49E-18	0.458	0.402				*PRKCB*	1stExon
cg00480331	3	187,455,371	1.3E-12	0.457	0.446				*BCL6*	5'UTR
cg00157477	6	29,855,473	1.66E-25	0.457	0.432				*HLA-H*	Body
cg16999254	10	86,016,931	4.19E-08	0.457	0.387				*RGR*	Body
cg16317273	5	38,258,028	1.69E-06	0.457	0.411				*EGFLAM*	TSS1500
cg03317517	19	20,188,803	7.38E-12	0.456	0.357				*ZNF90*	1stExon
cg15356923	3	68,981,011	1.32E-11	0.456	0.447				*FAM19A4*	5'UTR
cg03959186	2	9,893,528	1.94E-19	0.456	0.412			Yes		
cg14711997	7	150,652,864	6.87E-26	0.456	0.453				*KCNH2*	5'UTR
cg19790294	16	88,717,755	5.95E-38	0.456	0.440	3.96E-41	0.296		*CYBA*	TSS1500
cg26371731	1	156,390,240	4.64E-10	0.455	0.509				*MIR9-1*	TSS200
cg06532037	2	191,045,430	0.000202	0.455	0.439				*C2orf88*	5'UTR
cg18257814	2	55,646,430	4.94E-05	0.455	0.278				*CCDC88A*	5'UTR
cg16419441	8	105,478,683	1.5E-33	0.455	0.460				*DPYS*	Body
cg10187879	6	32,223,208	2.31E-16	0.455	0.453					
cg05613116	2	27,958,722	2.36E-22	0.455	0.385					
cg11660826	6	150,286,508	3.22E-25	0.455	0.424				*ULBP1*	Body
cg25561140	12	120,799,725	3.28E-39	0.455	0.446				*MSI1*	Body
cg05947181	X	50,557,283	9.76E-06	0.455	0.510				*SHROOM4*	TSS1500
cg20060685	12	106,533,898	2.56E-41	0.455	0.454				*NUAK1*	TSS200
cg22003435	2	191,045,885	1.46E-13	0.455	0.380				*C2orf88*	5'UTR
cg27299406	2	43,020,013	1.27E-08	0.455	0.309				*HAAO*	TSS1500
cg06206801	18	24,131,379	7.83E-28	0.454	0.422				*KCTD1*	5'UTR
cg18331855	17	1,131,781	8.71E-13	0.454	0.436					
cg01708411	12	57,631,839	4.01E-11	0.454	0.431				*NDUFA4L2*	5'UTR
cg25258879	X	49,022,996	8.2E-10	0.454	0.391				*MAGIX*	3'UTR
cg00179217	6	31,831,340	6.13E-41	0.454	0.432				*SLC44A4*	3'UTR
cg03344031	19	50,706,387	5.4E-05	0.454	0.290				*MYH14*	TSS1500

cg25556905	14	24,803,925	0.000194	0.454	0.473				*ADCY4*	TSS200
cg02386420	17	78,806,870	7.38E-20	0.454	0.450				*RPTOR*	Body
cg09087503	7	127,672,473	4.4E-117	0.454	0.458				*SND1*	Body
cg10224600	17	43,975,063	1.72E-24	0.454	0.454				*MAPT*	5'UTR
cg10262387	6	29,855,435	6.22E-17	0.453	0.424				*HLA-H*	Body
cg05562381	17	8,230,350	2.31E-07	0.453	0.381					
cg10626816	1	39,025,249	1.13E-06	0.453	0.430					
cg23780110	2	26,045,244	1.87E-23	0.453	0.445				*ASXL2*	Body
cg26071526	10	123,923,273	1.32E-39	0.453	0.447				*TACC2*	Body
cg12479047	3	25,469,573	5.3E-21	0.453	0.421				*RARB*	TSS1500
cg19846667	14	22,191,856	3.23E-38	0.453	0.457					
cg06428055	X	129,243,872	9.54E-17	0.453	0.447	1.56E-31	0.339		*ELF4*	5'UTR
cg25311637	1	146,556,000	3.58E-10	0.453	0.391			Yes		
cg11448068	2	191,045,026	4.14E-44	0.453	0.445				*C2orf88*	TSS1500
cg13727849	17	72,210,343	1.25E-26	0.453	0.435				*MGC16275*	TSS1500
cg06405186	11	61,283,968	3.14E-43	0.453	0.438				*SYT7*	3'UTR
cg15472092	7	150,653,079	1.48E-14	0.452	0.423				*KCNH2*	TSS200
cg08513472	6	1,625,238	8.41E-63	0.452	0.433				*GMDS*	Body
cg06301550	20	37,230,484	5.32E-31	0.452	0.449				*C20orf95*	TSS200
cg00778807	1	43,524,347	2.3E-14	0.452	0.466					
cg09953499	19	51,416,202	1.57E-21	0.452	0.411					
cg24519393	1	38,470,540	2.83E-40	0.452	0.400				*FHL3*	5'UTR
cg05402599	2	219,846,796	4.54E-41	0.452	0.447				*FEV*	Body
cg22750044	X	73,642,662	1.88E-21	0.452	0.401				*SLC16A2*	Body
cg24492202	4	2,802,612	2.28E-29	0.452	0.429				*SH3BP2*	5'UTR
cg08979352	4	99,580,530	1.52E-05	0.452	0.488				*TSPAN5*	TSS1500
cg15467646	1	22,141,014	1.48E-61	0.452	0.426				*LDLRAD2*	Body
cg10002178	14	94,254,726	0.013876	0.451	0.197				*PRIMA1*	5'UTR
cg16818740	20	4,803,300	8.21E-45	0.451	0.415				*RASSF2*	5'UTR
cg10286969	16	2,765,843	1.18E-17	0.451	0.452				*PRSS27*	Body
cg04704531	12	54,409,491	2.79E-05	0.451	0.434				*HOXC4*	TSS1500
cg07564962	5	139,076,670	2.82E-53	0.451	0.449					
cg11199810	1	150,123,146	0.00021	0.451	0.313				*PLEKHO1*	Body
cg15266705	12	115,136,096	7.32E-14	0.451	0.441					

cg13150171	1	41,326,892	4.03E-18	0.451	0.453				*CITED4*	3'UTR
cg07906520	20	37,435,716	6.31E-11	0.451	0.423				*PPP1R16B*	5'UTR
cg09841842	14	51,974,848	2.37E-31	0.451	0.464				*FRMD6*	5'UTR
cg06769296	11	133,906,761	1.9E-46	0.450	0.434				*LOC100128239*	Body
cg25191041	21	44,782,470	9.28E-47	0.450	0.432					
cg00722320	15	79,502,065	5.17E-06	0.450	0.463				*MIR184*	TSS200
cg08879559	5	151,066,486	2.09E-15	0.450	0.404				*SPARC*	1stExon
cg10940462	11	64,479,015	1.95E-06	0.450	0.430				*NRXN2*	Body
cg17278864	17	12,877,321	1.98E-05	0.450	0.261				*RICH2*	Body
cg07139301	3	170,746,207	2.22E-08	0.450	0.410				*SLC2A2*	TSS1500
cg09490603	15	74,658,166	1.46E-52	0.450	0.449				*CYP11A1*	1stExon
cg06654691	17	40,825,980	2.5E-109	0.450	0.435				*PLEKHH3*	Body
cg21799346	11	2,905,372	2.52E-06	0.450	0.370				*CDKN1C*	Body
cg16725050	2	220,117,771	4.63E-25	0.450	0.470				*TUBA4B*	TSS200
cg11566244	11	67,351,786	4.39E-28	0.450	0.445				*GSTP1*	Body
cg07795766	22	45,608,516	2.85E-08	0.450	0.413				*C22orf9*	Body
cg22829182	X	56,258,808	7.28E-08	0.450	0.392				*KLF8*	TSS200
cg08163199	6	29,974,858	8.32E-11	0.449	0.455				*HLA-J*	Body
cg14000361	6	10,882,336	4.36E-05	0.449	0.439				*GCM2*	TSS1500
cg02053896	X	70,151,117	6.65E-21	0.449	0.429				*SLC7A3*	TSS200
cg26751972	6	29,692,000	7.01E-10	0.449	0.395				*HLA-F*	Body
cg06540859	X	46,434,696	1.92E-13	0.449	0.322				*CHST7*	1stExon
cg14832998	7	157,530,956	2.09E-18	0.449	0.452				*PTPRN2*	Body
cg13717446	17	62,777,761	6.4E-05	0.449	0.353				*LOC146880*	Body
cg16107322	7	141,211,627	8.77E-77	0.449	0.430					
cg17861863	10	1,454,387	0.000807	0.448	0.210				*ADARB2*	Body
cg22399133	2	208,989,248	1.17E-05	0.448	0.427				*CRYGD*	1stExon
cg18878992	17	43,974,344	6.91E-06	0.448	0.397				*MAPT*	5'UTR
cg00601450	5	74,908,170	5.03E-15	0.447	0.377					
cg27209395	6	26,172,397	2.38E-07	0.447	0.488					
cg04285033	21	46,143,498	6.6E-06	0.447	0.545					
cg18698788	15	89,952,499	6.57E-08	0.447	0.395					
cg09659004	6	29,975,078	7.08E-08	0.447	0.435				*HLA-J*	Body
cg15707833	8	131,455,276	1.86E-06	0.447	0.289					

cg03892356	12	54,420,837	4.15E-10	0.447	0.341				*HOXC4*	5'UTR
cg10592478	12	53,612,641	2.55E-24	0.447	0.461				*RARG*	Body
cg10168635	2	191,044,980	1.65E-29	0.447	0.427				*C2orf88*	TSS1500
cg24816460	6	4,775,203	0.000183	0.447	0.351				*CDYL*	TSS1500
cg27280004	19	2,489,065	2.68E-24	0.447	0.447					
cg20893717	7	100,318,190	0.005109	0.446	0.277				*EPO*	TSS1500
cg18563413	19	51,843,854	5.87E-79	0.446	0.425				*VSIG10L*	Body
cg03276785	7	54,956,202	6.14E-17	0.446	0.449					
cg02090762	17	79,503,859	1.71E-05	0.446	0.418				*FSCN2*	Body
cg00227578	5	34,657,676	1.01E-18	0.446	0.380				*RAI14*	5'UTR
cg09064304	4	141,419,169	1.4E-05	0.446	0.433					
cg12451631	10	43,698,141	1.61E-18	0.446	0.448				*RASGEF1A*	Body
cg16998150	1	169,396,834	9.84E-33	0.446	0.431				*C1orf114*	TSS200
cg19439123	17	17,687,621	4.46E-09	0.446	0.402				*RAI1*	5'UTR
cg09920557	17	61,553,938	2.35E-15	0.445	0.400				*ACE*	TSS1500
cg18108818	7	102,574,475	6.41E-27	0.445	0.445				*FBXL13*	Body
cg20676696	1	110,210,444	9E-24	0.445	0.385				*GSTM2*	TSS200
cg03628719	15	89,438,964	9.8E-31	0.445	0.423				*HAPLN3*	TSS200
cg02532096	11	111,411,289	6.33E-14	0.444	0.453				*LAYN*	5'UTR
cg13975336	16	67,204,216	3.07E-06	0.444	0.488					
cg11859529	X	70,150,922	6.82E-06	0.444	0.417				*SLC7A3*	1stExon
cg20842253	2	27,529,788	1.56E-30	0.444	0.396				*TRIM54*	3'UTR
cg02586610	3	122,745,092	2.61E-12	0.443	0.416				*SEMA5B*	5'UTR
cg13531315	19	41,354,553	2.04E-20	0.443	0.411			Yes	*CYP2A6*	Body
cg02636041	10	43,698,008	1.52E-10	0.443	0.452				*RASGEF1A*	Body
cg06632027	4	90,757,378	4.97E-13	0.443	0.368				*SNCA*	5'UTR
cg16437908	2	85,640,810	2.69E-16	0.443	0.421					
cg03936721	17	42,386,419	1.46E-21	0.443	0.424				*RUNDC3A*	Body
cg08161922	12	2,163,608	0.008228	0.443	0.333				*CACNA1C*	Body
cg14433074	6	30,711,090	1.42E-20	0.443	0.408				*IER3*	3'UTR
cg12513880	12	115,136,298	4.79E-32	0.443	0.433					
cg12763668	1	19,687,257	3.62E-22	0.443	0.445				*CAPZB*	Body
cg21026830	14	60,973,517	5.79E-05	0.443	0.472					
cg21868134	1	45,792,660	0.000172	0.443	0.452				*HPDL*	1stExon

cg26792821	5	113,390,882	1.04E-33	0.443	0.433					
cg16318112	1	969,254	1.15E-07	0.442	0.475				*AGRN*	Body
cg23356769	X	129,245,245	2.17E-14	0.442	0.384				*ELF4*	TSS1500
cg05309989	14	50,527,872	1.28E-29	0.442	0.431					
cg13885357	12	53,184,041	0.000146	0.442	0.543				*KRT3*	Body
cg07249274	5	112,629,565	5.67E-05	0.442	0.352				*MCC*	Body
cg10575376	12	124,246,446	0.000648	0.442	0.201				*DNAH10*	TSS1500
cg15140902	6	21,667,815	1.91E-25	0.442	0.413				*FLJ22536*	Body
cg22097768	17	61,615,913	1.43E-54	0.442	0.428				*KCNH6*	Body
cg14709691	1	46,632,871	2.12E-23	0.442	0.452					
cg16482474	7	151,107,637	2.13E-07	0.442	0.433				*WDR86*	TSS1500
cg14862207	17	36,715,798	0.000317	0.442	0.411				*SRCIN1*	Body
cg09337254	2	85,640,762	2.71E-14	0.442	0.384					
cg05452524	17	53,343,029	0.001418	0.441	0.430				*HLF*	Body
cg09300114	17	73,084,329	2.9E-36	0.441	0.430	7.38E-37	0.274		*SLC16A5*	5'UTR
cg10742957	19	39,466,716	8.86E-17	0.441	0.458				*FBXO17*	TSS1500
cg06805513	X	73,641,955	0.001516	0.441	0.188				*SLC16A2*	Body
cg00742920	13	27,050,512	9.98E-18	0.441	0.452					
cg07016276	6	29,692,009	6.81E-09	0.441	0.420				*HLA-F*	Body
cg08022244	7	157,361,759	4.32E-25	0.441	0.429				*PTPRN2*	Body
cg14371731	10	81,003,175	7.88E-05	0.441	0.408				*ZMIZ1*	Body
cg09556515	16	85,470,485	3.47E-39	0.441	0.418					
cg03995122	6	29,894,642	7.71E-08	0.441	0.426				*HCG4P6*	TSS1500
cg01037637	18	59,560,289	9.15E-13	0.441	0.357				*RNF152*	1stExon
cg13605398	17	7,826,366	0.006105	0.441	0.415				*KCNAB3*	3'UTR
cg26937868	1	26,202,601	1.93E-32	0.441	0.393					
cg13208318	7	54,956,274	6.25E-17	0.441	0.401					
cg24974704	8	144,577,238	4.59E-22	0.441	0.417				*ZC3H3*	Body
cg16100120	2	56,150,475	1.25E-10	0.440	0.401				*EFEMP1*	TSS200
cg13516209	6	29,855,302	1.64E-30	0.440	0.432				*HLA-H*	TSS200
cg07147449	12	115,136,072	1.61E-15	0.440	0.429					
cg15613420	5	134,735,637	1.08E-09	0.440	0.432				*H2AFY*	TSS1500
cg15627710	1	154,972,140	1.2E-07	0.440	0.434					
cg02467990	7	49,813,102	0.001571	0.440	0.533				*VWC2*	TSS200

cg08879910	6	29,974,319	2.3E-09	0.440	0.435				*HLA-J*	Body
cg13261825	10	98,939,742	4.45E-23	0.440	0.413				*SLIT1*	Body
cg06947913	12	50,297,793	0.000508	0.440	0.260				*FAIM2*	TSS200
cg20098471	11	13,032,613	2.25E-06	0.440	0.366				*RASSF10*	Body
cg15407528	6	30,418,985	1.7E-07	0.439	0.509					
cg01552275	7	2,654,530	3.99E-09	0.439	0.365			Yes		
cg11930554	4	74,809,912	0.000186	0.439	0.392					
cg23235241	16	25,704,505	1.83E-11	0.439	0.452				*HS3ST4*	Body
cg10594090	19	46,800,198	1.41E-07	0.439	0.397				*HIF3A*	TSS200
cg16585972	14	23,835,212	4.06E-41	0.439	0.403				*EFS*	TSS1500
cg26704078	18	24,131,115	2.25E-25	0.439	0.412				*KCTD1*	5'UTR
cg02072495	15	60,689,285	4.01E-15	0.439	0.334				*ANXA2*	5'UTR
cg04170192	6	30,418,941	3.43E-11	0.439	0.464					
cg16260298	7	116,140,342	2.27E-20	0.439	0.422				*CAV2*	Body
cg16232979	19	16,187,631	0.009162	0.438	0.415	2.61E-25	0.287		*TPM4*	Body
cg23511432	5	137,610,255	0.002463	0.438	0.393				*GFRA3*	TSS200
cg20003494	4	90,757,398	3.11E-06	0.438	0.362				*SNCA*	5'UTR
cg21098557	5	43,191,993	3.99E-14	0.438	0.340				*MGC42105*	TSS1500
cg14134732	16	3,079,894	2.73E-25	0.438	0.431				*CCDC64B*	Body
cg02879662	19	46,800,467	1.05E-08	0.438	0.422	4.52E-31	0.315		*HIF3A*	Body
cg11871960	14	97,058,777	5E-05	0.438	0.404					
cg11246563	6	29,855,406	4.56E-29	0.438	0.389				*HLA-H*	Body
cg18652900	19	15,580,445	1.46E-22	0.438	0.433				*PGLYRP2*	Body
cg06363129	7	16,505,602	3.88E-22	0.438	0.447	2.99E-10	0.173		*SOSTDC1*	TSS200
cg14482902	17	36,714,803	8.1E-07	0.437	0.280				*SRCIN1*	Body
cg24779587	11	47,209,163	3.67E-29	0.437	0.424				*PACSIN3*	TSS1500
cg26870192	6	137,242,062	7.85E-07	0.437	0.514				*SLC35D3*	TSS1500
cg04791822	X	47,441,573	6.1E-08	0.437	0.423				*SYN1*	Body
cg06655100	X	56,259,373	1.04E-16	0.437	0.437	5.47E-21	0.212		*KLF8*	5'UTR
cg17329164	6	32,121,259	4.15E-22	0.437	0.447	1.15E-28	0.300		*PPT2*	TSS200
cg04505435	11	70,672,511	4.95E-27	0.437	0.418				*SHANK2*	Body
cg09910635	7	97,361,026	8.04E-19	0.437	0.406				*TAC1*	TSS1500
cg19537719	8	41,583,498	2.11E-12	0.437	0.412				*ANK1*	Body
cg27084746	6	31,831,532	4.07E-35	0.437	0.408				*NEU1*	TSS1500

cg15621260	11	27,015,813	1.56E-10	0.437	0.432				*FIBIN*	5'UTR
cg13940444	12	53,617,382	1.8E-22	0.436	0.428				*RARG*	Body
cg14594187	9	124,498,961	4.32E-35	0.436	0.436				*DAB2IP*	Body
cg06969287	22	28,838,631	7.73E-22	0.436	0.418				*TTC28*	Body
cg26591930	11	70,672,986	2.74E-11	0.436	0.389				*SHANK2*	Body
cg19254906	1	43,251,253	3.09E-06	0.436	0.435					
cg14928057	17	4,891,542	7.45E-06	0.436	0.340				*INCA1*	3'UTR
cg07347092	12	124,246,927	0.000672	0.436	0.189				*DNAH10*	TSS200
cg07116010	9	35,646,325	1.27E-06	0.436	0.342					
cg19003815	3	25,469,914	4.1E-32	0.435	0.423				*RARB*	5'UTR
cg24725574	6	29,974,717	8.06E-08	0.435	0.431				*HLA-J*	Body
cg07326438	11	57,194,562	0.000979	0.435	0.371				*SLC43A3*	5'UTR
cg06928838	11	67,351,490	2.31E-43	0.435	0.421				*GSTP1*	Body
cg14283569	19	51,416,213	7.59E-37	0.435	0.418					
cg21601837	3	125,900,065	4.92E-36	0.435	0.404				*ALDH1L1*	TSS1500
cg24716879	1	235,105,872	1.27E-19	0.435	0.369					
cg08244085	12	63,025,730	1.22E-08	0.435	0.351					
cg26625629	2	21,022,877	0.000752	0.435	0.395				*C2orf43*	TSS200
cg07484673	7	99,155,878	7.8E-05	0.435	0.302				*ZNF655*	TSS200
cg08042316	7	139,930,256	0.004902	0.435	0.319					
cg08101303	7	96,652,222	2.62E-08	0.435	0.439				*DLX5*	Body
cg09065876	17	45,949,756	1.28E-09	0.435	0.358					
cg25480336	20	50,720,908	4.89E-08	0.435	0.449				*ZFP64*	Body
cg01146875	3	170,746,172	2.9E-21	0.434	0.411				*SLC2A2*	TSS1500
cg11560280	12	54,089,497	3.32E-06	0.434	0.420					
cg07724670	20	45,440,228	3.82E-24	0.434	0.405					
cg21908235	1	146,551,744	7.39E-46	0.434	0.435					
cg14130039	6	32,121,225	2.49E-13	0.434	0.457				*PPT2*	TSS200
cg05467676	3	72,226,965	9.59E-19	0.434	0.433					
cg06102403	20	30,458,708	7.38E-06	0.434	0.332				*TTLL9*	5'UTR
cg08862890	5	169,064,451	3.18E-06	0.434	0.396				*DOCK2*	Body
cg18726691	15	90,039,613	2.32E-08	0.434	0.449				*RHCG*	1stExon
cg01897797	14	22,005,140	1.37E-14	0.434	0.417				*SALL2*	5'UTR
cg22235821	3	140,796,104	9.03E-07	0.434	0.436				*SPSB4*	Body

cg02460251	5	176,107,427	7.91E-05	0.433	0.399					
cg09856068	5	170,878,246	0.007331	0.433	0.545				*FGF18*	Body
cg18077971	2	223,164,867	3.7E-08	0.433	0.465				*PAX3*	TSS1500
cg20104456	19	51,568,523	5.61E-18	0.433	0.432				*KLK13*	TSS200
cg20424164	12	65,220,294	1.63E-23	0.433	0.384			Yes		
cg05196026	X	137,793,096	7.93E-07	0.433	0.372				*FGF13*	Body
cg18437209	11	35,105,199	1.61E-27	0.433	0.417					
cg08476890	X	138,774,601	2.46E-15	0.433	0.390				*MCF2*	5'UTR
cg18347010	17	55,888,532	3.93E-35	0.433	0.428					
cg04765848	1	53,527,576	1.14E-24	0.433	0.385				*PODN*	TSS1500
cg06359703	1	41,847,297	0.000275	0.432	0.233					
cg00174573	12	125,172,635	6.26E-30	0.432	0.408					
cg14991984	1	99,470,129	1.2E-05	0.432	0.500				*LPPR5*	1stExon
cg17588266	15	74,658,330	6.02E-27	0.432	0.421				*CYP11A1*	1stExon
cg17511968	2	219,721,165	1.07E-22	0.432	0.400					
cg24027937	19	709,809	9.38E-39	0.432	0.394				*PALM*	Body
cg00815013	X	107,681,737	2.84E-22	0.432	0.447				*COL4A5*	TSS1500
cg13958426	1	169,396,637	2.13E-05	0.432	0.419	1.22E-15	0.201		*C1orf114*	1stExon
cg26029345	8	22,960,820	0.009442	0.432	0.308				*TNFRSF10C*	Body
cg00279790	1	46,914,033	5.01E-20	0.431	0.393					
cg11988831	20	4,803,490	9.71E-12	0.431	0.365				*RASSF2*	5'UTR
cg23008871	17	45,949,743	1.38E-08	0.431	0.364					
cg27507687	3	48,632,484	0.02201	0.431	0.442				*COL7A1*	Body
cg08377924	17	41,363,628	7.43E-13	0.431	0.390				*NBR1*	3'UTR
cg20818417	1	76,082,690	2.21E-06	0.431	0.379					
cg10959711	1	153,509,096	4.7E-28	0.431	0.399				*S100A6*	TSS1500
cg19320816	6	26,087,776	0.002615	0.431	0.402				*HFE*	Body
cg22125968	13	43,566,472	0.000129	0.431	0.426				*EPSTI1*	TSS200
cg10024583	17	45,772,800	5.67E-30	0.431	0.418				*TBKBP1*	1stExon
cg15579650	X	101,906,119	0.022849	0.431	0.471				*GPRASP1*	TSS200
cg09907758	11	63,537,459	5.51E-32	0.430	0.423				*C11orf95*	TSS1500
cg15323528	10	73,156,427	6.71E-17	0.430	0.405				*CDH23*	TSS1500
cg00987031	5	140,893,101	7.5E-20	0.430	0.350					
cg10840277	8	124,829,410	1.03E-23	0.430	0.453					

cg23208176	5	1,876,168	3.5E-11	0.430	0.397					
cg18242030	5	58,335,271	5.16E-12	0.430	0.375				*PDE4D*	Body
cg03679305	1	9,129,487	8.29E-15	0.430	0.406	2.51E-23	0.175		*SLC2A5*	Body
cg01579535	12	54,447,292	1.76E-14	0.430	0.392				*HOXC4*	5'UTR
cg16371082	3	134,083,158	1.9E-32	0.430	0.460				*AMOTL2*	Body
cg00930615	15	60,690,667	8.68E-17	0.430	0.374				*ANXA2*	TSS1500
cg01663018	15	53,097,777	0.003508	0.429	0.357					
cg09287459	X	39,548,787	7.78E-06	0.429	0.345					
cg24818939	X	101,906,109	0.022063	0.429	0.481				*GPRASP1*	TSS200
cg04880618	17	74,706,567	0.001609	0.429	0.324				*MXRA7*	Body
cg03949008	X	2,846,196	1.49E-35	0.429	0.399				*ARSD*	Body
cg14206172	19	39,466,757	4.16E-29	0.429	0.431				*FBXO17*	TSS1500
cg01738650	6	32,223,076	1.35E-08	0.429	0.395					
cg18337963	11	46,383,209	2.25E-30	0.429	0.422				*DGKZ*	1stExon
cg00224083	4	139,773,135	5.17E-19	0.429	0.402					
cg10147666	2	219,646,481	0.000841	0.429	0.293				*CYP27A1*	5'UTR
cg06525551	X	137,792,939	6.31E-09	0.429	0.324				*FGF13*	Body
cg06968912	17	40,573,643	7.81E-23	0.429	0.420				*PTRF*	Body
cg10692693	7	101,688,648	1.5E-36	0.429	0.417				*CUX1*	Body
cg11267955	5	101,632,327	2.72E-06	0.429	0.444				*SLCO4C1*	TSS200
cg26360792	2	43,019,997	1.67E-07	0.429	0.263				*HAAO*	TSS1500
cg18856652	2	43,864,913	3.41E-13	0.429	0.381				*PLEKHH2*	5'UTR
cg07219542	1	110,210,770	4.4E-12	0.429	0.368				*GSTM2*	1stExon
cg21041775	12	54,425,418	3.63E-20	0.429	0.383				*HOXC4*	5'UTR
cg25181710	2	26,045,287	6.47E-32	0.429	0.417				*ASXL2*	Body
cg18709349	19	46,999,274	9.31E-06	0.429	0.442				*PNMAL2*	TSS200
cg03126946	12	2,944,310	0.002066	0.428	0.233				*NRIP2*	TSS200
cg19852958	4	13,545,760	3.54E-05	0.428	0.491				*NKX3-2*	1stExon
cg02711647	2	45,237,689	0.000205	0.428	0.301				*SIX2*	TSS1500
cg02156680	8	120,651,317	3.57E-12	0.428	0.431				*ENPP2*	TSS1500
cg14792081	1	65,468,460	0.000622	0.428	0.319					
cg18486102	12	50,297,777	0.003652	0.428	0.244				*FAIM2*	TSS200
cg15267232	10	8,097,689	4.65E-40	0.428	0.396				*GATA3*	Body
cg16927871	3	25,469,991	2.81E-32	0.428	0.410				*RARB*	5'UTR

cg09350274	5	137,610,396	0.001948	0.428	0.358	7.02E-12	0.184			*GFRA3*	TSS200
cg17393917	12	106,533,840	5.75E-25	0.428	0.412					*NUAK1*	TSS200
cg11664500	6	133,562,479	1.88E-06	0.427	0.516					*EYA4*	TSS200
cg09780231	17	873,452	7.64E-24	0.427	0.398					*NXN*	Body
cg09695033	2	29,337,988	5.62E-08	0.427	0.310					*CLIP4*	TSS1500
cg22187722	7	29,185,965	1.78E-09	0.427	0.384					*CPVL*	5'UTR
cg18267506	1	48,937,445	4.88E-07	0.427	0.313					*SPATA6*	Body
cg01893212	7	49,813,088	0.015338	0.427	0.505					*VWC2*	TSS200
cg00236832	17	38,465,489	8.03E-11	0.427	0.440	9.78E-28	0.288			*RARA*	5'UTR
cg12708994	6	116,691,460	1.17E-32	0.427	0.387					*DSE*	TSS1500
cg19561297	7	102,553,457	3.65E-44	0.427	0.404					*LRRC17*	5'UTR
cg06223162	1	101,003,688	1.54E-26	0.427	0.395					*GPR88*	TSS200
cg01610165	5	124,071,029	8.91E-05	0.427	0.356					*ZNF608*	Body
cg26477549	12	106,642,937	7.67E-12	0.427	0.414					*CKAP4*	TSS1500
cg21634602	5	112,073,570	0.00055	0.427	0.242	1.89E-13	0.220			*APC*	1stExon
cg13631572	14	24,803,903	0.00014	0.426	0.457					*ADCY4*	TSS200
cg12814550	X	119,134,684	2.99E-05	0.426	0.328						
cg12026563	19	18,415,804	1.06E-18	0.426	0.421						
cg23824801	12	54,653,403	5.73E-47	0.426	0.430					*CBX5*	TSS200
cg04669574	12	50,297,945	0.000401	0.426	0.244					*FAIM2*	TSS1500
cg08518631	10	102,905,681	4.61E-08	0.426	0.443						
cg04945541	17	43,299,826	1.66E-24	0.426	0.388	1.2E-34	0.232			*FMNL1*	Body
cg13000649	17	17,626,790	6.35E-27	0.426	0.410					*RAI1*	5'UTR
cg21347053	6	1,624,978	9.7E-19	0.426	0.405					*GMDS*	Body
cg01536987	13	43,566,492	8.94E-05	0.426	0.406					*EPSTI1*	TSS200
cg24852548	4	57,522,632	2.94E-10	0.426	0.439					*HOPX*	1stExon
cg09235583	6	30,711,141	3.43E-20	0.426	0.401					*IER3*	3'UTR
cg24480859	1	9,129,791	8.91E-21	0.426	0.391	1.19E-25	0.242			*SLC2A5*	5'UTR
cg23200020	19	35,068,555	4.05E-17	0.426	0.394					*LOC643719*	Body
cg11417025	7	16,505,589	3.03E-21	0.426	0.399					*SOSTDC1*	TSS200
cg25209842	10	103,536,342	2.19E-09	0.426	0.392					*FGF8*	TSS1500
cg16794576	6	29,974,971	3.04E-11	0.426	0.421					*HLA-J*	Body
cg13356133	17	12,877,772	4.3E-06	0.426	0.261					*RICH2*	Body
cg02773050	X	134,654,287	9.47E-09	0.425	0.335					*DDX26B*	TSS1500

cg25916282	2	177,023,760	0.001255	0.425	0.433					
cg05497345	2	96,990,914	5.96E-08	0.425	0.453				*ITPRIPL1*	TSS1500
cg14861089	10	102,895,043	9.72E-07	0.425	0.399				*TLX1*	Body
cg07186138	22	39,410,396	1.07E-15	0.425	0.391	7.7E-36	0.301		*APOBEC3C*	Body
cg07838272	7	116,164,670	1.02E-09	0.425	0.338				*CAV1*	TSS200
cg17723653	10	75,407,823	1.13E-12	0.425	0.355				*SYNPO2L*	Body
cg20569203	12	120,799,911	1.33E-18	0.425	0.419				*MSI1*	Body
cg00590761	1	55,008,319	6.54E-16	0.425	0.378					
cg14855334	15	74,658,553	1.53E-14	0.425	0.400				*CYP11A1*	Body
cg12727795	5	149,535,695	4.58E-33	0.425	0.432	2.61E-24	0.323		*PDGFRB*	TSS1500
cg12239742	X	18,116,923	2.28E-14	0.425	0.381					
cg06654549	5	1,875,058	1.51E-18	0.425	0.419					
cg13857811	X	70,151,209	2.52E-10	0.425	0.412				*SLC7A3*	TSS1500
cg25813864	17	38,334,176	3.51E-05	0.425	0.346				*RAPGEFL1*	TSS200
cg05910443	7	101,688,675	2.22E-34	0.425	0.407				*CUX1*	Body
cg11570367	13	114,896,683	0.000238	0.425	0.380				*RASA3*	Body
cg25950739	X	101,907,254	8.05E-09	0.425	0.440				*GPRASP1*	5'UTR
cg04931383	4	37,978,731	4.65E-47	0.425	0.405				*TBC1D1*	Body
cg12436377	5	87,440,296	0.003198	0.424	0.535					
cg03834574	X	56,259,094	6.43E-10	0.424	0.412				*KLF8*	5'UTR
cg14449180	6	29,894,619	4.72E-08	0.424	0.420				*HCG4P6*	TSS1500
cg00691999	4	55,094,011	0.001894	0.424	0.464				*PDGFRA*	TSS1500
cg19068385	X	47,483,024	0.010199	0.424	0.119					
cg11751645	11	56,947,754	0.000104	0.424	0.369				*LRRC55*	TSS1500
cg06088324	16	19,895,321	1.81E-05	0.424	0.451				*GPRC5B*	5'UTR
cg19447962	17	17,628,656	1.74E-27	0.424	0.432				*RAI1*	5'UTR
cg02501155	16	79,263,473	4.97E-18	0.424	0.366					
cg18260397	5	154,230,438	5.35E-31	0.424	0.422				*C5orf4*	TSS1500
cg22753340	6	31,831,489	4.7E-16	0.424	0.416				*NEU1*	TSS1500
cg20431135	17	19,290,762	1.49E-32	0.424	0.423				*MFAP4*	TSS1500
cg01765758	X	106,750,146	8.82E-12	0.424	0.427					
cg16642281	4	109,034,555	3.72E-12	0.424	0.440				*LEF1*	Body
cg02032778	7	29,186,228	1.29E-06	0.424	0.379				*CPVL*	TSS200
cg01185682	17	53,343,553	6.99E-10	0.424	0.402				*HLF*	Body

cg19259179	7	54,956,252	5.54E-25	0.424	0.430					
cg15176829	1	246,952,215	1.57E-31	0.424	0.371				*LOC149134*	TSS1500
cg25318809	6	29,974,863	1.02E-11	0.424	0.434				*HLA-J*	Body
cg27397850	7	27,281,941	3.52E-12	0.424	0.339				*EVX1*	TSS1500
cg07969619	12	115,136,279	1.15E-21	0.423	0.406					
cg09193347	15	79,381,867	8.5E-15	0.423	0.414				*RASGRF1*	Body
cg01698298	11	61,062,964	1.16E-12	0.423	0.345				*VWCE*	TSS200
cg14544666	5	179,634,789	7.29E-09	0.423	0.464				*RASGEF1C*	5'UTR
cg13488570	1	2,222,253	5.37E-10	0.423	0.408			Yes	*SKI*	Body
cg02823783	12	124,246,919	0.000834	0.423	0.188				*DNAH10*	TSS200
cg04705659	1	204,329,107	6.63E-12	0.423	0.424				*PLEKHA6*	TSS200
cg04937416	7	157,479,272	5.62E-10	0.423	0.459				*PTPRN2*	Body
cg21549195	19	52,452,368	3.44E-05	0.423	0.461					
cg18376288	1	8,181,555	1.3E-14	0.423	0.399					
cg00763725	X	154,842,689	6.64E-06	0.423	0.385				*TMLHE*	TSS200
cg19954537	2	97,204,401	0.000225	0.423	0.433				*ARID5A*	Body
cg15632826	6	31,831,740	1.83E-55	0.423	0.402				*NEU1*	TSS1500
cg09729613	2	201,450,601	4.74E-07	0.423	0.371				*AOX1*	TSS200
cg03667968	5	112,073,438	0.013784	0.423	0.237				*APC*	TSS200
cg11228682	6	16,320,530	0.000487	0.422	0.516				*ATXN1*	Body
cg10541755	3	170,625,872	8.86E-07	0.422	0.264	3.96E-41	0.351		*EIF5A2*	5'UTR
cg16629469	6	108,440,603	0.003131	0.422	0.429					
cg06234063	12	65,219,392	5.32E-40	0.422	0.411			Yes		
cg20557104	19	41,934,313	3.08E-23	0.422	0.445	2.29E-33	0.304		*B3GNT8*	5'UTR
cg08580254	17	38,478,807	9.2E-25	0.422	0.385				*RARA*	5'UTR
cg22116670	5	151,066,730	6.45E-20	0.422	0.430				*SPARC*	TSS1500
cg25923609	5	3,607,092	7.67E-14	0.422	0.425					
cg11617147	7	105,517,689	1.53E-05	0.422	0.236				*ATXN7L1*	TSS1500
cg20693607	3	184,319,566	1.82E-05	0.422	0.317					
cg05124021	7	157,487,066	3.65E-21	0.422	0.403				*PTPRN2*	Body
cg21707172	6	155,537,944	2.04E-17	0.421	0.423				*TIAM2*	Body
cg19423014	18	59,560,091	3.83E-12	0.421	0.347	9.33E-25	0.282		*RNF152*	1stExon
cg18442524	5	137,610,372	0.001977	0.421	0.347				*GFRA3*	TSS200
cg24980893	7	116,164,709	0.001185	0.421	0.347				*CAV1*	TSS200

cg11856897	1	47,998,999	6.2E-39	0.421	0.408					
cg21422700	1	201,229,247	1.35E-34	0.421	0.404					
cg26412532	10	103,881,068	2.71E-29	0.420	0.425				*LDB1*	TSS1500
cg04380340	2	201,450,506	3.97E-06	0.420	0.373				*AOX1*	TSS1500
cg03120555	7	630,473	1.32E-12	0.420	0.377				*PRKAR1B*	Body
cg22871668	6	133,562,492	3.56E-05	0.420	0.526				*EYA4*	TSS200
cg17851725	2	121,269,372	5.61E-32	0.420	0.390					
cg01996714	20	3,220,839	3.96E-40	0.420	0.378					
cg27636310	1	46,632,696	4.57E-08	0.420	0.454					
cg14557510	2	122,042,345	1.19E-24	0.420	0.404				*TFCP2L1*	Body
cg04109822	20	50,158,057	1.22E-07	0.420	0.412				*NFATC2*	Body
cg19764345	3	57,198,797	1E-07	0.420	0.306				*IL17RD*	Body
cg12448161	3	48,632,723	4.64E-08	0.420	0.399				*COL7A1*	TSS200
cg22773555	11	830,233	3.17E-06	0.420	0.474				*EFCAB4A*	Body
cg11400774	1	156,051,324	0.047686	0.420	0.213				*MEX3A*	Body
cg25272143	11	62,211,990	1.48E-16	0.420	0.417				*AHNAK*	Body
cg09641578	15	40,734,696	8.8E-34	0.420	0.404				*BAHD1*	5'UTR
cg02231729	15	89,438,611	0.001689	0.420	0.420				*HAPLN3*	5'UTR
cg15219228	6	35,479,628	0.002315	0.420	0.465				*TULP1*	Body
cg23069167	3	61,550,379	5.86E-30	0.420	0.421				*PTPRG*	Body
cg13490422	12	50,377,731	3.13E-36	0.420	0.406					
cg13204538	X	119,445,300	3.74E-14	0.420	0.384				*FAM70A*	1stExon
cg03078363	12	54,408,664	1.31E-10	0.420	0.410					
cg07696699	4	17,783,502	3.31E-07	0.420	0.443				*FAM184B*	TSS1500
cg07816687	16	67,197,186	4.17E-05	0.419	0.501				*HSF4*	TSS200
cg11097717	2	191,045,635	5.23E-18	0.419	0.379				*C2orf88*	5'UTR
cg02916102	15	74,658,244	5.99E-21	0.419	0.377				*CYP11A1*	1stExon
cg26871372	4	41,867,517	0.000264	0.419	0.467					
cg01025836	20	37,230,326	1.38E-19	0.419	0.418				*C20orf95*	TSS1500
cg11213690	7	149,112,402	3.06E-28	0.419	0.444					
cg14133945	11	60,809,985	0.000978	0.419	0.284					
cg01676962	8	91,997,001	9.28E-13	0.419	0.368					
cg21537736	X	149,534,089	3.82E-06	0.419	0.446					
cg16162970	14	105,779,952	2.56E-27	0.419	0.420				*PACS2*	TSS1500

cg20622056	X	70,151,134	6.04E-23	0.419	0.394	6.41E-13	0.239		*SLC7A3*	TSS200
cg01635086	17	42,386,326	8.17E-06	0.419	0.434				*RUNDC3A*	Body
cg06744545	2	27,958,421	6.07E-05	0.419	0.326					
cg18759209	20	55,842,071	1.43E-31	0.419	0.370				*BMP7*	TSS1500
cg13722431	6	2,903,533	3.4E-14	0.419	0.380				*SERPINB9*	1stExon
cg20101352	7	29,186,234	1.25E-05	0.418	0.373				*CPVL*	TSS200
cg14032025	20	50,721,129	1.22E-10	0.418	0.433				*ZFP64*	Body
cg13934406	6	32,120,878	7.65E-10	0.418	0.439				*PPT2*	TSS1500
cg00409917	6	29,894,679	7.55E-08	0.418	0.415				*HCG4P6*	TSS1500
cg01614237	4	6,660,771	3.69E-07	0.418	0.429					
cg15275625	3	13,343,118	1.58E-25	0.418	0.431					
cg23624008	1	70,035,479	3.51E-05	0.418	0.404					
cg13247663	8	59,058,254	8.75E-48	0.418	0.404				*FAM110B*	5'UTR
cg04557452	11	62,691,251	1.52E-09	0.418	0.386					
cg01853276	2	24,162,441	0.000199	0.418	0.444				*UBXN2A*	TSS1500
cg25020459	21	47,402,144	4.07E-07	0.418	0.404	1.62E-19	0.257		*COL6A1*	Body
cg02896361	X	47,441,581	0.002048	0.418	0.413				*SYN1*	Body
cg22855900	5	72,529,662	3.66E-08	0.418	0.391					
cg04889072	3	193,776,187	2.2E-16	0.418	0.376					
cg08320659	6	26,088,202	9.69E-05	0.418	0.387				*HFE*	Body
cg02534363	3	47,050,950	6.54E-08	0.418	0.355				*NBEAL2*	3'UTR
cg06711837	X	137,792,814	3.46E-14	0.417	0.317				*FGF13*	Body
cg01518607	6	27,235,891	0.000347	0.417	0.518					
cg03159396	6	50,808,813	1.72E-06	0.417	0.441				*TFAP2B*	Body
cg07777008	11	27,015,808	4.13E-13	0.417	0.424				*FIBIN*	5'UTR
cg23083672	X	13,587,603	1.72E-17	0.417	0.418	2.18E-18	0.263		*EGFL6*	TSS200
cg22902400	22	39,473,197	8.5E-13	0.417	0.408				*APOBEC3G*	5'UTR
cg10119075	10	7,709,738	1.28E-21	0.417	0.400				*ITIH5*	TSS1500
cg22222281	12	6,308,758	1.52E-19	0.417	0.383				*CD9*	TSS1500
cg12153794	12	115,131,549	1.65E-09	0.417	0.441			Yes		
cg26107890	3	124,860,686	3.59E-07	0.417	0.419				*SLC12A8*	Body
cg08396863	2	220,117,599	7.77E-25	0.417	0.417				*TUBA4B*	TSS1500
cg16515238	X	119,444,909	0.002807	0.417	0.288				*FAM70A*	Body
cg22598028	3	44,626,492	1.12E-06	0.417	0.264	2.86E-11	0.211		*ZNF660*	5'UTR

cg14095438	8	49,468,684	1.42E-11	0.417	0.366					
cg17239057	1	224,363,575	1.18E-20	0.417	0.411					
cg03774468	3	140,950,104	5.3E-05	0.417	0.371				*ACPL2*	TSS1500
cg01821058	16	55,512,870	4.07E-06	0.416	0.378				*MMP2*	TSS1500
cg06443533	1	1,182,424	0.001561	0.416	0.423				*FAM132A*	TSS1500
cg14553740	9	18,976,715	4.35E-16	0.416	0.363				*FAM154A*	Body
cg01340542	1	65,468,273	2.42E-11	0.416	0.320					
cg01655356	11	70,672,841	1.67E-33	0.416	0.400				*SHANK2*	Body
cg04448487	20	42,875,777	4.25E-08	0.416	0.273	5.32E-26	0.319		*GDAP1L1*	TSS200
cg16562730	16	71,560,187	1.05E-15	0.416	0.376				*CHST4*	1stExon
cg14318199	X	12,974,511	4.4E-07	0.416	0.403					
cg23130010	6	29,855,462	2.73E-21	0.416	0.356				*HLA-H*	Body
cg03049782	17	41,363,891	3.28E-22	0.416	0.390				*TMEM106A*	TSS200
cg07091016	1	36,174,005	2.96E-07	0.416	0.299					
cg22761670	17	74,268,201	0.003564	0.416	0.474					
cg12627583	2	201,450,731	7.19E-05	0.416	0.365	8.96E-25	0.304		*AOX1*	1stExon
cg18881873	20	52,276,947	4.17E-11	0.416	0.429					
cg08266417	2	201,450,575	7.5E-05	0.416	0.367				*AOX1*	TSS200
cg12277627	X	129,245,225	3.25E-06	0.416	0.324				*ELF4*	TSS1500
cg07402729	11	61,062,902	5.91E-13	0.416	0.353				*VWCE*	TSS200
cg11122493	17	74,706,551	0.003325	0.415	0.315				*MXRA7*	Body
cg08918274	10	17,270,300	5.78E-09	0.415	0.412				*VIM*	5'UTR
cg20785674	1	150,123,490	9.94E-22	0.415	0.384				*PLEKHO1*	Body
cg07602744	7	157,484,164	5.74E-18	0.415	0.406				*PTPRN2*	Body
cg00507008	12	115,131,421	6.22E-07	0.415	0.447					
cg21932800	X	135,056,858	1.47E-14	0.415	0.347	8.87E-31	0.209		*MMGT1*	TSS1500
cg01335367	12	110,172,208	9.92E-27	0.415	0.404				*MGC14436*	Body
cg18363192	6	31,831,434	1.87E-15	0.415	0.399				*NEU1*	TSS1500
cg08096786	16	57,571,099	6.16E-06	0.415	0.368				*CCDC102A*	TSS1500
cg14488317	11	3,181,446	1.32E-37	0.415	0.406				*OSBPL5*	5'UTR
cg01373189	13	33,002,820	8.64E-16	0.415	0.392				*N4BP2L1*	TSS1500
cg06092265	12	65,219,655	4.76E-74	0.415	0.409					
cg04920358	8	49,468,800	1.11E-05	0.415	0.398					
cg05542338	4	5,710,407	0.000111	0.415	0.330				*EVC2*	5'UTR

cg03340649	3	44,626,453	4.5E-11	0.415	0.297				*ZNF660*	TSS200
cg02264990	12	54,447,243	3.06E-27	0.415	0.407				*HOXC4*	5'UTR
cg17246140	2	43,020,264	1.87E-54	0.415	0.392				*HAAO*	TSS1500
cg23740882	20	44,098,387	2.98E-06	0.415	0.282				*WFDC2*	TSS200
cg24144083	3	36,805,814	1.98E-08	0.414	0.415					
cg10513702	1	200,003,800	3.9E-08	0.414	0.400				*NR5A2*	Body
cg17397592	2	237,416,338	1.98E-08	0.414	0.380				*IQCA1*	TSS1500
cg01908462	4	184,718,215	1.94E-08	0.414	0.395					
cg18531004	3	128,564,980	1.46E-08	0.414	0.364					
cg05452406	6	10,881,891	8.78E-23	0.414	0.393				*GCM2*	Body
cg10510586	X	68,348,597	1.91E-06	0.414	0.469					
cg20162652	12	66,629,018	9.61E-17	0.414	0.406				*IRAK3*	Body
cg03475285	8	54,789,207	5.74E-27	0.414	0.417				*RGS20*	Body
cg22369786	13	80,911,626	5.26E-06	0.414	0.406				*SPRY2*	Body
cg14088357	19	46,800,338	1.74E-35	0.414	0.407				*HIF3A*	TSS1500
cg11543592	12	115,124,067	3.85E-17	0.413	0.391					
cg06334134	7	142,986,693	1.47E-23	0.413	0.365				*CASP2*	Body
cg23839159	1	43,281,794	2.08E-22	0.413	0.407				*CCDC23*	Body
cg10030843	12	115,303,226	1.1E-24	0.413	0.397					
cg00755063	19	13,275,288	5.06E-17	0.413	0.327					
cg14914046	6	32,223,098	2.43E-10	0.413	0.385					
cg13420364	1	234,857,659	2.91E-25	0.413	0.395					
cg24645214	8	54,789,978	3.61E-15	0.413	0.404				*RGS20*	Body
cg26149167	19	16,187,889	0.002439	0.413	0.390				*TPM4*	Body
cg12317414	10	75,407,991	1.23E-28	0.413	0.394				*SYNPO2L*	Body
cg11638117	X	139,587,335	0.001649	0.413	0.531				*SOX3*	TSS200
cg13625875	4	54,959,419	2.4E-15	0.413	0.377					
cg08730348	16	31,214,307	3.2E-13	0.412	0.289				*PYCARD*	TSS200
cg03304815	3	134,083,139	3.98E-30	0.412	0.430				*AMOTL2*	Body
cg03376794	16	25,704,507	6.59E-10	0.412	0.423				*HS3ST4*	Body
cg18060199	1	94,146,495	1.98E-14	0.412	0.373	4.61E-27	0.211		*BCAR3*	5'UTR
cg18542056	19	51,523,205	8.51E-17	0.412	0.414				*KLK10*	5'UTR
cg13221363	2	12,859,103	5.95E-06	0.412	0.478				*TRIB2*	Body
cg05168015	5	171,880,461	9.52E-23	0.412	0.411				*SH3PXD2B*	Body

cg25801701	4	54,959,548	2.3E-12	0.412	0.432					
cg20927242	6	29,692,011	8.11E-11	0.412	0.400				*HLA-F*	Body
cg18132916	6	79,620,363	1.07E-07	0.412	0.433					
cg12911763	11	125,133,335	1.09E-17	0.412	0.405				*PKNOX2*	5'UTR
cg07961015	5	1,876,215	8.1E-15	0.412	0.401					
cg24302235	3	170,746,073	1.22E-09	0.412	0.381				*SLC2A2*	TSS1500
cg26250609	11	67,351,273	1.32E-70	0.412	0.416				*GSTP1*	1stExon
cg13804182	6	30,711,796	8.04E-09	0.412	0.430				*FLOT1*	TSS1500
cg21606115	7	157,361,752	1.47E-25	0.412	0.399				*PTPRN2*	Body
cg26621770	12	115,131,447	6.59E-09	0.412	0.420					
cg12177793	14	24,837,892	0.000236	0.411	0.395				*NFATC4*	Body
cg14518209	5	151,066,268	7.52E-19	0.411	0.454				*SPARC*	5'UTR
cg14553243	2	219,646,657	0.000597	0.411	0.293				*CYP27A1*	5'UTR
cg19113375	9	98,784,351	2.94E-09	0.411	0.299				*NCRNA00092*	TSS1500
cg00059652	17	67,605,498	4.2E-20	0.411	0.378					
cg03079733	21	43,183,466	7.15E-21	0.411	0.401				*RIPK4*	Body
cg20556517	19	18,540,330	1.31E-07	0.411	0.273				*SSBP4*	Body
cg05915293	16	2,041,512	9.76E-09	0.411	0.416				*SYNGR3*	Body
cg06537680	16	67,204,316	5.48E-05	0.411	0.452					
cg00734904	17	55,888,454	5.76E-40	0.411	0.398					
cg20092531	18	54,789,058	9.64E-05	0.411	0.334					
cg00330929	19	2,282,010	4.27E-07	0.411	0.343				*C19orf35*	5'UTR
cg23272399	1	53,068,579	1.27E-08	0.411	0.318				*GPX7*	Body
cg26205652	12	48,591,994	8.3E-05	0.410	0.416					
cg19640166	10	106,088,711	0.000513	0.410	0.446				*ITPRIP*	5'UTR
cg07836831	1	2,460,865	1.37E-15	0.410	0.428				*HES5*	3'UTR
cg08924430	4	106,067,052	1.03E-10	0.410	0.299	2.84E-38	0.275		*TET2*	TSS1500
cg16523424	X	47,441,779	3.3E-05	0.410	0.408	3.27E-18	0.108		*SYN1*	Body
cg04743654	3	50,378,292	1.26E-07	0.410	0.392				*RASSF1*	5'UTR
cg06632214	11	314,074	0.000161	0.410	0.228				*IFITM1*	1stExon
cg12053769	1	2,461,413	3.03E-27	0.410	0.403				*HES5*	Body
cg03191045	20	36,040,903	0.000177	0.410	0.498					
cg21604803	19	50,194,662	7.88E-05	0.409	0.306	2.03E-10	0.214		*CPT1C*	5'UTR
cg08946731	7	32,981,826	2.88E-06	0.409	0.201				*RP9P*	Body

cg02865822	11	46,383,141	5E-32	0.409	0.395				*DGKZ*	Body
cg20229534	6	10,882,250	0.000337	0.409	0.441				*GCM2*	TSS200
cg11682697	1	56,992,372	9.01E-22	0.409	0.410				*PPAP2B*	Body
cg13570656	15	75,019,196	7.68E-16	0.409	0.390				*CYP1A1*	TSS1500
cg04109898	6	29,943,455	3.86E-07	0.409	0.396				*HCG9*	Body
cg14502484	5	1,446,208	2.07E-26	0.409	0.372				*SLC6A3*	TSS1500
cg23489273	6	29,910,292	8.59E-51	0.409	0.376				*HLA-A*	TSS200
cg26955987	12	3,862,457	0.013211	0.409	0.290				*EFCAB4B*	TSS200
cg18108623	17	33,701,321	2.95E-14	0.409	0.417	5.26E-28	0.254		*SLFN11*	TSS1500
cg03085312	17	38,465,281	7.11E-32	0.409	0.398	2.75E-38	0.266		*RARA*	TSS200
cg09720701	12	54,447,283	9.21E-10	0.409	0.346				*HOXC4*	5'UTR
cg00758229	3	25,469,148	2.62E-11	0.409	0.359				*RARB*	TSS1500
cg15052447	17	74,696,872	5.18E-07	0.409	0.448				*MXRA7*	Body
cg26386409	12	63,025,771	0.000205	0.409	0.332					
cg06100756	17	43,221,575	2.31E-23	0.408	0.356					
cg14432143	6	29,975,061	8.54E-09	0.408	0.384				*HLA-J*	Body
cg09172548	12	3,862,221	0.025701	0.408	0.290				*EFCAB4B*	1stExon
cg19414967	8	49,468,296	5.41E-25	0.408	0.414					
cg21327343	X	56,258,579	1.2E-08	0.408	0.344				*KLF8*	TSS1500
cg03679544	6	155,537,972	1.45E-11	0.408	0.405				*TIAM2*	Body
cg27021986	15	41,805,273	2.72E-20	0.408	0.431				*LTK*	Body
cg18450254	3	64,200,005	2.65E-15	0.408	0.397				*PRICKLE2*	5'UTR
cg26260038	4	16,085,716	1.52E-32	0.408	0.394				*PROM1*	TSS200
cg22272840	8	10,586,882	5.4E-11	0.408	0.398				*SOX7*	Body
cg14210694	2	191,045,632	2.79E-14	0.408	0.364				*C2orf88*	5'UTR
cg00260634	2	219,847,233	2.04E-30	0.408	0.423				*FEV*	Body
cg19423622	7	158,362,729	1.02E-36	0.408	0.413				*PTPRN2*	Body
cg18094781	3	25,469,430	6.09E-34	0.408	0.386				*RARB*	TSS1500
cg09025501	16	88,717,472	1.87E-12	0.408	0.394				*CYBA*	TSS200
cg18403673	11	63,828,569	1.8E-26	0.408	0.411				*MACROD1*	Body
cg14547067	11	46,317,154	0.000544	0.408	0.328				*CREB3L1*	Body
cg26668837	11	8,615,356	7.92E-13	0.408	0.375				*STK33*	1stExon
cg09549813	16	4,587,862	5.5E-39	0.407	0.419				*C16orf5*	5'UTR
cg09275704	16	54,317,042	1.47E-18	0.407	0.373					

cg06404175	20	3,052,692	0.000991	0.407	0.349				*OXT*	Body	
cg14250833	6	10,882,240	0.003205	0.407	0.474				*GCM2*	TSS200	
cg01814945	6	29,974,723	2.1E-08	0.407	0.407				*HLA-J*	Body	
cg01622437	12	115,136,246	7.79E-26	0.407	0.391						
cg08412913	16	85,429,522	2.37E-13	0.407	0.327						
cg19714985	10	77,043,608	2.57E-10	0.407	0.433						
cg25110734	5	149,535,650	4.04E-42	0.407	0.374				*PDGFRB*	TSS1500	
cg25614853	X	149,534,258	8.38E-10	0.407	0.346						
cg18241962	17	75,385,432	7.96E-16	0.407	0.396				*SEPT9*	5'UTR	
cg18322546	14	77,422,738	8.39E-10	0.407	0.351						
cg23180938	5	115,152,485	0.000889	0.407	0.231				*CDO1*	TSS200	
cg18690729	10	128,250,156	9.9E-05	0.407	0.357						
cg17235702	7	54,956,419	1.84E-08	0.406	0.379						
cg15336765	12	50,355,307	1.36E-11	0.406	0.348				*AQP5*	1stExon	
cg12077963	17	4,079,306	1.39E-16	0.406	0.381				*ANKFY1*	Body	
cg15804105	6	26,172,302	0.000374	0.406	0.478						
cg00980978	20	44,098,724	4.9E-06	0.406	0.285	2.39E-18	0.208		*WFDC2*	Body	
cg03796321	15	86,162,442	2.59E-09	0.406	0.385				*AKAP13*	Body	
cg06426416	4	5,710,403	1.16E-05	0.406	0.291				*EVC2*	5'UTR	
cg09017619	7	27,196,320	0.000002	0.406	0.404				*HOXA7*	TSS200	
cg04890459	16	67,204,681	2.61E-05	0.406	0.424						
cg25200152	1	147,790,458	2.5E-08	0.406	0.360						
cg27538686	1	76,540,641	2.66E-12	0.406	0.335				*ST6GALNAC3*	Body	
cg03867465	4	183,062,301	1.19E-16	0.406	0.376				*MGC45800*	Body	
cg01414687	1	31,846,873	0.006862	0.406	0.216				*FABP3*	TSS1500	
cg09535960	8	23,193,704	1.18E-19	0.406	0.386				*LOXL2*	Body	
cg15255584	X	8,698,502	0.000828	0.406	0.291				*KAL1*	Body	
cg08548396	6	30,095,549	6.52E-05	0.406	0.413						
cg19653212	6	42,145,954	1.54E-05	0.406	0.466				*GUCA1A*	Body	
cg08750951	4	89,378,894	0.002084	0.406	0.222				*HERC5*	Body	
cg14724419	11	111,178,990	2.73E-07	0.406	0.420				*C11orf93*	Body	
cg26066597	14	21,540,022	1.32E-19	0.406	0.337				*FLJ10357*	Body	
cg23166865	1	63,539,729	6.81E-08	0.406	0.376						
cg25378916	5	1,875,013	1.88E-13	0.406	0.409						

cg04507515	11	74,178,268	0.000593	0.405	0.305				*KCNE3*	5'UTR
cg13999188	2	175,548,310	1.54E-06	0.405	0.288				*WIPF1*	TSS1500
cg22455450	19	53,038,972	4.57E-10	0.405	0.385				*ZNF808*	5'UTR
cg21334513	6	30,095,248	1.13E-18	0.405	0.422					
cg05372113	19	41,595,975	3.87E-24	0.405	0.386				*CYP2A13*	Body
cg23609985	1	119,870,339	1.18E-13	0.405	0.339					
cg09183124	1	173,092,323	1.28E-28	0.405	0.377					
cg13283765	2	200,853,063	6.86E-13	0.405	0.435					
cg26119367	2	17,699,620	0.000371	0.405	0.444				*RAD51AP2*	1stExon
cg23350336	10	70,321,554	4.12E-10	0.405	0.388				*TET1*	5'UTR
cg16364121	17	35,291,924	1.34E-05	0.405	0.440					
cg02051239	2	74,375,507	1.06E-26	0.405	0.349				*BOLA3*	TSS1500
cg26319579	6	35,466,014	1.12E-18	0.405	0.416				*TULP1*	3'UTR
cg04804377	2	17,699,746	0.02217	0.405	0.425				*RAD51AP2*	TSS200
cg14287112	6	133,562,485	4.86E-06	0.405	0.489				*EYA4*	TSS200
cg16670497	1	110,210,913	8.46E-16	0.405	0.364	3.06E-12	0.226		*GSTM2*	Body
cg00310940	1	9,129,648	5.66E-31	0.405	0.361				*SLC2A5*	5'UTR
cg23880581	15	78,912,387	4.97E-18	0.405	0.345				*CHRNA3*	Body
cg26010734	19	15,344,046	6E-07	0.404	0.379				*EPHX3*	TSS200
cg02054792	2	106,014,950	3.27E-05	0.404	0.455				*FHL2*	5'UTR
cg00506343	12	54,421,033	1.86E-11	0.404	0.330				*HOXC4*	5'UTR
cg01392772	17	53,342,740	1.45E-05	0.404	0.389				*HLF*	5'UTR
cg20145692	1	40,781,289	3.72E-06	0.404	0.381				*COL9A2*	Body
cg00447833	8	30,243,260	6.43E-10	0.404	0.319				*RBPMS*	Body
cg09143713	20	9,141,615	1.96E-09	0.404	0.391				*PLCB4*	5'UTR
cg26206183	14	24,457,993	1.68E-21	0.404	0.411				*DHRS4L2*	TSS200
cg12265829	14	24,804,022	7.58E-12	0.404	0.420	1.74E-19	0.287		*ADCY4*	TSS200
cg07561894	19	18,540,208	8.81E-07	0.404	0.257				*SSBP4*	Body
cg06899186	7	148,394,994	2.89E-05	0.404	0.261				*CUL1*	TSS1500
cg26105015	3	122,745,099	2.03E-20	0.404	0.388				*SEMA5B*	5'UTR
cg08790676	17	15,686,438	7.88E-05	0.404	0.369					
cg26272220	12	81,471,884	6.01E-05	0.404	0.379				*ACSS3*	1stExon
cg02303897	12	53,268,136	0.014879	0.404	0.315					
cg25568066	5	126,205,045	1.16E-34	0.404	0.403				*MARCH3*	3'UTR

cg05661282	19	58,220,370	9.31E-08	0.404	0.344				*ZNF154*	5'UTR
cg26670562	4	86,954,105	8.17E-24	0.403	0.380				*MAPK10*	Body
cg01005439	16	67,204,010	1.4E-06	0.403	0.438					
cg04367486	3	112,051,991	0.004363	0.403	0.483				*CD200*	1stExon
cg25720815	6	29,974,692	6.07E-08	0.403	0.406				*HLA-J*	Body
cg08668790	19	58,220,662	2.07E-12	0.403	0.363	2.25E-21	0.287		*ZNF154*	TSS200
cg03030717	12	65,218,069	0.012416	0.403	0.105					
cg17537073	13	24,902,500	0.004885	0.403	0.369					
cg27608999	10	45,914,276	1.89E-12	0.403	0.370				*ALOX5*	Body
cg01240056	7	1,263,927	2.32E-09	0.403	0.416					
cg05471296	7	27,196,302	6.73E-10	0.403	0.419				*HOXA7*	TSS200
cg15732502	10	93,647,300	4.66E-05	0.403	0.337					
cg26655340	5	154,230,141	1.89E-30	0.403	0.385				*C5orf4*	5'UTR
cg02215070	7	134,144,055	0.002207	0.403	0.256				*AKR1B1*	TSS200
cg06905453	19	14,089,284	8.87E-15	0.403	0.375				*RFX1*	Body
cg11155687	12	63,025,589	4.23E-09	0.403	0.312					
cg05687686	2	180,726,697	9.12E-08	0.403	0.284				*MIR1258*	TSS1500
cg26803268	10	18,549,536	8.4E-10	0.403	0.433				*CACNB2*	Body
cg14639847	12	54,389,982	0.001172	0.403	0.312					
cg12457529	7	99,155,892	0.000536	0.403	0.283				*ZNF655*	TSS200
cg07251099	3	112,052,405	5.63E-05	0.403	0.500				*CD200*	Body
cg03678039	17	74,525,066	1.01E-17	0.403	0.390			Yes	*CYGB*	Body
cg07285673	2	29,337,984	2.39E-07	0.403	0.284				*CLIP4*	TSS1500
cg10096645	18	24,130,851	1.08E-26	0.403	0.377				*KCTD1*	5'UTR
cg03118626	19	51,523,252	4.59E-19	0.403	0.397				*KLK10*	5'UTR
cg04012931	6	30,711,164	1.94E-19	0.403	0.403				*IER3*	3'UTR
cg13102585	17	42,386,208	1.92E-32	0.403	0.379	1.59E-29	0.256		*RUNDC3A*	1stExon
cg25593560	19	16,187,364	0.000631	0.402	0.390				*TPM4*	Body
cg19263700	6	32,223,236	2.72E-13	0.402	0.377					
cg27449131	6	30,712,027	0.003601	0.402	0.413				*IER3*	Body
cg05286653	19	46,800,602	7.15E-21	0.402	0.384				*HIF3A*	Body
cg14038391	10	102,900,130	9.94E-07	0.402	0.347					
cg08857063	6	50,808,667	4.59E-15	0.402	0.410				*TFAP2B*	Body
cg23398076	2	66,664,960	0.001111	0.402	0.467				*MEIS1*	Body

cg26733301	20	61,585,924	2.51E-18	0.402	0.380				*SLC17A9*	Body
cg04600466	5	139,076,830	4.58E-36	0.402	0.389					
cg07846311	4	57,371,927	4.54E-07	0.402	0.415				*ARL9*	5'UTR
cg01116067	1	2,461,838	4.41E-10	0.402	0.371				*HES5*	TSS200
cg16680677	10	80,008,481	8.1E-11	0.402	0.341					
cg17911788	17	44,343,683	3.33E-09	0.402	0.338					
cg27447477	12	111,023,252	6.77E-18	0.402	0.387					
cg08421126	2	43,019,854	2.02E-07	0.402	0.246				*HAAO*	TSS200
cg08693490	12	116,757,896	2.77E-15	0.402	0.398					
cg21386647	3	9,289,893	3.26E-07	0.402	0.269				*SRGAP3*	Body
cg16133088	12	3,862,428	0.019573	0.402	0.275				*EFCAB4B*	TSS200
cg00890485	14	70,653,919	9.71E-11	0.402	0.380				*SLC8A3*	5'UTR
cg09704415	1	48,937,250	4E-24	0.402	0.345	8.46E-33	0.274		*SPATA6*	Body
cg06463958	6	166,582,393	0.02384	0.402	0.250				*T*	TSS1500
cg23244790	5	140,811,102	3.46E-17	0.402	0.369				*PCDHGA4*	Body
cg25542878	4	6,474,782	1.14E-05	0.402	0.302				*PPP2R2C*	TSS1500
cg26332488	1	2,230,640	1.91E-25	0.402	0.396				*SKI*	Body
cg26121931	6	29,895,204	1.45E-09	0.402	0.387					
cg00084338	6	170,595,920	3.48E-11	0.402	0.389				*DLL1*	Body
cg20786074	2	56,150,255	1.16E-26	0.402	0.393	1.2E-27	0.282		*EFEMP1*	1stExon
cg19166302	3	122,296,613	6.65E-10	0.401	0.397				*PARP15*	1stExon
cg10400707	1	160,369,840	7.21E-12	0.401	0.360				*VANGL2*	TSS1500
cg24530250	11	71,955,599	1.14E-05	0.401	0.373				*PHOX2A*	TSS1500
cg17469978	7	116,164,704	3.26E-06	0.401	0.321	9.54E-26	0.236		*CAV1*	TSS200
cg19736608	4	47,840,050	3.4E-06	0.401	0.357				*CORIN*	5'UTR
cg03336086	5	79,331,047	1.27E-11	0.401	0.341				*THBS4*	1stExon
cg07664553	4	57,372,597	1.21E-22	0.401	0.409				*ARL9*	5'UTR
cg01446950	2	222,435,351	0.00027	0.401	0.286				*EPHA4*	Body
cg04987053	16	5,038,078	0.000202	0.401	0.470				*SEC14L5*	Body
cg11027354	5	10,761,677	0.015703	0.401	0.210				*DAP*	TSS1500
cg13928417	9	124,498,782	4.11E-32	0.401	0.383				*DAB2IP*	Body
cg17265994	11	2,905,024	3.97E-12	0.401	0.328				*CDKN1C*	3'UTR
cg13248778	7	128,337,725	0.00544	0.401	0.345					
cg20687242	12	54,653,427	4.61E-10	0.401	0.376				*CBX5*	TSS200

cg20365336	6	16,986,186	3.27E-15	0.401	0.361					
cg14173147	9	27,528,300	1.37E-22	0.401	0.381				*MOBKL2B*	5'UTR
cg07398614	3	128,564,918	0.000356	0.401	0.358					
cg12548341	10	104,420,963	3.08E-22	0.401	0.390					
cg27498387	8	41,655,789	2.51E-31	0.401	0.383				*ANK1*	TSS1500
cg00420348	11	830,190	7.04E-08	0.401	0.423				*EFCAB4A*	Body
cg01016662	1	110,673,082	0.00131	0.401	0.339					
cg24251193	1	156,676,542	4.84E-07	0.400	0.377				*CRABP2*	TSS1500
cg22844232	3	193,776,047	7.46E-13	0.400	0.374					
cg03405260	17	77,786,344	1.61E-16	0.400	0.393					
cg25228995	5	139,260,468	1.43E-25	0.400	0.352				*NRG2*	Body
cg03789645	7	148,394,842	5.24E-16	0.400	0.367				*CUL1*	TSS1500
cg27486427	3	25,469,919	3.02E-32	0.400	0.390	1.81E-26	0.258		*RARB*	5'UTR
cg19266578	20	62,711,729	5.02E-13	0.400	0.410				*RGS19*	TSS1500
cg23477967	2	188,419,087	5.47E-07	0.400	0.438	1.38E-14	0.234		*TFPI*	1stExon
cg16498913	5	1,851,563	3.71E-13	0.400	0.401					
cg23369529	1	68,149,989	2.05E-17	0.400	0.334				*GADD45A*	TSS1500
cg05972871	12	3,862,444	0.018095	0.400	0.275				*EFCAB4B*	TSS200
cg06786153	15	78,327,011	1.62E-08	0.400	0.350				*TBC1D2B*	Body
cg20577019	1	150,123,077	0.001663	0.400	0.255				*PLEKHO1*	Body
cg23155911	5	126,205,009	3E-36	0.400	0.387				*MARCH3*	3'UTR
cg12574408	7	25,219,543	1.17E-05	0.400	0.303				*C7orf31*	5'UTR
cg26310256	X	13,588,301	5.29E-18	0.400	0.421				*EGFL6*	Body
cg10708955	17	8,056,950	1.27E-27	0.399	0.388				*PER1*	TSS1500
cg00751021	2	206,474,068	2.18E-22	0.399	0.400				*PARD3B*	Body
cg21527621	1	55,013,419	1.99E-11	0.399	0.386				*ACOT11*	TSS1500
cg09651145	5	148,651,144	3.03E-09	0.399	0.346				*AFAP1L1*	TSS1500
cg14081667	21	44,782,497	3.68E-40	0.399	0.397					
cg21602557	8	119,122,878	0.000101	0.399	0.297				*EXT1*	1stExon
cg05422029	6	27,235,843	2.23E-05	0.399	0.483					
cg07311845	X	154,842,736	4.13E-09	0.399	0.319	5.72E-19	0.240		*TMLHE*	TSS200
cg21203569	11	47,279,365	8.46E-16	0.399	0.378				*NR1H3*	TSS200
cg05885936	2	132,088,912	2.1E-09	0.399	0.437					
cg03172947	20	44,035,850	1.92E-14	0.399	0.374				*DBNDD2*	5'UTR

cg05102394	4	148,402,899	9.68E-20	0.399	0.358				*EDNRA*	Body
cg06563300	12	100,750,811	1.06E-07	0.399	0.403	3.09E-17	0.291		*SLC17A8*	TSS200
cg10938374	6	30,712,058	0.00601	0.399	0.423				*IER3*	Body
cg06154084	10	102,792,249	2.26E-05	0.398	0.427				*PDZD7*	TSS1500
cg16570157	11	829,783	0.014512	0.398	0.427				*EFCAB4A*	Body
cg02667022	5	147,162,223	1.21E-09	0.398	0.361				*JAKMIP2*	5'UTR
cg03481274	3	25,469,708	5.27E-35	0.398	0.380				*RARB*	TSS200
cg13417862	17	855,238	1.46E-41	0.398	0.385				*NXN*	Body
cg16964025	7	157,482,215	1.71E-27	0.398	0.409				*PTPRN2*	Body
cg12098872	19	47,742,726	1.55E-08	0.398	0.370					
cg05573767	6	167,171,394	6.15E-07	0.398	0.393				*RPS6KA2*	Body
cg18296036	7	150,653,115	8.73E-24	0.398	0.378				*KCNH2*	Body
cg06747432	19	46,916,741	1.91E-05	0.398	0.477				*CCDC8*	1stExon
cg05852143	12	5,540,116	2.07E-07	0.398	0.453				*NTF3*	TSS1500
cg14907769	8	49,468,941	2.15E-19	0.398	0.391					
cg03810476	X	17,392,798	9.68E-29	0.398	0.358				*NHS*	TSS1500
cg04299389	20	25,129,296	4.1E-14	0.398	0.347				*LOC284798*	Body
cg25006194	12	94,288,553	3.65E-19	0.398	0.388					
cg03711016	16	12,997,684	2.4E-09	0.398	0.399				*SHISA9*	Body
cg05781504	5	145,713,607	1.15E-07	0.398	0.353					
cg06105694	17	36,585,340	1.9E-36	0.398	0.384					
cg19788741	5	101,632,310	3.06E-10	0.397	0.379				*SLCO4C1*	TSS200
cg21674704	8	131,455,249	4.02E-06	0.397	0.221					
cg03340119	1	246,952,508	2.01E-53	0.397	0.361				*LOC149134*	TSS1500
cg17160382	6	121,758,980	0.002313	0.397	0.444				*GJA1*	5'UTR
cg23079252	6	30,095,199	1.25E-27	0.397	0.396					
cg16079958	5	71,404,742	5.35E-10	0.397	0.336				*MAP1B*	Body
cg17568996	22	42,828,125	5.55E-27	0.397	0.357	1.16E-28	0.296		*NFAM1*	Body
cg07429623	1	205,326,201	1.14E-26	0.397	0.396				*KLHDC8A*	TSS200
cg15846886	17	79,790,116	1.24E-30	0.397	0.385				*FAM195B*	5'UTR
cg15726260	6	29,974,900	2.05E-10	0.397	0.391				*HLA-J*	Body
cg02783887	2	660,856	5.76E-20	0.397	0.390					
cg08253296	5	74,907,592	5.64E-19	0.397	0.343					
cg25272065	1	77,334,678	9.56E-09	0.397	0.341				*ST6GALNAC5*	Body

cg06244497	19	39,466,575	1.5E-08	0.397	0.432				*FBXO17*	TSS200
cg22674613	6	44,310,331	4.11E-07	0.397	0.417				*SPATS1*	TSS200
cg13114305	X	100,446,736	6.54E-10	0.397	0.401					
cg03257575	1	46,951,318	0.001676	0.397	0.344					
cg25896644	11	116,371,487	0.005215	0.397	0.375					
cg27663938	12	103,696,381	7.39E-08	0.397	0.327				*C12orf42*	Body
cg22935450	17	38,465,393	1.33E-32	0.396	0.381				*RARA*	TSS200
cg15866977	17	79,919,578	0.005869	0.396	0.420				*NOTUM*	TSS1500
cg05165378	7	99,155,739	6.98E-05	0.396	0.281				*ZNF655*	TSS1500
cg03336334	15	29,407,987	5.75E-08	0.396	0.362				*APBA2*	Body
cg23689428	2	121,223,964	1.88E-07	0.396	0.306				*LOC84931*	TSS200
cg23938220	5	112,073,538	0.001635	0.396	0.232				*APC*	TSS200
cg16909408	3	134,125,688	0.000181	0.396	0.374					
cg08818610	6	24,910,720	2.41E-07	0.396	0.439				*FAM65B*	5'UTR
cg13601435	9	120,507,431	1E-09	0.396	0.370					
cg17949969	4	8,073,790	0.003359	0.396	0.379				*ABLIM2*	Body
cg14736058	4	16,085,606	2.12E-66	0.396	0.386				*PROM1*	TSS200
cg20834178	4	6,944,071	2.87E-10	0.396	0.410				*TBC1D14*	Body
cg13319975	6	146,136,371	0.000146	0.396	0.276				*FBXO30*	TSS1500
cg26530485	1	23,496,304	3.33E-22	0.396	0.399				*LUZP1*	TSS1500
cg07182669	6	2,903,535	3.77E-18	0.396	0.352				*SERPINB9*	1stExon
cg08384314	6	30,711,680	2.18E-20	0.396	0.398				*IER3*	3'UTR
cg07067982	11	57,194,370	1.23E-09	0.396	0.392				*SLC43A3*	5'UTR
cg17696194	11	58,941,051	1.04E-25	0.396	0.384				*DTX4*	Body
cg18846883	12	3,451,308	3.34E-23	0.396	0.389					
cg26764250	8	104,090,700	3.16E-07	0.396	0.448					
cg21723903	12	3,862,575	7.52E-05	0.395	0.357				*EFCAB4B*	TSS1500
cg11452354	6	44,187,052	0.003913	0.395	0.280				*SLC29A1*	TSS200
cg06631944	7	158,365,160	6.05E-26	0.395	0.394				*PTPRN2*	Body
cg24441440	10	120,429,062	1.25E-07	0.395	0.298					
cg11712835	1	201,509,152	6.09E-11	0.395	0.334					
cg00687252	6	30,712,060	1E-05	0.395	0.397				*IER3*	Body
cg20248458	2	21,022,587	0.000356	0.395	0.321				*C2orf43*	5'UTR
cg17379325	17	7,555,411	3.49E-26	0.395	0.384				*ATP1B2*	Body

cg22831607	10	99,531,797	0.030052	0.395	0.264				*SFRP5*	TSS200
cg15553598	5	175,792,973	0.001361	0.395	0.483				*ARL10*	Body
cg09024126	4	16,085,603	2.34E-53	0.395	0.393				*PROM1*	TSS200
cg25456068	17	77,800,718	1.86E-16	0.395	0.421					
cg16703956	5	1,446,217	3.91E-24	0.395	0.379				*SLC6A3*	TSS1500
cg19167683	11	57,194,495	6.98E-05	0.395	0.356				*SLC43A3*	5'UTR
cg20115266	19	51,416,517	3.72E-14	0.395	0.403					
cg07475973	22	42,306,951	4.18E-06	0.395	0.337					
cg02970836	16	3,237,991	7.63E-05	0.395	0.411					
cg23502772	5	43,191,975	1.44E-12	0.395	0.296	7.48E-35	0.242		*MGC42105*	TSS1500
cg14281592	9	129,884,313	2.79E-29	0.395	0.385				*ANGPTL2*	5'UTR
cg01913970	1	45,308,593	0.000185	0.395	0.319				*PTCH2*	1stExon
cg16663419	8	145,033,566	1.28E-10	0.395	0.355				*PLEC1*	Body
cg23555120	12	106,533,863	2.94E-31	0.395	0.386	8.4E-31	0.276		*NUAK1*	TSS200
cg25721451	2	66,662,764	0.000402	0.395	0.440				*MEIS1*	5'UTR
cg17689799	2	27,529,540	2.4E-31	0.395	0.364				*TRIM54*	3'UTR
cg22846914	X	25,041,258	0.003521	0.395	0.365					
cg01582438	20	62,461,159	2.8E-16	0.395	0.425					
cg15232539	8	19,459,672	1.02E-06	0.395	0.290				*CSGALNACT1*	1stExon
cg13000082	2	201,450,527	0.000781	0.395	0.351				*AOX1*	TSS1500
cg05312305	7	99,156,387	0.018961	0.395	0.327				*ZNF655*	1stExon
cg03668718	17	33,701,350	8.06E-08	0.395	0.435				*SLFN11*	TSS1500
cg17052266	7	29,186,183	0.000695	0.395	0.354				*CPVL*	TSS200
cg03397307	12	3,862,423	0.016869	0.394	0.272				*EFCAB4B*	TSS200
cg12725760	1	192,873,912	7.16E-29	0.394	0.400					
cg21654383	2	85,641,076	0.002187	0.394	0.410					
cg11624479	11	9,285,586	7.15E-11	0.394	0.321				*DENND5A*	Body
cg12630147	10	70,321,574	3.76E-14	0.394	0.367				*TET1*	5'UTR
cg15338327	10	101,280,209	1.29E-14	0.394	0.406					
cg02724472	7	102,553,293	2.21E-25	0.394	0.366	9.23E-14	0.233		*LRRC17*	TSS200
cg15958289	11	126,188,994	6.08E-33	0.394	0.381				*DCPS*	Body
cg16001422	8	145,022,842	4.99E-18	0.394	0.344				*PLEC1*	Body
cg24938632	17	46,830,125	1E-123	0.394	0.385					
cg17485454	4	87,281,281	2.6E-07	0.394	0.384				*MAPK10*	5'UTR

cg20787173	6	133,562,475	1.22E-09	0.394	0.444				*EYA4*	TSS200	
cg03310939	7	101,688,730	1.84E-25	0.394	0.369				*CUX1*	Body	
cg14686113	4	54,965,829	2.32E-09	0.394	0.435				*GSX2*	TSS1500	
cg01985390	2	27,529,981	1.13E-17	0.394	0.366				*TRIM54*	3'UTR	
cg19769982	21	26,934,197	0.000571	0.394	0.327				*MIR155HG*	TSS1500	
cg26730416	11	4,233,502	9.78E-08	0.394	0.406						
cg01832036	17	48,050,274	2.17E-23	0.394	0.397				*DLX4*	Body	
cg24643706	6	28,603,779	0.00014	0.394	0.366						
cg10874556	X	100,446,569	1.06E-22	0.394	0.386						
cg25124406	12	89,744,662	6.39E-10	0.394	0.338				*DUSP6*	Body	
cg00759699	19	12,991,918	0.00091	0.393	0.193				*DNASE2*	Body	
cg08325845	6	29,974,886	1.62E-11	0.393	0.402				*HLA-J*	Body	
cg09723979	11	19,736,248	1.32E-09	0.393	0.388				*LOC100126784*	TSS200	
cg26701408	10	102,906,057	9.94E-14	0.393	0.418						
cg14370314	14	23,834,891	0.000295	0.393	0.390				*EFS*	TSS200	
cg25707722	12	63,025,490	9.92E-09	0.393	0.310						
cg01561916	2	43,020,169	1.82E-12	0.393	0.280	5.76E-19	0.315		*HAAO*	TSS1500	
cg17101165	12	115,136,226	2.5E-11	0.393	0.383						
cg07455757	16	54,968,985	0.000536	0.393	0.461						
cg01271455	7	157,362,062	1.24E-17	0.393	0.397				*PTPRN2*	Body	
cg03750144	2	131,595,152	1.53E-19	0.393	0.336						
cg09629657	5	179,220,795	3.52E-26	0.393	0.395				*LTC4S*	TSS200	
cg25223095	3	16,927,003	1.17E-07	0.393	0.259				*PLCL2*	Body	
cg08305551	19	12,978,611	0.000381	0.393	0.374				*MAST1*	Body	
cg23230584	7	95,025,740	4.35E-12	0.393	0.330				*PON3*	TSS200	
cg26718878	7	96,647,882	3.7E-09	0.393	0.385						
cg03826594	12	54,812,085	8.09E-08	0.393	0.402				*ITGA5*	Body	
cg04324758	19	16,436,671	7.1E-68	0.393	0.389				*KLF2*	Body	
cg08857201	15	56,536,865	1.28E-07	0.393	0.255				*RFX7*	TSS1500	
cg20286956	1	39,547,918	1.05E-15	0.393	0.386				*MACF1*	5'UTR	
cg18882819	13	43,566,496	8.22E-05	0.393	0.374				*EPSTI1*	TSS200	
cg06197769	12	12,868,983	2.4E-09	0.392	0.371				*CDKN1B*	TSS1500	
cg12691004	2	88,352,949	7.61E-14	0.392	0.421				*KRCC1*	5'UTR	
cg24846199	7	1,283,915	3.28E-08	0.392	0.350						

cg26348018	X	129,087,487	4.93E-12	0.392	0.380					
cg04207084	7	157,477,846	2.7E-57	0.392	0.378				*PTPRN2*	Body
cg22562494	17	41,607,896	3.53E-33	0.392	0.373				*ETV4*	Body
cg00455424	1	65,468,815	0.002732	0.392	0.252					
cg03146625	12	54,448,729	2.89E-14	0.392	0.377				*HOXC4*	Body
cg15846316	4	57,371,719	4.26E-07	0.392	0.413				*ARL9*	5'UTR
cg16600991	12	54,389,477	0.021003	0.392	0.327					
cg18161327	13	100,627,703	3.1E-06	0.392	0.426					
cg24604013	13	112,721,325	1.37E-15	0.392	0.381				*SOX1*	TSS1500
cg02058870	14	72,053,146	3.57E-16	0.392	0.382				*SIPA1L1*	5'UTR
cg27292588	1	45,285,892	5.97E-06	0.392	0.399				*PTCH2*	3'UTR
cg00840403	16	71,560,205	1.4E-11	0.392	0.386	3.12E-21	0.208		*CHST4*	1stExon
cg07657192	6	29,894,986	1.49E-05	0.392	0.351					
cg20303028	2	74,607,507	7.1E-12	0.392	0.401				*DCTN1*	TSS200
cg03930313	17	36,621,405	1.8E-16	0.392	0.395				*ARHGAP23*	Body
cg09636214	14	73,712,491	5.89E-10	0.392	0.393				*PAPLN*	Body
cg13648318	9	124,991,408	5.34E-05	0.392	0.274				*LHX6*	TSS1500
cg14672994	17	48,503,057	6.27E-34	0.392	0.368	3.99E-33	0.294		*ACSF2*	TSS1500
cg22830113	2	127,783,168	0.003385	0.392	0.260					
cg02227919	2	171,569,132	5.78E-08	0.392	0.360				*LOC440925*	Body
cg19595750	12	133,065,939	0.000896	0.392	0.325				*FBRSL1*	TSS1500
cg18959554	6	44,310,345	2.9E-15	0.392	0.409				*SPATS1*	TSS200
cg01946042	1	21,754,570	3.33E-09	0.392	0.327					
cg00557360	20	37,230,386	7.07E-45	0.392	0.396				*C20orf95*	TSS200
cg12109838	4	103,996,978	4.66E-08	0.392	0.341				*NHEDC2*	5'UTR
cg22817993	1	1,950,415	5.92E-14	0.391	0.353				*GABRD*	TSS1500
cg06560887	11	33,890,371	0.000133	0.391	0.373				*LMO2*	Body
cg00462570	9	98,784,441	1.23E-19	0.391	0.334				*NCRNA00092*	TSS1500
cg20399509	21	47,717,575	1.58E-12	0.391	0.380				*C21orf57*	3'UTR
cg24565274	11	19,736,253	3.76E-07	0.391	0.392				*LOC100126784*	TSS200
cg08663159	4	101,111,872	1.77E-07	0.391	0.370				*DDIT4L*	TSS1500
cg26210445	2	162,273,326	0.015671	0.391	0.402				*TBR1*	1stExon
cg13300480	1	64,485,696	2.38E-16	0.391	0.376				*ROR1*	Body
cg09792204	15	28,342,183	2.76E-08	0.391	0.364				*OCA2*	5'UTR

cg15788639	6	29,974,251	9.39E-08	0.391	0.386				*HLA-J*	Body
cg08481002	11	78,131,601	2.24E-10	0.391	0.383					
cg21790626	19	58,220,494	4.34E-06	0.391	0.334	2.94E-21	0.328		*ZNF154*	5'UTR
cg04153838	12	115,132,015	8.69E-09	0.391	0.415					
cg14262681	20	21,084,420	0.031861	0.391	0.499					
cg06767766	10	70,321,383	2.95E-05	0.391	0.218				*TET1*	5'UTR
cg16970232	5	112,073,433	0.001542	0.391	0.205	5.25E-10	0.259		*APC*	TSS200
cg14361409	14	104,584,098	4.35E-23	0.390	0.345					
cg03354508	6	147,769,498	4.74E-05	0.390	0.471					
cg13133835	12	125,239,803	3.5E-25	0.390	0.403					
cg19053239	2	149,645,394	3.71E-05	0.390	0.399				*KIF5C*	Body
cg04434593	11	67,139,546	3.17E-06	0.390	0.503				*LOC100130987*	Body
cg08457178	6	30,711,636	1.02E-20	0.390	0.392				*IER3*	3'UTR
cg25859972	3	64,670,515	9.44E-09	0.390	0.301				*ADAMTS9*	Body
cg23746347	22	43,058,692	1.92E-05	0.390	0.234					
cg02156380	1	180,882,591	9.16E-35	0.390	0.389				*KIAA1614*	Body
cg00099393	7	157,361,621	4.47E-29	0.390	0.378				*PTPRN2*	Body
cg22761431	17	7,609,416	1.3E-18	0.390	0.344				*EFNB3*	Body
cg02384967	5	159,738,801	6.35E-09	0.390	0.326				*CCNJL*	Body
cg00522883	11	57,194,120	1.54E-24	0.390	0.397				*SLC43A3*	5'UTR
cg15229124	3	139,258,912	7.47E-07	0.390	0.357				*RBP1*	TSS1500
cg03388786	8	56,852,253	1.93E-36	0.390	0.399				*LYN*	5'UTR
cg07745373	11	58,941,415	4.62E-22	0.389	0.376				*DTX4*	Body
cg12549300	13	113,594,776	1.09E-25	0.389	0.361					
cg05911704	X	129,304,869	3.31E-08	0.389	0.276				*RAB33A*	TSS1500
cg12763828	1	9,129,646	2.5E-25	0.389	0.354				*SLC2A5*	5'UTR
cg00347904	6	35,182,508	8.99E-08	0.389	0.375	2.82E-14	0.232		*SCUBE3*	Body
cg14192401	2	233,368,543	8.15E-25	0.389	0.346					
cg14603620	17	38,334,166	1.4E-06	0.389	0.330				*RAPGEFL1*	TSS200
cg23258881	1	150,123,037	0.006021	0.389	0.253				*PLEKHO1*	Body
cg03205103	5	38,257,541	6.02E-10	0.389	0.380				*EGFLAM*	TSS1500
cg05209518	2	227,288,518	5.14E-08	0.389	0.390					
cg18513062	17	763,049	7.22E-14	0.389	0.352				*NXN*	Body
cg09789590	19	46,800,479	2.11E-11	0.389	0.385				*HIF3A*	Body

cg06039161	11	70,672,835	1.42E-28	0.389	0.363				*SHANK2*	Body
cg02709432	8	120,651,236	4.96E-08	0.389	0.442				*ENPP2*	TSS200
cg24098326	10	123,923,050	7.13E-28	0.389	0.380				*TACC2*	Body
cg04510153	1	162,660,951	5.51E-29	0.389	0.372				*DDR2*	5'UTR
cg24035164	10	93,646,991	1.08E-07	0.389	0.344					
cg04452959	8	120,651,190	1.48E-05	0.389	0.396				*ENPP2*	TSS200
cg04804877	12	3,862,225	0.03327	0.389	0.266				*EFCAB4B*	1stExon
cg12435415	3	186,490,344	5.98E-06	0.389	0.420					
cg08378442	2	220,117,704	4.03E-10	0.389	0.393				*TUBA4B*	TSS1500
cg10648670	2	25,439,375	0.005199	0.389	0.299					
cg18674980	8	86,350,581	1.08E-09	0.389	0.363	5.39E-20	0.214		*CA3*	TSS1500
cg10365984	6	91,004,430	1.25E-06	0.389	0.332				*BACH2*	5'UTR
cg12737160	X	107,681,482	4.58E-05	0.389	0.365				*COL4A6*	1stExon
cg12232308	6	30,711,580	6.18E-36	0.389	0.385				*IER3*	3'UTR
cg01499815	6	29,895,074	4.5E-10	0.389	0.371					
cg21951457	10	123,923,450	4.5E-32	0.389	0.378				*TACC2*	Body
cg09109520	16	57,673,258	4.91E-25	0.389	0.367				*GPR56*	5'UTR
cg23660154	1	110,626,540	5.25E-05	0.389	0.366					
cg03367519	5	58,334,910	2.4E-20	0.389	0.388				*PDE4D*	Body
cg17648080	17	2,141,841	8.67E-11	0.389	0.386				*SMG6*	Body
cg27651229	4	780,336	3.39E-31	0.389	0.377				*CPLX1*	Body
cg07417745	2	228,582,488	2.69E-06	0.389	0.263				*SLC19A3*	5'UTR
cg14258341	1	46,914,023	3.54E-12	0.389	0.370					
cg06444282	1	20,669,975	0.000164	0.389	0.355				*VWA5B1*	Body
cg21918559	X	36,976,052	1.04E-12	0.389	0.320					
cg02486904	1	47,999,051	9.23E-26	0.388	0.364					
cg24886257	19	52,452,447	0.002939	0.388	0.397					
cg18607529	7	50,343,869	6.46E-08	0.388	0.378				*IKZF1*	TSS1500
cg22659676	5	139,525,919	1.91E-40	0.388	0.377					
cg02029926	4	74,734,874	7.23E-05	0.388	0.291	5.95E-31	0.263		*CXCL1*	TSS1500
cg00552704	6	32,121,420	4.92E-13	0.388	0.372				*PPT2*	TSS1500
cg23026024	6	29,974,512	7.94E-12	0.388	0.396				*HLA-J*	Body
cg27260772	6	50,791,202	0.000593	0.388	0.459				*TFAP2B*	Body
cg16127594	1	95,393,115	0.006654	0.388	0.171				*CNN3*	TSS1500

cg14207654	1	43,533,913	0.013287	0.388	0.169					
cg08879470	11	76,859,056	0.000604	0.388	0.340			Yes	*MYO7A*	Body
cg04175027	2	123,418,722	3.06E-28	0.388	0.386					
cg13434638	15	68,723,690	6.67E-07	0.388	0.378				*ITGA11*	Body
cg00113096	2	134,119,805	4.71E-15	0.388	0.347				*NCKAP5*	Body
cg22623223	7	157,484,045	8.65E-05	0.388	0.354				*PTPRN2*	Body
cg06970510	12	52,401,448	5.54E-10	0.388	0.383				*GRASP*	Body
cg07220448	7	16,505,592	8.26E-12	0.388	0.366				*SOSTDC1*	TSS200
cg15310857	3	64,253,534	5.07E-17	0.388	0.384					
cg13117164	4	54,960,232	5.53E-19	0.388	0.384					
cg19583819	5	139,283,051	3.73E-20	0.388	0.373				*NRG2*	Body
cg13539545	14	60,973,823	4.89E-05	0.388	0.326					
cg14382750	9	100,264,941	8.17E-11	0.388	0.345				*TMOD1*	5'UTR
cg23363832	3	139,258,939	1.64E-07	0.388	0.371				*RBP1*	TSS1500
cg25713185	2	228,582,486	3.21E-05	0.388	0.258	4.79E-32	0.264		*SLC19A3*	5'UTR
cg09025324	6	116,691,685	7.17E-08	0.388	0.293	2.77E-23	0.239		*DSE*	TSS1500
cg14337655	19	2,282,207	0.002368	0.388	0.322				*C19orf35*	TSS200
cg13421439	18	21,199,515	3.49E-05	0.388	0.386				*ANKRD29*	Body
cg15923947	1	2,461,929	2.6E-124	0.388	0.375				*HES5*	TSS1500
cg14029629	X	49,023,064	8.61E-10	0.388	0.332				*MAGIX*	3'UTR
cg21306329	17	73,083,876	0.00343	0.387	0.199				*SLC16A5*	TSS200
cg10320061	X	153,094,967	2.97E-10	0.387	0.347				*PDZD4*	Body
cg22324567	8	25,899,434	5.01E-16	0.387	0.370				*EBF2*	Body
cg24666993	6	29,974,991	1.16E-10	0.387	0.379				*HLA-J*	Body
cg06480736	5	101,632,321	1.27E-07	0.387	0.382				*SLCO4C1*	TSS200
cg06861841	11	62,212,114	1.06E-12	0.387	0.372				*AHNAK*	Body
cg04098270	15	72,489,686	1.45E-19	0.387	0.318				*GRAMD2*	Body
cg23808946	2	45,169,548	8.13E-07	0.387	0.316				*SIX3*	1stExon
cg23782734	10	3,109,041	2.29E-11	0.387	0.397				*PFKP*	TSS1500
cg20442697	7	102,553,432	1.38E-31	0.387	0.356	1.84E-23	0.275	Yes	*LRRC17*	5'UTR
cg15677957	18	47,088,780	3.11E-08	0.387	0.368				*LIPG*	Body
cg17403609	5	180,075,862	1.36E-20	0.387	0.387				*FLT4*	Body
cg12084388	3	42,056,663	3.13E-24	0.387	0.332					
cg04579966	13	24,846,221	0.026037	0.387	0.099				*SPATA13*	Body

cg00368636	7	64,734,674	3.06E-09	0.387	0.441					
cg21747330	X	122,319,602	3.54E-26	0.387	0.384				*GRIA3*	Body
cg13011388	14	23,834,909	1.45E-10	0.387	0.370				*EFS*	TSS200
cg26747885	17	76,355,701	1.02E-05	0.387	0.365				*SOCS3*	5'UTR
cg14202850	17	43,974,869	7E-22	0.387	0.360				*MAPT*	5'UTR
cg19010054	19	51,416,010	1.17E-18	0.387	0.378					
cg13853894	10	34,393,934	1.05E-15	0.387	0.342					
cg10397527	6	32,977,217	4.26E-06	0.386	0.385				*HLA-DOA*	Body
cg12749863	21	26,934,576	0.004289	0.386	0.339				*MIR155HG*	Body
cg07207490	11	118,492,463	1.91E-19	0.386	0.383				*PHLDB1*	Body
cg02564134	3	125,899,754	4.33E-06	0.386	0.338				*ALDH1L1*	TSS1500
cg14485185	11	118,051,260	8.7E-22	0.386	0.379					
cg17205324	14	23,835,595	1.22E-23	0.386	0.382				*EFS*	TSS1500
cg15244049	6	10,883,376	3.64E-10	0.386	0.416				*GCM2*	TSS1500
cg24158259	11	58,984,544	3.16E-05	0.386	0.341			Yes		
cg22700691	10	21,814,388	0.038524	0.386	0.326				*C10orf140*	5'UTR
cg14329059	11	58,940,964	1.94E-32	0.386	0.391				*DTX4*	Body
cg19214916	X	73,642,734	2.31E-29	0.386	0.357				*SLC16A2*	Body
cg14654886	4	5,710,319	0.001653	0.386	0.338				*EVC2*	5'UTR
cg02822259	17	61,615,504	1.8E-29	0.386	0.345				*KCNH6*	Body
cg15129506	2	27,958,712	7.54E-17	0.386	0.301					
cg11873482	7	97,361,244	2.83E-05	0.386	0.354				*TAC1*	TSS200
cg24512400	19	51,522,561	0.000433	0.386	0.376	6.43E-28	0.283		*KLK10*	5'UTR
cg18009321	20	25,129,457	1.4E-08	0.386	0.370				*LOC284798*	TSS1500
cg24680586	10	105,036,727	0.000547	0.386	0.379				*INA*	TSS200
cg07161429	12	105,114,162	8.45E-06	0.386	0.332				*CHST11*	Body
cg10558887	13	36,919,409	0.016614	0.386	0.470				*SPG20*	5'UTR
cg27219841	16	3,079,423	2.83E-22	0.386	0.392				*CCDC64B*	Body
cg08542865	6	2,842,248	1.41E-06	0.386	0.367				*SERPINB1*	TSS200
cg12506775	10	5,734,571	4.21E-09	0.386	0.346				*C10orf18*	5'UTR
cg14568259	9	127,421,654	0.002754	0.386	0.209				*NR6A1*	Body
cg19807257	1	46,951,354	0.001974	0.386	0.345					
cg03296797	16	84,870,066	2.17E-29	0.386	0.384				*CRISPLD2*	5'UTR
cg25799986	20	44,098,281	5E-08	0.386	0.284	1.04E-27	0.224		*WFDC2*	TSS200

cg06088745	16	54,317,413	0.000941	0.386	0.400				*IRX3*	3'UTR
cg02201914	4	85,299,913	1.82E-12	0.386	0.381					
cg06150195	16	11,275,874	1.47E-09	0.385	0.415				*CLEC16A*	3'UTR
cg12433395	12	106,533,667	1.37E-13	0.385	0.336				*NUAK1*	5'UTR
cg19403029	8	10,587,055	2.6E-15	0.385	0.359				*SOX7*	Body
cg17020562	18	59,560,495	7.56E-14	0.385	0.327				*RNF152*	TSS200
cg14315558	21	26,934,682	0.007972	0.385	0.357				*MIR155HG*	Body
cg19670286	5	137,610,127	0.000382	0.385	0.359				*GFRA3*	1stExon
cg18009325	X	47,441,605	0.000779	0.385	0.390				*SYN1*	Body
cg22035501	5	112,073,398	0.015669	0.385	0.200				*APC*	TSS200
cg02886589	2	39,892,450	0.000703	0.385	0.410				*TMEM178*	TSS1500
cg10614809	7	51,539,190	9.12E-31	0.385	0.379					
cg16859884	19	5,829,172	1.79E-07	0.385	0.261					
cg21116447	6	31,831,803	1.58E-10	0.385	0.367				*NEU1*	TSS1500
cg09866102	16	31,214,134	0.029351	0.385	0.181				*PYCARD*	5'UTR
cg23618344	12	54,423,428	2.02E-09	0.385	0.356				*HOXC4*	5'UTR
cg01163842	14	95,235,125	0.000492	0.385	0.428				*GSC*	Body
cg13455410	9	79,631,906	1.69E-05	0.385	0.357					
cg14781281	6	29,974,868	4.82E-10	0.385	0.393				*HLA-J*	Body
cg11670211	11	89,224,288	5.19E-11	0.385	0.443	2.27E-15	0.186		*NOX4*	5'UTR
cg16561266	17	62,777,777	9.32E-05	0.385	0.296				*LOC146880*	Body
cg16517523	19	42,927,074	7.26E-22	0.385	0.367				*LIPE*	Body
cg09425247	10	52,178,225	0.000332	0.385	0.415				*SGMS1*	5'UTR
cg14175690	1	119,527,638	2.55E-09	0.384	0.328				*TBX15*	5'UTR
cg12763824	X	153,094,947	6.27E-07	0.384	0.315				*PDZD4*	Body
cg14616251	1	24,648,696	1.42E-05	0.384	0.419				*GRHL3*	TSS1500
cg08772752	3	48,632,672	0.000318	0.384	0.414				*COL7A1*	TSS200
cg09500443	5	180,018,465	1.3E-52	0.384	0.385				*SCGB3A1*	5'UTR
cg00842351	9	71,789,653	4.19E-11	0.384	0.360	1.79E-10	0.219		*TJP2*	Body
cg05743713	11	66,885,240	4.6E-07	0.384	0.399				*KDM2A*	TSS1500
cg15998779	3	159,756,690	9.2E-15	0.384	0.399					
cg26233374	5	43,603,643	0.000181	0.384	0.245				*NNT*	5'UTR
cg25459558	6	33,161,290	0.00032	0.384	0.341				*COL11A2*	TSS1500
cg16337138	X	36,976,075	1.91E-08	0.384	0.391					

cg22058855	6	27,064,968	0.001928	0.384	0.332					
cg16807089	7	143,582,499	6.87E-14	0.384	0.407				*FAM115A*	5'UTR
cg06060135	5	3,596,704	0.00023	0.384	0.431				*IRX1*	Body
cg24428325	6	44,529,081	8.12E-07	0.384	0.323					
cg15574705	1	58,715,744	0.000143	0.384	0.389			Yes	*DAB1*	5'UTR
cg14470792	4	183,168,158	0.002761	0.384	0.235					
cg04542030	2	74,375,469	7.65E-21	0.384	0.316				*BOLA3*	TSS1500
cg10446179	6	121,758,653	2.31E-05	0.384	0.433				*GJA1*	5'UTR
cg22340072	8	61,822,740	1.66E-05	0.384	0.384					
cg09806262	10	45,914,949	1.01E-06	0.384	0.377				*ALOX5*	Body
cg20216854	X	19,817,872	2.51E-05	0.384	0.386				*SH3KBP1*	Body
cg13273396	12	81,471,867	5.74E-07	0.383	0.343				*ACSS3*	1stExon
cg26987597	6	1,391,207	0.001902	0.383	0.366				*FOXF2*	1stExon
cg04281464	14	70,014,873	0.000595	0.383	0.344					
cg27395066	17	43,221,220	8.23E-23	0.383	0.364				*ACBD4*	3'UTR
cg00329697	1	45,286,062	1.08E-13	0.383	0.410				*PTCH2*	3'UTR
cg01235820	20	642,782	4.05E-05	0.383	0.335				*SCRT2*	3'UTR
cg07478468	1	85,464,000	3.87E-11	0.383	0.375				*MCOLN2*	TSS1500
cg18552861	2	20,865,845	0.000178	0.383	0.360				*GDF7*	TSS1500
cg12594800	X	129,243,426	5.01E-11	0.383	0.362				*ELF4*	5'UTR
cg17121747	4	683,060	2.37E-22	0.383	0.381				*MFSD7*	TSS200
cg04784699	1	119,870,478	3.05E-26	0.383	0.352					
cg04407470	6	108,489,977	4.35E-06	0.383	0.376				*NR2E1*	Body
cg12689285	20	42,875,765	1.21E-19	0.383	0.304				*GDAP1L1*	TSS200
cg20988073	3	125,900,093	1.8E-15	0.383	0.337				*ALDH1L1*	TSS1500
cg11989330	1	65,468,827	0.00026	0.383	0.255					
cg18085998	1	33,938,224	0.018041	0.383	0.363				*ZSCAN20*	TSS200
cg13212283	12	57,632,120	6.26E-33	0.383	0.353				*NDUFA4L2*	5'UTR
cg23138678	14	75,894,283	0.037915	0.383	0.178				*JDP2*	TSS1500
cg26067203	1	1,935,289	0.000344	0.383	0.421				*KIAA1751*	TSS200
cg03356734	20	37,230,413	1.69E-47	0.383	0.374				*C20orf95*	TSS200
cg20311501	5	112,073,502	0.000326	0.383	0.206	3.48E-10	0.184		*APC*	TSS200
cg13379827	1	65,468,782	0.002489	0.383	0.244					
cg12506930	19	58,220,718	3.16E-09	0.383	0.330				*ZNF154*	TSS200

cg11849086	17	62,776,530	0.00052	0.382	0.301				*LOC146880*	Body
cg16898833	6	26,189,333	0.002172	0.382	0.373				*HIST1H4D*	TSS200
cg24578679	15	74,658,573	1.33E-05	0.382	0.359				*CYP11A1*	Body
cg16361890	5	179,220,545	1.31E-22	0.382	0.356	2.97E-30	0.210		*LTC4S*	TSS1500
cg19696083	12	54,438,419	5.32E-11	0.382	0.301				*HOXC4*	5'UTR
cg06186698	3	138,666,118	0.00526	0.382	0.280				*C3orf72*	5'UTR
cg22984571	3	186,490,656	1.31E-07	0.382	0.377					
cg23305408	1	32,169,701	2.69E-11	0.382	0.342				*COL16A1*	1stExon
cg21159128	1	54,693,933	2.06E-55	0.382	0.383				*SSBP3*	Body
cg01799653	5	10,563,501	6.72E-31	0.382	0.351				*ANKRD33B*	TSS1500
cg23041295	5	15,502,456	2.18E-17	0.382	0.384				*FBXL7*	Body
cg12138678	13	43,566,618	5.98E-06	0.382	0.372				*EPSTI1*	TSS1500
cg00218447	12	54,428,171	3.84E-07	0.382	0.335				*HOXC4*	5'UTR
cg17649385	8	91,997,985	1.01E-07	0.382	0.348					
cg15654259	1	116,371,187	2.33E-05	0.382	0.351					
cg00932276	X	13,587,976	1.72E-05	0.382	0.408	7.85E-24	0.304		*EGFL6*	5'UTR
cg03683587	X	50,557,066	0.031434	0.382	0.408				*SHROOM4*	TSS200
cg12092201	2	43,451,775	3.46E-06	0.382	0.357				*ZFP36L2*	Body
cg06085985	11	830,527	8.17E-09	0.382	0.404				*EFCAB4A*	Body
cg26348514	20	44,098,358	3.78E-06	0.382	0.251				*WFDC2*	TSS200
cg04849842	8	140,714,610	4.91E-08	0.382	0.396				*KCNK9*	Body
cg14110111	9	4,297,626	9.14E-05	0.381	0.273				*GLIS3*	5'UTR
cg11906781	2	28,183,394	1.6E-29	0.381	0.372				*BRE*	Body
cg27014380	3	133,393,454	0.000003	0.381	0.401					
cg27434509	4	5,710,337	0.000182	0.381	0.312				*EVC2*	5'UTR
cg23738624	7	142,553,157	0.007915	0.381	0.322				*EPHB6*	5'UTR
cg16510657	X	100,333,950	6.65E-06	0.381	0.345	3.01E-15	0.228		*TMEM35*	1stExon
cg05933736	13	109,248,331	4.16E-06	0.381	0.282				*MYO16*	TSS200
cg25764899	1	47,697,948	1.18E-06	0.381	0.387					
cg14597214	8	38,585,937	1.1E-18	0.381	0.382				*TACC1*	1stExon
cg25810484	12	54,089,486	2.5E-06	0.381	0.357					
cg10062049	7	116,140,267	1.28E-09	0.381	0.339				*CAV2*	Body
cg23941434	5	147,162,161	7.29E-05	0.381	0.331				*JAKMIP2*	5'UTR
cg08844698	16	85,185,607	1.2E-28	0.381	0.345					

cg08193579	3	169,529,701	6.8E-05	0.381	0.233				*LRRC34*	Body
cg05736236	2	222,435,214	2.24E-16	0.381	0.329				*EPHA4*	Body
cg06909469	2	96,990,800	4.83E-20	0.381	0.374				*ITPRIPL1*	TSS1500
cg27282900	6	30,095,258	3.98E-14	0.381	0.397					
cg17419626	12	53,268,268	1.46E-09	0.381	0.300					
cg00466108	1	65,731,689	0.000286	0.381	0.304				*DNAJC6*	Body
cg27432826	1	110,672,769	4.93E-17	0.381	0.364					
cg10219157	4	2,395,549	6.09E-07	0.381	0.253				*ZFYVE28*	Body
cg22444124	1	226,867,960	7.18E-06	0.381	0.400				*ITPKB*	Body
cg07489502	5	140,811,642	0.000115	0.381	0.351				*PCDHGA4*	Body
cg11940285	3	129,693,385	1.63E-11	0.381	0.381				*TRH*	5'UTR
cg23132268	5	147,162,169	6.01E-06	0.380	0.325				*JAKMIP2*	5'UTR
cg05326023	6	30,418,908	2.09E-09	0.380	0.380					
cg26573704	5	33,892,223	0.000243	0.380	0.345				*ADAMTS12*	TSS200
cg15777964	21	44,847,606	0.002623	0.380	0.320				*SIK1*	TSS1500
cg14777768	3	170,746,265	6.2E-11	0.380	0.335				*SLC2A2*	TSS1500
cg15835232	17	53,343,075	3.17E-10	0.380	0.369				*HLF*	Body
cg16277214	3	32,434,339	1.38E-06	0.380	0.309				*CMTM7*	Body
cg11335133	1	90,309,559	2.99E-05	0.380	0.297				*LRRC8D*	5'UTR
cg14826711	1	169,396,909	4.27E-16	0.380	0.353				*C1orf114*	TSS1500
cg09333221	7	30,804,200	3.31E-25	0.380	0.388					
cg27015057	5	141,703,946	0.001899	0.380	0.390				*SPRY4*	5'UTR
cg00778995	2	105,470,558	0.000904	0.380	0.354				*POU3F3*	TSS1500
cg25524962	13	20,735,819	1.66E-06	0.380	0.376				*GJA3*	TSS1500
cg00338801	1	70,035,208	9.86E-05	0.380	0.401					
cg04394267	3	55,523,950	0.018576	0.380	0.208					
cg15444597	10	103,881,107	2.12E-28	0.380	0.386				*LDB1*	TSS1500
cg03698393	15	89,438,795	1.15E-10	0.380	0.329	8.87E-26	0.204		*HAPLN3*	TSS200
cg19005168	13	27,092,836	1.07E-23	0.380	0.369					
cg00142266	15	77,789,307	0.003211	0.380	0.349					
cg18156204	17	71,948,613	9.51E-12	0.380	0.351					
cg02124446	16	85,783,537	7.22E-20	0.380	0.332				*C16orf74*	5'UTR
cg15523958	2	43,864,929	1.63E-08	0.380	0.304				*PLEKHH2*	5'UTR
cg07557560	5	134,914,642	3.35E-18	0.380	0.322				*CXCL14*	5'UTR

cg17407511	X	38,080,364	1.41E-09	0.380	0.336				*SRPX*	TSS200
cg02288341	5	137,610,226	0.002799	0.380	0.303				*GFRA3*	1stExon
cg13933043	7	130,696,871	9.72E-14	0.380	0.421				*FLJ43663*	Body
cg24543696	11	12,031,046	0.000112	0.380	0.300				*DKK3*	TSS1500
cg04026379	X	47,483,043	0.026266	0.380	0.107					
cg03220455	17	74,582,984	1.84E-19	0.380	0.344				*ST6GALNAC2*	TSS1500
cg08985979	4	183,062,576	1.26E-12	0.380	0.302				*MGC45800*	Body
cg17810176	19	36,035,831	0.002219	0.380	0.171				*GAPDHS*	Body
cg09548780	2	218,770,270	3.06E-27	0.379	0.382				*TNS1*	5'UTR
cg15949044	5	140,864,549	4.85E-08	0.379	0.381				*PCDHGA4*	Body
cg24034005	14	97,059,192	1.26E-06	0.379	0.367					
cg09089242	11	33,892,236	8.88E-19	0.379	0.330				*LMO2*	TSS1500
cg01379240	3	138,666,144	0.004433	0.379	0.299				*C3orf72*	5'UTR
cg18673954	10	105,036,611	2.88E-05	0.379	0.433				*INA*	TSS1500
cg27506442	13	30,948,716	3.94E-16	0.379	0.372				*LOC100188949*	TSS1500
cg22796507	1	47,882,739	2.48E-06	0.379	0.361				*FOXE3*	1stExon
cg19585556	7	18,535,332	1.05E-05	0.379	0.354				*HDAC9*	TSS1500
cg11720950	6	32,223,114	4.36E-12	0.379	0.382					
cg14574728	9	110,187,656	1.43E-10	0.379	0.367					
cg08772003	10	104,629,869	1.02E-14	0.379	0.297				*AS3MT*	Body
cg00685014	11	62,212,195	3.55E-14	0.379	0.386				*AHNAK*	Body
cg07330114	3	11,624,023	3.71E-07	0.379	0.317				*VGLL4*	Body
cg20300175	17	27,918,316	2.4E-55	0.379	0.374					
cg08195538	X	67,914,130	0.000529	0.379	0.359				*STARD8*	5'UTR
cg07114310	7	51,538,921	1.27E-11	0.379	0.332					
cg15410675	4	154,144,800	2E-05	0.379	0.421				*TRIM2*	Body
cg23605961	7	751,331	6.8E-25	0.379	0.390				*PRKAR1B*	5'UTR
cg12002882	20	62,461,365	4.1E-21	0.379	0.343					
cg20895877	14	94,254,586	2.1E-09	0.379	0.275	9.07E-21	0.285		*PRIMA1*	5'UTR
cg11183365	6	27,064,769	4.62E-06	0.379	0.322					
cg23912429	10	17,270,431	2.27E-08	0.379	0.438				*VIM*	5'UTR
cg19943330	14	24,505,554	1.58E-40	0.379	0.375				*DHRS4L1*	TSS200
cg21990316	6	29,894,836	4.94E-11	0.379	0.349				*HCG4P6*	TSS1500
cg26567423	12	54,500,619	1.63E-06	0.379	0.418				*FLJ12825*	Body

cg05998089	6	29,975,141	3.94E-14	0.379	0.367				*HLA-J*	Body
cg22081371	1	177,827,903	0.000937	0.379	0.241					
cg05952494	12	4,378,316	6.88E-06	0.379	0.372					
cg23072161	1	16,489,115	4.87E-09	0.379	0.318					
cg06530563	12	115,132,889	9.19E-10	0.379	0.395					
cg12178980	10	11,206,870	3.43E-06	0.379	0.210				*CUGBP2*	TSS200
cg07627119	15	89,906,172	0.000306	0.378	0.363					
cg24167314	12	103,311,500	0.000713	0.378	0.195				*PAH*	TSS200
cg10257049	5	154,230,308	3.79E-29	0.378	0.368	7.06E-26	0.241		*C5orf4*	TSS200
cg16674860	10	100,206,094	5.45E-05	0.378	0.319				*HPS1*	5'UTR
cg01779765	14	62,583,795	0.001714	0.378	0.393				*FLJ43390*	TSS1500
cg18401367	5	176,107,201	0.00141	0.378	0.427					
cg11010528	6	29,894,813	5.41E-11	0.378	0.368				*HCG4P6*	TSS1500
cg01959730	1	119,548,825	1.3E-05	0.378	0.380					
cg10588135	17	59,329,903	2.75E-21	0.378	0.365				*BCAS3*	Body
cg05835105	4	111,543,401	0.000303	0.378	0.433				*PITX2*	Body
cg24435571	10	104,436,140	1.1E-20	0.378	0.358				*ARL3*	3'UTR
cg11482719	4	57,372,411	1.06E-12	0.378	0.351				*ARL9*	5'UTR
cg08107689	15	29,033,901	2.63E-07	0.378	0.377					
cg09236284	7	97,361,114	1.92E-05	0.378	0.327				*TAC1*	TSS200
cg11640756	6	143,247,810	0.000526	0.378	0.226				*HIVEP2*	5'UTR
cg07104209	7	27,281,872	1.32E-09	0.378	0.308				*EVX1*	TSS1500
cg18031596	8	108,510,292	1.76E-13	0.378	0.390				*ANGPT1*	TSS200
cg17080163	2	123,418,757	1.27E-27	0.378	0.379					
cg23483840	6	29,974,844	1.66E-14	0.378	0.389				*HLA-J*	Body
cg11380530	12	54,389,179	0.006401	0.378	0.363					
cg21251385	20	52,277,050	4.77E-09	0.378	0.388					
cg02900356	16	31,214,494	4.65E-24	0.378	0.315				*PYCARD*	TSS1500
cg24864241	1	204,329,158	1.58E-33	0.378	0.371				*PLEKHA6*	TSS200
cg14301024	2	114,048,932	3.27E-09	0.378	0.356				*LOC440839*	Body
cg11809014	17	39,067,081	2.04E-16	0.378	0.383					
cg21448423	1	55,013,956	1.83E-19	0.377	0.364	6.01E-14	0.169		*ACOT11*	1stExon
cg12615766	8	59,058,273	1.41E-37	0.377	0.370				*FAM110B*	5'UTR
cg21737039	4	8,271,391	8.22E-09	0.377	0.336				*HTRA3*	TSS200

cg26268701	6	29,894,737	0.012228	0.377	0.494				*HCG4P6*	TSS1500
cg02535674	20	52,226,352	0.00778	0.377	0.291					
cg14115936	7	73,002,496	1.52E-09	0.377	0.347					
cg08450752	15	73,611,451	3.97E-08	0.377	0.363					
cg24161793	2	96,990,762	1.93E-21	0.377	0.355				*ITPRIPL1*	TSS1500
cg10765212	2	190,044,638	1.78E-18	0.377	0.353				*COL5A2*	TSS200
cg21215929	5	14,873,039	3.68E-06	0.377	0.263				*ANKH*	TSS1500
cg23685759	10	98,945,131	2.05E-13	0.377	0.300				*SLIT1*	Body
cg16217794	12	100,579,233	5.33E-23	0.377	0.370					
cg17032602	2	129,076,392	5.31E-18	0.377	0.326				*HS6ST1*	TSS1500
cg04781339	21	44,781,686	2.32E-07	0.377	0.341					
cg26786795	14	20,344,394	5.27E-35	0.377	0.350			Yes	*OR4K2*	TSS200
cg09495977	4	8,271,507	2.54E-06	0.377	0.259	1.18E-21	0.216		*HTRA3*	5'UTR
cg12211867	1	108,023,441	7.12E-13	0.377	0.332				*NTNG1*	Body
cg05152874	X	50,557,122	0.02514	0.377	0.460				*SHROOM4*	TSS200
cg14020762	7	100,465,657	5.87E-24	0.377	0.382				*TRIP6*	Body
cg19667811	10	30,405,328	1.03E-17	0.377	0.361					
cg02852959	12	2,944,436	1.29E-07	0.377	0.258				*NRIP2*	TSS1500
cg17410236	14	85,996,495	1.93E-05	0.377	0.331	8.64E-19	0.253		*FLRT2*	1stExon
cg06148118	11	33,892,246	1.13E-08	0.377	0.303				*LMO2*	TSS1500
cg03143849	11	2,904,951	5.53E-42	0.377	0.346	1.51E-15	0.259		*CDKN1C*	3'UTR
cg17777432	5	172,068,999	4.25E-12	0.377	0.319				*NEURL1B*	Body
cg09115984	16	31,214,426	9.82E-18	0.377	0.297				*PYCARD*	TSS200
cg26115228	2	85,116,113	1.67E-23	0.377	0.385					
cg01720033	17	62,777,744	6.64E-07	0.377	0.310				*LOC146880*	Body
cg21225170	5	137,610,391	0.003	0.377	0.296				*GFRA3*	TSS200
cg13634736	11	3,181,575	7.21E-21	0.376	0.332				*OSBPL5*	5'UTR
cg21304453	20	3,218,794	7.95E-37	0.376	0.358				*SLC4A11*	TSS1500
cg19729649	17	79,919,727	5.5E-15	0.376	0.368				*NOTUM*	TSS1500
cg22549041	15	75,019,251	2.84E-19	0.376	0.371				*CYP1A1*	TSS1500
cg13331354	19	18,539,841	6.99E-06	0.376	0.260				*SSBP4*	Body
cg08332908	17	38,465,326	3.39E-31	0.376	0.370				*RARA*	TSS200
cg00506168	21	45,148,460	0.004016	0.376	0.254				*PDXK*	Body
cg26416887	12	111,618,925	2.23E-10	0.376	0.307				*CUX2*	Body

cg18547371	19	18,539,843	7.19E-06	0.376	0.275				*SSBP4*	Body
cg10373003	17	18,057,079	0.000576	0.376	0.277				*MYO15A*	Body
cg19414598	22	38,965,745	2.81E-09	0.376	0.375				*DMC1*	5'UTR
cg22007861	19	2,282,439	2.71E-20	0.376	0.374				*C19orf35*	TSS1500
cg01268824	19	58,220,818	5.82E-07	0.376	0.353				*ZNF154*	TSS1500
cg06908244	14	100,126,358	5.66E-05	0.376	0.379				*HHIPL1*	Body
cg26469991	X	51,139,481	2.54E-06	0.376	0.353					
cg15423357	2	25,149,977	6.26E-08	0.376	0.423					
cg26315984	1	173,176,501	4.07E-14	0.376	0.374				*TNFSF4*	TSS200
cg03420881	2	130,763,516	2.39E-12	0.376	0.405					
cg12368612	12	115,135,999	4.72E-19	0.376	0.361					
cg25908973	15	45,670,865	3.59E-06	0.376	0.388				*LOC145663*	TSS200
cg14903097	2	21,023,042	0.001081	0.376	0.344				*C2orf43*	TSS1500
cg02320862	17	75,370,284	3.22E-17	0.376	0.307				*SEPT9*	5'UTR
cg09668344	11	77,122,506	8.45E-24	0.376	0.364				*PAK1*	5'UTR
cg03476673	16	84,870,203	4.01E-27	0.376	0.361				*CRISPLD2*	5'UTR
cg26777760	X	132,092,564	0.033648	0.376	0.236				*HS6ST2*	Body
cg20902783	14	21,494,071	4.08E-20	0.376	0.355				*NDRG2*	TSS1500
cg21890726	15	68,260,574	0.023121	0.376	0.052					
cg18005828	10	120,031,911	1.33E-09	0.376	0.318					
cg16288089	7	97,361,408	9.07E-05	0.376	0.366				*TAC1*	5'UTR
cg00744866	3	33,701,209	1.68E-06	0.376	0.269				*CLASP2*	Body
cg18435532	19	36,024,802	6.47E-07	0.376	0.336				*GAPDHS*	Body
cg21773872	7	30,722,320	0.002715	0.376	0.523				*CRHR2*	TSS200
cg18914211	6	29,795,815	4.25E-06	0.375	0.392				*HLA-G*	Body
cg23497020	12	50,616,562	3.25E-17	0.375	0.361				*LIMA1*	Body
cg20337996	12	124,780,809	1.79E-15	0.375	0.288				*FAM101A*	5'UTR
cg02590710	X	56,258,440	4.08E-16	0.375	0.354				*KLF8*	TSS1500
cg18653350	13	24,007,836	0.006922	0.375	0.274				*SACS*	5'UTR
cg09793609	X	119,445,399	4.4E-15	0.375	0.343				*FAM70A*	TSS200
cg01072076	5	1,930,801	0.014078	0.375	0.493					
cg05347878	9	98,784,231	0.002839	0.375	0.273				*NCRNA00092*	TSS200
cg21963535	20	44,098,285	1.26E-05	0.375	0.260				*WFDC2*	TSS200
cg19349369	6	2,842,146	8.99E-06	0.375	0.374				*SERPINB1*	TSS200

cg23720384	21	23,747,055	9.43E-15	0.375	0.323					
cg16937769	12	54,409,485	7.57E-06	0.375	0.372				*HOXC4*	TSS1500
cg24647015	11	114,191,367	1.73E-14	0.375	0.358					
cg09421111	16	57,673,122	2.5E-07	0.375	0.403				*GPR56*	5'UTR
cg20950465	1	53,068,181	9.28E-05	0.375	0.171				*GPX7*	1stExon
cg10101773	14	75,894,136	7.68E-20	0.375	0.323				*JDP2*	TSS1500
cg17034390	6	30,095,326	3.11E-13	0.375	0.356					
cg03515663	7	157,478,510	5.53E-30	0.375	0.363				*PTPRN2*	Body
cg05475524	11	66,326,798	6.27E-18	0.375	0.378				*ACTN3*	Body
cg14772439	6	29,911,104	0.016157	0.375	0.175				*HLA-A*	Body
cg22940848	1	2,222,486	1.14E-11	0.375	0.379				*SKI*	Body
cg19356191	1	116,371,337	0.000724	0.375	0.378					
cg05770238	1	26,686,587	1.08E-09	0.375	0.409					
cg00613284	8	67,419,936	1.86E-25	0.375	0.383				*C8orf46*	Body
cg12031863	16	4,587,854	3.42E-24	0.375	0.351				*C16orf5*	5'UTR
cg15673034	18	3,499,093	0.011084	0.375	0.257				*DLGAP1*	3'UTR
cg15375469	7	29,186,231	2.38E-05	0.375	0.343				*CPVL*	TSS200
cg24879335	3	133,465,180	0.000136	0.375	0.276				*TF*	5'UTR
cg14121282	9	137,268,074	1.4E-24	0.375	0.365				*RXRA*	Body
cg07541559	11	34,288,225	1.77E-26	0.374	0.353				*ABTB2*	Body
cg24431872	4	11,429,036	2.6E-31	0.374	0.375				*HS3ST1*	5'UTR
cg03917666	10	3,977,608	2.95E-23	0.374	0.360					
cg21286782	1	113,265,602	2.75E-59	0.374	0.368				*FAM19A3*	Body
cg27496526	15	41,805,530	1.34E-10	0.374	0.358				*LTK*	Body
cg01889574	11	44,590,889	1.03E-05	0.374	0.388				*CD82*	5'UTR
cg03051777	12	115,131,789	5.21E-10	0.374	0.425					
cg08613144	2	133,428,836	1.96E-06	0.374	0.404				*LYPD1*	1stExon
cg09180171	1	22,677,913	1.2E-05	0.374	0.336					
cg05721365	17	62,777,693	0.00038	0.374	0.281				*LOC146880*	Body
cg18502142	7	96,622,709	4.91E-11	0.374	0.364				*DLX6AS*	Body
cg05864326	2	177,030,150	0.003484	0.374	0.269				*HOXD3*	5'UTR
cg25974901	1	20,396,750	9.28E-47	0.374	0.388				*PLA2G5*	1stExon
cg21079345	7	134,144,036	0.000325	0.374	0.227				*AKR1B1*	TSS200
cg18056600	17	4,642,047	4.47E-10	0.374	0.225	3.89E-17	0.213		*ZMYND15*	TSS1500

cg22635676	2	241,975,971	4.57E-10	0.374	0.357				*SNED1*	Body
cg25831075	3	164,915,196	0.001698	0.374	0.240				*SLITRK3*	TSS1500
cg18383538	6	28,557,823	6.27E-08	0.374	0.389					
cg20415170	5	95,298,066	0.024825	0.374	0.237				*ELL2*	TSS1500
cg21554552	3	50,378,425	7.62E-13	0.374	0.378	7.8E-11	0.165		*RASSF1*	TSS200
cg15717183	3	181,438,122	1.4E-20	0.374	0.372				*SOX2OT*	Body
cg21042456	7	1,270,558	2.91E-06	0.374	0.354					
cg04909938	4	6,676,273	8.01E-09	0.374	0.379				*LOC93622*	Body
cg00158308	13	97,646,341	7.53E-05	0.374	0.368	1.85E-24	0.252		*OXGR1*	5'UTR
cg17932631	2	96,990,861	9.83E-16	0.374	0.323				*ITPRIPL1*	TSS1500
cg06488957	17	17,628,152	1.23E-66	0.374	0.388				*RAI1*	5'UTR
cg16695890	19	15,580,310	2.31E-13	0.374	0.362				*PGLYRP2*	Body
cg22869726	21	34,398,265	3.29E-09	0.374	0.391				*OLIG2*	1stExon
cg11044163	7	29,186,153	0.00587	0.374	0.367	2.22E-10	0.163		*CPVL*	TSS200
cg07904073	10	74,017,122	7.82E-17	0.374	0.379					
cg05476483	11	76,750,693	4.76E-17	0.374	0.367				*B3GNT6*	Body
cg21686812	6	168,078,911	1.21E-07	0.374	0.297					
cg00808305	1	7,434,171	2.5E-06	0.374	0.312				*CAMTA1*	Body
cg03248158	4	122,632,918	7.1E-06	0.374	0.346					
cg03126561	11	12,434,050	9.76E-17	0.373	0.360				*PARVA*	Body
cg10087036	16	19,130,461	2.05E-12	0.373	0.369				*ITPRIPL2*	1stExon
cg15575433	7	100,488,705	5.76E-05	0.373	0.396				*UFSP1*	TSS1500
cg17231524	X	134,556,505	6.41E-18	0.373	0.319	2.07E-32	0.333		*NCRNA00086*	Body
cg27117792	12	102,330,180	1.21E-11	0.373	0.386					
cg10249375	1	63,795,934	0.004754	0.373	0.388					
cg05986933	2	43,020,457	8.25E-15	0.373	0.333				*HAAO*	TSS1500
cg18344652	1	95,393,096	0.009585	0.373	0.149				*CNN3*	TSS1500
cg25054907	17	61,553,954	2.31E-22	0.373	0.325				*ACE*	TSS1500
cg10790698	19	18,539,756	0.000243	0.373	0.241				*SSBP4*	Body
cg11178148	11	116,371,781	1.38E-16	0.373	0.359					
cg15963417	12	103,311,300	0.000816	0.373	0.182	5.87E-14	0.210		*PAH*	5'UTR
cg22389730	1	161,697,241	0.00542	0.373	0.363				*FCRLB*	Body
cg22122174	6	44,187,055	0.004182	0.373	0.279				*SLC29A1*	TSS200
cg06025456	6	32,120,863	1.58E-07	0.373	0.365				*PPT2*	TSS1500

cg04153784	7	127,672,235	8.74E-05	0.373	0.256				*SND1*	Body
cg09944213	16	5,037,570	1.32E-07	0.373	0.375				*SEC14L5*	Body
cg11828180	1	41,848,985	8.91E-05	0.373	0.285					
cg13546935	2	29,033,780	0.010708	0.373	0.199				*SPDYA*	5'UTR
cg26870725	19	12,978,805	2.1E-08	0.373	0.329				*MAST1*	Body
cg12630714	4	54,975,894	1.96E-15	0.373	0.372					
cg27326642	6	26,189,001	0.016693	0.373	0.336				*HIST1H4D*	1stExon
cg17227967	20	9,496,556	9.1E-07	0.373	0.322				*C20orf103*	Body
cg07858113	6	30,711,561	4.07E-22	0.373	0.371				*IER3*	3'UTR
cg01250960	16	85,783,863	1.91E-19	0.373	0.324				*C16orf74*	5'UTR
cg06922635	4	57,371,607	9.04E-06	0.373	0.370				*ARL9*	5'UTR
cg02896496	X	54,385,394	4.35E-13	0.373	0.366				*WNK3*	TSS1500
cg08787251	7	27,281,964	5.71E-14	0.372	0.300				*EVX1*	TSS200
cg10916998	6	30,711,832	0.000424	0.372	0.362				*FLOT1*	TSS1500
cg17696629	8	94,713,190	0.000442	0.372	0.292				*FAM92A1*	Body
cg03428864	3	25,469,925	1.7E-25	0.372	0.365				*RARB*	5'UTR
cg03923561	12	54,447,220	2.76E-14	0.372	0.370				*HOXC4*	5'UTR
cg12768523	4	169,737,224	1.82E-17	0.372	0.347				*PALLD*	Body
cg05393297	12	53,359,155	1.48E-28	0.372	0.386					
cg12420900	10	123,922,863	3.49E-15	0.372	0.361				*TACC2*	TSS1500
cg17001034	1	55,008,516	4.49E-16	0.372	0.304					
cg23489434	12	54,500,175	9.16E-12	0.372	0.282				*FLJ12825*	Body
cg26061177	3	64,670,562	2.85E-06	0.372	0.290				*ADAMTS9*	Body
cg17029019	17	1,959,124	0.000593	0.372	0.268				*HIC1*	TSS1500
cg26213453	15	89,455,860	1.81E-06	0.372	0.327				*MFGE8*	Body
cg09308026	16	30,041,884	5.74E-06	0.372	0.395				*FAM57B*	5'UTR
cg02099543	22	45,899,186	0.000371	0.372	0.330				*FBLN1*	Body
cg24346905	1	108,023,249	1.36E-10	0.372	0.333				*NTNG1*	Body
cg26638444	5	150,400,180	6.38E-05	0.372	0.211				*GPX3*	5'UTR
cg15835396	7	128,809,228	0.000154	0.372	0.350					
cg26158897	15	53,083,135	1.01E-12	0.372	0.338				*ONECUT1*	TSS1500
cg24569637	2	201,450,560	0.00582	0.372	0.359				*AOX1*	TSS200
cg20383155	6	31,831,428	8.68E-09	0.372	0.339				*NEU1*	TSS1500
cg05192452	5	71,852,759	0.003254	0.372	0.251					

cg06126713	7	16,505,617	7E-07	0.372	0.374				*SOSTDC1*	TSS200
cg25123632	6	29,975,154	2.19E-14	0.372	0.356				*HLA-J*	Body
cg20293594	7	27,196,296	2.69E-05	0.372	0.333				*HOXA7*	TSS200
cg21111256	19	41,386,507	1.29E-14	0.372	0.325				*CYP2A7*	Body
cg09644722	17	60,885,519	2.04E-11	0.372	0.335	2.85E-27	0.262		*MARCH10*	5'UTR
cg09784335	4	41,875,193	1.76E-08	0.372	0.380					
cg00515457	19	35,396,203	7.09E-06	0.372	0.343					
cg21362478	9	78,507,352	0.000245	0.372	0.365				*PCSK5*	Body
cg13129612	14	24,505,547	3.96E-23	0.372	0.364				*DHRS4L1*	TSS200
cg20743134	3	69,061,356	6.47E-17	0.371	0.363				*C3orf64*	5'UTR
cg07068756	4	41,258,910	7.14E-06	0.371	0.362				*UCHL1*	1stExon
cg25456593	11	70,672,858	1.19E-39	0.371	0.354				*SHANK2*	Body
cg23882862	11	111,169,795	0.00047	0.371	0.393				*C11orf92*	5'UTR
cg25023596	12	118,627,943	1.13E-21	0.371	0.379				*TAOK3*	Body
cg19759135	5	179,221,059	1.09E-72	0.371	0.360				*LTC4S*	1stExon
cg22282405	6	50,810,682	3.97E-10	0.371	0.380				*TFAP2B*	Body
cg05635169	5	113,589,213	8.22E-30	0.371	0.336					
cg17670641	X	49,023,115	1.49E-08	0.371	0.303				*MAGIX*	3'UTR
cg05068206	6	30,095,195	9.44E-18	0.371	0.356					
cg17693420	7	99,157,059	0.012905	0.371	0.302				*ZNF655*	5'UTR
cg00474080	6	30,923,243	0.000687	0.371	0.427					
cg10715272	1	39,981,410	7E-21	0.371	0.390				*BMP8A*	Body
cg03432338	1	231,176,985	0.008191	0.371	0.328				*FAM89A*	TSS1500
cg21858255	12	104,609,609	2.93E-05	0.371	0.368				*TXNRD1*	1stExon
cg08276645	2	241,459,502	4.02E-19	0.371	0.351				*ANKMY1*	Body
cg00922748	12	2,944,322	0.00098	0.371	0.218				*NRIP2*	TSS200
cg26022183	1	156,215,655	0.016491	0.371	0.293				*PAQR6*	5'UTR
cg27633530	7	50,344,294	9.35E-12	0.371	0.369				*IKZF1*	TSS200
cg14588642	11	67,139,423	1.66E-23	0.371	0.395				*LOC100130987*	Body
cg18177414	7	149,389,929	0.006937	0.371	0.414					
cg02228815	15	31,685,131	1.46E-39	0.371	0.338					
cg09448677	11	70,672,740	2.62E-37	0.371	0.362				*SHANK2*	Body
cg17841765	1	22,560,699	2.63E-11	0.371	0.374					
cg14118515	2	176,948,728	0.038547	0.371	0.394				*EVX2*	TSS200

cg14820415	2	6,121,680	3.11E-05	0.371	0.340				*LOC400940*	TSS1500
cg16867584	19	49,200,180	1.54E-06	0.371	0.388				*FUT2*	5'UTR
cg24182521	20	44,541,013	7.64E-10	0.371	0.354				*PLTP*	TSS1500
cg14135025	5	55,776,721	0.020974	0.370	0.106					
cg19214408	10	111,981,687	1.2E-08	0.370	0.350				*MXI1*	Body
cg03207574	10	25,241,260	0.000445	0.370	0.385				*PRTFDC1*	Body
cg10572943	21	34,775,045	0.004329	0.370	0.215				*IFNGR2*	TSS200
cg19887700	12	115,136,200	2.63E-17	0.370	0.351					
cg23511118	3	67,840,535	1.29E-09	0.370	0.406					
cg10696085	12	50,444,522	3.11E-10	0.370	0.372					
cg19186145	2	45,169,562	1.68E-06	0.370	0.350				*SIX3*	1stExon
cg10992014	6	121,758,817	2.8E-07	0.370	0.374				*GJA1*	5'UTR
cg01559356	19	2,282,568	4.75E-13	0.370	0.354				*C19orf35*	TSS1500
cg19981568	14	60,977,300	5.73E-05	0.370	0.333				*SIX6*	Body
cg01292793	7	73,002,385	2.03E-17	0.370	0.325					
cg17255450	4	5,710,372	0.000424	0.370	0.299				*EVC2*	5'UTR
cg27616227	8	108,510,314	1.53E-11	0.370	0.371				*ANGPT1*	TSS200
cg26145959	6	44,310,257	3.3E-13	0.370	0.373				*SPATS1*	TSS200
cg18740893	3	136,538,934	0.012114	0.370	0.193				*TMEM22*	5'UTR
cg23152641	7	127,809,009	1.77E-05	0.370	0.359					
cg11942956	6	133,562,470	0.000006	0.370	0.426				*EYA4*	TSS200
cg03568858	7	100,465,221	7.56E-27	0.370	0.347				*TRIP6*	1stExon
cg20157753	2	113,594,343	2.32E-09	0.370	0.402				*IL1B*	1stExon
cg18049167	6	32,121,261	3.39E-10	0.370	0.357				*PPT2*	TSS200
cg19820372	11	111,411,216	4.61E-12	0.370	0.298				*LAYN*	TSS200
cg02077027	X	19,817,931	0.001363	0.370	0.360				*SH3KBP1*	Body
cg17727989	9	27,529,519	0.000871	0.370	0.167				*MOBKL2B*	5'UTR
cg23194719	10	52,177,823	7.76E-07	0.370	0.378				*SGMS1*	5'UTR
cg20840795	12	120,242,394	1.86E-21	0.370	0.362				*CIT*	Body
cg25104124	10	74,021,022	4.91E-08	0.370	0.349					
cg21982950	5	1,876,200	8.51E-21	0.370	0.361					
cg21879102	12	120,241,409	9.36E-19	0.370	0.356				*CIT*	Body
cg10848272	14	70,653,719	3.05E-44	0.370	0.360				*SLC8A3*	5'UTR
cg14753356	6	30,720,108	1.72E-11	0.370	0.276					

cg26968962	16	87,418,097	8.15E-12	0.370	0.316				*FBXO31*	TSS1500
cg13273147	X	153,192,582	0.000358	0.370	0.326				*ARHGAP4*	TSS1500
cg09881545	3	5,137,630	2.46E-15	0.370	0.375					
cg07569918	1	212,002,970	5.09E-37	0.370	0.362				*LPGAT1*	5'UTR
cg07641160	6	117,086,850	0.007392	0.370	0.277				*FAM162B*	1stExon
cg02245794	8	38,585,881	2.15E-08	0.369	0.337				*TACC1*	1stExon
cg02337836	1	110,210,641	5.18E-09	0.369	0.365				*GSTM2*	TSS200
cg06217769	17	2,126,306	3.82E-15	0.369	0.359				*SMG6*	Body
cg24954684	12	122,907,641	2.11E-19	0.369	0.318				*CLIP1*	TSS1500
cg26647332	10	31,073,467	0.000452	0.369	0.217					
cg06635952	2	70,025,869	2.08E-23	0.369	0.359				*ANXA4*	Body
cg25203962	3	170,746,291	4.51E-11	0.369	0.349					
cg17812788	17	40,333,009	0.001546	0.369	0.278				*KCNH4*	1stExon
cg13997668	10	73,847,532	2.44E-06	0.369	0.377				*SPOCK2*	Body
cg08217285	17	27,917,879	1.2E-19	0.369	0.360				*GIT1*	TSS1500
cg09092054	22	19,511,987	0.005047	0.369	0.392				*CLDN5*	1stExon
cg11641791	17	38,821,429	0.004402	0.369	0.347				*KRT222*	TSS200
cg27533119	7	124,404,472	1.01E-09	0.369	0.286				*GPR37*	1stExon
cg22498143	19	40,993,991	2.56E-05	0.369	0.290				*SPTBN4*	Body
cg10976772	1	6,390,595	4.58E-15	0.369	0.349				*ACOT7*	Body
cg08952506	2	201,450,610	7.39E-08	0.369	0.325				*AOX1*	TSS200
cg02006615	7	157,485,230	9.22E-21	0.369	0.361				*PTPRN2*	Body
cg04305621	17	73,084,263	1.49E-44	0.369	0.356				*SLC16A5*	5'UTR
cg22795891	2	192,543,908	0.001087	0.369	0.216				*OBFC2A*	Body
cg11207763	11	69,517,471	1.41E-10	0.369	0.394				*FGF19*	Body
cg00082219	2	219,846,299	1.01E-58	0.369	0.371				*FEV*	3'UTR
cg00017062	15	70,669,548	6.55E-11	0.369	0.363					
cg07121644	11	46,383,031	1.64E-38	0.369	0.359				*DGKZ*	Body
cg14270292	6	133,562,494	3.13E-06	0.369	0.446				*EYA4*	5'UTR
cg26480862	22	28,839,667	1.54E-09	0.369	0.323				*TTC28*	Body
cg20752903	17	60,885,671	0.000344	0.369	0.321				*MARCH10*	5'UTR
cg10824705	2	149,645,724	4.19E-10	0.369	0.380				*KIF5C*	Body
cg14957718	3	50,243,260	1.2E-22	0.369	0.337				*SLC38A3*	5'UTR
cg15012484	6	3,232,246	0.021714	0.369	0.213					

cg05783185	8	119,121,281	3.25E-08	0.369	0.415				*EXT1*	Body
cg09428251	8	68,658,408	6.82E-10	0.369	0.372				*CPA6*	1stExon
cg10758286	1	15,481,367	2.61E-20	0.369	0.301				*TMEM51*	5'UTR
cg01528052	6	30,095,728	5.89E-06	0.368	0.469					
cg25003777	6	2,841,626	3.94E-08	0.368	0.309				*SERPINB1*	5'UTR
cg01176363	20	62,369,445	0.005713	0.368	0.371				*LIME1*	Body
cg00063471	15	51,385,970	0.001067	0.368	0.411				*TNFAIP8L3*	Body
cg00730887	4	160,149,604	2.4E-25	0.368	0.354					
cg15198729	X	100,446,496	6.94E-12	0.368	0.361					
cg04006323	4	185,937,730	0.000001	0.368	0.362					
cg18573327	12	115,123,875	4.59E-15	0.368	0.320					
cg17180237	1	119,545,275	2.89E-11	0.368	0.311					
cg02259324	15	78,111,196	0.000217	0.368	0.308					
cg01814191	12	89,744,524	1.05E-15	0.368	0.332				*DUSP6*	Body
cg21950525	7	99,155,767	0.002224	0.368	0.281				*ZNF655*	TSS1500
cg07486895	18	59,560,433	7.2E-10	0.368	0.288				*RNF152*	TSS200
cg18617005	5	140,787,504	0.007575	0.368	0.489				*PCDHGA4*	Body
cg13372456	5	153,569,246	0.031645	0.368	0.169				*GALNT10*	TSS1500
cg01244877	X	153,598,902	4.13E-07	0.368	0.217				*FLNA*	Body
cg01985965	1	2,461,278	3.83E-14	0.368	0.350				*HES5*	Body
cg07817783	5	10,761,669	0.011518	0.368	0.171	1.6E-11	0.116		*DAP*	TSS1500
cg20583316	7	100,770,476	1.01E-05	0.368	0.372				*SERPINE1*	5'UTR
cg01240444	12	124,247,047	0.001125	0.368	0.130				*DNAH10*	1stExon
cg06132028	6	133,562,466	2.23E-07	0.368	0.426				*EYA4*	TSS200
cg24500959	10	99,474,813	2.12E-14	0.368	0.316				*MARVELD1*	Body
cg09155145	5	1,875,188	2.19E-17	0.368	0.356					
cg09971314	6	28,415,023	4.45E-05	0.368	0.310					
cg21730858	6	138,806,320	6.4E-11	0.368	0.293				*NHSL1*	Body
cg14657277	2	123,419,093	6.39E-35	0.368	0.370					
cg06616051	X	153,094,332	6.19E-26	0.368	0.362				*PDZD4*	Body
cg12200124	5	179,220,820	1.42E-38	0.368	0.360				*LTC4S*	TSS200
cg06732395	5	180,018,722	3.75E-33	0.368	0.351				*SCGB3A1*	TSS1500
cg11537946	4	139,773,369	1.14E-08	0.368	0.339					
cg14913111	1	45,792,733	0.000143	0.368	0.347				*HPDL*	1stExon

cg25423416	22	46,934,860	1.07E-13	0.367	0.335					
cg18233416	5	141,705,411	0.005671	0.367	0.213				*SPRY4*	TSS1500
cg06918474	5	126,205,081	1.46E-24	0.367	0.361				*MARCH3*	3'UTR
cg26683398	5	179,220,589	2.45E-33	0.367	0.350				*LTC4S*	TSS1500
cg09363735	6	50,808,699	5.83E-11	0.367	0.391				*TFAP2B*	Body
cg27188703	12	50,297,581	1.12E-07	0.367	0.259	1.27E-21	0.245		*FAIM2*	1stExon
cg07935264	2	113,594,375	9.2E-12	0.367	0.348	3.82E-24	0.172		*IL1B*	TSS200
cg01462545	4	42,401,242	5.81E-05	0.367	0.362				*SHISA3*	Body
cg08848711	X	118,827,659	0.006697	0.367	0.231				*SEPT6*	TSS1500
cg14599729	17	15,686,294	0.005775	0.367	0.243					
cg15125338	17	44,899,394	1.09E-16	0.367	0.358					
cg15817960	12	54,409,599	2.88E-06	0.367	0.296				*HOXC4*	TSS1500
cg12403148	X	154,842,718	5.9E-07	0.367	0.310				*TMLHE*	TSS200
cg22653140	10	81,214,225	1.56E-09	0.367	0.356					
cg00076195	5	140,892,677	1.03E-08	0.367	0.260					
cg06080793	6	1,625,457	1.42E-26	0.367	0.338				*GMDS*	Body
cg06710082	6	29,943,408	5.28E-08	0.367	0.359				*HCG9*	Body
cg13077262	6	44,310,350	3.2E-31	0.367	0.354				*SPATS1*	TSS200
cg24304789	2	43,451,423	0.000742	0.367	0.309				*ZFP36L2*	3'UTR
cg16993043	1	200,008,026	1.56E-16	0.367	0.385				*NR5A2*	Body
cg07400877	20	45,440,085	6.94E-07	0.367	0.319					
cg00397324	21	42,798,594	0.005198	0.367	0.378				*MX1*	5'UTR
cg24784416	4	169,525,923	0.001146	0.367	0.267			Yes	*PALLD*	Body
cg07243941	4	47,840,162	0.005843	0.367	0.356				*CORIN*	TSS200
cg15119221	17	19,290,755	1.55E-30	0.367	0.368				*MFAP4*	TSS1500
cg02231066	3	52,567,130	0.045014	0.367	0.143				*NT5DC2*	Body
cg01470551	13	95,620,874	1.53E-06	0.367	0.352					
cg16409339	1	146,556,239	1.6E-08	0.367	0.348					
cg18697991	3	64,823,081	1.75E-22	0.367	0.354				*MIR548A2*	Body
cg20059012	12	53,613,154	2.26E-06	0.367	0.386				*RARG*	Body
cg25153755	X	47,433,716	0.000466	0.367	0.372				*SYN1*	Body
cg18945842	22	42,306,589	0.017512	0.367	0.334					
cg20686234	17	26,699,485	0.000943	0.367	0.242				*SARM1*	1stExon
cg25594119	18	11,947,571	0.00012	0.367	0.345					

cg06916239	2	223,162,881	0.000574	0.367	0.370				*PAX3*	Body
cg09662584	6	44,310,445	4.53E-25	0.367	0.388				*SPATS1*	1stExon
cg08141615	12	110,172,214	9.42E-14	0.367	0.345				*MGC14436*	Body
cg22178238	7	158,362,809	8.78E-13	0.367	0.367				*PTPRN2*	Body
cg10641714	5	43,193,681	1.09E-09	0.367	0.314				*MGC42105*	5'UTR
cg23460843	2	127,644,075	0.023915	0.367	0.429					
cg15366353	6	116,691,382	7.98E-24	0.367	0.317				*DSE*	TSS1500
cg00714725	10	94,825,576	8.87E-05	0.367	0.212				*CYP26C1*	Body
cg06844968	18	24,131,604	2.11E-28	0.367	0.335				*KCTD1*	5'UTR
cg08500417	7	102,574,445	3.78E-20	0.367	0.366				*FBXL13*	Body
cg17733616	17	73,083,887	0.012063	0.367	0.162				*SLC16A5*	TSS200
cg24516106	2	72,079,413	2.3E-23	0.366	0.351					
cg06428620	5	140,864,276	6.68E-06	0.366	0.353				*PCDHGA4*	Body
cg06718073	7	142,495,582	6.01E-10	0.366	0.337					
cg08789022	2	27,484,942	1.31E-09	0.366	0.379				*SLC30A3*	Body
cg01972979	X	133,680,446	3.29E-08	0.366	0.352				*MGC16121*	Body
cg03367264	10	13,392,131	0.003939	0.366	0.252					
cg14174055	2	97,203,640	1.07E-15	0.366	0.299				*ARID5A*	Body
cg07778819	17	43,862,927	4.25E-06	0.366	0.260				*CRHR1*	Body
cg12296772	8	17,271,067	0.004338	0.366	0.261				*MTMR7*	TSS200
cg09354556	3	47,051,341	9.4E-11	0.366	0.324					
cg18653451	19	46,917,018	1.08E-47	0.366	0.363				*CCDC8*	TSS200
cg04234680	14	51,562,550	0.008662	0.366	0.240				*TRIM9*	TSS200
cg25489866	15	59,729,553	0.005754	0.366	0.421				*FAM81A*	TSS1500
cg02449166	10	131,748,481	1.62E-13	0.366	0.337				*EBF3*	Body
cg15572489	7	157,481,091	3.11E-12	0.366	0.352				*PTPRN2*	Body
cg05911774	X	99,899,345	5.6E-19	0.366	0.364				*SRPX2*	1stExon
cg21121496	19	55,592,930	1.15E-05	0.366	0.350				*EPS8L1*	Body
cg07345934	1	31,845,943	0.000197	0.366	0.286				*FABP3*	TSS200
cg22964842	10	102,495,141	0.000109	0.366	0.327					
cg11134334	13	94,822,726	1.53E-30	0.366	0.346				*GPC6*	Body
cg14024937	6	14,441,721	6.02E-22	0.366	0.365					
cg13868604	20	42,875,931	5.8E-06	0.366	0.305				*GDAP1L1*	5'UTR
cg08018585	13	46,425,866	0.007712	0.366	0.341				*SIAH3*	TSS200

cg01203385	2	21,022,844	0.00058	0.366	0.331				*C2orf43*	TSS200
cg14239655	12	46,005,115	1.63E-13	0.366	0.341					
cg04658021	17	8,056,967	3.04E-27	0.366	0.350				*PER1*	TSS1500
cg16052975	17	15,821,016	3.95E-13	0.366	0.330					
cg16399136	1	151,104,003	0.000153	0.366	0.374					
cg04121938	1	201,509,415	2.84E-23	0.366	0.371					
cg06291314	17	2,325,086	4E-17	0.366	0.365				*METT10D*	Body
cg05070518	17	7,555,322	2.78E-13	0.366	0.320				*ATP1B2*	Body
cg07621385	3	123,535,716	2.15E-22	0.366	0.350				*MYLK*	5'UTR
cg13916459	12	85,673,221	0.000261	0.366	0.393				*ALX1*	TSS1500
cg14995036	1	94,146,705	1.86E-06	0.365	0.360				*BCAR3*	5'UTR
cg17933722	1	116,711,077	0.000746	0.365	0.322					
cg24331598	10	101,280,256	2.71E-12	0.365	0.367					
cg04177426	X	150,863,969	0.010374	0.365	0.286					
cg17124829	2	171,568,791	0.003776	0.365	0.269					
cg05341781	1	164,723,567	4.17E-26	0.365	0.353				*PBX1*	Body
cg07868599	16	3,079,708	3.26E-39	0.365	0.372				*CCDC64B*	Body
cg24448259	7	29,186,110	0.000185	0.365	0.326	4.58E-15	0.192		*CPVL*	5'UTR
cg16692973	X	119,445,209	7.04E-05	0.365	0.339				*FAM70A*	1stExon
cg25387565	6	31,830,527	0.023132	0.365	0.331				*NEU1*	1stExon
cg11857704	2	3,750,952	6.9E-05	0.365	0.393					
cg11631271	19	38,700,487	0.002387	0.365	0.492					
cg07097417	1	212,002,770	1.14E-25	0.365	0.358				*LPGAT1*	5'UTR
cg20078466	7	50,344,331	7.29E-06	0.365	0.374				*IKZF1*	TSS200
cg12315302	6	26,189,340	0.003102	0.365	0.334			Yes	*HIST1H4D*	TSS200
cg09858022	17	38,465,333	5.32E-31	0.365	0.365				*RARA*	TSS200
cg14499577	8	1,414,612	0.000158	0.365	0.429					
cg06980169	12	111,127,139	5.42E-07	0.365	0.274				*HVCN1*	TSS200
cg03156893	16	23,847,675	5.7E-14	0.365	0.355				*PRKCB*	Body
cg02680314	16	3,097,387	4.06E-10	0.365	0.358				*MMP25*	Body
cg23288298	2	43,451,559	3.75E-05	0.365	0.314				*ZFP36L2*	Body
cg12929487	4	41,869,175	6.57E-08	0.365	0.333					
cg00684125	7	21,209,165	1.93E-19	0.365	0.307					
cg14024788	5	1,875,611	3.56E-09	0.365	0.374					

cg10266490	1	55,013,709	2.56E-23	0.365	0.372	8.45E-20	0.240		*ACOT11*	TSS200
cg15156975	17	43,449,055	0.0014	0.365	0.341					
cg27125645	12	124,246,434	0.000147	0.365	0.161				*DNAH10*	TSS1500
cg00793186	5	139,283,100	7.09E-25	0.365	0.341				*NRG2*	Body
cg25339566	1	67,218,165	6.12E-08	0.365	0.339				*TCTEX1D1*	5'UTR
cg03348397	8	54,789,648	1.44E-18	0.365	0.342				*RGS20*	Body
cg05126289	1	156,995,411	6.36E-20	0.365	0.342				*ARHGEF11*	Body
cg13409449	10	8,097,354	7.91E-06	0.365	0.341				*GATA3*	5'UTR
cg20800956	1	171,811,477	0.030927	0.365	0.165				*DNM3*	Body
cg23101464	8	144,640,755	0.000412	0.365	0.261				*GSDMD*	5'UTR
cg14632485	11	113,216,820	2.55E-12	0.365	0.321				*TTC12*	Body
cg10474350	7	27,196,555	7.62E-06	0.365	0.387				*HOXA7*	TSS1500
cg26459372	7	45,613,676	1.47E-14	0.365	0.358				*ADCY1*	TSS1500
cg07663732	19	41,882,741	5.56E-17	0.365	0.356				*TMEM91*	1stExon
cg20293650	6	16,180,549	7.02E-13	0.365	0.329					
cg23560546	2	159,652,019	5.4E-18	0.365	0.362				*DAPL1*	Body
cg26051755	2	97,198,107	1.27E-08	0.365	0.329					
cg04060356	20	52,226,170	9.66E-05	0.364	0.295					
cg26009486	14	24,838,008	5.17E-20	0.364	0.337				*NFATC4*	Body
cg25552843	10	106,088,702	0.001037	0.364	0.415				*ITPRIP*	5'UTR
cg07871590	7	127,671,193	0.016994	0.364	0.248				*LRRC4*	TSS200
cg11036833	5	115,152,494	0.00052	0.364	0.198				*CDO1*	TSS200
cg11613015	5	112,073,406	0.021411	0.364	0.185				*APC*	TSS200
cg22675937	17	59,481,571	3.52E-19	0.364	0.372				*TBX2*	Body
cg15590989	4	183,062,460	1.14E-08	0.364	0.289				*MGC45800*	Body
cg09983326	2	43,451,308	7.77E-06	0.364	0.321				*ZFP36L2*	3'UTR
cg04540383	3	50,378,611	2.49E-07	0.364	0.359				*RASSF1*	TSS1500
cg18395917	11	76,033,367	1.06E-35	0.364	0.359					
cg24340926	X	129,305,036	8.94E-05	0.364	0.223	4.34E-38	0.304		*RAB33A*	TSS1500
cg26450149	7	47,375,903	7.62E-45	0.364	0.365				*TNS3*	Body
cg02094273	11	86,636,018	1.44E-19	0.364	0.363					
cg24641993	5	150,537,046	0.003099	0.364	0.160				*ANXA6*	5'UTR
cg18347921	6	29,975,366	1.07E-19	0.364	0.363				*HLA-J*	Body
cg19437285	5	148,650,972	1.41E-30	0.364	0.358				*AFAP1L1*	TSS1500

cg01492330	12	53,184,005	8.77E-05	0.364	0.398				*KRT3*	Body
cg09003023	6	29,974,253	1.55E-09	0.364	0.350				*HLA-J*	Body
cg13656752	5	171,538,334	1.11E-09	0.364	0.351				*STK10*	Body
cg20640433	6	129,204,206	2.32E-05	0.364	0.337	5.35E-18	0.267		*LAMA2*	TSS200
cg18591496	19	41,595,977	9.87E-19	0.364	0.355				*CYP2A13*	Body
cg18393747	13	96,205,295	3.94E-05	0.364	0.385				*CLDN10*	Body
cg01642521	11	20,618,250	0.00347	0.364	0.329					
cg11659501	12	81,471,678	3.21E-07	0.364	0.328				*ACSS3*	TSS200
cg08233811	6	21,667,333	5.64E-13	0.364	0.335				*FLJ22536*	Body
cg16329197	12	53,359,506	2.75E-10	0.364	0.338					
cg05375728	1	58,715,539	0.000746	0.364	0.334				*DAB1*	5'UTR
cg12589143	6	29,894,341	3.2E-07	0.364	0.374				*HCG4P6*	TSS1500
cg00659931	15	102,010,125	4.55E-23	0.364	0.362				*PCSK6*	Body
cg13102294	6	32,121,393	9.95E-26	0.364	0.338				*PPT2*	TSS1500
cg01611017	14	95,780,344	2.3E-17	0.364	0.375				*CLMN*	Body
cg05906166	19	16,437,057	1.61E-17	0.364	0.352				*KLF2*	Body
cg13759905	2	233,741,920	1.35E-20	0.364	0.307					
cg00645339	15	45,670,875	7.42E-08	0.364	0.370				*LOC145663*	TSS200
cg13213923	17	60,885,491	0.000336	0.364	0.356				*MARCH10*	5'UTR
cg13038685	14	77,504,478	3.35E-22	0.364	0.385					
cg12902896	3	37,987,838	4.35E-24	0.364	0.322				*CTDSPL*	Body
cg00944193	13	36,044,860	0.002071	0.363	0.468				*NBEA*	Body
cg12175729	8	140,715,540	7.97E-07	0.363	0.387				*KCNK9*	TSS1500
cg27448110	7	157,484,647	6.71E-39	0.363	0.363				*PTPRN2*	Body
cg05312353	6	17,102,678	0.000113	0.363	0.244					
cg26107476	1	202,054,898	1.08E-16	0.363	0.358					
cg23217512	15	70,877,866	1.44E-08	0.363	0.299					
cg21195256	14	29,244,278	0.011876	0.363	0.263				*C14orf23*	Body
cg08118140	1	56,397,447	4.07E-49	0.363	0.331					
cg07227926	12	104,609,526	1.64E-12	0.363	0.344				*TXNRD1*	TSS200
cg24563094	2	26,395,458	0.007372	0.363	0.294				*FAM59B*	TSS1500
cg02348751	4	183,064,035	5.57E-08	0.363	0.361				*MGC45800*	Body
cg17990771	6	32,223,226	3.31E-05	0.363	0.383					
cg14383422	2	201,450,962	4.42E-05	0.363	0.306				*AOX1*	Body

cg15417365	17	56,609,082	4.62E-08	0.363	0.256				*SEPT4*	Body
cg09065413	1	45,285,821	1.62E-05	0.363	0.419				*PTCH2*	3'UTR
cg15062377	1	200,004,205	3.05E-17	0.363	0.352				*NR5A2*	Body
cg11076306	4	41,430,667	2.36E-10	0.363	0.357				*LIMCH1*	Body
cg20289949	2	43,019,614	7.41E-07	0.363	0.214				*HAAO*	1stExon
cg04787024	2	99,553,442	0.000157	0.363	0.391				*C2orf55*	TSS1500
cg18437710	19	51,522,894	4.67E-11	0.363	0.374				*KLK10*	5'UTR
cg25025879	12	53,359,317	5.13E-17	0.363	0.344					
cg22621867	3	51,990,301	0.000206	0.363	0.397				*GPR62*	1stExon
cg25677261	15	102,009,856	1.37E-32	0.363	0.361				*PCSK6*	Body
cg18818075	6	44,528,865	8.95E-10	0.363	0.279					
cg24679890	19	17,246,356	3.49E-11	0.363	0.394				*MYO9B*	Body
cg11028201	4	41,867,562	0.000179	0.363	0.363					
cg08285646	16	69,432,500	8.22E-24	0.363	0.327					
cg06720425	3	25,469,694	1.27E-19	0.363	0.325				*RARB*	TSS200
cg16049283	1	6,419,606	6.84E-25	0.363	0.363				*ACOT7*	Body
cg27494383	15	41,805,868	6.68E-07	0.363	0.392	6.33E-09	0.151		*LTK*	1stExon
cg07627556	11	115,530,590	4.94E-07	0.363	0.325					
cg15436123	4	3,390,332	5.17E-12	0.363	0.406				*RGS12*	Body
cg22773661	4	48,492,225	0.006489	0.363	0.335				*ZAR1*	TSS200
cg02058408	17	20,687,681	3.05E-05	0.362	0.313					
cg09242181	11	46,940,517	4.05E-10	0.362	0.362				*LRP4*	TSS1500
cg00371702	3	25,469,125	1.53E-14	0.362	0.314				*RARB*	TSS1500
cg01059100	16	31,214,607	6.76E-25	0.362	0.313				*PYCARD*	TSS1500
cg23376821	6	45,391,365	0.001884	0.362	0.371				*RUNX2*	Body
cg24192569	7	4,184,151	4.96E-23	0.362	0.386				*SDK1*	Body
cg15717448	5	171,234,429	3.87E-08	0.362	0.332					
cg13714344	12	6,938,635	1.07E-08	0.362	0.357				*LEPREL2*	Body
cg08687386	6	30,711,206	2.26E-29	0.362	0.331				*IER3*	3'UTR
cg01758418	X	70,151,160	2.21E-08	0.362	0.343				*SLC7A3*	TSS1500
cg23024158	10	78,011,952	2.69E-16	0.362	0.295				*C10orf11*	Body
cg14511739	5	112,073,373	0.021305	0.362	0.170				*APC*	TSS200
cg02614217	4	122,632,774	2.7E-08	0.362	0.352					
cg08167706	7	134,144,106	5.04E-08	0.362	0.244				*AKR1B1*	TSS1500

cg10445911	1	154,971,922	0.00291	0.362	0.365					
cg23426945	10	77,190,054	1.09E-10	0.362	0.342					
cg26459819	11	111,169,457	3.83E-22	0.362	0.362				*C11orf93*	TSS1500
cg09611490	2	97,193,080	2.46E-30	0.362	0.347					
cg09107344	12	53,612,734	6.56E-17	0.362	0.384				*RARG*	Body
cg21472506	2	63,283,967	1.86E-06	0.362	0.357				*OTX1*	3'UTR
cg00246451	1	155,949,012	5.93E-10	0.362	0.356				*ARHGEF2*	TSS1500
cg12134633	15	32,934,004	2.08E-09	0.362	0.275				*SCG5*	5'UTR
cg26195583	4	41,869,348	6.58E-09	0.362	0.344					
cg23016080	15	44,486,450	4.95E-06	0.362	0.313				*FRMD5*	Body
cg08076018	9	129,885,080	4.56E-07	0.362	0.315				*ANGPTL2*	TSS200
cg06760830	1	47,079,672	2.78E-09	0.362	0.399				*MOBKL2C*	Body
cg22489153	12	115,136,175	5.06E-12	0.362	0.331					
cg02273392	7	100,770,414	4.59E-09	0.362	0.358	9.11E-29	0.201		*SERPINE1*	5'UTR
cg04772683	19	51,522,641	0.006691	0.362	0.376				*KLK10*	5'UTR
cg04134279	8	496,327	5.05E-11	0.362	0.283				*C8orf42*	TSS1500
cg06599914	11	32,915,903	1.7E-05	0.362	0.425				*QSER1*	5'UTR
cg13084536	5	139,076,824	7.68E-18	0.362	0.336					
cg25620356	7	18,535,407	2.84E-05	0.362	0.319				*HDAC9*	TSS1500
cg08516516	5	115,152,492	0.001624	0.362	0.192				*CDO1*	TSS200
cg25054754	4	169,754,328	8.85E-06	0.362	0.402				*PALLD*	5'UTR
cg21903079	14	24,457,915	4.7E-25	0.362	0.331				*DHRS4L2*	TSS200
cg19896129	1	63,156,450	2.38E-18	0.362	0.340					
cg07035145	14	51,410,689	0.000128	0.362	0.374				*PYGL*	Body
cg03186486	3	68,980,947	2.93E-06	0.362	0.345				*FAM19A4*	5'UTR
cg00655913	1	228,633,920	1.19E-07	0.362	0.391					
cg12976581	6	29,975,032	6.47E-10	0.362	0.347				*HLA-J*	Body
cg10763594	2	218,258,333	1.72E-11	0.361	0.325			Yes	*DIRC3*	Body
cg22819422	10	3,108,507	2.6E-37	0.361	0.362				*PFKP*	TSS1500
cg08481075	6	33,161,328	0.001253	0.361	0.311				*COL11A2*	TSS1500
cg26973714	12	104,609,524	1.42E-13	0.361	0.326				*TXNRD1*	TSS200
cg04719903	1	1,181,956	1.04E-26	0.361	0.321				*FAM132A*	1stExon
cg20066782	7	102,574,504	3.58E-18	0.361	0.354				*FBXL13*	Body
cg07100000	5	170,877,840	3.69E-18	0.361	0.344				*FGF18*	Body

cg12126857	1	35,384,669	0.001505	0.361	0.353					
cg18738581	3	9,944,183	6.59E-11	0.361	0.358				*IL17RE*	TSS200
cg07978472	4	122,686,493	3.85E-06	0.361	0.252				*TMEM155*	TSS200
cg10935662	3	72,307,754	1.19E-15	0.361	0.341					
cg18082515	10	95,197,126	7.5E-16	0.361	0.350				*MYOF*	Body
cg11840125	14	23,834,860	0.004478	0.361	0.391				*EFS*	TSS200
cg23196756	10	72,165,580	1.24E-14	0.361	0.353				*EIF4EBP2*	Body
cg18995088	5	134,914,734	2.2E-06	0.361	0.334	6.94E-12	0.177		*CXCL14*	5'UTR
cg03131732	11	46,317,475	3.32E-10	0.361	0.275				*CREB3L1*	Body
cg11296826	1	40,236,125	7.15E-23	0.361	0.370				*BMP8B*	Body
cg13826247	12	54,424,203	3.69E-12	0.361	0.289				*HOXC4*	5'UTR
cg01680010	7	96,647,117	4.47E-08	0.361	0.373					
cg04276005	1	180,895,868	4.34E-31	0.361	0.355				*KIAA1614*	Body
cg21535657	12	11,806,362	1.82E-17	0.361	0.348				*ETV6*	Body
cg25588389	7	2,654,420	6.11E-06	0.361	0.274					
cg13837913	6	26,189,410	0.026618	0.361	0.327				*HIST1H4D*	TSS200
cg08598483	6	29,975,145	1.69E-15	0.361	0.358				*HLA-J*	Body
cg21081729	5	112,539,140	2.04E-07	0.361	0.334				*MCC*	Body
cg01356950	2	175,546,714	7.16E-08	0.361	0.255				*WIPF1*	5'UTR
cg09469554	13	96,204,917	0.013381	0.361	0.410				*CLDN10*	TSS200
cg09168808	12	115,124,584	9.59E-19	0.361	0.324					
cg26069562	6	29,691,981	3.38E-06	0.361	0.267				*HLA-F*	Body
cg25267808	11	95,779,454	5.01E-14	0.360	0.363				*MAML2*	Body
cg11608150	5	135,415,948	1.78E-05	0.360	0.316					
cg10215401	8	131,058,332	1.39E-20	0.360	0.356					
cg23258678	1	200,004,095	2.09E-10	0.360	0.353				*NR5A2*	Body
cg03070989	19	34,311,482	0.000139	0.360	0.358					
cg16355305	12	65,220,152	2.46E-17	0.360	0.313					
cg24394631	17	43,863,000	6.87E-07	0.360	0.303				*CRHR1*	Body
cg04791080	15	68,771,946	3.3E-23	0.360	0.356					
cg17511472	4	3,485,326	7.7E-15	0.360	0.312				*DOK7*	Body
cg10356613	17	35,294,491	0.001362	0.360	0.395				*LHX1*	TSS200
cg05313153	8	119,122,430	2.76E-15	0.360	0.379				*EXT1*	1stExon
cg11299991	3	157,154,687	3.26E-13	0.360	0.355				*PTX3*	5'UTR

cg26895804	8	145,917,243	1.65E-20	0.360	0.368					
cg26015416	1	108,023,482	1.71E-13	0.360	0.303				*NTNG1*	3'UTR
cg14687145	2	29,234,537	3.13E-05	0.360	0.376				*FAM179A*	Body
cg06616710	10	99,473,224	3.4E-08	0.360	0.317				*MARVELD1*	TSS1500
cg00011994	6	30,711,904	3.26E-09	0.360	0.359				*FLOT1*	TSS1500
cg27038634	8	49,468,341	1.26E-20	0.360	0.369					
cg18004701	7	157,481,628	1.38E-10	0.360	0.358				*PTPRN2*	Body
cg19067730	20	44,518,824	3.15E-11	0.360	0.259	6.73E-10	0.153		*CTSA*	TSS1500
cg19267596	19	15,334,821	3.37E-06	0.360	0.240					
cg06945523	14	36,974,800	0.002296	0.360	0.415				*SFTA3*	Body
cg09408571	1	101,003,634	2.25E-30	0.360	0.376				*GPR88*	TSS200
cg11024449	X	70,150,787	0.031688	0.360	0.397				*SLC7A3*	5'UTR
cg10625365	1	168,196,658	2.21E-16	0.360	0.348			Yes	*SFT2D2*	Body
cg07146104	1	16,489,048	3.76E-13	0.360	0.338					
cg24922129	11	129,242,530	6.78E-09	0.360	0.349					
cg10553890	11	83,074,588	2.94E-08	0.360	0.379					
cg10304637	6	6,003,220	0.002158	0.360	0.343				*NRN1*	Body
cg18111443	19	20,349,275	0.042045	0.360	0.193					
cg02550961	15	45,406,866	0.000158	0.360	0.353				*DUOXA2*	1stExon
cg06785746	17	44,896,166	1.45E-06	0.360	0.389				*WNT3*	TSS200
cg23183906	19	16,187,089	0.00221	0.360	0.341				*TPM4*	Body
cg08364561	11	31,846,844	9.05E-05	0.360	0.388					
cg26666133	12	90,101,389	0.000467	0.360	0.392				*LOC338758*	TSS1500
cg13039251	5	32,018,601	3.1E-20	0.360	0.332				*PDZD2*	Body
cg06832339	7	23,842,119	1.47E-19	0.360	0.320				*STK31*	Body
cg19545232	14	70,654,247	7.79E-08	0.359	0.266				*SLC8A3*	5'UTR
cg16414149	7	1,294,013	8.41E-18	0.359	0.362					
cg09480054	2	43,020,129	7.4E-07	0.359	0.204				*HAAO*	TSS1500
cg06077978	X	48,690,457	0.00033	0.359	0.451				*PCSK1N*	Body
cg25349820	22	25,346,354	4.7E-08	0.359	0.385					
cg08268798	20	42,955,472	0.00128	0.359	0.347					
cg15763670	10	73,473,235	7.68E-15	0.359	0.343				*C10orf105*	3'UTR
cg01146320	11	120,080,945	1.7E-11	0.359	0.332				*OAF*	TSS1500
cg17330251	7	94,953,956	4.51E-13	0.359	0.317				*PON1*	TSS200

cg03053374	17	38,465,422	1.23E-18	0.359	0.360				*RARA*	TSS200
cg06460983	10	91,092,340	8.22E-06	0.359	0.298				*IFIT3*	Body
cg23837109	10	75,670,435	4.88E-27	0.359	0.334				*PLAU*	TSS1500
cg21741562	3	87,138,387	4.19E-05	0.359	0.318					
cg10721782	15	90,039,809	4.35E-13	0.359	0.364				*RHCG*	TSS200
cg27510182	1	58,715,553	0.001803	0.359	0.353				*DAB1*	5'UTR
cg12914657	7	150,413,680	1.94E-13	0.359	0.369	4.4E-34	0.265		*GIMAP1*	TSS200
cg02635932	10	18,549,778	5.82E-20	0.359	0.344				*CACNB2*	1stExon
cg15045802	19	1,857,434	0.014349	0.359	0.293				*KLF16*	Body
cg05308656	2	235,406,291	3.9E-50	0.359	0.339				*ARL4C*	TSS1500
cg21606490	1	108,023,428	5.6E-13	0.359	0.296				*NTNG1*	Body
cg00510539	2	27,958,611	0.000318	0.359	0.224					
cg13573928	4	169,754,534	7.43E-09	0.359	0.373				*PALLD*	5'UTR
cg09516476	6	10,881,925	1.93E-14	0.359	0.338				*GCM2*	Body
cg07173972	20	61,583,979	1.94E-05	0.359	0.345				*SLC17A9*	TSS200
cg23122109	2	228,582,688	5.05E-05	0.359	0.237				*SLC19A3*	5'UTR
cg04775710	6	30,712,022	9.05E-05	0.359	0.359				*IER3*	Body
cg17389738	6	30,711,642	4.82E-28	0.359	0.361				*IER3*	3'UTR
cg27071460	5	40,681,893	1.89E-10	0.359	0.367				*PTGER4*	Body
cg10210594	1	208,132,787	0.003576	0.359	0.346					
cg08382226	6	108,440,339	0.000186	0.359	0.361					
cg11909912	17	43,974,919	1.01E-16	0.359	0.326				*MAPT*	5'UTR
cg22325530	12	111,127,346	7.81E-06	0.359	0.312				*HVCN1*	TSS1500
cg19624849	1	145,415,412	2.29E-06	0.358	0.409				*HFE2*	5'UTR
cg15202006	8	68,658,566	2.72E-09	0.358	0.331				*CPA6*	1stExon
cg02071670	15	89,438,918	1.17E-56	0.358	0.337				*HAPLN3*	TSS200
cg01616225	16	57,662,347	4.55E-14	0.358	0.342				*GPR56*	TSS200
cg20559385	6	106,049,591	4.63E-19	0.358	0.341					
cg10869581	X	102,000,905	0.002934	0.358	0.290				*BHLHB9*	TSS200
cg05226168	16	68,118,441	5.17E-32	0.358	0.338				*NFATC3*	TSS1500
cg12225220	4	87,281,434	3.03E-10	0.358	0.363				*MAPK10*	TSS200
cg01224366	21	45,148,694	0.001268	0.358	0.271				*PDXK*	Body
cg00158683	3	48,632,625	0.001674	0.358	0.348				*COL7A1*	TSS200
cg14808329	5	133,863,570	0.003097	0.358	0.334				*PHF15*	5'UTR

cg11045524	9	133,320,781	0.001802	0.358	0.217				*ASS1*	5'UTR
cg27624826	2	131,010,647	7.62E-08	0.358	0.317					
cg27545367	6	30,711,644	5.68E-22	0.358	0.359				*IER3*	3'UTR
cg06341027	6	21,667,407	1.03E-20	0.358	0.307				*FLJ22536*	Body
cg14479889	5	112,073,426	0.002772	0.358	0.180				*APC*	TSS200
cg15229275	19	46,800,054	6.38E-40	0.358	0.360				*HIF3A*	TSS1500
cg01167755	5	139,284,498	6.33E-07	0.358	0.405				*NRG2*	Body
cg25344503	10	47,083,398	0.000459	0.358	0.170				*PPYR1*	TSS200
cg23634087	20	50,721,797	2E-05	0.358	0.351				*ZFP64*	Body
cg15016771	2	235,403,218	1.96E-11	0.358	0.379				*ARL4C*	3'UTR
cg03567358	11	71,050,535	7.8E-21	0.358	0.356					
cg11872300	19	42,785,225	0.000452	0.358	0.295					
cg05774229	11	76,033,097	5.55E-10	0.358	0.341					
cg22454011	18	13,611,439	1.33E-05	0.358	0.315			Yes	*C18orf1*	TSS1500
cg11309496	17	33,701,353	0.000442	0.358	0.446				*SLFN11*	TSS1500
cg13662569	4	186,577,902	4.05E-09	0.358	0.351				*SORBS2*	1stExon
cg03086235	8	10,586,796	7.08E-14	0.358	0.341				*SOX7*	Body
cg08504812	3	157,260,627	1.94E-06	0.358	0.373				*C3orf55*	TSS1500
cg11694519	11	111,783,563	3.9E-06	0.358	0.346				*CRYAB*	TSS1500
cg12601757	1	76,540,465	1.92E-05	0.358	0.348	3.15E-30	0.283		*ST6GALNAC3*	1stExon
cg02878891	2	102,090,444	0.048249	0.358	0.174				*RFX8*	5'UTR
cg07080050	12	54,413,345	4.79E-07	0.358	0.318				*HOXC4*	5'UTR
cg11670789	15	93,208,459	0.001459	0.357	0.257					
cg13350478	X	20,286,613	6.74E-25	0.357	0.343					
cg17651959	20	21,498,100	6.34E-07	0.357	0.309					
cg26757221	17	21,477,761	9.78E-08	0.357	0.334					
cg02958263	1	174,769,548	8.95E-08	0.357	0.290				*RABGAP1L*	Body
cg23506042	5	50,262,673	3.11E-13	0.357	0.322					
cg12534150	5	112,073,613	0.006243	0.357	0.211				*APC*	1stExon
cg11867546	6	29,974,810	1.09E-09	0.357	0.353				*HLA-J*	Body
cg14615768	17	8,924,093	1.28E-06	0.357	0.261				*NTN1*	TSS1500
cg01780009	17	80,944,193	5.3E-21	0.357	0.356				*B3GNTL1*	Body
cg23558337	20	10,492,394	7.44E-10	0.357	0.346				*C20orf94*	Body
cg04679712	8	25,898,839	1.72E-09	0.357	0.350				*EBF2*	Body

cg16003256	1	116,710,945	1.69E-05	0.357	0.366					
cg27579711	6	26,172,931	4.17E-07	0.357	0.398					
cg13484546	1	16,084,939	0.000193	0.357	0.408				*FBLIM1*	TSS1500
cg00192031	7	47,620,790	9.78E-15	0.357	0.296					
cg24511404	1	212,607,111	2.71E-28	0.357	0.327				*NENF*	Body
cg25019989	4	54,961,835	7.79E-16	0.357	0.351					
cg02454806	14	42,075,554	1.36E-11	0.357	0.366				*LRFN5*	TSS1500
cg19630689	14	94,984,381	2.31E-10	0.357	0.333	9.07E-21	0.253		*SERPINA12*	TSS1500
cg21532325	7	151,107,400	7.67E-05	0.357	0.369				*WDR86*	TSS1500
cg04850731	12	57,618,943	5.54E-08	0.357	0.356				*NXPH4*	Body
cg06346505	10	116,164,640	4.28E-15	0.357	0.309				*AFAP1L2*	TSS200
cg21211367	2	162,094,118	1.13E-33	0.357	0.325					
cg13550694	9	73,835	0.000233	0.357	0.378					
cg23452969	11	62,691,182	6.55E-13	0.357	0.326					
cg02298094	12	111,844,676	1.8E-18	0.357	0.341				*SH2B3*	5'UTR
cg02561912	20	50,158,202	6.69E-10	0.357	0.361				*NFATC2*	Body
cg17028443	X	3,630,086	5.97E-15	0.357	0.344				*PRKX*	Body
cg21383810	12	65,515,470	8.58E-05	0.357	0.371				*WIF1*	TSS1500
cg08881019	1	156,096,178	7.02E-24	0.357	0.358				*LMNA*	Body
cg10901633	3	4,814,459	1.78E-12	0.357	0.331				*ITPR1*	Body
cg27363327	6	43,211,208	0.034184	0.357	0.416				*TTBK1*	TSS200
cg20857709	2	43,019,727	0.014209	0.357	0.163				*HAAO*	5'UTR
cg23740652	7	157,561,925	4.88E-22	0.357	0.350				*PTPRN2*	Body
cg00299943	22	41,613,692	2.17E-24	0.357	0.347				*L3MBTL2*	Body
cg23887396	17	26,699,551	0.000249	0.357	0.248	1.15E-08	0.172		*SARM1*	Body
cg08309809	8	22,960,572	0.007245	0.357	0.263				*TNFRSF10C*	5'UTR
cg14451382	5	1,876,397	5.33E-19	0.357	0.305					
cg17221758	8	30,243,241	3.18E-10	0.357	0.298				*RBPMS*	Body
cg12783776	11	57,365,948	7.94E-13	0.357	0.335	4.5E-17	0.246		*SERPING1*	Body
cg23719318	2	20,865,651	1.69E-05	0.357	0.388				*GDF7*	TSS1500
cg01074657	1	78,511,466	0.000112	0.357	0.344				*GIPC2*	TSS200
cg16761581	14	24,803,807	0.00015	0.357	0.383	3.3E-20	0.259		*ADCY4*	1stExon
cg27268352	10	30,387,860	2.22E-10	0.357	0.393					
cg18443437	5	3,326,731	3.73E-05	0.357	0.299					

cg26986180	12	124,246,525	0.001904	0.356	0.127				*DNAH10*	TSS1500
cg23060872	2	71,205,685	0.023996	0.356	0.431				*ANKRD53*	1stExon
cg20486569	22	19,512,228	5.58E-06	0.356	0.309				*CLDN5*	1stExon
cg08815398	12	65,218,869	1.43E-24	0.356	0.336					
cg15140465	11	77,184,194	8.18E-06	0.356	0.289				*PAK1*	5'UTR
cg07732143	6	36,807,193	1.57E-12	0.356	0.341				*CPNE5*	1stExon
cg10705306	8	37,337,009	4.69E-10	0.356	0.347					
cg21511941	15	72,489,609	3.89E-22	0.356	0.317				*GRAMD2*	Body
cg23445461	5	140,864,733	9.06E-07	0.356	0.353				*PCDHGA4*	Body
cg03587557	6	31,831,553	5.02E-06	0.356	0.341				*NEU1*	TSS1500
cg09676684	11	61,062,962	3.16E-17	0.356	0.300				*VWCE*	TSS200
cg13397632	X	106,750,861	2E-08	0.356	0.391					
cg17322044	12	125,140,105	1.65E-10	0.356	0.387					
cg07128973	2	130,901,432	1.4E-20	0.356	0.372				*CCDC74B*	Body
cg13651986	2	175,548,447	8.07E-20	0.356	0.288				*WIPF1*	TSS1500
cg15951557	5	137,610,421	0.003928	0.356	0.274				*GFRA3*	TSS200
cg24686074	10	102,497,666	0.000409	0.356	0.389					
cg25683325	1	158,151,058	0.000137	0.356	0.401				*CD1D*	Body
cg23251761	1	9,750,061	2.33E-11	0.356	0.341				*PIK3CD*	5'UTR
cg05119057	14	29,255,119	0.00033	0.356	0.307				*C14orf23*	Body
cg20143092	11	35,160,330	8.15E-05	0.356	0.409	4.07E-25	0.239		*CD44*	TSS200
cg17501823	12	47,219,793	7.92E-07	0.356	0.241				*SLC38A4*	TSS200
cg09590068	12	54,400,494	1.56E-20	0.356	0.316					
cg21323637	X	153,600,494	2.9E-11	0.356	0.316				*FLNA*	5'UTR
cg26195683	5	1,667,958	6.33E-11	0.356	0.327					
cg17399362	19	15,343,345	0.010845	0.356	0.419				*EPHX3*	5'UTR
cg00491927	3	120,003,642	4.17E-07	0.356	0.291					
cg21849932	20	62,369,462	0.004499	0.356	0.366				*LIME1*	Body
cg10703826	1	119,532,116	6.76E-06	0.356	0.395				*TBX15*	1stExon
cg23497752	X	153,598,077	0.000117	0.356	0.353				*FLNA*	Body
cg04608177	20	37,230,389	1.12E-41	0.356	0.360				*C20orf95*	TSS200
cg18753440	7	63,955,786	3.09E-12	0.356	0.322					
cg07160871	2	221,969,259	0.00014	0.355	0.384					
cg11359133	2	176,948,699	0.018257	0.355	0.416				*EVX2*	TSS200

cg14539231	13	43,566,262	1.24E-05	0.355	0.357	1.54E-22	0.279		*EPSTI1*	1stExon
cg20483324	13	46,189,838	0.000268	0.355	0.394				*FAM194B*	5'UTR
cg19307528	8	74,004,928	1.11E-07	0.355	0.417				*C8orf84*	Body
cg10748765	20	3,218,905	4.03E-06	0.355	0.300				*SLC4A11*	TSS1500
cg14550611	19	41,354,682	4.74E-10	0.355	0.341				*CYP2A6*	Body
cg22774472	2	190,044,636	6.29E-11	0.355	0.352	6.15E-31	0.234		*COL5A2*	TSS200
cg00663077	4	188,916,709	3.78E-06	0.355	0.345				*ZFP42*	TSS1500
cg12100791	16	31,214,417	6.82E-21	0.355	0.285	1.32E-23	0.202		*PYCARD*	TSS200
cg22176324	1	24,648,851	7.16E-11	0.355	0.386				*GRHL3*	TSS1500
cg22719241	19	36,524,058	1.8E-08	0.355	0.316				*CLIP3*	TSS1500
cg17741501	15	29,077,493	0.000263	0.355	0.395					
cg10864074	7	100,318,194	0.017469	0.355	0.189				*EPO*	TSS1500
cg14777342	12	57,480,090	6.51E-10	0.355	0.397					
cg01283834	4	141,488,964	0.000515	0.355	0.300				*UCP1*	Body
cg02502361	9	135,117,928	1.15E-11	0.355	0.341				*NTNG2*	3'UTR
cg07017114	6	29,855,465	2.73E-13	0.355	0.365				*HLA-H*	Body
cg10677787	14	24,457,893	4.1E-12	0.355	0.328	9.63E-25	0.143		*DHRS4L2*	TSS200
cg01711160	14	85,996,499	1.15E-06	0.355	0.317				*FLRT2*	1stExon
cg13656173	9	139,002,093	1.22E-18	0.355	0.344					
cg05355225	8	86,350,568	1.48E-10	0.355	0.323				*CA3*	TSS1500
cg19300568	4	2,060,112	3.44E-05	0.355	0.236				*NAT8L*	TSS1500
cg25680628	10	30,724,274	5.14E-05	0.355	0.320				*MAP3K8*	5'UTR
cg18103730	14	88,458,917	0.000868	0.355	0.253				*GALC*	Body
cg27011480	6	30,228,083	0.002293	0.355	0.272				*HLA-L*	Body
cg02074274	21	45,178,331	1.28E-12	0.355	0.371				*PDXK*	3'UTR
cg18252616	13	22,243,392	2.39E-11	0.355	0.303					
cg05259508	12	115,124,838	6.07E-09	0.355	0.303					
cg10972873	1	200,007,592	1.42E-08	0.355	0.377				*NR5A2*	Body
cg14666564	2	177,030,228	5.98E-07	0.355	0.303				*HOXD3*	5'UTR
cg11690979	1	151,694,320	3.33E-06	0.355	0.321				*C1orf230*	Body
cg08231349	6	29,894,644	3.26E-08	0.355	0.319				*HCG4P6*	TSS1500
cg00858599	20	3,220,929	5.76E-17	0.355	0.300					
cg14035860	14	55,707,715	2.37E-10	0.355	0.357					
cg24914355	2	176,959,229	2.46E-05	0.355	0.391				*HOXD13*	Body

cg27179041	3	61,889,812	1.43E-20	0.355	0.357				*PTPRG*	Body
cg09187007	1	3,514,990	9.27E-08	0.355	0.382				*MEGF6*	Body
cg02283485	10	81,003,020	0.000443	0.355	0.309				*ZMIZ1*	Body
cg03002352	2	17,699,688	0.000946	0.355	0.368				*RAD51AP2*	1stExon
cg11511777	2	219,725,959	4.34E-33	0.355	0.351				*WNT6*	Body
cg14603098	17	32,484,259	0.000398	0.355	0.405				*ACCN1*	TSS1500
cg06385583	2	12,596,843	1.6E-05	0.354	0.371					
cg06637517	3	52,407,344	0.000004	0.354	0.361				*DNAH1*	Body
cg27172557	14	91,012,362	6.5E-22	0.354	0.334				*TTC7B*	Body
cg26761826	8	67,419,572	2.05E-17	0.354	0.380				*C8orf46*	Body
cg18695839	12	54,446,944	5.17E-08	0.354	0.294				*HOXC4*	5'UTR
cg26906273	1	39,547,881	2.94E-06	0.354	0.317				*MACF1*	5'UTR
cg03947979	6	78,176,785	2.02E-05	0.354	0.326					
cg11321459	7	18,535,263	0.000156	0.354	0.306				*HDAC9*	TSS1500
cg07119434	1	151,103,642	3.27E-06	0.354	0.307					
cg08614290	7	158,938,491	1.06E-21	0.354	0.343				*VIPR2*	TSS1500
cg16179521	15	102,009,894	9.15E-18	0.354	0.333				*PCSK6*	Body
cg07118796	3	126,892,022	1.68E-07	0.354	0.329					
cg18519308	5	33,892,621	5.14E-14	0.354	0.332				*ADAMTS12*	TSS1500
cg00700485	20	44,098,223	1.62E-06	0.354	0.237				*WFDC2*	TSS200
cg06745944	12	124,246,391	0.000163	0.354	0.144				*DNAH10*	TSS1500
cg06594281	17	73,083,692	0.000499	0.354	0.180				*SLC16A5*	TSS1500
cg26574003	1	150,186,289	0.00027	0.354	0.245					
cg26837800	1	56,842,338	3.36E-21	0.354	0.344					
cg15826897	19	15,343,373	0.000344	0.354	0.397				*EPHX3*	5'UTR
cg03854238	6	22,071,933	1.71E-24	0.354	0.317				*FLJ22536*	Body
cg24613080	17	32,484,035	7.1E-17	0.354	0.388				*ACCN1*	TSS1500
cg06498232	3	72,227,063	5E-14	0.354	0.357					
cg26998850	16	50,300,322	1.36E-18	0.354	0.334					
cg10088041	15	45,670,872	7.9E-07	0.354	0.364				*LOC145663*	TSS200
cg03940848	1	229,543,112	0.004051	0.354	0.372					
cg08780711	17	6,560,593	4.73E-07	0.354	0.362					
cg20708842	5	127,875,163	7.84E-05	0.354	0.365				*FBN2*	TSS1500
cg14226182	3	158,450,251	0.011779	0.354	0.143				*RARRES1*	5'UTR

cg01390574	7	129,425,451	4.68E-07	0.354	0.360					
cg17298973	6	2,876,494	3.82E-15	0.354	0.290					
cg06438517	1	16,861,620	1.94E-11	0.354	0.330					
cg25417766	19	52,191,468	1.03E-12	0.354	0.313					
cg17390301	11	111,250,093	0.000206	0.354	0.353				*POU2AF1*	1stExon
cg01434608	16	66,637,927	0.035352	0.354	0.116				*CMTM3*	TSS1500
cg12045337	2	171,572,714	0.019992	0.354	0.371				*SP5*	Body
cg06829830	5	80,256,087	4.24E-06	0.354	0.292				*RASGRF2*	TSS1500
cg09445472	10	7,709,962	2.47E-11	0.354	0.378				*ITIH5*	TSS1500
cg10122865	2	63,284,132	1.77E-06	0.354	0.358				*OTX1*	3'UTR
cg07976064	11	120,435,056	0.013423	0.354	0.226					
cg00494337	20	30,194,024	0.010189	0.354	0.319				*ID1*	3'UTR
cg19869037	6	116,732,515	2.9E-10	0.354	0.385				*DSE*	Body
cg12042113	15	90,039,913	6.05E-10	0.354	0.356				*RHCG*	TSS200
cg25380021	5	171,601,682	1.49E-20	0.354	0.331				*STK10*	Body
cg11445094	6	28,521,837	2.51E-07	0.354	0.362					
cg04343891	4	81,106,663	0.001218	0.354	0.344				*PRDM8*	5'UTR
cg26158950	6	21,667,166	5.2E-10	0.354	0.305				*FLJ22536*	Body
cg01111041	6	32,121,055	1.81E-15	0.354	0.351				*PPT2*	TSS1500
cg14611152	9	124,463,026	3.88E-20	0.354	0.317				*DAB2IP*	Body
cg25528916	19	4,328,818	2.2E-07	0.354	0.356				*STAP2*	Body
cg12710510	12	3,862,497	0.036062	0.354	0.222				*EFCAB4B*	TSS200
cg12638737	1	204,120,607	1.52E-11	0.353	0.279				*ETNK2*	Body
cg22877509	1	155,290,607	1.78E-05	0.353	0.337				*RUSC1*	TSS200
cg17918002	10	28,035,719	4.72E-13	0.353	0.378				*MKX*	TSS1500
cg25440893	15	35,091,802	1.96E-15	0.353	0.281					
cg25236484	X	51,139,247	0.020496	0.353	0.368					
cg14817655	4	188,916,724	3.68E-08	0.353	0.321				*ZFP42*	TSS200
cg19997502	2	208,492,057	0.002578	0.353	0.366					
cg20543681	20	642,673	5.05E-07	0.353	0.354				*SCRT2*	3'UTR
cg02022733	5	140,864,578	1.01E-09	0.353	0.346				*PCDHGA4*	Body
cg10032668	4	187,065,417	1.66E-07	0.353	0.247				*FAM149A*	TSS1500
cg09397246	15	91,427,361	2.12E-15	0.353	0.349				*FES*	TSS1500
cg07014523	20	50,721,313	2.32E-15	0.353	0.353				*ZFP64*	Body

cg10882304	10	123,923,099	3.47E-31	0.353	0.354				*TACC2*	Body
cg05963821	3	115,377,939	0.002977	0.353	0.267				*GAP43*	Body
cg03962527	10	111,766,879	7.07E-17	0.353	0.344				*ADD3*	5'UTR
cg20485874	7	102,553,437	1.28E-31	0.353	0.324				*LRRC17*	5'UTR
cg08269389	1	181,074,009	0.008348	0.353	0.132					
cg27569446	3	50,378,431	1.83E-07	0.353	0.343				*RASSF1*	TSS200
cg03280622	8	145,023,013	2E-10	0.353	0.307				*PLEC1*	Body
cg12242450	6	10,555,808	1.58E-06	0.353	0.361				*GCNT2*	TSS200
cg14776321	10	27,220,441	7.23E-08	0.353	0.325				*NCRNA00202*	Body
cg16529477	2	223,164,854	2.64E-05	0.353	0.387				*PAX3*	TSS1500
cg09501687	5	150,169,781	3.87E-19	0.353	0.350				*C5orf62*	Body
cg14663177	2	38,373,737	2.3E-07	0.353	0.344				*C2orf58*	Body
cg13331550	1	151,118,416	5.03E-09	0.353	0.332				*SEMA6C*	5'UTR
cg07113642	20	2,781,262	0.004529	0.353	0.226				*CPXM1*	5'UTR
cg01749491	2	176,948,693	0.025036	0.353	0.432				*EVX2*	TSS200
cg01647632	15	89,438,905	1.29E-77	0.353	0.333				*HAPLN3*	TSS200
cg12739419	7	116,140,593	1.31E-40	0.353	0.322				*CAV2*	Body
cg00257296	12	120,799,373	1.19E-24	0.353	0.341				*MSI1*	Body
cg22230604	1	119,870,287	5.12E-05	0.353	0.250					
cg19596273	10	30,724,086	0.000114	0.353	0.283				*MAP3K8*	5'UTR
cg00097146	2	45,171,818	1.23E-07	0.353	0.340				*SIX3*	Body
cg02990368	1	204,120,649	6.15E-11	0.353	0.288				*ETNK2*	Body
cg09556700	1	2,230,668	3.21E-15	0.353	0.367				*SKI*	Body
cg21927177	14	65,172,436	3.02E-26	0.353	0.355				*PLEKHG3*	5'UTR
cg13511752	10	74,094,697	1.77E-14	0.353	0.315				*DNAJB12*	3'UTR
cg05194726	12	2,944,480	2.08E-06	0.353	0.212	5.51E-12	0.214		*NRIP2*	TSS1500
cg27182047	1	110,199,061	1.9E-08	0.353	0.346				*GSTM4*	Body
cg07599133	19	35,068,628	3.04E-11	0.353	0.327				*LOC643719*	TSS200
cg02453146	1	53,068,587	4.41E-09	0.353	0.251				*GPX7*	Body
cg00388391	1	18,459,578	1.53E-07	0.353	0.286				*IGSF21*	Body
cg13800209	15	37,390,284	0.02199	0.353	0.429				*MEIS2*	1stExon
cg11795854	5	72,598,965	9.77E-05	0.353	0.338					
cg10065823	9	96,108,467	8.11E-09	0.352	0.340				*C9orf129*	1stExon
cg04042468	22	43,506,033	2.92E-15	0.352	0.315				*BIK*	TSS1500

cg23515702	2	206,673,425	7.84E-36	0.352	0.347					
cg01861913	3	61,550,569	6.34E-23	0.352	0.339				*PTPRG*	Body
cg16173191	4	82,135,607	6.25E-05	0.352	0.272					
cg26150462	10	11,206,868	5.91E-05	0.352	0.171				*CUGBP2*	TSS200
cg15143037	7	75,639,754	1.16E-29	0.352	0.348				*STYXL1*	Body
cg11202265	7	158,362,907	4.04E-15	0.352	0.347				*PTPRN2*	Body
cg02388378	16	54,968,727	0.00113	0.352	0.421					
cg09938227	1	156,390,124	1.92E-11	0.352	0.374				*C1orf61*	5'UTR
cg14527439	7	158,266,198	4.57E-23	0.352	0.348				*PTPRN2*	Body
cg14470895	5	115,152,431	0.002236	0.352	0.191				*CDO1*	TSS200
cg02681842	8	145,033,310	1.5E-13	0.352	0.344				*PLEC1*	Body
cg13081720	5	150,399,890	4.04E-05	0.352	0.191				*GPX3*	TSS200
cg10953487	6	44,529,157	7.31E-14	0.352	0.302					
cg27641076	11	62,211,788	2.72E-22	0.352	0.333				*AHNAK*	Body
cg03563630	20	37,435,477	8.44E-09	0.352	0.323				*PPP1R16B*	5'UTR
cg10903903	6	27,647,843	0.018644	0.352	0.369					
cg18081258	14	21,494,161	3.27E-26	0.352	0.337	1.56E-20	0.223		*NDRG2*	TSS1500
cg08249424	6	4,775,222	8.38E-07	0.352	0.268				*CDYL*	TSS1500
cg10866060	12	83,592,588	2.21E-17	0.352	0.370					
cg17305266	6	1,391,265	0.002042	0.352	0.358				*FOXF2*	1stExon
cg18507379	5	140,787,507	0.011127	0.352	0.469				*PCDHGA4*	Body
cg05003322	1	32,169,706	9.58E-12	0.352	0.319				*COL16A1*	1stExon
cg01054354	15	100,272,751	2.42E-24	0.352	0.328				*LYSMD4*	5'UTR
cg25266629	10	102,894,148	6.84E-05	0.352	0.349				*TLX1*	Body
cg19111999	10	17,270,087	8.1E-07	0.352	0.328				*VIM*	TSS200
cg27554954	15	60,691,595	6.82E-28	0.352	0.346				*ANXA2*	TSS1500
cg24661595	11	70,456,848	1.23E-29	0.352	0.349				*SHANK2*	Body
cg24013620	8	144,798,688	4.07E-15	0.352	0.355				*MAPK15*	Body
cg16955889	3	181,445,647	0.000118	0.352	0.333				*SOX2OT*	Body
cg09820244	1	54,790,722	7.32E-23	0.352	0.341				*SSBP3*	Body
cg24725263	12	56,101,328	5.59E-24	0.352	0.312			Yes	*ITGA7*	1stExon
cg12441967	22	17,602,561	6.49E-16	0.352	0.283				*CECR6*	TSS1500
cg17017670	X	68,525,613	7.55E-07	0.352	0.329					
cg25913233	5	151,066,683	2.8E-21	0.352	0.334	6.09E-22	0.223		*SPARC*	TSS200

cg23483765	5	156,886,996	0.001708	0.352	0.235				*NIPAL4*	TSS200
cg08135379	12	47,474,763	2.51E-05	0.352	0.337				*AMIGO2*	TSS1500
cg02063759	1	119,543,295	8.92E-08	0.352	0.304					
cg23752752	7	4,778,908	2.25E-32	0.352	0.335				*FOXK1*	Body
cg07660671	8	10,586,967	6.01E-14	0.351	0.305				*SOX7*	Body
cg25905674	17	43,047,856	0.028222	0.351	0.287					
cg13696283	13	74,707,153	2.38E-05	0.351	0.398				*KLF12*	5'UTR
cg14913695	22	50,449,520	1.54E-23	0.351	0.342				*IL17REL*	5'UTR
cg10983208	10	73,848,320	4.67E-07	0.351	0.210	4.27E-11	0.163		*SPOCK2*	5'UTR
cg19915711	13	43,566,421	0.000311	0.351	0.347				*EPSTI1*	TSS200
cg03970036	2	220,174,232	0.000343	0.351	0.441				*PTPRN*	TSS200
cg21739934	11	116,371,391	7.63E-07	0.351	0.289					
cg27647152	20	3,220,985	9.62E-24	0.351	0.318					
cg07605211	1	223,538,359	0.00115	0.351	0.183				*SUSD4*	TSS1500
cg07891473	3	64,670,640	3.44E-07	0.351	0.269				*ADAMTS9*	Body
cg07848310	5	43,603,786	0.018757	0.351	0.200				*NNT*	5'UTR
cg11152302	14	24,867,472	7.37E-33	0.351	0.336				*NYNRIN*	TSS1500
cg07688604	12	133,065,928	0.002783	0.351	0.273				*FBRSL1*	TSS1500
cg27422722	6	28,574,662	0.000779	0.351	0.303					
cg19045644	4	57,976,756	0.022431	0.351	0.377				*IGFBP7*	TSS1500
cg13879495	22	22,005,669	2.32E-09	0.351	0.281					
cg22341310	19	48,048,583	7.38E-14	0.351	0.349	6.23E-18	0.224		*ZNF541*	Body
cg06145435	7	1,022,769	4.39E-20	0.351	0.324				*CYP2W1*	TSS200
cg19466563	4	88,450,506	2.21E-21	0.351	0.333	1.12E-27	0.236		*SPARCL1*	1stExon
cg22616881	7	96,642,096	3.95E-08	0.351	0.368				*DLX6AS*	Body
cg18328206	1	206,681,378	0.006182	0.351	0.347				*RASSF5*	1stExon
cg24865779	21	34,775,042	0.005947	0.351	0.200				*IFNGR2*	TSS200
cg21429394	12	100,750,899	4.79E-09	0.351	0.290				*SLC17A8*	1stExon
cg19647813	13	96,296,144	1.82E-06	0.351	0.353				*DZIP1*	5'UTR
cg22855020	8	42,356,731	5.77E-16	0.351	0.347				*SLC20A2*	5'UTR
cg09407917	X	130,192,151	8.72E-05	0.351	0.324				*FLJ30058*	TSS200
cg22645427	20	25,129,520	1.12E-08	0.351	0.300				*LOC284798*	TSS1500
cg19544662	2	88,752,056	0.016403	0.351	0.167				*FOXI3*	TSS200
cg08314603	12	81,471,311	1.15E-08	0.351	0.317				*ACSS3*	TSS1500

cg22535307	15	48,938,370	0.004042	0.351	0.168				*FBN1*	TSS1500
cg09382127	5	3,592,466	4.01E-11	0.351	0.365					
cg15233062	12	54,447,349	3.98E-06	0.351	0.352				*HOXC4*	5'UTR
cg25837803	1	186,649,909	0.007882	0.351	0.309				*PTGS2*	TSS1500
cg02503159	6	5,026,324	0.018534	0.351	0.371					
cg01047613	20	31,446,682	4.49E-10	0.351	0.360				*EFCAB8*	TSS200
cg01029638	7	120,628,874	6.64E-05	0.351	0.287				*C7orf58*	TSS1500
cg25185173	4	154,712,580	0.003285	0.351	0.452					
cg03762694	6	134,499,419	1.81E-11	0.351	0.360				*SGK1*	Body
cg08714753	11	35,639,590	4.58E-09	0.350	0.275				*FJX1*	TSS200
cg10106804	1	35,258,594	0.000612	0.350	0.357				*GJA4*	TSS200
cg21490980	12	103,311,138	0.002304	0.350	0.190				*PAH*	5'UTR
cg00764109	1	114,697,582	1.29E-06	0.350	0.326				*SYT6*	TSS1500
cg01297808	10	94,180,551	2.61E-10	0.350	0.294					
cg08260891	20	44,518,802	3.68E-11	0.350	0.232	2.54E-10	0.163		*CTSA*	TSS1500
cg26607337	5	145,758,881	2.65E-22	0.350	0.328					
cg27223047	5	127,874,825	0.000165	0.350	0.380	4.92E-32	0.300		*FBN2*	TSS1500
cg19309616	1	41,851,836	1.16E-20	0.350	0.338					
cg00221185	6	30,652,268	9.52E-12	0.350	0.370				*KIAA1949*	Body
cg10391212	4	47,840,136	1.99E-08	0.350	0.316				*CORIN*	TSS200
cg16587010	3	58,523,130	3.07E-32	0.350	0.354				*ACOX2*	TSS1500
cg23274561	1	156,084,271	4.88E-22	0.350	0.313				*LMNA*	TSS200
cg23391107	17	45,924,227	1.8E-06	0.350	0.309				*SP6*	3'UTR
cg18334392	5	9,547,825	4.65E-06	0.350	0.318				*SNORD123*	TSS1500
cg17450733	12	45,443,875	2.45E-32	0.350	0.330				*DBX2*	Body
cg14164044	15	89,952,135	4.41E-09	0.350	0.367					
cg14196395	2	71,823,517	1.24E-07	0.350	0.265				*DYSF*	Body
cg17101450	10	102,900,365	0.000217	0.350	0.323					
cg24896649	4	188,916,726	2.51E-08	0.350	0.307				*ZFP42*	TSS200
cg22233512	17	55,543,388	6.49E-22	0.350	0.336				*MSI2*	Body
cg20066496	15	43,213,993	0.000244	0.350	0.183				*TTBK2*	TSS1500
cg21655480	X	21,677,052	1.08E-07	0.350	0.302	8.5E-16	0.194		*KLHL34*	TSS1500
cg16231923	15	73,923,634	1.69E-08	0.350	0.321				*NPTN*	Body
cg06568535	3	29,323,042	5.08E-06	0.350	0.379				*RBMS3*	1stExon

cg24862743	19	2,719,889	2.32E-22	0.350	0.365				*DIRAS1*	5'UTR
cg26579601	10	30,724,020	7.01E-05	0.350	0.274				*MAP3K8*	5'UTR
cg12169365	14	92,979,709	5.31E-07	0.350	0.265				*RIN3*	TSS1500
cg05315321	14	68,844,276	1.5E-14	0.350	0.353				*RAD51L1*	Body
cg10512203	1	40,780,905	9.07E-16	0.350	0.327				*COL9A2*	Body
cg25293325	17	873,413	2.16E-17	0.350	0.336				*NXN*	Body
cg08059845	19	50,015,975	0.000134	0.349	0.266				*FCGRT*	5'UTR
cg15132565	5	72,740,154	0.003141	0.349	0.312					
cg11986813	10	43,429,144	0.001399	0.349	0.404					
cg12126901	1	61,623,366	2.98E-11	0.349	0.351				*NFIA*	Body
cg07686872	7	156,794,879	3.44E-09	0.349	0.362					
cg01906944	20	45,439,769	7.39E-21	0.349	0.344					
cg26924440	1	19,276,353	5.68E-34	0.349	0.331				*IFFO2*	Body
cg09127400	6	30,712,331	0.002309	0.349	0.349				*IER3*	TSS200
cg23189044	11	74,178,114	8.19E-05	0.349	0.270	6.12E-09	0.163		*KCNE3*	5'UTR
cg09465698	5	140,787,623	2.03E-07	0.349	0.413				*PCDHGA4*	Body
cg14136420	9	128,728,906	2.15E-22	0.349	0.307				*PBX3*	Body
cg19769827	1	203,259,772	0.013101	0.349	0.172					
cg14102988	9	113,801,337	5.69E-06	0.349	0.263				*LPAR1*	TSS1500
cg15447787	15	29,967,531	4.73E-32	0.349	0.325					
cg15608277	12	53,184,086	7.86E-06	0.349	0.410				*KRT3*	Body
cg23561979	2	42,329,535	6.78E-05	0.349	0.364					
cg13279244	7	20,838,581	1.43E-05	0.349	0.289					
cg03864000	11	111,411,381	5.77E-05	0.349	0.330				*LAYN*	5'UTR
cg20634514	1	44,703,750	3.14E-24	0.349	0.353				*ERI3*	Body
cg25154801	8	41,655,826	2.04E-10	0.349	0.297				*ANK1*	TSS1500
cg08229358	1	179,544,765	1.43E-16	0.349	0.319				*NPHS2*	1stExon
cg10375890	1	50,892,511	0.005611	0.349	0.421					
cg19074496	2	220,117,868	0.000292	0.349	0.403				*TUBA4B*	TSS200
cg14999001	7	45,197,441	0.003738	0.349	0.217				*RAMP3*	1stExon
cg05745631	6	30,095,178	3.68E-35	0.349	0.348					
cg10608596	19	11,785,125	2.79E-12	0.349	0.324				*ZNF833*	Body
cg11394785	5	179,221,090	3.95E-34	0.349	0.330	6.79E-23	0.179		*LTC4S*	1stExon
cg18919225	12	103,311,494	0.000548	0.349	0.184				*PAH*	TSS200

cg15797110	7	27,196,314	2.48E-06	0.349	0.354				*HOXA7*	TSS200
cg01791587	1	110,693,985	1.82E-26	0.349	0.347				*SLC6A17*	5'UTR
cg26268276	11	33,038,313	1.03E-17	0.349	0.368				*DEPDC7*	Body
cg24467291	12	122,907,814	3.11E-16	0.349	0.334	2.71E-33	0.264		*CLIP1*	TSS1500
cg16032894	11	30,605,411	1.71E-14	0.349	0.306				*MPPED2*	5'UTR
cg13337731	7	73,011,308	4.16E-07	0.349	0.379				*MLXIPL*	Body
cg17347389	14	51,562,646	0.000787	0.349	0.233	1.5E-11	0.115		*TRIM9*	TSS1500
cg18107232	13	95,620,997	2.87E-06	0.349	0.331					
cg00727630	20	50,721,366	5.97E-11	0.349	0.337				*ZFP64*	Body
cg06264679	6	32,121,433	3.9E-23	0.349	0.318				*PPT2*	Body
cg10804678	3	72,788,309	5.05E-06	0.349	0.255					
cg24595606	6	108,492,358	6.07E-07	0.349	0.360				*NR2E1*	Body
cg12298268	3	139,257,866	7.67E-10	0.349	0.351				*RBP1*	Body
cg06329684	4	140,187,743	1.17E-08	0.349	0.348				*C4orf49*	3'UTR
cg18783429	5	92,414,398	1.05E-10	0.349	0.279					
cg08965235	11	65,325,158	4.9E-05	0.348	0.252	1.81E-37	0.278		*LTBP3*	1stExon
cg19034038	12	45,443,996	1.67E-27	0.348	0.313				*DBX2*	Body
cg04108939	1	45,250,181	0.013784	0.348	0.401				*BEST4*	Body
cg18454685	17	48,639,239	5.43E-07	0.348	0.323	1.12E-24	0.227		*CACNA1G*	Body
cg13763783	10	95,517,562	2.41E-06	0.348	0.395				*LGI1*	TSS200
cg16335762	16	66,637,919	0.016261	0.348	0.122	5.23E-10	0.151		*CMTM3*	TSS1500
cg09704750	21	27,011,139	2.02E-07	0.348	0.383				*JAM2*	TSS1500
cg13337949	6	30,711,586	1.71E-26	0.348	0.331				*IER3*	3'UTR
cg00031256	6	2,842,255	4.29E-06	0.348	0.336				*SERPINB1*	TSS200
cg09671837	7	143,582,308	5.2E-16	0.348	0.334				*FAM115A*	5'UTR
cg13453424	9	98,260,248	1.51E-24	0.348	0.349				*PTCH1*	5'UTR
cg00363080	10	1,454,292	1.7E-24	0.348	0.320				*ADARB2*	Body
cg00169617	3	87,138,700	0.000281	0.348	0.219					
cg25792593	19	8,559,219	6.04E-25	0.348	0.327				*PRAM1*	Body
cg15182643	5	38,257,516	9.56E-15	0.348	0.326				*EGFLAM*	TSS1500
cg10712623	3	25,469,577	6.37E-14	0.348	0.318	3.77E-18	0.129		*RARB*	TSS1500
cg24335070	2	179,914,864	2.12E-08	0.348	0.322					
cg14039306	4	4,862,240	2E-09	0.348	0.333				*MSX1*	Body
cg11756095	1	32,410,614	2.67E-06	0.348	0.358					

cg04636402	5	139,284,774	4.73E-39	0.348	0.341				*NRG2*	Body
cg11709405	1	24,649,398	3.96E-15	0.348	0.365				*GRHL3*	TSS200
cg25433316	2	10,197,543	1.59E-05	0.348	0.206				*CYS1*	3'UTR
cg20219035	12	14,720,858	3.83E-07	0.348	0.230				*PLBD1*	TSS200
cg19906093	2	191,045,697	3.3E-12	0.348	0.330				*C2orf88*	5'UTR
cg17480760	8	86,350,889	2.7E-06	0.348	0.356				*CA3*	TSS200
cg18141830	6	137,242,085	7.85E-09	0.348	0.373				*SLC35D3*	TSS1500
cg10277365	X	49,022,793	1.14E-09	0.348	0.305				*MAGIX*	3'UTR
cg02394955	10	47,083,426	0.000373	0.348	0.179				*PPYR1*	TSS200
cg19929126	7	28,998,195	0.008037	0.348	0.244				*TRIL*	TSS200
cg12055515	2	66,735,203	2.13E-19	0.348	0.320				*MEIS1*	Body
cg26296894	3	20,183,665	1.32E-23	0.348	0.344				*KAT2B*	Body
cg12926938	8	93,114,150	0.000181	0.348	0.374					
cg24393055	5	141,705,521	4.58E-07	0.348	0.247				*SPRY4*	TSS1500
cg24935217	11	34,517,699	5.93E-16	0.348	0.371				*ELF5*	Body
cg17165580	1	156,676,581	0.000203	0.348	0.335				*CRABP2*	TSS1500
cg05870108	X	106,749,745	1.9E-46	0.348	0.352					
cg22396555	13	28,502,461	3.31E-09	0.348	0.340					
cg04691781	16	89,668,842	5.72E-11	0.348	0.303					
cg10024587	10	105,211,753	3.22E-07	0.348	0.297				*CALHM2*	5'UTR
cg20443254	12	108,169,020	1.03E-06	0.348	0.282				*ASCL4*	1stExon
cg25767345	18	54,788,969	2.64E-12	0.348	0.318					
cg08940787	17	62,777,648	0.000468	0.348	0.259				*LOC146880*	Body
cg11142705	3	42,306,974	0.000153	0.348	0.379				*CCK*	TSS1500
cg22911898	11	94,473,405	2.82E-11	0.348	0.269					
cg26560222	1	50,888,826	0.000117	0.348	0.358				*DMRTA2*	1stExon
cg16304521	14	22,191,827	6.3E-12	0.348	0.338					
cg27090201	5	134,914,717	0.002025	0.348	0.304				*CXCL14*	5'UTR
cg02043994	6	15,244,878	8.65E-13	0.348	0.304					
cg19059495	6	30,095,495	1.32E-15	0.347	0.319					
cg21645554	11	111,169,473	3.25E-26	0.347	0.350				*C11orf93*	TSS1500
cg10236452	1	208,132,590	0.001196	0.347	0.335					
cg09435929	8	86,350,913	0.001252	0.347	0.341				*CA3*	TSS200
cg17764549	7	158,287,907	2.47E-22	0.347	0.332				*PTPRN2*	Body

cg02786012	4	57,371,589	4.49E-05	0.347	0.348				*ARL9*	5'UTR
cg15750501	2	123,419,027	7.16E-25	0.347	0.351					
cg24634333	6	111,887,834	2.64E-22	0.347	0.355				*TRAF3IP2*	Body
cg02622316	6	28,367,410	1.25E-05	0.347	0.303	6.74E-25	0.256		*ZSCAN12*	Body
cg21389753	17	59,480,674	3.84E-11	0.347	0.344				*TBX2*	Body
cg04003500	9	71,788,759	9.93E-24	0.347	0.355				*TJP2*	TSS1500
cg22418737	7	139,930,066	0.000213	0.347	0.233					
cg22136365	16	71,611,043	5.54E-21	0.347	0.340	1.1E-25	0.265		*TAT*	TSS200
cg14380270	17	33,700,747	0.00471	0.347	0.391				*SLFN11*	TSS200
cg01682504	12	57,620,607	6.55E-07	0.347	0.382					
cg16796458	16	85,254,655	2.4E-21	0.347	0.342					
cg25334934	2	121,269,348	2.33E-14	0.347	0.345					
cg12081743	7	18,535,499	6.31E-05	0.347	0.325				*HDAC9*	TSS1500
cg02174092	10	43,846,539	0.01226	0.347	0.197					
cg19788186	21	39,669,558	1.22E-15	0.347	0.317				*KCNJ15*	5'UTR
cg17199325	14	23,821,229	6.47E-15	0.347	0.299				*SLC22A17*	Body
cg03117976	18	11,149,435	0.006172	0.347	0.369				*FAM38B*	TSS1500
cg13476133	7	44,185,646	0.001557	0.347	0.369				*GCK*	Body
cg04555220	5	9,546,695	1.09E-07	0.347	0.294				*SEMA5A*	TSS1500
cg01406988	2	66,662,810	6.03E-05	0.347	0.395				*MEIS1*	5'UTR
cg17285883	1	17,896,290	1.21E-18	0.347	0.342				*ARHGEF10L*	5'UTR
cg26713109	17	754,463	8.47E-15	0.347	0.320				*NXN*	Body
cg02659086	11	67,350,976	0.002026	0.347	0.337				*GSTP1*	TSS200
cg26196385	5	113,392,117	3.23E-26	0.347	0.341					
cg11770080	5	78,408,179	4.37E-09	0.347	0.344				*BHMT*	Body
cg16425038	14	24,641,194	1.83E-11	0.347	0.352				*REC8*	TSS200
cg27539986	1	6,664,103	1.16E-20	0.347	0.337				*KLHL21*	TSS1500
cg07266404	12	54,447,584	1.9E-05	0.347	0.275				*HOXC4*	5'UTR
cg05079548	7	128,337,621	2.41E-06	0.347	0.274					
cg11536474	2	63,286,049	1.8E-06	0.347	0.335					
cg19057001	5	150,538,165	4.83E-21	0.347	0.319				*ANXA6*	TSS1500
cg13887205	5	139,525,946	1.75E-14	0.347	0.332					
cg24676120	12	102,821,557	1.02E-20	0.346	0.325				*IGF1*	Body
cg00165323	1	70,035,080	0.000682	0.346	0.314					

cg24115232	5	1,243,907	3.19E-18	0.346	0.365				*SLC6A18*	Body
cg23425970	2	129,076,394	0.009268	0.346	0.288				*HS6ST1*	TSS1500
cg06492744	11	65,406,254	1.4E-13	0.346	0.321				*SIPA1*	5'UTR
cg20134598	X	54,947,308	1.2E-08	0.346	0.381				*TRO*	5'UTR
cg02076598	22	25,423,801	2.78E-19	0.346	0.339				*KIAA1671*	TSS200
cg17822706	19	18,415,538	6.93E-26	0.346	0.338					
cg12379948	17	44,896,424	0.003322	0.346	0.416				*WNT3*	TSS1500
cg26311610	7	73,593,138	3.3E-24	0.346	0.364				*EIF4H*	Body
cg03046886	1	18,869,151	3.02E-25	0.346	0.326					
cg06592987	7	149,112,142	1.02E-07	0.346	0.347					
cg12136950	1	226,849,798	2.42E-14	0.346	0.357				*ITPKB*	Body
cg25374649	18	13,611,400	0.000207	0.346	0.318				*C18orf1*	TSS1500
cg10502231	1	224,363,449	1.94E-26	0.346	0.357					
cg24091270	6	29,894,908	0.00163	0.346	0.360				*HCG4P6*	TSS1500
cg02579022	5	137,828,267	1.88E-06	0.346	0.315					
cg20295992	6	108,440,280	0.001366	0.346	0.365					
cg26435178	10	73,848,458	3.14E-19	0.346	0.286				*SPOCK2*	5'UTR
cg20014398	11	31,825,792	0.003572	0.346	0.320				*PAX6*	Body
cg23089272	15	39,985,431	1.65E-08	0.346	0.331				*FSIP1*	Body
cg06040206	2	172,961,280	0.014515	0.346	0.217					
cg20927661	2	5,837,002	0.002938	0.346	0.318				*SOX11*	3'UTR
cg06692785	1	180,882,681	7.49E-20	0.346	0.366				*KIAA1614*	Body
cg06263461	20	45,280,237	1.83E-15	0.346	0.338				*SLC13A3*	TSS200
cg13104713	1	46,632,811	7.32E-10	0.346	0.367					
cg25608973	2	21,022,431	0.002403	0.346	0.336				*C2orf43*	5'UTR
cg00809863	1	15,251,881	4.85E-16	0.346	0.304				*KIAA1026*	Body
cg23168425	7	157,561,848	7.29E-13	0.346	0.342				*PTPRN2*	Body
cg18343437	8	142,528,415	1.24E-14	0.346	0.350					
cg21091128	14	104,583,799	9.03E-14	0.346	0.312				*MIR203*	Body
cg16517394	1	173,176,362	1.78E-14	0.346	0.343	2.03E-15	0.238		*TNFSF4*	5'UTR
cg03741406	1	40,255,103	0.000328	0.346	0.256				*BMP8B*	TSS1500
cg09846875	7	143,582,630	1.37E-16	0.346	0.344				*FAM115A*	5'UTR
cg10462187	10	124,067,402	1.25E-15	0.346	0.362				*BTBD16*	Body
cg03575641	7	128,809,442	3.91E-06	0.345	0.325					

cg18536496	X	101,906,035	0.002254	0.345	0.385				*GPRASP1*	TSS1500
cg08794940	6	30,711,073	3.31E-05	0.345	0.243				*IER3*	3'UTR
cg03122624	2	56,150,341	1.48E-14	0.345	0.306				*EFEMP1*	1stExon
cg25865108	6	45,559,104	1.48E-23	0.345	0.316					
cg25670330	3	138,665,984	0.034284	0.345	0.277				*FOXL2*	TSS200
cg20457732	1	71,172,486	1.36E-05	0.345	0.271					
cg14578894	4	87,281,511	2.72E-09	0.345	0.350				*MAPK10*	TSS200
cg16483725	5	122,621,240	0.001104	0.345	0.340					
cg27056119	6	2,842,239	4.57E-05	0.345	0.337	4.62E-21	0.258		*SERPINB1*	TSS200
cg10633526	3	13,560,246	7.45E-18	0.345	0.333					
cg07984133	13	28,544,592	1.54E-05	0.345	0.307				*CDX2*	TSS1500
cg24519146	6	33,160,318	0.004267	0.345	0.303				*COL11A2*	TSS200
cg16519192	12	116,037,126	1.9E-06	0.345	0.294					
cg22290648	19	39,694,709	2.35E-05	0.345	0.308				*SYCN*	1stExon
cg11648594	1	1,566,860	0.00021	0.345	0.378				*MMP23A*	TSS1500
cg01287975	7	97,361,241	2.47E-07	0.345	0.303				*TAC1*	TSS200
cg01597387	14	24,458,208	1.82E-07	0.345	0.340				*DHRS4L2*	1stExon
cg19204958	10	415,937	2.94E-28	0.345	0.331				*DIP2C*	Body
cg08588180	6	25,138,905	0.000644	0.345	0.257				*CMAH*	TSS1500
cg02599716	2	10,231,486	4.37E-05	0.345	0.316					
cg17926950	19	13,983,613	5.76E-06	0.345	0.415					
cg14973798	1	171,723,758	2.21E-14	0.345	0.327					
cg01308913	X	25,040,907	0.001202	0.345	0.385					
cg02280532	2	160,761,244	3.9E-08	0.345	0.283				*LY75*	5'UTR
cg02589791	7	63,955,666	2.41E-29	0.345	0.321					
cg01383955	22	42,828,447	1.7E-19	0.345	0.331				*NFAM1*	TSS200
cg00456651	1	31,380,425	4.64E-24	0.345	0.341				*SDC3*	Body
cg11626007	20	43,743,835	6.87E-11	0.345	0.332				*WFDC5*	TSS200
cg08340755	20	30,176,106	2.59E-14	0.345	0.301					
cg07703462	7	149,487,338	0.002755	0.345	0.262				*SSPO*	Body
cg09668030	1	27,852,173	2.15E-17	0.345	0.333					
cg05890620	19	10,397,612	3.74E-10	0.345	0.339				*ICAM4*	TSS200
cg03513363	20	30,458,745	1.17E-08	0.345	0.289	1.27E-33	0.216		*TTLL9*	5'UTR
cg21461649	7	117,119,938	1.73E-06	0.345	0.329				*CFTR*	TSS200

cg04737881	4	183,060,943	1.81E-14	0.345	0.327				*MGC45800*	Body
cg04066019	3	16,554,466	0.019609	0.345	0.410				*RFTN1*	5'UTR
cg26187876	2	191,514,902	0.000505	0.344	0.177				*NAB1*	5'UTR
cg03940047	20	62,120,117	1.79E-17	0.344	0.315				*EEF1A2*	Body
cg08318283	15	73,611,202	4.72E-06	0.344	0.276					
cg14381919	11	12,701,969	0.001993	0.344	0.351				*TEAD1*	5'UTR
cg03714619	2	45,160,445	4.87E-06	0.344	0.356					
cg22175814	17	71,739,339	2.95E-05	0.344	0.272					
cg02144933	2	201,450,690	0.000122	0.344	0.319	2.77E-23	0.326		*AOX1*	TSS200
cg11846236	3	48,632,568	2.8E-06	0.344	0.327	2.19E-25	0.242		*COL7A1*	1stExon
cg17199320	1	231,177,008	0.000313	0.344	0.343				*FAM89A*	TSS1500
cg18279326	10	102,475,324	3.4E-14	0.344	0.341					
cg03096126	18	56,531,159	0.00019	0.344	0.197				*ZNF532*	5'UTR
cg23018117	7	150,413,687	9.31E-17	0.344	0.338				*GIMAP1*	TSS200
cg12245706	2	71,823,484	3.39E-07	0.344	0.249				*DYSF*	Body
cg14824052	10	2,951,326	4.17E-11	0.344	0.321					
cg21088420	5	7,849,836	3.25E-24	0.344	0.326				*C5orf49*	Body
cg14732324	5	528,621	0.008309	0.344	0.433					
cg26911787	12	96,587,734	0.004729	0.344	0.281	7.22E-13	0.105		*ELK3*	TSS1500
cg18648037	3	106,917,496	8.94E-28	0.344	0.346				*LOC100302640*	Body
cg21439763	1	173,176,366	6.68E-13	0.344	0.335				*TNFSF4*	5'UTR
cg25987744	19	46,916,588	3.7E-09	0.344	0.297				*CCDC8*	1stExon
cg12298212	6	27,065,003	1.73E-07	0.344	0.283					
cg19884345	6	26,189,379	0.00336	0.344	0.320				*HIST1H4D*	TSS200
cg12776171	17	72,442,928	5.59E-17	0.344	0.335				*GPRC5C*	Body
cg05535113	16	71,559,579	2.18E-10	0.344	0.313	8.22E-17	0.236		*CHST4*	TSS1500
cg00465970	3	129,024,601	2.16E-17	0.344	0.350					
cg01405004	3	68,980,539	9.9E-11	0.344	0.307				*FAM19A4*	5'UTR
cg19019849	4	13,533,145	8.74E-07	0.344	0.367					
cg19818890	11	76,033,391	1.19E-07	0.344	0.312					
cg03307401	19	51,568,260	0.03186	0.344	0.403				*KLK13*	Body
cg13705284	3	58,523,313	2.14E-22	0.344	0.369				*ACOX2*	TSS1500
cg05977109	10	52,178,181	4.42E-07	0.343	0.362				*SGMS1*	5'UTR
cg13917560	7	157,372,255	9.56E-37	0.343	0.333				*PTPRN2*	Body

cg10512745	1	50,884,480	0.004111	0.343	0.398				*DMRTA2*	Body
cg16890428	19	55,866,412	0.000838	0.343	0.350				*FAM71E2*	3'UTR
cg11615395	4	140,925,869	0.02552	0.343	0.275				*MAML3*	Body
cg02598441	17	62,777,298	0.000226	0.343	0.275				*LOC146880*	Body
cg05264279	7	158,266,136	9.05E-11	0.343	0.336				*PTPRN2*	Body
cg06561329	22	17,602,610	3.43E-18	0.343	0.313				*CECR6*	TSS1500
cg09211763	2	190,044,504	7.26E-06	0.343	0.352				*COL5A2*	1stExon
cg06211837	1	76,540,438	0.000814	0.343	0.314				*ST6GALNAC3*	1stExon
cg22864077	2	131,185,333	1.03E-07	0.343	0.353					
cg10663628	2	23,798,452	4.76E-21	0.343	0.327				*KLHL29*	Body
cg07223177	X	46,434,198	0.000757	0.343	0.121				*CHST7*	1stExon
cg26371061	5	71,014,857	2.28E-12	0.343	0.347				*CARTPT*	TSS200
cg05463589	16	88,706,426	1.82E-09	0.343	0.281				*IL17C*	Body
cg18465484	19	17,138,051	0.000511	0.343	0.279				*CPAMD8*	TSS1500
cg18368411	1	31,845,959	0.003483	0.343	0.237				*FABP3*	TSS200
cg13338877	6	29,974,531	3.71E-13	0.343	0.337				*HLA-J*	Body
cg20291855	4	13,524,143	0.01542	0.343	0.378					
cg10480343	15	48,938,347	0.009606	0.343	0.149				*FBN1*	TSS1500
cg12698813	12	115,136,060	4.08E-12	0.343	0.321					
cg11421255	15	60,690,974	3.06E-13	0.343	0.302				*ANXA2*	TSS1500
cg18069125	20	45,439,836	6.55E-13	0.343	0.324					
cg22524061	8	99,959,430	3.76E-11	0.343	0.348				*OSR2*	5'UTR
cg22783308	14	75,894,315	0.001385	0.343	0.154				*JDP2*	TSS200
cg00465319	3	123,339,568	3.59E-10	0.343	0.348				*MYLK*	TSS200
cg25247859	6	30,095,174	6.65E-23	0.343	0.331					
cg06372850	2	164,205,188	5.78E-12	0.343	0.333					
cg08572315	2	85,133,831	0.016043	0.343	0.234					
cg14113919	X	20,134,908	0.00045	0.343	0.294				*MAP7D2*	TSS200
cg09676129	20	60,540,388	0.000002	0.343	0.321					
cg15850390	X	72,668,487	0.004074	0.343	0.311				*CDX4*	Body
cg19472078	17	77,020,166	1.87E-05	0.343	0.307				*C1QTNF1*	TSS200
cg04510919	4	7,282,819	1.66E-08	0.343	0.354				*SORCS2*	Body
cg08146483	1	75,602,412	0.000923	0.343	0.332				*LHX8*	Body
cg04728482	5	72,529,902	0.006226	0.343	0.350					

cg19228034	7	92,672,812	1.83E-15	0.343	0.319					
cg14669379	1	112,058,559	7.85E-23	0.343	0.329				*ADORA3*	Body
cg24479590	17	7,348,322	8.78E-14	0.343	0.307				*CHRNB1*	TSS200
cg07866212	8	23,836,952	1.03E-09	0.343	0.307					
cg13591204	9	38,064,182	2.9E-13	0.343	0.328				*SHB*	Body
cg27018070	1	38,470,578	2.8E-11	0.343	0.274	1.26E-24	0.120		*FHL3*	5'UTR
cg01277506	5	80,690,205	2.25E-09	0.343	0.335				*RNU5E*	Body
cg03345454	2	202,901,428	3.86E-07	0.343	0.366				*FZD7*	1stExon
cg19237879	11	9,113,458	1.26E-05	0.343	0.255	2.42E-27	0.197		*SCUBE2*	TSS1500
cg08377819	6	28,956,268	2.77E-06	0.343	0.270					
cg00011616	17	75,672,423	0.001397	0.343	0.360					
cg13045134	8	103,741,780	8.25E-17	0.343	0.369					
cg09570682	6	106,429,443	1.04E-07	0.342	0.366					
cg09999194	17	77,765,305	0.006765	0.342	0.318					
cg09114581	6	32,077,103	2.29E-09	0.342	0.316				*TNXB*	5'UTR
cg08856529	8	140,714,586	1.65E-07	0.342	0.402				*KCNK9*	Body
cg05119316	6	29,716,135	7.53E-08	0.342	0.263				*LOC285830*	Body
cg07351192	3	5,137,773	1.53E-12	0.342	0.327					
cg27058988	15	67,200,879	5.64E-10	0.342	0.350					
cg22725685	4	74,810,310	0.002202	0.342	0.350					
cg07702750	7	28,998,214	0.004375	0.342	0.246				*TRIL*	TSS200
cg19982230	17	27,045,302	6.87E-14	0.342	0.363				*RAB34*	TSS1500
cg27513574	4	25,657,440	0.003459	0.342	0.192				*SLC34A2*	1stExon
cg24021113	6	3,752,849	1.06E-08	0.342	0.310				*C6orf145*	TSS1500
cg06188744	10	3,108,651	1.08E-21	0.342	0.341				*PFKP*	TSS1500
cg00990022	1	40,138,052	6.1E-18	0.342	0.342				*NT5C1A*	TSS1500
cg09206294	15	42,072,687	1.12E-16	0.342	0.334				*MAPKBP1*	Body
cg24954668	7	20,833,585	0.015972	0.342	0.389					
cg15853125	21	32,932,267	4.84E-05	0.342	0.316				*TIAM1*	TSS1500
cg25315362	3	116,164,576	9.64E-23	0.342	0.310	2E-36	0.231		*LSAMP*	TSS200
cg19871940	14	57,264,287	0.000698	0.342	0.365					
cg00691830	15	37,388,540	4.96E-06	0.342	0.418				*MEIS2*	Body
cg00531137	16	57,643,932	4.72E-06	0.342	0.337					
cg06892726	6	26,087,607	0.000926	0.342	0.353	1.96E-11	0.122		*HFE*	1stExon

cg00450784	12	106,979,062	0.000837	0.342	0.369				*RFX4*	Body
cg18451588	7	29,606,289	1.72E-14	0.342	0.328				*PRR15*	Body
cg24738311	15	89,952,632	8.74E-06	0.342	0.359					
cg08892600	1	223,538,601	3.87E-08	0.342	0.316				*SUSD4*	TSS1500
cg14474520	12	124,781,038	1.07E-20	0.342	0.303				*FAM101A*	5'UTR
cg16370398	12	54,448,913	9.08E-14	0.342	0.325				*HOXC4*	Body
cg18282849	11	86,085,694	4.13E-07	0.342	0.353				*CCDC81*	TSS200
cg08116137	2	73,870,653	3.82E-16	0.342	0.336	2.64E-24	0.212		*NAT8*	TSS1500
cg09106903	1	217,309,568	7.99E-12	0.342	0.341				*ESRRG*	5'UTR
cg06935438	9	4,116,989	2.88E-22	0.342	0.331				*GLIS3*	Body
cg01972751	6	137,814,728	0.001051	0.342	0.291				*OLIG3*	1stExon
cg07915884	1	245,474,048	4.12E-21	0.342	0.333				*KIF26B*	Body
cg11678461	11	3,181,822	2.1E-12	0.342	0.290				*OSBPL5*	5'UTR
cg10126181	6	21,832,051	0.000216	0.342	0.308				*FLJ22536*	Body
cg24158594	8	60,030,763	0.047357	0.342	0.378				*TOX*	Body
cg03299336	11	11,465,911	3.69E-15	0.342	0.277				*GALNTL4*	Body
cg15780398	2	97,439,827	3.11E-07	0.342	0.280			Yes	*CNNM4*	Body
cg24676071	7	45,613,410	1.17E-08	0.342	0.317				*ADCY1*	TSS1500
cg14957943	3	48,632,783	0.001105	0.342	0.372				*COL7A1*	TSS200
cg18356974	7	128,809,100	6.97E-06	0.342	0.318					
cg08352755	5	145,758,879	3.87E-17	0.342	0.304					
cg09789426	X	102,000,698	7.05E-13	0.342	0.266				*BHLHB9*	5'UTR
cg27063372	16	616,857	0.00049	0.342	0.368				*NHLRC4*	TSS200
cg08199758	16	3,062,426	3.03E-08	0.342	0.340				*CLDN9*	TSS200
cg22905097	13	43,566,401	0.000271	0.342	0.331	1.3E-16	0.171		*EPSTI1*	TSS200
cg19323951	11	64,993,076	0.000395	0.342	0.332				*SLC22A20*	Body
cg27488348	12	14,720,834	8.39E-07	0.342	0.270				*PLBD1*	TSS200
cg00558689	6	41,607,109	5.83E-07	0.342	0.306				*MDFI*	Body
cg02090033	3	65,583,617	5.08E-05	0.342	0.352				*MAGI1*	Body
cg25120945	6	29,975,416	6.71E-14	0.342	0.319				*HLA-J*	Body
cg05214748	16	31,214,335	2.61E-18	0.342	0.272				*PYCARD*	TSS200
cg13931925	1	179,545,216	1.13E-05	0.342	0.262				*NPHS2*	TSS200
cg15059804	1	33,766,318	5.12E-18	0.342	0.325				*ZNF362*	3'UTR
cg16201596	6	29,974,568	1.75E-08	0.341	0.316				*HLA-J*	Body

cg17741014	5	80,690,220	2.69E-16	0.341	0.291				*RNU5E*	Body
cg02746725	2	177,054,306	3.63E-14	0.341	0.316				*HOXD1*	Body
cg27453857	17	4,689,640	8.4E-05	0.341	0.241				*VMO1*	1stExon
cg13056495	7	134,143,249	0.000249	0.341	0.278				*AKR1B1*	Body
cg17524136	11	84,028,382	2.23E-09	0.341	0.387				*DLG2*	Body
cg25371919	2	63,284,481	1.61E-07	0.341	0.334					
cg18484034	10	93,647,648	8.42E-05	0.341	0.294					
cg15902390	15	37,387,438	0.001871	0.341	0.395				*MEIS2*	Body
cg07194250	X	133,680,463	0.001414	0.341	0.229				*MGC16121*	Body
cg02067712	13	28,364,724	0.000007	0.341	0.354					
cg21961771	12	100,750,652	4.18E-06	0.341	0.335				*SLC17A8*	TSS1500
cg18544564	2	189,839,120	3.09E-14	0.341	0.340				*COL3A1*	5'UTR
cg24547885	2	179,914,868	9.1E-05	0.341	0.293					
cg04767500	6	38,682,956	2.13E-07	0.341	0.324					
cg12018098	19	38,885,481	2.49E-09	0.341	0.344				*SPRED3*	Body
cg00028013	15	74,218,697	1.38E-20	0.341	0.322				*LOXL1*	TSS200
cg10740054	19	18,548,004	0.000425	0.341	0.210				*ISYNA1*	Body
cg26605683	5	172,751,269	1.46E-05	0.341	0.391				*STC2*	Body
cg06967228	X	68,525,725	9.12E-08	0.341	0.365					
cg15677293	1	236,559,595	0.004455	0.341	0.106				*EDARADD*	Body
cg13343687	3	49,843,809	1.13E-31	0.341	0.334				*UBA7*	Body
cg15057061	3	181,437,299	0.009408	0.341	0.374				*SOX2OT*	Body
cg04384626	4	41,882,580	3.35E-07	0.341	0.302					
cg11282676	6	26,018,003	2.27E-05	0.341	0.292				*HIST1H1A*	5'UTR
cg22590761	15	74,218,921	8.24E-19	0.341	0.297				*LOXL1*	5'UTR
cg17415451	5	11,589,049	3.22E-06	0.341	0.308				*CTNND2*	Body
cg12254845	15	89,952,272	3.77E-09	0.341	0.378					
cg27402429	3	72,788,294	4.54E-10	0.341	0.265					
cg13997864	2	96,990,982	2.52E-06	0.341	0.297				*ITPRIPL1*	TSS1500
cg27142536	11	20,618,198	6.99E-14	0.341	0.323					
cg16438525	3	52,517,742	1.18E-19	0.341	0.331				*NISCH*	Body
cg14665813	11	59,333,490	0.001594	0.341	0.310					
cg07881405	5	3,597,487	0.000286	0.341	0.375				*IRX1*	Body
cg20658918	2	131,185,698	0.000143	0.341	0.307					

cg00081799	1	6,390,793	1.72E-63	0.341	0.326				*ACOT7*	Body
cg18664667	7	105,517,962	5.72E-07	0.341	0.244				*ATXN7L1*	TSS1500
cg04338652	18	13,136,597	4.16E-07	0.341	0.287					
cg01566127	11	63,828,762	4.06E-31	0.341	0.332				*MACROD1*	Body
cg05485137	10	28,035,864	2.21E-08	0.341	0.359				*MKX*	TSS1500
cg00347563	1	78,511,600	0.035841	0.341	0.448				*GIPC2*	1stExon
cg27619475	17	73,083,881	0.001169	0.341	0.152	7.84E-16	0.260		*SLC16A5*	TSS200
cg25291387	6	29,910,237	5.63E-05	0.341	0.259			Yes	*HLA-A*	TSS200
cg00489401	5	180,075,875	2.64E-10	0.341	0.330	2E-33	0.348		*FLT4*	Body
cg17437939	7	97,361,252	0.000133	0.341	0.342				*TAC1*	TSS200
cg26874693	12	111,618,936	1.36E-06	0.341	0.251				*CUX2*	Body
cg14188232	15	68,724,065	0.000226	0.341	0.287	1.6E-31	0.266		*ITGA11*	Body
cg14652638	6	2,842,215	1.73E-06	0.341	0.320				*SERPINB1*	TSS200
cg25402480	12	53,719,777	7.24E-08	0.341	0.362					
cg12738718	6	32,121,204	5.4E-07	0.341	0.408				*PPT2*	TSS200
cg14251870	6	137,809,529	0.000201	0.341	0.394					
cg26237168	8	10,872,648	0.000101	0.341	0.358				*XKR6*	Body
cg07133749	17	29,249,870	0.000258	0.341	0.348				*ADAP2*	Body
cg18267049	10	101,875,028	2.85E-06	0.341	0.331					
cg21766898	6	50,814,089	0.000551	0.341	0.351				*TFAP2B*	3'UTR
cg26104932	2	219,647,360	7.4E-05	0.341	0.213				*CYP27A1*	Body
cg21819468	1	13,910,569	0.00344	0.341	0.413				*PDPN*	TSS1500
cg05669497	13	113,655,468	2.64E-10	0.341	0.268				*MCF2L*	Body
cg11014463	6	56,818,479	7.1E-07	0.341	0.338				*BEND6*	TSS1500
cg15826479	17	78,806,493	1.44E-13	0.341	0.334				*RPTOR*	Body
cg13096007	4	24,802,340	1.07E-08	0.341	0.351				*SOD3*	3'UTR
cg14703002	6	26,018,058	0.001439	0.340	0.278				*HIST1H1A*	TSS200
cg06457011	20	39,767,490	0.003381	0.340	0.245				*PLCG1*	Body
cg03585778	1	205,537,837	2.66E-06	0.340	0.253				*MFSD4*	TSS1500
cg14807945	2	145,189,102	9.24E-26	0.340	0.332				*ZEB2*	Body
cg23971170	1	165,326,269	7.07E-12	0.340	0.328				*LMX1A*	TSS1500
cg01678415	16	5,037,461	0.000189	0.340	0.366				*SEC14L5*	Body
cg03953626	6	30,652,396	8.61E-15	0.340	0.334				*KIAA1949*	Body
cg02774862	10	70,321,580	6.96E-14	0.340	0.325				*TET1*	5'UTR

cg00083833	16	50,583,273	1.62E-22	0.340	0.317				*NKD1*	Body
cg18605031	3	49,314,155	1.68E-05	0.340	0.271				*C3orf62*	1stExon
cg01875784	5	1,874,703	1.14E-13	0.340	0.354					
cg02930667	2	219,671,206	3.14E-08	0.340	0.334				*CYP27A1*	Body
cg01031032	3	112,051,894	0.006313	0.340	0.439				*CD200*	TSS200
cg20663376	15	31,372,997	6.06E-11	0.340	0.330				*TRPM1*	5'UTR
cg01560873	5	3,116,792	5.05E-12	0.340	0.364					
cg00458932	2	208,199,465	2.17E-15	0.340	0.333					
cg21207028	20	327,619	0.035742	0.340	0.053				*NRSN2*	1stExon
cg26540943	X	154,842,296	0.004916	0.340	0.317				*TMLHE*	5'UTR
cg13445796	11	20,181,725	0.020509	0.340	0.341				*DBX1*	1stExon
cg24323958	1	108,741,884	3.13E-05	0.340	0.218				*SLC25A24*	Body
cg12191938	10	88,730,946	0.008709	0.340	0.293				*AGAP11*	5'UTR
cg08186924	1	179,544,721	4.82E-10	0.340	0.259				*NPHS2*	Body
cg10631716	8	135,469,436	0.000186	0.340	0.217					
cg12783819	17	75,369,657	0.009827	0.340	0.307				*SEPT9*	5'UTR
cg02861178	20	10,492,319	1.11E-08	0.340	0.327				*C20orf94*	Body
cg19767131	14	92,979,698	1.19E-05	0.340	0.226				*RIN3*	TSS1500
cg18239431	8	25,898,220	2.79E-15	0.340	0.312				*EBF2*	Body
cg27408285	12	54,653,364	6.13E-26	0.340	0.361				*CBX5*	1stExon
cg25647300	8	49,670,412	1.82E-20	0.340	0.349					
cg27045447	22	28,198,218	0.004196	0.340	0.326				*MN1*	TSS1500
cg01439876	1	119,543,336	0.000989	0.340	0.360					
cg15826437	17	38,334,186	0.002079	0.340	0.298				*RAPGEFL1*	TSS200
cg19755813	17	650,208	9.23E-07	0.340	0.306				*GEMIN4*	Body
cg03632704	5	87,973,439	9.37E-06	0.340	0.316				*LOC645323*	Body
cg14151259	6	19,837,015	0.000174	0.340	0.191				*ID4*	TSS1500
cg11654024	1	85,230,119	1.31E-15	0.340	0.306					
cg14230696	2	176,968,920	4.76E-05	0.340	0.314					
cg19901801	1	114,697,032	6.79E-05	0.340	0.336				*SYT6*	TSS1500
cg19748424	5	6,583,601	7.42E-10	0.340	0.300				*LOC255167*	Body
cg05675514	6	22,026,015	2.32E-08	0.340	0.346				*FLJ22536*	Body
cg22549870	15	89,949,776	0.001273	0.340	0.380					
cg11523712	2	176,957,055	5.32E-08	0.340	0.305				*HOXD13*	TSS1500

cg15355146	1	40,368,366	1.79E-07	0.339	0.332				*MYCL1*	TSS1500
cg24653181	15	90,039,582	8.81E-10	0.339	0.350	9.01E-32	0.237		*RHCG*	Body
cg22609785	10	80,416,672	4.44E-11	0.339	0.312					
cg03448362	1	172,864,347	4.97E-05	0.339	0.302					
cg09626894	7	117,119,637	2.04E-10	0.339	0.335				*CFTR*	TSS1500
cg06132400	7	38,350,938	0.00113	0.339	0.110					
cg11649376	12	81,473,234	2.79E-23	0.339	0.315				*ACSS3*	Body
cg16917193	12	54,089,295	6.15E-05	0.339	0.369					
cg20433275	1	156,358,408	0.010127	0.339	0.183					
cg16266579	12	48,591,673	2.07E-05	0.339	0.362					
cg04097639	1	200,009,830	0.000175	0.339	0.391				*NR5A2*	Body
cg08440556	4	83,718,952	1.31E-05	0.339	0.229				*SCD5*	Body
cg27614319	17	35,079,061	3.62E-15	0.339	0.343					
cg24711649	12	81,472,118	3.15E-11	0.339	0.355				*ACSS3*	1stExon
cg10527300	13	110,874,326	8.9E-45	0.339	0.315				*COL4A1*	Body
cg01833675	2	225,266,876	0.005096	0.339	0.351				*FAM124B*	TSS200
cg22604316	1	46,958,801	2.42E-05	0.339	0.349					
cg25032745	10	15,481,695	3.87E-09	0.339	0.296					
cg05757757	10	80,008,637	5.16E-29	0.339	0.317					
cg19295451	15	102,010,161	5.01E-15	0.339	0.331				*PCSK6*	Body
cg26940178	1	76,082,888	2.08E-05	0.339	0.282					
cg09349604	4	41,880,747	0.000914	0.339	0.308					
cg08713365	20	327,623	0.026017	0.339	0.044	2.11E-09	0.159		*NRSN2*	1stExon
cg05364884	16	17,107,768	3.03E-10	0.339	0.361					
cg08275638	3	64,253,818	2.03E-31	0.339	0.349					
cg02517337	20	51,590,841	4.56E-08	0.339	0.388				*TSHZ2*	Body
cg27537164	6	168,079,127	0.00029	0.339	0.242					
cg00460049	1	66,998,806	3.11E-06	0.339	0.243				*SGIP1*	TSS1500
cg26869417	10	130,274,509	7.65E-07	0.339	0.262					
cg16264966	22	41,613,790	9.67E-24	0.339	0.340				*L3MBTL2*	Body
cg13532547	17	185,957	2.18E-16	0.339	0.301				*RPH3AL*	5'UTR
cg08190858	12	54,321,346	0.00136	0.339	0.350					
cg19870512	12	4,919,081	0.000484	0.339	0.142				*KCNA6*	5'UTR
cg04096619	5	9,547,595	5.18E-08	0.339	0.302				*SNORD123*	TSS1500

cg08949655	2	176,940,301	3.83E-05	0.339	0.319					
cg18206027	7	49,813,486	0.001656	0.339	0.467				*VWC2*	1stExon
cg22234930	15	72,519,739	1.18E-14	0.339	0.341				*PKM2*	5'UTR
cg21516478	5	150,400,328	0.003786	0.339	0.129	3.48E-11	0.190		*GPX3*	Body
cg22097731	15	63,334,436	0.017893	0.339	0.315				*TPM1*	TSS1500
cg09278708	X	101,905,837	3.18E-07	0.339	0.318				*GPRASP1*	TSS1500
cg22500428	12	4,381,882	0.025835	0.338	0.205				*CCND2*	TSS1500
cg20691205	13	36,919,674	0.017207	0.338	0.398				*SPG20*	5'UTR
cg07815287	2	9,345,967	1.02E-13	0.338	0.376				*ASAP2*	TSS1500
cg14025053	8	104,153,637	1.23E-05	0.338	0.357				*C8orf56*	TSS200
cg19380001	1	50,889,427	0.000626	0.338	0.306				*DMRTA2*	TSS1500
cg13303553	1	33,938,226	0.022926	0.338	0.336				*ZSCAN20*	TSS200
cg03554749	4	85,405,132	7.17E-06	0.338	0.306					
cg00301660	8	49,783,269	4.98E-20	0.338	0.331					
cg25440811	5	149,535,765	4.18E-06	0.338	0.358				*PDGFRB*	TSS1500
cg26994377	4	140,579,069	1.44E-07	0.338	0.338					
cg17852385	15	75,019,188	4.64E-13	0.338	0.294				*CYP1A1*	TSS1500
cg09745430	6	91,004,452	5.23E-13	0.338	0.289				*BACH2*	5'UTR
cg07483811	18	2,846,237	5.94E-12	0.338	0.271				*EMILIN2*	TSS1500
cg12591770	15	100,913,571	3.46E-11	0.338	0.329					
cg01316378	10	63,629,580	0.019017	0.338	0.267					
cg02407266	10	79,637,979	3.03E-05	0.338	0.358				*DLG5*	Body
cg01228355	4	47,839,876	0.000388	0.338	0.304				*CORIN*	Body
cg05140648	12	49,391,920	5.66E-05	0.338	0.440				*DDN*	Body
cg16751493	2	242,101,289	1.55E-08	0.338	0.342				*PPP1R7*	Body
cg17639394	6	29,943,480	2E-08	0.338	0.341				*HCG9*	Body
cg08077337	2	202,900,063	0.000201	0.338	0.369				*FZD7*	1stExon
cg02363950	1	156,676,793	4.81E-20	0.338	0.316				*CRABP2*	TSS1500
cg18328334	2	218,808,652	1.93E-27	0.338	0.321	6.45E-32	0.267		*TNS1*	1stExon
cg07875385	2	190,044,601	4.05E-06	0.338	0.314				*COL5A2*	1stExon
cg13398482	9	96,715,256	4.18E-09	0.338	0.389				*BARX1*	Body
cg02674352	11	61,275,826	5.48E-08	0.338	0.275				*LRRC10B*	TSS1500
cg18336453	6	43,082,296	1.63E-36	0.338	0.324				*PTK7*	Body
cg24549507	6	3,752,754	2.8E-11	0.338	0.307	1.77E-27	0.234		*C6orf145*	TSS1500

cg00926894	X	46,938,229	5.69E-11	0.338	0.337				*RGN*	5'UTR
cg00172597	1	223,538,379	0.002005	0.338	0.174				*SUSD4*	TSS1500
cg18454133	14	23,835,870	1.21E-20	0.338	0.350				*EFS*	TSS1500
cg04554652	10	103,880,979	4.16E-09	0.338	0.308				*LDB1*	TSS1500
cg02486253	17	55,122,828	6.28E-06	0.338	0.349				*RNF126P1*	TSS200
cg24383056	17	48,071,706	0.000759	0.338	0.258				*DLX3*	Body
cg19066273	17	37,366,365	0.000238	0.338	0.291					
cg22053945	17	46,651,360	8.66E-10	0.338	0.330				*HOXB3*	5'UTR
cg07560510	8	86,350,592	2.07E-09	0.338	0.357				*CA3*	TSS1500
cg04228709	6	155,537,930	3.12E-16	0.338	0.313				*TIAM2*	Body
cg07931391	13	100,608,263	6.38E-05	0.338	0.321					
cg24053514	1	146,556,859	1.68E-12	0.338	0.297					
cg05743885	7	27,196,365	5E-10	0.338	0.349				*HOXA7*	TSS200
cg12174804	8	18,244,861	1.59E-10	0.337	0.327					
cg23614791	12	81,471,754	0.002371	0.337	0.367				*ACSS3*	TSS200
cg11256607	1	58,716,306	7.49E-12	0.337	0.317				*DAB1*	TSS200
cg21935981	22	45,608,465	2.95E-07	0.337	0.295				*C22orf9*	Body
cg12408990	5	179,634,671	4.15E-22	0.337	0.326				*RASGEF1C*	5'UTR
cg16019142	6	30,418,900	1.59E-06	0.337	0.325					
cg07492240	4	121,994,123	1.35E-11	0.337	0.280				*C4orf31*	TSS1500
cg18065318	4	140,201,433	0.000431	0.337	0.287				*C4orf49*	5'UTR
cg25598159	12	111,127,533	5.56E-19	0.337	0.311				*HVCN1*	1stExon
cg27150417	20	44,462,035	7.1E-22	0.337	0.290				*SNX21*	TSS1500
cg16585234	1	25,944,802	3.9E-20	0.337	0.300				*MAN1C1*	1stExon
cg06866163	6	28,557,486	0.0008	0.337	0.275					
cg26263234	3	26,666,060	0.01973	0.337	0.322				*LRRC3B*	5'UTR
cg14337614	17	70,262,836	4.93E-19	0.337	0.323					
cg26205771	8	53,851,156	0.002348	0.337	0.407				*NPBWR1*	TSS1500
cg24859236	1	9,750,213	5.69E-22	0.337	0.333				*PIK3CD*	5'UTR
cg01385327	10	123,356,336	4.31E-06	0.337	0.321				*FGFR2*	5'UTR
cg19035232	20	51,589,985	0.003904	0.337	0.427				*TSHZ2*	Body
cg22332233	X	48,897,515	1.57E-10	0.337	0.375				*TFE3*	Body
cg02924943	3	134,125,326	3.73E-07	0.337	0.339					
cg08845123	1	62,784,332	2.52E-07	0.337	0.372				*KANK4*	5'UTR

cg01363714	14	103,655,773	9.17E-11	0.337	0.371					
cg19914919	2	161,992,878	4.3E-15	0.337	0.330				*TANK*	TSS1500
cg03662014	16	29,086,271	9.44E-06	0.337	0.361				*RRN3P2*	Body
cg14923157	19	17,138,063	0.007665	0.337	0.202				*CPAMD8*	TSS1500
cg18054172	12	54,424,902	1.43E-15	0.337	0.283				*HOXC4*	5'UTR
cg03485694	12	3,862,473	0.02188	0.337	0.193				*EFCAB4B*	TSS200
cg27527108	1	116,711,075	0.00625	0.337	0.325					
cg25649038	6	6,546,777	0.047828	0.337	0.171				*LOC285780*	Body
cg06959635	X	138,724,627	0.000377	0.337	0.329	7.18E-26	0.218		*MCF2*	1stExon
cg09792926	16	49,316,053	0.013043	0.337	0.415				*CBLN1*	TSS1500
cg12894325	11	67,418,113	1.48E-32	0.337	0.323				*ACY3*	5'UTR
cg02164046	3	187,388,148	0.000986	0.337	0.269	8.17E-19	0.238		*SST*	1stExon
cg05245533	16	795,877	0.000151	0.337	0.409					
cg03702236	5	9,546,755	1.22E-07	0.337	0.255	1.11E-20	0.263		*SEMA5A*	TSS1500
cg00914041	8	91,997,810	4.56E-05	0.337	0.336					
cg10043865	17	35,294,481	0.031401	0.337	0.359				*LHX1*	TSS200
cg22794704	2	162,273,437	0.017715	0.337	0.339				*TBR1*	1stExon
cg22084642	3	52,351,963	0.001945	0.337	0.347				*DNAH1*	5'UTR
cg07222505	5	74,161,231	0.000975	0.336	0.144				*FAM169A*	5'UTR
cg13568106	17	26,835,115	9.34E-05	0.336	0.321					
cg26330416	11	62,691,066	1.39E-07	0.336	0.321					
cg00596048	1	41,847,267	6.54E-05	0.336	0.150					
cg15910208	19	51,522,588	9.66E-05	0.336	0.343	1.68E-28	0.262		*KLK10*	5'UTR
cg02712145	7	30,722,433	1.02E-07	0.336	0.362				*CRHR2*	TSS1500
cg14288235	9	135,036,556	3.49E-23	0.336	0.317				*NTNG2*	TSS1500
cg11216064	5	50,263,586	0.012209	0.336	0.333					
cg25062539	8	79,718,482	3.57E-19	0.336	0.347				*IL7*	TSS1500
cg10498502	3	185,928,164	9.16E-26	0.336	0.314				*DGKG*	Body
cg06612088	5	11,589,016	9.06E-12	0.336	0.329				*CTNND2*	Body
cg08967106	17	35,014,412	0.026521	0.336	0.308					
cg12647497	1	110,210,265	4.94E-19	0.336	0.297				*GSTM2*	TSS1500
cg18881778	12	120,241,287	1.62E-17	0.336	0.331				*CIT*	Body
cg25792518	17	48,545,950	7.69E-17	0.336	0.324				*ACSF2*	Body
cg00354572	12	81,471,419	1.69E-07	0.336	0.300	8.25E-17	0.156		*ACSS3*	TSS1500

cg20408693	12	46,767,289	1.02E-07	0.336	0.328				*SLC38A2*	TSS1500
cg07793207	10	95,517,529	2.59E-07	0.336	0.332				*LGI1*	TSS200
cg02909711	16	67,360,805	0.012348	0.336	0.255				*LRRC36*	1stExon
cg02584756	1	43,533,796	6E-07	0.336	0.262					
cg08519905	12	6,308,774	9.98E-19	0.336	0.305	1.34E-17	0.198		*CD9*	TSS1500
cg10304922	12	133,065,924	0.004708	0.336	0.301				*FBRSL1*	TSS1500
cg18912160	10	3,281,074	5.23E-14	0.336	0.310					
cg16262614	3	133,464,971	7.62E-06	0.336	0.256				*TF*	TSS200
cg05106294	11	12,031,097	0.012768	0.336	0.315				*DKK3*	TSS1500
cg13990926	12	54,133,447	0.001089	0.336	0.254					
cg12290635	X	119,148,809	4.73E-11	0.336	0.296					
cg12506005	11	111,411,176	3.93E-06	0.336	0.314				*LAYN*	TSS200
cg03234186	19	58,220,657	2.16E-10	0.336	0.301				*ZNF154*	TSS200
cg11855325	7	30,029,717	0.011348	0.336	0.202				*SCRN1*	TSS1500
cg00155310	6	50,814,011	3.14E-10	0.336	0.381				*TFAP2B*	3'UTR
cg15659132	6	26,577,336	0.000178	0.336	0.377					
cg20899581	6	27,841,230	1.09E-10	0.336	0.320				*HIST1H4L*	1stExon
cg11646762	5	68,180,149	2.45E-21	0.336	0.328					
cg12829141	7	128,000,780	0.000591	0.336	0.375				*PRRT4*	5'UTR
cg01537995	14	85,996,352	8.09E-10	0.336	0.346				*FLRT2*	TSS200
cg10331424	16	70,696,450	4.86E-49	0.336	0.338				*MTSS1L*	3'UTR
cg18940843	6	30,418,773	1.24E-07	0.336	0.282					
cg02644510	7	121,946,608	3.72E-07	0.336	0.373					
cg24281267	6	35,479,648	0.006355	0.336	0.379				*TULP1*	Body
cg11724135	20	25,129,507	3.53E-09	0.336	0.286				*LOC284798*	TSS1500
cg07437373	3	134,125,814	7.33E-14	0.336	0.332					
cg12319534	4	54,959,841	9.95E-13	0.336	0.339					
cg22202692	2	6,121,939	0.000115	0.336	0.354				*LOC400940*	TSS200
cg09657963	11	124,790,875	0.02951	0.336	0.241				*HEPACAM*	3'UTR
cg17538572	10	94,834,763	0.000749	0.336	0.193				*CYP26A1*	Body
cg17935217	21	36,042,170	0.002315	0.335	0.290				*CLIC6*	1stExon
cg14610403	1	66,998,812	1E-05	0.335	0.238				*SGIP1*	TSS1500
cg00878038	7	151,107,807	2.55E-06	0.335	0.367				*WDR86*	TSS1500
cg05209996	11	76,033,054	0.000119	0.335	0.313					

cg06971418	17	41,995,241	1.82E-05	0.335	0.365				*C17orf88*	Body
cg15229747	19	56,097,663	0.00107	0.335	0.205					
cg24800655	12	133,065,941	0.001953	0.335	0.260				*FBRSL1*	TSS1500
cg01385959	2	190,119,363	1.01E-12	0.335	0.314					
cg19467964	17	80,806,135	0.000492	0.335	0.393				*TBCD*	Body
cg23204908	11	9,824,185	3.07E-18	0.335	0.329				*SBF2*	Body
cg11691298	2	26,396,787	5.3E-06	0.335	0.325				*FAM59B*	Body
cg27336068	10	1,336,103	3.93E-06	0.335	0.375				*ADARB2*	Body
cg10706039	X	119,134,323	0.000612	0.335	0.279					
cg21557231	X	138,725,077	1.61E-08	0.335	0.347	3.84E-20	0.223		*MCF2*	TSS200
cg10941747	20	55,841,898	0.030229	0.335	0.196				*BMP7*	TSS200
cg08482436	7	96,647,888	2.86E-08	0.335	0.328					
cg14825976	18	13,611,370	7.31E-06	0.335	0.310				*C18orf1*	TSS1500
cg16400631	1	91,300,215	0.03905	0.335	0.465					
cg19372602	1	156,116,207	1.92E-24	0.335	0.336					
cg26503877	11	128,391,429	1.26E-05	0.335	0.303				*ETS1*	Body
cg06782090	17	77,787,880	5.27E-07	0.335	0.286					
cg01446627	10	1,508,061	3.93E-08	0.335	0.256				*ADARB2*	Body
cg01338255	20	30,176,054	1.68E-13	0.335	0.286					
cg14121845	20	25,566,513	0.000123	0.335	0.221				*NINL*	TSS1500
cg13354872	15	65,550,813	1.96E-21	0.335	0.338				*PARP16*	3'UTR
cg02776659	21	47,717,406	1.61E-08	0.335	0.296				*C21orf57*	Body
cg14966613	22	25,423,700	1.18E-15	0.335	0.363				*KIAA1671*	TSS1500
cg27022933	11	67,418,106	4.61E-21	0.335	0.324				*ACY3*	5'UTR
cg06530338	2	19,561,612	5.21E-07	0.335	0.305					
cg07691152	12	103,355,123	0.000262	0.335	0.344					
cg01571001	3	128,215,433	2.27E-55	0.335	0.323					
cg16331929	4	185,937,863	1.62E-07	0.334	0.312					
cg13946341	9	128,799,328	1.44E-09	0.334	0.292					
cg05163709	Y	7,141,248	1.09E-07	0.334	0.246				*PRKY*	TSS1500
cg12857638	11	62,212,169	1.04E-09	0.334	0.344				*AHNAK*	Body
cg00397635	1	53,309,162	2.16E-05	0.334	0.313				*ZYG11A*	Body
cg18720905	2	96,990,858	9.32E-10	0.334	0.334				*ITPRIPL1*	TSS1500
cg04514123	16	2,041,225	4.86E-05	0.334	0.410				*SYNGR3*	Body

cg19646112	14	24,804,342	9.39E-09	0.334	0.341				*ADCY4*	TSS1500
cg09085216	1	204,329,081	3.87E-27	0.334	0.317				*PLEKHA6*	TSS200
cg00735923	7	117,119,963	2.01E-06	0.334	0.326				*CFTR*	TSS200
cg02518216	12	106,533,903	2.76E-24	0.334	0.306				*NUAK1*	TSS200
cg09079818	7	155,604,091	2.41E-06	0.334	0.201				*SHH*	Body
cg10227331	1	8,181,647	7.63E-24	0.334	0.323					
cg15552249	4	88,450,824	1.57E-21	0.334	0.337				*SPARCL1*	TSS200
cg18879590	1	236,306,640	0.000821	0.334	0.229				*GPR137B*	Body
cg05527869	17	35,294,476	0.018613	0.334	0.332				*LHX1*	TSS200
cg05622465	1	110,613,072	2.43E-07	0.334	0.329				*ALX3*	1stExon
cg11458217	X	134,125,022	0.000141	0.334	0.323				*LOC644538*	1stExon
cg12989128	20	42,875,933	3.8E-06	0.334	0.279				*GDAP1L1*	5'UTR
cg14709253	8	17,519,419	1.58E-08	0.334	0.290				*MTUS1*	Body
cg06460869	10	17,270,094	8.48E-05	0.334	0.329				*VIM*	TSS200
cg00555905	17	79,140,481	9.03E-06	0.334	0.250				*AATK*	TSS1500
cg05905176	15	51,385,916	0.006877	0.334	0.401				*TNFAIP8L3*	Body
cg23829024	6	26,018,074	0.001477	0.334	0.285				*HIST1H1A*	TSS200
cg08887400	6	30,652,376	9.89E-09	0.334	0.330				*KIAA1949*	Body
cg09706021	6	33,399,109	2.64E-32	0.334	0.330				*SYNGAP1*	Body
cg18813545	3	9,167,185	4.95E-24	0.334	0.326				*SRGAP3*	Body
cg11994674	17	47,547,767	5.06E-17	0.334	0.352					
cg05005609	11	57,250,204	3.64E-13	0.334	0.340					
cg16855845	19	12,991,665	0.003105	0.334	0.147				*DNASE2*	Body
cg22387369	15	85,360,691	9.54E-11	0.334	0.330				*ALPK3*	1stExon
cg17466795	20	9,497,330	8.21E-09	0.334	0.333				*C20orf103*	Body
cg06572465	5	140,800,929	1.34E-05	0.334	0.376				*PCDHGA4*	Body
cg08664487	6	56,818,409	3.96E-08	0.334	0.326				*BEND6*	TSS1500
cg24446178	12	100,750,702	1.05E-06	0.334	0.301				*SLC17A8*	TSS200
cg07481320	6	42,739,021	1.5E-32	0.334	0.309					
cg08963132	3	130,064,261	1.58E-05	0.334	0.231				*COL29A1*	TSS200
cg02374745	17	6,679,564	3.74E-05	0.334	0.252				*FBXO39*	1stExon
cg16909733	2	241,459,847	7.31E-13	0.334	0.307				*ANKMY1*	Body
cg10913846	14	37,133,714	8.07E-05	0.334	0.202				*PAX9*	Body
cg04188862	15	88,801,474	2.14E-08	0.334	0.330					

cg08192350	12	3,862,417	0.003002	0.334	0.238				*EFCAB4B*	TSS200
cg01442843	1	206,729,040	3E-26	0.334	0.302				*RASSF5*	Body
cg04306024	1	54,203,844	1.48E-05	0.334	0.316			Yes		
cg13575161	12	4,381,792	0.000598	0.334	0.273				*CCND2*	TSS1500
cg02991469	20	50,720,731	3.3E-07	0.334	0.347				*ZFP64*	Body
cg17199800	7	96,632,777	3.41E-05	0.334	0.366				*DLX6AS*	Body
cg17401179	5	400,517	2.56E-05	0.334	0.383				*AHRR*	Body
cg19736094	X	38,080,327	2.72E-06	0.334	0.286				*SRPX*	TSS200
cg11935248	6	4,942,483	1.04E-11	0.333	0.343				*CDYL*	Body
cg27505047	X	133,684,884	0.000628	0.333	0.285					
cg24812837	10	102,894,120	0.000517	0.333	0.314				*TLX1*	Body
cg27134365	14	24,458,004	3.9E-50	0.333	0.330				*DHRS4L2*	TSS200
cg19858576	5	191,806	5.23E-20	0.333	0.315				*LRRC14B*	1stExon
cg21141024	14	59,422,098	5.91E-06	0.333	0.267					
cg12111309	4	57,624,039	4.41E-10	0.333	0.304					
cg23842796	1	156,358,404	0.005307	0.333	0.196					
cg20734421	12	14,720,805	1.36E-08	0.333	0.252				*PLBD1*	TSS200
cg07374509	6	30,419,070	0.00029	0.333	0.365					
cg15536165	11	35,639,492	6.13E-06	0.333	0.253				*FJX1*	TSS1500
cg22280475	8	25,897,534	5.47E-09	0.333	0.310				*EBF2*	Body
cg25864762	7	1,850,388	3.43E-11	0.333	0.331					
cg11278506	3	72,787,955	3.09E-06	0.333	0.242					
cg02680669	8	40,181,105	3.02E-12	0.333	0.349					
cg00422638	10	25,466,027	8.49E-05	0.333	0.327				*LOC100128811*	TSS1500
cg01346013	17	74,836,383	2.35E-09	0.333	0.360					
cg05931423	5	115,697,214	8.78E-16	0.333	0.345					
cg26288577	14	91,580,429	0.009932	0.333	0.147				*C14orf159*	1stExon
cg11756518	6	107,809,929	2.24E-06	0.333	0.251				*SOBP*	TSS1500
cg16754467	6	10,426,588	1.18E-08	0.333	0.337					
cg01308827	19	36,132,627	5.44E-07	0.333	0.340				*ETV2*	TSS200
cg23267759	18	19,751,335	3.08E-07	0.333	0.321				*GATA6*	Body
cg00087244	7	74,042,487	9.13E-19	0.333	0.334					
cg23958524	3	9,289,787	3.84E-08	0.333	0.223				*SRGAP3*	Body
cg06866938	22	46,934,811	6.76E-10	0.333	0.324					

cg22370379	6	32,223,090	3.16E-05	0.333	0.340					
cg25806307	19	39,466,182	0.008444	0.333	0.368				*FBXO17*	5'UTR
cg16201038	13	28,544,760	3.26E-08	0.333	0.297				*CDX2*	TSS1500
cg09453076	2	235,406,275	1.17E-31	0.333	0.325				*ARL4C*	TSS1500
cg01777397	2	29,337,946	9.47E-08	0.333	0.264	1.19E-32	0.278		*CLIP4*	TSS1500
cg12147198	4	47,838,169	3.54E-16	0.333	0.292				*CORIN*	Body
cg19308375	2	228,324,937	2.74E-20	0.333	0.332					
cg10382221	17	46,114,672	8.85E-07	0.333	0.323				*MIR152*	TSS200
cg21056706	7	139,929,714	0.00035	0.333	0.279					
cg02806452	2	233,499,070	1.1E-05	0.333	0.307				*EFHD1*	Body
cg14196225	4	57,522,762	0.000313	0.333	0.311				*HOPX*	5'UTR
cg10143811	12	16,757,985	2.82E-05	0.333	0.272				*LMO3*	5'UTR
cg04543008	11	71,955,332	0.001084	0.333	0.321				*PHOX2A*	TSS200
cg15867125	14	77,624,538	4.13E-09	0.333	0.354					
cg09662034	20	55,206,467	0.001743	0.333	0.226				*TFAP2C*	Body
cg26163458	13	95,620,976	1.46E-05	0.333	0.295					
cg08757924	4	85,306,687	4.39E-10	0.333	0.319					
cg11961138	17	38,599,366	2.62E-26	0.333	0.316				*IGFBP4*	TSS1500
cg13708158	12	116,037,223	1.39E-06	0.333	0.275					
cg04669815	10	101,296,619	4.19E-06	0.333	0.355					
cg15688619	3	44,626,860	4.27E-15	0.333	0.291				*ZNF660*	5'UTR
cg08268017	8	94,712,118	6.8E-09	0.332	0.288				*FAM92A1*	TSS1500
cg24389730	17	46,114,615	1.86E-06	0.332	0.336				*MIR152*	TSS200
cg14428146	8	23,563,925	3.21E-05	0.332	0.325				*NKX2-6*	TSS200
cg14350382	9	133,320,728	0.003607	0.332	0.219				*ASS1*	5'UTR
cg25410739	1	223,743,086	4.05E-11	0.332	0.311				*CAPN8*	Body
cg24137543	5	140,893,634	2.2E-11	0.332	0.327					
cg18245160	7	27,244,370	0.010486	0.332	0.424					
cg14348741	9	139,880,223	6.81E-06	0.332	0.347					
cg15948536	4	169,770,092	3.31E-12	0.332	0.319				*PALLD*	5'UTR
cg08637618	7	27,279,329	9.29E-09	0.332	0.291					
cg12117658	17	56,406,294	3.21E-05	0.332	0.335				*BZRAP1*	TSS200
cg18456523	5	54,516,805	0.000226	0.332	0.335					
cg11331445	12	47,219,877	7.53E-08	0.332	0.311				*SLC38A4*	TSS200

cg06476685	2	241,459,638	6.27E-15	0.332	0.312				*ANKMY1*	Body
cg27049766	19	58,220,516	9.09E-06	0.332	0.277				*ZNF154*	5'UTR
cg14397690	15	60,688,948	2.15E-22	0.332	0.305				*ANXA2*	5'UTR
cg17771031	20	44,875,334	0.001688	0.332	0.198				*CDH22*	Body
cg17344906	19	13,202,507	2.68E-28	0.332	0.349				*NFIX*	3'UTR
cg00063174	2	228,736,253	0.003145	0.332	0.425				*WDR69*	TSS200
cg03989617	16	57,662,541	1.69E-19	0.332	0.300				*GPR56*	5'UTR
cg06815950	12	111,619,455	1.27E-12	0.332	0.315				*CUX2*	Body
cg26707709	2	241,975,756	9.62E-21	0.332	0.306				*SNED1*	Body
cg23536629	10	101,279,944	1.31E-07	0.332	0.250					
cg20852226	11	76,859,010	0.002163	0.332	0.278				*MYO7A*	Body
cg09183316	12	58,238,232	1.36E-09	0.332	0.306				*CTDSP2*	Body
cg18713646	6	29,974,952	5.96E-11	0.332	0.324				*HLA-J*	Body
cg01283289	12	81,472,177	9.78E-12	0.332	0.319	3.69E-24	0.214		*ACSS3*	1stExon
cg02096396	2	26,395,556	0.002024	0.332	0.172				*FAM59B*	TSS1500
cg15880846	6	28,557,424	0.001335	0.332	0.297					
cg11479165	15	65,068,594	1.55E-18	0.332	0.327				*RBPMS2*	TSS1500
cg11673244	22	22,007,100	4.1E-25	0.332	0.321				*MIR130B*	TSS1500
cg24468070	19	54,976,501	7.54E-09	0.332	0.352				*CDC42EP5*	Body
cg21695661	1	63,796,263	1.26E-08	0.332	0.284					
cg00628788	18	33,529,997	8.01E-08	0.332	0.246					
cg22222413	3	42,066,796	2.29E-16	0.332	0.306					
cg08980711	10	98,945,025	1.94E-12	0.332	0.309				*SLIT1*	Body
cg07485916	15	90,039,805	1.57E-12	0.332	0.332				*RHCG*	TSS200
cg15130102	1	213,090,116	6.3E-10	0.332	0.332					
cg00991875	6	161,100,376	0.001671	0.332	0.256					
cg02114084	14	75,894,209	1.07E-09	0.332	0.245				*JDP2*	TSS1500
cg06856720	20	19,870,217	0.011946	0.332	0.247				*RIN2*	1stExon
cg17915961	10	103,881,480	3.07E-10	0.332	0.307				*LDB1*	TSS1500
cg27358154	10	72,453,975	0.000197	0.332	0.243				*ADAMTS14*	Body
cg15696662	1	46,669,221	0.016662	0.332	0.499				*C1orf190*	1stExon
cg23627335	6	17,102,653	0.0072	0.332	0.116					
cg06200244	2	12,862,337	6.35E-20	0.332	0.333				*TRIB2*	Body
cg26262482	1	64,937,345	0.000268	0.332	0.310				*CACHD1*	Body

cg13832372	9	124,983,482	5.46E-09	0.332	0.329				*LHX6*	Body
cg23831143	8	22,960,427	0.001213	0.332	0.257				*TNFRSF10C*	TSS200
cg17763354	10	86,016,954	2.31E-05	0.332	0.264				*RGR*	Body
cg22694191	19	19,373,575	1.18E-09	0.332	0.332				*HAPLN4*	5'UTR
cg21038156	7	158,936,739	0.000102	0.332	0.314				*VIPR2*	Body
cg13670057	9	133,721,945	2.94E-19	0.331	0.319				*ABL1*	Body
cg09031823	4	83,674,996	1.29E-28	0.331	0.321				*MIR575*	TSS1500
cg16651537	19	51,226,536	0.00035	0.331	0.450				*CLEC11A*	TSS200
cg03640756	5	140,864,593	9.55E-05	0.331	0.316				*PCDHGA4*	Body
cg16792842	8	25,899,459	6.34E-10	0.331	0.304				*EBF2*	Body
cg03536375	18	18,821,328	8.41E-07	0.331	0.291				*GREB1L*	TSS1500
cg09661010	10	73,848,153	0.001529	0.331	0.129				*SPOCK2*	5'UTR
cg18016254	3	122,296,505	1.36E-06	0.331	0.328				*PARP15*	5'UTR
cg09509179	10	91,598,023	3.39E-10	0.331	0.244					
cg15935965	1	59,550,124	2.4E-09	0.331	0.315					
cg09736669	5	1,851,828	1.08E-11	0.331	0.311					
cg04021697	1	3,567,303	0.004649	0.331	0.332				*WDR8*	TSS1500
cg11574975	1	112,058,304	7.56E-07	0.331	0.349				*ADORA3*	Body
cg20198108	10	17,270,091	9.53E-07	0.331	0.317				*VIM*	TSS200
cg22803868	17	27,045,164	1.99E-18	0.331	0.328				*RAB34*	Body
cg07143418	8	23,567,548	1.45E-07	0.331	0.312					
cg26008908	12	120,799,332	1.9E-20	0.331	0.318				*MSI1*	Body
cg26995992	16	87,417,792	0.022675	0.331	0.265				*FBXO31*	TSS1500
cg11521404	13	20,735,532	0.032018	0.331	0.373				*GJA3*	TSS1500
cg15339720	2	20,425,649	1.16E-29	0.331	0.299				*SDC1*	TSS1500
cg07239716	12	89,744,488	2.06E-13	0.331	0.316				*DUSP6*	Body
cg18090004	7	1,283,896	2.92E-09	0.331	0.294					
cg19177125	18	19,752,414	1.87E-19	0.331	0.292				*GATA6*	Body
cg04650654	6	28,505,429	0.000002	0.331	0.314					
cg16686733	20	25,566,563	0.001586	0.331	0.206				*NINL*	TSS1500
cg02035751	1	206,729,099	1.01E-27	0.331	0.298				*RASSF5*	Body
cg19164987	12	54,413,000	2.79E-16	0.331	0.273				*HOXC4*	5'UTR
cg05461734	10	122,705,955	7.84E-26	0.331	0.295					
cg21703322	11	111,411,076	8.94E-20	0.331	0.295				*LAYN*	TSS200

cg12248807	6	25,156,302	1.49E-15	0.331	0.341						
cg20595752	6	35,458,416	7.15E-24	0.331	0.327					*TEAD3*	5'UTR
cg14346243	4	90,757,452	5.25E-07	0.331	0.268					*SNCA*	5'UTR
cg17504394	17	70,339,295	5.23E-19	0.331	0.331						
cg03431846	14	24,641,183	4.74E-12	0.331	0.319					*REC8*	TSS200
cg15173134	10	119,000,638	0.004913	0.331	0.296					*SLC18A2*	TSS200
cg09040078	16	23,291,476	6.12E-10	0.331	0.346						
cg20119798	7	94,954,144	1.79E-11	0.331	0.316					*PON1*	TSS1500
cg00903676	6	26,172,091	7.15E-05	0.331	0.398						
cg08858649	6	30,139,903	0.000228	0.331	0.351					*TRIM15*	Body
cg13720581	6	32,077,017	1.81E-05	0.331	0.270					*TNXB*	5'UTR
cg00626702	4	1,647,016	1.46E-08	0.331	0.326					*FAM53A*	Body
cg02602541	5	7,850,438	0.009414	0.331	0.458					*C5orf49*	Body
cg14077898	12	100,750,695	3.77E-07	0.331	0.280					*SLC17A8*	TSS200
cg04249706	10	11,206,772	0.017628	0.331	0.225					*CUGBP2*	Body
cg26168907	6	32,977,223	3.23E-06	0.331	0.310					*HLA-DOA*	Body
cg08949408	10	16,562,470	0.000619	0.331	0.231					*C1QL3*	Body
cg10190863	5	139,077,047	0.000599	0.331	0.223						
cg22413056	6	30,650,202	1.89E-09	0.331	0.355					*KIAA1949*	Body
cg25278941	6	139,795,527	1.05E-21	0.331	0.320						
cg04812556	11	9,112,715	0.000644	0.331	0.165					*SCUBE2*	Body
cg18038544	3	14,576,099	9.89E-14	0.331	0.325					*GRIP2*	Body
cg00995986	2	66,665,428	6.78E-05	0.331	0.380					*MEIS1*	Body
cg01691182	7	55,639,118	8.36E-12	0.331	0.263					*VOPP1*	Body
cg19225602	11	75,269,316	5.92E-20	0.331	0.327						
cg08043592	19	56,687,388	1.74E-07	0.331	0.369					*GALP*	1stExon
cg23097402	1	50,883,394	9.19E-06	0.331	0.360					*DMRTA2*	3'UTR
cg26294995	5	36,067,091	4.78E-07	0.331	0.318					*UGT3A2*	TSS200
cg08943714	6	139,470,282	1.58E-33	0.331	0.324					*HECA*	Body
cg01646639	14	70,654,290	1.58E-06	0.330	0.274					*SLC8A3*	5'UTR
cg04621020	5	101,632,341	0.000645	0.330	0.284					*SLCO4C1*	TSS200
cg09793121	21	34,398,263	2.88E-07	0.330	0.336					*OLIG2*	1stExon
cg21620778	2	175,548,020	0.022108	0.330	0.220					*WIPF1*	TSS1500
cg03281981	12	115,123,757	6.46E-12	0.330	0.277						

cg25433595	X	100,183,764	1.48E-05	0.330	0.162				*XKRX*	5'UTR
cg26018901	4	47,840,011	3.68E-07	0.330	0.287	1.72E-10	0.144		*CORIN*	5'UTR
cg00795341	5	79,330,929	0.002948	0.330	0.270				*THBS4*	TSS200
cg15836505	19	20,349,252	0.020153	0.330	0.170					
cg05317090	16	54,969,706	4.81E-05	0.330	0.409					
cg20810222	2	109,947,649	8.97E-12	0.330	0.286				*SH3RF3*	Body
cg05590619	3	42,056,814	9.59E-13	0.330	0.262					
cg14262740	14	20,612,220	1.16E-17	0.330	0.296				*OR4N5*	1stExon
cg20647610	2	228,736,258	0.005518	0.330	0.428				*WDR69*	TSS200
cg00842687	3	73,816,009	3.64E-23	0.330	0.335					
cg15020645	5	112,073,769	0.000775	0.330	0.196	5.44E-20	0.237		*APC*	5'UTR
cg10968631	14	69,986,021	1.1E-23	0.330	0.320			Yes	*FLJ44817*	Body
cg13617795	7	105,137,683	6.69E-17	0.330	0.332				*PUS7*	Body
cg14530275	9	16,705,212	2.26E-19	0.330	0.314				*BNC2*	Body
cg08726522	11	8,739,587	1.79E-06	0.330	0.373				*ST5*	Body
cg25942990	2	177,014,996	7.71E-07	0.330	0.278				*HOXD4*	TSS1500
cg03040165	2	131,185,700	0.00337	0.330	0.318					
cg17210933	5	3,428,696	2.73E-06	0.330	0.332					
cg24542714	1	46,669,345	2.76E-10	0.330	0.350				*POMGNT1*	Body
cg19841506	17	4,642,056	1.53E-08	0.330	0.191	4.7E-17	0.197		*ZMYND15*	TSS1500
cg01047555	6	127,835,990	1.25E-18	0.330	0.328				*C6orf174*	Body
cg12606911	2	87,036,626	3.72E-06	0.330	0.335				*CD8A*	TSS1500
cg19092981	22	19,751,654	4.39E-16	0.330	0.304				*TBX1*	Body
cg08275602	12	29,935,873	0.001042	0.330	0.206				*TMTC1*	5'UTR
cg07779387	2	95,873,465	0.000807	0.330	0.228					
cg16515523	1	171,811,529	0.000143	0.330	0.203				*DNM3*	Body
cg24859937	8	38,411,708	0.016887	0.330	0.259					
cg10784386	5	79,330,943	8.91E-05	0.330	0.261				*THBS4*	TSS200
cg07367232	17	7,826,266	0.007575	0.330	0.321				*KCNAB3*	3'UTR
cg10788618	17	46,622,022	0.000369	0.330	0.313				*HOXB2*	1stExon
cg11573219	1	32,083,031	8.4E-37	0.330	0.317				*HCRTR1*	TSS1500
cg00523604	6	32,223,341	7.49E-09	0.330	0.330					
cg17074762	X	68,052,208	6.75E-27	0.330	0.315				*EFNB1*	Body
cg20177650	3	181,445,156	0.001545	0.330	0.336				*SOX2OT*	Body

cg07121856	7	95,025,736	2.42E-09	0.330	0.285				*PON3*	TSS200
cg00814733	12	4,382,051	7.86E-12	0.330	0.296				*CCND2*	TSS1500
cg00809111	X	130,192,134	1.55E-05	0.329	0.214				*FLJ30058*	TSS200
cg11205552	3	112,995,690	1.1E-09	0.329	0.277				*BOC*	Body
cg03744043	18	45,662,275	2.29E-10	0.329	0.308					
cg03429644	2	9,252,446	1.98E-07	0.329	0.346					
cg08832906	7	139,208,852	0.000144	0.329	0.313				*CLEC2L*	1stExon
cg16375290	5	177,412,718	2.71E-07	0.329	0.345					
cg20646556	7	1,022,806	2.57E-26	0.329	0.318				*CYP2W1*	TSS200
cg02927346	17	34,059,260	4.37E-12	0.329	0.322	1.05E-22	0.243		*RASL10B*	5'UTR
cg09072216	13	36,919,344	0.000266	0.329	0.427				*SPG20*	5'UTR
cg05031016	14	24,804,153	1.5E-05	0.329	0.347				*ADCY4*	TSS1500
cg03195164	3	128,998,642	0.020048	0.329	0.085				*C3orf37*	Body
cg14141458	2	46,770,817	1.01E-06	0.329	0.234				*RHOQ*	Body
cg06638529	1	44,704,073	6.24E-29	0.329	0.307				*ERI3*	Body
cg22592142	15	45,409,170	4.29E-11	0.329	0.319				*DUOXA2*	Body
cg27379109	5	74,907,287	3.04E-15	0.329	0.286					
cg00201468	9	88,137,984	0.004344	0.329	0.277					
cg09774842	20	3,052,483	1.09E-11	0.329	0.348				*OXT*	Body
cg00866556	1	29,449,356	2.96E-05	0.329	0.364				*TMEM200B*	5'UTR
cg04837025	1	236,228,744	0.016035	0.329	0.134				*NID1*	TSS1500
cg18652367	5	180,017,689	0.00038	0.329	0.292				*SCGB3A1*	Body
cg23552977	14	21,492,433	1.27E-07	0.329	0.366				*NDRG2*	5'UTR
cg22287064	17	73,584,070	0.000216	0.329	0.285				*MYO15B*	TSS200
cg12812583	8	23,567,310	0.000151	0.329	0.294					
cg09505788	7	117,854,511	0.000122	0.329	0.292					
cg03529003	1	11,994,281	1.47E-06	0.329	0.284				*PLOD1*	TSS1500
cg22692511	11	117,069,966	1.15E-11	0.329	0.321				*TAGLN*	TSS200
cg24377179	3	55,523,815	0.001602	0.329	0.219					
cg24451123	17	6,559,656	0.009528	0.329	0.315					
cg17627629	5	179,517,140	0.000783	0.329	0.214					
cg27481555	7	102,553,369	5.96E-10	0.329	0.316				*LRRC17*	5'UTR
cg02582940	2	97,308,417	1.08E-09	0.329	0.326				*FER1L5*	TSS200
cg07152605	1	164,793,520	2.6E-27	0.329	0.320				*PBX1*	Body

cg12702981	3	119,528,656	2.05E-07	0.329	0.322				*NR1I2*	Body
cg27494647	7	150,038,898	2.83E-07	0.329	0.335				*RARRES2*	TSS200
cg17194270	22	39,759,992	1.11E-17	0.329	0.323				*SYNGR1*	Body
cg05603881	4	42,401,149	0.000212	0.329	0.320				*SHISA3*	Body
cg22289434	20	34,188,940	5.44E-12	0.329	0.359				*FER1L4*	Body
cg24122124	19	5,339,092	4.21E-06	0.329	0.346				*PTPRS*	5'UTR
cg00925616	1	32,410,211	7.1E-08	0.329	0.326					
cg18320766	19	55,598,782	0.000111	0.329	0.273				*EPS8L1*	Body
cg01532168	8	140,715,557	8.17E-06	0.329	0.367				*KCNK9*	TSS1500
cg15124757	2	17,699,904	0.007071	0.329	0.379				*RAD51AP2*	TSS200
cg17120578	8	38,329,836	3.28E-14	0.329	0.278					
cg18427091	17	30,115,417	4.23E-18	0.329	0.321					
cg05907835	16	31,214,346	1.86E-15	0.329	0.269				*PYCARD*	TSS200
cg09165441	10	103,044,194	4.69E-05	0.329	0.282					
cg04226536	11	111,411,071	3.73E-19	0.329	0.296				*LAYN*	TSS200
cg10720723	7	157,476,443	3.76E-14	0.329	0.312				*PTPRN2*	Body
cg20512711	11	31,841,524	8.79E-05	0.329	0.370					
cg18862767	1	40,137,970	3.34E-08	0.329	0.293				*NT5C1A*	TSS1500
cg06222062	1	20,396,690	7.02E-13	0.328	0.306				*PLA2G5*	TSS200
cg18959207	10	18,549,686	9.66E-20	0.328	0.323				*CACNB2*	1stExon
cg19349217	6	29,894,903	0.007195	0.328	0.296				*HCG4P6*	TSS1500
cg14870792	12	2,163,532	0.000209	0.328	0.237				*CACNA1C*	Body
cg07066369	12	4,384,888	0.000254	0.328	0.362				*CCND2*	Body
cg04752680	19	14,114,100	7.16E-25	0.328	0.305				*RFX1*	5'UTR
cg13347910	6	10,555,853	6.53E-08	0.328	0.307				*GCNT2*	TSS200
cg24254196	15	101,719,523	1.72E-12	0.328	0.264				*CHSY1*	Body
cg12024969	19	6,768,022	0.000733	0.328	0.227				*SH2D3A*	TSS1500
cg12472483	7	157,996,945	4.02E-43	0.328	0.314				*PTPRN2*	Body
cg22970435	6	44,310,228	6.19E-16	0.328	0.322	7.77E-25	0.226		*SPATS1*	TSS200
cg01678084	15	67,022,087	7.07E-25	0.328	0.323				*SMAD6*	Body
cg18628371	14	24,641,189	7.1E-12	0.328	0.324				*REC8*	TSS200
cg02466815	2	177,054,573	4.19E-08	0.328	0.310				*HOXD1*	Body
cg10071643	6	137,814,356	6.44E-10	0.328	0.328				*OLIG3*	1stExon
cg12602409	2	177,039,568	0.009908	0.328	0.357					

cg24832985	5	81,148,310	1.58E-11	0.328	0.290					
cg23092823	1	53,528,612	6.23E-15	0.328	0.306	7.23E-23	0.263		*PODN*	Body
cg20276377	3	99,595,026	0.002347	0.328	0.370				*C3orf26*	Body
cg08692423	21	45,148,864	0.000752	0.328	0.203				*PDXK*	Body
cg07529534	8	41,511,314	0.040363	0.328	0.327				*ANK1*	3'UTR
cg10981580	1	236,306,767	0.008032	0.328	0.175				*GPR137B*	Body
cg20254354	12	99,287,558	0.00043	0.328	0.240				*ANKS1B*	Body
cg06581175	7	96,632,635	0.000155	0.328	0.309				*DLX6AS*	Body
cg08725455	X	53,077,301	1.46E-10	0.328	0.303				*GPR173*	TSS1500
cg21316772	9	33,167,933	1.35E-22	0.328	0.309				*B4GALT1*	TSS1500
cg13908315	15	29,033,896	1E-12	0.328	0.326					
cg22892110	8	144,798,631	4.48E-15	0.328	0.342	1.29E-29	0.325		*MAPK15*	Body
cg13654836	17	55,365,912	3.87E-23	0.328	0.300				*MSI2*	Body
cg21853510	17	80,285,525	1.96E-09	0.328	0.368				*SECTM1*	5'UTR
cg07573727	14	74,706,196	8.13E-06	0.328	0.305				*VSX2*	5'UTR
cg12268565	X	133,685,325	1.44E-08	0.328	0.319					
cg15885440	12	29,302,714	0.000273	0.328	0.208					
cg21428710	12	47,219,797	6.45E-13	0.328	0.286				*SLC38A4*	TSS200
cg05258757	3	125,690,986	4.6E-05	0.328	0.272				*ROPN1B*	Body
cg14824386	8	41,167,923	0.014576	0.328	0.223				*SFRP1*	TSS1500
cg03452174	17	27,045,113	4.54E-10	0.328	0.370				*RAB34*	Body
cg14706852	2	127,976,351	3.69E-10	0.328	0.311					
cg03731268	1	62,660,861	1.3E-05	0.328	0.370				*L1TD1*	5'UTR
cg00365499	7	104,909,430	0.000149	0.328	0.302				*SRPK2*	5'UTR
cg07223180	13	20,989,142	7.16E-17	0.328	0.307				*CRYL1*	Body
cg18139020	1	33,938,191	0.027092	0.328	0.320				*ZSCAN20*	TSS200
cg23161999	2	162,283,189	0.021722	0.328	0.304					
cg14088921	8	98,944,936	1.43E-05	0.328	0.311				*MATN2*	Body
cg12074780	12	53,719,569	0.000296	0.328	0.384					
cg05183931	11	17,717,588	0.010927	0.328	0.347					
cg21963854	15	69,366,348	0.000238	0.328	0.160				*MIR548H4*	Body
cg01121022	14	104,338,788	3.93E-16	0.328	0.303					
cg12518442	20	55,841,906	0.039162	0.328	0.197				*BMP7*	TSS200
cg10511249	1	43,533,955	0.007658	0.328	0.109					

cg19028462	13	113,677,628	7.21E-21	0.328	0.310				*MCF2L*	Body
cg06899970	8	42,234,669	5.03E-10	0.327	0.308			Yes	*DKK4*	5'UTR
cg21001198	20	6,033,205	0.011793	0.327	0.110				*LRRN4*	Body
cg19350360	15	100,913,948	0.000399	0.327	0.290					
cg03423123	X	134,125,181	1.35E-05	0.327	0.334				*LOC644538*	1stExon
cg02595575	19	19,221,316	3.12E-05	0.327	0.336				*SLC25A42*	Body
cg13793145	6	44,187,109	0.008289	0.327	0.235				*SLC29A1*	TSS200
cg10651121	12	109,549,097	3E-08	0.327	0.221					
cg08266366	12	50,354,998	3.46E-13	0.327	0.288				*AQP5*	TSS1500
cg05539515	1	62,784,269	2.12E-10	0.327	0.332				*KANK4*	5'UTR
cg27267227	16	29,888,023	8.61E-07	0.327	0.331				*SEZ6L2*	Body
cg22735222	10	101,295,724	0.004895	0.327	0.304				*NKX2-3*	3'UTR
cg16430510	11	35,639,686	0.006818	0.327	0.243				*FJX1*	TSS200
cg06811478	8	10,872,626	1.47E-05	0.327	0.318				*XKR6*	Body
cg04365224	3	72,788,183	1.77E-08	0.327	0.268					
cg07696516	4	41,614,958	4.15E-10	0.327	0.247			Yes	*LIMCH1*	1stExon
cg08950287	7	99,725,807	2.16E-12	0.327	0.261				*MBLAC1*	Body
cg24096855	19	38,700,334	4.7E-12	0.327	0.360					
cg08310866	17	21,281,163	2.28E-12	0.327	0.351				*KCNJ12*	5'UTR
cg00112042	1	146,555,865	1.47E-06	0.327	0.321					
cg11241541	8	38,586,066	5.6E-05	0.327	0.284				*TACC1*	1stExon
cg13073773	14	95,234,658	1.5E-05	0.327	0.370				*GSC*	3'UTR
cg18894200	7	157,484,250	5.24E-28	0.327	0.312				*PTPRN2*	Body
cg24827726	3	14,581,937	8.43E-12	0.327	0.361				*GRIP2*	Body
cg18285788	7	157,475,692	6.47E-10	0.327	0.313				*PTPRN2*	Body
cg18877506	1	13,910,555	5.55E-07	0.327	0.375	1.24E-18	0.182		*PDPN*	TSS1500
cg25338134	3	72,940,510	5.64E-21	0.327	0.316				*GXYLT2*	Body
cg19305681	10	94,821,657	4.93E-10	0.327	0.272				*CYP26C1*	Body
cg24470119	19	8,432,416	0.004598	0.327	0.314				*ANGPTL4*	Body
cg01401641	19	31,839,171	2.04E-19	0.327	0.321				*TSHZ3*	Body
cg14405137	12	50,219,283	1.59E-18	0.327	0.325				*NCKAP5L*	5'UTR
cg19591881	1	208,084,640	2.55E-09	0.327	0.306	4.97E-21	0.243		*CD34*	5'UTR
cg01964152	6	30,095,546	5.08E-06	0.327	0.328					
cg24351410	1	58,716,033	1.25E-06	0.327	0.278				*DAB1*	5'UTR

cg13699887	6	16,320,488	0.005439	0.327	0.396				*ATXN1*	Body
cg01596834	3	183,993,711	1.09E-06	0.327	0.229				*ECE2*	TSS200
cg02874942	19	11,689,649	1.7E-05	0.327	0.320				*ACP5*	TSS1500
cg14225678	7	54,612,325	0.007114	0.327	0.346				*VSTM2A*	Body
cg07968031	3	129,346,101	0.000375	0.327	0.341					
cg20945738	13	108,922,032	1.57E-06	0.327	0.289				*TNFSF13B*	5'UTR
cg17820365	8	97,157,856	1.65E-05	0.327	0.327				*GDF6*	Body
cg14753355	19	16,999,668	0.000376	0.327	0.283				*F2RL3*	TSS200
cg17163760	7	29,186,267	0.000704	0.327	0.282				*CPVL*	TSS200
cg05243438	1	113,265,611	4.25E-23	0.327	0.316				*FAM19A3*	Body
cg27507295	15	90,039,794	5.27E-10	0.327	0.325				*RHCG*	1stExon
cg08148891	11	13,030,309	2E-08	0.327	0.270				*RASSF10*	TSS1500
cg06126421	6	30,720,080	1.95E-15	0.327	0.263					
cg06557644	7	30,510,463	1.18E-06	0.326	0.259				*NOD1*	5'UTR
cg16264526	4	148,401,273	7.1E-15	0.326	0.330				*EDNRA*	TSS1500
cg22837714	2	198,120,525	1.73E-09	0.326	0.318					
cg11859584	19	3,361,374	7.46E-07	0.326	0.283				*NFIC*	Body
cg00301239	8	103,801,487	2.04E-13	0.326	0.323					
cg08690876	22	43,040,721	9.02E-13	0.326	0.327				*CYB5R3*	5'UTR
cg08815081	10	28,035,731	6.73E-14	0.326	0.337				*MKX*	TSS1500
cg18862481	3	129,693,370	8.56E-08	0.326	0.326				*TRH*	5'UTR
cg27146050	19	46,801,557	1.15E-12	0.326	0.355				*HIF3A*	Body
cg26251270	1	53,067,832	8.88E-05	0.326	0.175				*GPX7*	TSS1500
cg26282655	20	2,505,978	8.92E-06	0.326	0.260					
cg21754400	12	81,471,649	2.14E-12	0.326	0.284				*ACSS3*	TSS200
cg25025646	7	150,711,449	1.13E-16	0.326	0.331				*NOS3*	3'UTR
cg08260959	6	26,240,920	2.93E-05	0.326	0.314	1.38E-11	0.176		*HIST1H4F*	1stExon
cg21905630	4	54,965,904	1.15E-05	0.326	0.359	1.67E-15	0.206		*GSX2*	TSS1500
cg07072475	14	90,850,234	6.49E-05	0.326	0.347					
cg13180315	2	177,024,294	0.03004	0.326	0.381					
cg14644001	6	32,116,653	0.020981	0.326	0.330				*PRRT1*	3'UTR
cg05816868	1	26,686,860	1.89E-06	0.326	0.370					
cg08290850	6	44,238,228	0.000841	0.326	0.203				*TMEM151B*	TSS1500
cg12399536	11	12,884,884	3.87E-16	0.326	0.306				*TEAD1*	Body

cg05985303	2	219,723,705	1.27E-20	0.326	0.309				*WNT6*	TSS1500
cg08390172	2	192,711,905	9.57E-05	0.326	0.369				*SDPR*	5'UTR
cg27586487	10	79,687,289	0.000116	0.326	0.243				*LOC100128292*	Body
cg06117341	16	3,062,653	6.34E-16	0.326	0.294				*CLDN9*	1stExon
cg11337945	5	2,755,158	3.39E-07	0.326	0.354				*C5orf38*	3'UTR
cg18097189	3	154,147,955	2.26E-10	0.326	0.345				*GPR149*	TSS1500
cg00347757	13	20,768,560	4.03E-08	0.326	0.319				*GJB2*	TSS1500
cg06962177	1	63,785,946	0.014795	0.326	0.417					
cg04069374	X	119,445,076	0.000251	0.326	0.271				*FAM70A*	Body
cg24779941	14	103,524,901	0.010472	0.326	0.418				*CDC42BPB*	TSS1500
cg18809076	7	55,177,623	1.04E-18	0.326	0.302				*EGFR*	Body
cg04920761	2	43,455,103	6.86E-08	0.326	0.255				*ZFP36L2*	TSS1500
cg03686593	18	11,149,470	0.031495	0.326	0.299				*FAM38B*	TSS1500
cg18546689	13	43,148,295	6.41E-06	0.326	0.306				*TNFSF11*	1stExon
cg05376810	17	74,511,493	2.33E-15	0.326	0.297					
cg08750440	2	43,447,375	0.005788	0.326	0.239					
cg01627483	1	32,708,341	1.11E-06	0.326	0.284				*MTMR9L*	TSS1500
cg21105787	10	73,847,257	1.79E-08	0.326	0.327				*SPOCK2*	Body
cg17951713	8	130,698,161	3E-05	0.326	0.345					
cg23523215	2	202,508,694	7.9E-11	0.326	0.281				*ALS2CR4*	TSS1500
cg05813818	16	875,966	8.62E-05	0.326	0.256					
cg27454883	10	102,626,432	0.007759	0.326	0.376					
cg00126320	8	23,584,644	4.91E-05	0.326	0.226					
cg18711066	16	68,118,822	1.09E-06	0.326	0.346	3.83E-41	0.242		*NFATC3*	TSS1500
cg26985119	X	49,022,801	1.07E-08	0.326	0.249				*MAGIX*	3'UTR
cg16103959	1	200,004,529	1.26E-06	0.326	0.357				*NR5A2*	Body
cg26073885	12	31,506,955	6.28E-11	0.326	0.262					
cg26295921	7	157,476,575	5.02E-10	0.326	0.329				*PTPRN2*	Body
cg09493505	7	49,813,111	0.029131	0.326	0.467				*VWC2*	TSS200
cg06858555	14	105,144,735	5.07E-15	0.326	0.283					
cg00651829	22	23,413,066	0.016969	0.326	0.155				*RTDR1*	Body
cg10026473	1	45,792,540	0.000261	0.326	0.327				*HPDL*	TSS200
cg21489622	6	143,247,772	1.74E-06	0.326	0.235				*HIVEP2*	5'UTR
cg24715680	2	215,675,429	8.09E-20	0.326	0.265				*BARD1*	TSS1500

cg11972677	11	30,605,704	3.84E-05	0.325	0.298				*MPPED2*	5'UTR
cg26266431	7	157,361,564	0.000265	0.325	0.311			Yes	*PTPRN2*	Body
cg03905795	11	70,670,880	1.53E-07	0.325	0.261				*SHANK2*	Body
cg04622802	11	27,015,872	2.96E-06	0.325	0.303	3.49E-33	0.307		*FIBIN*	5'UTR
cg00462951	12	5,539,736	4.91E-09	0.325	0.351					
cg08110785	17	55,952,217	2.16E-08	0.325	0.310				*CUEDC1*	Body
cg08993878	12	98,151,379	2.68E-22	0.325	0.307					
cg01399219	7	44,145,110	1.4E-09	0.325	0.375				*AEBP1*	Body
cg07529754	2	50,575,098	2.26E-05	0.325	0.244				*NRXN1*	Body
cg20495819	2	30,448,529	9.29E-06	0.325	0.240					
cg08317263	5	150,603,169	1.99E-11	0.325	0.290	2.72E-18	0.176		*CCDC69*	Body
cg06231618	X	40,504,059	1.28E-09	0.325	0.285				*CXorf38*	Body
cg13231700	2	222,435,709	0.004215	0.325	0.188				*EPHA4*	Body
cg19642402	15	77,287,762	1.96E-16	0.325	0.316				*PSTPIP1*	5'UTR
cg06795971	4	106,067,365	0.000211	0.325	0.255				*TET2*	TSS1500
cg08067566	4	13,529,282	0.004272	0.325	0.314					
cg26236177	14	61,110,649	3.03E-07	0.325	0.245					
cg15043615	17	74,025,708	1.77E-06	0.325	0.323					
cg20351171	22	37,720,723	4.87E-15	0.325	0.319					
cg02474799	19	19,072,780	0.004936	0.325	0.241					
cg02874376	14	101,193,397	0.004407	0.325	0.246				*DLK1*	1stExon
cg16697214	7	50,343,361	4.92E-09	0.325	0.351				*IKZF1*	TSS1500
cg05738152	2	287,354	4.35E-11	0.325	0.283				*FAM150B*	Body
cg26582768	10	102,792,043	0.008474	0.325	0.385				*PDZD7*	TSS1500
cg01889143	5	134,825,791	1.03E-07	0.325	0.323					
cg12087004	3	195,906,436	3.63E-09	0.325	0.329					
cg21146184	22	46,262,436	5.06E-07	0.325	0.336					
cg05740243	10	135,160,775	8.22E-09	0.325	0.288				*PRAP1*	TSS200
cg02236650	10	17,269,775	2.82E-06	0.325	0.321				*VIM*	TSS1500
cg12355172	11	67,418,315	8.39E-11	0.325	0.317				*ACY3*	TSS200
cg09938462	22	19,706,365	0.000267	0.325	0.319				*SEPT5*	Body
cg22151644	12	54,444,047	7.87E-05	0.325	0.232				*HOXC4*	5'UTR
cg26390041	17	70,201,666	2.18E-07	0.325	0.317					
cg05010648	7	73,446,865	1.33E-14	0.325	0.338				*ELN*	Body

cg08183724	20	21,497,429	0.021571	0.325	0.395					
cg10762199	14	61,934,948	7.65E-15	0.325	0.315				*PRKCH*	Body
cg17645677	5	149,185,704	3.43E-24	0.325	0.305				*PPARGC1B*	Body
cg21150901	17	34,957,586	3.33E-06	0.325	0.210				*MRM1*	TSS1500
cg19129842	3	128,565,090	5.52E-11	0.325	0.284					
cg04074945	11	46,071,833	1.76E-20	0.325	0.324				*PHF21A*	Body
cg24939845	1	110,627,473	4.2E-06	0.325	0.327					
cg18330866	7	94,673,120	8.27E-14	0.325	0.326				*PPP1R9A*	Body
cg23821329	10	17,269,643	6.57E-08	0.325	0.315				*VIM*	TSS1500
cg10916429	15	64,799,784	9.41E-14	0.325	0.318				*ZNF609*	Body
cg20178172	6	30,711,655	4.18E-23	0.325	0.321				*IER3*	3'UTR
cg05350879	4	88,451,223	3.21E-12	0.325	0.326				*SPARCL1*	TSS1500
cg05549655	15	75,019,143	1.73E-14	0.325	0.308				*CYP1A1*	TSS1500
cg24973993	12	120,242,513	5.08E-15	0.325	0.319				*CIT*	Body
cg10130900	16	68,003,410	1.13E-07	0.325	0.224				*SLC12A4*	TSS1500
cg21193484	14	61,938,892	5.25E-07	0.325	0.292				*PRKCH*	Body
cg27072996	19	822,635	5.81E-05	0.325	0.334				*LPPR3*	TSS1500
cg15181598	7	38,350,921	0.007131	0.325	0.049					
cg06744119	10	88,730,599	0.005158	0.325	0.239				*C10orf116*	3'UTR
cg07058988	17	80,297,159	0.000124	0.325	0.380					
cg27234864	13	21,295,941	0.043475	0.325	0.179				*IL17D*	Body
cg02351381	12	110,172,346	1.96E-13	0.325	0.329	1.1E-17	0.190		*MGC14436*	Body
cg20438404	7	100,770,192	4.74E-09	0.325	0.326				*SERPINE1*	TSS200
cg16620382	6	43,211,213	0.046642	0.325	0.335				*TTBK1*	TSS200
cg00498305	10	119,000,236	2.51E-13	0.324	0.307				*SLC18A2*	TSS1500
cg26227186	1	95,393,138	0.01455	0.324	0.121				*CNN3*	TSS1500
cg20198242	6	29,855,110	6.45E-19	0.324	0.268				*HLA-H*	TSS1500
cg26405020	15	91,427,363	3.29E-14	0.324	0.299				*FES*	TSS1500
cg16800165	14	85,996,364	1.01E-09	0.324	0.336				*FLRT2*	TSS200
cg17655415	10	89,802,559	1.18E-07	0.324	0.297					
cg02259081	12	53,268,433	3.84E-18	0.324	0.286					
cg07638650	10	43,697,849	2.01E-06	0.324	0.334				*RASGEF1A*	Body
cg04574090	11	74,178,749	1.25E-06	0.324	0.270				*KCNE3*	TSS200
cg08015801	6	26,088,040	0.001093	0.324	0.322				*HFE*	Body

cg15233183	19	16,187,102	0.00219	0.324	0.309				*TPM4*	Body
cg07070728	3	141,399,954	1.31E-08	0.324	0.314					
cg25888561	10	44,163,067	4.1E-05	0.324	0.274					
cg07411620	2	176,948,759	0.045472	0.324	0.289				*EVX2*	TSS200
cg25533774	7	16,505,409	0.000426	0.324	0.338	6.21E-15	0.159		*SOSTDC1*	1stExon
cg27372920	5	52,778,745	7.77E-10	0.324	0.304				*FST*	Body
cg27451672	7	99,156,362	0.007125	0.324	0.269				*ZNF655*	1stExon
cg18130044	20	21,682,362	0.003777	0.324	0.353					
cg13501793	14	55,031,733	1.11E-05	0.324	0.313					
cg15575982	1	87,796,349	0.000493	0.324	0.311				*LMO4*	5'UTR
cg22614142	8	145,698,232	0.044108	0.324	0.381				*KIFC2*	Body
cg21843616	4	85,424,469	0.001115	0.324	0.299					
cg05334053	1	3,474,202	4.76E-05	0.324	0.287				*MEGF6*	Body
cg09829319	6	10,882,238	5.96E-05	0.324	0.382	4.3E-12	0.211		*GCM2*	TSS200
cg00063828	16	85,749,496	1.5E-12	0.324	0.304				*C16orf74*	Body
cg17287767	2	192,015,279	0.0183	0.324	0.215				*STAT4*	5'UTR
cg00559249	5	131,831,244	3.51E-12	0.324	0.257					
cg14283454	18	46,449,389	0.000244	0.324	0.276				*SMAD7*	Body
cg24960799	6	24,908,812	6.07E-11	0.324	0.306				*FAM65B*	5'UTR
cg16017979	8	74,333,589	1.37E-27	0.324	0.313				*STAU2*	3'UTR
cg21328779	3	111,629,875	6.88E-12	0.324	0.312				*PHLDB2*	Body
cg18723937	7	37,955,713	2.74E-05	0.324	0.338				*SFRP4*	1stExon
cg09858188	7	156,814,480	0.000274	0.324	0.312					
cg14537533	12	52,626,904	0.000275	0.324	0.353				*KRT7*	TSS200
cg26219092	8	134,388,022	3.02E-11	0.324	0.285					
cg00466116	1	59,221,344	0.002952	0.324	0.384			Yes		
cg10308253	6	149,803,111	2.18E-09	0.324	0.350				*ZC3H12D*	5'UTR
cg12963656	3	101,659,687	1.16E-13	0.324	0.297				*LOC152225*	TSS200
cg09226986	6	160,852,328	5.92E-13	0.324	0.295				*SLC22A3*	Body
cg16682899	16	85,508,110	5.11E-35	0.324	0.300					
cg10191725	X	25,034,625	3.13E-07	0.324	0.315				*ARX*	TSS1500
cg00887153	16	71,559,593	3.47E-13	0.324	0.281				*CHST4*	TSS1500
cg14270346	9	38,026,076	2.78E-15	0.324	0.324				*SHB*	Body
cg09077934	15	29,407,792	7.58E-06	0.324	0.289				*APBA2*	Body

cg10752158	4	81,185,022	7.47E-05	0.324	0.273					
cg00961866	10	102,905,737	4.76E-08	0.324	0.354					
cg13078563	15	27,016,796	0.000839	0.324	0.237				*GABRB3*	Body
cg25757115	1	46,913,787	1.55E-10	0.324	0.285					
cg09049251	12	50,297,477	4.7E-07	0.324	0.266				*FAIM2*	Body
cg15963552	3	99,594,931	0.048519	0.324	0.412				*C3orf26*	Body
cg21495612	14	96,342,250	0.010948	0.324	0.441					
cg25044876	22	43,041,146	7.6E-09	0.324	0.284				*CYB5R3*	5'UTR
cg09276998	10	29,991,884	0.000747	0.324	0.249				*SVIL*	5'UTR
cg02968175	16	4,690,020	1.9E-19	0.324	0.289				*MGRN1*	Body
cg14602839	17	63,060,161	2.01E-20	0.324	0.313					
cg23741863	8	97,657,833	2.15E-05	0.324	0.235				*PGCP*	5'UTR
cg11716270	1	39,025,311	0.001558	0.324	0.276					
cg13739115	10	121,094,427	5.32E-11	0.324	0.315				*GRK5*	Body
cg09327714	1	91,316,253	0.000325	0.324	0.319					
cg09967633	16	84,918,794	1.37E-05	0.323	0.281				*CRISPLD2*	Body
cg12664038	1	156,611,194	1.02E-09	0.323	0.365				*BCAN*	TSS1500
cg23352146	11	12,701,929	6.16E-05	0.323	0.352				*TEAD1*	5'UTR
cg10758057	15	29,033,893	9.07E-06	0.323	0.341					
cg08876932	11	71,955,547	5.41E-06	0.323	0.267	8.91E-11	0.189		*PHOX2A*	TSS1500
cg04874782	2	241,900,108	0.000217	0.323	0.260					
cg10630155	4	16,085,666	4.31E-28	0.323	0.304				*PROM1*	TSS200
cg12686055	12	45,628,262	1.5E-16	0.323	0.280				*ANO6*	Body
cg10303487	8	105,479,058	0.007735	0.323	0.315	7.42E-20	0.263		*DPYS*	1stExon
cg14327531	10	8,097,331	2.65E-06	0.323	0.328				*GATA3*	5'UTR
cg16169675	13	113,594,722	2.16E-16	0.323	0.306					
cg05507490	2	242,808,479	0.000503	0.323	0.376					
cg10932018	5	54,516,487	2.35E-06	0.323	0.337					
cg21438527	6	26,189,355	0.001955	0.323	0.285				*HIST1H4D*	TSS200
cg02656560	17	19,967,600	5.41E-30	0.323	0.306					
cg05621157	13	99,404,279	2.25E-05	0.323	0.307				*SLC15A1*	Body
cg13618979	19	16,186,840	4.38E-07	0.323	0.306				*TPM4*	Body
cg06949439	1	206,729,034	1.17E-22	0.323	0.300				*RASSF5*	Body
cg17443359	1	91,300,231	0.006884	0.323	0.424					

cg22059073	22	17,602,570	1.91E-28	0.323	0.270				*CECR6*	TSS1500
cg27603915	11	20,692,113	0.003104	0.323	0.320				*NELL1*	Body
cg24909548	19	36,736,272	1.36E-06	0.323	0.227					
cg04401986	4	81,189,927	0.005565	0.323	0.307				*FGF5*	Body
cg25119690	3	55,524,048	0.022465	0.323	0.147					
cg01718447	7	30,722,327	0.004228	0.323	0.487				*CRHR2*	TSS200
cg17133668	1	162,792,177	6.42E-06	0.323	0.363					
cg23483251	X	119,445,319	3.68E-10	0.323	0.296				*FAM70A*	1stExon
cg01313977	3	44,626,204	2.19E-07	0.323	0.220				*ZNF660*	TSS1500
cg04273431	6	30,523,215	1.07E-30	0.323	0.311				*GNL1*	Body
cg25122629	2	54,936,822	0.000297	0.323	0.266					
cg09625066	X	46,937,571	2.02E-21	0.323	0.314	1.65E-18	0.236		*RGN*	TSS1500
cg03007522	8	11,562,685	6.64E-05	0.323	0.366				*GATA4*	5'UTR
cg21306240	1	38,230,845	0.004592	0.323	0.355				*EPHA10*	TSS200
cg07654896	X	46,937,829	0.000164	0.323	0.287	1.03E-15	0.273		*RGN*	1stExon
cg02214783	1	3,688,257	3.06E-18	0.323	0.286				*LOC388588*	TSS1500
cg05407338	17	8,213,522	2.19E-07	0.323	0.333				*ARHGEF15*	TSS200
cg02790379	1	22,140,769	1.58E-10	0.323	0.275				*LDLRAD2*	Body
cg26503125	10	80,181,190	1.15E-20	0.323	0.298					
cg04622024	1	38,201,001	4.47E-16	0.323	0.280				*EPHA10*	Body
cg04319873	2	164,205,343	1.47E-12	0.323	0.319					
cg23937816	8	91,997,231	1.17E-09	0.323	0.346					
cg14127046	2	236,147,233	6.48E-09	0.323	0.309					
cg01656955	1	40,137,966	5.69E-07	0.323	0.293				*NT5C1A*	TSS1500
cg13031611	12	115,132,761	1.48E-26	0.323	0.286					
cg14239515	19	38,885,242	0.00038	0.323	0.339				*SPRED3*	Body
cg19782243	11	125,133,120	3.06E-17	0.323	0.324				*PKNOX2*	5'UTR
cg18180415	11	115,530,547	0.00018	0.323	0.285					
cg26332253	6	121,758,729	3.76E-06	0.323	0.357				*GJA1*	5'UTR
cg17299437	4	54,974,644	0.000148	0.323	0.320					
cg21079003	15	93,615,146	1.04E-05	0.323	0.306				*RGMA*	Body
cg13514049	9	110,495,709	1.28E-15	0.323	0.329					
cg00785170	1	201,438,757	3.64E-05	0.323	0.322				*PHLDA3*	TSS1500
cg24405567	15	70,787,565	1.3E-13	0.323	0.300					

cg05031521	16	1,030,561	1.64E-10	0.323	0.320				*SOX8*	TSS1500
cg23616139	1	221,051,907	4.26E-07	0.323	0.319				*HLX*	TSS1500
cg09896412	1	98,519,101	2.76E-12	0.323	0.308					
cg12264626	8	86,350,381	7.45E-10	0.323	0.320				*CA3*	TSS1500
cg00536939	11	47,279,352	1.24E-09	0.323	0.293				*NR1H3*	TSS200
cg04699104	2	12,858,912	0.00188	0.323	0.393				*TRIB2*	Body
cg17385175	X	23,350,552	0.011417	0.323	0.242					
cg06169131	10	103,536,348	1.09E-09	0.323	0.305				*FGF8*	TSS1500
cg17318990	12	90,101,606	0.001909	0.322	0.276				*LOC338758*	TSS1500
cg04178858	17	38,334,170	0.007773	0.322	0.235				*RAPGEFL1*	TSS200
cg24577131	6	139,613,426	2.81E-09	0.322	0.329				*TXLNB*	TSS1500
cg01472882	2	85,980,609	0.000002	0.322	0.272				*ATOH8*	TSS1500
cg05664072	2	239,198,294	3.02E-11	0.322	0.269	3.69E-36	0.222		*PER2*	TSS1500
cg12435551	1	118,679,290	2.88E-05	0.322	0.286				*SPAG17*	Body
cg05151811	10	17,270,250	3.77E-06	0.322	0.356				*VIM*	TSS200
cg23178308	21	45,148,879	3.78E-06	0.322	0.234	4.23E-25	0.224		*PDXK*	Body
cg17892159	2	42,274,610	0.003391	0.322	0.329				*PKDCC*	TSS1500
cg07974511	2	63,283,013	4.97E-07	0.322	0.323				*OTX1*	Body
cg17343563	6	50,803,945	4.24E-07	0.322	0.331				*TFAP2B*	Body
cg17410295	12	109,729,809	0.008849	0.322	0.227				*FOXN4*	Body
cg20142377	6	29,910,206	3.02E-07	0.322	0.302				*HLA-A*	TSS200
cg25750228	12	53,267,626	0.000621	0.322	0.247					
cg08533773	5	113,392,004	9.24E-09	0.322	0.351					
cg25833672	8	144,853,229	2.06E-05	0.322	0.280					
cg03298700	2	220,295,753	6.33E-06	0.322	0.316					
cg05050657	1	46,957,105	3.01E-06	0.322	0.342					
cg23106912	1	85,464,261	1.61E-15	0.322	0.280				*MCOLN2*	TSS1500
cg11500660	21	44,036,692	0.000208	0.322	0.254					
cg11343211	17	15,686,309	0.003013	0.322	0.308					
cg22746681	10	51,912,631	3.04E-06	0.322	0.249					
cg14439761	9	19,788,317	0.007566	0.322	0.268				*SLC24A2*	TSS1500
cg27432653	1	92,952,461	1.02E-07	0.322	0.326			Yes	*GFI1*	TSS1500
cg12817840	5	124,070,989	1.4E-08	0.322	0.248				*ZNF608*	Body
cg21335378	2	173,150,101	2.04E-09	0.322	0.274					

cg05096161	17	46,114,674	9.56E-06	0.322	0.333				*MIR152*	TSS200
cg25773259	12	94,019,073	1.47E-11	0.322	0.340					
cg00174901	19	709,889	9.23E-15	0.322	0.334	8.45E-31	0.194		*PALM*	Body
cg01175020	10	102,896,475	1.12E-11	0.322	0.303				*TLX1*	Body
cg03127334	21	40,033,634	0.000241	0.322	0.221				*ERG*	5'UTR
cg16886987	21	38,378,634	0.00332	0.322	0.229				*DSCR6*	TSS1500
cg23320056	1	155,948,742	5.24E-13	0.322	0.263	2.25E-31	0.140		*ARHGEF2*	TSS1500
cg18725573	20	62,368,956	0.000875	0.322	0.295				*LIME1*	Body
cg20723355	17	6,679,597	4.96E-05	0.322	0.276	4.45E-15	0.217		*FBXO39*	5'UTR
cg01524853	12	54,447,807	5.94E-14	0.322	0.288				*HOXC4*	1stExon
cg24520538	6	42,739,049	9.03E-23	0.322	0.305					
cg00462430	1	232,608,508	6.67E-11	0.322	0.333				*SIPA1L2*	Body
cg25585712	4	41,869,213	9.15E-18	0.322	0.305					
cg24803391	7	21,209,338	3.45E-10	0.322	0.285					
cg19058865	19	4,327,350	1.4E-06	0.322	0.321				*STAP2*	Body
cg02151779	X	48,327,875	5.7E-07	0.322	0.311				*SLC38A5*	5'UTR
cg02900441	1	15,911,134	0.020849	0.322	0.252				*AGMAT*	Body
cg01572891	7	4,184,354	8.67E-20	0.322	0.337				*SDK1*	Body
cg21000227	17	70,216,183	2.01E-05	0.322	0.344					
cg06855337	2	189,489,191	9.69E-09	0.322	0.308					
cg04060366	6	168,079,025	4.91E-07	0.322	0.254					
cg01076971	8	97,657,803	0.008874	0.322	0.197				*PGCP*	5'UTR
cg09770579	16	17,407,429	4.08E-08	0.322	0.296				*XYLT1*	Body
cg24360909	15	49,189,538	1.8E-13	0.322	0.286				*SHC4*	Body
cg19777067	4	174,440,103	0.000431	0.322	0.298					
cg20134151	6	150,463,500	4.54E-08	0.322	0.235	2.51E-28	0.242		*PPP1R14C*	TSS1500
cg26596964	10	102,495,354	0.002217	0.322	0.309					
cg11664987	1	38,201,123	4.74E-07	0.322	0.268				*EPHA10*	Body
cg25370658	6	7,673,306	1.05E-22	0.322	0.293					
cg17719053	1	75,198,211	7.73E-05	0.322	0.349				*TYW3*	TSS1500
cg26624398	5	168,726,889	2.6E-05	0.321	0.262				*SLIT3*	Body
cg19989295	14	24,641,077	1.96E-10	0.321	0.318				*REC8*	TSS200
cg03469862	11	68,924,853	2.71E-14	0.321	0.305					
cg14601444	2	74,607,536	3.87E-23	0.321	0.331				*DCTN1*	TSS200

cg05411132	X	99,195,115	2.32E-09	0.321	0.283				*LOC442459*	TSS1500
cg25835179	11	67,418,291	4.07E-13	0.321	0.320				*ACY3*	TSS200
cg02028751	17	74,378,849	6.18E-05	0.321	0.241					
cg25551714	11	116,452,003	0.001556	0.321	0.388					
cg27645955	22	21,386,885	0.013854	0.321	0.131				*SLC7A4*	TSS200
cg06223539	11	70,517,374	6.76E-06	0.321	0.321				*SHANK2*	Body
cg09999348	2	46,398,848	3.17E-18	0.321	0.304				*PRKCE*	Body
cg20717768	19	46,379,763	2.03E-18	0.321	0.302					
cg13774080	X	134,156,809	9.79E-14	0.321	0.301				*FAM127C*	TSS1500
cg04858616	1	40,255,010	0.014406	0.321	0.195				*BMP8B*	TSS1500
cg02081266	17	59,529,618	1.57E-07	0.321	0.315					
cg24367957	1	161,931,993	5.36E-20	0.321	0.317					
cg05166871	17	46,233,714	6.07E-16	0.321	0.284				*SKAP1*	Body
cg25486143	3	50,378,527	7.06E-10	0.321	0.318				*RASSF1*	TSS200
cg00152041	20	61,274,867	6.04E-14	0.321	0.319				*SLCO4A1*	5'UTR
cg16195091	17	76,227,996	0.00065	0.321	0.321				*LOC283999*	Body
cg23733123	10	99,472,988	1.86E-12	0.321	0.319				*MARVELD1*	TSS1500
cg03467825	2	171,551,893	1.11E-06	0.321	0.322					
cg06223767	6	393,616	0.003321	0.321	0.261				*IRF4*	Body
cg11456756	6	30,228,069	0.00037	0.321	0.311				*HLA-L*	Body
cg16516400	1	231,177,201	0.001371	0.321	0.295	0.0000266	0.099		*FAM89A*	TSS1500
cg18147543	1	16,276,294	1.52E-36	0.321	0.304				*ZBTB17*	5'UTR
cg13636404	7	99,155,673	0.000371	0.321	0.243	8.5E-19	0.131		*ZNF655*	TSS1500
cg09730500	16	57,662,690	1.09E-20	0.321	0.296				*GPR56*	5'UTR
cg27239280	16	30,907,679	2.84E-06	0.321	0.308				*CTF1*	TSS1500
cg22455667	6	29,895,037	1.94E-09	0.321	0.268					
cg03096954	16	85,347,426	6.89E-17	0.321	0.339					
cg04607844	1	213,090,203	2.76E-08	0.321	0.314					
cg04682791	6	25,313,030	2.97E-17	0.321	0.340				*LRRC16A*	Body
cg00338116	13	43,566,313	4.46E-06	0.321	0.333				*EPSTI1*	5'UTR
cg14858267	3	44,037,760	3.76E-06	0.321	0.390					
cg24685601	1	20,512,550	5.1E-05	0.321	0.294				*UBXN10*	TSS200
cg22907789	17	75,567,709	0.000341	0.321	0.290					
cg19516515	1	155,290,614	7.67E-05	0.321	0.334				*RUSC1*	TSS200

cg10120657	17	77,786,354	3.7E-07	0.321	0.316					
cg10536999	7	26,193,109	0.00414	0.321	0.197				*NFE2L3*	Body
cg07536910	2	176,948,105	2.35E-05	0.321	0.326				*EVX2*	1stExon
cg08812692	5	178,052,235	2.67E-10	0.321	0.324				*CLK4*	5'UTR
cg25335841	7	38,351,094	0.005083	0.321	0.122					
cg05794117	1	228,394,559	1.69E-14	0.321	0.271				*OBSCN*	TSS1500
cg16082336	14	21,539,712	0.000789	0.321	0.216				*FLJ10357*	Body
cg13644317	18	70,536,461	8.44E-09	0.321	0.282					
cg10442157	11	61,284,134	1.59E-20	0.321	0.316				*SYT7*	3'UTR
cg17083323	6	50,803,820	2.09E-06	0.321	0.313				*TFAP2B*	Body
cg03671075	4	78,573,680	7.44E-07	0.321	0.282					
cg13507084	9	34,555,042	5.89E-10	0.321	0.290				*CNTFR*	Body
cg04421973	3	64,670,907	0.00074	0.321	0.188				*ADAMTS9*	Body
cg09815769	1	119,539,738	9.82E-12	0.321	0.282					
cg18327157	7	157,486,718	6.64E-19	0.321	0.318				*PTPRN2*	Body
cg26173847	7	127,671,017	0.029431	0.321	0.196				*LRRC4*	TSS200
cg18391694	1	43,250,900	1.36E-09	0.321	0.288					
cg04566848	6	29,974,715	2.91E-07	0.320	0.327				*HLA-J*	Body
cg04111789	6	41,908,262	0.000221	0.320	0.205				*CCND3*	Body
cg24663970	13	97,647,084	0.00074	0.320	0.360				*OXGR1*	TSS1500
cg10574566	1	203,377,808	2.75E-12	0.320	0.344					
cg08712932	6	133,562,463	8.82E-05	0.320	0.377				*EYA4*	TSS200
cg21468728	17	27,918,207	7.28E-10	0.320	0.306					
cg16688582	7	28,386,631	5.07E-17	0.320	0.301				*CREB5*	5'UTR
cg02928664	19	54,976,506	5.93E-09	0.320	0.312				*CDC42EP5*	Body
cg24375409	1	38,200,920	9.32E-13	0.320	0.303				*EPHA10*	Body
cg18727742	11	288,301	0.002277	0.320	0.250				*ATHL1*	TSS1500
cg20619808	17	27,313,499	8.2E-20	0.320	0.271				*SEZ6*	Body
cg09210933	X	73,641,881	0.001013	0.320	0.141				*SLC16A2*	1stExon
cg15006175	8	145,698,660	1.09E-08	0.320	0.308				*KIFC2*	Body
cg00557947	10	102,419,245	0.023174	0.320	0.417					
cg19997946	6	166,399,986	4.12E-05	0.320	0.285				*C6orf176*	Body
cg25103417	3	157,812,018	1.47E-08	0.320	0.329					
cg13612083	6	127,836,053	1.67E-11	0.320	0.306				*C6orf174*	Body

cg13481638	11	91,957,660	5.69E-07	0.320	0.315					
cg10347326	X	133,684,177	0.005975	0.320	0.191					
cg00920348	1	66,999,304	0.001722	0.320	0.251				*SGIP1*	TSS1500
cg20544372	7	74,064,549	4.1E-05	0.320	0.376					
cg03586474	6	137,242,147	5.3E-06	0.320	0.340				*SLC35D3*	TSS1500
cg08942939	1	119,532,542	2.18E-05	0.320	0.297				*TBX15*	TSS1500
cg07695771	10	126,029,702	5.28E-18	0.320	0.315					
cg07907386	4	41,749,443	0.000347	0.320	0.365				*PHOX2B*	Body
cg04579493	13	100,609,947	0.001597	0.320	0.339					
cg07186765	7	30,633,504	6.94E-11	0.320	0.254				*GARS*	TSS1500
cg05240059	X	124,340,398	5.49E-12	0.320	0.274					
cg18891824	8	49,783,652	6.25E-36	0.320	0.290					
cg23205426	X	152,066,221	0.035385	0.320	0.127					
cg26403843	5	158,634,085	3.41E-05	0.320	0.350				*RNF145*	Body
cg01816515	2	21,022,699	0.000209	0.320	0.280				*C2orf43*	5'UTR
cg17980404	17	38,601,676	1.29E-21	0.320	0.315				*IGFBP4*	Body
cg01407244	8	22,961,007	0.000991	0.320	0.258	8.61E-14	0.176		*TNFRSF10C*	Body
cg12840719	9	21,968,233	7.92E-06	0.320	0.325				*CDKN2A*	Body
cg14483244	17	42,173,343	0.000789	0.320	0.264				*HDAC5*	Body
cg06834506	2	26,396,964	0.00019	0.320	0.337				*FAM59B*	Body
cg25691167	7	19,184,961	0.000918	0.320	0.298	3.31E-13	0.206		*FERD3L*	1stExon
cg11731596	17	15,821,130	6.98E-18	0.320	0.291					
cg07713896	8	94,712,367	0.000657	0.320	0.198				*FAM92A1*	TSS1500
cg00725401	8	70,945,169	2.25E-12	0.320	0.342					
cg02558026	16	30,904,573	2.29E-10	0.320	0.292				*MIR762*	TSS1500
cg05209514	12	69,353,500	9.24E-12	0.320	0.287				*CPM*	5'UTR
cg26890354	5	140,864,020	2.83E-05	0.320	0.343				*PCDHGA4*	Body
cg01180552	16	54,316,931	8.08E-17	0.320	0.293					
cg04308040	13	44,942,303	4.95E-11	0.320	0.323					
cg10375954	1	6,419,608	4.03E-17	0.320	0.307				*ACOT7*	Body
cg01984743	5	172,751,331	0.031585	0.320	0.397				*STC2*	Body
cg08336425	12	125,140,167	4.99E-08	0.320	0.344					
cg19414980	7	92,673,176	1.05E-13	0.320	0.338					
cg22242148	15	74,215,283	4.96E-19	0.320	0.306					

cg12252135	17	79,503,877	0.000727	0.320	0.289				*FSCN2*	Body
cg03660115	3	87,138,400	6.88E-05	0.320	0.251					
cg19847283	19	20,349,250	0.019568	0.320	0.158					
cg08217163	2	66,809,959	1.1E-05	0.320	0.338					
cg20922422	X	24,665,669	0.007936	0.320	0.336	0.0000151	0.120		*PCYT1B*	Body
cg10242602	4	188,916,875	7.51E-06	0.320	0.292				*ZFP42*	TSS200
cg22192069	6	13,305,324	6.25E-49	0.320	0.302				*TBC1D7*	3'UTR
cg00268185	3	123,602,768	0.002166	0.320	0.316				*MYLK*	5'UTR
cg10873171	7	44,145,333	4.66E-15	0.320	0.307				*AEBP1*	Body
cg14889643	18	60,985,636	0.002461	0.320	0.334				*BCL2*	Body
cg01177167	10	101,280,523	5.02E-07	0.319	0.332					
cg07890238	8	8,182,454	2.41E-14	0.319	0.303				*PRAGMIN*	Body
cg26069230	17	29,250,073	2.91E-10	0.319	0.322				*ADAP2*	Body
cg04569837	5	60,921,535	0.000909	0.319	0.308					
cg25599902	1	51,567,771	3.11E-07	0.319	0.266				*C1orf185*	TSS200
cg24128931	21	44,013,597	0.018551	0.319	0.235					
cg00178850	1	236,559,675	0.005786	0.319	0.101				*EDARADD*	Body
cg10101600	2	43,478,743	3.09E-23	0.319	0.305				*THADA*	Body
cg27272677	10	102,891,080	0.013005	0.319	0.240				*TLX1NB*	TSS200
cg21620246	3	126,854,423	0.037225	0.319	0.232					
cg26773567	10	82,173,535	3.85E-08	0.319	0.296				*C10orf58*	TSS200
cg19706516	1	151,103,869	0.000491	0.319	0.281					
cg17558126	1	206,680,250	1.22E-13	0.319	0.262	9.62E-21	0.218		*RASSF5*	TSS1500
cg22471726	17	46,719,276	2.7E-06	0.319	0.321					
cg15994467	12	52,401,523	2.58E-09	0.319	0.314				*GRASP*	Body
cg05907976	6	29,760,028	0.000814	0.319	0.361				*HCG4*	Body
cg08511651	6	26,017,619	3.57E-10	0.319	0.293				*HIST1H1A*	1stExon
cg13523557	7	45,613,725	2.73E-07	0.319	0.245	1.5E-29	0.193		*ADCY1*	TSS1500
cg24522254	2	174,878,113	4.27E-15	0.319	0.299					
cg07352345	5	175,793,049	0.018251	0.319	0.365				*ARL10*	Body
cg18448949	2	176,993,089	1.81E-05	0.319	0.287				*HOXD8*	TSS1500
cg22637538	17	7,348,327	2.51E-10	0.319	0.278				*CHRNB1*	TSS200
cg13150925	12	124,529,439	5.06E-11	0.319	0.300					
cg14482308	2	18,059,109	0.000103	0.319	0.233				*KCNS3*	TSS1500

cg25565383	2	17,699,773	0.029066	0.319	0.342				*RAD51AP2*	TSS200
cg04976685	17	43,449,285	0.006141	0.319	0.314					
cg16331823	6	30,711,835	0.008438	0.319	0.345				*FLOT1*	TSS1500
cg20945853	5	133,406,542	0.000108	0.319	0.158					
cg15126957	5	179,246,594	2.21E-10	0.319	0.281				*SQSTM1*	5'UTR
cg12161228	11	89,224,506	1.08E-12	0.319	0.326				*NOX4*	1stExon
cg26788916	20	62,461,539	1.02E-11	0.319	0.254					
cg08308801	10	119,867,973	3.05E-09	0.319	0.266				*CASC2*	Body
cg23533419	12	54,090,519	3.06E-08	0.319	0.245					
cg20396510	5	66,124,588	0.001161	0.319	0.139				*MAST4*	TSS200
cg02433882	6	44,187,031	0.000634	0.319	0.262				*SLC29A1*	TSS1500
cg08030987	4	83,675,202	1.99E-20	0.319	0.302				*MIR575*	TSS1500
cg12659883	1	119,543,057	2.87E-08	0.319	0.305					
cg25498489	17	8,230,483	1.78E-06	0.319	0.276					
cg14323199	17	60,705,839	8.62E-05	0.319	0.302				*MRC2*	Body
cg01786048	2	239,035,964	9.54E-10	0.319	0.286				*ESPNL*	Body
cg08120986	2	218,816,280	1.96E-18	0.319	0.302					
cg16650529	2	182,548,750	0.000498	0.319	0.220					
cg06755262	6	14,824,437	5.82E-22	0.319	0.336					
cg26090107	11	60,718,882	7.06E-08	0.319	0.319				*SLC15A3*	1stExon
cg05001389	9	135,853,034	1.79E-07	0.319	0.235				*GFI1B*	TSS1500
cg26663609	5	691,161	8.8E-09	0.319	0.299				*TPPP*	5'UTR
cg08105396	1	153,539,058	0.000161	0.319	0.348				*S100A2*	TSS1500
cg04991805	10	99,790,910	3.28E-05	0.319	0.270				*CRTAC1*	TSS1500
cg00146951	10	17,270,376	5.4E-07	0.319	0.331				*VIM*	5'UTR
cg09159285	1	6,664,134	1E-20	0.319	0.295				*KLHL21*	TSS1500
cg08146609	7	18,535,232	6.73E-05	0.319	0.281				*HDAC9*	TSS1500
cg15391601	19	35,849,176	0.003076	0.319	0.332				*FFAR3*	TSS1500
cg19416073	2	96,093,031	2.41E-20	0.319	0.299					
cg03064940	2	129,320,987	1.92E-08	0.319	0.294					
cg06206881	12	5,620,842	3.05E-05	0.319	0.312					
cg17082405	8	145,033,328	4.58E-13	0.319	0.310				*PLEC1*	Body
cg00874055	1	236,306,673	0.004384	0.319	0.183				*GPR137B*	Body
cg05020252	2	233,499,283	5.92E-08	0.319	0.291				*EFHD1*	Body

cg21384402	10	105,036,701	0.006241	0.319	0.277				*INA*	TSS1500
cg12638731	13	36,045,281	0.000125	0.319	0.323				*NBEA*	Body
cg16132520	7	134,143,906	0.004678	0.319	0.224				*AKR1B1*	TSS200
cg11014124	5	140,782,297	1.61E-05	0.319	0.381				*PCDHGA4*	Body
cg23893898	4	6,247,571	1.87E-05	0.319	0.255					
cg10251070	21	44,781,958	3.82E-19	0.318	0.301					
cg01589129	4	74,810,017	0.017269	0.318	0.350					
cg17577122	22	19,511,967	0.000914	0.318	0.327				*CLDN5*	1stExon
cg13198321	15	58,357,891	0.006221	0.318	0.317				*ALDH1A2*	5'UTR
cg10426464	15	62,457,529	0.000298	0.318	0.358				*C2CD4B*	TSS200
cg24909309	8	38,387,291	5.08E-20	0.318	0.305				*C8orf86*	TSS1500
cg02639634	8	54,792,161	1.16E-08	0.318	0.286				*RGS20*	Body
cg08980198	7	150,676,529	2.71E-05	0.318	0.239					
cg17429772	7	158,269,409	5.7E-11	0.318	0.326				*PTPRN2*	Body
cg20999347	6	30,095,517	5.72E-20	0.318	0.283			Yes		
cg05850327	13	20,768,236	2E-08	0.318	0.299				*GJB2*	TSS1500
cg10588362	11	64,728,445	0.002894	0.318	0.315				*C11orf85*	TSS1500
cg18614735	8	41,583,523	7.26E-10	0.318	0.319				*ANK1*	Body
cg05897030	12	27,702,201	6.46E-13	0.318	0.339				*PPFIBP1*	5'UTR
cg08549661	2	131,185,585	3.97E-05	0.318	0.335					
cg14775423	4	57,372,356	7.29E-11	0.318	0.334				*ARL9*	5'UTR
cg20894686	6	50,803,903	1.32E-07	0.318	0.339				*TFAP2B*	Body
cg06420903	16	88,186,669	1.48E-28	0.318	0.304					
cg17985646	7	35,293,759	0.008575	0.318	0.339				*TBX20*	TSS200
cg12349884	6	27,106,988	7.92E-07	0.318	0.299				*HIST1H2BK*	3'UTR
cg15110403	19	17,392,923	0.002558	0.318	0.273				*ANKLE1*	Body
cg19276110	1	20,396,593	2.59E-12	0.318	0.316				*PLA2G5*	TSS200
cg03965496	1	147,718,157	2.94E-08	0.318	0.319					
cg22474464	20	21,492,914	0.010438	0.318	0.444				*NKX2-2*	Body
cg16530177	6	6,320,324	0.003697	0.318	0.267				*F13A1*	5'UTR
cg17204129	7	117,119,601	3.47E-08	0.318	0.312				*CFTR*	TSS1500
cg08380379	2	201,652,352	1.23E-14	0.318	0.299				*AOX2P*	Body
cg12516359	11	17,717,280	0.004407	0.318	0.405					
cg17871993	7	65,673,056	3.44E-12	0.318	0.316				*TPST1*	5'UTR

cg23338503	5	169,064,530	0.008057	0.318	0.319				*DOCK2*	Body
cg08151731	15	48,938,576	9.07E-10	0.318	0.247				*FBN1*	TSS1500
cg17173663	12	115,135,859	2.39E-09	0.318	0.287					
cg19043574	2	99,439,533	8.97E-08	0.318	0.339				*C2orf55*	Body
cg26335633	7	64,029,999	8.18E-23	0.318	0.322					
cg12648074	5	140,864,474	2.11E-11	0.318	0.304				*PCDHGA4*	Body
cg00959636	6	134,499,464	0.000765	0.318	0.323				*SGK1*	Body
cg07862461	20	1,914,231	5.18E-08	0.318	0.293				*SIRPA*	Body
cg25975621	1	217,311,177	3.44E-05	0.318	0.295				*ESRRG*	TSS200
cg14186641	6	88,876,741	0.023845	0.318	0.250				*CNR1*	TSS1500
cg20669834	3	123,339,417	5.29E-08	0.318	0.317				*MYLK*	1stExon
cg20129082	11	19,736,150	7.66E-05	0.318	0.322				*LOC100126784*	TSS200
cg01094950	2	43,455,097	4.24E-06	0.318	0.241				*ZFP36L2*	TSS1500
cg07570498	1	33,813,321	1.02E-21	0.318	0.301				*PHC2*	5'UTR
cg07674834	17	8,230,235	0.001075	0.318	0.343					
cg00467854	X	25,040,358	0.000461	0.318	0.278					
cg04482110	17	41,364,121	1.51E-09	0.318	0.306	6.36E-25	0.169		*TMEM106A*	5'UTR
cg11558551	19	17,516,442	3.49E-08	0.318	0.298				*BST2*	TSS200
cg21346043	8	86,351,067	3.53E-09	0.318	0.322				*CA3*	1stExon
cg03699904	3	170,746,029	1.06E-09	0.318	0.271	4.57E-17	0.215		*SLC2A2*	TSS1500
cg21884231	3	170,303,721	0.002007	0.318	0.416				*SLC7A14*	5'UTR
cg02683114	2	24,398,427	1.3E-06	0.318	0.248				*C2orf84*	Body
cg13914893	9	137,980,958	0.000134	0.318	0.270				*OLFM1*	Body
cg21899310	11	57,365,725	0.001746	0.318	0.296				*SERPING1*	1stExon
cg03257179	15	41,805,946	2.18E-08	0.318	0.310				*LTK*	1stExon
cg08170869	6	26,188,969	0.000207	0.318	0.312				*HIST1H4D*	3'UTR
cg11833968	6	79,620,685	9.51E-07	0.318	0.330					
cg05729490	7	27,404,187	1.93E-16	0.318	0.284					
cg11996592	8	144,577,094	5.99E-26	0.318	0.311				*ZC3H3*	Body
cg06942445	X	54,947,373	0.00081	0.318	0.435	5.15E-27	0.258		*TRO*	5'UTR
cg08184792	12	48,592,270	2.13E-05	0.318	0.324					
cg07550362	7	97,361,201	6.28E-05	0.318	0.298	1.55E-29	0.217		*TAC1*	TSS200
cg24544490	1	155,290,961	6.57E-08	0.318	0.264				*RUSC1*	5'UTR
cg08362628	3	54,861,701	9.76E-06	0.318	0.334				*CACNA2D3*	Body

cg15241084	X	12,886,121	2.41E-06	0.318	0.353				*TLR7*	Body
cg13036352	6	30,651,825	1.78E-09	0.318	0.329				*KIAA1949*	Body
cg10864952	7	65,671,987	5.74E-11	0.317	0.328				*TPST1*	5'UTR
cg19728226	2	176,968,744	1.14E-05	0.317	0.312					
cg15081566	11	111,169,865	0.00861	0.317	0.365				*C11orf92*	5'UTR
cg00578638	17	38,334,161	1.13E-08	0.317	0.272				*RAPGEFL1*	TSS200
cg15052747	9	138,942,591	1.48E-11	0.317	0.301				*NACC2*	5'UTR
cg09645574	6	42,879,382	6.83E-07	0.317	0.320					
cg11508595	2	192,135,315	2.96E-15	0.317	0.313				*MYO1B*	5'UTR
cg15920906	12	111,619,414	9.46E-08	0.317	0.277				*CUX2*	Body
cg16732616	1	50,886,782	0.004738	0.317	0.337				*DMRTA2*	Body
cg17284804	1	67,218,135	1.59E-06	0.317	0.333				*TCTEX1D1*	TSS200
cg03951374	15	37,390,176	5.21E-06	0.317	0.368				*MEIS2*	1stExon
cg21737444	22	38,071,591	7.47E-09	0.317	0.291				*LGALS1*	TSS200
cg03932723	11	63,258,523	0.000159	0.317	0.272				*HRASLS5*	1stExon
cg10881128	1	44,031,826	4.23E-07	0.317	0.326				*PTPRF*	Body
cg23160016	4	46,391,929	1.95E-05	0.317	0.298				*GABRA2*	TSS1500
cg08935118	15	35,014,229	7.12E-07	0.317	0.322					
cg04923020	12	46,005,007	1.97E-18	0.317	0.320					
cg19660812	8	38,617,447	1.15E-08	0.317	0.315				*TACC1*	5'UTR
cg03872783	21	26,934,885	0.001211	0.317	0.259				*MIR155HG*	Body
cg10811474	19	8,428,787	0.009318	0.317	0.236				*ANGPTL4*	TSS1500
cg07803375	7	811,206	3.84E-05	0.317	0.316				*HEATR2*	Body
cg26290543	11	45,377,324	1.42E-13	0.317	0.335					
cg18272353	14	95,238,706	0.000391	0.317	0.314					
cg12866112	7	157,372,332	7.04E-24	0.317	0.323				*PTPRN2*	Body
cg15198094	1	200,003,342	3.4E-10	0.317	0.301				*NR5A2*	Body
cg15549973	1	159,823,687	2.38E-05	0.317	0.255				*C1orf204*	Body
cg09752627	4	25,657,160	0.008319	0.317	0.210				*SLC34A2*	TSS1500
cg00079219	12	54,360,131	3.47E-06	0.317	0.253				*HOTAIR*	Body
cg06148264	6	2,841,468	1.15E-08	0.317	0.284	1.01E-21	0.186		*SERPINB1*	5'UTR
cg12360736	3	151,985,869	1.15E-08	0.317	0.273	2.37E-21	0.171		*MBNL1*	1stExon
cg12956688	19	18,415,646	2.47E-15	0.317	0.318					
cg22336806	6	2,903,704	7.87E-08	0.317	0.274				*SERPINB9*	TSS200

cg01841641	2	223,169,965	0.004364	0.317	0.426					
cg22982811	7	28,032,986	4.01E-08	0.317	0.304				*JAZF1*	Body
cg07149609	1	170,630,734	1.55E-08	0.317	0.322					
cg26465391	19	58,220,773	9.42E-07	0.317	0.266				*ZNF154*	TSS200
cg27168780	6	28,521,796	4.85E-05	0.317	0.302					
cg00057231	3	48,631,958	1.91E-13	0.317	0.278				*COL7A1*	Body
cg00089814	3	72,641,008	9.35E-12	0.317	0.312					
cg06312813	4	74,964,493	3.31E-07	0.317	0.309				*CXCL2*	Body
cg14021375	19	16,999,800	1.24E-09	0.317	0.283				*F2RL3*	TSS200
cg03434384	12	27,703,097	3.94E-25	0.317	0.313				*PPFIBP1*	5'UTR
cg17138769	10	118,892,211	0.006807	0.317	0.393				*VAX1*	Body
cg19268119	2	225,266,880	0.02429	0.317	0.341				*FAM124B*	TSS200
cg17606785	14	23,834,808	1.47E-09	0.317	0.287	8.11E-33	0.229		*EFS*	1stExon
cg17773502	X	41,302,010	6.44E-08	0.317	0.210					
cg04993975	17	35,294,472	0.016022	0.317	0.340				*LHX1*	TSS200
cg02553486	4	47,840,095	1.49E-10	0.317	0.295	2.03E-12	0.169		*CORIN*	TSS200
cg02990291	7	96,625,586	0.00026	0.317	0.345				*DLX6AS*	Body
cg01370541	4	41,882,812	6.6E-05	0.317	0.290					
cg09313745	12	3,373,666	8E-10	0.317	0.277				*TSPAN9*	Body
cg03329165	3	112,052,218	0.000183	0.317	0.415	6.7E-12	0.154		*CD200*	Body
cg12118269	10	102,893,980	6.48E-05	0.317	0.294				*TLX1*	Body
cg10242496	6	137,242,316	4.91E-06	0.317	0.326				*SLC35D3*	TSS1500
cg13267264	8	70,983,600	0.014871	0.317	0.421				*PRDM14*	TSS200
cg03276022	11	117,069,900	9.63E-13	0.317	0.317				*TAGLN*	TSS200
cg20742009	3	182,879,817	0.00027	0.317	0.166				*LAMP3*	Body
cg27603796	7	117,512,803	0.000236	0.317	0.358	1.62E-23	0.224		*CTTNBP2*	Body
cg20943084	5	140,012,769	0.002527	0.317	0.359				*CD14*	1stExon
cg03559682	11	6,439,864	0.000266	0.316	0.133				*APBB1*	5'UTR
cg24710320	3	192,958,832	0.013183	0.316	0.192				*MGC2889*	TSS1500
cg26343883	2	164,205,032	1.09E-05	0.316	0.254					
cg06355129	1	156,646,293	4.29E-07	0.316	0.343				*NES*	1stExon
cg17357479	1	184,005,717	0.000138	0.316	0.222				*GLT25D2*	Body
cg03362215	6	78,176,806	0.00042	0.316	0.314					
cg17291136	8	26,087,851	1.43E-18	0.316	0.308					

cg05319305	4	101,111,927	9.24E-08	0.316	0.271				*DDIT4L*	TSS1500
cg00966099	4	184,718,401	4.55E-05	0.316	0.327					
cg06650847	X	63,004,884	0.003827	0.316	0.359					
cg10800833	4	57,521,657	0.027286	0.316	0.165				*HOPX*	Body
cg23982858	12	95,941,869	5.09E-06	0.316	0.252				*USP44*	5'UTR
cg04192867	10	93,647,053	1.29E-06	0.316	0.288					
cg21202716	11	70,508,022	7.28E-07	0.316	0.302				*SHANK2*	Body
cg13787982	19	20,348,926	0.017849	0.316	0.158					
cg02956248	6	32,120,901	2.24E-13	0.316	0.325				*PPT2*	TSS1500
cg05227549	12	1,770,782	0.010353	0.316	0.229					
cg07017597	15	32,983,650	1.19E-12	0.316	0.308				*SCG5*	Body
cg08832603	7	96,652,481	3.98E-07	0.316	0.304				*DLX5*	Body
cg16476975	7	155,164,995	0.000179	0.316	0.296					
cg13974773	6	27,463,217	0.001248	0.316	0.367					
cg07749485	X	119,124,509	0.001226	0.316	0.393					
cg16158863	13	32,605,218	0.002246	0.316	0.149				*FRY*	TSS1500
cg24212268	10	29,936,149	1.12E-08	0.316	0.259				*SVIL*	5'UTR
cg24188415	10	119,304,586	0.039151	0.316	0.316				*EMX2*	Body
cg08481491	3	125,900,108	2.3E-15	0.316	0.262				*ALDH1L1*	TSS1500
cg18008019	13	100,641,646	9.46E-12	0.316	0.287					
cg13639936	9	102,586,628	0.001775	0.316	0.205				*NR4A3*	5'UTR
cg20215007	7	149,468,365	2.05E-27	0.316	0.298				*ZNF467*	5'UTR
cg25663755	3	38,035,463	6.79E-05	0.316	0.198				*VILL*	Body
cg00545705	1	208,382,186	5.19E-22	0.316	0.275				*PLXNA2*	Body
cg17621718	3	42,306,737	1.97E-05	0.316	0.314				*CCK*	TSS1500
cg05097861	17	15,820,610	4.48E-18	0.316	0.294					
cg08350776	5	54,516,538	4.68E-09	0.316	0.297					
cg25035631	1	35,586,358	1.22E-07	0.316	0.324					
cg08298946	12	13,301,010	9.26E-06	0.316	0.237					
cg21443699	7	142,637,936	3.96E-17	0.316	0.307				*C7orf34*	3'UTR
cg02921269	14	75,894,323	0.025772	0.316	0.118				*JDP2*	TSS200
cg23684878	12	65,218,260	0.031682	0.316	0.078					
cg12457909	7	39,649,359	1.73E-06	0.316	0.247				*LOC646999*	Body
cg08178940	6	50,808,627	1.65E-08	0.316	0.330				*TFAP2B*	Body

cg00772407	12	89,744,471	3.15E-06	0.316	0.282				*DUSP6*	Body
cg00788028	2	114,049,041	1.95E-16	0.316	0.312				*LOC440839*	Body
cg08091439	1	110,546,703	2.82E-24	0.316	0.323				*AHCYL1*	Body
cg14243778	12	41,086,879	9.25E-07	0.316	0.334				*CNTN1*	5'UTR
cg24550010	1	65,731,107	7.37E-09	0.316	0.280				*DNAJC6*	Body
cg04926881	7	29,024,378	2.67E-06	0.316	0.345					
cg05635754	19	17,958,736	4.56E-06	0.316	0.279				*JAK3*	5'UTR
cg01101344	16	1,973,654	0.000366	0.315	0.288					
cg06132069	20	44,686,038	4.95E-09	0.315	0.299				*SLC12A5*	Body
cg05602356	5	58,334,856	1.06E-15	0.315	0.308				*PDE4D*	Body
cg05587394	2	23,777,246	1.21E-19	0.315	0.318				*KLHL29*	5'UTR
cg25769732	1	92,295,946	4.11E-07	0.315	0.288				*TGFBR3*	Body
cg02453462	15	73,662,002	4.21E-05	0.315	0.374				*HCN4*	TSS1500
cg02721000	12	54,441,213	4.98E-08	0.315	0.280				*HOXC4*	5'UTR
cg15676455	3	128,564,943	0.000124	0.315	0.282					
cg03478249	13	43,566,547	0.000186	0.315	0.309				*EPSTI1*	TSS200
cg26474043	5	148,442,857	2.12E-17	0.315	0.311				*SH3TC2*	TSS200
cg10322504	20	3,229,239	1.26E-09	0.315	0.301					
cg02986266	7	20,833,552	0.01896	0.315	0.353					
cg06183338	5	178,004,204	1.03E-11	0.315	0.324				*COL23A1*	Body
cg02058358	14	76,819,107	1.07E-10	0.315	0.225					
cg25927215	6	34,677,450	1.59E-16	0.315	0.324					
cg11154552	7	87,848,242	0.005669	0.315	0.260				*SRI*	Body
cg20233073	4	54,960,226	1.03E-12	0.315	0.285					
cg03400828	6	44,310,324	2.92E-05	0.315	0.365				*SPATS1*	TSS200
cg06796713	2	66,664,871	0.001663	0.315	0.389				*MEIS1*	Body
cg14663984	1	969,042	0.000729	0.315	0.260				*AGRN*	Body
cg03738247	X	119,134,526	1.46E-06	0.315	0.277					
cg04588840	20	2,781,685	2.05E-05	0.315	0.234				*CPXM1*	TSS1500
cg16835233	22	22,862,479	3.02E-09	0.315	0.197				*ZNF280B*	5'UTR
cg25747192	3	50,378,423	1.01E-06	0.315	0.311				*RASSF1*	TSS200
cg21039708	14	57,278,729	0.027461	0.315	0.368				*OTX2OS1*	TSS1500
cg25832771	8	72,756,058	0.001059	0.315	0.365				*MSC*	1stExon
cg01888601	11	47,736,182	1.14E-11	0.315	0.261	3.03E-27	0.195		*AGBL2*	1stExon

cg06714180	12	54,409,525	0.001098	0.315	0.313				*HOXC4*	TSS1500
cg18145505	15	33,009,498	0.000838	0.315	0.269	2.53E-24	0.300		*GREM1*	TSS1500
cg19843891	8	48,675,647	3.18E-06	0.315	0.257					
cg11827910	11	31,825,756	0.017518	0.315	0.277				*PAX6*	Body
cg25971727	12	81,471,533	1.87E-05	0.315	0.304				*ACSS3*	TSS1500
cg08662017	11	12,031,093	0.000346	0.315	0.279				*DKK3*	TSS1500
cg05901357	2	27,529,325	4.03E-17	0.315	0.298				*TRIM54*	Body
cg08472142	1	155,007,166	1.01E-07	0.315	0.244				*DCST1*	Body
cg06736785	7	132,260,175	5.23E-12	0.315	0.268				*PLXNA4*	5'UTR
cg05143887	12	81,471,938	0.0102	0.315	0.337				*ACSS3*	1stExon
cg02305765	12	53,107,910	5.78E-22	0.315	0.258					
cg05847960	20	10,152,774	0.020498	0.315	0.163					
cg08278108	17	48,042,917	1.62E-11	0.315	0.321					
cg09969043	15	96,868,971	5.54E-07	0.315	0.258				*NR2F2*	TSS200
cg10332649	2	149,932,635	5.81E-15	0.315	0.306				*LYPD6B*	5'UTR
cg14360865	18	59,560,371	4.96E-08	0.315	0.250				*RNF152*	TSS200
cg18809126	3	11,623,526	8.36E-07	0.315	0.272				*VGLL4*	Body
cg06087421	1	47,695,138	0.002252	0.315	0.336				*TAL1*	5'UTR
cg06673178	X	50,557,022	0.027205	0.315	0.392				*SHROOM4*	5'UTR
cg07201456	16	75,528,813	0.005301	0.315	0.292				*CHST6*	5'UTR
cg21480420	X	119,445,407	1.34E-15	0.315	0.314				*FAM70A*	TSS200
cg06127885	15	45,421,068	7.6E-26	0.315	0.314				*DUOX1*	TSS1500
cg03891590	10	21,814,313	1.17E-17	0.315	0.268				*C10orf140*	5'UTR
cg24030507	5	139,089,274	5.03E-05	0.315	0.342					
cg02388150	8	41,165,699	0.000427	0.315	0.160	1.39E-14	0.184		*SFRP1*	Body
cg26613811	7	150,054,588	3.37E-19	0.315	0.301					
cg01110759	16	56,672,387	2.25E-07	0.315	0.296				*MT1A*	TSS200
cg09076334	13	24,477,870	1.95E-10	0.315	0.331					
cg06815411	4	57,737,651	6.49E-11	0.315	0.325					
cg16407699	10	74,020,428	2.77E-12	0.315	0.304					
cg21593030	11	70,952,175	0.001458	0.315	0.304					
cg22395765	7	157,362,094	6.1E-18	0.315	0.317				*PTPRN2*	Body
cg16440629	17	44,896,147	3.46E-07	0.315	0.336				*WNT3*	TSS200
cg17616554	7	117,119,611	7.72E-07	0.315	0.303				*CFTR*	TSS1500

cg12975230	18	73,167,671	0.01319	0.315	0.096					
cg07189401	11	19,736,333	2.66E-06	0.315	0.309				*LOC100126784*	TSS200
cg08690459	11	47,279,586	6.52E-23	0.315	0.299				*NR1H3*	1stExon
cg06320380	2	218,770,208	3.2E-16	0.315	0.307				*TNS1*	5'UTR
cg21679391	1	1,286,917	2.27E-27	0.315	0.288					
cg07841815	7	100,318,223	0.001083	0.315	0.196				*EPO*	TSS200
cg13398947	6	30,418,494	2.34E-07	0.315	0.295					
cg27347290	6	31,831,439	2.68E-06	0.315	0.301				*NEU1*	TSS1500
cg09292244	8	8,182,618	9.47E-20	0.315	0.304				*PRAGMIN*	Body
cg13036381	3	159,942,495	8.13E-07	0.315	0.280				*LOC401097*	TSS1500
cg20930202	6	139,613,456	7.75E-10	0.315	0.328				*TXLNB*	TSS1500
cg00956964	18	12,420,862	2.75E-13	0.315	0.266				*SLMO1*	Body
cg19744936	19	15,343,395	0.001771	0.315	0.349				*EPHX3*	5'UTR
cg19623012	11	116,371,330	0.000974	0.315	0.275					
cg22884656	2	45,157,296	0.001221	0.315	0.318					
cg17012202	6	31,830,656	0.042954	0.315	0.285				*NEU1*	5'UTR
cg05741937	12	104,609,797	3.81E-07	0.315	0.314				*TXNRD1*	Body
cg16065922	7	71,912,239	0.000143	0.315	0.370					
cg18427787	20	45,087,990	0.00341	0.315	0.132					
cg11601252	15	68,122,139	0.006896	0.314	0.315				*LBXCOR1*	Body
cg25129414	X	152,848,639	1.89E-19	0.314	0.308					
cg06644669	6	30,653,799	8.98E-10	0.314	0.317				*KIAA1949*	5'UTR
cg13611347	18	31,739,207	0.018418	0.314	0.382				*NOL4*	Body
cg06511330	1	234,635,574	2.6E-19	0.314	0.293					
cg15298059	7	157,668,141	5.02E-09	0.314	0.243				*PTPRN2*	Body
cg17175208	1	6,241,179	0.000276	0.314	0.222				*CHD5*	TSS1500
cg14009098	1	234,041,306	3.59E-08	0.314	0.278				*SLC35F3*	Body
cg08161323	4	74,734,762	0.000538	0.314	0.222				*CXCL1*	TSS1500
cg17406248	9	97,807,693	9.57E-16	0.314	0.300				*C9orf3*	Body
cg16281276	11	7,695,528	0.001463	0.314	0.251				*CYB5R2*	TSS1500
cg18135771	3	13,323,959	6.8E-06	0.314	0.348					
cg06890747	7	39,649,290	0.000605	0.314	0.276				*LOC646999*	Body
cg09715059	6	32,223,336	6.76E-08	0.314	0.308					
cg13651833	9	71,788,748	1.69E-19	0.314	0.325				*TJP2*	TSS1500

cg19873536	10	116,475,283	1.74E-17	0.314	0.277					
cg20288341	6	31,683,131	6.99E-20	0.314	0.300				*LY6G6E*	TSS1500
cg12841929	X	50,557,522	1.12E-06	0.314	0.310				*SHROOM4*	TSS1500
cg08351336	5	71,014,820	2.55E-07	0.314	0.276				*CARTPT*	TSS200
cg08571859	5	112,073,350	0.005412	0.314	0.145				*APC*	TSS1500
cg24006126	1	155,290,813	1.19E-07	0.314	0.275				*RUSC1*	5'UTR
cg14661148	5	71,852,862	0.018578	0.314	0.237					
cg15068733	17	7,342,846	1.19E-06	0.314	0.189	2.9E-11	0.150		*FGF11*	1stExon
cg16854986	4	77,872,417	0.038517	0.314	0.096				*SEPT11*	Body
cg13186286	14	91,580,451	0.012713	0.314	0.142				*C14orf159*	1stExon
cg21462428	12	4,381,448	0.000211	0.314	0.306				*CCND2*	TSS1500
cg21251140	2	181,544,642	3.15E-06	0.314	0.255					
cg16448636	7	21,209,509	1.2E-15	0.314	0.299					
cg00301159	3	157,260,788	4.55E-07	0.314	0.335				*C3orf55*	TSS1500
cg23207676	19	41,073,603	0.004289	0.314	0.217				*SPTBN4*	Body
cg17282004	11	47,615,333	2.93E-10	0.314	0.260				*C1QTNF4*	5'UTR
cg00901051	1	208,132,490	0.000761	0.314	0.293					
cg22491548	7	128,000,861	0.002414	0.314	0.325				*PRRT4*	5'UTR
cg05490712	11	31,838,674	0.000493	0.314	0.355				*PAX6*	5'UTR
cg20024850	X	138,774,368	0.026947	0.314	0.349				*MCF2*	5'UTR
cg23965061	8	22,960,599	0.005867	0.314	0.268				*TNFRSF10C*	5'UTR
cg26120958	11	6,439,806	1.2E-06	0.314	0.188				*APBB1*	5'UTR
cg07537562	7	51,539,514	5.38E-08	0.314	0.275					
cg09340741	X	46,432,858	3.91E-05	0.314	0.308				*CHST7*	TSS1500
cg13768137	2	177,039,426	1.26E-09	0.314	0.301					
cg07197059	14	23,836,012	5.51E-15	0.314	0.314	4.76E-23	0.172		*EFS*	TSS1500
cg11736020	6	30,651,511	2.4E-06	0.314	0.348				*KIAA1949*	Body
cg23931734	15	63,673,013	2.69E-13	0.314	0.311				*CA12*	Body
cg17913386	3	48,632,582	0.000188	0.314	0.309				*COL7A1*	1stExon
cg21929473	14	51,862,102	7E-21	0.314	0.316					
cg26830038	2	219,846,925	4.15E-29	0.314	0.324				*FEV*	Body
cg03607179	9	139,236,708	7.16E-24	0.314	0.300				*GPSM1*	Body
cg25943118	11	121,260,362	1.53E-12	0.314	0.321					
cg20638675	10	45,374,906	0.028467	0.314	0.176					

cg21817737	11	89,867,704	3.95E-05	0.314	0.386				*NAALAD2*	TSS200
cg02017718	12	54,441,055	1.73E-07	0.314	0.258				*HOXC4*	5'UTR
cg08313757	2	97,428,708	1.76E-08	0.314	0.235				*CNNM4*	Body
cg23647143	X	9,432,556	0.009625	0.314	0.296				*TBL1X*	5'UTR
cg03848381	15	40,575,645	0.000621	0.314	0.220					
cg05924540	4	140,200,723	0.000366	0.314	0.253				*C4orf49*	Body
cg27305383	4	57,522,795	1.78E-08	0.314	0.300				*HOPX*	5'UTR
cg22579265	10	94,180,383	1.19E-06	0.314	0.250					
cg21708058	8	38,585,602	4.67E-12	0.314	0.315				*TACC1*	TSS200
cg24505687	6	33,548,425	1.01E-05	0.314	0.242				*BAK1*	TSS1500
cg02256049	11	115,531,001	1.52E-08	0.314	0.287					
cg23473396	X	25,041,134	0.015795	0.313	0.331					
cg05360958	12	15,038,440	3.49E-07	0.313	0.300				*MGP*	Body
cg20657849	6	28,557,400	0.004444	0.313	0.269					
cg04502985	5	77,268,452	0.000297	0.313	0.343					
cg05447008	6	73,331,114	0.044984	0.313	0.400				*KCNQ5*	TSS1500
cg01349903	7	71,148,142	3.19E-07	0.313	0.270				*WBSCR17*	Body
cg21581873	1	204,329,307	2.85E-09	0.313	0.321	5.7E-38	0.236		*PLEKHA6*	TSS1500
cg07041883	5	76,476,158	0.008648	0.313	0.296					
cg18602913	19	16,022,934	2.28E-06	0.313	0.274					
cg15661753	12	49,627,010	7.51E-05	0.313	0.364					
cg23836904	2	25,599,488	0.000146	0.313	0.232					
cg04285275	16	31,549,141	1.81E-06	0.313	0.408					
cg00913949	1	217,311,511	2.23E-07	0.313	0.312				*ESRRG*	TSS1500
cg19610905	11	61,596,333	0.011827	0.313	0.433				*FADS2*	Body
cg18793215	1	156,674,685	1.71E-10	0.313	0.262				*CRABP2*	Body
cg02142483	16	84,560,555	3.11E-05	0.313	0.284					
cg03061435	1	205,537,833	2.19E-07	0.313	0.245				*MFSD4*	TSS1500
cg25528646	X	151,143,302	0.011558	0.313	0.411				*GABRE*	TSS200
cg10990170	6	29,974,521	1.9E-14	0.313	0.317				*HLA-J*	Body
cg15031661	1	240,256,603	0.024152	0.313	0.319				*FMN2*	1stExon
cg16965552	1	48,937,016	6.16E-20	0.313	0.304				*SPATA6*	Body
cg06825512	18	10,453,969	3.15E-22	0.313	0.304				*APCDD1*	TSS1500
cg01749753	21	38,580,812	5.64E-09	0.313	0.245				*DSCR9*	TSS200

cg05854114	12	77,719,408	0.008974	0.313	0.259					
cg23723410	1	59,486,037	5.45E-07	0.313	0.301					
cg07687766	7	129,421,353	0.007835	0.313	0.321					
cg16661628	20	32,857,227	0.000174	0.313	0.210					
cg22381068	1	906,363	1.01E-09	0.313	0.342				*PLEKHN1*	Body
cg02210123	14	63,670,835	0.029977	0.313	0.143	0.00017193	0.051		*RHOJ*	TSS1500
cg04474049	7	116,164,533	1.94E-09	0.313	0.228				*CAV1*	TSS1500
cg22450968	7	156,871,234	3E-05	0.313	0.307					
cg23586322	8	121,138,232	0.004434	0.313	0.377				*COL14A1*	5'UTR
cg06607384	14	91,579,859	6.99E-10	0.313	0.279				*C14orf159*	TSS1500
cg20931973	6	110,679,832	9.26E-07	0.313	0.190				*C6orf186*	TSS1500
cg21579399	2	11,890,700	8.51E-13	0.313	0.300				*LPIN1*	5'UTR
cg01635061	6	33,160,234	5.03E-05	0.313	0.302				*COL11A2*	5'UTR
cg23244913	6	29,943,401	1.19E-07	0.313	0.314	9.08E-12	0.192		*HCG9*	Body
cg08269485	11	1,769,382	0.035268	0.313	0.199				*HCCA2*	Body
cg12136608	3	3,840,360	0.01881	0.313	0.074				*LRRN1*	TSS1500
cg10777851	3	112,051,808	0.001039	0.313	0.424	5.02E-19	0.138		*CD200*	TSS200
cg25340966	1	119,532,195	0.001873	0.313	0.384				*TBX15*	TSS200
cg17547524	X	117,629,324	0.044108	0.313	0.241				*DOCK11*	TSS1500
cg12439325	12	51,236,768	0.00028	0.313	0.222				*TMPRSS12*	1stExon
cg02776664	19	12,978,624	0.002585	0.313	0.349				*MAST1*	Body
cg03443331	2	97,302,668	3.51E-11	0.313	0.316				*KIAA1310*	Body
cg13939204	9	130,667,057	1.51E-17	0.313	0.294					
cg16895261	4	3,391,305	8.77E-21	0.313	0.316				*RGS12*	Body
cg22876812	2	71,116,188	0.000884	0.313	0.286					
cg18704110	2	73,143,534	0.004335	0.313	0.209				*EMX1*	TSS1500
cg12669543	3	9,225,565	2.96E-15	0.313	0.298				*SRGAP3*	Body
cg05036846	1	24,648,984	4.11E-12	0.313	0.335				*GRHL3*	TSS1500
cg12978800	7	151,403,164	3.19E-12	0.313	0.333				*PRKAG2*	Body
cg07317017	15	74,218,715	1.99E-21	0.313	0.297				*LOXL1*	TSS200
cg25513433	11	10,316,555	8.07E-09	0.313	0.235				*SBF2*	TSS1500
cg06679334	17	35,241,844	0.00343	0.313	0.343					
cg13668207	12	65,904,452	3.8E-13	0.313	0.329					
cg10664112	2	149,645,559	9.24E-06	0.313	0.298				*KIF5C*	Body

cg19679123	15	67,430,336	3.1E-19	0.313	0.302				*SMAD3*	Body
cg02147764	2	45,169,681	5.04E-08	0.313	0.317				*SIX3*	1stExon
cg09296957	11	67,418,310	4.78E-13	0.313	0.312				*ACY3*	TSS200
cg18121066	17	80,794,274	9.4E-05	0.313	0.307				*TBCD*	Body
cg03026373	17	36,715,787	0.002833	0.313	0.327				*SRCIN1*	Body
cg25529319	X	154,842,320	0.001446	0.313	0.304				*TMLHE*	5'UTR
cg04843555	5	17,360,014	1.5E-28	0.313	0.299					
cg09266771	12	63,207,138	1.77E-18	0.313	0.310				*PPM1H*	Body
cg03405909	11	31,845,639	0.000346	0.313	0.259					
cg19756790	2	45,176,863	3.48E-05	0.313	0.208					
cg10357657	19	17,958,633	1.12E-06	0.313	0.276				*JAK3*	5'UTR
cg16585682	5	180,017,623	0.001197	0.313	0.260	1.33E-25	0.202		*SCGB3A1*	Body
cg05949640	4	2,627,057	1.14E-24	0.313	0.305				*FAM193A*	TSS200
cg14167596	4	4,862,910	4.44E-07	0.313	0.328	3.27E-13	0.190		*MSX1*	Body
cg21264055	6	30,523,242	2.23E-13	0.313	0.252	6.36E-26	0.250		*GNL1*	Body
cg08892236	1	21,978,698	4.81E-07	0.312	0.280				*RAP1GAP*	5'UTR
cg15100135	17	4,891,775	6.9E-09	0.312	0.226				*INCA1*	Body
cg27549944	1	204,328,843	4.03E-22	0.312	0.295	2.39E-25	0.160		*PLEKHA6*	5'UTR
cg06453916	16	29,690,524	5.44E-15	0.312	0.331				*QPRT*	1stExon
cg06060835	6	31,913,809	0.000352	0.312	0.325				*CFB*	1stExon
cg05391318	1	200,011,684	0.023004	0.312	0.372				*NR5A2*	Body
cg24847366	12	125,034,283	7E-45	0.312	0.309					
cg00324161	3	156,009,680	7.3E-06	0.312	0.281				*KCNAB1*	Body
cg20114732	6	166,579,135	1.77E-06	0.312	0.280				*T*	Body
cg19766441	4	25,657,365	0.005861	0.312	0.175				*SLC34A2*	TSS200
cg21687563	22	28,838,019	2.33E-06	0.312	0.297				*TTC28*	Body
cg25076459	7	96,655,889	5.3E-10	0.312	0.263					
cg23255964	5	138,862,353	5.72E-07	0.312	0.291				*TMEM173*	TSS200
cg26073933	1	156,390,102	4.02E-07	0.312	0.323				*C1orf61*	5'UTR
cg09581551	6	107,810,162	0.000532	0.312	0.180				*SOBP*	TSS1500
cg04631202	1	25,942,460	0.002073	0.312	0.198	4.66E-24	0.209		*MAN1C1*	TSS1500
cg23255934	2	151,025,188	3.44E-07	0.312	0.266					
cg14871601	7	64,030,039	1.15E-08	0.312	0.320					
cg20014822	11	69,517,103	1.28E-05	0.312	0.293				*FGF19*	Body

cg01753263	5	171,879,957	4.6E-17	0.312	0.312				*SH3PXD2B*	Body
cg09256201	2	43,019,484	8.97E-10	0.312	0.201				*HAAO*	Body
cg10111629	1	113,067,124	1.44E-24	0.312	0.296				*ST7L*	3'UTR
cg23999170	1	115,628,111	4.3E-07	0.312	0.312				*TSPAN2*	Body
cg21942082	5	80,690,225	3.13E-20	0.312	0.288	9.9E-18	0.155		*RNU5E*	Body
cg13308137	11	47,528,955	8.24E-18	0.312	0.294				*CUGBP1*	5'UTR
cg13945224	9	98,111,970	2.05E-05	0.312	0.303					
cg05937737	3	170,303,540	6.32E-11	0.312	0.301				*SLC7A14*	5'UTR
cg25127852	22	50,523,703	2.11E-08	0.312	0.302				*MLC1*	5'UTR
cg19601636	22	20,001,066	9.16E-19	0.312	0.295				*ARVCF*	5'UTR
cg12434571	17	4,511,132	1.15E-07	0.312	0.265				*SMTNL2*	3'UTR
cg05486035	16	56,697,088	0.001303	0.312	0.338					
cg05994850	6	30,095,341	4.29E-17	0.312	0.290					
cg14167389	1	32,708,250	7.36E-23	0.312	0.305				*MTMR9L*	TSS1500
cg04918175	1	53,527,424	1.68E-16	0.312	0.281				*PODN*	TSS1500
cg16735495	7	105,690,812	2.86E-15	0.312	0.299					
cg02737619	1	2,222,440	2.84E-08	0.312	0.315				*SKI*	Body
cg23918923	7	134,144,118	0.000174	0.312	0.175				*AKR1B1*	TSS1500
cg00446414	8	29,387,350	0.000128	0.312	0.288					
cg22626659	3	52,334,181	1.16E-08	0.312	0.275					
cg11651220	1	20,512,944	1.84E-06	0.312	0.287				*UBXN10*	5'UTR
cg14759043	12	3,862,597	0.001348	0.312	0.225	1.43E-11	0.129		*EFCAB4B*	TSS1500
cg13740187	1	154,164,699	8.7E-09	0.312	0.313				*TPM3*	TSS200
cg19839655	20	44,746,499	0.00117	0.312	0.428				*CD40*	TSS1500
cg06242243	12	124,992,461	1.19E-15	0.312	0.322				*NCOR2*	5'UTR
cg14176966	12	2,944,286	0.001543	0.312	0.175				*NRIP2*	TSS200
cg04151062	1	116,710,943	0.000141	0.312	0.271					
cg04276508	6	50,819,054	9.99E-07	0.312	0.309					
cg12985929	17	75,370,611	5.7E-06	0.312	0.241				*SEPT9*	5'UTR
cg19766820	5	783,220	1.37E-08	0.312	0.296					
cg09161412	17	18,057,145	0.028263	0.312	0.240				*MYO15A*	Body
cg01340991	6	33,397,233	5.78E-22	0.312	0.301				*SYNGAP1*	Body
cg09603829	2	54,054,349	1.67E-14	0.312	0.303				*LOC100302652*	Body
cg08829299	11	288,305	0.00067	0.312	0.258				*ATHL1*	TSS1500

cg09775533	20	32,857,151	0.000118	0.312	0.210					
cg04001842	15	45,406,939	1.99E-06	0.312	0.310	5.59E-20	0.181		*DUOXA2*	1stExon
cg00499837	11	119,542,542	7.5E-18	0.312	0.291				*PVRL1*	Body
cg18840956	5	95,769,005	0.031877	0.312	0.271				*PCSK1*	TSS200
cg00537210	2	23,786,588	2.78E-14	0.312	0.313				*KLHL29*	Body
cg19968840	15	45,409,319	8.22E-09	0.312	0.316				*DUOXA2*	Body
cg04916091	19	17,958,647	8.63E-05	0.312	0.274				*JAK3*	5'UTR
cg03154690	5	59,188,920	1.14E-16	0.312	0.298				*PDE4D*	Body
cg07090980	17	873,255	8.59E-14	0.312	0.286				*NXN*	Body
cg19124509	4	87,281,412	3.69E-10	0.312	0.309				*MAPK10*	TSS200
cg18036081	22	29,605,147	4.19E-23	0.311	0.279				*EMID1*	Body
cg18390610	17	48,278,927	0.001645	0.311	0.200				*COL1A1*	1stExon
cg02481934	6	26,271,193	1.78E-08	0.311	0.306				*HIST1H3G*	3'UTR
cg05571820	4	5,709,858	0.000558	0.311	0.241				*EVC2*	5'UTR
cg25694349	12	133,065,906	0.040325	0.311	0.311				*FBRSL1*	TSS1500
cg00482488	17	70,216,332	0.000511	0.311	0.323					
cg12252090	8	49,833,279	4.6E-07	0.311	0.327				*SNAI2*	Body
cg21450228	3	23,727,711	2.86E-17	0.311	0.312					
cg25587431	3	177,542,099	8.74E-06	0.311	0.342					
cg25650256	5	171,538,557	5.24E-08	0.311	0.295				*STK10*	Body
cg02979711	6	29,855,338	3.27E-22	0.311	0.287				*HLA-H*	TSS200
cg05636175	8	22,960,397	0.002504	0.311	0.218	6.96E-24	0.227		*TNFRSF10C*	TSS200
cg13578841	15	57,719,922	7.47E-40	0.311	0.288				*CGNL1*	5'UTR
cg25542041	9	124,982,087	1.03E-05	0.311	0.339				*LHX6*	Body
cg00747975	14	77,333,987	4.31E-05	0.311	0.291				*C14orf166B*	Body
cg02003184	19	51,324,465	1.86E-06	0.311	0.289				*KLK1*	Body
cg09442613	12	76,054,089	0.000683	0.311	0.332					
cg21944491	19	41,119,829	0.006111	0.311	0.170			Yes	*LTBP4*	Body
cg03913921	7	120,496,827	2.47E-13	0.311	0.261				*TSPAN12*	5'UTR
cg07717632	9	140,172,631	2.67E-11	0.311	0.298	1.73E-16	0.154		*C9orf167*	5'UTR
cg15965716	1	164,688,315	5.38E-28	0.311	0.307				*PBX1*	Body
cg02753187	17	46,919,180	1E-11	0.311	0.292				*CALCOCO2*	Body
cg20299703	1	65,731,886	1.59E-08	0.311	0.266				*DNAJC6*	Body
cg26230651	2	114,048,973	1.14E-08	0.311	0.279				*LOC440839*	Body

cg09137696	16	56,672,415	1.76E-05	0.311	0.271				*MT1A*	TSS200
cg03239552	12	54,339,653	0.000328	0.311	0.343				*HOXC13*	3'UTR
cg20388732	17	40,439,433	7.3E-08	0.311	0.278				*STAT5A*	TSS200
cg16875629	19	36,389,575	0.012566	0.311	0.195				*NFKBID*	5'UTR
cg11064454	8	74,363,469	2.54E-21	0.311	0.283				*STAU2*	Body
cg25888700	X	21,676,344	0.034244	0.311	0.336				*KLHL34*	5'UTR
cg20618441	19	53,039,444	3.43E-06	0.311	0.313				*ZNF808*	5'UTR
cg26677584	13	28,541,468	0.000345	0.311	0.227				*CDX2*	Body
cg10244368	19	53,039,145	2.44E-06	0.311	0.311				*ZNF808*	5'UTR
cg26232553	8	101,471,097	4.26E-07	0.311	0.302					
cg03738352	4	25,657,381	0.003745	0.311	0.175				*SLC34A2*	TSS200
cg10351914	17	19,290,690	4.58E-39	0.311	0.304				*MFAP4*	TSS200
cg20027133	11	35,547,514	1.02E-13	0.311	0.300				*PAMR1*	TSS1500
cg20885179	3	38,035,831	0.046173	0.311	0.120				*VILL*	Body
cg14019323	12	65,218,413	0.041103	0.311	0.064					
cg00237268	4	7,287,615	1.35E-05	0.311	0.241				*SORCS2*	Body
cg11605903	6	78,176,622	0.006148	0.311	0.320					
cg00388195	7	96,647,323	3.27E-09	0.311	0.306					
cg20911718	14	24,867,491	1.91E-30	0.311	0.300				*NYNRIN*	TSS1500
cg26567163	10	66,268,067	9.68E-07	0.311	0.368					
cg12317456	16	55,512,868	3.37E-06	0.311	0.277	3.54E-24	0.199		*MMP2*	TSS1500
cg10986455	X	69,642,862	2.7E-27	0.311	0.299				*GDPD2*	TSS200
cg05548912	17	40,464,318	0.010118	0.311	0.388					
cg11448683	1	45,274,099	1.27E-06	0.311	0.305				*TCTEX1D4*	TSS1500
cg18276112	7	4,755,032	3.63E-27	0.311	0.300				*FOXK1*	Body
cg22763593	2	135,125,439	5.09E-16	0.311	0.313			Yes	*MGAT5*	Body
cg04124352	5	33,892,429	0.000151	0.311	0.344				*ADAMTS12*	TSS1500
cg07200348	13	36,045,201	1.5E-06	0.311	0.341				*NBEA*	Body
cg01791371	6	50,817,193	6.3E-05	0.311	0.365					
cg16590821	X	118,107,876	0.046147	0.311	0.409				*LONRF3*	TSS1500
cg16933386	10	124,906,011	1.36E-06	0.311	0.319					
cg24279017	12	11,877,740	2.81E-13	0.311	0.312			Yes	*ETV6*	Body
cg19007269	10	105,420,501	5.13E-09	0.311	0.239				*SH3PXD2A*	Body
cg07810726	5	158,524,649	0.015569	0.311	0.363				*EBF1*	Body

cg13419869	1	200,863,686	6.64E-08	0.311	0.343				*C1orf106*	TSS1500
cg17766007	19	51,522,921	2.85E-07	0.311	0.339				*KLK10*	5'UTR
cg07451886	16	3,097,405	2.49E-10	0.311	0.298				*MMP25*	Body
cg15965190	2	208,370,032	3.46E-12	0.311	0.317					
cg00514407	2	224,904,626	2.34E-05	0.311	0.215	5.66E-29	0.264		*SERPINE2*	TSS1500
cg22149516	5	101,632,314	2.06E-09	0.311	0.280				*SLCO4C1*	TSS200
cg27345757	3	123,602,795	1.85E-06	0.311	0.284				*MYLK*	5'UTR
cg17053251	10	43,429,459	1.54E-07	0.311	0.326					
cg24476033	19	6,710,658	0.000278	0.311	0.349				*C3*	Body
cg01034993	15	68,992,751	2.32E-20	0.310	0.293				*CORO2B*	Body
cg01486558	3	42,132,870	7.32E-12	0.310	0.297				*TRAK1*	1stExon
cg05669418	2	177,022,962	0.001666	0.310	0.311					
cg06588556	11	18,477,299	5.78E-07	0.310	0.248				*LDHAL6A*	TSS200
cg17858328	1	92,952,521	5.97E-06	0.310	0.280				*GFI1*	TSS1500
cg25099516	1	48,288,813	1.47E-19	0.310	0.337					
cg11825059	7	14,876,371	1.64E-17	0.310	0.297				*DGKB*	Body
cg04436755	15	74,218,767	5.05E-16	0.310	0.296				*LOXL1*	TSS200
cg23387401	17	4,582,204	3.64E-14	0.310	0.313				*PELP1*	Body
cg15719255	19	18,508,620	9.13E-12	0.310	0.294				*LRRC25*	TSS1500
cg18061197	8	101,117,958	1.53E-16	0.310	0.301				*RGS22*	Body
cg27549720	1	19,992,167	1.36E-06	0.310	0.303				*HTR6*	5'UTR
cg10377903	10	101,290,699	0.015413	0.310	0.389					
cg15374435	13	80,911,692	7.66E-06	0.310	0.292				*SPRY2*	Body
cg19713460	22	39,745,530	4.34E-22	0.310	0.284				*SYNGR1*	TSS1500
cg24080793	17	62,097,951	0.000456	0.310	0.279				*ICAM2*	5'UTR
cg00091953	4	143,326,541	0.035252	0.310	0.193				*INPP4B*	Body
cg09486778	2	223,169,609	9.9E-05	0.310	0.351				*CCDC140*	3'UTR
cg14780837	22	39,760,267	2.71E-15	0.310	0.321				*SYNGR1*	Body
cg16202470	17	8,907,213	1.37E-06	0.310	0.301					
cg23330006	12	106,507,713	2.22E-09	0.310	0.273				*NUAK1*	Body
cg25120326	3	13,591,084	3.05E-10	0.310	0.298				*FBLN2*	5'UTR
cg06776898	15	73,611,079	0.001607	0.310	0.338					
cg07775292	22	30,720,496	2.28E-10	0.310	0.330				*TBC1D10A*	Body
cg24596132	15	89,456,136	4.55E-05	0.310	0.243				*MFGE8*	Body

cg07891531	16	3,221,328	0.000422	0.310	0.357					
cg23965931	4	184,828,386	2.84E-07	0.310	0.314				*STOX2*	Body
cg25741533	19	5,829,199	1.83E-07	0.310	0.216					
cg12726120	1	244,892,984	1.43E-07	0.310	0.243					
cg17140469	8	18,238,381	8.16E-10	0.310	0.301					
cg18703238	13	108,922,361	8.86E-07	0.310	0.276				*TNFSF13B*	1stExon
cg13430464	11	74,408,131	1.34E-22	0.310	0.299				*CHRDL2*	Body
cg15648389	12	54,448,769	3.47E-12	0.310	0.273				*HOXC4*	Body
cg03010301	19	52,195,499	5.59E-10	0.310	0.274				*MIR125A*	TSS1500
cg17905084	1	161,697,281	0.000259	0.310	0.302				*FCRLB*	Body
cg27521476	10	102,896,376	1.48E-10	0.310	0.257				*TLX1*	Body
cg20805133	2	242,802,192	2.79E-06	0.310	0.307				*PDCD1*	TSS1500
cg05700129	1	1,935,125	0.000335	0.310	0.344				*KIAA1751*	5'UTR
cg08263589	3	14,852,659	0.004855	0.310	0.345					
cg16115588	12	50,297,588	2.33E-05	0.310	0.210				*FAIM2*	1stExon
cg06721528	1	40,781,824	0.001686	0.310	0.197				*COL9A2*	Body
cg04974290	17	5,001,047	0.000941	0.310	0.322					
cg00368055	4	11,429,689	1.03E-08	0.310	0.315				*HS3ST1*	5'UTR
cg08965276	16	1,030,586	1.79E-19	0.310	0.297				*SOX8*	TSS1500
cg05204123	17	1,613,017	1.29E-05	0.310	0.213				*TLCD2*	Body
cg16405026	2	145,281,942	0.017808	0.310	0.343					
cg14291663	9	135,454,951	2.68E-09	0.310	0.300					
cg26596719	7	142,986,429	1.45E-22	0.310	0.299				*CASP2*	Body
cg03442712	15	86,292,964	3.17E-37	0.310	0.309					
cg25616777	1	27,645,056	2.17E-06	0.310	0.254					
cg09639151	5	140,810,920	1.17E-06	0.310	0.331				*PCDHGA4*	Body
cg17918556	2	169,372,300	4.95E-16	0.310	0.313				*LASS6*	Body
cg23710748	7	124,431,027	1.83E-05	0.310	0.341					
cg09811860	1	211,803,715	3.92E-24	0.310	0.290					
cg22304255	6	29,975,204	2.31E-13	0.310	0.295				*HLA-J*	Body
cg13376290	17	4,614,420	0.021787	0.310	0.108				*ARRB2*	Body
cg19275261	18	5,630,072	0.004559	0.310	0.372					
cg08760398	X	119,442,971	0.008837	0.310	0.402				*FAM70A*	Body
cg09109450	2	21,022,565	0.000277	0.310	0.274	7.69E-22	0.193		*C2orf43*	5'UTR

cg04528829	12	115,133,294	1.29E-14	0.310	0.292					
cg13564825	19	38,747,201	0.016902	0.310	0.270				*PPP1R14A*	TSS200
cg00037457	17	59,531,744	3.41E-08	0.310	0.307					
cg22336337	8	53,161,949	1.19E-21	0.310	0.306				*ST18*	5'UTR
cg01812599	10	103,043,991	3.92E-06	0.310	0.253					
cg03365751	22	42,828,386	1.39E-17	0.310	0.294				*NFAM1*	1stExon
cg06539694	X	68,348,545	4.38E-08	0.310	0.308					
cg06148685	9	113,800,909	0.003767	0.310	0.227				*LPAR1*	TSS1500
cg03162045	3	142,443,933	0.000324	0.310	0.231				*TRPC1*	Body
cg11019430	10	29,096,857	1.2E-07	0.310	0.319					
cg03162492	3	134,010,471	1.8E-19	0.310	0.312					
cg23621729	1	1,935,445	0.000232	0.310	0.322				*KIAA1751*	TSS200
cg10512875	12	29,936,174	0.001939	0.310	0.238				*TMTC1*	5'UTR
cg16167741	1	41,174,793	0.014847	0.310	0.285				*NFYC*	5'UTR
cg02364610	11	46,317,036	0.000128	0.310	0.237				*CREB3L1*	Body
cg18741404	6	29,894,253	5.49E-08	0.310	0.296				*HCG4P6*	TSS1500
cg26034516	17	76,228,121	0.001239	0.310	0.344				*LOC283999*	Body
cg12071888	1	91,195,159	0.010621	0.310	0.342					
cg19013611	7	157,668,051	9.18E-08	0.310	0.238				*PTPRN2*	Body
cg10701640	17	43,249,399	8.94E-08	0.309	0.299					
cg26165269	17	77,765,514	0.003486	0.309	0.310					
cg09760963	4	154,680,808	0.003424	0.309	0.400				*RNF175*	Body
cg26606256	17	43,974,804	0.000189	0.309	0.287				*MAPT*	5'UTR
cg22921760	14	22,005,368	3.58E-24	0.309	0.318				*SALL2*	TSS200
cg09709472	8	10,586,959	6.36E-15	0.309	0.294				*SOX7*	Body
cg03870138	16	88,717,850	3.39E-08	0.309	0.311				*CYBA*	TSS1500
cg17010100	5	59,188,112	1.86E-13	0.309	0.289				*PDE4D*	Body
cg03717588	11	56,948,018	0.006741	0.309	0.303				*LRRC55*	TSS1500
cg11703796	12	6,055,201	1.24E-10	0.309	0.263				*ANO2*	Body
cg14765959	6	106,429,316	0.001222	0.309	0.312					
cg12611243	5	149,897,230	1.24E-06	0.309	0.277				*NDST1*	5'UTR
cg04844714	8	10,872,604	0.000373	0.309	0.307				*XKR6*	Body
cg14563260	9	113,801,320	1.21E-08	0.309	0.212	7.46E-30	0.321		*LPAR1*	TSS1500
cg06018401	5	71,404,897	5.41E-06	0.309	0.294				*MAP1B*	Body

cg12973941	4	13,545,639	1.57E-06	0.309	0.289				*NKX3-2*	1stExon
cg25203031	1	66,999,813	0.005451	0.309	0.292				*SGIP1*	TSS200
cg00920892	8	37,732,224	1.52E-14	0.309	0.265				*RAB11FIP1*	Body
cg22274539	12	103,696,209	2.41E-05	0.309	0.220				*C12orf42*	Body
cg06580551	3	42,947,481	0.043654	0.309	0.210				*ZNF662*	TSS200
cg22247664	1	110,199,126	2.61E-09	0.309	0.304				*GSTM4*	Body
cg25834415	2	241,758,805	4.76E-05	0.309	0.250				*KIF1A*	5'UTR
cg01907098	21	45,149,141	6.61E-07	0.309	0.236				*PDXK*	Body
cg18586886	6	30,652,399	6.72E-11	0.309	0.316				*KIAA1949*	Body
cg01484686	6	106,258,629	2.69E-07	0.309	0.306					
cg20435725	2	107,103,716	0.020887	0.309	0.102					
cg24434800	1	119,542,295	0.001722	0.309	0.344					
cg11483789	3	122,745,560	2.16E-05	0.309	0.212				*SEMA5B*	5'UTR
cg00575003	1	217,261,949	4.15E-10	0.309	0.254				*ESRRG*	5'UTR
cg01242903	12	89,744,877	2.09E-11	0.309	0.325				*DUSP6*	Body
cg08703857	7	2,653,651	3.57E-14	0.309	0.307				*IQCE*	3'UTR
cg09213964	12	122,668,037	6.05E-08	0.309	0.275				*LRRC43*	5'UTR
cg09515442	X	48,689,942	0.000345	0.309	0.317				*PCSK1N*	Body
cg04243822	17	77,030,221	9.51E-13	0.309	0.283				*C1QTNF1*	5'UTR
cg10113101	7	30,029,743	0.006939	0.309	0.173				*SCRN1*	TSS1500
cg07831351	20	3,229,402	2.07E-06	0.309	0.311					
cg00005847	2	177,029,073	0.000352	0.309	0.242	1.03E-10	0.125		*HOXD3*	5'UTR
cg19006008	19	16,999,768	5.88E-07	0.309	0.266	3.79E-17	0.171		*F2RL3*	TSS200
cg02642117	6	21,033,836	6.52E-19	0.309	0.295				*CDKAL1*	Body
cg10136354	2	73,151,457	0.008343	0.309	0.234				*EMX1*	Body
cg23543318	4	1,195,845	2.12E-06	0.309	0.307				*LOC100130872*	Body
cg15985184	19	51,830,311	0.000233	0.309	0.382				*IGLON5*	Body
cg04738464	2	218,868,754	9.18E-22	0.309	0.292					
cg07844977	6	2,903,699	1.6E-06	0.309	0.277				*SERPINB9*	TSS200
cg19595012	4	81,106,312	0.016182	0.309	0.366				*PRDM8*	TSS200
cg05482942	10	102,899,285	0.000244	0.309	0.295					
cg26340149	11	69,988,497	8.71E-17	0.309	0.302				*ANO1*	Body
cg07095230	17	59,480,730	9.98E-06	0.309	0.260				*TBX2*	Body
cg07193234	15	28,341,783	3.52E-10	0.309	0.282				*OCA2*	5'UTR

cg13337740	17	33,446,298	1.82E-16	0.309	0.314				*RAD51L3*	Body
cg16731240	19	52,391,250	0.003059	0.309	0.283	7.45E-16	0.343		*ZNF577*	TSS200
cg14781778	22	45,608,692	1.33E-07	0.309	0.275				*C22orf9*	Body
cg21737226	8	53,326,668	1.23E-09	0.309	0.339					
cg06339706	19	49,371,285	2.19E-12	0.309	0.303	5.35E-23	0.220		*PLEKHA4*	5'UTR
cg18534077	10	104,628,846	5.67E-12	0.309	0.259				*AS3MT*	TSS1500
cg14001664	2	132,088,801	0.000182	0.309	0.297					
cg03449398	7	92,673,015	2.3E-13	0.309	0.281					
cg15936066	8	42,356,637	3.53E-12	0.309	0.295				*SLC20A2*	5'UTR
cg10259701	15	31,645,503	0.009367	0.309	0.189				*KLF13*	Body
cg01238435	5	141,595,696	0.000736	0.309	0.327					
cg22812684	1	70,035,392	7.21E-06	0.309	0.293					
cg11758283	6	7,998,697	5.49E-17	0.308	0.281					
cg26719062	6	30,095,349	3.2E-14	0.308	0.285					
cg25214310	3	149,934,218	3.11E-09	0.308	0.324					
cg00585901	1	155,035,388	0.015012	0.308	0.180				*EFNA4*	TSS1500
cg01704924	12	54,446,996	7.57E-09	0.308	0.264				*HOXC4*	5'UTR
cg11500797	7	96,652,123	4E-08	0.308	0.296	4.88E-14	0.212		*DLX5*	Body
cg12892471	12	50,616,779	7.42E-24	0.308	0.309				*LIMA1*	Body
cg24472939	4	175,139,486	0.001266	0.308	0.233					
cg06179011	11	843,897	3.52E-17	0.308	0.276				*TSPAN4*	TSS1500
cg07168526	7	116,797,907	3.42E-30	0.308	0.304				*ST7*	Body
cg20333836	1	156,215,418	6.08E-08	0.308	0.329				*PAQR6*	Body
cg10629004	20	21,696,467	2.22E-06	0.308	0.354				*PAX1*	3'UTR
cg07515422	19	40,993,713	6E-07	0.308	0.266				*SPTBN4*	Body
cg04596071	7	2,155,899	5.04E-21	0.308	0.309				*MAD1L1*	Body
cg12414174	12	89,748,895	7.37E-12	0.308	0.288					
cg15724945	1	119,548,527	4.16E-13	0.308	0.269					
cg23623270	17	42,402,962	6.53E-09	0.308	0.302				*SLC25A39*	TSS1500
cg10959198	15	93,632,896	1.23E-05	0.308	0.325				*RGMA*	TSS1500
cg26004759	4	141,489,018	3.97E-06	0.308	0.270				*UCP1*	Body
cg25666433	6	28,367,279	0.000503	0.308	0.301				*ZSCAN12*	Body
cg00852846	2	85,664,713	7.94E-05	0.308	0.207					
cg02366320	16	88,967,522	4.83E-70	0.308	0.309				*CBFA2T3*	Body

cg10629165	5	66,124,563	0.001511	0.308	0.133				*MAST4*	TSS200
cg10974980	15	74,243,781	5.07E-05	0.308	0.280				*LOXL1*	Body
cg06231244	1	2,460,761	2E-09	0.308	0.297				*HES5*	3'UTR
cg25199322	1	151,693,838	0.000192	0.308	0.292				*C1orf230*	TSS200
cg24934400	4	4,873,966	0.041793	0.308	0.176					
cg26064774	4	6,666,009	0.000311	0.308	0.332					
cg21821833	3	42,306,493	0.000295	0.308	0.349				*CCK*	TSS200
cg26091021	2	177,021,826	7.46E-05	0.308	0.285					
cg20164887	22	50,968,250	0.038592	0.308	0.236				*TYMP*	5'UTR
cg01348733	17	33,700,757	0.000289	0.308	0.333				*SLFN11*	TSS200
cg10465866	6	28,574,471	0.00516	0.308	0.275					
cg15093997	10	73,769,828	1.06E-14	0.308	0.290				*CHST3*	3'UTR
cg03395558	11	70,508,329	7.79E-09	0.308	0.298				*SHANK2*	Body
cg05740959	X	49,087,234	0.000589	0.308	0.307				*CACNA1F*	Body
cg06807397	13	44,948,413	8.2E-09	0.308	0.245				*SERP2*	Body
cg11413133	17	21,281,318	2.1E-26	0.308	0.320				*KCNJ12*	5'UTR
cg26772854	3	159,756,793	0.002652	0.308	0.338					
cg03605542	5	59,929,339	8.82E-31	0.308	0.290				*DEPDC1B*	Body
cg07887891	3	33,429,950	2.83E-07	0.308	0.320				*UBP1*	3'UTR
cg09734791	8	72,756,155	0.026751	0.308	0.360				*MSC*	1stExon
cg19831077	7	151,107,924	1.18E-15	0.308	0.321	1.5E-28	0.233		*WDR86*	TSS1500
cg22660207	4	174,320,772	4.34E-10	0.308	0.351				*SCRG1*	TSS200
cg25027125	14	35,182,801	1.19E-15	0.308	0.257				*CFL2*	Body
cg04844574	12	122,028,594	4.89E-06	0.308	0.286					
cg10503083	2	177,003,280	5.4E-05	0.308	0.298					
cg04132540	8	56,851,846	8.38E-41	0.308	0.306				*LYN*	5'UTR
cg26043257	4	15,780,238	0.020871	0.308	0.280	6.29E-18	0.241		*CD38*	1stExon
cg21101743	7	39,649,254	1.66E-05	0.308	0.314				*LOC646999*	Body
cg22289837	8	86,350,278	1.22E-11	0.308	0.327	5.67E-21	0.160		*CA3*	TSS1500
cg12453905	4	154,143,694	0.045814	0.308	0.260				*TRIM2*	Body
cg18499201	17	77,395,887	2.47E-06	0.308	0.275				*HRNBP3*	5'UTR
cg26201811	22	51,111,714	3.05E-09	0.308	0.277				*SHANK3*	TSS1500
cg12700449	17	34,121,837	5.42E-20	0.308	0.310				*MMP28*	Body
cg19513321	2	85,811,432	3.48E-07	0.308	0.303				*VAMP5*	TSS200

cg04184019	4	85,424,288	8.71E-07	0.308	0.282					
cg21416692	1	33,803,805	7.53E-15	0.308	0.298				*PHC2*	5'UTR
cg05858889	7	39,649,224	1.21E-05	0.308	0.345				*LOC646999*	Body
cg07158503	5	135,415,693	1.04E-10	0.308	0.286					
cg05923687	1	63,795,711	2.41E-07	0.308	0.274					
cg05204104	2	235,403,141	1.13E-05	0.308	0.334				*ARL4C*	3'UTR
cg10278318	2	235,276,581	1.9E-11	0.308	0.288					
cg26624026	19	18,416,001	2.57E-09	0.308	0.338					
cg25054580	4	54,976,019	6.57E-10	0.308	0.280					
cg10210369	15	90,197,924	0.000199	0.308	0.225				*KIF7*	5'UTR
cg16913064	17	45,949,878	3.64E-13	0.308	0.254					
cg25451249	11	19,736,241	9.76E-06	0.308	0.307				*LOC100126784*	TSS200
cg23851088	X	40,004,432	6.22E-07	0.308	0.223				*BCOR*	5'UTR
cg17863743	10	100,993,587	8.17E-06	0.308	0.338				*HPSE2*	Body
cg09357934	2	159,651,918	6.4E-10	0.308	0.273				*DAPL1*	1stExon
cg13799919	13	28,212,848	8.5E-14	0.308	0.319				*POLR1D*	Body
cg04105511	12	54,409,207	0.010204	0.308	0.292				*HOXC4*	TSS1500
cg06958829	17	48,546,118	6.41E-09	0.307	0.315				*ACSF2*	Body
cg22916722	14	76,605,056	0.024764	0.307	0.239					
cg10665891	12	117,042,917	0.008883	0.307	0.352					
cg26996569	12	121,829,743	9.87E-08	0.307	0.310					
cg08050748	14	51,562,890	9.4E-08	0.307	0.323				*TRIM9*	TSS1500
cg12022772	2	131,130,468	0.002107	0.307	0.253				*PTPN18*	Body
cg09969806	2	241,758,399	3.37E-08	0.307	0.299				*KIF1A*	5'UTR
cg06315607	22	19,512,084	7.79E-05	0.307	0.322				*CLDN5*	1stExon
cg25499397	3	51,990,575	0.003946	0.307	0.315				*GPR62*	1stExon
cg23460943	12	125,333,358	1.24E-34	0.307	0.288				*SCARB1*	Body
cg12403889	1	11,753,338	1.04E-08	0.307	0.313				*C1orf187*	5'UTR
cg13451127	3	47,050,803	1.42E-14	0.307	0.266				*NBEAL2*	Body
cg25424856	X	50,557,036	0.022073	0.307	0.380				*SHROOM4*	5'UTR
cg25308508	5	3,592,638	1.72E-11	0.307	0.330					
cg02340053	13	36,044,886	0.000172	0.307	0.359				*NBEA*	Body
cg14212504	3	128,564,815	0.010286	0.307	0.374					
cg08983217	2	96,991,149	2.11E-08	0.307	0.275				*ITPRIPL1*	TSS1500

cg19238415	11	32,916,120	1.37E-09	0.307	0.331				*QSER1*	5'UTR
cg10277651	20	623,361	0.002005	0.307	0.264					
cg11909137	18	46,461,202	3.3E-07	0.307	0.285				*SMAD7*	Body
cg14099595	5	145,717,283	0.023178	0.307	0.298				*POU4F3*	TSS1500
cg01781417	18	78,004,045	8.81E-23	0.307	0.282				*PARD6G*	Body
cg01968402	6	137,817,775	0.002464	0.307	0.350					
cg20371266	2	233,390,781	6.22E-07	0.307	0.267				*CHRND*	TSS200
cg24020152	4	154,419,554	3.71E-16	0.307	0.295				*KIAA0922*	Body
cg02345747	3	182,879,754	7.91E-08	0.307	0.227				*LAMP3*	Body
cg04078410	5	77,269,250	0.000177	0.307	0.232					
cg09499136	6	168,975,608	1.01E-17	0.307	0.292				*SMOC2*	Body
cg11509907	2	235,404,686	2.59E-05	0.307	0.204				*ARL4C*	1stExon
cg08954277	4	1,535,673	3.83E-05	0.307	0.313					
cg21575509	3	138,658,704	0.001651	0.307	0.345					
cg23353432	20	44,639,847	7.65E-10	0.307	0.291				*MMP9*	Body
cg02619001	17	47,592,333	4.59E-19	0.307	0.294				*NGFR*	3'UTR
cg23671221	1	44,031,300	0.000005	0.307	0.311				*PTPRF*	Body
cg26271591	2	178,125,956	3.67E-11	0.307	0.272				*NFE2L2*	Body
cg04981492	19	15,218,713	3.93E-06	0.307	0.325	7.12E-29	0.200		*SYDE1*	Body
cg09404551	8	121,136,951	3.88E-07	0.307	0.321				*COL14A1*	TSS1500
cg14276379	9	97,663,142	8.27E-12	0.307	0.306				*C9orf3*	Body
cg01029623	12	122,016,779	4.51E-20	0.307	0.314				*KDM2B*	Body
cg09957386	7	134,144,062	0.001935	0.307	0.198				*AKR1B1*	TSS200
cg09424526	2	223,163,809	0.002161	0.307	0.293				*PAX3*	TSS200
cg13742526	19	2,252,432	0.002189	0.307	0.322				*JSRP1*	Body
cg18172881	3	136,538,801	0.007668	0.307	0.154				*TMEM22*	5'UTR
cg06522768	2	38,265,635	1.05E-05	0.307	0.278				*FAM82A1*	Body
cg14080475	9	117,150,495	2.23E-05	0.307	0.185				*AKNA*	5'UTR
cg02695609	7	124,430,950	0.000155	0.307	0.316					
cg10453365	15	90,039,909	4.74E-09	0.307	0.316	2E-36	0.287		*RHCG*	TSS200
cg22489321	13	100,643,388	2.62E-08	0.307	0.299					
cg01325465	16	85,123,554	5.43E-24	0.307	0.291				*KIAA0513*	3'UTR
cg27413008	16	28,519,597	5.84E-10	0.307	0.306				*IL27*	TSS1500
cg25756241	12	50,221,559	0.026675	0.307	0.162				*NCKAP5L*	5'UTR

cg04623837	6	29,943,414	2.23E-06	0.307	0.317	7.57E-10	0.175		*HCG9*	Body
cg02220078	5	132,386,904	1.32E-20	0.307	0.292				*HSPA4*	TSS1500
cg03778669	6	28,457,246	0.022509	0.307	0.294					
cg13161961	7	120,970,240	0.00275	0.307	0.333				*WNT16*	Body
cg03883555	13	24,007,434	0.014739	0.307	0.185				*SACS*	5'UTR
cg26191447	2	191,656,418	1.17E-05	0.307	0.268					
cg08407114	5	36,690,259	0.002708	0.307	0.104					
cg02873263	14	70,476,756	2.95E-17	0.307	0.300				*SMOC1*	Body
cg06211323	13	109,248,488	1.11E-12	0.307	0.229				*MYO16*	TSS200
cg05404698	6	28,956,247	2.88E-06	0.307	0.299					
cg12863693	15	85,201,151	7.01E-06	0.307	0.259				*NMB*	Body
cg01079658	7	99,155,872	0.000299	0.306	0.199				*ZNF655*	TSS200
cg13467672	17	40,464,625	5.86E-23	0.306	0.310					
cg09175834	12	78,225,315	4.5E-12	0.306	0.310				*NAV3*	1stExon
cg14855519	8	25,898,191	4.23E-10	0.306	0.258				*EBF2*	Body
cg25850808	6	74,405,048	1.33E-12	0.306	0.308				*CD109*	TSS1500
cg09381178	4	106,067,367	5E-05	0.306	0.230				*TET2*	TSS1500
cg18559739	6	4,775,132	0.000901	0.306	0.240				*CDYL*	Body
cg10047572	6	30,711,399	2.93E-21	0.306	0.287				*IER3*	3'UTR
cg01576677	2	45,029,908	0.005077	0.306	0.324					
cg22425474	6	29,818,086	0.000382	0.306	0.325					
cg07870237	5	131,348,431	1.4E-11	0.306	0.253				*ACSL6*	TSS1500
cg24075113	7	100,465,289	3.91E-75	0.306	0.306				*TRIP6*	Body
cg26190890	6	27,107,718	1.13E-08	0.306	0.297				*HIST1H2BK*	3'UTR
cg01534253	7	28,447,591	2.36E-06	0.306	0.269				*CREB5*	5'UTR
cg06483432	4	55,523,233	8.73E-09	0.306	0.332				*KIT*	TSS1500
cg05360477	2	12,959,313	3.85E-06	0.306	0.359					
cg09235539	1	65,730,481	1.04E-07	0.306	0.300				*DNAJC6*	5'UTR
cg07485357	14	24,803,679	2.83E-05	0.306	0.332				*ADCY4*	Body
cg13721134	8	86,350,390	3.07E-06	0.306	0.289				*CA3*	TSS1500
cg17431280	19	47,507,461	0.039382	0.306	0.239				*GRLF1*	3'UTR
cg15753746	2	171,679,591	1.44E-06	0.306	0.314				*GAD1*	Body
cg19042390	6	166,261,046	3.13E-05	0.306	0.178					
cg26279668	4	21,949,853	3.16E-05	0.306	0.208				*KCNIP4*	5'UTR

cg16966315	7	149,112,318	6.72E-08	0.306	0.298					
cg17162576	1	66,999,059	8.71E-05	0.306	0.261				*SGIP1*	TSS1500
cg14480531	17	55,700,565	1.01E-09	0.306	0.258				*MSI2*	Body
cg00893471	2	177,029,921	5.56E-07	0.306	0.276				*HOXD3*	5'UTR
cg20954533	5	71,462,729	1.7E-17	0.306	0.280				*MAP1B*	Body
cg15845792	6	28,175,446	0.000167	0.306	0.233					
cg07727884	17	19,290,503	1.12E-21	0.306	0.296				*MFAP4*	TSS200
cg06764239	1	119,544,772	3.26E-06	0.306	0.296					
cg10604002	2	131,130,131	0.000765	0.306	0.233				*PTPN18*	Body
cg13744039	3	184,475,074	1.46E-15	0.306	0.293					
cg25520679	17	1,959,121	1.95E-05	0.306	0.257				*HIC1*	TSS1500
cg16915316	12	58,237,272	3.25E-12	0.306	0.292				*CTDSP2*	Body
cg19918230	2	219,745,647	4.25E-08	0.306	0.233				*WNT10A*	1stExon
cg25137711	1	48,059,173	3.54E-12	0.306	0.255					
cg18575809	14	21,540,329	4.92E-16	0.306	0.302				*FLJ10357*	Body
cg15364169	6	29,975,080	5.37E-05	0.306	0.332				*HLA-J*	Body
cg04683149	8	134,203,304	1.32E-18	0.306	0.310				*WISP1*	TSS200
cg22510376	5	178,957,930	0.000123	0.306	0.226					
cg27296071	20	30,458,612	0.024111	0.306	0.124				*TTLL9*	5'UTR
cg06652329	12	102,874,566	4.72E-06	0.306	0.334				*IGF1*	TSS200
cg06871074	1	146,551,565	6.87E-14	0.306	0.281					
cg19735698	2	23,729,523	1.59E-07	0.306	0.325				*KLHL29*	5'UTR
cg27357571	21	34,398,226	0.001865	0.306	0.312				*OLIG2*	TSS200
cg05655001	8	94,713,206	0.00028	0.306	0.213				*FAM92A1*	Body
cg25246692	22	37,545,924	1.02E-09	0.306	0.275				*IL2RB*	5'UTR
cg01218619	4	25,090,298	1.06E-05	0.306	0.294					
cg05494467	5	140,892,308	2.89E-10	0.306	0.231				*PCDHGB5*	3'UTR
cg08673716	3	14,852,905	0.002512	0.306	0.383					
cg06181069	1	154,293,494	2.61E-05	0.306	0.328				*AQP10*	TSS200
cg08079908	2	176,997,277	4.69E-07	0.306	0.274					
cg08794157	17	15,164,627	9.17E-09	0.306	0.294				*PMP22*	5'UTR
cg10755235	14	60,974,039	0.000108	0.306	0.276					
cg09491410	5	174,178,884	0.011528	0.306	0.190					
cg15384589	5	54,873,393	2.23E-11	0.306	0.305					

cg00374492	6	42,015,867	0.000652	0.306	0.308				*CCND3*	5'UTR
cg00691123	3	11,632,974	1.16E-10	0.306	0.247				*VGLL4*	Body
cg17100761	7	100,318,080	0.006951	0.306	0.224				*EPO*	TSS1500
cg13801416	7	134,143,919	0.003645	0.306	0.234	1.78E-15	0.259		*AKR1B1*	TSS200
cg03460682	2	219,646,316	0.006513	0.306	0.222				*CYP27A1*	TSS200
cg09687738	X	113,818,934	0.021557	0.305	0.355				*HTR2C*	5'UTR
cg18947951	2	63,285,739	3.5E-06	0.305	0.292					
cg25207248	1	110,672,912	5.01E-15	0.305	0.303					
cg01993208	9	97,808,906	1.48E-13	0.305	0.311				*C9orf3*	Body
cg08781140	17	73,584,617	2.88E-06	0.305	0.305				*MYO15B*	Body
cg17965019	6	27,858,545	3.59E-05	0.305	0.316	1.37E-33	0.316		*HIST1H3J*	1stExon
cg21776583	6	30,923,545	0.000589	0.305	0.341					
cg25465483	3	178,432,932	3.2E-24	0.305	0.294				*KCNMB2*	5'UTR
cg16055185	10	79,150,517	2.76E-10	0.305	0.267				*KCNMA1*	Body
cg06339573	7	157,372,262	1.61E-20	0.305	0.273				*PTPRN2*	Body
cg25457832	8	49,647,579	3.79E-07	0.305	0.240				*EFCAB1*	Body
cg26635219	7	117,119,424	2.04E-06	0.305	0.312				*CFTR*	TSS1500
cg15241074	2	12,860,614	1.53E-10	0.305	0.324				*TRIB2*	Body
cg09607488	8	99,963,657	8.07E-10	0.305	0.276				*OSR2*	Body
cg19142043	3	29,724,842	5.43E-19	0.305	0.307				*RBMS3*	Body
cg22358236	11	62,691,452	2.74E-06	0.305	0.286					
cg00754357	1	53,019,727	4.85E-23	0.305	0.274				*ZCCHC11*	TSS1500
cg08438690	3	52,279,403	3.95E-20	0.305	0.287				*PPM1M*	TSS1500
cg05422957	4	122,873,181	0.004578	0.305	0.155				*TRPC3*	TSS1500
cg20918957	3	50,275,112	1.05E-05	0.305	0.336				*GNAI2*	Body
cg15076145	4	94,748,686	2.62E-05	0.305	0.321				*ATOH1*	TSS1500
cg26104143	4	41,869,579	4.07E-06	0.305	0.260					
cg13519918	12	89,748,985	1.78E-09	0.305	0.289					
cg06093379	17	44,896,080	0.005297	0.305	0.379				*WNT3*	5'UTR
cg12934884	6	28,558,070	6.53E-06	0.305	0.327					
cg03243226	11	105,480,979	4.24E-06	0.305	0.322				*GRIA4*	1stExon
cg27231335	1	95,393,429	0.00496	0.305	0.157				*CNN3*	TSS1500
cg04786857	2	29,033,897	0.006188	0.305	0.204	1.15E-13	0.198		*SPDYA*	5'UTR
cg11195797	13	100,547,331	0.000388	0.305	0.230				*CLYBL*	3'UTR

cg08755040	1	180,124,539	0.021837	0.305	0.257	5.14E-08	0.137		*QSOX1*	Body
cg14255824	9	71,795,999	2.35E-13	0.305	0.302				*TJP2*	Body
cg17982102	18	9,707,896	3.14E-05	0.305	0.254	5.63E-18	0.217		*RAB31*	TSS1500
cg03149560	6	29,796,379	9.91E-05	0.305	0.331				*HLA-G*	Body
cg00483526	12	122,907,647	4.32E-14	0.305	0.274				*CLIP1*	TSS1500
cg23642747	10	105,036,645	5.36E-09	0.305	0.344	1.27E-19	0.187		*INA*	TSS1500
cg15929797	5	172,660,796	1.19E-06	0.305	0.317				*NKX2-5*	Body
cg12592716	7	148,395,181	0.008584	0.305	0.136	1.02E-07	0.082	Yes	*CUL1*	TSS1500
cg00918522	4	175,132,871	0.000116	0.305	0.333					
cg13989852	15	68,260,324	2.56E-18	0.305	0.272					
cg18550262	7	72,812,030	9.91E-23	0.305	0.281					
cg22758454	17	16,570,483	7.78E-06	0.305	0.310					
cg23089825	1	170,630,558	0.000473	0.305	0.313					
cg18181201	1	242,686,709	0.004907	0.305	0.159				*PLD5*	5'UTR
cg09975171	X	154,842,841	6.96E-05	0.305	0.187				*TMLHE*	TSS1500
cg06130360	X	117,633,362	5.56E-08	0.305	0.306				*DOCK11*	Body
cg04701034	14	52,536,175	0.001414	0.305	0.339				*NID2*	TSS1500
cg18034737	4	188,916,865	0.000639	0.305	0.299				*ZFP42*	TSS200
cg25868998	13	95,655,172	0.021958	0.305	0.146					
cg13912573	9	92,293,297	6.62E-29	0.305	0.279				*LOC100129066*	Body
cg15829056	1	54,856,415	2.86E-15	0.305	0.279				*SSBP3*	Body
cg05009389	15	90,208,810	0.043068	0.305	0.202				*PLIN1*	3'UTR
cg08739651	10	51,784,888	9.36E-09	0.305	0.250					
cg24776142	6	16,513,829	0.000114	0.305	0.230				*ATXN1*	5'UTR
cg25950625	5	41,510,116	0.046761	0.305	0.426				*PLCXD3*	Body
cg10146929	6	26,017,939	0.000205	0.305	0.262	7.78E-21	0.258		*HIST1H1A*	1stExon
cg08523343	5	14,872,934	9.39E-05	0.305	0.229				*ANKH*	TSS1500
cg09873164	1	152,488,093	0.020927	0.305	0.316				*CRCT1*	Body
cg18055610	6	30,095,419	7.21E-06	0.305	0.318					
cg23395749	5	176,984,246	0.000102	0.305	0.264					
cg13065206	15	41,062,017	5.36E-13	0.305	0.302				*DNAJC17*	Body
cg21342910	17	48,042,779	4.47E-06	0.305	0.366					
cg22956060	3	9,289,747	4.55E-07	0.305	0.197				*SRGAP3*	Body
cg04829853	15	89,438,208	0.001447	0.305	0.373				*HAPLN3*	5'UTR

cg22008625	8	10,588,386	0.011295	0.305	0.200				*SOX7*	TSS1500
cg03383941	20	44,099,418	0.017366	0.305	0.198				*WFDC2*	Body
cg17399545	12	88,972,226	1.78E-15	0.305	0.288				*KITLG*	Body
cg17800442	16	30,908,233	0.006358	0.305	0.361	1.5E-18	0.228		*CTF1*	Body
cg19870698	14	95,239,570	0.011335	0.305	0.330					
cg09478396	7	140,339,480	5.37E-06	0.305	0.307					
cg04878000	1	32,237,923	3.91E-05	0.305	0.323					
cg12101586	15	75,019,203	1.12E-16	0.305	0.299				*CYP1A1*	TSS1500
cg20039048	11	8,305,302	7.93E-10	0.305	0.306					
cg22222461	8	98,000,885	3.63E-09	0.305	0.282				*PGCP*	Body
cg18235000	8	26,372,829	8.76E-05	0.305	0.257				*PNMA2*	TSS1500
cg22698272	1	221,054,615	4.93E-13	0.305	0.309				*HLX*	Body
cg02713832	11	60,772,559	4.15E-12	0.304	0.278				*CD6*	Body
cg19760241	17	35,300,010	1.92E-05	0.304	0.311				*LHX1*	Body
cg09813276	X	100,805,521	0.043257	0.304	0.162				*ARMCX1*	1stExon
cg04358214	16	67,143,304	3.71E-05	0.304	0.282				*C16orf70*	TSS1500
cg25216704	4	3,485,393	2.23E-10	0.304	0.302				*DOK7*	Body
cg00036011	6	26,271,718	0.007825	0.304	0.330				*HIST1H3G*	TSS200
cg08189989	2	105,459,164	0.025478	0.304	0.401					
cg00184203	2	187,558,402	6.62E-05	0.304	0.213				*FAM171B*	TSS1500
cg01451808	8	49,676,562	3.07E-15	0.304	0.279					
cg27405791	4	41,882,953	0.000555	0.304	0.312					
cg04582364	5	52,778,441	7.34E-05	0.304	0.224				*FST*	Body
cg27365825	2	18,059,130	0.001447	0.304	0.223				*KCNS3*	TSS1500
cg25060829	6	28,367,571	0.002848	0.304	0.282				*ZSCAN12*	TSS200
cg14511698	14	60,976,898	4.74E-08	0.304	0.309				*SIX6*	Body
cg25324047	10	22,765,840	4.05E-08	0.304	0.334					
cg13910046	12	25,538,221	5.73E-05	0.304	0.197					
cg14409958	8	120,651,652	5.84E-05	0.304	0.341	4.2E-18	0.153		*ENPP2*	TSS1500
cg12054869	13	33,001,250	0.001284	0.304	0.196				*N4BP2L1*	Body
cg01287054	7	156,795,156	0.000354	0.304	0.256					
cg13892570	1	159,893,182	1.87E-07	0.304	0.278				*TAGLN2*	5'UTR
cg16405012	12	5,542,395	0.001482	0.304	0.299				*NTF3*	Body
cg22982093	17	41,669,401	8.98E-26	0.304	0.292					

cg18790597	4	17,783,331	0.002252	0.304	0.290				*FAM184B*	TSS200
cg14093715	9	126,776,180	6.54E-08	0.304	0.289				*LHX2*	Body
cg19619003	1	41,846,758	6.6E-05	0.304	0.159					
cg23493412	17	63,491,688	1E-10	0.304	0.287					
cg05725004	1	76,082,568	3.66E-08	0.304	0.234					
cg21872764	22	19,510,977	6.49E-05	0.304	0.317				*CLDN5*	1stExon
cg10445315	21	38,066,047	0.005961	0.304	0.331					
cg23396786	2	73,299,151	0.000958	0.304	0.233				*SFXN5*	TSS200
cg27369013	2	219,472,724	0.002532	0.304	0.238				*PLCD4*	1stExon
cg04138112	1	167,090,618	0.00664	0.304	0.185				*DUSP27*	Body
cg19856273	1	46,668,472	1.58E-14	0.304	0.314				*C1orf190*	TSS1500
cg21096399	11	119,188,145	2.93E-19	0.304	0.299	2.45E-30	0.229		*MCAM*	TSS1500
cg04641171	15	79,381,805	1.04E-05	0.304	0.301				*RASGRF1*	Body
cg08121925	3	180,588,041	6.9E-05	0.304	0.208					
cg10334741	11	128,321,749	2.28E-13	0.304	0.301					
cg04159905	5	691,354	4.2E-10	0.304	0.307				*TPPP*	5'UTR
cg27640794	4	169,555,699	1.86E-10	0.304	0.297				*PALLD*	5'UTR
cg10184889	8	86,350,919	0.000107	0.304	0.300				*CA3*	TSS200
cg14374337	14	75,446,498	1.34E-20	0.304	0.284					
cg11525280	2	26,624,667	0.045576	0.304	0.079				*C2orf39*	TSS200
cg26848524	8	55,294,566	1.19E-05	0.304	0.280					
cg21007342	14	94,917,627	6.24E-10	0.304	0.290				*SERPINA11*	5'UTR
cg13966628	1	240,629,046	8.79E-07	0.304	0.304				*FMN2*	Body
cg14159342	1	3,624,292	1.58E-09	0.304	0.286				*TP73*	Body
cg19484420	2	127,414,108	0.030805	0.304	0.417				*GYPC*	Body
cg04317962	16	79,623,625	1.04E-09	0.304	0.270					
cg19500479	2	20,865,674	0.000106	0.304	0.288				*GDF7*	TSS1500
cg10883284	6	27,059,715	0.007837	0.304	0.256					
cg08214995	16	22,826,243	7.12E-07	0.304	0.257				*HS3ST2*	1stExon
cg24110050	1	67,217,853	1.4E-05	0.304	0.267	1.42E-15	0.188		*TCTEX1D1*	TSS1500
cg22546374	6	27,301,172	1.35E-06	0.304	0.314					
cg22792176	10	130,274,452	0.000136	0.304	0.234					
cg18395327	2	220,379,109	0.041523	0.304	0.303				*ACCN4*	1stExon
cg24902339	10	119,867,927	8.92E-09	0.304	0.246				*CASC2*	Body

cg08384999	X	40,004,346	3.29E-07	0.304	0.214				*BCOR*	5'UTR
cg05187508	6	29,974,306	2.66E-08	0.304	0.296				*HLA-J*	Body
cg01649773	11	27,015,991	3.68E-18	0.304	0.301				*FIBIN*	5'UTR
cg09723635	6	17,866,039	5.95E-22	0.304	0.309				*KIF13A*	Body
cg17807172	5	122,620,929	2.25E-05	0.304	0.308					
cg00178511	3	48,632,778	0.008415	0.304	0.330				*COL7A1*	TSS200
cg06630799	20	55,203,840	0.000324	0.304	0.241				*TFAP2C*	TSS1500
cg05211836	19	16,999,576	3.25E-06	0.304	0.327				*F2RL3*	TSS1500
cg00577935	5	112,073,348	0.006154	0.304	0.154				*APC*	TSS1500
cg01244015	2	225,266,843	0.014238	0.304	0.352				*FAM124B*	TSS200
cg02381898	4	54,970,175	2.71E-05	0.304	0.294					
cg18769074	3	133,464,867	1.1E-07	0.304	0.239				*TF*	TSS200
cg06774787	X	56,258,973	9E-08	0.304	0.270				*KLF8*	5'UTR
cg07366670	12	111,619,077	0.009681	0.304	0.188				*CUX2*	Body
cg24389412	X	70,323,743	1.9E-11	0.304	0.300				*CXorf65*	Body
cg24495017	5	134,370,530	4.25E-15	0.304	0.290	1.66E-30	0.213		*PITX1*	TSS1500
cg25143990	12	28,127,783	2.29E-06	0.304	0.311					
cg20252022	12	77,272,172	0.001007	0.304	0.156				*CSRP2*	5'UTR
cg21131024	8	104,153,643	5.48E-07	0.304	0.298				*C8orf56*	TSS200
cg13714026	10	73,385,503	2.15E-23	0.304	0.301				*CDH23*	Body
cg20215212	17	28,618,041	6.72E-11	0.304	0.302				*BLMH*	Body
cg05692123	5	122,620,950	5.74E-06	0.304	0.302					
cg26822438	X	119,134,368	7.73E-06	0.304	0.281					
cg03705784	11	95,888,212	4.15E-20	0.304	0.287				*MAML2*	Body
cg09756225	7	73,894,999	0.001946	0.304	0.245				*GTF2IRD1*	5'UTR
cg25260543	7	100,946,792	5.35E-06	0.304	0.255					
cg11325273	13	36,642,977	1.71E-15	0.304	0.328				*DCLK1*	Body
cg09567485	16	67,203,753	0.001316	0.304	0.394				*HSF4*	3'UTR
cg21996227	1	156,130,826	1.71E-06	0.304	0.298				*SEMA4A*	Body
cg04649530	9	132,113,372	6.72E-19	0.304	0.273					
cg20859731	20	36,226,820	1.16E-05	0.304	0.331					
cg22356428	1	206,687,071	5.1E-22	0.304	0.295				*RASSF5*	Body
cg13569051	9	124,051,703	7.14E-25	0.304	0.296				*GSN*	5'UTR
cg05297854	22	50,623,687	4.78E-05	0.304	0.293				*TRABD*	TSS1500

cg21425842	6	26,240,939	0.000741	0.304	0.295				*HIST1H4F*	1stExon
cg11239720	4	152,967,415	1.01E-08	0.304	0.272					
cg19502359	11	72,912,441	6.26E-16	0.304	0.287					
cg20972214	12	8,088,818	4.35E-05	0.303	0.304	1.71E-30	0.217		*SLC2A3*	1stExon
cg05809668	7	35,301,188	0.015078	0.303	0.296					
cg13413955	9	842,914	8.24E-07	0.303	0.270				*DMRT1*	Body
cg22738219	10	13,043,313	7.33E-06	0.303	0.276				*CCDC3*	1stExon
cg16481332	7	2,654,053	3.97E-08	0.303	0.265				*IQCE*	3'UTR
cg20536041	2	45,029,004	0.002884	0.303	0.304					
cg05840412	13	49,145,436	2.05E-06	0.303	0.251					
cg12988117	7	51,658,747	7.9E-13	0.303	0.306					
cg25513924	3	187,009,486	4.07E-09	0.303	0.306			Yes	*MASP1*	5'UTR
cg11969002	6	30,712,282	0.016698	0.303	0.297				*IER3*	1stExon
cg20045320	11	319,555	6.26E-07	0.303	0.208					
cg04529938	17	73,084,494	1.29E-17	0.303	0.276				*SLC16A5*	5'UTR
cg08047457	3	50,378,413	4.44E-06	0.303	0.292	1.89E-08	0.123		*RASSF1*	TSS200
cg13448197	10	43,846,281	0.007895	0.303	0.187					
cg26040816	20	42,816,135	2.25E-07	0.303	0.220				*JPH2*	1stExon
cg21216015	1	26,230,906	5.16E-15	0.303	0.281				*STMN1*	Body
cg17936721	5	77,140,543	0.002955	0.303	0.319					
cg04119462	16	2,415,808	5.48E-09	0.303	0.297				*ABCA17P*	Body
cg05285244	7	149,412,760	1.45E-08	0.303	0.203				*KRBA1*	5'UTR
cg10508993	5	180,018,495	4.7E-130	0.303	0.302				*SCGB3A1*	TSS200
cg21971621	11	65,600,773	3.54E-07	0.303	0.304				*SNX32*	TSS1500
cg27558693	1	85,710,632	0.000422	0.303	0.282					
cg03884132	15	33,009,531	0.017859	0.303	0.317				*GREM1*	TSS1500
cg08802841	12	89,748,726	3.15E-11	0.303	0.288					
cg04881103	1	32,410,413	1.49E-06	0.303	0.267					
cg13207333	6	29,974,798	1.09E-10	0.303	0.285				*HLA-J*	Body
cg13864794	7	16,505,664	0.000643	0.303	0.348				*SOSTDC1*	TSS200
cg10148280	3	148,846,750	1.21E-05	0.303	0.255	0.0000047	0.112		*HPS3*	TSS1500
cg13620051	9	126,759,535	0.000484	0.303	0.343					
cg18612255	12	1,770,715	1.04E-07	0.303	0.283					
cg19934381	17	80,944,090	2.05E-16	0.303	0.277				*B3GNTL1*	Body

cg26271045	2	238,535,754	0.000122	0.303	0.236				*LRRFIP1*	TSS1500
cg10372921	15	74,218,733	1.37E-30	0.303	0.294				*LOXL1*	TSS200
cg14328457	17	37,381,475	0.002024	0.303	0.360				*STAC2*	Body
cg03257172	1	200,010,283	0.016325	0.303	0.383				*NR5A2*	Body
cg06520003	17	48,504,157	2.83E-13	0.303	0.300				*ACSF2*	Body
cg05644921	4	141,489,114	0.001096	0.303	0.258				*UCP1*	Body
cg23808165	22	25,421,713	2.16E-18	0.303	0.310					
cg22851295	6	10,881,949	7.9E-26	0.303	0.282				*GCM2*	1stExon
cg00286102	20	61,047,376	0.012591	0.303	0.137			Yes	*GATA5*	Body
cg09122035	11	319,667	2.41E-06	0.303	0.248					
cg16985778	1	32,708,234	3.98E-13	0.303	0.295				*MTMR9L*	TSS1500
cg15876825	3	11,651,881	9.95E-15	0.303	0.275				*VGLL4*	Body
cg26331172	1	151,118,299	3.83E-11	0.303	0.302				*SEMA6C*	5'UTR
cg26877596	12	106,534,217	1.05E-16	0.303	0.289				*NUAK1*	TSS1500
cg17602451	18	60,985,645	0.003207	0.303	0.330	1.29E-10	0.188		*BCL2*	Body
cg22070991	4	4,856,904	0.002096	0.303	0.277					
cg24341129	9	16,727,830	1.72E-23	0.303	0.264	8.29E-27	0.218		*BNC2*	Body
cg25117600	4	10,023,201	8.39E-11	0.303	0.289				*SLC2A9*	TSS200
cg09143673	5	172,664,439	4.14E-06	0.303	0.294					
cg18946226	13	109,248,503	1.08E-08	0.303	0.209	2.17E-27	0.257		*MYO16*	5'UTR
cg13203394	12	26,951,217	1.01E-08	0.303	0.319				*ITPR2*	Body
cg26055054	X	134,654,347	0.001202	0.303	0.190				*DDX26B*	TSS1500
cg09151979	6	28,457,624	0.018209	0.303	0.329					
cg18792146	1	235,105,751	3.62E-11	0.303	0.259					
cg01763453	16	81,764,614	6.09E-13	0.303	0.278					
cg09142313	5	72,677,859	8.21E-08	0.303	0.303					
cg02254461	3	39,195,904	7.28E-17	0.303	0.306	3.3E-33	0.320		*CSRNP1*	TSS1500
cg10484211	1	218,547,935	1.67E-05	0.303	0.268				*TGFB2*	Body
cg07029873	1	217,311,014	3.81E-06	0.303	0.295				*ESRRG*	1stExon
cg21120951	4	152,611,527	1.83E-15	0.303	0.307			Yes	*PET112L*	Body
cg17619311	3	42,947,565	0.007883	0.303	0.212				*ZNF662*	TSS200
cg09537620	11	31,826,574	0.000153	0.303	0.296				*PAX6*	Body
cg08235220	8	40,835,933	5.7E-14	0.303	0.295					
cg11920449	6	36,645,608	1.43E-07	0.303	0.320				*CDKN1A*	TSS1500

cg25558103	17	42,835,859	6.96E-09	0.303	0.309				*ADAM11*	TSS1500
cg25883066	8	127,647,405	4E-27	0.303	0.295					
cg06096336	2	231,989,800	0.001573	0.303	0.255	0.00680158	0.070		*PSMD1*	Body
cg13470462	3	112,965,787	3.37E-12	0.303	0.305				*BOC*	5'UTR
cg17754510	6	10,391,412	0.000161	0.303	0.314					
cg19751300	18	44,336,399	2.51E-06	0.303	0.277	7.2E-19	0.173		*ST8SIA5*	1stExon
cg18949886	X	3,265,318	0.00031	0.303	0.280				*MXRA5*	TSS1500
cg07134230	1	156,631,248	0.001196	0.303	0.379					
cg05234415	19	38,743,744	1.64E-31	0.303	0.281				*PPP1R14A*	Body
cg11722971	2	1,286,544	6.02E-05	0.303	0.195				*SNTG2*	Body
cg08247397	19	4,607,146	0.020267	0.303	0.219					
cg26248173	1	166,853,578	0.02369	0.303	0.191					
cg18355902	4	154,681,128	0.002409	0.303	0.355				*RNF175*	5'UTR
cg00188748	9	130,564,509	4.05E-17	0.303	0.282				*FPGS*	TSS1500
cg09767126	8	103,877,365	1.96E-06	0.303	0.306				*AZIN1*	TSS1500
cg18496653	21	38,593,042	0.037703	0.302	0.154					
cg00338391	7	1,283,981	0.000145	0.302	0.313					
cg24025550	15	59,157,523	3.95E-08	0.302	0.305					
cg00249511	11	627,152	4.68E-05	0.302	0.309				*SCT*	5'UTR
cg16283183	3	45,718,805	6.79E-07	0.302	0.285				*LIMD1*	3'UTR
cg06374079	6	27,182,908	0.009229	0.302	0.319					
cg06985993	16	30,040,055	9.19E-08	0.302	0.282				*FAM57B*	Body
cg04208434	3	129,513,427	3.9E-08	0.302	0.269				*TMCC1*	Body
cg08323075	4	5,053,504	0.037314	0.302	0.183				*STK32B*	TSS200
cg15084851	1	36,317,380	5.66E-16	0.302	0.324				*EIF2C4*	Body
cg07471614	8	125,855,152	1.8E-22	0.302	0.300					
cg17326090	22	28,198,472	9.8E-12	0.302	0.319				*MN1*	TSS1500
cg25216196	17	19,290,520	1.63E-19	0.302	0.291				*MFAP4*	TSS200
cg26783727	16	67,204,807	1.3E-12	0.302	0.310					
cg04376312	3	123,602,485	1.85E-12	0.302	0.291	9.19E-31	0.213		*MYLK*	5'UTR
cg23125200	13	20,806,732	0.002513	0.302	0.218				*GJB6*	TSS200
cg19256731	20	1,874,527	1.68E-09	0.302	0.317				*SIRPA*	TSS1500
cg17968795	2	99,553,406	4.32E-05	0.302	0.314				*C2orf55*	TSS1500
cg02639927	1	77,334,748	2.24E-05	0.302	0.294				*ST6GALNAC5*	Body

cg17296482	12	4,382,188	0.000117	0.302	0.287				*CCND2*	TSS1500
cg01678309	15	68,930,255	2.23E-08	0.302	0.249				*CORO2B*	Body
cg07676859	20	23,015,932	0.001079	0.302	0.187				*SSTR4*	TSS200
cg16389901	14	54,423,807	0.000219	0.302	0.253				*BMP4*	TSS1500
cg12439852	7	16,627,383	2.21E-09	0.302	0.271					
cg20658100	13	74,707,145	0.015711	0.302	0.350				*KLF12*	5'UTR
cg24154839	4	46,995,741	8.32E-11	0.302	0.325				*GABRA4*	TSS200
cg11165158	16	86,613,094	9.56E-10	0.302	0.279				*FOXL1*	1stExon
cg22492099	15	43,414,974	4.04E-12	0.302	0.287					
cg08075452	3	184,056,843	9.2E-16	0.302	0.292				*FAM131A*	5'UTR
cg08201451	16	70,760,142	6.59E-20	0.302	0.287				*VAC14*	Body
cg03208951	19	48,828,835	0.026198	0.302	0.265				*EMP3*	1stExon
cg00252282	12	5,542,736	2.53E-05	0.302	0.270				*NTF3*	Body
cg04175292	6	44,187,226	0.014604	0.302	0.228				*SLC29A1*	TSS200
cg25790133	4	2,627,014	5.46E-27	0.302	0.287				*FAM193A*	TSS200
cg18438124	1	21,996,315	0.008377	0.302	0.283				*RAP1GAP*	TSS1500
cg03089717	6	28,557,411	0.001593	0.302	0.248					
cg11533881	6	29,894,322	3.19E-08	0.302	0.301				*HCG4P6*	TSS1500
cg02300652	2	152,804,107	4.4E-10	0.302	0.236				*CACNB4*	Body
cg06707286	15	102,010,195	5.73E-14	0.302	0.284				*PCSK6*	Body
cg17037963	12	30,949,050	0.001513	0.302	0.268					
cg14507337	14	60,978,161	0.000117	0.302	0.301				*SIX6*	3'UTR
cg24913825	2	198,134,173	2.75E-14	0.302	0.312					
cg24946597	8	31,497,464	0.014426	0.302	0.245				*NRG1*	5'UTR
cg25074493	2	131,186,495	1.01E-14	0.302	0.295					
cg05085230	6	133,562,461	0.002856	0.302	0.378				*EYA4*	TSS200
cg17852104	X	102,000,694	3.71E-15	0.302	0.273				*BHLHB9*	5'UTR
cg24687970	1	1,397,896	7.06E-15	0.302	0.302				*ATAD3C*	Body
cg15442728	14	91,580,280	0.000274	0.302	0.178				*C14orf159*	TSS200
cg16058797	8	102,236,617	2.5E-07	0.302	0.319					
cg10680264	15	75,470,927	8.93E-07	0.302	0.293					
cg00384539	8	70,983,567	9.67E-10	0.302	0.316				*PRDM14*	TSS200
cg08750459	17	6,558,815	4.77E-05	0.302	0.308					
cg16204205	18	19,753,432	2.65E-22	0.302	0.268				*GATA6*	Body

cg18447876	3	54,121,761	3.22E-06	0.302	0.338					
cg21752211	7	27,243,463	0.015147	0.302	0.389					
cg14580211	5	150,161,299	5.07E-13	0.302	0.290				*C5orf62*	Body
cg15485700	1	117,421,329	0.008619	0.302	0.102					
cg04963514	10	44,162,879	0.001196	0.302	0.247					
cg03470207	6	28,574,981	0.009427	0.302	0.311					
cg02871995	11	111,410,960	5.24E-11	0.302	0.262				*LAYN*	TSS1500
cg00161791	21	45,575,014	4.42E-13	0.302	0.277					
cg04458368	8	38,411,682	0.00057	0.302	0.271					
cg27081634	11	68,778,745	4.74E-11	0.302	0.297				*MRGPRF*	5'UTR
cg00619126	2	105,459,775	3.55E-09	0.302	0.291					
cg21530419	3	186,490,728	1.42E-06	0.302	0.287					
cg23845168	17	31,271,015	2.76E-15	0.302	0.281					
cg18121003	11	86,085,757	1.08E-06	0.302	0.317				*CCDC81*	TSS200
cg27009812	16	3,062,597	0.000101	0.302	0.305				*CLDN9*	1stExon
cg09013220	17	45,944,385	3.26E-16	0.302	0.288					
cg07394914	4	185,371,335	2.04E-09	0.302	0.307				*IRF2*	5'UTR
cg24923226	X	129,255,138	0.040173	0.302	0.394					
cg15803065	6	29,973,988	3.25E-07	0.302	0.332			Yes	*HLA-J*	Body
cg05000331	16	79,623,678	6.43E-08	0.302	0.232					
cg09168153	13	110,779,117	2.98E-14	0.302	0.271					
cg05911003	4	187,065,516	1.79E-07	0.302	0.214				*FAM149A*	TSS1500
cg26851107	11	70,508,659	7.06E-15	0.302	0.258				*SHANK2*	Body
cg13298199	1	208,132,388	6.06E-08	0.302	0.295					
cg15596666	10	135,160,792	1.02E-09	0.302	0.267				*PRAP1*	TSS200
cg10829727	17	73,084,309	1.75E-20	0.302	0.307				*SLC16A5*	5'UTR
cg13842305	1	151,033,007	8.02E-05	0.302	0.189				*MLLT11*	5'UTR
cg24199599	20	62,681,243	1.86E-06	0.302	0.294				*SOX18*	TSS1500
cg08783090	X	151,143,125	0.000116	0.301	0.324				*GABRE*	1stExon
cg10248878	4	54,965,109	1.56E-09	0.301	0.302				*GSX2*	TSS1500
cg05496203	2	66,664,798	0.002955	0.301	0.353				*MEIS1*	Body
cg11891395	7	96,652,153	6.75E-09	0.301	0.290				*DLX5*	Body
cg03124636	6	30,711,426	6.66E-19	0.301	0.289				*IER3*	3'UTR
cg15127227	7	131,242,878	0.013952	0.301	0.189					

cg24035484	12	20,000,505	6.26E-11	0.301	0.320					
cg05388880	4	183,807,081	8.83E-08	0.301	0.271					
cg03745002	16	52,581,405	0.045182	0.301	0.216				*TOX3*	5'UTR
cg22462235	1	226,129,481	3.8E-12	0.301	0.330	2.57E-18	0.222	Yes	*LEFTY2*	TSS1500
cg13073224	12	117,131,523	0.008068	0.301	0.154					
cg06074896	7	1,500,176	1.11E-14	0.301	0.309				*MICALL2*	TSS1500
cg14847502	10	30,724,194	3.99E-05	0.301	0.252				*MAP3K8*	5'UTR
cg04248271	5	158,524,404	0.00132	0.301	0.363				*EBF1*	Body
cg06808498	14	60,974,525	2.08E-05	0.301	0.272				*SIX6*	TSS1500
cg18643093	5	150,521,257	1.8E-09	0.301	0.266				*ANXA6*	Body
cg08443845	21	36,421,955	0.014728	0.301	0.175				*RUNX1*	TSS1500
cg17800654	1	50,888,590	3.32E-06	0.301	0.315				*DMRTA2*	5'UTR
cg04278110	2	198,063,759	9.61E-05	0.301	0.240				*ANKRD44*	TSS1500
cg26607748	9	35,690,226	3.09E-07	0.301	0.255				*TPM2*	TSS200
cg02115599	7	27,244,564	0.032645	0.301	0.397					
cg15104644	1	231,297,103	0.005224	0.301	0.351					
cg00170003	6	108,490,371	6.37E-05	0.301	0.310				*NR2E1*	Body
cg00395210	12	48,592,465	2.18E-07	0.301	0.307					
cg07201620	5	83,679,941	0.000107	0.301	0.264				*EDIL3*	Body
cg16371229	1	233,248,845	8.43E-08	0.301	0.294				*PCNXL2*	Body
cg14335429	8	42,342,083	1.13E-22	0.301	0.264				*SLC20A2*	5'UTR
cg27545611	1	68,517,177	7.2E-16	0.301	0.309				*DIRAS3*	TSS1500
cg05036656	4	41,875,470	0.000275	0.301	0.319					
cg14920289	14	23,821,149	2.72E-28	0.301	0.290				*SLC22A17*	Body
cg09513990	6	27,235,822	0.000735	0.301	0.381					
cg10163776	3	32,433,833	0.00655	0.301	0.221				*CMTM7*	Body
cg25588844	2	10,037,561	9.36E-15	0.301	0.264				*TAF1B*	Body
cg16310415	8	25,898,539	9.01E-07	0.301	0.263				*EBF2*	Body
cg09849405	16	66,612,955	5.97E-05	0.301	0.264				*CMTM1*	3'UTR
cg13112361	11	63,258,739	0.042866	0.301	0.249				*HRASLS5*	TSS200
cg02909790	6	26,271,587	0.00073	0.301	0.339	7.3E-19	0.262		*HIST1H3G*	1stExon
cg23398047	8	49,783,505	2.07E-26	0.301	0.278					
cg15234492	12	122,019,076	1.24E-10	0.301	0.261				*KDM2B*	TSS200
cg05221748	5	122,621,289	8.5E-05	0.301	0.304					

cg14678774	19	12,978,360	6.15E-08	0.301	0.281				*MAST1*	Body
cg07910560	7	157,361,396	1.08E-17	0.301	0.281				*PTPRN2*	Body
cg07027116	1	65,731,738	0.037168	0.301	0.293				*DNAJC6*	Body
cg17740822	12	89,744,609	3.97E-11	0.301	0.301				*DUSP6*	Body
cg02970525	1	201,915,999	0.00231	0.301	0.117				*LMOD1*	TSS1500
cg10251229	7	630,581	7.84E-12	0.301	0.279				*PRKAR1B*	Body
cg15476790	2	218,786,102	6.77E-20	0.301	0.287				*TNS1*	5'UTR
cg01916115	6	129,204,339	0.000698	0.301	0.318				*LAMA2*	5'UTR
cg15056966	3	120,003,702	1.34E-05	0.301	0.229					
cg14089984	1	1,182,418	0.001542	0.301	0.313				*FAM132A*	TSS1500
cg09803262	7	96,622,608	1.44E-07	0.301	0.268				*DLX6AS*	Body
cg00353773	10	44,880,708	0.000282	0.301	0.259				*CXCL12*	TSS200
cg03148889	1	78,706,562	4.89E-12	0.301	0.285				*MGC27382*	Body
cg13294849	3	181,441,571	7.98E-06	0.301	0.285				*SOX2OT*	Body
cg08207876	2	181,844,876	0.028222	0.301	0.287			Yes	*UBE2E3*	TSS1500
cg03832522	2	218,898,668	8.91E-06	0.301	0.296					
cg08162372	14	54,422,925	0.039233	0.301	0.376				*BMP4*	5'UTR
cg06169590	2	43,455,088	0.000308	0.301	0.210				*ZFP36L2*	TSS1500
cg11102858	17	66,255,427	0.000857	0.301	0.322				*ARSG*	5'UTR
cg02897667	7	51,539,131	2.04E-08	0.301	0.235					
cg04965987	21	38,378,539	6.06E-07	0.301	0.255				*DSCR6*	TSS1500
cg07303912	20	6,032,782	0.004936	0.301	0.120				*LRRN4*	Body
cg16856833	3	87,138,640	7.37E-07	0.301	0.246					
cg23923856	1	113,051,685	0.000757	0.301	0.285				*WNT2B*	Body
cg07080224	14	21,574,644	2.92E-13	0.301	0.307					
cg17227926	10	115,860,799	0.013205	0.301	0.063					
cg07773769	3	52,280,329	5.4E-09	0.301	0.226				*PPM1M*	1stExon
cg19536664	17	6,899,085	1.26E-10	0.301	0.241				*ALOX12*	TSS1500
cg22352717	2	132,285,972	0.000123	0.301	0.223				*CCDC74A*	Body
cg05707492	17	78,833,484	5.2E-07	0.301	0.198				*RPTOR*	Body
cg20078807	22	45,608,713	5.14E-13	0.301	0.269				*C22orf9*	Body
cg15366127	X	40,003,935	1.59E-15	0.301	0.280				*BCOR*	5'UTR
cg21504918	5	150,400,001	0.001361	0.301	0.127	2.15E-11	0.166		*GPX3*	5'UTR
cg23965588	11	71,699,167	1.23E-11	0.301	0.292				*RNF121*	Body

cg08309183	7	150,674,375	3.54E-14	0.301	0.299				*KCNH2*	Body
cg11213150	9	129,884,457	2.69E-22	0.300	0.285	3.6E-16	0.163		*ANGPTL2*	5'UTR
cg18905461	14	52,534,596	0.00279	0.300	0.332				*NID2*	Body
cg26022526	4	8,271,319	1.5E-07	0.300	0.243				*HTRA3*	TSS200
cg13023623	14	57,276,257	4.23E-07	0.300	0.317				*OTX2*	5'UTR
cg13787932	5	191,793	1.16E-19	0.300	0.283				*LRRC14B*	1stExon
cg13692082	9	136,447,400	1.74E-16	0.300	0.288					
cg08864042	2	74,607,470	5.64E-14	0.300	0.311				*DCTN1*	5'UTR
cg10062193	4	47,533,905	7.25E-14	0.300	0.314				*ATP10D*	Body
cg15982972	5	50,260,129	3.61E-05	0.300	0.232					
cg20648847	11	66,326,767	4.42E-05	0.300	0.335				*ACTN3*	Body
cg12366772	2	122,041,891	1.34E-11	0.300	0.281				*TFCP2L1*	Body
cg12552771	22	29,709,320	4.27E-05	0.300	0.335				*RASL10A*	Body
cg02798576	10	101,290,168	7.62E-06	0.300	0.291					
cg17534540	3	129,024,712	5.78E-13	0.300	0.315					
cg00026474	8	134,585,500	4.03E-11	0.300	0.296				*ST3GAL1*	TSS1500
cg13329912	6	16,215,786	9.47E-11	0.300	0.299					
cg19572637	2	66,660,175	2.16E-07	0.300	0.260					
cg18137704	22	17,602,681	9.85E-13	0.300	0.265	5.06E-30	0.196		*CECR6*	TSS1500
cg03323696	5	59,189,120	6.24E-05	0.300	0.296				*PDE4D*	Body
cg03635766	17	38,607,865	1.28E-18	0.300	0.308				*IGFBP4*	Body
cg14019186	3	158,390,074	0.015872	0.300	0.231				*GFM1*	Body
cg26734888	19	37,825,307	0.004314	0.300	0.178				*HKR1*	TSS1500
cg25844133	17	21,477,738	6.97E-06	0.300	0.284					
cg05687834	12	106,534,276	3.35E-09	0.300	0.282				*NUAK1*	TSS1500
cg02769145	1	160,952,126	0.002129	0.300	0.318					
cg01355757	2	74,272,773	1.4E-20	0.300	0.311				*TET3*	TSS1500
cg04645914	19	52,452,317	0.000183	0.300	0.312					
cg20652404	15	74,218,904	2.99E-17	0.300	0.270				*LOXL1*	5'UTR
cg07517893	19	45,889,164	1.68E-06	0.300	0.298				*PPP1R13L*	Body
cg05065948	4	26,275,089	4.04E-26	0.300	0.279					
cg15292275	13	109,149,011	0.001145	0.300	0.229					
cg17459215	2	66,298,242	2.23E-08	0.300	0.322					
cg10082841	9	130,689,321	1.03E-11	0.300	0.250				*PIP5KL1*	Body

cg14380111	15	81,426,360	0.000263	0.300	0.235				*C15orf26*	TSS1500
cg15970666	2	162,095,662	6.46E-10	0.300	0.292					
cg21770060	3	159,583,545	2.93E-15	0.300	0.269				*SCHIP1*	Body
cg00912746	1	33,350,709	4.36E-21	0.300	0.301				*HPCA*	TSS1500
cg06108383	6	32,120,899	9.77E-13	0.300	0.292				*PPT2*	TSS1500
cg02327530	7	100,091,786	1.53E-14	0.300	0.304				*C7orf51*	3'UTR
cg17143518	18	20,716,545	1.54E-06	0.300	0.176				*CABLES1*	1stExon
cg20133129	X	153,192,433	0.014717	0.300	0.277				*ARHGAP4*	TSS1500
cg25149251	7	100,488,527	0.006609	0.300	0.310				*UFSP1*	TSS1500
cg00690082	17	40,438,511	1.49E-17	0.300	0.278				*STAT5A*	TSS1500
cg16937268	20	21,684,417	0.002578	0.300	0.329					
cg13551814	20	45,280,276	3.62E-16	0.300	0.293				*SLC13A3*	TSS200
cg17180284	13	31,783,983	2.6E-07	0.300	0.348				*B3GALTL*	Body
cg17260706	11	118,782,879	1.06E-19	0.300	0.267				*BCL9L*	TSS1500
cg01744822	16	73,100,510	6.92E-19	0.300	0.289					
cg15184386	3	136,538,601	0.006749	0.300	0.135				*TMEM22*	5'UTR
cg17608103	6	169,653,612	0.001309	0.300	0.334				*THBS2*	5'UTR
cg18755783	13	36,919,905	0.02413	0.300	0.341	1.92E-18	0.251		*SPG20*	5'UTR
cg00488692	2	174,877,566	1.17E-05	0.300	0.269					
cg08958294	6	146,350,131	0.012785	0.300	0.396				*GRM1*	5'UTR
cg04970352	11	44,327,399	6.54E-07	0.300	0.298	1.24E-22	0.272		*ALX4*	Body
cg21820656	15	59,157,075	5.6E-08	0.300	0.314					
cg12596505	1	213,090,240	3.49E-05	0.300	0.335					
cg11759875	8	1,405,060	4.39E-34	0.300	0.255			Yes		
cg00531748	4	55,992,689	3.35E-07	0.300	0.269				*KDR*	TSS1500
cg17674042	6	34,482,479	6.88E-09	0.300	0.289				*PACSIN1*	5'UTR
cg18128164	19	2,253,492	3.51E-12	0.300	0.286				*JSRP1*	Body
cg11498607	21	36,399,226	0.000155	0.300	0.316				*RUNX1*	Body
cg19626138	8	37,731,969	3.35E-23	0.300	0.287				*RAB11FIP1*	Body
cg10861599	1	173,176,523	6.27E-09	0.300	0.296	1.76E-09	0.167		*TNFSF4*	TSS200
cg14015044	8	22,960,619	0.008103	0.300	0.268	3.9E-22	0.260		*TNFRSF10C*	5'UTR
cg16126286	1	243,646,235	3.85E-06	0.300	0.235				*SDCCAG8*	Body
cg14442421	9	19,788,257	0.000716	0.300	0.134				*SLC24A2*	TSS1500
cg26027669	1	92,952,467	0.000162	0.300	0.262				*GFI1*	TSS1500

cg04376123	7	73,895,109	6.6E-06	0.299	0.253				*GTF2IRD1*	5'UTR
cg04621728	2	234,934,131	4.04E-07	0.299	0.275					
cg19214707	7	3,157,722	8.25E-05	0.299	0.237					
cg20229496	19	35,395,800	1.96E-09	0.299	0.306					
cg18514922	2	160,761,262	0.000525	0.299	0.296				*LY75*	TSS200
cg23298862	7	158,159,286	5.07E-08	0.299	0.286				*PTPRN2*	Body
cg26071135	11	843,943	1.54E-05	0.299	0.232				*TSPAN4*	TSS1500
cg15426035	19	36,024,685	3.16E-05	0.299	0.320				*GAPDHS*	Body
cg17716765	15	29,407,853	9.1E-05	0.299	0.266				*APBA2*	Body
cg02804510	19	49,199,965	6.73E-05	0.299	0.271				*FUT2*	5'UTR
cg12378753	10	116,527,201	1.14E-09	0.299	0.224					
cg08465346	12	54,441,458	0.000126	0.299	0.264				*HOXC4*	5'UTR
cg11334771	11	71,955,337	0.000173	0.299	0.285				*PHOX2A*	TSS200
cg17589341	18	43,304,079	8.4E-10	0.299	0.303	5.62E-29	0.274		*SLC14A1*	TSS200
cg10587854	3	37,011,630	6.64E-08	0.299	0.321					
cg25099065	2	69,994,225	2.71E-41	0.299	0.302				*ANXA4*	5'UTR
cg27277463	12	62,585,031	0.026086	0.299	0.366				*FAM19A2*	5'UTR
cg05001334	13	35,391,234	1.33E-05	0.299	0.229					
cg16778107	16	381,873	5.03E-15	0.299	0.313				*AXIN1*	Body
cg17269633	17	42,733,600	2.51E-05	0.299	0.261				*C17orf104*	TSS200
cg22139166	5	124,071,107	2.11E-07	0.299	0.256				*ZNF608*	Body
cg00530492	6	29,943,464	3.97E-05	0.299	0.315				*HCG9*	Body
cg22111078	5	172,655,925	1.02E-06	0.299	0.258					
cg01142710	5	145,713,754	1.81E-05	0.299	0.277					
cg16509851	4	174,430,614	0.000624	0.299	0.309					
cg21004104	1	15,954,209	5.15E-14	0.299	0.250				*DDI2*	Body
cg19428722	10	100,994,478	7.19E-06	0.299	0.274				*HPSE2*	Body
cg26130488	17	59,530,104	3.03E-09	0.299	0.298					
cg07220922	3	10,806,022	0.00048	0.299	0.273				*LOC285370*	TSS200
cg12585943	6	32,121,368	5.49E-17	0.299	0.290	2.8E-33	0.246		*PPT2*	TSS1500
cg03166753	6	38,683,221	2.94E-12	0.299	0.287					
cg16557944	1	53,068,197	0.002839	0.299	0.090	1.4E-11	0.258		*GPX7*	1stExon
cg11586832	22	37,721,305	3.08E-06	0.299	0.239					
cg00762613	13	36,045,224	0.000269	0.299	0.327				*NBEA*	Body

cg20175702	15	70,053,398	1.91E-06	0.299	0.297					
cg25157524	13	30,002,931	0.000192	0.299	0.236				*MTUS2*	1stExon
cg01860897	1	90,309,605	1.46E-07	0.299	0.233				*LRRC8D*	5'UTR
cg04111435	11	63,448,455	0.000487	0.299	0.164				*RTN3*	TSS1500
cg19108771	6	32,076,337	1.04E-06	0.299	0.333				*TNXB*	5'UTR
cg26818820	5	135,468,000	2.63E-07	0.299	0.259				*SMAD5*	TSS1500
cg26156256	1	116,370,891	1.67E-39	0.299	0.282					
cg20313963	12	8,089,207	2.88E-09	0.299	0.299				*SLC2A3*	TSS1500
cg26995244	22	45,125,395	3.61E-25	0.299	0.295				*PRR5*	Body
cg11445109	10	135,343,248	0.01993	0.299	0.240				*CYP2E1*	Body
cg06274159	4	188,916,867	0.001303	0.299	0.320	3.78E-12	0.265		*ZFP42*	TSS200
cg21143446	13	108,922,340	3.39E-06	0.299	0.255				*TNFSF13B*	1stExon
cg01544903	16	56,697,229	1.59E-07	0.299	0.293					
cg14654171	9	130,151,001	5.49E-07	0.299	0.292				*GARNL3*	Body
cg04217778	7	5,111,916	0.007805	0.299	0.317				*LOC389458*	Body
cg26181939	11	61,063,062	3.34E-19	0.299	0.257				*VWCE*	TSS1500
cg07964848	10	28,035,894	1.41E-07	0.299	0.309				*MKX*	TSS1500
cg17011709	10	94,826,053	1.75E-05	0.299	0.186				*CYP26C1*	Body
cg06894334	4	1,398,599	8.92E-10	0.299	0.267					
cg23562538	8	144,798,877	1.72E-19	0.299	0.323				*MAPK15*	Body
cg23313031	6	29,974,745	1.26E-09	0.299	0.284				*HLA-J*	Body
cg02792792	5	115,152,420	0.001226	0.299	0.152				*CDO1*	TSS200
cg06370069	10	73,728,417	5.92E-09	0.299	0.305				*CHST3*	5'UTR
cg06585233	1	229,543,009	1.77E-06	0.299	0.297					
cg15856626	3	140,796,013	5.63E-06	0.299	0.263				*SPSB4*	Body
cg23489038	12	111,619,190	0.00183	0.299	0.175				*CUX2*	Body
cg04282607	22	22,007,286	1.56E-25	0.299	0.288				*MIR301B*	Body
cg24314564	3	188,668,940	1.31E-08	0.299	0.322					
cg11861562	11	117,069,780	2.27E-18	0.299	0.307				*TAGLN*	TSS1500
cg19792802	11	65,647,270	1.25E-12	0.299	0.288				*CTSW*	TSS200
cg03269060	3	158,450,208	0.031937	0.299	0.120				*RARRES1*	5'UTR
cg13692446	13	112,759,719	0.013842	0.299	0.368					
cg10776919	1	50,889,442	4.92E-10	0.299	0.279				*DMRTA2*	TSS1500
cg19696441	1	20,512,463	4.14E-06	0.299	0.293				*UBXN10*	TSS200

cg18093120	22	50,523,955	3.33E-05	0.299	0.263				*MLC1*	1stExon
cg18187244	6	10,881,782	6.61E-07	0.299	0.289				*GCM2*	Body
cg26781150	1	45,792,542	6.67E-05	0.299	0.287	1.55E-26	0.201		*HPDL*	TSS200
cg12516959	21	47,718,080	1.43E-17	0.299	0.283					
cg15232240	6	90,143,704	0.005658	0.299	0.236				*ANKRD6*	5'UTR
cg22466678	12	89,749,033	3.52E-08	0.299	0.293					
cg04212092	1	27,889,261	1.02E-15	0.299	0.291				*AHDC1*	5'UTR
cg08034440	2	176,940,341	2.85E-05	0.299	0.227					
cg01449144	8	49,782,592	3.48E-05	0.299	0.223					
cg18834878	X	105,067,311	0.001097	0.298	0.258				*NRK*	Body
cg22979093	7	137,028,410	0.002844	0.298	0.207				*PTN*	5'UTR
cg14635269	9	129,439,339	1.12E-06	0.298	0.283				*LMX1B*	Body
cg15988320	5	140,868,422	1.73E-11	0.298	0.322				*PCDHGA4*	Body
cg03794213	16	22,220,727	5.16E-11	0.298	0.331				*EEF2K*	5'UTR
cg27592331	18	19,756,582	1.13E-06	0.298	0.284				*GATA6*	Body
cg14473575	3	193,788,394	0.001143	0.298	0.209					
cg16707227	2	19,157,847	0.033063	0.298	0.194					
cg13463203	9	132,961,972	1.44E-20	0.298	0.306				*FREQ*	TSS1500
cg11603463	8	10,588,486	8.04E-07	0.298	0.181				*SOX7*	TSS1500
cg22560979	5	10,565,263	0.000436	0.298	0.272				*ANKRD33B*	Body
cg17516247	1	174,933,574	4.77E-27	0.298	0.288					
cg06897478	1	20,799,799	4.9E-11	0.298	0.272					
cg14626725	2	238,080,231	1.63E-17	0.298	0.281					
cg11385003	21	45,148,332	0.023634	0.298	0.189				*PDXK*	Body
cg03943218	10	102,419,600	0.011339	0.298	0.304					
cg02276070	5	149,683,066	3.46E-23	0.298	0.261				*ARSI*	TSS1500
cg04192393	15	63,673,286	1.03E-14	0.298	0.292				*CA12*	Body
cg13797425	14	102,554,977	3.09E-06	0.298	0.309				*HSP90AA1*	TSS1500
cg07636194	6	27,107,282	9.23E-06	0.298	0.295				*HIST1H2BK*	3'UTR
cg16709232	17	72,322,154	0.001166	0.298	0.189				*KIF19*	TSS200
cg19546583	19	16,022,523	1.44E-07	0.298	0.283					
cg08857994	2	130,763,641	2.68E-05	0.298	0.298					
cg02367655	1	178,064,623	0.001561	0.298	0.343				*RASAL2*	Body
cg02491396	2	172,966,428	5.55E-05	0.298	0.364				*DLX2*	Body

cg21241675	9	93,925,541	4.21E-15	0.298	0.281					
cg05086798	X	23,350,313	0.000775	0.298	0.285					
cg22660578	17	35,294,029	3.29E-05	0.298	0.334	2.61E-13	0.200		*LHX1*	TSS1500
cg15367247	3	185,960,440	6.31E-08	0.298	0.310				*DGKG*	Body
cg06807117	1	204,329,004	4.34E-28	0.298	0.288				*PLEKHA6*	5'UTR
cg17344321	7	22,617,382	0.000481	0.298	0.228					
cg12248614	19	41,018,880	0.041217	0.298	0.298				*SPTBN4*	Body
cg18566883	21	38,919,676	0.007091	0.298	0.217					
cg07126525	6	166,218,519	0.043294	0.298	0.160					
cg17495912	13	37,006,340	0.008343	0.298	0.276				*CCNA1*	5'UTR
cg24374161	11	46,582,058	0.001411	0.298	0.181				*AMBRA1*	5'UTR
cg15087147	7	127,672,169	9.06E-07	0.298	0.215	1.12E-10	0.186		*SND1*	Body
cg11843238	5	131,593,191	0.00677	0.298	0.218				*PDLIM4*	TSS200
cg02539083	7	97,361,148	2.8E-07	0.298	0.287				*TAC1*	TSS200
cg24404630	18	13,611,824	8.93E-10	0.298	0.276				*C18orf1*	5'UTR
cg10648139	2	206,551,364	0.000975	0.298	0.301				*NRP2*	Body
cg12116288	17	70,215,735	6.93E-07	0.298	0.275					
cg10440578	1	115,397,857	0.008676	0.298	0.250				*SYCP1*	5'UTR
cg15496864	10	119,312,767	0.005176	0.298	0.366					
cg25451893	12	25,538,177	2.98E-05	0.298	0.228					
cg07679836	6	33,548,423	2.44E-06	0.298	0.238				*BAK1*	TSS1500
cg12966367	3	50,378,407	1.13E-06	0.298	0.277				*RASSF1*	TSS200
cg18040354	17	53,800,484	0.00348	0.298	0.315				*TMEM100*	TSS1500
cg18070676	14	85,997,519	0.000634	0.298	0.328				*FLRT2*	5'UTR
cg17177632	2	131,512,961	0.004244	0.298	0.281				*FAM123C*	TSS1500
cg17972013	5	9,547,468	1.37E-15	0.298	0.260				*SNORD123*	TSS1500
cg22881435	8	37,732,086	2.97E-18	0.298	0.267				*RAB11FIP1*	Body
cg05690644	8	97,158,015	2.54E-09	0.298	0.303				*GDF6*	Body
cg13934625	15	52,472,770	6.65E-05	0.298	0.208				*GNB5*	TSS1500
cg20191066	17	56,413,977	1.42E-08	0.298	0.297					
cg03512637	6	33,215,685	6.9E-09	0.298	0.308					
cg02413040	21	36,419,648	4.71E-06	0.298	0.260				*RUNX1*	Body
cg08477106	4	75,895,596	3.2E-24	0.298	0.277				*PARM1*	Body
cg11334165	5	145,758,782	1.45E-20	0.298	0.283					

cg18412777	X	133,680,665	0.008168	0.298	0.238				*MGC16121*	TSS200
cg25786366	22	24,817,597	1.17E-07	0.298	0.316					
cg02527527	22	38,303,310	1.36E-12	0.298	0.243				*MICALL1*	Body
cg00376553	7	100,073,281	4.68E-29	0.298	0.310				*TSC22D4*	Body
cg23874437	19	38,886,110	1.32E-11	0.298	0.296				*SPRED3*	Body
cg19103169	7	1,280,040	0.000315	0.298	0.323					
cg26113512	3	38,035,601	0.022213	0.298	0.127	6.78E-09	0.188		*VILL*	Body
cg27031435	15	37,403,184	0.0226	0.298	0.208					
cg15280358	10	127,904,472	4.37E-12	0.298	0.286				*ADAM12*	Body
cg11365617	12	110,174,620	1.53E-08	0.298	0.290				*MGC14436*	Body
cg12149299	12	96,762,309	5.53E-15	0.298	0.303				*CDK17*	5'UTR
cg14885762	17	75,446,450	6.08E-09	0.298	0.224				*SEPT9*	TSS200
cg23722334	X	106,515,943	0.000605	0.298	0.330					
cg11386011	6	32,121,156	8.22E-07	0.298	0.329				*PPT2*	TSS200
cg02541592	22	45,608,686	1.27E-07	0.298	0.264				*C22orf9*	Body
cg14227486	4	41,867,415	4.61E-06	0.298	0.321					
cg07216036	12	132,950,611	0.049407	0.298	0.343					
cg13291283	17	10,101,195	0.009199	0.298	0.229				*GAS7*	Body
cg01187920	5	71,015,162	3.29E-07	0.298	0.272				*CARTPT*	1stExon
cg04166042	1	50,896,246	2.23E-08	0.298	0.267					
cg03628000	16	14,596,062	8.1E-11	0.298	0.334				*PARN*	Body
cg09444392	5	179,781,245	0.001856	0.298	0.268				*GFPT2*	TSS1500
cg18665334	2	231,735,434	3.32E-13	0.298	0.282				*ITM2C*	Body
cg16353006	1	197,882,469	0.002122	0.298	0.318				*LHX9*	Body
cg19869035	7	2,653,955	7.59E-09	0.298	0.261				*IQCE*	3'UTR
cg07723510	6	30,228,063	8.59E-05	0.297	0.289				*HLA-L*	Body
cg00497232	4	6,665,905	1.8E-10	0.297	0.288					
cg03210314	16	22,959,594	5.03E-05	0.297	0.286					
cg20323175	12	68,758,857	1.28E-05	0.297	0.261					
cg00797821	16	49,888,944	0.023614	0.297	0.209					
cg03847716	22	30,938,483	0.017805	0.297	0.210					
cg13571479	7	2,077,345	3.63E-13	0.297	0.322				*MAD1L1*	Body
cg20151221	11	325,030	3.36E-14	0.297	0.279					
cg08369368	5	176,559,881	6.92E-08	0.297	0.286				*NSD1*	TSS200

cg08402365	2	172,945,274	6.12E-08	0.297	0.255				*MAP1D*	3'UTR
cg09746279	6	29,895,016	5.5E-05	0.297	0.276					
cg22777979	11	134,283,252	2.93E-08	0.297	0.313				*B3GAT1*	TSS1500
cg17319007	14	92,414,300	0.024282	0.297	0.127				*FBLN5*	TSS1500
cg05495546	5	113,390,551	3.89E-12	0.297	0.285					
cg08291098	20	62,285,239	6.38E-05	0.297	0.202	1.2E-16	0.133		*STMN3*	TSS1500
cg14045872	7	49,813,065	0.049439	0.297	0.340				*VWC2*	TSS200
cg08929399	3	151,778,972	4.62E-13	0.297	0.267					
cg03407747	17	6,899,364	9.12E-07	0.297	0.234				*ALOX12*	TSS200
cg04223044	22	45,608,428	2.9E-05	0.297	0.264				*C22orf9*	Body
cg26453588	22	43,506,021	5.16E-20	0.297	0.276	5.36E-26	0.234		*BIK*	TSS1500
cg05310249	8	23,560,590	0.008482	0.297	0.226				*NKX2-6*	Body
cg02232839	1	156,404,965	2.39E-05	0.297	0.341					
cg03149565	4	109,092,769	0.041636	0.297	0.365				*LOC641518*	TSS1500
cg23287547	15	52,820,479	0.000406	0.297	0.175	0.00000187	0.086		*MYO5A*	Body
cg00875805	4	140,656,749	4.57E-06	0.297	0.299				*MAML3*	Body
cg04219946	7	87,104,724	1.77E-05	0.297	0.238				*ABCB4*	Body
cg13691247	12	54,071,090	0.015172	0.297	0.269				*ATP5G2*	TSS1500
cg06802522	8	124,173,231	0.000657	0.297	0.256					
cg09142578	17	79,140,486	2.05E-05	0.297	0.236				*AATK*	TSS1500
cg17495250	5	2,727,907	0.000265	0.297	0.300					
cg24115040	7	96,652,375	7.95E-05	0.297	0.279	2.62E-15	0.252		*DLX5*	Body
cg02640604	16	29,690,271	3.92E-29	0.297	0.315				*QPRT*	TSS200
cg06357561	17	40,439,501	7.77E-10	0.297	0.268				*STAT5A*	TSS200
cg07263235	8	19,796,394	1.77E-05	0.297	0.228				*LPL*	TSS200
cg09157176	11	44,599,232	8.22E-10	0.297	0.239				*CD82*	5'UTR
cg14630206	11	77,121,864	7.42E-15	0.297	0.290				*PAK1*	5'UTR
cg15070894	6	29,894,162	3.73E-09	0.297	0.291				*HCG4P6*	TSS1500
cg25735648	15	35,998,009	1.11E-08	0.297	0.318					
cg27272235	1	221,061,070	1.49E-05	0.297	0.224					
cg22627841	17	48,546,258	1.95E-06	0.297	0.273				*CHAD*	TSS200
cg00514609	11	111,783,348	0.001425	0.297	0.379				*CRYAB*	TSS1500
cg19113530	2	178,257,923	0.006783	0.297	0.201				*LOC100130691*	TSS1500
cg14346208	11	35,547,548	0.002283	0.297	0.276				*PAMR1*	TSS1500

cg08041308	17	73,028,425	0.001286	0.297	0.243					
cg11231958	1	9,509,421	7.2E-06	0.297	0.277					
cg19604907	12	50,444,723	2.16E-06	0.297	0.252					
cg07394266	9	71,155,970	2.5E-19	0.297	0.285				*C9orf71*	TSS200
cg22753382	4	3,660,005	0.007922	0.297	0.138					
cg10020356	17	7,906,042	0.004894	0.297	0.364				*GUCY2D*	1stExon
cg08901901	1	160,951,907	0.002222	0.297	0.295					
cg11097433	10	108,924,557	0.003408	0.297	0.369				*SORCS1*	TSS1500
cg00109344	9	116,344,532	2.27E-12	0.297	0.289				*RGS3*	Body
cg01233722	14	24,838,126	4.58E-13	0.297	0.256				*NFATC4*	Body
cg17142183	2	102,608,192	5.85E-06	0.297	0.265	2.96E-11	0.262		*IL1R2*	TSS200
cg25058482	2	161,993,315	4.8E-05	0.297	0.292				*TANK*	TSS200
cg03216846	1	119,543,883	1.64E-09	0.297	0.268					
cg04176888	19	41,596,066	3.23E-05	0.297	0.305				*CYP2A13*	Body
cg14381908	10	43,697,817	1.26E-07	0.297	0.261			Yes	*RASGEF1A*	Body
cg11590053	6	41,343,459	0.02671	0.297	0.421					
cg02305377	12	103,355,958	0.028028	0.297	0.281					
cg06911110	1	17,293,971	2.27E-17	0.297	0.281				*CROCC*	Body
cg23499956	1	153,584,920	1.88E-12	0.297	0.315	8.93E-32	0.168		*S100A16*	5'UTR
cg24293507	1	10,511,793	4.95E-06	0.297	0.243				*APITD1*	3'UTR
cg26337998	5	140,749,783	0.011449	0.297	0.371				*PCDHGA4*	Body
cg07372520	1	180,086,434	6.15E-38	0.297	0.273					
cg08409482	X	132,551,364	0.000467	0.297	0.265					
cg01414185	2	177,017,449	2.3E-08	0.297	0.235				*HOXD4*	Body
cg00982919	9	126,775,263	8.85E-12	0.297	0.314				*LHX2*	Body
cg18438944	16	56,672,525	7.13E-06	0.297	0.293				*MT1A*	TSS200
cg09981361	8	109,206,108	7.51E-07	0.297	0.307					
cg04908077	3	150,187,338	7.24E-11	0.297	0.343					
cg15261247	7	129,425,731	9.09E-06	0.296	0.297					
cg00393825	20	42,137,061	1.29E-05	0.296	0.298					
cg06487301	19	11,531,313	0.000738	0.296	0.300				*RGL3*	TSS1500
cg21642176	2	17,699,740	0.019902	0.296	0.305				*RAD51AP2*	TSS200
cg01774894	17	46,674,395	4.75E-06	0.296	0.289				*LOC404266*	Body
cg20744163	10	80,999,841	4.3E-15	0.296	0.308				*ZMIZ1*	Body

cg16446288	7	98,424,232	1.81E-06	0.296	0.294					
cg26855801	2	45,176,983	0.000132	0.296	0.181					
cg20300343	1	149,719,461	3.5E-08	0.296	0.303					
cg07157830	1	236,228,741	0.015728	0.296	0.116				*NID1*	TSS1500
cg25947878	2	20,068,702	0.018267	0.296	0.155					
cg06609866	3	57,929,568	4.73E-27	0.296	0.282					
cg07483244	13	95,358,924	3.88E-06	0.296	0.267					
cg27416337	3	32,748,004	9.49E-14	0.296	0.307				*CNOT10*	Body
cg11935153	6	30,923,306	0.012445	0.296	0.373					
cg08280468	16	12,997,534	4.11E-09	0.296	0.289				*SHISA9*	Body
cg21655710	1	54,856,511	4.7E-12	0.296	0.269				*SSBP3*	Body
cg23901918	10	105,420,747	2.22E-10	0.296	0.232				*SH3PXD2A*	Body
cg27252696	6	100,912,940	0.012425	0.296	0.358				*SIM1*	TSS1500
cg02779016	15	43,809,689	3.2E-08	0.296	0.275				*MAP1A*	TSS200
cg09237846	12	105,114,399	1.54E-08	0.296	0.277				*CHST11*	Body
cg06775523	1	3,514,921	1.71E-09	0.296	0.291				*MEGF6*	Body
cg18337363	3	52,569,053	1.92E-09	0.296	0.234				*NT5DC2*	5'UTR
cg23752000	17	78,442,298	0.000436	0.296	0.236				*NPTX1*	3'UTR
cg25046720	4	183,801,938	3.83E-09	0.296	0.328					
cg00612595	21	47,717,864	1.35E-09	0.296	0.294					
cg04293307	17	63,553,581	1.08E-09	0.296	0.235				*AXIN2*	Body
cg16640855	2	182,543,233	0.001794	0.296	0.228				*NEUROD1*	Body
cg02640104	13	30,476,615	3.34E-10	0.296	0.305					
cg11288144	6	30,649,909	1.17E-05	0.296	0.312				*KIAA1949*	Body
cg24268343	X	56,260,186	2.66E-15	0.296	0.298				*KLF8*	Body
cg17237063	3	29,323,182	0.002452	0.296	0.342	1.37E-20	0.200		*RBMS3*	1stExon
cg02059626	8	76,319,264	1.18E-07	0.296	0.276					
cg09505809	1	180,123,222	4.74E-12	0.296	0.278				*QSOX1*	TSS1500
cg22481950	6	161,395,786	4.43E-10	0.296	0.305					
cg15984661	19	46,916,520	6.3E-05	0.296	0.310	1.92E-10	0.142		*CCDC8*	1stExon
cg06590173	19	16,189,360	0.001015	0.296	0.266				*TPM4*	Body
cg23571457	X	101,907,558	4.15E-13	0.296	0.285	8.52E-29	0.204		*GPRASP1*	5'UTR
cg15754153	2	43,865,055	2.56E-05	0.296	0.304				*PLEKHH2*	5'UTR
cg19381811	3	49,851,713	1.77E-14	0.296	0.287				*UBA7*	TSS1500

cg13297960	21	22,370,237	2.24E-05	0.296	0.311				*NCAM2*	TSS1500
cg10616216	11	128,321,662	3.31E-24	0.296	0.296					
cg12608692	5	16,742,020	3.01E-26	0.296	0.291				*MYO10*	Body
cg21781979	13	100,547,292	0.001642	0.296	0.216				*CLYBL*	3'UTR
cg03220945	1	205,537,877	0.000133	0.296	0.179				*MFSD4*	TSS1500
cg10521153	1	91,307,077	0.000102	0.296	0.362					
cg26917694	1	221,061,058	6.51E-05	0.296	0.227					
cg00930873	15	58,357,973	9.93E-06	0.296	0.293	2.57E-15	0.159		*ALDH1A2*	TSS200
cg04270048	20	33,296,729	3.62E-10	0.296	0.211				*TP53INP2*	Body
cg04242021	8	121,137,288	0.002025	0.296	0.243				*COL14A1*	TSS200
cg09969277	15	89,922,328	2.88E-05	0.296	0.265				*LOC254559*	Body
cg10043253	17	6,616,425	0.002604	0.296	0.346				*SLC13A5*	Body
cg26175971	2	219,647,167	5.54E-05	0.296	0.202				*CYP27A1*	Body
cg23031975	2	67,601,521	5.11E-24	0.296	0.248					
cg25150953	4	41,540,229	2.16E-11	0.296	0.314				*LIMCH1*	Body
cg00670942	17	74,836,683	2.45E-07	0.296	0.316					
cg05917460	14	37,126,902	5.62E-09	0.296	0.300				*PAX9*	1stExon
cg19853364	2	99,082,357	4.85E-18	0.296	0.278				*INPP4A*	5'UTR
cg08551725	7	30,029,776	0.014143	0.296	0.141				*SCRN1*	TSS1500
cg20584157	3	64,149,428	2.75E-11	0.296	0.287				*PRICKLE2*	Body
cg17718302	6	27,858,637	0.000317	0.296	0.321	5.02E-20	0.228		*HIST1H3J*	TSS200
cg09608652	16	53,105,688	1.18E-10	0.296	0.278				*CHD9*	5'UTR
cg12535476	6	55,691,833	3.15E-17	0.296	0.244				*BMP5*	Body
cg27616661	16	2,040,981	0.00467	0.296	0.390				*SYNGR3*	Body
cg22410382	X	134,124,941	0.008155	0.296	0.299				*LOC644538*	TSS200
cg27315157	1	79,086,197	0.000158	0.296	0.185				*IFI44L*	5'UTR
cg01901262	16	54,966,837	0.002266	0.296	0.339				*IRX5*	Body
cg01937840	5	11,588,971	6.68E-08	0.296	0.294				*CTNND2*	Body
cg03753331	2	115,919,006	0.014375	0.296	0.302				*DPP10*	TSS1500
cg26667946	13	46,425,874	0.00099	0.296	0.269				*SIAH3*	TSS200
cg01638193	14	68,571,056	1.93E-16	0.296	0.297				*RAD51L1*	Body
cg23795217	12	54,812,000	1.31E-06	0.296	0.287				*ITGA5*	Body
cg08164484	1	58,713,941	1.83E-07	0.296	0.266				*DAB1*	5'UTR
cg19755318	3	50,243,323	1.24E-09	0.296	0.257				*SLC38A3*	5'UTR

cg07921371	13	20,768,536	6.23E-08	0.296	0.264				*GJB2*	TSS1500
cg04056576	3	160,475,090	1.06E-09	0.296	0.296				*PPM1L*	Body
cg10712173	4	8,271,414	0.001263	0.296	0.237				*HTRA3*	TSS200
cg17214107	4	123,747,542	4.71E-09	0.296	0.276				*FGF2*	TSS1500
cg01141459	22	30,476,452	1.08E-12	0.296	0.292				*HORMAD2*	1stExon
cg09282497	2	18,059,200	0.021661	0.296	0.164				*KCNS3*	TSS1500
cg12972233	9	91,605,893	1.51E-19	0.296	0.278				*C9orf47*	1stExon
cg25594899	1	1,182,226	0.035813	0.295	0.380				*FAM132A*	TSS200
cg15616310	11	94,570,647	1.73E-18	0.295	0.287				*AMOTL1*	Body
cg10789281	12	81,471,882	0.000153	0.295	0.280				*ACSS3*	1stExon
cg05447023	15	40,531,952	3.39E-07	0.295	0.290				*PAK6*	5'UTR
cg15678825	18	77,266,975	3.86E-13	0.295	0.266				*NFATC1*	Body
cg04257984	2	171,568,984	0.002482	0.295	0.211				*LOC440925*	Body
cg25756780	2	121,345,047	1.39E-33	0.295	0.274					
cg00986824	12	75,601,465	0.001374	0.295	0.234				*KCNC2*	Body
cg21301805	1	119,534,644	4.54E-07	0.295	0.332					
cg03199651	4	4,862,770	9.17E-08	0.295	0.284				*MSX1*	Body
cg08003102	4	88,451,496	2.75E-10	0.295	0.284				*SPARCL1*	TSS1500
cg04602939	20	62,184,207	2.36E-05	0.295	0.314				*C20orf195*	TSS200
cg16188209	12	103,311,596	0.002123	0.295	0.151				*PAH*	TSS1500
cg10547969	17	35,084,975	0.003158	0.295	0.298					
cg18926212	12	50,297,936	0.002096	0.295	0.261				*FAIM2*	TSS1500
cg20907456	11	57,405,372	3.37E-24	0.295	0.270					
cg25761326	10	11,206,792	0.006128	0.295	0.183				*CUGBP2*	TSS200
cg11480627	11	70,672,876	1.9E-16	0.295	0.273				*SHANK2*	Body
cg01363736	20	37,172,289	9.23E-11	0.295	0.267				*RALGAPB*	Body
cg23480730	14	33,402,027	5.56E-07	0.295	0.292					
cg15360478	2	43,094,440	1.93E-22	0.295	0.290					
cg20024403	17	81,039,326	0.000165	0.295	0.236				*METRNL*	Body
cg23172664	6	86,169,204	6.04E-10	0.295	0.305				*NT5E*	Body
cg02792740	6	2,876,395	0.000444	0.295	0.303					
cg12996913	3	72,338,454	0.030293	0.295	0.137					
cg07537443	17	47,572,132	2.65E-32	0.295	0.255				*NGFR*	TSS1500
cg03861427	6	29,855,325	2.09E-15	0.295	0.265				*HLA-H*	TSS200

cg05890484	5	78,407,552	1.63E-11	0.295	0.285	1.74E-24	0.238		*BHMT*	TSS200
cg23049458	1	62,660,624	0.001249	0.295	0.282				*L1TD1*	5'UTR
cg01513307	6	108,479,557	0.001302	0.295	0.293					
cg09070799	6	151,561,236	0.000111	0.295	0.173				*AKAP12*	5'UTR
cg18793404	7	52,156,489	6.39E-05	0.295	0.248					
cg22877570	3	171,175,950	5.98E-07	0.295	0.328				*TNIK*	Body
cg12198841	5	115,941,397	5.77E-19	0.295	0.275					
cg13921605	9	133,808,174	2.18E-12	0.295	0.307				*FIBCD1*	Body
cg25193276	1	41,901,646	1.79E-11	0.295	0.283					
cg14097568	1	94,792,942	2.13E-08	0.295	0.235					
cg01968657	1	237,207,256	0.016064	0.295	0.197				*RYR2*	Body
cg25165701	3	151,985,697	4.98E-08	0.295	0.299				*LOC401093*	Body
cg12345672	7	127,672,658	1.98E-05	0.295	0.305				*SND1*	Body
cg01661350	13	70,682,098	0.00127	0.295	0.284				*KLHL1*	1stExon
cg06152586	7	157,890,659	1.28E-13	0.295	0.266			Yes	*PTPRN2*	Body
cg17545346	6	28,956,609	1.85E-08	0.295	0.304					
cg18702689	4	110,223,138	0.004805	0.295	0.350				*COL25A1*	Body
cg26075747	6	30,457,407	3.02E-16	0.295	0.289				*HLA-E*	Body
cg10320410	7	64,343,315	0.023315	0.295	0.166					
cg16849268	8	125,721,998	0.000241	0.295	0.240				*MTSS1*	Body
cg10418289	14	23,308,294	1.62E-16	0.295	0.294				*MMP14*	Body
cg20216655	6	2,665,911	3.5E-06	0.295	0.250				*MYLK4*	3'UTR
cg22867816	4	16,081,205	8.37E-23	0.295	0.277				*PROM1*	5'UTR
cg01630690	14	36,992,001	0.000328	0.295	0.368					
cg04713531	7	158,197,288	4.17E-12	0.295	0.294				*PTPRN2*	Body
cg10879397	5	148,555,266	5.54E-21	0.295	0.288				*ABLIM3*	Body
cg13759852	17	8,056,985	1.55E-20	0.295	0.299				*PER1*	TSS1500
cg26234658	7	36,343,787	4.3E-16	0.295	0.258					
cg15701237	3	187,086,154	0.006807	0.295	0.115				*RTP4*	TSS200
cg12120292	17	3,867,227	0.002628	0.295	0.253				*ATP2A3*	Body
cg02773086	2	177,027,440	0.001214	0.295	0.181				*HOXD3*	TSS1500
cg14458932	20	45,439,805	3.85E-08	0.295	0.317					
cg17853216	10	81,002,480	0.041006	0.295	0.175				*ZMIZ1*	Body
cg25885684	15	100,048,426	1.25E-06	0.295	0.241					

cg09363604	4	83,675,306	1.22E-07	0.295	0.302				*MIR575*	TSS1500
cg05624862	16	1,349,657	0.002491	0.295	0.302					
cg05735180	18	55,095,177	0.01169	0.295	0.303					
cg23878906	18	29,304,818	1.42E-07	0.295	0.269					
cg13525428	2	129,076,361	3.55E-07	0.295	0.254				*HS6ST1*	TSS200
cg15103584	18	56,943,576	3.61E-05	0.295	0.319					
cg26987855	6	10,426,637	0.001708	0.295	0.396					
cg01942226	4	154,218,350	1.53E-08	0.295	0.249				*TRIM2*	Body
cg17820459	5	150,400,531	0.000176	0.295	0.143	1.02E-14	0.197		*GPX3*	Body
cg00369811	6	37,673,597	0.012216	0.295	0.309					
cg08619932	19	49,200,058	7.54E-06	0.295	0.293				*FUT2*	5'UTR
cg12829045	10	77,228,852	8.65E-08	0.295	0.295					
cg14838248	11	64,147,847	3.39E-31	0.295	0.287					
cg17550566	7	2,681,436	1.77E-16	0.295	0.294				*TTYH3*	Body
cg23625660	5	135,266,516	6.26E-09	0.295	0.250				*FBXL21*	5'UTR
cg07785447	14	95,235,127	0.00823	0.295	0.336				*GSC*	Body
cg10851413	10	99,532,174	3.91E-05	0.295	0.304				*SFRP5*	TSS1500
cg13104938	11	843,956	2.79E-09	0.295	0.235				*TSPAN4*	TSS1500
cg08069249	4	6,676,075	2.29E-19	0.294	0.301				*LOC93622*	Body
cg01461552	11	20,692,867	0.000965	0.294	0.275				*NELL1*	Body
cg20281962	10	8,089,733	5.2E-05	0.294	0.272					
cg07507251	3	52,567,010	0.000604	0.294	0.147				*NT5DC2*	Body
cg15396686	10	124,897,048	0.000292	0.294	0.273				*HMX3*	Body
cg18660345	20	35,169,539	2.85E-09	0.294	0.284				*MYL9*	TSS1500
cg09803951	6	29,910,796	5.89E-07	0.294	0.289				*HLA-A*	Body
cg23174932	6	31,831,502	2.64E-06	0.294	0.257				*NEU1*	TSS1500
cg08925720	11	61,314,936	1.36E-65	0.294	0.291				*SYT7*	Body
cg09550810	1	61,519,890	0.000161	0.294	0.226					
cg13756380	9	135,455,174	0.000109	0.294	0.302					
cg22723502	6	26,240,528	0.023424	0.294	0.343				*HIST1H4F*	TSS200
cg02649547	10	95,848,791	4.46E-06	0.294	0.270				*PLCE1*	TSS200
cg04906283	14	77,965,859	3.57E-10	0.294	0.270				*ISM2*	TSS1500
cg05135644	6	108,489,795	0.000105	0.294	0.312				*NR2E1*	Body
cg14549256	4	2,627,142	2.95E-19	0.294	0.305				*FAM193A*	TSS200

cg07099331	10	102,419,617	0.000716	0.294	0.305					
cg13799941	7	96,632,294	2.84E-25	0.294	0.289				*DLX6AS*	Body
cg11423684	6	29,691,993	4.82E-05	0.294	0.215				*HLA-F*	Body
cg13605988	15	45,421,612	0.008974	0.294	0.349				*DUOX1*	TSS1500
cg08072992	19	14,229,581	0.000329	0.294	0.260				*PRKACA*	TSS1500
cg11609001	19	18,901,843	0.004802	0.294	0.256				*COMP*	Body
cg16300637	14	97,059,005	0.003975	0.294	0.234					
cg01515543	9	92,254,510	2.11E-10	0.294	0.281				*LOC100129066*	TSS200
cg04609635	2	24,398,695	3.25E-06	0.294	0.247				*C2orf84*	Body
cg07766263	3	170,303,045	6.57E-17	0.294	0.289				*SLC7A14*	5'UTR
cg12406391	1	6,520,194	1.01E-07	0.294	0.280				*ESPN*	Body
cg13888593	2	74,642,470	0.000206	0.294	0.288				*C2orf81*	Body
cg10008757	10	8,097,183	0.00038	0.294	0.314				*GATA3*	5'UTR
cg14615784	5	94,955,756	0.009626	0.294	0.229				*GPR150*	TSS1500
cg26245697	19	2,282,203	0.024103	0.294	0.245				*C19orf35*	TSS200
cg15879316	22	46,934,089	0.000388	0.294	0.166	1.94E-14	0.077		*CELSR1*	TSS1500
cg13338798	6	28,557,846	3.02E-06	0.294	0.283					
cg20467412	19	5,799,340	0.000847	0.294	0.348					
cg20061654	22	45,608,492	9.54E-08	0.294	0.273				*C22orf9*	Body
cg10208485	3	350,602	0.02091	0.294	0.256				*CHL1*	5'UTR
cg12642693	X	50,556,917	0.030655	0.294	0.304				*SHROOM4*	1stExon
cg01246520	17	17,644,344	1.53E-05	0.294	0.278				*RAI1*	5'UTR
cg00959249	5	95,768,997	0.016457	0.294	0.303				*PCSK1*	TSS200
cg17063929	11	89,224,799	7.16E-10	0.294	0.265	5.11E-30	0.241		*NOX4*	5'UTR
cg19507206	10	103,044,008	0.001368	0.294	0.292					
cg24751894	6	29,894,141	4.21E-08	0.294	0.286				*HCG4P6*	TSS1500
cg00748938	14	94,641,781	0.037635	0.294	0.223				*PPP4R4*	Body
cg09450367	2	11,294,738	8.1E-05	0.294	0.273				*PQLC3*	TSS1500
cg00668150	17	2,615,720	2.59E-05	0.294	0.239				*KIAA0664*	TSS1500
cg23405212	2	208,989,382	0.000204	0.294	0.277				*CRYGD*	TSS200
cg09912512	22	42,761,285	1.48E-20	0.294	0.281					
cg09980176	19	11,532,175	7.55E-08	0.294	0.293				*CCDC151*	Body
cg15736165	15	83,951,732	4.81E-11	0.294	0.296	4.1E-21	0.182		*BNC1*	Body
cg14913216	8	145,033,104	8.61E-16	0.294	0.297				*PLEC1*	Body

cg22471255	22	42,315,642	0.020703	0.294	0.294					
cg24357619	15	37,389,384	0.000756	0.294	0.333				*MEIS2*	Body
cg11096993	11	67,417,958	1.3E-11	0.294	0.283	4.56E-24	0.200		*ACY3*	5'UTR
cg00174851	7	134,142,981	7.27E-30	0.294	0.271				*AKR1B1*	Body
cg12232388	12	125,027,851	3.15E-07	0.294	0.270					
cg06917763	7	130,033,247	1.64E-22	0.294	0.295					
cg11588197	11	128,391,494	1.19E-05	0.294	0.251				*ETS1*	Body
cg02161900	10	28,035,631	5.56E-10	0.294	0.306	5.61E-27	0.236		*MKX*	TSS1500
cg03238359	11	60,623,567	6.67E-17	0.294	0.262				*GPR44*	TSS200
cg08833675	8	10,586,790	2.3E-09	0.294	0.285				*SOX7*	Body
cg00829858	2	179,148,245	1.43E-19	0.294	0.284				*OSBPL6*	5'UTR
cg10286983	6	24,494,717	2.56E-08	0.294	0.199				*ALDH5A1*	TSS1500
cg00046499	3	154,146,183	0.000294	0.294	0.276				*GPR149*	Body
cg02104162	6	4,610,716	5.31E-06	0.294	0.240					
cg09258804	17	59,532,564	1.81E-08	0.294	0.258				*TBX4*	TSS1500
cg08906194	9	34,577,972	0.000909	0.294	0.305				*CNTFR*	5'UTR
cg13726682	17	29,718,306	0.00037	0.294	0.245				*RAB11FIP4*	TSS1500
cg03018496	15	96,868,989	1.08E-06	0.294	0.239				*NR2F2*	TSS200
cg12929027	12	110,150,180	1.79E-13	0.294	0.309					
cg26371320	6	28,778,091	0.004059	0.294	0.131					
cg24940967	3	45,837,197	0.002608	0.294	0.210				*SLC6A20*	Body
cg00126034	17	44,896,162	1.33E-05	0.294	0.319	2.0478E-05	0.111		*WNT3*	TSS200
cg07112337	16	57,917,946	0.009113	0.294	0.247				*CNGB1*	3'UTR
cg20113824	8	70,946,946	0.001182	0.294	0.296					
cg10050661	17	38,821,420	0.002743	0.294	0.266				*KRT222*	TSS200
cg10856045	10	73,848,463	1.41E-12	0.294	0.236				*SPOCK2*	5'UTR
cg12145907	5	140,864,834	6.42E-09	0.294	0.280	1.28E-08	0.121		*PCDHGA4*	Body
cg21374048	17	56,013,560	1.23E-18	0.294	0.289					
cg07974504	2	220,325,375	4.35E-19	0.294	0.289				*SPEG*	Body
cg05303057	17	54,756,052	0.000799	0.294	0.343					
cg18953104	6	139,613,145	5.54E-10	0.294	0.300				*TXLNB*	5'UTR
cg05215748	17	6,898,738	7.14E-13	0.294	0.291				*ALOX12*	TSS1500
cg07026666	1	204,050,380	2.8E-06	0.294	0.315				*SOX13*	5'UTR
cg10965575	2	182,321,496	1.65E-07	0.294	0.226				*ITGA4*	TSS200

cg00848728	1	58,716,018	6.13E-07	0.294	0.243	2.52E-32	0.287		*DAB1*	5'UTR
cg17536166	3	44,626,697	0.000185	0.294	0.238				*ZNF660*	5'UTR
cg07687119	12	54,423,549	9.03E-07	0.294	0.261				*HOXC4*	5'UTR
cg24717695	7	156,809,644	2.03E-05	0.294	0.285					
cg21327194	17	72,443,018	1.56E-14	0.294	0.295				*GPRC5C*	Body
cg10783371	3	181,437,548	9.47E-06	0.294	0.344				*SOX2OT*	Body
cg00199007	20	61,583,910	2.14E-08	0.294	0.293				*SLC17A9*	TSS200
cg20881942	4	154,710,826	0.004043	0.293	0.374				*SFRP2*	TSS1500
cg22560083	21	36,038,533	2.16E-21	0.293	0.280					
cg23518541	3	25,472,652	1.56E-11	0.293	0.314				*RARB*	5'UTR
cg02001390	19	5,338,977	3.94E-06	0.293	0.326				*PTPRS*	5'UTR
cg01821113	2	218,277,531	1.24E-18	0.293	0.285				*DIRC3*	Body
cg13306921	1	54,863,529	1.05E-18	0.293	0.270				*SSBP3*	Body
cg14335894	17	47,075,281	0.000419	0.293	0.277				*IGF2BP1*	1stExon
cg00687714	16	21,272,887	9.29E-14	0.293	0.308				*CRYM*	Body
cg14940165	2	177,021,702	6.87E-06	0.293	0.283					
cg18032164	3	129,024,676	9.59E-20	0.293	0.271					
cg14702570	2	74,259,524	3.2E-11	0.293	0.296					
cg06241101	17	77,895,684	9.23E-05	0.293	0.279					
cg16234557	10	119,292,062	0.027478	0.293	0.235				*EMX2OS*	Body
cg27165884	10	99,473,085	5.46E-07	0.293	0.337				*MARVELD1*	TSS1500
cg11424376	1	230,220,668	1.47E-20	0.293	0.279				*GALNT2*	Body
cg26027233	X	75,648,961	0.04418	0.293	0.161				*MAGEE1*	1stExon
cg02693127	4	148,652,654	0.000379	0.293	0.298				*ARHGAP10*	TSS1500
cg27543103	4	54,975,677	1.79E-07	0.293	0.340					
cg25580581	10	3,853,668	3.7E-06	0.293	0.335					
cg08574629	1	155,290,542	1.88E-07	0.293	0.238				*RUSC1*	TSS200
cg01837410	5	101,631,990	0.000422	0.293	0.170				*SLCO4C1*	1stExon
cg10802379	5	150,400,395	0.003552	0.293	0.117				*GPX3*	Body
cg04611203	20	2,781,438	9.68E-06	0.293	0.198				*CPXM1*	TSS200
cg14700679	2	9,898,228	5.82E-10	0.293	0.280					
cg15443822	17	3,867,014	1.51E-05	0.293	0.264	5.15E-27	0.200		*ATP2A3*	Body
cg21762523	6	108,497,717	7.51E-05	0.293	0.296				*NR2E1*	Body
cg13414212	2	26,624,823	0.020736	0.293	0.097				*C2orf39*	5'UTR

cg25191628	1	50,886,949	0.000151	0.293	0.289				*DMRTA2*	Body
cg17370981	13	114,311,583	0.043113	0.293	0.130			Yes	*ATP4B*	Body
cg05224741	6	10,422,322	6.69E-05	0.293	0.299					
cg06896987	2	177,003,735	5.11E-07	0.293	0.236					
cg17945789	2	236,444,284	0.002644	0.293	0.248				*AGAP1*	Body
cg04131799	2	97,193,006	2.99E-11	0.293	0.298					
cg09926486	15	44,486,207	1.25E-06	0.293	0.241				*FRMD5*	Body
cg07511668	11	68,622,177	1.61E-08	0.293	0.306					
cg13640071	X	102,585,147	6.96E-08	0.293	0.306				*TCEAL7*	TSS200
cg18193764	5	134,879,961	1.1E-06	0.293	0.307					
cg05491854	6	24,910,562	3.55E-05	0.293	0.299				*FAM65B*	5'UTR
cg26983835	17	59,481,280	2.53E-10	0.293	0.293				*TBX2*	Body
cg02639608	1	68,150,326	6.96E-09	0.293	0.237				*GADD45A*	TSS1500
cg07687548	2	172,965,280	0.001325	0.293	0.389				*DLX2*	Body
cg11953272	1	53,067,965	1.29E-05	0.293	0.157				*GPX7*	TSS200
cg24250374	1	184,591,081	7.76E-34	0.293	0.286				*C1orf21*	3'UTR
cg01471428	12	115,133,663	1.7E-18	0.293	0.303					
cg22376864	19	42,497,705	7.14E-12	0.293	0.261				*ATP1A3*	Body
cg17276590	10	119,304,487	0.012131	0.293	0.305				*EMX2OS*	Body
cg01392518	6	166,583,226	3.27E-05	0.293	0.282				*T*	TSS1500
cg09465142	20	50,109,375	4.6E-17	0.293	0.284				*NFATC2*	Body
cg18736101	10	126,043,549	0.000465	0.293	0.201					
cg08860070	5	140,892,822	3.13E-09	0.293	0.188					
cg16499656	7	50,344,471	0.018511	0.293	0.301				*IKZF1*	1stExon
cg14317609	9	36,986,006	0.00985	0.293	0.314				*PAX5*	Body
cg08815340	6	5,026,435	0.007358	0.293	0.292					
cg16487653	16	73,200,705	2.91E-08	0.293	0.260					
cg08848958	16	1,384,655	1.4E-06	0.293	0.286				*BAIAP3*	TSS200
cg13558971	1	203,597,085	0.00231	0.293	0.376				*ATP2B4*	5'UTR
cg15647296	11	77,751,173	0.003972	0.293	0.201					
cg13553997	6	42,161,424	1.99E-07	0.293	0.258				*GUCA1B*	Body
cg11142826	17	44,896,223	0.014432	0.293	0.417				*WNT3*	TSS200
cg09726240	11	70,672,878	1.33E-15	0.293	0.273				*SHANK2*	Body
cg07313504	2	208,995,664	4.29E-17	0.293	0.303				*CRYGC*	TSS1500

cg00110832	6	32,121,130	3.37E-08	0.293	0.319				*PPT2*	TSS200
cg03135351	2	29,338,258	0.021235	0.293	0.241				*CLIP4*	TSS200
cg08154107	3	183,994,073	0.008308	0.293	0.316				*ECE2*	Body
cg13932029	9	23,824,172	9.36E-13	0.293	0.322				*ELAVL2*	5'UTR
cg23035602	19	53,935,117	1.1E-05	0.293	0.293				*ZNF761*	TSS200
cg19725903	12	14,721,020	3.52E-11	0.293	0.282				*PLBD1*	TSS1500
cg10807894	12	125,169,804	2.76E-08	0.293	0.307					
cg27254482	21	34,398,085	8.21E-06	0.293	0.256				*OLIG2*	TSS200
cg12828075	11	15,136,150	0.005824	0.293	0.198				*INSC*	Body
cg27558095	1	66,999,588	0.006837	0.293	0.272				*SGIP1*	TSS1500
cg24164564	8	76,317,040	4.19E-06	0.293	0.330					
cg18752854	2	218,809,254	3.38E-30	0.293	0.277	5.34E-16	0.168		*TNS1*	TSS1500
cg19904425	14	94,984,530	4.34E-13	0.293	0.282				*SERPINA12*	TSS1500
cg01965939	5	148,442,694	3.16E-07	0.293	0.241	7.07E-31	0.236		*SH3TC2*	1stExon
cg16683160	1	87,617,710	0.000944	0.293	0.327				*LOC339524*	Body
cg05726239	6	107,816,677	0.02941	0.293	0.180				*SOBP*	Body
cg18135132	11	10,814,092	0.002919	0.293	0.255					
cg24405999	3	133,393,119	0.000703	0.293	0.363					
cg05966241	16	67,515,938	4.25E-13	0.293	0.292				*ATP6V0D1*	TSS1500
cg07963705	10	33,212,865	2.01E-11	0.293	0.278				*ITGB1*	Body
cg08059112	19	2,294,887	7.25E-18	0.293	0.268				*LINGO3*	5'UTR
cg21815063	5	131,800,430	3.92E-08	0.293	0.221					
cg23245720	X	133,680,549	0.035213	0.293	0.331				*MGC16121*	Body
cg06371502	1	197,879,766	0.030224	0.293	0.404					
cg21672292	8	145,025,128	1.39E-09	0.293	0.307				*PLEC1*	Body
cg05667348	10	118,892,581	0.009977	0.293	0.371				*VAX1*	Body
cg08206665	11	64,479,111	0.004132	0.293	0.358				*NRXN2*	Body
cg10926487	17	76,917,906	1.38E-17	0.293	0.278				*TIMP2*	Body
cg14092045	9	116,450,241	0.000123	0.293	0.318					
cg25051341	6	100,061,307	0.000932	0.292	0.270				*PRDM13*	Body
cg14816482	10	12,390,916	0.01034	0.292	0.137				*CAMK1D*	TSS1500
cg14391855	11	66,104,174	9.5E-13	0.292	0.326	2.53E-27	0.112		*RIN1*	TSS200
cg06314333	7	129,425,532	4.27E-07	0.292	0.295					
cg13615338	4	55,094,005	0.020857	0.292	0.361				*PDGFRA*	TSS1500

cg10693390	11	12,411,772	1.16E-12	0.292	0.297				*PARVA*	Body
cg25386234	1	119,542,159	0.000228	0.292	0.310					
cg12034345	6	29,975,427	1.58E-14	0.292	0.258				*HLA-J*	Body
cg08175591	14	101,176,152	0.009659	0.292	0.215					
cg17071446	4	85,402,497	6.41E-06	0.292	0.252					
cg02446647	6	133,562,101	4.12E-08	0.292	0.303				*EYA4*	TSS1500
cg24660670	1	116,371,083	3.08E-08	0.292	0.247					
cg26751792	19	15,580,383	0.000439	0.292	0.287				*PGLYRP2*	Body
cg05092308	2	63,285,365	4.84E-05	0.292	0.294					
cg19744211	14	95,079,224	6.8E-14	0.292	0.296				*SERPINA3*	5'UTR
cg06496344	19	23,253,834	0.000861	0.292	0.341					
cg16297011	4	13,539,023	0.000273	0.292	0.331					
cg06118384	2	149,645,798	5.01E-06	0.292	0.291				*KIF5C*	Body
cg20560075	5	146,257,484	0.017182	0.292	0.330				*PPP2R2B*	Body
cg10537807	17	56,415,478	1.4E-07	0.292	0.322					
cg07813249	4	46,392,253	0.001768	0.292	0.275				*GABRA2*	TSS200
cg04370314	13	100,643,312	0.040964	0.292	0.348					
cg08063911	5	133,451,474	1.35E-14	0.292	0.255				*TCF7*	5'UTR
cg09434193	16	227,233	0.000432	0.292	0.349				*HBA1*	Body
cg02577436	4	8,271,369	1.25E-07	0.292	0.233				*HTRA3*	TSS200
cg09092093	5	58,335,954	1.7E-07	0.292	0.243				*PDE4D*	Body
cg15128226	4	123,747,672	2.13E-09	0.292	0.268				*FGF2*	TSS200
cg16686951	20	44,747,351	6.13E-05	0.292	0.363				*CD40*	Body
cg06659128	X	154,842,535	0.000994	0.292	0.262	2.58E-27	0.242		*TMLHE*	5'UTR
cg00868131	1	54,952,901	0.000923	0.292	0.214					
cg05389236	6	36,635,087	1.21E-12	0.292	0.314					
cg16485975	20	25,065,418	0.038852	0.292	0.198					
cg03654299	12	54,389,264	0.031701	0.292	0.184					
cg04077363	X	102,531,881	0.00022	0.292	0.243				*TCEAL5*	TSS200
cg27531366	1	27,627,058	5.27E-18	0.292	0.279				*WDTC1*	Body
cg17589633	1	203,595,283	3.19E-07	0.292	0.226				*ATP2B4*	TSS1500
cg06905130	2	287,148	3.23E-07	0.292	0.274				*FAM150B*	Body
cg11199046	12	115,136,308	1.96E-24	0.292	0.281					
cg08142344	1	110,210,699	1.46E-06	0.292	0.252				*GSTM2*	5'UTR

cg05617413	12	133,065,912	0.029164	0.292	0.282				*FBRSL1*	TSS1500
cg07494518	21	44,847,608	1.07E-05	0.292	0.235	2.5E-21	0.186		*SIK1*	TSS1500
cg18731398	3	123,414,733	2.7E-15	0.292	0.293				*MYLK*	Body
cg06893296	6	30,095,136	9.05E-19	0.292	0.285					
cg22388634	20	25,058,429	0.023417	0.292	0.352				*VSX1*	Body
cg06356912	3	38,035,642	0.02258	0.292	0.113				*VILL*	Body
cg02998240	1	16,508,668	1.88E-21	0.292	0.273					
cg23986590	2	109,934,728	6.61E-18	0.292	0.283				*SH3RF3*	Body
cg24287239	13	43,148,278	5.42E-06	0.292	0.297				*TNFSF11*	TSS200
cg09422450	16	79,623,618	7.58E-14	0.292	0.256					
cg23217153	2	242,808,611	7.85E-07	0.292	0.292					
cg05614013	19	11,253,754	0.003414	0.292	0.182					
cg11550865	15	59,501,408	5.92E-15	0.292	0.276				*MYO1E*	Body
cg21158411	9	130,689,627	0.003254	0.292	0.244				*PIP5KL1*	Body
cg03610280	10	97,592,418	5.2E-06	0.292	0.240				*ENTPD1*	Body
cg06161600	16	1,393,797	0.001564	0.292	0.205				*BAIAP3*	Body
cg10773698	1	61,519,473	7.41E-06	0.292	0.269					
cg10551329	6	32,120,933	0.001305	0.292	0.320				*PPT2*	TSS1500
cg20025238	7	128,829,789	2.16E-14	0.292	0.253				*SMO*	Body
cg22339338	1	159,891,638	3.64E-27	0.292	0.286				*TAGLN2*	5'UTR
cg06426114	17	6,355,097	1.69E-09	0.292	0.286				*PITPNM3*	3'UTR
cg01056004	10	98,948,259	5.57E-13	0.292	0.286					
cg15428435	14	102,247,681	0.000162	0.292	0.190				*PPP2R5C*	Body
cg19290504	17	3,867,060	2.32E-05	0.292	0.262				*ATP2A3*	Body
cg09396895	7	25,021,067	0.001355	0.292	0.175				*OSBPL3*	TSS1500
cg11682083	5	172,782,844	1.06E-10	0.292	0.320					
cg20592766	2	109,934,860	1.67E-16	0.292	0.277				*SH3RF3*	Body
cg25547580	18	55,103,734	0.003682	0.292	0.323				*ONECUT2*	1stExon
cg11896151	7	223,592	3.13E-17	0.292	0.290				*FAM20C*	Body
cg23064609	2	175,208,594	0.02645	0.292	0.331					
cg06776173	1	46,932,780	8.56E-06	0.292	0.281					
cg13545297	12	54,404,315	5.98E-11	0.292	0.256				*HOXC8*	Body
cg03718183	1	26,231,082	1.14E-18	0.292	0.260				*STMN1*	Body
cg02489958	4	85,417,416	0.000539	0.292	0.308				*NKX6-1*	Body

cg07406897	17	27,347,178	0.014305	0.292	0.094						
cg14343652	7	130,789,013	8.83E-11	0.292	0.353				*FLJ43663*	Body
cg17164954	6	157,345,266	4.35E-19	0.292	0.300				*ARID1B*	Body
cg26929700	16	49,732,567	2.57E-11	0.292	0.278				*ZNF423*	Body
cg05386570	7	99,177,695	1.02E-17	0.292	0.255					
cg19630629	7	136,556,193	6.99E-05	0.292	0.212				*CHRM2*	5'UTR
cg11481582	10	74,080,805	1.42E-12	0.292	0.278					
cg16657340	7	131,242,787	0.003448	0.292	0.195				*PODXL*	TSS1500
cg13385210	17	38,599,271	3.81E-11	0.292	0.263				*IGFBP4*	TSS1500
cg06261066	20	2,360,396	2.86E-06	0.292	0.247				*TGM6*	TSS1500
cg04849985	1	68,215,783	2.22E-05	0.291	0.276				*GNG12*	5'UTR
cg07629454	3	121,903,520	0.000286	0.291	0.275				*CASR*	5'UTR
cg19925204	10	119,307,692	0.00845	0.291	0.255				*EMX2*	3'UTR
cg21117965	2	220,325,369	2.48E-15	0.291	0.282				*SPEG*	Body
cg19080053	6	5,132,887	8.78E-10	0.291	0.280				*LYRM4*	3'UTR
cg15920654	4	4,869,091	0.012016	0.291	0.346					
cg09234567	15	72,489,828	0.048876	0.291	0.162				*GRAMD2*	Body
cg22996170	19	12,895,529	6.91E-20	0.291	0.287					
cg00979803	17	38,646,740	4.19E-15	0.291	0.279			Yes	*TNS4*	Body
cg02181624	6	168,716,070	2.66E-13	0.291	0.282				*DACT2*	Body
cg06611426	12	52,404,161	9.51E-06	0.291	0.267				*GRASP*	Body
cg00935993	2	21,022,933	0.000484	0.291	0.252				*C2orf43*	TSS200
cg26132320	20	44,640,353	0.000928	0.291	0.400				*MMP9*	Body
cg02595219	11	74,178,761	2.72E-08	0.291	0.255	6.29E-18	0.221		*KCNE3*	TSS200
cg16783744	8	105,478,855	3.51E-07	0.291	0.280				*DPYS*	Body
cg02387326	12	54,345,679	3E-05	0.291	0.276					
cg09170903	15	62,457,209	0.00599	0.291	0.348				*C2CD4B*	5'UTR
cg20844851	6	117,591,827	0.04171	0.291	0.122				*VGLL2*	Body
cg20427865	16	57,406,353	3.19E-12	0.291	0.298	1.83E-21	0.231		*CX3CL1*	TSS200
cg04010446	6	162,311,879	0.000139	0.291	0.244				*PARK2*	Body
cg14185547	8	145,497,339	7.31E-14	0.291	0.313				*BOP1*	Body
cg24804060	17	48,425,014	4.16E-18	0.291	0.295				*XYLT2*	Body
cg26987928	15	62,767,926	3.71E-14	0.291	0.292					
cg10556384	10	94,828,103	0.000438	0.291	0.168				*CYP26C1*	Body

cg10201685	7	44,315,903	2.64E-12	0.291	0.276				*CAMK2B*	Body
cg11332928	1	45,273,680	0.010515	0.291	0.242				*BTBD19*	TSS1500
cg20615879	10	102,892,391	0.00697	0.291	0.291				*TLX1NB*	TSS1500
cg24738346	1	27,479,312	1.04E-07	0.291	0.276				*SLC9A1*	Body
cg21601919	5	170,877,932	2.62E-06	0.291	0.260				*FGF18*	Body
cg02285791	6	27,550,747	0.000771	0.291	0.342					
cg03650550	4	83,675,797	2.6E-14	0.291	0.280				*MIR575*	TSS1500
cg11038340	14	103,389,966	4.53E-05	0.291	0.309				*AMN*	Body
cg26186239	20	62,119,492	1.67E-09	0.291	0.239				*EEF1A2*	3'UTR
cg10655236	17	753,974	2.15E-10	0.291	0.287				*NXN*	Body
cg04258335	6	123,121,585	3.81E-10	0.291	0.271				*SMPDL3A*	Body
cg11294513	19	58,220,295	4.47E-06	0.291	0.234				*ZNF154*	Body
cg11841649	5	131,989,505	1.22E-08	0.291	0.311					
cg13051071	1	145,473,392	0.032709	0.291	0.173				*ANKRD34A*	Body
cg13803976	2	87,016,129	1.92E-06	0.291	0.296				*CD8A*	Body
cg09236434	2	99,553,642	8.49E-07	0.291	0.285				*C2orf55*	TSS1500
cg02984614	17	31,618,409	0.027926	0.291	0.283				*ACCN1*	Body
cg01264639	5	73,839,484	6.41E-10	0.291	0.251					
cg03951603	15	89,903,565	0.003722	0.291	0.289					
cg16529599	20	42,137,055	2.4E-06	0.291	0.293					
cg04800503	17	46,648,533	4.94E-12	0.291	0.244				*HOXB3*	5'UTR
cg01762827	16	69,141,442	0.03634	0.291	0.294				*HAS3*	5'UTR
cg15536490	20	55,203,849	0.002128	0.291	0.219	0.00462803	0.069		*TFAP2C*	TSS1500
cg19320476	11	82,443,592	3.81E-07	0.291	0.281				*FAM181B*	1stExon
cg10896862	1	161,275,561	0.002339	0.291	0.326				*MPZ*	3'UTR
cg18608369	17	33,700,759	0.010716	0.291	0.337				*SLFN11*	TSS200
cg08134504	2	16,245,936	1.36E-14	0.291	0.253					
cg17947992	6	2,510,180	2.43E-13	0.291	0.270					
cg04858553	17	80,549,621	1.55E-14	0.291	0.300				*FOXK2*	Body
cg19519964	2	223,290,012	8.71E-14	0.291	0.235				*SGPP2*	Body
cg18912484	14	97,058,964	0.002264	0.291	0.266					
cg01765111	1	37,784,244	0.03852	0.291	0.293					
cg06097580	16	56,456,934	2.97E-32	0.291	0.275				*AMFR*	Body
cg18566515	10	123,356,236	2.2E-07	0.291	0.280				*FGFR2*	5'UTR

cg14345131	2	233,741,790	3.5E-17	0.291	0.241					
cg09990446	6	29,716,108	1.63E-07	0.291	0.237				*LOC285830*	Body
cg07915921	12	54,321,502	0.027807	0.291	0.275					
cg22245096	2	238,681,052	2.36E-08	0.291	0.295				*LRRFIP1*	Body
cg15258447	10	99,798,430	1.13E-06	0.291	0.283					
cg04656070	8	116,661,063	4.68E-22	0.291	0.301				*TRPS1*	5'UTR
cg20326704	10	70,321,770	1.72E-06	0.291	0.275				*TET1*	5'UTR
cg02050376	13	109,984,684	1.65E-07	0.291	0.211					
cg06159340	16	30,907,560	4.82E-06	0.291	0.277				*CTF1*	TSS1500
cg01258949	11	47,092,992	5.06E-15	0.291	0.286				*C11orf49*	Body
cg14100184	16	851,298	2.64E-08	0.291	0.298	7.9E-16	0.157		*GNG13*	TSS1500
cg08593157	17	17,684,240	1.13E-16	0.291	0.286				*SMCR5*	TSS1500
cg00618626	17	39,807,901	3.85E-13	0.291	0.277					
cg17207552	X	39,767,775	3.4E-23	0.291	0.267					
cg18438793	1	40,149,837	0.018679	0.291	0.337				*HPCAL4*	Body
cg00862116	7	87,230,344	0.001194	0.291	0.265				*ABCB1*	5'UTR
cg17214381	4	188,916,668	0.015322	0.291	0.290				*ZFP42*	TSS1500
cg06452354	17	43,449,123	0.005506	0.291	0.278					
cg17768491	19	41,018,876	0.032898	0.291	0.289				*SPTBN4*	Body
cg01530482	11	1,976,432	1.43E-19	0.291	0.299				*MRPL23*	Body
cg09155362	15	33,487,289	1.85E-05	0.291	0.291					
cg25577023	14	103,394,899	0.042886	0.291	0.388				*AMN*	Body
cg12946808	11	115,493,909	3.24E-07	0.291	0.270					
cg18140268	1	147,781,827	1.75E-08	0.291	0.285					
cg13646785	9	135,461,935	0.000937	0.291	0.293				*BARHL1*	Body
cg14604470	9	130,713,376	2.02E-17	0.291	0.301				*FAM102A*	TSS1500
cg04089901	7	7,557,590	7.64E-11	0.291	0.272				*COL28A1*	Body
cg14498745	7	142,638,303	6.3E-10	0.291	0.298				*KEL*	3'UTR
cg20389472	2	171,568,526	0.028071	0.291	0.198					
cg03143486	7	811,491	2.75E-10	0.291	0.300				*HEATR2*	Body
cg14836602	5	3,592,718	0.000426	0.291	0.390					
cg12573871	18	72,509,555	1.25E-06	0.291	0.249				*ZNF407*	Body
cg08019736	11	115,529,822	8.71E-09	0.291	0.305					
cg11059357	8	41,649,676	3.3E-07	0.291	0.269				*ANK1*	Body

cg03673965	6	108,497,734	3.43E-05	0.290	0.303				*NR2E1*	Body
cg20645966	12	117,131,719	1.28E-07	0.290	0.211					
cg25596297	2	223,162,875	0.00056	0.290	0.278				*PAX3*	Body
cg26983177	19	35,395,973	0.00056	0.290	0.257					
cg06536868	2	27,530,884	2.86E-07	0.290	0.259				*UCN*	5'UTR
cg18254850	6	116,691,851	0.027265	0.290	0.188				*DSE*	TSS1500
cg26249873	10	25,465,430	1.35E-08	0.290	0.335				*LOC100128811*	TSS1500
cg07402669	20	57,090,015	0.000204	0.290	0.295				*APCDD1L*	TSS200
cg12563839	14	91,580,507	0.019067	0.290	0.123				*C14orf159*	1stExon
cg00568550	1	61,784,017	0.002285	0.290	0.256			Yes	*NFIA*	Body
cg21852513	1	116,371,475	0.000153	0.290	0.255					
cg26311932	6	31,549,563	2.69E-18	0.290	0.279				*LTB*	Body
cg08849150	6	117,087,010	4.12E-07	0.290	0.215				*FAM162B*	TSS200
cg22488750	1	22,678,029	2.54E-05	0.290	0.264					
cg21051972	11	18,067,927	0.003832	0.290	0.263					
cg22134162	3	63,841,271	6.72E-17	0.290	0.276				*THOC7*	Body
cg22991148	21	46,131,099	0.00704	0.290	0.254	7.47E-21	0.223		*C21orf29*	Body
cg09032314	8	69,426,081	8.28E-11	0.290	0.295				*C8orf34*	Body
cg14796305	1	46,632,581	1.45E-06	0.290	0.320					
cg18451814	14	57,278,188	1.1E-13	0.290	0.321				*OTX2*	TSS1500
cg16324314	14	77,480,847	2.4E-06	0.290	0.236					
cg15959252	12	48,578,724	2.8E-10	0.290	0.311				*C12orf68*	3'UTR
cg18495563	11	33,061,907	0.000673	0.290	0.271	9.89E-20	0.167		*TCP11L1*	5'UTR
cg25096264	1	112,860,317	2.38E-12	0.290	0.288					
cg01856529	12	54,653,091	3.91E-17	0.290	0.292				*CBX5*	1stExon
cg14575356	6	130,013,903	3.59E-08	0.290	0.296				*ARHGAP18*	Body
cg16714091	9	18,473,902	7.69E-07	0.290	0.223	3.67E-14	0.170		*ADAMTSL1*	TSS1500
cg10580293	7	29,605,897	1.16E-10	0.290	0.287				*PRR15*	5'UTR
cg14604777	12	101,988,756	1.9E-06	0.290	0.223				*MYBPC1*	5'UTR
cg21148362	3	62,353,708	0.003526	0.290	0.270					
cg26515460	20	983,354	0.012437	0.290	0.316				*RSPO4*	TSS1500
cg14189141	9	1,042,605	0.001285	0.290	0.277					
cg07516252	14	24,641,201	4.78E-11	0.290	0.288				*REC8*	TSS200
cg18168101	8	104,153,627	7.27E-09	0.290	0.294				*C8orf56*	TSS200

cg10746936	14	95,240,143	0.003698	0.290	0.306					
cg08334034	15	93,615,039	5.76E-15	0.290	0.271				*RGMA*	Body
cg05210480	7	24,539,647	4.69E-26	0.290	0.288					
cg26433955	12	96,558,950	1.64E-10	0.290	0.302					
cg07756483	19	44,281,665	0.000119	0.290	0.207				*KCNN4*	Body
cg12537329	13	113,677,777	4.95E-19	0.290	0.287				*MCF2L*	Body
cg20485758	6	26,088,446	1.09E-05	0.290	0.279				*HFE*	Body
cg05627522	15	75,251,581	2.46E-09	0.290	0.241					
cg17128417	2	20,865,088	9.9E-06	0.290	0.266			Yes	*GDF7*	TSS1500
cg24376776	10	101,297,245	9.9E-05	0.290	0.264					
cg14464718	X	100,334,126	0.003874	0.290	0.314				*TMEM35*	Body
cg22698489	1	159,893,696	0.007443	0.290	0.283				*TAGLN2*	5'UTR
cg01417625	1	183,187,433	0.000493	0.290	0.262				*LAMC2*	Body
cg02490736	6	1,391,758	9.24E-06	0.290	0.253				*FOXF2*	Body
cg13721404	18	31,739,202	0.016779	0.290	0.311				*NOL4*	Body
cg22544863	19	15,580,400	3.38E-09	0.290	0.286				*PGLYRP2*	Body
cg22673999	6	37,752,301	0.000112	0.290	0.214					
cg17356056	5	139,526,226	2.46E-16	0.290	0.269					
cg13784235	6	108,495,865	9.31E-07	0.290	0.271				*NR2E1*	Body
cg26451547	11	36,397,596	6.12E-08	0.290	0.234				*PRR5L*	5'UTR
cg04615460	12	115,125,098	4.5E-13	0.290	0.271					
cg05589784	5	2,915,123	1.93E-07	0.290	0.309					
cg09632907	4	54,969,963	2.91E-05	0.290	0.296					
cg24035245	1	119,535,928	7.35E-10	0.290	0.247					
cg08058191	17	39,781,130	2.36E-07	0.290	0.284				*KRT17*	TSS1500
cg00582971	5	178,422,128	0.018721	0.290	0.369				*GRM6*	TSS200
cg13661109	9	129,433,708	2.35E-11	0.290	0.304				*LMX1B*	Body
cg14132388	12	120,242,490	3.7E-06	0.290	0.277				*CIT*	Body
cg00020474	1	8,214,963	0.000213	0.290	0.139					
cg24153044	1	6,485,201	0.004408	0.290	0.325				*ESPN*	1stExon
cg15639684	1	85,464,110	6.11E-15	0.290	0.265				*MCOLN2*	TSS1500
cg10670396	X	101,906,537	0.026956	0.290	0.319				*GPRASP1*	5'UTR
cg01629716	15	45,996,671	3.12E-05	0.290	0.289					
cg27090216	8	22,960,461	0.003201	0.290	0.209	5.92E-14	0.225		*TNFRSF10C*	5'UTR

cg15829642	5	140,744,983	0.004652	0.290	0.341				*PCDHGA4*	Body
cg23546512	12	125,160,999	4.31E-15	0.290	0.280					
cg25678088	7	96,632,460	1.58E-10	0.290	0.247				*DLX6AS*	Body
cg25061914	22	37,447,468	0.004527	0.289	0.160			Yes	*KCTD17*	TSS1500
cg01014615	2	177,027,621	4.45E-05	0.289	0.310				*HOXD3*	TSS1500
cg24306340	1	64,937,451	6.06E-06	0.289	0.270				*CACHD1*	Body
cg11437784	13	112,710,823	0.015958	0.289	0.273					
cg06973595	4	55,093,096	0.00042	0.289	0.316					
cg18265608	11	44,087,367	1.04E-09	0.289	0.255				*ACCS*	TSS1500
cg25280433	6	105,584,216	0.004119	0.289	0.178				*BVES*	5'UTR
cg12781915	3	185,544,099	5.38E-06	0.289	0.283				*IGF2BP2*	TSS1500
cg15616496	17	73,860,607	3.25E-07	0.289	0.284					
cg02764093	12	120,241,760	1.44E-18	0.289	0.278				*CIT*	Body
cg09963123	4	38,664,654	4.27E-05	0.289	0.216	5.17E-08	0.109		*FLJ13197*	Body
cg05750047	1	149,908,085	3.24E-23	0.289	0.286				*MTMR11*	Body
cg08764465	8	15,308,417	8.6E-15	0.289	0.294					
cg12920180	14	31,344,444	1.85E-05	0.289	0.305				*COCH*	Body
cg07631309	10	106,020,930	1.28E-08	0.289	0.274				*GSTO1*	Body
cg13782866	1	231,761,845	1.13E-08	0.289	0.260				*TSNAX-DISC1*	Body
cg15204861	11	111,783,352	1.9E-05	0.289	0.325				*CRYAB*	TSS1500
cg14470978	9	132,427,640	0.017625	0.289	0.268				*PRRX2*	TSS1500
cg03430633	11	119,580,588	1.76E-07	0.289	0.312				*PVRL1*	Body
cg12477452	3	11,237,939	0.000138	0.289	0.315				*HRH1*	5'UTR
cg07019438	14	34,419,546	0.007157	0.289	0.321				*EGLN3*	Body
cg20680163	8	145,033,743	8.45E-09	0.289	0.259				*PLEC1*	Body
cg08545463	11	108,459,708	3.07E-08	0.289	0.288				*EXPH5*	Body
cg05599160	5	124,536,183	3.26E-07	0.289	0.290					
cg15681358	10	99,474,521	0.000209	0.289	0.143				*MARVELD1*	Body
cg20567847	7	155,167,038	0.022799	0.289	0.289					
cg24770158	17	8,241,765	3.03E-07	0.289	0.284				*ODF4*	TSS1500
cg25001190	1	61,668,835	5.26E-13	0.289	0.269				*NFIA*	Body
cg09799983	2	38,301,756	0.021467	0.289	0.340				*CYP1B1*	Body
cg06427779	5	54,180,079	2.54E-08	0.289	0.335					
cg16400350	21	46,414,569	2.65E-28	0.289	0.267					

cg18843682	12	54,425,156	7.48E-10	0.289	0.231				*HOXC4*	5'UTR
cg18972013	17	78,078,605	2.86E-12	0.289	0.287				*GAA*	Body
cg16755924	X	25,041,099	0.009936	0.289	0.265					
cg05159804	17	44,343,776	8.09E-08	0.289	0.199					
cg04857033	1	120,258,412	3.15E-12	0.289	0.257				*PHGDH*	Body
cg13529335	9	2,157,942	0.014812	0.289	0.263				*SMARCA2*	Body
cg17715896	7	33,943,437	4.49E-10	0.289	0.270					
cg08441170	10	26,223,100	0.031579	0.289	0.346				*MYO3A*	1stExon
cg06975048	17	7,826,155	0.000221	0.289	0.260				*KCNAB3*	3'UTR
cg17616646	11	67,271,660	0.001274	0.289	0.083				*PITPNM1*	5'UTR
cg19678392	7	94,953,810	7.97E-13	0.289	0.258				*PON1*	1stExon
cg01589998	12	109,729,478	0.004265	0.289	0.195				*FOXN4*	Body
cg14150907	8	142,275,722	0.00507	0.289	0.265					
cg04748497	X	101,906,989	0.000342	0.289	0.268				*GPRASP1*	5'UTR
cg03609666	19	49,250,439	1.61E-06	0.289	0.283				*IZUMO1*	TSS1500
cg14448830	9	79,627,216	0.015069	0.289	0.308					
cg12361088	11	19,695,688	2.31E-06	0.289	0.220				*NAV2*	Body
cg07121182	7	99,156,011	0.001995	0.289	0.261				*ZNF655*	TSS200
cg07870074	10	706,006	3.02E-08	0.289	0.294				*DIP2C*	Body
cg22475071	3	64,338,594	2.74E-10	0.289	0.289					
cg09642925	2	114,261,793	0.00293	0.289	0.182					
cg25090510	5	156,886,990	0.00094	0.289	0.203				*NIPAL4*	TSS200
cg05262436	6	137,815,569	0.006543	0.289	0.358				*OLIG3*	TSS200
cg22088219	7	74,064,567	0.001165	0.289	0.352					
cg16673477	19	18,812,017	0.003457	0.289	0.191				*CRTC1*	Body
cg25572367	12	25,538,380	0.01536	0.289	0.162					
cg01519225	6	29,895,005	0.000443	0.289	0.371					
cg03890691	16	30,023,615	8.98E-12	0.289	0.296				*DOC2A*	TSS1500
cg16082058	9	100,265,072	6.73E-18	0.289	0.277				*TMOD1*	5'UTR
cg15046818	12	96,587,840	0.012997	0.289	0.237				*ELK3*	TSS1500
cg01377268	2	239,925,497	1.47E-08	0.289	0.253					
cg07018404	5	126,193,470	1.16E-13	0.289	0.291					
cg01874867	7	94,954,059	8.42E-09	0.289	0.254				*PON1*	TSS200
cg04120686	3	169,384,410	2.3E-08	0.289	0.288					

cg22771759	13	24,902,376	0.030805	0.289	0.322					
cg24274579	17	59,534,637	2.75E-08	0.289	0.261				*TBX4*	Body
cg06924459	6	17,102,643	0.000436	0.289	0.148					
cg08049853	4	41,875,784	0.00028	0.289	0.293					
cg08941457	20	25,129,562	8.23E-09	0.289	0.285				*LOC284798*	TSS1500
cg17001627	11	47,599,391	4.95E-21	0.289	0.255				*KBTBD4*	Body
cg20532370	3	139,258,730	0.012272	0.289	0.124				*RBP1*	TSS200
cg19592439	3	138,665,957	0.006767	0.289	0.218				*FOXL2*	1stExon
cg24304093	11	14,926,738	0.001884	0.289	0.289					
cg00697992	2	139,537,131	0.009745	0.289	0.295				*NXPH2*	Body
cg13462843	9	37,025,769	0.003939	0.289	0.382				*PAX5*	Body
cg08691567	12	53,183,930	8.46E-07	0.289	0.318				*KRT3*	Body
cg26296488	4	9,783,192	0.014355	0.289	0.388				*DRD5*	TSS200
cg13738299	13	46,189,906	0.002499	0.289	0.330				*FAM194B*	TSS200
cg08278357	19	59,050,122	0.003304	0.289	0.273					
cg00788521	12	2,229,269	9.72E-12	0.289	0.303				*CACNA1C*	Body
cg02457623	1	203,599,089	3.87E-05	0.289	0.248				*ATP2B4*	5'UTR
cg00737978	18	13,611,807	1.24E-07	0.289	0.248				*C18orf1*	5'UTR
cg18864674	1	21,058,007	9.12E-09	0.289	0.285				*SH2D5*	5'UTR
cg07811110	12	85,307,131	0.025973	0.289	0.284				*SLC6A15*	TSS1500
cg16354201	1	33,938,307	0.01921	0.289	0.226				*ZSCAN20*	5'UTR
cg13063274	3	112,870,238	6.58E-19	0.289	0.295					
cg14665901	11	12,885,131	6.76E-09	0.289	0.262				*TEAD1*	Body
cg25874119	1	228,633,904	0.000373	0.289	0.357					
cg25913520	4	13,524,041	0.005336	0.289	0.314					
cg05287372	3	69,058,876	6.39E-20	0.289	0.293				*C3orf64*	Body
cg00641409	6	21,666,778	0.018469	0.288	0.327				*FLJ22536*	Body
cg02866639	17	1,808,743	5.15E-12	0.288	0.223					
cg16355945	12	132,654,742	0.006798	0.288	0.261					
cg05345286	6	41,606,641	0.000303	0.288	0.198	3E-09	0.158		*MDFI*	Body
cg23949389	5	122,621,186	4.96E-06	0.288	0.283					
cg24444188	1	200,992,385	0.023746	0.288	0.168				*KIF21B*	Body
cg12549600	1	113,287,034	1.64E-05	0.288	0.244					
cg03188000	12	68,634,913	3.15E-08	0.288	0.237					

cg07314829	1	112,058,397	6.32E-11	0.288	0.291				*ADORA3*	Body
cg16672562	19	46,801,672	1.64E-12	0.288	0.293				*HIF3A*	5'UTR
cg05510665	6	137,834,607	1.1E-05	0.288	0.288					
cg07179669	1	221,054,707	2.05E-07	0.288	0.268				*HLX*	Body
cg15035437	X	63,005,077	0.027054	0.288	0.344					
cg04518342	5	131,593,106	0.003519	0.288	0.196				*PDLIM4*	TSS1500
cg06430509	21	44,853,470	2.91E-22	0.288	0.276					
cg00656735	6	41,470,922	5.96E-15	0.288	0.287					
cg01813139	9	139,580,970	9.48E-05	0.288	0.217				*AGPAT2*	Body
cg02016195	9	132,198,822	3.02E-14	0.288	0.283					
cg02399524	11	63,258,509	1.8E-05	0.288	0.282				*HRASLS5*	1stExon
cg10017626	2	65,085,243	2.05E-11	0.288	0.254					
cg12182937	6	33,215,649	4.03E-17	0.288	0.281					
cg24856140	1	146,556,382	0.000794	0.288	0.280					
cg23273897	3	154,796,861	0.000972	0.288	0.161	9.66E-24	0.162		*MME*	TSS1500
cg11482422	10	11,206,813	0.002118	0.288	0.184				*CUGBP2*	TSS200
cg17928286	1	205,414,220	2.04E-12	0.288	0.280					
cg23754918	7	2,653,733	3.54E-20	0.288	0.267				*IQCE*	3'UTR
cg21938261	5	146,257,862	0.026004	0.288	0.309				*PPP2R2B*	5'UTR
cg01010188	5	172,753,741	0.001478	0.288	0.237				*STC2*	Body
cg18735810	5	50,260,204	1.63E-05	0.288	0.222					
cg25178683	17	76,976,267	1.21E-06	0.288	0.240				*LGALS3BP*	TSS1500
cg02120582	11	104,034,754	0.043405	0.288	0.320				*PDGFD*	5'UTR
cg00957641	12	103,406,953	0.00117	0.288	0.190					
cg11852579	4	3,657,880	0.002204	0.288	0.278					
cg14876117	14	51,428,973	7.01E-14	0.288	0.290					
cg03620376	19	54,984,272	1.45E-05	0.288	0.289	2.6E-09	0.130		*CDC42EP5*	5'UTR
cg11721100	X	20,134,346	5.58E-05	0.288	0.297				*MAP7D2*	Body
cg17881208	12	55,378,912	5.69E-08	0.288	0.267				*KIAA0748*	TSS1500
cg21951301	12	5,542,527	0.001682	0.288	0.246				*NTF3*	Body
cg24430419	1	61,519,532	1.03E-05	0.288	0.260					
cg26123256	2	27,530,670	8.1E-10	0.288	0.259				*UCN*	Body
cg04937080	1	58,715,532	0.002136	0.288	0.252				*DAB1*	5'UTR
cg18607411	13	80,911,808	3.71E-06	0.288	0.282				*SPRY2*	Body

cg23108728	2	220,117,875	0.003386	0.288	0.364				*TUBA4B*	TSS200
cg02580986	X	105,421,602	0.000359	0.288	0.206				*MUM1L1*	5'UTR
cg24667115	6	91,004,482	4.58E-07	0.288	0.235				*BACH2*	5'UTR
cg27262054	16	73,067,525	1.41E-08	0.288	0.270				*ZFHX3*	5'UTR
cg03177042	6	166,422,671	4.15E-06	0.288	0.325					
cg19463256	19	46,915,351	5.84E-10	0.288	0.257				*CCDC8*	1stExon
cg14071612	9	112,081,275	0.012353	0.288	0.069				*EPB41L4B*	Body
cg12251779	22	45,608,440	1.8E-05	0.288	0.254				*C22orf9*	Body
cg17302155	6	100,061,111	0.003007	0.288	0.364				*PRDM13*	Body
cg26809043	11	34,285,529	1.34E-22	0.288	0.280				*ABTB2*	Body
cg13855924	6	39,281,183	3.41E-08	0.288	0.263				*KCNK17*	Body
cg26795540	10	35,082,070	2.05E-19	0.288	0.270				*PARD3*	Body
cg06390079	17	19,648,855	0.000891	0.288	0.281				*ALDH3A1*	1stExon
cg19964273	6	168,745,827	1.44E-20	0.288	0.259					
cg09417209	16	11,715,746	2.77E-06	0.288	0.269					
cg12449685	11	124,768,950	8.66E-05	0.288	0.283				*ROBO4*	TSS1500
cg02251349	8	74,004,752	5.25E-08	0.288	0.245				*C8orf84*	Body
cg05436064	16	67,197,180	0.000739	0.288	0.346				*HSF4*	TSS200
cg25601949	X	3,630,407	0.003132	0.288	0.285				*PRKX*	Body
cg22129364	1	53,067,894	2.55E-06	0.288	0.235				*GPX7*	TSS200
cg22891070	19	46,801,642	8.47E-10	0.288	0.288				*HIF3A*	Body
cg00155063	1	12,227,825	1.33E-15	0.288	0.273				*TNFRSF1B*	Body
cg12193731	12	52,300,911	0.000351	0.288	0.267				*ACVRL1*	TSS1500
cg25300386	7	94,023,988	4.52E-05	0.288	0.261	6.67E-09	0.152		*COL1A2*	1stExon
cg24437737	1	149,400,352	1.11E-19	0.288	0.266				*HIST2H2BF*	TSS1500
cg11433866	12	115,126,908	6.81E-17	0.288	0.258					
cg16626067	16	66,613,266	1.85E-06	0.288	0.250				*CMTM2*	TSS200
cg00353923	7	127,671,044	0.002661	0.288	0.195				*LRRC4*	TSS200
cg05052324	1	18,957,020	0.00797	0.288	0.320				*PAX7*	TSS1500
cg13861122	9	91,849,710	0.023621	0.288	0.169					
cg06851000	1	33,627,109	1.06E-10	0.288	0.261				*TRIM62*	Body
cg15980797	6	137,813,648	3.47E-07	0.288	0.290				*OLIG3*	1stExon
cg11956953	17	27,347,092	0.043683	0.288	0.072					
cg07553475	6	21,665,800	0.03536	0.288	0.355				*FLJ22536*	TSS1500

cg00746446	2	23,879,137	9.44E-37	0.288	0.281				*KLHL29*	Body
cg19048950	13	30,915,492	4.2E-09	0.287	0.270				*LOC100188949*	Body
cg25485392	19	3,358,039	5.76E-18	0.287	0.259					
cg12683342	6	28,557,518	0.001718	0.287	0.239					
cg19845249	17	77,819,279	2.81E-11	0.287	0.268					
cg11269599	19	52,391,304	0.00386	0.287	0.278				*ZNF577*	TSS200
cg26631984	2	17,719,375	0.002511	0.287	0.317					
cg13779265	X	105,970,597	0.000136	0.287	0.359				*RNF128*	1stExon
cg01135546	17	45,949,828	0.000322	0.287	0.344					
cg11749828	19	35,531,150	1.76E-07	0.287	0.280				*SCN1B*	3'UTR
cg00862041	X	101,967,017	2.57E-09	0.287	0.266	1.61E-17	0.217		*GPRASP2*	TSS1500
cg06833110	2	66,673,316	0.000103	0.287	0.313				*MEIS1*	Body
cg21773967	17	15,166,047	0.013828	0.287	0.174				*PMP22*	TSS200
cg09293816	19	50,015,532	1.63E-20	0.287	0.292				*FCGRT*	TSS200
cg11911648	6	30,418,902	8.99E-06	0.287	0.288					
cg03509106	4	121,992,261	1.34E-08	0.287	0.247				*C4orf31*	5'UTR
cg14752089	8	128,773,042	3.61E-10	0.287	0.279					
cg19008877	10	78,157,854	3.14E-13	0.287	0.279				*C10orf11*	Body
cg20707222	1	76,540,222	0.00231	0.287	0.250				*ST6GALNAC3*	TSS200
cg08032476	17	27,045,176	7.36E-17	0.287	0.272				*RAB34*	Body
cg13597397	9	133,320,948	0.004549	0.287	0.173				*ASS1*	5'UTR
cg15341350	12	119,446,891	0.006176	0.287	0.334				*SRRM4*	Body
cg04839991	2	237,880,790	4.07E-08	0.287	0.251					
cg11431957	3	38,035,999	0.015397	0.287	0.112				*VILL*	Body
cg27211284	2	200,524,012	0.006558	0.287	0.266					
cg23793104	4	85,401,758	3.96E-08	0.287	0.287					
cg07475654	2	219,151,444	8.02E-05	0.287	0.376				*PNKD*	Body
cg02613418	15	68,723,914	3.75E-08	0.287	0.273				*ITGA11*	Body
cg17461735	9	82,185,498	0.00027	0.287	0.252				*TLE4*	TSS1500
cg15744005	10	104,629,667	1.5E-05	0.287	0.206				*AS3MT*	Body
cg17817532	10	70,322,874	3.16E-07	0.287	0.296				*TET1*	5'UTR
cg15271616	9	35,490,515	0.001176	0.287	0.145	5.96E-17	0.271		*RUSC2*	5'UTR
cg03381047	6	168,078,772	0.000379	0.287	0.234					
cg22081832	3	64,670,459	3.69E-07	0.287	0.196				*ADAMTS9*	Body

cg03507534	1	36,789,937	1.77E-12	0.287	0.269				*FAM176B*	TSS200
cg17727929	21	38,069,338	0.004102	0.287	0.308					
cg11928064	3	124,537,810	9.33E-24	0.287	0.281				*ITGB5*	Body
cg03784628	11	64,059,937	4.54E-16	0.287	0.253				*KCNK4*	5'UTR
cg23229261	2	63,284,066	2.09E-06	0.287	0.302				*OTX1*	3'UTR
cg07992044	11	68,096,049	0.044051	0.287	0.169				*LRP5*	Body
cg14273450	2	26,624,934	0.007756	0.287	0.108				*C2orf39*	1stExon
cg09479818	19	33,880,790	2.32E-18	0.287	0.287				*PEPD*	Body
cg13917047	17	15,406,213	0.020367	0.287	0.500				*FAM18B2*	Body
cg12250049	15	83,951,663	4.22E-07	0.287	0.296				*BNC1*	Body
cg00908833	6	50,810,432	0.00028	0.287	0.343				*TFAP2B*	Body
cg27525524	1	16,468,313	1.65E-14	0.287	0.271				*EPHA2*	Body
cg03071808	2	242,908,131	3.82E-07	0.287	0.258					
cg04015962	1	10,949,192	2.37E-14	0.287	0.294					
cg05526015	6	170,553,845	4.08E-50	0.287	0.279					
cg00305281	22	42,316,176	5.94E-08	0.287	0.261					
cg06605704	16	4,551,147	4.97E-06	0.287	0.319				*HMOX2*	5'UTR
cg09107912	6	159,590,059	0.000476	0.287	0.322				*FNDC1*	TSS1500
cg09561125	7	108,097,477	5.74E-09	0.287	0.276				*NRCAM*	TSS1500
cg26419656	13	74,707,400	0.00098	0.287	0.316				*KLF12*	5'UTR
cg26983469	10	17,271,051	0.010165	0.287	0.334				*VIM*	5'UTR
cg01083633	10	44,162,781	1.64E-05	0.287	0.206					
cg08807892	2	162,101,083	0.047349	0.287	0.297					
cg13433012	12	57,635,954	0.000854	0.287	0.225				*NDUFA4L2*	TSS1500
cg01165355	3	64,225,108	3.66E-14	0.287	0.242					
cg09065714	2	27,531,535	5.94E-14	0.287	0.292				*UCN*	TSS1500
cg27473895	10	102,497,354	9.39E-09	0.287	0.296					
cg23209990	4	55,097,576	0.004858	0.287	0.262				*PDGFRA*	5'UTR
cg10967191	16	49,888,604	1.4E-05	0.287	0.216					
cg20589883	4	111,115,135	5.79E-06	0.287	0.248				*ELOVL6*	Body
cg08551248	1	40,255,129	0.000175	0.287	0.176				*BMP8B*	TSS1500
cg22620090	6	105,400,993	0.042332	0.287	0.341					
cg26919186	18	56,701,751	2.62E-09	0.287	0.252				*LOC390858*	TSS1500
cg27327911	16	54,969,146	0.001373	0.287	0.366					

cg24892362	3	184,319,417	4.4E-06	0.287	0.230					
cg01566555	3	125,931,744	0.020727	0.287	0.207					
cg23451221	10	135,160,726	6.07E-08	0.287	0.255				*PRAP1*	TSS200
cg23348764	5	140,749,834	0.002265	0.287	0.365				*PCDHGA4*	Body
cg23695707	14	95,234,826	5.82E-05	0.287	0.299				*GSC*	3'UTR
cg19908556	11	32,355,022	0.014426	0.287	0.231					
cg19258665	7	157,482,508	0.000696	0.287	0.276				*PTPRN2*	Body
cg00349061	1	26,371,865	1.23E-13	0.287	0.272				*SLC30A2*	Body
cg08639379	1	95,018,889	1.27E-13	0.287	0.274					
cg15222899	18	55,095,249	0.000237	0.287	0.304					
cg23645199	2	233,498,625	5.1E-08	0.287	0.260				*EFHD1*	1stExon
cg08124446	11	43,596,529	0.00111	0.287	0.312					
cg16725974	14	64,341,178	3.42E-10	0.287	0.292				*SYNE2*	5'UTR
cg20498859	3	154,145,810	0.001507	0.286	0.236				*GPR149*	Body
cg02480199	5	145,725,913	1.47E-07	0.286	0.289					
cg15756227	4	185,937,405	1.31E-07	0.286	0.305					
cg00731785	4	15,704,680	0.01358	0.286	0.329				*BST1*	1stExon
cg01763749	22	21,368,291	0.000517	0.286	0.253				*P2RX6*	TSS1500
cg01806741	6	1,527,072	3.75E-07	0.286	0.227					
cg12131828	17	46,663,648	2.72E-09	0.286	0.282					
cg14555350	5	142,580,618	5.05E-17	0.286	0.275				*ARHGAP26*	Body
cg18795461	1	36,591,112	0.004296	0.286	0.242					
cg04463386	11	75,295,186	2.04E-15	0.286	0.256					
cg18501395	8	12,293,513	0.005708	0.286	0.200				*FAM86B2*	Body
cg02722657	17	38,334,439	5.54E-12	0.286	0.270				*RAPGEFL1*	1stExon
cg12788878	3	124,860,616	1.48E-06	0.286	0.248				*SLC12A8*	Body
cg19385331	13	100,641,867	0.000453	0.286	0.282					
cg20427486	2	162,849,137	2.64E-15	0.286	0.269				*DPP4*	3'UTR
cg03389653	16	49,316,197	0.027964	0.286	0.380				*CBLN1*	TSS1500
cg08264895	14	70,060,335	4.07E-28	0.286	0.293					
cg12864389	8	145,025,178	2.16E-07	0.286	0.313				*PLEC1*	Body
cg24478096	2	160,761,085	4.24E-06	0.286	0.287				*LY75*	Body
cg02858512	17	1,992,954	3.86E-05	0.286	0.247				*SMG6*	Body
cg11731890	6	26,271,827	0.011961	0.286	0.294				*HIST1H3G*	TSS1500

cg05899618	2	20,865,847	0.001449	0.286	0.268	1.86E-16	0.178		*GDF7*	TSS1500
cg18043690	X	68,348,774	7.55E-07	0.286	0.318					
cg20314331	2	131,129,567	0.000671	0.286	0.233				*PTPN18*	Body
cg05160558	4	70,389,517	1.51E-06	0.286	0.311			Yes		
cg06496484	2	176,946,509	0.001521	0.286	0.300			Yes	*EVX2*	Body
cg17486263	1	200,011,726	9.7E-05	0.286	0.339				*NR5A2*	Body
cg07239592	7	39,453,625	0.015533	0.286	0.217				*POU6F2*	Body
cg13899718	1	150,947,832	1.91E-07	0.286	0.252				*LASS2*	TSS1500
cg09854626	16	1,030,568	4.67E-09	0.286	0.283				*SOX8*	TSS1500
cg06260709	20	48,626,621	2.93E-17	0.286	0.273					
cg11926610	15	37,403,211	2.68E-07	0.286	0.240					
cg12267236	1	117,527,582	1.19E-21	0.286	0.299				*PTGFRN*	Body
cg21627409	5	140,810,106	0.001065	0.286	0.341				*PCDHGA4*	Body
cg14935626	2	17,776,015	1.43E-06	0.286	0.268				*VSNL1*	Body
cg02030008	7	157,476,930	0.022641	0.286	0.129				*PTPRN2*	Body
cg06135689	17	35,085,060	9.45E-10	0.286	0.283					
cg16136290	1	112,280,233	1.17E-26	0.286	0.297				*C1orf183*	Body
cg22675922	11	2,889,840	0.000118	0.286	0.291				*KCNQ1DN*	TSS1500
cg11905624	19	16,186,848	4.62E-05	0.286	0.271				*TPM4*	Body
cg03426023	16	54,972,078	0.0015	0.286	0.293					
cg25029264	15	81,426,347	0.00459	0.286	0.240				*C15orf26*	TSS1500
cg19690403	12	117,470,568	1.61E-14	0.286	0.248					
cg00599564	6	29,943,209	2.38E-06	0.286	0.289				*HCG9*	Body
cg19682013	15	45,996,608	1.49E-05	0.286	0.274					
cg13947167	9	34,555,109	3.07E-15	0.286	0.257				*CNTFR*	Body
cg18400324	2	132,286,430	3.47E-11	0.286	0.278				*CCDC74A*	Body
cg20403557	5	115,299,088	5.64E-05	0.286	0.324				*LVRN*	Body
cg22241045	4	54,363,911	3.42E-17	0.286	0.267				*LNX1*	Body
cg15924332	10	7,710,179	1.24E-10	0.286	0.285				*ITIH5*	TSS1500
cg19192590	2	178,524,533	1.33E-06	0.286	0.307				*PDE11A*	Body
cg01422262	1	108,506,291	0.000148	0.286	0.160				*VAV3*	Body
cg08582485	20	22,557,518	1.16E-07	0.286	0.280				*C20orf56*	Body
cg12051762	11	117,069,788	3.75E-07	0.286	0.315				*TAGLN*	TSS1500
cg13939978	4	57,522,881	1.94E-11	0.286	0.276				*HOPX*	5'UTR

cg23207077	1	905,988	4.07E-13	0.286	0.275				*PLEKHN1*	Body
cg11284797	11	18,067,816	0.005262	0.286	0.258					
cg10167837	2	17,699,762	0.040262	0.286	0.334				*RAD51AP2*	TSS200
cg14908245	8	23,619,180	4.98E-05	0.286	0.288					
cg04607372	5	54,523,900	3.63E-11	0.286	0.283					
cg16047144	17	62,097,953	0.00071	0.286	0.279				*ICAM2*	5'UTR
cg17785773	5	78,408,347	8.9E-10	0.286	0.279				*BHMT*	Body
cg24960947	11	65,816,463	0.000316	0.286	0.285				*GAL3ST3*	5'UTR
cg26570179	8	99,960,500	1.04E-06	0.286	0.296				*OSR2*	5'UTR
cg19535672	2	27,530,455	1.05E-13	0.286	0.265				*UCN*	Body
cg07475885	2	85,107,320	0.016704	0.286	0.388				*C2orf89*	Body
cg22092811	3	99,595,145	0.002557	0.286	0.302				*C3orf26*	Body
cg23274680	10	94,828,605	0.0002	0.286	0.187					
cg19542753	5	180,252,309	6.49E-19	0.286	0.263			Yes		
cg23729417	2	118,593,926	1.51E-05	0.286	0.304					
cg07665510	17	55,952,063	2.87E-06	0.286	0.291				*CUEDC1*	Body
cg00089798	12	62,585,204	0.001552	0.286	0.285				*FAM19A2*	5'UTR
cg07045089	1	3,620,218	0.00617	0.286	0.463				*TP73*	Body
cg09507790	18	19,752,343	2.72E-12	0.286	0.264				*GATA6*	Body
cg04887494	19	2,252,786	0.00142	0.286	0.266				*JSRP1*	Body
cg17854416	22	37,720,647	7.34E-10	0.286	0.269					
cg20897119	7	116,164,422	0.000782	0.286	0.303				*CAV1*	TSS1500
cg09476067	6	30,418,581	7.78E-11	0.286	0.282					
cg10616974	17	74,511,719	7.72E-16	0.286	0.277					
cg13609645	6	143,555,173	3.13E-20	0.286	0.264				*AIG1*	Body
cg05083414	4	2,627,039	4.04E-19	0.286	0.294				*FAM193A*	TSS200
cg23109344	8	496,440	4.53E-13	0.286	0.253				*C8orf42*	TSS1500
cg03426888	6	32,156,527	1.4E-15	0.286	0.253				*PBX2*	Body
cg09919570	16	49,311,782	0.008241	0.286	0.235					
cg02010682	8	99,954,432	0.000143	0.286	0.301					
cg02936049	3	141,102,599	0.04545	0.286	0.181				*ZBTB38*	5'UTR
cg10839385	1	159,825,049	2.42E-06	0.286	0.253				*VSIG8*	Body
cg19412791	5	180,633,063	0.0005	0.286	0.227				*TRIM7*	TSS1500
cg15411272	6	29,895,187	3.3E-08	0.286	0.269					

cg22056595	7	158,293,463	4.38E-11	0.286	0.258			Yes	*PTPRN2*	Body
cg25953350	11	45,167,368	3.5E-07	0.286	0.308				*PRDM11*	Body
cg00262776	12	14,720,726	3.27E-06	0.286	0.172				*PLBD1*	1stExon
cg02049405	6	30,095,265	1E-16	0.286	0.283					
cg11467015	17	77,786,853	0.003894	0.286	0.301					
cg21493127	5	16,617,587	1.57E-14	0.286	0.237				*FAM134B*	TSS1500
cg08372619	8	109,799,518	0.003691	0.286	0.165				*TMEM74*	5'UTR
cg27282530	3	125,898,680	3.43E-08	0.286	0.299				*ALDH1L1*	5'UTR
cg10378032	1	65,214,497	1.48E-21	0.285	0.276				*RAVER2*	Body
cg10786043	8	38,643,568	6.32E-13	0.285	0.283				*TACC1*	TSS1500
cg10708761	2	39,892,612	0.009352	0.285	0.309				*TMEM178*	TSS1500
cg25575732	10	52,177,880	1.31E-06	0.285	0.289				*SGMS1*	5'UTR
cg23183069	6	168,778,242	1.82E-13	0.285	0.258					
cg05298922	2	175,191,045	0.010932	0.285	0.260					
cg23019889	1	146,544,044	3.29E-27	0.285	0.299					
cg14875171	2	50,574,196	0.000234	0.285	0.257				*NRXN1*	Body
cg26338453	15	82,340,023	0.017469	0.285	0.203					
cg02657832	2	201,450,633	0.000217	0.285	0.260				*AOX1*	TSS200
cg17058383	13	113,624,866	1.06E-17	0.285	0.268				*MCF2L*	Body
cg17799033	7	157,476,329	9.86E-11	0.285	0.263				*PTPRN2*	Body
cg13862927	9	72,158,091	2.03E-09	0.285	0.294				*APBA1*	5'UTR
cg13883696	1	61,519,888	0.000115	0.285	0.270					
cg05143735	2	127,978,490	9.35E-08	0.285	0.270					
cg17071045	4	54,969,609	3.05E-09	0.285	0.291					
cg08586423	X	102,508,853	0.000205	0.285	0.342				*TCEAL8*	Body
cg18713028	12	54,329,935	1.91E-12	0.285	0.272					
cg09952395	11	63,258,712	0.022901	0.285	0.226				*HRASLS5*	TSS200
cg27454064	12	64,215,611	1.86E-18	0.285	0.275					
cg09526758	12	16,758,047	2.41E-05	0.285	0.246				*LMO3*	5'UTR
cg14120784	7	73,867,457	0.002067	0.285	0.228	0.0000041	0.101		*GTF2IRD1*	TSS1500
cg09251292	6	117,087,056	6.53E-09	0.285	0.225				*FAM162B*	TSS200
cg19406182	22	37,721,149	3.98E-05	0.285	0.185					
cg26579556	14	103,673,450	5.08E-05	0.285	0.170					
cg22030032	19	41,312,792	2.09E-06	0.285	0.281				*EGLN2*	Body

cg25644740	6	29,894,152	6.9E-07	0.285	0.278				*HCG4P6*	TSS1500
cg07385423	1	161,697,574	5.58E-07	0.285	0.262				*FCRLB*	3'UTR
cg12536279	11	711,129	2.1E-19	0.285	0.270				*EPS8L2*	Body
cg04135498	3	120,628,544	0.000334	0.285	0.239				*STXBP5L*	Body
cg04812351	4	73,177,995	2.37E-10	0.285	0.281				*ADAMTS3*	Body
cg07353303	5	145,713,747	9.64E-05	0.285	0.226					
cg19391247	20	2,360,385	1.09E-06	0.285	0.240				*TGM6*	TSS1500
cg24187351	X	68,525,883	1.56E-05	0.285	0.318					
cg09297903	8	42,234,798	1.14E-10	0.285	0.246				*DKK4*	TSS200
cg19978312	5	179,634,688	7.76E-18	0.285	0.270				*RASGEF1C*	5'UTR
cg06871999	7	104,460,407	3.5E-14	0.285	0.290				*LHFPL3*	Body
cg14602530	4	4,859,772	0.004297	0.285	0.284					
cg09275956	1	50,896,251	0.001623	0.285	0.295					
cg18006568	2	71,205,891	0.001236	0.285	0.303	6.33E-25	0.278		*ANKRD53*	1stExon
cg01468514	5	10,563,358	1.04E-11	0.285	0.234				*ANKRD33B*	TSS1500
cg25229172	12	96,336,121	0.003164	0.285	0.144	5.19E-21	0.171		*AMDHD1*	TSS1500
cg07959771	7	121,956,740	0.003433	0.285	0.270					
cg16481280	6	32,120,955	6.72E-05	0.285	0.308				*PPT2*	TSS1500
cg16707405	5	115,152,413	0.004256	0.285	0.173				*CDO1*	TSS200
cg11802027	19	14,049,442	2.75E-10	0.285	0.260				*PODNL1*	Body
cg18628732	6	30,095,295	4.59E-13	0.285	0.268					
cg23709913	2	132,152,429	7.81E-09	0.285	0.258					
cg01297721	19	19,373,566	0.000189	0.285	0.291				*HAPLN4*	5'UTR
cg10165801	17	46,651,186	0.000622	0.285	0.233				*HOXB3*	5'UTR
cg04796181	6	27,839,850	0.029907	0.285	0.313				*HIST1H3I*	1stExon
cg01650776	7	150,748,518	7.57E-14	0.285	0.233				*ACCN3*	Body
cg08166362	3	128,998,952	1.91E-14	0.285	0.244				*C3orf37*	Body
cg03721195	5	137,610,448	0.000833	0.285	0.251				*GFRA3*	TSS200
cg11059159	2	128,453,445	0.004385	0.285	0.205					
cg19402991	2	164,204,915	1.99E-05	0.285	0.212					
cg24660009	6	28,458,175	0.004029	0.285	0.271					
cg14161412	1	76,095,838	0.004001	0.285	0.285					
cg04844977	3	125,691,054	0.017261	0.285	0.261				*ROPN1B*	Body
cg04951947	12	26,349,129	0.022467	0.285	0.209				*SSPN*	5'UTR

cg06422467	6	30,720,484	0.000224	0.285	0.164					
cg26654807	10	81,002,218	0.04006	0.285	0.172				*ZMIZ1*	Body
cg10045354	11	111,169,427	1.62E-14	0.285	0.286				*C11orf93*	TSS1500
cg21590021	8	145,240,760	8.27E-05	0.285	0.275				*HEATR7A*	Body
cg22080436	11	4,212,471	2.75E-07	0.285	0.259					
cg18547299	19	14,049,578	1.33E-07	0.285	0.292				*PODNL1*	Body
cg11903133	20	50,722,613	0.00682	0.285	0.313				*ZFP64*	Body
cg10144198	10	134,001,728	2.3E-06	0.285	0.298				*DPYSL4*	Body
cg14451926	8	23,562,918	8.08E-09	0.285	0.280				*NKX2-6*	Body
cg27573591	7	127,672,152	0.001087	0.285	0.210				*SND1*	Body
cg07578695	4	25,657,414	0.005768	0.285	0.160				*SLC34A2*	TSS200
cg08592890	16	30,907,246	1.07E-07	0.285	0.261				*CTF1*	TSS1500
cg07101841	17	75,315,527	0.044927	0.285	0.278				*SEPT9*	5'UTR
cg22210145	13	28,545,263	8.75E-07	0.285	0.259					
cg19674878	12	117,470,549	4.31E-19	0.285	0.262					
cg16014085	16	30,406,122	4.44E-05	0.285	0.196	1.47E-20	0.214		*ZNF48*	TSS1500
cg18988685	2	233,368,473	4.14E-05	0.285	0.250					
cg20977794	14	51,560,748	4.22E-06	0.285	0.223				*TRIM9*	Body
cg24610231	15	29,967,620	8.12E-12	0.285	0.265					
cg11548303	8	23,567,350	3.95E-19	0.285	0.254					
cg13906811	2	50,575,093	9.97E-08	0.285	0.243				*NRXN1*	Body
cg14699112	4	141,170,338	1.39E-07	0.285	0.249					
cg23891273	12	102,036,400	5.45E-06	0.285	0.282				*MYBPC1*	Body
cg07810164	X	13,588,174	0.000137	0.285	0.292				*EGFL6*	Body
cg03507241	18	12,307,649	3.37E-05	0.285	0.274				*TUBB6*	TSS1500
cg23915769	5	115,297,488	0.023268	0.285	0.375				*LVRN*	TSS1500
cg24332422	5	112,073,686	0.001066	0.285	0.156	1.84E-18	0.177		*APC*	5'UTR
cg11028769	6	25,138,951	0.029034	0.285	0.157				*CMAH*	TSS1500
cg24760467	10	102,760,784	6.71E-09	0.285	0.298				*LZTS2*	5'UTR
cg04786287	16	54,970,523	0.003766	0.285	0.334					
cg12821539	13	100,642,775	0.009419	0.285	0.306					
cg02948476	14	95,235,026	0.013582	0.285	0.339				*GSC*	Body
cg22502382	12	32,701,732	2.31E-08	0.285	0.323				*FGD4*	5'UTR
cg01966091	16	69,141,478	0.014603	0.285	0.270				*HAS3*	5'UTR

cg08398132	2	8,557,381	3.2E-12	0.285	0.268					
cg26826183	19	10,231,701	1.6E-13	0.285	0.246				*EIF3G*	TSS1500
cg20691507	6	28,956,683	0.000694	0.285	0.349			Yes		
cg26814276	4	172,734,266	0.013265	0.285	0.390				*GALNTL6*	TSS1500
cg16108059	14	81,421,989	0.004113	0.285	0.371				*TSHR*	5'UTR
cg19761272	14	76,605,176	0.002155	0.285	0.274					
cg09456782	13	114,146,038	6.03E-06	0.285	0.208				*TMCO3*	5'UTR
cg26149275	2	176,950,007	0.001609	0.285	0.342				*EVX2*	TSS1500
cg04064644	5	1,876,719	1.33E-05	0.285	0.244					
cg04157263	17	37,752,991	2.81E-09	0.285	0.255					
cg17286326	20	57,796,491	1.61E-07	0.285	0.269				*ZNF831*	Body
cg18081863	19	50,015,527	2.47E-16	0.285	0.308				*FCGRT*	TSS200
cg22820316	1	119,532,736	6.02E-07	0.285	0.303				*TBX15*	TSS1500
cg17930194	9	124,982,834	5.33E-06	0.284	0.270				*LHX6*	Body
cg02674384	13	46,425,837	0.004328	0.284	0.253				*SIAH3*	5'UTR
cg17683336	19	11,305,924	7.93E-05	0.284	0.254				*KANK2*	5'UTR
cg01970716	14	24,867,164	2.25E-25	0.284	0.296				*NYNRIN*	TSS1500
cg25827022	17	7,287,652	2.21E-07	0.284	0.263				*TNK1*	Body
cg19045293	X	39,864,984	0.015033	0.284	0.322					
cg13631916	6	117,086,931	0.012132	0.284	0.243				*FAM162B*	TSS200
cg14272084	14	61,685,564	0.000527	0.284	0.202					
cg08304190	20	26,188,997	0.01482	0.284	0.243				*MIR663*	TSS200
cg21985590	6	84,563,910	1.28E-07	0.284	0.288				*RIPPLY2*	Body
cg01737100	17	8,230,130	1.68E-06	0.284	0.263					
cg16700924	1	146,552,102	0.000209	0.284	0.293					
cg21784383	1	217,311,172	0.00021	0.284	0.268				*ESRRG*	TSS200
cg04467162	3	128,764,824	0.00021	0.284	0.239					
cg19423196	10	82,049,429	0.001398	0.284	0.250	1.22E-34	0.254		*MAT1A*	1stExon
cg22557662	19	38,747,374	0.001529	0.284	0.292				*PPP1R14A*	TSS1500
cg15289466	4	103,740,913	3.65E-07	0.284	0.285				*UBE2D3*	Body
cg02591487	12	8,088,741	4.87E-08	0.284	0.286				*SLC2A3*	1stExon
cg01787574	12	16,758,057	1.13E-06	0.284	0.259				*LMO3*	5'UTR
cg07063068	14	91,711,033	2.99E-05	0.284	0.248				*GPR68*	5'UTR
cg20180061	3	181,445,385	0.001077	0.284	0.292				*SOX2OT*	Body

cg12526834	11	120,081,089	2.62E-06	0.284	0.252				*OAF*	TSS1500
cg21109265	6	43,276,647	0.030047	0.284	0.205				*CRIP3*	TSS200
cg17590101	X	48,327,749	3.22E-09	0.284	0.275				*SLC38A5*	5'UTR
cg25530583	18	43,305,617	1.59E-07	0.284	0.258				*SLC14A1*	5'UTR
cg02530022	7	96,642,708	2.85E-07	0.284	0.291				*DLX6AS*	Body
cg09235983	11	58,612,814	0.002694	0.284	0.279				*GLYATL2*	TSS1500
cg01179012	2	131,595,107	1.74E-07	0.284	0.273					
cg13726459	12	54,409,657	4.49E-05	0.284	0.218				*HOXC4*	TSS1500
cg15132165	17	773,137	5.51E-06	0.284	0.278				*NXN*	Body
cg09896120	11	46,317,577	0.00024	0.284	0.208				*CREB3L1*	Body
cg16191307	12	63,754,169	4.88E-06	0.284	0.199					
cg00832555	3	169,529,716	0.000852	0.284	0.209				*LRRC34*	Body
cg27057027	7	27,243,276	0.018015	0.284	0.365					
cg15718581	4	188,916,581	0.015413	0.284	0.283				*ZFP42*	TSS1500
cg23704195	17	59,531,104	6.21E-12	0.284	0.264					
cg17113856	6	32,120,895	6.57E-10	0.284	0.284				*PPT2*	TSS1500
cg19309580	1	9,747,263	0.049545	0.284	0.275				*PIK3CD*	5'UTR
cg08767820	7	51,539,583	2.06E-06	0.284	0.266					
cg16510793	9	116,450,346	1.33E-06	0.284	0.272					
cg17119387	17	2,615,431	0.003788	0.284	0.207	1.61E-22	0.177		*KIAA0664*	TSS1500
cg25950112	5	170,740,870	0.022621	0.284	0.300					
cg25991569	13	35,324,682	0.000603	0.284	0.299					
cg10684686	6	28,557,047	0.002567	0.284	0.229					
cg13321661	1	62,319,602	2.05E-08	0.284	0.266				*INADL*	Body
cg03200166	11	61,335,254	0.008697	0.284	0.143				*SYT7*	Body
cg08515442	6	168,778,225	6.11E-12	0.284	0.252					
cg04442631	22	36,593,144	1.21E-11	0.284	0.268				*APOL4*	Body
cg06197966	10	29,948,324	1.05E-08	0.284	0.242				*SVIL*	5'UTR
cg22252475	X	134,654,383	0.006261	0.284	0.157				*DDX26B*	TSS200
cg00739120	19	36,035,875	0.000542	0.284	0.151	1.03E-25	0.214		*GAPDHS*	Body
cg05534148	7	1,398,159	0.000437	0.284	0.316					
cg18147605	12	6,663,049	4.28E-10	0.284	0.293				*IFFO1*	Body
cg19695266	1	1,241,672	2.07E-18	0.284	0.307				*ACAP3*	Body
cg14629509	7	134,143,991	0.001104	0.284	0.176				*AKR1B1*	TSS200

cg16832407	9	73,736,539	3.89E-05	0.284	0.262	1.29E-17	0.197		*TRPM3*	TSS200
cg02463253	17	27,369,851	6.27E-08	0.284	0.287				*PIPOX*	TSS200
cg24553170	1	49,243,472	3.36E-16	0.284	0.263				*AGBL4*	Body
cg11649981	5	174,178,104	0.019112	0.284	0.225					
cg19350020	11	118,489,198	1.25E-18	0.284	0.278				*PHLDB1*	Body
cg22215815	12	57,619,178	1.19E-06	0.284	0.294				*NXPH4*	Body
cg09671258	1	180,202,530	0.029105	0.284	0.315				*LHX4*	Body
cg02336762	12	118,313,990	0.000774	0.284	0.322				*KSR2*	Body
cg08198176	3	37,494,602	3.26E-11	0.284	0.274				*ITGA9*	Body
cg00101715	3	101,497,475	1.99E-05	0.284	0.203				*FAM55C*	TSS1500
cg02745847	17	47,075,880	8.64E-06	0.284	0.253				*IGF2BP1*	Body
cg13023870	14	37,124,018	8.28E-06	0.284	0.300					
cg16546503	18	12,307,329	6.94E-05	0.284	0.276				*TUBB6*	TSS1500
cg18196782	1	232,941,176	0.016495	0.284	0.243				*KIAA1383*	1stExon
cg14298457	2	177,012,117	4.58E-08	0.284	0.219					
cg07860213	8	70,982,039	0.000521	0.284	0.294				*PRDM14*	Body
cg20247486	1	119,544,329	6.49E-08	0.284	0.248					
cg18255595	13	20,735,871	2.21E-06	0.284	0.265				*GJA3*	TSS1500
cg22610620	10	77,524,487	3.03E-08	0.284	0.260					
cg01355242	X	56,258,387	7.68E-13	0.284	0.250				*KLF8*	TSS1500
cg07393734	11	44,341,451	0.002317	0.284	0.281					
cg25527090	10	118,894,180	0.000132	0.284	0.221				*VAX1*	Body
cg01154046	10	17,270,601	0.030807	0.284	0.339				*VIM*	5'UTR
cg08814148	X	118,407,645	0.001244	0.284	0.246					
cg24404823	1	247,496,053	0.029953	0.284	0.149				*ZNF496*	TSS1500
cg04455137	7	120,629,316	1.54E-07	0.284	0.256				*C7orf58*	5'UTR
cg18125573	17	38,506,350	0.009714	0.284	0.227				*RARA*	Body
cg19883813	10	8,098,005	2.88E-08	0.284	0.227				*GATA3*	Body
cg04098194	1	1,241,087	6.15E-14	0.284	0.303				*ACAP3*	Body
cg07598755	16	30,023,028	0.000676	0.283	0.212				*DOC2A*	TSS1500
cg17110920	1	27,893,925	3.81E-18	0.283	0.275				*AHDC1*	5'UTR
cg00469207	7	152,622,455	0.012065	0.283	0.390					
cg22250498	11	63,258,744	0.023206	0.283	0.251				*HRASLS5*	TSS200
cg01162672	6	133,562,275	6.52E-08	0.283	0.313				*EYA4*	TSS1500

cg14071579	9	126,794,830	0.000458	0.283	0.382				*LHX2*	Body
cg26065488	3	171,176,016	4.71E-08	0.283	0.288				*TNIK*	Body
cg07962303	19	11,253,944	0.000465	0.283	0.211					
cg18734330	14	95,876,455	4.44E-13	0.283	0.280				*C14orf139*	TSS200
cg04660410	3	38,036,034	0.018952	0.283	0.111	0.0000212	0.121		*VILL*	Body
cg17208748	3	197,194,394	1.25E-10	0.283	0.291					
cg00278392	6	16,802,505	4.94E-11	0.283	0.257					
cg07011913	2	177,014,626	5.34E-08	0.283	0.258				*HOXD4*	TSS1500
cg04193484	12	57,620,856	3.37E-06	0.283	0.306					
cg27175634	1	77,748,728	0.024566	0.283	0.075				*AK5*	Body
cg18356276	7	17,337,342	7.29E-40	0.283	0.269				*AHR*	TSS1500
cg07862488	14	60,977,054	6.41E-07	0.283	0.251				*SIX6*	Body
cg24784350	21	47,518,998	0.00131	0.283	0.185				*COL6A2*	5'UTR
cg00185066	13	80,910,763	1.95E-06	0.283	0.290				*SPRY2*	3'UTR
cg16331883	12	53,886,170	4.14E-07	0.283	0.250				*MAP3K12*	5'UTR
cg07083464	7	27,240,758	0.034813	0.283	0.348				*HOXA13*	TSS1500
cg06470822	6	28,175,283	3.82E-05	0.283	0.251					
cg22757019	7	91,808,805	1.9E-06	0.283	0.221					
cg12980127	5	2,129,874	0.024469	0.283	0.380					
cg09941363	1	51,730,449	5.16E-07	0.283	0.268				*RNF11*	Body
cg12417775	1	212,463,238	2.32E-23	0.283	0.295				*PPP2R5A*	Body
cg20980055	6	133,562,258	0.001401	0.283	0.334				*EYA4*	TSS1500
cg14059835	9	132,598,917	1.58E-07	0.283	0.257				*C9orf78*	TSS1500
cg09041207	16	87,417,818	0.012058	0.283	0.215				*FBXO31*	TSS1500
cg11274314	2	242,480,504	3.53E-07	0.283	0.280					
cg27548752	19	47,263,614	4.61E-18	0.283	0.277					
cg02628823	4	141,419,587	0.000128	0.283	0.277					
cg25417335	17	15,313,159	3.56E-23	0.283	0.300					
cg23799901	7	124,404,431	0.000178	0.283	0.186				*GPR37*	1stExon
cg00651400	6	3,230,007	1.07E-08	0.283	0.260					
cg11139090	5	176,294,068	2.6E-06	0.283	0.270				*UNC5A*	Body
cg01817521	20	61,584,108	1.74E-08	0.283	0.265				*SLC17A9*	1stExon
cg00952054	1	9,437,047	1.58E-10	0.283	0.300					
cg06640593	2	568,321	1.23E-09	0.283	0.239					

cg20770175	2	189,839,474	9.27E-09	0.283	0.306	2.28E-08	0.164		*COL3A1*	Body
cg10112943	4	148,401,351	1.16E-12	0.283	0.278				*EDNRA*	TSS1500
cg21146537	22	35,695,301	6.6E-08	0.283	0.181				*TOM1*	TSS1500
cg25900280	17	59,534,847	7.84E-08	0.283	0.249				*TBX4*	Body
cg14367995	9	110,253,800	5.02E-06	0.283	0.273					
cg04293930	6	35,465,845	0.000161	0.283	0.200				*TULP1*	3'UTR
cg02959609	1	151,693,503	2.47E-08	0.283	0.251				*C1orf230*	TSS1500
cg06846752	1	32,929,715	8.45E-05	0.283	0.220				*ZBTB8B*	TSS1500
cg18075315	12	106,988,393	2.31E-09	0.283	0.289				*RFX4*	Body
cg20772590	17	75,446,431	3.46E-05	0.283	0.179				*SEPT9*	TSS200
cg14258853	12	29,935,411	0.000467	0.283	0.208				*TMTC1*	5'UTR
cg18009621	11	114,129,200	1.21E-11	0.283	0.338					
cg02909688	1	147,752,823	0.001368	0.283	0.266					
cg19776515	8	57,232,395	0.00957	0.283	0.367				*SDR16C5*	5'UTR
cg10149836	17	29,719,312	1.11E-09	0.283	0.204	2.5E-17	0.177		*RAB11FIP4*	Body
cg20016673	1	108,023,366	3.62E-09	0.283	0.252				*NTNG1*	Body
cg02249292	10	28,035,655	4.96E-09	0.283	0.284				*MKX*	TSS1500
cg02580923	12	117,470,541	2.13E-18	0.283	0.247					
cg18878437	17	7,826,413	0.002022	0.283	0.285				*KCNAB3*	3'UTR
cg26327071	17	46,655,818	0.023765	0.283	0.303				*HOXB4*	TSS200
cg27189803	10	80,416,566	4.59E-13	0.283	0.267					
cg14435807	15	74,218,780	1.91E-17	0.283	0.262	2.01E-21	0.191		*LOXL1*	TSS200
cg22884020	3	38,081,186	4.29E-05	0.283	0.182				*DLEC1*	Body
cg24410453	6	53,919,041	0.004069	0.283	0.366				*C6orf142*	Body
cg19792599	11	125,774,406	1.58E-06	0.283	0.240				*DDX25*	5'UTR
cg05348973	1	110,697,298	0.001415	0.283	0.287				*SLC6A17*	5'UTR
cg13475665	3	65,582,925	9.61E-07	0.283	0.292				*MAGI1*	Body
cg07471461	6	37,751,706	0.036129	0.283	0.189					
cg04184143	5	50,263,500	0.008497	0.283	0.275					
cg16712127	X	119,149,186	0.00119	0.283	0.229					
cg14571710	6	30,655,896	8.48E-23	0.283	0.270				*KIAA1949*	TSS1500
cg15392819	1	180,585,679	1.89E-19	0.283	0.265					
cg00777121	3	50,378,191	6.95E-06	0.283	0.283	3.55E-10	0.144		*RASSF1*	1stExon
cg03671052	5	180,086,260	0.001916	0.283	0.320					

cg06844012	3	159,944,430	4.11E-05	0.283	0.162				*LOC401097*	3'UTR
cg07544796	6	36,817,048	0.032222	0.283	0.094					
cg10786645	19	36,352,376	0.001979	0.283	0.304				*KIRREL2*	Body
cg27078824	6	44,187,399	0.029133	0.283	0.197				*SLC29A1*	1stExon
cg15651928	20	32,290,811	4.72E-07	0.283	0.256				*PXMP4*	3'UTR
cg18295744	10	80,872,926	1.52E-15	0.283	0.256				*ZMIZ1*	5'UTR
cg16716883	10	74,079,772	0.018484	0.283	0.194					
cg14482998	6	111,984,370	1.38E-11	0.283	0.249				*FYN*	Body
cg22152728	11	20,185,382	0.019989	0.283	0.337					
cg24319381	15	83,316,911	0.000803	0.283	0.255				*CPEB1*	TSS200
cg25649765	2	11,672,761	1.81E-13	0.283	0.267				*GREB1*	TSS1500
cg07505018	16	54,968,831	0.00468	0.283	0.354					
cg13215970	4	74,864,596	1.96E-05	0.283	0.226				*CXCL5*	TSS200
cg15571561	3	35,706,161	0.001132	0.283	0.278				*ARPP-21*	5'UTR
cg26708220	15	37,387,577	0.028095	0.283	0.378				*MEIS2*	Body
cg20633818	6	27,799,380	0.003907	0.283	0.224				*HIST1H4K*	TSS200
cg16582517	3	5,025,885	0.001135	0.283	0.129				*BHLHE40*	3'UTR
cg18729001	12	45,443,808	4.26E-38	0.282	0.277				*DBX2*	Body
cg21582112	12	54,421,335	1.39E-07	0.282	0.223				*HOXC4*	5'UTR
cg27645968	19	406,407	4.32E-15	0.282	0.273				*C2CD4C*	3'UTR
cg25529393	6	27,858,380	1.01E-05	0.282	0.284				*HIST1H3J*	1stExon
cg21683708	6	36,628,755	0.003504	0.282	0.228					
cg03355101	19	16,449,718	1.77E-06	0.282	0.285					
cg11085282	22	46,262,203	0.00229	0.282	0.318					
cg00308438	1	92,329,399	6.21E-09	0.282	0.292				*TGFBR3*	5'UTR
cg00437102	2	10,231,846	2.17E-07	0.282	0.265					
cg27649971	11	711,205	2.62E-17	0.282	0.276				*EPS8L2*	Body
cg09488203	10	95,327,884	1.71E-05	0.282	0.305				*GPR120*	Body
cg06811094	1	200,272,112	1.55E-07	0.282	0.268					
cg21370856	16	23,848,003	3.17E-06	0.282	0.238				*PRKCB*	Body
cg04829485	14	104,550,970	1.5E-10	0.282	0.263				*ASPG*	TSS1500
cg09427311	9	129,885,730	7.61E-05	0.282	0.232				*ANGPTL2*	TSS1500
cg11225297	3	130,064,318	0.001304	0.282	0.200				*COL29A1*	TSS200
cg17229678	6	32,121,555	4.38E-08	0.282	0.249				*PPT2*	Body

cg22867719	1	19,276,236	7.51E-14	0.282	0.265				*IFFO2*	Body
cg15878619	6	30,687,373	4.42E-08	0.282	0.248				*TUBB*	TSS1500
cg17567700	22	51,112,218	3.83E-05	0.282	0.293				*SHANK3*	TSS1500
cg04652097	7	79,083,445	0.012438	0.282	0.195				*MAGI2*	TSS1500
cg27114640	1	18,437,320	9.61E-05	0.282	0.247				*IGSF21*	Body
cg09209510	14	100,288,389	8.51E-14	0.282	0.256				*EML1*	Body
cg07788369	7	96,619,068	1.64E-07	0.282	0.269				*DLX6AS*	Body
cg14550985	11	66,104,159	5.98E-13	0.282	0.293				*RIN1*	TSS200
cg22534145	20	23,015,936	0.000687	0.282	0.202				*SSTR4*	TSS200
cg02590886	7	87,848,358	0.005687	0.282	0.193				*SRI*	Body
cg03107888	7	27,241,203	0.019201	0.282	0.326				*HOXA13*	TSS1500
cg16608018	10	122,739,544	2.87E-12	0.282	0.231					
cg27296341	6	154,485,839	2.09E-19	0.282	0.295				*IPCEF1*	Body
cg17456100	1	22,140,859	5.72E-10	0.282	0.222				*LDLRAD2*	Body
cg00970325	3	142,683,176	0.007227	0.282	0.299	3.25E-09	0.217		*PAQR9*	TSS1500
cg00694258	10	32,038,939	1.47E-08	0.282	0.264					
cg15741433	6	26,018,084	0.000344	0.282	0.241				*HIST1H1A*	TSS200
cg11197916	1	26,249,040	1.72E-06	0.282	0.161					
cg00118808	9	132,097,931	2.67E-05	0.282	0.231					
cg17380661	6	100,912,946	0.010384	0.282	0.371				*SIM1*	TSS1500
cg01460276	8	18,871,817	0.007629	0.282	0.187				*PSD3*	TSS1500
cg24803719	17	45,855,879	2.64E-05	0.282	0.218					
cg25777540	X	101,906,428	0.015051	0.282	0.297				*GPRASP1*	1stExon
cg26162932	4	81,106,342	0.013393	0.282	0.368				*PRDM8*	TSS200
cg23090632	8	94,712,373	0.007196	0.282	0.161				*FAM92A1*	TSS1500
cg02431672	1	43,534,324	0.001353	0.282	0.100					
cg16584431	X	117,250,153	1.21E-07	0.282	0.248				*KLHL13*	5'UTR
cg19268434	6	32,076,967	6.59E-05	0.282	0.270				*TNXB*	5'UTR
cg22682811	20	21,372,462	7E-12	0.282	0.292					
cg10022722	16	2,041,826	5.45E-05	0.282	0.235				*SYNGR3*	Body
cg18556834	14	24,641,070	2.41E-11	0.282	0.282				*REC8*	TSS200
cg03966514	13	36,919,738	0.011467	0.282	0.303				*SPG20*	5'UTR
cg19248676	12	115,135,926	2.7E-10	0.282	0.250					
cg24761507	17	35,293,930	0.045625	0.282	0.171				*LHX1*	TSS1500

cg03599147	11	78,551,798	9.58E-12	0.282	0.289				*ODZ4*	Body
cg04804717	7	1,300,320	0.008319	0.282	0.281					
cg07341624	20	2,539,167	6.33E-05	0.282	0.278				*TMC2*	Body
cg23760687	13	25,621,735	0.008174	0.282	0.145					
cg19796981	22	50,438,501	3.9E-07	0.282	0.277				*IL17REL*	Body
cg24054180	11	112,157,241	1.49E-21	0.282	0.286					
cg26724975	12	46,554,412	2.31E-38	0.282	0.263					
cg19045239	19	46,799,505	7.4E-10	0.282	0.254				*HIF3A*	TSS1500
cg21229055	X	13,957,233	1.07E-07	0.282	0.260				*GPM6B*	TSS1500
cg12073779	2	208,989,469	6.12E-09	0.282	0.287	1.28E-13	0.196		*CRYGD*	TSS200
cg13922291	1	46,914,528	6.31E-09	0.282	0.275					
cg17155018	6	111,953,066	1.46E-25	0.282	0.267					
cg04774469	14	21,562,632	3.78E-14	0.282	0.292				*ZNF219*	5'UTR
cg16572876	4	56,024,045	6.86E-06	0.282	0.287					
cg10562586	2	210,444,154	3.19E-07	0.282	0.283	1.1E-08	0.102		*MAP2*	TSS1500
cg06509837	19	610,955	4.52E-05	0.282	0.207				*HCN2*	Body
cg10625533	2	233,498,682	1.56E-07	0.282	0.251				*EFHD1*	1stExon
cg18021286	11	115,530,371	3.08E-06	0.282	0.229					
cg13482308	9	37,002,685	0.000442	0.282	0.253				*PAX5*	Body
cg15373592	6	56,405,167	0.001429	0.282	0.224				*DST*	Body
cg21757872	12	107,974,453	4.07E-14	0.282	0.279				*BTBD11*	TSS200
cg06593753	7	51,539,323	1.56E-11	0.282	0.284					
cg18184071	1	149,670,096	1.21E-23	0.282	0.291					
cg19352038	2	223,164,869	1.48E-07	0.282	0.296	1.78E-16	0.189		*PAX3*	TSS1500
cg23970275	2	208,008,052	1.46E-09	0.282	0.293				*KLF7*	Body
cg15573040	15	92,938,295	0.012179	0.282	0.219				*ST8SIA2*	Body
cg02701825	17	73,083,632	9.07E-12	0.282	0.223				*SLC16A5*	TSS1500
cg13920090	1	27,872,303	3.04E-12	0.282	0.296				*AHDC1*	3'UTR
cg25007447	12	93,521,330	2.6E-16	0.282	0.271					
cg10233654	12	89,744,621	1.09E-11	0.282	0.261				*DUSP6*	Body
cg15922057	17	25,784,714	2E-07	0.282	0.348					
cg22488256	1	66,258,046	0.00013	0.282	0.278				*PDE4B*	TSS200
cg16053580	19	51,568,863	1.4E-07	0.282	0.274				*KLK13*	TSS1500
cg08873266	1	40,255,052	0.000565	0.282	0.147				*BMP8B*	TSS1500

cg10206344	16	31,483,277	0.001071	0.282	0.125				*TGFB1I1*	TSS1500
cg12645616	10	103,070,480	0.0006	0.282	0.256					
cg21543859	6	45,391,645	0.003796	0.282	0.201				*RUNX2*	Body
cg10402698	15	67,000,045	9.69E-08	0.281	0.312				*SMAD6*	TSS1500
cg00108715	3	52,565,015	1.09E-21	0.281	0.257				*NT5DC2*	Body
cg03614193	18	55,021,542	0.001453	0.281	0.195				*ST8SIA3*	Body
cg23346544	8	80,731,144	0.008078	0.281	0.185					
cg18178197	22	50,098,317	5.83E-07	0.281	0.304					
cg23221603	2	26,963,883	1.51E-11	0.281	0.268					
cg08431536	2	223,161,894	0.022777	0.281	0.327				*PAX3*	Body
cg16501625	16	54,628,759	0.001731	0.281	0.216					
cg11941920	6	79,339,195	0.000151	0.281	0.297					
cg14651249	9	135,465,030	0.030167	0.281	0.338				*BARHL1*	3'UTR
cg24940155	13	93,896,820	8.45E-14	0.281	0.247				*GPC6*	Body
cg23738708	1	206,240,661	1.25E-08	0.281	0.264				*C1orf186*	Body
cg07161827	2	71,680,559	0.037647	0.281	0.187				*DYSF*	TSS200
cg00114029	1	35,351,407	0.002077	0.281	0.259				*DLGAP3*	Body
cg11784631	6	30,658,014	4.07E-15	0.281	0.266				*NRM*	Body
cg22689630	19	51,568,918	1.2E-06	0.281	0.286				*KLK13*	TSS1500
cg25338563	8	103,669,495	0.018818	0.281	0.185					
cg03637769	3	195,870,092	0.000143	0.281	0.168					
cg13306478	13	31,317,745	4.19E-16	0.281	0.260				*ALOX5AP*	Body
cg00042837	17	37,028,650	1.45E-12	0.281	0.279				*LASP1*	Body
cg06272916	22	42,306,237	0.037366	0.281	0.258					
cg11719362	7	25,900,355	0.001515	0.281	0.281					
cg20116805	12	54,363,054	2.52E-05	0.281	0.308				*HOTAIR*	TSS1500
cg20586076	10	133,252,332	1.76E-05	0.281	0.293					
cg05997350	10	50,817,104	0.047515	0.281	0.349				*SLC18A3*	TSS1500
cg12961080	14	99,736,143	0.001175	0.281	0.236				*BCL11B*	Body
cg26803305	18	43,304,452	3.01E-12	0.281	0.261	2.13E-12	0.184		*SLC14A1*	5'UTR
cg05137450	2	233,368,263	0.022843	0.281	0.291					
cg02493602	11	86,383,696	0.025437	0.281	0.177				*ME3*	TSS1500
cg14238325	6	43,276,781	0.001682	0.281	0.248				*CRIP3*	TSS1500
cg16038738	1	109,103,731	4.32E-10	0.281	0.274				*FAM102B*	Body

cg03226666	6	124,151,503	4.79E-05	0.281	0.267				*NKAIN2*	Body
cg12215294	3	40,350,768	0.000558	0.281	0.294				*EIF1B*	TSS1500
cg19831403	10	88,453,678	1.2E-07	0.281	0.236				*LDB3*	Body
cg25701114	6	26,031,939	0.000114	0.281	0.279				*HIST1H3B*	1stExon
cg08995609	11	66,104,115	3.16E-12	0.281	0.275				*RIN1*	TSS200
cg13492692	9	126,777,673	2.13E-05	0.281	0.346				*LHX2*	Body
cg15027606	X	53,077,819	0.012527	0.281	0.317				*GPR173*	TSS1500
cg16097858	2	46,765,700	2.28E-12	0.281	0.283					
cg17543488	5	172,672,959	0.000194	0.281	0.331					
cg22780193	13	74,825,252	1.19E-09	0.281	0.231					
cg07909444	17	67,585,094	2.57E-14	0.281	0.277					
cg10338787	17	72,931,767	2.66E-07	0.281	0.231				*OTOP3*	TSS200
cg16863294	X	72,668,847	0.004112	0.281	0.274				*CDX4*	Body
cg25714115	7	73,895,061	3.41E-05	0.281	0.221				*GTF2IRD1*	5'UTR
cg10046388	7	129,425,858	8.21E-06	0.281	0.280			Yes		
cg25292098	20	31,446,183	1.2E-13	0.281	0.287				*EFCAB8*	TSS1500
cg26429925	15	83,953,775	3.64E-05	0.281	0.251				*BNC1*	TSS1500
cg12431299	2	241,758,282	2.14E-05	0.281	0.291				*KIF1A*	5'UTR
cg16876823	19	2,987,082	1.15E-12	0.281	0.252				*TLE6*	Body
cg05673098	3	131,752,709	0.038941	0.281	0.437				*CPNE4*	5'UTR
cg12359279	21	42,797,953	2.25E-05	0.281	0.179				*MX1*	TSS200
cg14666113	8	11,561,724	0.019402	0.281	0.285				*GATA4*	1stExon
cg14684709	13	100,547,249	0.01157	0.281	0.209				*CLYBL*	3'UTR
cg03408135	1	207,842,833	0.000369	0.281	0.263				*CR1L*	Body
cg01505878	11	61,690,927	6.33E-15	0.281	0.277					
cg18245316	8	144,962,981	1.37E-21	0.281	0.275					
cg19704853	11	125,133,609	4.19E-18	0.281	0.288				*PKNOX2*	5'UTR
cg21637392	7	5,735,123	3.32E-06	0.281	0.269				*RNF216*	Body
cg12654412	16	71,459,826	8.13E-05	0.281	0.271					
cg26035171	17	28,705,464	0.000201	0.281	0.230				*CPD*	TSS1500
cg23718026	3	16,555,379	0.046323	0.281	0.073				*RFTN1*	TSS200
cg16408970	10	28,965,584	1.77E-07	0.281	0.221	1.49E-10	0.125		*BAMBI*	TSS1500
cg09417809	12	115,131,223	0.002265	0.281	0.344					
cg04520169	6	29,894,195	3.6E-05	0.281	0.276				*HCG4P6*	TSS1500

cg05291374	11	111,847,475	6.63E-07	0.281	0.235				*DIXDC1*	TSS1500
cg06026468	11	76,315,513	2.79E-13	0.281	0.275					
cg08103988	17	6,558,365	0.002741	0.281	0.316					
cg08762247	17	53,800,372	0.002655	0.281	0.301	1.05E-22	0.268		*TMEM100*	TSS200
cg05060949	7	156,798,581	0.000396	0.281	0.317				*MNX1*	Body
cg18367631	7	653,309	1.7E-06	0.281	0.281				*PRKAR1B*	Body
cg06414921	6	31,276,504	0.030614	0.281	0.242					
cg10494684	17	26,645,200	0.0037	0.281	0.295				*TMEM97*	TSS1500
cg04182483	10	121,259,610	0.000357	0.281	0.216			Yes	*RGS10*	3'UTR
cg13480493	9	102,613,887	1.18E-09	0.281	0.255				*NR4A3*	Body
cg08290076	11	110,581,434	2.05E-05	0.281	0.270				*ARHGAP20*	Body
cg25685519	14	52,536,721	2.63E-08	0.281	0.270				*NID2*	TSS1500
cg06480171	2	37,853,482	1.18E-10	0.281	0.270					
cg03120289	1	99,470,550	0.005451	0.281	0.333				*LPPR5*	TSS200
cg13413982	13	44,868,172	5.98E-17	0.281	0.294					
cg18534491	14	24,641,155	3.98E-12	0.281	0.282				*REC8*	TSS200
cg04759376	10	77,190,437	5.95E-11	0.281	0.245					
cg05194346	15	101,305,443	1.3E-13	0.281	0.261					
cg09004287	6	10,426,387	3.68E-06	0.281	0.276					
cg26232308	1	18,201,537	1.68E-15	0.281	0.275					
cg23906738	14	36,987,301	0.048421	0.281	0.286				*NKX2-1*	Body
cg21337832	X	24,665,655	0.025534	0.281	0.256				*PCYT1B*	Body
cg18750960	2	177,016,417	3.07E-05	0.281	0.266	1.94E-13	0.155		*HOXD4*	1stExon
cg19237113	1	6,803,800	1.47E-05	0.281	0.295					
cg21596858	1	155,007,254	2.27E-10	0.281	0.230	7.12E-18	0.178		*DCST1*	Body
cg06721601	7	101,762,633	4.46E-14	0.281	0.254				*CUX1*	Body
cg19763461	2	63,284,557	2.74E-05	0.281	0.251					
cg21317965	6	50,803,850	4.06E-05	0.281	0.267				*TFAP2B*	Body
cg25928986	7	96,647,079	2.49E-05	0.281	0.299					
cg10049789	4	8,202,531	4.91E-05	0.281	0.267				*SH3TC1*	5'UTR
cg18041884	12	109,162,555	0.001531	0.281	0.168					
cg14630933	6	32,076,093	1.86E-06	0.281	0.320				*TNXB*	5'UTR
cg14769786	17	1,881,333	0.002067	0.281	0.283				*RTN4RL1*	Body
cg10835584	18	55,108,852	0.030486	0.281	0.307				*ONECUT2*	Body

cg26391832	2	45,237,929	6.15E-06	0.281	0.216				*SIX2*	TSS1500
cg22226592	1	109,439,213	5.63E-19	0.281	0.263				*GPSM2*	Body
cg13409645	19	55,866,261	0.001546	0.281	0.186				*COX6B2*	TSS200
cg17677030	2	50,575,069	7.73E-06	0.281	0.187				*NRXN1*	Body
cg25790298	3	172,031,063	6.2E-19	0.281	0.276				*FNDC3B*	Body
cg02506527	X	39,868,153	2.62E-06	0.281	0.265					
cg26665891	1	204,159,832	0.016445	0.281	0.234				*KISS1*	Body
cg21210224	15	65,186,306	0.001485	0.281	0.236					
cg27559661	16	57,125,836	0.047286	0.281	0.222				*CPNE2*	TSS1500
cg03733760	11	69,517,841	1.45E-05	0.281	0.301				*FGF19*	Body
cg07890839	15	67,359,525	0.000185	0.281	0.187				*SMAD3*	Body
cg01922613	1	94,560,851	3.41E-11	0.281	0.236				*ABCA4*	Body
cg12811449	14	60,972,853	1.56E-06	0.280	0.266					
cg17338430	5	170,739,853	0.012617	0.280	0.317					
cg03404566	17	6,899,310	1.65E-06	0.280	0.207				*ALOX12*	TSS200
cg16137928	6	30,720,491	0.000459	0.280	0.154					
cg03741862	17	63,225,392	0.00089	0.280	0.316					
cg06418113	18	43,915,901	6.76E-19	0.280	0.273				*RNF165*	Body
cg08599448	19	11,689,614	2.68E-05	0.280	0.276				*ACP5*	TSS1500
cg06402325	1	85,464,166	2.39E-12	0.280	0.263				*MCOLN2*	TSS1500
cg19084726	1	145,075,538	2.56E-05	0.280	0.201				*PDE4DIP*	Body
cg07448795	22	21,368,419	4.6E-05	0.280	0.297				*P2RX6*	TSS1500
cg11303301	8	144,577,185	9.12E-20	0.280	0.259				*ZC3H3*	Body
cg19132069	11	124,631,666	1.36E-16	0.280	0.274				*ESAM*	Body
cg24319171	12	187,480	1.46E-13	0.280	0.230				*IQSEC3*	Body
cg05857999	6	31,650,760	1.02E-11	0.280	0.290					
cg10617784	2	71,134,586	0.000142	0.280	0.285				*VAX2*	Body
cg08618463	4	146,467,791	3.1E-13	0.280	0.273				*SMAD1*	Body
cg23119604	6	30,227,854	1.71E-09	0.280	0.282				*HLA-L*	Body
cg01636505	14	94,408,232	3.74E-25	0.280	0.249				*ASB2*	Body
cg03255953	3	127,056,837	3.76E-06	0.280	0.340					
cg19312667	4	25,090,491	0.000619	0.280	0.264					
cg22209929	3	172,394,700	3.68E-12	0.280	0.287				*NCEH1*	5'UTR
cg08865350	19	408,639	7.7E-06	0.280	0.215				*C2CD4C*	5'UTR

cg07431286	5	72,526,496	0.024975	0.280	0.273					
cg14328115	11	2,554,562	1.96E-07	0.280	0.270				*KCNQ1*	Body
cg02666566	6	2,510,173	9.93E-17	0.280	0.260					
cg06452129	16	85,265,566	4.78E-09	0.280	0.222					
cg08961621	21	39,644,244	2.08E-12	0.280	0.247				*KCNJ15*	5'UTR
cg17495087	8	96,085,689	4.58E-06	0.280	0.263					
cg06638787	6	5,997,243	0.000281	0.280	0.270					
cg07023459	1	113,265,410	1.74E-07	0.280	0.287				*FAM19A3*	Body
cg16546016	4	121,994,092	3.05E-05	0.280	0.280				*C4orf31*	TSS1500
cg11225405	1	117,038,246	1.13E-11	0.280	0.285					
cg19388776	3	71,835,112	4.01E-07	0.280	0.258				*PROK2*	TSS1500
cg26597539	5	40,680,670	0.009299	0.280	0.366				*PTGER4*	5'UTR
cg03749016	8	23,564,591	0.000439	0.280	0.199				*NKX2-6*	TSS1500
cg19885625	11	64,815,313	2.15E-05	0.280	0.282				*NAALADL1*	Body
cg02479497	16	85,548,895	9.18E-06	0.280	0.263					
cg08883078	2	97,291,520	1.62E-10	0.280	0.259				*KIAA1310*	Body
cg14598478	15	84,363,061	1.35E-07	0.280	0.263				*ADAMTSL3*	Body
cg10253457	15	101,460,077	0.045617	0.280	0.135				*LRRK1*	5'UTR
cg19024969	17	32,905,966	0.004037	0.280	0.265				*C17orf102*	1stExon
cg22928082	20	3,218,522	2.64E-14	0.280	0.257				*SLC4A11*	TSS200
cg11015893	7	135,433,540	1.02E-09	0.280	0.230				*FAM180A*	1stExon
cg02816816	4	17,783,356	2.44E-06	0.280	0.257				*FAM184B*	TSS1500
cg00968638	2	43,450,965	0.002846	0.280	0.224				*ZFP36L2*	3'UTR
cg06001519	13	23,500,838	0.000804	0.280	0.289					
cg23642827	10	98,751,284	6.07E-18	0.280	0.276					
cg07176692	1	113,500,329	4.84E-05	0.280	0.174				*SLC16A1*	TSS1500
cg14784372	10	2,951,257	7.63E-08	0.280	0.236					
cg01357135	3	160,475,128	1.02E-09	0.280	0.289				*PPM1L*	Body
cg25360181	6	43,970,084	0.022306	0.280	0.339				*C6orf223*	Body
cg10327400	14	42,075,406	2.42E-06	0.280	0.350				*LRFN5*	TSS1500
cg10809134	2	47,879,734	8.58E-15	0.280	0.274					
cg13012916	14	36,973,691	0.000819	0.280	0.380				*SFTA3*	Body
cg04422003	7	28,448,295	5.22E-11	0.280	0.258				*CREB5*	5'UTR
cg10893278	12	54,377,225	0.000206	0.280	0.233					

cg18537730	19	49,250,080	2.97E-08	0.280	0.274				*IZUMO1*	1stExon
cg22525688	7	74,001,908	2.09E-09	0.280	0.266				*GTF2IRD1*	Body
cg04689048	19	46,999,289	1E-09	0.280	0.289				*PNMAL2*	TSS200
cg00503383	1	91,301,651	2.19E-05	0.280	0.320					
cg02853616	4	94,755,787	1.39E-06	0.280	0.282					
cg13736811	19	41,873,930	8.55E-11	0.280	0.263				*TMEM91*	5'UTR
cg26701289	15	73,611,289	8.22E-05	0.280	0.248					
cg25012961	5	124,034,288	6.55E-09	0.280	0.265				*ZNF608*	Body
cg01368217	10	127,836,603	0.000166	0.280	0.275				*ADAM12*	Body
cg23619365	13	112,712,009	0.029174	0.280	0.350					
cg08342194	6	31,683,075	1.03E-09	0.280	0.295				*LY6G6E*	TSS1500
cg18270378	7	3,284,394	8.57E-13	0.280	0.273					
cg22488857	7	100,489,022	3.78E-05	0.280	0.277				*ACHE*	Body
cg13813458	17	35,085,134	7.81E-09	0.280	0.289					
cg20041567	6	27,841,067	0.000194	0.280	0.290				*HIST1H4L*	1stExon
cg08667128	17	29,886,885	0.000868	0.280	0.353				*MIR193A*	TSS200
cg02340083	1	50,882,006	0.025333	0.280	0.234					
cg03020810	10	102,890,984	0.048272	0.280	0.197				*TLX1*	TSS200
cg00151370	6	16,323,285	2.98E-20	0.280	0.273				*ATXN1*	Body
cg13458609	9	130,608,923	1.35E-10	0.280	0.300				*ENG*	Body
cg13702629	15	66,546,648	1.05E-05	0.280	0.256				*MEGF11*	TSS1500
cg27646729	12	3,373,764	2.08E-17	0.280	0.251				*TSPAN9*	Body
cg03543593	6	32,076,417	1.01E-05	0.280	0.323	2.4E-15	0.199	Yes	*TNXB*	5'UTR
cg04295144	19	10,407,184	6.47E-07	0.280	0.226				*ICAM5*	Body
cg10149996	X	118,369,686	5.88E-15	0.280	0.248				*PGRMC1*	TSS1500
cg19879075	10	73,846,858	9.82E-07	0.280	0.321				*SPOCK2*	Body
cg12830671	10	73,335,297	1.64E-12	0.279	0.263				*CDH23*	Body
cg23138608	14	91,770,145	2.05E-14	0.279	0.239				*CCDC88C*	Body
cg02132284	17	15,686,473	3.35E-10	0.279	0.245					
cg09088834	20	25,565,460	1.14E-06	0.279	0.171	1.93E-30	0.240		*NINL*	5'UTR
cg17265829	4	126,238,422	0.001314	0.279	0.262				*FAT4*	1stExon
cg11230435	10	30,724,374	8.85E-06	0.279	0.205				*MAP3K8*	5'UTR
cg14823789	17	72,322,612	5.65E-07	0.279	0.248				*KIF19*	Body
cg22044566	2	14,772,312	2.17E-08	0.279	0.222				*FAM84A*	TSS1500

cg00927495	9	96,715,687	8.15E-08	0.279	0.308				*BARX1*	Body
cg22617819	1	44,378,782	3.52E-16	0.279	0.234				*ST3GAL3*	Body
cg12110750	19	31,839,577	0.001333	0.279	0.133				*TSHZ3*	Body
cg11306441	12	54,520,919	0.015777	0.279	0.258				*LOC400043*	Body
cg20738500	15	89,921,156	0.000125	0.279	0.256				*LOC254559*	TSS200
cg26459700	10	12,390,907	0.016633	0.279	0.135				*CAMK1D*	TSS1500
cg05057720	14	38,724,675	7.86E-07	0.279	0.289				*CLEC14A*	1stExon
cg20780998	X	12,996,931	0.000136	0.279	0.300					
cg23167259	10	102,495,509	0.002678	0.279	0.291					
cg09887284	12	54,388,147	0.01097	0.279	0.332					
cg04524477	17	41,363,597	1.49E-16	0.279	0.266				*NBR1*	3'UTR
cg05263113	16	47,921,291	0.030478	0.279	0.187					
cg19426827	14	105,191,118	9.57E-08	0.279	0.275	1.63E-27	0.204		*ADSSL1*	Body
cg01107185	7	138,795,041	7.82E-08	0.279	0.211				*ZC3HAV1*	TSS1500
cg02409878	8	99,960,498	1.27E-06	0.279	0.283				*OSR2*	5'UTR
cg23774988	11	128,419,199	2.55E-06	0.279	0.293				*ETS1*	Body
cg18618206	17	754,296	5.05E-17	0.279	0.266				*NXN*	Body
cg03702533	15	77,830,579	2.24E-07	0.279	0.273					
cg06776703	19	4,328,759	2.67E-06	0.279	0.286				*STAP2*	Body
cg13683990	6	33,161,292	0.000382	0.279	0.244				*COL11A2*	TSS1500
cg02951847	4	41,614,490	5.58E-08	0.279	0.235				*LIMCH1*	Body
cg20264543	3	128,787,213	3.71E-07	0.279	0.269					
cg01286655	11	12,031,099	0.001139	0.279	0.254				*DKK3*	TSS1500
cg02718725	1	95,392,925	0.024548	0.279	0.104	2.11E-17	0.174		*CNN3*	TSS200
cg08509586	11	16,797,315	5.82E-29	0.279	0.257					
cg12892506	3	147,113,918	0.048752	0.279	0.326				*ZIC4*	Body
cg13570101	5	172,661,859	0.001056	0.279	0.184				*NKX2-5*	1stExon
cg12910087	4	175,140,004	1.31E-07	0.279	0.270					
cg23972860	1	7,395,024	9.59E-05	0.279	0.168				*CAMTA1*	Body
cg06649048	6	114,176,811	0.002773	0.279	0.132					
cg04442426	5	37,832,522	1.46E-07	0.279	0.282				*GDNF*	Body
cg19307545	10	35,028,929	0.000377	0.279	0.178				*PARD3*	Body
cg23325963	2	45,160,093	1.68E-06	0.279	0.262					
cg00842231	19	11,352,474	6.58E-75	0.279	0.269				*LOC55908*	3'UTR

cg14189049	15	89,909,748	1.96E-05	0.279	0.259				*MIR9-3*	TSS1500
cg19708901	1	180,882,655	3.49E-20	0.279	0.268				*KIAA1614*	Body
cg11213574	22	50,623,692	4.36E-05	0.279	0.288				*TRABD*	TSS1500
cg16713262	16	79,623,751	0.003743	0.279	0.259					
cg02430645	13	20,989,325	1.81E-15	0.279	0.248				*CRYL1*	Body
cg04191989	7	630,914	2.7E-09	0.279	0.245				*PRKAR1B*	Body
cg14957186	10	72,977,773	7.92E-11	0.279	0.262				*UNC5B*	Body
cg21739530	4	174,186,068	3.17E-07	0.279	0.321				*GALNT7*	Body
cg16711011	2	568,198	2.47E-09	0.279	0.220					
cg25012739	2	132,058,031	9.39E-09	0.279	0.272					
cg06333719	11	100,557,736	5.52E-07	0.279	0.253				*FLJ32810*	TSS1500
cg21183256	8	73,163,868	0.016668	0.279	0.315					
cg21333674	8	96,705,765	0.000313	0.279	0.227					
cg06976096	3	87,138,510	0.000414	0.279	0.169					
cg07355157	10	23,385,780	2.9E-10	0.279	0.248				*MSRB2*	Body
cg26559804	11	128,391,271	6.19E-06	0.279	0.268				*ETS1*	Body
cg26961808	7	128,470,913	2.73E-05	0.279	0.289				*FLNC*	1stExon
cg11116274	10	134,334,283	3.75E-16	0.279	0.281					
cg18856581	14	48,145,000	0.009811	0.279	0.342				*MDGA2*	TSS1500
cg05143530	3	197,439,242	3.56E-07	0.279	0.270				*KIAA0226*	Body
cg11225090	4	185,276,573	6.15E-05	0.279	0.232					
cg16946997	X	51,076,330	0.037089	0.279	0.129				*NUDT10*	Body
cg07950002	9	131,465,811	6.87E-08	0.279	0.286				*PKN3*	Body
cg02575448	5	140,749,735	0.004079	0.279	0.326				*PCDHGA4*	Body
cg13995427	9	117,134,861	0.000267	0.279	0.288				*AKNA*	Body
cg02938601	8	22,961,088	0.013187	0.279	0.233				*TNFRSF10C*	Body
cg23632985	11	20,184,637	0.011687	0.279	0.286					
cg26525342	16	3,221,466	1.14E-09	0.279	0.273					
cg26946821	17	35,292,390	0.001571	0.279	0.229					
cg04164415	18	21,199,797	0.000903	0.279	0.292				*ANKRD29*	Body
cg20231084	11	130,700,487	6.91E-18	0.279	0.279					
cg23947376	10	27,548,601	5E-11	0.279	0.282					
cg25358289	5	140,012,728	0.011065	0.279	0.331	5.49E-11	0.123		*CD14*	1stExon
cg00577560	1	15,271,830	1.28E-10	0.279	0.251				*KIAA1026*	Body

cg26883974	13	20,768,523	1.36E-05	0.279	0.291				*GJB2*	TSS1500
cg01343424	10	31,073,383	0.003402	0.279	0.141					
cg02082083	1	20,396,560	5.54E-21	0.279	0.276				*PLA2G5*	TSS200
cg01900413	11	128,419,356	1.56E-09	0.279	0.259				*ETS1*	Body
cg06668065	5	92,909,069	7.65E-05	0.279	0.212				*FLJ42709*	Body
cg19822518	3	71,834,840	7.58E-05	0.279	0.295				*PROK2*	TSS1500
cg23688817	7	87,105,293	5.79E-05	0.279	0.238				*ABCB4*	TSS1500
cg26797073	2	131,129,811	0.002154	0.279	0.224				*PTPN18*	Body
cg13643914	4	122,302,226	0.035338	0.279	0.256				*QRFPR*	TSS200
cg11293610	21	34,755,681	3.42E-08	0.279	0.297					
cg05608790	7	69,427,388	2.14E-20	0.279	0.289				*AUTS2*	Body
cg15996397	2	100,831,189	9.67E-15	0.279	0.271					
cg10058779	14	23,821,435	8.73E-08	0.279	0.217				*SLC22A17*	5'UTR
cg11940040	17	154,420	0.001267	0.279	0.220				*RPH3AL*	Body
cg27423228	3	43,021,399	4.51E-07	0.279	0.158				*FAM198A*	Body
cg01529637	20	50,721,911	0.001948	0.278	0.317				*ZFP64*	Body
cg13818424	17	70,339,099	2.2E-11	0.278	0.285					
cg07724309	6	6,009,556	0.020418	0.278	0.297					
cg15820062	8	23,584,404	0.001439	0.278	0.175					
cg02456451	7	156,871,073	4.55E-07	0.278	0.264					
cg14269807	14	22,191,888	6.14E-13	0.278	0.254					
cg15426660	7	140,347,007	1.7E-12	0.278	0.262					
cg01658421	2	176,945,899	6.01E-06	0.278	0.295				*EVX2*	Body
cg03949284	6	33,161,333	0.007426	0.278	0.256				*COL11A2*	TSS1500
cg12254517	12	93,454,991	1.28E-07	0.278	0.291					
cg23551494	17	39,495,535	2.32E-07	0.278	0.252					
cg12143439	5	140,012,660	0.011144	0.278	0.318				*CD14*	1stExon
cg02332982	12	45,444,420	0.000377	0.278	0.299				*DBX2*	1stExon
cg18375441	4	123,749,187	0.002678	0.278	0.127				*FGF2*	Body
cg18444673	19	36,389,500	4.33E-13	0.278	0.224				*NFKBID*	5'UTR
cg18974921	11	78,131,895	4.36E-10	0.278	0.254					
cg01597398	17	40,250,727	2.2E-22	0.278	0.267					
cg24405179	1	226,411,259	0.002828	0.278	0.350				*MIXL1*	TSS200
cg13052034	1	66,999,238	0.039357	0.278	0.300				*SGIP1*	TSS1500

cg23170347	3	64,253,795	1.55E-14	0.278	0.279					
cg15135286	2	33,359,281	1.97E-07	0.278	0.238				*LTBP1*	Body
cg15361590	5	140,864,701	6.31E-08	0.278	0.262	3.71E-15	0.198		*PCDHGA4*	Body
cg12691382	11	33,037,792	0.012225	0.278	0.392				*DEPDC7*	5'UTR
cg22109795	1	210,468,650	2.6E-06	0.278	0.288					
cg21707187	12	54,345,862	0.026484	0.278	0.286					
cg05793013	16	54,546,975	2.9E-108	0.278	0.268					
cg03260566	4	20,256,841	9.57E-08	0.278	0.291				*SLIT2*	Body
cg16644023	7	27,154,845	2.53E-11	0.278	0.245				*HOXA3*	5'UTR
cg17042098	1	61,519,631	1E-05	0.278	0.265					
cg03212133	X	135,057,092	5.65E-05	0.278	0.252				*MMGT1*	TSS1500
cg17922359	4	13,530,923	0.001816	0.278	0.289					
cg18073240	15	40,731,970	4.08E-20	0.278	0.267				*BAHD1*	TSS1500
cg26392367	17	17,566,891	1.72E-11	0.278	0.275					
cg23901852	4	126,238,384	0.001165	0.278	0.290				*FAT4*	1stExon
cg07459252	14	37,130,212	0.000143	0.278	0.287				*PAX9*	5'UTR
cg14976276	6	31,830,630	0.019159	0.278	0.241	4.46E-15	0.236		*NEU1*	5'UTR
cg03679755	10	101,875,035	0.002338	0.278	0.302					
cg10582860	2	55,347,991	5.74E-15	0.278	0.274					
cg11680055	1	110,230,252	1.2E-05	0.278	0.282				*GSTM1*	TSS200
cg18927077	14	103,605,358	0.000111	0.278	0.227					
cg25426302	6	32,120,826	0.000713	0.278	0.289	3.65E-22	0.246		*PPT2*	TSS1500
cg09030672	16	67,197,150	3.94E-05	0.278	0.326				*HSF4*	TSS200
cg16142306	4	41,258,935	1.52E-06	0.278	0.262				*UCHL1*	1stExon
cg18311871	7	157,754,600	2.11E-07	0.278	0.214				*PTPRN2*	Body
cg07145284	11	66,084,631	8.94E-05	0.278	0.229				*CD248*	TSS200
cg11367159	12	110,044,531	0.010046	0.278	0.284					
cg24942922	3	120,003,547	2.21E-07	0.278	0.232					
cg26544072	6	29,795,992	0.021235	0.278	0.246				*HLA-G*	Body
cg04396791	11	70,508,180	2.88E-09	0.278	0.276	1.76E-18	0.244		*SHANK2*	Body
cg13762691	22	17,956,560	2.57E-12	0.278	0.239				*CECR2*	TSS200
cg20432671	1	167,682,922	3.15E-07	0.278	0.258					
cg14242557	9	114,288,341	2.06E-05	0.278	0.186			Yes	*ZNF483*	5'UTR
cg06783737	6	26,752,689	0.001387	0.278	0.245					

cg18154567	15	51,464,013	8.15E-20	0.278	0.261					
cg22930859	14	78,108,189	4.33E-07	0.278	0.301					
cg00765686	X	17,545,806	3.06E-05	0.278	0.178				*NHS*	Body
cg03669282	1	228,566,718	0.01778	0.278	0.316					
cg18825119	6	163,149,453	3.25E-13	0.278	0.232				*PACRG*	Body
cg04562138	1	53,966,412	1.71E-07	0.278	0.174					
cg16565031	14	70,186,289	5.49E-16	0.278	0.256					
cg02447879	17	79,393,682	0.000289	0.278	0.113				*BAHCC1*	Body
cg05890377	2	74,357,713	0.001476	0.278	0.315					
cg01903654	5	139,225,955	1.01E-12	0.278	0.288					
cg27582007	1	167,682,788	5.61E-07	0.278	0.256					
cg00541718	7	630,823	6.36E-10	0.278	0.252				*PRKAR1B*	Body
cg02125316	5	170,878,209	3.28E-34	0.278	0.276				*FGF18*	Body
cg02629106	2	120,302,419	0.001071	0.278	0.291				*PCDP1*	5'UTR
cg05027533	2	153,290,479	1.19E-10	0.278	0.285				*FMNL2*	Body
cg01382864	2	220,174,251	7.79E-06	0.278	0.308				*PTPRN*	TSS200
cg02643782	3	14,853,225	1.45E-16	0.278	0.262					
cg06993307	16	54,969,051	0.000966	0.278	0.347					
cg08602008	19	48,076,841	3.92E-08	0.278	0.279					
cg25830182	4	85,417,077	0.017973	0.278	0.344				*NKX6-1*	Body
cg03883228	11	68,778,570	8.35E-06	0.278	0.234				*MRGPRF*	5'UTR
cg18016826	5	176,560,001	1.24E-05	0.278	0.250				*NSD1*	TSS200
cg26155697	5	52,150,386	7.29E-15	0.278	0.288				*ITGA1*	Body
cg24714905	13	100,637,241	0.000407	0.278	0.227				*ZIC2*	Body
cg01739354	6	170,481,630	0.00014	0.278	0.212					
cg25610702	6	43,276,744	0.011043	0.278	0.234				*CRIP3*	TSS1500
cg13972124	9	132,539,744	2.46E-05	0.278	0.283					
cg11078221	12	115,135,934	1.76E-08	0.278	0.261					
cg11551216	12	115,125,619	8.78E-05	0.278	0.283					
cg06015781	11	119,613,693	3.39E-07	0.278	0.298					
cg14207728	1	67,217,886	0.003497	0.278	0.265				*TCTEX1D1*	TSS1500
cg06468454	17	3,591,377	9.52E-08	0.278	0.256				*P2RX5*	Body
cg26279840	X	153,770,418	2.64E-09	0.278	0.251				*IKBKG*	TSS200
cg07541701	12	54,389,934	1.21E-07	0.278	0.206					

cg25503903	3	138,679,512	0.00156	0.278	0.284					
cg03989273	5	133,468,435	0.007025	0.278	0.240				*TCF7*	Body
cg05797224	1	94,277,532	1.2E-13	0.278	0.285					
cg16071681	18	19,925,575	1.18E-06	0.278	0.232					
cg21237418	17	27,045,043	1.77E-06	0.278	0.252	7.41E-27	0.205		*RAB34*	TSS200
cg14276286	11	111,782,364	2.15E-11	0.278	0.303				*HSPB2*	TSS1500
cg24336398	9	139,236,777	2.73E-13	0.278	0.274				*GPSM1*	Body
cg24452128	6	10,390,919	0.000867	0.278	0.317					
cg02700891	3	129,693,586	0.00375	0.278	0.339				*TRH*	5'UTR
cg26149244	17	80,329,964	0.026486	0.278	0.353					
cg14586373	22	37,447,398	3.2E-07	0.278	0.200				*KCTD17*	TSS1500
cg24030587	8	122,653,639	0.000416	0.278	0.329				*HAS2*	TSS200
cg03957885	10	89,855,267	2.34E-09	0.278	0.246					
cg26115633	10	129,535,893	9.06E-11	0.278	0.237				*FOXI2*	1stExon
cg04086531	16	49,732,376	9.8E-12	0.278	0.295				*ZNF423*	Body
cg09220326	3	45,175,421	2.28E-14	0.278	0.264			Yes	*CDCP1*	Body
cg11866303	19	40,732,691	0.005128	0.278	0.119				*CNTD2*	TSS200
cg15396367	9	139,972,779	0.005478	0.278	0.168	3.01E-11	0.168		*UAP1L1*	Body
cg00740822	19	49,939,611	0.00326	0.278	0.349				*SLC17A7*	Body
cg10009830	X	20,134,904	0.004638	0.278	0.253	1.39E-17	0.245		*MAP7D2*	TSS200
cg03611487	11	19,737,237	0.000116	0.277	0.290				*LOC100126784*	TSS1500
cg05275576	11	130,523,347	2.8E-08	0.277	0.257					
cg16006349	12	54,360,460	5.54E-12	0.277	0.264				*HOTAIR*	Body
cg05673966	16	85,431,095	3.46E-15	0.277	0.253					
cg06454951	X	15,683,334	3.1E-05	0.277	0.239				*TMEM27*	TSS200
cg05987154	X	3,264,853	0.020248	0.277	0.133				*MXRA5*	TSS200
cg08901098	17	66,255,244	0.000368	0.277	0.278				*ARSG*	TSS200
cg15309006	16	23,766,116	0.000978	0.277	0.325	3.09E-27	0.260		*CHP2*	1stExon
cg24227984	19	4,474,970	2.97E-24	0.277	0.288				*HDGF2*	Body
cg22304612	20	2,781,241	3.48E-05	0.277	0.212				*CPXM1*	5'UTR
cg02093112	17	6,679,495	0.001827	0.277	0.257				*FBXO39*	TSS200
cg17975443	17	59,534,998	4.21E-05	0.277	0.288				*TBX4*	Body
cg09446908	22	19,512,320	0.005473	0.277	0.326				*CLDN5*	1stExon
cg02454595	15	45,406,534	0.001642	0.277	0.263				*DUOXA2*	1stExon

cg15079885	1	32,041,940	7.3E-07	0.277	0.188				*TINAGL1*	TSS200
cg23415212	17	37,331,429	0.007131	0.277	0.373				*CACNB1*	3'UTR
cg27467383	22	38,073,472	0.003384	0.277	0.247				*LGALS1*	Body
cg00018606	2	208,989,425	1.4E-07	0.277	0.261				*CRYGD*	TSS200
cg00016913	17	27,369,892	9.42E-10	0.277	0.250				*PIPOX*	TSS200
cg13461890	9	4,793,992	8.84E-16	0.277	0.249				*RCL1*	Body
cg01481989	2	183,731,169	0.001527	0.277	0.205				*FRZB*	1stExon
cg00683788	20	1,974,878	2.78E-06	0.277	0.286				*PDYN*	TSS200
cg17611647	17	75,260,857	8.57E-19	0.277	0.273					
cg21141329	17	48,290,459	4.83E-08	0.277	0.301					
cg06212637	16	67,184,953	0.045953	0.277	0.135				*B3GNT9*	TSS200
cg16303353	12	2,393,684	3.27E-15	0.277	0.246				*CACNA1C*	Body
cg14688962	17	66,596,275	9.26E-08	0.277	0.248				*FAM20A*	Body
cg21192063	7	150,948,686	0.000533	0.277	0.163				*SMARCD3*	Body
cg01019875	17	6,945,620	0.011917	0.277	0.239				*SLC16A11*	Body
cg23837799	15	31,684,836	0.002261	0.277	0.300					
cg00348762	16	56,228,114	0.000299	0.277	0.206				*DKFZP434H168*	Body
cg15544654	1	197,882,295	0.001391	0.277	0.276				*LHX9*	Body
cg25797887	7	96,627,821	0.000308	0.277	0.319				*DLX6AS*	Body
cg17670496	6	29,759,972	0.001224	0.277	0.306				*HCG4*	Body
cg27017658	14	21,093,081	8.28E-05	0.277	0.266					
cg27207079	9	123,656,751	4.61E-15	0.277	0.269					
cg24631482	2	42,329,446	1.48E-05	0.277	0.278					
cg10715265	1	58,715,499	0.009891	0.277	0.263				*DAB1*	5'UTR
cg01045132	19	11,649,571	8.59E-08	0.277	0.286				*CNN1*	TSS200
cg07235253	7	99,156,031	0.000188	0.277	0.223				*ZNF655*	TSS200
cg18705279	16	67,197,129	9.49E-05	0.277	0.317				*HSF4*	TSS200
cg26407571	12	54,473,534	1.32E-48	0.277	0.254				*FLJ12825*	Body
cg16868298	7	120,969,033	1.78E-05	0.277	0.245				*WNT16*	TSS200
cg01145903	5	115,697,588	2.89E-09	0.277	0.273					
cg05626738	19	53,238,413	0.000789	0.277	0.301				*ZNF611*	TSS200
cg21185686	5	140,810,433	0.000881	0.277	0.308				*PCDHGA4*	Body
cg16374663	15	41,805,031	0.000278	0.277	0.382				*LTK*	Body
cg02639108	2	242,711,009	1.44E-12	0.277	0.278					

cg17757602	5	42,952,113	0.000446	0.277	0.272					
cg04164058	15	89,949,694	7.23E-05	0.277	0.315					
cg13717434	9	97,854,044	1.05E-10	0.277	0.247					
cg12377701	X	75,648,992	0.029762	0.277	0.228				*MAGEE1*	1stExon
cg23786625	10	106,402,741	0.00131	0.277	0.220				*SORCS3*	Body
cg26677394	6	46,258,880	2.51E-11	0.277	0.303				*RCAN2*	Body
cg25958283	18	11,752,089	0.042115	0.277	0.406				*GNAL*	5'UTR
cg02961707	19	7,927,974	3.83E-07	0.277	0.271				*EVI5L*	Body
cg07694025	4	154,709,441	0.002927	0.277	0.271	7.71E-10	0.122		*SFRP2*	Body
cg12999366	7	30,499,283	9.18E-09	0.277	0.292				*NOD1*	5'UTR
cg18724430	15	89,951,787	1.16E-05	0.277	0.230					
cg20325921	8	142,368,332	1.19E-56	0.277	0.278				*GPR20*	5'UTR
cg11918450	2	33,359,198	1.2E-08	0.277	0.230				*LTBP1*	Body
cg23414876	16	67,197,396	2.37E-06	0.277	0.311				*HSF4*	5'UTR
cg05954411	6	28,521,811	0.000128	0.277	0.254					
cg18337222	3	99,595,254	8.71E-13	0.277	0.285				*C3orf26*	Body
cg23542075	8	91,952,780	2.58E-09	0.277	0.285				*NECAB1*	Body
cg03950476	1	53,019,814	3.65E-12	0.277	0.238				*ZCCHC11*	TSS1500
cg07368069	12	107,631,018	6.64E-07	0.277	0.272					
cg21406402	2	121,223,847	6.5E-06	0.277	0.225				*LOC84931*	Body
cg13518992	18	55,109,119	0.007388	0.277	0.299				*ONECUT2*	Body
cg01720742	1	22,351,395	0.037982	0.277	0.281				*HSPC157*	TSS1500
cg27550270	6	117,086,996	5.56E-06	0.277	0.200				*FAM162B*	TSS200
cg03218396	8	96,377,380	2.08E-06	0.277	0.209					
cg03577944	X	106,694,654	9.86E-08	0.277	0.235					
cg03593578	2	45,028,225	1.57E-09	0.277	0.279					
cg08151292	20	3,758,189	0.002729	0.277	0.091				*SPEF1*	3'UTR
cg25629790	1	53,527,412	6.1E-18	0.277	0.249				*PODN*	TSS1500
cg19446165	17	185,756	1.31E-08	0.277	0.256				*RPH3AL*	5'UTR
cg24723331	12	22,487,219	2.91E-05	0.277	0.265	9.04E-20	0.179		*ST8SIA1*	5'UTR
cg14689338	5	16,742,054	3.47E-28	0.277	0.271				*MYO10*	Body
cg21292033	17	75,462,189	3.51E-09	0.277	0.270				*SEPT9*	Body
cg17203063	12	75,601,711	0.005397	0.277	0.265				*KCNC2*	Body
cg26477573	19	15,342,915	0.006631	0.277	0.293				*EPHX3*	1stExon

cg00807464	12	111,618,977	0.016957	0.277	0.150				*CUX2*	Body
cg00866870	7	107,579,250	4.82E-08	0.277	0.285				*LAMB1*	Body
cg01033205	8	107,876,626	0.000563	0.277	0.195					
cg01344273	4	110,223,040	3.97E-14	0.277	0.278				*COL25A1*	Body
cg05193470	8	135,469,552	0.011743	0.277	0.188					
cg04832767	7	96,632,841	0.01063	0.277	0.274				*DLX6AS*	Body
cg08857310	17	75,567,635	0.000117	0.277	0.203					
cg27649239	15	68,120,393	3.5E-05	0.277	0.287				*LBXCOR1*	Body
cg18339788	19	41,641,434	1.04E-05	0.277	0.264					
cg26582733	17	47,547,707	1.69E-05	0.277	0.312					
cg06786219	3	124,499,653	0.002506	0.277	0.182				*ITGB5*	Body
cg12085570	19	8,674,501	0.001725	0.277	0.348				*ADAMTS10*	5'UTR
cg19138538	11	60,623,415	2.36E-15	0.277	0.250				*GPR44*	5'UTR
cg11455444	2	121,223,947	1.83E-09	0.277	0.220				*LOC84931*	TSS200
cg26767632	19	46,526,508	4.52E-15	0.277	0.257				*PGLYRP1*	TSS200
cg04452432	16	57,406,511	2.23E-15	0.276	0.275				*CX3CL1*	1stExon
cg00059374	17	21,477,756	3.5E-07	0.276	0.241					
cg18818866	12	50,221,061	1.67E-20	0.276	0.261				*NCKAP5L*	5'UTR
cg03257341	12	49,375,499	0.023325	0.276	0.369				*WNT1*	3'UTR
cg08036309	4	13,536,991	1.76E-06	0.276	0.339					
cg14468692	11	504,784	0.00056	0.276	0.214				*RNH1*	5'UTR
cg17306747	5	1,446,165	2.87E-06	0.276	0.230				*SLC6A3*	TSS1500
cg11248896	2	177,003,747	1.41E-07	0.276	0.226					
cg04912843	1	78,511,856	0.000272	0.276	0.307				*GIPC2*	1stExon
cg13279774	X	102,632,528	1.04E-13	0.276	0.257				*NGFRAP1*	Body
cg21117734	20	9,489,749	9.7E-09	0.276	0.250					
cg19153763	7	27,196,371	1.5E-08	0.276	0.271				*HOXA7*	TSS200
cg21902772	3	25,468,324	1.56E-13	0.276	0.240				*RARB*	TSS1500
cg20312971	14	79,746,780	0.001319	0.276	0.275				*NRXN3*	Body
cg13481132	9	138,606,811	0.004228	0.276	0.203				*KCNT1*	Body
cg08248285	14	35,182,889	2.73E-06	0.276	0.182				*CFL2*	1stExon
cg26865446	3	181,438,328	2.21E-07	0.276	0.261				*SOX2OT*	Body
cg03352287	1	152,488,262	0.030559	0.276	0.272				*CRCT1*	3'UTR
cg10181330	6	29,818,243	0.00166	0.276	0.367					

cg22955387	X	133,680,468	0.004089	0.276	0.182				*MGC16121*	Body
cg04954111	11	34,363,786	2.4E-11	0.276	0.233				*ABTB2*	Body
cg18063495	5	50,262,796	0.00508	0.276	0.299					
cg18167466	1	6,390,473	2.71E-24	0.276	0.269				*ACOT7*	Body
cg05112555	17	71,739,385	2.19E-05	0.276	0.223					
cg09370982	16	2,547,559	3.32E-07	0.276	0.236				*TBC1D24*	Body
cg19966212	5	149,792,783	0.003484	0.276	0.103				*CD74*	TSS1500
cg03225093	5	2,008,713	4.81E-06	0.276	0.288					
cg19789505	1	228,394,509	6.24E-07	0.276	0.215	2.76E-21	0.128		*OBSCN*	TSS1500
cg20649663	2	197,112,771	6.26E-12	0.276	0.246				*HECW2*	Body
cg05921579	17	61,776,513	1.63E-11	0.276	0.216				*LIMD2*	Body
cg20582655	1	156,390,237	0.000417	0.276	0.296				*MIR9-1*	TSS200
cg03027800	17	7,651,017	4.42E-05	0.276	0.315				*DNAH2*	Body
cg18588323	15	51,385,891	1.78E-08	0.276	0.269				*TNFAIP8L3*	Body
cg05882680	17	48,042,605	0.000229	0.276	0.342					
cg11948905	5	53,920,730	1.53E-06	0.276	0.263			Yes		
cg02710296	1	182,921,851	0.004136	0.276	0.196				*C1orf14*	Body
cg07600533	22	50,986,031	3.11E-05	0.276	0.281				*KLHDC7B*	TSS1500
cg08278741	19	36,736,477	0.002035	0.276	0.254					
cg14613540	18	61,616,369	0.01909	0.276	0.299				*HMSD*	TSS1500
cg08227526	4	155,663,529	0.004665	0.276	0.321					
cg06505273	16	24,850,292	1.17E-06	0.276	0.288					
cg06486093	19	51,875,986	2.92E-05	0.276	0.254				*NKG7*	TSS200
cg17562247	X	119,443,277	0.03199	0.276	0.283				*FAM70A*	Body
cg21233688	5	134,880,353	1.07E-07	0.276	0.274					
cg02032125	14	101,000,186	2.59E-12	0.276	0.262					
cg14983728	22	35,695,234	8.15E-15	0.276	0.231				*TOM1*	TSS1500
cg19654743	17	75,446,592	0.000257	0.276	0.175				*SEPT9*	TSS200
cg24395710	3	58,163,815	0.019815	0.276	0.226					
cg02200207	7	29,605,237	1.43E-14	0.276	0.264				*PRR15*	5'UTR
cg08747277	6	96,048,126	1.19E-16	0.276	0.275				*MANEA*	Body
cg22720790	19	58,716,032	1.76E-16	0.276	0.266				*ZNF274*	Body
cg19456540	14	60,976,285	0.00493	0.276	0.216	6.39E-23	0.238		*SIX6*	1stExon
cg04115680	7	75,889,229	0.026016	0.276	0.281				*SRRM3*	Body

cg14555996	9	86,785,905	1.57E-09	0.276	0.304					
cg02679012	17	773,283	0.000265	0.276	0.263				*NXN*	Body
cg07061260	16	68,676,557	7.62E-07	0.276	0.256					
cg15347342	6	108,490,485	3.08E-09	0.276	0.272				*NR2E1*	Body
cg23224142	8	37,082,537	2.86E-09	0.276	0.279					
cg26253438	1	203,598,623	0.016698	0.276	0.225				*ATP2B4*	5'UTR
cg16175263	8	22,960,385	0.002013	0.276	0.208	2.05E-19	0.193		*TNFRSF10C*	TSS200
cg01404317	13	36,919,819	0.025986	0.276	0.336				*SPG20*	5'UTR
cg06160973	12	57,522,813	0.049998	0.276	0.117				*LRP1*	1stExon
cg22490134	10	22,624,847	0.001145	0.276	0.336					
cg06952416	5	142,781,736	0.011897	0.276	0.205				*NR3C1*	5'UTR
cg15936446	5	42,952,369	1.67E-05	0.276	0.246					
cg06953130	10	11,912,410	1.01E-18	0.276	0.247				*C10orf47*	3'UTR
cg25923450	3	159,944,518	0.000492	0.276	0.145				*LOC401097*	3'UTR
cg05987823	12	54,428,592	1.24E-33	0.276	0.257				*HOXC4*	5'UTR
cg00158528	7	121,956,859	0.00791	0.276	0.358					
cg04053798	12	47,219,705	4.87E-07	0.276	0.235				*SLC38A4*	1stExon
cg01412762	2	29,751,968	4.98E-05	0.276	0.257				*ALK*	Body
cg18781966	19	51,523,159	1.06E-13	0.276	0.271				*KLK10*	5'UTR
cg21237687	17	6,899,380	1.14E-08	0.276	0.204				*ALOX12*	TSS200
cg00210994	3	99,594,893	0.037827	0.276	0.363				*C3orf26*	Body
cg16171281	18	73,167,422	0.049291	0.276	0.074					
cg18320111	18	9,400,501	8.92E-16	0.276	0.297				*TWSG1*	3'UTR
cg09556826	6	28,956,259	6.57E-05	0.276	0.281					
cg01533258	14	95,235,489	0.000616	0.276	0.309				*GSC*	Body
cg13941760	2	198,063,781	2.95E-06	0.276	0.209				*ANKRD44*	TSS1500
cg15883716	1	173,638,701	0.001647	0.276	0.202	2.53E-13	0.141		*ANKRD45*	5'UTR
cg21108215	1	183,791,720	4.98E-19	0.276	0.282				*RGL1*	Body
cg15085603	19	49,250,252	3.91E-06	0.276	0.279				*IZUMO1*	TSS200
cg16426714	17	77,773,042	0.048127	0.276	0.202					
cg12287785	6	99,295,470	1.31E-14	0.276	0.240					
cg13305793	1	210,517,139	1.21E-05	0.276	0.174			Yes	*HHAT*	5'UTR
cg26718213	2	241,976,080	1.81E-11	0.276	0.244				*SNED1*	Body
cg17265419	2	182,321,489	5.19E-11	0.276	0.239				*ITGA4*	TSS200

cg25115537	8	123,801,352	0.003682	0.276	0.171				*ZHX2*	5'UTR
cg00095018	6	36,101,338	8.68E-10	0.276	0.235				*MAPK13*	Body
cg11859607	18	909,154	0.000225	0.276	0.273				*ADCYAP1*	Body
cg21383487	10	22,624,094	5.6E-05	0.276	0.330					
cg26723045	11	9,042,581	1.83E-12	0.276	0.269				*SCUBE2*	3'UTR
cg18283386	8	11,561,728	0.015177	0.276	0.276				*GATA4*	1stExon
cg08498833	8	134,003,896	4.75E-10	0.276	0.282				*TG*	Body
cg13786863	11	68,610,743	1.62E-06	0.276	0.271				*CPT1A*	TSS1500
cg02728483	2	172,965,081	4.54E-05	0.276	0.262				*DLX2*	3'UTR
cg08055087	6	7,107,129	0.002546	0.276	0.184				*RREB1*	TSS1500
cg19056772	17	7,341,616	0.003382	0.276	0.283				*FGF11*	TSS1500
cg08939481	14	21,494,269	0.00011	0.276	0.274				*NDRG2*	TSS1500
cg25249539	20	56,803,358	9.07E-16	0.276	0.282					
cg07551545	19	39,466,313	0.036589	0.276	0.399				*FBXO17*	5'UTR
cg16573374	2	97,761,334	2.36E-05	0.276	0.203				*FAHD2B*	TSS1500
cg18456459	18	9,707,753	4.63E-06	0.276	0.271				*RAB31*	TSS1500
cg09717261	20	35,579,347	0.00732	0.276	0.099				*SAMHD1*	Body
cg20824989	2	208,491,939	1.96E-06	0.276	0.277					
cg14637919	2	227,288,327	0.003626	0.275	0.240					
cg18584067	2	64,975,916	1.41E-07	0.275	0.298					
cg26853640	19	38,879,673	0.000396	0.275	0.224	1.22E-25	0.147		*SPRED3*	TSS1500
cg10137231	14	24,641,021	2.84E-14	0.275	0.281				*REC8*	TSS1500
cg18205770	5	87,439,353	0.001333	0.275	0.272					
cg23058405	3	105,072,537	2.51E-13	0.275	0.272					
cg12176783	20	62,694,000	4.71E-12	0.275	0.257				*TCEA2*	TSS200
cg19645639	1	113,500,384	0.000473	0.275	0.169				*SLC16A1*	TSS1500
cg21497990	7	155,255,122	0.000624	0.275	0.281				*EN2*	Body
cg16291276	1	208,084,071	0.000543	0.275	0.246				*CD34*	Body
cg09182533	11	63,775,071	0.045377	0.275	0.246				*MACROD1*	Body
cg22660370	8	64,132,778	1.86E-05	0.275	0.240					
cg25281029	12	88,969,462	1.68E-06	0.275	0.311				*KITLG*	Body
cg00107488	22	19,930,437	0.015072	0.275	0.202				*COMT*	5'UTR
cg14590331	9	138,782,572	2.64E-14	0.275	0.235				*CAMSAP1*	Body
cg02501410	3	143,255,785	4.72E-15	0.275	0.239				*SLC9A9*	Body

cg08081805	19	49,242,953	0.011783	0.275	0.275				*RASIP1*	Body
cg13054419	13	31,438,929	1.77E-09	0.275	0.298					
cg04995717	9	27,109,293	7.34E-17	0.275	0.267	3.46E-15	0.136		*TEK*	5'UTR
cg11832404	12	65,218,258	0.015356	0.275	0.060					
cg07068761	14	77,505,182	4.48E-15	0.275	0.273					
cg24313303	1	27,113,219	3.49E-07	0.275	0.265				*PIGV*	TSS1500
cg01264106	22	38,071,602	1.86E-06	0.275	0.305				*LGALS1*	TSS200
cg01809281	2	24,162,256	4.74E-17	0.275	0.279				*UBXN2A*	TSS1500
cg06307153	X	134,429,853	4.36E-07	0.275	0.267				*ZNF75D*	5'UTR
cg20705321	19	10,958,970	1.86E-11	0.275	0.262				*C19orf38*	TSS200
cg00399175	2	26,395,448	0.002264	0.275	0.168				*FAM59B*	TSS1500
cg07329360	11	31,841,768	2.85E-05	0.275	0.283					
cg08070022	3	101,497,478	0.000178	0.275	0.189				*FAM55C*	TSS1500
cg20216139	11	16,632,509	0.000149	0.275	0.259					
cg21358336	17	6,558,440	2.36E-05	0.275	0.275					
cg07185131	2	62,798,365	0.006656	0.275	0.272					
cg04100595	12	27,615,138	3.17E-05	0.275	0.305					
cg20152309	10	36,054,255	0.004547	0.275	0.310					
cg11833001	3	14,852,756	0.008948	0.275	0.313					
cg08924554	4	57,976,775	0.013049	0.275	0.314				*IGFBP7*	TSS1500
cg21883598	15	45,404,157	0.000621	0.275	0.315				*DUOX2*	Body
cg00927554	12	95,941,920	5.29E-05	0.275	0.228				*USP44*	5'UTR
cg03367387	3	197,439,334	2.18E-07	0.275	0.239				*KIAA0226*	Body
cg25316749	11	111,169,284	1.56E-14	0.275	0.292				*C11orf93*	TSS1500
cg02599464	6	27,107,097	8.94E-06	0.275	0.256	6.09E-20	0.189		*HIST1H2BK*	3'UTR
cg03142586	19	58,220,080	0.008386	0.275	0.186				*ZNF154*	Body
cg09375033	3	137,833,634	1.17E-13	0.275	0.221				*DZIP1L*	5'UTR
cg16448058	10	102,880,841	0.018644	0.275	0.322				*TLX1NB*	5'UTR
cg05158538	4	23,891,486	0.004836	0.275	0.307	2.01E-07	0.144		*PPARGC1A*	Body
cg23771603	10	26,223,310	0.007728	0.275	0.329	1.84E-19	0.265		*MYO3A*	5'UTR
cg03724964	19	39,465,969	0.007146	0.275	0.301				*FBXO17*	5'UTR
cg04246763	1	87,617,811	0.01363	0.275	0.272				*LOC339524*	Body
cg02159731	11	119,613,405	8.78E-05	0.275	0.244					
cg09227563	19	54,984,705	0.00033	0.275	0.257	4.1E-22	0.197		*CDC42EP5*	TSS1500

cg24105287	2	66,809,985	1.86E-07	0.275	0.260					
cg13211683	8	97,171,827	0.024778	0.275	0.391				*GDF6*	Body
cg06710648	1	58,716,377	3.09E-11	0.275	0.228	2.94E-16	0.192		*DAB1*	TSS200
cg01454215	2	27,530,363	0.003671	0.275	0.304				*UCN*	3'UTR
cg01415937	1	10,134,620	1.91E-08	0.275	0.233				*UBE4B*	Body
cg14375937	10	94,825,667	0.000447	0.275	0.158				*CYP26C1*	Body
cg00653790	15	78,031,418	0.000218	0.275	0.282					
cg08773462	16	17,107,610	6.76E-05	0.275	0.261					
cg12640469	1	53,067,717	5.98E-08	0.275	0.266				*GPX7*	TSS1500
cg18767278	5	1,119,025	1.25E-17	0.275	0.264					
cg16489259	10	32,438,418	1.55E-05	0.275	0.288					
cg10048349	11	111,783,267	0.000007	0.275	0.334				*CRYAB*	TSS1500
cg12467264	5	175,200,071	0.001048	0.275	0.226					
cg18721420	19	15,121,913	8.75E-05	0.275	0.217				*CCDC105*	1stExon
cg17991823	18	12,254,473	0.021877	0.275	0.239				*CIDEA*	Body
cg22810423	1	200,272,180	3.06E-08	0.275	0.263					
cg25032094	17	73,583,839	0.000116	0.275	0.267				*MYO15B*	TSS1500
cg27132335	8	74,002,760	0.000187	0.275	0.203				*C8orf84*	Body
cg02094252	10	102,807,849	1.72E-05	0.275	0.258					
cg08508431	17	13,319,838	3.48E-05	0.275	0.252					
cg08900404	7	29,605,808	8.22E-12	0.275	0.266				*PRR15*	5'UTR
cg26377000	17	33,814,891	6.14E-06	0.275	0.240				*SLFN12L*	Body
cg15938050	17	68,984,117	3.09E-05	0.275	0.261					
cg03694713	8	23,564,156	0.002706	0.275	0.308				*NKX2-6*	TSS1500
cg05376617	2	237,145,141	0.001053	0.275	0.234				*ASB18*	Body
cg09354380	6	106,258,324	2.71E-10	0.275	0.290					
cg14538537	12	6,939,048	6.74E-14	0.275	0.254				*LEPREL2*	Body
cg18235734	1	91,301,731	0.000116	0.275	0.298					
cg20312077	8	327,768	6.04E-11	0.275	0.242					
cg16842053	7	3,083,333	0.025421	0.275	0.407				*CARD11*	1stExon
cg19942495	17	32,484,027	4.96E-07	0.275	0.287				*ACCN1*	TSS1500
cg03231735	16	9,184,099	1.04E-06	0.275	0.214				*C16orf72*	TSS1500
cg04024827	12	54,454,163	4.76E-08	0.275	0.218				*FLJ12825*	Body
cg07589393	7	26,984,305	7.96E-16	0.275	0.284					

cg18327056	2	26,915,355	0.00506	0.275	0.164				*KCNK3*	TSS1500
cg05185926	5	142,602,110	2.16E-12	0.275	0.244				*ARHGAP26*	3'UTR
cg13833923	14	79,746,627	0.000126	0.275	0.240				*NRXN3*	Body
cg09983897	16	31,483,285	0.029455	0.275	0.114				*TGFB1I1*	TSS1500
cg01606023	12	57,613,744	1.56E-14	0.275	0.256				*NXPH4*	Body
cg02634222	1	41,944,775	4.63E-09	0.275	0.241				*EDN2*	3'UTR
cg23514936	7	20,838,523	0.001709	0.275	0.223					
cg14409810	9	140,172,908	1.27E-06	0.275	0.248				*C9orf167*	5'UTR
cg04311496	1	67,773,719	0.001412	0.275	0.140				*IL12RB2*	5'UTR
cg03169508	6	28,557,901	1.33E-06	0.275	0.265					
cg07124687	6	28,956,332	5.01E-05	0.275	0.273					
cg03207151	12	54,442,288	9.82E-07	0.275	0.201				*HOXC4*	5'UTR
cg20072171	3	62,356,962	0.036929	0.275	0.230				*FEZF2*	Body
cg24306982	1	119,549,312	2.53E-05	0.275	0.335					
cg24810735	8	108,441,547	3.84E-08	0.275	0.292				*ANGPT1*	Body
cg14014796	2	45,029,977	0.037168	0.275	0.220					
cg23440058	3	124,103,696	1.57E-09	0.275	0.264				*KALRN*	Body
cg20328588	11	117,747,934	1.03E-12	0.275	0.205				*FXYD6*	5'UTR
cg24947764	4	41,881,359	0.002989	0.275	0.278					
cg15000966	17	73,128,123	0.00024	0.275	0.258				*NT5C*	TSS1500
cg02918577	15	93,633,145	2.33E-07	0.275	0.265				*RGMA*	TSS1500
cg09831105	12	99,287,469	7.72E-07	0.275	0.238				*ANKS1B*	Body
cg07693238	7	99,595,437	0.002898	0.275	0.292					
cg25113157	12	111,537,104	1.01E-10	0.275	0.260				*CUX2*	Body
cg01563704	6	26,271,816	0.026437	0.274	0.304				*HIST1H3G*	TSS1500
cg06528645	1	119,534,088	0.000264	0.274	0.346					
cg17214455	6	32,977,138	3.6E-17	0.274	0.272				*HLA-DOA*	Body
cg04020309	1	247,496,094	0.017203	0.274	0.148				*ZNF496*	TSS1500
cg20979921	22	37,584,018	0.023857	0.274	0.172				*C1QTNF6*	Body
cg06745511	4	10,585,774	2.24E-06	0.274	0.250				*CLNK*	Body
cg15135047	3	72,149,514	0.004016	0.274	0.347					
cg23767994	7	96,621,323	5.3E-09	0.274	0.280				*DLX6AS*	Body
cg04453471	10	30,726,531	0.000171	0.274	0.210				*MAP3K8*	5'UTR
cg14631386	8	93,114,305	0.000116	0.274	0.321					

cg02010481	7	28,218,524	1.75E-05	0.274	0.286				*JAZF1*	Body
cg03811629	5	139,176,077	1.17E-09	0.274	0.254				*PSD2*	5'UTR
cg07197230	22	17,956,641	6.14E-20	0.274	0.246				*CECR2*	1stExon
cg07884255	7	33,804,562	9.12E-07	0.274	0.304					
cg15339231	7	96,650,407	2.39E-06	0.274	0.269				*DLX5*	Body
cg14970515	17	77,816,034	7.32E-10	0.274	0.253					
cg08538034	1	14,928,945	1.27E-09	0.274	0.281				*KIAA1026*	Body
cg27204474	X	130,191,834	1.17E-05	0.274	0.220				*FLJ30058*	TSS1500
cg00951972	3	186,490,777	9.19E-06	0.274	0.263					
cg08572565	14	51,298,737	3.02E-06	0.274	0.280				*NIN*	TSS1500
cg18771553	19	6,710,827	8.98E-05	0.274	0.337				*C3*	Body
cg26329715	20	61,583,987	1.32E-05	0.274	0.297				*SLC17A9*	TSS200
cg06481122	15	37,403,088	0.002675	0.274	0.271					
cg11743528	1	218,339,560	0.0021	0.274	0.320					
cg12502577	12	57,615,581	1.61E-08	0.274	0.269				*NXPH4*	Body
cg08696931	12	123,754,071	0.000653	0.274	0.138				*CDK2AP1*	Body
cg24981058	11	89,224,736	8.53E-06	0.274	0.317				*NOX4*	5'UTR
cg02205739	9	132,427,570	7.91E-05	0.274	0.210	6.54E-26	0.159		*PRRX2*	TSS1500
cg06271652	16	23,144,103	1.72E-09	0.274	0.283				*USP31*	Body
cg10203211	4	185,018,682	0.009551	0.274	0.244				*ENPP6*	Body
cg02556634	4	6,449,000	1.86E-09	0.274	0.273				*PPP2R2C*	Body
cg22925274	14	71,288,210	1.59E-10	0.274	0.235					
cg11660146	11	120,110,688	0.038251	0.274	0.197				*POU2F3*	TSS1500
cg02878244	11	20,181,911	0.013204	0.274	0.315				*DBX1*	TSS200
cg06868100	7	29,606,349	1.1E-09	0.274	0.259				*PRR15*	3'UTR
cg20430047	20	20,257,861	0.000407	0.274	0.199				*C20orf26*	Body
cg06719900	11	109,292,894	0.000275	0.274	0.316				*C11orf87*	5'UTR
cg22762844	2	95,692,424	0.004504	0.274	0.194				*MAL*	Body
cg23090603	17	855,187	7.94E-12	0.274	0.245				*NXN*	Body
cg09798536	6	28,457,348	0.003406	0.274	0.248					
cg19884658	1	6,664,268	7.2E-16	0.274	0.268	2.48E-16	0.206		*KLHL21*	TSS1500
cg05133437	15	40,729,033	0.001941	0.274	0.258					
cg27234090	11	119,251,854	0.008449	0.274	0.118	2.84E-13	0.176		*USP2*	5'UTR
cg10519140	6	44,187,076	0.005268	0.274	0.191	1.2E-15	0.172		*SLC29A1*	TSS200

cg26517376	10	126,428,818	2.48E-12	0.274	0.252				*FAM53B*	5'UTR
cg17862152	1	203,044,930	0.004417	0.274	0.156				*PPFIA4*	Body
cg08545129	7	117,854,304	4.15E-05	0.274	0.230					
cg13519035	12	34,261,006	5.49E-08	0.274	0.236					
cg18592447	3	32,857,857	0.001539	0.274	0.287					
cg27122213	14	57,274,763	0.027745	0.274	0.321				*OTX2*	5'UTR
cg20615910	6	11,607,923	1.41E-20	0.274	0.268					
cg02516419	12	47,771,422	2.17E-11	0.274	0.283					
cg01020696	2	27,958,142	3.1E-05	0.274	0.208					
cg03393769	2	54,611,906	0.000607	0.274	0.245			Yes		
cg14577707	4	184,797,463	2.2E-05	0.274	0.265					
cg13149442	18	43,303,777	1.12E-14	0.274	0.261				*SLC14A1*	TSS1500
cg19776616	7	129,425,330	3.81E-06	0.274	0.283					
cg04357965	5	77,945,347	0.000102	0.274	0.209				*LHFPL2*	TSS1500
cg19697239	12	752,646	3.19E-05	0.274	0.218				*NINJ2*	Body
cg21960624	19	35,085,474	1.28E-10	0.274	0.271				*SCGBL*	1stExon
cg18891712	6	76,059,576	0.044855	0.274	0.183				*FILIP1*	Body
cg13038216	15	33,487,120	0.020276	0.274	0.176					
cg12650635	12	50,344,463	1.38E-18	0.274	0.255	5.03E-14	0.088		*AQP2*	TSS200
cg04059568	7	157,486,012	2.22E-05	0.274	0.302				*PTPRN2*	Body
cg17093485	1	149,294,708	0.007282	0.274	0.403					
cg21051519	5	137,225,191	0.040015	0.274	0.186				*PKD2L2*	1stExon
cg21550483	17	6,984,106	2.32E-05	0.274	0.233	2.37E-18	0.223		*CLEC10A*	TSS1500
cg23999932	4	54,976,184	5.67E-09	0.274	0.275					
cg07144296	1	182,584,520	0.025787	0.274	0.172					
cg13115683	6	29,943,460	3.09E-06	0.274	0.277				*HCG9*	Body
cg12144100	7	157,890,277	4.32E-10	0.274	0.250				*PTPRN2*	Body
cg11676189	2	69,875,470	2.77E-17	0.274	0.259					
cg12374123	15	45,406,327	5.88E-05	0.274	0.267				*DUOX2*	5'UTR
cg13437581	18	44,777,799	0.001761	0.274	0.251					
cg05764238	1	16,536,990	5.08E-10	0.274	0.278				*ARHGEF19*	5'UTR
cg09164580	8	97,157,878	1.13E-05	0.274	0.253				*GDF6*	Body
cg14550519	17	58,180,026	1.37E-05	0.274	0.248				*LOC653653*	Body
cg24484138	20	31,070,190	5.38E-05	0.274	0.203				*C20orf112*	5'UTR

cg02315489	2	24,398,859	4.25E-12	0.274	0.279				*C2orf84*	Body
cg04364728	1	845,810	3.31E-06	0.274	0.262					
cg07350470	4	4,765,329	2.5E-07	0.274	0.251					
cg09240763	2	200,330,851	1.19E-06	0.274	0.272					
cg22172038	1	231,176,991	1.25E-05	0.274	0.214				*FAM89A*	TSS1500
cg18201679	1	149,672,149	5.34E-08	0.274	0.259					
cg04169747	Y	2,655,740	1.35E-05	0.274	0.306				*SRY*	5'UTR
cg11660879	19	13,319,417	2.96E-06	0.274	0.256				*CACNA1A*	Body
cg13729210	5	3,606,561	1.13E-07	0.274	0.276					
cg16670554	15	68,118,985	0.000473	0.274	0.299				*LBXCOR1*	Body
cg12858577	17	75,385,278	7.8E-07	0.274	0.301				*SEPT9*	5'UTR
cg18372930	5	137,939,469	2.89E-12	0.274	0.282					
cg02544037	12	68,759,069	0.000877	0.274	0.205					
cg13735819	19	2,253,850	1.29E-10	0.274	0.270				*JSRP1*	Body
cg25928819	12	133,484,026	1.35E-05	0.274	0.270					
cg12744859	17	46,669,492	5.84E-14	0.274	0.260				*LOC404266*	TSS200
cg16758598	4	106,729,791	1.39E-16	0.274	0.264				*GSTCD*	Body
cg05336698	10	102,497,298	0.02819	0.274	0.240					
cg01804193	12	63,026,212	0.006191	0.274	0.205					
cg08286219	21	38,580,802	2.56E-18	0.274	0.268				*DSCR9*	TSS200
cg00307214	14	69,726,319	0.010566	0.274	0.127				*GALNTL1*	TSS1500
cg20820107	1	12,193,099	0.000371	0.274	0.176				*TNFRSF8*	5'UTR
cg10189135	16	67,516,162	1.7E-18	0.273	0.238				*ATP6V0D1*	TSS1500
cg19448822	6	29,974,773	1.83E-10	0.273	0.262				*HLA-J*	Body
cg27339550	7	6,654,880	0.000873	0.273	0.259				*ZNF853*	TSS1500
cg19270505	1	25,256,705	0.044552	0.273	0.242				*RUNX3*	1stExon
cg01521258	1	64,937,528	0.000123	0.273	0.260				*CACHD1*	Body
cg05817845	20	42,816,226	2.41E-07	0.273	0.235				*JPH2*	TSS200
cg09667289	15	33,165,563	0.043314	0.273	0.167				*FMN1*	Body
cg06726820	3	139,259,742	1.58E-12	0.273	0.246				*RBP1*	TSS1500
cg01808736	5	219,908	2.62E-15	0.273	0.260				*SDHA*	Body
cg12260798	6	26,240,519	0.000838	0.273	0.282				*HIST1H4F*	TSS200
cg23667127	12	1,739,917	1.05E-08	0.273	0.254				*WNT5B*	5'UTR
cg25283465	1	111,323,341	0.000216	0.273	0.291					

cg00324725	16	56,581,212	3.84E-10	0.273	0.250						
cg21962450	18	24,444,196	4.85E-10	0.273	0.269					*AQP4*	Body
cg22274813	21	38,067,101	0.007413	0.273	0.305						
cg03697809	X	106,750,775	3.06E-07	0.273	0.250						
cg26348226	1	21,617,442	4.98E-07	0.273	0.211					*ECE1*	TSS1500
cg15200418	7	4,755,010	2.27E-17	0.273	0.247					*FOXK1*	Body
cg10764101	2	241,374,174	4.95E-07	0.273	0.234					*GPC1*	TSS1500
cg18110483	5	79,330,959	5.32E-06	0.273	0.225	8.05E-08	0.147			*THBS4*	TSS200
cg07576175	19	59,049,843	0.021694	0.273	0.317						
cg00705059	1	244,080,368	9.31E-05	0.273	0.194						
cg12991050	5	87,979,504	0.00061	0.273	0.305					*LOC645323*	Body
cg13493766	9	97,365,869	0.013946	0.273	0.183					*FBP1*	Body
cg17980508	1	79,085,713	0.000694	0.273	0.221	0.00000452	0.102			*IFI44L*	TSS1500
cg03562044	19	15,342,749	0.032796	0.273	0.269					*EPHX3*	Body
cg09086442	6	168,079,212	0.000205	0.273	0.202						
cg26824216	3	187,086,147	0.016344	0.273	0.087					*RTP4*	TSS200
cg03573446	20	55,841,893	0.020685	0.273	0.153					*BMP7*	TSS200
cg15385139	6	29,595,506	6.53E-05	0.273	0.238					*GABBR1*	Body
cg19451167	7	96,655,981	2.9E-15	0.273	0.259						
cg11510586	9	72,027,409	0.04788	0.273	0.335						
cg25681728	2	233,153,284	1.88E-21	0.273	0.263					*DIS3L2*	Body
cg23681866	6	29,895,175	0.000001	0.273	0.244						
cg17632299	13	53,312,966	0.028725	0.273	0.154					*LECT1*	Body
cg26470101	2	173,099,597	0.006355	0.273	0.227						
cg18141622	2	238,525,524	1.2E-07	0.273	0.277						
cg04109923	13	28,495,600	0.00242	0.273	0.237					*PDX1*	Body
cg09468836	19	55,889,611	1.61E-07	0.273	0.251					*TMEM190*	3'UTR
cg22617773	6	29,521,751	0.028351	0.273	0.216						
cg06607764	17	76,719,635	0.000111	0.273	0.323					*CYTH1*	Body
cg07842862	2	73,299,145	0.003954	0.273	0.210					*SFXN5*	TSS200
cg24129589	5	1,008,558	2.78E-05	0.273	0.186					*NKD2*	TSS1500
cg19815596	1	201,583,478	3.43E-07	0.273	0.230						
cg01346501	17	38,254,714	3.21E-05	0.273	0.267					*NR1D1*	Body
cg01965476	15	40,530,997	5.4E-09	0.273	0.225					*PAK6*	5'UTR

cg00914726	1	60,539,400	1.55E-07	0.273	0.243				*C1orf87*	1stExon
cg01876130	2	97,308,580	1.17E-13	0.273	0.255				*FER1L5*	1stExon
cg11548446	2	28,867,073	1.07E-09	0.273	0.271					
cg00947032	13	36,919,960	0.012955	0.273	0.286				*SPG20*	5'UTR
cg27424370	X	23,350,651	0.014134	0.273	0.164					
cg04075781	3	54,154,438	1.46E-10	0.273	0.224					
cg01602621	19	11,689,813	8.79E-05	0.273	0.263				*ACP5*	TSS1500
cg14375499	17	6,899,207	1.84E-08	0.273	0.212				*ALOX12*	TSS200
cg15442678	3	79,065,762	3.34E-07	0.273	0.222				*ROBO1*	Body
cg23397741	17	35,014,588	0.009014	0.273	0.200					
cg01010211	22	17,956,453	1.21E-13	0.273	0.255				*CECR2*	TSS200
cg19836199	21	36,421,941	0.021779	0.273	0.143				*RUNX1*	TSS1500
cg18442286	11	59,333,608	0.001911	0.273	0.269					
cg14805347	15	58,357,879	0.022145	0.273	0.293				*ALDH1A2*	5'UTR
cg11071682	1	210,411,064	0.00178	0.273	0.314				*SERTAD4*	5'UTR
cg27634344	4	188,917,251	1.86E-12	0.273	0.261				*ZFP42*	5'UTR
cg19411146	6	152,128,471	0.030037	0.273	0.143				*ESR1*	TSS1500
cg23497436	19	55,767,121	9.25E-10	0.273	0.239				*SAPS1*	5'UTR
cg05253480	3	9,944,537	1.04E-05	0.273	0.286				*IL17RE*	1stExon
cg26189983	1	12,227,700	2.59E-09	0.273	0.266	4.79E-16	0.164		*TNFRSF1B*	Body
cg23278196	12	54,071,111	0.014248	0.273	0.254				*ATP5G2*	TSS1500
cg06687640	5	159,560,692	1.14E-05	0.273	0.260					
cg13019143	7	96,633,060	1.35E-07	0.273	0.233				*DLX6AS*	Body
cg01498231	10	73,847,259	0.002285	0.273	0.301				*SPOCK2*	Body
cg03445151	2	23,516,881	0.045292	0.273	0.338					
cg13588086	9	6,644,369	0.001637	0.273	0.307				*GLDC*	Body
cg23309670	6	36,756,830	2.95E-05	0.273	0.299				*CPNE5*	Body
cg12184886	17	73,584,029	3.58E-06	0.273	0.256				*MYO15B*	TSS200
cg04295543	1	244,080,447	7.56E-11	0.273	0.234					
cg06069187	17	26,698,283	0.000234	0.273	0.189				*SARM1*	TSS1500
cg25077778	14	51,562,591	0.000264	0.273	0.178				*TRIM9*	TSS200
cg14417784	2	131,009,681	8.92E-06	0.273	0.245					
cg21848191	14	68,607,773	4.32E-12	0.273	0.257				*RAD51L1*	Body
cg09626867	3	45,053,351	2.54E-06	0.273	0.299				*EXOSC7*	Body

cg23363754	7	29,606,082	4.72E-11	0.273	0.262				*PRR15*	Body
cg03760483	17	6,899,297	1.34E-06	0.273	0.194	1.65E-18	0.235		*ALOX12*	TSS200
cg04875041	1	12,514,747	2.99E-11	0.273	0.260				*VPS13D*	Body
cg21011616	2	177,015,992	1.53E-07	0.273	0.239				*HOXD4*	TSS200
cg25951981	4	46,995,743	2.03E-07	0.273	0.313				*GABRA4*	TSS200
cg18786873	1	110,610,899	2.36E-07	0.273	0.289				*ALX3*	Body
cg13604445	9	116,342,046	6.51E-20	0.273	0.251				*RGS3*	Body
cg16657152	8	125,784,769	0.014437	0.273	0.248					
cg08256939	1	112,058,236	5.3E-22	0.273	0.244				*ADORA3*	Body
cg25436127	X	36,976,108	5.25E-09	0.273	0.286					
cg02286091	5	78,407,678	7.71E-10	0.273	0.275				*BHMT*	5'UTR
cg09262171	16	4,140,361	0.000447	0.273	0.201				*ADCY9*	Body
cg17580045	12	4,384,890	0.01391	0.273	0.349	7.87E-27	0.202		*CCND2*	Body
cg03594515	3	129,346,178	0.003643	0.273	0.287					
cg10821050	7	155,603,913	0.014283	0.273	0.170				*SHH*	Body
cg25611756	2	65,086,991	3.72E-06	0.273	0.213					
cg02619661	12	116,036,878	2.18E-05	0.273	0.213					
cg12257156	7	158,823,799	2.24E-06	0.273	0.236				*VIPR2*	Body
cg07419680	X	134,429,972	1.95E-06	0.273	0.266				*ZNF75D*	5'UTR
cg13964726	9	71,062,328	5.69E-11	0.273	0.238				*PGM5*	Body
cg02825977	10	94,825,848	1.86E-05	0.273	0.164				*CYP26C1*	Body
cg20741105	7	43,686,889	3.19E-07	0.273	0.275				*C7orf44*	Body
cg11923296	3	149,374,285	2.37E-21	0.273	0.262			Yes	*WWTR1*	Body
cg00760729	2	161,236,088	2.93E-08	0.273	0.260				*RBMS1*	Body
cg01581084	8	99,961,874	4.1E-08	0.273	0.277				*OSR2*	Body
cg20165694	13	30,077,315	7.55E-06	0.272	0.195				*MTUS2*	Body
cg11505661	12	57,618,965	1.84E-08	0.272	0.293				*NXPH4*	Body
cg13781744	1	43,250,691	1.25E-10	0.272	0.259					
cg00067414	6	151,188,081	0.000835	0.272	0.204				*MTHFD1L*	Body
cg11312495	5	115,697,370	3.91E-11	0.272	0.279					
cg12147622	10	74,021,432	7.05E-16	0.272	0.260					
cg16745616	19	8,428,856	0.002453	0.272	0.168	1.27E-14	0.104		*ANGPTL4*	TSS200
cg02228185	17	3,379,567	2.47E-05	0.272	0.294	6.45E-10	0.087		*ASPA*	1stExon
cg11377730	8	17,270,604	0.001178	0.272	0.268				*MTMR7*	Body

cg09530163	16	55,512,822	3.63E-13	0.272	0.236				*MMP2*	TSS1500
cg09316122	1	228,195,522	0.007666	0.272	0.344				*WNT3A*	Body
cg23807890	2	228,736,357	0.010026	0.272	0.302				*WDR69*	1stExon
cg02824888	12	115,129,011	0.032664	0.272	0.391					
cg20549620	2	220,970,010	6.74E-07	0.272	0.217					
cg24257776	3	47,051,546	1.6E-17	0.272	0.264				*LOC100129354*	TSS1500
cg03231163	19	39,360,465	0.018371	0.272	0.269				*RINL*	Body
cg13769548	19	54,984,708	0.000218	0.272	0.278				*CDC42EP5*	TSS1500
cg20799268	8	142,275,649	0.007588	0.272	0.259					
cg23286646	21	38,120,350	0.000371	0.272	0.217				*SIM2*	Body
cg00397986	8	11,561,593	3.4E-06	0.272	0.238				*GATA4*	TSS200
cg22233974	1	39,546,511	1.52E-14	0.272	0.266	2.26E-23	0.174		*MACF1*	TSS1500
cg26905768	7	34,098,803	1.74E-06	0.272	0.214				*BMPER*	Body
cg07204280	18	12,254,551	0.034608	0.272	0.294				*CIDEA*	Body
cg01975591	X	9,432,530	3.94E-06	0.272	0.266				*TBL1X*	5'UTR
cg24322131	21	45,971,503	0.005129	0.272	0.247				*KRTAP10-2*	TSS200
cg10988628	10	135,160,697	3.33E-08	0.272	0.231				*PRAP1*	TSS200
cg14624546	11	8,800,270	2.95E-21	0.272	0.255				*ST5*	5'UTR
cg00035316	2	176,993,017	3.36E-05	0.272	0.242				*HOXD8*	TSS1500
cg00901982	2	70,257,298	1.73E-05	0.272	0.267					
cg02282774	5	50,264,516	0.006778	0.272	0.298					
cg21325485	7	158,364,932	1.51E-19	0.272	0.269				*PTPRN2*	Body
cg16801374	12	102,872,708	0.00014	0.272	0.236				*IGF1*	Body
cg17479501	17	80,806,191	0.000142	0.272	0.304				*TBCD*	Body
cg02583633	2	175,192,013	1.07E-09	0.272	0.253					
cg24345062	2	200,334,439	0.000367	0.272	0.286				*FLJ32063*	Body
cg18827954	4	5,053,585	0.001058	0.272	0.252				*STK32B*	1stExon
cg25740565	2	200,335,101	5.56E-06	0.272	0.277				*FLJ32063*	Body
cg10192893	4	41,747,895	0.02918	0.272	0.297				*PHOX2B*	Body
cg20034792	6	29,521,781	0.000219	0.272	0.259					
cg05097306	10	45,374,683	8.75E-13	0.272	0.237					
cg13652008	2	33,359,356	0.000197	0.272	0.190				*LTBP1*	Body
cg18096987	3	11,623,873	0.002763	0.272	0.216				*VGLL4*	Body
cg00028211	2	45,028,802	1.5E-07	0.272	0.275					

cg10416963	4	121,844,401	0.000916	0.272	0.145				*PRDM5*	TSS1500
cg11179081	6	27,107,756	8.3E-08	0.272	0.262				*HIST1H2BK*	3'UTR
cg20377305	7	96,650,668	3.47E-10	0.272	0.272				*DLX5*	Body
cg01296889	3	54,122,032	1.93E-08	0.272	0.277					
cg25349034	3	149,265,300	3.67E-08	0.272	0.261				*WWTR1*	Body
cg01551350	1	160,951,675	0.000207	0.272	0.289					
cg20653955	7	6,566,649	3.62E-09	0.272	0.227				*GRID2IP*	Body
cg10966582	1	17,085,860	1.03E-08	0.272	0.263				*MST1P9*	Body
cg03464514	7	137,028,442	0.0034	0.272	0.191				*PTN*	5'UTR
cg01481159	20	21,489,196	0.003721	0.272	0.390					
cg04886703	1	45,250,266	9.38E-05	0.272	0.310				*BEST4*	Body
cg23715700	19	16,188,274	0.002466	0.272	0.285				*TPM4*	Body
cg05942508	11	113,846,922	9.72E-06	0.272	0.208				*HTR3A*	Body
cg20000562	14	36,978,633	0.004953	0.272	0.390				*SFTA3*	Body
cg26784201	5	150,050,950	5.44E-08	0.272	0.258				*MYOZ3*	Body
cg02081701	2	139,537,197	0.045428	0.272	0.327				*NXPH2*	Body
cg23175074	10	105,212,490	9.23E-07	0.272	0.255				*CALHM2*	TSS1500
cg04770088	17	63,225,020	0.016222	0.272	0.330					
cg16389399	11	43,603,991	8.35E-05	0.272	0.265					
cg19917412	11	64,815,189	1.06E-06	0.272	0.249				*NAALADL1*	Body
cg24572745	3	112,932,345	6.56E-05	0.272	0.294				*BOC*	5'UTR
cg27099262	12	20,263,199	3.47E-13	0.272	0.275					
cg00876332	Y	21,664,717	0.001694	0.272	0.251				*BCORL2*	Body
cg02951552	1	156,051,441	0.039719	0.272	0.105				*MEX3A*	1stExon
cg05436658	16	23,847,568	0.000289	0.272	0.191	5.88E-15	0.219		*PRKCB*	1stExon
cg15978561	2	240,196,996	1.87E-07	0.272	0.241				*HDAC4*	Body
cg06907680	14	91,580,129	0.002825	0.272	0.158				*C14orf159*	TSS1500
cg24654177	15	58,791,257	3.53E-29	0.272	0.263				*LIPC*	Body
cg06012347	11	65,066,368	6.44E-12	0.272	0.273					
cg25622366	2	63,281,139	2.4E-05	0.272	0.266				*OTX1*	Body
cg25881804	20	21,081,901	0.0118	0.272	0.251					
cg12462816	6	127,836,107	6.82E-05	0.272	0.244				*C6orf174*	Body
cg15028458	3	58,860,290	4.77E-09	0.272	0.281				*C3orf67*	Body
cg18526008	3	177,076,224	0.007214	0.272	0.364					

cg08547691	17	9,143,754	7.34E-05	0.272	0.235				*NTN1*	3'UTR
cg20973210	19	2,282,729	1.76E-07	0.272	0.258				*C19orf35*	TSS1500
cg00668426	X	102,319,340	0.004854	0.272	0.256				*BEX1*	TSS200
cg05385377	1	15,480,844	5.16E-08	0.272	0.255	8.42E-27	0.182		*TMEM51*	5'UTR
cg12284802	15	79,723,655	7.15E-05	0.272	0.183				*KIAA1024*	TSS1500
cg07813142	2	171,573,223	0.000472	0.272	0.230				*SP5*	Body
cg07042104	20	34,206,951	0.000155	0.272	0.206				*SPAG4*	Body
cg04231042	4	54,729,555	1.46E-17	0.272	0.256					
cg00627029	17	72,322,791	0.00021	0.272	0.295				*KIF19*	Body
cg12014547	11	20,692,092	0.018282	0.272	0.260				*NELL1*	Body
cg10372188	11	117,748,169	2.91E-06	0.272	0.211				*FXYD6*	TSS1500
cg00587922	6	30,651,601	1.82E-09	0.272	0.262				*KIAA1949*	Body
cg14469436	3	127,441,359	4.72E-19	0.272	0.260				*MGLL*	Body
cg24079361	13	44,948,039	0.019047	0.272	0.310				*SERP2*	1stExon
cg21504815	6	27,791,876	0.003761	0.272	0.219				*HIST1H4J*	TSS200
cg16814483	1	146,550,074	8.49E-05	0.272	0.307					
cg01942863	7	99,769,432	0.021957	0.272	0.221				*GPC2*	Body
cg22559034	5	92,910,534	1.79E-06	0.272	0.236				*FLJ42709*	Body
cg15986276	14	101,176,310	0.023891	0.272	0.207					
cg17880014	2	71,693,165	0.002205	0.272	0.335				*DYSF*	TSS1500
cg23217622	1	156,405,438	6.49E-05	0.272	0.268					
cg00495860	21	38,065,524	0.029122	0.272	0.323					
cg09989996	1	753,376	6.74E-05	0.272	0.200					
cg05804047	4	77,752,568	1.46E-10	0.271	0.279					
cg03474202	17	45,855,739	1.23E-06	0.271	0.250					
cg03535793	12	3,862,594	0.000135	0.271	0.236				*EFCAB4B*	TSS1500
cg06352750	2	192,711,926	0.000459	0.271	0.300	2.77E-21	0.185		*SDPR*	5'UTR
cg22904048	4	6,665,672	4.04E-18	0.271	0.279					
cg23732608	20	20,180,469	1.33E-06	0.271	0.211				*C20orf26*	Body
cg03113038	16	85,181,211	2.37E-09	0.271	0.222					
cg23537887	3	54,157,647	0.000415	0.271	0.211				*CACNA2D3*	Body
cg01679516	18	30,352,505	2.73E-05	0.271	0.275				*KLHL14*	5'UTR
cg06459000	11	111,411,199	0.000247	0.271	0.247				*LAYN*	TSS200
cg01400468	2	54,087,469	7.47E-07	0.271	0.241				*GPR75*	TSS1500

cg19718309	14	100,632,904	0.013777	0.271	0.355					
cg01559726	3	54,157,894	0.005994	0.271	0.252				*CACNA2D3*	Body
cg00503866	1	39,546,656	9.82E-13	0.271	0.264				*MACF1*	TSS1500
cg07553115	X	107,976,167	0.013471	0.271	0.350				*IRS4*	1stExon
cg19499884	10	102,760,724	1.53E-05	0.271	0.285				*LZTS2*	5'UTR
cg17291251	10	49,678,358	2.31E-09	0.271	0.265				*ARHGAP22*	Body
cg23010048	19	52,391,257	0.00515	0.271	0.232				*ZNF577*	TSS200
cg25499067	8	23,559,988	6.76E-09	0.271	0.266				*NKX2-6*	Body
cg05904716	5	179,779,205	4.86E-06	0.271	0.261				*GFPT2*	Body
cg01824919	1	116,711,339	4.74E-06	0.271	0.225					
cg09844474	6	33,161,276	0.000631	0.271	0.229				*COL11A2*	TSS1500
cg15384383	10	99,798,415	2.3E-06	0.271	0.265					
cg15661311	11	504,551	9.46E-05	0.271	0.218				*RNH1*	5'UTR
cg12737152	11	129,565,909	3.66E-05	0.271	0.269					
cg15545035	12	28,128,288	0.001358	0.271	0.208					
cg00249032	20	43,975,752	0.029576	0.271	0.137				*SDC4*	Body
cg01637175	17	21,281,507	1.77E-29	0.271	0.279				*KCNJ12*	5'UTR
cg21824733	15	42,197,055	6.08E-13	0.271	0.257				*EHD4*	Body
cg23245007	3	195,682,326	8.19E-09	0.271	0.238					
cg02303016	7	130,020,130	7.12E-07	0.271	0.323				*CPA1*	TSS200
cg11594833	12	54,444,985	2.01E-06	0.271	0.205				*HOXC4*	5'UTR
cg16661209	13	29,107,069	0.001219	0.271	0.312					
cg05368966	12	14,392,531	4.22E-09	0.271	0.246					
cg17131553	8	116,676,938	5.36E-07	0.271	0.259				*TRPS1*	5'UTR
cg23697202	6	91,320,190	0.00048	0.271	0.194					
cg05485241	6	137,311,162	0.003735	0.271	0.179				*NHEG1*	Body
cg01646109	4	183,064,633	0.014993	0.271	0.339				*MGC45800*	Body
cg26239725	21	46,824,732	0.001146	0.271	0.181				*COL18A1*	TSS1500
cg02115117	2	24,398,668	2.47E-08	0.271	0.244				*C2orf84*	Body
cg20628994	10	118,429,852	0.011675	0.271	0.271				*C10orf82*	TSS1500
cg06819935	16	50,003,431	7.74E-09	0.271	0.222					
cg03536474	12	54,367,460	0.010402	0.271	0.276				*HOXC11*	1stExon
cg17807663	17	46,622,012	0.001444	0.271	0.256				*HOXB2*	1stExon
cg23776012	17	38,821,403	0.008901	0.271	0.260	0.00000535	0.112		*KRT222*	5'UTR

cg01205087	2	47,008,202	8.09E-21	0.271	0.240					
cg25895948	15	85,360,655	7.5E-09	0.271	0.254				*ALPK3*	1stExon
cg01508380	14	23,305,585	0.001104	0.271	0.300				*MMP14*	TSS1500
cg12014818	2	219,744,634	1.34E-11	0.271	0.269				*WNT10A*	TSS1500
cg02337873	11	65,659,393	1.01E-12	0.271	0.272					
cg17261120	11	12,578,707	2.15E-12	0.271	0.267					
cg09082287	1	65,730,494	0.000007	0.271	0.238	2.92E-30	0.255		*DNAJC6*	5'UTR
cg26671281	11	2,828,214	1.34E-23	0.271	0.268				*KCNQ1*	Body
cg27100022	11	107,800,031	0.000292	0.271	0.181				*RAB39*	Body
cg00422474	8	67,261,068	0.001989	0.271	0.235					
cg15105703	15	45,421,860	0.000474	0.271	0.252	4.12E-14	0.171		*DUOX1*	TSS1500
cg06941230	16	11,484,506	7.08E-17	0.271	0.269					
cg06255004	9	139,580,799	1.32E-08	0.271	0.239				*AGPAT2*	Body
cg11174855	10	134,598,352	9.44E-06	0.271	0.245				*NKX6-2*	3'UTR
cg14669514	13	22,243,722	0.000448	0.271	0.113				*FGF9*	TSS1500
cg08684879	12	5,369,360	4.19E-07	0.271	0.244					
cg10164640	1	45,792,557	0.001126	0.271	0.254	2.81E-24	0.192		*HPDL*	1stExon
cg04557883	6	3,753,083	1.6E-05	0.271	0.253				*C6orf145*	TSS1500
cg23896816	22	39,473,253	2.18E-08	0.271	0.257				*APOBEC3G*	5'UTR
cg13917273	9	132,099,943	7.7E-19	0.271	0.247					
cg03224572	8	97,172,012	0.012835	0.271	0.326				*GDF6*	Body
cg05157171	6	29,910,411	1.37E-07	0.271	0.268				*HLA-A*	Body
cg22821981	3	122,296,435	3.26E-11	0.271	0.263				*PARP15*	TSS200
cg00334177	6	28,511,757	5.58E-06	0.271	0.277					
cg01799671	16	81,560,752	7.36E-14	0.271	0.263				*CMIP*	Body
cg13033898	6	151,561,055	2.7E-07	0.271	0.246				*AKAP12*	TSS200
cg23688719	1	65,935,654	1.24E-16	0.271	0.259				*LEPR*	5'UTR
cg01229787	15	100,048,371	7.05E-08	0.271	0.260					
cg00374672	7	100,463,416	1.1E-08	0.271	0.233				*SLC12A9*	Body
cg10611580	17	75,319,942	4.36E-11	0.271	0.215				*SEPT9*	5'UTR
cg24474193	4	108,662,252	1.37E-14	0.271	0.242					
cg10333808	12	22,487,459	0.008052	0.271	0.280				*ST8SIA1*	5'UTR
cg02648057	2	102,867,466	0.001502	0.271	0.248					
cg22473333	5	54,287,462	9.5E-06	0.271	0.290					

cg19629053	3	44,626,193	6.19E-05	0.271	0.162				*ZNF660*	TSS1500
cg10461547	3	13,323,567	8.88E-06	0.271	0.309					
cg18272889	17	66,197,318	1.27E-07	0.271	0.198					
cg24756378	14	33,401,638	2.21E-20	0.271	0.245					
cg19210770	2	220,379,091	0.028339	0.271	0.276	4.92E-09	0.141		*ACCN4*	1stExon
cg00345443	3	54,121,778	8.75E-10	0.271	0.281					
cg10806711	1	113,265,679	1.65E-19	0.271	0.263				*FAM19A3*	Body
cg18349911	5	1,877,135	2.22E-07	0.271	0.267					
cg24427724	12	6,234,257	3.22E-12	0.271	0.277				*VWF*	TSS1500
cg07605620	19	21,512,141	0.001411	0.271	0.172				*ZNF708*	1stExon
cg10512292	12	4,378,267	9.73E-05	0.271	0.264					
cg01681847	11	43,605,269	0.00018	0.271	0.261					
cg04637478	5	140,753,655	0.003567	0.271	0.352				*PCDHGA4*	Body
cg05242371	19	46,286,475	1.87E-23	0.271	0.272				*DMPK*	TSS1500
cg11970252	13	42,188,629	6.81E-08	0.271	0.276				*KIAA0564*	Body
cg17701073	3	55,523,895	0.001171	0.271	0.173					
cg22756866	7	1,279,131	0.00252	0.271	0.287					
cg15146834	6	150,463,775	0.00182	0.271	0.241				*PPP1R14C*	TSS1500
cg19317600	4	8,395,941	0.001433	0.271	0.325				*ACOX3*	Body
cg24374636	15	41,852,586	6.28E-08	0.271	0.254				*TYRO3*	Body
cg00764307	14	94,408,121	3.22E-10	0.271	0.246				*ASB2*	Body
cg18282455	13	36,729,109	0.00101	0.270	0.231					
cg08856601	1	65,730,351	0.000461	0.270	0.234				*DNAJC6*	TSS200
cg09295252	6	5,026,552	0.035166	0.270	0.308					
cg12938068	13	28,549,698	0.01731	0.270	0.169					
cg24884142	1	119,532,056	1.61E-09	0.270	0.247				*TBX15*	1stExon
cg21266975	18	2,846,181	5.24E-12	0.270	0.256				*EMILIN2*	TSS1500
cg23792314	12	25,056,243	0.000645	0.270	0.260				*BCAT1*	Body
cg04191427	17	78,833,259	0.000781	0.270	0.177				*RPTOR*	Body
cg05768427	3	127,634,188	0.021187	0.270	0.140					
cg07034329	5	66,124,395	0.004614	0.270	0.109				*MAST4*	TSS1500
cg05997941	2	219,845,912	2.86E-25	0.270	0.272				*FEV*	3'UTR
cg21839984	17	30,846,986	1.21E-11	0.270	0.261				*MYO1D*	Body
cg25652029	2	182,321,786	0.013258	0.270	0.266				*ITGA4*	5'UTR

cg23359665	6	32,120,907	4.71E-09	0.270	0.284				*PPT2*	TSS1500
cg01884057	2	25,150,051	1.63E-13	0.270	0.250					
cg09696044	20	55,968,294	1.35E-14	0.270	0.247				*RBM38*	Body
cg08276171	14	104,850,405	4.03E-05	0.270	0.241					
cg18091046	13	30,077,344	0.000225	0.270	0.221				*MTUS2*	3'UTR
cg15424989	18	19,757,468	4.26E-23	0.270	0.238				*GATA6*	Body
cg13870539	10	95,848,649	2.43E-09	0.270	0.237				*PLCE1*	TSS200
cg09272217	11	45,179,364	3.2E-22	0.270	0.270				*PRDM11*	Body
cg25650964	13	33,544,014	1.51E-05	0.270	0.283					
cg25924694	12	102,872,603	7.61E-05	0.270	0.251				*IGF1*	Body
cg02685896	17	27,038,861	2.33E-05	0.270	0.270				*PROCA1*	5'UTR
cg11109845	7	148,394,888	9.08E-09	0.270	0.250				*CUL1*	TSS1500
cg20935741	2	176,968,584	0.001688	0.270	0.267					
cg18092474	15	75,019,302	2.37E-11	0.270	0.273				*CYP1A1*	TSS1500
cg04926361	14	36,982,447	1.91E-09	0.270	0.305				*SFTA3*	5'UTR
cg15453836	15	77,711,506	2.46E-07	0.270	0.235					
cg18599206	6	29,911,087	0.010515	0.270	0.141				*HLA-A*	Body
cg18298920	5	3,607,033	7.07E-09	0.270	0.304					
cg11216632	1	155,944,655	4.36E-08	0.270	0.240				*ARHGEF2*	Body
cg16586594	16	8,806,569	1.87E-09	0.270	0.276				*ABAT*	5'UTR
cg05485379	3	197,121,410	3.98E-22	0.270	0.244					
cg08370077	16	851,288	1.08E-07	0.270	0.271				*GNG13*	TSS1500
cg10440639	17	154,499	0.003966	0.270	0.213				*RPH3AL*	Body
cg10781468	6	168,778,312	9.53E-10	0.270	0.229					
cg05222995	12	115,124,973	5.12E-10	0.270	0.241					
cg20167366	6	30,094,980	1.76E-08	0.270	0.227					
cg18639180	14	91,580,605	0.040349	0.270	0.086				*C14orf159*	1stExon
cg14299940	9	131,684,923	0.010321	0.270	0.278				*PHYHD1*	Body
cg19571617	15	90,209,190	0.000926	0.270	0.170				*PLIN1*	Body
cg19894975	17	76,355,674	7.91E-05	0.270	0.245				*SOCS3*	5'UTR
cg17067528	6	30,712,517	0.023597	0.270	0.250	1.54E-10	0.131		*IER3*	TSS200
cg02745822	8	134,203,435	2.42E-14	0.270	0.271				*WISP1*	1stExon
cg07048832	13	50,883,529	1.32E-05	0.270	0.239					
cg10437217	6	33,215,814	3.46E-07	0.270	0.263					

cg14058647	9	88,137,909	0.00994	0.270	0.209						
cg03102442	8	125,738,432	3.97E-15	0.270	0.258					*MTSS1*	Body
cg26503073	7	6,704,074	1.22E-09	0.270	0.242						
cg01240599	11	67,418,045	1.54E-10	0.270	0.285					*ACY3*	5'UTR
cg04270799	12	57,944,059	0.00062	0.270	0.207	1.12E-13	0.185			*KIF5A*	1stExon
cg14013195	2	26,624,767	0.025583	0.270	0.063					*C2orf39*	TSS200
cg00950265	14	60,974,389	3.52E-08	0.270	0.238						
cg17169998	22	50,524,124	3.46E-05	0.270	0.270	1.75E-14	0.153			*MLC1*	TSS1500
cg15213210	1	18,962,937	0.009523	0.270	0.265					*PAX7*	Body
cg05328461	19	10,231,566	7.65E-18	0.270	0.264					*EIF3G*	TSS1500
cg23052830	14	61,119,919	2.55E-05	0.270	0.314						
cg09980477	17	66,255,444	0.001722	0.270	0.279					*ARSG*	5'UTR
cg01075271	14	38,080,314	1.06E-06	0.270	0.313						
cg04203646	19	1,467,008	0.001673	0.270	0.267					*APC2*	Body
cg15608483	2	175,192,815	7.01E-05	0.270	0.299						
cg20370678	17	58,216,297	0.000345	0.270	0.259						
cg22564583	22	37,720,976	0.003564	0.270	0.171						
cg17123338	X	153,361,684	1.81E-05	0.270	0.245					*MECP2*	Body
cg01152073	5	179,246,575	6.7E-10	0.270	0.235					*SQSTM1*	5'UTR
cg03322234	7	1,022,643	1.29E-21	0.270	0.261					*CYP2W1*	TSS200
cg08972081	3	139,259,435	1.16E-06	0.270	0.292					*RBP1*	TSS1500
cg26272623	1	119,532,320	5.31E-12	0.270	0.255					*TBX15*	TSS200
cg00295744	1	151,689,985	0.001237	0.270	0.248					*TNRC4*	TSS1500
cg25172243	X	138,724,688	0.004375	0.270	0.267					*MCF2*	1stExon
cg05382956	11	10,627,849	1.03E-09	0.270	0.270					*MRVI1*	Body
cg09802835	11	71,952,131	7.57E-09	0.270	0.233					*PHOX2A*	Body
cg23217097	14	42,074,635	1.99E-06	0.270	0.223						
cg05103064	10	7,311,500	0.002683	0.270	0.296					*SFMBT2*	Body
cg07015424	7	124,360,326	1.17E-10	0.270	0.287						
cg20260127	7	56,355,681	1.08E-08	0.270	0.234						
cg14516948	22	42,196,187	0.006045	0.270	0.239					*CCDC134*	TSS1500
cg01757168	3	128,215,565	1.14E-23	0.270	0.238						
cg05589489	12	89,748,821	2.14E-11	0.270	0.257						
cg23094674	1	204,329,186	1.94E-21	0.270	0.257					*PLEKHA6*	TSS200

cg13285968	2	180,725,645	3.82E-07	0.270	0.200				*ZNF385B*	5'UTR
cg01022780	3	48,598,236	5.7E-12	0.270	0.263					
cg15054274	3	105,072,683	1.68E-06	0.270	0.287					
cg05726600	7	150,498,594	1.02E-14	0.270	0.266				*TMEM176B*	TSS200
cg23187653	5	95,769,008	0.010887	0.270	0.244	1.55E-09	0.119		*PCSK1*	TSS200
cg23634124	17	48,049,952	0.000231	0.270	0.195				*DLX4*	TSS200
cg05331143	15	52,820,584	0.020337	0.270	0.135				*MYO5A*	Body
cg11991617	4	153,512,727	0.020024	0.270	0.255					
cg17177995	1	70,035,846	1.26E-08	0.270	0.276					
cg12187586	17	2,627,661	0.024871	0.270	0.307					
cg26433034	7	128,430,626	9.64E-09	0.270	0.260				*CCDC136*	TSS1500
cg12189835	11	61,335,071	1.25E-05	0.270	0.174				*SYT7*	Body
cg17146738	7	98,739,698	0.048517	0.270	0.266				*SMURF1*	Body
cg07435331	17	21,178,476	1.21E-31	0.270	0.260					
cg00851088	5	133,406,550	0.001852	0.270	0.140					
cg03733219	19	38,886,319	4.32E-05	0.270	0.253				*SPRED3*	Body
cg10758824	1	164,606,935	3.42E-14	0.270	0.261				*PBX1*	Body
cg19923650	5	172,659,730	0.021268	0.270	0.423				*NKX2-5*	3'UTR
cg07209634	7	156,796,550	0.003502	0.270	0.213					
cg08528170	19	13,947,914	4.24E-10	0.270	0.266				*MIR24-2*	TSS1500
cg09044785	20	30,458,634	0.008972	0.270	0.178				*TTLL9*	5'UTR
cg06110458	16	85,256,305	2.69E-07	0.270	0.279					
cg03730428	11	32,009,004	0.000111	0.270	0.282					
cg06523224	15	83,953,883	1.04E-05	0.270	0.255				*BNC1*	TSS1500
cg18642503	19	14,089,711	0.000132	0.270	0.222				*RFX1*	Body
cg00839584	2	113,542,091	5.13E-06	0.270	0.308	1.9E-30	0.229		*IL1A*	1stExon
cg01558501	X	99,667,223	0.002645	0.270	0.232					
cg03039974	19	49,467,169	5.79E-05	0.270	0.288				*FTL*	TSS1500
cg27529555	1	205,325,859	1.01E-13	0.270	0.266				*KLHDC8A*	5'UTR
cg12598089	3	156,008,770	2.5E-07	0.270	0.324				*KCNAB1*	TSS200
cg13543436	9	79,488,657	2.12E-22	0.270	0.268				*PRUNE2*	Body
cg02863073	19	39,694,831	0.034658	0.270	0.346				*SYCN*	1stExon
cg14453201	5	320,888	0.033008	0.270	0.208				*AHRR*	Body
cg19814981	5	3,592,435	8.01E-15	0.270	0.264					

cg06624337	15	47,477,411	0.003894	0.269	0.221					
cg23209976	5	138,872,772	4.6E-08	0.269	0.292					
cg02873954	21	45,161,415	9.19E-08	0.269	0.228				*PDXK*	Body
cg23288059	14	38,724,945	7.06E-05	0.269	0.291				*CLEC14A*	1stExon
cg02941781	2	191,045,563	0.000269	0.269	0.256				*C2orf88*	5'UTR
cg09414673	7	1,022,797	2.39E-16	0.269	0.251				*CYP2W1*	TSS200
cg04726446	2	26,624,865	0.014728	0.269	0.075	2.73E-14	0.159		*C2orf39*	1stExon
cg22004069	19	5,585,648	5.61E-19	0.269	0.247					
cg24280832	10	77,871,958	1.94E-18	0.269	0.249				*C10orf11*	Body
cg24399924	2	85,980,533	0.002833	0.269	0.254				*ATOH8*	TSS1500
cg06552366	2	161,263,994	3.07E-15	0.269	0.270				*RBMS1*	Body
cg16068038	15	69,099,678	1.61E-08	0.269	0.268				*C15orf28*	TSS1500
cg26926612	1	214,153,294	0.000912	0.269	0.349					
cg06846669	5	134,879,739	2.76E-08	0.269	0.265					
cg06593715	3	42,814,427	2.28E-06	0.269	0.277				*CCDC13*	5'UTR
cg02026062	6	117,086,479	0.003999	0.269	0.287				*FAM162B*	Body
cg04569152	14	21,555,603	5.62E-08	0.269	0.263				*FLJ10357*	Body
cg19764436	22	23,413,260	1.49E-07	0.269	0.175	2.15E-29	0.180		*RTDR1*	Body
cg08944026	5	10,626,811	1.39E-15	0.269	0.245				*ANKRD33B*	Body
cg13874290	2	70,017,056	1.09E-07	0.269	0.259				*ANXA4*	Body
cg01932956	16	22,959,525	0.002194	0.269	0.225					
cg05604079	4	122,302,268	0.042664	0.269	0.250				*QRFPR*	TSS200
cg01141339	5	72,530,198	1.04E-06	0.269	0.241					
cg12064372	12	30,948,792	0.001258	0.269	0.260					
cg09737314	17	6,899,359	1.04E-06	0.269	0.213				*ALOX12*	TSS200
cg10911287	12	125,271,306	5.48E-15	0.269	0.239				*SCARB1*	Body
cg00221494	13	98,794,593	2.61E-10	0.269	0.175				*FARP1*	TSS1500
cg15686334	8	60,030,291	0.001138	0.269	0.251				*TOX*	Body
cg13464990	9	135,421,719	0.008382	0.269	0.210				*C9orf171*	Body
cg16023415	1	20,692,812	0.02419	0.269	0.355					
cg04122873	6	28,956,226	3.16E-06	0.269	0.264					
cg15945129	7	120,629,051	0.001341	0.269	0.181				*C7orf58*	TSS1500
cg22001208	19	18,782,374	1.26E-11	0.269	0.261					
cg16555537	7	100,464,771	3.28E-08	0.269	0.295				*TRIP6*	TSS200

cg19616230	4	25,657,429	0.000859	0.269	0.154	3.79E-14	0.271		*SLC34A2*	TSS200
cg21459969	19	36,343,521	4.82E-07	0.269	0.257				*NPHS1*	TSS1500
cg13097407	6	43,276,658	0.039569	0.269	0.190	5.63E-21	0.185		*CRIP3*	TSS200
cg20431191	1	86,043,795	7.77E-05	0.269	0.277				*DDAH1*	1stExon
cg00247571	13	50,707,065	4.12E-05	0.269	0.260					
cg05492660	2	71,205,564	0.019958	0.269	0.338				*ANKRD53*	TSS200
cg13645732	19	45,901,649	0.00127	0.269	0.310				*PPP1R13L*	5'UTR
cg08855938	1	214,398,483	1.36E-07	0.269	0.266					
cg25864523	6	28,557,388	0.002609	0.269	0.214					
cg27542552	19	11,689,488	2.04E-05	0.269	0.262				*ACP5*	TSS1500
cg23740940	11	68,924,746	8.84E-13	0.269	0.267					
cg21080336	6	10,426,657	0.00012	0.269	0.327					
cg00158333	17	47,075,855	0.000205	0.269	0.219				*IGF2BP1*	Body
cg15827779	14	103,524,899	0.001471	0.269	0.340				*CDC42BPB*	TSS1500
cg09599027	X	43,745,111	3.37E-08	0.269	0.245					
cg07461572	8	1,955,117	2.43E-14	0.269	0.251					
cg08032924	16	66,613,096	2.04E-07	0.269	0.249				*CMTM2*	TSS1500
cg01255894	10	8,099,218	3.42E-12	0.269	0.278				*GATA3*	Body
cg04011829	20	61,275,033	2.47E-09	0.269	0.256				*SLCO4A1*	5'UTR
cg08043998	12	121,890,503	4E-10	0.269	0.274				*KDM2B*	Body
cg26127662	7	27,153,944	5.37E-06	0.269	0.214				*HOXA3*	5'UTR
cg08312412	5	141,443,781	1.7E-07	0.269	0.273					
cg22675801	7	28,998,346	4.87E-05	0.269	0.199				*TRIL*	TSS1500
cg03699566	11	71,900,652	1.04E-14	0.269	0.265	6.61E-21	0.167		*FOLR1*	1stExon
cg22508969	6	55,444,091	0.028594	0.269	0.271				*HMGCLL1*	TSS200
cg04562217	3	79,815,718	0.000307	0.269	0.225				*ROBO1*	5'UTR
cg20902817	15	30,277,796	2.88E-06	0.269	0.258					
cg19304410	1	64,971,188	0.001151	0.269	0.289	3.65E-21	0.147		*CACHD1*	Body
cg00939682	10	16,933,505	9.82E-05	0.269	0.266				*CUBN*	Body
cg02474563	10	134,442,032	1.59E-06	0.269	0.261				*INPP5A*	Body
cg02857325	16	86,564,928	2.59E-06	0.269	0.257				*MTHFSD*	3'UTR
cg22224704	11	67,352,041	1.05E-14	0.269	0.259	2.08E-21	0.165		*GSTP1*	Body
cg01286319	19	2,695,343	3.8E-06	0.269	0.259				*GNG7*	5'UTR
cg00901320	12	129,338,534	0.006798	0.269	0.175				*GLT1D1*	Body

cg26199241	17	59,529,486	9.05E-06	0.269	0.273					
cg27062571	8	124,890,314	5.44E-06	0.269	0.283				*FER1L6*	5'UTR
cg01850179	19	10,340,795	3.33E-10	0.269	0.272				*S1PR2*	5'UTR
cg00907288	3	9,178,249	8.21E-06	0.269	0.279				*SRGAP3*	Body
cg23052826	2	232,479,675	1.3E-05	0.269	0.274					
cg00459232	12	6,309,025	1.17E-06	0.269	0.272				*CD9*	TSS1500
cg12356951	17	35,165,018	0.00075	0.269	0.243					
cg01428589	19	33,167,718	1.18E-08	0.269	0.264				*RGS9BP*	1stExon
cg18831409	6	10,585,851	3.73E-19	0.269	0.265			Yes	*GCNT2*	Body
cg19376851	2	200,334,137	0.004678	0.269	0.314				*FLJ32063*	Body
cg03678566	11	68,106,018	2.07E-16	0.269	0.270				*LRP5*	Body
cg08802482	7	2,657,119	1.39E-18	0.269	0.258					
cg10395685	11	86,085,715	5.78E-06	0.269	0.261				*CCDC81*	TSS200
cg12065285	1	155,946,346	1.61E-20	0.269	0.275				*ARHGEF2*	Body
cg15639581	19	13,318,873	3.61E-06	0.269	0.275				*CACNA1A*	Body
cg17835180	X	12,995,592	0.000619	0.269	0.288					
cg01762581	8	42,234,614	3.65E-11	0.269	0.246	1.7E-22	0.206		*DKK4*	5'UTR
cg27456468	2	200,334,001	1.54E-06	0.269	0.238				*FLJ32063*	Body
cg16564033	21	38,067,741	0.004422	0.269	0.313					
cg24848599	1	228,604,800	1.41E-05	0.269	0.255				*TRIM17*	TSS1500
cg09396217	8	108,510,286	2.5E-13	0.269	0.276	2.22E-12	0.167		*ANGPT1*	TSS200
cg17280299	5	132,082,690	3.15E-08	0.269	0.263				*CCNI2*	TSS1500
cg21268578	22	38,029,478	3.38E-09	0.269	0.260				*GGA1*	3'UTR
cg21743907	7	129,423,053	2.9E-06	0.269	0.269			Yes		
cg03522245	20	25,566,470	0.003897	0.269	0.184				*NINL*	TSS1500
cg17681491	10	123,356,205	0.000291	0.269	0.222				*FGFR2*	5'UTR
cg12839172	4	16,085,077	0.015764	0.269	0.344				*PROM1*	5'UTR
cg23644624	22	21,386,989	0.017549	0.269	0.069				*SLC7A4*	TSS200
cg25769889	18	28,620,915	0.001502	0.269	0.224				*DSC3*	Body
cg04539503	19	34,311,327	0.007218	0.269	0.216					
cg13383058	11	66,084,660	1.09E-05	0.269	0.313				*CD248*	TSS200
cg14476745	9	124,979,356	1.06E-18	0.269	0.265				*LHX6*	Body
cg06818307	20	6,750,782	0.030315	0.269	0.238				*BMP2*	Body
cg22881247	15	101,460,222	0.02789	0.269	0.140				*LRRK1*	5'UTR

cg16519300	1	16,084,830	0.000297	0.269	0.319				*FBLIM1*	TSS1500
cg19827182	1	21,640,802	9.12E-20	0.269	0.264				*ECE1*	Body
cg01092108	5	33,362,853	2.76E-10	0.269	0.273					
cg17415326	1	79,350,918	3.41E-16	0.269	0.255					
cg27323784	11	116,451,730	0.025017	0.269	0.297					
cg15522171	1	33,547,626	1.47E-06	0.269	0.270				*ADC*	5'UTR
cg17241776	13	51,417,469	2.65E-05	0.269	0.260				*DLEU7*	1stExon
cg25171685	15	63,334,355	0.008585	0.268	0.212				*TPM1*	TSS1500
cg03580358	8	41,981,423	3.29E-07	0.268	0.244					
cg04646987	5	14,406,732	5.66E-15	0.268	0.257				*TRIO*	Body
cg11765288	7	18,535,767	0.00401	0.268	0.238				*HDAC9*	TSS200
cg16742874	10	77,055,847	0.001175	0.268	0.218					
cg25017060	14	103,389,017	2.56E-06	0.268	0.232				*AMN*	1stExon
cg27485906	13	42,136,511	6.04E-09	0.268	0.273					
cg05872387	2	242,716,801	2.89E-20	0.268	0.249				*GAL3ST2*	Body
cg09236382	11	6,676,366	5.31E-13	0.268	0.220				*DCHS1*	5'UTR
cg12099952	20	3,053,037	0.022232	0.268	0.172				*OXT*	Body
cg14204462	1	119,540,044	1.24E-05	0.268	0.242					
cg01447112	7	6,703,803	0.036702	0.268	0.249					
cg21750887	8	68,658,237	2.64E-11	0.268	0.246	9.63E-26	0.203		*CPA6*	Body
cg07034362	3	125,899,552	0.03964	0.268	0.175				*ALDH1L1*	TSS200
cg21944234	3	177,234,095	6.09E-15	0.268	0.271					
cg00850068	19	8,428,494	0.0035	0.268	0.200				*ANGPTL4*	TSS1500
cg02290168	1	151,255,971	1.49E-05	0.268	0.249				*ZNF687*	5'UTR
cg02963866	15	29,612,015	5.32E-17	0.268	0.275				*FAM189A1*	Body
cg11009362	12	118,313,924	8.64E-07	0.268	0.253				*KSR2*	Body
cg14141880	7	47,649,923	2.53E-18	0.268	0.262			Yes		
cg27246129	6	170,595,947	8.4E-10	0.268	0.246				*DLL1*	Body
cg12484113	1	27,898,757	2.27E-07	0.268	0.308				*AHDC1*	5'UTR
cg11052668	6	28,626,564	0.011572	0.268	0.270					
cg14233604	9	83,412,508	5.63E-19	0.268	0.256					
cg05037662	16	25,704,296	6.02E-05	0.268	0.293				*HS3ST4*	1stExon
cg06720669	X	108,868,282	0.00027	0.268	0.309				*KCNE1L*	1stExon
cg06148175	11	67,418,148	1.87E-09	0.268	0.262	1.13E-09	0.099		*ACY3*	TSS200

cg11862551	6	29,817,963	0.007123	0.268	0.298					
cg14926717	14	62,583,919	0.018828	0.268	0.333				*FLJ43390*	TSS200
cg10436257	16	30,959,496	1.17E-16	0.268	0.251				*FBXL19*	3'UTR
cg27429195	10	1,602,108	0.001079	0.268	0.329				*ADARB2*	Body
cg26907842	9	37,036,052	0.00901	0.268	0.295					
cg00204782	22	19,753,364	9.19E-06	0.268	0.225				*TBX1*	Body
cg01919401	7	130,985,828	5E-07	0.268	0.327				*MKLN1*	Body
cg14002960	18	31,739,423	0.038854	0.268	0.362				*NOL4*	Body
cg15025103	5	177,411,380	1.12E-06	0.268	0.267					
cg13187764	6	28,677,521	0.002351	0.268	0.291					
cg04809274	6	32,977,254	0.000897	0.268	0.253				*HLA-DOA*	1stExon
cg23079727	3	11,598,978	2.25E-13	0.268	0.275				*VGLL4*	3'UTR
cg18197392	20	23,015,908	0.009134	0.268	0.244				*SSTR4*	TSS200
cg23973417	7	116,164,753	0.026973	0.268	0.218				*CAV1*	TSS200
cg06469229	5	59,188,213	7.95E-09	0.268	0.253				*PDE4D*	Body
cg21514834	2	132,285,991	0.003087	0.268	0.173				*CCDC74A*	Body
cg03452737	3	117,385,277	1.17E-08	0.268	0.268					
cg16290866	8	97,167,768	1.67E-07	0.268	0.245				*GDF6*	Body
cg18058747	5	78,408,253	1.6E-12	0.268	0.266				*BHMT*	Body
cg22982570	3	51,997,033	9.83E-17	0.268	0.253				*PCBP4*	TSS200
cg13592721	6	27,107,393	2.33E-06	0.268	0.263			Yes	*HIST1H2BK*	3'UTR
cg21386766	12	94,286,778	1.05E-05	0.268	0.217					
cg03557857	10	103,589,693	6.5E-05	0.268	0.251				*KCNIP2*	Body
cg09982773	7	128,550,860	4.39E-16	0.268	0.244				*KCP*	TSS200
cg13123927	4	41,746,697	1.96E-05	0.268	0.255				*PHOX2B*	3'UTR
cg26276327	1	247,511,469	4.27E-05	0.268	0.243					
cg08828819	7	95,025,836	0.037614	0.268	0.242				*PON3*	TSS200
cg03233426	17	53,800,554	0.001922	0.268	0.295				*TMEM100*	TSS1500
cg01466741	5	134,733,519	0.000172	0.268	0.339				*H2AFY*	5'UTR
cg14702307	7	64,700,697	3.78E-06	0.268	0.196					
cg06641357	16	29,197,753	2.21E-10	0.268	0.282					
cg26168881	2	9,451,170	0.015719	0.268	0.263				*ASAP2*	Body
cg09547226	19	5,058,863	1.53E-21	0.268	0.243			Yes	*KDM4B*	Body
cg21618620	12	54,408,427	4.76E-09	0.268	0.255					

cg25370416	11	68,894,672	1.02E-11	0.268	0.257					
cg09113070	2	86,114,036	2.24E-09	0.268	0.280				*ST3GAL5*	Body
cg14248106	17	43,046,371	0.007112	0.268	0.146				*C1QL1*	TSS1500
cg03745372	3	57,197,870	9.18E-09	0.268	0.258				*IL17RD*	Body
cg04297907	X	10,126,668	0.000221	0.268	0.253				*CLCN4*	5'UTR
cg13094752	14	23,305,548	7.04E-06	0.268	0.298				*MMP14*	TSS1500
cg20406460	5	3,606,489	0.005936	0.268	0.381					
cg20719001	17	79,297,435	5.99E-11	0.268	0.267				*TMEM105*	5'UTR
cg21447550	1	223,538,623	0.001306	0.268	0.202				*SUSD4*	TSS1500
cg01215761	X	129,254,804	0.045861	0.268	0.379					
cg12749132	1	151,811,364	3.24E-05	0.268	0.278				*LOC100132111*	Body
cg23291534	8	110,988,043	0.014326	0.268	0.343				*KCNV1*	TSS1500
cg07571734	7	151,479,894	1.08E-05	0.268	0.276				*PRKAG2*	Body
cg03847373	5	50,265,767	0.006565	0.268	0.248					
cg10771480	14	64,319,275	0.0151	0.268	0.162				*SYNE2*	TSS1500
cg16348470	1	34,642,609	0.013222	0.268	0.192				*C1orf94*	5'UTR
cg19572766	17	48,206,424	1.05E-06	0.268	0.272				*SAMD14*	5'UTR
cg24313597	9	93,955,675	0.025249	0.268	0.133					
cg04305062	6	39,399,200	0.000707	0.268	0.213				*KIF6*	Body
cg10110367	21	46,131,464	3.68E-05	0.268	0.244				*C21orf29*	1stExon
cg16269733	1	156,616,804	0.0211	0.268	0.264				*BCAN*	Body
cg19541697	2	26,045,189	1.79E-23	0.268	0.254				*ASXL2*	Body
cg21604042	6	35,181,401	8.15E-07	0.268	0.169	2.62E-09	0.168		*SCUBE3*	TSS1500
cg04918402	17	61,778,550	2.05E-07	0.268	0.254				*LIMD2*	TSS1500
cg26864766	1	39,857,950	7.22E-05	0.268	0.268			Yes	*MACF1*	Body
cg08402963	13	99,135,625	0.002935	0.268	0.191				*STK24*	Body
cg25092838	14	57,279,115	0.00173	0.268	0.296				*OTX2OS1*	TSS1500
cg15192750	16	69,999,425	0.023071	0.268	0.268					
cg14126020	20	61,149,009	0.001758	0.268	0.297				*C20orf166*	5'UTR
cg18693395	14	52,735,421	0.022408	0.268	0.275				*PTGDR*	Body
cg10631356	11	34,517,541	3.86E-20	0.268	0.268				*ELF5*	Body
cg23764900	11	8,616,102	0.00118	0.268	0.105				*STK33*	TSS1500
cg06086731	1	38,942,075	0.000437	0.268	0.297					
cg08125125	5	149,980,486	2.02E-06	0.268	0.261				*SYNPO*	TSS200

cg13475583	1	235,116,731	0.000779	0.268	0.266					
cg03281661	16	3,239,458	1.28E-07	0.268	0.285					
cg06666025	16	66,613,278	5.85E-07	0.268	0.225				*CMTM2*	TSS200
cg17826344	19	51,169,660	0.000356	0.268	0.299				*SHANK1*	Body
cg26663490	17	72,443,066	3.88E-10	0.268	0.274				*GPRC5C*	Body
cg12460870	7	1,293,669	2.17E-09	0.268	0.276					
cg02220617	14	102,312,533	3.46E-09	0.268	0.232				*PPP2R5C*	Body
cg12905836	12	54,409,770	5.56E-07	0.268	0.233				*HOXC4*	TSS1500
cg23039195	10	22,624,663	0.003893	0.268	0.321					
cg05816193	6	26,018,127	2.94E-07	0.268	0.230				*HIST1H1A*	TSS200
cg07776847	4	140,201,065	0.000869	0.268	0.240				*C4orf49*	Body
cg08893692	5	72,595,741	0.011698	0.268	0.272					
cg27478242	12	54,611,928	1.88E-05	0.268	0.285					
cg15850851	2	43,446,987	0.018052	0.268	0.193					
cg07774424	X	101,915,091	0.000957	0.268	0.276					
cg24366211	11	68,610,706	5.57E-06	0.268	0.269				*CPT1A*	TSS1500
cg01427848	11	56,972,618	0.019474	0.268	0.248					
cg05713206	17	61,615,622	2.86E-08	0.268	0.245				*KCNH6*	Body
cg10338338	12	8,088,931	5.33E-09	0.268	0.278	1.51E-19	0.148		*SLC2A3*	TSS200
cg07005067	13	31,782,610	1.16E-14	0.268	0.262				*B3GALTL*	Body
cg08506654	5	172,673,098	5.75E-07	0.268	0.274					
cg14396995	7	42,278,089	1.42E-08	0.268	0.256				*GLI3*	TSS1500
cg00766964	13	24,693,844	4.19E-10	0.268	0.252					
cg03573945	8	25,897,342	5.97E-06	0.268	0.238				*EBF2*	Body
cg06133145	14	94,254,351	2.39E-10	0.268	0.260				*PRIMA1*	5'UTR
cg18048405	17	27,333,617	0.000338	0.268	0.270				*SEZ6*	TSS1500
cg22254104	9	124,991,432	0.000107	0.268	0.194				*LHX6*	TSS1500
cg15175162	4	15,657,657	9.76E-07	0.268	0.201				*FBXL5*	TSS1500
cg22558825	10	103,329,449	1.15E-07	0.268	0.236					
cg03513464	1	58,898,672	1.13E-05	0.268	0.167					
cg26198700	5	42,360,789	5.69E-05	0.268	0.243					
cg02092885	17	29,379,458	1.1E-14	0.268	0.252					
cg08378830	5	134,825,466	8.01E-07	0.268	0.272					
cg04318855	2	225,906,085	0.000611	0.268	0.326				*DOCK10*	Body

cg05062333	6	133,562,269	8.49E-05	0.268	0.317				*EYA4*	TSS1500
cg04155289	7	94,953,770	2.22E-10	0.267	0.252				*PON1*	1stExon
cg15635368	2	191,208,116	0.002523	0.267	0.174				*INPP1*	TSS200
cg25731261	19	47,736,018	9.31E-07	0.267	0.290				*BBC3*	1stExon
cg00502662	7	76,053,224	6.76E-22	0.267	0.251				*ZP3*	TSS1500
cg17594131	17	43,046,361	0.003908	0.267	0.161				*C1QL1*	TSS1500
cg21570209	19	46,367,987	7.85E-08	0.267	0.251				*FOXA3*	Body
cg04573550	14	38,680,313	3.56E-05	0.267	0.167				*SSTR1*	3'UTR
cg10996258	14	38,975,251	1.01E-05	0.267	0.267					
cg13073564	4	8,508,604	1.85E-07	0.267	0.293					
cg04896949	1	28,918,475	6.49E-12	0.267	0.242				*RAB42*	TSS1500
cg06629130	18	44,787,585	0.0008	0.267	0.242					
cg14673722	17	71,065,768	3.76E-17	0.267	0.269				*SLC39A11*	Body
cg20693209	11	13,984,067	2.97E-07	0.267	0.217				*SPON1*	1stExon
cg05342515	2	177,024,172	1.39E-05	0.267	0.219					
cg02319354	5	72,530,186	2.02E-07	0.267	0.231					
cg06907053	2	106,015,869	5.16E-05	0.267	0.218				*FHL2*	TSS200
cg07300846	16	29,888,571	1.09E-05	0.267	0.266				*SEZ6L2*	Body
cg12026967	18	19,780,728	4.28E-44	0.267	0.253				*GATA6*	Body
cg19486070	7	1,626,153	0.001393	0.267	0.235				*KIAA1908*	Body
cg26559209	10	13,570,012	0.000193	0.267	0.255					
cg03387092	3	123,493,248	7.81E-17	0.267	0.265				*MYLK*	Body
cg19798881	6	7,758,958	9.91E-15	0.267	0.257				*BMP6*	Body
cg11619961	5	149,792,840	0.000813	0.267	0.119				*CD74*	TSS1500
cg22592475	5	67,547,334	4.05E-09	0.267	0.244				*PIK3R1*	Body
cg14710465	1	160,369,869	3.3E-23	0.267	0.231				*VANGL2*	TSS1500
cg05185021	17	2,324,794	2.04E-14	0.267	0.267				*METT10D*	Body
cg20316549	7	156,810,507	0.0006	0.267	0.271					
cg26365925	3	44,063,315	0.000144	0.267	0.269					
cg13001963	5	10,467,973	5.36E-09	0.267	0.251					
cg12580156	10	112,588,051	1.12E-35	0.267	0.258				*RBM20*	Body
cg05184938	17	75,369,939	6.98E-05	0.267	0.221				*SEPT9*	5'UTR
cg06861044	16	20,359,815	0.046602	0.267	0.361				*UMOD*	Body
cg06215984	1	203,258,903	9.63E-15	0.267	0.240					

cg16097357	17	73,584,360	0.003985	0.267	0.217				*MYO15B*	Body
cg22898783	2	26,204,699	2.27E-10	0.267	0.211				*KIF3C*	1stExon
cg03800922	6	74,290,220	0.000154	0.267	0.291					
cg25219329	22	42,679,444	0.001352	0.267	0.234					
cg25305879	2	106,814,630	3.84E-11	0.267	0.289					
cg12643732	5	691,580	0.000381	0.267	0.292				*TPPP*	5'UTR
cg01760475	8	145,104,359	0.00105	0.267	0.266					
cg15168995	22	37,914,206	1.34E-10	0.267	0.284				*CARD10*	Body
cg03773647	11	57,417,949	9.15E-07	0.267	0.228				*YPEL4*	TSS1500
cg19228647	6	6,901,002	1.86E-08	0.267	0.231					
cg26279336	17	46,674,438	0.007691	0.267	0.218				*LOC404266*	Body
cg15034300	2	85,134,468	1.4E-05	0.267	0.167					
cg19126910	11	111,249,659	2.05E-12	0.267	0.249				*POU2AF1*	Body
cg06061257	17	71,361,901	1.79E-07	0.267	0.268				*SDK2*	Body
cg18749404	17	76,975,944	0.001065	0.267	0.213				*LGALS3BP*	1stExon
cg14831380	8	144,510,421	0.000308	0.267	0.332					
cg19643252	7	151,264,653	1.81E-05	0.267	0.242				*PRKAG2*	Body
cg10279204	10	71,176,853	2.39E-16	0.267	0.252				*TACR2*	TSS200
cg24375938	17	13,505,799	0.007055	0.267	0.160				*HS3ST3A1*	TSS1500
cg00811132	22	19,511,707	0.0064	0.267	0.304				*CLDN5*	1stExon
cg12769994	1	149,784,571	2.23E-05	0.267	0.241				*HIST2H2BF*	TSS1500
cg00014830	17	77,765,733	0.001033	0.267	0.226					
cg02059867	17	38,334,623	2.36E-13	0.267	0.249				*RAPGEFL1*	1stExon
cg12440549	13	28,541,477	0.003149	0.267	0.191				*CDX2*	Body
cg02882448	15	33,487,058	0.019486	0.267	0.155					
cg15235999	19	52,996,352	0.001334	0.267	0.265				*ZNF578*	5'UTR
cg16379910	14	78,636,730	0.025069	0.267	0.285					
cg17977173	2	129,076,335	0.000135	0.267	0.253				*HS6ST1*	TSS200
cg10395711	12	6,938,638	2.56E-05	0.267	0.268				*LEPREL2*	Body
cg10983484	15	52,018,762	2.59E-09	0.267	0.297				*LYSMD2*	Body
cg15032098	4	41,258,560	7.04E-05	0.267	0.255				*UCHL1*	TSS1500
cg26728517	20	39,319,540	7.84E-08	0.267	0.269					
cg21395577	1	221,060,851	9.75E-06	0.267	0.260					
cg24473500	7	871,436	0.000138	0.267	0.355				*UNC84A*	5'UTR

cg24395504	6	154,869,909	8.54E-10	0.267	0.244					
cg11711057	12	107,974,444	8.04E-13	0.267	0.261				*BTBD11*	TSS200
cg13066043	14	24,808,987	5.22E-08	0.267	0.273				*RIPK3*	Body
cg22213386	5	156,887,005	0.009896	0.267	0.194				*NIPAL4*	TSS200
cg24074448	19	18,768,688	1.95E-33	0.267	0.258				*KLHL26*	Body
cg26651950	22	37,976,179	5.71E-13	0.267	0.259				*LGALS2*	TSS200
cg14557288	10	72,454,026	0.006459	0.267	0.175				*ADAMTS14*	Body
cg04337944	22	45,899,303	1.76E-13	0.267	0.245	4.08E-37	0.222		*FBLN1*	Body
cg13019491	14	60,977,856	1.03E-05	0.267	0.263				*SIX6*	Body
cg20956738	18	55,108,537	0.008333	0.267	0.301				*ONECUT2*	Body
cg12628061	1	56,453,730	1.01E-12	0.267	0.214					
cg13457165	1	40,105,079	1.34E-05	0.267	0.282				*HEYL*	Body
cg14286456	1	8,570,225	2.32E-15	0.267	0.253				*RERE*	Body
cg19170015	4	74,734,714	0.00037	0.267	0.183				*CXCL1*	TSS1500
cg02022808	5	140,744,914	0.002455	0.267	0.312				*PCDHGA4*	Body
cg12606409	2	27,958,270	0.000119	0.267	0.176					
cg27406975	2	172,955,512	0.048453	0.267	0.333					
cg17860198	3	5,138,170	9.02E-05	0.267	0.262					
cg18815343	6	28,367,644	0.000516	0.267	0.216				*ZSCAN12*	TSS200
cg21853707	1	164,676,874	8.29E-16	0.267	0.258				*PBX1*	Body
cg24813736	5	63,255,045	1.78E-05	0.267	0.234					
cg09371112	11	12,222,570	2.31E-16	0.267	0.258				*MICAL2*	Body
cg16237891	1	146,544,645	8.76E-07	0.267	0.299					
cg26570165	1	119,541,833	6.56E-09	0.267	0.260					
cg23999695	15	89,943,625	0.041427	0.267	0.239					
cg24021956	7	79,083,509	0.006223	0.267	0.227				*MAGI2*	TSS1500
cg24877675	10	133,995,387	4.42E-11	0.267	0.257				*JAKMIP3*	3'UTR
cg15820574	1	156,406,711	0.001854	0.267	0.319					
cg00990385	12	113,587,690	5.11E-06	0.267	0.273				*CCDC42B*	1stExon
cg13809441	6	6,737,631	5.37E-09	0.267	0.216					
cg19246977	17	17,280,399	0.010413	0.267	0.156					
cg13300273	1	200,842,890	7.38E-08	0.267	0.237				*GPR25*	1stExon
cg03512076	8	103,907,077	4.02E-17	0.267	0.260					
cg12438430	10	1,507,896	1.63E-07	0.267	0.183				*ADARB2*	Body

cg13501279	1	167,523,555	0.002713	0.267	0.198				*CREG1*	TSS1500
cg14689479	8	53,326,618	0.000146	0.267	0.314					
cg13655250	9	134,544,528	1.44E-10	0.267	0.249				*RAPGEF1*	Body
cg07621749	15	41,806,141	1.27E-05	0.267	0.262				*LTK*	TSS200
cg00720047	16	1,186,227	5.82E-05	0.267	0.288					
cg02309273	1	38,412,744	1.89E-05	0.267	0.280	2.72E-27	0.182		*INPP5B*	TSS200
cg07881210	17	398,090	3.95E-12	0.267	0.248					
cg19776793	17	79,225,974	2.78E-15	0.267	0.246				*SLC38A10*	Body
cg04674508	6	29,975,239	2.85E-09	0.267	0.239				*HLA-J*	Body
cg05940984	11	32,448,678	0.020066	0.267	0.268				*WT1*	Body
cg03803086	21	34,185,960	2.47E-09	0.266	0.248				*C21orf62*	5'UTR
cg20322093	17	72,931,788	3.66E-12	0.266	0.224				*OTOP3*	TSS200
cg27391934	1	1,369,948	0.035266	0.266	0.152				*VWA1*	TSS1500
cg06677890	5	149,887,486	1.67E-13	0.266	0.249				*NDST1*	TSS200
cg08394412	16	50,875,140	0.008672	0.266	0.308					
cg04927502	1	149,889,361	0.008175	0.266	0.308				*SV2A*	5'UTR
cg04615865	12	121,678,720	0.005436	0.266	0.364				*CAMKK2*	Body
cg27315333	2	63,285,950	3.51E-05	0.266	0.247					
cg13668314	9	130,692,638	0.000911	0.266	0.130				*PIP5KL1*	Body
cg19479609	2	208,390,216	3.05E-06	0.266	0.250					
cg20635389	2	207,859,049	2.74E-05	0.266	0.234			Yes		
cg11628739	17	60,731,254	3.01E-18	0.266	0.249				*MRC2*	Body
cg14354895	22	50,607,824	3.5E-05	0.266	0.267				*PANX2*	TSS1500
cg04360793	1	79,472,361	5.94E-07	0.266	0.266				*ELTD1*	5'UTR
cg08814105	5	172,662,130	0.04056	0.266	0.174				*NKX2-5*	5'UTR
cg00647859	1	185,534,625	3.9E-12	0.266	0.263					
cg18373855	6	159,360,209	1.01E-11	0.266	0.266					
cg05357152	20	61,049,813	7.31E-07	0.266	0.247				*GATA5*	Body
cg21568661	15	62,411,575	0.000338	0.266	0.276					
cg09519327	7	150,710,730	0.025722	0.266	0.323				*ATG9B*	3'UTR
cg00290974	11	10,316,461	0.007575	0.266	0.136				*SBF2*	TSS1500
cg09281101	1	236,227,410	0.001722	0.266	0.203				*NID1*	Body
cg18676237	6	2,903,258	0.005683	0.266	0.291	2.53E-13	0.187		*SERPINB9*	5'UTR
cg14546076	6	29,796,420	8.33E-07	0.266	0.241				*HLA-G*	Body

cg00282510	9	100,611,516	0.001223	0.266	0.271					
cg24628744	5	134,735,654	3.48E-05	0.266	0.216	1.04E-13	0.238		*H2AFY*	TSS1500
cg01275133	16	85,229,580	4.21E-14	0.266	0.249					
cg16922279	11	71,955,439	7.66E-07	0.266	0.299				*PHOX2A*	TSS1500
cg18968196	2	39,892,502	0.000155	0.266	0.241				*TMEM178*	TSS1500
cg02953912	11	2,904,591	3.96E-27	0.266	0.257				*CDKN1C*	3'UTR
cg04826040	12	132,933,643	1.62E-05	0.266	0.256					
cg20176388	2	17,724,532	1.49E-08	0.266	0.245				*VSNL1*	5'UTR
cg13187827	6	31,734,192	6.9E-13	0.266	0.256				*C6orf27*	Body
cg23615741	10	101,297,642	1.24E-05	0.266	0.245					
cg15464821	1	167,682,648	2.69E-09	0.266	0.247					
cg02325832	4	13,542,883	7.64E-09	0.266	0.246				*NKX3-2*	3'UTR
cg00321234	4	141,217,605	4.63E-10	0.266	0.217				*SCOC*	5'UTR
cg03052099	16	67,951,373	5.37E-10	0.266	0.280				*PSKH1*	Body
cg12148515	8	41,517,301	3.76E-14	0.266	0.269				*ANK1*	3'UTR
cg22465479	19	50,306,072	0.000286	0.266	0.272				*AP2A1*	Body
cg12133048	5	141,293,785	0.017283	0.266	0.206					
cg13889415	9	116,943,379	0.000309	0.266	0.244				*COL27A1*	Body
cg26822986	18	21,199,691	6.15E-05	0.266	0.247				*ANKRD29*	Body
cg13302869	2	131,010,522	0.00013	0.266	0.224					
cg12020396	19	55,593,580	1.2E-05	0.266	0.271				*EPS8L1*	Body
cg24381412	15	45,996,787	0.021777	0.266	0.230					
cg18050804	13	113,677,188	9.18E-09	0.266	0.279				*MCF2L*	Body
cg04394967	14	68,189,457	0.005206	0.266	0.263	0.00000097	0.088		*RDH12*	Body
cg17202313	3	181,444,989	0.036125	0.266	0.324				*SOX2OT*	Body
cg19841369	14	64,663,928	3.28E-06	0.266	0.253				*SYNE2*	Body
cg03933572	2	150,187,926	6.87E-06	0.266	0.199				*LYPD6*	5'UTR
cg08833577	10	118,899,288	6.46E-06	0.266	0.255				*VAX1*	TSS1500
cg09699056	17	79,503,642	9.44E-09	0.266	0.254			Yes	*FSCN2*	Body
cg09984479	22	38,635,620	1.69E-05	0.266	0.314				*TMEM184B*	Body
cg22272282	6	27,107,670	2.99E-07	0.266	0.260				*HIST1H2BK*	3'UTR
cg23383189	5	176,560,116	1.14E-06	0.266	0.237				*NSD1*	1stExon
cg16120833	17	72,443,085	8.14E-11	0.266	0.273				*GPRC5C*	Body
cg21163444	12	54,765,670	2.36E-16	0.266	0.247				*ZNF385A*	Body

cg19695041	8	38,615,330	8.96E-08	0.266	0.208				*TACC1*	5'UTR
cg21560142	19	47,730,821	0.006553	0.266	0.334				*BBC3*	Body
cg14094646	9	37,030,262	1.91E-10	0.266	0.267				*PAX5*	Body
cg22122321	4	85,407,081	9.53E-12	0.266	0.256					
cg04093349	15	66,998,947	1.57E-55	0.266	0.259				*SMAD6*	Body
cg03562414	19	52,391,078	0.011008	0.266	0.255				*ZNF577*	Body
cg23647037	X	119,150,375	0.022605	0.266	0.228					
cg21139587	1	201,455,370	1.43E-05	0.266	0.295				*CSRP1*	Body
cg18948199	12	118,313,708	7.84E-08	0.266	0.265				*KSR2*	Body
cg00299972	10	102,892,528	7.31E-07	0.266	0.255				*TLX1*	Body
cg03969996	19	46,999,055	1.18E-06	0.266	0.277				*PNMAL2*	1stExon
cg10124710	1	146,544,267	2.68E-09	0.266	0.267					
cg12173409	3	54,154,746	0.00551	0.266	0.230					
cg13700552	X	101,968,505	1.39E-08	0.266	0.260				*GPRASP2*	5'UTR
cg20346189	X	71,524,766	1.7E-06	0.266	0.246				*CITED1*	5'UTR
cg20514322	2	134,023,662	5.98E-08	0.266	0.246				*NCKAP5*	Body
cg10421979	17	48,546,321	0.024451	0.266	0.239				*CHAD*	TSS200
cg27274135	X	129,093,798	0.023707	0.266	0.260					
cg14845020	20	55,833,416	1.18E-11	0.266	0.285				*BMP7*	Body
cg06201861	16	84,877,964	2.64E-14	0.266	0.256				*CRISPLD2*	Body
cg04904099	16	68,678,010	4.73E-08	0.266	0.235				*CDH3*	TSS200
cg07448060	7	79,083,753	0.004249	0.266	0.303				*MAGI2*	TSS1500
cg14482313	12	52,626,889	3.96E-05	0.266	0.291				*KRT7*	TSS200
cg25623768	2	45,241,008	0.025314	0.266	0.302					
cg14420245	2	148,601,095	1.78E-08	0.266	0.225				*ACVR2A*	TSS1500
cg22920700	8	99,955,610	6.49E-10	0.266	0.269				*OSR2*	TSS1500
cg24643417	9	503,937	0.001352	0.266	0.174				*KANK1*	TSS1500
cg00188055	6	32,076,079	1.75E-08	0.266	0.260				*TNXB*	5'UTR
cg12177220	3	38,080,925	0.020804	0.266	0.180				*DLEC1*	1stExon
cg10322124	8	74,331,865	2.95E-18	0.266	0.268					
cg17099016	11	17,568,889	1.8E-18	0.266	0.273					
cg20253855	7	101,762,511	2.56E-19	0.266	0.257				*CUX1*	Body
cg01859228	15	64,208,452	1.86E-13	0.266	0.284				*DAPK2*	Body
cg27520776	1	217,311,982	8.78E-08	0.266	0.270				*ESRRG*	TSS1500

cg00112465	3	128,719,999	0.006941	0.266	0.229				*CCDC48*	TSS1500
cg12439439	11	129,243,219	0.000134	0.266	0.286					
cg05282518	14	20,344,920	4.36E-18	0.266	0.234				*OR4K2*	1stExon
cg12604950	1	180,882,287	0.001558	0.266	0.236				*KIAA1614*	TSS200
cg19370054	2	98,964,186	0.002096	0.266	0.231				*CNGA3*	5'UTR
cg13490403	9	124,982,413	3.91E-05	0.266	0.283				*LHX6*	Body
cg01763575	20	44,875,431	0.004132	0.266	0.191				*CDH22*	Body
cg04083712	7	99,156,968	0.004012	0.266	0.208				*ZNF655*	5'UTR
cg09628195	1	119,551,303	0.008733	0.266	0.286					
cg22870092	4	7,918,691	3.94E-23	0.266	0.253				*AFAP1*	5'UTR
cg13699414	5	178,957,880	6.33E-10	0.266	0.236					
cg19756821	1	54,940,702	0.005821	0.266	0.116					
cg26472133	7	156,797,104	1.82E-08	0.266	0.228					
cg19023847	10	99,344,164	4.35E-07	0.266	0.285				*DHDPSL*	1stExon
cg25044651	5	115,298,720	4.41E-06	0.266	0.243	9.26E-24	0.242		*LVRN*	1stExon
cg02649597	2	45,171,621	1.99E-08	0.266	0.258				*SIX3*	Body
cg04706201	5	131,773,770	1.6E-09	0.266	0.260				*C5orf56*	Body
cg26575622	10	77,872,458	0.000666	0.266	0.154			Yes	*C10orf11*	Body
cg11743000	1	65,355,578	8.61E-10	0.265	0.271				*JAK1*	5'UTR
cg23239574	1	228,653,136	0.00335	0.265	0.353					
cg06571407	20	44,746,823	6.63E-05	0.265	0.320				*CD40*	TSS200
cg17459443	17	6,559,109	0.006267	0.265	0.332					
cg27563985	20	54,580,677	8.28E-07	0.265	0.287				*CBLN4*	TSS1500
cg25763036	4	13,540,946	0.027413	0.265	0.354					
cg05253110	7	141,130,687	2.21E-05	0.265	0.234					
cg09976984	6	27,182,943	0.00142	0.265	0.320					
cg25120290	14	85,996,873	0.009368	0.265	0.282				*FLRT2*	1stExon
cg15027426	Y	21,664,604	0.00034	0.265	0.269				*BCORL2*	Body
cg08376310	11	2,858,621	1.59E-13	0.265	0.250				*KCNQ1*	Body
cg13366537	7	30,029,754	0.003406	0.265	0.133				*SCRN1*	TSS1500
cg14338526	3	172,394,394	9.59E-18	0.265	0.241				*NCEH1*	5'UTR
cg18119977	8	96,226,844	0.00017	0.265	0.316					
cg03396151	15	37,387,304	0.00884	0.265	0.322				*MEIS2*	Body
cg03465499	11	68,781,279	0.000843	0.265	0.201				*MRGPRF*	TSS1500

cg07382998	17	76,880,071	3.29E-08	0.265	0.243				*TIMP2*	Body
cg13720108	3	62,364,032	0.007297	0.265	0.331					
cg22481263	1	906,493	1.77E-35	0.265	0.255				*PLEKHN1*	Body
cg07391141	21	44,035,034	1.15E-14	0.265	0.233					
cg12103951	10	88,162,314	4.6E-05	0.265	0.252					
cg22861587	7	91,196,675	3.34E-09	0.265	0.288					
cg03297163	7	2,548,137	0.001336	0.265	0.303					
cg26063904	1	101,313,199	3.81E-06	0.265	0.265					
cg07063583	X	8,698,228	1.16E-07	0.265	0.242				*KAL1*	Body
cg22304399	19	13,319,324	0.002073	0.265	0.321				*CACNA1A*	Body
cg12127282	2	177,014,685	1.08E-08	0.265	0.250	0.02732237	0.037		*HOXD4*	TSS1500
cg21800258	6	28,557,080	0.000882	0.265	0.219					
cg25428494	4	84,255,411	1.59E-13	0.265	0.267				*HPSE*	Body
cg27392904	19	50,400,714	0.001479	0.265	0.325				*IL4I1*	TSS1500
cg18551877	11	58,612,087	7.35E-06	0.265	0.271				*GLYATL2*	TSS200
cg07753510	3	171,111,243	2.14E-06	0.265	0.180				*TNIK*	Body
cg07721569	6	10,420,287	0.006347	0.265	0.292				*TFAP2A*	TSS1500
cg00544436	6	34,203,564	3.59E-06	0.265	0.252				*HMGA1*	TSS1500
cg12671030	2	109,647,956	2.6E-06	0.265	0.243					
cg14611399	3	87,138,203	8.64E-07	0.265	0.256					
cg07222863	8	104,153,592	1.95E-08	0.265	0.284				*C8orf56*	TSS200
cg16184495	19	15,343,350	0.003588	0.265	0.269				*EPHX3*	5'UTR
cg24527881	1	45,187,630	2.72E-14	0.265	0.251				*C1orf228*	Body
cg07540103	17	6,679,499	0.005691	0.265	0.247				*FBXO39*	TSS200
cg12869615	X	101,906,288	0.043889	0.265	0.321				*GPRASP1*	TSS200
cg08302480	19	54,369,133	9.26E-19	0.265	0.253				*MYADM*	TSS1500
cg09474229	19	39,360,330	0.010964	0.265	0.311				*RINL*	Body
cg14996220	12	85,673,270	0.015854	0.265	0.295				*ALX1*	TSS1500
cg04610224	11	111,411,756	0.007767	0.265	0.297				*LAYN*	Body
cg10694152	13	99,404,506	0.004428	0.265	0.235	2.44E-16	0.206		*SLC15A1*	Body
cg00877887	15	45,406,654	0.000474	0.265	0.233	8.26E-11	0.111		*DUOXA2*	1stExon
cg21056992	19	17,392,414	0.015906	0.265	0.243				*ANKLE1*	TSS200
cg13980799	10	119,000,215	2.59E-07	0.265	0.251				*SLC18A2*	TSS1500
cg15854847	8	23,563,970	0.001972	0.265	0.220				*NKX2-6*	TSS200

cg17265380	21	26,934,383	0.02291	0.265	0.238				*MIR155HG*	TSS200
cg04303382	17	77,766,421	1.44E-06	0.265	0.264					
cg00919971	7	1,286,149	0.000304	0.265	0.235					
cg09578794	14	104,690,040	1.09E-13	0.265	0.238					
cg26305817	5	172,656,055	0.000446	0.265	0.227					
cg01476568	10	5,734,716	4.01E-11	0.265	0.229				*C10orf18*	5'UTR
cg00173659	7	156,871,088	9.41E-09	0.265	0.273					
cg12523526	5	2,756,005	0.020471	0.265	0.246					
cg01539133	19	4,607,324	0.005276	0.265	0.165					
cg11764900	6	38,683,035	1.81E-12	0.265	0.249					
cg04934382	17	77,766,335	4.92E-07	0.265	0.266					
cg14121142	12	49,391,363	0.000347	0.265	0.337				*DDN*	Body
cg21091547	6	36,645,500	2.51E-05	0.265	0.277				*CDKN1A*	TSS1500
cg24828151	5	54,516,779	0.001679	0.265	0.264					
cg00918944	9	35,908,117	1.22E-11	0.265	0.272				*LOC158376*	TSS1500
cg01383997	3	126,423,799	7.14E-06	0.265	0.188				*CHCHD6*	Body
cg04666027	15	74,583,589	0.000498	0.265	0.204				*CCDC33*	Body
cg07265743	17	59,535,253	1.92E-06	0.265	0.274				*TBX4*	Body
cg25959149	20	17,674,250	5.26E-05	0.265	0.259				*BANF2*	TSS200
cg01899130	14	55,348,681	0.010693	0.265	0.250				*GCH1*	Body
cg00347677	1	63,795,636	5.24E-07	0.265	0.278					
cg26430059	14	89,770,384	0.023871	0.265	0.240				*FOXN3*	Body
cg03743720	2	241,460,002	1.22E-35	0.265	0.256				*ANKMY1*	Body
cg18031850	19	33,716,452	3.47E-06	0.265	0.257				*SLC7A10*	Body
cg18062140	20	55,967,678	7.68E-14	0.265	0.248				*RBM38*	Body
cg15792487	17	7,348,316	7.04E-13	0.265	0.223				*CHRNB1*	TSS200
cg26644853	16	57,406,257	5.22E-09	0.265	0.248				*CX3CL1*	TSS200
cg16144883	17	398,066	3.87E-41	0.265	0.263					
cg00490075	13	27,359,547	7.22E-05	0.265	0.274					
cg08282145	14	24,868,755	1.65E-09	0.265	0.278				*NYNRIN*	Body
cg23003500	3	42,543,530	0.024191	0.265	0.277				*VIPR1*	TSS1500
cg03804400	3	143,505,340	5.09E-27	0.265	0.264				*SLC9A9*	Body
cg05668342	16	1,340,422	4.26E-09	0.265	0.270					
cg16805068	20	31,410,931	3.45E-22	0.265	0.239				*MAPRE1*	5'UTR

cg00884221	4	57,976,915	0.01535	0.265	0.299	2.53E-07	0.135		*IGFBP7*	TSS1500
cg12951849	18	11,148,932	0.001038	0.265	0.231				*FAM38B*	TSS200
cg02511809	5	112,073,544	0.000346	0.265	0.148				*APC*	TSS200
cg11931727	2	58,655,790	0.010881	0.265	0.093					
cg19355079	19	23,253,218	0.001024	0.265	0.296					
cg21633059	5	146,863,675	1.43E-07	0.265	0.270					
cg05493001	19	52,195,559	4.24E-12	0.265	0.243				*MIR125A*	TSS1500
cg08750439	11	128,321,880	1.81E-19	0.265	0.261			Yes		
cg20587808	3	184,320,123	2.53E-11	0.265	0.249					
cg07714420	16	85,123,342	2.62E-14	0.265	0.262				*KIAA0513*	3'UTR
cg19764143	14	60,974,499	0.016343	0.265	0.216				*SIX6*	TSS1500
cg00177388	5	72,677,886	0.00014	0.265	0.246					
cg25298161	15	41,806,135	7.94E-07	0.265	0.254				*LTK*	TSS200
cg14659662	1	54,151,053	6.68E-14	0.265	0.268				*GLIS1*	5'UTR
cg23164183	5	2,754,894	0.000177	0.265	0.294				*C5orf38*	Body
cg14076258	11	77,183,807	2.43E-07	0.265	0.257				*PAK1*	5'UTR
cg15033068	19	3,801,922	6.3E-07	0.265	0.278				*MATK*	TSS200
cg07044708	15	75,471,034	7.66E-07	0.265	0.257					
cg16356622	1	36,351,841	1.12E-16	0.265	0.253				*EIF2C1*	Body
cg20189244	7	96,648,205	1.43E-10	0.265	0.257					
cg19465165	2	131,130,103	0.002118	0.265	0.212				*PTPN18*	Body
cg05349016	17	43,170,343	2.28E-12	0.265	0.270				*NMT1*	Body
cg10397678	14	103,655,712	6.23E-11	0.265	0.256					
cg07157030	14	63,671,356	9.13E-08	0.265	0.236				*RHOJ*	5'UTR
cg10422777	9	79,631,227	1.56E-05	0.265	0.222					
cg02115781	2	172,175,273	3.26E-15	0.265	0.238				*METTL8*	3'UTR
cg03955537	17	80,806,388	2.03E-10	0.264	0.286				*TBCD*	Body
cg01055281	16	50,746,052	6E-09	0.264	0.215				*NOD2*	Body
cg07165066	13	23,952,576	8.11E-06	0.264	0.238				*SACS*	Body
cg19529551	3	133,615,241	2.16E-07	0.264	0.187				*RAB6B*	TSS1500
cg08149253	22	45,899,292	3.59E-08	0.264	0.225				*FBLN1*	Body
cg12459718	17	8,129,997	0.001013	0.264	0.193				*C17orf68*	3'UTR
cg22110896	17	59,533,081	8.06E-16	0.264	0.238				*TBX4*	TSS1500
cg23003534	10	105,036,747	0.018985	0.264	0.250				*INA*	TSS200

cg01178624	11	65,360,327	0.001379	0.264	0.267				*KCNK7*	3'UTR
cg15809959	17	36,720,382	3.26E-06	0.264	0.270				*SRCIN1*	Body
cg16525435	3	193,994,972	1.25E-18	0.264	0.252					
cg22737624	5	140,750,111	0.002197	0.264	0.317				*PCDHGA4*	Body
cg25572105	7	95,025,955	0.000232	0.264	0.212				*PON3*	TSS1500
cg26697605	7	1,850,801	0.028803	0.264	0.172					
cg04454571	11	61,525,216	2.41E-20	0.264	0.254				*DKFZP434K028*	TSS200
cg08615596	12	68,758,840	6.26E-06	0.264	0.241					
cg13340269	X	100,333,778	0.007855	0.264	0.283	4.73E-07	0.112		*TMEM35*	TSS200
cg16649560	16	27,338,391	4.4E-13	0.264	0.259				*IL4R*	5'UTR
cg21122225	2	238,832,508	0.00778	0.264	0.167					
cg22153181	4	172,734,431	3.47E-05	0.264	0.281				*GALNTL6*	TSS200
cg25753817	2	191,208,232	0.000833	0.264	0.143	3.2E-19	0.171		*INPP1*	1stExon
cg00864474	17	9,143,838	0.001444	0.264	0.202				*NTN1*	3'UTR
cg06377106	1	20,618,331	9.73E-06	0.264	0.235				*VWA5B1*	5'UTR
cg19611886	8	103,821,171	1.13E-09	0.264	0.268					
cg22372930	19	55,630,001	1.7E-07	0.264	0.279				*PPP1R12C*	TSS1500
cg26557756	7	42,278,096	7.45E-13	0.264	0.281				*GLI3*	TSS1500
cg18499443	10	100,993,553	8.99E-05	0.264	0.279				*HPSE2*	Body
cg19883384	8	25,905,478	0.002522	0.264	0.226					
cg00834796	5	147,161,924	0.000294	0.264	0.219	0.0000353	0.078	Yes	*JAKMIP2*	5'UTR
cg02779592	X	99,899,134	0.000243	0.264	0.281				*SRPX2*	TSS200
cg19016332	1	179,545,294	9.21E-05	0.264	0.216				*NPHS2*	TSS1500
cg05571970	6	166,422,182	1.97E-06	0.264	0.226					
cg09400316	6	12,957,697	0.010568	0.264	0.244				*PHACTR1*	Body
cg09683350	15	89,914,466	0.000246	0.264	0.180					
cg02059509	10	30,405,144	3.24E-06	0.264	0.240					
cg03281561	3	159,942,181	0.001965	0.264	0.317				*LOC401097*	TSS1500
cg20291321	X	36,981,602	0.00195	0.264	0.335					
cg15962744	12	114,328,074	1.91E-11	0.264	0.255				*RBM19*	Body
cg17568707	6	137,818,327	0.000394	0.264	0.276					
cg04392209	X	119,124,215	0.013448	0.264	0.353					
cg06469542	14	77,965,869	1.24E-11	0.264	0.246				*ISM2*	TSS1500
cg26630231	13	112,726,482	0.011589	0.264	0.338					

cg00834923	16	85,393,998	3.51E-12	0.264	0.246					
cg05265607	10	98,900,817	5.76E-07	0.264	0.305			Yes	*SLIT1*	Body
cg26332310	1	91,182,305	0.000645	0.264	0.295				*BARHL2*	1stExon
cg09333785	4	8,202,491	0.000307	0.264	0.240				*SH3TC1*	5'UTR
cg20100910	10	102,475,429	0.034888	0.264	0.357					
cg23318812	12	66,123,127	0.028233	0.264	0.282					
cg03911745	8	49,486,641	2.71E-17	0.264	0.286					
cg21903817	17	56,406,118	2.51E-05	0.264	0.287				*BZRAP1*	1stExon
cg02238178	22	37,545,969	3.6E-05	0.264	0.211				*IL2RB*	TSS200
cg17178761	17	55,682,851	1.92E-05	0.264	0.205				*MSI2*	Body
cg05215004	6	6,546,556	0.005202	0.264	0.152				*LOC285780*	Body
cg13871633	3	42,947,385	0.011323	0.264	0.155				*ZNF662*	TSS1500
cg06204062	3	116,164,512	0.00017	0.264	0.210				*LSAMP*	TSS200
cg17969560	11	65,324,768	6.85E-18	0.264	0.234				*LTBP3*	Body
cg18309255	2	218,179,272	4.42E-10	0.264	0.220				*DIRC3*	Body
cg22578916	6	31,830,651	0.035959	0.264	0.238				*NEU1*	5'UTR
cg02855778	7	157,462,914	2.1E-15	0.264	0.234				*PTPRN2*	Body
cg04986899	16	17,553,784	1.41E-24	0.264	0.264				*XYLT1*	Body
cg17453456	1	156,094,519	2.33E-16	0.264	0.266				*LMNA*	Body
cg01601050	14	25,519,778	0.007848	0.264	0.216				*STXBP6*	TSS1500
cg17495715	2	176,950,273	2.69E-06	0.264	0.269					
cg04361551	3	134,125,573	0.044813	0.264	0.357					
cg24903893	5	158,522,515	6.73E-05	0.264	0.264				*EBF1*	Body
cg03224396	1	221,017,300	0.002473	0.264	0.266					
cg08838158	2	118,594,167	6.6E-36	0.264	0.247					
cg19240857	6	31,683,109	4.98E-20	0.264	0.257				*LY6G6E*	TSS1500
cg23610453	10	79,470,966	8.5E-08	0.264	0.162					
cg06204711	1	119,528,424	0.002318	0.264	0.340				*TBX15*	5'UTR
cg09778422	X	64,887,118	1.05E-07	0.264	0.189				*MSN*	TSS1500
cg13770461	11	46,016,312	1.27E-13	0.264	0.262				*PHF21A*	Body
cg15459780	12	104,609,980	1.26E-06	0.264	0.257				*TXNRD1*	Body
cg19339179	4	113,324,678	3.3E-24	0.264	0.249				*ALPK1*	Body
cg27638727	5	36,066,944	3.02E-06	0.264	0.209				*UGT3A2*	1stExon
cg02593205	12	26,348,343	0.020109	0.264	0.210				*SSPN*	5'UTR

cg18234115	1	245,673,856	7.66E-06	0.264	0.279				*KIF26B*	Body
cg13080487	11	34,622,359	8.2E-05	0.264	0.260					
cg22592160	5	124,995,555	8.84E-11	0.264	0.276					
cg22747746	8	23,564,193	5.17E-05	0.264	0.250				*NKX2-6*	TSS1500
cg21692666	1	17,199,302	7.65E-06	0.264	0.272					
cg00319774	8	33,455,184	0.000834	0.264	0.234				*DUSP26*	5'UTR
cg13114497	22	18,986,775	0.001354	0.264	0.275					
cg06085011	4	54,965,999	0.001439	0.264	0.270				*GSX2*	TSS1500
cg02892772	1	44,991,042	3.07E-24	0.264	0.244				*RNF220*	Body
cg13951311	9	139,499,374	6.14E-07	0.264	0.237					
cg19188306	11	111,410,935	4.73E-12	0.264	0.231				*LAYN*	TSS1500
cg22249789	2	63,284,574	9.31E-06	0.264	0.237					
cg14608318	19	17,392,429	0.019131	0.264	0.242				*ANKLE1*	TSS200
cg20377627	16	23,194,721	0.001121	0.264	0.176				*SCNN1G*	5'UTR
cg03161476	1	203,596,940	0.028312	0.264	0.360				*ATP2B4*	5'UTR
cg03704700	16	75,529,019	0.00615	0.264	0.223				*CHST6*	TSS200
cg01548300	5	10,333,766	0.0194	0.264	0.286					
cg02751731	20	642,436	0.000132	0.264	0.292				*SCRT2*	3'UTR
cg17839721	2	97,193,174	0.001822	0.264	0.350					
cg13802013	20	37,303,351	2.18E-08	0.264	0.203					
cg20036207	19	10,397,031	3.11E-12	0.264	0.253				*ICAM4*	TSS1500
cg15120085	1	32,041,898	4.73E-05	0.264	0.209				*TINAGL1*	TSS200
cg16726201	X	49,087,385	4.37E-05	0.264	0.255				*CACNA1F*	Body
cg20312012	2	97,331,035	3.45E-14	0.264	0.249				*FER1L5*	Body
cg06192497	2	63,276,216	0.024703	0.264	0.271				*LOC100132215*	TSS1500
cg02493798	17	6,899,577	7.32E-10	0.264	0.209				*ALOX12*	Body
cg12091944	2	215,675,105	0.000323	0.264	0.169				*BARD1*	TSS1500
cg12351587	4	85,405,251	6.81E-06	0.264	0.209					
cg04194947	2	177,012,191	8.23E-09	0.263	0.225					
cg10603004	8	23,564,294	0.00043	0.263	0.225				*NKX2-6*	TSS1500
cg15441392	8	25,900,020	1.96E-06	0.263	0.223				*EBF2*	Body
cg19046826	17	48,546,297	0.001281	0.263	0.228				*CHAD*	TSS200
cg02155658	20	21,690,036	3.56E-06	0.263	0.282				*PAX1*	Body
cg03163783	2	200,326,591	2.94E-07	0.263	0.250				*SATB2*	TSS1500

cg05781984	6	43,237,642	2.22E-09	0.263	0.256				*TTBK1*	Body
cg02681370	6	32,156,482	0.001087	0.263	0.253				*PBX2*	Body
cg16764274	2	223,290,168	3.35E-14	0.263	0.246				*SGPP2*	Body
cg24164254	9	137,329,341	1.51E-10	0.263	0.256				*RXRA*	3'UTR
cg08871052	2	42,274,619	0.042657	0.263	0.300				*PKDCC*	TSS1500
cg04544858	5	67,489,737	1.38E-08	0.263	0.297					
cg27324426	19	58,220,837	8.3E-05	0.263	0.232				*ZNF154*	TSS1500
cg04605287	1	54,953,486	0.002889	0.263	0.189					
cg15540820	3	27,765,283	0.002252	0.263	0.231	3.38E-10	0.168		*EOMES*	TSS1500
cg24559969	X	111,326,800	3.72E-05	0.263	0.270				*TRPC5*	TSS1500
cg03671597	17	27,333,463	0.000105	0.263	0.282				*SEZ6*	TSS1500
cg13050240	14	24,809,068	3.76E-06	0.263	0.227				*RIPK3*	5'UTR
cg02134353	6	108,440,118	0.001726	0.263	0.274					
cg14429204	1	16,553,327	9.69E-07	0.263	0.231					
cg15644764	1	202,592,914	7.64E-13	0.263	0.261				*SYT2*	5'UTR
cg17920789	13	47,152,525	8.5E-07	0.263	0.261				*LRCH1*	Body
cg01243371	16	86,547,386	0.002258	0.263	0.268				*FOXF1*	3'UTR
cg09502015	5	87,441,873	3.27E-08	0.263	0.263					
cg07448742	6	30,657,984	4.73E-25	0.263	0.255				*NRM*	Body
cg08770870	17	154,410	0.002768	0.263	0.211				*RPH3AL*	Body
cg10588962	17	46,667,587	5.8E-12	0.263	0.246				*LOC404266*	TSS1500
cg14515614	9	116,969,440	6.1E-11	0.263	0.286				*COL27A1*	Body
cg23827488	6	24,908,452	6.07E-05	0.263	0.200				*FAM65B*	5'UTR
cg26056498	8	1,030,319	0.04863	0.263	0.232					
cg01097384	11	1,358,675	4.96E-07	0.263	0.290					
cg01423335	13	93,881,110	0.000627	0.263	0.189				*GPC6*	Body
cg04927537	17	76,976,091	0.001579	0.263	0.215				*LGALS3BP*	TSS200
cg07931411	19	15,580,721	0.036071	0.263	0.359				*PGLYRP2*	Body
cg10189661	6	31,683,120	1.25E-16	0.263	0.241				*LY6G6E*	TSS1500
cg20600729	12	50,344,475	2.44E-16	0.263	0.244				*AQP2*	TSS200
cg21209395	9	35,406,764	1.51E-05	0.263	0.183				*LOC158381*	Body
cg11674130	22	50,523,689	6E-11	0.263	0.252				*MLC1*	5'UTR
cg18164611	1	46,958,628	4.38E-07	0.263	0.269					
cg09457469	17	7,197,507	0.021535	0.263	0.133				*YBX2*	Body

cg19403377	17	53,800,225	0.004705	0.263	0.284				*TMEM100*	TSS200
cg15126975	2	175,208,605	0.018988	0.263	0.292					
cg03352428	19	51,843,601	1.75E-08	0.263	0.243				*VSIG10L*	Body
cg07595602	1	240,783,371	0.001192	0.263	0.184					
cg20956278	22	38,478,457	3.95E-06	0.263	0.278				*SLC16A8*	Body
cg07821354	19	6,768,010	0.004605	0.263	0.157				*SH2D3A*	TSS1500
cg26783057	10	118,891,670	0.022621	0.263	0.314				*VAX1*	3'UTR
cg15982700	7	27,154,911	3.52E-09	0.263	0.248				*HOXA3*	5'UTR
cg12304520	5	140,810,123	0.002791	0.263	0.303				*PCDHGA4*	Body
cg01397141	15	79,502,137	0.001665	0.263	0.268				*MIR184*	Body
cg01856645	5	78,365,647	3.35E-09	0.263	0.209				*DMGDH*	TSS200
cg01883425	6	41,606,770	0.009255	0.263	0.184				*MDFI*	Body
cg03790740	13	24,337,521	3.03E-16	0.263	0.257				*MIPEP*	Body
cg18741958	3	124,913,784	1.05E-10	0.263	0.216				*SLC12A8*	Body
cg16793187	11	115,530,431	8.25E-05	0.263	0.221					
cg07642638	20	5,891,786	2.31E-06	0.263	0.193	3.9E-23	0.147		*CHGB*	TSS200
cg15792653	10	118,924,342	4.83E-05	0.263	0.281					
cg08034379	2	228,736,324	0.004495	0.263	0.299				*WDR69*	TSS200
cg02381279	19	16,394,366	5.21E-09	0.263	0.266					
cg22321572	6	168,225,923	9.06E-10	0.263	0.207				*C6orf124*	Body
cg25296103	2	145,268,533	2.69E-08	0.263	0.253				*ZEB2*	Body
cg19762657	2	95,692,524	7.32E-06	0.263	0.232				*MAL*	Body
cg07152196	2	87,016,436	0.000362	0.263	0.298				*CD8A*	Body
cg00727673	3	53,190,763	2.22E-11	0.263	0.240					
cg16573755	2	172,958,337	0.002472	0.263	0.235					
cg05294159	17	43,047,548	1.13E-13	0.263	0.262					
cg18049571	8	54,791,151	5.94E-07	0.263	0.225				*RGS20*	Body
cg05429448	3	101,659,630	6.37E-10	0.263	0.268				*LOC152225*	TSS200
cg06482188	17	77,644,545	1.74E-16	0.263	0.260					
cg17264618	3	40,429,014	0.000641	0.263	0.212	1.15E-18	0.201		*ENTPD3*	5'UTR
cg22502502	6	25,962,567	0.000509	0.263	0.259	0.0002059	0.119		*TRIM38*	TSS1500
cg06559274	6	108,487,903	0.001945	0.263	0.330				*NR2E1*	5'UTR
cg04255230	2	74,727,010	8.34E-22	0.263	0.258				*LBX2*	Body
cg05111603	4	38,213,171	2.92E-10	0.263	0.289					

cg14171486	1	155,139,557	2.02E-06	0.263	0.292						
cg16536399	18	77,230,665	2.01E-14	0.263	0.262				*NFATC1*	Body	
cg18044383	18	59,001,158	9.83E-05	0.263	0.266						
cg24727399	4	122,302,265	0.029576	0.263	0.267				*QRFPR*	TSS200	
cg04947764	15	79,502,127	0.002468	0.263	0.276				*MIR184*	TSS200	
cg12059864	6	111,989,790	7.32E-17	0.263	0.272				*FYN*	Body	
cg12924309	12	125,105,776	9.78E-20	0.263	0.249						
cg03461110	7	4,778,881	4.48E-15	0.263	0.246				*FOXK1*	Body	
cg13532885	X	47,478,770	0.007034	0.263	0.181				*SYN1*	1stExon	
cg13469595	1	111,813,852	6.06E-06	0.263	0.294						
cg04724735	19	7,927,554	0.003625	0.263	0.261				*EVI5L*	Body	
cg06840491	10	48,416,966	1.28E-16	0.263	0.250				*GDF2*	TSS200	
cg19443023	17	78,923,344	2.76E-65	0.263	0.262				*RPTOR*	Body	
cg02571142	8	42,234,803	3.86E-19	0.263	0.256				*DKK4*	TSS200	
cg03202564	10	50,977,130	4.7E-06	0.263	0.270						
cg23697796	10	93,334,974	2.96E-09	0.263	0.257				*LOC100188947*	Body	
cg19377250	7	100,463,206	6.05E-19	0.263	0.237				*SLC12A9*	Body	
cg19971716	4	122,301,740	0.011902	0.263	0.239				*QRFPR*	1stExon	
cg01796166	14	95,239,586	0.000252	0.263	0.268						
cg24559983	21	38,592,988	0.009212	0.263	0.132						
cg03366986	17	59,530,021	2.79E-06	0.263	0.254						
cg05986505	8	99,955,563	5.7E-12	0.263	0.260				*OSR2*	TSS1500	
cg04642923	11	33,398,366	0.007176	0.263	0.204						
cg19691778	8	97,157,756	0.001502	0.263	0.220				*GDF6*	Body	
cg08983097	6	117,586,538	0.000294	0.263	0.293				*VGLL2*	TSS200	
cg19559891	15	31,515,984	6.09E-06	0.263	0.287						
cg23082339	5	140,810,051	0.027361	0.263	0.335				*PCDHGA4*	Body	
cg04421553	3	38,036,060	0.021393	0.263	0.107				*VILL*	Body	
cg00093087	12	50,344,548	1.23E-43	0.263	0.240				*AQP2*	5'UTR	
cg17298239	18	3,499,253	0.046858	0.263	0.165				*DLGAP1*	Body	
cg04003850	3	192,126,165	0.026284	0.263	0.242				*FGF12*	5'UTR	
cg15351951	18	2,907,472	7.76E-07	0.263	0.245				*EMILIN2*	Body	
cg15826810	4	124,572,578	0.000283	0.263	0.174				*LOC285419*	TSS1500	
cg27295642	5	74,952,444	0.00278	0.263	0.174						

cg23520422	12	76,423,956	0.007249	0.263	0.113				*PHLDA1*	3'UTR
cg24336989	6	10,385,903	0.003261	0.263	0.286					
cg25429719	2	208,989,324	9.06E-07	0.263	0.273				*CRYGD*	TSS200
cg19592235	15	65,360,486	0.016453	0.263	0.168				*RASL12*	TSS200
cg02923856	3	23,989,124	7.57E-16	0.263	0.275				*NR1D2*	5'UTR
cg10390305	15	70,878,049	4.38E-05	0.263	0.196					
cg06746118	10	118,922,887	0.028128	0.263	0.279					
cg02177231	1	119,529,930	0.004221	0.263	0.182				*TBX15*	5'UTR
cg08198377	14	51,707,975	8.26E-06	0.263	0.227				*TMX1*	Body
cg08762432	10	21,358,991	3.68E-08	0.263	0.268				*NEBL*	Body
cg03152187	17	75,417,165	0.005665	0.263	0.152				*SEPT9*	Body
cg23728669	11	63,258,734	0.045683	0.263	0.228				*HRASLS5*	TSS200
cg04118124	1	43,231,249	2.72E-05	0.263	0.143				*LEPRE1*	Body
cg06478085	19	33,876,779	2.15E-14	0.263	0.268					
cg08034070	17	40,805,868	0.049352	0.263	0.131					
cg14269973	13	46,425,904	0.001928	0.263	0.235				*SIAH3*	TSS200
cg00768359	19	51,456,308	0.004039	0.263	0.275				*KLK5*	5'UTR
cg05301470	6	28,558,006	1.04E-05	0.263	0.266					
cg19480198	19	18,896,853	1.68E-08	0.263	0.217				*COMP*	Body
cg21032564	15	65,381,956	1.92E-17	0.263	0.243					
cg12995113	11	123,008,408	1.92E-05	0.263	0.157				*ASAM*	Body
cg08775137	4	41,880,319	7.93E-11	0.262	0.262					
cg26470798	16	54,971,598	3.33E-06	0.262	0.264					
cg01850783	6	129,204,540	0.011301	0.262	0.320				*LAMA2*	Body
cg17634781	4	186,508,880	5.58E-10	0.262	0.269				*SORBS2*	Body
cg25451303	19	30,720,025	0.001583	0.262	0.244					
cg06152533	3	3,840,333	0.037266	0.262	0.059				*LRRN1*	TSS1500
cg10199763	14	24,458,299	2.54E-08	0.262	0.250				*DHRS4L2*	Body
cg15159104	15	43,809,865	8.35E-14	0.262	0.243				*MAP1A*	5'UTR
cg17820591	17	4,853,706	0.015626	0.262	0.204	8.44E-19	0.157		*ENO3*	TSS1500
cg21267231	15	45,406,706	2.84E-06	0.262	0.236				*DUOXA2*	1stExon
cg16624888	12	109,711,141	3.89E-11	0.262	0.268					
cg00767581	2	177,015,125	2.6E-08	0.262	0.211	0.0000543	0.072		*HOXD4*	TSS1500
cg10589443	7	30,028,281	0.016114	0.262	0.169				*SCRN1*	5'UTR

cg10754215	4	8,073,804	0.016033	0.262	0.215				*ABLIM2*	Body
cg12441066	17	55,456,535	1.43E-13	0.262	0.239				*MSI2*	Body
cg07522516	4	48,492,196	0.001871	0.262	0.295				*ZAR1*	TSS200
cg08774871	16	85,783,575	3.2E-13	0.262	0.229				*C16orf74*	5'UTR
cg15415452	13	112,759,355	0.021333	0.262	0.306					
cg26253898	2	163,006,202	1.6E-09	0.262	0.197				*GCG*	5'UTR
cg01062395	6	29,855,636	0.005724	0.262	0.230				*HLA-H*	Body
cg17640147	8	142,302,237	3.36E-22	0.262	0.263					
cg02855833	8	12,293,509	0.010032	0.262	0.179				*FAM86B2*	Body
cg11145461	1	201,475,451	3.83E-05	0.262	0.276				*CSRP1*	5'UTR
cg11213369	7	1,270,925	1.41E-14	0.262	0.257					
cg14659346	2	233,246,431	0.017341	0.262	0.220				*ALPP*	Body
cg25807487	11	68,782,106	4.53E-09	0.262	0.271				*MRGPRF*	TSS1500
cg15916192	12	63,025,948	0.018553	0.262	0.215					
cg14852394	2	67,116,039	5.7E-12	0.262	0.244					
cg25979543	1	17,888,104	6.47E-20	0.262	0.258				*ARHGEF10L*	5'UTR
cg24303731	7	14,942,359	0.003121	0.262	0.322					
cg10975889	1	221,057,808	4.81E-11	0.262	0.236				*HLX*	Body
cg03719509	5	77,945,178	0.031512	0.262	0.188				*LHFPL2*	TSS1500
cg21619653	2	45,163,188	0.004679	0.262	0.362					
cg16849024	19	41,934,210	6.33E-76	0.262	0.254				*B3GNT8*	5'UTR
cg18415686	14	36,005,164	2.24E-14	0.262	0.243				*INSM2*	3'UTR
cg25302087	6	21,668,049	7.37E-10	0.262	0.294				*FLJ22536*	Body
cg04233193	16	1,340,362	1.28E-06	0.262	0.244					
cg01807688	X	133,680,740	0.020433	0.262	0.242				*MGC16121*	TSS200
cg16220788	11	46,005,268	9.4E-21	0.262	0.259				*PHF21A*	Body
cg19580937	15	70,740,429	1.09E-15	0.262	0.245					
cg18500988	4	54,959,708	1.76E-12	0.262	0.238					
cg02400740	7	19,157,938	0.012836	0.262	0.254				*TWIST1*	TSS1500
cg05822633	20	11,898,531	0.022507	0.262	0.069				*BTBD3*	5'UTR
cg08594554	17	72,231,318	5.57E-09	0.262	0.254				*TTYH2*	Body
cg11504517	1	3,569,899	6.21E-10	0.262	0.245				*TP73*	5'UTR
cg02508651	17	46,604,554	2.25E-05	0.262	0.214					
cg02320740	10	106,400,259	0.03822	0.262	0.194				*SORCS3*	TSS1500

cg26940479	16	85,201,840	1.01E-05	0.262	0.205					
cg05638493	5	140,683,367	3.15E-05	0.262	0.233				*SLC25A2*	1stExon
cg02892388	7	155,260,310	0.000653	0.262	0.255					
cg09671955	1	169,429,972	4.58E-10	0.262	0.267					
cg20652899	11	117,747,940	4.6E-17	0.262	0.253				*FXYD6*	5'UTR
cg20664636	17	1,959,596	1.22E-07	0.262	0.257				*HIC1*	TSS200
cg20019985	11	32,008,961	0.00171	0.262	0.310					
cg11668419	1	68,025,026	2.87E-06	0.262	0.250					
cg18926409	15	65,174,571	1.32E-17	0.262	0.251					
cg02473781	22	22,007,095	1.46E-33	0.262	0.238				*MIR130B*	TSS1500
cg20849109	8	1,954,777	5.27E-06	0.262	0.283				*KBTBD11*	3'UTR
cg14846965	18	13,611,481	2.99E-05	0.262	0.241				*C18orf1*	TSS200
cg27191312	19	12,750,958	0.004851	0.262	0.229					
cg05961809	4	154,710,523	0.003131	0.262	0.340				*SFRP2*	TSS1500
cg15608939	17	8,907,074	0.000274	0.262	0.236					
cg16073143	6	159,360,073	2.25E-07	0.262	0.269					
cg21011139	11	7,695,245	1.16E-07	0.262	0.234				*CYB5R2*	TSS1500
cg19942459	11	70,508,110	1.41E-06	0.262	0.242				*SHANK2*	Body
cg22745354	2	928,720	7.24E-19	0.262	0.240					
cg20980783	8	67,874,178	0.002331	0.262	0.155					
cg11159604	1	91,316,223	2.42E-05	0.262	0.221					
cg16239482	3	181,437,145	0.034991	0.262	0.330				*SOX2OT*	Body
cg02751808	2	71,125,521	4.41E-05	0.262	0.290					
cg08128274	1	206,832,043	6.14E-05	0.262	0.262					
cg11746813	12	54,448,090	3.83E-07	0.262	0.245				*HOXC4*	1stExon
cg26053073	8	131,455,383	0.01909	0.262	0.133					
cg15454486	2	192,543,810	0.032971	0.262	0.162				*OBFC2A*	Body
cg20939723	2	171,570,700	7.34E-05	0.262	0.213				*LOC440925*	Body
cg22894896	17	29,886,890	0.001702	0.262	0.323				*MIR193A*	TSS200
cg04099705	16	1,397,981	4.5E-08	0.262	0.254				*BAIAP3*	Body
cg06215569	1	110,611,465	0.001916	0.262	0.327				*ALX3*	Body
cg26595643	10	118,899,291	7.26E-06	0.262	0.238				*VAX1*	TSS1500
cg09569850	2	64,955,034	2.33E-09	0.262	0.304					
cg13537237	2	241,908,681	2.41E-08	0.262	0.247					

cg16604035	1	27,863,299	3.98E-12	0.262	0.234				*AHDC1*	3'UTR
cg19599386	2	113,420,964	3.17E-09	0.262	0.272				*SLC20A1*	3'UTR
cg01876531	1	208,405,868	3.16E-07	0.262	0.261				*PLXNA2*	5'UTR
cg09581649	16	4,572,735	2.33E-22	0.262	0.251				*C16orf5*	5'UTR
cg25839877	17	76,361,930	6.2E-13	0.262	0.257					
cg17059658	18	905,101	0.019391	0.262	0.275				*ADCYAP1*	5'UTR
cg14353137	8	67,873,799	0.039238	0.262	0.187					
cg03075709	5	2,755,820	0.003895	0.262	0.299					
cg25947408	5	133,451,383	9.69E-16	0.262	0.237				*TCF7*	5'UTR
cg13510327	6	31,830,729	0.047206	0.262	0.244				*NEU1*	TSS200
cg10635122	19	52,391,090	0.011424	0.262	0.258				*ZNF577*	Body
cg15746187	1	11,713,123	1.44E-09	0.262	0.240				*FBXO2*	Body
cg20378690	8	22,723,551	6.09E-08	0.262	0.196			Yes	*PEBP4*	Body
cg18417373	6	32,077,097	8.39E-06	0.262	0.233				*TNXB*	5'UTR
cg20406267	11	34,377,921	0.0049	0.262	0.219				*ABTB2*	Body
cg04363624	1	47,697,965	0.000138	0.262	0.246					
cg24343310	11	57,249,947	2.86E-23	0.262	0.272					
cg03989068	2	42,106,106	2.76E-13	0.262	0.238					
cg22488367	22	45,608,448	0.003236	0.262	0.269				*C22orf9*	Body
cg10880485	14	60,974,384	3.18E-06	0.262	0.238					
cg27154163	4	55,523,756	2.49E-05	0.262	0.174	8.67E-22	0.196		*KIT*	TSS1500
cg06879222	16	85,730,838	8.73E-08	0.262	0.224					
cg01996567	7	149,484,880	0.002242	0.262	0.215				*SSPO*	Body
cg08045906	6	32,120,625	9.14E-05	0.262	0.287				*PPT2*	TSS1500
cg12061069	17	2,755,793	6.98E-12	0.262	0.237				*RAP1GAP2*	Body
cg16852440	2	237,570,117	3.64E-08	0.262	0.247					
cg01467266	19	2,252,961	7.3E-16	0.261	0.271				*JSRP1*	Body
cg11697427	10	102,590,168	1.32E-06	0.261	0.275					
cg26142412	11	20,185,077	0.036339	0.261	0.282					
cg14595252	18	9,709,061	9.37E-06	0.261	0.164				*RAB31*	Body
cg16561893	6	85,483,536	0.003991	0.261	0.253					
cg00076538	5	38,257,555	7.98E-11	0.261	0.250				*EGFLAM*	TSS1500
cg22861561	12	48,397,730	0.001398	0.261	0.166				*COL2A1*	Body
cg24114154	17	46,655,829	0.00771	0.261	0.252				*HOXB4*	TSS200

cg20064106	2	178,257,871	0.001862	0.261	0.177				*LOC100130691*	TSS1500
cg03761750	12	6,184,355	1.54E-06	0.261	0.262				*VWF*	Body
cg08510658	6	10,882,927	5.75E-05	0.261	0.277				*GCM2*	TSS1500
cg08641579	7	79,083,447	0.000647	0.261	0.190				*MAGI2*	TSS1500
cg18390345	10	98,939,912	2.69E-16	0.261	0.252				*SLIT1*	Body
cg00332680	10	74,079,303	0.027918	0.261	0.187					
cg25158320	17	1,665,262	3.08E-08	0.261	0.279				*SERPINF1*	1stExon
cg04163638	2	16,832,247	8.81E-07	0.261	0.277				*FAM49A*	5'UTR
cg02337898	12	93,578,655	2.38E-09	0.261	0.242					
cg04036070	16	53,957,219	1.66E-06	0.261	0.268				*FTO*	Body
cg08555612	3	71,834,640	0.000192	0.261	0.210	2.97E-25	0.226		*PROK2*	TSS1500
cg10366178	3	13,323,866	1.45E-06	0.261	0.265					
cg26334154	3	42,132,310	5.03E-10	0.261	0.195				*TRAK1*	TSS1500
cg08218734	10	128,993,810	0.017714	0.261	0.178				*FAM196A*	5'UTR
cg08275025	11	314,493	0.000681	0.261	0.128				*IFITM1*	Body
cg08799997	6	167,737,937	5.63E-10	0.261	0.240				*TTLL2*	TSS1500
cg19805748	17	71,003,041	3.06E-18	0.261	0.258				*SLC39A11*	Body
cg19120717	6	41,343,647	1.77E-05	0.261	0.299					
cg27541454	1	975,551	1.08E-06	0.261	0.253				*AGRN*	Body
cg13580029	7	123,569,748	8.96E-12	0.261	0.236			Yes	*SPAM1*	5'UTR
cg15603568	11	105,481,283	0.009396	0.261	0.262				*GRIA4*	5'UTR
cg06636195	2	109,533,679	0.016547	0.261	0.250				*EDAR*	Body
cg07846167	1	16,084,758	4.65E-05	0.261	0.295				*FBLIM1*	TSS1500
cg21082028	17	46,659,993	2.22E-06	0.261	0.234					
cg05224998	17	33,700,801	0.038367	0.261	0.362				*SLFN11*	TSS200
cg01709518	3	68,053,484	0.002118	0.261	0.338				*FAM19A1*	5'UTR
cg03972560	X	17,877,110	2.23E-11	0.261	0.272				*RAI2*	5'UTR
cg21821214	2	176,969,223	0.001081	0.261	0.293					
cg19885926	X	71,350,942	1.75E-08	0.261	0.249				*NHSL2*	Body
cg20605818	10	72,166,322	3.74E-12	0.261	0.241				*EIF4EBP2*	Body
cg10263682	3	181,413,847	0.028081	0.261	0.261				*SOX2OT*	Body
cg02711872	7	150,652,702	1.88E-17	0.261	0.270				*KCNH2*	5'UTR
cg08748913	5	137,071,788	4.04E-07	0.261	0.126				*KLHL3*	TSS200
cg22705959	2	208,635,887	7.07E-09	0.261	0.257					

cg23261919	10	135,072,960	5.64E-08	0.261	0.239					
cg04848693	7	2,078,786	3.57E-19	0.261	0.214				*MAD1L1*	Body
cg01619345	9	4,297,433	0.00051	0.261	0.203				*GLIS3*	5'UTR
cg02078525	16	66,400,395	3.16E-07	0.261	0.201				*CDH5*	TSS200
cg00157796	6	159,590,231	0.040363	0.261	0.199				*FNDC1*	TSS200
cg04792863	1	110,627,197	4.16E-06	0.261	0.246					
cg08566455	2	130,971,164	0.040803	0.261	0.212					
cg09437808	5	176,107,069	1.26E-07	0.261	0.311					
cg23334662	2	198,540,518	7.85E-11	0.261	0.271				*RFTN2*	1stExon
cg05301383	2	171,569,144	1.01E-05	0.261	0.245				*LOC440925*	Body
cg23588462	13	95,357,223	2.5E-06	0.261	0.260					
cg27221193	20	62,466,924	1.71E-17	0.261	0.265					
cg07298473	11	47,279,183	1.39E-05	0.261	0.221				*NR1H3*	5'UTR
cg24409539	2	114,034,595	1.23E-06	0.261	0.299				*PAX8*	Body
cg09775263	6	10,884,094	7.2E-05	0.261	0.248					
cg10772304	3	179,245,127	3.66E-07	0.261	0.290					
cg24049493	1	42,385,941	6.53E-06	0.261	0.251				*HIVEP3*	TSS1500
cg12118013	3	134,031,948	6.1E-08	0.261	0.249					
cg15270892	3	27,762,352	0.002055	0.261	0.089				*EOMES*	Body
cg03109913	1	94,445,292	1.2E-06	0.261	0.213					
cg04486382	17	55,483,894	3.88E-06	0.261	0.222				*MSI2*	Body
cg06401021	6	55,443,868	0.027554	0.261	0.295				*HMGCLL1*	1stExon
cg02881929	4	41,882,868	4.14E-07	0.261	0.238					
cg08359956	7	150,498,205	1.13E-08	0.261	0.265	2.86E-36	0.252		*TMEM176B*	1stExon
cg02624701	19	49,937,176	0.021811	0.261	0.298				*SLC17A7*	Body
cg07451524	10	123,923,518	3.77E-10	0.261	0.252				*TACC2*	Body
cg25101405	1	1,398,028	5.71E-23	0.261	0.261				*ATAD3C*	Body
cg05966228	16	67,666,442	2.13E-11	0.261	0.230				*CTCF*	Body
cg10619129	6	33,085,755	4.39E-06	0.261	0.259				*HLA-DPB2*	Body
cg14254720	1	90,097,981	0.008388	0.261	0.224				*LRRC8C*	TSS1500
cg01257171	5	172,656,713	0.001214	0.261	0.359					
cg24421668	5	179,244,452	0.00019	0.261	0.201				*SQSTM1*	5'UTR
cg26606327	7	98,972,104	0.004866	0.261	0.192				*ARPC1B*	TSS1500
cg14315444	17	48,636,344	4E-07	0.261	0.257					

cg15837212	1	63,795,970	2.28E-06	0.261	0.272					
cg06277657	7	137,532,374	1.83E-05	0.261	0.223	3.4E-22	0.253		*DGKI*	TSS1500
cg25053018	2	173,477,995	1.44E-10	0.261	0.243					
cg27173151	1	203,598,330	0.000167	0.261	0.258				*ATP2B4*	5'UTR
cg08763617	6	138,183,963	1.51E-06	0.261	0.235					
cg20186067	7	129,422,439	1.83E-05	0.261	0.264					
cg25734421	1	210,501,368	6.61E-14	0.261	0.273				*HHAT*	TSS1500
cg21282282	1	109,705,170	1.15E-12	0.261	0.246				*KIAA1324*	Body
cg02707176	5	140,798,758	0.000184	0.261	0.304				*PCDHGA4*	Body
cg17021218	10	88,731,069	0.019223	0.261	0.224				*AGAP11*	5'UTR
cg23740491	6	27,228,267	0.000376	0.261	0.298					
cg00421624	1	153,746,588	2.3E-08	0.261	0.275				*SLC27A3*	TSS1500
cg11741189	11	31,843,886	0.000213	0.261	0.253					
cg18089397	4	2,264,747	0.006583	0.261	0.177				*MXD4*	TSS1500
cg21366688	6	134,491,421	1.03E-13	0.261	0.271				*SGK1*	Body
cg24537836	12	51,782,159	1.28E-09	0.261	0.277				*GALNT6*	5'UTR
cg02844545	6	10,882,043	4.25E-11	0.261	0.235	4.37E-19	0.212		*GCM2*	1stExon
cg03305185	X	134,429,653	3.97E-05	0.261	0.233				*ZNF75D*	5'UTR
cg16055869	4	74,864,612	6.22E-07	0.261	0.249				*CXCL5*	TSS200
cg05157360	13	113,613,660	0.004378	0.261	0.174					
cg09321400	16	55,690,443	0.000704	0.261	0.203				*SLC6A2*	TSS200
cg17125267	4	115,520,931	3.03E-05	0.261	0.281				*UGT8*	5'UTR
cg26827247	11	93,063,208	0.003096	0.261	0.167				*CCDC67*	TSS1500
cg04086468	3	185,911,208	9.41E-06	0.261	0.207				*DGKG*	Body
cg22292345	3	47,051,533	2.98E-14	0.261	0.232				*LOC100129354*	TSS1500
cg25965471	6	43,211,235	0.005105	0.261	0.263				*TTBK1*	5'UTR
cg25579180	7	71,098,623	0.007536	0.261	0.245				*WBSCR17*	Body
cg13173111	17	66,255,265	0.000835	0.261	0.272				*ARSG*	TSS200
cg03730533	7	26,897,685	7.22E-06	0.261	0.273				*SKAP2*	Body
cg22659049	4	41,646,672	1.07E-15	0.261	0.264				*LIMCH1*	Body
cg03688699	11	62,694,712	3.05E-09	0.261	0.262					
cg22799132	X	111,325,495	6.21E-06	0.261	0.262	3.7E-14	0.191		*TRPC5*	5'UTR
cg02274869	19	11,784,955	3.91E-06	0.261	0.206				*ZNF833*	Body
cg09481483	16	11,734,383	5.65E-08	0.261	0.281					

cg18678645	5	135,416,331	5.76E-05	0.261	0.217				*MIR886*	TSS200
cg03184366	X	9,984,977	0.005788	0.261	0.286				*WWC3*	5'UTR
cg18944451	2	177,023,716	0.005768	0.261	0.283					
cg02487331	1	146,550,467	7.45E-09	0.261	0.285					
cg26147098	8	145,103,393	8.86E-06	0.261	0.266					
cg12752627	10	94,827,528	9.71E-17	0.261	0.257				*CYP26C1*	Body
cg24435924	19	12,978,255	0.00096	0.261	0.281				*MAST1*	Body
cg00783104	8	41,655,894	0.000149	0.261	0.278				*ANK1*	TSS1500
cg05732750	7	6,704,045	7.5E-14	0.261	0.229					
cg21253721	17	1,202,092	0.002371	0.261	0.267				*TUSC5*	3'UTR
cg06390536	17	73,584,068	0.001538	0.261	0.213				*MYO15B*	TSS200
cg26874367	2	129,381,792	6.09E-09	0.261	0.262					
cg11255159	19	18,401,021	1.86E-06	0.261	0.264					
cg15898874	15	96,515,411	2.75E-08	0.261	0.246					
cg15374686	1	110,694,263	3.1E-12	0.260	0.246				*SLC6A17*	5'UTR
cg22402007	9	87,282,823	0.002678	0.260	0.138	3.29E-16	0.133		*NTRK2*	TSS1500
cg27214960	10	135,343,280	0.007184	0.260	0.190				*CYP2E1*	Body
cg00543976	1	112,058,750	2.2E-08	0.260	0.234				*ADORA3*	Body
cg02839824	X	119,133,091	0.021045	0.260	0.268					
cg19641804	12	54,653,065	8.08E-17	0.260	0.253				*CBX5*	1stExon
cg03116258	7	27,135,214	4.98E-14	0.260	0.226				*HOXA1*	1stExon
cg06170215	X	71,525,152	0.001798	0.260	0.161				*CITED1*	5'UTR
cg13922451	6	28,557,542	0.001685	0.260	0.222					
cg14722140	6	49,518,627	0.007922	0.260	0.244				*C6orf141*	1stExon
cg22709192	12	54,366,909	0.000307	0.260	0.240	2.83E-17	0.196		*HOXC11*	5'UTR
cg11752430	10	79,273,606	9.34E-15	0.260	0.256				*KCNMA1*	Body
cg01275038	4	7,287,492	0.001851	0.260	0.260				*SORCS2*	Body
cg06204126	9	139,890,188	5.27E-21	0.260	0.238				*CLIC3*	Body
cg15812322	11	45,868,398	1.9E-08	0.260	0.189				*CRY2*	TSS1500
cg25554917	2	170,220,129	0.016586	0.260	0.253				*LRP2*	TSS1500
cg02351655	16	4,442,918	1.29E-15	0.260	0.266				*CORO7*	Body
cg02428792	4	25,090,597	0.003034	0.260	0.205					
cg06395298	17	46,651,225	0.002116	0.260	0.227				*HOXB3*	5'UTR
cg15840462	1	969,744	2.08E-11	0.260	0.264				*AGRN*	Body

cg15772924	12	54,413,384	2.69E-09	0.260	0.242				*HOXC4*	5'UTR	
cg17657037	19	3,933,856	0.003663	0.260	0.367				*ITGB1BP3*	Body	
cg25171348	7	28,448,200	6.53E-09	0.260	0.233				*CREB5*	5'UTR	
cg04821993	7	73,002,468	1.04E-06	0.260	0.221						
cg11729934	6	1,601,693	1.54E-06	0.260	0.202						
cg14643586	9	95,569,618	9.49E-06	0.260	0.234						
cg26354128	14	93,897,195	0.012213	0.260	0.243				*KIAA1409*	5'UTR	
cg06680397	5	135,527,376	1.21E-07	0.260	0.184				*LOC389332*	Body	
cg08669096	11	121,229,765	1.03E-05	0.260	0.248						
cg09249588	12	58,986,386	5.52E-10	0.260	0.283						
cg00447970	17	29,719,431	1.02E-09	0.260	0.245				*RAB11FIP4*	Body	
cg06678678	3	55,523,099	0.00754	0.260	0.190						
cg17857974	5	140,800,474	8.78E-05	0.260	0.299				*PCDHGA4*	Body	
cg08021564	10	73,847,318	5.62E-06	0.260	0.282				*SPOCK2*	Body	
cg18566594	12	4,381,435	7.03E-06	0.260	0.256				*CCND2*	TSS1500	
cg10806146	8	42,356,871	2.35E-11	0.260	0.260				*SLC20A2*	5'UTR	
cg03036592	10	101,287,839	0.002291	0.260	0.297						
cg02483661	1	2,460,621	1.77E-06	0.260	0.270				*HES5*	3'UTR	
cg15887459	11	18,230,903	1.22E-07	0.260	0.279				*LOC494141*	Body	
cg17737014	X	70,323,912	1.13E-06	0.260	0.284				*CXorf65*	Body	
cg25356719	10	1,638,893	1.52E-05	0.260	0.202				*ADARB2*	Body	
cg17846334	20	49,639,134	0.010439	0.260	0.151				*KCNG1*	5'UTR	
cg06620896	15	37,395,115	0.049596	0.260	0.322						
cg02888131	15	58,357,987	0.000612	0.260	0.295				*ALDH1A2*	TSS200	
cg06974930	1	46,633,153	1.82E-10	0.260	0.252						
cg19866866	1	182,026,044	1.86E-07	0.260	0.272				*ZNF648*	Body	
cg12483476	5	178,421,711	0.031512	0.260	0.184				*GRM6*	1stExon	
cg09827833	9	27,109,492	5.58E-06	0.260	0.272	0.00036595	0.093		*TEK*	5'UTR	
cg12378187	3	62,355,443	2.29E-05	0.260	0.269				*FEZF2*	3'UTR	
cg22857085	4	85,403,223	9.72E-05	0.260	0.289						
cg09288894	19	12,894,309	0.000224	0.260	0.177						
cg21748207	2	26,395,359	9.13E-05	0.260	0.158				*FAM59B*	TSS1500	
cg18302606	19	39,904,402	0.012471	0.260	0.084				*PLEKHG2*	5'UTR	
cg05021896	14	51,562,526	0.000341	0.260	0.174				*TRIM9*	TSS200	

cg07642259	X	43,833,130	0.003697	0.260	0.284				*NDP*	TSS1500
cg11838224	17	7,287,740	0.000883	0.260	0.235				*TNK1*	Body
cg01783662	3	181,442,676	1.86E-05	0.260	0.250				*SOX2OT*	Body
cg26153410	12	103,358,619	0.000241	0.260	0.181					
cg27554782	15	78,933,106	5.77E-08	0.260	0.196	6.85E-37	0.250		*CHRNB4*	Body
cg08835221	22	38,071,607	8.79E-07	0.260	0.279				*LGALS1*	TSS200
cg14186321	2	151,544,796	4.12E-13	0.260	0.210			Yes		
cg15199678	7	96,642,510	3.83E-05	0.260	0.277				*DLX6AS*	Body
cg17062109	17	46,670,029	6.04E-09	0.260	0.253				*LOC404266*	Body
cg17115250	6	28,557,291	0.001221	0.260	0.199					
cg23704517	13	109,147,317	0.000202	0.260	0.130					
cg18650716	6	30,711,863	0.000806	0.260	0.254				*FLOT1*	TSS1500
cg14676825	17	27,038,886	0.000324	0.260	0.293				*PROCA1*	TSS200
cg16163756	2	192,807,194	1.26E-16	0.260	0.249					
cg17527404	X	119,135,074	2.06E-07	0.260	0.262					
cg21158502	5	74,348,187	1.93E-05	0.260	0.172					
cg24398061	10	102,626,652	0.000611	0.260	0.322					
cg27565450	X	72,667,088	3.51E-05	0.260	0.175				*CDX4*	TSS200
cg26788672	11	91,958,396	0.004611	0.260	0.280					
cg01460436	20	44,687,454	4.13E-07	0.260	0.230				*SLC12A5*	3'UTR
cg04171235	17	46,604,393	3.1E-06	0.260	0.205					
cg09127607	10	69,914,213	5.39E-05	0.260	0.259				*MYPN*	Body
cg10204868	2	131,512,971	5.83E-06	0.260	0.269				*FAM123C*	TSS1500
cg14772107	X	23,350,419	0.002473	0.260	0.282					
cg20062681	11	94,988,642	2.39E-05	0.260	0.238					
cg19452802	6	46,459,961	0.006656	0.260	0.127					
cg00123762	6	41,343,697	0.003779	0.260	0.286					
cg13169011	14	60,973,462	0.000314	0.260	0.223					
cg24059115	7	134,143,489	0.021456	0.260	0.233				*AKR1B1*	Body
cg10012059	19	4,327,106	2.1E-05	0.260	0.273				*STAP2*	Body
cg06871582	12	59,990,420	0.004682	0.260	0.224					
cg15599668	5	150,005,953	3.01E-09	0.260	0.269				*SYNPO*	Body
cg03771840	6	30,140,145	1.74E-09	0.260	0.250				*TRIM15*	3'UTR
cg22769941	11	20,690,628	0.018877	0.260	0.265				*NELL1*	TSS1500

cg12681370	2	218,794,733	2.42E-16	0.260	0.257				*TNS1*	5'UTR
cg07002403	10	68,687,951	0.004039	0.260	0.245				*CTNNA3*	Body
cg08795279	16	56,623,816	5.91E-07	0.260	0.191				*MT3*	Body
cg13390441	9	132,962,957	4.62E-15	0.260	0.273				*FREQ*	Body
cg20898283	14	75,920,198	1E-11	0.260	0.220				*JDP2*	Body
cg01100068	1	58,715,747	1.34E-05	0.260	0.227				*DAB1*	5'UTR
cg02391713	6	105,400,985	0.047306	0.260	0.251					
cg14662355	1	165,324,810	6.6E-05	0.260	0.321				*LMX1A*	5'UTR
cg17753212	17	6,659,562	0.001405	0.260	0.233				*XAF1*	Body
cg14377703	17	19,647,973	6.08E-05	0.260	0.305				*ALDH3A1*	Body
cg15735157	7	22,974,608	2.94E-20	0.260	0.242					
cg08432013	11	83,393,570	0.001055	0.260	0.260				*DLG2*	Body
cg13377264	6	30,419,135	2.93E-05	0.260	0.305					
cg24996161	5	140,735,397	4.66E-05	0.260	0.255				*PCDHGA2*	Body
cg00959431	9	79,638,117	1.4E-08	0.260	0.214					
cg24874350	20	45,440,346	1.65E-53	0.260	0.252					
cg08305942	16	79,692,354	9.99E-14	0.260	0.240					
cg11888511	7	33,663,100	1.49E-16	0.260	0.271					
cg13321114	6	29,760,023	0.000125	0.260	0.305				*HCG4*	Body
cg26257411	6	33,397,122	2.8E-13	0.260	0.259				*SYNGAP1*	Body
cg19566774	14	67,656,082	2.67E-18	0.260	0.246				*FAM71D*	TSS200
cg21476299	13	43,148,214	0.000943	0.260	0.313				*TNFSF11*	TSS200
cg21388327	1	22,351,548	0.012288	0.260	0.242				*HSPC157*	TSS1500
cg02385840	12	5,539,588	0.00026	0.260	0.286					
cg13465477	9	133,534,961	0.008496	0.260	0.309					
cg16001722	6	127,836,159	6.15E-05	0.260	0.241				*C6orf174*	Body
cg26301565	3	194,117,261	6.49E-06	0.260	0.240				*GP5*	3'UTR
cg00084770	10	88,149,210	0.035151	0.260	0.312					
cg11362010	3	181,442,713	5.45E-06	0.260	0.246				*SOX2OT*	Body
cg19793376	9	135,462,522	0.006554	0.260	0.269				*BARHL1*	Body
cg22934200	2	61,407,270	0.001024	0.260	0.301				*AHSA2*	5'UTR
cg00121626	Y	21,664,296	0.004351	0.260	0.193				*BCORL2*	Body
cg13692543	9	35,510,933	4.62E-06	0.260	0.226				*RUSC2*	5'UTR
cg15397231	6	31,239,074	1.42E-08	0.260	0.213				*HLA-C*	Body

cg06175981	2	220,387,980	4.91E-08	0.259	0.232				*ACCN4*	Body
cg12219325	8	59,058,585	7.32E-06	0.259	0.236				*FAM110B*	5'UTR
cg23082862	10	128,993,115	9.32E-08	0.259	0.235				*FAM196A*	5'UTR
cg27393010	7	121,940,216	8.35E-05	0.259	0.284					
cg01552731	19	46,806,907	5.46E-32	0.259	0.237				*HIF3A*	1stExon
cg09263118	19	11,531,294	0.008452	0.259	0.322				*RGL3*	TSS1500
cg27208307	10	70,847,399	0.006093	0.259	0.113	2.85E-11	0.158		*SRGN*	TSS1500
cg00777079	17	1,666,607	6.32E-11	0.259	0.244				*SERPINF1*	5'UTR
cg24390913	10	118,899,788	0.007169	0.259	0.366					
cg00089915	1	66,999,684	0.016657	0.259	0.228				*SGIP1*	TSS200
cg20372230	7	127,911,967	5.41E-07	0.259	0.232					
cg26180126	2	70,779,764	0.006605	0.259	0.220				*TGFA*	Body
cg00964997	4	13,531,195	5.2E-07	0.259	0.243					
cg25197238	1	18,335,878	8.28E-08	0.259	0.247					
cg23385492	7	158,325,118	4.94E-06	0.259	0.202			Yes	*PTPRN2*	Body
cg23668195	2	62,805,851	6.6E-07	0.259	0.305					
cg08195491	16	50,746,063	1.36E-07	0.259	0.211				*NOD2*	Body
cg23244488	7	19,146,032	0.002139	0.259	0.292					
cg05675373	1	110,754,257	8.91E-05	0.259	0.229	1.72E-09	0.152		*KCNC4*	1stExon
cg16302441	2	25,391,830	2.73E-05	0.259	0.228	7.68E-18	0.199		*POMC*	TSS1500
cg03625676	5	156,887,393	0.021977	0.259	0.110				*NIPAL4*	Body
cg03520683	13	95,355,814	3.79E-05	0.259	0.284					
cg02965078	11	60,623,504	1.47E-10	0.259	0.245	1.74E-19	0.113		*GPR44*	TSS200
cg11498156	10	102,890,145	3.27E-12	0.259	0.254	2.7E-08	0.122		*TLX1*	TSS1500
cg13197216	8	23,567,658	1.21E-06	0.259	0.223					
cg16785291	7	133,974,228	3.06E-22	0.259	0.237				*SLC35B4*	3'UTR
cg26824006	7	121,957,254	0.000162	0.259	0.240					
cg01385708	16	4,373,570	2.54E-23	0.259	0.247					
cg06387842	3	184,099,096	3.25E-05	0.259	0.247				*CHRD*	Body
cg13923648	6	27,173,910	3.48E-05	0.259	0.258					
cg19619014	8	37,250,930	2.26E-05	0.259	0.237					
cg20328456	6	32,121,113	2.14E-05	0.259	0.302				*PPT2*	TSS200
cg24570303	10	102,900,491	0.003127	0.259	0.234					
cg24683680	2	176,948,864	3.55E-05	0.259	0.287				*EVX2*	TSS200

cg15450098	1	221,057,561	1.06E-12	0.259	0.231				*HLX*	Body
cg16083838	11	2,889,809	1.2E-07	0.259	0.237				*KCNQ1DN*	TSS1500
cg04080282	7	95,026,106	1.26E-05	0.259	0.225				*PON3*	TSS1500
cg20725021	3	187,009,575	5.13E-12	0.259	0.250	2.69E-23	0.166		*MASP1*	5'UTR
cg21002146	14	92,303,622	1.41E-05	0.259	0.228				*TC2N*	TSS1500
cg03894490	3	159,942,832	0.001752	0.259	0.190				*LOC401097*	TSS1500
cg07462756	4	4,859,985	3.22E-09	0.259	0.210				*MSX1*	TSS1500
cg01759597	7	96,626,788	4.07E-05	0.259	0.253				*DLX6AS*	Body
cg07842521	16	85,157,762	1.7E-15	0.259	0.245					
cg09084462	10	27,220,234	1.1E-08	0.259	0.253				*NCRNA00202*	Body
cg21147709	1	12,004,662	1.96E-06	0.259	0.252				*PLOD1*	Body
cg03805475	22	46,972,501	0.021722	0.259	0.215					
cg01009664	3	129,693,613	0.003302	0.259	0.299	1.96E-10	0.189		*TRH*	5'UTR
cg03990033	1	208,084,030	1.11E-06	0.259	0.211				*CD34*	Body
cg04567445	1	65,730,409	1.15E-06	0.259	0.230				*DNAJC6*	TSS200
cg09821007	X	68,348,763	2.79E-07	0.259	0.268					
cg01858517	10	79,396,584	0.005354	0.259	0.195				*KCNMA1*	Body
cg02338271	1	171,868,115	2.42E-13	0.259	0.238				*DNM3*	Body
cg00211821	17	16,978,673	3.49E-05	0.259	0.269				*MPRIP*	Body
cg12873454	1	14,924,611	0.005549	0.259	0.083				*KIAA1026*	TSS1500
cg17310600	4	165,305,334	1E-05	0.259	0.285				*MARCH1*	TSS1500
cg22476027	6	108,492,642	1.44E-05	0.259	0.289				*NR2E1*	Body
cg07500347	16	875,881	0.021329	0.259	0.232					
cg20600210	20	57,090,013	0.000132	0.259	0.253				*APCDD1L*	TSS200
cg06065019	2	177,356,276	7.03E-10	0.259	0.230					
cg12626589	6	32,120,783	4.67E-05	0.259	0.308				*PPT2*	TSS1500
cg22879282	15	32,634,505	0.040343	0.259	0.344					
cg26288249	9	112,810,954	0.003407	0.259	0.136				*AKAP2*	TSS200
cg24254387	1	45,273,495	0.040167	0.259	0.198				*BTBD19*	TSS1500
cg05605029	17	16,189,554	0.001176	0.259	0.308				*PIGL*	Body
cg14090647	22	24,108,000	1.04E-20	0.259	0.242			Yes	*C22orf15*	3'UTR
cg27662832	2	210,444,327	2.27E-08	0.259	0.272				*MAP2*	TSS200
cg03899054	8	143,206,674	2.34E-05	0.259	0.295					
cg05017199	7	150,497,065	0.003173	0.259	0.165				*TMEM176B*	TSS1500

cg12598524	2	46,088,325	4.38E-06	0.259	0.279				*PRKCE*	Body
cg12753606	6	135,504,989	0.01012	0.259	0.128				*MYB*	Body
cg13687570	19	37,825,320	0.01327	0.259	0.148				*HKR1*	TSS1500
cg08335389	12	109,554,302	8.57E-14	0.259	0.272					
cg14184400	3	49,460,057	3.42E-12	0.259	0.268				*AMT*	Body
cg01777341	12	51,784,862	9.24E-12	0.259	0.211				*GALNT6*	5'UTR
cg10927461	2	122,530,159	1.59E-08	0.259	0.272					
cg16746576	8	99,962,118	3.05E-07	0.259	0.260				*OSR2*	Body
cg19634315	15	71,679,485	1.06E-05	0.259	0.230				*THSD4*	Body
cg08425203	3	128,764,940	1.84E-05	0.259	0.223					
cg08537660	11	12,699,661	4.2E-08	0.259	0.245				*TEAD1*	5'UTR
cg08610862	1	197,888,469	0.031109	0.259	0.376				*LHX9*	Body
cg13672078	5	87,439,429	0.014109	0.259	0.271					
cg20466166	1	32,666,735	0.025042	0.259	0.138				*CCDC28B*	5'UTR
cg07475000	7	22,162,928	2.54E-17	0.259	0.240				*RAPGEF5*	Body
cg11505026	2	46,067,299	3.24E-12	0.259	0.240				*PRKCE*	Body
cg23854488	11	69,588,848	0.007565	0.259	0.280				*FGF4*	Body
cg18349298	3	158,450,550	2.05E-21	0.259	0.252				*RARRES1*	TSS1500
cg06728055	3	149,294,286	0.003457	0.259	0.209				*WWTR1*	Body
cg13298538	12	1,759,721	7.27E-12	0.259	0.269					
cg16336494	6	151,188,259	1E-09	0.259	0.257				*MTHFD1L*	Body
cg14609025	1	119,550,983	0.004886	0.259	0.261					
cg16887264	4	147,561,775	0.020228	0.259	0.211				*POU4F2*	Body
cg00908292	17	38,699,009	8.12E-16	0.259	0.243					
cg18396533	10	82,116,208	2.28E-06	0.259	0.186	4.98E-15	0.198		*DYDC2*	TSS1500
cg06630413	12	54,366,875	0.001259	0.259	0.333				*HOXC11*	TSS200
cg06763161	1	33,813,745	2.67E-19	0.259	0.259				*PHC2*	5'UTR
cg01673674	8	97,166,036	3.65E-06	0.259	0.253				*GDF6*	Body
cg10373891	13	52,338,758	0.0009	0.259	0.266					
cg16758813	6	50,818,862	2.11E-05	0.259	0.259					
cg23664396	1	23,895,366	1.55E-17	0.259	0.259					
cg17024199	17	74,881,747	1.47E-06	0.259	0.222				*MGAT5B*	Body
cg02466572	10	94,826,319	1.79E-10	0.259	0.221				*CYP26C1*	Body
cg14249564	11	12,340,382	5.11E-14	0.259	0.273				*MICALCL*	Body

cg20267775	1	171,623,695	6.91E-12	0.259	0.250					
cg23325673	19	34,397,439	3.93E-06	0.259	0.195					
cg00197058	17	59,554,672	2.3E-14	0.259	0.243				*TBX4*	Body
cg01122167	5	149,650,243	4.11E-09	0.259	0.252				*CAMK2A*	Body
cg03344332	8	116,452,420	1.4E-20	0.259	0.265				*TRPS1*	Body
cg06634716	1	217,311,151	0.000606	0.259	0.265				*ESRRG*	TSS200
cg12537796	X	106,515,818	0.028917	0.259	0.298	3.16E-13	0.149			
cg18270687	1	39,035,982	0.027901	0.259	0.307					
cg20421058	4	7,337,225	1.21E-12	0.259	0.271				*SORCS2*	Body
cg03111404	6	28,175,984	0.000477	0.259	0.218					
cg01704474	11	504,918	0.003717	0.259	0.231				*RNH1*	5'UTR
cg07296447	7	1,286,023	0.000445	0.259	0.246					
cg13718539	5	140,801,354	3.38E-06	0.259	0.268				*PCDHGA4*	Body
cg14036143	1	145,415,441	3.37E-10	0.259	0.260				*HFE2*	5'UTR
cg19717326	19	54,370,016	3.49E-14	0.259	0.263	6.59E-24	0.168		*MYADM*	TSS1500
cg24169669	14	38,092,080	0.000165	0.259	0.262					
cg05686497	5	127,874,587	0.021205	0.259	0.339				*FBN2*	TSS1500
cg00957901	4	41,614,842	6.91E-06	0.259	0.202				*LIMCH1*	TSS200
cg12476188	15	66,999,484	6.56E-26	0.259	0.266				*SMAD6*	TSS1500
cg21992932	2	159,651,813	0.000104	0.259	0.265				*DAPL1*	TSS200
cg08835113	7	96,650,192	6.77E-06	0.259	0.254				*DLX5*	Body
cg11533712	22	46,934,981	1.85E-08	0.259	0.236					
cg09272948	20	39,958,363	1.07E-06	0.259	0.239					
cg18474072	10	664,762	2.92E-06	0.259	0.287				*DIP2C*	Body
cg20691080	4	6,200,500	0.004175	0.259	0.186				*JAKMIP1*	5'UTR
cg16251647	6	25,832,228	0.000924	0.258	0.175				*SLC17A1*	1stExon
cg05080940	16	11,256,561	3.32E-06	0.258	0.232				*CLEC16A*	Body
cg13406145	15	89,952,052	0.005527	0.258	0.264					
cg14502980	11	125,133,439	1.29E-11	0.258	0.283				*PKNOX2*	5'UTR
cg09094674	16	23,194,733	4.35E-05	0.258	0.197				*SCNN1G*	5'UTR
cg14177084	7	75,269,642	1.34E-17	0.258	0.230				*HIP1*	Body
cg27367721	16	20,917,257	4.52E-06	0.258	0.254				*LYRM1*	5'UTR
cg03521044	7	20,838,564	5.41E-05	0.258	0.189					
cg07838943	1	12,493,840	5.27E-12	0.258	0.209				*VPS13D*	Body

cg16994941	5	141,218,887	3.37E-08	0.258	0.250					
cg02943578	16	86,600,499	0.004711	0.258	0.239				*FOXC2*	TSS1500
cg07833019	5	38,258,007	0.003714	0.258	0.296				*EGFLAM*	TSS1500
cg24713529	22	50,965,126	0.031679	0.258	0.187				*TYMP*	Body
cg10723962	6	26,240,782	0.001224	0.258	0.309				*HIST1H4F*	1stExon
cg17974767	X	48,690,187	0.000229	0.258	0.267				*PCSK1N*	Body
cg11849281	7	35,494,507	0.000793	0.258	0.170					
cg01181415	12	16,757,954	9.37E-06	0.258	0.190				*LMO3*	5'UTR
cg01440841	4	154,681,066	0.000377	0.258	0.270				*RNF175*	5'UTR
cg26477488	5	153,858,936	0.025682	0.258	0.312				*HAND1*	TSS1500
cg00883831	2	208,636,032	1.71E-11	0.258	0.244					
cg00336700	2	176,943,704	5.7E-10	0.258	0.212					
cg07180646	1	15,481,541	9.58E-12	0.258	0.250				*TMEM51*	5'UTR
cg23144681	10	23,488,710	0.025044	0.258	0.262					
cg03621841	16	49,673,985	0.011923	0.258	0.210				*ZNF423*	Body
cg18147485	8	110,986,685	0.046885	0.258	0.308				*KCNV1*	5'UTR
cg19465605	14	91,770,229	3.36E-21	0.258	0.217				*CCDC88C*	Body
cg23619399	13	79,169,753	0.028258	0.258	0.340					
cg04469167	9	27,529,907	0.024677	0.258	0.104				*MOBKL2B*	TSS200
cg07541744	16	55,363,277	7.64E-05	0.258	0.279				*IRX6*	Body
cg19862899	10	82,173,556	3.91E-07	0.258	0.227				*C10orf58*	TSS200
cg27054162	16	54,628,834	0.000486	0.258	0.203					
cg14195216	12	99,548,950	5.26E-14	0.258	0.262				*ANKS1B*	TSS1500
cg02360980	15	41,804,283	0.020405	0.258	0.231				*LTK*	Body
cg02650401	3	181,441,501	2.08E-08	0.258	0.244				*SOX2OT*	Body
cg26926138	4	54,960,034	2.66E-10	0.258	0.247					
cg23777956	19	4,153,671	2.9E-06	0.258	0.245				*CREB3L3*	1stExon
cg19766988	19	10,231,711	1.09E-16	0.258	0.241				*EIF3G*	TSS1500
cg26275431	5	140,773,122	0.004831	0.258	0.334				*PCDHGA4*	Body
cg08354053	17	30,630,872	9.53E-07	0.258	0.230				*RHBDL3*	Body
cg10784570	11	69,452,398	0.00057	0.258	0.293					
cg16488580	5	173,097,570	9.08E-12	0.258	0.239					
cg15988350	7	32,110,650	0.027486	0.258	0.281				*PDE1C*	5'UTR
cg11763830	17	72,231,400	1.16E-05	0.258	0.289				*TTYH2*	Body

cg24856726	12	108,169,333	9.25E-06	0.258	0.215				*ASCL4*	1stExon
cg12075247	12	47,226,029	0.000646	0.258	0.220					
cg00425764	16	15,949,616	0.000337	0.258	0.277				*MYH11*	5'UTR
cg03954667	12	11,806,065	7.62E-06	0.258	0.258				*ETV6*	Body
cg09006015	3	112,051,908	0.04581	0.258	0.333				*CD200*	TSS200
cg22559013	1	78,693,899	0.002046	0.258	0.146				*MGC27382*	TSS1500
cg00295794	13	100,641,409	0.046193	0.258	0.291					
cg07882059	11	68,924,751	8.79E-14	0.258	0.228					
cg22232207	20	44,746,825	0.000288	0.258	0.308				*CD40*	TSS200
cg00387220	17	48,071,685	0.006228	0.258	0.220				*DLX3*	Body
cg19520763	3	38,534,710	8.29E-18	0.258	0.267					
cg19868364	14	104,734,368	0.001305	0.258	0.210					
cg00576250	20	45,087,765	0.000277	0.258	0.146	1.95E-13	0.145			
cg14535068	2	220,416,419	0.000982	0.258	0.208				*OBSL1*	Body
cg26361533	12	2,445,561	8.57E-06	0.258	0.252				*CACNA1C*	Body
cg07825347	10	43,600,497	5.1E-06	0.258	0.248				*RET*	Body
cg00344443	11	115,530,150	2.7E-07	0.258	0.209					
cg15142477	18	43,915,662	3.27E-27	0.258	0.248				*RNF165*	Body
cg08089301	17	46,655,561	0.041761	0.258	0.316	4.93E-19	0.251		*HOXB4*	1stExon
cg05873889	13	44,948,152	0.004724	0.258	0.260				*SERP2*	1stExon
cg19648612	15	66,274,894	0.000473	0.258	0.258				*MEGF11*	Body
cg13713922	2	121,625,577	2.62E-07	0.258	0.263				*GLI2*	Body
cg12091642	14	61,104,566	1.05E-05	0.258	0.262					
cg17266233	11	46,382,725	4.04E-13	0.258	0.250				*DGKZ*	Body
cg22695532	1	84,158,538	1.92E-06	0.258	0.200					
cg10515731	2	220,378,996	0.037011	0.258	0.241				*ACCN4*	5'UTR
cg00379720	19	23,185,968	0.000499	0.258	0.291					
cg09354309	12	22,487,437	0.007463	0.258	0.267				*ST8SIA1*	5'UTR
cg09258150	19	10,397,429	5.11E-06	0.258	0.272				*ICAM4*	TSS1500
cg10145246	1	119,527,884	2.24E-05	0.258	0.280				*TBX15*	5'UTR
cg11128216	11	31,825,833	0.000113	0.258	0.264				*PAX6*	Body
cg13190879	22	28,198,404	7.5E-08	0.258	0.229				*MN1*	TSS1500
cg09027133	1	25,945,042	4.06E-18	0.258	0.244				*MAN1C1*	Body
cg16905506	1	155,035,748	0.001242	0.258	0.163				*EFNA4*	TSS1500

cg10539700	2	182,644,898	8.9E-13	0.258	0.218					
cg09166091	2	216,484,055	0.006849	0.258	0.217					
cg22234897	17	7,826,291	0.015798	0.258	0.265				*KCNAB3*	3'UTR
cg22344830	1	1,935,048	0.019878	0.258	0.303				*KIAA1751*	5'UTR
cg05052633	17	29,249,485	0.015057	0.258	0.223	4.87E-09	0.135		*ADAP2*	Body
cg05098432	10	95,848,734	2.28E-05	0.258	0.225				*PLCE1*	TSS200
cg21974358	12	52,115,678	0.00207	0.258	0.274			Yes	*SCN8A*	Body
cg08169778	11	115,846,336	1.28E-07	0.258	0.218					
cg04131101	11	94,427,846	1.05E-18	0.258	0.242					
cg09171562	17	17,011,443	1.08E-15	0.258	0.242				*MPRIP*	Body
cg20083676	9	91,605,999	3.67E-14	0.258	0.226	2.07E-34	0.268		*C9orf47*	1stExon
cg17531456	X	134,124,939	0.042258	0.258	0.276				*LOC644538*	TSS200
cg04414451	2	108,993,844	8.23E-10	0.258	0.212				*SULT1C4*	TSS1500
cg12804755	11	15,796,715	2.29E-10	0.258	0.245					
cg13695535	5	66,124,603	0.000622	0.258	0.115				*MAST4*	1stExon
cg15016701	12	63,211,683	0.001136	0.258	0.171				*PPM1H*	Body
cg00881207	7	2,728,912	0.001125	0.258	0.310				*AMZ1*	5'UTR
cg17128947	4	779,480	3.85E-05	0.258	0.231				*CPLX1*	3'UTR
cg24634568	1	43,250,769	2.98E-10	0.258	0.249					
cg04075191	2	115,919,785	1.31E-06	0.258	0.294				*DPP10*	Body
cg05183646	6	134,491,143	2.32E-15	0.258	0.260				*SGK1*	3'UTR
cg24855781	10	68,688,167	0.004238	0.258	0.261				*CTNNA3*	Body
cg21649089	17	14,198,392	2.08E-06	0.258	0.227					
cg04110902	6	45,500,999	1.25E-06	0.258	0.203				*RUNX2*	Body
cg13341412	6	124,151,453	0.00069	0.258	0.250				*NKAIN2*	Body
cg23941527	5	92,868,380	1.29E-05	0.258	0.284				*FLJ42709*	Body
cg20961045	17	55,952,128	3.56E-06	0.258	0.249				*CUEDC1*	Body
cg25073708	9	132,083,538	0.004913	0.258	0.190				*C9orf106*	5'UTR
cg25304107	2	177,017,331	4.22E-07	0.258	0.220				*HOXD4*	Body
cg16711792	21	32,930,054	0.015753	0.258	0.198				*TIAM1*	5'UTR
cg00367463	1	45,249,899	0.000565	0.258	0.322				*BEST4*	Body
cg01554963	17	773,023	0.001689	0.258	0.211				*NXN*	Body
cg19917989	6	14,925,093	2.76E-05	0.258	0.282					
cg20170533	13	51,417,846	0.000695	0.258	0.282				*DLEU7*	1stExon

cg15069295	2	137,522,461	3.12E-05	0.258	0.189					
cg20752878	17	7,080,538	1.22E-09	0.258	0.201				*ASGR1*	Body
cg08081424	2	159,749,591	7.74E-09	0.258	0.255					
cg13016732	11	76,858,947	9.77E-10	0.258	0.206				*MYO7A*	Body
cg02061431	1	92,150,549	1.14E-07	0.258	0.256				*TGFBR3*	Body
cg06071058	9	96,716,091	2.57E-06	0.258	0.233				*BARX1*	Body
cg09513259	9	136,039,474	5.85E-05	0.258	0.169				*GBGT1*	TSS200
cg22732101	19	16,394,638	8.97E-06	0.258	0.246					
cg23370536	4	55,523,824	0.041931	0.258	0.153				*KIT*	TSS1500
cg00575851	X	134,232,238	0.004071	0.258	0.274				*NCRNA00087*	Body
cg07165756	11	66,311,422	1.76E-14	0.258	0.249				*ZDHHC24*	Body
cg00852573	6	73,331,405	0.03003	0.258	0.397				*KCNQ5*	TSS200
cg04883911	2	208,977,452	2.82E-06	0.258	0.265					
cg13622450	9	91,147,514	0.000122	0.258	0.192					
cg20969194	2	223,163,175	2.32E-06	0.258	0.200				*PAX3*	Body
cg11633102	7	67,084,376	2.04E-12	0.258	0.245					
cg20331288	7	155,326,803	1.15E-05	0.258	0.290				*CNPY1*	TSS1500
cg00314029	2	161,348,665	6.79E-07	0.258	0.244				*RBMS1*	Body
cg02388865	1	26,737,318	0.001432	0.258	0.241				*LIN28*	1stExon
cg00953201	13	30,156,486	8.95E-35	0.257	0.242				*SLC7A1*	5'UTR
cg02030542	16	70,722,454	1.06E-13	0.257	0.242				*VAC14*	Body
cg21077684	14	95,233,665	1.79E-06	0.257	0.248					
cg04550737	1	119,530,600	0.032889	0.257	0.172				*TBX15*	5'UTR
cg13181164	15	37,388,828	0.00772	0.257	0.302				*MEIS2*	Body
cg15157455	1	149,400,292	2.68E-25	0.257	0.234				*HIST2H2BF*	TSS1500
cg27022956	3	181,445,025	0.000151	0.257	0.275				*SOX2OT*	Body
cg15859496	12	118,490,276	1.12E-11	0.257	0.239				*WSB2*	Body
cg22800743	6	21,954,586	0.011503	0.257	0.173				*FLJ22536*	Body
cg26968378	6	28,447,087	0.002595	0.257	0.264					
cg22175764	20	56,803,964	0.041645	0.257	0.106	0.01077803	0.048			
cg00584840	3	112,903,054	9.38E-09	0.257	0.256					
cg15538767	5	149,870,395	1.08E-18	0.257	0.231					
cg04025675	15	45,671,028	0.000207	0.257	0.242				*LOC145663*	Body
cg12053004	1	221,057,751	2.7E-09	0.257	0.221				*HLX*	Body

cg17563235	3	54,157,725	0.005302	0.257	0.257				*CACNA2D3*	Body
cg07622493	8	93,114,294	0.000264	0.257	0.295					
cg05166820	6	55,444,153	1.31E-09	0.257	0.250				*HMGCLL1*	TSS200
cg02351277	3	177,397,780	1.39E-12	0.257	0.245					
cg09674170	16	3,062,382	3.61E-09	0.257	0.258				*CLDN9*	TSS200
cg09232478	1	119,548,852	0.000133	0.257	0.259					
cg01277372	3	169,378,764	2.45E-06	0.257	0.245				*MECOM*	Body
cg09296270	2	65,087,288	1.61E-10	0.257	0.182					
cg23068621	5	77,139,964	0.000133	0.257	0.241					
cg12434747	15	70,740,470	2.37E-18	0.257	0.224					
cg16331051	2	75,788,552	0.00513	0.257	0.156				*FAM176A*	5'UTR
cg18842363	6	31,629,088	3.88E-09	0.257	0.230				*C6orf47*	TSS1500
cg21156129	1	54,941,031	0.000816	0.257	0.202					
cg06035815	6	26,189,597	0.003577	0.257	0.224				*HIST1H4D*	TSS1500
cg18144647	8	41,113,257	3.04E-10	0.257	0.232					
cg21629500	5	2,754,753	1.47E-10	0.257	0.241				*C5orf38*	Body
cg26530319	1	221,060,923	4.33E-09	0.257	0.253					
cg16355771	3	13,242,707	3.1E-10	0.257	0.243					
cg24672833	17	46,659,318	0.003251	0.257	0.150					
cg05523897	1	2,139,175	1.06E-12	0.257	0.225				*C1orf86*	TSS200
cg09160589	16	11,654,880	1.16E-07	0.257	0.248				*LITAF*	5'UTR
cg11590420	5	150,051,566	0.000399	0.257	0.198				*MYOZ3*	Body
cg13711950	20	43,729,527	4.61E-05	0.257	0.260				*KCNS1*	5'UTR
cg15859189	3	65,832,744	1.42E-17	0.257	0.251				*MAGI1*	Body
cg22118524	1	217,311,547	1.8E-05	0.257	0.248				*ESRRG*	TSS1500
cg20318166	15	85,360,664	3.05E-09	0.257	0.239				*ALPK3*	1stExon
cg03304610	4	172,734,276	0.012965	0.257	0.331				*GALNTL6*	TSS1500
cg00790307	17	17,684,042	6.05E-14	0.257	0.254				*SMCR5*	TSS1500
cg01364253	X	39,679,986	2.23E-09	0.257	0.247					
cg26532621	5	177,423,340	1.65E-05	0.257	0.271				*PROP1*	TSS200
cg15101392	11	117,748,132	9.19E-08	0.257	0.268				*FXYD6*	TSS1500
cg17023034	2	152,919,487	1.01E-08	0.257	0.275				*CACNB4*	Body
cg26304237	1	65,730,384	1.53E-06	0.257	0.244	1.58E-28	0.241		*DNAJC6*	TSS200
cg15006881	8	97,158,052	6.36E-10	0.257	0.227				*GDF6*	Body

cg11652360	6	33,160,120	9.32E-05	0.257	0.231				*COL11A2*	5'UTR
cg20318845	2	45,237,781	1.69E-06	0.257	0.184				*SIX2*	TSS1500
cg23504246	15	49,715,342	7.37E-07	0.257	0.256	4.77E-16	0.154		*C15orf33*	Body
cg26247508	10	82,299,669	8.73E-09	0.257	0.245				*SH2D4B*	TSS1500
cg09208010	14	23,305,780	0.00359	0.257	0.234			Yes	*MMP14*	TSS200
cg02627657	17	16,257,165	0.000126	0.257	0.169				*CENPV*	TSS1500
cg12434362	5	95,419,503	1.18E-09	0.257	0.264					
cg04555373	8	31,497,042	0.008954	0.257	0.243				*NRG1*	TSS1500
cg18422268	15	66,274,767	0.004345	0.257	0.154				*MEGF11*	Body
cg17177455	2	176,969,218	0.000513	0.257	0.274					
cg23917844	11	61,314,901	9.71E-15	0.257	0.264				*SYT7*	Body
cg14761454	11	108,092,087	0.000761	0.257	0.240				*ATM*	TSS1500
cg10127490	3	15,563,072	3.26E-08	0.257	0.244				*COLQ*	1stExon
cg13090478	11	116,706,372	0.028842	0.257	0.258					
cg25147026	1	186,650,441	0.00015	0.257	0.266				*PTGS2*	TSS1500
cg21063282	14	95,235,402	0.004096	0.257	0.275				*GSC*	Body
cg09222023	3	138,669,322	0.019787	0.257	0.153				*C3orf72*	Body
cg04541368	5	92,909,429	0.0001	0.257	0.195				*FLJ42709*	Body
cg24859433	6	30,720,203	2.82E-09	0.257	0.190					
cg09109383	16	16,083,164	6.21E-11	0.257	0.261				*ABCC1*	Body
cg00262446	4	101,439,228	9.18E-05	0.257	0.248				*EMCN*	5'UTR
cg06741112	14	97,059,243	5.1E-05	0.257	0.197					
cg12612393	1	3,474,376	2.56E-08	0.257	0.248				*MEGF6*	Body
cg16473125	16	55,090,720	0.032735	0.257	0.335					
cg02745912	7	142,494,204	0.000161	0.257	0.277					
cg10168149	2	200,335,558	3.45E-06	0.257	0.249				*FLJ32063*	Body
cg06835156	14	101,131,459	0.002262	0.257	0.257				*C14orf70*	Body
cg17775621	10	11,206,838	0.000439	0.257	0.128				*CUGBP2*	TSS200
cg14947429	10	28,036,151	3.65E-07	0.257	0.277				*MKX*	TSS1500
cg20129210	13	100,547,192	0.005841	0.257	0.173				*CLYBL*	3'UTR
cg19098796	3	183,993,726	0.007665	0.257	0.227				*ECE2*	TSS200
cg27364741	2	63,281,069	2.12E-05	0.257	0.243				*OTX1*	Body
cg13146674	11	68,095,623	0.022686	0.257	0.156				*LRP5*	Body
cg14005211	2	171,676,925	5.94E-13	0.257	0.224				*GAD1*	Body

cg15774391	17	40,439,628	4.69E-07	0.257	0.237				*STAT5A*	5'UTR
cg04751631	7	149,917,170	0.000329	0.257	0.221					
cg07260927	4	11,428,822	1.4E-19	0.257	0.259				*HS3ST1*	5'UTR
cg15069906	20	55,841,888	0.035447	0.257	0.172				*BMP7*	TSS200
cg19116429	10	93,647,563	0.006177	0.257	0.221					
cg13013900	17	6,659,326	0.002003	0.257	0.202				*XAF1*	5'UTR
cg05618426	4	148,401,958	1.95E-08	0.257	0.246				*EDNRA*	TSS200
cg09513276	20	55,836,676	1.5E-17	0.257	0.261				*BMP7*	Body
cg09740450	12	125,223,650	6.39E-07	0.257	0.205					
cg01881444	3	69,061,575	2.55E-07	0.257	0.287				*C3orf64*	5'UTR
cg23266968	14	105,265,793	3.38E-12	0.257	0.248				*ZBTB42*	TSS1500
cg25431535	22	43,084,985	6.41E-06	0.257	0.202					
cg05171584	6	152,128,535	0.000483	0.257	0.190				*ESR1*	TSS1500
cg22494725	22	38,709,636	4.62E-06	0.257	0.224				*CSNK1E*	Body
cg06997549	12	2,944,163	9.44E-05	0.257	0.154				*NRIP2*	5'UTR
cg16306654	1	6,419,767	8.23E-13	0.257	0.238				*ACOT7*	Body
cg08584759	10	11,912,126	3.97E-11	0.257	0.221				*C10orf47*	Body
cg11701148	8	1,993,035	3.13E-05	0.257	0.254	8.32E-08	0.086		*MYOM2*	TSS200
cg08067998	21	38,580,856	5.14E-15	0.257	0.250			Yes	*DSCR9*	TSS200
cg18375642	1	171,154,475	1.44E-14	0.257	0.241				*FMO2*	5'UTR
cg22881914	14	52,536,147	0.012232	0.257	0.259	3.78E-23	0.243		*NID2*	TSS1500
cg01941755	1	200,004,582	3.27E-05	0.257	0.263			Yes	*NR5A2*	Body
cg17836612	17	76,976,357	2.97E-07	0.257	0.217				*LGALS3BP*	TSS1500
cg22377043	15	43,809,486	2.76E-05	0.257	0.238				*MAP1A*	TSS1500
cg06861426	5	179,244,317	0.002361	0.256	0.123				*SQSTM1*	5'UTR
cg03232056	7	157,461,310	0.000594	0.256	0.191				*PTPRN2*	Body
cg07135614	3	138,663,230	0.000152	0.256	0.265				*FOXL2*	1stExon
cg22473846	5	38,846,743	0.000423	0.256	0.203				*OSMR*	5'UTR
cg18533525	1	217,581,184	1.22E-06	0.256	0.273					
cg18088242	15	45,422,325	0.006242	0.256	0.218				*DUOX1*	5'UTR
cg19378036	17	36,105,364	0.027057	0.256	0.258				*HNF1B*	TSS1500
cg21571060	5	92,414,263	8.85E-09	0.256	0.231					
cg01683883	16	66,613,053	9.1E-09	0.256	0.242	6.85E-29	0.211		*CMTM2*	TSS1500
cg21762788	8	9,756,501	0.020319	0.256	0.267					

cg04922803	12	104,444,418	5.17E-08	0.256	0.244				*GLT8D2*	TSS1500
cg03083443	16	1,969,712	4.58E-08	0.256	0.284				*HS3ST6*	TSS1500
cg19683410	21	46,129,392	0.000133	0.256	0.186				*C21orf29*	Body
cg25735305	11	124,629,580	4.99E-11	0.256	0.245				*ESAM*	Body
cg03398481	15	81,666,671	5.26E-09	0.256	0.277				*TMC3*	TSS1500
cg20065768	16	29,796,685	9.95E-06	0.256	0.272					
cg06109894	2	131,185,499	0.022213	0.256	0.331					
cg08734477	6	7,833,720	4.75E-07	0.256	0.252				*BMP6*	Body
cg17826834	15	96,868,857	2.2E-06	0.256	0.224				*NR2F2*	TSS1500
cg02259384	3	58,523,373	3.6E-25	0.256	0.231				*ACOX2*	TSS1500
cg21497082	6	31,111,270	3.27E-10	0.256	0.245				*CCHCR1*	Body
cg22713958	17	76,976,245	1.47E-05	0.256	0.206				*LGALS3BP*	TSS200
cg11111740	6	28,584,288	5.6E-08	0.256	0.249					
cg19507032	2	42,991,422	9.05E-14	0.256	0.264				*OXER1*	TSS200
cg22641201	10	60,401,105	0.000834	0.256	0.169				*BICC1*	Body
cg24718971	5	80,256,166	0.019099	0.256	0.252				*RASGRF2*	TSS1500
cg00039176	5	140,753,076	0.013096	0.256	0.329				*PCDHGA4*	Body
cg10149870	2	129,106,448	5.53E-12	0.256	0.272					
cg12751644	20	60,527,061	6.83E-09	0.256	0.233					
cg13654344	Y	2,654,978	6.75E-05	0.256	0.280				*SRY*	3'UTR
cg13867255	9	138,108,675	0.001397	0.256	0.277					
cg14706475	6	25,218,752	1.66E-09	0.256	0.230					
cg20571592	3	40,657,387	2.13E-05	0.256	0.294					
cg19567689	14	97,059,467	2.57E-08	0.256	0.252					
cg18769353	7	137,028,617	0.013099	0.256	0.158				*PTN*	TSS200
cg03521696	6	29,795,595	1.79E-05	0.256	0.267				*HLA-G*	5'UTR
cg02468728	19	611,107	0.000382	0.256	0.204				*HCN2*	Body
cg07830436	6	30,419,066	0.003841	0.256	0.285					
cg27622017	3	181,447,418	0.000444	0.256	0.262				*SOX2OT*	Body
cg01499197	13	28,544,814	1.49E-07	0.256	0.205				*CDX2*	TSS1500
cg24801587	7	155,244,101	0.020459	0.256	0.222					
cg25021359	7	123,315,035	1.31E-11	0.256	0.269					
cg24621437	10	108,924,560	0.027725	0.256	0.319				*SORCS1*	TSS1500
cg15712310	16	73,100,790	3.68E-07	0.256	0.243					

cg16105620	11	74,178,684	0.000803	0.256	0.212				*KCNE3*	TSS200
cg08898155	7	95,026,097	0.007859	0.256	0.231				*PON3*	TSS1500
cg02121672	X	24,665,932	0.007994	0.256	0.228				*PCYT1B*	Body
cg07575518	14	92,413,497	0.009327	0.256	0.281				*FBLN5*	Body
cg20284239	1	168,091,000	2.94E-18	0.256	0.245				*GPR161*	5'UTR
cg11324832	3	181,441,806	3.39E-05	0.256	0.250				*SOX2OT*	Body
cg14711108	1	44,813,163	2.03E-16	0.256	0.250				*ERI3*	Body
cg16299576	8	10,872,819	0.000345	0.256	0.274				*XKR6*	Body
cg18002862	17	2,718,230	4.06E-06	0.256	0.194				*RAP1GAP2*	Body
cg23236370	5	33,892,705	1.34E-22	0.256	0.240				*ADAMTS12*	TSS1500
cg04848137	8	38,483,364	8.17E-06	0.256	0.199					
cg03669782	4	54,970,254	0.000451	0.256	0.252					
cg15507690	3	176,919,597	2.43E-07	0.256	0.187					
cg06544405	18	70,536,807	1.76E-05	0.256	0.211					
cg19421368	20	45,280,288	4.22E-15	0.256	0.274				*SLC13A3*	TSS200
cg11213248	13	28,534,648	0.010894	0.256	0.269					
cg03316934	2	220,510,231	1.42E-09	0.256	0.274					
cg11523350	11	117,171,073	2.4E-10	0.256	0.268				*BACE1*	Body
cg05048377	16	3,237,964	0.02927	0.256	0.275					
cg24452260	4	158,143,538	1.31E-05	0.256	0.243				*GRIA2*	Body
cg01288724	5	142,575,122	4.93E-10	0.256	0.265				*ARHGAP26*	Body
cg18557131	13	100,624,207	0.00284	0.256	0.235				*ZIC5*	TSS200
cg13048911	5	139,691,821	0.000444	0.256	0.309					
cg24601030	11	111,847,747	0.00513	0.256	0.205				*DIXDC1*	TSS1500
cg03241461	10	29,924,258	5.08E-09	0.256	0.266				*SVIL*	TSS1500
cg08701183	7	21,209,781	5.36E-09	0.256	0.260					
cg16596440	4	140,684,596	0.001764	0.256	0.155				*MAML3*	Body
cg14917244	8	27,469,001	0.000335	0.256	0.215				*CLU*	TSS200
cg07585069	17	27,039,120	1.82E-05	0.256	0.266				*PROCA1*	TSS1500
cg09398550	3	68,056,976	0.033585	0.256	0.297				*FAM19A1*	Body
cg16002305	14	97,879,656	0.011618	0.256	0.333					
cg16991515	6	27,107,019	1.09E-05	0.256	0.238				*HIST1H2BK*	3'UTR
cg26197915	11	48,085,137	5.99E-08	0.256	0.282				*PTPRJ*	Body
cg15867728	4	72,898,212	0.000126	0.256	0.223				*NPFFR2*	Body

cg17974166	1	23,280,069	4.82E-08	0.256	0.257					
cg05797623	4	73,178,007	8.37E-10	0.256	0.254				*ADAMTS3*	Body
cg21182196	14	95,786,801	0.015975	0.256	0.271				*CLMN*	TSS1500
cg24087736	1	68,301,529	2.63E-17	0.256	0.263					
cg18358894	7	121,956,890	0.000834	0.256	0.302					
cg23233200	6	26,272,695	0.014932	0.256	0.331				*HIST1H3G*	TSS1500
cg26833120	1	32,169,568	2.08E-48	0.256	0.256				*COL16A1*	1stExon
cg12890903	14	24,867,954	1.72E-10	0.256	0.218				*NYNRIN*	TSS200
cg15123159	6	43,045,686	2.47E-11	0.256	0.190				*PTK7*	Body
cg09054333	X	142,723,768	0.019348	0.256	0.343				*SLITRK4*	TSS1500
cg00489394	11	63,259,326	0.000774	0.256	0.205				*HRASLS5*	TSS1500
cg05898452	7	55,224,863	3.26E-09	0.256	0.221				*EGFR*	Body
cg22304799	2	28,877,884	4.91E-13	0.256	0.237					
cg00929635	20	44,035,918	4.19E-14	0.256	0.256				*DBNDD2*	5'UTR
cg04387396	10	72,593,165	1.33E-05	0.256	0.275				*SGPL1*	Body
cg18793806	2	95,826,087	4.26E-13	0.256	0.213	4.19E-21	0.163		*ZNF514*	TSS1500
cg25358039	12	81,471,757	0.017825	0.256	0.290				*ACSS3*	TSS200
cg26117023	2	74,782,096	0.002057	0.256	0.278	4.49E-14	0.225		*LOXL3*	TSS1500
cg09614479	10	81,967,536	6.95E-07	0.256	0.222					
cg20495737	X	48,327,768	6.89E-05	0.256	0.201				*SLC38A5*	5'UTR
cg26268277	8	26,372,879	0.000219	0.256	0.229				*PNMA2*	TSS1500
cg02305242	14	85,996,202	0.012672	0.256	0.235				*FLRT2*	TSS1500
cg12029804	17	38,821,390	0.012548	0.256	0.243				*KRT222*	5'UTR
cg25423004	2	176,993,211	1.61E-06	0.256	0.228				*HOXD8*	TSS1500
cg02619116	17	40,819,808	4.11E-05	0.256	0.259					
cg07918545	10	129,535,669	0.010185	0.256	0.245				*FOXI2*	1stExon
cg11638071	17	56,405,967	9.08E-09	0.256	0.244				*BZRAP1*	1stExon
cg21729992	20	55,200,370	2.78E-05	0.256	0.268					
cg01413632	12	76,078,445	0.014001	0.256	0.303					
cg15475650	10	120,938,899	7.91E-06	0.256	0.228				*PRDX3*	TSS1500
cg20059140	18	11,751,516	0.011732	0.256	0.286				*GNAL*	TSS200
cg01022200	1	180,881,086	0.000389	0.255	0.220				*KIAA1614*	TSS1500
cg25668058	12	115,183,175	8.31E-24	0.255	0.239					
cg02495310	5	134,366,831	0.008862	0.255	0.330				*PITX1*	Body

cg06192866	12	89,748,300	0.003438	0.255	0.280					
cg26248114	1	56,901,693	5.8E-10	0.255	0.241					
cg10110083	6	30,418,845	0.001236	0.255	0.316					
cg20705893	7	157,869,970	2.95E-09	0.255	0.238				*PTPRN2*	Body
cg01488770	17	59,474,078	9.41E-07	0.255	0.247					
cg16209444	3	58,522,771	7.9E-19	0.255	0.246				*ACOX2*	5'UTR
cg25762679	1	101,185,311	0.01187	0.255	0.221				*VCAM1*	1stExon
cg06813578	15	51,386,254	0.001182	0.255	0.255				*TNFAIP8L3*	Body
cg11141652	22	24,348,549	0.029738	0.255	0.002				*GSTTP1*	TSS1500
cg13031171	13	29,106,870	0.000453	0.255	0.221					
cg05248655	10	1,454,265	2.12E-13	0.255	0.224				*ADARB2*	Body
cg16704560	2	15,777,810	1.08E-21	0.255	0.248			Yes		
cg03606772	1	152,487,856	0.001275	0.255	0.272				*CRCT1*	5'UTR
cg20790648	3	151,619,923	4.79E-12	0.255	0.243					
cg07725206	1	85,251,175	1.43E-16	0.255	0.234					
cg22430861	17	55,584,079	1.15E-07	0.255	0.254				*MSI2*	Body
cg23637494	3	138,663,400	0.002248	0.255	0.262				*FOXL2*	1stExon
cg06221393	5	139,089,315	2.49E-07	0.255	0.250					
cg11285496	5	81,882,123	0.000268	0.255	0.222					
cg12272369	10	95,848,841	8.7E-05	0.255	0.235				*PLCE1*	Body
cg07046818	8	97,166,291	2E-07	0.255	0.257				*GDF6*	Body
cg19610869	5	157,098,619	4.02E-05	0.255	0.213				*C5orf52*	5'UTR
cg22301854	4	8,081,109	0.000416	0.255	0.243				*ABLIM2*	Body
cg20260721	15	63,245,298	1.7E-11	0.255	0.249					
cg22974982	12	30,323,251	0.000787	0.255	0.207					
cg09735465	9	120,507,228	4.18E-07	0.255	0.225					
cg23783653	6	27,059,850	0.014013	0.255	0.238					
cg26695445	16	87,902,677	0.005701	0.255	0.291				*SLC7A5*	1stExon
cg26979473	7	137,028,389	0.009359	0.255	0.167				*PTN*	5'UTR
cg20934596	22	31,481,480	0.019191	0.255	0.069				*SMTN*	Body
cg06193578	19	35,396,475	0.010468	0.255	0.261					
cg06948222	16	87,958,493	5.05E-08	0.255	0.218				*CA5A*	Body
cg07905054	12	102,272,136	1.71E-09	0.255	0.244				*DRAM1*	Body
cg18612461	15	75,251,733	7.91E-07	0.255	0.210					

cg21279510	1	8,277,627	0.015607	0.255	0.189					
cg22430700	1	180,511,495	5.64E-14	0.255	0.251					
cg19084031	15	38,361,362	0.000165	0.255	0.193					
cg07961994	4	93,227,195	0.042568	0.255	0.156				*GRID2*	Body
cg02448597	6	32,160,605	1.67E-07	0.255	0.204				*GPSM3*	5'UTR
cg08537783	1	10,795,394	9.2E-14	0.255	0.261				*CASZ1*	5'UTR
cg20699586	1	227,748,719	1.27E-13	0.255	0.247					
cg25330016	17	77,030,281	2.01E-08	0.255	0.213				*C1QTNF1*	TSS200
cg01192077	5	158,523,507	0.004286	0.255	0.269				*EBF1*	Body
cg00418027	9	96,716,998	4.8E-05	0.255	0.307				*BARX1*	Body
cg13169132	1	1,362,340	3.63E-07	0.255	0.238				*TMEM88B*	Body
cg22193276	2	11,965,198	7.21E-18	0.255	0.246				*LPIN1*	3'UTR
cg01897496	2	182,545,209	7.42E-06	0.255	0.234				*NEUROD1*	1stExon
cg02611848	2	74,875,387	3.71E-07	0.255	0.241				*C2orf65*	TSS1500
cg03465028	15	31,658,323	0.04999	0.255	0.225				*KLF13*	Body
cg09278107	14	105,779,826	2.83E-22	0.255	0.239				*PACS2*	TSS1500
cg09670128	12	52,627,047	0.042797	0.255	0.310				*KRT7*	5'UTR
cg00419094	12	106,480,579	5.9E-58	0.255	0.255				*NUAK1*	Body
cg19856897	7	142,495,846	7.95E-05	0.255	0.265					
cg20747577	7	26,897,253	2.15E-07	0.255	0.239				*SKAP2*	Body
cg25382400	3	181,445,514	4.52E-05	0.255	0.233				*SOX2OT*	Body
cg17759274	19	39,260,954	1.57E-08	0.255	0.246					
cg08660915	22	50,523,790	1.34E-07	0.255	0.225				*MLC1*	1stExon
cg17710779	10	123,356,331	0.000956	0.255	0.275				*FGFR2*	5'UTR
cg18430555	5	170,740,937	0.03307	0.255	0.262					
cg20432960	6	50,804,174	0.000547	0.255	0.266				*TFAP2B*	Body
cg05924583	1	3,569,624	1.9E-06	0.255	0.213	0.0000146	0.082		*TP73*	5'UTR
cg10159630	2	177,037,364	7.96E-07	0.255	0.204				*HOXD3*	3'UTR
cg13321164	10	48,416,781	2.86E-12	0.255	0.243				*GDF2*	5'UTR
cg19566405	17	33,759,965	0.039427	0.255	0.181				*SLFN12*	TSS1500
cg22031614	15	76,628,424	0.001819	0.255	0.247				*ISL2*	TSS1500
cg22005990	17	46,659,939	2.17E-06	0.255	0.219					
cg06819923	16	21,214,508	8.96E-11	0.255	0.265				*ZP2*	Body
cg17157725	22	45,667,757	5.74E-12	0.255	0.234					

cg09233651	12	114,838,567	0.002762	0.255	0.249				*TBX5*	Body
cg09916783	2	25,391,911	6.68E-06	0.255	0.206				*POMC*	TSS1500
cg13021619	8	23,563,859	0.00134	0.255	0.251				*NKX2-6*	1stExon
cg17729667	20	25,566,382	0.005057	0.255	0.140	6.58E-26	0.188		*NINL*	TSS1500
cg06705122	19	49,371,079	3.86E-05	0.255	0.273				*PLEKHA4*	5'UTR
cg07451803	5	53,927,032	1.69E-14	0.255	0.254					
cg08678044	19	51,226,849	0.006968	0.255	0.350				*CLEC11A*	1stExon
cg11231751	19	2,302,783	1.02E-07	0.255	0.268				*LINGO3*	5'UTR
cg19451673	12	48,398,730	0.020566	0.255	0.119				*COL2A1*	TSS1500
cg27248029	17	71,039,644	2.17E-05	0.255	0.195				*SLC39A11*	Body
cg24399540	2	207,307,150	0.000186	0.255	0.237				*ADAM23*	TSS1500
cg25611057	3	197,121,419	1E-14	0.255	0.211					
cg05720261	1	40,105,304	0.018909	0.255	0.277				*HEYL*	1stExon
cg12526997	2	242,974,096	9.06E-09	0.255	0.218					
cg26209005	7	132,262,526	0.001716	0.255	0.184				*PLXNA4*	TSS1500
cg04477962	12	51,317,375	7.03E-17	0.255	0.253				*METTL7A*	TSS1500
cg06232807	16	51,185,132	0.00063	0.255	0.196				*SALL1*	TSS1500
cg04311403	1	236,559,646	0.01453	0.255	0.062				*EDARADD*	Body
cg26202753	7	1,850,203	1.09E-13	0.255	0.255					
cg26251506	7	96,642,163	4.08E-06	0.255	0.249				*DLX6AS*	Body
cg03922126	6	73,330,297	0.010275	0.255	0.299				*KCNQ5*	TSS1500
cg17491622	1	1,369,616	0.001079	0.255	0.158				*VWA1*	TSS1500
cg04032066	11	20,622,721	0.000734	0.255	0.296				*SLC6A5*	Body
cg12390344	18	21,270,348	1.32E-05	0.255	0.224				*LAMA3*	Body
cg12501761	2	240,898,385	8.43E-20	0.255	0.232				*NDUFA10*	3'UTR
cg19722748	7	96,627,700	0.00845	0.255	0.310				*DLX6AS*	Body
cg21106407	14	57,262,177	0.000878	0.255	0.251					
cg27406678	4	85,417,612	0.0073	0.255	0.266				*NKX6-1*	Body
cg24181771	6	28,706,472	0.044702	0.255	0.241					
cg07102229	12	47,219,958	4.91E-11	0.255	0.234				*SLC38A4*	TSS200
cg08473660	8	61,777,765	5.11E-07	0.255	0.231				*CHD7*	Body
cg13761949	10	35,098,359	0.003415	0.255	0.156				*PARD3*	Body
cg17949727	1	38,412,684	1.29E-05	0.255	0.248				*INPP5B*	5'UTR
cg00044995	19	408,683	0.000305	0.255	0.244				*C2CD4C*	5'UTR

cg01824969	8	67,875,261	0.001207	0.255	0.318					
cg02921624	19	35,068,788	1.19E-05	0.255	0.203				*LOC643719*	TSS200
cg14190534	14	90,848,416	1.71E-06	0.255	0.220					
cg20945055	17	836,474	2.65E-08	0.255	0.260				*NXN*	Body
cg08136809	19	41,882,642	0.003352	0.255	0.150				*TMEM91*	1stExon
cg09855140	13	100,004,097	5.88E-14	0.255	0.247				*UBAC2*	Body
cg08839210	17	27,045,048	0.000103	0.255	0.236				*RAB34*	TSS200
cg08698854	7	134,575,306	3.41E-16	0.255	0.253				*CALD1*	TSS1500
cg15177359	17	73,840,750	5.6E-20	0.255	0.281	5.46E-38	0.161		*UNC13D*	5'UTR
cg19954017	17	80,333,257	0.000423	0.255	0.278				*UTS2R*	1stExon
cg04221461	1	243,877,024	4.1E-15	0.255	0.230				*AKT3*	Body
cg15291243	17	35,298,199	0.00191	0.255	0.242				*LHX1*	Body
cg03517776	2	64,834,430	0.00217	0.255	0.265					
cg17990365	11	319,718	5.02E-06	0.255	0.179				*IFITM3*	3'UTR
cg17567560	10	105,036,863	0.00836	0.255	0.104				*INA*	TSS200
cg13175329	6	25,035,073	5.15E-05	0.255	0.270					
cg16675485	2	6,055,439	3.68E-22	0.255	0.239					
cg16379716	15	75,965,427	3.03E-13	0.255	0.202					
cg03589296	2	66,666,292	2.48E-06	0.255	0.250				*MEIS1*	Body
cg09136878	1	236,135,584	3.1E-06	0.255	0.241					
cg11643510	2	16,124,837	8.15E-14	0.255	0.221					
cg01120165	22	50,690,152	0.000722	0.255	0.316	4.12E-08	0.128		*HDAC10*	TSS1500
cg19598293	7	117,854,765	0.003563	0.255	0.241					
cg15111495	3	112,951,743	6.09E-06	0.255	0.236				*BOC*	5'UTR
cg18358723	5	87,955,618	2.17E-06	0.255	0.246				*LOC645323*	Body
cg20432507	20	21,689,861	0.000313	0.255	0.269				*PAX1*	Body
cg03475182	11	110,581,454	0.007079	0.254	0.281				*ARHGAP20*	Body
cg08655206	1	221,058,198	1.05E-17	0.254	0.235				*HLX*	3'UTR
cg11009817	5	101,632,372	0.017135	0.254	0.187				*SLCO4C1*	TSS200
cg16655240	20	32,857,072	0.000171	0.254	0.167				*ASIP*	3'UTR
cg03793270	11	89,224,684	2.12E-12	0.254	0.273				*NOX4*	5'UTR
cg13462129	7	96,651,281	6.99E-08	0.254	0.241	0.00000213	0.099		*DLX5*	Body
cg06809252	1	110,612,044	5.46E-09	0.254	0.223				*ALX3*	Body
cg17566175	6	56,389,866	0.001104	0.254	0.236				*DST*	Body

cg19084508	5	150,530,152	1.15E-06	0.254	0.235				*ANXA6*	5'UTR
cg21451998	1	110,198,240	1.35E-05	0.254	0.274				*GSTM4*	TSS1500
cg23866687	11	96,026,956	6.56E-07	0.254	0.278				*MAML2*	Body
cg10576051	15	73,661,652	5.01E-05	0.254	0.265				*HCN4*	TSS200
cg19709355	17	38,504,102	2.34E-12	0.254	0.269				*RARA*	Body
cg27425146	1	25,062,925	4.77E-12	0.254	0.266					
cg06316886	2	177,027,043	0.026105	0.254	0.265					
cg16336436	14	76,178,685	8.5E-20	0.254	0.259				*TTLL5*	Body
cg03292222	5	170,734,312	0.00626	0.254	0.155					
cg03816707	20	43,726,544	4.69E-07	0.254	0.283				*KCNS1*	Body
cg10386298	1	221,050,491	9.59E-06	0.254	0.248					
cg25306277	1	200,003,267	1.01E-10	0.254	0.237				*NR5A2*	Body
cg00324097	17	75,446,549	0.006116	0.254	0.143				*SEPT9*	TSS200
cg02392696	12	52,258,264	0.00248	0.254	0.250					
cg06513247	17	75,446,661	0.002102	0.254	0.152				*SEPT9*	1stExon
cg00794881	1	19,043,573	5.45E-07	0.254	0.222				*PAX7*	Body
cg03494430	16	2,177,269	1.14E-09	0.254	0.260				*PKD1*	Body
cg05990080	3	37,965,818	2.36E-23	0.254	0.241				*CTDSPL*	Body
cg19452248	5	143,883,482	0.003073	0.254	0.204					
cg19096571	7	129,423,354	2.12E-05	0.254	0.252					
cg21885107	14	73,712,289	1.29E-09	0.254	0.231				*PAPLN*	Body
cg25729445	6	29,595,347	7.64E-12	0.254	0.260				*GABBR1*	Body
cg27606396	2	113,542,117	2.5E-06	0.254	0.302				*IL1A*	1stExon
cg09126636	1	113,265,753	4.89E-14	0.254	0.234				*FAM19A3*	Body
cg16365445	10	33,230,686	9.19E-16	0.254	0.248				*ITGB1*	5'UTR
cg01826117	16	85,181,279	4.75E-13	0.254	0.244					
cg05531482	7	124,430,753	0.000388	0.254	0.288					
cg13997435	1	153,538,406	1.8E-11	0.254	0.262	3.99E-32	0.186		*S100A2*	TSS200
cg06611810	19	41,317,067	0.007807	0.254	0.271					
cg01192745	3	31,239,040	0.000243	0.254	0.180					
cg10293403	6	10,421,670	5.92E-06	0.254	0.256					
cg13485230	18	9,709,310	3.05E-06	0.254	0.247				*RAB31*	Body
cg04612444	8	57,358,713	0.000724	0.254	0.219				*PENK*	5'UTR
cg05267204	2	55,360,999	0.000204	0.254	0.221			Yes		

cg10583893	19	53,039,743	1.18E-05	0.254	0.237				*ZNF808*	5'UTR	
cg15603424	11	13,300,592	7.3E-07	0.254	0.259				*ARNTL*	5'UTR	
cg17646820	3	66,848,679	6.2E-07	0.254	0.226						
cg05893709	8	39,836,064	0.010953	0.254	0.105				*IDO2*	Body	
cg25516803	3	42,307,519	5.42E-07	0.254	0.266				*CCK*	TSS1500	
cg15454726	1	241,587,609	0.004649	0.254	0.313						
cg14970046	5	1,880,021	1.47E-05	0.254	0.225				*IRX4*	Body	
cg20557801	15	68,121,177	0.001389	0.254	0.192				*LBXCOR1*	Body	
cg09728102	5	66,124,492	0.008831	0.254	0.070				*MAST4*	TSS200	
cg24329557	6	10,882,326	1.93E-05	0.254	0.252				*GCM2*	TSS1500	
cg19468029	3	46,854,231	0.010744	0.254	0.220						
cg26299169	13	28,498,956	0.030443	0.254	0.322				*PDX1*	3'UTR	
cg01323148	2	206,551,212	0.003488	0.254	0.259				*NRP2*	Body	
cg04764624	17	29,718,129	2.8E-09	0.254	0.231				*RAB11FIP4*	TSS1500	
cg06622408	15	89,952,299	0.001259	0.254	0.296						
cg13814351	9	136,446,836	1.65E-18	0.254	0.241				*FAM163B*	TSS1500	
cg16112276	11	58,806,952	3.12E-14	0.254	0.236			Yes			
cg04193065	15	31,528,995	4.31E-06	0.254	0.264						
cg04332927	6	44,039,484	1.37E-05	0.254	0.248						
cg04589008	2	660,692	1.05E-14	0.254	0.240						
cg07791490	8	99,949,391	5.53E-08	0.254	0.265						
cg26635451	12	89,749,142	1.36E-10	0.254	0.249						
cg23164203	6	28,367,475	0.004776	0.254	0.226				*ZSCAN12*	Body	
cg05516272	11	66,311,334	2.74E-20	0.254	0.254				*ZDHHC24*	Body	
cg17827017	1	208,333,467	1.05E-17	0.254	0.236				*PLXNA2*	Body	
cg23858410	22	46,276,678	0.001526	0.254	0.269						
cg18359530	5	77,945,219	0.007301	0.254	0.191				*LHFPL2*	TSS1500	
cg26302986	7	90,291,360	7.09E-17	0.254	0.239						
cg27140872	5	147,162,461	0.000973	0.254	0.231				*JAKMIP2*	TSS1500	
cg12110262	10	94,827,200	8.3E-11	0.254	0.240				*CYP26C1*	Body	
cg27346528	17	44,896,212	0.00645	0.254	0.337				*WNT3*	TSS200	
cg08977270	3	158,450,833	6.11E-18	0.254	0.257				*RARRES1*	TSS1500	
cg25825506	7	96,626,659	0.001767	0.254	0.235				*DLX6AS*	Body	
cg24952328	19	19,383,704	0.000297	0.254	0.127				*TM6SF2*	Body	

cg04722391	7	137,032,114	0.005054	0.254	0.198					
cg18310639	1	223,904,225	4.54E-12	0.254	0.235				*CAPN2*	Body
cg25432738	13	21,009,861	2.39E-18	0.254	0.213				*CRYL1*	Body
cg00808648	14	105,779,910	1.44E-09	0.254	0.265				*PACS2*	TSS1500
cg19086540	11	36,397,458	3.28E-05	0.254	0.269				*PRR5L*	5'UTR
cg25509871	19	40,871,557	0.004943	0.254	0.267				*PLD3*	5'UTR
cg27599211	12	117,483,006	1.14E-09	0.254	0.181				*TESC*	Body
cg01639898	1	32,083,012	9.83E-09	0.254	0.229				*HCRTR1*	TSS1500
cg21504624	9	34,653,580	1.13E-22	0.254	0.241	5.53E-28	0.151		*IL11RA*	5'UTR
cg07611334	20	327,811	0.035891	0.254	0.034	1.79E-15	0.192		*NRSN2*	5'UTR
cg02627216	17	38,599,056	4.07E-12	0.254	0.254				*IGFBP4*	TSS1500
cg03428047	4	140,730,656	8.23E-09	0.254	0.257				*MAML3*	Body
cg06065125	4	85,402,870	0.001701	0.254	0.236					
cg06478886	5	135,416,029	1.94E-05	0.254	0.195					
cg07207982	6	34,984,930	0.002187	0.254	0.197				*ANKS1A*	Body
cg07958387	5	172,069,173	2.09E-07	0.254	0.225				*NEURL1B*	Body
cg12307840	1	223,538,334	0.000905	0.254	0.137				*SUSD4*	TSS1500
cg10204320	1	32,708,276	4.32E-11	0.254	0.253				*MTMR9L*	TSS1500
cg14565781	1	45,274,513	2.43E-09	0.254	0.253				*BTBD19*	1stExon
cg17075998	X	40,104,353	1.55E-09	0.254	0.243					
cg14786652	1	1,109,192	0.012457	0.254	0.292				*TTLL10*	TSS200
cg18346576	2	27,938,071	4.03E-15	0.254	0.255					
cg03546235	10	102,590,290	1.35E-06	0.254	0.244					
cg24292235	12	8,171,463	0.003238	0.254	0.282					
cg00695799	6	149,803,087	8.08E-09	0.254	0.242				*ZC3H12D*	5'UTR
cg07202214	11	76,381,081	1.54E-08	0.254	0.230				*LRRC32*	5'UTR
cg09450063	13	108,922,504	1.56E-06	0.254	0.232				*TNFSF13B*	1stExon
cg02362103	1	119,532,773	6.62E-09	0.254	0.287				*TBX15*	TSS1500
cg07389611	17	59,540,267	2.31E-05	0.254	0.193				*TBX4*	Body
cg09066326	5	140,753,415	0.027628	0.254	0.329				*PCDHGA4*	Body
cg17919966	15	78,384,712	2.36E-14	0.254	0.241				*SH2D7*	TSS1500
cg08878323	7	96,652,464	0.002213	0.254	0.233	1.31E-16	0.157		*DLX5*	Body
cg12798157	1	19,600,719	4.47E-05	0.254	0.227				*AKR7L*	TSS200
cg08846566	8	80,803,954	0.000191	0.254	0.254					

cg11137160	12	125,674,249	5.28E-08	0.254	0.239					
cg07933926	12	6,485,881	4.72E-08	0.254	0.257				*SCNN1A*	Body
cg16524733	11	117,070,046	6.56E-11	0.254	0.254				*TAGLN*	5'UTR
cg10514097	16	86,544,787	0.001625	0.254	0.153				*FOXF1*	1stExon
cg17033080	2	217,508,851	2.86E-12	0.254	0.268				*IGFBP2*	Body
cg20555674	17	37,321,631	0.022717	0.254	0.263				*ARL5C*	Body
cg00766188	15	89,953,370	1.67E-31	0.254	0.245					
cg14006181	14	29,254,942	1.21E-05	0.254	0.263				*C14orf23*	Body
cg21625570	12	50,268,448	2.47E-08	0.254	0.269				*FAIM2*	Body
cg21977377	20	21,492,823	0.026195	0.254	0.325				*NKX2-2*	Body
cg09943560	14	91,581,064	0.011362	0.254	0.118				*C14orf159*	5'UTR
cg09284275	16	15,923,487	8.36E-13	0.254	0.256				*MYH11*	Body
cg26509318	12	122,019,760	1.86E-05	0.254	0.264				*KDM2B*	TSS1500
cg27557378	16	2,286,786	0.046727	0.254	0.086				*DNASE1L2*	5'UTR
cg08548659	4	32,149,506	0.001399	0.254	0.246					
cg12262378	17	6,899,522	1.99E-08	0.254	0.201				*ALOX12*	1stExon
cg01484487	2	74,726,892	4.18E-07	0.254	0.259				*LBX2*	Body
cg10165585	1	29,449,042	0.002906	0.254	0.322				*TMEM200B*	5'UTR
cg11518846	6	133,562,246	0.010021	0.254	0.305				*EYA4*	TSS1500
cg14684457	7	26,192,966	0.001585	0.254	0.177				*NFE2L3*	Body
cg15072619	10	18,294,563	1.16E-06	0.254	0.211				*SLC39A12*	Body
cg10146203	15	27,111,435	0.000771	0.254	0.252				*GABRA5*	TSS1500
cg10495164	11	64,631,357	1.76E-15	0.254	0.254				*EHD1*	Body
cg08424184	1	36,043,633	8.98E-07	0.254	0.248				*TFAP2E*	Body
cg11018604	5	71,507,163	1.25E-13	0.254	0.241					
cg12850892	11	75,945,585	6.69E-06	0.254	0.218					
cg16902746	19	5,338,881	2.6E-08	0.254	0.245				*PTPRS*	5'UTR
cg03514351	1	65,991,765	4.29E-06	0.254	0.204				*LEPR*	5'UTR
cg26886066	13	113,677,034	7.15E-07	0.254	0.252				*MCF2L*	Body
cg10075976	12	99,303,715	2.37E-05	0.253	0.215				*ANKS1B*	Body
cg15835542	6	45,709,808	7.24E-07	0.253	0.218					
cg09272748	10	43,428,910	0.009496	0.253	0.293					
cg26348487	19	2,488,560	2.34E-10	0.253	0.240					
cg06562372	7	87,230,196	2.55E-07	0.253	0.237				*ABCB1*	5'UTR

cg14958635	5	134,870,977	0.000281	0.253	0.248	4.29E-09	0.141		*NEUROG1*	1stExon
cg22731373	X	24,665,296	0.007742	0.253	0.291				*PCYT1B*	1stExon
cg26122451	7	92,672,950	2.57E-15	0.253	0.232					
cg08487063	10	102,894,639	0.001083	0.253	0.271				*TLX1*	Body
cg13715631	5	134,366,914	0.000347	0.253	0.307				*PITX1*	Body
cg07452312	5	153,775,466	6.49E-10	0.253	0.262				*GALNT10*	Body
cg24935896	1	201,431,492	5.2E-10	0.253	0.240					
cg01560464	X	142,721,195	0.010603	0.253	0.255				*SLITRK4*	5'UTR
cg14001023	19	46,526,321	1.58E-08	0.253	0.222				*PGLYRP1*	1stExon
cg08935418	7	158,110,570	1.19E-07	0.253	0.239				*PTPRN2*	Body
cg04255201	19	46,999,307	4.92E-12	0.253	0.256				*PNMAL2*	TSS200
cg19376461	7	110,358,708	1.84E-16	0.253	0.239				*IMMP2L*	Body
cg20984188	16	67,185,059	0.038119	0.253	0.119				*B3GNT9*	TSS200
cg22373225	11	45,234,334	8.8E-62	0.253	0.240				*PRDM11*	Body
cg07269682	11	843,915	1.69E-06	0.253	0.210				*TSPAN4*	TSS1500
cg18435674	17	38,821,155	0.001207	0.253	0.213				*KRT222*	Body
cg25977206	14	97,499,484	0.001561	0.253	0.219					
cg08659204	17	35,242,555	2.12E-17	0.253	0.251					
cg14548272	8	99,961,122	2.28E-07	0.253	0.244				*OSR2*	5'UTR
cg11440030	3	147,137,031	0.019658	0.253	0.340			Yes		
cg22595443	10	14,510,114	1.05E-10	0.253	0.262			Yes		
cg24683414	1	92,952,581	4.09E-06	0.253	0.234				*GFI1*	TSS1500
cg13526469	3	13,245,693	4.39E-06	0.253	0.216					
cg23677243	15	37,387,525	0.000002	0.253	0.301				*MEIS2*	Body
cg06346138	1	17,086,071	1.44E-09	0.253	0.239				*MST1P9*	Body
cg11004890	20	3,218,500	2.39E-14	0.253	0.235	4.58E-08	0.112		*SLC4A11*	TSS200
cg24518098	1	2,146,288	3.93E-17	0.253	0.252					
cg05546763	14	95,983,383	3.69E-19	0.253	0.213					
cg14166009	19	37,825,309	0.00613	0.253	0.147	0.00000109	0.115	Yes	*HKR1*	TSS1500
cg12099279	19	40,732,518	0.024842	0.253	0.080				*CNTD2*	1stExon
cg01414407	1	15,495,100	0.000325	0.253	0.223			Yes	*TMEM51*	5'UTR
cg10626063	19	38,881,032	1.28E-09	0.253	0.246				*SPRED3*	1stExon
cg15032142	6	28,414,780	0.009375	0.253	0.258					
cg06506863	7	150,896,449	1.12E-08	0.253	0.272					

cg24725789	10	119,310,288	0.009983	0.253	0.282					
cg20467929	17	72,321,958	1.57E-08	0.253	0.178				*KIF19*	TSS1500
cg21699330	7	26,193,032	0.006659	0.253	0.150				*NFE2L3*	Body
cg22237425	17	738,822	1.45E-13	0.253	0.240				*NXN*	Body
cg18732172	11	504,301	1.24E-11	0.253	0.233				*RNH1*	5'UTR
cg19083265	17	75,300,284	1.88E-17	0.253	0.217				*SEPT9*	5'UTR
cg07855319	16	69,123,283	0.001798	0.253	0.274					
cg17384889	6	28,227,068	0.001129	0.253	0.228				*NKAPL*	TSS200
cg13598010	7	72,838,776	1.59E-06	0.253	0.220					
cg14087413	9	35,848,330	5.63E-07	0.253	0.252				*TMEM8B*	Body
cg21963436	13	28,541,142	0.009518	0.253	0.196				*CDX2*	Body
cg10784030	1	38,412,688	1.21E-05	0.253	0.244	7.57E-26	0.207		*INPP5B*	5'UTR
cg19924619	1	165,323,692	1.99E-05	0.253	0.269				*LMX1A*	Body
cg01295339	2	74,729,710	4E-05	0.253	0.252				*LBX2*	Body
cg07446753	7	2,527,391	8.98E-15	0.253	0.243					
cg15905656	17	8,584,536	1.45E-05	0.253	0.267					
cg00478361	1	26,552,116	0.000196	0.253	0.260					
cg15622912	5	175,225,021	0.009548	0.253	0.242				*CPLX2*	5'UTR
cg18389639	10	97,049,610	3.39E-07	0.253	0.251				*PDLIM1*	Body
cg27350104	11	70,958,638	1.28E-22	0.253	0.233					
cg05861512	14	94,442,960	0.002846	0.253	0.265					
cg08141518	X	133,680,974	2.88E-05	0.253	0.232				*MIR424*	TSS1500
cg22313574	8	27,468,981	2.21E-06	0.253	0.229				*CLU*	TSS200
cg04832245	6	32,829,144	0.007597	0.253	0.209					
cg03379797	6	138,892,911	0.001511	0.253	0.182				*NHSL1*	1stExon
cg16137147	1	221,067,896	0.022829	0.253	0.257					
cg16299455	14	75,894,309	0.015212	0.253	0.081				*JDP2*	TSS200
cg02772995	2	161,348,781	0.000265	0.253	0.278				*RBMS1*	Body
cg05826255	18	55,019,307	0.000688	0.253	0.232				*ST8SIA3*	TSS1500
cg23369564	1	150,123,734	1.54E-07	0.253	0.256				*PLEKHO1*	Body
cg24360197	15	66,662,775	0.038449	0.253	0.122					
cg25323057	10	88,730,481	0.000921	0.253	0.204				*C10orf116*	3'UTR
cg19488874	2	175,193,551	0.000401	0.253	0.289					
cg09048129	11	119,234,607	5.98E-05	0.253	0.226				*USP2*	1stExon

cg16045423	22	39,377,790	0.000903	0.253	0.253				*APOBEC3B*	TSS1500
cg05479130	3	52,316,296	4.15E-22	0.253	0.244					
cg10167978	15	57,825,722	1.18E-22	0.253	0.242			Yes	*CGNL1*	Body
cg21590108	8	23,609,057	6.12E-12	0.253	0.227					
cg14500300	9	211,689	0.000213	0.253	0.318					
cg23989336	17	79,283,915	1.73E-07	0.253	0.203				*C17orf55*	TSS1500
cg00088688	6	167,560,504	6.68E-11	0.253	0.197					
cg03451178	2	172,864,273	9.5E-09	0.253	0.244				*MAP1D*	TSS1500
cg11584690	19	42,574,196	3.16E-07	0.253	0.222	5.64E-25	0.190			
cg15092219	1	247,511,364	3.83E-05	0.253	0.246					
cg18719157	X	133,680,925	8.35E-08	0.253	0.264				*MGC16121*	TSS1500
cg02587648	1	2,023,258	0.000459	0.253	0.293				*PRKCZ*	Body
cg11124260	10	79,709,078	1.18E-08	0.253	0.215					
cg15393022	1	54,775,837	1.35E-08	0.253	0.221				*SSBP3*	Body
cg20599798	2	31,361,760	0.012312	0.253	0.139				*GALNT14*	TSS200
cg00255142	6	31,830,812	0.045471	0.253	0.249				*NEU1*	TSS200
cg27361914	19	51,567,972	0.000133	0.253	0.255				*KLK13*	Body
cg18450500	14	104,690,201	8.51E-11	0.253	0.239					
cg20696432	17	18,881,907	3.5E-10	0.253	0.220				*SLC5A10*	Body
cg22186831	5	176,544,048	0.04313	0.253	0.265					
cg18871289	17	72,210,278	7.2E-09	0.253	0.202				*MGC16275*	TSS1500
cg05937292	5	77,269,342	0.001318	0.253	0.275					
cg18546384	7	2,077,057	9.52E-22	0.253	0.232				*MAD1L1*	Body
cg21197219	1	3,374,906	6.35E-12	0.253	0.237				*ARHGEF16*	5'UTR
cg18292394	10	82,049,450	4.9E-05	0.253	0.204	2.03E-28	0.236		*MAT1A*	TSS200
cg19391535	7	148,398,020	2.28E-18	0.253	0.244				*CUL1*	5'UTR
cg07177380	4	175,133,151	0.001572	0.253	0.242					
cg13941669	5	156,886,982	0.002141	0.253	0.150				*NIPAL4*	TSS200
cg09292226	6	1,392,250	4.58E-11	0.253	0.217				*FOXF2*	Body
cg18786623	6	29,894,050	2.18E-05	0.253	0.179				*HCG4P6*	TSS1500
cg20709530	20	36,040,249	6.51E-10	0.253	0.270					
cg00083042	10	30,404,617	2.65E-12	0.253	0.241					
cg05996671	3	123,518,635	1.34E-09	0.253	0.232				*MYLK*	5'UTR
cg24981593	2	45,160,253	0.013527	0.253	0.226					

cg19302183	1	56,397,400	8.79E-12	0.253	0.243					
cg25204319	2	12,984,815	4.47E-10	0.253	0.227					
cg01302656	14	29,254,853	0.001398	0.253	0.176				*C14orf23*	Body
cg01695225	2	73,152,672	0.000383	0.253	0.246				*EMX1*	Body
cg06626184	9	126,775,931	8.79E-07	0.253	0.241				*LHX2*	Body
cg15931949	3	138,662,315	1.06E-08	0.253	0.255					
cg19925849	19	54,445,377	0.000501	0.253	0.262				*CACNG7*	Body
cg14682922	5	1,016,768	0.000606	0.253	0.244				*NKD2*	Body
cg17349352	7	45,066,738	1.84E-10	0.253	0.227				*CCM2*	Body
cg11130317	14	103,739,856	4.07E-05	0.253	0.185					
cg26624796	16	30,406,176	5.73E-05	0.253	0.212				*ZNF48*	TSS1500
cg01362455	6	29,855,490	7.72E-21	0.253	0.218				*HLA-H*	Body
cg12426652	21	37,915,056	3.91E-11	0.253	0.236				*CLDN14*	TSS200
cg18917517	2	242,743,958	0.002535	0.253	0.321					
cg00512280	19	2,282,161	0.002631	0.253	0.224				*C19orf35*	5'UTR
cg10497871	14	92,041,114	2.14E-05	0.253	0.224				*C14orf184*	5'UTR
cg15342087	6	30,720,209	4.74E-09	0.253	0.175					
cg20797905	17	78,747,227	3.3E-07	0.253	0.241				*RPTOR*	Body
cg25878366	11	12,767,438	4.75E-22	0.253	0.256				*TEAD1*	5'UTR
cg25510753	7	130,008,536	2.11E-05	0.253	0.211				*CPA5*	3'UTR
cg26140790	10	126,301,281	9.49E-08	0.253	0.269				*LHPP*	Body
cg18887230	7	98,739,627	0.03522	0.253	0.247				*SMURF1*	Body
cg20011326	11	59,333,844	0.024193	0.253	0.251					
cg10274022	19	40,871,697	0.00117	0.253	0.253				*PLD3*	5'UTR
cg11559446	8	141,728,568	5.93E-28	0.253	0.262				*PTK2*	Body
cg04682802	20	11,898,478	0.00958	0.253	0.083				*BTBD3*	5'UTR
cg03454711	8	144,852,716	0.000122	0.252	0.240					
cg05349837	12	54,430,991	3.63E-15	0.252	0.221				*HOXC4*	5'UTR
cg06016684	X	20,134,557	0.008542	0.252	0.227				*MAP7D2*	Body
cg25741215	2	63,287,082	0.034838	0.252	0.318					
cg03244411	6	99,295,790	0.000439	0.252	0.205					
cg11848487	6	48,035,985	3.96E-07	0.252	0.267				*C6orf138*	1stExon
cg02898159	19	5,346,237	4.6E-05	0.252	0.259					
cg23165899	6	19,837,095	0.021786	0.252	0.148				*ID4*	TSS1500

cg26895569	1	204,329,222	4.45E-11	0.252	0.250				*PLEKHA6*	TSS200
cg09024435	3	62,360,893	0.034607	0.252	0.268					
cg08543966	19	35,646,150	0.012292	0.252	0.295				*FXYD5*	5'UTR
cg07533422	5	92,935,283	4.69E-12	0.252	0.245					
cg16117799	4	81,106,857	0.000447	0.252	0.220				*PRDM8*	5'UTR
cg17301216	15	89,920,348	6.42E-09	0.252	0.269				*LOC254559*	TSS1500
cg17827328	1	36,817,299	5.2E-48	0.252	0.241				*STK40*	Body
cg14606328	21	45,177,639	1.33E-09	0.252	0.260				*PDXK*	3'UTR
cg22968966	16	22,959,875	0.00193	0.252	0.254					
cg11685547	9	135,455,303	2.96E-06	0.252	0.256					
cg14789818	1	227,748,712	1.85E-15	0.252	0.232					
cg21880222	2	103,327,770	3.37E-06	0.252	0.299				*SLC9A2*	3'UTR
cg05250119	15	79,123,384	1.11E-08	0.252	0.248					
cg16509239	11	62,212,431	1.03E-12	0.252	0.240				*AHNAK*	Body
cg26544247	2	25,473,781	8.51E-17	0.252	0.227				*DNMT3A*	Body
cg00326908	8	66,755,120	0.005713	0.252	0.161				*PDE7A*	TSS1500
cg03035653	X	99,662,932	0.000138	0.252	0.283				*PCDH19*	1stExon
cg06390484	7	134,575,271	1.69E-10	0.252	0.250				*CALD1*	TSS1500
cg06991300	4	122,301,724	0.000414	0.252	0.235				*QRFPR*	1stExon
cg13356540	1	7,449,057	1.69E-21	0.252	0.234				*CAMTA1*	Body
cg16860686	22	42,311,634	1.65E-07	0.252	0.237					
cg02576468	19	41,074,006	0.017687	0.252	0.139				*SPTBN4*	Body
cg09555124	6	160,451,213	2.05E-13	0.252	0.271				*IGF2R*	Body
cg24205914	10	62,761,575	1.15E-07	0.252	0.198				*RHOBTB1*	TSS1500
cg11932249	X	71,524,953	2.31E-06	0.252	0.225				*CITED1*	5'UTR
cg25698741	10	120,006,292	1.7E-05	0.252	0.252					
cg03982831	1	182,669,315	0.001369	0.252	0.244					
cg20608895	13	109,896,380	0.000749	0.252	0.227					
cg05858042	11	569,232	2.13E-05	0.252	0.282				*MIR210*	TSS1500
cg23883058	2	5,813,866	0.000911	0.252	0.188					
cg24633771	8	104,149,619	0.002131	0.252	0.176				*C8orf56*	Body
cg26314722	1	234,867,300	3.23E-09	0.252	0.234					
cg04548856	14	57,284,528	0.000227	0.252	0.214				*OTX2OS1*	Body
cg15981734	2	200,334,558	3.94E-05	0.252	0.255				*FLJ32063*	Body

cg24753061	18	19,752,362	1.44E-11	0.252	0.222				*GATA6*	Body
cg26977936	6	28,558,022	2.72E-05	0.252	0.270					
cg13391820	12	4,384,404	0.007327	0.252	0.272				*CCND2*	Body
cg01105948	13	32,605,196	0.001484	0.252	0.131				*FRY*	TSS1500
cg01196788	2	19,561,709	2E-16	0.252	0.254					
cg04029933	20	31,068,427	2.23E-38	0.252	0.257				*C20orf112*	5'UTR
cg07007400	12	52,626,831	1.35E-05	0.252	0.273	3.45E-22	0.193		*KRT7*	TSS200
cg13407975	19	18,705,946	3.39E-05	0.252	0.253				*CRLF1*	Body
cg26624134	11	128,761,419	0.003844	0.252	0.166	4.26E-13	0.146		*KCNJ5*	1stExon
cg03221483	17	75,315,081	0.023211	0.252	0.208				*SEPT9*	5'UTR
cg04495354	16	57,294,356	6.72E-06	0.252	0.275				*PLLP*	Body
cg12741645	15	89,167,736	8.28E-05	0.252	0.258				*AEN*	5'UTR
cg14686555	2	6,121,915	0.000375	0.252	0.272				*LOC400940*	TSS200
cg02779944	1	200,004,584	8.38E-06	0.252	0.265				*NR5A2*	Body
cg26621699	4	145,566,903	6.68E-05	0.252	0.224				*HHIP*	TSS1500
cg02101773	20	20,347,593	0.007559	0.252	0.182				*INSM1*	TSS1500
cg02457680	1	154,475,139	0.010384	0.252	0.148				*TDRD10*	5'UTR
cg19047868	17	46,669,485	1.19E-14	0.252	0.235				*LOC404266*	TSS200
cg07865216	10	98,910,008	1.8E-13	0.252	0.266				*SLIT1*	Body
cg06869454	6	3,770,466	2.02E-07	0.252	0.248					
cg06946797	16	11,422,409	7.59E-06	0.252	0.288					
cg14742937	8	39,172,097	5.46E-05	0.252	0.275				*ADAM5P*	TSS200
cg04501840	1	226,737,975	0.004881	0.252	0.238				*C1orf95*	Body
cg02767634	5	179,036,637	4.69E-05	0.252	0.272				*RUFY1*	3'UTR
cg18457851	X	40,104,514	2.48E-06	0.252	0.236					
cg26445608	20	2,780,114	2.17E-17	0.252	0.239				*CPXM1*	Body
cg01324702	5	138,290,007	3.87E-19	0.252	0.215				*SIL1*	Body
cg20073153	10	30,238,815	0.000339	0.252	0.283					
cg22776856	16	3,072,572	1.74E-10	0.252	0.251				*THOC6*	TSS1500
cg23643814	17	2,627,585	0.00039	0.252	0.313					
cg09565397	8	145,543,014	4.3E-15	0.252	0.242				*DGAT1*	Body
cg19131313	8	1,704,013	0.000265	0.252	0.245					
cg03361164	6	100,915,805	0.005106	0.252	0.300					
cg06202585	2	38,325,802	2.79E-06	0.252	0.240					

cg12046414	2	220,379,199	0.011902	0.252	0.207				*ACCN4*	1stExon
cg04688351	2	223,154,140	0.008525	0.252	0.280				*PAX3*	Body
cg21568106	2	66,805,366	0.009078	0.252	0.257					
cg05817758	11	13,030,567	0.004088	0.252	0.107				*RASSF10*	TSS200
cg09050331	8	70,984,917	1.72E-07	0.252	0.243				*PRDM14*	TSS1500
cg10660256	5	78,407,683	5.63E-05	0.252	0.262	1.74E-22	0.261		*BHMT*	5'UTR
cg20114113	10	93,391,963	0.000415	0.252	0.212			Yes	*PPP1R3C*	Body
cg24072451	2	133,888,263	2.13E-15	0.252	0.252				*NCKAP5*	Body
cg27048684	16	27,791,167	0.000742	0.252	0.192				*KIAA0556*	3'UTR
cg01408508	20	26,188,639	0.001302	0.252	0.229					
cg03171465	2	99,553,830	1.34E-05	0.252	0.257				*C2orf55*	TSS1500
cg07644368	5	115,152,785	0.037846	0.252	0.032	0.0009046	0.082		*CDO1*	TSS1500
cg15148933	4	94,756,031	8.47E-06	0.252	0.235					
cg02132714	17	46,656,690	0.00119	0.252	0.235				*HOXB4*	TSS1500
cg08010865	13	24,807,902	2.83E-09	0.252	0.237			Yes	*SPATA13*	Body
cg12627354	11	46,940,434	4.21E-05	0.252	0.227				*LRP4*	TSS1500
cg22795212	1	33,938,026	0.039368	0.252	0.233				*ZSCAN20*	TSS1500
cg19383430	3	187,086,187	0.019798	0.252	0.090				*RTP4*	5'UTR
cg07734975	11	9,780,844	1.34E-14	0.252	0.226					
cg13305336	1	229,366,001	6.48E-05	0.252	0.300					
cg15344504	3	31,677,078	3.18E-15	0.252	0.257				*STT3B*	Body
cg26939195	6	32,077,020	0.000386	0.252	0.223				*TNXB*	5'UTR
cg06862374	2	219,736,549	1.67E-05	0.252	0.236				*WNT6*	Body
cg02202193	6	6,546,851	0.025677	0.252	0.103				*LOC285780*	Body
cg13308964	17	69,029,659	0.000329	0.252	0.254					
cg16028753	1	53,527,435	2.64E-08	0.252	0.192	6.29E-25	0.180		*PODN*	TSS1500
cg01466288	1	119,550,803	0.007052	0.252	0.251					
cg26624914	9	33,448,382	1.4E-19	0.252	0.245	1.07E-16	0.215		*SUGT1P1*	Body
cg02770252	6	14,450,312	1.88E-22	0.252	0.240					
cg11944797	13	99,135,711	1.57E-06	0.252	0.171				*STK24*	Body
cg16343134	6	136,657,129	4.27E-12	0.252	0.252					
cg02383785	7	127,808,848	0.000111	0.252	0.247					
cg12298745	18	76,737,545	6.81E-05	0.252	0.258					
cg10210397	8	42,410,091	9.34E-08	0.252	0.250					

cg14511652	9	133,783,802	7.78E-08	0.252	0.246				*FIBCD1*	Body
cg00711072	17	46,669,489	6.1E-18	0.252	0.237				*LOC404266*	TSS200
cg12382153	15	58,357,204	0.005514	0.252	0.141				*ALDH1A2*	Body
cg20543544	10	81,003,657	0.000369	0.252	0.239				*ZMIZ1*	Body
cg02541778	8	55,382,901	0.041954	0.252	0.320					
cg07016556	17	79,393,532	0.003662	0.252	0.084				*BAHCC1*	Body
cg23477460	3	66,848,765	0.000314	0.252	0.279					
cg02932355	8	102,184,648	3.21E-10	0.252	0.250					
cg03671802	18	56,941,473	0.011422	0.252	0.233				*RAX*	TSS1500
cg08297985	16	85,343,488	1.46E-25	0.252	0.236					
cg01591025	7	25,018,595	2.51E-11	0.252	0.247				*OSBPL3*	5'UTR
cg00490606	8	102,217,748	1.56E-10	0.252	0.247				*ZNF706*	5'UTR
cg15278646	15	28,341,980	0.028081	0.252	0.319				*OCA2*	5'UTR
cg00084094	12	109,096,204	0.000621	0.252	0.264				*CORO1C*	5'UTR
cg09587549	16	31,214,126	0.024654	0.252	0.128	1.47E-10	0.156		*PYCARD*	5'UTR
cg07240834	4	175,139,755	3.55E-07	0.252	0.216					
cg08475096	4	158,143,750	9.58E-06	0.252	0.207				*GRIA2*	Body
cg13032463	6	166,582,585	0.000638	0.252	0.326				*T*	TSS1500
cg18302225	5	55,776,401	9.18E-05	0.252	0.152					
cg01444716	20	11,898,557	0.013053	0.252	0.079				*BTBD3*	5'UTR
cg04092800	5	40,681,444	3.09E-08	0.252	0.259				*PTGER4*	Body
cg06212224	2	175,198,768	0.004602	0.252	0.196				*SP9*	TSS1500
cg07345734	14	36,986,363	0.005956	0.252	0.327				*NKX2-1*	3'UTR
cg19455840	10	102,906,171	0.000249	0.252	0.262			Yes		
cg08358166	17	18,538,271	0.033408	0.252	0.291					
cg11080549	5	92,909,968	5.26E-06	0.252	0.211				*FLJ42709*	Body
cg13514772	8	38,770,414	2.36E-12	0.252	0.261				*PLEKHA2*	5'UTR
cg05393726	3	133,615,119	0.027094	0.252	0.193				*RAB6B*	TSS1500
cg18151012	1	19,600,909	7.25E-05	0.252	0.204				*AKR7L*	TSS1500
cg05133179	11	116,451,176	0.012537	0.252	0.229					
cg23095743	10	104,000,831	0.005008	0.252	0.303				*PITX3*	5'UTR
cg02236679	16	78,782,477	3.8E-18	0.251	0.231				*WWOX*	Body
cg02817030	7	20,833,508	0.005503	0.251	0.248					
cg04167854	11	18,031,127	5.58E-08	0.251	0.259				*SERGEF*	Body

cg16162324	12	104,852,047	4.52E-05	0.251	0.221				*CHST11*	Body
cg24794531	3	142,443,837	0.005971	0.251	0.236	8.69E-18	0.168		*TRPC1*	Body
cg19623418	20	55,499,548	1.53E-09	0.251	0.267					
cg26885268	7	149,389,941	0.00036	0.251	0.248					
cg27641532	6	27,648,317	8.78E-06	0.251	0.296					
cg21999939	5	2,755,004	3.07E-05	0.251	0.268				*C5orf38*	Body
cg21771173	5	169,064,071	0.005019	0.251	0.316				*DOCK2*	TSS200
cg27342781	16	84,566,279	5.92E-13	0.251	0.239					
cg24642844	7	1,081,250	2.32E-19	0.251	0.237				*C7orf50*	Body
cg24701575	11	31,845,389	0.016894	0.251	0.269					
cg14424538	20	1,784,365	0.030576	0.251	0.088					
cg18278184	10	70,847,430	0.015022	0.251	0.083				*SRGN*	TSS1500
cg23323891	14	57,200,519	5.55E-08	0.251	0.213					
cg08293075	6	32,156,294	2.58E-20	0.251	0.244				*PBX2*	Body
cg09385936	14	100,791,876	1.87E-09	0.251	0.249				*C14orf68*	Body
cg17489871	2	439,337	1.03E-07	0.251	0.234					
cg23502162	2	189,839,015	9.22E-13	0.251	0.233				*COL3A1*	TSS200
cg14516100	4	186,560,083	1.17E-05	0.251	0.246				*SORBS2*	Body
cg26718433	10	44,880,730	0.002149	0.251	0.226				*CXCL12*	TSS200
cg12140851	7	26,897,612	6.52E-13	0.251	0.243				*SKAP2*	Body
cg18145759	2	109,901,466	1.94E-16	0.251	0.284				*SH3RF3*	Body
cg18270675	11	85,422,118	7.69E-06	0.251	0.247				*SYTL2*	Body
cg05614333	15	27,111,774	0.000623	0.251	0.242				*GABRA5*	TSS1500
cg05746024	1	154,540,074	0.004968	0.251	0.249				*CHRNB2*	TSS200
cg25738714	1	50,893,658	0.018487	0.251	0.227					
cg18300492	5	2,129,206	0.026747	0.251	0.223					
cg10304824	2	177,028,804	4.05E-05	0.251	0.235				*HOXD3*	1stExon
cg22490780	5	16,867,064	7.24E-14	0.251	0.246				*MYO10*	Body
cg02025879	16	22,326,203	2.81E-15	0.251	0.241				*POLR3E*	Body
cg03356778	11	120,110,544	1.93E-12	0.251	0.232				*POU2F3*	TSS1500
cg05353869	11	75,139,544	0.006374	0.251	0.204				*KLHL35*	Body
cg24446429	18	74,798,054	4.13E-08	0.251	0.206				*MBP*	Body
cg17251265	17	5,487,585	8.2E-11	0.251	0.251			Yes	*NLRP1*	5'UTR
cg11873372	10	130,274,436	0.000272	0.251	0.183					

cg15806857	17	2,305,903	0.000589	0.251	0.254					
cg10653163	12	109,124,059	0.000222	0.251	0.107				*CORO1C*	5'UTR
cg17470251	8	19,459,996	1.26E-06	0.251	0.230				*CSGALNACT1*	1stExon
cg04477010	8	38,175,544	2.07E-16	0.251	0.246				*WHSC1L1*	Body
cg06707663	11	61,381,267	6.98E-09	0.251	0.252				*RPLP0P2*	TSS1500
cg26767389	13	30,594,417	1.73E-17	0.251	0.244					
cg18944047	17	72,322,143	0.006615	0.251	0.187				*KIF19*	TSS1500
cg12787405	20	42,955,658	6.63E-05	0.251	0.244					
cg03287111	2	121,625,634	4.01E-06	0.251	0.250			Yes	*GLI2*	Body
cg09638944	3	149,424,680	1.23E-11	0.251	0.285					
cg02672493	3	136,537,709	0.000874	0.251	0.282	1.37E-33	0.249		*TMEM22*	TSS200
cg03857553	22	29,976,613	0.000205	0.251	0.138				*NIPSNAP1*	Body
cg24885937	11	16,898,482	0.000568	0.251	0.224				*PLEKHA7*	Body
cg05875017	2	233,390,771	4.95E-05	0.251	0.230				*CHRND*	TSS200
cg17186066	8	80,740,613	0.003697	0.251	0.240					
cg08196032	14	95,239,751	0.017278	0.251	0.258					
cg11739633	1	156,459,986	1.62E-07	0.251	0.248				*MEF2D*	5'UTR
cg01612824	14	56,313,920	5.06E-11	0.251	0.258					
cg04478905	21	37,915,091	1.08E-12	0.251	0.226				*CLDN14*	TSS200
cg15531512	8	123,706,630	5.16E-07	0.251	0.279					
cg19351026	2	63,285,425	0.010024	0.251	0.269					
cg15645287	10	34,569,552	1.04E-05	0.251	0.254				*PARD3*	Body
cg17823004	5	34,804,746	0.001915	0.251	0.221				*RAI14*	Body
cg15700437	1	152,487,844	0.003462	0.251	0.264				*CRCT1*	5'UTR
cg15264806	18	59,000,800	0.019137	0.251	0.245					
cg01597480	7	120,629,404	4.92E-09	0.251	0.225				*C7orf58*	5'UTR
cg10635895	12	2,339,439	3.38E-15	0.251	0.245				*CACNA1C*	Body
cg14928932	6	33,401,520	2.94E-13	0.251	0.255				*SYNGAP1*	Body
cg16683746	8	93,113,829	0.000288	0.251	0.279					
cg22891862	10	101,279,697	3.23E-05	0.251	0.232					
cg21239997	2	24,398,519	1.13E-07	0.251	0.240				*C2orf84*	Body
cg06779449	20	42,544,663	1.28E-07	0.251	0.249				*TOX2*	TSS200
cg14912728	17	15,164,634	0.006828	0.251	0.216				*PMP22*	5'UTR
cg08071609	6	117,086,040	0.000105	0.251	0.268				*FAM162B*	Body

cg10123421	19	11,785,062	5.56E-06	0.251	0.198				*ZNF833*	Body
cg19020855	17	41,669,266	6.44E-16	0.251	0.248					
cg03480346	2	24,232,603	0.00856	0.251	0.159				*MFSD2B*	TSS1500
cg13722700	10	46,993,502	1.48E-08	0.251	0.236				*GPRIN2*	TSS200
cg15991175	3	52,313,923	2.61E-05	0.251	0.219				*WDR82*	TSS1500
cg16651877	1	45,276,338	5.53E-15	0.251	0.250				*BTBD19*	Body
cg18663382	6	30,418,789	0.000103	0.251	0.252					
cg14067419	9	98,783,308	0.007673	0.251	0.196				*NCRNA00092*	Body
cg01253160	11	124,747,172	8.02E-05	0.251	0.301				*ROBO3*	Body
cg04638014	4	57,371,368	0.002734	0.251	0.278				*ARL9*	TSS200
cg14942424	20	55,207,002	0.000934	0.251	0.171				*TFAP2C*	Body
cg15008124	12	114,337,844	1.11E-06	0.251	0.244				*RBM19*	Body
cg00371418	17	35,014,438	0.00376	0.251	0.191					
cg04517738	8	66,196,608	0.001137	0.251	0.263					
cg08563601	2	215,675,518	3.16E-10	0.251	0.166				*BARD1*	TSS1500
cg11136654	2	235,286,867	5.84E-07	0.251	0.273					
cg12646649	10	102,987,257	0.000158	0.251	0.270				*LBX1*	Body
cg10552523	11	313,478	8.12E-11	0.251	0.212				*IFITM1*	TSS1500
cg13066983	3	68,057,306	0.006685	0.251	0.239				*FAM19A1*	Body
cg13569342	12	44,255,515	3.2E-16	0.251	0.241				*TMEM117*	Body
cg26768558	16	88,751,897	2.84E-13	0.251	0.239				*MGC23284*	Body
cg20036706	10	131,711,529	2.91E-13	0.251	0.242				*EBF3*	Body
cg20848988	7	36,247,779	1.99E-06	0.251	0.249				*EEPD1*	Body
cg26440142	1	221,057,573	1.05E-14	0.251	0.229				*HLX*	Body
cg04640675	16	30,572,739	0.006406	0.251	0.237					
cg12103037	14	36,985,711	1.79E-05	0.251	0.265				*NKX2-1*	3'UTR
cg12880658	5	115,152,386	0.012591	0.251	0.147	6.31E-09	0.160		*CDO1*	1stExon
cg22670329	4	74,702,075	2.29E-08	0.251	0.239	1.24E-18	0.181		*CXCL6*	TSS200
cg00376544	17	6,679,348	0.015673	0.251	0.174				*FBXO39*	TSS1500
cg11472521	17	48,050,512	1.35E-05	0.251	0.219				*DLX4*	Body
cg26196626	8	121,989,053	1.34E-07	0.251	0.211					
cg03133266	22	23,908,776	0.022552	0.251	0.271					
cg02282833	5	32,726,189	1.3E-07	0.251	0.246				*NPR3*	Body
cg02123734	10	50,649,666	4.1E-07	0.251	0.220					

cg03943605	10	76,818,994	8.69E-18	0.251	0.245				*DUPD1*	TSS1500
cg05347431	X	132,547,568	0.007876	0.251	0.018				*GPC4*	Body
cg13522882	2	102,316,496	0.00118	0.251	0.234				*MAP4K4*	Body
cg11429044	19	13,947,473	6.46E-08	0.251	0.256				*MIR23A*	TSS200
cg13476058	6	28,557,210	0.014203	0.251	0.197					
cg00240880	20	43,343,233	1.67E-07	0.251	0.265	2.01E-25	0.190		*WISP2*	TSS1500
cg07660236	6	28,367,883	6.62E-06	0.251	0.221				*ZSCAN12*	TSS1500
cg08855395	1	231,296,818	0.00163	0.251	0.259					
cg21028946	20	34,680,004	5.14E-10	0.251	0.188					
cg13106484	7	157,692,127	7.21E-11	0.251	0.254				*PTPRN2*	Body
cg00201414	13	52,769,768	0.000271	0.251	0.295				*THSD1P*	TSS1500
cg04304802	10	73,499,965	8.54E-05	0.251	0.233				*CDH23*	Body
cg26988215	2	31,361,757	0.038388	0.251	0.137				*GALNT14*	TSS200
cg15531433	10	1,639,090	1.61E-33	0.251	0.233				*ADARB2*	Body
cg26839871	1	171,811,299	0.00791	0.251	0.150				*DNM3*	Body
cg09626521	3	113,933,104	0.030639	0.251	0.133					
cg16678099	17	40,250,199	0.003902	0.251	0.270					
cg26991025	15	99,091,228	1.08E-05	0.251	0.230					
cg18946473	2	97,152,025	0.001116	0.251	0.236					
cg04213841	13	49,792,685	0.00188	0.251	0.215					
cg01024668	15	69,099,680	9.91E-15	0.251	0.246				*C15orf28*	TSS1500
cg11147278	10	34,062,234	0.002177	0.251	0.243					
cg18152523	11	93,063,302	0.009154	0.251	0.152				*CCDC67*	TSS1500
cg17215278	1	117,114,263	4.15E-10	0.250	0.224				*CD58*	TSS1500
cg01142676	11	117,695,591	0.003541	0.250	0.267				*FXYD2*	Body
cg17918558	1	46,945,595	3.82E-11	0.250	0.248					
cg04807106	3	124,103,560	1.54E-08	0.250	0.218				*KALRN*	Body
cg11362604	15	37,389,585	1.87E-05	0.250	0.292				*MEIS2*	Body
cg21292068	7	127,907,884	7.6E-19	0.250	0.248					
cg09010707	12	26,392,567	2.62E-07	0.250	0.261					
cg15878670	12	49,485,085	8.37E-05	0.250	0.220				*DHH*	Body
cg19885037	3	4,762,242	0.000928	0.250	0.095				*ITPR1*	Body
cg06523744	22	39,410,145	4.96E-05	0.250	0.236				*APOBEC3C*	TSS200
cg06026136	6	27,173,753	0.010148	0.250	0.261					

cg05867448	X	130,192,575	0.044381	0.250	0.189				*FLJ30058*	5'UTR
cg07919443	10	102,419,409	0.000754	0.250	0.264					
cg13033814	6	31,830,035	0.017879	0.250	0.191				*NEU1*	Body
cg13466694	9	129,380,728	9.38E-09	0.250	0.248				*LMX1B*	Body
cg03963219	2	84,709,147	5.11E-12	0.250	0.244					
cg04353483	4	26,493,496	1.86E-09	0.250	0.237	1.74E-11	0.171		*CCKAR*	TSS1500
cg24770624	2	20,068,871	0.01826	0.250	0.106					
cg01911077	11	64,531,743	6.17E-12	0.250	0.247					
cg13762082	5	31,640,096	1.59E-05	0.250	0.236					
cg14016177	9	131,965,610	0.000643	0.250	0.166					
cg24752267	20	20,352,002	0.000266	0.250	0.189					
cg04111071	7	19,813,299	0.003839	0.250	0.250				*TMEM196*	TSS1500
cg05200628	1	160,681,760	0.004955	0.250	0.264				*CD48*	TSS200
cg00096104	13	100,547,897	0.001189	0.250	0.231				*CLYBL*	3'UTR
cg08813925	10	31,996,740	0.019172	0.250	0.271					
cg02798394	2	19,974,694	5.43E-13	0.250	0.222					
cg03655683	2	63,281,383	4.91E-05	0.250	0.228				*OTX1*	Body
cg00954841	12	3,373,534	2.94E-08	0.250	0.211				*TSPAN9*	Body
cg06397161	22	39,760,059	1.34E-16	0.250	0.243			Yes	*SYNGR1*	Body
cg17406383	17	71,948,506	6.6E-14	0.250	0.222					
cg20395789	11	107,570,881	2.91E-14	0.250	0.252					
cg07484739	2	177,356,020	4.53E-08	0.250	0.210					
cg07581883	3	125,676,865	0.00029	0.250	0.194					
cg20971970	12	57,632,495	1.64E-07	0.250	0.253				*NDUFA4L2*	5'UTR
cg03171003	2	111,875,934	9.25E-06	0.250	0.262					
cg13808012	1	244,094,935	9.84E-15	0.250	0.224					
cg24160421	8	48,675,771	0.00022	0.250	0.186					
cg07450037	7	27,135,147	2.06E-07	0.250	0.239				*HOXA1*	Body
cg26019295	12	54,409,212	0.039463	0.250	0.256				*HOXC4*	TSS1500
cg24581462	8	38,759,627	0.000646	0.250	0.192				*PLEKHA2*	5'UTR
cg08194879	6	123,317,569	0.010602	0.250	0.234				*CLVS2*	TSS200
cg24467349	1	110,254,835	8.66E-06	0.250	0.256				*GSTM5*	TSS200
cg01961105	16	54,968,783	0.007915	0.250	0.317					
cg03689092	2	128,439,345	7.59E-16	0.250	0.263				*LIMS2*	1stExon

cg00222341	15	83,951,913	0.000256	0.250	0.240				*BNC1*	Body
cg25365014	5	2,727,713	1.33E-05	0.250	0.266					
cg17353698	6	31,831,599	0.001514	0.250	0.202				*NEU1*	TSS1500
cg05438378	15	67,383,736	1.64E-05	0.250	0.233				*SMAD3*	Body
cg01748627	11	67,272,202	0.001245	0.250	0.144				*PITPNM1*	5'UTR
cg04780086	2	111,875,790	0.000102	0.250	0.256				*ACOXL*	3'UTR
cg27560796	6	33,864,689	4.28E-11	0.250	0.195					
cg05208605	11	116,451,437	1.85E-05	0.250	0.237					
cg15056105	18	13,611,489	0.000299	0.250	0.206				*C18orf1*	TSS200
cg21611682	11	68,138,269	1.22E-05	0.250	0.219				*LRP5*	Body
cg02976588	1	150,135,546	0.006955	0.250	0.311					
cg14062261	7	156,736,159	0.000247	0.250	0.292					
cg21699894	20	42,545,443	1.93E-07	0.250	0.228				*TOX2*	5'UTR
cg23237314	6	29,894,197	1.75E-06	0.250	0.240				*HCG4P6*	TSS1500
cg20556402	6	41,616,858	1.35E-10	0.250	0.248				*MDFI*	Body
cg07546293	16	851,255	0.000125	0.250	0.236			Yes	*GNG13*	TSS1500
cg20161791	7	2,558,431	1.57E-07	0.250	0.257				*LFNG*	Body
cg01161204	11	115,530,932	3.43E-07	0.250	0.233					
cg06323896	16	85,410,525	7.25E-09	0.250	0.244					
cg00897875	17	43,392,165	0.000157	0.250	0.200				*MAP3K14*	5'UTR
cg02055540	5	165,510,804	6.82E-11	0.250	0.210					
cg22354646	3	58,550,723	0.001731	0.250	0.266				*FAM107A*	3'UTR
cg04500239	1	118,502,178	2.39E-06	0.250	0.233				*WDR3*	3'UTR
cg07063883	16	3,079,953	8.52E-43	0.250	0.246				*CCDC64B*	Body
cg13523576	8	21,909,636	2.36E-15	0.250	0.248				*EPB49*	TSS1500
cg26508444	10	126,330,845	7.21E-12	0.250	0.226				*FAM53B*	Body
cg27302263	15	66,915,218	1.38E-06	0.250	0.311					
cg02730492	13	110,874,299	5.6E-05	0.250	0.248				*COL4A1*	Body
cg05123976	17	45,949,677	7.27E-08	0.250	0.233					
cg06081199	7	117,120,064	4.63E-06	0.250	0.227				*CFTR*	1stExon
cg13643594	X	107,681,825	5.76E-10	0.250	0.230				*COL4A5*	TSS1500
cg22828581	9	93,880,502	0.000969	0.250	0.199					
cg26927190	17	76,875,678	1.76E-07	0.250	0.252				*TIMP2*	Body
cg09123188	15	100,913,934	8.25E-05	0.250	0.188					

cg00093643	15	44,579,510	7.84E-16	0.250	0.257				*CASC4*	TSS1500
cg15474402	4	188,953,969	1.22E-06	0.250	0.265					
cg16704246	10	112,594,670	1.13E-15	0.250	0.234				*RBM20*	Body
cg21415530	8	140,715,802	3.62E-05	0.250	0.278				*KCNK9*	TSS1500
cg01219426	11	95,856,658	9.41E-09	0.250	0.206				*MAML2*	Body
cg09069072	1	15,482,753	1.58E-09	0.250	0.264				*TMEM51*	5'UTR
cg23424407	12	115,131,846	1.87E-11	0.250	0.252					
cg01683794	14	97,059,349	0.00143	0.250	0.204					
cg23118086	15	65,360,058	0.001766	0.250	0.259				*RASL12*	Body
cg23900203	12	127,765,331	0.020208	0.250	0.216					
cg01078408	6	3,232,129	0.004586	0.250	0.167					
cg08936706	3	46,906,385	1.15E-07	0.250	0.260				*MYL3*	TSS1500
cg09511190	17	33,416,862	5.81E-07	0.250	0.199				*RFFL*	TSS1500
cg13725826	14	100,211,213	9.2E-06	0.250	0.263					
cg23730575	6	27,799,385	0.001636	0.250	0.209				*HIST1H4K*	TSS200
cg08095452	17	55,958,800	2.9E-18	0.250	0.235				*CUEDC1*	Body
cg16232183	13	96,204,854	0.020725	0.250	0.248				*CLDN10*	TSS200
cg20039443	20	50,108,912	1.26E-08	0.250	0.250				*NFATC2*	Body
cg25799059	3	45,134,300	1.34E-05	0.250	0.224				*CDCP1*	Body
cg26081841	5	87,441,969	0.000992	0.250	0.301					
cg26906629	11	68,564,367	5.11E-09	0.250	0.256				*CPT1A*	Body
cg10770742	7	151,107,285	0.040298	0.250	0.351				*WDR86*	TSS200
cg09942999	1	32,707,389	8.45E-05	0.250	0.179				*MTMR9L*	TSS200
cg24683222	4	41,880,440	2.74E-10	0.250	0.249					
cg16293656	8	21,900,438	9.26E-05	0.250	0.195	5.15E-15	0.140		*FGF17*	5'UTR
cg08781365	16	85,320,072	4.35E-08	0.250	0.245					
cg18717967	8	8,182,747	1.66E-24	0.250	0.241				*PRAGMIN*	Body
cg09161043	1	53,068,834	1.6E-13	0.250	0.240				*GPX7*	Body
cg09715285	2	220,108,267	0.004657	0.250	0.165				*GLB1L*	Body
cg12717533	2	26,397,154	1.39E-07	0.250	0.220				*FAM59B*	Body
cg07937271	4	13,542,719	0.001872	0.250	0.255				*NKX3-2*	3'UTR
cg10350722	12	3,490,214	1.88E-09	0.250	0.255					
cg03100639	16	31,483,137	6.23E-10	0.250	0.210				*TGFB1I1*	TSS1500
cg10281996	11	69,290,977	9.56E-21	0.250	0.236					

cg24702341	7	73,895,278	1.25E-08	0.250	0.235				*GTF2IRD1*	5'UTR
cg11520719	6	157,391,679	5.76E-21	0.250	0.236				*ARID1B*	Body
cg17427491	7	190,423	1.21E-13	0.250	0.219					
cg26366091	1	111,770,274	9.69E-05	0.250	0.244	5.31E-10	0.162		*CHI3L2*	TSS200
cg15058210	2	240,196,877	2.37E-06	0.250	0.218				*HDAC4*	Body
cg23870587	14	91,060,122	2.9E-09	0.250	0.236				*TTC7B*	Body
cg12052765	10	50,816,963	0.038321	0.250	0.310	2.7E-22	0.190		*SLC18A3*	TSS1500
cg20624391	6	105,584,149	0.033983	0.250	0.127	1.33E-11	0.181		*BVES*	5'UTR
cg02300379	10	95,536,738	5.73E-05	0.250	0.246				*LGI1*	Body
cg08350549	3	99,591,722	1.01E-09	0.250	0.189				*C3orf26*	Body
cg17154395	17	37,752,605	0.008004	0.250	0.232					
cg26020069	6	52,382,441	1.18E-06	0.250	0.297				*TRAM2*	Body
cg05241461	19	22,816,980	0.017881	0.250	0.247				*ZNF492*	TSS200
cg15677364	6	30,687,221	5.25E-08	0.250	0.229				*TUBB*	TSS1500
cg20068058	11	46,317,859	3.69E-09	0.250	0.243				*CREB3L1*	Body
cg15914863	7	1,022,675	6.48E-19	0.250	0.234	4.28E-21	0.173		*CYP2W1*	TSS200
cg19428417	16	66,959,729	1.93E-08	0.250	0.234	2.88E-21	0.161		*RRAD*	TSS1500
cg08048268	3	133,502,702	0.003876	0.250	0.271					
cg00890208	9	37,025,102	1.63E-07	0.250	0.211				*PAX5*	Body
cg06564232	6	50,810,993	0.001699	0.250	0.305				*TFAP2B*	Body
cg19423543	5	172,655,948	0.000541	0.250	0.256					
cg20386487	15	96,898,045	0.00123	0.250	0.263					
cg21433125	7	18,535,786	0.003436	0.250	0.206				*HDAC9*	TSS200
cg02058267	14	76,605,088	0.009339	0.250	0.223					
cg07739369	17	80,241,456	3.61E-10	0.250	0.227					
cg14558529	6	27,648,341	0.00173	0.250	0.294					
cg17297305	1	185,287,148	1.31E-06	0.250	0.211				*IVNS1ABP*	TSS1500
cg02839593	16	66,934,539	2.35E-20	0.250	0.231					
cg15885337	11	132,813,819	0.035742	0.250	0.326				*OPCML*	TSS1500
cg03351003	16	75,272,306	4.33E-16	0.250	0.235				*BCAR1*	Body
cg26378982	2	11,053,937	1.53E-62	0.250	0.241				*KCNF1*	1stExon
cg12338417	3	32,860,178	0.006888	0.250	0.196				*TRIM71*	1stExon
cg08891071	5	150,399,909	0.00014	0.250	0.141				*GPX3*	TSS200
cg24860534	1	227,506,868	0.01116	0.250	0.121	2.75E-16	0.160		*CDC42BPA*	TSS1500

cg16450577	17	80,806,375	2.88E-07	0.250	0.279			Yes	*TBCD*	Body
cg07447868	X	17,395,220	0.006183	0.250	0.156				*NHS*	Body
cg06128608	9	136,834,256	2.79E-17	0.250	0.226				*VAV2*	Body
cg14657834	8	67,875,441	0.000514	0.250	0.247					
cg15983026	7	155,325,552	3.18E-08	0.249	0.237				*CNPY1*	5'UTR
cg02213260	14	23,834,995	4.24E-36	0.249	0.226				*EFS*	TSS200
cg25666919	7	102,074,615	0.001578	0.249	0.128				*ORAI2*	5'UTR
cg23467008	6	152,128,537	0.018011	0.249	0.154				*ESR1*	TSS1500
cg05913906	6	30,923,370	0.013311	0.249	0.325					
cg00050375	6	43,404,828	6.22E-08	0.249	0.245				*ABCC10*	Body
cg03115558	10	72,200,789	9.42E-05	0.249	0.261				*NODAL*	Body
cg17200137	10	48,416,896	3.29E-19	0.249	0.249				*GDF2*	TSS200
cg21857598	5	66,492,721	1.13E-05	0.249	0.252				*CD180*	TSS200
cg21890239	X	136,509,643	0.047397	0.249	0.274					
cg14003627	9	123,204,555	3.27E-33	0.249	0.248				*CDK5RAP2*	Body
cg18103859	14	61,110,002	6.69E-06	0.249	0.263					
cg04685442	8	49,776,492	8.9E-13	0.249	0.259					
cg00473633	17	75,260,903	0.00019	0.249	0.285					
cg06604589	17	800,512	1.41E-06	0.249	0.221				*NXN*	Body
cg13850606	1	229,543,554	0.025966	0.249	0.330					
cg06022664	15	69,136,313	0.029629	0.249	0.257				*MIR548H4*	Body
cg19709468	20	21,082,942	0.015879	0.249	0.302					
cg00044097	2	106,055,153	1.36E-09	0.249	0.247				*FHL2*	1stExon
cg20355301	9	97,848,107	1.68E-05	0.249	0.223				*C9orf3*	3'UTR
cg17221315	6	27,791,827	0.01442	0.249	0.187				*HIST1H4J*	TSS200
cg01644731	12	58,236,712	1.23E-29	0.249	0.252				*CTDSP2*	Body
cg09543255	3	170,302,640	0.013445	0.249	0.337				*SLC7A14*	5'UTR
cg07017214	6	30,433,749	0.001175	0.249	0.229					
cg01521220	17	46,233,799	1.88E-09	0.249	0.224				*MIR1203*	Body
cg08271229	1	2,222,674	1.25E-06	0.249	0.248				*SKI*	Body
cg07727170	15	70,458,214	3.01E-18	0.249	0.250					
cg08235161	14	57,262,049	2.17E-06	0.249	0.243					
cg11425788	1	60,136,100	2.43E-08	0.249	0.261				*FGGY*	Body
cg15105476	6	1,335,849	0.000908	0.249	0.161			Yes		

cg18977727	22	37,952,950	1.53E-07	0.249	0.279					
cg20491914	2	26,915,349	0.001776	0.249	0.166				*KCNK3*	TSS1500
cg07023704	2	172,965,890	1.32E-06	0.249	0.254				*DLX2*	Body
cg03297783	3	50,378,664	0.000309	0.249	0.257				*RASSF1*	TSS1500
cg16342439	3	126,006,835	0.012669	0.249	0.148					
cg11412288	6	117,802,725	1.38E-05	0.249	0.259				*DCBLD1*	TSS1500
cg13241681	13	114,187,990	2.81E-05	0.249	0.261				*TMCO3*	Body
cg20321153	1	63,795,435	2.54E-06	0.249	0.269					
cg00980058	1	233,248,709	1.12E-09	0.249	0.252				*PCNXL2*	Body
cg19049194	2	175,193,754	0.000286	0.249	0.220					
cg12287358	6	29,796,032	0.010479	0.249	0.289				*HLA-G*	Body
cg20670679	7	157,561,786	1.53E-08	0.249	0.249				*PTPRN2*	Body
cg23040305	1	183,846,243	2.34E-10	0.249	0.270				*RGL1*	Body
cg22129545	2	27,531,320	1.78E-18	0.249	0.261				*UCN*	TSS200
cg10985145	5	135,227,049	4.64E-20	0.249	0.236					
cg20731469	6	108,495,678	3.27E-05	0.249	0.252				*NR2E1*	Body
cg00025496	17	1,057,343	0.020372	0.249	0.296				*ABR*	Body
cg20245822	12	104,734,362	1.89E-15	0.249	0.237				*TXNRD1*	Body
cg00040455	X	30,326,676	0.016416	0.249	0.375				*NR0B1*	1stExon
cg07414961	17	70,587,753	0.004596	0.249	0.055					
cg11428724	1	18,957,632	0.047915	0.249	0.181	3.01E-14	0.173		*PAX7*	5'UTR
cg13932501	9	94,060,853	0.000329	0.249	0.262				*AUH*	Body
cg20132775	3	142,444,202	8.97E-06	0.249	0.241				*TRPC1*	Body
cg23871507	6	44,528,765	7.83E-20	0.249	0.226					
cg26984805	10	71,176,501	7.58E-13	0.249	0.239	9.44E-22	0.177		*TACR2*	1stExon
cg04571189	2	10,638,072	0.00045	0.249	0.214					
cg21604450	1	66,998,563	4.58E-05	0.249	0.193				*SGIP1*	TSS1500
cg24844534	17	77,020,267	0.004739	0.249	0.215	9.53E-24	0.195		*C1QTNF1*	1stExon
cg16751451	7	55,086,091	1.47E-11	0.249	0.208				*EGFR*	TSS1500
cg18803306	20	623,421	7.76E-05	0.249	0.169					
cg00394180	8	97,167,170	0.000254	0.249	0.244				*GDF6*	Body
cg22727783	5	14,441,074	0.000236	0.249	0.220				*TRIO*	Body
cg26507725	11	69,987,633	7.8E-15	0.249	0.269				*ANO1*	Body
cg14752336	10	106,400,454	0.002552	0.249	0.173				*SORCS3*	TSS1500

cg02149446	19	47,812,519	1.57E-09	0.249	0.263	8.79E-21	0.214		*C5AR1*	TSS1500
cg09820180	6	140,168,822	4.22E-05	0.249	0.262					
cg03619352	5	179,219,098	1.07E-05	0.249	0.271					
cg05729200	2	102,091,321	1.53E-10	0.249	0.226				*RFX8*	TSS200
cg27638615	2	202,901,352	4.25E-08	0.249	0.228				*FZD7*	1stExon
cg03740167	10	23,463,746	0.000396	0.249	0.275					
cg12007166	6	30,228,058	0.000282	0.249	0.214				*HLA-L*	Body
cg14231297	19	58,629,901	0.014008	0.249	0.284				*ZSCAN18*	TSS200
cg19416570	19	58,715,677	2.57E-13	0.249	0.248				*ZNF274*	Body
cg10002915	4	149,369,175	5E-10	0.249	0.229					
cg16440442	5	149,980,671	1.76E-05	0.249	0.251				*SYNPO*	1stExon
cg15249639	12	4,382,174	0.023799	0.249	0.236				*CCND2*	TSS1500
cg04377609	3	9,358,914	0.000123	0.249	0.184					
cg14467181	17	47,516,312	0.000754	0.249	0.289					
cg17649293	2	71,134,791	0.002287	0.249	0.182				*VAX2*	Body
cg00310215	16	51,185,346	0.002118	0.249	0.177				*SALL1*	TSS1500
cg11201751	16	85,158,047	8.47E-15	0.249	0.265					
cg26342637	20	43,743,872	5.18E-11	0.249	0.253				*WFDC5*	TSS200
cg04314624	6	28,458,328	0.03373	0.249	0.231					
cg13561855	14	52,735,840	5.5E-06	0.249	0.271				*PTGDR*	Body
cg09615353	4	187,026,710	0.029015	0.249	0.192					
cg24590788	13	95,655,324	0.028331	0.249	0.132					
cg01093878	2	131,512,663	0.005128	0.249	0.281				*FAM123C*	TSS1500
cg07068674	14	37,123,778	1.08E-05	0.249	0.253					
cg22736718	5	691,229	0.000614	0.249	0.278				*TPPP*	5'UTR
cg13066963	1	6,485,317	2.89E-05	0.249	0.256	2.58E-18	0.168		*ESPN*	Body
cg05372242	14	36,983,542	0.014736	0.249	0.307				*SFTA3*	TSS1500
cg11095319	11	19,263,864	0.004986	0.249	0.254				*E2F8*	TSS1500
cg17743381	1	39,024,825	2.65E-18	0.249	0.235					
cg26541218	7	47,826,387	5.02E-06	0.249	0.229				*PKD1L1*	Body
cg14471064	9	139,236,663	1.21E-15	0.249	0.247				*GPSM1*	Body
cg17553353	3	111,718,565	6.31E-09	0.249	0.230				*TAGLN3*	Body
cg24375399	17	40,825,798	3.38E-27	0.249	0.242				*PLEKHH3*	Body
cg00632374	X	46,432,929	0.017241	0.249	0.159				*CHST7*	TSS1500

cg15246085	3	49,460,111	5.3E-14	0.249	0.265				*AMT*	TSS200
cg27041049	14	31,345,003	1.17E-14	0.249	0.229				*COCH*	Body
cg14420519	9	139,515,751	1.46E-05	0.249	0.215					
cg16547027	18	24,127,588	3.56E-09	0.249	0.206				*KCTD1*	5'UTR
cg19130973	3	12,979,917	1.03E-14	0.249	0.242				*IQSEC1*	Body
cg06578342	16	4,349,215	0.006616	0.249	0.271					
cg16602747	10	119,313,075	8.46E-05	0.249	0.216					
cg17068623	8	144,853,065	4.53E-05	0.249	0.225					
cg21234471	9	92,023,797	5.82E-11	0.249	0.233				*SEMA4D*	5'UTR
cg27240158	11	12,698,019	7.05E-09	0.249	0.257				*TEAD1*	5'UTR
cg13530938	2	239,140,190	0.000129	0.249	0.232				*LOC151174*	Body
cg06653026	7	84,892,267	9.1E-11	0.249	0.243					
cg23177095	17	30,873,196	2.73E-07	0.249	0.256				*MYO1D*	Body
cg06014588	15	88,801,339	1.22E-05	0.249	0.246					
cg08390209	9	22,005,563	4.06E-12	0.249	0.209	3.77E-23	0.170		*CDKN2BAS*	Body
cg08745965	5	135,416,529	6.96E-05	0.249	0.222				*MIR886*	TSS1500
cg10305789	20	37,434,167	0.000699	0.249	0.166				*PPP1R16B*	TSS200
cg12746557	21	39,644,332	1.02E-10	0.249	0.227				*KCNJ15*	5'UTR
cg11055991	7	158,280,410	9.71E-05	0.249	0.243				*PTPRN2*	Body
cg05828005	6	28,504,908	1.3E-07	0.249	0.245					
cg18512948	14	24,641,706	1.49E-12	0.249	0.229				*REC8*	1stExon
cg01804679	1	32,707,303	0.000493	0.249	0.170				*MTMR9L*	Body
cg20935165	2	172,972,840	6.63E-05	0.249	0.238					
cg12786198	14	55,741,714	4.96E-10	0.249	0.266				*FBXO34*	5'UTR
cg27426707	17	48,639,585	5.13E-05	0.249	0.263	2.62E-34	0.209		*CACNA1G*	Body
cg05689208	8	49,496,391	3.48E-19	0.249	0.272					
cg12302402	11	18,230,629	0.000478	0.249	0.290				*LOC494141*	TSS200
cg09279544	19	8,674,388	0.001342	0.249	0.302				*ADAMTS10*	5'UTR
cg11170179	19	51,522,767	3.44E-14	0.249	0.220	8.23E-22	0.150		*KLK10*	5'UTR
cg07937427	15	59,592,763	0.031456	0.249	0.225				*MYO1E*	Body
cg02782426	3	40,428,986	0.02341	0.249	0.189				*ENTPD3*	5'UTR
cg14904923	8	122,651,570	0.001247	0.249	0.237				*HAS2*	5'UTR
cg16979070	6	150,463,417	0.000129	0.249	0.157				*PPP1R14C*	TSS1500
cg27621745	8	82,671,510	0.00012	0.249	0.237				*CHMP4C*	3'UTR

cg17542408	11	78,673,100	0.006543	0.249	0.199				*ODZ4*	Body
cg17840719	5	178,368,253	0.033747	0.249	0.187				*ZNF454*	1stExon
cg18093572	5	122,598,329	1.7E-12	0.249	0.234					
cg24835051	1	15,480,853	1.01E-06	0.249	0.229				*TMEM51*	5'UTR
cg15154229	7	130,020,011	4.32E-13	0.249	0.242	2.04E-22	0.180		*CPA1*	TSS1500
cg21650866	8	142,297,256	6.02E-05	0.249	0.278					
cg07196992	4	78,098,654	4.24E-14	0.249	0.251					
cg10395252	14	76,236,901	8.7E-26	0.249	0.250				*TTLL5*	Body
cg17429000	5	76,936,636	0.007715	0.249	0.303					
cg24127861	14	24,640,947	1.44E-07	0.249	0.260				*REC8*	TSS1500
cg14665413	4	38,859,728	0.000311	0.249	0.308				*TLR6*	TSS1500
cg24745495	19	15,344,239	1.44E-08	0.249	0.235				*EPHX3*	TSS1500
cg09530810	17	46,831,490	8.51E-07	0.249	0.240					
cg09560979	8	38,615,235	0.000423	0.249	0.156				*TACC1*	5'UTR
cg02358362	5	172,096,266	1.18E-15	0.249	0.225				*NEURL1B*	Body
cg02609692	9	129,389,125	1.28E-06	0.249	0.215				*LMX1B*	Body
cg21046148	5	72,731,905	0.00532	0.249	0.227					
cg24851651	11	66,362,959	9.67E-09	0.249	0.221				*CCS*	Body
cg01174140	5	180,018,735	4.98E-51	0.249	0.251				*SCGB3A1*	TSS1500
cg07384708	19	372,718	8.13E-12	0.249	0.226				*THEG*	Body
cg12505170	5	72,595,685	0.006927	0.249	0.239					
cg17669581	4	54,968,251	0.000349	0.249	0.259					
cg07120346	6	58,141,890	0.007514	0.249	0.187					
cg16420756	6	5,026,219	0.02045	0.249	0.293					
cg23136799	6	167,763,819	0.023411	0.249	0.156					
cg12544951	20	21,695,343	0.04136	0.249	0.339				*PAX1*	Body
cg06581718	19	7,541,760	6.78E-20	0.248	0.241				*PEX11G*	3'UTR
cg10000952	11	116,451,628	0.005669	0.248	0.190					
cg14508572	11	119,778,410	0.000148	0.248	0.202					
cg05481257	2	20,870,211	1.08E-06	0.248	0.218				*GDF7*	Body
cg02154252	1	79,472,532	9.95E-11	0.248	0.226				*ELTD1*	TSS200
cg11044575	6	105,401,636	0.040822	0.248	0.266					
cg21593409	16	88,706,389	0.002156	0.248	0.191				*IL17C*	Body
cg20548888	14	36,974,929	0.003544	0.248	0.303				*SFTA3*	Body

cg00830419	2	20,371,971	2.41E-16	0.248	0.227					
cg06609094	4	175,132,842	0.00846	0.248	0.252					
cg27635267	11	35,547,519	2.64E-05	0.248	0.201				*PAMR1*	TSS1500
cg05513806	3	181,438,216	4.91E-06	0.248	0.248				*SOX2OT*	Body
cg12687617	15	89,948,930	1.04E-07	0.248	0.257					
cg16107628	1	159,894,061	3.53E-05	0.248	0.231				*TAGLN2*	5'UTR
cg26296337	17	72,931,792	2.83E-07	0.248	0.198				*OTOP3*	TSS200
cg08053103	19	5,799,467	1.03E-05	0.248	0.289					
cg14793406	12	115,135,906	1.58E-06	0.248	0.225					
cg01148127	17	1,478,604	5.28E-11	0.248	0.236				*SLC43A2*	3'UTR
cg13762244	11	64,951,960	2.07E-08	0.248	0.288				*CAPN1*	Body
cg22784386	6	100,903,817	0.000455	0.248	0.257				*SIM1*	Body
cg26365668	4	84,375,973	7.78E-07	0.248	0.194	0.00058912	0.065		*MRPS18C*	TSS1500
cg14504269	3	138,658,941	0.002359	0.248	0.287					
cg00494665	12	65,218,728	0.000216	0.248	0.128					
cg02048377	17	37,219,764	0.000462	0.248	0.191				*PLXDC1*	3'UTR
cg07147475	4	141,419,555	3.99E-07	0.248	0.200					
cg10778736	15	45,406,362	2.89E-05	0.248	0.247				*DUOX2*	TSS200
cg12251804	10	135,171,083	2.75E-05	0.248	0.251	1.88E-23	0.247		*C10orf125*	Body
cg16068263	13	36,729,231	2.78E-05	0.248	0.232					
cg02925229	3	139,258,948	1.32E-08	0.248	0.254				*RBP1*	TSS1500
cg12213062	2	220,299,653	8.48E-07	0.248	0.246				*SPEG*	TSS200
cg15583014	1	65,468,659	0.004262	0.248	0.150					
cg02828785	12	12,846,546	9.13E-16	0.248	0.255				*GPR19*	5'UTR
cg02853553	7	44,258,054	2.89E-20	0.248	0.245				*CAMK2B*	3'UTR
cg13897833	13	74,562,744	5E-18	0.248	0.210				*KLF12*	Body
cg21949305	22	24,828,655	1.53E-12	0.248	0.240	2.56E-27	0.252		*C22orf45*	Body
cg23267558	13	70,682,604	0.000635	0.248	0.259				*KLHL1*	1stExon
cg17760391	5	66,124,501	0.001006	0.248	0.111				*MAST4*	TSS200
cg10785622	4	85,403,409	0.006396	0.248	0.255					
cg14664759	1	196,577,723	0.006141	0.248	0.334				*KCNT2*	TSS1500
cg24940370	5	1,835,328	1.78E-06	0.248	0.240					
cg02775927	10	79,470,820	2.04E-06	0.248	0.170					
cg02926266	2	205,847,300	6.03E-11	0.248	0.233				*PARD3B*	Body

cg12796306	8	67,406,954	0.001507	0.248	0.253				*C8orf46*	Body
cg05815196	22	31,536,540	3.43E-05	0.248	0.250				*PLA2G3*	TSS200
cg02167021	3	121,379,791	8.48E-07	0.248	0.220				*HCLS1*	TSS200
cg08690412	3	9,240,454	0.000551	0.248	0.187				*SRGAP3*	Body
cg12646804	5	2,008,188	0.020395	0.248	0.125					
cg21154793	19	54,495,222	0.030195	0.248	0.239				*CACNG6*	TSS1500
cg24641302	1	165,087,025	0.00068	0.248	0.233					
cg11885098	19	1,285,397	1.86E-09	0.248	0.235	2.13E-19	0.088		*EFNA2*	TSS1500
cg15382538	5	72,678,193	2.58E-05	0.248	0.306					
cg23317501	5	35,991,215	7.11E-05	0.248	0.233	6.37E-12	0.155		*UGT3A1*	Body
cg23414595	22	30,129,607	7.64E-12	0.248	0.219				*ZMAT5*	Body
cg24689895	17	27,346,926	0.044769	0.248	0.045					
cg25712005	7	73,443,113	0.002156	0.248	0.359				*ELN*	Body
cg02705958	6	6,901,065	1.47E-10	0.248	0.215					
cg08548089	1	156,408,887	2.82E-08	0.248	0.248					
cg17869514	9	1,042,770	0.001052	0.248	0.291					
cg20454002	1	221,054,365	8.91E-10	0.248	0.250				*HLX*	Body
cg08015382	6	12,365,899	1.39E-06	0.248	0.236					
cg10237765	17	9,863,081	3.4E-05	0.248	0.269				*GAS7*	Body
cg26953453	12	54,346,784	0.001895	0.248	0.279					
cg01351300	4	114,873,079	1.69E-18	0.248	0.216				*ARSJ*	Body
cg10799055	6	27,725,188	0.03395	0.248	0.311					
cg09714458	13	113,807,560	0.028235	0.248	0.312					
cg09918657	16	14,397,207	0.004162	0.248	0.295				*MIR193B*	TSS1500
cg18181496	7	107,642,539	8.33E-06	0.248	0.236				*LAMB1*	Body
cg08957564	16	12,071,814	3.13E-19	0.248	0.251				*RUNDC2A*	Body
cg16786102	4	41,867,462	1.83E-08	0.248	0.233					
cg09317884	1	165,204,512	4.81E-05	0.248	0.246				*LMX1A*	Body
cg14382719	9	124,291,856	1.5E-10	0.248	0.227					
cg19631648	19	33,764,284	2.72E-14	0.248	0.241					
cg27559870	1	31,380,976	0.012173	0.248	0.193				*SDC3*	Body
cg19597382	4	147,559,423	0.023863	0.248	0.181				*POU4F2*	TSS1500
cg04555966	7	44,185,986	0.0002	0.248	0.275				*GCK*	Body
cg22621272	12	54,409,336	0.039855	0.248	0.235				*HOXC4*	TSS1500

cg24140801	8	30,588,816	1.21E-10	0.248	0.207					
cg08843283	1	32,826,969	3.88E-22	0.248	0.233				*LOC100128071*	3'UTR
cg04418091	12	54,071,168	0.011378	0.248	0.213				*ATP5G2*	TSS1500
cg16905100	3	72,149,974	0.032565	0.248	0.147					
cg04932340	14	95,239,381	0.009591	0.248	0.315					
cg03667086	2	130,763,427	1.6E-09	0.248	0.243					
cg06743610	19	35,630,305	5.32E-08	0.248	0.248				*FXYD1*	5'UTR
cg18284089	4	153,601,384	0.032034	0.248	0.236				*TMEM154*	TSS200
cg12479878	1	221,054,825	4.16E-10	0.248	0.232				*HLX*	Body
cg14252661	1	162,792,613	0.002409	0.248	0.292			Yes		
cg05222982	13	28,545,214	1.56E-07	0.248	0.233					
cg22512438	3	129,693,489	0.016312	0.248	0.320				*TRH*	5'UTR
cg08567279	13	53,313,529	0.030945	0.248	0.227				*LECT1*	Body
cg05314679	8	101,821,998	4.69E-05	0.248	0.219			Yes		
cg08994384	3	62,363,642	0.008403	0.248	0.267					
cg19860353	3	194,756,349	8.18E-15	0.248	0.208					
cg26472636	5	1,594,282	0.042894	0.248	0.024				*SDHAP3*	Body
cg01886663	17	78,846,179	8.45E-26	0.248	0.245				*RPTOR*	Body
cg12890346	3	14,567,529	2.82E-09	0.248	0.229				*GRIP2*	Body
cg24775496	14	85,998,576	0.000209	0.248	0.193				*FLRT2*	5'UTR
cg07539798	5	140,811,312	1.55E-07	0.248	0.234				*PCDHGA4*	Body
cg22531904	7	99,595,276	0.003343	0.248	0.230					
cg21684021	1	109,800,400	1.71E-07	0.248	0.264				*CELSR2*	Body
cg27016307	19	49,658,913	3.03E-09	0.248	0.232	3.67E-15	0.096		*HRC*	TSS1500
cg00581422	3	127,794,831	9.26E-08	0.248	0.249					
cg17883914	8	144,344,745	1.12E-14	0.248	0.239				*ZFP41*	3'UTR
cg23270523	18	4,450,489	0.025594	0.248	0.223					
cg00377320	17	47,988,100	2.08E-05	0.248	0.283					
cg04606020	12	56,139,467	0.002079	0.248	0.212				*GDF11*	Body
cg19462635	11	121,237,843	1.45E-09	0.248	0.244					
cg27093944	2	242,908,218	3.12E-06	0.248	0.232					
cg01455568	7	155,261,034	9.33E-06	0.248	0.190					
cg07235805	18	78,004,237	9.96E-07	0.248	0.179				*PARD6G*	Body
cg05151709	1	846,155	1.36E-10	0.248	0.262					

cg02002258	2	26,700,976	2.67E-08	0.248	0.246				*OTOF*	TSS200
cg13455434	8	30,243,930	1.39E-09	0.248	0.221				*RBPMS*	Body
cg13460168	1	16,501,540	1.25E-27	0.248	0.249					
cg16336556	2	33,295,138	6.25E-05	0.248	0.285				*LTBP1*	Body
cg17383842	3	50,553,718	4.56E-08	0.248	0.235					
cg11684647	12	40,618,356	0.004291	0.248	0.300				*LRRK2*	TSS1500
cg07455789	2	232,419,317	9.69E-10	0.248	0.237					
cg24376793	1	202,545,589	3.91E-07	0.248	0.257				*PPP1R12B*	3'UTR
cg02925601	6	27,648,605	0.001972	0.248	0.321					
cg14497054	12	34,261,116	8.47E-24	0.248	0.235					
cg03975690	7	87,848,292	0.049235	0.248	0.198				*SRI*	Body
cg07951347	6	27,463,250	0.010676	0.248	0.309					
cg03703839	16	87,990,084	0.000133	0.248	0.253				*BANP*	5'UTR
cg08291024	1	165,324,411	0.007466	0.248	0.332				*LMX1A*	Body
cg16046444	1	200,004,265	0.001108	0.248	0.295				*NR5A2*	Body
cg15572907	19	40,995,921	0.001869	0.248	0.245				*SPTBN4*	Body
cg17003260	17	8,907,561	3.44E-05	0.248	0.228					
cg20489847	8	144,095,483	1.58E-08	0.248	0.249				*LOC100133669*	Body
cg01451162	1	58,714,452	0.007854	0.248	0.214				*DAB1*	5'UTR
cg20743744	4	1,243,849	3.97E-06	0.248	0.217	3.27E-18	0.221		*C4orf42*	TSS1500
cg03053826	15	96,868,935	0.001341	0.248	0.217				*NR2F2*	TSS1500
cg07042914	11	27,015,656	0.017714	0.248	0.295				*FIBIN*	5'UTR
cg09368936	2	43,019,819	0.008166	0.248	0.157				*HAAO*	TSS200
cg23737737	12	6,665,370	5.99E-05	0.248	0.251				*IFFO1*	TSS200
cg03519157	11	46,367,033	1.48E-10	0.248	0.249				*DGKZ*	5'UTR
cg02371631	2	202,748,718	8.15E-11	0.248	0.234				*CDK15*	Body
cg04460847	7	127,841,335	1.11E-06	0.248	0.195					
cg08951638	5	141,706,213	8.42E-11	0.248	0.234					
cg27516568	4	122,686,667	0.001099	0.248	0.189				*TMEM155*	TSS1500
cg01305981	5	135,266,673	8.96E-08	0.248	0.235				*FBXL21*	5'UTR
cg09605636	2	200,334,584	4.81E-07	0.248	0.228				*FLJ32063*	Body
cg12967327	17	1,906,190	7.18E-06	0.248	0.210				*RTN4RL1*	Body
cg08475953	5	178,004,091	0.002708	0.248	0.255				*COL23A1*	Body
cg21115608	13	20,735,860	9.35E-08	0.248	0.244				*GJA3*	TSS1500

cg02671880	13	95,355,117	0.001385	0.248	0.262					
cg04288241	2	236,444,202	8.49E-05	0.248	0.226				*AGAP1*	Body
cg11328436	12	54,360,511	3.54E-11	0.248	0.224				*HOTAIR*	Body
cg16987754	7	155,242,911	0.003536	0.248	0.200					
cg01213447	15	96,871,576	3.91E-06	0.247	0.253				*NR2F2*	Body
cg10900455	20	42,545,099	4.6E-06	0.247	0.213				*TOX2*	5'UTR
cg10928294	17	41,739,326	0.000559	0.247	0.222				*MEOX1*	TSS1500
cg01146232	16	51,185,147	0.006234	0.247	0.211				*SALL1*	TSS1500
cg02269083	18	56,296,607	1.42E-07	0.247	0.264				*ALPK2*	TSS1500
cg19149463	3	11,651,759	1.06E-13	0.247	0.203				*VGLL4*	Body
cg21545390	7	96,627,190	0.035208	0.247	0.283				*DLX6AS*	Body
cg05255351	5	1,503,979	5.12E-14	0.247	0.232				*LPCAT1*	Body
cg18274480	5	145,713,863	1.67E-05	0.247	0.242					
cg12492345	16	54,971,007	0.013333	0.247	0.297					
cg04846243	17	47,074,883	0.005583	0.247	0.274				*IGF2BP1*	1stExon
cg07303450	16	30,023,400	8.33E-07	0.247	0.252				*DOC2A*	TSS1500
cg08559364	3	11,634,536	2.27E-16	0.247	0.245				*VGLL4*	Body
cg21815667	2	176,993,841	0.000408	0.247	0.247	3.48E-11	0.152		*HOXD8*	TSS1500
cg23085662	18	59,000,860	4.92E-08	0.247	0.208					
cg01933836	11	122,854,796	4.65E-05	0.247	0.238					
cg15128801	1	68,202,053	0.0002	0.247	0.246				*GNG12*	5'UTR
cg16022049	12	13,359,372	2.12E-06	0.247	0.248				*EMP1*	5'UTR
cg20965469	2	100,266,111	6.31E-08	0.247	0.243				*AFF3*	Body
cg08185345	1	155,055,102	0.000626	0.247	0.210				*EFNA3*	Body
cg21165812	20	54,931,343	9.41E-08	0.247	0.245					
cg14263858	19	4,328,745	7.72E-05	0.247	0.260				*STAP2*	Body
cg27290624	3	194,208,441	0.000277	0.247	0.240					
cg14398661	9	129,433,779	9.44E-07	0.247	0.247				*LMX1B*	Body
cg14569644	12	54,408,091	3E-05	0.247	0.223					
cg23346408	10	81,001,957	0.001909	0.247	0.179				*ZMIZ1*	Body
cg27069921	5	54,523,727	1.32E-06	0.247	0.238					
cg09234616	11	32,452,592	0.007517	0.247	0.257				*WT1*	Body
cg25219188	4	165,898,926	0.000149	0.247	0.238				*TRIM61*	TSS200
cg26922678	6	30,420,981	0.004587	0.247	0.239					

cg00298065	19	58,715,577	1.82E-11	0.247	0.240				*ZNF274*	Body
cg11226913	14	29,254,961	7.18E-07	0.247	0.240				*C14orf23*	Body
cg19851909	11	94,134,827	0.000201	0.247	0.153				*GPR83*	TSS1500
cg13896328	1	29,652,476	9.66E-18	0.247	0.241				*PTPRU*	3'UTR
cg01069311	X	47,373,245	2.29E-16	0.247	0.218					
cg01113811	11	95,927,950	1.81E-06	0.247	0.273				*MAML2*	Body
cg04037970	7	54,955,965	1.31E-29	0.247	0.220					
cg20030711	17	75,471,308	4.48E-16	0.247	0.234				*SEPT9*	Body
cg19216792	7	26,897,202	1.22E-10	0.247	0.219				*SKAP2*	Body
cg12423482	X	21,676,871	0.004071	0.247	0.225				*KLHL34*	TSS1500
cg12776287	18	24,125,939	0.000644	0.247	0.201				*KCTD1*	5'UTR
cg15460348	4	88,896,909	1.4E-05	0.247	0.229				*SPP1*	1stExon
cg15281774	15	73,661,908	0.022766	0.247	0.289				*HCN4*	TSS1500
cg18184411	1	53,309,261	0.000507	0.247	0.204				*ZYG11A*	Body
cg05959459	1	147,752,860	0.00281	0.247	0.268					
cg05081751	20	62,108,923	4.86E-05	0.247	0.278					
cg07452306	2	73,299,297	1.27E-11	0.247	0.175				*SFXN5*	TSS1500
cg14959729	6	166,580,693	1.81E-06	0.247	0.271				*T*	Body
cg11996428	9	135,466,211	0.00067	0.247	0.220					
cg12074182	2	176,948,596	3.96E-06	0.247	0.207				*EVX2*	5'UTR
cg13998850	14	24,540,773	7.07E-10	0.247	0.292				*CPNE6*	1stExon
cg05921207	X	110,038,886	7.61E-05	0.247	0.167	4.56E-32	0.166		*CHRDL1*	5'UTR
cg26279070	2	130,971,343	0.000896	0.247	0.203					
cg05068452	4	115,521,241	7.75E-08	0.247	0.256				*UGT8*	5'UTR
cg18448746	17	27,039,132	5.28E-07	0.247	0.246				*PROCA1*	TSS1500
cg22740603	6	33,397,959	3E-18	0.247	0.239				*SYNGAP1*	Body
cg12743638	22	20,192,485	1.2E-14	0.247	0.246				*LOC150197*	TSS1500
cg24000859	7	71,023,890	0.002431	0.247	0.168				*WBSCR17*	Body
cg00828762	8	42,234,732	2.16E-06	0.247	0.256				*DKK4*	TSS200
cg00914222	1	29,585,898	0.000143	0.247	0.193				*PTPRU*	Body
cg16473141	14	24,641,501	5.3E-12	0.247	0.237				*REC8*	1stExon
cg19286687	2	220,282,243	2.97E-10	0.247	0.224				*DES*	TSS1500
cg09822192	14	24,801,191	8.01E-21	0.247	0.223				*ADCY4*	Body
cg14360917	17	45,992,122	7.32E-12	0.247	0.259	7.38E-37	0.193		*SP2*	Body

cg07737560	12	101,470,827	0.003645	0.247	0.236				*ANO4*	Body
cg01620701	15	75,953,954	5.56E-11	0.247	0.220					
cg07242540	1	12,600,348	2.57E-10	0.247	0.264					
cg26987660	11	71,952,431	5.87E-09	0.247	0.217				*PHOX2A*	Body
cg16861031	12	105,477,526	0.000278	0.247	0.212				*ALDH1L2*	Body
cg19184885	17	45,699,322	2.28E-16	0.247	0.263				*NPEPPS*	3'UTR
cg16941302	18	55,108,947	0.008382	0.247	0.268				*ONECUT2*	Body
cg11250081	21	46,126,104	4.02E-05	0.247	0.249				*C21orf29*	Body
cg14383658	10	102,899,262	5.25E-06	0.247	0.225					
cg25389328	17	38,222,062	1.82E-18	0.247	0.236				*THRA*	5'UTR
cg26568372	1	91,190,173	0.026194	0.247	0.338					
cg06321596	16	17,562,960	2E-08	0.247	0.269				*XYLT1*	Body
cg20010506	12	62,585,467	0.000141	0.247	0.207				*FAM19A2*	5'UTR
cg13403586	1	22,141,170	0.031314	0.247	0.330				*LDLRAD2*	Body
cg01291336	7	150,949,495	0.004631	0.247	0.152				*SMARCD3*	Body
cg12684999	2	161,099,777	5.27E-08	0.247	0.242					
cg19589939	12	103,355,326	0.000358	0.247	0.250					
cg24411961	1	34,642,396	0.007798	0.247	0.207				*C1orf94*	5'UTR
cg20803796	8	145,103,842	0.003091	0.247	0.225					
cg23837265	10	88,730,407	3.13E-05	0.247	0.192				*C10orf116*	3'UTR
cg24249778	4	55,095,226	0.014003	0.247	0.342				*PDGFRA*	TSS200
cg14183329	7	131,242,962	0.012307	0.247	0.175					
cg12093241	6	157,465,138	8.39E-17	0.247	0.245				*ARID1B*	Body
cg12434944	7	48,031,559	0.003907	0.247	0.216				*SUNC1*	Body
cg19540425	12	32,655,836	3.65E-18	0.247	0.246				*FGD4*	5'UTR
cg08194989	8	41,655,154	8.57E-15	0.247	0.229				*ANK1*	TSS200
cg11131384	7	155,325,597	0.000786	0.247	0.215				*CNPY1*	5'UTR
cg23695133	20	21,087,075	0.017762	0.247	0.273					
cg02249713	6	6,547,074	0.045279	0.247	0.096			Yes	*LOC285780*	Body
cg18378811	4	114,900,050	0.01508	0.247	0.232				*ARSJ*	1stExon
cg09407650	16	85,320,156	4.43E-10	0.247	0.235					
cg12237784	7	44,187,017	9.81E-27	0.247	0.226				*GCK*	Body
cg16727774	16	87,958,281	4.79E-05	0.247	0.197				*CA5A*	Body
cg25774643	11	627,175	7.77E-05	0.247	0.238				*SCT*	TSS200

cg14031054	1	164,681,652	1.46E-08	0.247	0.236				*PBX1*	Body
cg16310491	5	87,973,597	0.000277	0.247	0.197				*LOC645323*	Body
cg03070194	1	110,210,684	6.73E-07	0.247	0.247	1.76E-19	0.235		*GSTM2*	5'UTR
cg00722631	5	9,548,098	0.000952	0.247	0.273			Yes	*SNORD123*	TSS1500
cg02996801	4	113,441,944	0.017295	0.247	0.323					
cg05226932	22	37,720,696	8.21E-07	0.247	0.196					
cg06836849	12	100,751,051	3.67E-05	0.247	0.214	2.28E-24	0.188		*SLC17A8*	1stExon
cg14758204	20	9,487,871	1.29E-07	0.247	0.216					
cg16297271	14	94,984,308	2.86E-06	0.247	0.243				*SERPINA12*	TSS200
cg22627981	1	165,204,410	1.37E-05	0.247	0.262				*LMX1A*	Body
cg00683332	16	51,188,697	0.003745	0.247	0.198					
cg00868875	18	24,127,237	2.22E-06	0.247	0.199				*KCTD1*	5'UTR
cg06830319	16	57,406,074	6.43E-11	0.247	0.249				*CX3CL1*	TSS1500
cg17884327	3	197,840,760	2.07E-05	0.247	0.247					
cg16829453	17	48,636,646	8.23E-05	0.247	0.208					
cg19601328	17	43,368,729	0.013505	0.247	0.325	2.69E-17	0.266		*MAP3K14*	5'UTR
cg04819096	12	54,339,167	0.000313	0.247	0.239				*HOXC13*	3'UTR
cg17455088	2	206,551,466	2.75E-06	0.247	0.228				*NRP2*	Body
cg16105461	11	61,485,853	7.67E-12	0.247	0.250				*DAGLA*	5'UTR
cg19003304	8	60,029,914	1.47E-05	0.247	0.223				*TOX*	Body
cg27033805	8	145,499,436	7.29E-05	0.247	0.258				*BOP1*	Body
cg08584947	8	93,468,317	5.26E-05	0.247	0.259					
cg11528328	21	36,041,683	0.000533	0.247	0.248				*CLIC6*	TSS200
cg18987335	2	177,039,862	0.000378	0.247	0.209					
cg23712458	17	189,002	1.84E-12	0.247	0.217				*RPH3AL*	5'UTR
cg25101657	17	32,751,628	0.003187	0.247	0.339					
cg19668476	4	153,506,267	7.9E-06	0.247	0.257					
cg23681339	12	133,065,808	8.59E-05	0.247	0.182				*FBRSL1*	TSS1500
cg03578926	11	70,508,032	6.48E-06	0.247	0.231				*SHANK2*	Body
cg18024113	1	16,862,202	8.07E-05	0.247	0.299					
cg06299307	16	10,133,466	0.035513	0.247	0.232				*GRIN2A*	Body
cg05955675	16	85,206,653	1.28E-09	0.247	0.236					
cg19664945	2	54,087,343	0.007808	0.247	0.129	1.39E-11	0.191		*LOC100302652*	TSS200
cg24603113	7	1,105,321	1.63E-13	0.247	0.237				*C7orf50*	Body

cg05323725	17	35,294,713	0.000949	0.247	0.182				*LHX1*	1stExon
cg24883265	1	5,917,845	7.1E-12	0.247	0.226					
cg03942051	1	119,532,655	5.86E-09	0.247	0.242				*TBX15*	TSS1500
cg15734553	19	11,960,208	0.049738	0.247	0.110					
cg16554148	1	3,630,882	3.11E-12	0.247	0.231			Yes	*TP73*	Body
cg16656979	15	37,403,242	0.036153	0.247	0.169					
cg00930833	8	41,168,264	2.11E-08	0.247	0.218				*SFRP1*	TSS1500
cg19194233	22	50,523,801	0.000254	0.247	0.258				*MLC1*	1stExon
cg24232444	13	99,545,448	2.35E-08	0.247	0.231				*DOCK9*	Body
cg09346546	2	176,943,669	1.06E-06	0.247	0.222					
cg18294691	14	38,080,669	1.06E-06	0.247	0.263					
cg19051416	7	74,064,585	0.001843	0.247	0.290					
cg18523477	20	55,967,503	2.35E-16	0.247	0.231				*RBM38*	Body
cg13679691	22	50,608,033	0.002051	0.247	0.161				*PANX2*	TSS1500
cg15671450	6	29,895,116	5.5E-07	0.247	0.210					
cg21118367	1	184,460,875	2.9E-05	0.247	0.224				*C1orf21*	Body
cg10820551	22	45,608,345	5.19E-08	0.247	0.213				*C22orf9*	Body
cg21651000	8	71,129,472	8.37E-10	0.246	0.265				*NCOA2*	5'UTR
cg08927006	10	100,993,556	1.15E-06	0.246	0.241				*HPSE2*	Body
cg09712464	17	2,627,328	2.03E-05	0.246	0.240					
cg25605307	1	183,965,312	1.96E-10	0.246	0.232				*GLT25D2*	Body
cg23248887	14	38,679,643	3.05E-08	0.246	0.218				*SSTR1*	Body
cg16658255	5	598,906	0.000383	0.246	0.247					
cg24736989	14	60,973,904	0.017695	0.246	0.231					
cg06864853	7	134,143,971	0.006876	0.246	0.202				*AKR1B1*	TSS200
cg24378559	7	156,889,254	7.08E-06	0.246	0.212					
cg01112452	X	117,958,532	7.05E-05	0.246	0.248	3.89E-19	0.155		*ZCCHC12*	5'UTR
cg19770292	5	1,868,693	8.19E-06	0.246	0.278			Yes		
cg07489869	13	20,806,730	4.37E-05	0.246	0.179				*GJB6*	TSS200
cg15323253	6	3,766,662	9.94E-06	0.246	0.195					
cg18063312	5	72,740,737	0.012711	0.246	0.186					
cg21268984	1	8,720,599	1.28E-07	0.246	0.263				*RERE*	5'UTR
cg08354406	10	115,370,978	0.012566	0.246	0.278				*NRAP*	Body
cg08743751	5	66,462,471	0.002659	0.246	0.244				*MAST4*	Body

cg19653417	12	132,654,924	0.002614	0.246	0.221					
cg01273232	15	93,652,756	2.03E-05	0.246	0.239					
cg05869699	10	82,258,734	0.032867	0.246	0.183				*TSPAN14*	Body
cg13721989	9	130,689,253	6.91E-20	0.246	0.217				*PIP5KL1*	Body
cg18669209	18	44,777,736	0.0484	0.246	0.224					
cg08404028	16	23,766,112	0.008645	0.246	0.306				*CHP2*	1stExon
cg02299328	17	38,346,960	2.53E-16	0.246	0.228				*RAPGEFL1*	Body
cg17631390	15	42,174,823	3.94E-09	0.246	0.221				*SPTBN5*	Body
cg17854454	5	77,140,243	0.001255	0.246	0.185					
cg24159636	6	18,043,467	1.53E-14	0.246	0.254					
cg24630419	1	217,311,608	1.15E-08	0.246	0.237				*ESRRG*	TSS1500
cg16197636	8	42,234,724	9.8E-08	0.246	0.250				*DKK4*	TSS200
cg18991601	2	208,635,549	5.97E-08	0.246	0.244				*FZD5*	TSS1500
cg06636289	1	40,781,617	0.005442	0.246	0.194				*COL9A2*	Body
cg01825213	9	98,979,965	1.92E-06	0.246	0.247					
cg14612428	18	11,751,566	7.74E-07	0.246	0.230				*GNAL*	5'UTR
cg00090261	8	101,118,548	0.000263	0.246	0.246				*RGS22*	TSS1500
cg06100570	7	2,078,988	1.49E-11	0.246	0.237				*MAD1L1*	Body
cg12393318	5	87,981,068	3.55E-07	0.246	0.288				*LOC645323*	TSS1500
cg15972560	4	82,136,940	0.001877	0.246	0.212					
cg17890928	17	48,546,193	0.008838	0.246	0.233				*ACSF2*	Body
cg11218954	2	45,164,864	1.71E-07	0.246	0.241					
cg06391412	3	71,295,684	8.34E-13	0.246	0.203				*FOXP1*	5'UTR
cg02589685	11	2,889,629	5.4E-14	0.246	0.245					
cg03283990	15	35,129,641	2.15E-05	0.246	0.175					
cg15517113	2	620,265	1.69E-05	0.246	0.245					
cg22775000	9	103,235,181	0.000897	0.246	0.157	2.54E-15	0.116		*TMEFF1*	TSS1500
cg12081870	1	205,475,130	5.87E-14	0.246	0.226				*CDK18*	5'UTR
cg07990546	2	74,742,309	0.00413	0.246	0.297				*TLX2*	1stExon
cg08129092	1	153,746,211	4.37E-10	0.246	0.254				*INTS3*	3'UTR
cg08828733	12	45,268,613	6.52E-08	0.246	0.218				*NELL2*	Body
cg27223827	2	106,054,920	5.4E-18	0.246	0.236	1.24E-22	0.169	Yes	*FHL2*	1stExon
cg14968300	8	23,585,186	1.79E-05	0.246	0.229					
cg15986251	2	65,519,810	0.001442	0.246	0.259					

cg24541426	2	177,029,459	3.73E-06	0.246	0.199				*HOXD3*	5'UTR
cg27117828	2	175,499,377	0.01207	0.246	0.211				*WIPF1*	5'UTR
cg26375010	X	118,407,852	0.021736	0.246	0.229					
cg26705724	15	41,061,984	4.63E-11	0.246	0.242				*DNAJC17*	Body
cg25513659	17	183,931	0.041304	0.246	0.137				*RPH3AL*	5'UTR
cg09827761	15	66,947,392	0.014922	0.246	0.148					
cg14023881	1	77,230,704	2.38E-09	0.246	0.186					
cg15481172	1	2,059,086	2.21E-10	0.246	0.240				*PRKCZ*	5'UTR
cg12817924	X	13,588,162	0.000921	0.246	0.231				*EGFL6*	Body
cg13423383	9	139,889,616	1.05E-15	0.246	0.204				*CLIC3*	Body
cg11667020	20	21,378,045	0.018549	0.246	0.231				*NKX2-4*	5'UTR
cg23054181	15	99,048,945	0.00499	0.246	0.200				*FAM169B*	5'UTR
cg25485913	19	49,568,962	2.12E-10	0.246	0.249	7.51E-27	0.132			
cg09095777	12	53,465,912	1.81E-14	0.246	0.252				*SPRYD3*	Body
cg10906415	17	36,719,937	4.56E-05	0.246	0.233				*SRCIN1*	Body
cg13977835	1	23,883,338	2.43E-07	0.246	0.275					
cg17588800	7	42,278,067	2.24E-11	0.246	0.229				*GLI3*	TSS1500
cg19988426	10	130,064,021	0.040077	0.246	0.234					
cg26125625	3	124,860,871	2.8E-05	0.246	0.249				*SLC12A8*	Body
cg19929189	6	41,340,058	0.013335	0.246	0.266					
cg11529346	6	168,665,455	2.64E-07	0.246	0.262					
cg12573849	2	105,470,711	1.55E-08	0.246	0.230				*POU3F3*	TSS1500
cg14453145	2	242,801,896	7.16E-07	0.246	0.225				*PDCD1*	TSS1500
cg03782157	16	54,970,302	0.011849	0.246	0.302					
cg06495501	22	50,523,761	0.000189	0.246	0.257				*MLC1*	5'UTR
cg14311670	3	181,444,999	0.004788	0.246	0.269				*SOX2OT*	Body
cg22635541	17	1,808,573	1.39E-20	0.246	0.195					
cg26345888	1	58,716,257	9.1E-07	0.246	0.193				*DAB1*	TSS200
cg27282564	19	55,577,746	9.7E-09	0.246	0.269				*RDH13*	5'UTR
cg25457886	3	14,339,565	8.98E-06	0.246	0.249					
cg22996489	13	97,646,736	0.045122	0.246	0.334				*OXGR1*	TSS200
cg09682128	13	100,613,240	6E-05	0.246	0.221					
cg11660018	11	86,510,915	9.72E-23	0.246	0.214				*PRSS23*	TSS1500
cg21287054	19	54,928,012	0.012315	0.246	0.202				*TTYH1*	Body

cg05460226	17	8,804,279	0.000249	0.246	0.320				*PIK3R5*	Body
cg02388882	10	88,731,631	1.49E-05	0.246	0.236				*AGAP11*	5'UTR
cg07057744	17	4,545,074	0.003704	0.246	0.343				*ALOX15*	TSS200
cg26415789	17	75,475,973	1.89E-20	0.246	0.232				*SEPT9*	Body
cg11931762	20	9,488,920	2.49E-06	0.246	0.223					
cg13157980	8	141,599,141	0.000142	0.246	0.196				*EIF2C2*	Body
cg17105834	1	177,133,566	0.045774	0.246	0.293				*ASTN1*	1stExon
cg10068989	11	129,244,122	0.000302	0.246	0.268					
cg11485154	11	46,265,393	0.000821	0.246	0.152					
cg26644885	16	72,981,599	1.14E-10	0.246	0.232				*ZFHX3*	Body
cg03301582	7	95,026,095	0.000153	0.246	0.233				*PON3*	TSS1500
cg04565008	11	67,418,140	1.3E-14	0.246	0.236				*ACY3*	TSS200
cg05756029	11	18,230,754	5.35E-12	0.246	0.247				*LOC494141*	Body
cg08131184	19	33,764,378	2.48E-15	0.246	0.239					
cg18446336	7	2,847,575	4.07E-06	0.246	0.176				*GNA12*	Body
cg08767182	5	92,940,163	8.44E-05	0.246	0.195					
cg20255723	22	45,073,031	3.42E-14	0.246	0.225				*PRR5*	5'UTR
cg13647523	9	120,178,198	6.11E-07	0.246	0.285				*ASTN2*	TSS1500
cg22523050	7	137,564,212	9.94E-11	0.246	0.247				*CREB3L2*	3'UTR
cg05766064	18	19,262,576	0.003123	0.246	0.217				*ABHD3*	Body
cg12615535	3	139,257,713	7.5E-09	0.246	0.248				*RBP1*	Body
cg20308679	2	183,731,407	0.049939	0.246	0.142	0.00131609	0.087		*FRZB*	5'UTR
cg05446860	20	25,129,126	0.004196	0.246	0.276				*LOC284798*	Body
cg04090347	21	44,061,597	1.37E-05	0.246	0.253					
cg13969327	7	127,526,253	9.07E-12	0.246	0.244				*SND1*	Body
cg10064871	11	6,341,908	3.74E-05	0.246	0.246				*PRKCDBP*	TSS200
cg11435506	7	95,026,073	0.000976	0.246	0.197				*PON3*	TSS1500
cg02856338	8	101,822,108	1.89E-07	0.246	0.243					
cg06299284	11	636,659	0.003262	0.246	0.240				*DRD4*	TSS1500
cg10635061	2	106,055,006	5.72E-11	0.246	0.264	2.28E-25	0.218		*FHL2*	1stExon
cg11746924	17	62,007,912	2.1E-12	0.246	0.240				*CD79B*	Body
cg27212729	2	171,570,838	0.008967	0.246	0.154				*LOC440925*	Body
cg26634121	16	88,897,219	7.29E-24	0.246	0.225				*GALNS*	Body
cg01598596	3	187,464,648	0.000239	0.246	0.149				*BCL6*	TSS1500

cg03936449	16	56,696,967	1.79E-05	0.246	0.248					
cg18045100	5	3,764,541	0.000409	0.246	0.258					
cg01416712	4	41,750,864	0.000495	0.246	0.272				*PHOX2B*	1stExon
cg11344729	7	151,335,190	1.09E-21	0.246	0.249				*PRKAG2*	Body
cg08812802	12	49,686,619	2.16E-06	0.246	0.270					
cg15626285	12	7,167,781	0.002978	0.246	0.298				*C1S*	TSS200
cg01940964	7	128,471,600	4.04E-05	0.246	0.230				*FLNC*	Body
cg04474277	7	134,376,946	0.003479	0.246	0.285					
cg05392244	6	1,575,286	1.37E-08	0.246	0.228					
cg23217419	1	180,881,088	0.003828	0.246	0.215				*KIAA1614*	TSS1500
cg24624901	7	19,184,273	0.025256	0.246	0.280					
cg26937778	14	96,562,667	1.06E-05	0.246	0.239					
cg13035743	6	32,119,685	0.014579	0.246	0.225	4.35E-16	0.206		*PRRT1*	1stExon
cg18631996	5	139,526,080	1.89E-13	0.246	0.219					
cg05749969	1	38,412,711	2.65E-06	0.246	0.241				*INPP5B*	5'UTR
cg10168494	3	187,635,183	9.02E-08	0.246	0.220					
cg16033633	13	100,624,855	0.000168	0.246	0.239				*ZIC5*	TSS1500
cg16448890	3	8,783,613	0.000229	0.246	0.209				*CAV3*	Body
cg20431250	6	108,492,653	9.39E-05	0.246	0.257				*NR2E1*	Body
cg21779301	7	30,029,757	0.014776	0.246	0.103				*SCRN1*	TSS1500
cg04112211	5	87,436,327	0.009236	0.245	0.189					
cg06601581	16	85,404,654	0.00189	0.245	0.159					
cg00107772	14	57,261,886	0.000627	0.245	0.192					
cg22820364	14	81,901,540	1.57E-05	0.245	0.235					
cg16990168	1	119,526,060	0.000604	0.245	0.238				*TBX15*	5'UTR
cg25429640	5	176,302,913	0.001099	0.245	0.263				*UNC5A*	Body
cg17124224	15	83,953,880	6.92E-06	0.245	0.234				*BNC1*	TSS1500
cg18142262	2	180,293,643	0.000276	0.245	0.239					
cg09870568	3	138,154,278	1.05E-05	0.245	0.246			Yes	*ESYT3*	Body
cg05259836	6	74,290,516	0.004303	0.245	0.247					
cg07839742	6	84,761,960	0.000422	0.245	0.288				*MRAP2*	5'UTR
cg14189571	4	188,916,943	8.55E-06	0.245	0.238	4.75E-10	0.205		*ZFP42*	1stExon
cg15721666	12	120,799,597	2.58E-45	0.245	0.242				*MSI1*	Body
cg26708483	5	131,422,110	2.39E-09	0.245	0.251			Yes		

cg08634318	3	13,323,642	6.18E-06	0.245	0.223					
cg18798099	9	140,189,618	2.69E-17	0.245	0.224					
cg24219033	17	7,485,218	1.11E-07	0.245	0.265				*CD68*	3'UTR
cg09479650	16	85,578,516	1.38E-20	0.245	0.217					
cg10864680	13	108,922,274	0.000141	0.245	0.238				*TNFSF13B*	1stExon
cg27177554	10	16,562,102	2.1E-05	0.245	0.261				*C1QL3*	Body
cg13371788	6	117,586,817	0.030753	0.245	0.254				*VGLL2*	1stExon
cg16431436	5	82,768,907	0.020492	0.245	0.115				*VCAN*	5'UTR
cg16170708	1	6,390,830	3.55E-25	0.245	0.229				*ACOT7*	Body
cg00741609	10	102,893,925	0.000549	0.245	0.258				*TLX1*	Body
cg23861803	6	31,830,030	0.026027	0.245	0.211				*NEU1*	Body
cg26314055	1	1,182,658	0.002891	0.245	0.289				*FAM132A*	TSS1500
cg03144232	16	31,022,351	0.026378	0.245	0.270				*STX1B*	TSS1500
cg07729130	11	45,167,493	0.027019	0.245	0.224				*PRDM11*	Body
cg09145546	16	56,696,748	5.65E-06	0.245	0.236					
cg27036111	11	20,620,680	0.000622	0.245	0.289				*SLC6A5*	TSS1500
cg10540626	2	71,124,470	0.000001	0.245	0.253					
cg14319235	1	79,472,282	0.0004	0.245	0.215				*ELTD1*	Body
cg14334389	4	88,451,346	0.000954	0.245	0.299				*SPARCL1*	TSS1500
cg05022789	8	135,469,681	0.038589	0.245	0.173					
cg14405893	9	127,539,942	0.011794	0.245	0.198				*OLFML2A*	Body
cg05384646	15	30,206,862	1.03E-12	0.245	0.249					
cg09840968	15	37,394,840	0.032367	0.245	0.336				*MEIS2*	TSS1500
cg12727529	3	42,307,761	3.14E-20	0.245	0.248				*CCK*	TSS1500
cg14211310	7	7,557,741	4.46E-08	0.245	0.221				*COL28A1*	Body
cg14396619	7	98,424,185	0.000118	0.245	0.218					
cg26183870	14	100,142,298	1.73E-07	0.245	0.197				*HHIPL1*	3'UTR
cg10796603	X	133,676,860	1.75E-16	0.245	0.222				*MIR542*	TSS1500
cg18888461	11	18,477,379	2.28E-05	0.245	0.186				*LDHAL6A*	1stExon
cg03704031	X	70,712,215	1.55E-05	0.245	0.192	0.0000131	0.144		*INGX*	Body
cg03339247	16	73,059,888	3.16E-05	0.245	0.222				*ZFHX3*	5'UTR
cg06619938	12	54,376,127	1.9E-07	0.245	0.229					
cg07525782	5	33,324,641	3.18E-15	0.245	0.237					
cg18084609	14	31,345,041	7.23E-17	0.245	0.241				*COCH*	Body

cg22522598	19	15,360,953	2.12E-20	0.245	0.241				*BRD4*	Body
cg01916088	6	41,528,531	3.99E-08	0.245	0.236				*FOXP4*	5'UTR
cg04203587	4	75,895,612	8.63E-20	0.245	0.237				*PARM1*	Body
cg12386646	15	41,787,261	0.000228	0.245	0.251				*ITPKA*	Body
cg00164458	1	225,676,425	2.77E-10	0.245	0.236				*ENAH*	3'UTR
cg02697649	2	202,901,045	4.36E-07	0.245	0.253				*FZD7*	1stExon
cg09949775	19	18,902,107	0.000123	0.245	0.195	3.69E-17	0.158		*COMP*	1stExon
cg09224689	11	31,825,166	0.024931	0.245	0.304				*PAX6*	Body
cg25617328	5	76,939,333	1.97E-12	0.245	0.216					
cg19772399	11	133,825,319	0.002556	0.245	0.217				*IGSF9B*	Body
cg10703784	12	28,127,353	0.00132	0.245	0.277					
cg14952949	12	81,109,806	1.01E-13	0.245	0.229				*MYF5*	TSS1500
cg27105390	19	23,253,316	1.1E-05	0.245	0.266					
cg00268086	17	5,487,376	3.28E-06	0.245	0.258				*NLRP1*	5'UTR
cg15608301	13	100,085,187	5.4E-07	0.245	0.191					
cg04452770	12	53,267,981	0.029618	0.245	0.192					
cg24989193	10	105,540,623	1.84E-07	0.245	0.216				*SH3PXD2A*	Body
cg25250968	6	11,318,770	5.1E-06	0.245	0.236				*NEDD9*	5'UTR
cg03858703	3	68,316,096	0.00534	0.245	0.179				*FAM19A1*	Body
cg06945399	7	127,671,195	0.007204	0.245	0.163				*LRRC4*	TSS200
cg20168495	4	148,283,066	1.1E-06	0.245	0.243					
cg12974599	22	46,770,195	3.35E-10	0.245	0.228				*CELSR1*	Body
cg26608174	17	46,711,035	9.4E-06	0.245	0.217				*MIR196A1*	TSS1500
cg06212263	21	34,392,851	0.000808	0.245	0.219					
cg17788832	8	87,081,654	1.01E-11	0.245	0.248	3.55E-31	0.195		*PSKH2*	Body
cg04948892	3	181,428,462	3.87E-06	0.245	0.238				*SOX2OT*	Body
cg13925809	9	84,646,696	9.36E-05	0.245	0.291					
cg21195468	9	126,773,095	1.98E-09	0.245	0.256				*LHX2*	TSS1500
cg10431481	1	45,249,875	0.003817	0.245	0.323				*BEST4*	3'UTR
cg16026813	10	103,245,720	8.79E-06	0.245	0.251				*BTRC*	Body
cg18584387	12	4,384,879	0.002523	0.245	0.264				*CCND2*	Body
cg04076682	11	32,915,823	0.005795	0.245	0.304				*QSER1*	5'UTR
cg17296589	13	113,366,074	0.000133	0.245	0.145				*ATP11A*	Body
cg22076941	1	70,036,106	0.000218	0.245	0.251					

cg08271804	19	22,816,896	0.014036	0.245	0.237				*ZNF492*	TSS1500
cg16642520	2	54,936,739	6.4E-05	0.245	0.196					
cg07885629	7	129,422,915	3.23E-05	0.245	0.247					
cg11348293	2	219,926,334	0.004562	0.245	0.156				*IHH*	TSS1500
cg07082484	4	170,075,731	3E-05	0.245	0.254				*SH3RF1*	Body
cg15662251	1	26,197,855	2.43E-14	0.245	0.229	4.39E-24	0.224		*PAQR7*	TSS200
cg09555736	16	474,271	0.002875	0.245	0.300				*RAB11FIP3*	TSS1500
cg11878016	7	84,816,843	0.001621	0.245	0.253					
cg26221105	1	223,914,343	2.77E-06	0.245	0.240				*CAPN2*	Body
cg04666975	14	60,977,353	0.002499	0.245	0.198				*SIX6*	Body
cg08190450	17	800,717	3.67E-07	0.245	0.234				*NXN*	Body
cg23475955	11	1,985,762	1.47E-10	0.245	0.246					
cg04737114	7	96,654,931	0.00024	0.245	0.217				*DLX5*	TSS1500
cg06352807	7	157,648,699	1.45E-11	0.245	0.228				*PTPRN2*	Body
cg12621514	10	54,073,047	0.002172	0.245	0.272	5.02E-13	0.187		*DKK1*	TSS1500
cg01337813	7	127,808,512	0.001628	0.245	0.216					
cg16115418	X	3,630,582	1.83E-05	0.245	0.162				*PRKX*	Body
cg10109500	3	172,165,884	0.011054	0.245	0.275				*GHSR*	1stExon
cg15773794	2	172,957,583	0.006148	0.245	0.270					
cg00192882	17	19,291,120	2.02E-17	0.245	0.246				*MFAP4*	TSS1500
cg19701879	4	23,881,105	3.22E-05	0.245	0.249				*PPARGC1A*	Body
cg00161124	1	234,366,259	0.001124	0.245	0.181				*SLC35F3*	Body
cg05470554	7	120,969,079	0.000645	0.245	0.251				*WNT16*	TSS200
cg20438286	11	11,371,451	2.23E-14	0.245	0.241				*GALNTL4*	Body
cg03994959	13	108,866,993	0.000121	0.245	0.117				*LIG4*	5'UTR
cg16884902	11	20,617,974	0.002172	0.245	0.266					
cg09180848	15	83,953,780	0.001976	0.245	0.224				*BNC1*	TSS1500
cg21031345	11	121,800,497	0.010984	0.245	0.214					
cg06400704	1	240,256,644	0.040274	0.245	0.338				*FMN2*	1stExon
cg09597806	15	28,351,607	0.012453	0.245	0.092					
cg23318893	6	29,974,083	5.09E-06	0.245	0.255				*HLA-J*	Body
cg19348676	20	43,743,967	5.98E-16	0.245	0.246				*WFDC5*	TSS200
cg20822579	14	24,809,073	1.38E-07	0.245	0.222	2.87E-16	0.182		*RIPK3*	5'UTR
cg03410359	6	28,574,635	0.007741	0.245	0.241					

cg04468551	12	59,990,894	0.00494	0.245	0.339					
cg18020065	13	114,829,733	0.010308	0.245	0.140				*RASA3*	Body
cg26101485	8	142,264,769	8.04E-15	0.245	0.264					
cg15292765	10	34,405,759	3.51E-09	0.245	0.237				*PARD3*	Body
cg01408363	12	125,033,903	3.89E-12	0.245	0.230					
cg02621287	12	11,653,278	0.036009	0.245	0.250					
cg06865451	1	39,035,934	0.00038	0.245	0.208					
cg13957126	7	5,597,849	0.000514	0.245	0.214					
cg25009504	18	21,269,793	0.018907	0.245	0.255				*LAMA3*	1stExon
cg08486575	16	30,908,490	0.048305	0.245	0.332				*CTF1*	Body
cg05527507	1	861,265	0.000495	0.245	0.242				*SAMD11*	5'UTR
cg22957135	14	33,409,651	3.42E-09	0.245	0.237				*NPAS3*	Body
cg04190618	11	46,938,492	0.014385	0.245	0.180				*LRP4*	Body
cg04578903	1	21,754,899	1.47E-05	0.245	0.198					
cg18072282	22	37,976,192	8.1E-10	0.245	0.259				*LGALS2*	TSS200
cg05945782	17	1,954,986	1.43E-07	0.245	0.264				*MIR212*	TSS1500
cg13305444	1	225,838,196	0.000345	0.245	0.298				*ENAH*	Body
cg19868691	2	219,744,597	0.001566	0.245	0.271				*WNT10A*	TSS1500
cg16700920	10	133,810,060	1.17E-66	0.245	0.246					
cg27116888	18	76,746,337	0.038646	0.245	0.312				*SALL3*	Body
cg02213684	5	134,366,534	0.007835	0.245	0.315				*PITX1*	Body
cg03494022	18	43,992,823	1.96E-08	0.245	0.277				*RNF165*	Body
cg22192489	5	134,521,633	1.66E-10	0.245	0.217					
cg06913330	4	2,060,313	0.009389	0.245	0.125				*NAT8L*	TSS1500
cg07200957	5	58,334,676	7.84E-05	0.245	0.248				*PDE4D*	Body
cg11530678	15	67,081,012	3E-26	0.245	0.227					
cg06632098	10	43,605,906	7.02E-12	0.245	0.244				*RET*	Body
cg14463068	2	1,820,315	1.55E-06	0.245	0.239				*MYT1L*	Body
cg17877071	3	49,460,177	1.39E-12	0.245	0.246				*AMT*	TSS200
cg25752163	12	111,470,657	1.93E-10	0.245	0.249				*CUX2*	TSS1500
cg12317601	10	119,301,760	0.049922	0.244	0.315				*EMX2*	TSS200
cg14152222	11	68,780,661	2.03E-08	0.244	0.260				*MRGPRF*	5'UTR
cg17876497	10	88,731,295	0.002275	0.244	0.187				*AGAP11*	5'UTR
cg14718065	2	131,010,763	1.02E-12	0.244	0.192					

cg16034168	1	6,336,711	2.67E-11	0.244	0.234				*ACOT7*	Body
cg24585203	11	27,105,814	6.22E-17	0.244	0.236				*BBOX1*	Body
cg13288195	15	63,889,711	4.93E-13	0.244	0.234	4.09E-14	0.235		*FBXL22*	1stExon
cg14437725	9	137,787,387	0.000239	0.244	0.279					
cg25705148	2	72,079,498	5.87E-12	0.244	0.224					
cg10800464	12	109,162,641	0.012298	0.244	0.129					
cg18391937	14	55,539,537	0.000226	0.244	0.245					
cg05922610	6	108,495,985	3.11E-05	0.244	0.251				*NR2E1*	Body
cg08582356	8	86,351,018	4.88E-05	0.244	0.270				*CA3*	TSS200
cg13033722	6	43,111,240	1.66E-11	0.244	0.215				*PTK7*	Body
cg25403283	10	63,629,487	0.000501	0.244	0.241					
cg18742814	1	41,828,276	1.33E-05	0.244	0.246					
cg24994355	10	99,445,520	5.39E-07	0.244	0.208			Yes	*AVPI1*	5'UTR
cg01882113	2	206,724,023	1.2E-13	0.244	0.223					
cg03915432	2	101,428,924	3.9E-12	0.244	0.243					
cg04293152	16	3,085,468	1.71E-12	0.244	0.241				*CCDC64B*	1stExon
cg05260966	22	33,197,394	1.09E-08	0.244	0.227	3.58E-27	0.166		*SYN3*	Body
cg06556244	7	143,089,459	8E-11	0.244	0.243				*EPHA1*	Body
cg19485202	19	18,902,081	1.57E-05	0.244	0.192				*COMP*	1stExon
cg13459035	7	135,369,001	1.84E-15	0.244	0.247				*SLC13A4*	Body
cg17966344	7	102,071,463	0.001591	0.244	0.213					
cg06490225	17	35,298,825	1.56E-06	0.244	0.253				*LHX1*	Body
cg15493618	4	154,714,069	0.00382	0.244	0.277					
cg22730464	5	94,955,816	0.010047	0.244	0.202				*GPR150*	TSS200
cg23056703	3	5,470,788	1.01E-11	0.244	0.262					
cg02974898	1	23,105,600	9.97E-12	0.244	0.245				*EPHB2*	Body
cg16032102	1	206,680,459	0.001088	0.244	0.203				*RASSF5*	TSS1500
cg14578087	9	133,796,926	2.16E-20	0.244	0.247				*FIBCD1*	Body
cg18868483	6	71,667,067	0.025438	0.244	0.102				*B3GAT2*	TSS1500
cg03506640	14	85,997,753	0.008673	0.244	0.258				*FLRT2*	5'UTR
cg01734112	2	26,624,760	0.023427	0.244	0.056				*C2orf39*	TSS200
cg24128292	8	30,419,935	7.17E-14	0.244	0.241				*RBPMS*	3'UTR
cg15468976	1	2,106,222	2.83E-05	0.244	0.289				*PRKCZ*	Body
cg22386583	17	78,753,756	1.37E-14	0.244	0.233				*RPTOR*	Body

cg04166432	2	181,843,717	4.15E-11	0.244	0.204				*UBE2E3*	TSS1500
cg17827208	15	99,470,414	1.14E-10	0.244	0.225				*IGF1R*	Body
cg19375537	1	46,951,589	0.001323	0.244	0.240					
cg17075352	4	71,533,675	4.8E-11	0.244	0.267				*IGJ*	TSS1500
cg09420907	12	4,273,763	0.021069	0.244	0.266					
cg16004706	12	114,273,782	1.21E-06	0.244	0.193				*RBM19*	Body
cg23279335	11	75,863,631	6.14E-10	0.244	0.263					
cg23671279	8	140,969,608	1.56E-18	0.244	0.231				*TRAPPC9*	Body
cg00594099	1	27,893,886	2.28E-07	0.244	0.259				*AHDC1*	5'UTR
cg07501233	13	100,641,900	0.000101	0.244	0.232					
cg04517258	8	144,094,955	6.74E-09	0.244	0.217				*LOC100133669*	Body
cg05294243	19	51,569,106	0.00012	0.244	0.270	1.23E-12	0.168		*KLK13*	TSS1500
cg11814235	18	28,621,490	0.006255	0.244	0.256				*DSC3*	Body
cg16617301	19	46,999,109	4.45E-06	0.244	0.263				*PNMAL2*	1stExon
cg11124021	4	10,024,412	0.000376	0.244	0.176				*SLC2A9*	TSS1500
cg04052706	16	72,981,543	3.66E-13	0.244	0.222				*ZFHX3*	Body
cg23504411	6	27,859,160	0.000162	0.244	0.244				*HIST1H3J*	TSS1500
cg15539420	17	46,692,248	4.64E-06	0.244	0.191	2.57E-31	0.216		*HOXB8*	5'UTR
cg15717617	8	38,782,090	0.035615	0.244	0.155				*PLEKHA2*	Body
cg02809404	14	67,656,029	2.9E-17	0.244	0.213				*FAM71D*	TSS200
cg25105561	1	91,316,318	1.4E-06	0.244	0.218					
cg06009215	5	113,647,903	1.74E-20	0.244	0.251					
cg26037504	12	56,113,808	4.11E-11	0.244	0.249				*RDH5*	TSS1500
cg06196755	19	31,839,734	0.034395	0.244	0.118				*TSHZ3*	Body
cg11150074	1	200,863,559	1.76E-23	0.244	0.248				*C1orf106*	TSS1500
cg12793733	4	47,488,620	2.01E-15	0.244	0.222				*ATP10D*	5'UTR
cg14645027	12	52,642,706	9.14E-18	0.244	0.221				*KRT7*	3'UTR
cg13217590	2	6,122,107	0.003166	0.244	0.274				*LOC400940*	TSS200
cg04747322	5	121,647,308	0.03507	0.244	0.283	4.97E-18	0.199		*SNCAIP*	TSS1500
cg08848140	9	124,982,018	7.4E-08	0.244	0.241				*LHX6*	Body
cg10299976	10	94,455,543	9.97E-08	0.244	0.219					
cg19494587	16	1,398,004	1.68E-06	0.244	0.225				*BAIAP3*	Body
cg02523844	7	79,083,507	0.014323	0.244	0.206				*MAGI2*	TSS1500
cg17411190	22	19,510,877	8.7E-05	0.244	0.235				*CLDN5*	1stExon

cg27646965	2	238,814,032	0.000104	0.244	0.216				*RAMP1*	Body
cg07313162	5	34,587,700	6.71E-08	0.244	0.226					
cg21901277	2	132,152,297	9.04E-10	0.244	0.233					
cg15649236	17	46,657,504	0.000268	0.244	0.236				*MIR10A*	TSS200
cg20120491	22	38,965,934	4E-05	0.244	0.234	1.55E-16	0.154		*DMC1*	5'UTR
cg00306311	4	17,783,936	3.07E-08	0.244	0.224				*FAM184B*	TSS1500
cg12041387	7	96,650,171	8.25E-08	0.244	0.232	0.00714665	0.067		*DLX5*	Body
cg14482968	9	137,599,175	1.02E-09	0.244	0.240				*COL5A1*	Body
cg15371881	5	142,065,819	4.42E-10	0.244	0.215				*FGF1*	5'UTR
cg09621708	16	50,003,457	1.53E-09	0.244	0.216					
cg06179765	7	23,287,352	0.002184	0.244	0.272				*GPNMB*	Body
cg00253379	16	54,404,709	7.02E-06	0.244	0.221					
cg24879560	3	65,342,971	1.21E-10	0.244	0.224				*MAGI1*	Body
cg27457941	3	139,258,754	0.004591	0.244	0.109	4.2E-13	0.126		*RBP1*	TSS200
cg06845853	11	32,461,240	0.046135	0.244	0.275				*WIT1*	Body
cg07589381	1	10,764,512	1.2E-10	0.244	0.208				*CASZ1*	5'UTR
cg16877087	2	161,504,834	0.002131	0.244	0.190					
cg15909981	22	37,465,178	1.35E-07	0.244	0.265				*TMPRSS6*	Body
cg02341197	21	34,185,927	1.23E-13	0.244	0.230				*C21orf62*	5'UTR
cg15716680	11	12,864,037	0.023349	0.244	0.139				*TEAD1*	Body
cg12996903	3	50,275,575	0.029465	0.244	0.144				*GNAI2*	Body
cg27310024	15	90,950,101	1.3E-16	0.244	0.232				*IQGAP1*	Body
cg25365260	17	46,669,781	9.92E-08	0.244	0.219				*LOC404266*	Body
cg07413251	3	27,771,667	0.049512	0.244	0.219					
cg07279442	3	124,848,635	4.14E-21	0.244	0.227				*SLC12A8*	Body
cg23163200	21	34,186,122	8.17E-07	0.244	0.225				*C21orf62*	TSS200
cg26390660	3	110,344,165	2.68E-10	0.244	0.232					
cg16181043	6	31,239,175	0.004356	0.244	0.256				*HLA-C*	Body
cg00571041	3	62,352,633	0.001535	0.244	0.191			Yes		
cg05351887	16	3,988,869	0.02488	0.244	0.308					
cg05959170	3	62,353,312	0.000215	0.244	0.208					
cg26755097	6	30,711,844	0.007844	0.244	0.211				*FLOT1*	TSS1500
cg07785737	10	71,176,850	6.17E-19	0.244	0.239				*TACR2*	TSS200
cg27481559	2	87,037,038	1.06E-06	0.244	0.231					

cg11207353	19	18,508,493	5.93E-09	0.244	0.225				*LRRC25*	TSS200
cg26865084	6	169,223,825	7.64E-08	0.244	0.227					
cg01588379	7	151,433,326	1.2E-06	0.244	0.177				*PRKAG2*	Body
cg21182715	12	95,567,919	3.77E-06	0.244	0.210				*FGD6*	Body
cg05130081	7	157,867,264	4.51E-07	0.244	0.238				*PTPRN2*	Body
cg18512963	3	131,430,364	6.62E-11	0.244	0.227				*CPNE4*	Body
cg25256099	17	79,484,960	1.69E-09	0.244	0.230					
cg06379721	10	102,590,401	0.001162	0.244	0.234					
cg06794543	15	42,749,748	0.025562	0.244	0.180				*ZFP106*	TSS200
cg07914866	12	66,583,431	2.01E-05	0.244	0.263	2.79E-10	0.109		*IRAK3*	Body
cg26667550	1	156,098,772	3.94E-14	0.244	0.243				*LMNA*	Body
cg04497512	21	36,042,224	0.001907	0.244	0.174				*CLIC6*	1stExon
cg26551200	7	2,681,369	5.45E-14	0.244	0.239				*TTYH3*	Body
cg03455762	X	128,873,475	5.26E-14	0.244	0.257				*XPNPEP2*	Body
cg04414946	X	9,435,372	6.97E-21	0.244	0.258				*TBL1X*	5'UTR
cg05932042	17	40,822,653	1.13E-12	0.244	0.221				*PLEKHH3*	Body
cg15133719	2	223,163,804	0.000412	0.244	0.225				*PAX3*	TSS200
cg25707676	7	96,643,539	0.004406	0.244	0.277				*DLX6AS*	TSS200
cg07504393	15	67,136,043	0.029837	0.244	0.086					
cg08319130	6	100,038,353	0.0014	0.244	0.275					
cg08419450	10	90,013,989	0.00023	0.244	0.204					
cg22777433	14	100,784,665	0.027883	0.244	0.194					
cg25782983	11	74,950,764	1.87E-12	0.244	0.238					
cg05377162	1	185,286,996	0.001745	0.244	0.190				*IVNS1ABP*	TSS1500
cg27656459	12	116,796,562	5.09E-16	0.244	0.256					
cg10996148	17	925,701	1.89E-16	0.244	0.229				*ABR*	Body
cg13634242	5	60,921,891	0.032983	0.244	0.305					
cg04754315	4	57,522,801	1.72E-05	0.244	0.242				*HOPX*	5'UTR
cg06108782	10	50,817,213	0.003742	0.244	0.261				*SLC18A3*	TSS1500
cg19593490	6	29,943,188	1.26E-05	0.244	0.231				*HCG9*	Body
cg03001305	17	40,439,492	9.32E-09	0.244	0.231	2.78E-13	0.121		*STAT5A*	TSS200
cg11532433	2	24,735,298	2.49E-07	0.244	0.239					
cg10447977	3	64,598,405	3.46E-07	0.244	0.272				*ADAMTS9*	Body
cg16263152	1	183,287,819	0.003722	0.244	0.265				*NMNAT2*	Body

cg17861277	5	150,385,659	7.04E-12	0.244	0.231					
cg14155416	18	6,414,330	0.001206	0.244	0.230	1.51E-13	0.158		*L3MBTL4*	5'UTR
cg11316887	10	29,924,694	0.005635	0.244	0.197				*SVIL*	TSS1500
cg16401770	7	112,739,805	3.02E-06	0.244	0.240					
cg24384918	7	43,153,950	0.000681	0.244	0.269				*HECW1*	5'UTR
cg15126544	2	171,678,954	0.004022	0.244	0.263				*GAD1*	Body
cg06967828	10	73,723,753	0.000375	0.244	0.160				*CHST3*	TSS1500
cg24516901	2	225,266,801	0.014623	0.244	0.283	1.35E-07	0.143		*FAM124B*	TSS200
cg05316463	14	95,786,663	0.001641	0.244	0.210				*CLMN*	TSS1500
cg05900140	12	6,560,162	0.046027	0.243	0.085				*TAPBPL*	TSS1500
cg08488945	3	181,422,202	0.000176	0.243	0.264				*SOX2OT*	Body
cg22336004	1	66,258,035	0.004203	0.243	0.222				*PDE4B*	TSS200
cg09940188	17	1,686,671	0.014567	0.243	0.131				*SMYD4*	Body
cg11702085	6	28,956,804	1.26E-05	0.243	0.244					
cg15808426	19	57,154,302	0.00459	0.243	0.234					
cg05374654	11	94,134,666	1.48E-05	0.243	0.180				*GPR83*	TSS200
cg06103624	15	66,998,926	4.13E-39	0.243	0.232				*SMAD6*	Body
cg18120446	4	13,531,076	0.00093	0.243	0.239					
cg00711090	19	57,018,614	0.001932	0.243	0.213				*ZNF471*	TSS1500
cg07130150	12	96,883,863	0.01411	0.243	0.059					
cg26735846	10	124,894,943	6.51E-06	0.243	0.147				*HMX3*	TSS1500
cg12926596	12	56,111,944	1.46E-12	0.243	0.252			Yes	*BLOC1S1*	Body
cg12597472	11	69,339,188	0.00044	0.243	0.218					
cg07496545	5	140,683,737	1.53E-10	0.243	0.255				*SLC25A2*	TSS200
cg08070646	12	52,616,097	1.05E-10	0.243	0.252				*LOC283404*	Body
cg11319389	20	42,544,648	1.8E-05	0.243	0.242	5.83E-11	0.134		*TOX2*	TSS200
cg15120732	6	73,330,106	0.00661	0.243	0.275				*KCNQ5*	TSS1500
cg23655651	12	20,365,720	0.004717	0.243	0.326					
cg25254074	6	41,607,326	3.28E-30	0.243	0.241				*MDFI*	Body
cg02381853	5	33,321,127	5.06E-09	0.243	0.228					
cg23542968	14	36,983,129	0.002048	0.243	0.251				*SFTA3*	TSS200
cg06368184	21	38,069,416	0.006505	0.243	0.270					
cg06878361	19	52,390,810	0.006927	0.243	0.219				*ZNF577*	Body
cg11461670	8	22,454,935	2.2E-11	0.243	0.236				*PDLIM2*	3'UTR

cg04854637	11	134,285,140	2.22E-10	0.243	0.230					
cg01172640	15	53,096,035	0.006172	0.243	0.179					
cg23033518	14	59,101,719	0.005111	0.243	0.200					
cg25436886	3	127,056,972	0.000157	0.243	0.216					
cg11813265	14	95,238,671	1.7E-06	0.243	0.243					
cg22210157	3	46,853,986	0.004068	0.243	0.258					
cg23262036	7	96,647,021	1.47E-08	0.243	0.248					
cg01794103	15	78,342,494	0.008324	0.243	0.217				*TBC1D2B*	Body
cg16255156	1	113,265,709	1.48E-23	0.243	0.227				*FAM19A3*	Body
cg07436152	6	50,819,236	0.007002	0.243	0.244					
cg24641186	6	50,804,147	0.000126	0.243	0.240				*TFAP2B*	Body
cg03730249	1	42,692,013	4.63E-19	0.243	0.233				*FOXJ3*	Body
cg15754594	2	106,184,688	6.59E-09	0.243	0.236					
cg09427605	4	146,740,968	0.021729	0.243	0.319				*ZNF827*	Body
cg09654300	3	42,132,991	7.87E-13	0.243	0.238				*TRAK1*	1stExon
cg26212328	12	46,767,665	0.000106	0.243	0.235				*SLC38A2*	TSS1500
cg03835609	5	140,753,418	0.025891	0.243	0.301				*PCDHGA4*	Body
cg00985729	6	30,712,559	0.016794	0.243	0.238				*IER3*	TSS1500
cg03762994	17	6,899,332	4.23E-06	0.243	0.192				*ALOX12*	TSS200
cg09789768	1	119,524,323	4.27E-10	0.243	0.236				*TBX15*	5'UTR
cg11524235	1	33,351,587	4.19E-08	0.243	0.210				*HPCA*	TSS1500
cg07308257	7	36,265,036	1.97E-15	0.243	0.239				*EEPD1*	Body
cg15253816	17	66,255,259	0.000269	0.243	0.252				*ARSG*	TSS200
cg08602500	7	157,996,648	7.09E-14	0.243	0.226				*PTPRN2*	Body
cg15246590	19	48,983,600	4.86E-19	0.243	0.247				*CYTH2*	3'UTR
cg22175873	5	140,801,482	6.15E-05	0.243	0.233				*PCDHGA4*	Body
cg12271487	6	109,266,451	0.000264	0.243	0.255				*ARMC2*	Body
cg21211253	5	139,168,323	1.06E-09	0.243	0.240					
cg01209635	19	3,607,269	1.28E-08	0.243	0.222				*TBXA2R*	TSS1500
cg04619882	1	109,741,037	3.64E-05	0.243	0.345				*KIAA1324*	Body
cg14819504	2	160,761,413	0.034352	0.243	0.218				*LY75*	TSS200
cg03859090	11	69,522,216	2.93E-11	0.243	0.219			Yes		
cg11173131	20	45,179,226	2.23E-14	0.243	0.240				*C20orf123*	TSS200
cg26697320	7	26,437,681	0.015932	0.243	0.151					

cg00401972	16	66,400,404	3.41E-07	0.243	0.203				*CDH5*	TSS200
cg06820621	5	92,938,232	3.98E-14	0.243	0.233					
cg06884718	6	32,191,821	4.31E-06	0.243	0.261				*NOTCH4*	1stExon
cg18394340	5	134,735,613	0.000109	0.243	0.208				*H2AFY*	TSS1500
cg14692854	2	97,198,126	9.51E-06	0.243	0.295					
cg02818811	12	75,728,458	0.022893	0.243	0.211				*GLIPR1L1*	TSS200
cg24799460	15	102,251,213	4.95E-07	0.243	0.233				*TARSL2*	Body
cg05178734	15	59,729,760	0.000468	0.243	0.247				*FAM81A*	TSS1500
cg17125585	1	68,176,471	1.64E-10	0.243	0.234				*GNG12*	5'UTR
cg23426473	7	155,255,232	0.001544	0.243	0.261				*EN2*	Body
cg13986840	7	130,132,453	3.62E-06	0.243	0.234				*MEST*	5'UTR
cg23876056	6	24,950,870	7.63E-14	0.243	0.244					
cg00044796	1	146,552,390	3.6E-06	0.243	0.250					
cg03217471	6	11,687,689	4.65E-24	0.243	0.237					
cg15473017	1	166,853,615	0.010875	0.243	0.151					
cg11718030	11	27,744,363	1.18E-05	0.243	0.254				*BDNF*	TSS1500
cg02852327	7	121,950,975	1.81E-06	0.243	0.231					
cg00765705	12	124,865,130	3.41E-16	0.243	0.237				*NCOR2*	Body
cg02967969	11	66,443,874	1.76E-16	0.243	0.255				*RBM4B*	Body
cg08671647	4	30,724,564	1.4E-07	0.243	0.241				*PCDH7*	1stExon
cg22904737	17	36,735,245	5.42E-07	0.243	0.204				*SRCIN1*	Body
cg18131141	1	36,043,002	3.47E-05	0.243	0.236				*TFAP2E*	Body
cg20320575	14	24,458,099	3.51E-19	0.243	0.231				*DHRS4L2*	5'UTR
cg05783998	6	74,295,921	0.000948	0.243	0.240					
cg18810664	10	31,074,367	0.021816	0.243	0.139					
cg26554505	3	55,516,711	0.001792	0.243	0.180				*WNT5A*	Body
cg03346415	16	69,854,895	1.5E-14	0.243	0.222				*WWP2*	Body
cg07383092	5	172,663,058	0.000157	0.243	0.237				*NKX2-5*	TSS1500
cg08660876	4	154,681,323	3.36E-05	0.243	0.220				*RNF175*	5'UTR
cg04268950	11	130,298,854	0.002113	0.243	0.254				*ADAMTS8*	TSS1500
cg08693172	19	46,526,333	0.000605	0.243	0.169				*PGLYRP1*	TSS200
cg17984022	14	105,156,566	0.002301	0.243	0.203				*INF2*	5'UTR
cg13198709	10	102,473,456	8.98E-05	0.243	0.250					
cg17311074	15	71,507,695	1.44E-14	0.243	0.219				*THSD4*	Body

cg07835844	2	45,879,684	0.045082	0.243	0.198				*PRKCE*	Body
cg16553500	1	32,169,868	0.001444	0.243	0.241				*COL16A1*	TSS200
cg19636519	7	99,541,626	1.29E-12	0.243	0.246					
cg02448922	1	21,754,947	0.002791	0.243	0.223					
cg03803008	7	70,055,327	3.95E-19	0.243	0.226				*AUTS2*	Body
cg14037728	9	116,645,936	8.04E-07	0.243	0.284				*ZNF618*	Body
cg11094122	20	35,579,338	0.023535	0.243	0.070				*SAMHD1*	Body
cg00161225	17	3,814,785	8.48E-07	0.243	0.214				*P2RX1*	Body
cg11770664	10	45,470,023	4.32E-17	0.243	0.192				*RASSF4*	Body
cg12562076	6	137,808,930	3.76E-06	0.243	0.274					
cg20873416	6	30,737,068	0.001661	0.243	0.291					
cg24539517	10	121,161,258	1.75E-06	0.243	0.205				*GRK5*	Body
cg16169604	2	131,485,486	0.000455	0.243	0.225	1.02E-14	0.113		*GPR148*	TSS1500
cg12038696	1	36,043,352	3.05E-05	0.243	0.231				*TFAP2E*	Body
cg12277495	16	66,462,184	3.49E-06	0.243	0.204				*BEAN*	5'UTR
cg13164537	18	67,624,071	2.77E-07	0.243	0.216	5.37E-16	0.182		*CD226*	5'UTR
cg17859448	1	101,703,749	0.005358	0.243	0.277				*S1PR1*	5'UTR
cg19230867	3	42,103,367	9.65E-10	0.243	0.271					
cg24447042	X	128,657,893	0.005259	0.243	0.159	8.08E-23	0.222		*SMARCA1*	TSS1500
cg24500441	15	66,274,709	0.032492	0.243	0.198				*MEGF11*	Body
cg04436554	5	172,669,976	0.000389	0.243	0.241					
cg22698177	15	49,207,945	4.65E-05	0.243	0.245				*SHC4*	Body
cg15542798	1	232,941,253	0.049813	0.243	0.178				*KIAA1383*	1stExon
cg08971771	17	48,049,979	0.00021	0.243	0.175				*DLX4*	TSS200
cg00014998	2	177,014,962	7.63E-12	0.243	0.205				*HOXD4*	TSS1500
cg00048743	5	140,782,367	0.000327	0.243	0.275				*PCDHGA4*	Body
cg11448896	1	50,889,182	0.00445	0.243	0.206				*DMRTA2*	TSS200
cg12252069	7	26,897,597	8.58E-10	0.243	0.226				*SKAP2*	Body
cg17161718	6	33,160,156	4.86E-06	0.243	0.173				*COL11A2*	5'UTR
cg07113230	1	8,277,394	0.024985	0.243	0.129					
cg09153895	17	58,179,869	0.000126	0.243	0.231				*LOC653653*	Body
cg18985133	8	39,835,730	5.48E-10	0.243	0.163				*IDO2*	Body
cg01624229	2	153,252,785	4.2E-20	0.243	0.254				*FMNL2*	Body
cg24720355	1	119,526,255	5.12E-12	0.243	0.212				*TBX15*	5'UTR

cg12379940	13	44,947,923	0.016715	0.243	0.250				*SERP2*	TSS200
cg00574563	17	35,299,270	0.002058	0.243	0.223				*LHX1*	Body
cg01880463	20	45,179,413	1.05E-11	0.243	0.236				*C20orf123*	TSS1500
cg18345219	1	43,919,084	9.22E-05	0.243	0.193				*HYI*	Body
cg22382836	19	723,036	1.4E-06	0.243	0.263				*PALM*	Body
cg27175093	14	57,265,910	0.006038	0.243	0.229					
cg19223579	10	115,370,894	0.017247	0.243	0.243				*NRAP*	Body
cg04180086	5	1,878,499	5.23E-08	0.243	0.239				*IRX4*	Body
cg08546494	2	102,867,826	0.037444	0.243	0.175					
cg20352178	15	69,099,262	8.79E-23	0.243	0.230				*C15orf28*	Body
cg00128482	8	75,737,456	0.003133	0.243	0.303				*PI15*	5'UTR
cg01192554	11	70,228,615	0.000783	0.243	0.192			Yes	*PPFIA1*	3'UTR
cg02505749	8	140,715,735	0.000876	0.243	0.303				*KCNK9*	TSS1500
cg03049691	17	27,039,032	8.09E-06	0.243	0.231				*PROCA1*	TSS200
cg26632897	17	77,030,546	1.95E-07	0.243	0.192				*C1QTNF1*	1stExon
cg00241281	14	105,779,794	5.39E-18	0.243	0.225				*PACS2*	TSS1500
cg25301767	12	49,731,393	1.6E-12	0.243	0.215				*C1QL4*	TSS1500
cg00305071	1	34,504,017	0.00176	0.243	0.231				*CSMD2*	Body
cg04958794	14	97,685,274	0.000247	0.243	0.297					
cg18952647	15	83,953,744	0.008044	0.243	0.207	3.35E-09	0.133		*BNC1*	TSS1500
cg16224951	6	31,528,154	1.66E-13	0.243	0.229					
cg01190692	12	106,976,711	0.008914	0.243	0.208				*RFX4*	TSS1500
cg02567839	1	242,612,787	3.38E-08	0.243	0.237				*PLD5*	5'UTR
cg09567511	4	6,155,914	3.78E-22	0.243	0.232				*JAKMIP1*	5'UTR
cg15251385	12	8,025,582	3.11E-07	0.243	0.238				*SLC2A14*	TSS200
cg16705744	3	27,756,039	0.003623	0.243	0.204					
cg22537474	20	10,198,659	0.000447	0.243	0.222				*SNAP25*	TSS1500
cg04561727	14	104,010,865	6.27E-08	0.243	0.251			Yes		
cg14626309	4	299,051	0.008517	0.243	0.207					
cg07812877	19	11,531,469	0.000766	0.243	0.260				*RGL3*	TSS1500
cg11201710	3	192,232,468	0.002926	0.243	0.236				*FGF12*	Body
cg11332519	12	26,348,093	9.51E-05	0.243	0.189				*SSPN*	TSS200
cg25718035	10	8,085,029	1.98E-06	0.242	0.239					
cg26102728	7	28,218,933	0.005655	0.242	0.224				*JAZF1*	Body

cg12875241	X	9,734,063	5.95E-19	0.242	0.236				*GPR143*	TSS200
cg24694743	1	32,088,013	1.73E-07	0.242	0.241				*HCRTR1*	Body
cg02432280	7	152,622,368	0.002539	0.242	0.248					
cg02940147	11	124,747,263	1E-22	0.242	0.227				*ROBO3*	Body
cg17206850	11	62,693,456	3.63E-05	0.242	0.246					
cg08315174	14	36,990,430	0.000351	0.242	0.217			Yes	*NKX2-1*	TSS1500
cg05704955	16	70,759,914	2.16E-17	0.242	0.243				*VAC14*	Body
cg13885965	6	30,712,409	0.031837	0.242	0.237				*IER3*	TSS200
cg07690127	7	2,077,293	5.06E-10	0.242	0.235				*MAD1L1*	Body
cg09904497	18	76,737,834	0.000316	0.242	0.257					
cg23165310	3	181,422,217	3.43E-06	0.242	0.231				*SOX2OT*	Body
cg24897141	5	139,283,199	0.001343	0.242	0.279				*NRG2*	Body
cg12510708	7	26,193,805	3.81E-11	0.242	0.219				*NFE2L3*	Body
cg17299899	18	76,738,478	0.023004	0.242	0.270					
cg21587238	5	42,953,590	0.0305	0.242	0.238					
cg22027433	17	62,208,049	2.96E-06	0.242	0.201				*ERN1*	TSS1500
cg22085751	6	28,921,976	0.028643	0.242	0.217					
cg23702727	17	73,642,607	5.57E-05	0.242	0.189				*RECQL5*	Body
cg23694533	17	40,806,193	0.022855	0.242	0.146					
cg17580935	3	65,583,240	6.35E-08	0.242	0.252				*MAGI1*	Body
cg21608192	16	17,563,827	0.012756	0.242	0.157	1.27E-12	0.120		*XYLT1*	Body
cg12714665	11	15,739,896	1.46E-08	0.242	0.244					
cg14916079	10	120,788,696	0.000156	0.242	0.168				*NANOS1*	TSS1500
cg26326607	11	1,976,270	5.86E-22	0.242	0.240				*MRPL23*	Body
cg04224247	X	9,984,515	0.024852	0.242	0.215				*WWC3*	5'UTR
cg22661893	11	89,224,559	0.01382	0.242	0.260				*NOX4*	1stExon
cg24685006	1	36,043,014	2.07E-05	0.242	0.237				*TFAP2E*	Body
cg14653977	9	136,038,692	5.95E-11	0.242	0.228				*GBGT1*	5'UTR
cg18185665	17	16,257,111	0.000241	0.242	0.146				*CENPV*	TSS1500
cg19513727	12	110,708,125	1.24E-07	0.242	0.191					
cg23122901	22	19,880,135	1.19E-06	0.242	0.235				*TXNRD2*	Body
cg25073700	7	157,628,207	1.4E-07	0.242	0.251				*PTPRN2*	Body
cg27564792	3	62,360,162	0.024617	0.242	0.316				*FEZF2*	TSS1500
cg04767734	2	172,958,009	0.018707	0.242	0.258					

cg05377949	3	127,951,287	8.23E-14	0.242	0.240				*EEFSEC*	Body
cg14035238	2	175,499,365	0.03454	0.242	0.210				*WIPF1*	5'UTR
cg15928347	2	64,837,018	1.64E-05	0.242	0.247					
cg20971045	5	53,075,687	0.000368	0.242	0.245					
cg04805181	15	66,914,759	0.044127	0.242	0.187					
cg12569013	8	11,549,572	0.003521	0.242	0.180					
cg16725984	16	89,735,184	1.09E-06	0.242	0.193				*C16orf55*	Body
cg24946795	12	52,115,674	0.001539	0.242	0.272				*SCN8A*	Body
cg04972759	5	180,596,634	0.004162	0.242	0.311					
cg07830160	1	159,880,327	0.000117	0.242	0.227					
cg10005224	12	54,424,964	7.42E-05	0.242	0.197				*HOXC4*	5'UTR
cg16265348	20	25,129,542	3.7E-06	0.242	0.224				*LOC284798*	TSS1500
cg11750133	10	93,647,217	0.022257	0.242	0.242					
cg06394874	19	22,235,850	0.00296	0.242	0.312				*ZNF257*	Body
cg05281603	11	111,384,124	3.68E-07	0.242	0.222				*BTG4*	TSS1500
cg06979669	11	119,835,317	0.009755	0.242	0.232					
cg03479065	3	134,124,988	1.38E-05	0.242	0.260					
cg04157138	14	58,331,898	6.04E-06	0.242	0.195					
cg09521719	2	237,416,370	0.000185	0.242	0.243				*IQCA1*	TSS1500
cg14044388	9	94,579,826	3.12E-19	0.242	0.241				*ROR2*	Body
cg03719428	12	54,376,094	6.14E-18	0.242	0.223					
cg06553841	7	120,628,572	0.001467	0.242	0.171				*C7orf58*	TSS1500
cg22581896	7	4,779,225	1E-11	0.242	0.216				*FOXK1*	Body
cg24530246	3	53,118,167	3.04E-22	0.242	0.224					
cg24838316	6	29,895,260	1.15E-07	0.242	0.191					
cg24828683	6	29,856,210	0.016475	0.242	0.221				*HLA-H*	Body
cg02614129	5	134,733,615	0.002682	0.242	0.227				*H2AFY*	5'UTR
cg20006618	4	186,652,454	6.16E-16	0.242	0.214				*SORBS2*	5'UTR
cg22258315	8	48,675,723	2.19E-06	0.242	0.177					
cg01718116	2	115,919,829	0.015748	0.242	0.328				*DPP10*	Body
cg24398450	12	109,029,099	1.08E-05	0.242	0.262				*SELPLG*	TSS1500
cg23643375	17	45,933,306	0.011495	0.242	0.225				*SP6*	TSS200
cg09092713	X	119,133,965	0.043087	0.242	0.262					
cg01574481	14	24,641,852	1.33E-17	0.242	0.231				*REC8*	Body

cg04529406	X	118,897,145	1.58E-05	0.242	0.267					
cg21274067	2	114,341,111	9.49E-05	0.242	0.158				*WASH2P*	TSS200
cg01022219	18	13,641,735	3.98E-16	0.242	0.224				*C18orf1*	Body
cg04125350	21	36,042,344	0.001156	0.242	0.221				*CLIC6*	1stExon
cg20910490	14	37,117,947	0.000731	0.242	0.249					
cg25910261	7	157,405,965	5.94E-10	0.242	0.230				*PTPRN2*	Body
cg26033466	7	96,644,782	0.034032	0.242	0.211				*DLX6AS*	TSS1500
cg01295392	12	108,169,569	3.43E-07	0.242	0.234				*ASCL4*	1stExon
cg18536148	17	59,533,844	1.19E-10	0.242	0.212	0.00879198	0.052		*TBX4*	5'UTR
cg00587115	7	47,621,025	2.57E-05	0.242	0.213					
cg06688989	6	41,606,869	0.012867	0.242	0.157				*MDFI*	Body
cg08258582	1	48,058,985	0.001789	0.242	0.182					
cg05584361	11	1,481,525	3.63E-17	0.242	0.248				*BRSK2*	Body
cg08560874	6	29,759,947	0.037905	0.242	0.269				*HCG4*	Body
cg10158541	13	37,006,265	7.57E-06	0.242	0.206				*CCNA1*	1stExon
cg16304656	7	641,337	2.76E-06	0.242	0.242				*PRKAR1B*	Body
cg11357670	13	114,143,948	0.003249	0.242	0.160				*DCUN1D2*	Body
cg27348266	14	104,583,972	4.43E-09	0.242	0.193					
cg27358469	18	55,107,176	0.000661	0.242	0.284				*ONECUT2*	Body
cg04406436	1	38,942,313	0.012933	0.242	0.257					
cg06549607	13	110,440,352	0.001822	0.242	0.170				*IRS2*	TSS1500
cg17918501	2	27,717,709	0.022276	0.242	0.132	3.17E-12	0.106		*FNDC4*	5'UTR
cg07709148	8	29,387,238	0.000313	0.242	0.213					
cg07823492	17	46,608,099	6.57E-09	0.242	0.245				*HOXB1*	1stExon
cg07941927	13	100,644,041	0.011348	0.242	0.238					
cg20092892	12	52,258,077	0.006017	0.242	0.260					
cg23981871	2	177,028,714	0.00587	0.242	0.238				*HOXD3*	TSS200
cg26279550	12	66,582,805	0.03876	0.242	0.159				*IRAK3*	TSS200
cg00849692	6	99,295,724	6.11E-06	0.242	0.168					
cg07355189	10	45,923,374	2.39E-11	0.242	0.256				*ALOX5*	Body
cg00463767	2	63,282,043	1.76E-06	0.242	0.241				*OTX1*	Body
cg03483632	11	35,639,276	3.66E-11	0.242	0.213				*FJX1*	TSS1500
cg14480446	5	87,989,154	0.045812	0.242	0.249					
cg01106572	7	150,744,384	9.96E-29	0.242	0.231				*ACCN3*	TSS1500

cg04234474	10	119,312,950	7.43E-07	0.242	0.239					
cg14658867	20	443,632	0.001282	0.242	0.286				*TBC1D20*	TSS1500
cg26661034	1	18,957,335	0.026355	0.242	0.291				*PAX7*	TSS200
cg00646019	15	29,034,163	0.000992	0.242	0.263					
cg08884571	19	45,901,453	0.000377	0.242	0.262				*PPP1R13L*	Body
cg11920122	18	11,751,556	5.52E-07	0.242	0.236				*GNAL*	TSS200
cg19233001	1	201,346,876	0.004686	0.242	0.288				*TNNT2*	TSS200
cg06285727	11	72,524,028	4.58E-12	0.242	0.231				*ATG16L2*	TSS1500
cg21252909	X	30,326,328	0.000184	0.242	0.292				*NR0B1*	1stExon
cg24041541	8	77,586,395	0.002506	0.242	0.288				*LOC100192378*	Body
cg03239178	12	103,358,881	0.005342	0.242	0.269					
cg04893741	8	142,692,382	0.002084	0.242	0.284					
cg20491991	1	167,683,013	1.98E-06	0.242	0.243					
cg22337605	1	12,227,417	0.008642	0.242	0.217				*TNFRSF1B*	Body
cg21350860	2	173,080,153	4.41E-05	0.242	0.203					
cg15681626	11	64,739,253	0.020296	0.242	0.209					
cg19700787	6	3,997,547	2.1E-11	0.242	0.236					
cg18099096	4	23,936,764	0.000792	0.242	0.265					
cg14900471	8	11,561,620	0.002556	0.242	0.265				*GATA4*	TSS200
cg08481464	10	77,871,618	4.06E-13	0.242	0.218				*C10orf11*	Body
cg24475782	1	119,530,932	0.029984	0.242	0.208				*TBX15*	5'UTR
cg01956614	15	62,838,506	4.81E-11	0.242	0.245					
cg10513852	12	81,109,337	1.25E-10	0.242	0.231				*MYF5*	TSS1500
cg23341085	2	149,063,092	0.000207	0.242	0.193					
cg20415053	1	26,527,928	4.41E-05	0.242	0.243				*CATSPER4*	Body
cg23072383	22	31,031,044	9.94E-05	0.242	0.236				*SLC35E4*	TSS1500
cg26185508	1	54,619,445	9.71E-14	0.242	0.185				*CDCP2*	TSS200
cg00970313	14	37,125,914	5.39E-05	0.242	0.260				*PAX9*	TSS1500
cg06085204	17	6,659,164	1.45E-05	0.242	0.196				*XAF1*	5'UTR
cg11008243	6	73,331,238	0.023863	0.242	0.319				*KCNQ5*	TSS1500
cg08536841	19	49,250,452	3.96E-07	0.242	0.239	2.61E-13	0.164		*IZUMO1*	TSS1500
cg19808620	1	153,514,442	0.001725	0.242	0.260				*S100A5*	TSS1500
cg10881071	1	24,612,045	0.000277	0.242	0.225			Yes		
cg12368542	13	50,570,250	0.00802	0.242	0.115				*TRIM13*	TSS1500

cg26575738	17	44,896,168	5.71E-05	0.242	0.277				*WNT3*	TSS200
cg07458308	5	134,827,512	0.000943	0.242	0.184					
cg12892303	17	42,733,592	0.006051	0.242	0.263				*C17orf104*	TSS200
cg25027832	2	176,948,544	2.14E-05	0.242	0.235				*EVX2*	5'UTR
cg06535308	11	17,497,693	2.73E-06	0.242	0.226				*ABCC8*	Body
cg09853936	1	3,388,196	8.82E-15	0.242	0.236				*ARHGEF16*	Body
cg25813936	9	36,276,879	5.34E-08	0.242	0.249				*GNE*	Body
cg01408010	3	150,102,779	3.96E-06	0.242	0.232					
cg12463851	2	237,477,186	0.001814	0.242	0.138				*CXCR7*	TSS1500
cg10424417	5	87,989,191	0.00055	0.242	0.258					
cg22370057	6	12,957,918	0.001363	0.242	0.216				*PHACTR1*	Body
cg10814005	14	91,711,041	2.32E-05	0.242	0.209				*GPR68*	5'UTR
cg24889694	8	101,522,535	0.004793	0.242	0.127					
cg26487157	2	70,313,295	0.039708	0.242	0.271				*PCBP1*	TSS1500
cg26057751	2	87,016,259	6.41E-09	0.242	0.237				*CD8A*	Body
cg08875705	12	6,665,330	0.00026	0.242	0.243				*IFFO1*	TSS200
cg00328965	X	129,243,062	2.38E-09	0.242	0.229				*ELF4*	5'UTR
cg27203560	3	193,629,645	3.76E-09	0.242	0.241					
cg00821400	14	55,895,678	4.46E-12	0.242	0.254				*TBPL2*	Body
cg25578028	17	7,905,704	1.77E-06	0.242	0.244				*GUCY2D*	TSS1500
cg03744440	17	73,584,108	2.68E-05	0.242	0.235				*MYO15B*	TSS200
cg07870999	7	155,260,895	9.3E-05	0.242	0.192					
cg07098902	2	17,720,407	0.015439	0.242	0.240				*VSNL1*	TSS1500
cg27470373	2	64,836,764	0.000357	0.242	0.255					
cg01782024	1	91,989,267	6.98E-05	0.242	0.249				*CDC7*	Body
cg04961582	14	95,239,464	8.98E-07	0.241	0.238					
cg12877039	5	140,857,192	1.16E-06	0.241	0.207				*PCDHGA4*	Body
cg18257810	3	54,121,929	0.005805	0.241	0.309					
cg23847381	18	5,544,031	0.039282	0.241	0.350				*EPB41L3*	TSS200
cg05812089	11	19,736,648	6.99E-05	0.241	0.203				*LOC100126784*	TSS1500
cg13732857	2	45,029,729	0.014287	0.241	0.195					
cg19031085	5	135,228,288	2.24E-14	0.241	0.243				*IL9*	Body
cg21163960	11	35,441,777	1.44E-05	0.241	0.251				*SLC1A2*	TSS1500
cg04506114	1	179,322,762	2.94E-14	0.241	0.236				*SOAT1*	Body

cg13072717	3	186,743,989	5.24E-05	0.241	0.190				*ST6GAL1*	5'UTR
cg16820186	11	17,373,103	0.035442	0.241	0.043				*DKFZp686O24166*	TSS1500
cg13416381	17	46,090,231	3.64E-09	0.241	0.200					
cg04902729	1	181,452,707	0.004991	0.241	0.234				*CACNA1E*	TSS200
cg06772309	1	17,571,896	3.3E-07	0.241	0.225				*PADI1*	3'UTR
cg11155356	8	134,114,892	3.53E-12	0.241	0.220				*TG*	Body
cg06583412	X	102,001,318	0.029872	0.241	0.096				*BHLHB9*	5'UTR
cg05965392	8	53,478,624	0.016586	0.241	0.210				*FAM150A*	TSS1500
cg15802091	14	65,009,101	0.000127	0.241	0.231				*HSPA2*	1stExon
cg01710189	8	22,454,888	2.67E-09	0.241	0.241				*PDLIM2*	3'UTR
cg25020286	1	18,956,947	2.25E-05	0.241	0.218				*PAX7*	TSS1500
cg02017534	13	46,425,999	0.001603	0.241	0.206				*SIAH3*	TSS200
cg02516134	7	134,575,187	5.5E-09	0.241	0.269				*CALD1*	TSS1500
cg24848035	8	101,118,525	8.78E-06	0.241	0.286				*RGS22*	TSS200
cg04308769	1	200,007,623	1.12E-08	0.241	0.218				*NR5A2*	Body
cg08000684	19	11,530,025	0.000857	0.241	0.229				*RGL3*	TSS200
cg24416660	17	73,865,998	4.98E-21	0.241	0.244					
cg10002860	11	1,482,176	0.000164	0.241	0.244				*BRSK2*	Body
cg27423669	13	97,766,320	4.32E-07	0.241	0.205					
cg23415995	4	128,704,651	0.015759	0.241	0.258				*HSPA4L*	Body
cg01804925	6	27,107,588	7.23E-06	0.241	0.257				*HIST1H2BK*	3'UTR
cg09872737	6	32,076,270	1.37E-06	0.241	0.268				*TNXB*	5'UTR
cg14727777	4	140,201,652	2.82E-05	0.241	0.212				*C4orf49*	TSS200
cg17920936	9	94,710,553	1.49E-06	0.241	0.191				*ROR2*	Body
cg03526459	1	146,549,940	1.38E-10	0.241	0.254					
cg23490638	6	28,557,467	0.001063	0.241	0.194					
cg26187308	8	70,947,440	0.000108	0.241	0.224					
cg01294808	5	3,599,686	5.15E-14	0.241	0.224				*IRX1*	Body
cg08624472	7	98,971,852	0.007726	0.241	0.191				*ARPC1B*	TSS1500
cg08044097	8	55,371,013	5.01E-05	0.241	0.320				*SOX17*	Body
cg09832911	10	45,923,678	2.99E-09	0.241	0.254				*ALOX5*	Body
cg00752099	4	94,748,619	5.71E-07	0.241	0.241				*ATOH1*	TSS1500
cg04937144	17	46,832,631	3.26E-06	0.241	0.233					
cg13521842	14	101,036,202	1.13E-08	0.241	0.233				*BEGAIN*	TSS200

cg19995654	12	66,628,777	0.000431	0.241	0.209				*IRAK3*	Body
cg04027328	1	11,372,138	1.3E-15	0.241	0.216					
cg04365481	1	17,086,061	1.96E-05	0.241	0.209				*MST1P9*	Body
cg14397893	2	23,777,302	3.9E-15	0.241	0.226				*KLHL29*	5'UTR
cg06445944	5	67,586,928	0.01906	0.241	0.139				*PIK3R1*	Body
cg09808235	1	2,542,588	0.000304	0.241	0.215				*MMEL1*	Body
cg24158160	1	151,693,222	1.19E-05	0.241	0.208				*C1orf230*	TSS1500
cg13943217	9	133,542,125	0.029291	0.241	0.233				*PRDM12*	Body
cg16086559	15	96,911,531	0.002581	0.241	0.216					
cg10427430	11	12,783,675	4.59E-13	0.241	0.221				*TEAD1*	5'UTR
cg11384924	6	33,160,224	4.41E-06	0.241	0.241				*COL11A2*	5'UTR
cg17817168	12	22,486,454	3.64E-05	0.241	0.216				*ST8SIA1*	Body
cg23462514	4	1,107,585	0.048379	0.241	0.158				*RNF212*	TSS200
cg26595520	7	127,671,033	0.011484	0.241	0.174				*LRRC4*	TSS200
cg13233461	12	54,350,209	0.023056	0.241	0.229				*HOXC12*	Body
cg19657174	22	19,746,268	2.55E-07	0.241	0.230				*TBX1*	5'UTR
cg24955592	6	26,271,227	4.4E-05	0.241	0.248				*HIST1H3G*	1stExon
cg12198334	11	70,692,032	1.54E-05	0.241	0.210			Yes	*SHANK2*	Body
cg08969855	16	23,417,536	1.47E-30	0.241	0.240				*COG7*	Body
cg23004527	19	3,390,353	8.61E-08	0.241	0.232				*NFIC*	Body
cg18038207	12	80,581,062	0.000288	0.241	0.246					
cg26590744	2	177,017,389	3.22E-05	0.241	0.205				*HOXD4*	Body
cg02874569	6	5,851,436	0.01556	0.241	0.086					
cg12244647	22	50,737,978	5.25E-17	0.241	0.260				*PLXNB2*	5'UTR
cg16257051	5	153,862,183	0.027635	0.241	0.304					
cg10231675	12	2,944,493	2.14E-07	0.241	0.171				*NRIP2*	TSS1500
cg12593812	3	51,990,638	0.00267	0.241	0.242				*GPR62*	1stExon
cg18297736	4	85,416,865	0.015396	0.241	0.291				*NKX6-1*	Body
cg01315572	10	116,391,550	1.7E-07	0.241	0.246				*ABLIM1*	Body
cg04025964	1	200,010,788	0.010833	0.241	0.290				*NR5A2*	Body
cg21529533	6	29,796,015	0.010522	0.241	0.271	9.29E-13	0.203		*HLA-G*	Body
cg12856183	15	89,953,033	1.42E-05	0.241	0.217					
cg22431992	8	30,244,571	0.002423	0.241	0.214				*RBPMS*	Body
cg18797653	5	135,416,613	0.002607	0.241	0.237				*MIR886*	TSS1500

cg08347042	19	45,579,378	0.021848	0.241	0.271				*ZNF296*	1stExon
cg06549238	11	93,277,161	2.03E-05	0.241	0.185			Yes	*C11orf75*	TSS1500
cg12710899	14	57,261,702	0.015235	0.241	0.238					
cg03945800	16	4,165,515	2.62E-08	0.241	0.197	1.43E-23	0.219		*ADCY9*	5'UTR
cg01115857	14	73,695,018	1.41E-07	0.241	0.230					
cg12350474	17	46,578,476	2.24E-17	0.241	0.216					
cg15617155	1	63,785,321	0.012664	0.241	0.308					
cg15921876	3	133,393,656	0.000443	0.241	0.217					
cg00674004	10	101,280,750	4.09E-06	0.241	0.247					
cg04508701	6	137,813,878	8.02E-05	0.241	0.218				*OLIG3*	1stExon
cg25986200	2	131,010,423	1.43E-07	0.241	0.203					
cg14261272	2	127,976,400	0.010911	0.241	0.322					
cg00351472	9	97,846,551	2.54E-12	0.241	0.234				*MIR23B*	TSS1500
cg00636527	5	176,859,356	1.11E-08	0.241	0.225				*GRK6*	Body
cg20154618	19	4,153,677	0.005638	0.241	0.255				*CREB3L3*	1stExon
cg25409809	15	22,920,168	6.32E-10	0.241	0.212				*CYFIP1*	5'UTR
cg01288372	2	209,271,383	0.020236	0.241	0.228				*PTH2R*	TSS200
cg03957109	15	60,654,693	0.000124	0.241	0.218				*ANXA2*	Body
cg03801871	2	30,453,395	4.68E-06	0.241	0.231				*LBH*	TSS1500
cg09797337	13	112,709,538	0.001352	0.241	0.247					
cg10501360	10	49,671,665	1.11E-13	0.241	0.228				*ARHGAP22*	Body
cg14260169	10	72,344,881	1.84E-05	0.241	0.266					
cg20457051	6	47,668,044	1.76E-07	0.241	0.251				*GPR115*	5'UTR
cg11192416	1	231,296,968	0.002401	0.241	0.260					
cg15664152	7	129,008,407	2.32E-09	0.241	0.240				*AHCYL2*	Body
cg22328396	4	6,116,698	6.11E-06	0.241	0.219				*JAKMIP1*	5'UTR
cg03167763	1	20,512,397	2.21E-07	0.241	0.211	2.33E-09	0.114		*UBXN10*	TSS200
cg06570476	19	33,764,439	4.37E-10	0.241	0.228					
cg15833843	17	53,341,243	0.000188	0.241	0.224				*HLF*	TSS1500
cg23662097	3	4,873,008	3.67E-06	0.241	0.202				*ITPR1*	Body
cg12308279	11	8,731,033	1.15E-14	0.241	0.224				*ST5*	Body
cg22750254	10	13,043,421	0.025356	0.241	0.288	1.28E-19	0.245		*CCDC3*	1stExon
cg06245037	11	44,332,938	0.018835	0.241	0.279				*ALX4*	TSS1500
cg01452847	17	46,711,341	0.000655	0.241	0.167				*MIR196A1*	TSS1500

cg01981252	6	150,286,646	0.00642	0.241	0.216				*ULBP1*	Body
cg05782445	2	26,915,207	8.98E-05	0.241	0.170				*KCNK3*	TSS1500
cg20038493	14	24,809,226	2.52E-05	0.241	0.206				*RIPK3*	5'UTR
cg04922681	13	43,149,234	0.030579	0.241	0.316				*TNFSF11*	Body
cg06533244	6	27,258,460	0.032716	0.241	0.267					
cg12904904	5	155,754,151	0.001547	0.241	0.199				*SGCD*	1stExon
cg18454863	9	70,972,325	1.9E-07	0.241	0.242				*LOC572558*	Body
cg07271302	6	114,177,535	0.012241	0.241	0.190				*MARCKS*	TSS1500
cg18596947	14	94,393,816	4.39E-09	0.241	0.244				*FAM181A*	Body
cg14923652	18	12,277,384	0.000163	0.241	0.253				*CIDEA*	3'UTR
cg00528902	8	9,760,528	0.00053	0.241	0.226				*LOC157627*	Body
cg05609218	10	15,760,947	5.83E-05	0.241	0.229			Yes	*ITGA8*	Body
cg09313482	8	120,685,317	4.93E-06	0.241	0.279					
cg00398130	8	80,525,764	0.014117	0.241	0.201				*STMN2*	Body
cg05260469	8	62,621,327	3.91E-07	0.241	0.253				*ASPH*	Body
cg13952840	9	136,114,300	0.001268	0.241	0.232					
cg17301223	8	145,106,438	2.05E-05	0.241	0.259				*OPLAH*	Body
cg23424043	7	138,795,004	3.76E-08	0.241	0.185				*ZC3HAV1*	TSS1500
cg08867471	5	56,738,244	2.12E-17	0.241	0.210					
cg16634404	6	33,161,438	2.59E-19	0.241	0.226				*COL11A2*	TSS1500
cg21897034	3	38,567,652	2.22E-05	0.241	0.286				*EXOG*	3'UTR
cg23705098	7	1,289,853	4.24E-07	0.241	0.215					
cg24759821	12	103,311,462	0.006656	0.241	0.092	7.17E-13	0.175		*PAH*	TSS200
cg25911551	4	144,266,241	3.68E-10	0.241	0.258				*GAB1*	Body
cg00517532	6	3,232,025	0.036545	0.241	0.198					
cg12891160	2	119,916,686	0.00313	0.241	0.252				*C1QL2*	TSS1500
cg22374525	1	116,370,764	1.11E-08	0.241	0.225					
cg25718383	7	130,020,096	4.21E-06	0.241	0.273				*CPA1*	TSS200
cg16754364	16	29,677,969	0.018687	0.241	0.266				*SPN*	3'UTR
cg01402735	1	79,472,558	3.48E-06	0.241	0.213				*ELTD1*	TSS200
cg12110327	10	38,264,581	3.24E-07	0.241	0.193				*ZNF25*	5'UTR
cg15469272	1	91,189,314	0.009297	0.241	0.271					
cg21692450	1	46,940,325	1.49E-08	0.241	0.222					
cg26740494	1	1,567,131	1.71E-11	0.241	0.238				*MMP23A*	TSS1500

cg08605656	X	73,642,885	4.71E-22	0.241	0.234				*SLC16A2*	Body
cg18026631	8	23,584,746	0.001069	0.241	0.206					
cg16470716	6	28,956,719	9.09E-07	0.241	0.226					
cg22489510	2	8,675,599	1.31E-14	0.241	0.226					
cg18424072	20	39,656,880	0.002574	0.240	0.185				*TOP1*	TSS1500
cg26889659	6	684,090	1.22E-13	0.240	0.235				*EXOC2*	5'UTR
cg13031251	5	77,268,734	0.009309	0.240	0.221					
cg05370101	2	43,188,839	8.31E-06	0.240	0.263					
cg05758861	8	143,580,823	0.026165	0.240	0.362				*BAI1*	Body
cg22244192	6	33,943,837	1.15E-06	0.240	0.229					
cg05266321	3	46,401,927	1.19E-07	0.240	0.178				*CCR2*	3'UTR
cg07441518	11	31,846,849	4.47E-05	0.240	0.269					
cg15999311	14	65,749,247	2.42E-08	0.240	0.244					
cg20256245	22	29,877,285	0.000322	0.240	0.232				*NEFH*	Body
cg00133857	17	15,317,624	2.05E-20	0.240	0.216					
cg14651446	6	28,574,571	0.012284	0.240	0.288					
cg04922566	3	57,198,244	5.56E-10	0.240	0.222				*IL17RD*	Body
cg17387577	12	124,864,657	2.77E-16	0.240	0.274				*NCOR2*	Body
cg19238380	1	156,093,948	8.38E-12	0.240	0.282				*LMNA*	Body
cg25493589	3	42,307,193	2.13E-08	0.240	0.252				*CCK*	TSS1500
cg01821923	5	134,914,371	5.48E-05	0.240	0.220				*CXCL14*	Body
cg05778494	1	155,165,346	3.74E-05	0.240	0.141					
cg07007118	2	237,477,262	0.001127	0.240	0.133				*CXCR7*	TSS1500
cg10095777	6	31,734,292	2.29E-06	0.240	0.231				*C6orf27*	Body
cg11212451	5	173,198,508	0.004497	0.240	0.170					
cg11353598	15	70,740,451	7.18E-14	0.240	0.225					
cg11980129	3	79,815,639	0.000268	0.240	0.189				*ROBO1*	5'UTR
cg01724917	16	17,563,028	6.01E-14	0.240	0.238				*XYLT1*	Body
cg06902607	1	247,511,400	7.04E-08	0.240	0.191					
cg15321401	6	166,884,430	9.76E-14	0.240	0.208				*RPS6KA2*	Body
cg19597529	3	122,399,230	2.48E-06	0.240	0.166				*PARP14*	TSS1500
cg01843768	7	2,078,650	1.4E-16	0.240	0.201				*MAD1L1*	Body
cg17793776	5	77,140,381	2.57E-05	0.240	0.207					
cg00538458	16	22,959,868	0.005077	0.240	0.238					

cg19940644	1	12,112,386	0.002231	0.240	0.280					
cg22345878	12	6,234,313	4.55E-08	0.240	0.264				*VWF*	TSS1500
cg04924185	16	56,710,098	3.24E-05	0.240	0.231				*MT1IP*	Body
cg19865237	14	104,690,244	3.44E-17	0.240	0.244					
cg03543600	17	4,614,422	0.003503	0.240	0.099				*ARRB2*	Body
cg01494551	6	28,367,070	0.001164	0.240	0.234				*ZSCAN12*	Body
cg02392179	10	106,400,114	1.23E-05	0.240	0.256				*SORCS3*	TSS1500
cg07601320	12	47,219,841	0.000537	0.240	0.159	1.52E-20	0.197		*SLC38A4*	TSS200
cg20474425	12	54,746,067	1.88E-19	0.240	0.243					
cg16165748	11	65,030,448	0.000229	0.240	0.212				*POLA2*	Body
cg21521518	4	53,727,714	0.000194	0.240	0.152				*RASL11B*	TSS1500
cg04711852	14	37,126,283	0.001465	0.240	0.232			Yes	*PAX9*	TSS1500
cg05876917	17	35,014,676	0.002306	0.240	0.195					
cg16589830	1	212,284,214	0.000167	0.240	0.233					
cg01366595	5	42,952,307	9.49E-05	0.240	0.220					
cg14345676	5	175,109,098	0.003743	0.240	0.236				*HRH2*	5'UTR
cg14843868	1	53,791,688	0.003125	0.240	0.137				*LRP8*	Body
cg18103394	X	47,433,374	0.001347	0.240	0.289				*SYN1*	Body
cg27499185	19	6,138,355	2.19E-09	0.240	0.247				*ACSBG2*	5'UTR
cg00815214	21	47,717,953	7.72E-10	0.240	0.220					
cg20125091	1	92,952,641	1.38E-05	0.240	0.223	2.05E-20	0.152		*GFI1*	TSS1500
cg22047703	20	55,841,896	0.019377	0.240	0.121				*BMP7*	TSS200
cg00730441	17	59,483,863	1.6E-13	0.240	0.241				*TBX2*	Body
cg13322252	1	15,392,793	1.75E-11	0.240	0.243				*KIAA1026*	Body
cg14966782	2	109,812,089	3.96E-14	0.240	0.243				*SH3RF3*	Body
cg03983213	7	157,475,737	0.00013	0.240	0.211				*PTPRN2*	Body
cg09802066	4	183,063,534	9.03E-07	0.240	0.244				*MGC45800*	Body
cg11317690	4	6,347,280	5.02E-12	0.240	0.249				*PPP2R2C*	Body
cg12733607	10	21,813,501	1.22E-07	0.240	0.272				*C10orf140*	5'UTR
cg27138223	2	109,561,317	1.67E-14	0.240	0.202				*EDAR*	5'UTR
cg01353903	X	99,662,650	1.25E-06	0.240	0.227				*PCDH19*	1stExon
cg05238069	17	16,257,135	0.000224	0.240	0.153				*CENPV*	TSS1500
cg05650674	16	68,677,997	2.01E-14	0.240	0.215				*CDH3*	TSS200
cg09674468	1	234,300,299	3.61E-05	0.240	0.280				*SLC35F3*	Body

cg10393598	6	87,862,264	0.014717	0.240	0.184						
cg26467528	6	31,632,248	6.12E-07	0.240	0.254					*BAT4*	Body
cg20836412	8	82,543,285	0.000143	0.240	0.223						
cg07097722	20	43,247,325	2.66E-08	0.240	0.256					*PKIG*	3'UTR
cg25105745	1	110,141,275	1.44E-12	0.240	0.249					*MIR197*	TSS1500
cg25988710	X	72,667,379	0.000631	0.240	0.227					*CDX4*	1stExon
cg06321998	6	27,173,991	0.042281	0.240	0.282						
cg13277939	14	39,735,211	0.000495	0.240	0.277	1.2E-12	0.177			*CTAGE5*	TSS1500
cg15630458	8	142,986,649	0.001036	0.240	0.179						
cg19593949	1	205,312,444	1.85E-09	0.240	0.183					*KLHDC8A*	Body
cg17881600	6	17,012,979	0.005905	0.240	0.105						
cg19869746	6	7,107,108	0.005999	0.240	0.139					*RREB1*	TSS1500
cg08193322	2	74,730,586	0.032739	0.240	0.328					*LOC151534*	Body
cg03220124	17	54,756,137	4.76E-08	0.240	0.223						
cg22047977	17	62,962,070	0.000344	0.240	0.265						
cg04344565	8	140,715,646	0.008549	0.240	0.278					*KCNK9*	TSS1500
cg07160746	20	43,726,765	9.5E-08	0.240	0.292					*KCNS1*	Body
cg11895696	7	105,283,982	1.78E-12	0.240	0.227					*ATXN7L1*	Body
cg05949903	16	21,831,636	0.048008	0.240	0.088					*RRN3P1*	TSS1500
cg24886267	5	167,956,306	0.041162	0.240	0.164					*FBLL1*	TSS1500
cg01636582	11	844,593	7.5E-12	0.240	0.215					*TSPAN4*	5'UTR
cg13071386	3	37,494,403	3.4E-16	0.240	0.219					*ITGA9*	Body
cg06202276	4	154,713,333	0.011319	0.240	0.248						
cg18268547	17	79,615,552	0.001861	0.240	0.249					*TSPAN10*	3'UTR
cg27426835	2	19,963,298	8.22E-07	0.240	0.213						
cg27060391	19	42,416,708	0.001996	0.240	0.319						
cg07537370	2	27,531,124	2.7E-07	0.240	0.213					*UCN*	5'UTR
cg15107368	15	68,260,709	0.01065	0.240	0.056						
cg17786642	1	54,203,678	0.000878	0.240	0.184						
cg10671510	11	15,494,161	1.43E-05	0.240	0.266						
cg03157027	8	99,959,723	2.27E-05	0.240	0.265					*OSR2*	5'UTR
cg05360714	17	75,543,046	2.54E-09	0.240	0.238						
cg09028204	20	43,729,808	5.1E-07	0.240	0.226					*KCNS1*	TSS200
cg14606431	2	71,126,505	1.8E-06	0.240	0.226					*VAX2*	TSS1500

cg14719959	8	61,777,711	1.39E-07	0.240	0.230				*CHD7*	Body
cg18866599	2	208,635,723	2.83E-05	0.240	0.242					
cg24514391	1	20,518,904	1.5E-12	0.240	0.238				*UBXN10*	3'UTR
cg24673771	2	45,180,267	0.000645	0.240	0.218					
cg10128164	14	31,344,535	0.007949	0.240	0.307	8.47E-08	0.145		*COCH*	Body
cg08812555	10	54,074,788	0.029034	0.240	0.138				*DKK1*	Body
cg06346335	1	76,489,892	2.33E-22	0.240	0.207					
cg10862567	17	80,745,169	7.29E-08	0.240	0.241				*TBCD*	Body
cg14485633	9	136,783,440	9.61E-18	0.240	0.243				*VAV2*	Body
cg21356535	6	137,311,287	0.046207	0.240	0.127				*NHEG1*	Body
cg01435109	1	23,496,648	5.6E-11	0.240	0.224				*LUZP1*	TSS1500
cg20050484	3	123,339,334	3.01E-11	0.240	0.229				*MYLK*	1stExon
cg00044665	16	66,400,411	4.33E-07	0.240	0.187				*CDH5*	TSS200
cg06153925	17	78,755,379	1.99E-44	0.240	0.236				*RPTOR*	Body
cg14038058	4	165,898,848	2.91E-08	0.240	0.232				*TRIM61*	TSS200
cg21481275	13	100,639,669	0.002179	0.240	0.277					
cg22367310	2	241,856,114	9.1E-05	0.240	0.231					
cg00088797	3	12,976,288	3.25E-06	0.240	0.227				*IQSEC1*	Body
cg00339913	8	22,085,227	6.72E-06	0.240	0.203				*PHYHIP*	Body
cg08622757	7	156,799,393	4.12E-05	0.240	0.223				*MNX1*	Body
cg19433697	15	60,294,787	0.005469	0.240	0.283					
cg14956197	14	36,993,831	0.009384	0.240	0.355					
cg15198000	4	75,230,839	0.010523	0.240	0.271				*EREG*	TSS200
cg05276469	17	16,570,473	0.004052	0.240	0.232					
cg08180070	2	109,647,958	0.000252	0.240	0.201					
cg10505580	1	32,238,660	3.42E-08	0.240	0.247					
cg23726526	9	96,711,316	0.034648	0.240	0.334					
cg20784768	1	234,041,456	7.71E-06	0.240	0.215				*SLC35F3*	Body
cg11283429	13	28,549,574	6.7E-05	0.240	0.246					
cg00998451	13	100,608,123	5.05E-05	0.240	0.227					
cg06902071	5	134,879,326	0.000236	0.240	0.243					
cg07913197	2	200,334,901	0.000962	0.240	0.263				*FLJ32063*	Body
cg12885751	13	100,085,073	1.45E-05	0.240	0.215					
cg16318349	1	154,917,307	7.51E-18	0.240	0.237				*PBXIP1*	3'UTR

cg02366262	11	121,297,716	8.87E-07	0.240	0.232					
cg01979888	12	6,665,424	9.99E-05	0.240	0.218				*IFFO1*	TSS200
cg04935109	3	187,086,530	0.000274	0.240	0.149				*RTP4*	Body
cg07301795	5	157,001,698	0.000448	0.240	0.262				*ADAM19*	Body
cg26007189	5	176,166,980	1.87E-06	0.240	0.243					
cg03322338	11	1,403,751	0.000357	0.240	0.309					
cg04945457	16	56,894,587	7.93E-07	0.240	0.261					
cg07540652	8	81,805,956	3.01E-06	0.240	0.195					
cg21160842	11	32,112,091	2.55E-07	0.240	0.219				*RCN1*	TSS1500
cg04657325	4	185,435,601	0.000745	0.240	0.216					
cg19386336	X	100,546,064	0.001255	0.240	0.197				*TAF7L*	Body
cg21618017	12	124,016,861	0.000121	0.240	0.217				*RILPL1*	Body
cg13426351	9	132,956,279	3.34E-12	0.240	0.229				*FREQ*	Body
cg00162231	3	11,035,357	0.022689	0.240	0.261				*SLC6A1*	5'UTR
cg20954971	21	44,593,642	0.005743	0.240	0.265					
cg23205629	8	33,421,410	1.05E-14	0.240	0.231				*RNF122*	Body
cg10060574	6	30,712,338	0.002096	0.240	0.221				*IER3*	TSS200
cg08509172	19	19,383,838	0.006462	0.240	0.127				*TM6SF2*	Body
cg02994349	1	66,999,753	0.004167	0.240	0.212				*SGIP1*	TSS200
cg03731303	3	181,443,009	2.51E-06	0.240	0.220				*SOX2OT*	Body
cg05191175	5	134,827,218	0.000851	0.240	0.167					
cg18956933	3	147,136,904	0.009926	0.240	0.281					
cg08791347	10	13,831,250	7.78E-07	0.240	0.257				*FRMD4A*	Body
cg12798118	17	15,166,031	0.033336	0.240	0.161				*PMP22*	TSS200
cg02180498	6	31,830,572	0.044954	0.240	0.229				*NEU1*	5'UTR
cg02278646	12	45,608,691	9.54E-13	0.240	0.230				*ANO6*	TSS1500
cg17910813	19	47,923,387	4.67E-05	0.240	0.266				*MEIS3*	TSS1500
cg19717773	7	2,847,554	0.004046	0.240	0.140				*GNA12*	Body
cg01413582	6	30,652,688	8.03E-05	0.240	0.232				*KIAA1949*	Body
cg04207385	6	88,877,064	0.000139	0.240	0.266				*CNR1*	TSS1500
cg06606386	19	13,947,469	2.32E-08	0.240	0.236				*MIR27A*	TSS200
cg11731221	6	30,418,508	1.97E-05	0.240	0.272					
cg18406255	2	144,995,067	0.000954	0.240	0.235				*GTDC1*	5'UTR
cg15355388	11	57,004,919	8.27E-06	0.240	0.273				*APLNR*	Body

cg08569678	8	143,781,397	0.004866	0.240	0.212				*LY6K*	TSS200
cg14349667	4	81,189,706	0.001416	0.240	0.249				*FGF5*	Body
cg24699719	5	74,913,041	5.96E-08	0.240	0.232					
cg03032512	17	77,030,418	1.35E-08	0.240	0.193				*C1QTNF1*	TSS200
cg09088836	6	2,510,144	2.64E-12	0.240	0.227					
cg15559674	6	149,803,426	9.91E-07	0.240	0.237				*ZC3H12D*	5'UTR
cg19938199	15	63,233,164	5.66E-07	0.240	0.230					
cg24162251	14	32,420,205	2.28E-05	0.240	0.214					
cg05363616	1	119,529,219	1.44E-07	0.240	0.266				*TBX15*	5'UTR
cg25662822	3	116,755,155	1.1E-13	0.240	0.243					
cg05341539	15	86,233,214	0.011056	0.240	0.233				*AKAP13*	Body
cg01438365	17	59,480,470	4.47E-06	0.240	0.226				*TBX2*	Body
cg02928928	11	1,975,304	1.28E-11	0.239	0.220				*MRPL23*	Body
cg14326472	9	126,164,083	8.58E-20	0.239	0.227				*DENND1A*	3'UTR
cg14952457	6	50,818,685	8.65E-05	0.239	0.247					
cg11039476	2	127,978,579	1.83E-06	0.239	0.230					
cg06390651	2	177,023,667	0.000565	0.239	0.188					
cg06857289	4	13,524,306	0.040873	0.239	0.335					
cg13831540	7	130,128,420	3.87E-21	0.239	0.230				*MESTIT1*	Body
cg27128761	5	151,055,650	8.71E-12	0.239	0.224				*SPARC*	Body
cg09462924	2	66,666,470	0.042393	0.239	0.328				*MEIS1*	Body
cg03114255	6	150,358,985	0.044616	0.239	0.168					
cg05222794	20	60,540,550	5.08E-06	0.239	0.235					
cg10603592	17	40,896,097	3.05E-12	0.239	0.227				*EZH1*	5'UTR
cg10397932	1	2,166,155	0.000171	0.239	0.325				*SKI*	Body
cg24312680	4	128,704,518	0.008852	0.239	0.248				*HSPA4L*	Body
cg27584013	1	23,012,439	1.13E-10	0.239	0.264					
cg10753889	11	116,706,520	0.028706	0.239	0.184				*APOA1*	3'UTR
cg20523861	13	70,682,324	0.001546	0.239	0.199	5.64E-22	0.230		*KLHL1*	1stExon
cg26104293	6	91,499,812	5.06E-12	0.239	0.217					
cg10368049	7	94,023,796	0.002127	0.239	0.190				*COL1A2*	TSS200
cg19960891	11	63,776,653	0.015478	0.239	0.116				*MACROD1*	Body
cg15129679	1	85,513,315	1.37E-06	0.239	0.211				*MCOLN3*	5'UTR
cg22629111	1	166,135,208	3.79E-06	0.239	0.264				*FAM78B*	Body

cg16264705	12	54,071,194	0.024254	0.239	0.216				*ATP5G2*	TSS1500
cg18168249	7	134,946,296	0.000374	0.239	0.253					
cg20137746	10	27,235,598	4.92E-06	0.239	0.258					
cg00754253	11	63,259,187	1.51E-09	0.239	0.249				*HRASLS5*	TSS1500
cg06312072	12	99,287,129	1.29E-05	0.239	0.181				*ANKS1B*	Body
cg15443952	7	100,817,963	0.000979	0.239	0.323				*C7orf52*	Body
cg12757155	18	5,628,325	0.011911	0.239	0.284					
cg14592399	4	41,881,933	3.07E-06	0.239	0.205					
cg23590187	7	29,686,098	2.2E-05	0.239	0.260				*LOC646762*	Body
cg14582226	7	116,164,743	0.00986	0.239	0.170				*CAV1*	TSS200
cg18224993	11	78,521,705	2.92E-15	0.239	0.241				*ODZ4*	Body
cg18661868	15	91,427,965	0.001637	0.239	0.169	2.39E-08	0.081		*FES*	5'UTR
cg19723734	7	2,271,673	2.33E-06	0.239	0.252				*MAD1L1*	5'UTR
cg06772268	5	81,342,312	4.22E-19	0.239	0.211				*ATG10*	Body
cg13348944	11	118,047,121	0.00052	0.239	0.256	4.03E-17	0.149		*SCN2B*	1stExon
cg22900415	13	20,736,075	7.24E-05	0.239	0.210				*GJA3*	TSS1500
cg02915422	X	150,863,331	0.001071	0.239	0.262					
cg02508830	7	102,576,358	6.29E-07	0.239	0.252				*FBXL13*	Body
cg06570025	21	34,444,245	0.032183	0.239	0.241				*OLIG1*	1stExon
cg11883737	3	129,276,179	6.73E-31	0.239	0.246				*PLXND1*	Body
cg20040765	8	67,358,546	1.08E-06	0.239	0.259				*ADHFE1*	Body
cg23112192	17	27,893,542	6.57E-07	0.239	0.216				*ABHD15*	1stExon
cg07653695	2	102,175,888	7.63E-05	0.239	0.226					
cg21932416	2	176,997,707	2.18E-08	0.239	0.216					
cg24227728	19	11,590,393	0.006414	0.239	0.258				*ELAVL3*	Body
cg03314569	1	32,041,732	1.52E-11	0.239	0.247				*TINAGL1*	TSS1500
cg05147273	17	74,696,804	2.32E-12	0.239	0.230				*MXRA7*	Body
cg10543035	1	179,545,790	2.46E-06	0.239	0.190				*NPHS2*	TSS1500
cg06017559	16	1,026,760	1.27E-11	0.239	0.221					
cg26449787	6	133,562,087	1.96E-05	0.239	0.304				*EYA4*	TSS1500
cg04537567	6	99,296,068	3.34E-05	0.239	0.197					
cg13391244	7	120,629,638	5.02E-10	0.239	0.214				*C7orf58*	5'UTR
cg27405988	3	48,171,915	3.54E-06	0.239	0.253					
cg03059247	8	11,287,657	1.71E-10	0.239	0.228				*C8orf12*	Body

cg11911305	19	45,428,924	1.62E-11	0.239	0.242				*APOC1P1*	TSS1500
cg16050897	17	71,361,894	5.13E-07	0.239	0.240				*SDK2*	Body
cg20531550	7	128,550,902	3.13E-16	0.239	0.234				*KCP*	TSS200
cg03190219	7	25,898,688	1.09E-09	0.239	0.220					
cg19839026	14	51,410,501	0.001561	0.239	0.244				*PYGL*	Body
cg20930815	11	9,099,393	1.04E-11	0.239	0.214				*SCUBE2*	Body
cg09364590	6	155,537,901	3.42E-13	0.239	0.210				*TIAM2*	Body
cg11242303	1	67,773,725	0.02149	0.239	0.084				*IL12RB2*	5'UTR
cg16987638	6	1,601,509	0.000877	0.239	0.155					
cg17207266	2	25,562,957	1.75E-09	0.239	0.201				*DNMT3A*	5'UTR
cg00204465	17	73,583,945	3.25E-05	0.239	0.242				*MYO15B*	TSS200
cg27627006	11	96,072,706	5.72E-06	0.239	0.222				*MAML2*	Body
cg07328888	13	33,002,693	0.009599	0.239	0.167				*N4BP2L1*	TSS1500
cg27452217	1	116,371,037	2.01E-05	0.239	0.232					
cg02569115	17	76,922,138	1.59E-06	0.239	0.251				*TIMP2*	TSS1500
cg13500072	1	36,788,387	3.55E-06	0.239	0.225				*FAM176B*	Body
cg01466330	8	39,835,697	4.93E-11	0.239	0.174				*IDO2*	Body
cg03784793	3	116,058,982	0.004342	0.239	0.165				*LSAMP*	Body
cg13152535	19	51,226,557	0.001748	0.239	0.328	0.0000137	0.083		*CLEC11A*	TSS200
cg24815529	19	55,592,342	4.95E-05	0.239	0.213				*EPS8L1*	Body
cg01858698	1	46,954,407	5.11E-07	0.239	0.225					
cg07546334	3	152,557,119	4.3E-07	0.239	0.222					
cg15109744	1	180,917,873	4.57E-15	0.239	0.228					
cg25978487	6	32,157,150	0.000104	0.239	0.204				*PBX2*	Body
cg27428520	1	204,100,192	5.26E-12	0.239	0.224				*ETNK2*	3'UTR
cg02864619	17	78,933,961	1.17E-84	0.239	0.235				*RPTOR*	Body
cg01194371	5	79,331,477	0.000597	0.239	0.236				*THBS4*	Body
cg12405136	11	68,781,357	2.77E-05	0.239	0.203				*MRGPRF*	TSS1500
cg15624624	10	114,819,357	3.52E-05	0.239	0.200				*TCF7L2*	Body
cg04084892	2	17,722,836	0.00134	0.239	0.245				*VSNL1*	5'UTR
cg18407955	7	158,110,685	1.45E-06	0.239	0.224				*PTPRN2*	Body
cg12061236	6	151,560,886	8.47E-06	0.239	0.229	2.64E-08	0.098		*AKAP12*	TSS1500
cg22796922	7	48,031,800	0.010185	0.239	0.226				*SUNC1*	Body
cg06808089	3	45,708,680	3.5E-17	0.239	0.236				*LIMD1*	Body

cg26632948	13	25,711,193	3.09E-05	0.239	0.194					
cg04944806	6	27,798,932	0.012083	0.239	0.184					
cg03548415	11	111,991,342	2.43E-08	0.239	0.251					
cg16908257	12	54,404,893	0.000326	0.239	0.176				*HOXC8*	Body
cg17386213	6	108,488,335	0.002431	0.239	0.256				*NR2E1*	Body
cg26899651	17	75,441,392	2.48E-09	0.239	0.223				*SEPT9*	Body
cg01876548	5	124,126,863	1.22E-09	0.239	0.247					
cg02333730	17	12,199,913	9.39E-09	0.239	0.204					
cg00409356	5	1,879,525	3.05E-05	0.239	0.230				*IRX4*	Body
cg04279973	16	23,846,968	0.001397	0.239	0.196				*PRKCB*	TSS1500
cg12836280	5	50,260,240	0.000472	0.239	0.175					
cg17611193	19	35,395,942	0.007397	0.239	0.203					
cg17862558	4	106,137,048	7.98E-08	0.239	0.236				*TET2*	5'UTR
cg19501909	22	38,001,083	1.96E-05	0.239	0.228					
cg25544075	1	1,109,315	0.00025	0.239	0.233				*TTLL10*	5'UTR
cg26472684	6	14,763,072	7.41E-20	0.239	0.224					
cg18642177	1	228,195,779	0.000159	0.239	0.250				*WNT3A*	Body
cg09722609	5	149,887,422	2.29E-16	0.239	0.230				*NDST1*	TSS1500
cg12942552	5	57,749,839	4.9E-09	0.239	0.257				*PLK2*	3'UTR
cg24777399	2	109,855,574	1.87E-10	0.239	0.247				*SH3RF3*	Body
cg04948014	12	120,241,494	2.77E-11	0.239	0.220				*CIT*	Body
cg12111783	X	40,027,674	1.71E-25	0.239	0.216				*BCOR*	5'UTR
cg12340454	15	61,519,486	0.000237	0.239	0.169				*RORA*	Body
cg13128937	13	95,357,743	0.00163	0.239	0.252					
cg22891868	2	223,536,069	2.26E-15	0.239	0.208				*MOGAT1*	TSS1500
cg25843866	6	168,079,818	3.03E-05	0.239	0.230					
cg21209876	14	23,447,792	6.39E-08	0.239	0.253				*JUB*	Body
cg15482931	15	27,019,448	9.93E-05	0.239	0.194				*GABRB3*	TSS1500
cg24690479	6	10,521,142	4.44E-08	0.239	0.203				*GCNT2*	TSS1500
cg13351583	9	91,793,648	2.12E-05	0.239	0.238	1.46E-08	0.079		*SHC3*	1stExon
cg07714276	6	7,169,632	7.67E-12	0.239	0.219				*RREB1*	5'UTR
cg09788586	12	114,337,676	3.64E-07	0.239	0.223				*RBM19*	Body
cg11590714	X	49,832,148	1.59E-13	0.239	0.246				*CLCN5*	Body
cg13480738	2	46,110,087	4.9E-11	0.239	0.241				*PRKCE*	Body

cg15514496	18	12,287,561	1.5E-08	0.239	0.208					
cg25103337	1	9,293,583	1.81E-08	0.239	0.237				*H6PD*	TSS1500
cg11832210	10	91,295,346	0.048771	0.239	0.304				*SLC16A12*	TSS200
cg14458619	9	8,858,644	0.001817	0.239	0.188				*PTPRD*	5'UTR
cg25964728	3	136,539,328	0.001859	0.239	0.157				*TMEM22*	5'UTR
cg12974388	2	237,072,998	0.045401	0.239	0.260					
cg01723876	1	21,978,856	2.29E-10	0.239	0.224				*RAP1GAP*	5'UTR
cg16366546	10	24,672,795	9.97E-07	0.239	0.213				*KIAA1217*	Body
cg05576992	8	969,029	1.09E-07	0.239	0.196					
cg12988424	3	62,355,884	0.021949	0.239	0.286				*FEZF2*	Body
cg19279973	3	15,563,149	0.000136	0.239	0.252				*COLQ*	1stExon
cg21166964	5	72,529,816	6.39E-07	0.239	0.175					
cg00157016	2	24,344,064	1.51E-12	0.239	0.253				*PFN4*	Body
cg06832449	10	35,069,982	5.48E-06	0.239	0.228				*PARD3*	Body
cg09238180	2	176,993,632	0.000482	0.239	0.254				*HOXD8*	TSS1500
cg15009198	2	97,429,502	7.75E-13	0.239	0.211				*CNNM4*	Body
cg15136975	1	19,781,337	2.36E-06	0.239	0.266				*CAPZB*	Body
cg23205387	7	152,623,043	0.00491	0.239	0.264					
cg01210589	14	89,895,742	0.000161	0.239	0.180				*FOXN3*	5'UTR
cg05005332	9	98,979,142	7.74E-14	0.239	0.251					
cg23115907	6	137,817,260	0.000988	0.239	0.250					
cg00304185	16	1,033,074	9.49E-05	0.239	0.223				*SOX8*	Body
cg01104119	2	131,186,487	7.13E-15	0.239	0.223					
cg07155013	6	140,030,345	2.93E-20	0.239	0.233					
cg05467716	17	62,774,079	2.71E-15	0.239	0.235				*LOC146880*	Body
cg23647784	10	24,386,611	1.38E-11	0.239	0.237				*KIAA1217*	5'UTR
cg05142765	14	95,234,822	3.84E-05	0.239	0.241				*GSC*	3'UTR
cg04503600	5	140,798,188	0.000733	0.239	0.258				*PCDHGA4*	Body
cg00562180	16	89,638,519	6.54E-10	0.239	0.236					
cg20023898	16	67,048,469	5.57E-05	0.239	0.205					
cg01305421	12	102,874,286	2.84E-05	0.239	0.240	1.8E-10	0.121		*IGF1*	5'UTR
cg02237629	14	57,264,095	0.000499	0.239	0.245					
cg06625244	19	54,175,187	4.23E-06	0.239	0.260				*MIR1323*	TSS200
cg08679238	3	46,449,100	1.29E-06	0.239	0.232				*CCRL2*	1stExon

cg22959260	15	40,382,862	1.31E-25	0.239	0.229				*BMF*	3'UTR
cg11704005	1	19,002,948	5.05E-08	0.238	0.245				*PAX7*	Body
cg23732781	6	29,595,016	1.38E-06	0.238	0.229				*GABBR1*	Body
cg23978504	3	181,413,373	0.007982	0.238	0.278				*SOX2OT*	Body
cg14336737	10	74,079,845	0.01389	0.238	0.153					
cg16002355	4	111,544,387	0.046916	0.238	0.203				*PITX2*	Body
cg08463758	20	39,995,649	2.22E-05	0.238	0.202				*EMILIN3*	TSS200
cg22165175	1	111,148,915	6.61E-06	0.238	0.197	8.18E-17	0.176		*KCNA2*	TSS1500
cg25775494	6	108,490,895	1.14E-07	0.238	0.243				*NR2E1*	Body
cg04835613	22	39,410,292	2.37E-06	0.238	0.215				*APOBEC3C*	1stExon
cg05401364	3	112,967,129	1.44E-07	0.238	0.230				*BOC*	5'UTR
cg04237918	22	38,435,259	2.38E-05	0.238	0.267					
cg14509153	9	127,274,614	3.3E-08	0.238	0.228					
cg25379036	6	125,544,500	8.57E-13	0.238	0.225				*TPD52L1*	Body
cg26327804	8	140,714,489	3.95E-05	0.238	0.205				*KCNK9*	Body
cg03317811	2	42,329,494	5.64E-05	0.238	0.235					
cg02361903	1	110,452,616	4.53E-10	0.238	0.235				*CSF1*	TSS1500
cg04158367	1	92,952,440	0.001957	0.238	0.217				*GFI1*	TSS1500
cg26725502	11	27,034,225	9.16E-11	0.238	0.222					
cg04629204	1	26,348,325	8.12E-12	0.238	0.212	1.59E-26	0.133		*EXTL1*	1stExon
cg07703690	11	27,062,963	6.27E-25	0.238	0.221				*BBOX1*	5'UTR
cg26486663	16	2,040,863	0.005772	0.238	0.282	0.0000172	0.085		*SYNGR3*	Body
cg09169779	13	20,751,710	9.17E-14	0.238	0.211					
cg02054667	14	54,687,314	0.009124	0.238	0.228					
cg13488013	17	47,592,680	4.71E-05	0.238	0.249					
cg13835168	6	29,648,756	0.003761	0.238	0.174					
cg15237857	2	45,511	4.69E-05	0.238	0.131				*FAM110C*	1stExon
cg23198707	3	112,023,284	1.32E-06	0.238	0.229					
cg25821781	17	2,325,143	5.3E-15	0.238	0.226				*METT10D*	Body
cg10375078	10	22,624,374	0.01226	0.238	0.305					
cg20191453	3	49,459,855	1.54E-08	0.238	0.243	1.88E-11	0.178		*AMT*	Body
cg08084860	2	237,080,340	0.00109	0.238	0.213					
cg08530414	11	35,160,400	0.00486	0.238	0.317	2.77E-20	0.226		*CD44*	TSS200
cg10595547	10	119,310,911	0.000476	0.238	0.271					

cg11851174	17	17,712,609	1.46E-30	0.238	0.238				*RAI1*	Body
cg18823647	4	99,580,449	6.37E-05	0.238	0.216			Yes	*TSPAN5*	TSS1500
cg25500050	12	8,171,633	0.005536	0.238	0.262					
cg02245998	2	100,210,626	4.57E-10	0.238	0.196				*AFF3*	Body
cg11194545	X	105,066,793	0.035719	0.238	0.323				*NRK*	5'UTR
cg08251815	2	85,106,968	0.000139	0.238	0.280				*C2orf89*	Body
cg10215414	16	67,361,708	5.44E-15	0.238	0.231				*KCTD19*	TSS1500
cg22399984	6	1,601,802	2.54E-07	0.238	0.194					
cg17755907	5	141,293,917	0.00916	0.238	0.142					
cg05784193	1	192,847,589	1.08E-08	0.238	0.270					
cg12858902	1	110,254,854	4.67E-10	0.238	0.217				*GSTM5*	TSS200
cg03200052	1	223,538,627	0.001111	0.238	0.163				*SUSD4*	TSS1500
cg09169874	5	603,562	2.2E-16	0.238	0.208					
cg16675758	11	115,531,171	1.05E-07	0.238	0.203					
cg21067652	13	41,548,815	0.000248	0.238	0.258				*ELF1*	Body
cg22025232	6	29,894,874	0.020641	0.238	0.211				*HCG4P6*	TSS1500
cg14671384	12	47,219,920	7.57E-08	0.238	0.222				*SLC38A4*	TSS200
cg08718490	4	52,943,099	0.000655	0.238	0.243				*SPATA18*	Body
cg03983223	2	175,499,283	0.020781	0.238	0.205				*WIPF1*	1stExon
cg06298346	5	10,565,679	5.35E-08	0.238	0.235				*ANKRD33B*	Body
cg09599130	10	104,170,691	0.008281	0.238	0.242				*PSD*	Body
cg14072027	17	15,301,904	1.74E-06	0.238	0.225					
cg15205636	13	21,520,468	3.6E-05	0.238	0.229					
cg05861567	22	50,523,686	7.51E-11	0.238	0.226	0.00108251	0.099		*MLC1*	5'UTR
cg08279008	2	86,263,224	0.038526	0.238	0.360				*POLR1A*	Body
cg12860635	6	30,095,140	1.81E-15	0.238	0.225					
cg24945701	3	62,362,783	7.23E-05	0.238	0.264					
cg18798922	2	171,572,863	0.03362	0.238	0.254				*SP5*	Body
cg06170683	16	3,740,774	0.000181	0.238	0.224				*TRAP1*	Body
cg16038120	14	29,235,196	0.037051	0.238	0.162				*FOXG1*	TSS1500
cg06407657	20	43,937,138	2.46E-10	0.238	0.233				*MATN4*	TSS200
cg13528344	13	20,768,138	0.01111	0.238	0.272				*GJB2*	TSS1500
cg00050402	7	55,073,022	1.41E-08	0.238	0.209					
cg06362543	20	42,135,794	1.94E-06	0.238	0.201					

cg08195512	17	71,948,430	0.002055	0.238	0.265					
cg10069155	15	41,852,638	3E-05	0.238	0.218			Yes	*TYRO3*	Body
cg04487296	7	149,389,669	0.007114	0.238	0.271					
cg11851804	1	848,379	2.02E-05	0.238	0.246					
cg27210464	10	101,283,479	0.049965	0.238	0.236					
cg07219769	22	38,071,455	0.00019	0.238	0.292				*LGALS1*	TSS200
cg08975803	12	52,626,818	1.07E-06	0.238	0.218				*KRT7*	TSS200
cg13033858	12	109,248,326	5.79E-06	0.238	0.231				*SSH1*	Body
cg01388757	2	102,091,195	0.002202	0.238	0.238				*RFX8*	TSS200
cg09557313	13	53,313,174	0.007762	0.238	0.164				*LECT1*	Body
cg16615357	5	135,416,594	0.00037	0.238	0.230				*MIR886*	TSS1500
cg07719492	8	70,983,348	0.019104	0.238	0.252				*PRDM14*	5'UTR
cg26514793	10	73,769,127	2.34E-08	0.238	0.237				*CHST3*	3'UTR
cg26668713	11	65,405,903	0.02401	0.238	0.169	2.41E-08	0.124		*SIPA1*	5'UTR
cg00524220	19	3,098,963	4.59E-05	0.238	0.263				*GNA11*	Body
cg01246617	5	53,751,493	7.59E-30	0.238	0.242				*HSPB3*	5'UTR
cg18291437	X	119,445,843	3.49E-08	0.238	0.245				*FAM70A*	TSS1500
cg19785066	20	44,746,655	4.75E-05	0.238	0.297				*CD40*	TSS1500
cg24392548	3	46,854,173	0.018629	0.238	0.227					
cg04567307	4	48,492,122	0.000762	0.238	0.201				*ZAR1*	TSS200
cg05637536	1	154,475,068	0.000488	0.238	0.127				*TDRD10*	5'UTR
cg10779492	1	106,623,781	0.002665	0.238	0.226					
cg15927699	1	37,856,881	3.76E-09	0.238	0.228					
cg07188513	7	156,807,474	8.98E-06	0.238	0.225					
cg12598235	15	89,921,182	4.32E-05	0.238	0.206				*LOC254559*	TSS200
cg25659902	6	30,652,202	7.28E-06	0.238	0.242				*KIAA1949*	Body
cg00302983	13	24,007,545	0.003728	0.238	0.162				*SACS*	5'UTR
cg22976313	14	105,070,340	3.6E-07	0.238	0.262				*TMEM179*	Body
cg04205034	5	134,824,947	0.009892	0.238	0.199					
cg09169117	2	153,302,353	2.13E-06	0.238	0.240				*FMNL2*	Body
cg11775488	10	95,586,237	0.001554	0.238	0.254					
cg20438306	4	41,747,092	0.000151	0.238	0.238				*PHOX2B*	3'UTR
cg14983586	6	27,206,503	0.001728	0.238	0.256					
cg01077100	10	124,067,280	6.8E-10	0.238	0.223				*BTBD16*	Body

cg03155027	3	49,907,692	4.16E-07	0.238	0.203				*CAMKV*	TSS1500
cg05184394	2	131,512,773	1.06E-12	0.238	0.249	3.3E-33	0.210		*FAM123C*	TSS1500
cg09045681	6	31,127,178	2.39E-05	0.238	0.245	1.44E-08	0.131		*TCF19*	5'UTR
cg14265068	17	77,763,497	2.59E-12	0.238	0.197					
cg19656282	2	74,742,786	0.033608	0.238	0.274				*TLX2*	Body
cg21880903	4	39,408,665	3.52E-05	0.238	0.278	3.48E-10	0.168		*KLB*	1stExon
cg12166610	8	31,498,256	0.000192	0.238	0.264				*NRG1*	Body
cg18372607	11	31,827,084	0.009517	0.238	0.267				*PAX6*	Body
cg18497508	6	41,528,345	1.94E-06	0.238	0.222				*FOXP4*	5'UTR
cg19054158	7	1,099,421	0.007229	0.238	0.257				*C7orf50*	Body
cg10298741	16	73,019,173	4.52E-05	0.238	0.224				*ZFHX3*	5'UTR
cg14538320	8	22,722,360	0.000263	0.238	0.145				*PEBP4*	Body
cg01493517	12	6,665,447	4.29E-05	0.238	0.226				*IFFO1*	TSS1500
cg15287594	20	42,938,795	5.12E-11	0.238	0.264				*FITM2*	Body
cg21394729	15	31,316,860	1.09E-13	0.238	0.244				*TRPM1*	Body
cg21831174	3	187,011,187	3.51E-12	0.238	0.224	0.00091119	0.076		*MASP1*	TSS1500
cg03920442	20	25,848,969	0.002445	0.238	0.195					
cg04633600	5	101,632,286	0.011854	0.238	0.197				*SLCO4C1*	TSS200
cg00767010	9	99,482,094	0.011911	0.238	0.235					
cg05940231	1	119,532,189	1.01E-05	0.238	0.257				*TBX15*	TSS200
cg11765312	2	84,714,510	9.31E-05	0.238	0.173					
cg06657529	5	783,288	0.000975	0.238	0.268					
cg09905511	2	77,621,916	1.38E-06	0.238	0.233				*LRRTM4*	Body
cg13577076	7	752,456	0.002788	0.238	0.167	1.75E-12	0.231		*PRKAR1B*	5'UTR
cg22902089	11	46,318,223	3.34E-21	0.238	0.225				*CREB3L1*	Body
cg26541780	1	156,217,981	5.88E-07	0.238	0.287				*PAQR6*	TSS200
cg05016408	5	150,326,174	0.049784	0.238	0.265				*LOC134466*	TSS200
cg12598865	11	2,901,843	5.76E-10	0.238	0.233					
cg13537353	13	26,586,651	0.000191	0.238	0.196				*ATP8A2*	Body
cg10966139	1	236,228,837	0.000842	0.238	0.195				*NID1*	TSS1500
cg23947326	19	46,504,128	0.033529	0.238	0.152				*CCDC61*	Body
cg24924958	6	142,409,993	0.01398	0.238	0.181				*NMBR*	TSS200
cg02634272	16	71,459,870	0.01848	0.238	0.231					
cg03340026	19	1,403,530	0.000345	0.238	0.268					

cg04607865	7	20,834,362	0.001373	0.238	0.195					
cg04628438	6	10,585,889	3.79E-08	0.238	0.228				*GCNT2*	Body
cg07820868	1	119,528,638	0.006615	0.238	0.253				*TBX15*	5'UTR
cg22202689	19	49,298,227	7.71E-10	0.238	0.267					
cg19906672	4	6,918,868	0.000676	0.238	0.188				*TBC1D14*	5'UTR
cg22967542	7	96,647,483	0.000231	0.238	0.262					
cg25034155	10	80,945,359	1.18E-05	0.238	0.227				*ZMIZ1*	5'UTR
cg03534504	5	171,594,998	4.76E-15	0.238	0.222				*STK10*	Body
cg21684012	6	100,912,906	0.014537	0.238	0.302				*SIM1*	TSS1500
cg18893793	12	47,472,810	0.014011	0.238	0.156				*AMIGO2*	5'UTR
cg25929533	6	127,840,616	0.015497	0.238	0.192				*C6orf174*	TSS200
cg04370442	16	58,019,866	1.62E-20	0.238	0.226				*TEPP*	Body
cg05399718	12	123,555,535	7.64E-15	0.238	0.246				*PITPNM2*	5'UTR
cg08636083	1	149,785,211	2.64E-09	0.238	0.204				*HIST2H2BF*	TSS1500
cg09643340	11	129,244,105	6.45E-08	0.238	0.258					
cg12894524	12	116,997,069	1.11E-08	0.238	0.217				*MAP1LC3B2*	TSS200
cg15835817	3	32,433,905	8.8E-05	0.238	0.150				*CMTM7*	Body
cg06103657	12	33,030,976	2.26E-12	0.238	0.202				*PKP2*	Body
cg23972298	1	32,665,582	0.000121	0.238	0.166				*CCDC28B*	TSS1500
cg03402235	15	42,749,336	6.82E-08	0.238	0.193				*ZFP106*	1stExon
cg12848808	15	68,125,599	0.000966	0.238	0.246				*LBXCOR1*	Body
cg24900983	6	152,128,528	0.000578	0.238	0.178				*ESR1*	TSS1500
cg25714956	7	70,096,264	1.2E-09	0.238	0.228				*AUTS2*	Body
cg02116953	20	20,346,166	0.032469	0.238	0.188					
cg25642476	6	29,595,011	7.91E-08	0.238	0.241				*GABBR1*	Body
cg08318842	16	55,512,806	8.23E-07	0.238	0.226				*MMP2*	TSS1500
cg17543296	1	20,880,378	0.005401	0.238	0.129				*FAM43B*	1stExon
cg09720573	12	110,150,261	3.75E-10	0.238	0.232					
cg16353624	6	74,160,974	0.013148	0.237	0.129				*C6orf150*	Body
cg18668813	6	114,177,341	0.020395	0.237	0.178				*MARCKS*	TSS1500
cg21661379	2	228,324,983	6E-16	0.237	0.239					
cg10970143	1	159,866,558	3.44E-05	0.237	0.210				*CCDC19*	Body
cg11111460	4	183,063,637	6.05E-05	0.237	0.217				*MGC45800*	Body
cg15920887	2	190,928,430	1.4E-07	0.237	0.240				*MSTN*	TSS1500

cg17347104	14	75,034,677	1.32E-08	0.237	0.230				*LTBP2*	Body
cg05734722	2	85,122,547	0.033438	0.237	0.105					
cg19779166	14	56,890,696	0.000134	0.237	0.241					
cg21211271	1	6,265,071	0.006372	0.237	0.245				*RNF207*	TSS1500
cg05116382	11	66,327,000	0.000101	0.237	0.269				*ACTN3*	Body
cg24909660	11	47,276,469	0.000125	0.237	0.169				*NR1H3*	5'UTR
cg19984911	5	150,020,338	2.34E-06	0.237	0.238				*SYNPO*	1stExon
cg19910201	7	64,734,691	0.00145	0.237	0.275					
cg19876672	10	118,891,266	0.006463	0.237	0.230				*VAX1*	3'UTR
cg12406726	1	162,792,568	0.027313	0.237	0.328					
cg11854259	7	155,325,815	0.000313	0.237	0.235				*CNPY1*	5'UTR
cg10927449	2	63,286,621	1.15E-06	0.237	0.220					
cg19014941	10	88,127,363	0.003915	0.237	0.125				*GRID1*	TSS1500
cg07234865	1	156,390,220	0.000183	0.237	0.337				*MIR9-1*	Body
cg16596367	16	89,070,757	0.003903	0.237	0.145					
cg17816357	7	124,430,776	0.001429	0.237	0.264					
cg18378551	11	84,844,436	5.94E-08	0.237	0.233				*DLG2*	Body
cg25960038	17	46,667,789	1.53E-12	0.237	0.218				*LOC404266*	TSS200
cg00339682	4	13,531,163	0.013757	0.237	0.315					
cg00994250	6	151,087,821	1.64E-07	0.237	0.237				*PLEKHG1*	Body
cg06005197	11	58,612,853	9.38E-06	0.237	0.230				*GLYATL2*	TSS1500
cg10556772	6	29,795,434	8.96E-08	0.237	0.231				*HLA-G*	5'UTR
cg16426482	16	21,831,587	0.04417	0.237	0.058				*RRN3P1*	TSS1500
cg24368031	17	60,726,097	0.007007	0.237	0.302				*MRC2*	Body
cg05680973	X	132,090,141	3.67E-06	0.237	0.213				*HS6ST2*	Body
cg06613480	3	181,437,383	0.002369	0.237	0.347				*SOX2OT*	Body
cg00961640	1	110,933,767	4.43E-10	0.237	0.234				*SLC16A4*	TSS200
cg13426138	6	31,830,455	0.045187	0.237	0.197				*NEU1*	1stExon
cg00767000	16	87,525,307	3.53E-07	0.237	0.260				*ZCCHC14*	1stExon
cg04428826	X	75,649,367	1.43E-23	0.237	0.228				*MAGEE1*	1stExon
cg12979844	13	46,425,929	0.004695	0.237	0.190				*SIAH3*	TSS200
cg14855931	3	138,631,456	0.001306	0.237	0.261					
cg19200589	21	36,041,605	0.000469	0.237	0.229				*CLIC6*	TSS200
cg18226915	11	126,009,830	1.1E-20	0.237	0.236					

cg01388260	16	85,399,742	2.86E-17	0.237	0.230					
cg19656974	2	66,653,415	1.94E-05	0.237	0.233					
cg25630910	12	123,753,186	2.3E-05	0.237	0.142				*CDK2AP1*	Body
cg12010198	8	145,939,499	3.38E-05	0.237	0.211					
cg07606384	10	94,823,251	2.9E-05	0.237	0.205				*CYP26C1*	Body
cg11869049	3	48,278,910	0.001072	0.237	0.288					
cg20712631	5	172,074,065	2.69E-11	0.237	0.233				*NEURL1B*	Body
cg10532489	16	29,888,304	0.000005	0.237	0.232				*SEZ6L2*	Body
cg11205636	5	107,108,407	1.05E-10	0.237	0.251					
cg15500865	7	95,025,924	0.000277	0.237	0.208				*PON3*	TSS1500
cg07929768	2	232,055,508	0.000697	0.237	0.256					
cg11042214	10	102,906,231	2.82E-05	0.237	0.271					
cg14219620	6	151,561,168	0.000937	0.237	0.129			Yes	*AKAP12*	1stExon
cg05892568	19	30,015,782	0.000979	0.237	0.180					
cg03132556	12	54,366,760	0.00261	0.237	0.242				*HOXC11*	TSS200
cg12542255	19	45,976,195	2.36E-05	0.237	0.156				*FOSB*	Body
cg20067049	2	121,345,149	2.38E-09	0.237	0.229					
cg07999845	4	154,710,961	0.025074	0.237	0.360				*SFRP2*	TSS1500
cg16379462	14	55,119,082	2.56E-08	0.237	0.249				*SAMD4A*	Body
cg04209913	5	37,836,246	5.4E-15	0.237	0.236				*GDNF*	TSS1500
cg04422990	6	137,241,923	3.82E-06	0.237	0.236				*SLC35D3*	TSS1500
cg05961294	2	45,028,264	1.71E-06	0.237	0.219					
cg13571460	9	124,989,337	6.53E-05	0.237	0.257				*LHX6*	Body
cg19209066	10	8,085,349	0.003531	0.237	0.280					
cg24051242	11	68,096,138	1.31E-10	0.237	0.182				*LRP5*	Body
cg18485596	6	111,435,084	2.89E-06	0.237	0.269				*SLC16A10*	Body
cg19911329	6	29,975,345	3.25E-14	0.237	0.235				*HLA-J*	Body
cg02523640	10	129,534,613	0.00339	0.237	0.229				*FOXI2*	TSS1500
cg08823209	19	35,611,675	3.91E-05	0.237	0.233				*FXYD3*	Body
cg01128482	2	177,028,621	0.00829	0.237	0.251				*HOXD3*	TSS200
cg21865845	3	79,815,848	7.76E-05	0.237	0.189				*ROBO1*	5'UTR
cg00889398	13	28,502,089	0.002083	0.237	0.213					
cg09385692	5	180,597,400	0.000939	0.237	0.271					
cg26158279	20	25,565,667	0.022914	0.237	0.112				*NINL*	5'UTR

cg25755575	1	217,311,531	2.67E-05	0.237	0.238				*ESRRG*	TSS1500
cg22706186	3	68,053,371	0.012835	0.237	0.268				*FAM19A1*	TSS200
cg23007665	7	43,690,247	3.17E-09	0.237	0.241				*C7orf44*	5'UTR
cg08469845	17	39,676,096	7.81E-08	0.237	0.224				*KRT15*	TSS1500
cg09408917	21	38,069,321	0.006114	0.237	0.261					
cg07434457	2	220,222,765	3.44E-06	0.237	0.237					
cg09816693	14	74,706,113	1.2E-06	0.237	0.226				*VSX2*	TSS200
cg14977608	1	55,506,512	0.00149	0.237	0.221				*PCSK9*	Body
cg02973792	12	117,408,122	3.3E-42	0.237	0.228			Yes	*FBXW8*	Body
cg02923831	8	38,386,436	2.9E-06	0.237	0.254				*C8orf86*	TSS1500
cg12775181	7	1,284,732	3.26E-06	0.237	0.208					
cg17037355	20	3,218,545	5.3E-17	0.237	0.217				*SLC4A11*	TSS200
cg25399367	6	146,136,238	0.017603	0.237	0.139				*FBXO30*	TSS1500
cg12175311	15	100,538,092	3.53E-13	0.237	0.207				*ADAMTS17*	Body
cg13711424	14	65,017,091	1.91E-08	0.237	0.216				*C14orf50*	Body
cg18433784	21	48,025,155	1.27E-08	0.237	0.209				*S100B*	TSS200
cg22839531	6	91,123,560	0.000738	0.237	0.196					
cg13902210	1	110,754,436	1.75E-05	0.237	0.240				*KCNC4*	1stExon
cg18179931	3	194,119,058	3.56E-05	0.237	0.216				*GP5*	5'UTR
cg01298991	2	20,422,849	3.49E-08	0.237	0.190				*SDC1*	Body
cg21209859	1	167,682,516	9.96E-11	0.237	0.201					
cg06003986	18	35,104,868	0.002677	0.237	0.203				*BRUNOL4*	Body
cg05950572	11	14,004,854	0.000771	0.237	0.217				*SPON1*	Body
cg07981266	6	30,720,311	0.00248	0.237	0.137			Yes		
cg17771652	5	82,767,968	0.007073	0.237	0.309				*VCAN*	5'UTR
cg22178613	4	154,710,499	0.008521	0.237	0.299				*SFRP2*	TSS1500
cg22881652	4	13,532,951	0.013101	0.237	0.340					
cg01857475	12	104,697,193	0.018679	0.237	0.180				*TXNRD1*	5'UTR
cg07847273	3	43,195,345	1.39E-13	0.237	0.237					
cg07892597	1	119,532,093	2.37E-08	0.237	0.219				*TBX15*	1stExon
cg14314071	17	15,165,812	0.002952	0.237	0.223				*PMP22*	5'UTR
cg21099148	17	36,611,691	1.18E-06	0.237	0.193					
cg11323985	8	18,541,497	0.000616	0.237	0.181				*PSD3*	Body
cg24167422	1	9,243,600	3.64E-06	0.237	0.225					

cg25431463	20	36,012,946	5.32E-06	0.237	0.253				*SRC*	Body
cg19773546	17	45,944,339	5.42E-18	0.237	0.221					
cg25212090	1	197,882,170	0.003821	0.237	0.220				*LHX9*	Body
cg01295782	3	71,739,283	1.67E-23	0.237	0.245				*EIF4E3*	Body
cg01521274	14	71,822,452	0.002179	0.237	0.203					
cg02857760	2	177,012,562	1.43E-06	0.237	0.194					
cg05874561	4	154,709,828	0.010402	0.237	0.277				*SFRP2*	1stExon
cg24033224	12	131,438,016	1.42E-09	0.237	0.243				*GPR133*	TSS1500
cg25826546	7	100,770,060	0.013799	0.237	0.231				*SERPINE1*	TSS1500
cg09627520	3	58,410,836	6.31E-07	0.237	0.272				*PXK*	3'UTR
cg14600877	17	78,922,220	1.61E-05	0.237	0.210				*RPTOR*	Body
cg24341611	17	50,235,279	0.018554	0.237	0.255				*CA10*	1stExon
cg12583095	10	92,618,141	0.00779	0.237	0.087				*HTR7*	TSS1500
cg23695990	12	95,967,811	9E-10	0.237	0.221					
cg24908198	11	128,812,992	0.002639	0.237	0.151				*TP53AIP1*	TSS200
cg14473030	9	119,336,987	0.00067	0.237	0.212				*ASTN2*	Body
cg17349389	2	80,530,770	0.00824	0.237	0.270				*CTNNA2*	Body
cg09416188	3	49,538,552	0.000689	0.237	0.206				*DAG1*	5'UTR
cg12224030	17	48,047,608	1.27E-05	0.237	0.260				*DLX4*	Body
cg17346952	12	57,630,107	4.09E-07	0.237	0.242				*NDUFA4L2*	Body
cg04697454	15	93,633,408	9.34E-05	0.237	0.242				*RGMA*	TSS1500
cg18969232	2	45,156,937	4.71E-05	0.237	0.204					
cg01310750	19	52,996,548	2.37E-05	0.237	0.202				*ZNF578*	5'UTR
cg03107235	13	26,586,713	0.000245	0.237	0.233				*ATP8A2*	Body
cg21670944	8	144,962,931	2.13E-14	0.237	0.248					
cg00134602	3	64,251,663	9.05E-05	0.237	0.260					
cg02469095	8	99,960,921	7.31E-07	0.237	0.234				*OSR2*	5'UTR
cg09992184	1	118,151,821	7.07E-05	0.237	0.230				*FAM46C*	5'UTR
cg18771512	X	153,532,633	3.31E-12	0.237	0.251				*TKTL1*	TSS1500
cg06873316	11	20,690,682	0.015648	0.237	0.143				*NELL1*	TSS1500
cg02791331	3	183,993,724	0.008568	0.237	0.210				*ECE2*	TSS200
cg03885399	1	47,691,550	3.72E-05	0.237	0.253				*TAL1*	Body
cg17775490	20	45,179,354	5.1E-07	0.237	0.217				*C20orf123*	TSS200
cg03662459	9	34,654,290	8.93E-31	0.237	0.223	5.64E-10	0.079		*IL11RA*	5'UTR

cg07576931	8	117,530,081	1.83E-07	0.237	0.204					
cg14400292	22	46,367,203	0.00164	0.237	0.182				*WNT7B*	Body
cg16935980	7	155,255,310	0.014891	0.237	0.247				*EN2*	Body
cg04728296	4	166,414,646	2.52E-05	0.237	0.276				*CPE*	Body
cg05337681	15	58,844,500	1.66E-08	0.237	0.237				*LIPC*	Body
cg06142351	2	8,683,945	8.71E-07	0.237	0.219					
cg15822765	6	28,956,387	2.79E-05	0.237	0.237					
cg15146752	1	16,482,767	1.88E-05	0.237	0.147	1.64E-41	0.262		*EPHA2*	TSS1500
cg00191052	X	46,432,770	1.22E-20	0.237	0.231	1.4E-10	0.103		*CHST7*	TSS1500
cg09455225	X	133,684,399	0.033647	0.236	0.181					
cg01201797	2	208,635,605	1.02E-06	0.236	0.233				*FZD5*	TSS1500
cg16087412	17	21,281,017	2.62E-09	0.236	0.239				*KCNJ12*	5'UTR
cg06326971	16	57,836,692	0.000634	0.236	0.197				*KIFC3*	TSS1500
cg07421596	6	31,913,770	3E-05	0.236	0.221				*CFB*	1stExon
cg24425727	6	36,645,648	0.000429	0.236	0.292				*CDKN1A*	TSS1500
cg07776419	X	133,680,476	0.005704	0.236	0.155				*MGC16121*	Body
cg18075666	7	134,550,430	4.34E-06	0.236	0.235				*CALD1*	5'UTR
cg24465685	1	47,009,809	0.028799	0.236	0.313					
cg02170525	2	87,018,382	0.00012	0.236	0.168	3.11E-15	0.121		*CD8A*	5'UTR
cg07713493	1	205,473,393	2.49E-16	0.236	0.218	2.2E-12	0.133		*CDK18*	TSS1500
cg16602460	6	32,156,071	1.86E-16	0.236	0.227				*PBX2*	Body
cg24914185	2	200,663,789	8.81E-15	0.236	0.208					
cg05527785	17	46,656,543	0.002544	0.236	0.154				*HOXB4*	TSS1500
cg15874872	7	100,770,434	0.000394	0.236	0.287				*SERPINE1*	5'UTR
cg18687675	14	95,234,965	3.1E-05	0.236	0.250				*GSC*	Body
cg00339782	1	149,908,099	3.31E-16	0.236	0.228				*MTMR11*	Body
cg10937302	11	33,037,916	2.08E-05	0.236	0.258				*DEPDC7*	5'UTR
cg13740815	19	51,170,356	0.016868	0.236	0.225				*SHANK1*	Body
cg06495184	19	406,256	3.35E-14	0.236	0.216				*C2CD4C*	3'UTR
cg07930539	2	177,025,975	0.000325	0.236	0.179					
cg26690742	11	2,889,602	7.37E-12	0.236	0.226					
cg10345920	8	23,560,319	0.006334	0.236	0.178				*NKX2-6*	Body
cg19893751	2	45,029,285	1.28E-06	0.236	0.215					
cg19928046	5	149,792,249	0.030424	0.236	0.087	3.16E-11	0.127		*CD74*	1stExon

cg27357306	10	63,657,059	4.44E-05	0.236	0.240					
cg05033333	16	3,222,180	0.039994	0.236	0.165					
cg06710596	19	36,024,279	0.000377	0.236	0.249				*GAPDHS*	TSS200
cg14411503	14	103,589,028	2.42E-07	0.236	0.208					
cg21127735	17	47,547,353	0.00068	0.236	0.209					
cg25125652	X	110,039,604	0.007313	0.236	0.087				*CHRDL1*	TSS1500
cg11193342	12	116,872,185	1.14E-06	0.236	0.175					
cg15487498	1	230,372,938	2.4E-13	0.236	0.242				*GALNT2*	Body
cg21165064	1	156,402,678	4.89E-06	0.236	0.244					
cg26856924	1	119,542,573	8.51E-09	0.236	0.243					
cg01946191	12	54,444,463	0.000953	0.236	0.155				*HOXC4*	5'UTR
cg26612165	1	181,059,499	0.036658	0.236	0.121				*IER5*	3'UTR
cg09684846	16	10,912,718	5.64E-12	0.236	0.228				*FAM18A*	TSS200
cg23886978	8	1,272,628	2.12E-05	0.236	0.254					
cg25840237	15	29,967,593	3.64E-28	0.236	0.209					
cg05392323	1	23,698,143	1.1E-06	0.236	0.212				*C1orf213*	3'UTR
cg07058377	17	2,041,773	1.65E-05	0.236	0.166				*SMG6*	Body
cg07722360	20	17,691,454	4.4E-12	0.236	0.214				*BANF2*	5'UTR
cg09664550	10	33,294,358	0.000365	0.236	0.238					
cg23675930	1	26,054,700	2.23E-16	0.236	0.230				*MAN1C1*	Body
cg06452665	13	43,148,436	1.65E-09	0.236	0.238				*TNFSF11*	1stExon
cg08239804	6	134,497,755	1.65E-09	0.236	0.247				*SGK1*	TSS1500
cg25102370	4	174,451,141	0.000184	0.236	0.296				*HAND2*	5'UTR
cg04349243	10	91,295,159	0.012072	0.236	0.267				*SLC16A12*	5'UTR
cg06047587	3	117,118,875	3.09E-10	0.236	0.258					
cg10741760	1	19,992,024	0.004932	0.236	0.183	1.86E-15	0.196		*HTR6*	5'UTR
cg23783862	8	82,658,145	2.24E-14	0.236	0.245				*CHMP4C*	Body
cg26646659	5	55,776,364	2.97E-07	0.236	0.155					
cg07205883	10	116,187,407	2.05E-14	0.236	0.237					
cg10319893	13	37,248,953	0.005889	0.236	0.214				*C13orf36*	5'UTR
cg26229752	10	131,697,345	0.018617	0.236	0.171				*EBF3*	Body
cg22573003	X	15,402,648	2.09E-06	0.236	0.205				*FIGF*	TSS200
cg26618965	8	67,874,206	0.007393	0.236	0.109					
cg09820101	1	162,792,092	5.97E-07	0.236	0.238					

cg03490331	5	172,672,817	1.15E-08	0.236	0.220					
cg23520688	14	103,294,740	9.09E-14	0.236	0.211				*TRAF3*	5'UTR
cg26750742	2	209,054,670	2.88E-10	0.236	0.236				*C2orf80*	5'UTR
cg13164157	4	16,085,180	0.014842	0.236	0.340				*PROM1*	5'UTR
cg08068820	19	11,530,021	0.001778	0.236	0.229				*RGL3*	TSS200
cg26847866	8	27,492,077	3.7E-11	0.236	0.223	2.98E-29	0.146		*SCARA3*	Body
cg16722931	17	75,312,494	1.9E-17	0.236	0.222				*SEPT9*	5'UTR
cg03261800	9	36,985,761	9.6E-06	0.236	0.240				*PAX5*	Body
cg19457477	19	39,108,609	0.020783	0.236	0.225				*MAP4K1*	1stExon
cg03402605	10	134,257,269	0.000777	0.236	0.270				*C10orf91*	TSS1500
cg04850999	10	77,872,084	4.77E-07	0.236	0.215				*C10orf11*	Body
cg12758082	13	113,407,769	0.000232	0.236	0.123				*ATP11A*	Body
cg02028339	6	125,991,844	1.28E-08	0.236	0.236					
cg03277049	3	156,534,076	1.41E-05	0.236	0.265					
cg15989926	12	124,607,875	8.46E-30	0.236	0.234					
cg21219903	1	43,920,090	0.006363	0.236	0.225				*HYI*	TSS200
cg26953640	17	1,174,473	0.000515	0.236	0.273				*BHLHA9*	1stExon
cg01704698	16	84,941,294	2.68E-05	0.236	0.196				*CRISPLD2*	3'UTR
cg18561261	7	123,284,476	2.44E-12	0.236	0.226					
cg12284382	22	35,940,438	1.91E-06	0.236	0.215				*RASD2*	5'UTR
cg26581504	11	112,046,963	3.26E-18	0.236	0.216				*BCO2*	TSS200
cg01793445	1	167,632,415	1.66E-08	0.236	0.252				*RCSD1*	Body
cg02511456	7	156,801,419	0.000786	0.236	0.231				*MNX1*	Body
cg09832245	16	85,494,611	3.68E-11	0.236	0.228					
cg17173369	6	169,224,099	1.24E-08	0.236	0.219					
cg03489023	2	19,557,201	9.94E-06	0.236	0.244				*OSR1*	5'UTR
cg07786995	1	162,114,714	4.95E-06	0.236	0.223				*NOS1AP*	Body
cg09515953	19	38,747,355	0.002859	0.236	0.303				*PPP1R14A*	TSS200
cg02101486	7	96,652,245	0.004322	0.236	0.248	1.97E-14	0.230		*DLX5*	Body
cg13575011	19	16,022,472	9.89E-05	0.236	0.233					
cg14317414	9	109,991,376	8.45E-08	0.236	0.238					
cg01857019	15	57,210,107	0.001386	0.236	0.206				*LOC145783*	Body
cg11854183	19	38,741,928	0.00012	0.236	0.218				*PPP1R14A*	3'UTR
cg05144259	7	157,808,584	1.41E-13	0.236	0.256				*PTPRN2*	Body

cg13638427	6	29,617,320	0.002456	0.236	0.147					
cg20099830	12	114,842,031	0.005282	0.236	0.260				*TBX5*	5'UTR
cg20917077	7	129,996,313	2.73E-12	0.236	0.207				*CPA5*	Body
cg16655084	1	184,005,896	0.011405	0.236	0.168				*GLT25D2*	Body
cg13278353	2	208,635,942	3.6E-10	0.236	0.226					
cg13218688	18	72,125,544	2.3E-08	0.236	0.195				*FAM69C*	TSS1500
cg06420480	17	78,923,244	1.03E-20	0.236	0.239				*RPTOR*	Body
cg08555653	3	138,659,021	0.000175	0.236	0.239					
cg02773945	1	200,011,858	0.019246	0.236	0.261				*NR5A2*	Body
cg13502125	1	147,826,191	3.29E-09	0.236	0.244					
cg15794778	Y	21,665,021	0.014256	0.236	0.192				*BCORL2*	Body
cg24056567	8	18,871,909	0.007174	0.236	0.127	8.24E-14	0.158		*PSD3*	TSS1500
cg04555312	12	54,389,114	0.006922	0.236	0.205					
cg06324373	10	99,734,805	0.000597	0.236	0.257				*CRTAC1*	Body
cg08119655	11	7,595,925	1.92E-10	0.236	0.231				*PPFIBP2*	Body
cg16519842	1	149,399,911	7.4E-09	0.236	0.180				*HIST2H2BF*	TSS1500
cg07212778	2	119,916,510	0.00294	0.236	0.220				*C1QL2*	TSS200
cg02235562	13	36,729,137	0.000835	0.236	0.189					
cg04277821	6	150,572,395	6.23E-09	0.236	0.185					
cg14344989	9	137,329,237	6.88E-13	0.236	0.238				*RXRA*	3'UTR
cg18376817	15	64,438,372	1.06E-26	0.236	0.227					
cg13943560	X	40,004,481	4.56E-06	0.236	0.159				*BCOR*	5'UTR
cg09000800	17	4,545,081	0.020553	0.236	0.367				*ALOX15*	TSS200
cg26754262	8	75,617,860	3.33E-13	0.236	0.254				*MIR2052*	TSS200
cg02694884	8	99,411,691	1.86E-24	0.236	0.240					
cg16924045	2	191,343,262	0.000124	0.236	0.210				*MFSD6*	Body
cg24460369	12	109,549,041	2.46E-07	0.236	0.175					
cg26376168	6	30,712,439	0.006957	0.236	0.207				*IER3*	TSS200
cg19592637	20	25,063,052	1.09E-08	0.236	0.211				*VSX1*	TSS1500
cg21553980	17	109,444	1.27E-09	0.236	0.212				*RPH3AL*	Body
cg27333706	1	209,602,002	6.55E-05	0.236	0.135				*LOC642587*	TSS200
cg09664054	1	42,179,097	1.38E-22	0.236	0.236				*HIVEP3*	5'UTR
cg19556613	5	116,280,275	1.41E-11	0.236	0.232					
cg24674635	20	6,750,696	0.007389	0.236	0.277				*BMP2*	5'UTR

cg15423872	8	58,906,736	0.028045	0.236	0.133				*FAM110B*	TSS1500
cg16214653	15	100,048,500	5.98E-05	0.236	0.205					
cg19250989	1	147,752,892	5.64E-05	0.236	0.252					
cg01494399	16	81,709,890	8.1E-11	0.236	0.247				*CMIP*	Body
cg05730368	6	42,146,052	7.28E-10	0.236	0.217				*GUCA1A*	Body
cg07502417	1	230,849,801	0.00048	0.236	0.245				*AGT*	5'UTR
cg14457691	X	100,740,937	0.000653	0.236	0.171	2.06E-17	0.184		*ARMCX4*	Body
cg04447362	2	237,654,635	5.77E-08	0.236	0.193					
cg05551825	7	5,735,129	8.17E-06	0.236	0.216				*RNF216*	Body
cg13303069	2	85,361,842	0.000745	0.236	0.280				*TCF7L1*	Body
cg11676353	1	39,548,001	6.76E-12	0.236	0.248				*MACF1*	5'UTR
cg03423916	1	153,670,787	8.92E-05	0.236	0.233					
cg17585615	21	46,128,908	0.019559	0.236	0.258				*C21orf29*	Body
cg02222324	2	10,231,621	0.002417	0.236	0.227					
cg13290371	7	5,518,071	3.6E-11	0.236	0.224				*FBXL18*	3'UTR
cg23986620	2	177,012,706	7.96E-09	0.236	0.214					
cg10189261	14	101,176,105	0.001863	0.236	0.205					
cg25529875	6	13,413,705	1.26E-16	0.236	0.242				*GFOD1*	Body
cg04510874	15	91,427,884	0.005671	0.236	0.164				*FES*	5'UTR
cg09493406	7	91,765,513	1.38E-18	0.236	0.199				*CYP51A1*	TSS1500
cg10572355	1	119,549,145	0.007266	0.236	0.217					
cg11642106	3	27,772,805	0.000641	0.236	0.255					
cg14582917	9	132,262,821	9.06E-18	0.236	0.247					
cg25625968	5	38,464,319	1.13E-07	0.236	0.256				*EGFLAM*	3'UTR
cg13274938	17	38,493,822	3.45E-17	0.236	0.229				*RARA*	Body
cg27385193	7	21,208,954	1.26E-07	0.236	0.213					
cg14410476	2	95,693,023	0.003622	0.236	0.247				*MAL*	Body
cg23227259	2	132,152,543	0.00027	0.236	0.261					
cg01336781	8	23,563,182	0.001064	0.236	0.189				*NKX2-6*	Body
cg01402631	5	136,359,400	7.03E-10	0.236	0.208				*SPOCK1*	Body
cg05121006	4	85,402,397	0.006879	0.236	0.237					
cg21540621	11	115,531,033	1.01E-06	0.236	0.200					
cg01697487	17	48,588,559	7.55E-06	0.236	0.241				*MYCBPAP*	Body
cg12619509	16	2,286,601	0.003891	0.236	0.129	9.86E-11	0.134		*DNASE1L2*	5'UTR

cg06872822	5	6,582,121	6.17E-15	0.236	0.249				*LOC255167*	TSS1500
cg03130910	1	234,908,226	0.029539	0.236	0.149					
cg20599967	2	119,592,754	0.001339	0.236	0.204					
cg22010052	5	115,298,704	1.4E-06	0.236	0.229				*LVRN*	1stExon
cg26031954	1	2,139,327	1.09E-12	0.236	0.230				*C1orf86*	TSS200
cg06083669	20	3,189,437	0.003041	0.236	0.221				*ITPA*	TSS1500
cg08639389	4	26,995,889	1.2E-16	0.236	0.234				*STIM2*	Body
cg20264966	10	68,688,049	0.000538	0.236	0.223				*CTNNA3*	Body
cg09430647	10	106,247,914	0.012298	0.236	0.192					
cg03131724	11	58,731,433	0.00014	0.236	0.201					
cg26259363	2	220,282,281	5.22E-09	0.236	0.252	4.71E-26	0.260		*DES*	TSS1500
cg13720639	14	72,061,746	3.43E-08	0.235	0.220			Yes	*SIPA1L1*	Body
cg23012600	1	244,088,110	1.02E-12	0.235	0.218					
cg25508679	6	28,956,403	3.86E-06	0.235	0.232					
cg14916924	9	129,433,969	6.95E-10	0.235	0.231				*LMX1B*	Body
cg00040837	1	236,081,097	5.68E-05	0.235	0.224			Yes		
cg03605610	6	41,394,765	4.24E-17	0.235	0.220					
cg09943716	20	55,202,236	3.99E-05	0.235	0.236					
cg27002699	19	11,689,538	0.000184	0.235	0.235				*ACP5*	TSS1500
cg01776188	5	172,664,082	0.00015	0.235	0.216					
cg14213692	4	6,732,388	1.43E-06	0.235	0.251					
cg17864046	6	166,422,341	0.002323	0.235	0.198					
cg20292636	1	204,328,377	9.9E-18	0.235	0.237				*PLEKHA6*	5'UTR
cg26310273	3	55,377,420	5.9E-15	0.235	0.220					
cg04221521	5	72,597,715	0.000139	0.235	0.174					
cg04659582	2	231,276,073	1.1E-05	0.235	0.180					
cg12018969	2	43,350,413	2.32E-14	0.235	0.234			Yes		
cg20822858	6	134,499,519	0.006599	0.235	0.273				*SGK1*	Body
cg09940032	8	142,215,736	0.047377	0.235	0.173					
cg23207990	4	154,710,535	0.00328	0.235	0.298				*SFRP2*	TSS1500
cg18297437	3	147,131,332	4.12E-07	0.235	0.260				*ZIC1*	Body
cg03085785	7	27,247,651	4.69E-05	0.235	0.235					
cg26397549	14	37,116,121	3.8E-05	0.235	0.227					
cg03692563	11	31,846,414	0.000838	0.235	0.264					

cg16365064	5	172,984,486	4.4E-14	0.235	0.213					
cg27204671	17	46,831,623	5.59E-06	0.235	0.222					
cg17759535	1	200,010,137	0.007495	0.235	0.293				*NR5A2*	Body
cg02027878	7	150,711,138	2.32E-06	0.235	0.211				*ATG9B*	3'UTR
cg09229918	16	2,041,916	4.31E-05	0.235	0.206				*SYNGR3*	Body
cg09267056	1	90,410,109	2.88E-10	0.235	0.214					
cg22624907	1	80,447,732	0.014559	0.235	0.135					
cg04224064	14	36,992,233	1.97E-05	0.235	0.257					
cg08884974	15	31,505,024	0.017754	0.235	0.127					
cg09550083	2	66,672,337	2.11E-06	0.235	0.244				*MEIS1*	Body
cg12235877	12	323,392	0.012964	0.235	0.307				*SLC6A12*	TSS1500
cg15087907	10	3,169,259	0.033098	0.235	0.077				*PFKP*	Body
cg04864807	2	121,412,139	1.25E-09	0.235	0.231					
cg09018299	16	24,748,339	2.25E-08	0.235	0.248				*TNRC6A*	Body
cg10184983	17	36,734,911	1.49E-05	0.235	0.224				*SRCIN1*	Body
cg06393679	7	7,140,086	1.48E-07	0.235	0.203					
cg09178385	1	35,246,759	4.01E-08	0.235	0.186				*GJB3*	TSS200
cg14015656	9	100,070,142	0.006879	0.235	0.169				*KIAA1529*	Body
cg17654970	2	163,027,484	0.000252	0.235	0.179				*FAP*	3'UTR
cg00743094	13	100,547,968	0.000412	0.235	0.210				*CLYBL*	3'UTR
cg09571713	4	168,154,775	0.004906	0.235	0.280				*SPOCK3*	Body
cg01777643	1	224,805,287	0.01186	0.235	0.201				*CNIH3*	Body
cg22798214	1	212,692,190	4.42E-07	0.235	0.213					
cg24530432	4	174,428,060	0.008785	0.235	0.238					
cg03475190	7	30,919,932	1.55E-08	0.235	0.201				*FAM188B*	Body
cg20643029	21	37,915,044	5.48E-08	0.235	0.215				*CLDN14*	TSS200
cg25320780	1	110,933,345	4.94E-08	0.235	0.223				*SLC16A4*	5'UTR
cg27662789	12	43,666,281	2.55E-12	0.235	0.199					
cg03350138	6	28,446,794	0.000166	0.235	0.202					
cg25600766	12	22,486,484	0.000134	0.235	0.202				*ST8SIA1*	Body
cg26382697	11	2,406,712	3.36E-17	0.235	0.212				*CD81*	Body
cg24985060	15	25,920,220	4.09E-06	0.235	0.199					
cg01254505	19	17,516,470	1.21E-06	0.235	0.201	3.37E-14	0.127		*BST2*	TSS200
cg01478234	12	107,975,299	3.35E-13	0.235	0.216				*BTBD11*	Body

cg06133205	13	50,701,960	5.09E-05	0.235	0.241					
cg18698884	11	16,632,724	0.004058	0.235	0.195					
cg09912079	4	3,409,828	1.17E-11	0.235	0.209				*RGS12*	Body
cg16231241	6	33,215,838	0.000725	0.235	0.243					
cg16563171	2	223,156,106	0.001884	0.235	0.202				*PAX3*	Body
cg26626663	8	121,137,879	0.004191	0.235	0.291				*COL14A1*	5'UTR
cg27577554	17	30,412,142	1.09E-06	0.235	0.211					
cg01347682	4	140,656,794	9.13E-05	0.235	0.209				*MAML3*	Body
cg14098223	1	2,262,333	3.37E-06	0.235	0.247				*MORN1*	Body
cg25701646	3	57,969,522	7.44E-06	0.235	0.217					
cg00525383	X	136,647,793	0.000145	0.235	0.273				*ZIC3*	TSS1500
cg05239310	14	95,651,984	2.29E-07	0.235	0.224				*CLMN*	3'UTR
cg09507761	5	96,152,293	1.4E-05	0.235	0.222					
cg01202519	11	85,645,743	9.22E-16	0.235	0.248					
cg18836366	13	24,007,560	0.006138	0.235	0.140				*SACS*	5'UTR
cg03547017	17	37,031,281	2.72E-09	0.235	0.226				*LASP1*	Body
cg23452752	2	10,572,946	0.000556	0.235	0.170					
cg26506007	17	41,939,449	8.17E-13	0.235	0.249				*CD300LG*	3'UTR
cg03731473	2	130,901,674	6.22E-07	0.235	0.206				*CCDC74B*	Body
cg08545287	16	6,069,047	0.031054	0.235	0.234				*A2BP1*	TSS200
cg16565394	6	100,896,674	0.001159	0.235	0.268				*SIM1*	Body
cg05477457	4	169,799,308	0.002809	0.235	0.134				*PALLD*	Body
cg20823772	8	99,962,013	1.42E-07	0.235	0.236				*OSR2*	Body
cg03020951	6	34,203,887	1.48E-05	0.235	0.225	3.89E-16	0.191		*HMGA1*	TSS1500
cg16079347	9	100,265,181	2.44E-08	0.235	0.198				*TMOD1*	5'UTR
cg01341487	22	43,505,954	7.67E-10	0.235	0.176				*BIK*	TSS1500
cg04441405	5	1,877,309	6.78E-07	0.235	0.212					
cg08461451	19	2,295,092	2.21E-11	0.235	0.210				*LINGO3*	5'UTR
cg07696842	12	105,089,508	0.001044	0.235	0.179				*CHST11*	Body
cg08566449	6	31,631,706	9.57E-08	0.235	0.246				*BAT4*	Body
cg14221171	7	97,361,759	0.000272	0.235	0.247	1.03E-18	0.168		*TAC1*	5'UTR
cg06267617	1	172,291,727	5.42E-14	0.235	0.225				*DNM3*	Body
cg15530351	8	6,407,676	7.16E-06	0.235	0.210				*ANGPT2*	Body
cg03339609	19	34,662,636	1.9E-05	0.235	0.239				*LSM14A*	TSS1500

cg09100271	8	48,675,911	1.3E-05	0.235	0.171					
cg18892169	1	150,737,762	0.000715	0.235	0.194				*CTSS*	5'UTR
cg24805875	6	117,086,941	0.017595	0.235	0.198				*FAM162B*	TSS200
cg04110105	3	46,395,356	0.001599	0.235	0.225				*CCR2*	5'UTR
cg25819602	7	96,627,634	0.036524	0.235	0.310				*DLX6AS*	Body
cg04971534	17	27,333,432	0.005228	0.235	0.239				*SEZ6*	TSS1500
cg06126593	16	20,910,405	0.000262	0.235	0.249				*LYRM1*	TSS1500
cg12172441	6	28,176,163	0.003374	0.235	0.284					
cg26530713	1	153,928,406	3.35E-31	0.235	0.221				*CRTC2*	Body
cg11390690	10	86,120,572	7.7E-08	0.235	0.244				*FAM190B*	5'UTR
cg12338137	2	218,701,429	2.63E-08	0.235	0.221				*TNS1*	Body
cg13677415	17	35,291,900	0.000443	0.235	0.235					
cg17882660	13	100,637,258	0.000422	0.235	0.212				*ZIC2*	Body
cg10129665	12	54,133,618	0.003839	0.235	0.170					
cg14037769	1	9,895,799	3.43E-12	0.235	0.220					
cg21049587	X	46,938,148	6.91E-08	0.235	0.246				*RGN*	5'UTR
cg26365545	5	76,932,016	0.006989	0.235	0.270				*OTP*	Body
cg23244289	5	79,331,135	0.035889	0.235	0.138				*THBS4*	5'UTR
cg20492922	1	39,194,066	0.008246	0.235	0.140					
cg07919145	22	39,780,408	1.3E-11	0.235	0.229				*SYNGR1*	3'UTR
cg13023646	4	165,898,835	2.12E-07	0.235	0.219				*TRIM61*	TSS200
cg15312264	14	55,907,299	0.000105	0.235	0.218				*TBPL2*	TSS200
cg23262274	1	38,412,519	3.9E-06	0.235	0.217				*INPP5B*	5'UTR
cg04496791	6	41,395,094	0.004735	0.235	0.158					
cg01490094	8	72,917,363	0.019489	0.235	0.298					
cg11072113	11	61,063,124	2.02E-19	0.235	0.212				*VWCE*	TSS1500
cg14260530	9	117,157,871	1.65E-22	0.235	0.206				*AKNA*	TSS1500
cg16546861	X	49,022,452	1.35E-07	0.235	0.174				*MAGIX*	Body
cg24771804	18	55,107,755	0.002348	0.235	0.302				*ONECUT2*	Body
cg16592832	22	24,891,141	0.034196	0.235	0.266				*C22orf45*	TSS1500
cg27342837	2	188,419,568	0.00924	0.235	0.215				*TFPI*	TSS1500
cg00169725	5	176,543,901	0.041208	0.235	0.224					
cg17924476	5	323,794	2.86E-09	0.235	0.246				*AHRR*	Body
cg22578843	13	28,495,598	0.017599	0.235	0.208				*PDX1*	Body

cg04339553	15	37,307,513	0.010655	0.235	0.240				*MEIS2*	Body
cg09477232	7	106,061,801	1.6E-06	0.235	0.230					
cg01363324	9	124,979,353	3.16E-11	0.235	0.223				*LHX6*	Body
cg23047271	3	64,210,991	0.000332	0.235	0.197	1.01E-07	0.102		*PRICKLE2*	1stExon
cg07492518	20	443,641	0.001463	0.235	0.291				*TBC1D20*	TSS1500
cg15053022	19	36,048,484	2.65E-06	0.235	0.223				*ATP4A*	Body
cg03821689	11	117,748,187	5.26E-05	0.235	0.195				*FXYD6*	TSS1500
cg20026939	18	44,788,898	0.005684	0.235	0.213					
cg01779383	10	94,180,753	7.99E-06	0.235	0.202					
cg03763874	19	933,707	6.37E-12	0.235	0.242				*ARID3A*	Body
cg05469885	3	124,103,652	4.05E-08	0.235	0.208				*KALRN*	Body
cg24479524	1	20,512,796	3.58E-05	0.235	0.220				*UBXN10*	5'UTR
cg16966962	1	201,915,989	0.013282	0.235	0.086				*LMOD1*	TSS1500
cg10749912	10	99,531,934	0.029712	0.235	0.194				*SFRP5*	TSS200
cg11071207	1	110,611,218	0.000321	0.235	0.242				*ALX3*	Body
cg11635839	11	129,243,456	0.001087	0.235	0.292					
cg24936799	10	99,798,395	5.36E-05	0.235	0.244					
cg00901493	17	7,197,375	0.000179	0.235	0.172	8.77E-18	0.174		*YBX2*	Body
cg04352288	16	87,958,407	0.001109	0.235	0.133				*CA5A*	Body
cg05979400	2	27,295,591	5.45E-11	0.235	0.228				*LOC100128731*	TSS1500
cg27209729	11	64,428,925	5.81E-09	0.235	0.239				*NRXN2*	Body
cg14031365	7	20,816,695	5.96E-06	0.235	0.194					
cg17374433	10	119,876,327	0.014436	0.235	0.189				*CASC2*	Body
cg00318947	7	27,154,914	2.3E-10	0.235	0.227				*HOXA3*	5'UTR
cg24262325	21	43,305,576	1.08E-10	0.235	0.180				*C2CD2*	3'UTR
cg03270710	2	74,729,592	0.000398	0.235	0.248				*LBX2*	Body
cg05265484	17	52,977,445	0.000436	0.235	0.201				*TOM1L1*	TSS1500
cg06470626	16	22,364,961	8.88E-06	0.235	0.232				*CDR2*	Body
cg07021052	2	20,871,279	0.000304	0.235	0.271					
cg23881926	8	59,058,656	1.57E-06	0.235	0.223				*FAM110B*	5'UTR
cg07312701	13	46,189,883	0.022181	0.235	0.282				*FAM194B*	TSS200
cg12150784	11	119,613,308	0.000513	0.235	0.217					
cg05576245	7	138,719,784	2.69E-16	0.235	0.230				*ZC3HAV1L*	Body
cg13877315	9	140,172,089	4.56E-06	0.235	0.233				*C9orf167*	TSS200

cg15632862	1	38,471,572	0.000992	0.235	0.153				*FHL3*	TSS1500
cg19803952	20	44,036,136	1.91E-12	0.235	0.218				*DBNDD2*	5'UTR
cg20820622	15	70,354,647	1.39E-17	0.235	0.239				*TLE3*	Body
cg13918433	7	115,779,350	8.01E-15	0.235	0.235					
cg17714010	17	78,968,450	7.51E-10	0.235	0.235				*CHMP6*	Body
cg17868936	1	76,053,565	0.004194	0.235	0.232				*SLC44A5*	5'UTR
cg07229760	11	130,596,374	6.67E-20	0.235	0.230					
cg14834903	14	73,712,902	0.000954	0.235	0.260				*PAPLN*	Body
cg26061357	7	150,969,275	2.1E-05	0.235	0.254				*SMARCD3*	Body
cg04739880	6	35,017,865	0.00011	0.235	0.223				*ANKS1A*	Body
cg12938020	2	177,027,075	0.005772	0.235	0.164					
cg00698916	6	13,767,628	1.78E-08	0.235	0.232					
cg20417500	5	73,109,241	8.3E-07	0.235	0.270				*RGNEF*	Body
cg18233786	8	23,260,961	0.003161	0.235	0.183				*LOXL2*	5'UTR
cg01922437	12	67,461,690	5.48E-17	0.235	0.228					
cg05927714	17	36,734,660	1.06E-05	0.235	0.223				*SRCIN1*	Body
cg21494776	19	10,397,780	4.29E-08	0.235	0.218	2.92E-13	0.115		*ICAM4*	1stExon
cg03329019	1	221,051,117	4.43E-10	0.234	0.231					
cg10020892	1	147,016,901	5.77E-10	0.234	0.230				*BCL9*	5'UTR
cg10061736	X	133,756,545	4.35E-07	0.234	0.221				*PLAC1*	5'UTR
cg22280258	17	4,545,157	0.021506	0.234	0.260				*ALOX15*	TSS200
cg01816186	2	15,310,191	2.52E-08	0.234	0.218				*NBAS*	Body
cg04243401	14	37,128,378	0.000177	0.234	0.238				*PAX9*	5'UTR
cg15611624	5	156,886,970	0.016153	0.234	0.119				*NIPAL4*	TSS200
cg25065716	10	1,422,865	1.53E-10	0.234	0.242				*ADARB2*	Body
cg02378006	10	73,026,288	1.23E-06	0.234	0.226				*UNC5B*	Body
cg03863486	6	138,724,410	7.59E-08	0.234	0.226				*HEBP2*	TSS1500
cg05118764	3	183,993,721	0.009732	0.234	0.215				*ECE2*	TSS200
cg19741167	2	86,263,270	0.015105	0.234	0.321				*POLR1A*	Body
cg04601957	2	105,467,144	0.000457	0.234	0.258					
cg22310648	3	58,603,027	0.000799	0.234	0.222					
cg23130075	11	67,418,365	3.14E-11	0.234	0.238				*ACY3*	TSS1500
cg26564117	22	28,249,567	9.16E-12	0.234	0.236				*PITPNB*	3'UTR
cg01093786	11	96,047,698	0.00212	0.234	0.226				*MAML2*	Body

cg03774463	7	96,636,616	7.32E-06	0.234	0.227					*DLX6AS*	Body
cg04552451	8	144,852,873	0.000606	0.234	0.232						
cg23250528	15	29,423,177	4.43E-06	0.234	0.226					*FAM189A1*	Body
cg25313930	2	225,266,783	0.020851	0.234	0.276					*FAM124B*	TSS200
cg24864511	1	6,480,516	0.00083	0.234	0.218					*HES2*	TSS1500
cg03820828	2	219,922,362	1.22E-05	0.234	0.268					*IHH*	Body
cg27544405	1	14,108,975	1.22E-09	0.234	0.211					*PRDM2*	Body
cg24328539	15	45,671,016	0.000871	0.234	0.208					*LOC145663*	Body
cg01207473	6	159,359,940	5.41E-09	0.234	0.214						
cg02620769	12	49,298,070	0.006826	0.234	0.220	6.66E-15	0.164			*CCDC65*	1stExon
cg21684845	18	19,755,501	1.02E-22	0.234	0.209					*GATA6*	Body
cg04455999	X	119,444,838	0.036942	0.234	0.182	8.22E-09	0.126			*FAM70A*	Body
cg12400025	6	111,913,206	4.83E-10	0.234	0.225					*TRAF3IP2*	Body
cg25075794	7	30,951,392	6.22E-07	0.234	0.218					*AQP1*	TSS200
cg25168494	2	63,286,355	3.85E-22	0.234	0.229						
cg19868007	6	29,691,890	0.002253	0.234	0.232					*HLA-F*	Body
cg26992010	15	89,951,870	0.000126	0.234	0.258						
cg18771300	14	63,671,737	1.07E-06	0.234	0.205	3.1E-08	0.100			*RHOJ*	1stExon
cg19024632	2	18,059,103	0.000846	0.234	0.124					*KCNS3*	TSS1500
cg24000814	3	184,301,730	0.000406	0.234	0.219						
cg25644224	7	158,195,876	3.26E-08	0.234	0.205					*PTPRN2*	Body
cg20507963	4	13,541,863	0.008293	0.234	0.203						
cg26711508	8	119,750,651	3.25E-05	0.234	0.235						
cg02066681	21	46,823,800	3.76E-08	0.234	0.190	3.01E-23	0.174			*COL18A1*	TSS1500
cg07204662	11	64,081,430	6.22E-21	0.234	0.271					*ESRRA*	Body
cg09830083	5	131,561,058	3.03E-23	0.234	0.222					*P4HA2*	5'UTR
cg18789547	3	42,307,799	3.08E-07	0.234	0.231					*CCK*	TSS1500
cg00046899	13	97,777,690	0.000839	0.234	0.243						
cg03693105	7	151,108,340	1.66E-05	0.234	0.230					*WDR86*	TSS1500
cg10135197	3	126,854,676	3.31E-10	0.234	0.204						
cg10639585	12	130,387,131	0.006722	0.234	0.228					*TMEM132D*	Body
cg24008577	3	62,353,504	0.007097	0.234	0.194						
cg19285800	13	21,520,379	1.46E-06	0.234	0.210						
cg25720804	5	170,736,389	0.009542	0.234	0.252	1.31E-13	0.239			*TLX3*	1stExon

cg26113448	6	27,248,065	0.016178	0.234	0.199					
cg07650504	12	48,397,055	0.001261	0.234	0.185				*COL2A1*	Body
cg11571124	12	122,851,438	4.56E-06	0.234	0.188				*CLIP1*	Body
cg16283208	11	59,323,398	0.000201	0.234	0.226					
cg07451080	3	44,041,025	0.041762	0.234	0.209					
cg03215422	5	135,266,509	8.91E-07	0.234	0.231				*FBXL21*	5'UTR
cg17705334	10	81,002,615	0.011184	0.234	0.154				*ZMIZ1*	Body
cg04153740	16	54,971,018	0.014011	0.234	0.294					
cg08111832	14	24,868,082	0.005339	0.234	0.114				*NYNRIN*	1stExon
cg08549332	7	101,558,461	3.73E-32	0.234	0.230				*CUX1*	Body
cg11308643	11	105,480,788	5.45E-05	0.234	0.213				*GRIA4*	TSS200
cg14415629	11	33,033,607	3.69E-05	0.234	0.215					
cg27152890	19	45,900,241	1.64E-15	0.234	0.228				*PPP1R13L*	Body
cg13792913	11	118,026,593	1.52E-15	0.234	0.218					
cg27405731	7	101,571,203	2.15E-14	0.234	0.210				*CUX1*	Body
cg04105342	4	30,724,841	3.66E-10	0.234	0.232				*PCDH7*	1stExon
cg14794298	11	13,336,674	3.64E-14	0.234	0.228				*ARNTL*	5'UTR
cg18964775	10	102,890,986	0.018618	0.234	0.155				*TLX1*	TSS200
cg25828185	12	54,455,656	1.13E-05	0.234	0.184				*FLJ12825*	Body
cg00253228	8	145,025,610	1.51E-07	0.234	0.226				*PLEC1*	Body
cg15739913	3	4,722,338	5.1E-17	0.234	0.226				*ITPR1*	Body
cg23758703	10	114,610,508	1.65E-05	0.234	0.275				*LOC143188*	TSS200
cg26109199	21	34,755,747	2.49E-08	0.234	0.262					
cg14262955	9	132,114,769	5.07E-10	0.234	0.185					
cg05915892	18	673,911	3.16E-07	0.234	0.253				*ENOSF1*	3'UTR
cg07243147	7	150,744,274	1.06E-09	0.234	0.201				*ACCN3*	TSS1500
cg18522038	17	30,568,596	4.49E-05	0.234	0.239					
cg19812432	17	19,351,904	1.33E-11	0.234	0.240					
cg23734137	15	96,868,114	1.15E-07	0.234	0.217				*NR2F2*	TSS1500
cg15759056	7	87,229,552	0.001519	0.234	0.171				*ABCB1*	5'UTR
cg07741192	16	4,690,013	2.47E-20	0.234	0.215				*MGRN1*	Body
cg15684724	8	67,875,033	0.009563	0.234	0.204					
cg02602182	X	40,026,844	0.043343	0.234	0.274				*BCOR*	5'UTR
cg07208853	1	200,005,219	0.003315	0.234	0.272				*NR5A2*	Body

cg13608733	11	8,228,022	1.4E-05	0.234	0.221					
cg16170614	4	24,802,387	1.15E-08	0.234	0.219				*SOD3*	3'UTR
cg19952015	7	105,432,653	7.77E-09	0.234	0.227				*ATXN7L1*	Body
cg04466549	8	23,567,747	1.57E-05	0.234	0.222					
cg06793867	19	7,927,374	3.63E-08	0.234	0.206				*EVI5L*	Body
cg23913995	20	21,490,665	0.014017	0.234	0.259					
cg25890640	11	68,610,632	0.000102	0.234	0.225				*CPT1A*	TSS1500
cg02732252	16	3,068,014	0.001626	0.234	0.254				*CLDN6*	5'UTR
cg04121415	3	38,325,566	3.42E-13	0.234	0.231					
cg07847030	2	85,361,959	0.004815	0.234	0.259				*TCF7L1*	Body
cg09784934	2	177,023,062	0.000425	0.234	0.268					
cg25030888	1	67,156,909	2.99E-05	0.234	0.228				*SGIP1*	Body
cg06133670	7	127,744,553	0.002815	0.234	0.207					
cg08460812	4	109,087,419	9.73E-05	0.234	0.233				*LOC641518*	TSS1500
cg20034617	9	140,172,277	3.37E-07	0.234	0.222				*C9orf167*	TSS200
cg15833534	1	31,845,985	0.007461	0.234	0.132				*FABP3*	TSS200
cg02319318	8	81,639,711	0.007449	0.234	0.191				*ZNF704*	Body
cg14751369	12	117,042,332	4.87E-05	0.234	0.238					
cg24713122	11	46,389,164	1.22E-13	0.234	0.233				*DGKZ*	Body
cg25298725	6	30,228,047	0.00247	0.234	0.205				*HLA-L*	Body
cg06427054	4	54,963,174	7.58E-09	0.234	0.255					
cg06854842	2	26,947,187	9.23E-20	0.234	0.212				*KCNK3*	Body
cg23651826	11	115,630,531	0.001493	0.234	0.176					
cg00328075	8	12,989,417	2.44E-07	0.234	0.189				*DLC1*	Body
cg19401033	3	107,148,372	4.11E-05	0.234	0.203					
cg23037133	13	97,646,811	0.006953	0.234	0.295	4.39E-19	0.223		*OXGR1*	TSS1500
cg15333426	4	10,463,620	0.000613	0.234	0.252					
cg10828904	10	126,703,991	0.000102	0.234	0.218				*CTBP2*	Body
cg11898347	Y	2,655,866	0.022781	0.234	0.256				*SRY*	TSS200
cg00001583	1	200,011,786	0.039099	0.234	0.217				*NR5A2*	Body
cg02689863	13	95,357,832	1.88E-05	0.234	0.214					
cg08553437	4	122,686,456	0.018744	0.234	0.144				*TMEM155*	TSS200
cg01081263	11	9,112,471	9.06E-09	0.234	0.168	1.41E-08	0.127		*SCUBE2*	Body
cg22024120	6	30,696,029	0.003219	0.234	0.293				*FLOT1*	Body

cg00980581	6	36,238,095	0.000429	0.234	0.237				*PNPLA1*	5'UTR
cg09246103	1	8,456,466	0.000247	0.234	0.164				*RERE*	5'UTR
cg22480558	5	150,461,211	0.000524	0.234	0.178				*TNIP1*	TSS1500
cg03241909	11	64,952,034	4.41E-11	0.234	0.224				*CAPN1*	Body
cg09718037	12	117,042,854	0.007673	0.234	0.238					
cg21878149	1	221,052,023	0.007149	0.234	0.222				*HLX*	TSS1500
cg20972142	19	20,189,112	0.001735	0.234	0.220				*ZNF90*	Body
cg23553576	11	85,451,557	0.000238	0.234	0.166				*SYTL2*	Body
cg01300317	12	110,316,883	4.43E-06	0.234	0.202				*GLTP*	Body
cg20963227	4	41,868,203	3.16E-05	0.234	0.219					
cg00723997	X	48,690,473	0.020721	0.234	0.303				*PCSK1N*	Body
cg12897164	8	124,529,273	4.95E-05	0.234	0.236				*FBXO32*	5'UTR
cg00022997	6	31,650,808	8.6E-07	0.234	0.276					
cg06492372	2	97,573,176	0.014078	0.234	0.234				*FAM178B*	Body
cg13547644	1	229,569,608	0.000588	0.234	0.240	4.28E-14	0.217		*ACTA1*	5'UTR
cg19784721	10	126,043,521	0.000439	0.234	0.166					
cg24932585	19	12,666,317	3.5E-06	0.234	0.235					
cg05580671	8	38,006,196	0.022285	0.234	0.234				*STAR*	Body
cg15393275	2	45,165,193	0.000483	0.234	0.230					
cg24458609	11	56,948,015	0.012624	0.234	0.236				*LRRC55*	TSS1500
cg25149238	2	45,030,384	2.08E-05	0.234	0.242					
cg27065254	17	71,289,332	5.87E-13	0.234	0.222				*CDC42EP4*	5'UTR
cg03740162	9	1,050,680	0.022698	0.234	0.251				*DMRT2*	1stExon
cg06193628	2	228,526,145	1E-09	0.234	0.226					
cg06984848	16	85,320,722	1.06E-18	0.234	0.210					
cg08310837	16	67,572,860	2.82E-07	0.234	0.207				*FAM65A*	Body
cg16736018	1	156,661,648	1.13E-14	0.234	0.219					
cg23657099	7	1,102,138	7.75E-15	0.234	0.231				*C7orf50*	Body
cg19651341	4	8,012,338	2.85E-05	0.234	0.194				*ABLIM2*	Body
cg27177983	4	25,033,126	0.005741	0.234	0.201				*LGI2*	TSS1500
cg17929951	20	44,746,681	0.003757	0.234	0.313				*CD40*	TSS1500
cg21871232	14	95,693,881	2.88E-05	0.234	0.157				*CLMN*	Body
cg23146833	3	171,175,866	5.99E-06	0.234	0.264				*TNIK*	Body
cg11616547	6	28,782,301	0.015819	0.234	0.253					

cg02407720	6	10,421,372	6.89E-05	0.234	0.267					
cg04314652	16	69,597,925	8.47E-08	0.234	0.216				*NFAT5*	TSS1500
cg04940329	17	39,093,054	8E-08	0.234	0.216				*KRT23*	5'UTR
cg16732175	19	49,250,180	0.002195	0.234	0.246				*IZUMO1*	TSS200
cg12235788	3	123,476,518	1.37E-11	0.234	0.180				*MYLK*	Body
cg17176619	13	99,218,657	3.23E-15	0.234	0.227				*STK24*	Body
cg19474865	11	83,733,268	0.002681	0.234	0.217				*DLG2*	Body
cg00456299	1	22,677,736	0.00239	0.234	0.187					
cg07828024	6	149,772,892	2.56E-16	0.234	0.222				*ZC3H12D*	Body
cg17351439	13	93,888,067	2.08E-05	0.234	0.253				*GPC6*	Body
cg07160932	16	29,888,733	1.94E-06	0.234	0.238				*SEZ6L2*	Body
cg08743812	5	71,014,736	0.020129	0.234	0.279				*CARTPT*	TSS1500
cg08844701	6	27,064,705	2.77E-05	0.234	0.181					
cg13234863	12	130,389,138	3.13E-05	0.234	0.202	5.38E-23	0.221		*TMEM132D*	TSS1500
cg06555468	7	2,094,790	1.02E-07	0.234	0.212				*MAD1L1*	Body
cg08387426	16	14,042,426	5.51E-14	0.234	0.226				*ERCC4*	3'UTR
cg16674484	5	149,887,497	3.41E-13	0.234	0.214				*NDST1*	TSS200
cg14870539	5	150,530,752	5.24E-09	0.234	0.224				*ANXA6*	5'UTR
cg03763518	1	150,245,044	9.12E-07	0.234	0.232				*C1orf54*	TSS200
cg05093169	11	71,955,401	0.005074	0.234	0.302				*PHOX2A*	TSS200
cg14115756	9	125,795,935	4.39E-14	0.234	0.232				*GPR21*	TSS1500
cg24645671	7	42,656,871	1.6E-15	0.234	0.212					
cg15849864	15	73,661,642	3.02E-07	0.233	0.242				*HCN4*	TSS200
cg01955203	8	124,515,502	4.77E-11	0.233	0.241				*FBXO32*	3'UTR
cg05685892	6	30,421,091	1E-16	0.233	0.212					
cg19002462	11	36,036,892	1.7E-10	0.233	0.227				*LDLRAD3*	Body
cg23058435	14	36,983,949	0.00013	0.233	0.260				*SFTA3*	TSS1500
cg02532824	6	108,444,810	4.52E-06	0.233	0.241			Yes		
cg13816321	9	973,198	1.56E-06	0.233	0.257					
cg00160583	1	32,167,512	3.74E-11	0.233	0.219				*COL16A1*	Body
cg26416466	15	66,546,708	5.54E-07	0.233	0.234	1.84E-19	0.173		*MEGF11*	TSS1500
cg02856768	12	94,385,202	3.7E-12	0.233	0.237			Yes		
cg22132508	3	138,658,878	0.009872	0.233	0.271					
cg05706696	X	129,254,556	0.000128	0.233	0.219					

cg02963248	2	66,809,780	1.18E-05	0.233	0.234					
cg10043155	6	25,218,855	5.95E-06	0.233	0.208					
cg02318527	19	41,318,069	4.74E-05	0.233	0.200					
cg06306751	X	154,251,005	6.1E-07	0.233	0.285	0.01106387	0.056		*F8*	TSS200
cg05635036	12	110,149,962	4.3E-09	0.233	0.227					
cg00379467	4	148,401,873	7.69E-18	0.233	0.230				*EDNRA*	TSS200
cg01965552	5	40,478,425	1.6E-07	0.233	0.172					
cg04856896	1	32,665,450	4E-16	0.233	0.212				*CCDC28B*	TSS1500
cg24472574	10	103,070,503	0.001084	0.233	0.212					
cg00960305	20	22,558,297	7.9E-05	0.233	0.198				*C20orf56*	Body
cg15992730	12	7,848,093	0.038327	0.233	0.276	0.00063329	0.114		*GDF3*	1stExon
cg26021627	8	53,478,706	0.003982	0.233	0.188	1.44E-25	0.189		*FAM150A*	TSS1500
cg27508055	2	19,286,187	7.62E-13	0.233	0.234					
cg05420057	17	36,721,297	2.06E-06	0.233	0.228				*SRCIN1*	Body
cg16334795	21	42,538,894	2.52E-06	0.233	0.189	0.00000182	0.151		*BACE2*	TSS1500
cg19805005	17	50,234,775	0.027063	0.233	0.196				*CA10*	Body
cg25116388	17	46,692,422	0.00051	0.233	0.270				*HOXB8*	TSS200
cg07655487	11	66,849,820	0.001154	0.233	0.177					
cg08669447	19	44,203,749	0.01563	0.233	0.113					
cg20504791	20	22,562,478	0.028852	0.233	0.239				*FOXA2*	3'UTR
cg02223323	X	20,134,399	4.02E-07	0.233	0.239				*MAP7D2*	Body
cg04967578	18	13,641,894	5.58E-06	0.233	0.199				*C18orf1*	Body
cg07480090	6	29,818,230	0.017427	0.233	0.263					
cg02652597	2	85,811,292	7.28E-07	0.233	0.250				*VAMP5*	TSS1500
cg17618327	7	2,143,942	0.000132	0.233	0.253				*MAD1L1*	Body
cg23677000	19	35,773,317	0.003466	0.233	0.239				*HAMP*	TSS200
cg01458510	2	109,752,622	6.47E-30	0.233	0.220				*SH3RF3*	Body
cg05413277	2	74,642,087	3.79E-06	0.233	0.196				*C2orf81*	Body
cg18366919	19	15,344,364	1.04E-09	0.233	0.227				*EPHX3*	TSS1500
cg24119607	5	139,047,739	1.61E-08	0.233	0.244				*CXXC5*	5'UTR
cg07211259	9	5,510,497	0.001792	0.233	0.150	8.63E-14	0.121		*PDCD1LG2*	TSS200
cg10950028	1	110,230,633	4.43E-08	0.233	0.215				*GSTM1*	Body
cg13969001	1	18,958,084	0.032267	0.233	0.278				*PAX7*	5'UTR
cg20462512	12	5,543,155	2.55E-07	0.233	0.226				*NTF3*	Body

cg04228935	21	36,399,258	0.011041	0.233	0.277				*RUNX1*	Body
cg17787134	17	47,073,522	0.040636	0.233	0.239				*IGF2BP1*	TSS1500
cg03900378	8	54,442,083	5.4E-07	0.233	0.214					
cg04290586	12	108,168,987	2.52E-05	0.233	0.181				*ASCL4*	1stExon
cg12324970	10	104,000,446	2.11E-07	0.233	0.218				*PITX3*	5'UTR
cg13084731	7	20,834,411	0.000296	0.233	0.179					
cg06959514	6	29,856,141	0.001373	0.233	0.193				*HLA-H*	Body
cg26848718	11	32,454,975	0.004022	0.233	0.201				*WT1*	Body
cg02490989	8	17,271,322	0.00069	0.233	0.190				*MTMR7*	TSS1500
cg04267526	10	109,674,297	1.95E-07	0.233	0.207					
cg06849477	22	45,072,724	2.16E-14	0.233	0.229	2.43E-39	0.158		*PRR5*	5'UTR
cg22768487	22	46,770,253	9.69E-08	0.233	0.217				*CELSR1*	Body
cg04481923	5	135,416,205	0.004422	0.233	0.259				*MIR886*	Body
cg15832662	11	63,448,437	0.000445	0.233	0.133				*RTN3*	TSS1500
cg19913563	6	30,720,261	0.002184	0.233	0.099					
cg20629315	2	43,685,377	5.13E-09	0.233	0.246				*THADA*	Body
cg22627406	19	10,958,968	9.44E-09	0.233	0.233				*C19orf38*	TSS200
cg03595533	6	28,784,788	0.036112	0.233	0.302					
cg05228186	11	83,393,344	0.00206	0.233	0.202				*DLG2*	1stExon
cg08638180	16	67,283,238	7.63E-07	0.233	0.133				*SLC9A5*	Body
cg09864245	11	33,746,426	0.000179	0.233	0.227				*CD59*	5'UTR
cg23669081	17	46,685,353	3.29E-07	0.233	0.235				*HOXB7*	Body
cg25383605	6	155,538,047	5.81E-08	0.233	0.184				*TIAM2*	Body
cg06844526	2	192,169,585	0.005526	0.233	0.146				*MYO1B*	Body
cg13480549	1	2,058,790	1.5E-08	0.233	0.199				*PRKCZ*	5'UTR
cg17918937	15	64,992,780	8.67E-16	0.233	0.240				*OAZ2*	Body
cg18576957	6	31,651,020	0.01845	0.233	0.283					
cg22632523	10	3,711,048	0.008189	0.233	0.106					
cg02567119	10	102,891,045	0.035974	0.233	0.160				*TLX1*	TSS200
cg00831237	12	50,344,289	1.73E-08	0.233	0.199				*AQP2*	TSS1500
cg01363734	12	117,482,953	1.49E-08	0.233	0.163				*TESC*	Body
cg10663316	12	124,702,334	0.014299	0.233	0.071					
cg23532454	14	75,910,026	2.61E-08	0.233	0.228				*JDP2*	Body
cg24161018	7	131,847,456	1.22E-13	0.233	0.225				*PLXNA4*	Body

cg04925129	3	149,247,133	5.04E-16	0.233	0.253				*WWTR1*	Body
cg06642219	1	5,586,147	0.002658	0.233	0.181					
cg17315500	12	103,359,572	0.010382	0.233	0.179					
cg00351537	19	7,580,275	8.15E-11	0.233	0.217				*ZNF358*	TSS1500
cg10500737	21	17,566,381	0.000416	0.233	0.171				*C21orf34*	Body
cg02039815	2	224,904,724	9.74E-06	0.233	0.148				*SERPINE2*	TSS1500
cg09473315	17	76,228,233	7.98E-06	0.233	0.236				*LOC283999*	Body
cg13035268	7	103,085,736	3.24E-06	0.233	0.217				*SLC26A5*	5'UTR
cg04709035	2	468,737	1.86E-06	0.233	0.229					
cg06496666	16	87,241,473	2.3E-05	0.233	0.180					
cg01320579	17	75,405,842	1.89E-08	0.233	0.236				*SEPT9*	Body
cg08640475	16	85,551,478	1.6E-63	0.233	0.228					
cg21249595	6	30,848,811	2.78E-12	0.233	0.215					
cg00733978	7	80,352,237	1.41E-05	0.233	0.163					
cg04007303	9	100,618,041	2.17E-05	0.233	0.208				*FOXE1*	3'UTR
cg05379634	19	1,856,726	4.7E-07	0.233	0.216				*KLF16*	Body
cg07781736	20	55,839,008	0.00657	0.233	0.227				*BMP7*	Body
cg11089489	14	37,124,313	2.91E-05	0.233	0.221					
cg14720024	5	115,697,696	2.18E-09	0.233	0.237					
cg17504999	4	134,072,723	0.036716	0.233	0.225				*PCDH10*	1stExon
cg07799299	8	131,344,855	2.57E-15	0.233	0.236				*ASAP1*	Body
cg08064488	21	38,068,043	0.0124	0.233	0.257					
cg10982443	14	20,903,611	9.61E-08	0.233	0.246				*KLHL33*	5'UTR
cg27441486	1	201,708,522	0.006894	0.233	0.156				*NAV1*	TSS1500
cg06715672	X	136,655,987	0.02594	0.233	0.219					
cg02953559	3	123,164,964	1.43E-14	0.233	0.237				*ADCY5*	Body
cg06640763	7	149,473,097	0.001553	0.233	0.179				*SSPO*	TSS200
cg13508369	9	129,884,026	7.49E-15	0.233	0.214				*ANGPTL2*	5'UTR
cg24611608	8	72,274,615	0.019192	0.233	0.314				*EYA1*	TSS200
cg00760014	1	19,267,827	0.006059	0.233	0.123				*IFFO2*	Body
cg06126019	11	9,253,566	3.62E-07	0.233	0.234				*DENND5A*	Body
cg04271801	6	26,272,516	4.37E-05	0.233	0.252				*HIST1H3G*	TSS1500
cg10732611	13	33,700,592	0.000646	0.233	0.214				*STARD13*	Body
cg12214003	2	235,859,792	1.8E-05	0.233	0.199				*SH3BP4*	TSS1500

cg01948226	6	32,829,208	0.005496	0.233	0.185					
cg10140794	5	36,066,972	0.000349	0.233	0.227				*UGT3A2*	1stExon
cg13694466	10	13,382,122	5.17E-12	0.233	0.235				*SEPHS1*	Body
cg18738190	10	73,740,291	2.42E-09	0.233	0.228				*CHST3*	5'UTR
cg07065111	2	5,831,213	0.002513	0.233	0.243					
cg19458787	5	173,116,776	0.002868	0.233	0.137					
cg21139046	3	14,339,700	4.63E-07	0.233	0.252					
cg07676145	10	73,506,646	1.29E-08	0.233	0.234				*CDH23*	Body
cg08369810	3	194,522,571	4.82E-05	0.233	0.214					
cg13300710	11	45,167,419	0.001009	0.233	0.260				*PRDM11*	Body
cg02766845	6	28,956,577	0.013008	0.233	0.223					
cg16967578	1	218,575,437	7.92E-05	0.233	0.234				*TGFB2*	Body
cg18273412	10	93,647,857	2.29E-07	0.233	0.207					
cg26224060	5	94,957,243	0.000263	0.233	0.195				*GPR150*	1stExon
cg08507136	8	28,515,641	4.42E-05	0.233	0.177					
cg15534755	11	117,069,859	1.01E-09	0.233	0.235				*TAGLN*	TSS200
cg23283423	17	821,546	6.6E-05	0.233	0.190				*NXN*	Body
cg02109162	15	99,646,443	0.000582	0.233	0.180				*SYNM*	Body
cg17284168	6	134,491,531	1.69E-19	0.233	0.233				*SGK1*	Body
cg17611046	6	5,672,954	1.72E-07	0.233	0.210				*FARS2*	Body
cg01687189	12	102,225,365	1.46E-12	0.233	0.214				*GNPTAB*	TSS1500
cg07069844	19	1,617,027	1.43E-08	0.233	0.293				*TCF3*	Body
cg15980076	10	94,822,747	2.71E-10	0.233	0.200				*CYP26C1*	Body
cg02805106	15	90,039,409	3.87E-07	0.233	0.239				*RHCG*	Body
cg07956751	7	150,211,939	0.000493	0.233	0.206				*GIMAP7*	TSS200
cg19333507	10	116,852,186	0.009832	0.233	0.099				*ATRNL1*	TSS1500
cg07302677	7	128,550,862	3.02E-18	0.233	0.221				*KCP*	TSS200
cg20813996	6	166,422,741	0.006227	0.233	0.257					
cg00062245	17	42,462,260	0.000519	0.233	0.241				*ITGA2B*	Body
cg04421348	10	82,299,737	2.13E-07	0.233	0.248				*SH2D4B*	TSS1500
cg13587180	4	42,403,248	4.62E-12	0.233	0.225				*SHISA3*	Body
cg24437535	1	150,121,441	0.004819	0.233	0.122				*PLEKHO1*	TSS1500
cg25890168	18	19,262,819	0.012944	0.233	0.212				*ABHD3*	Body
cg15666214	6	108,438,316	0.00089	0.233	0.245					

cg00532411	1	6,241,506	2.4E-07	0.233	0.193				*CHD5*	TSS1500
cg05134426	5	124,080,479	7.11E-05	0.233	0.267				*ZNF608*	1stExon
cg10486820	6	51,972,234	1.32E-09	0.233	0.210					
cg19416417	2	223,290,324	1.86E-14	0.233	0.220				*SGPP2*	Body
cg22900229	2	25,391,773	0.005231	0.233	0.180				*POMC*	TSS1500
cg02285386	22	35,695,157	4.92E-14	0.233	0.217				*TOM1*	TSS1500
cg20193363	2	947,589	4.95E-06	0.233	0.170				*SNTG2*	Body
cg16136262	1	169,429,604	8.38E-05	0.233	0.218					
cg17828689	3	136,539,113	0.016694	0.233	0.088				*TMEM22*	5'UTR
cg21890423	3	55,395,559	1.26E-06	0.233	0.245					
cg09736968	2	233,351,609	0.041997	0.233	0.179				*ECEL1*	5'UTR
cg04929703	1	91,300,390	0.00632	0.233	0.302					
cg12935170	7	23,508,189	0.041194	0.233	0.254				*IGF2BP3*	Body
cg12980549	7	158,084,782	1.17E-24	0.233	0.223				*PTPRN2*	Body
cg19781462	12	124,744,261	0.000583	0.233	0.272					
cg25614364	4	129,747,274	3.72E-14	0.233	0.234				*PHF17*	5'UTR
cg15679892	7	1,267,057	2.11E-10	0.233	0.235					
cg22510727	12	64,541,117	5.35E-10	0.233	0.229				*SRGAP1*	3'UTR
cg09484032	12	54,329,854	2.36E-09	0.233	0.226					
cg15095906	1	36,789,786	4.47E-05	0.233	0.194				*FAM176B*	TSS200
cg21706229	17	70,112,877	5.37E-06	0.233	0.212					
cg24927800	2	220,283,214	0.015595	0.233	0.144				*DES*	1stExon
cg11401568	17	19,648,157	0.000288	0.233	0.241				*ALDH3A1*	Body
cg25884399	9	139,237,461	0.043194	0.233	0.258				*GPSM1*	Body
cg09978996	10	70,321,668	2.72E-05	0.233	0.239				*TET1*	5'UTR
cg17744078	5	171,023,504	5.34E-07	0.233	0.220					
cg13903070	1	61,456,527	1.2E-06	0.232	0.211					
cg24082677	5	38,105,272	5.91E-09	0.232	0.221					
cg26819718	10	77,872,170	1.63E-11	0.232	0.197				*C10orf11*	Body
cg05532178	10	70,979,827	2.28E-07	0.232	0.253				*HKDC1*	TSS1500
cg09028008	1	19,615,080	2.74E-08	0.232	0.166				*AKR7A3*	1stExon
cg12883279	6	32,120,773	9.32E-05	0.232	0.270				*PPT2*	TSS1500
cg27441045	X	100,446,383	2.83E-13	0.232	0.236					
cg22160000	1	45,252,019	0.013797	0.232	0.271				*BEST4*	Body

cg07846737	19	5,068,701	1.94E-38	0.232	0.217				*KDM4B*	Body
cg24796272	8	99,954,679	1.33E-07	0.232	0.220					
cg04334011	14	21,561,590	7.57E-09	0.232	0.222				*ZNF219*	Body
cg04674956	8	65,499,048	0.000769	0.232	0.233					
cg09658497	7	2,847,517	0.001389	0.232	0.144				*GNA12*	Body
cg15607664	12	57,630,422	2.67E-13	0.232	0.219				*NDUFA4L2*	Body
cg23201938	5	134,451,535	1.21E-23	0.232	0.233					
cg04322202	4	66,533,472	0.036427	0.232	0.247				*EPHA5*	Body
cg04371726	2	63,285,645	0.001773	0.232	0.232					
cg02750065	12	117,155,382	1.66E-12	0.232	0.220				*C12orf49*	3'UTR
cg22218806	1	203,434,261	1.95E-17	0.232	0.228					
cg08318587	2	216,484,453	0.023839	0.232	0.157			Yes		
cg26924480	1	22,463,884	1.3E-07	0.232	0.168				*WNT4*	Body
cg04337653	6	100,905,587	0.01186	0.232	0.284				*SIM1*	Body
cg20460541	X	129,253,297	0.00296	0.232	0.180					
cg26809635	12	54,355,087	0.014817	0.232	0.270					
cg05762671	16	67,345,737	4.88E-06	0.232	0.207				*KCTD19*	Body
cg15416329	14	74,706,016	2.2E-08	0.232	0.219				*VSX2*	TSS200
cg17540545	19	35,630,355	1.98E-08	0.232	0.252				*FXYD1*	5'UTR
cg24979288	7	27,242,424	0.04127	0.232	0.312					
cg14567085	9	133,411,807	3.81E-05	0.232	0.172					
cg14906976	21	45,177,538	2.75E-10	0.232	0.239				*PDXK*	3'UTR
cg16552271	1	15,392,907	1.17E-08	0.232	0.212				*KIAA1026*	Body
cg20782117	7	95,865,631	3.05E-08	0.232	0.221				*SLC25A13*	Body
cg13333954	12	1,098,663	3.14E-07	0.232	0.249					
cg05656486	1	161,171,383	3.08E-05	0.232	0.236				*NDUFS2*	5'UTR
cg20209499	2	26,929,892	1.07E-45	0.232	0.233				*KCNK3*	Body
cg09285683	2	216,691,985	3.13E-05	0.232	0.202					
cg10879867	11	68,622,263	4.2E-06	0.232	0.205					
cg01896424	11	20,010,227	0.015775	0.232	0.169				*NAV2*	Body
cg16705245	2	180,725,637	1.14E-06	0.232	0.170				*ZNF385B*	5'UTR
cg25074023	2	179,316,491	0.004456	0.232	0.242				*PRKRA*	TSS1500
cg07730848	5	6,379,108	1.97E-05	0.232	0.197				*MED10*	TSS1500
cg00370047	11	1,481,436	1.38E-13	0.232	0.233				*BRSK2*	Body

cg19107291	15	100,537,865	2.11E-12	0.232	0.211				*ADAMTS17*	Body
cg27112146	2	148,641,953	5.95E-08	0.232	0.240				*ACVR2A*	Body
cg21244177	1	63,795,848	1.14E-07	0.232	0.228					
cg22855968	6	134,673,162	3.22E-06	0.232	0.227					
cg05223396	5	171,830,155	6.83E-23	0.232	0.217				*SH3PXD2B*	Body
cg25930591	7	96,627,602	0.000553	0.232	0.279				*DLX6AS*	Body
cg20627916	6	152,128,328	0.002697	0.232	0.204	0.03716632	0.059		*ESR1*	TSS1500
cg18326021	10	106,401,479	0.0347	0.232	0.218				*SORCS3*	1stExon
cg11412703	6	44,629,403	0.00022	0.232	0.278					
cg15990972	2	177,012,372	1.87E-08	0.232	0.176					
cg22294740	19	2,294,961	0.000571	0.232	0.295				*LINGO3*	5'UTR
cg20596162	15	45,408,861	7.01E-11	0.232	0.228				*DUOXA2*	Body
cg06403156	10	1,651,293	0.002344	0.232	0.220				*ADARB2*	Body
cg10949007	5	95,159,614	0.000706	0.232	0.194				*GLRX*	TSS1500
cg16207110	17	37,024,401	2.12E-05	0.232	0.252					
cg16354117	17	73,829,262	0.000184	0.232	0.242				*UNC13D*	Body
cg19897071	5	35,991,382	0.000195	0.232	0.230				*UGT3A1*	5'UTR
cg05774801	4	154,709,756	0.003193	0.232	0.220	2.63E-15	0.149		*SFRP2*	1stExon
cg06055229	5	124,077,966	0.000466	0.232	0.208				*ZNF608*	Body
cg11612266	2	947,167	6.14E-07	0.232	0.215				*SNTG2*	Body
cg15895339	10	20,210,338	3.9E-21	0.232	0.231				*PLXDC2*	Body
cg22723675	13	95,358,092	0.021804	0.232	0.208					
cg03882585	6	152,957,910	0.017706	0.232	0.189				*SYNE1*	5'UTR
cg08244262	21	42,797,939	0.003976	0.232	0.143				*MX1*	TSS200
cg00333483	17	79,504,180	0.033671	0.232	0.329					
cg06365303	20	2,801,844	0.016789	0.232	0.288					
cg01476003	16	71,460,486	0.001482	0.232	0.244					
cg04846781	6	32,077,084	5.83E-05	0.232	0.219				*TNXB*	5'UTR
cg09048500	20	21,085,073	1.89E-05	0.232	0.262					
cg11647108	10	81,967,516	3.34E-06	0.232	0.208					
cg00010445	19	14,089,630	4.72E-07	0.232	0.171				*RFX1*	Body
cg25353142	20	43,729,869	1.89E-07	0.232	0.222				*KCNS1*	TSS200
cg26517663	15	60,295,294	0.028597	0.232	0.290				*FOXB1*	TSS1500
cg00092518	13	50,070,550	0.000282	0.232	0.206				*PHF11*	Body

cg21895319	3	150,874,026	1.03E-20	0.232	0.221				*MED12L*	Body
cg25631352	2	70,995,527	0.018566	0.232	0.107				*ADD2*	TSS200
cg05965863	3	137,480,182	0.001293	0.232	0.185					
cg10169241	19	1,467,032	0.001143	0.232	0.249				*APC2*	Body
cg13531886	10	3,281,434	3.7E-15	0.232	0.221					
cg13833437	2	27,531,236	2.94E-09	0.232	0.237				*UCN*	TSS200
cg13935577	12	107,974,897	3.82E-18	0.232	0.229				*BTBD11*	Body
cg16072192	5	2,741,047	0.000897	0.232	0.223					
cg04205616	16	79,692,516	1.41E-11	0.232	0.217					
cg05209330	5	131,550,942	6.71E-09	0.232	0.242				*P4HA2*	Body
cg09744051	22	43,115,937	0.000283	0.232	0.233	6.01E-08	0.099		*A4GALT*	5'UTR
cg09329936	10	80,523,167	9.51E-07	0.232	0.220					
cg11029634	6	130,088,757	0.007799	0.232	0.123					
cg13748267	X	47,483,418	0.042153	0.232	0.033					
cg19429599	5	50,266,099	0.000682	0.232	0.263					
cg16524960	16	69,141,345	0.031627	0.232	0.227				*HAS3*	5'UTR
cg20843277	11	70,672,365	4.65E-21	0.232	0.193				*SHANK2*	Body
cg14754787	17	35,299,600	4.48E-07	0.232	0.213				*LHX1*	Body
cg23487201	20	57,090,122	2.52E-07	0.232	0.257				*APCDD1L*	TSS200
cg11691189	2	47,743,741	3.16E-09	0.232	0.234					
cg15621656	5	118,609,258	4.01E-05	0.232	0.234				*TNFAIP8*	Body
cg12951488	11	115,516,632	1.86E-08	0.232	0.209					
cg11806178	20	39,319,929	2.1E-08	0.232	0.217					
cg04691277	1	6,479,352	0.000489	0.232	0.158				*HES2*	Body
cg05131623	3	179,755,285	0.03646	0.232	0.328				*PEX5L*	TSS1500
cg09221867	4	134,071,663	0.008473	0.232	0.160				*PCDH10*	1stExon
cg13292202	5	112,771,367	0.0002	0.232	0.236				*TSSK1B*	TSS1500
cg03994820	3	350,682	0.005141	0.232	0.198				*CHL1*	5'UTR
cg04624228	8	38,411,598	0.033762	0.232	0.184					
cg11179759	15	89,947,346	0.014612	0.232	0.222					
cg21226965	6	169,852,806	0.000105	0.232	0.254					
cg27170985	14	37,124,063	4.02E-06	0.232	0.236					
cg16077055	2	106,428,750	1.67E-09	0.232	0.210				*NCK2*	5'UTR
cg17320707	10	119,307,931	0.016713	0.232	0.293				*EMX2*	3'UTR

cg24765394	9	129,088,647	7.87E-07	0.232	0.212				*FAM125B*	TSS1500
cg17683593	11	61,283,872	0.001285	0.232	0.258				*SYT7*	3'UTR
cg23914842	1	9,327,170	0.033943	0.232	0.252				*H6PD*	3'UTR
cg04307594	1	205,525,860	1.47E-11	0.232	0.231				*LOC284578*	TSS200
cg04319276	19	42,445,244	0.000475	0.232	0.255					
cg22948288	17	41,739,246	6.79E-08	0.232	0.211				*MEOX1*	TSS1500
cg17599241	5	82,858,676	1.09E-05	0.232	0.202				*VCAN*	Body
cg19265103	12	51,784,644	1.01E-15	0.232	0.204				*GALNT6*	5'UTR
cg19548652	14	34,419,316	0.000136	0.232	0.254				*EGLN3*	Body
cg22681784	4	57,687,546	5.96E-08	0.232	0.200	2.24E-13	0.123		*SPINK2*	Body
cg24929538	3	73,164,119	1.69E-09	0.232	0.249					
cg07167946	5	1,878,205	1.02E-10	0.232	0.213				*IRX4*	Body
cg15152362	20	31,173,500	0.003343	0.232	0.219					
cg18780257	3	192,126,023	0.015607	0.232	0.238				*FGF12*	5'UTR
cg16620390	12	125,670,262	2.19E-05	0.232	0.187					
cg00321007	17	15,340,112	4.59E-19	0.232	0.229				*CDRT4*	3'UTR
cg09771468	9	135,462,169	0.000229	0.232	0.232				*BARHL1*	Body
cg18006379	10	132,907,652	1.84E-09	0.232	0.220				*TCERG1L*	Body
cg18231025	1	59,573,471	3.65E-15	0.232	0.215					
cg21484547	13	28,319,144	5.43E-06	0.232	0.215					
cg25467634	10	49,684,658	3.34E-09	0.232	0.178				*ARHGAP22*	Body
cg05455409	2	202,897,006	0.005819	0.232	0.192					
cg09819083	1	110,673,179	0.001153	0.232	0.244					
cg17444738	4	85,417,969	7.81E-08	0.232	0.271				*NKX6-1*	Body
cg17838427	1	204,653,956	0.034195	0.232	0.067				*LRRN2*	5'UTR
cg14565725	1	119,532,044	2.13E-05	0.232	0.225				*TBX15*	1stExon
cg20119487	11	61,723,124	5.55E-05	0.232	0.184				*BEST1*	Body
cg24417499	1	33,351,653	0.00044	0.232	0.181	2.08E-11	0.133		*HPCA*	TSS1500
cg25162948	13	50,070,718	0.000108	0.232	0.198				*PHF11*	Body
cg26821745	2	177,023,183	0.002906	0.232	0.282					
cg07425204	17	66,511,183	8.97E-10	0.232	0.191				*PRKAR1A*	5'UTR
cg20813965	4	57,976,944	0.007261	0.232	0.248				*IGFBP7*	TSS1500
cg04110224	11	70,508,463	1.37E-05	0.232	0.231				*SHANK2*	Body
cg27518976	1	23,886,730	4.43E-07	0.232	0.260				*ID3*	TSS1500

cg01089249	2	171,676,553	0.000001	0.232	0.206				*GAD1*	Body
cg02614024	15	93,652,766	4.88E-07	0.232	0.223					
cg11803251	4	41,659,108	0.000752	0.232	0.199				*LIMCH1*	Body
cg12756396	1	50,886,969	2.83E-07	0.232	0.226				*DMRTA2*	Body
cg22256631	3	181,443,971	0.000283	0.232	0.212				*SOX2OT*	Body
cg00290892	3	87,842,398	0.032395	0.232	0.324					
cg04768501	3	9,956,823	0.035914	0.232	0.223				*IL17RE*	Body
cg14579864	9	79,632,225	0.027897	0.232	0.192					
cg16294013	1	66,258,022	0.004846	0.232	0.219				*PDE4B*	TSS200
cg22817352	1	204,653,629	0.001221	0.232	0.158				*LRRN2*	5'UTR
cg06467040	5	34,670,172	8.88E-12	0.232	0.227				*RAI14*	5'UTR
cg10238080	12	50,445,058	1.65E-06	0.232	0.259					
cg15172785	11	103,105,918	2.33E-13	0.232	0.255				*DYNC2H1*	Body
cg22484737	7	115,117,568	6.66E-06	0.232	0.235					
cg23847712	4	9,783,196	0.004885	0.232	0.311				*DRD5*	TSS200
cg00639615	15	89,786,496	0.002264	0.232	0.310				*FANCI*	TSS1500
cg10148473	12	75,601,824	0.010399	0.232	0.162				*KCNC2*	5'UTR
cg16764778	15	41,061,410	6.53E-06	0.232	0.218				*DNAJC17*	Body
cg05004665	1	20,800,052	1.53E-07	0.232	0.244					
cg18209323	7	92,672,878	3.38E-12	0.232	0.224					
cg18220920	12	115,126,061	0.006123	0.232	0.252					
cg19061690	17	46,080,304	1.36E-06	0.232	0.235					
cg21340621	12	122,020,166	0.008505	0.232	0.250				*KDM2B*	TSS1500
cg22400703	2	68,545,995	9.51E-09	0.232	0.244				*CNRIP1*	Body
cg26493188	16	2,975,209	1.39E-08	0.232	0.226				*FLYWCH1*	5'UTR
cg15770575	22	42,760,647	4.76E-10	0.232	0.228					
cg08302913	2	182,548,786	0.012813	0.232	0.206					
cg27161197	12	47,224,649	1.29E-07	0.232	0.200					
cg26673012	7	35,293,753	0.046137	0.232	0.262				*TBX20*	TSS200
cg14471364	2	157,177,936	0.009389	0.232	0.212					
cg04854328	5	92,914,828	6.15E-06	0.232	0.209				*FLJ42709*	Body
cg16404157	14	38,724,648	3.84E-05	0.232	0.241				*CLEC14A*	1stExon
cg19062108	12	57,630,834	5.54E-06	0.232	0.205				*NDUFA4L2*	Body
cg15115171	10	17,604,829	5.65E-06	0.232	0.196					

cg19071452	2	152,146,944	5.51E-08	0.232	0.199				*NMI*	TSS1500
cg22295864	11	74,233,472	4.29E-10	0.232	0.198					
cg19494811	6	34,356,253	6.72E-12	0.232	0.233				*NUDT3*	Body
cg08126875	6	125,743,207	8.37E-05	0.231	0.209					
cg20018782	14	23,317,496	1.69E-08	0.231	0.242					
cg20045394	17	36,184,576	1.49E-06	0.231	0.224					
cg20972453	13	20,805,460	5.58E-09	0.231	0.200				*GJB6*	5'UTR
cg23838005	17	74,868,604	7.69E-15	0.231	0.226				*MGAT5B*	Body
cg01154445	2	206,548,171	0.000989	0.231	0.225				*NRP2*	Body
cg01389386	16	70,471,055	1.51E-14	0.231	0.208				*ST3GAL2*	5'UTR
cg14394550	8	22,548,400	0.000305	0.231	0.184				*EGR3*	Body
cg15607672	14	57,277,227	1.23E-05	0.231	0.291	2.36E-17	0.172		*OTX2*	TSS200
cg15934958	3	37,212,084	2.65E-07	0.231	0.203				*LRRFIP2*	5'UTR
cg00651020	2	180,726,576	0.004383	0.231	0.099				*MIR1258*	TSS1500
cg13522075	9	99,481,726	3.61E-06	0.231	0.237					
cg13907146	3	50,243,565	6.5E-13	0.231	0.196				*SLC38A3*	5'UTR
cg07420274	2	171,676,306	2.02E-07	0.231	0.206				*GAD1*	Body
cg21900073	4	82,005,992	3.34E-07	0.231	0.221					
cg22844669	X	136,510,760	0.022336	0.231	0.249					
cg26427498	7	105,987,258	3.35E-07	0.231	0.238					
cg27577928	4	54,975,388	1.28E-08	0.231	0.190					
cg17557766	6	10,420,696	8.57E-08	0.231	0.237				*TFAP2A*	TSS1500
cg15092343	4	4,860,061	0.002137	0.231	0.258				*MSX1*	TSS1500
cg02806733	2	231,731,972	3.55E-06	0.231	0.267				*ITM2C*	Body
cg06049774	10	81,892,109	0.005551	0.231	0.217				*PLAC9*	TSS200
cg06012872	10	45,374,841	0.011722	0.231	0.147					
cg10567964	1	205,312,695	1.72E-05	0.231	0.194				*KLHDC8A*	Body
cg18020955	1	147,790,806	0.000246	0.231	0.255					
cg20904489	6	6,002,032	5.7E-05	0.231	0.189				*NRN1*	Body
cg26143719	22	37,584,227	3.63E-09	0.231	0.220	3.73E-19	0.154		*C1QTNF6*	1stExon
cg04864327	7	111,396,894	8.95E-09	0.231	0.241				*DOCK4*	Body
cg10473369	17	7,492,352	1.48E-24	0.231	0.224				*SOX15*	Body
cg12642289	11	78,948,859	8.06E-08	0.231	0.200				*ODZ4*	5'UTR
cg07281879	11	32,459,626	0.003806	0.231	0.295				*WIT1*	Body

cg07536847	1	18,957,232	0.003405	0.231	0.216	5.25E-13	0.174		*PAX7*	TSS1500
cg23316060	19	33,761,225	2.95E-07	0.231	0.214					
cg01775265	20	31,780,882	4.13E-23	0.231	0.212				*BASE*	TSS1500
cg24370475	11	69,518,003	0.000002	0.231	0.275				*FGF19*	Body
cg08144172	1	41,849,203	3.8E-05	0.231	0.157					
cg10859192	12	54,071,672	2.95E-06	0.231	0.230				*ATP5G2*	TSS1500
cg10934129	5	172,655,841	6.33E-06	0.231	0.192					
cg15084543	1	79,472,408	9.1E-09	0.231	0.237				*ELTD1*	5'UTR
cg16859420	8	41,219,116	0.003212	0.231	0.123					
cg03854796	11	31,819,162	0.00671	0.231	0.195				*PAX6*	Body
cg05967403	13	28,367,245	0.000214	0.231	0.230				*GSX1*	Body
cg06755992	3	123,753,198	0.003832	0.231	0.141					
cg07188896	10	59,546,843	7.61E-05	0.231	0.129					
cg16130857	10	102,900,262	1.67E-05	0.231	0.208					
cg12615235	16	1,969,016	1.29E-05	0.231	0.195				*HS3ST6*	TSS1500
cg20798740	8	37,560,840	0.000102	0.231	0.207					
cg14064694	6	26,273,556	0.038523	0.231	0.145				*HIST1H2BI*	1stExon
cg07286123	20	23,067,126	0.007735	0.231	0.182				*CD93*	TSS200
cg03729251	4	151,501,035	1.37E-11	0.231	0.233				*LRBA*	Body
cg18128969	21	22,369,908	1.3E-05	0.231	0.204				*NCAM2*	TSS1500
cg04861904	1	147,737,182	4.55E-05	0.231	0.221					
cg05506446	11	46,409,501	2.32E-08	0.231	0.240				*CHRM4*	TSS1500
cg26118435	2	161,350,826	0.00116	0.231	0.107				*RBMS1*	TSS1500
cg12595281	15	93,633,172	0.000972	0.231	0.218				*RGMA*	TSS1500
cg25595442	1	39,981,352	0.001422	0.231	0.264				*BMP8A*	Body
cg26777456	12	54,454,250	0.001747	0.231	0.167				*FLJ12825*	Body
cg05621843	9	97,806,397	1E-12	0.231	0.217				*C9orf3*	Body
cg07611887	16	21,831,372	8.32E-10	0.231	0.195				*RRN3P1*	TSS1500
cg21170796	1	119,528,164	0.004582	0.231	0.269				*TBX15*	5'UTR
cg23880533	12	124,896,079	0.000153	0.231	0.196				*NCOR2*	Body
cg03715305	19	5,894,715	2.72E-14	0.231	0.202				*NDUFA11*	3'UTR
cg05137975	6	99,786,314	2.98E-11	0.231	0.227				*C6orf168*	Body
cg15726807	19	41,857,198	0.000342	0.231	0.216				*TGFB1*	Body
cg16101346	1	186,650,479	8.68E-06	0.231	0.237				*PTGS2*	TSS1500

cg18533201	8	97,157,453	0.000518	0.231	0.316				*GDF6*	Body
cg16238815	14	36,741,794	0.010997	0.231	0.287					
cg03942871	7	102,574,105	4.75E-12	0.231	0.226				*LRRC17*	5'UTR
cg07817686	4	16,085,401	0.000872	0.231	0.272				*PROM1*	5'UTR
cg16479539	12	54,345,212	0.00523	0.231	0.246					
cg23371746	1	119,532,925	7.25E-06	0.231	0.222				*TBX15*	TSS1500
cg13358636	2	124,782,263	0.048064	0.231	0.141				*CNTNAP5*	TSS1500
cg09184832	6	79,620,586	2.9E-10	0.231	0.237					
cg12171761	8	61,910,949	2.68E-18	0.231	0.228					
cg11795276	12	54,454,252	0.000149	0.231	0.155				*FLJ12825*	Body
cg27138293	17	79,503,701	1.34E-12	0.231	0.210				*FSCN2*	Body
cg03356689	11	128,586,365	0.049483	0.231	0.258				*FLI1*	Body
cg24251035	7	138,721,065	0.005493	0.231	0.129	4.3E-23	0.171		*ZC3HAV1L*	TSS1500
cg05197667	12	64,383,297	0.000805	0.231	0.275				*SRGAP1*	Body
cg06827038	17	48,912,952	1.47E-13	0.231	0.216	2.49E-23	0.159		*WFIKKN2*	1stExon
cg10641258	21	43,484,279	5.7E-14	0.231	0.200					
cg18166150	10	3,169,037	0.000503	0.231	0.129				*PFKP*	Body
cg09579310	6	10,395,947	9.56E-05	0.231	0.240					
cg09990194	14	76,289,228	2.38E-07	0.231	0.235				*TTLL5*	Body
cg10632969	11	134,023,334	1.06E-09	0.231	0.237				*NCAPD3*	Body
cg14886850	14	75,122,289	4.06E-13	0.231	0.227					
cg20735682	6	168,665,386	2.79E-13	0.231	0.202					
cg11234688	2	1,609,660	1.96E-07	0.231	0.218					
cg17438030	5	72,526,379	3.24E-06	0.231	0.198					
cg22057234	20	34,206,710	9.67E-06	0.231	0.168				*SPAG4*	Body
cg12917718	13	70,682,019	0.000936	0.231	0.182				*KLHL1*	1stExon
cg19025525	1	228,566,824	0.008409	0.231	0.218					
cg23248357	5	2,755,376	1.12E-06	0.231	0.219				*C5orf38*	3'UTR
cg26380291	10	114,787,843	5.46E-07	0.231	0.221				*TCF7L2*	Body
cg05250797	16	70,222,502	0.000142	0.231	0.159					
cg07589342	1	212,392,508	1.1E-07	0.231	0.234					
cg02588107	1	207,669,716	0.0266	0.231	0.236				*CR1*	1stExon
cg04376709	2	106,055,450	3.07E-22	0.231	0.222				*FHL2*	TSS1500
cg25018049	1	201,472,826	1.11E-06	0.231	0.239				*CSRP1*	5'UTR

cg02430797	10	50,969,719	0.038931	0.231	0.230				*OGDHL*	5'UTR
cg18991169	11	31,843,388	0.001705	0.231	0.231					
cg06151243	17	5,000,872	0.008087	0.231	0.139					
cg09814029	1	39,212,920	0.004286	0.231	0.185					
cg24425171	2	25,391,711	0.022571	0.231	0.134				*POMC*	TSS200
cg13510429	X	153,094,195	1.6E-06	0.231	0.176				*PDZD4*	Body
cg14921884	8	54,442,043	4.94E-08	0.231	0.225					
cg22519545	5	50,263,682	0.018384	0.231	0.215					
cg07419144	18	55,100,658	0.010032	0.231	0.242					
cg07942990	20	23,432,977	7.38E-08	0.231	0.256				*CST11*	Body
cg09549240	15	75,250,560	0.024742	0.231	0.257				*RPP25*	TSS1500
cg11432962	13	95,354,741	0.00033	0.231	0.243					
cg04942516	2	106,055,458	1.13E-25	0.231	0.224				*FHL2*	TSS1500
cg21690921	1	240,562,805	6.96E-10	0.231	0.238				*FMN2*	Body
cg13414629	9	132,935,646	0.04908	0.231	0.161				*FREQ*	Body
cg04569897	6	45,609,407	0.011562	0.231	0.108					
cg14557487	2	162,279,686	0.00013	0.231	0.209				*TBR1*	Body
cg20737812	15	86,336,631	1.17E-16	0.231	0.217				*KLHL25*	5'UTR
cg00505001	13	36,049,807	0.039379	0.231	0.225				*MAB21L1*	1stExon
cg01511806	16	85,157,939	7.74E-12	0.231	0.225					
cg04567731	17	59,532,519	4.37E-05	0.231	0.200				*TBX4*	TSS1500
cg07501919	2	219,857,231	1.09E-05	0.231	0.223				*CRYBA2*	Body
cg14129775	4	107,957,941	0.000272	0.231	0.209				*DKK2*	TSS1500
cg21390363	17	18,171,734	2.87E-16	0.231	0.242					
cg07476508	11	111,783,355	0.00171	0.231	0.304				*CRYAB*	TSS1500
cg14328415	9	139,847,314	1.4E-99	0.231	0.232				*LCN12*	Body
cg11982546	8	56,832,362	2.11E-28	0.231	0.230				*LYN*	5'UTR
cg18282791	6	26,332,501	0.016674	0.231	0.187					
cg22637890	11	95,954,652	2.38E-08	0.231	0.211				*MAML2*	Body
cg26756396	19	7,459,296	0.004357	0.231	0.246				*ARHGEF18*	TSS1500
cg07072704	22	45,899,736	2.56E-13	0.231	0.228				*FBLN1*	Body
cg11591485	3	124,839,640	1.36E-15	0.231	0.218				*SLC12A8*	Body
cg16072304	5	153,859,646	0.013207	0.231	0.257					
cg19797376	1	47,695,475	0.002564	0.231	0.168	2.56E-20	0.205		*TAL1*	TSS200

cg08390877	2	119,615,998	0.008185	0.231	0.310					
cg15013617	20	2,795,378	8.77E-08	0.231	0.205				*C20orf141*	TSS1500
cg22462010	8	124,173,385	1.74E-06	0.231	0.216					
cg22705929	11	57,417,974	1.44E-05	0.231	0.163	1.62E-09	0.147		*YPEL4*	TSS1500
cg24183566	7	101,629,430	5.49E-13	0.231	0.241				*CUX1*	Body
cg02973171	11	82,443,614	7.17E-07	0.231	0.226				*FAM181B*	1stExon
cg18837542	4	139,610,152	9.22E-14	0.231	0.218					
cg10472919	5	135,266,805	3.04E-07	0.231	0.211				*FBXL21*	5'UTR
cg18733548	17	77,766,817	1.05E-06	0.231	0.219					
cg23967293	6	37,550,213	1.33E-11	0.231	0.218					
cg14381712	1	12,546,623	0.001522	0.231	0.138				*VPS13D*	Body
cg26414521	12	132,899,974	1.62E-05	0.231	0.270				*GALNT9*	Body
cg03601920	1	17,445,310	1.29E-07	0.231	0.217				*PADI2*	Body
cg04707332	1	119,528,018	9.72E-23	0.231	0.220				*TBX15*	5'UTR
cg19572487	17	38,476,024	0.000148	0.231	0.176				*RARA*	5'UTR
cg07986058	15	26,094,704	0.000465	0.231	0.139				*ATP10A*	Body
cg10230190	1	85,405,081	5.7E-12	0.231	0.235				*MCOLN2*	Body
cg20480642	8	52,722,185	1.02E-09	0.231	0.216				*PXDNL*	TSS200
cg07119973	17	43,109,221	3.72E-09	0.231	0.237				*DCAKD*	Body
cg15448210	6	33,401,514	7.21E-21	0.231	0.230				*SYNGAP1*	Body
cg17085688	7	93,540,134	0.033512	0.231	0.113				*GNGT1*	Body
cg22019158	1	27,926,683	6.64E-09	0.231	0.229				*AHDC1*	5'UTR
cg06758644	1	12,533,120	2.35E-09	0.231	0.210				*VPS13D*	Body
cg06770429	6	143,206,075	1.42E-21	0.231	0.210				*HIVEP2*	5'UTR
cg07747133	3	181,428,307	3.95E-06	0.231	0.218				*SOX2OT*	Body
cg22012659	1	112,506,949	9.94E-12	0.231	0.210				*KCND3*	Body
cg10996327	10	94,180,743	0.00091	0.231	0.205					
cg07078225	12	52,652,377	0.016773	0.231	0.151					
cg08757037	7	75,933,294	0.013807	0.231	0.313				*HSPB1*	Body
cg21525032	17	80,372,493	0.003099	0.231	0.228				*C17orf101*	Body
cg11744777	6	27,799,370	0.002495	0.231	0.190				*HIST1H4K*	TSS200
cg14041778	1	33,938,210	0.02355	0.231	0.189				*ZSCAN20*	TSS200
cg24396358	6	32,977,400	0.000463	0.231	0.249				*HLA-DOA*	TSS200
cg14990644	5	112,771,691	0.000196	0.231	0.226				*TSSK1B*	TSS1500

cg22932427	1	155,615,664	2.35E-09	0.231	0.246					
cg07267294	5	58,893,722	3.57E-19	0.231	0.221				*PDE4D*	Body
cg22668218	6	161,100,017	0.00028	0.231	0.193					
cg27532722	22	24,115,568	2.32E-08	0.231	0.179	3.79E-14	0.134		*MMP11*	Body
cg21620540	11	12,031,314	1.43E-05	0.231	0.150				*DKK3*	TSS1500
cg26014634	17	70,113,514	5.63E-05	0.231	0.230					
cg26444951	4	13,530,269	0.035735	0.231	0.276					
cg11986760	2	112,420,098	6.3E-10	0.231	0.242					
cg25726080	1	167,682,729	2.06E-05	0.231	0.200					
cg08911368	8	11,471,085	0.002134	0.231	0.163					
cg18087477	1	115,397,597	1.55E-08	0.231	0.215	8.02E-13	0.149		*SYCP1*	1stExon
cg03096746	6	29,795,436	1.27E-06	0.231	0.232				*HLA-G*	5'UTR
cg07730329	5	140,810,137	0.000315	0.230	0.249	1.31E-19	0.188		*PCDHGA4*	Body
cg23930313	12	25,056,083	0.023458	0.230	0.275				*BCAT1*	Body
cg24837370	2	5,833,774	0.011304	0.230	0.226				*SOX11*	1stExon
cg26678138	4	184,642,625	5.21E-05	0.230	0.213					
cg06515144	1	179,545,091	0.049747	0.230	0.252				*NPHS2*	TSS200
cg19821297	19	12,890,029	1.07E-08	0.230	0.217					
cg20086517	8	30,588,468	2.08E-54	0.230	0.221					
cg03388193	10	100,996,070	0.030347	0.230	0.241	6.22E-11	0.170		*HPSE2*	TSS1500
cg08795320	16	66,982,803	7.88E-11	0.230	0.231					
cg11638298	4	188,917,173	4.79E-09	0.230	0.225				*ZFP42*	5'UTR
cg14039246	1	53,365,446	4.72E-08	0.230	0.201				*ECHDC2*	Body
cg26393015	7	128,809,301	9.44E-05	0.230	0.202					
cg01640808	X	21,674,815	1.43E-06	0.230	0.203				*KLHL34*	1stExon
cg13931292	1	114,698,448	0.000447	0.230	0.239					
cg25446789	2	25,810,393	8.5E-08	0.230	0.234				*DTNB*	Body
cg05293365	15	77,287,338	8.54E-14	0.230	0.227				*PSTPIP1*	TSS200
cg20080624	7	96,651,111	4.58E-06	0.230	0.215	0.02595709	0.053		*DLX5*	Body
cg05000994	7	27,131,807	6.18E-08	0.230	0.226					
cg07307078	18	12,307,630	0.000194	0.230	0.220	3.77E-07	0.087		*TUBB6*	TSS1500
cg10885779	11	34,331,908	7.13E-10	0.230	0.232				*ABTB2*	Body
cg15904283	10	23,484,867	0.010411	0.230	0.322					
cg27297441	10	134,918,671	0.000588	0.230	0.227				*GPR123*	Body

cg08281371	17	76,662,056	1.04E-06	0.230	0.219					
cg18912855	14	105,830,859	0.049432	0.230	0.113				*PACS2*	Body
cg18539461	1	201,709,675	0.003683	0.230	0.195				*NAV1*	Body
cg03760457	15	70,326,230	1.09E-10	0.230	0.209					
cg09015433	8	57,359,970	0.002261	0.230	0.224				*PENK*	TSS1500
cg10550882	20	1,825,647	0.003552	0.230	0.181					
cg13496662	19	45,416,369	1.01E-10	0.230	0.243					
cg18603577	12	123,900,102	1.89E-07	0.230	0.236				*RILPL2*	3'UTR
cg23690904	4	4,387,697	0.003148	0.230	0.242				*D4S234E*	TSS1500
cg03421358	17	17,479,100	9.49E-06	0.230	0.285				*PEMT*	Body
cg16683770	20	21,000,992	0.01224	0.230	0.263					
cg03612413	21	38,066,918	0.000963	0.230	0.280					
cg07542748	7	4,885,643	2.74E-05	0.230	0.196				*RADIL*	Body
cg08208460	19	39,687,865	0.038443	0.230	0.322				*NCCRP1*	1stExon
cg09556527	1	1,239,259	1.22E-17	0.230	0.230				*ACAP3*	Body
cg11585893	12	54,447,430	6.12E-07	0.230	0.198				*HOXC4*	5'UTR
cg24438217	4	39,980,114	2.72E-14	0.230	0.228				*PDS5A*	TSS1500
cg26339162	14	68,509,470	0.000192	0.230	0.204				*RAD51L1*	Body
cg13861244	1	18,214,635	8.66E-07	0.230	0.191					
cg23514016	5	78,407,564	1.32E-06	0.230	0.223				*BHMT*	TSS200
cg09396295	17	66,196,681	0.000515	0.230	0.231					
cg14533390	3	65,342,843	7.45E-09	0.230	0.226				*MAGI1*	Body
cg21397588	1	203,598,954	0.008973	0.230	0.187				*ATP2B4*	5'UTR
cg10477905	2	177,039,295	0.001665	0.230	0.221					
cg21996351	1	205,326,059	4.59E-42	0.230	0.221				*KLHDC8A*	TSS200
cg11336311	1	23,887,324	3.77E-20	0.230	0.236			Yes	*ID3*	TSS1500
cg25082959	13	103,046,930	0.048582	0.230	0.176				*FGF14*	Body
cg27387239	1	218,667,548	2.04E-08	0.230	0.225					
cg05810170	4	1,049,609	0.003086	0.230	0.169					
cg23901857	2	176,993,221	7.37E-06	0.230	0.196				*HOXD8*	TSS1500
cg24876187	6	31,126,871	0.001202	0.230	0.185				*CCHCR1*	TSS1500
cg07888912	19	11,305,913	4.12E-05	0.230	0.189				*KANK2*	5'UTR
cg16822407	1	15,392,852	2.79E-05	0.230	0.233				*KIAA1026*	Body
cg24452591	12	47,224,823	0.007596	0.230	0.112					

cg17421991	7	156,795,356	0.045478	0.230	0.204					
cg15554023	1	144,008,937	3.38E-09	0.230	0.239					
cg23206209	15	53,079,494	0.013831	0.230	0.153				*ONECUT1*	Body
cg26654798	15	83,953,864	4.2E-07	0.230	0.223				*BNC1*	TSS1500
cg01517500	2	10,637,974	0.000114	0.230	0.219					
cg08696165	2	45,172,169	0.002597	0.230	0.293				*SIX3*	3'UTR
cg14780276	4	148,743,200	1.29E-09	0.230	0.213				*ARHGAP10*	Body
cg23754924	15	93,616,943	4.2E-07	0.230	0.191				*RGMA*	Body
cg02633371	17	35,298,618	1.11E-05	0.230	0.196				*LHX1*	Body
cg09024228	1	116,707,400	6.12E-09	0.230	0.250					
cg15591803	1	111,733,668	5.99E-08	0.230	0.191				*DENND2D*	Body
cg21929183	5	140,807,225	0.024464	0.230	0.291				*PCDHGA4*	Body
cg24693341	4	41,746,947	0.03673	0.230	0.248				*PHOX2B*	3'UTR
cg06373648	6	33,401,536	2.21E-15	0.230	0.226				*SYNGAP1*	Body
cg01558645	4	71,587,904	4.17E-13	0.230	0.218				*RUFY3*	1stExon
cg27160952	21	44,089,142	0.000237	0.230	0.249				*PDE9A*	Body
cg20699141	3	182,698,915	0.000182	0.230	0.142				*DCUN1D1*	TSS1500
cg12165546	8	23,566,799	0.001395	0.230	0.225					
cg14085358	2	176,949,017	0.009432	0.230	0.237				*EVX2*	TSS1500
cg20023120	11	69,830,611	0.004331	0.230	0.220					
cg23676151	6	108,491,372	0.00044	0.230	0.268				*NR2E1*	Body
cg24139639	5	3,605,884	0.002821	0.230	0.291					
cg26607897	1	167,510,839	0.000689	0.230	0.242				*CREG1*	3'UTR
cg11441379	12	63,026,424	0.022706	0.230	0.180					
cg12403137	11	89,867,809	0.003942	0.230	0.277				*NAALAD2*	TSS200
cg10603156	10	13,732,625	1.02E-10	0.230	0.240				*FRMD4A*	Body
cg14554869	5	74,231,171	0.007545	0.230	0.276					
cg18390991	2	217,672,659	6.95E-16	0.230	0.241					
cg25349276	4	41,614,591	1.67E-09	0.230	0.205				*LIMCH1*	Body
cg10056203	10	13,571,816	4.31E-11	0.230	0.200					
cg13523649	20	37,230,741	5.99E-12	0.230	0.234				*C20orf95*	Body
cg25406779	19	40,732,712	0.006092	0.230	0.120				*CNTD2*	TSS200
cg25647583	15	91,427,184	2.32E-14	0.230	0.235				*FES*	TSS1500
cg02538248	16	75,045,643	1.2E-14	0.230	0.209				*ZNRF1*	Body

cg11070193	1	26,527,870	8.76E-08	0.230	0.225				*CATSPER4*	Body
cg09319061	12	109,128,198	1.32E-07	0.230	0.274					
cg09690483	2	202,992,863	4.04E-08	0.230	0.254					
cg21045608	11	8,615,456	0.016523	0.230	0.209				*STK33*	1stExon
cg01528028	13	100,649,800	0.000321	0.230	0.241					
cg11755796	20	19,192,616	0.015644	0.230	0.125				*SLC24A3*	TSS1500
cg00991994	3	99,639,553	7.4E-15	0.230	0.240				*C3orf26*	Body
cg10227187	16	2,030,509	0.024929	0.230	0.305				*NOXO1*	Body
cg17427305	15	57,881,059	2.81E-20	0.230	0.210					
cg21911276	18	55,289,797	3.67E-05	0.230	0.192				*NARS*	TSS1500
cg25070373	2	200,966,980	2.84E-09	0.230	0.203					
cg01091951	5	42,993,450	0.012062	0.230	0.232					
cg01638185	17	64,530,027	1.12E-09	0.230	0.231				*PRKCA*	Body
cg09888468	15	81,410,853	0.004087	0.230	0.208					
cg07123069	12	54,367,250	0.000154	0.230	0.189	7.14E-13	0.169		*HOXC11*	1stExon
cg07910724	3	117,604,411	0.034147	0.230	0.219					
cg14875081	2	96,814,660	6.62E-08	0.230	0.218					
cg17279839	7	150,038,598	1.96E-05	0.230	0.231	1.19E-25	0.200		*RARRES2*	5'UTR
cg17912835	2	105,478,804	2.61E-07	0.230	0.224					
cg26512226	2	220,282,165	5.21E-13	0.230	0.213				*DES*	TSS1500
cg27534599	22	17,680,477	1.45E-05	0.230	0.200				*CECR1*	Body
cg02833172	16	84,774,950	1.35E-05	0.230	0.222				*USP10*	Body
cg11050612	3	149,374,066	0.017991	0.230	0.168				*WWTR1*	Body
cg16002660	17	2,310,278	2.06E-05	0.230	0.216				*LOC284009*	Body
cg02273889	5	123,941,379	3.87E-08	0.230	0.241					
cg08917933	1	19,615,303	7.98E-06	0.230	0.231				*AKR7A3*	TSS200
cg09426383	7	26,897,182	3.66E-08	0.230	0.196				*SKAP2*	Body
cg16646024	5	175,560,307	0.003903	0.230	0.211					
cg19572014	2	24,232,681	9.78E-05	0.230	0.165				*MFSD2B*	TSS1500
cg23447239	7	157,697,628	7.98E-12	0.230	0.221				*PTPRN2*	Body
cg26221992	6	74,024,572	0.018944	0.230	0.243					
cg09612786	7	150,715,214	0.004118	0.230	0.266				*ATG9B*	Body
cg15511381	18	35,147,532	0.023248	0.230	0.163					
cg26606286	4	174,440,471	0.001272	0.230	0.225					

cg15732768	7	96,651,537	1.36E-06	0.230	0.224				*DLX5*	Body
cg24335895	19	36,643,771	1.46E-07	0.230	0.244				*COX7A1*	TSS200
cg04518808	19	54,384,822	0.000934	0.230	0.277				*PRKCG*	TSS1500
cg05322916	6	36,880,416	3.36E-06	0.230	0.198				*C6orf89*	Body
cg24375218	18	46,461,399	3.5E-10	0.230	0.221				*SMAD7*	Body
cg26364899	3	12,329,070	0.029074	0.230	0.384				*PPARG*	TSS1500
cg02137183	7	141,250,424	4.35E-19	0.230	0.227				*AGK*	TSS1500
cg00731232	9	18,437,917	2.5E-14	0.230	0.237					
cg11577716	20	20,258,001	0.001175	0.230	0.175				*C20orf26*	Body
cg13484553	9	137,990,330	0.012238	0.230	0.173				*OLFM1*	Body
cg18803104	6	28,956,324	0.000161	0.230	0.221					
cg21968515	11	62,694,099	2.7E-05	0.230	0.212					
cg00912580	2	135,169,533	3.86E-11	0.230	0.216				*MGAT5*	Body
cg26129545	5	149,792,330	0.039179	0.230	0.058				*CD74*	1stExon
cg02249657	5	140,873,765	1.17E-10	0.230	0.240				*PCDHGA4*	Body
cg18728025	4	121,991,645	1.04E-08	0.230	0.230				*C4orf31*	5'UTR
cg25747427	15	89,561,135	0.007663	0.230	0.198					
cg06172942	3	50,378,529	2.02E-06	0.230	0.232				*RASSF1*	TSS200
cg13342435	11	30,605,787	0.021927	0.230	0.236				*MPPED2*	5'UTR
cg14787269	19	46,380,394	1.1E-13	0.230	0.219					
cg04182378	X	21,958,416	0.004743	0.230	0.280				*SMS*	TSS1500
cg16345647	21	38,378,406	0.000244	0.230	0.202			Yes	*DSCR6*	TSS1500
cg01920232	12	106,976,668	0.00143	0.230	0.199	2.18E-18	0.151		*RFX4*	TSS1500
cg11241627	7	19,185,165	0.008192	0.230	0.269	0.0000164	0.068		*FERD3L*	TSS200
cg27485646	X	38,080,412	0.016066	0.230	0.203				*SRPX*	TSS1500
cg03276408	13	112,712,475	0.000149	0.230	0.268					
cg00374016	15	24,722,944	0.00012	0.230	0.238					
cg19012170	15	65,715,664	0.001005	0.230	0.280				*IGDCC4*	TSS1500
cg21405761	8	11,304,273	4.66E-15	0.230	0.219			Yes	*FAM167A*	5'UTR
cg27624162	2	111,876,008	3.76E-06	0.230	0.226					
cg24234899	10	118,922,493	0.034828	0.230	0.174					
cg13572309	12	16,758,267	0.001813	0.230	0.207				*LMO3*	5'UTR
cg26429783	1	245,753,302	1.03E-14	0.230	0.219				*KIF26B*	Body
cg00920139	8	22,455,473	4.85E-12	0.230	0.236				*PDLIM2*	3'UTR

cg06663149	4	41,457,558	4.92E-05	0.230	0.250				*LIMCH1*	Body
cg23698476	19	4,607,318	0.008406	0.230	0.156					
cg26734335	16	87,166,401	0.000117	0.230	0.251					
cg00854172	2	23,748,104	8.57E-14	0.230	0.212				*KLHL29*	5'UTR
cg06214007	1	89,829,308	0.000588	0.230	0.238	2.65E-17	0.214		*GBP6*	TSS200
cg00797651	1	207,494,336	1.52E-05	0.230	0.208				*CD55*	TSS1500
cg10227830	4	144,332,828	4.58E-06	0.230	0.235				*GAB1*	Body
cg01138867	5	160,975,792	0.027115	0.230	0.248				*GABRB2*	TSS1500
cg04296203	2	242,985,752	0.000411	0.230	0.232					
cg15315056	3	184,504,579	5.78E-06	0.229	0.213					
cg04581214	19	1,267,741	7.27E-12	0.229	0.217				*C19orf23*	Body
cg06087019	3	27,770,182	0.00212	0.229	0.208					
cg17498296	10	124,907,540	0.043032	0.229	0.159				*HMX2*	TSS200
cg00579520	12	128,752,586	4.76E-06	0.229	0.190				*TMEM132C*	Body
cg04755365	14	100,625,253	0.00219	0.229	0.222				*DEGS2*	Body
cg21735668	15	67,072,353	2.68E-13	0.229	0.224				*SMAD6*	Body
cg21052608	5	81,652,962	0.004373	0.229	0.159					
cg25514273	13	70,681,804	0.001301	0.229	0.207				*KLHL1*	1stExon
cg09656020	15	33,009,409	0.000546	0.229	0.245				*GREM1*	TSS1500
cg12345778	12	49,364,094	7.64E-07	0.229	0.181				*WNT10B*	Body
cg12560421	7	2,656,527	3.58E-19	0.229	0.233					
cg12831863	6	28,457,210	2.57E-06	0.229	0.219					
cg00849990	8	86,090,773	4.73E-07	0.229	0.205				*E2F5*	Body
cg13575422	9	91,608,364	5.27E-09	0.229	0.254				*C9orf47*	3'UTR
cg23375552	6	10,426,310	4.42E-08	0.229	0.263					
cg03964111	7	150,498,493	1.12E-10	0.229	0.234	1.38E-16	0.127		*TMEM176B*	TSS200
cg15239238	11	16,629,389	0.001193	0.229	0.126					
cg23679982	17	1,686,737	0.000909	0.229	0.137				*SMYD4*	Body
cg00473179	15	29,033,881	3.07E-05	0.229	0.265					
cg02285920	19	17,958,956	2.11E-06	0.229	0.191				*JAK3*	TSS200
cg06226156	2	74,726,478	0.00115	0.229	0.236				*LBX2*	Body
cg10136560	4	140,737,518	2.42E-07	0.229	0.241				*MAML3*	Body
cg14614754	1	120,289,923	4.92E-13	0.229	0.222					
cg15742751	1	155,213,747	0.001894	0.229	0.246				*GBA*	5'UTR

cg14531834	9	87,655,988	0.00472	0.229	0.297					
cg18814699	7	36,342,851	1.02E-09	0.229	0.163					
cg26297344	1	111,151,163	8.12E-08	0.229	0.226					
cg23123731	10	80,161,611	1.07E-06	0.229	0.217					
cg17620199	20	21,685,925	6.72E-06	0.229	0.269				*PAX1*	TSS1500
cg16397884	5	132,082,837	0.003505	0.229	0.193				*CCNI2*	TSS1500
cg16653620	2	162,284,135	0.015442	0.229	0.246					
cg23990272	1	25,906,648	2.72E-17	0.229	0.223					
cg05210258	13	102,569,538	0.041563	0.229	0.259				*FGF14*	Body
cg26685246	6	32,805,692	4.96E-06	0.229	0.162				*TAP2*	Body
cg12978433	20	52,789,956	0.028814	0.229	0.129				*CYP24A1*	1stExon
cg00031162	17	7,453,377	6.11E-12	0.229	0.218	4.46E-14	0.136		*TNFSF12*	Body
cg09978860	4	56,023,921	7.35E-05	0.229	0.241					
cg09553982	10	77,875,676	0.034388	0.229	0.095				*C10orf11*	Body
cg14486905	2	171,677,602	0.00024	0.229	0.223				*GAD1*	Body
cg12215739	2	177,014,966	5.62E-08	0.229	0.202				*HOXD4*	TSS1500
cg25397636	7	1,896,220	3.06E-24	0.229	0.208				*MAD1L1*	Body
cg01033938	11	130,298,880	0.00734	0.229	0.269	5.69E-21	0.192		*ADAMTS8*	TSS1500
cg03636532	18	55,107,236	0.009519	0.229	0.239				*ONECUT2*	Body
cg04007726	10	80,981,129	0.000418	0.229	0.260				*ZMIZ1*	Body
cg04993276	1	65,730,339	6.02E-05	0.229	0.209				*DNAJC6*	TSS200
cg09503045	19	55,592,453	0.000172	0.229	0.223				*EPS8L1*	Body
cg25673591	17	39,945,814	5.51E-07	0.229	0.225					
cg17771050	10	47,083,342	0.000434	0.229	0.107				*PPYR1*	TSS200
cg12195742	18	72,123,456	1.64E-08	0.229	0.179				*FAM69C*	Body
cg12229555	17	80,260,329	2.84E-09	0.229	0.203					
cg17419888	2	23,840,555	5.23E-06	0.229	0.227				*KLHL29*	Body
cg26181727	3	14,581,959	4.31E-06	0.229	0.254				*GRIP2*	Body
cg02369820	4	6,200,609	0.020059	0.229	0.147				*JAKMIP1*	5'UTR
cg06660332	1	47,009,707	8.82E-06	0.229	0.244					
cg09920605	7	140,341,335	0.000111	0.229	0.224					
cg14300730	2	108,994,366	0.007021	0.229	0.131				*SULT1C4*	TSS200
cg21614408	6	52,228,023	0.009604	0.229	0.131				*PAQR8*	5'UTR
cg01748757	17	54,230,996	3.12E-09	0.229	0.207				*ANKFN1*	Body

cg07917150	7	27,154,264	7.74E-06	0.229	0.211				*HOXA3*	5'UTR
cg14477581	9	72,899,321	1.27E-08	0.229	0.225				*SMC5*	Body
cg02629070	6	41,394,156	5.25E-09	0.229	0.225					
cg08776014	21	43,183,039	8.08E-10	0.229	0.196				*RIPK4*	Body
cg26618058	12	118,022,239	4.01E-07	0.229	0.219				*KSR2*	Body
cg12801917	17	15,417,503	1.25E-05	0.229	0.221				*FAM18B2*	Body
cg25140419	1	221,050,742	4.34E-05	0.229	0.210					
cg06890415	8	67,405,405	2.52E-06	0.229	0.206				*C8orf46*	TSS200
cg14417760	15	27,111,749	0.000212	0.229	0.240				*GABRA5*	TSS1500
cg11549972	12	103,358,649	0.000109	0.229	0.208					
cg22980079	4	2,627,118	1.62E-13	0.229	0.205	3.09E-19	0.184		*FAM193A*	TSS200
cg00976949	3	127,950,782	4E-20	0.229	0.208				*EEFSEC*	Body
cg01231108	2	155,556,016	0.000263	0.229	0.232				*KCNJ3*	Body
cg27656658	17	46,628,224	3.49E-07	0.229	0.220				*HOXB3*	Body
cg12105183	1	89,829,876	4.4E-21	0.229	0.231				*GBP6*	5'UTR
cg13623495	6	10,426,099	1.96E-05	0.229	0.221					
cg14508021	15	85,987,419	1.22E-07	0.229	0.205				*AKAP13*	5'UTR
cg08607018	19	31,842,873	2.46E-05	0.229	0.214					
cg02064360	2	219,854,101	7.25E-06	0.229	0.226			Yes		
cg10454327	16	85,204,789	5.4E-18	0.229	0.241					
cg20597172	1	246,646,424	2.28E-08	0.229	0.212				*SMYD3*	Body
cg00203568	X	135,850,876	2.15E-08	0.229	0.221				*ARHGEF6*	Body
cg11280525	5	87,955,695	0.000273	0.229	0.253				*LOC645323*	Body
cg20890989	2	223,574,238	0.000409	0.229	0.237				*MOGAT1*	Body
cg04055345	10	116,264,862	7.83E-10	0.229	0.237				*ABLIM1*	Body
cg00036976	16	85,343,281	9.24E-19	0.229	0.219					
cg01222180	X	150,345,616	0.008629	0.229	0.227				*GPR50*	Body
cg04729730	11	69,258,477	0.022031	0.229	0.107					
cg23856138	10	35,658,321	0.001297	0.229	0.218				*CCNY*	Body
cg26307117	3	46,506,552	0.000135	0.229	0.177				*LTF*	TSS200
cg02456406	19	36,288,702	0.000984	0.229	0.265					
cg00687306	15	37,398,943	0.01723	0.229	0.258					
cg02438164	16	4,651,732	0.000121	0.229	0.264					
cg13530507	X	54,833,796	1.21E-07	0.229	0.224				*MAGED2*	TSS1500

cg07776049	2	928,870	1.15E-05	0.229	0.219					
cg13030582	17	19,290,708	4.9E-20	0.229	0.221	7.68E-16	0.117		*MFAP4*	TSS1500
cg25125298	5	71,853,215	3E-09	0.229	0.202					
cg27413563	7	95,026,090	1.01E-05	0.229	0.219				*PON3*	TSS1500
cg01096199	14	90,191,045	0.005823	0.229	0.174					
cg04805577	7	27,286,684	7.28E-10	0.229	0.199					
cg13737332	10	100,993,583	0.004658	0.229	0.297				*HPSE2*	Body
cg27450668	1	3,460,070	0.005702	0.229	0.186				*MEGF6*	Body
cg01427769	6	31,830,600	0.045091	0.229	0.223				*NEU1*	5'UTR
cg01212071	2	122,041,650	2.13E-08	0.229	0.214				*TFCP2L1*	Body
cg00194519	X	133,679,545	0.00132	0.229	0.235				*MGC16121*	Body
cg19134347	14	104,584,279	1.95E-09	0.229	0.217					
cg00077904	5	151,078,909	6.49E-11	0.229	0.219					
cg10303107	11	45,230,795	1.3E-62	0.229	0.212				*PRDM11*	Body
cg16160493	14	89,837,519	2.46E-14	0.229	0.220				*FOXN3*	Body
cg26266041	14	88,852,686	0.003748	0.229	0.103				*SPATA7*	Body
cg03572011	17	43,229,244	7.31E-06	0.229	0.249				*HEXIM1*	1stExon
cg13743231	10	94,958,683	0.031464	0.229	0.155					
cg04340435	13	114,145,973	0.000513	0.229	0.183				*TMCO3*	5'UTR
cg04202892	2	111,875,749	4.49E-05	0.229	0.236				*ACOXL*	3'UTR
cg09104660	12	57,635,479	6.96E-10	0.229	0.196				*NDUFA4L2*	TSS1500
cg12600692	2	198,809,390	0.000431	0.229	0.217				*PLCL1*	Body
cg23015118	1	2,232,527	0.000207	0.229	0.218				*SKI*	Body
cg09792881	1	50,884,544	0.011597	0.229	0.283				*DMRTA2*	Body
cg18178715	1	43,795,999	0.000325	0.229	0.246					
cg06124975	6	107,810,578	0.003071	0.229	0.118				*SOBP*	TSS1500
cg01938018	1	179,545,425	0.000105	0.229	0.196				*NPHS2*	TSS1500
cg04478428	20	61,584,072	1.21E-07	0.229	0.225				*SLC17A9*	1stExon
cg08162976	2	67,032,496	2.9E-08	0.229	0.183					
cg09606564	17	19,290,353	1.4E-08	0.229	0.253	0.00000792	0.094		*MFAP4*	Body
cg07963345	22	39,436,852	5.96E-07	0.229	0.207				*APOBEC3F*	1stExon
cg22158992	5	1,021,831	3.59E-13	0.229	0.202				*NKD2*	Body
cg26777836	3	26,665,815	0.039539	0.229	0.095				*LRRC3B*	5'UTR
cg13067221	14	23,449,582	7.35E-05	0.229	0.236				*JUB*	Body

cg23636571	6	32,828,996	0.024047	0.229	0.167					
cg26668276	7	96,633,311	0.011544	0.229	0.270				*DLX6AS*	Body
cg02773756	6	27,799,308	0.001212	0.229	0.186				*HIST1H4K*	TSS200
cg15406469	11	35,546,824	6.23E-05	0.229	0.204				*PAMR1*	Body
cg16134717	1	11,752,494	0.001065	0.229	0.238				*MAD2L2*	TSS1500
cg20325547	1	9,431,594	2.93E-12	0.229	0.223					
cg18614381	19	31,844,182	5.03E-07	0.229	0.211					
cg27246147	17	47,987,056	0.00025	0.229	0.246					
cg00801512	17	28,996,047	7.53E-06	0.229	0.283					
cg08355659	12	54,399,190	2.62E-10	0.229	0.196					
cg12061886	14	75,746,793	4.03E-09	0.229	0.204				*FOS*	Body
cg21046160	22	24,105,147	0.001913	0.229	0.149				*C22orf15*	TSS200
cg04105091	6	32,121,355	0.000708	0.229	0.235				*PPT2*	TSS1500
cg07197785	1	111,813,690	0.000877	0.229	0.282					
cg25706257	1	6,390,530	8.78E-23	0.229	0.209				*ACOT7*	Body
cg06558014	6	100,051,116	0.045262	0.229	0.371					
cg08910558	15	75,471,194	4.26E-05	0.229	0.229					
cg13382288	1	202,614,046	3.58E-07	0.229	0.215				*SYT2*	TSS1500
cg15852963	3	181,440,178	1.29E-05	0.229	0.211				*SOX2OT*	Body
cg26575450	2	64,834,280	0.000854	0.229	0.213					
cg00178119	8	105,235,184	9.66E-07	0.229	0.217				*RIMS2*	Body
cg07910680	18	56,296,449	1.21E-15	0.229	0.216				*ALPK2*	TSS1500
cg01084154	1	20,692,892	0.020133	0.229	0.303					
cg09731996	5	3,599,090	0.00368	0.229	0.265				*IRX1*	Body
cg16601359	12	2,955,294	1.12E-13	0.229	0.228					
cg04074153	12	29,568,207	0.00076	0.229	0.287					
cg21357996	20	33,578,276	6.43E-17	0.229	0.225				*MYH7B*	Body
cg21784254	13	108,922,428	8.97E-06	0.229	0.218				*TNFSF13B*	1stExon
cg01289103	17	38,821,277	0.029216	0.229	0.232	0.0000128	0.114		*KRT222*	1stExon
cg10780949	15	67,418,316	0.000375	0.229	0.166				*SMAD3*	Body
cg11028052	15	81,154,387	7.81E-10	0.229	0.232				*KIAA1199*	5'UTR
cg07417284	17	1,082,225	1.57E-05	0.229	0.196				*ABR*	Body
cg13045555	8	105,342,365	1.53E-10	0.229	0.216					
cg17032005	1	92,891,301	3.98E-14	0.229	0.236					

cg17172851	1	18,957,328	0.002991	0.229	0.244				*PAX7*	TSS200
cg24848787	15	58,844,359	3.59E-06	0.229	0.195				*LIPC*	Body
cg27485596	13	111,829,530	1.44E-09	0.229	0.223				*ARHGEF7*	Body
cg16999370	4	122,302,232	0.030095	0.229	0.224				*QRFPR*	TSS200
cg08072507	7	133,897,858	6.88E-07	0.229	0.199				*LRGUK*	Body
cg24213719	18	60,263,646	0.015375	0.229	0.201					
cg00162969	12	132,933,532	0.013749	0.229	0.299					
cg00702126	1	1,369,793	0.01489	0.229	0.142				*VWA1*	TSS1500
cg04995300	3	66,848,608	0.00015	0.229	0.250					
cg06017212	17	1,478,463	2.53E-12	0.228	0.215				*SLC43A2*	3'UTR
cg27176614	16	88,717,989	2.13E-11	0.228	0.227				*CYBA*	TSS1500
cg26421310	1	25,257,058	0.005799	0.228	0.189				*RUNX3*	TSS1500
cg13341668	3	50,359,909	9.12E-14	0.228	0.247				*HYAL2*	5'UTR
cg15976388	1	114,696,946	0.011963	0.228	0.266				*SYT6*	TSS1500
cg17533927	7	128,550,898	3.11E-18	0.228	0.203				*KCP*	TSS200
cg02582057	7	155,258,253	0.011712	0.228	0.206					
cg11993173	8	54,857,695	2.97E-05	0.228	0.262				*RGS20*	Body
cg17069396	20	2,731,102	4.75E-09	0.228	0.209				*EBF4*	Body
cg15603311	3	54,157,900	0.006877	0.228	0.221				*CACNA2D3*	Body
cg03804255	12	90,278,335	8.55E-07	0.228	0.223					
cg09260773	1	119,529,266	0.003687	0.228	0.310				*TBX15*	5'UTR
cg22101190	17	77,896,096	0.001152	0.228	0.189					
cg04143876	15	76,639,558	0.000452	0.228	0.131					
cg08818092	X	24,665,074	0.048632	0.228	0.184				*PCYT1B*	Body
cg10499651	3	12,465,415	0.004479	0.228	0.177				*PPARG*	Body
cg03230711	2	45,228,334	2.92E-10	0.228	0.251					
cg11620672	6	19,298,476	1.2E-14	0.228	0.225					
cg07010228	19	2,253,854	1.17E-07	0.228	0.214				*JSRP1*	Body
cg08662620	10	34,061,705	8.7E-07	0.228	0.233					
cg14780466	2	20,870,812	3.53E-10	0.228	0.216				*GDF7*	Body
cg20779964	20	54,580,070	6.53E-12	0.228	0.210				*CBLN4*	TSS200
cg18496447	15	45,480,917	0.019099	0.228	0.126				*SHF*	Body
cg25910519	7	157,389,729	1.24E-17	0.228	0.220				*PTPRN2*	Body
cg06948492	19	34,398,264	4.87E-07	0.228	0.226					

cg15843262	2	25,473,895	1.8E-06	0.228	0.223				*DNMT3A*	Body
cg16198103	8	53,471,884	9.78E-09	0.228	0.209				*FAM150A*	Body
cg06159404	10	43,846,376	0.01462	0.228	0.136					
cg26006870	8	11,559,039	1.71E-06	0.228	0.239					
cg01942962	4	158,143,439	0.03986	0.228	0.167				*GRIA2*	Body
cg00953480	9	37,034,933	0.043273	0.228	0.377				*PAX5*	TSS1500
cg25929173	2	182,548,308	0.038767	0.228	0.049					
cg08290668	16	876,164	1.41E-07	0.228	0.197					
cg10130564	11	117,069,849	4.28E-13	0.228	0.225				*TAGLN*	TSS200
cg24399204	2	11,636,294	2.69E-07	0.228	0.191					
cg04633683	16	29,837,056	2.05E-06	0.228	0.229				*MVP*	5'UTR
cg07942135	7	27,154,262	0.000327	0.228	0.203				*HOXA3*	5'UTR
cg27262850	18	74,826,769	3.47E-07	0.228	0.214				*MBP*	5'UTR
cg05053978	6	117,869,098	1.69E-05	0.228	0.221				*DCBLD1*	Body
cg03795847	3	44,596,512	5.1E-05	0.228	0.157				*ZNF167*	TSS200
cg13344095	6	26,233,352	0.014963	0.228	0.216					
cg00761125	12	96,590,115	1.54E-07	0.228	0.228				*ELK3*	5'UTR
cg26522361	5	55,625,247	2.68E-10	0.228	0.209					
cg05673882	5	74,862,702	0.000165	0.228	0.210				*POLK*	Body
cg10397440	8	57,359,258	5.28E-05	0.228	0.253				*PENK*	TSS1500
cg17046823	17	16,256,992	0.006156	0.228	0.143				*CENPV*	TSS200
cg18722841	11	71,954,982	0.000579	0.228	0.277	4.24E-08	0.163		*PHOX2A*	1stExon
cg01547915	2	38,947,762	0.00189	0.228	0.243				*GALM*	Body
cg06191390	2	73,299,285	7.36E-06	0.228	0.175				*SFXN5*	TSS1500
cg26395761	7	107,700,124	2.08E-11	0.228	0.220				*LAMB4*	Body
cg02329430	15	73,921,385	1.37E-23	0.228	0.215				*NPTN*	Body
cg09090017	15	23,158,623	0.000279	0.228	0.241					
cg22729539	12	122,519,119	2.36E-07	0.228	0.226				*MLXIP*	Body
cg08832414	3	183,993,571	6.3E-10	0.228	0.191				*ECE2*	Body
cg12854483	19	11,649,671	7.68E-07	0.228	0.234	6.84E-28	0.211		*CNN1*	1stExon
cg23733328	X	53,311,101	6.46E-06	0.228	0.242				*IQSEC2*	Body
cg22494858	1	110,611,712	3.71E-06	0.228	0.228				*ALX3*	Body
cg06201393	3	169,378,835	2.55E-05	0.228	0.216				*MECOM*	Body
cg09553651	15	60,411,555	0.004838	0.228	0.185					

cg26346108	19	35,329,641	0.001035	0.228	0.262					
cg07355926	19	1,852,127	9.28E-06	0.228	0.210					
cg09473576	1	20,813,647	0.033153	0.228	0.092				*CAMK2N1*	TSS1500
cg05230392	17	55,976,522	0.000262	0.228	0.279				*CUEDC1*	5'UTR
cg10546817	14	95,233,855	1.99E-06	0.228	0.207					
cg06710259	5	55,775,841	8.81E-12	0.228	0.214					
cg14376625	12	111,736,686	2.36E-08	0.228	0.246				*CUX2*	Body
cg14886572	17	40,934,398	0.045292	0.228	0.075				*WNK4*	Body
cg16585333	1	223,302,861	0.004751	0.228	0.236				*TLR5*	5'UTR
cg25670685	13	102,068,432	0.008936	0.228	0.129				*NALCN*	5'UTR
cg08430329	2	73,430,374	0.015133	0.228	0.264				*NOTO*	Body
cg10902144	3	28,618,700	2.81E-06	0.228	0.162					
cg13900817	5	172,070,153	1.55E-05	0.228	0.219				*NEURL1B*	Body
cg02852670	14	20,903,410	1.1E-11	0.228	0.257				*KLHL33*	5'UTR
cg19659741	8	39,172,099	9.86E-05	0.228	0.246				*ADAM5P*	TSS200
cg27343917	2	75,751,211	5.8E-05	0.228	0.244				*FAM176A*	5'UTR
cg27638453	6	157,470,150	6.59E-06	0.228	0.217				*ARID1B*	Body
cg02246426	8	97,162,431	0.000361	0.228	0.216				*GDF6*	Body
cg18358869	10	102,808,077	1.12E-10	0.228	0.217					
cg19317211	20	2,801,711	0.002439	0.228	0.270					
cg05771342	11	68,782,202	6.02E-09	0.228	0.221				*MRGPRF*	TSS1500
cg15282819	16	12,997,592	0.016989	0.228	0.257				*SHISA9*	Body
cg24366665	13	31,371,618	0.002529	0.228	0.200					
cg00395063	11	120,210,293	6.95E-07	0.228	0.227				*ARHGEF12*	Body
cg05033803	6	27,182,976	0.017349	0.228	0.315					
cg05619598	19	33,569,228	3.06E-05	0.228	0.229					
cg07802949	8	1,404,980	1.98E-07	0.228	0.192					
cg10333214	16	1,382,952	0.00011	0.228	0.203					
cg25444386	4	55,990,944	3.89E-10	0.228	0.237				*KDR*	Body
cg10205310	12	117,131,820	9.62E-14	0.228	0.181					
cg08914623	11	44,327,572	2.2E-06	0.228	0.252	4.03E-23	0.211		*ALX4*	Body
cg13598256	5	140,801,286	0.00286	0.228	0.277				*PCDHGA4*	Body
cg15927196	7	95,025,905	1.68E-05	0.228	0.199				*PON3*	TSS1500
cg21413219	1	6,299,374	9.12E-05	0.228	0.275					

cg15081720	1	119,866,618	2.34E-16	0.228	0.217						
cg15895925	1	33,810,859	1.6E-18	0.228	0.240					*PHC2*	5'UTR
cg02854922	1	144,930,964	2.57E-06	0.228	0.135					*PDE4DIP*	1stExon
cg11581248	1	9,887,811	2.24E-14	0.228	0.222						
cg12067764	1	153,762,244	0.002352	0.228	0.176						
cg21506800	19	11,531,519	9.37E-05	0.228	0.235					*CCDC151*	Body
cg22663872	7	149,120,266	0.000137	0.228	0.231						
cg03391657	1	121,260,690	0.000616	0.228	0.095					*LOC647121*	TSS1500
cg11721464	19	42,703,763	6.62E-09	0.228	0.202					*DEDD2*	Body
cg14270581	14	103,394,548	6.59E-09	0.228	0.204					*AMN*	Body
cg06759189	17	77,777,947	0.021966	0.228	0.187						
cg01691710	8	49,292,285	3.67E-11	0.228	0.201						
cg02168584	2	172,972,681	0.001664	0.228	0.222						
cg04431143	3	37,986,017	2.47E-05	0.228	0.240					*CTDSPL*	Body
cg17173187	15	85,201,210	0.0256	0.228	0.202					*NMB*	Body
cg19547972	7	73,389,575	8.18E-09	0.228	0.231						
cg26818573	2	46,717,839	2.57E-09	0.228	0.227						
cg17964510	2	175,199,694	0.033455	0.228	0.279					*SP9*	TSS200
cg00767496	15	45,671,279	7.47E-06	0.228	0.223					*LOC145663*	Body
cg01043845	2	177,013,071	6.34E-06	0.228	0.187						
cg14377594	9	1,051,505	9.8E-08	0.228	0.198					*DMRT2*	5'UTR
cg04335355	X	44,204,116	1.27E-05	0.228	0.218					*EFHC2*	TSS1500
cg21858380	2	119,916,486	0.002981	0.228	0.230					*C1QL2*	TSS200
cg01815538	6	100,054,615	5.46E-05	0.228	0.148					*PRDM13*	TSS200
cg02718825	20	55,202,842	2.9E-07	0.228	0.219						
cg02801786	12	113,914,222	0.005787	0.228	0.254						
cg16552454	20	61,583,924	5.16E-09	0.228	0.222					*SLC17A9*	TSS200
cg03233918	4	84,991,657	0.031789	0.228	0.254						
cg06118312	5	54,398,555	0.000808	0.228	0.242	1.18E-13	0.217			*GZMA*	1stExon
cg08529345	16	54,971,700	0.002593	0.228	0.249						
cg22254983	8	136,181,389	0.049822	0.228	0.323						
cg07227671	12	124,780,924	0.011683	0.228	0.282					*FAM101A*	5'UTR
cg01944288	9	135,036,217	1.26E-09	0.228	0.237					*NTNG2*	TSS1500
cg11652668	18	13,136,899	0.007275	0.228	0.069						

cg13929328	10	129,535,898	4.22E-07	0.228	0.173	5.15E-23	0.207		*FOXI2*	1stExon
cg15438548	10	123,098,791	1.31E-11	0.228	0.213					
cg22751954	6	143,023,932	2.55E-13	0.228	0.214					
cg26525121	6	14,129,370	3.36E-17	0.228	0.218				*CD83*	Body
cg26860613	12	6,661,647	2.18E-13	0.228	0.221				*IFFO1*	Body
cg10885338	2	106,682,640	2.51E-06	0.228	0.206	5.67E-09	0.089		*C2orf40*	Body
cg24116965	4	148,654,701	2.63E-08	0.228	0.207				*ARHGAP10*	Body
cg01323049	2	19,557,396	2.97E-05	0.228	0.187				*OSR1*	5'UTR
cg09314702	12	76,271,607	3.81E-09	0.228	0.223					
cg03946671	20	62,693,973	4.6E-13	0.228	0.233				*TCEA2*	TSS200
cg04754011	11	60,623,144	7.58E-09	0.228	0.216	1.78E-20	0.101		*GPR44*	5'UTR
cg06772671	16	85,123,778	8.35E-18	0.228	0.220				*KIAA0513*	3'UTR
cg15506703	2	242,844,601	0.01847	0.228	0.185					
cg18249173	7	35,293,245	0.007369	0.228	0.244				*TBX20*	1stExon
cg23319982	19	41,641,847	0.000194	0.228	0.224					
cg06829760	2	16,845,412	9.5E-08	0.228	0.246				*FAM49A*	5'UTR
cg10066186	14	23,083,160	7.09E-13	0.228	0.235					
cg11410682	19	46,999,224	0.001727	0.228	0.279				*PNMAL2*	TSS200
cg12999932	3	177,412,249	3.98E-09	0.228	0.234					
cg17818471	2	177,012,640	6.33E-07	0.228	0.174					
cg07016258	15	83,953,929	3.54E-06	0.228	0.243				*BNC1*	TSS1500
cg14799457	18	19,927,125	0.026323	0.228	0.149					
cg03982087	18	9,708,920	0.044965	0.228	0.110				*RAB31*	Body
cg06108459	6	43,089,049	4.99E-10	0.228	0.225				*PTK7*	Body
cg22459204	5	10,565,940	1.75E-07	0.228	0.221				*ANKRD33B*	Body
cg10362172	3	138,679,578	0.001971	0.228	0.252					
cg14252141	6	1,388,159	1.89E-06	0.228	0.172					
cg20387775	6	158,136,705	3.14E-13	0.228	0.233					
cg05670461	3	157,155,088	8.69E-05	0.228	0.208				*VEPH1*	Body
cg16858415	11	116,967,348	8.45E-06	0.228	0.233				*SIK3*	Body
cg01387771	16	1,036,944	0.002425	0.228	0.228				*SOX8*	3'UTR
cg11944284	11	2,904,663	2.87E-15	0.228	0.208				*CDKN1C*	3'UTR
cg05488474	12	54,388,934	0.0021	0.228	0.174					
cg05826677	7	48,031,468	0.013686	0.228	0.271				*SUNC1*	Body

cg10687131	2	20,871,002	0.032888	0.228	0.302				*GDF7*	Body
cg11848254	7	134,939,464	9.61E-08	0.228	0.229				*STRA8*	Body
cg11841529	20	44,746,751	0.028131	0.228	0.338				*CD40*	TSS200
cg04365202	1	197,889,248	0.007592	0.228	0.260				*LHX9*	Body
cg20459687	13	99,128,579	2.7E-07	0.228	0.219				*STK24*	Body
cg24927890	5	149,980,526	0.000302	0.228	0.212				*SYNPO*	TSS200
cg10502220	4	176,733,966	2.49E-05	0.228	0.173				*GPM6A*	1stExon
cg14087880	10	47,083,316	0.006783	0.228	0.091				*PPYR1*	TSS1500
cg11132534	2	218,114,431	1.56E-06	0.228	0.187					
cg21752601	2	27,531,310	9.95E-26	0.228	0.232				*UCN*	TSS200
cg03950655	1	64,379,958	1.15E-06	0.228	0.250				*ROR1*	Body
cg08355260	2	74,729,429	0.027671	0.228	0.298				*LOC151534*	TSS1500
cg14700707	6	32,191,840	2.11E-07	0.228	0.251	1.1E-30	0.162		*NOTCH4*	1stExon
cg03509898	14	104,190,750	3.18E-21	0.228	0.219				*ZFYVE21*	Body
cg19503640	5	1,503,763	1.33E-43	0.228	0.228				*LPCAT1*	Body
cg01270593	14	51,339,072	1.72E-06	0.228	0.248				*ABHD12B*	5'UTR
cg04451570	10	81,892,128	5.97E-05	0.228	0.199				*PLAC9*	TSS200
cg09807875	7	34,136,112	0.001283	0.228	0.205				*BMPER*	Body
cg10679688	13	100,624,965	0.013125	0.228	0.234				*ZIC5*	TSS1500
cg18468354	14	36,993,517	0.002103	0.228	0.247					
cg07557630	5	34,503,180	8.71E-11	0.228	0.216					
cg12729305	1	208,497,546	0.012122	0.228	0.152					
cg14334441	6	27,235,820	0.00038	0.228	0.253					
cg21476000	12	54,430,684	2.47E-15	0.228	0.204				*HOXC4*	5'UTR
cg21550024	4	123,747,533	5.96E-08	0.228	0.216				*FGF2*	TSS1500
cg03583050	19	3,358,182	5.6E-08	0.228	0.189				*NFIC*	TSS1500
cg11012061	5	145,722,468	0.032025	0.228	0.204					
cg13011961	3	67,291,183	5.51E-12	0.228	0.223					
cg22059812	1	19,992,564	9.72E-05	0.228	0.198				*HTR6*	1stExon
cg08827358	16	66,461,802	0.002901	0.228	0.246				*BEAN*	5'UTR
cg15996644	12	54,370,352	0.006447	0.227	0.137					
cg17470942	6	134,210,279	4.16E-05	0.227	0.246				*TCF21*	1stExon
cg18469813	6	168,148,018	1.02E-16	0.227	0.221					
cg06043908	8	49,467,831	3.79E-19	0.227	0.218					

cg08761350	X	101,967,081	0.032535	0.227	0.236				*GPRASP2*	TSS1500
cg08848847	11	36,399,468	6.4E-05	0.227	0.123				*PRR5L*	5'UTR
cg10553103	5	140,619,567	0.004527	0.227	0.301				*PCDHB19P*	TSS200
cg12485727	20	39,762,753	1.29E-12	0.227	0.218					
cg16831623	20	6,750,617	0.022735	0.227	0.275				*BMP2*	5'UTR
cg23357257	16	29,887,967	1.26E-05	0.227	0.234				*SEZ6L2*	Body
cg08373610	2	198,540,621	0.002386	0.227	0.265	0.0000873	0.073		*RFTN2*	TSS200
cg06989610	12	54,451,877	1.82E-06	0.227	0.167				*FLJ12825*	TSS200
cg07568117	16	2,139,114	1.47E-07	0.227	0.219				*PKD1*	3'UTR
cg13222091	20	3,758,207	0.000904	0.227	0.076				*SPEF1*	3'UTR
cg01751605	7	137,028,546	0.007351	0.227	0.151				*PTN*	TSS200
cg05996512	15	63,032,491	9E-13	0.227	0.224				*TLN2*	Body
cg22927247	8	86,351,195	4.43E-10	0.227	0.223				*CA3*	Body
cg02185666	2	206,549,667	1.69E-05	0.227	0.218				*NRP2*	Body
cg05534403	20	61,583,830	3.69E-05	0.227	0.219				*SLC17A9*	TSS200
cg18708252	22	39,545,030	3.55E-08	0.227	0.263				*CBX7*	Body
cg10646402	12	15,475,490	0.003328	0.227	0.231	5.57E-08	0.134		*PTPRO*	1stExon
cg19519310	6	1,389,367	0.003826	0.227	0.127				*FOXF2*	TSS1500
cg03783663	13	70,682,628	0.000131	0.227	0.249				*KLHL1*	TSS200
cg13154880	4	48,486,472	0.000128	0.227	0.228				*SLC10A4*	Body
cg15000841	11	64,815,642	0.000275	0.227	0.217				*NAALADL1*	Body
cg16153547	2	74,725,925	0.00251	0.227	0.260				*LBX2*	Body
cg21141812	3	48,556,323	3.96E-07	0.227	0.190				*PFKFB4*	3'UTR
cg22579075	7	94,250,352	0.001431	0.227	0.292				*SGCE*	Body
cg07250162	17	46,832,247	1.05E-05	0.227	0.210					
cg09829263	13	49,066,969	0.001724	0.227	0.115				*RCBTB2*	Body
cg11469061	11	31,826,421	0.009998	0.227	0.216				*PAX6*	Body
cg01671895	11	100,997,799	0.000377	0.227	0.211				*PGR*	Body
cg08479476	2	70,313,163	0.034081	0.227	0.284				*PCBP1*	TSS1500
cg14081465	9	119,722,239	2.9E-07	0.227	0.218				*ASTN2*	Body
cg23280568	17	46,534,400	1.66E-09	0.227	0.188					
cg05082708	4	4,765,299	0.000106	0.227	0.207					
cg05874806	2	102,350,276	4.89E-06	0.227	0.231				*MAP4K4*	Body
cg07719621	16	54,970,469	0.007837	0.227	0.284					

cg15158977	17	62,962,238	0.000219	0.227	0.228					
cg26620147	12	58,210,716	1.68E-08	0.227	0.238				*AVIL*	TSS1500
cg02119938	15	78,505,051	1.05E-09	0.227	0.232				*ACSBG1*	Body
cg03408904	22	17,956,462	5.3E-12	0.227	0.199				*CECR2*	TSS200
cg08714590	7	90,895,894	4.86E-05	0.227	0.248				*FZD1*	1stExon
cg10105237	8	135,726,091	5.13E-14	0.227	0.229				*ZFAT*	TSS1500
cg02124184	5	72,950,691	1.48E-20	0.227	0.214			Yes	*RGNEF*	5'UTR
cg06654127	6	41,395,230	0.001786	0.227	0.184					
cg27178922	1	213,476,578	7.85E-20	0.227	0.196					
cg08108750	15	96,889,464	0.000627	0.227	0.240					
cg21280510	1	40,137,089	0.001675	0.227	0.147	1.32E-11	0.120		*NT5C1A*	Body
cg17104258	1	167,090,646	0.01966	0.227	0.098				*DUSP27*	Body
cg07021447	14	69,418,951	6.37E-09	0.227	0.217				*ACTN1*	Body
cg10714061	6	33,714,631	6.37E-12	0.227	0.217				*IP6K3*	1stExon
cg06769082	7	99,156,634	0.016126	0.227	0.175				*ZNF655*	5'UTR
cg03774514	20	22,563,702	0.013585	0.227	0.189				*FOXA2*	Body
cg06911084	7	96,652,894	0.000204	0.227	0.181	6.09E-20	0.232		*DLX5*	Body
cg12091498	12	47,226,255	9.22E-06	0.227	0.228					
cg21145245	5	150,593,684	3.93E-06	0.227	0.223				*CCDC69*	Body
cg01334682	6	21,666,939	0.001159	0.227	0.245				*FLJ22536*	Body
cg01304890	4	81,106,772	0.000127	0.227	0.229				*PRDM8*	5'UTR
cg27149179	11	86,451,848	1.53E-06	0.227	0.211					
cg23558213	22	41,215,939	3.14E-12	0.227	0.223				*SLC25A17*	TSS1500
cg24889708	17	46,678,719	2.21E-08	0.227	0.223				*LOC404266*	Body
cg16481365	14	105,554,352	0.006898	0.227	0.143					
cg01554437	14	21,091,422	2.91E-05	0.227	0.254					
cg02389949	7	30,176,264	5.52E-07	0.227	0.163				*C7orf41*	Body
cg20561830	14	81,893,829	1.49E-05	0.227	0.190					
cg03284866	8	124,173,487	0.000218	0.227	0.215					
cg18503912	19	35,630,279	2.27E-08	0.227	0.218				*FXYD1*	5'UTR
cg22958090	12	52,627,438	1.73E-08	0.227	0.221				*KRT7*	Body
cg12418190	15	37,403,304	0.007172	0.227	0.131					
cg04424215	X	111,325,143	5.01E-06	0.227	0.217				*TRPC5*	5'UTR
cg06686156	16	20,420,222	5.17E-16	0.227	0.235				*ACSM5*	TSS1500

cg09712234	16	30,614,413	5.2E-10	0.227	0.226					
cg10803577	2	177,016,837	0.000165	0.227	0.175				*HOXD4*	Body
cg13283952	2	121,412,005	1.58E-09	0.227	0.234					
cg01250001	8	103,758,088	3.05E-09	0.227	0.212					
cg04549460	8	109,095,975	0.007431	0.227	0.231				*RSPO2*	TSS200
cg12057563	X	135,230,841	1.27E-05	0.227	0.246				*FHL1*	Body
cg02241986	10	5,566,543	2.31E-06	0.227	0.258				*CALML3*	TSS1500
cg26493353	9	91,793,721	0.01415	0.227	0.283				*SHC3*	TSS200
cg13324546	8	23,564,031	0.000197	0.227	0.204				*NKX2-6*	TSS200
cg21899640	6	168,609,219	1.44E-07	0.227	0.209					
cg02770751	19	795,467	2.97E-07	0.227	0.235					
cg07852825	3	172,166,182	1.95E-08	0.227	0.217				*GHSR*	1stExon
cg23331961	15	72,564,179	1.22E-06	0.227	0.174				*PARP6*	TSS1500
cg25658983	13	46,426,026	0.000331	0.227	0.206				*SIAH3*	TSS200
cg27229613	1	229,569,046	0.001068	0.227	0.246				*ACTA1*	5'UTR
cg06145736	3	143,559,620	2.52E-24	0.227	0.224			Yes	*SLC9A9*	Body
cg16429975	1	6,445,632	4.26E-07	0.227	0.184				*ACOT7*	Body
cg07200280	3	16,554,619	0.025997	0.227	0.324	2.59E-07	0.100		*RFTN1*	5'UTR
cg09685182	3	130,236,037	0.000573	0.227	0.240					
cg02646779	2	72,342,554	3.12E-08	0.227	0.194					
cg15787039	15	32,934,185	2.85E-10	0.227	0.164	4.03E-19	0.201		*SCG5*	5'UTR
cg18530551	5	172,670,879	0.013053	0.227	0.294					
cg06157170	6	167,764,523	0.013829	0.227	0.112					
cg17094985	3	159,756,929	0.047608	0.227	0.250					
cg08474786	17	17,491,465	2.23E-17	0.227	0.238				*PEMT*	Body
cg00456672	15	62,358,751	4.74E-12	0.227	0.184				*C2CD4A*	TSS1500
cg06550984	10	23,480,002	0.031999	0.227	0.271				*PTF1A*	TSS1500
cg27543403	5	58,335,880	2.5E-06	0.227	0.201				*PDE4D*	Body
cg08160091	4	1,398,383	0.000487	0.227	0.135					
cg26082368	6	37,673,708	2.14E-05	0.227	0.204					
cg06137852	13	28,545,283	1.58E-06	0.227	0.206					
cg19719930	17	70,339,343	2.13E-07	0.227	0.199					
cg26174583	6	1,392,624	1.6E-12	0.227	0.199				*FOXF2*	Body
cg05855039	14	85,996,751	0.044331	0.227	0.333				*FLRT2*	1stExon

cg13396713	17	505,019	3.53E-14	0.227	0.210				*VPS53*	Body
cg14478713	9	16,653,688	0.029671	0.227	0.139				*BNC2*	Body
cg26686732	20	22,562,737	0.00302	0.227	0.233				*FOXA2*	Body
cg00225623	13	24,845,891	0.029224	0.227	0.057				*SPATA13*	Body
cg04887321	16	55,090,590	0.001982	0.227	0.258					
cg13547127	6	145,217,150	0.000524	0.227	0.165					
cg20663495	17	80,943,851	2.72E-11	0.227	0.228				*B3GNTL1*	Body
cg10591771	19	3,671,118	0.000213	0.227	0.222				*PIP5K1C*	Body
cg12594244	15	74,218,754	3.97E-13	0.227	0.221				*LOXL1*	TSS200
cg13372658	8	49,783,907	1.28E-14	0.227	0.209					
cg02966332	20	62,369,605	1.85E-12	0.227	0.227				*LIME1*	Body
cg03602280	18	11,148,914	0.026282	0.227	0.249				*FAM38B*	TSS200
cg09219877	16	2,287,294	0.002543	0.227	0.149				*DNASE1L2*	Body
cg07005778	4	188,953,586	3.16E-05	0.227	0.235					
cg22951509	5	94,955,893	0.009593	0.227	0.153				*GPR150*	TSS200
cg09119688	X	119,133,700	0.029669	0.227	0.264					
cg16617723	18	24,443,905	9.69E-09	0.227	0.208				*AQP4*	TSS1500
cg24276105	11	45,686,055	0.001567	0.227	0.196				*CHST1*	5'UTR
cg05733463	11	13,282,917	1.06E-06	0.227	0.219			Yes		
cg25424299	3	13,319,638	0.000547	0.227	0.245					
cg03245590	11	15,329,459	5.32E-05	0.227	0.206					
cg05255275	14	60,952,097	0.026313	0.227	0.177				*C14orf39*	5'UTR
cg16122427	1	36,947,989	2.26E-07	0.227	0.252				*CSF3R*	5'UTR
cg19363579	9	135,460,728	4.41E-05	0.227	0.220				*BARHL1*	Body
cg14310703	3	126,006,903	0.014051	0.227	0.139					
cg02061956	1	146,697,547	0.022196	0.227	0.301				*FMO5*	TSS1500
cg04282694	6	5,996,412	0.001212	0.227	0.259					
cg16424082	12	51,323,740	2.94E-20	0.227	0.218				*METTL7A*	Body
cg17533951	X	68,115,082	6.41E-22	0.227	0.228					
cg22635096	21	46,550,644	1.18E-06	0.227	0.217				*ADARB1*	5'UTR
cg00561775	16	1,159,805	0.000257	0.227	0.200					
cg09303936	6	73,331,290	0.02907	0.227	0.298				*KCNQ5*	TSS1500
cg13351161	8	27,490,921	8.84E-05	0.227	0.172	6.02E-12	0.122		*SCARA3*	TSS1500
cg20314884	2	220,108,094	0.008748	0.227	0.154				*GLB1L*	Body

cg00258316	1	8,194,341	1.28E-08	0.227	0.219					
cg14528374	7	99,178,047	0.004225	0.227	0.145					
cg01310096	13	35,474,735	9.48E-10	0.227	0.217					
cg05196905	21	47,877,665	0.00011	0.227	0.183				*DIP2A*	TSS1500
cg25205489	13	24,125,993	2.91E-10	0.227	0.200					
cg00979931	11	72,533,664	0.000116	0.227	0.116				*ATG16L2*	Body
cg02955098	1	3,473,665	9.02E-06	0.227	0.249				*MEGF6*	Body
cg18362330	5	134,872,659	8.47E-07	0.227	0.237				*NEUROG1*	TSS1500
cg18453446	21	28,219,387	0.012475	0.227	0.190					
cg19047068	17	34,119,162	5.2E-07	0.227	0.257				*MMP28*	Body
cg24794228	19	52,391,166	0.034608	0.227	0.211				*ZNF577*	Body
cg09456289	2	43,038,399	0.036255	0.227	0.149					
cg13904481	5	132,118,820	9.99E-06	0.227	0.220					
cg17782974	10	104,406,990	4.54E-05	0.227	0.258				*TRIM8*	Body
cg25333529	7	73,536,420	7.5E-18	0.227	0.213				*LIMK1*	3'UTR
cg04750421	14	97,208,333	1.26E-05	0.227	0.226					
cg24529736	13	96,205,679	0.002586	0.227	0.223				*CLDN10*	Body
cg25946059	2	242,973,936	1.05E-06	0.227	0.200					
cg03954144	13	20,751,679	7.31E-17	0.227	0.201					
cg10548805	14	101,538,070	0.00035	0.227	0.249					
cg19515245	2	97,151,922	1.64E-05	0.227	0.184					
cg25071651	4	101,112,317	4.32E-07	0.227	0.188				*DDIT4L*	TSS1500
cg25115460	1	3,607,110	0.007669	0.227	0.202	1.47E-13	0.168		*TP73*	Body
cg21488066	6	28,367,898	3.31E-05	0.227	0.185				*ZSCAN12*	TSS1500
cg13906377	1	63,792,695	0.039999	0.227	0.280					
cg04261556	7	20,373,143	9.48E-14	0.227	0.222				*ITGB8*	Body
cg09635954	7	29,605,624	4E-12	0.227	0.211				*PRR15*	5'UTR
cg10784519	6	30,737,231	1.55E-06	0.227	0.223					
cg25394505	10	134,399,880	1.55E-12	0.227	0.217				*INPP5A*	Body
cg25535982	11	74,030,400	4.6E-07	0.227	0.233					
cg05336982	16	69,564,735	0.002173	0.227	0.222					
cg19092163	17	4,403,417	0.001633	0.227	0.208				*SPNS2*	Body
cg13853591	1	65,468,921	0.001113	0.227	0.135					
cg05457768	6	28,751,790	0.04141	0.227	0.262					

cg15256492	12	115,125,475	1.41E-13	0.227	0.213					
cg15944766	8	29,210,790	0.045459	0.227	0.231					
cg03506028	7	35,494,689	0.001418	0.227	0.215					
cg26916780	15	64,889,554	1.11E-05	0.227	0.174				*ZNF609*	Body
cg01840020	17	4,648,949	0.001508	0.227	0.229				*ZMYND15*	Body
cg00143249	7	156,798,810	0.001028	0.227	0.233				*MNX1*	Body
cg03825231	3	125,898,467	0.019513	0.227	0.243				*ALDH1L1*	5'UTR
cg03866862	6	33,401,542	5.82E-15	0.227	0.220				*SYNGAP1*	Body
cg07220152	6	30,737,270	0.002295	0.227	0.260					
cg09439817	17	45,933,066	1.29E-08	0.227	0.242				*SP6*	5'UTR
cg01845476	9	136,833,401	3.03E-17	0.226	0.197				*VAV2*	Body
cg17679604	5	115,790,387	8.68E-06	0.226	0.224				*SEMA6A*	Body
cg18280830	14	57,265,875	1.11E-07	0.226	0.229					
cg11536940	8	97,658,076	0.012799	0.226	0.135	4.56E-23	0.203		*PGCP*	5'UTR
cg17161130	10	113,544,977	2.82E-05	0.226	0.200					
cg09754272	1	156,399,638	0.000121	0.226	0.303				*C1orf61*	TSS1500
cg00152008	1	50,889,225	0.028812	0.226	0.196				*DMRTA2*	TSS200
cg05002166	1	217,119,469	1.06E-14	0.226	0.234				*ESRRG*	5'UTR
cg22886649	7	190,349	2.94E-30	0.226	0.218					
cg25277809	2	128,453,484	0.034069	0.226	0.159					
cg16931664	11	11,169,707	1.22E-05	0.226	0.229					
cg09336988	19	42,386,872	1.9E-06	0.226	0.205				*ARHGEF1*	TSS1500
cg13673514	14	101,035,818	1.86E-06	0.226	0.232	1.62E-12	0.128		*BEGAIN*	TSS1500
cg24790912	7	66,162,803	1.23E-15	0.226	0.197					
cg00708598	13	53,313,895	0.000891	0.226	0.195	2.1E-22	0.168		*LECT1*	1stExon
cg07959574	4	42,401,056	0.020472	0.226	0.249				*SHISA3*	Body
cg15365032	9	100,610,697	0.01142	0.226	0.181					
cg02455346	1	221,055,401	2.28E-17	0.226	0.224				*HLX*	Body
cg05738240	12	114,337,927	1.22E-05	0.226	0.205				*RBM19*	Body
cg05940514	17	39,791,696	0.000155	0.226	0.223					
cg06447424	13	20,875,743	3.4E-05	0.226	0.240					
cg16820615	13	114,884,918	0.006446	0.226	0.253				*RASA3*	Body
cg01907106	22	29,875,592	0.001637	0.226	0.267				*NEFH*	TSS1500
cg02748089	16	78,540,172	7.94E-10	0.226	0.210				*WWOX*	Body

cg16590189	5	175,299,798	0.018783	0.226	0.263				*CPLX2*	5'UTR
cg21697381	17	33,759,957	0.01629	0.226	0.163				*SLFN12*	TSS1500
cg01536803	7	157,968,578	2E-11	0.226	0.221				*PTPRN2*	Body
cg02372786	2	45,167,549	6.56E-05	0.226	0.230				*SIX3*	TSS1500
cg12091331	8	42,065,314	0.00046	0.226	0.142	3.39E-26	0.238		*PLAT*	TSS200
cg09269891	16	3,843,415	9.22E-07	0.226	0.209				*CREBBP*	Body
cg11430077	10	8,099,018	2.24E-08	0.226	0.225				*GATA3*	Body
cg05501584	15	92,936,306	0.025487	0.226	0.227				*ST8SIA2*	TSS1500
cg05545272	X	43,741,376	0.003411	0.226	0.081				*MAOB*	Body
cg03322057	1	172,114,419	5.85E-06	0.226	0.235				*DNM3*	Body
cg26968812	19	16,186,873	0.000565	0.226	0.220	3.88E-31	0.188		*TPM4*	Body
cg26950568	13	30,626,440	0.001374	0.226	0.206					
cg05544396	16	66,959,780	4.26E-07	0.226	0.217				*RRAD*	TSS1500
cg08877374	1	31,845,904	4.3E-05	0.226	0.187				*FABP3*	1stExon
cg09151742	12	122,018,897	0.010435	0.226	0.186				*KDM2B*	5'UTR
cg17573720	12	54,359,712	0.011001	0.226	0.283				*HOTAIR*	Body
cg18942298	20	42,809,578	5.45E-06	0.226	0.203				*JPH2*	Body
cg26310551	14	24,780,540	0.000498	0.226	0.201				*LTB4R2*	Body
cg10363397	Y	15,864,778	0.004176	0.226	0.192					
cg11525020	20	17,674,233	0.00554	0.226	0.237				*BANF2*	TSS200
cg27501686	12	2,184,874	1.45E-15	0.226	0.206				*CACNA1C*	Body
cg18645906	4	15,704,599	0.039162	0.226	0.256				*BST1*	1stExon
cg17566560	20	35,094,679	7.08E-24	0.226	0.228				*DLGAP4*	Body
cg26382173	14	105,994,265	0.00088	0.226	0.175				*TMEM121*	5'UTR
cg07333715	11	2,292,691	0.007157	0.226	0.234				*ASCL2*	TSS1500
cg14295696	X	135,230,717	1.91E-07	0.226	0.223				*FHL1*	Body
cg19748341	17	48,503,357	2.69E-06	0.226	0.154				*ACSF2*	TSS200
cg08353973	15	66,914,175	1.25E-07	0.226	0.223					
cg08993690	4	124,621,838	2.37E-09	0.226	0.215				*LOC285419*	Body
cg09220235	1	204,162,877	1.64E-13	0.226	0.221				*KISS1*	5'UTR
cg24780240	X	68,052,409	1.05E-22	0.226	0.225				*EFNB1*	Body
cg17091312	2	46,558,320	3.27E-12	0.226	0.217				*EPAS1*	Body
cg00049323	5	472,564	0.022668	0.226	0.255				*LOC25845*	Body
cg03416521	20	19,958,192	0.013361	0.226	0.134				*RIN2*	Body

cg08930904	17	80,297,325	0.000583	0.226	0.270					
cg01049870	14	95,601,239	2.58E-23	0.226	0.207				*DICER1*	5'UTR
cg04697946	2	3,654,582	1.32E-05	0.226	0.216				*COLEC11*	Body
cg20270653	7	70,962,720	0.002128	0.226	0.178				*WBSCR17*	Body
cg03018679	12	120,241,922	2.01E-14	0.226	0.210				*CIT*	Body
cg13372811	1	110,627,590	3.47E-07	0.226	0.219					
cg18684951	5	141,700,490	1.81E-10	0.226	0.220				*SPRY4*	5'UTR
cg19005955	5	132,938,694	5.46E-06	0.226	0.198				*FSTL4*	Body
cg20907806	12	570,249	7.02E-14	0.226	0.199				*B4GALNT3*	Body
cg25681339	17	1,174,148	0.000317	0.226	0.222				*BHLHA9*	1stExon
cg27385757	6	30,656,024	4.87E-13	0.226	0.193				*KIAA1949*	TSS1500
cg09299055	15	41,793,629	0.001983	0.226	0.128				*ITPKA*	Body
cg13702421	2	172,955,759	1.49E-06	0.226	0.236					
cg00586817	2	200,329,872	4.47E-08	0.226	0.217					
cg11616384	X	114,849,448	0.02083	0.226	0.205				*PLS3*	Body
cg14687029	11	41,519,708	0.031927	0.226	0.130					
cg16962008	7	48,075,950	0.003905	0.226	0.095				*C7orf57*	Body
cg09527670	19	5,059,110	7.36E-15	0.226	0.211				*KDM4B*	Body
cg12329275	10	11,912,597	7.81E-22	0.226	0.206				*C10orf47*	3'UTR
cg23882658	17	1,959,132	0.005077	0.226	0.219				*HIC1*	TSS1500
cg13616314	17	13,472,852	0.000191	0.226	0.199			Yes	*HS3ST3A1*	Body
cg25216696	8	11,565,351	0.000348	0.226	0.234	1.66E-21	0.182		*GATA4*	5'UTR
cg05387399	10	124,893,766	0.004346	0.226	0.253					
cg18052274	11	19,737,158	0.001621	0.226	0.172				*LOC100126784*	TSS1500
cg26660656	1	245,753,539	7.75E-24	0.226	0.196				*KIF26B*	Body
cg26778345	16	8,806,586	1.01E-06	0.226	0.236				*ABAT*	5'UTR
cg02898493	17	62,097,918	0.011763	0.226	0.217				*ICAM2*	5'UTR
cg02540827	2	201,221,975	1.74E-15	0.226	0.241				*SPATS2L*	5'UTR
cg02793948	11	68,095,662	0.001843	0.226	0.153				*LRP5*	Body
cg12350762	18	55,106,285	0.044447	0.226	0.211				*ONECUT2*	Body
cg13292607	10	126,751,950	0.049258	0.226	0.234				*CTBP2*	5'UTR
cg16719560	2	85,811,421	5.55E-05	0.226	0.222				*VAMP5*	TSS200
cg23492779	6	36,928,572	0.000105	0.226	0.203				*PI16*	Body
cg06409824	6	30,227,825	4.36E-08	0.226	0.224				*HLA-L*	Body

cg10643691	8	95,246,514	0.023361	0.226	0.283					
cg15027300	19	38,877,874	5.63E-16	0.226	0.184				*GGN*	Body
cg03175771	16	30,661,534	0.000606	0.226	0.244				*PRR14*	TSS1500
cg18321976	12	53,893,000	7.77E-05	0.226	0.132				*MAP3K12*	5'UTR
cg22268231	19	50,922,484	0.004335	0.226	0.224	0.00607046	0.050		*SPIB*	Body
cg24178740	2	219,850,465	5.13E-14	0.226	0.183	1.89E-09	0.081		*FEV*	TSS200
cg25734913	5	172,672,390	0.000183	0.226	0.217					
cg09336406	2	20,068,906	0.007084	0.226	0.125					
cg11823511	1	91,183,697	0.037985	0.226	0.255				*BARHL2*	TSS1500
cg00318111	7	6,010,084	0.000413	0.226	0.235				*RSPH10B*	5'UTR
cg04065086	1	151,104,186	0.001858	0.226	0.226				*SEMA6C*	3'UTR
cg08965888	11	44,341,446	0.002453	0.226	0.236					
cg09650907	17	71,224,983	0.017794	0.226	0.273				*FAM104A*	Body
cg11320187	20	33,578,094	1.7E-16	0.226	0.231				*MYH7B*	Body
cg04159302	5	150,162,068	0.003928	0.226	0.225				*C5orf62*	Body
cg12702354	1	77,043,128	4.21E-09	0.226	0.213				*ST6GALNAC3*	Body
cg25271892	11	62,690,462	3.86E-10	0.226	0.211				*CHRM1*	TSS1500
cg03447967	2	45,163,099	1.49E-09	0.226	0.209					
cg03648020	1	931,327	1.17E-11	0.226	0.199					
cg20939662	9	126,777,160	8E-08	0.226	0.226				*LHX2*	Body
cg18106312	14	51,560,488	0.038475	0.226	0.219				*TRIM9*	Body
cg26233253	22	31,032,613	6.27E-08	0.226	0.182	8.14E-17	0.139		*SLC35E4*	1stExon
cg27527922	1	6,260,181	6.53E-05	0.226	0.189				*RPL22*	TSS1500
cg10613707	5	3,394,463	1.35E-07	0.226	0.209					
cg11983196	1	98,518,557	1.16E-14	0.226	0.211					
cg00124095	3	72,305,082	0.024932	0.226	0.088					
cg02192520	12	56,114,155	5.67E-05	0.226	0.254	3.13E-21	0.252		*RDH5*	1stExon
cg04598121	8	57,358,505	0.000329	0.226	0.244				*PENK*	Body
cg07247419	20	21,376,484	0.00052	0.226	0.242				*NKX2-4*	3'UTR
cg07809589	2	66,664,535	0.024259	0.226	0.293				*MEIS1*	Body
cg16810031	17	38,024,146	1.91E-11	0.226	0.234				*ZPBP2*	TSS1500
cg21249091	2	173,204,540	1.98E-10	0.226	0.212					
cg23155717	8	145,033,622	2.85E-10	0.226	0.207				*PLEC1*	Body
cg27657333	12	30,975,867	0.000429	0.226	0.219					

cg00426822	1	39,546,536	7.39E-12	0.226	0.228				*MACF1*	TSS1500
cg01042246	2	171,572,902	0.048811	0.226	0.185				*SP5*	Body
cg13099330	3	139,257,799	1.52E-06	0.226	0.228	1.31E-16	0.152		*RBP1*	Body
cg03733831	X	17,653,562	3.81E-08	0.226	0.233				*NHS*	1stExon
cg13229972	1	110,335,018	8.85E-09	0.226	0.226					
cg19264571	18	10,454,085	5.56E-13	0.226	0.225	3.09E-19	0.173		*APCDD1*	TSS1500
cg07983905	20	3,073,561	0.002332	0.226	0.139					
cg01170045	14	60,982,832	5.27E-10	0.226	0.205					
cg04228468	3	164,913,627	0.000112	0.226	0.278				*SLITRK3*	5'UTR
cg18587340	20	39,995,654	8.56E-06	0.226	0.191				*EMILIN3*	TSS200
cg01705587	1	149,889,364	9.06E-05	0.226	0.266				*SV2A*	5'UTR
cg10086659	2	177,040,226	6.56E-06	0.226	0.161					
cg26683259	5	77,943,417	0.003526	0.226	0.182				*LHFPL2*	5'UTR
cg02311193	17	46,660,002	2.2E-11	0.226	0.204					
cg14439622	18	19,752,292	4.98E-09	0.226	0.197				*GATA6*	Body
cg25109721	17	48,125,064	1.56E-07	0.226	0.233					
cg25195334	8	23,710,946	0.001815	0.226	0.186			Yes	*STC1*	Body
cg24234651	12	75,728,471	0.001031	0.226	0.185				*GLIPR1L1*	5'UTR
cg25397973	6	5,996,562	0.009617	0.226	0.181					
cg03883256	11	119,235,802	6.79E-05	0.226	0.223				*USP2*	TSS1500
cg10308785	17	46,673,625	1.05E-07	0.226	0.215				*LOC404266*	Body
cg01158277	11	111,783,327	0.004627	0.226	0.321				*CRYAB*	TSS1500
cg14693194	18	6,415,252	0.011706	0.226	0.209				*L3MBTL4*	TSS1500
cg13615136	9	116,362,889	1.01E-11	0.226	0.218					
cg25147158	4	165,898,707	2.2E-08	0.226	0.223				*TRIM61*	1stExon
cg04141796	5	42,950,062	0.00142	0.226	0.186					
cg13619054	9	129,370,554	0.007861	0.226	0.117					
cg26371327	1	16,473,143	6.84E-15	0.226	0.220				*EPHA2*	Body
cg03462322	16	126,397	3.4E-10	0.226	0.223				*MPG*	TSS1500
cg11354105	2	25,475,805	1.91E-05	0.226	0.206				*DNMT3A*	Body
cg12777520	9	129,387,303	0.029307	0.226	0.256				*LMX1B*	Body
cg24253904	1	201,644,923	0.000782	0.226	0.233			Yes	*NAV1*	Body
cg03341413	15	68,125,566	0.000621	0.226	0.234				*LBXCOR1*	Body
cg10572274	12	104,697,224	0.002037	0.226	0.158				*TXNRD1*	5'UTR

cg13114921	12	30,976,001	4.23E-05	0.226	0.215					
cg16516639	1	111,050,810	6.25E-09	0.226	0.223					
cg20122518	7	810,958	0.000267	0.226	0.218				*HEATR2*	Body
cg02099390	3	31,959,312	8.96E-05	0.226	0.231				*OSBPL10*	Body
cg03456938	16	67,197,084	0.002553	0.226	0.281				*HSF4*	TSS1500
cg12351310	5	52,937,992	0.001998	0.226	0.227				*NDUFS4*	Body
cg05072470	13	100,643,858	0.041752	0.226	0.269					
cg07387286	8	39,172,120	0.000724	0.226	0.244				*ADAM5P*	TSS200
cg20301308	1	65,534,742	2.37E-11	0.226	0.197					
cg21235078	9	135,645,974	0.013613	0.226	0.219				*C9orf98*	Body
cg26115667	14	103,294,656	6.13E-05	0.226	0.194				*TRAF3*	5'UTR
cg09384035	8	104,131,768	8.63E-09	0.226	0.219					
cg09601505	12	109,162,445	2E-08	0.226	0.180					
cg11354906	4	154,710,371	0.013318	0.226	0.290	3.17E-16	0.159		*SFRP2*	TSS200
cg17209471	22	18,558,411	2.13E-29	0.226	0.227					
cg17552116	3	126,721,513	8.28E-09	0.226	0.248				*PLXNA1*	Body
cg00810971	2	65,085,764	1.7E-14	0.226	0.230					
cg05386769	2	218,681,558	2.16E-08	0.226	0.236				*TNS1*	Body
cg18827470	15	75,471,200	8.26E-05	0.226	0.217					
cg18616243	19	7,542,191	3.8E-109	0.226	0.217				*PEX11G*	Body
cg06788790	17	55,123,356	0.00019	0.226	0.223				*RNF126P1*	Body
cg14518658	22	37,726,676	3.2E-06	0.226	0.234					
cg09812070	4	46,995,689	0.019496	0.226	0.223				*GABRA4*	TSS200
cg00149716	20	13,200,069	1.89E-07	0.226	0.193					
cg05845376	5	140,683,632	3.34E-07	0.226	0.225				*SLC25A2*	TSS200
cg20802392	1	150,780,684	1.71E-07	0.226	0.220	6.27E-15	0.141		*CTSK*	5'UTR
cg26002259	14	36,976,428	0.020706	0.226	0.278				*SFTA3*	Body
cg19622874	14	85,998,492	0.007008	0.225	0.287				*FLRT2*	5'UTR
cg20029745	18	28,621,985	0.022642	0.225	0.167				*DSC3*	Body
cg14137381	5	502,291	7.21E-05	0.225	0.256			Yes	*SLC9A3*	Body
cg18844301	2	220,379,000	0.019935	0.225	0.209				*ACCN4*	5'UTR
cg03851159	10	118,891,090	0.02923	0.225	0.301				*VAX1*	3'UTR
cg07826859	7	45,020,086	7.27E-11	0.225	0.200				*MYO1G*	TSS1500
cg27361370	3	157,813,327	0.004368	0.225	0.187					

cg04972459	1	9,460,204	1.19E-10	0.225	0.228					
cg22267770	6	10,422,139	0.001121	0.225	0.235					
cg10671668	12	4,919,230	0.013119	0.225	0.127				*KCNA6*	1stExon
cg02426623	12	3,259,078	2.21E-17	0.225	0.204				*TSPAN9*	5'UTR
cg19577080	4	143,470,771	1.11E-07	0.225	0.207				*INPP4B*	5'UTR
cg22256027	4	4,863,016	3.08E-06	0.225	0.225				*MSX1*	Body
cg06329735	11	75,139,390	0.021569	0.225	0.246				*KLHL35*	Body
cg12686273	16	3,085,867	7.2E-11	0.225	0.234				*CCDC64B*	TSS1500
cg16084857	X	130,216,575	0.039532	0.225	0.164				*FLJ30058*	Body
cg23432345	7	27,196,153	0.011985	0.225	0.189	5.51E-16	0.177		*HOXA7*	1stExon
cg17842189	22	47,425,596	6.38E-10	0.225	0.225				*TBC1D22A*	Body
cg20626840	2	38,201,011	6.7E-06	0.225	0.216				*FAM82A1*	Body
cg23197405	12	54,451,938	6.12E-11	0.225	0.200				*FLJ12825*	TSS200
cg06039988	11	65,158,399	7.55E-09	0.225	0.203				*FRMD8*	Body
cg09147777	11	105,480,771	0.00011	0.225	0.192				*GRIA4*	TSS200
cg09828714	22	29,875,578	0.013865	0.225	0.290				*NEFH*	TSS1500
cg17024482	17	54,230,730	1.87E-16	0.225	0.206				*ANKFN1*	TSS200
cg18917486	15	74,093,431	4.74E-05	0.225	0.208					
cg23855319	4	2,062,392	0.000169	0.225	0.237				*NAT8L*	Body
cg01654862	3	159,480,947	0.000806	0.225	0.217				*SCHIP1*	Body
cg12931554	8	37,119,428	4.23E-07	0.225	0.211					
cg19514721	5	141,813,298	4.72E-13	0.225	0.206					
cg22637867	19	12,624,466	0.000393	0.225	0.227				*ZNF709*	5'UTR
cg04569615	11	20,622,198	0.000419	0.225	0.280				*SLC6A5*	Body
cg05560472	X	99,986,046	0.016024	0.225	0.261				*SYTL4*	5'UTR
cg15329133	5	135,467,951	1.72E-10	0.225	0.194				*SMAD5*	TSS1500
cg18932968	11	102,576,469	2.28E-08	0.225	0.219				*MMP27*	TSS200
cg19672546	8	145,025,123	4.39E-09	0.225	0.221				*PLEC1*	Body
cg02495604	1	40,158,076	0.000208	0.225	0.235				*HPCAL4*	TSS1500
cg03024720	8	9,765,601	2.81E-05	0.225	0.247					
cg07404485	7	94,953,653	8.77E-08	0.225	0.193	1.65E-15	0.159		*PON1*	Body
cg16762684	18	74,820,493	0.007858	0.225	0.190				*MBP*	5'UTR
cg00537673	6	43,141,823	9.03E-15	0.225	0.199				*SRF*	Body
cg10325497	1	32,739,049	1.65E-09	0.225	0.206				*LCK*	5'UTR

cg22667178	13	44,947,976	0.005889	0.225	0.222				*SERP2*	TSS200
cg22915945	8	24,151,625	1.81E-05	0.225	0.228				*ADAM28*	1stExon
cg23554686	X	40,122,371	8.85E-09	0.225	0.225					
cg24954895	5	140,800,761	0.004109	0.225	0.280				*PCDHGA4*	Body
cg08159989	1	205,326,071	3.55E-19	0.225	0.207				*KLHDC8A*	TSS200
cg09001939	2	11,810,534	0.002647	0.225	0.198				*NTSR2*	TSS1500
cg19630681	3	64,429,607	5.17E-08	0.225	0.220					
cg06421774	5	2,066,690	0.003426	0.225	0.264					
cg20777398	6	28,956,244	5.3E-05	0.225	0.213					
cg19258425	3	181,428,280	3.8E-05	0.225	0.220				*SOX2OT*	Body
cg00028308	12	54,090,754	1.87E-05	0.225	0.236					
cg10658307	11	95,071,108	3.12E-15	0.225	0.225					
cg07282889	15	89,912,141	0.01219	0.225	0.232					
cg13966575	8	127,592,341	6.33E-15	0.225	0.205					
cg17933493	X	153,532,656	5.43E-14	0.225	0.218				*TKTL1*	TSS1500
cg02080857	1	85,279,323	7.58E-05	0.225	0.212				*LPAR3*	3'UTR
cg03941587	4	123,747,634	2.2E-06	0.225	0.219				*FGF2*	TSS1500
cg25688583	15	57,510,460	8.7E-06	0.225	0.245				*TCF12*	TSS1500
cg01899676	12	106,988,413	1.85E-07	0.225	0.229				*RFX4*	Body
cg26840437	5	1,851,071	4.83E-09	0.225	0.183					
cg27362525	17	5,019,638	0.016586	0.225	0.274				*ZNF232*	5'UTR
cg00961418	11	8,616,172	0.000191	0.225	0.181				*STK33*	TSS1500
cg12539415	2	177,016,167	1.37E-06	0.225	0.193				*HOXD4*	5'UTR
cg04002794	3	171,805,805	1.32E-05	0.225	0.249				*FNDC3B*	5'UTR
cg09238992	10	23,484,228	0.003411	0.225	0.273					
cg16228323	10	43,818,243	0.032044	0.225	0.196					
cg27440150	22	24,561,080	5.55E-36	0.225	0.217				*CABIN1*	Body
cg08179525	15	29,033,879	0.000305	0.225	0.236					
cg18850728	18	48,346,838	6.16E-11	0.225	0.213				*MRO*	TSS1500
cg17739428	19	23,598,854	0.00795	0.225	0.139					
cg10588211	1	162,792,246	0.03166	0.225	0.351					
cg18944010	1	119,522,855	0.001211	0.225	0.267				*TBX15*	5'UTR
cg20813589	11	504,930	0.008648	0.225	0.193				*RNH1*	5'UTR
cg08661007	3	79,817,232	0.016963	0.225	0.267				*ROBO1*	TSS200

cg08927016	7	156,796,145	0.00109	0.225	0.218					
cg00181327	13	31,439,278	2.2E-10	0.225	0.219					
cg22969524	17	41,909,716	1.29E-05	0.225	0.201				*MPP3*	5'UTR
cg26704293	12	125,073,750	6.83E-06	0.225	0.213					
cg06008926	5	170,740,673	0.021749	0.225	0.300					
cg13459217	1	205,326,437	1.56E-13	0.225	0.233				*KLHDC8A*	TSS1500
cg15829654	6	108,482,863	0.001647	0.225	0.256					
cg01375263	X	57,163,746	2.77E-07	0.225	0.210				*SPIN2A*	5'UTR
cg09380805	3	169,384,034	9.88E-07	0.225	0.220					
cg09777237	7	73,445,236	2.35E-15	0.225	0.212				*ELN*	Body
cg15821319	20	37,434,158	0.000255	0.225	0.145				*PPP1R16B*	TSS200
cg02441765	13	33,590,838	0.003779	0.225	0.126				*KL*	1stExon
cg09308580	2	43,405,947	0.001813	0.225	0.233					
cg15247509	10	48,416,977	1.28E-08	0.225	0.236				*GDF2*	TSS200
cg20921890	18	19,753,208	7.7E-14	0.225	0.206				*GATA6*	Body
cg14157107	9	16,704,404	2.8E-07	0.225	0.210				*BNC2*	Body
cg27119919	11	60,684,332	3.61E-07	0.225	0.199				*TMEM109*	5'UTR
cg07649862	16	88,897,202	4.97E-44	0.225	0.220				*GALNS*	Body
cg17203647	8	25,897,201	1.65E-05	0.225	0.210				*EBF2*	Body
cg20168806	6	10,382,828	0.007146	0.225	0.245					
cg01330221	4	57,522,689	0.030381	0.225	0.243			Yes	*HOPX*	5'UTR
cg24023258	8	143,781,297	4.46E-11	0.225	0.227				*LY6K*	TSS1500
cg16973098	13	95,359,203	0.004281	0.225	0.180					
cg00468380	8	11,549,521	6.27E-05	0.225	0.223					
cg17838420	5	1,877,906	4.02E-09	0.225	0.204				*IRX4*	3'UTR
cg06872964	1	79,085,250	0.003033	0.225	0.196				*IFI44L*	TSS1500
cg07162085	10	95,751,894	0.000162	0.225	0.242					
cg10078829	19	51,415,452	2.13E-07	0.225	0.226				*KLK4*	TSS1500
cg23214071	6	32,627,784	0.022335	0.225	0.151			Yes	*HLA-DQB1*	3'UTR
cg04586332	22	45,659,810	0.020646	0.225	0.246					
cg13303358	1	245,644,687	1.44E-06	0.225	0.172				*KIF26B*	Body
cg16166968	1	120,439,036	5.07E-08	0.225	0.237				*ADAM30*	1stExon
cg01443452	19	36,024,439	4.26E-10	0.225	0.214	2.8E-08	0.096		*GAPDHS*	1stExon
cg06631160	16	29,086,456	0.000122	0.225	0.151				*RRN3P2*	Body

cg23413506	20	3,218,579	6.77E-17	0.225	0.207				*SLC4A11*	TSS1500
cg23771366	11	86,510,998	0.000589	0.225	0.127				*PRSS23*	TSS1500
cg24014143	13	70,682,127	0.019902	0.225	0.269				*KLHL1*	1stExon
cg04602747	6	118,273,169	0.041625	0.225	0.050				*SLC35F1*	Body
cg13790353	11	85,565,559	0.012126	0.225	0.170				*CCDC83*	TSS1500
cg14701491	3	127,634,449	0.040867	0.225	0.203					
cg17104824	2	177,014,959	9.03E-07	0.225	0.173				*HOXD4*	TSS1500
cg07180458	1	19,600,912	0.002514	0.225	0.163				*AKR7L*	TSS1500
cg06530441	22	51,016,950	3.17E-08	0.225	0.237				*CPT1B*	TSS200
cg07167423	1	36,042,986	1.47E-05	0.225	0.226				*TFAP2E*	Body
cg07138399	1	200,272,215	1.43E-05	0.225	0.198					
cg03555299	2	206,830,681	0.000126	0.225	0.223					
cg13223107	6	50,810,715	9.92E-05	0.225	0.248				*TFAP2B*	Body
cg17941572	1	63,784,454	0.00491	0.225	0.247					
cg23088510	10	126,428,199	0.00033	0.225	0.219				*FAM53B*	5'UTR
cg01300341	6	137,311,021	0.000789	0.225	0.151				*NHEG1*	Body
cg27326452	3	138,668,931	0.036772	0.225	0.223				*C3orf72*	Body
cg06511312	1	3,459,949	0.003002	0.225	0.189			Yes	*MEGF6*	Body
cg07489048	14	29,236,898	0.000274	0.225	0.268				*FOXG1*	1stExon
cg15165122	2	71,206,291	3.5E-06	0.225	0.210				*ANKRD53*	Body
cg13971892	5	146,258,512	0.022139	0.225	0.208				*PPP2R2B*	TSS200
cg01624571	15	67,417,697	8.6E-11	0.225	0.201				*SMAD3*	Body
cg04569429	17	37,024,625	2.02E-05	0.225	0.235				*LASP1*	TSS1500
cg05048976	3	55,516,243	0.000113	0.225	0.189				*WNT5A*	Body
cg05346256	12	25,959,372	2.67E-06	0.225	0.234					
cg07545037	12	54,423,920	2.75E-10	0.225	0.193				*HOXC4*	5'UTR
cg10184289	2	64,870,490	1.72E-06	0.225	0.196				*SERTAD2*	5'UTR
cg22660542	12	54,402,431	5.17E-05	0.225	0.195				*HOXC8*	TSS1500
cg11772661	10	118,765,335	0.011263	0.225	0.111				*KIAA1598*	TSS1500
cg23713520	16	57,318,087	1.72E-05	0.225	0.185	3.03E-13	0.180		*PLLP*	Body
cg02725065	15	101,718,610	4.14E-07	0.225	0.212				*CHSY1*	Body
cg01476969	19	719,323	3.72E-13	0.225	0.215				*PALM*	Body
cg17280106	17	77,767,084	5.99E-06	0.225	0.260					
cg18273417	1	153,518,418	2.86E-09	0.225	0.227				*S100A4*	TSS200

cg13876325	17	16,190,098	2.02E-07	0.225	0.225				*PIGL*	Body
cg15268136	1	44,031,309	8.82E-06	0.225	0.230				*PTPRF*	Body
cg20989443	1	230,301,268	9.23E-19	0.225	0.216				*GALNT2*	Body
cg04367197	11	13,300,320	0.007626	0.225	0.146				*ARNTL*	5'UTR
cg11089595	8	85,094,437	0.006789	0.225	0.184				*RALYL*	TSS1500
cg17328631	3	124,609,514	1.34E-07	0.225	0.213					
cg14018429	9	129,138,146	6.51E-14	0.225	0.219				*FAM125B*	Body
cg22825644	7	29,186,347	0.001964	0.225	0.226				*CPVL*	TSS200
cg18397450	14	105,830,631	0.04818	0.225	0.104				*PACS2*	Body
cg09566763	17	7,553,760	0.002561	0.225	0.208				*ATP1B2*	TSS1500
cg17693604	19	3,046,805	4.12E-23	0.225	0.215				*TLE2*	5'UTR
cg00008629	9	115,093,661	2.85E-10	0.225	0.208				*ROD1*	Body
cg06891491	X	134,429,749	1.63E-05	0.225	0.211				*ZNF75D*	5'UTR
cg06901711	1	146,649,635	0.018804	0.225	0.199				*PDIA3P*	Body
cg14331316	9	99,139,396	1.92E-07	0.225	0.210				*SLC35D2*	Body
cg19944876	17	8,905,866	5.59E-05	0.225	0.211					
cg27319216	8	6,693,540	5.09E-09	0.225	0.225				*XKR5*	TSS1500
cg27417609	16	68,676,806	6.46E-06	0.225	0.198				*CDH3*	TSS1500
cg02197277	16	21,245,235	1.38E-08	0.225	0.195				*ANKS4B*	Body
cg16232126	2	108,603,005	0.01482	0.225	0.195	6.25E-17	0.183		*SLC5A7*	1stExon
cg22083053	4	13,541,444	0.022967	0.225	0.264					
cg27149973	17	74,868,725	2.89E-09	0.225	0.239				*MGAT5B*	Body
cg17063780	12	103,816,811	0.000325	0.225	0.184				*C12orf42*	Body
cg24211908	17	72,443,401	3.68E-05	0.225	0.251				*GPRC5C*	3'UTR
cg06616245	12	57,854,236	3.19E-06	0.225	0.183	2.9E-13	0.061		*GLI1*	5'UTR
cg24451872	1	32,177,995	6.34E-05	0.225	0.240			Yes		
cg18147366	1	207,669,544	0.000468	0.225	0.271				*CR1*	5'UTR
cg18736448	8	134,087,171	0.00029	0.225	0.237				*TG*	Body
cg09580336	1	116,915,122	0.003831	0.225	0.178	2.22E-14	0.104		*ATP1A1*	TSS1500
cg10508317	17	76,355,146	0.000233	0.225	0.225				*SOCS3*	Body
cg18481230	2	155,555,347	0.018939	0.225	0.255				*KCNJ3*	1stExon
cg12691631	4	23,891,835	0.043393	0.225	0.283				*PPARGC1A*	TSS200
cg13710969	17	79,393,463	0.000774	0.225	0.083				*BAHCC1*	Body
cg08062822	X	103,356,845	0.016599	0.225	0.105				*MCART6*	5'UTR

cg03602029	6	137,814,078	7.23E-05	0.225	0.242				*OLIG3*	1stExon
cg15440661	2	43,395,871	8.64E-16	0.225	0.229					
cg19620724	6	50,810,852	0.001049	0.225	0.249				*TFAP2B*	Body
cg14050053	17	33,416,834	4.31E-07	0.225	0.190				*RFFL*	TSS1500
cg14686919	20	61,584,159	0.000122	0.225	0.193				*SLC17A9*	1stExon
cg02994246	1	21,587,174	7.77E-12	0.225	0.209				*ECE1*	Body
cg11605835	5	3,597,695	0.004652	0.225	0.262				*IRX1*	Body
cg20547575	7	69,065,351	0.008275	0.225	0.135				*AUTS2*	Body
cg13679772	14	89,933,549	4.95E-07	0.225	0.195				*FOXN3*	5'UTR
cg10497692	1	21,996,199	0.018514	0.225	0.155				*RAP1GAP*	TSS1500
cg12902206	3	52,351,487	1.97E-06	0.225	0.251				*DNAH1*	5'UTR
cg14704669	10	32,046,612	6.1E-20	0.225	0.226					
cg11858226	7	2,548,262	0.003104	0.225	0.226					
cg17856063	1	146,543,992	9.16E-10	0.225	0.191					
cg21371809	10	14,052,028	0.000262	0.225	0.176				*FRMD4A*	Body
cg04457317	3	50,567,932	0.001429	0.224	0.202					
cg09171882	12	75,874,499	0.02402	0.224	0.299				*GLIPR1*	TSS200
cg11604182	5	177,433,673	0.000162	0.224	0.236					
cg24993576	6	168,502,767	1.77E-05	0.224	0.214					
cg13251484	14	74,180,217	0.004694	0.224	0.180				*PNMA1*	1stExon
cg21092808	2	177,029,850	0.000992	0.224	0.207				*HOXD3*	5'UTR
cg07056299	7	73,393,148	1.79E-09	0.224	0.220					
cg15017278	16	127,449	0.004581	0.224	0.153				*MPG*	5'UTR
cg16297569	1	92,952,517	0.000188	0.224	0.183				*GFI1*	TSS1500
cg25577212	7	24,740,509	2.6E-09	0.224	0.230				*DFNA5*	Body
cg06399427	2	161,504,772	0.002093	0.224	0.169					
cg09261593	1	2,058,612	2.76E-12	0.224	0.212				*PRKCZ*	5'UTR
cg15269394	17	40,672,083	2.42E-09	0.224	0.224				*ATP6V0A1*	Body
cg17988320	7	30,361,459	1.46E-09	0.224	0.201				*ZNRF2*	Body
cg09103619	15	79,575,048	0.045997	0.224	0.211				*ANKRD34C*	TSS200
cg02762440	5	71,014,792	1.93E-08	0.224	0.215				*CARTPT*	TSS200
cg04551619	1	10,692,799	1.7E-28	0.224	0.243					
cg06386478	8	19,460,136	0.000359	0.224	0.201				*CSGALNACT1*	TSS200
cg06473276	6	39,399,221	0.002119	0.224	0.174				*KIF6*	Body

cg23851027	19	51,857,538	4.37E-06	0.224	0.174				*ETFB*	1stExon
cg10069809	2	45,028,604	5.38E-07	0.224	0.224					
cg24472823	7	51,296,131	1.39E-12	0.224	0.236				*COBL*	Body
cg27423216	12	5,543,269	0.000157	0.224	0.226				*NTF3*	Body
cg04464085	6	30,923,327	0.014043	0.224	0.291					
cg19201009	10	99,475,406	4.67E-23	0.224	0.212				*MARVELD1*	Body
cg25174438	4	85,403,915	0.000187	0.224	0.235					
cg09473359	13	24,477,933	4.07E-11	0.224	0.227					
cg26923410	7	30,951,432	9.46E-05	0.224	0.175				*AQP1*	TSS200
cg04309093	14	92,980,608	0.00427	0.224	0.152				*RIN3*	Body
cg17299456	11	10,696,311	0.001177	0.224	0.231				*MRVI1*	5'UTR
cg22460123	12	52,638,294	9.91E-13	0.224	0.198				*KRT7*	Body
cg00868523	19	54,387,036	0.002952	0.224	0.277				*PRKCG*	Body
cg02858132	3	136,469,005	1.74E-12	0.224	0.244				*STAG1*	5'UTR
cg12845268	10	63,657,363	1.59E-09	0.224	0.191					
cg15559833	2	218,829,609	0.005994	0.224	0.195			Yes		
cg08461692	17	8,481,454	5.37E-13	0.224	0.197				*MYH10*	Body
cg09100338	8	48,676,054	0.005036	0.224	0.172					
cg14176930	6	10,884,891	2.02E-06	0.224	0.247					
cg18652721	5	2,754,074	0.043891	0.224	0.204				*C5orf38*	Body
cg09267051	7	100,943,468	0.015001	0.224	0.216					
cg17651821	6	27,841,683	0.017727	0.224	0.235	4.36E-11	0.178		*HIST1H4L*	TSS1500
cg25751198	12	109,729,573	0.00375	0.224	0.172				*FOXN4*	Body
cg04792712	8	17,271,105	0.001121	0.224	0.154				*MTMR7*	TSS200
cg05416911	12	7,176,049	5.33E-05	0.224	0.211				*C1S*	Body
cg09387749	2	177,028,680	0.030618	0.224	0.227				*HOXD3*	TSS200
cg13985437	11	76,381,044	3.58E-07	0.224	0.200				*LRRC32*	5'UTR
cg15854394	8	22,769,830	2.58E-09	0.224	0.201				*PEBP4*	Body
cg23419907	2	220,154,319	1.45E-10	0.224	0.206					
cg13604794	17	48,050,338	3.1E-06	0.224	0.224				*DLX4*	Body
cg14594111	9	124,463,457	6.55E-13	0.224	0.200			Yes	*DAB2IP*	Body
cg18145937	5	132,010,740	1.83E-20	0.224	0.243				*IL4*	Body
cg14726637	1	207,669,308	1.82E-06	0.224	0.211	1.72E-20	0.202		*CR1*	TSS200
cg18455523	3	24,563,685	0.000414	0.224	0.153					

cg22871653	1	119,535,817	0.013534	0.224	0.227					
cg27345924	5	2,727,758	0.002649	0.224	0.252					
cg01558110	22	50,607,681	4.99E-21	0.224	0.237				*PANX2*	TSS1500
cg07519536	6	26,501,950	0.012806	0.224	0.207				*BTN1A1*	Body
cg27560864	11	118,024,339	6.41E-10	0.224	0.209	3.1E-19	0.140		*SCN4B*	TSS1500
cg15885672	17	59,488,116	8.07E-06	0.224	0.212				*C17orf82*	TSS1500
cg27264462	1	2,106,365	5.14E-05	0.224	0.260				*PRKCZ*	Body
cg17864646	17	80,297,148	1.17E-07	0.224	0.243					
cg26343958	12	5,539,662	0.000734	0.224	0.249					
cg10237817	1	154,519,846	2.15E-07	0.224	0.223				*TDRD10*	Body
cg02172819	10	79,261,503	1.53E-07	0.224	0.209				*KCNMA1*	Body
cg03810479	X	38,666,254	2.64E-12	0.224	0.212					
cg12111714	13	26,043,472	0.002845	0.224	0.190	3.1E-11	0.179		*ATP8A2*	Body
cg14096207	1	223,407,010	5.25E-05	0.224	0.248				*SUSD4*	Body
cg24569492	2	66,808,811	0.011121	0.224	0.239					
cg08368875	10	134,334,241	5.78E-09	0.224	0.237					
cg22598426	4	85,417,755	1.79E-07	0.224	0.207				*NKX6-1*	Body
cg23097006	20	25,063,817	0.009489	0.224	0.321	1.67E-11	0.174		*VSX1*	TSS1500
cg02117721	4	74,702,421	0.002768	0.224	0.082				*CXCL6*	5'UTR
cg06615445	17	36,759,100	9.38E-13	0.224	0.213				*SRCIN1*	Body
cg18055467	9	129,384,767	1.82E-06	0.224	0.222				*LMX1B*	Body
cg21814178	12	51,720,755	6.7E-06	0.224	0.226					
cg27148800	16	54,325,929	1.34E-06	0.224	0.187					
cg04566677	12	54,779,175	1.19E-09	0.224	0.211				*ZNF385A*	Body
cg26256223	8	145,106,582	1.46E-05	0.224	0.226				*OPLAH*	Body
cg11731868	17	7,344,050	0.039674	0.224	0.289				*FGF11*	Body
cg21831931	3	5,137,953	0.009143	0.224	0.222					
cg21940308	1	167,766,195	8.65E-07	0.224	0.202					
cg19403534	10	120,355,419	0.00058	0.224	0.229				*PRLHR*	TSS1500
cg26546537	8	122,651,555	0.015185	0.224	0.248				*HAS2*	5'UTR
cg10366797	12	54,441,509	6.64E-08	0.224	0.207				*HOXC4*	5'UTR
cg22540514	8	41,050,652	1.07E-09	0.224	0.213					
cg01919377	12	5,539,832	0.011762	0.224	0.238				*NTF3*	TSS1500
cg06405341	17	63,535,223	2.5E-14	0.224	0.223				*AXIN2*	Body

cg07213414	16	23,153,611	1.97E-24	0.224	0.227				*USP31*	Body
cg09682051	11	128,813,008	0.008165	0.224	0.130				*TP53AIP1*	TSS200
cg09694921	11	126,215,608	1.49E-13	0.224	0.201				*DCPS*	3'UTR
cg09773769	X	132,547,387	2.59E-12	0.224	0.187				*GPC4*	Body
cg17071855	17	3,819,880	8.07E-07	0.224	0.223				*P2RX1*	1stExon
cg03602233	16	75,144,040	7.37E-12	0.224	0.212				*ZNRF1*	3'UTR
cg08819022	22	18,531,447	1.01E-12	0.224	0.217					
cg09094355	X	3,632,358	0.002705	0.224	0.172				*PRKX*	TSS1500
cg25638611	11	70,508,420	5.88E-09	0.224	0.217				*SHANK2*	Body
cg21199165	2	45,166,884	8.44E-07	0.224	0.209					
cg23926436	17	12,133,847	4.33E-07	0.224	0.159					
cg00209038	2	48,982,683	0.025315	0.224	0.313				*LHCGR*	1stExon
cg08250880	11	86,526,022	0.000834	0.224	0.167					
cg14409032	9	129,445,219	0.000183	0.224	0.195				*LMX1B*	Body
cg20207763	10	13,392,088	0.004631	0.224	0.171					
cg00232453	6	31,864,574	1.11E-10	0.224	0.209				*EHMT2*	Body
cg03239925	18	77,230,795	3.65E-10	0.224	0.218				*NFATC1*	Body
cg22023627	8	20,125,819	1.11E-14	0.224	0.226					
cg24144574	7	653,445	5.37E-07	0.224	0.219				*PRKAR1B*	Body
cg01893695	14	77,370,443	7E-11	0.224	0.227					
cg21100518	6	29,595,002	5.13E-08	0.224	0.226				*GABBR1*	Body
cg11907729	6	55,443,757	0.026499	0.224	0.285				*HMGCLL1*	1stExon
cg02360514	19	45,975,990	2.81E-11	0.224	0.189				*FOSB*	Body
cg18504989	19	46,286,198	6.7E-06	0.224	0.235				*DMPK*	TSS1500
cg22331740	19	48,983,508	4.92E-13	0.224	0.226				*CYTH2*	3'UTR
cg04838832	10	72,502,473	0.00319	0.224	0.230				*ADAMTS14*	Body
cg05668853	17	27,044,169	0.000222	0.224	0.245	2.48E-22	0.165		*RAB34*	Body
cg24659328	3	58,563,666	1.97E-08	0.224	0.246				*FAM107A*	TSS200
cg06098693	11	70,002,283	6.27E-08	0.224	0.204				*ANO1*	Body
cg13577628	1	119,534,899	0.004377	0.224	0.187					
cg04054731	22	21,386,798	0.034902	0.224	0.081				*SLC7A4*	5'UTR
cg19615017	3	30,673,459	4.11E-19	0.224	0.222				*TGFBR2*	Body
cg20196291	10	116,164,849	1.79E-08	0.224	0.168				*AFAP1L2*	TSS1500
cg00208504	4	140,200,520	0.014417	0.224	0.147				*C4orf49*	Body

cg00961517	10	24,491,007	1.33E-05	0.224	0.223				*KIAA1217*	5'UTR
cg22065923	22	30,938,383	0.01214	0.224	0.179					
cg05228404	10	125,851,279	0.000886	0.224	0.279					
cg11947187	8	134,142,317	0.000324	0.224	0.221				*TG*	Body
cg03646189	5	2,756,101	0.032871	0.224	0.189					
cg16765487	16	89,548,175	9.39E-09	0.224	0.216				*ANKRD11*	5'UTR
cg25577130	17	58,226,188	2.58E-08	0.224	0.217				*CA4*	TSS1500
cg24001644	X	20,134,719	0.0101	0.224	0.199				*MAP7D2*	Body
cg20341504	2	54,900,998	0.028223	0.224	0.221					
cg22518208	14	85,997,469	0.004261	0.224	0.242				*FLRT2*	5'UTR
cg11746996	13	69,459,533	1.58E-08	0.224	0.216					
cg14067873	12	81,110,706	1.66E-17	0.224	0.204				*MYF5*	TSS200
cg19793499	7	70,096,378	1.94E-10	0.224	0.216				*AUTS2*	Body
cg16099804	5	83,680,253	0.005626	0.224	0.218				*EDIL3*	5'UTR
cg05030553	21	44,061,403	0.000448	0.224	0.181					
cg16422907	13	37,006,734	0.000109	0.224	0.193	8.97E-24	0.197		*CCNA1*	5'UTR
cg22141459	17	27,899,465	7.4E-17	0.224	0.214				*TP53I13*	Body
cg25623727	5	15,932,416	5.8E-10	0.224	0.208				*FBXL7*	Body
cg26612727	17	38,024,636	5.4E-06	0.224	0.184				*ZPBP2*	1stExon
cg09350411	7	157,667,935	5.67E-07	0.224	0.159				*PTPRN2*	Body
cg04915044	2	86,263,895	2.95E-05	0.224	0.238				*POLR1A*	Body
cg19640500	14	55,982,148	2.62E-09	0.224	0.224					
cg21958524	12	99,139,867	1.7E-06	0.224	0.147				*ANKS1B*	Body
cg24607642	17	42,345,612	0.001093	0.224	0.233			Yes	*SLC4A1*	TSS200
cg26867575	9	96,723,088	0.001935	0.224	0.241					
cg06174078	11	1,325,718	0.030567	0.224	0.206				*TOLLIP*	Body
cg16411668	4	123,704,747	1.18E-19	0.224	0.218					
cg20429250	17	33,814,359	0.005527	0.224	0.228				*SLFN12L*	Body
cg19256400	2	131,512,768	6.37E-09	0.224	0.246				*FAM123C*	TSS1500
cg17839166	5	53,751,272	1.53E-06	0.224	0.263				*HSPB3*	TSS200
cg08336287	10	11,206,892	5.43E-05	0.224	0.112				*CUGBP2*	TSS200
cg08128361	16	67,336,052	0.018391	0.224	0.210				*KCTD19*	Body
cg08188886	14	105,633,697	1.29E-07	0.224	0.181				*JAG2*	Body
cg12938003	4	109,092,724	0.044973	0.224	0.287				*LOC641518*	TSS1500

cg22304507	3	136,537,863	0.029447	0.224	0.168				*TMEM22*	1stExon
cg13990947	9	19,128,801	0.000652	0.224	0.178				*PLIN2*	TSS1500
cg22490454	2	468,934	2.86E-06	0.224	0.215					
cg04550823	1	87,617,916	0.003884	0.224	0.256				*LOC339524*	Body
cg07392601	9	133,413,095	0.000631	0.224	0.200					
cg21728840	19	57,829,009	1.54E-05	0.224	0.260					
cg24512005	11	3,014,291	7.09E-05	0.224	0.176				*NAP1L4*	TSS1500
cg01602153	8	59,058,660	2.36E-06	0.224	0.212				*FAM110B*	5'UTR
cg01992935	X	108,868,271	1.66E-05	0.224	0.240				*KCNE1L*	1stExon
cg14437996	9	91,794,687	2.73E-06	0.224	0.215				*SHC3*	TSS1500
cg02864688	1	11,752,342	4.37E-07	0.224	0.181				*MAD2L2*	TSS1500
cg11826961	17	38,221,639	2.31E-05	0.224	0.224				*THRA*	5'UTR
cg16691593	5	76,934,634	0.001394	0.224	0.244				*OTP*	TSS200
cg24095374	5	176,799,366	6.47E-10	0.224	0.195				*RGS14*	3'UTR
cg14842777	13	112,723,226	0.02484	0.224	0.192				*SOX1*	1stExon
cg00176210	8	41,654,967	4.98E-07	0.224	0.159	1.18E-10	0.109		*ANK1*	Body
cg03138928	1	226,004,826	0.000138	0.224	0.220				*EPHX1*	5'UTR
cg07589612	8	97,171,668	0.000107	0.224	0.269				*GDF6*	Body
cg05998607	2	119,067,928	0.001957	0.224	0.225					
cg06394621	10	29,236,369	7.52E-05	0.224	0.240					
cg21097036	6	127,840,640	0.025654	0.224	0.220				*C6orf174*	TSS200
cg04768602	8	99,960,148	1.03E-05	0.224	0.225				*OSR2*	5'UTR
cg10385303	14	36,983,146	0.014307	0.224	0.271				*SFTA3*	TSS200
cg17557141	11	43,947,677	4.22E-09	0.224	0.226					
cg20121142	7	19,158,001	0.003052	0.224	0.218				*TWIST1*	TSS1500
cg20880080	18	19,780,644	1.31E-44	0.224	0.210				*GATA6*	Body
cg14762436	7	24,917,750	9.88E-12	0.224	0.216				*OSBPL3*	Body
cg05907933	5	77,268,888	0.03914	0.224	0.216					
cg10221896	16	79,263,595	1.27E-08	0.224	0.240					
cg14974772	14	92,414,425	0.003553	0.224	0.094	2.49E-22	0.142		*FBLN5*	TSS1500
cg16105687	19	22,805,801	0.00574	0.224	0.233					
cg05134847	22	19,742,426	5.75E-09	0.224	0.201					
cg21658968	11	92,754,025	0.002494	0.224	0.177					
cg27637521	17	76,355,202	1.81E-05	0.224	0.222	4.85E-22	0.225		*SOCS3*	5'UTR

cg10214640	16	54,973,047	2.9E-05	0.224	0.211					
cg15016597	1	156,401,319	1.09E-12	0.224	0.203					
cg16286925	5	108,339,964	1.7E-10	0.224	0.217				*FER*	Body
cg20355644	10	29,747,440	1.47E-18	0.224	0.221				*SVIL*	Body
cg20970205	17	77,766,040	8.23E-06	0.224	0.215					
cg26099834	15	66,947,568	0.005371	0.224	0.142					
cg27141517	15	66,923,526	3.84E-12	0.224	0.209					
cg18397279	X	91,036,807	0.037838	0.224	0.210				*PCDH11X*	5'UTR
cg07853425	5	134,408,820	2.89E-09	0.224	0.233					
cg23369632	6	56,438,637	0.000201	0.224	0.266				*DST*	Body
cg01603234	1	218,990,709	6.85E-10	0.224	0.260					
cg23300372	5	71,015,432	0.028567	0.224	0.202	1.22E-22	0.262		*CARTPT*	Body
cg02683197	6	28,174,875	0.003281	0.224	0.243					
cg14062002	6	139,117,468	1.52E-05	0.224	0.225					
cg04638150	11	62,273,418	1.61E-09	0.223	0.202				*AHNAK*	Body
cg08660959	6	32,044,163	2.98E-37	0.223	0.225				*TNXB*	Body
cg09213124	17	38,599,508	0.000129	0.223	0.172				*IGFBP4*	TSS200
cg16549694	1	42,204,925	5.12E-05	0.223	0.199				*HIVEP3*	5'UTR
cg08581937	17	7,555,132	0.012838	0.223	0.149				*ATP1B2*	Body
cg04396896	11	125,364,611	0.005105	0.223	0.254				*FEZ1*	5'UTR
cg11055493	10	94,451,351	4.04E-05	0.223	0.196				*HHEX*	Body
cg12532095	6	166,260,964	0.008221	0.223	0.087					
cg02473866	12	28,128,105	0.022872	0.223	0.264					
cg01733638	4	140,201,100	0.042726	0.223	0.202				*C4orf49*	Body
cg13808083	9	96,716,527	8.29E-07	0.223	0.205				*BARX1*	Body
cg17633234	15	93,617,065	3.6E-07	0.223	0.178				*RGMA*	1stExon
cg04132452	3	160,475,035	3.26E-08	0.223	0.231				*PPM1L*	Body
cg15300753	11	48,057,861	2E-10	0.223	0.229				*PTPRJ*	Body
cg14216285	6	28,778,124	0.043181	0.223	0.080					
cg04364695	8	40,618,425	0.000595	0.223	0.194				*ZMAT4*	Body
cg14496753	9	124,979,184	1.09E-15	0.223	0.207				*LHX6*	Body
cg26180469	11	64,081,563	1.86E-32	0.223	0.225				*ESRRA*	Body
cg04042861	2	231,989,824	0.001629	0.223	0.192				*HTR2B*	TSS200
cg09655666	17	34,820,190	0.019203	0.223	0.303					

cg17790928	7	1,277,790	0.006671	0.223	0.169					
cg02711510	16	85,220,747	1.08E-17	0.223	0.226					
cg06173663	7	101,579,929	0.00661	0.223	0.189				*CUX1*	Body
cg11631427	17	59,478,579	0.014307	0.223	0.168				*TBX2*	Body
cg15572396	3	58,321,349	1.13E-09	0.223	0.214				*PXK*	Body
cg18576301	8	100,716,071	2.59E-13	0.223	0.216				*VPS13B*	Body
cg18890556	2	225,266,580	6.84E-07	0.223	0.198				*FAM124B*	5'UTR
cg01616394	12	125,138,263	5.72E-11	0.223	0.234					
cg06958211	15	40,532,180	1.76E-05	0.223	0.254	2.59E-23	0.282		*PAK6*	1stExon
cg06812795	6	32,829,025	0.031162	0.223	0.166					
cg16847637	7	130,020,328	0.000386	0.223	0.259				*CPA1*	1stExon
cg02457328	12	103,355,190	0.001234	0.223	0.202					
cg08966208	11	1,769,409	0.008606	0.223	0.148				*HCCA2*	Body
cg14422093	1	227,009,245	4.36E-10	0.223	0.222					
cg27141871	19	41,318,235	3.08E-14	0.223	0.224					
cg26814883	X	70,328,127	2.28E-07	0.223	0.211				*CXorf65*	TSS1500
cg06142946	11	17,373,124	0.048975	0.223	0.039				*DKFZp686O24166*	TSS200
cg09595185	17	46,667,812	1.99E-08	0.223	0.223				*LOC404266*	TSS200
cg11616676	7	87,848,143	0.022719	0.223	0.182				*SRI*	Body
cg24684077	12	30,948,712	3.19E-06	0.223	0.213					
cg27176129	20	43,937,155	0.000208	0.223	0.230				*MATN4*	TSS200
cg01515515	1	92,414,295	0.000481	0.223	0.249				*BRDT*	TSS1500
cg14323539	2	928,750	6.11E-20	0.223	0.201					
cg18406033	2	223,159,869	8.78E-07	0.223	0.186				*PAX3*	Body
cg19836471	10	79,638,108	0.000195	0.223	0.239				*DLG5*	Body
cg21282907	6	74,289,980	0.000386	0.223	0.251					
cg05267394	20	32,856,898	1.42E-05	0.223	0.175				*ASIP*	Body
cg11808936	4	7,894,203	0.000676	0.223	0.171				*AFAP1*	5'UTR
cg25711779	2	56,149,825	6.14E-11	0.223	0.202	7.72E-16	0.175		*EFEMP1*	5'UTR
cg03813151	6	149,778,058	1.16E-17	0.223	0.236				*ZC3H12D*	Body
cg12236164	9	972,622	0.006033	0.223	0.255					
cg01682285	14	81,422,160	5.65E-07	0.223	0.199				*TSHR*	1stExon
cg14814167	6	139,293,014	4.14E-19	0.223	0.229				*REPS1*	Body
cg18207741	15	60,294,120	0.00426	0.223	0.266					

cg27339452	3	136,537,752	0.009867	0.223	0.243				*TMEM22*	TSS200
cg01500844	19	11,530,060	0.001461	0.223	0.215				*RGL3*	TSS200
cg13773631	9	96,715,843	0.000666	0.223	0.235				*BARX1*	Body
cg20345562	2	175,197,892	0.004505	0.223	0.270					
cg21795544	5	42,993,440	0.010285	0.223	0.208					
cg00278547	2	177,037,212	4.86E-06	0.223	0.170				*HOXD3*	3'UTR
cg26814835	20	30,195,969	0.001502	0.223	0.227					
cg02100011	22	38,635,773	0.000136	0.223	0.251				*TMEM184B*	Body
cg03171770	10	43,393,728	0.012539	0.223	0.232					
cg06158650	1	119,522,637	0.011435	0.223	0.228				*TBX15*	5'UTR
cg07786776	17	40,250,289	0.048971	0.223	0.264					
cg11453076	6	137,812,735	1.12E-06	0.223	0.234					
cg21033965	1	178,455,800	4.84E-05	0.223	0.234					
cg05379541	7	27,252,541	1.85E-06	0.223	0.203					
cg15304404	6	163,826,969	0.006931	0.223	0.155					
cg00352218	6	19,691,654	0.012927	0.223	0.264					
cg24206827	2	214,098,074	8.69E-05	0.223	0.199					
cg00667862	1	91,300,446	0.037723	0.223	0.272					
cg01884681	17	70,339,149	1.18E-05	0.223	0.207					
cg12497374	3	14,339,534	4.85E-07	0.223	0.227					
cg19194167	15	57,846,210	2.18E-09	0.223	0.222					
cg11875044	10	8,546,509	0.019413	0.223	0.162					
cg01997270	22	44,418,018	6.55E-17	0.223	0.204				*PARVB*	Body
cg11978884	5	135,415,819	8.49E-05	0.223	0.163					
cg26597734	16	11,150,952	3.41E-05	0.223	0.221			Yes	*CLEC16A*	Body
cg11947981	2	182,322,749	0.03716	0.223	0.219				*ITGA4*	Body
cg22132509	3	160,475,102	4.71E-08	0.223	0.216				*PPM1L*	Body
cg26643856	6	32,977,349	5.06E-05	0.223	0.242	7.95E-26	0.143		*HLA-DOA*	1stExon
cg02108033	4	17,783,294	0.012037	0.223	0.198				*FAM184B*	TSS200
cg04992588	3	58,563,674	1.03E-09	0.223	0.216				*FAM107A*	TSS200
cg17291521	5	50,260,045	2.91E-08	0.223	0.195					
cg01281911	1	47,882,686	9.84E-05	0.223	0.293				*FOXE3*	1stExon
cg05754861	8	99,961,768	2.49E-07	0.223	0.213				*OSR2*	Body
cg16754788	8	97,803,770	1.08E-09	0.223	0.225				*PGCP*	Body

cg27183030	7	99,595,458	0.005188	0.223	0.192					
cg06036471	10	93,861,385	0.000182	0.223	0.198				*CPEB3*	Body
cg17733820	22	31,536,784	0.001061	0.223	0.156			Yes	*PLA2G3*	TSS1500
cg24718465	19	46,999,118	9.97E-05	0.223	0.238				*PNMAL2*	1stExon
cg05945290	17	39,792,147	0.001351	0.223	0.250					
cg15237152	19	11,649,631	1.15E-07	0.223	0.207				*CNN1*	1stExon
cg15681255	8	27,192,141	4.31E-10	0.223	0.197				*PTK2B*	5'UTR
cg18516557	2	177,004,015	3.22E-08	0.223	0.192					
cg07310406	22	50,524,374	2.47E-05	0.223	0.228				*MLC1*	TSS1500
cg19577548	20	48,178,928	1.39E-16	0.223	0.236				*PTGIS*	Body
cg04347414	1	2,084,519	5.26E-05	0.223	0.178				*PRKCZ*	Body
cg08348422	2	236,935,654	1.14E-10	0.223	0.216				*AGAP1*	Body
cg12731671	20	6,033,516	1.67E-06	0.223	0.198				*LRRN4*	5'UTR
cg06609197	8	80,524,611	0.014631	0.223	0.287				*STMN2*	Body
cg20690125	17	74,476,978	2.64E-08	0.223	0.227				*RHBDF2*	Body
cg16477774	11	65,325,249	0.008058	0.223	0.128				*LTBP3*	1stExon
cg03396354	19	46,519,603	0.008178	0.223	0.171				*CCDC61*	Body
cg18961681	7	32,467,657	0.009699	0.223	0.245					
cg00376022	7	19,183,880	0.008883	0.223	0.337					
cg01026503	20	62,025,366	8.31E-07	0.223	0.240					
cg04146745	3	42,947,263	0.000848	0.223	0.140				*ZNF662*	TSS1500
cg10425005	16	10,133,433	0.007672	0.223	0.246				*GRIN2A*	Body
cg11871421	17	6,679,476	0.026434	0.223	0.217				*FBXO39*	TSS200
cg14094242	4	56,020,979	1.67E-05	0.223	0.221					
cg23363971	12	56,415,271	0.000126	0.223	0.222				*IKZF4*	1stExon
cg02744524	3	138,655,827	0.038707	0.223	0.237					
cg09590377	2	8,597,389	0.003764	0.223	0.220					
cg13768290	2	63,285,491	0.000342	0.223	0.217					
cg19921377	19	7,580,209	1.88E-11	0.223	0.218			Yes	*ZNF358*	TSS1500
cg24377070	3	128,182,041	1.74E-17	0.223	0.195				*DNAJB8*	Body
cg00380924	6	166,967,377	1.69E-12	0.223	0.221				*RPS6KA2*	Body
cg10635443	8	23,557,486	3.1E-06	0.223	0.195					
cg15824100	1	77,980,005	8.99E-11	0.223	0.240				*AK5*	Body
cg26660800	17	59,480,926	6.93E-06	0.223	0.188				*TBX2*	Body

cg18085176	4	7,939,940	1.48E-08	0.223	0.235				*AFAP1*	5'UTR
cg21414905	6	11,901,382	1.65E-14	0.223	0.217					
cg17193155	6	27,203,343	0.000156	0.223	0.242					
cg00852595	12	99,548,437	2.34E-07	0.223	0.237				*ANKS1B*	1stExon
cg02197228	2	220,402,771	1.7E-08	0.223	0.250				*ACCN4*	3'UTR
cg06103243	3	193,923,516	2.62E-12	0.223	0.209					
cg10600534	15	60,971,920	2.53E-14	0.223	0.231				*RORA*	Body
cg18861547	1	22,196,887	1.2E-11	0.223	0.180				*HSPG2*	Body
cg16864658	3	42,306,150	0.024416	0.223	0.199	0.00000401	0.116		*CCK*	5'UTR
cg17327331	14	67,736,448	8.24E-10	0.223	0.236				*MPP5*	5'UTR
cg25771271	1	119,550,191	0.002452	0.223	0.254					
cg06207120	15	45,996,521	1.32E-07	0.223	0.207					
cg05583240	12	105,477,579	0.012044	0.223	0.169				*ALDH1L2*	Body
cg09970120	7	155,167,844	0.013839	0.223	0.194					
cg05218245	6	31,683,352	1.05E-23	0.223	0.222				*LY6G6D*	Body
cg26558799	17	80,806,435	3.56E-07	0.223	0.243				*TBCD*	Body
cg07533511	12	94,580,931	6.83E-08	0.223	0.206				*PLXNC1*	Body
cg10236239	2	108,994,514	9.84E-08	0.223	0.208	6.45E-13	0.125		*SULT1C4*	5'UTR
cg18112953	20	47,448,545	2.21E-12	0.223	0.203					
cg13526915	14	24,164,078	0.002581	0.223	0.156					
cg18264486	6	31,650,916	0.017892	0.223	0.275					
cg20862097	6	39,760,776	0.006302	0.223	0.277				*DAAM2*	TSS200
cg23448348	3	139,257,907	7.9E-07	0.223	0.227				*RBP1*	Body
cg01804036	12	117,483,310	1.22E-07	0.223	0.186				*TESC*	Body
cg10114725	2	225,266,733	0.010716	0.223	0.266				*FAM124B*	TSS200
cg18901140	17	8,908,225	2.55E-10	0.223	0.194					
cg07958575	7	140,206,867	6.48E-05	0.223	0.243					
cg26950867	2	216,299,856	1.89E-07	0.223	0.219				*FN1*	Body
cg26965695	X	23,926,980	7.5E-23	0.223	0.211				*CXorf58*	5'UTR
cg13764778	17	48,546,503	0.021667	0.223	0.219				*CHAD*	TSS1500
cg26074349	12	50,426,727	0.011128	0.223	0.266					
cg24953596	1	3,515,194	0.00615	0.223	0.249				*MEGF6*	Body
cg13466456	1	29,587,088	1.77E-05	0.223	0.214				*PTPRU*	Body
cg07981013	7	156,795,767	0.020602	0.223	0.184					

cg15992347	11	85,565,475	0.02058	0.223	0.149				*CCDC83*	TSS1500
cg22367872	1	4,718,573	0.003819	0.223	0.178				*AJAP1*	Body
cg15029935	15	91,126,012	9.87E-29	0.223	0.205				*CRTC3*	Body
cg14025021	15	43,809,510	2.13E-08	0.223	0.217				*MAP1A*	TSS1500
cg22236066	8	103,742,123	1.54E-10	0.223	0.233					
cg17360338	19	19,500,060	8.16E-10	0.223	0.215				*GATAD2A*	5'UTR
cg20647118	13	92,051,786	0.001135	0.223	0.233				*GPC5*	Body
cg26176204	2	236,401,677	3.77E-06	0.223	0.199				*AGAP1*	TSS1500
cg01379344	17	70,609,699	1.69E-08	0.223	0.231					
cg08380556	13	31,020,218	0.003826	0.223	0.142					
cg09533305	22	38,853,600	1.44E-09	0.223	0.215					
cg04328562	19	11,649,701	2.38E-05	0.223	0.245				*CNN1*	1stExon
cg14235846	17	800,745	1.25E-08	0.223	0.222				*NXN*	Body
cg19160624	12	68,221,623	8.47E-05	0.223	0.208					
cg03744842	7	45,067,319	1.83E-08	0.223	0.186				*CCM2*	Body
cg14368473	9	123,694,717	8.89E-12	0.223	0.204					
cg01539484	7	130,040,067	3.15E-12	0.223	0.230				*TSGA14*	Body
cg13861294	12	100,750,473	2.06E-05	0.223	0.290				*SLC17A8*	TSS1500
cg15206981	4	124,323,013	2.54E-06	0.223	0.251				*SPRY1*	Body
cg22452837	6	28,664,155	0.025131	0.223	0.270					
cg00318756	1	67,773,020	0.006806	0.223	0.169				*IL12RB2*	TSS200
cg10174333	1	116,336,029	0.003625	0.223	0.217					
cg10421029	16	30,936,028	2.17E-06	0.223	0.198				*FBXL19*	1stExon
cg18017908	17	16,394,618	0.000209	0.223	0.135	4.51E-13	0.108		*C17orf76*	Body
cg06964190	11	61,275,247	3.61E-08	0.223	0.185				*LRRC10B*	TSS1500
cg19907725	1	153,539,397	9.07E-10	0.223	0.212				*S100A2*	TSS1500
cg14507146	5	34,043,333	5.84E-14	0.223	0.198				*C1QTNF3*	TSS200
cg26323077	11	95,831,121	3.49E-16	0.223	0.236				*MAML2*	Body
cg25033990	17	32,484,014	6.88E-05	0.223	0.242				*ACCN1*	TSS200
cg26498247	16	50,003,613	4.38E-12	0.223	0.189					
cg07997434	2	238,864,969	0.008284	0.222	0.207					
cg22711111	6	30,139,979	0.006388	0.222	0.318				*TRIM15*	Body
cg22729681	1	164,717,625	2.24E-06	0.222	0.245				*PBX1*	Body
cg10503842	15	23,158,338	0.001707	0.222	0.261					

cg04905421	16	1,581,129	1.62E-08	0.222	0.230				*IFT140*	Body
cg13850401	9	133,413,080	0.000296	0.222	0.211					
cg22663372	2	45,161,107	0.001839	0.222	0.188					
cg01668281	21	37,915,281	2.88E-11	0.222	0.222				*CLDN14*	5'UTR
cg02218506	10	132,975,885	0.0005	0.222	0.251				*TCERG1L*	Body
cg05710562	3	13,323,633	1.59E-05	0.222	0.203					
cg05557233	6	27,482,950	0.036431	0.222	0.248					
cg11700800	5	92,906,633	3.93E-06	0.222	0.244				*FLJ42709*	Body
cg20296924	2	3,654,470	1.82E-16	0.222	0.220				*COLEC11*	Body
cg22459664	7	139,929,764	0.022491	0.222	0.151					
cg06275859	4	1,407,694	0.005489	0.222	0.247					
cg09106510	11	71,050,395	9.86E-10	0.222	0.201					
cg14580628	5	43,602,116	0.003975	0.222	0.111				*NNT*	TSS1500
cg03486832	12	122,019,525	4.65E-05	0.222	0.228				*KDM2B*	TSS1500
cg10632395	6	137,815,648	0.004229	0.222	0.261				*OLIG3*	TSS200
cg25007705	1	21,588,799	2.18E-10	0.222	0.212				*ECE1*	Body
cg01947130	15	60,287,196	0.021246	0.222	0.235					
cg01611777	2	102,359,027	0.019442	0.222	0.125				*MAP4K4*	Body
cg07145843	5	95,769,025	0.006342	0.222	0.184				*PCSK1*	TSS200
cg04859826	1	59,479,179	0.000677	0.222	0.173					
cg19483007	3	149,327,651	0.000175	0.222	0.210				*WWTR1*	Body
cg00053393	4	183,064,388	5.48E-06	0.222	0.249				*MGC45800*	Body
cg15339164	12	3,589,869	3.65E-06	0.222	0.242					
cg19344322	7	105,650,550	6.76E-12	0.222	0.216				*FLJ23834*	Body
cg27134827	6	32,121,566	0.001747	0.222	0.194				*PPT2*	Body
cg24135939	6	42,419,091	0.008073	0.222	0.209				*TRERF1*	5'UTR
cg06261926	2	161,264,607	0.01152	0.222	0.148				*RBMS1*	Body
cg00171092	3	124,531,371	7.2E-10	0.222	0.200			Yes	*ITGB5*	Body
cg03430846	8	31,497,082	0.038383	0.222	0.216				*NRG1*	TSS200
cg07316846	2	105,484,694	0.00844	0.222	0.272					
cg00294382	12	56,732,721	0.000128	0.222	0.210	5.13E-19	0.172		*IL23A*	5'UTR
cg01296593	16	86,599,976	0.007458	0.222	0.255				*FOXC2*	TSS1500
cg24210813	17	46,669,644	8.19E-09	0.222	0.191				*LOC404266*	TSS200
cg00824767	10	23,488,272	0.00169	0.222	0.213					

cg13793157	19	51,415,450	1.14E-11	0.222	0.223				*KLK4*	TSS1500
cg15090211	6	3,232,464	0.034381	0.222	0.097					
cg18390495	20	239,809	2.08E-06	0.222	0.194				*DEFB132*	Body
cg19909787	10	124,907,708	0.019552	0.222	0.170				*HMX2*	1stExon
cg14989164	1	180,205,157	0.007343	0.222	0.278				*LHX4*	Body
cg15668533	3	10,858,446	1.5E-06	0.222	0.194				*SLC6A11*	Body
cg00983904	12	6,665,288	0.000111	0.222	0.220				*IFFO1*	TSS200
cg00090619	6	33,160,286	0.042762	0.222	0.298				*COL11A2*	TSS200
cg12936335	6	150,286,212	0.016536	0.222	0.196				*ULBP1*	Body
cg15175129	12	65,672,052	2.28E-06	0.222	0.204				*MSRB3*	TSS1500
cg22038738	8	42,064,673	1.83E-11	0.222	0.170	2.57E-21	0.148		*PLAT*	5'UTR
cg22976533	14	105,784,090	1.65E-06	0.222	0.208				*PACS2*	Body
cg15205547	7	23,514,347	0.044608	0.222	0.219					
cg16097191	1	9,262,511	1.53E-05	0.222	0.200					
cg24378440	22	25,816,699	0.006006	0.222	0.198					
cg02590287	X	106,161,451	1.19E-08	0.222	0.218				*CLDN2*	5'UTR
cg16299747	10	118,429,952	0.003042	0.222	0.195				*C10orf82*	TSS1500
cg00575066	6	10,390,039	0.000126	0.222	0.235					
cg08959094	16	88,186,048	3.51E-32	0.222	0.221					
cg09940311	1	183,901,666	7.82E-05	0.222	0.190					
cg19092096	1	110,612,474	3.82E-07	0.222	0.211				*ALX3*	Body
cg21566081	7	127,841,626	0.010023	0.222	0.195					
cg00099602	6	87,993,447	5.28E-08	0.222	0.265				*GJB7*	3'UTR
cg15616358	11	68,610,296	1.77E-07	0.222	0.233				*CPT1A*	TSS1500
cg04214430	13	50,702,707	1.09E-11	0.222	0.201					
cg01835695	5	180,230,959	2.62E-09	0.222	0.202				*MGAT1*	5'UTR
cg07278054	3	55,523,594	2.01E-08	0.222	0.194					
cg07515989	1	43,250,763	0.000621	0.222	0.238					
cg14537332	11	70,508,113	1.28E-05	0.222	0.202				*SHANK2*	Body
cg04784839	2	114,299,047	0.006791	0.222	0.168					
cg16104076	5	40,682,069	3.19E-06	0.222	0.234				*PTGER4*	Body
cg18344930	5	87,441,081	0.017628	0.222	0.289					
cg19700872	5	145,895,766	0.040164	0.222	0.259				*GPR151*	TSS200
cg20495365	4	3,371,291	0.000113	0.222	0.201				*RGS12*	TSS1500

cg00634665	8	54,790,957	0.000483	0.222	0.243				*RGS20*	Body
cg12308407	6	32,081,491	1.51E-14	0.222	0.229					
cg02097014	1	203,156,374	0.007651	0.222	0.164				*CHI3L1*	TSS1500
cg26046136	20	45,526,613	1.24E-09	0.222	0.196				*EYA2*	5'UTR
cg18143296	3	157,812,763	0.001542	0.222	0.231					
cg06230839	21	43,189,892	2.1E-17	0.222	0.206					
cg06829686	1	47,910,843	1.4E-05	0.222	0.202					
cg11325578	X	9,733,287	1.08E-10	0.222	0.216				*GPR143*	Body
cg08235970	1	18,957,023	0.010083	0.222	0.189				*PAX7*	TSS1500
cg08783616	17	16,389,293	0.000517	0.222	0.152				*C17orf76*	Body
cg20954987	12	48,578,223	0.003569	0.222	0.257				*C12orf68*	1stExon
cg15414638	6	28,956,731	9.66E-05	0.222	0.209					
cg18425434	10	118,429,648	0.000192	0.222	0.286				*C10orf82*	TSS200
cg20248056	13	113,704,976	8.42E-08	0.222	0.197				*MCF2L*	Body
cg10282491	16	3,062,368	3.76E-06	0.222	0.230	2.24E-25	0.200		*CLDN9*	TSS200
cg16895719	8	25,899,257	2.9E-05	0.222	0.206				*EBF2*	Body
cg23015917	1	67,485,337	2.47E-09	0.222	0.217				*SLC35D1*	Body
cg04407762	4	113,442,791	0.001777	0.222	0.183					
cg05939970	3	127,795,340	3.1E-05	0.222	0.234					
cg05643964	20	62,369,504	0.00494	0.222	0.258				*LIME1*	Body
cg15633699	1	1,356,728	2.41E-12	0.222	0.198				*LOC441869*	TSS200
cg15909232	7	156,235,420	3.17E-05	0.222	0.264			Yes		
cg17445765	7	64,403,162	0.000347	0.222	0.144					
cg18569734	6	108,496,460	4.93E-05	0.222	0.218				*NR2E1*	Body
cg19759671	4	183,063,459	0.002394	0.222	0.203				*MGC45800*	Body
cg26590537	12	5,020,202	7.77E-05	0.222	0.241	4.79E-09	0.104		*KCNA1*	5'UTR
cg11840197	20	60,540,469	0.005545	0.222	0.201					
cg16639691	13	28,597,227	1.49E-05	0.222	0.235				*FLT3*	Body
cg18564686	2	10,169,675	9.13E-08	0.222	0.212					
cg20671920	8	10,586,678	1.57E-09	0.222	0.212				*SOX7*	Body
cg05779272	9	33,168,018	2.56E-13	0.222	0.205	1.59E-14	0.118		*B4GALT1*	TSS1500
cg09630706	7	229,695	2.53E-13	0.222	0.218				*FAM20C*	Body
cg11049075	17	42,199,063	5.57E-15	0.222	0.238				*HDAC5*	5'UTR
cg16592596	17	79,620,347	7.7E-09	0.222	0.209				*PDE6G*	5'UTR

cg24332783	11	31,818,791	0.00124	0.222	0.230				*PAX6*	Body
cg12406559	18	56,530,302	4.2E-07	0.222	0.138	2.78E-08	0.124		*ZNF532*	1stExon
cg17316316	5	180,525,108	0.034864	0.222	0.216					
cg00873517	8	136,511,832	3.03E-06	0.222	0.248				*KHDRBS3*	Body
cg04330449	5	134,871,166	0.039637	0.222	0.260	0.00035374	0.080		*NEUROG1*	1stExon
cg05813297	8	82,543,035	0.000329	0.222	0.210					
cg11202345	17	76,976,057	0.0015	0.222	0.180				*LGALS3BP*	1stExon
cg23528975	2	128,403,133	1.09E-26	0.222	0.212				*LIMS2*	Body
cg12047391	1	160,957,458	4.74E-07	0.222	0.240					
cg13450266	13	113,625,160	5.9E-22	0.222	0.223				*MCF2L*	Body
cg14254480	2	26,927,693	3.2E-14	0.222	0.218				*KCNK3*	Body
cg14553895	3	178,899,170	1.37E-05	0.222	0.232				*PIK3CA*	5'UTR
cg19926444	19	12,624,742	7.48E-06	0.222	0.245				*ZNF709*	TSS200
cg25316769	5	72,607,540	0.000103	0.222	0.190					
cg27315742	6	117,586,681	0.000831	0.222	0.252				*VGLL2*	TSS200
cg04039799	11	19,745,484	0.004653	0.222	0.181				*NAV2*	Body
cg11203512	11	93,064,092	0.000822	0.222	0.171				*CCDC67*	5'UTR
cg24028260	6	139,899,284	1.22E-17	0.222	0.223					
cg25836567	17	44,929,689	0.002025	0.222	0.185				*WNT9B*	Body
cg26898166	2	73,143,799	6.96E-05	0.222	0.245	2.85E-20	0.162		*EMX1*	TSS1500
cg00024443	16	21,569,473	3.38E-06	0.222	0.221					
cg02882375	11	34,517,751	7.18E-13	0.222	0.219				*ELF5*	Body
cg06834637	18	9,017,502	0.001041	0.222	0.190					
cg17421417	5	92,934,644	7.61E-07	0.222	0.208					
cg08461760	7	25,703,644	1.73E-06	0.222	0.242					
cg16968985	17	27,313,254	0.006994	0.222	0.131				*SEZ6*	Body
cg04513549	10	1,651,238	0.006531	0.222	0.218				*ADARB2*	Body
cg13262467	3	169,538,890	1.14E-12	0.222	0.243				*LRRIQ4*	TSS1500
cg13713830	9	79,631,737	0.000288	0.222	0.193					
cg17583077	7	108,097,436	0.000853	0.222	0.181				*NRCAM*	TSS1500
cg25217100	13	37,249,426	0.000606	0.222	0.249				*C13orf36*	5'UTR
cg05657792	17	6,899,758	0.000163	0.222	0.169				*ALOX12*	Body
cg07033292	15	78,287,690	9.26E-16	0.222	0.198				*TBC1D2B*	3'UTR
cg14317639	14	92,721,505	0.000386	0.222	0.182					

cg07722722	1	45,187,551	2.7E-09	0.222	0.210				*C1orf228*	Body
cg10892950	12	45,626,938	0.001132	0.222	0.191				*ANO6*	Body
cg14829606	6	166,579,974	0.00036	0.222	0.240				*T*	Body
cg05512561	13	33,925,656	6.83E-05	0.222	0.197					
cg16142218	8	23,101,221	2.55E-09	0.222	0.196				*CHMP7*	1stExon
cg26315862	13	21,520,519	7.71E-06	0.222	0.208					
cg07711397	10	82,297,615	8.57E-17	0.222	0.214				*SH2D4B*	TSS200
cg09035882	20	50,720,331	2.24E-06	0.222	0.241				*ZFP64*	Body
cg02103924	1	110,672,857	8.78E-05	0.222	0.182					
cg10158997	6	31,734,106	1.9E-14	0.222	0.209				*C6orf27*	Body
cg22749589	20	21,501,710	0.013072	0.222	0.218					
cg06875227	5	50,683,378	0.020304	0.222	0.277				*ISL1*	Body
cg02056653	5	140,888,468	1.05E-05	0.222	0.208				*PCDHGA4*	Body
cg06857304	10	94,826,314	1.4E-11	0.222	0.195				*CYP26C1*	Body
cg10160276	16	52,583,062	5.43E-05	0.222	0.160				*TOX3*	TSS1500
cg19719391	4	26,789,915	7.5E-08	0.222	0.216					
cg20722210	5	134,827,136	7.96E-09	0.222	0.195					
cg27044570	X	111,325,176	0.000002	0.222	0.207				*TRPC5*	5'UTR
cg21331088	18	908,290	0.025012	0.222	0.219				*ADCYAP1*	Body
cg00328729	11	45,231,175	1.57E-20	0.222	0.214				*PRDM11*	Body
cg03866607	17	59,532,826	1.33E-07	0.222	0.190				*TBX4*	TSS1500
cg26517714	5	170,736,245	0.002205	0.222	0.246				*TLX3*	TSS200
cg26585701	6	30,418,565	1.11E-05	0.222	0.197					
cg04410715	19	39,283,334	4.57E-06	0.222	0.213					
cg09408098	1	170,631,331	0.000788	0.222	0.196					
cg16659284	16	12,189,952	1.03E-12	0.222	0.223				*SNX29*	Body
cg03978242	17	40,822,497	3.98E-09	0.222	0.192				*PLEKHH3*	Body
cg05234035	5	172,673,203	1.91E-10	0.222	0.216					
cg26312951	21	42,797,847	1.07E-10	0.222	0.194				*MX1*	TSS200
cg05238917	6	28,504,946	1.79E-05	0.222	0.208					
cg07809831	16	48,428,016	1.6E-11	0.222	0.210					
cg09246479	22	24,891,129	0.04913	0.222	0.242				*C22orf45*	TSS1500
cg20803910	17	6,659,375	0.009508	0.222	0.192				*XAF1*	5'UTR
cg07723251	17	50,237,742	0.007794	0.222	0.243				*CA10*	TSS1500

cg17786206	1	29,142,254	2.55E-09	0.222	0.244				*OPRD1*	Body
cg01169778	9	136,038,690	1.22E-16	0.222	0.229	2.87E-22	0.173		*GBGT1*	5'UTR
cg04317047	5	148,808,388	0.000195	0.222	0.269				*MIR143*	TSS200
cg10987840	2	9,319,526	0.029174	0.222	0.156					
cg14355482	21	22,373,039	9.45E-05	0.222	0.146				*NCAM2*	Body
cg17826594	1	55,506,014	0.004303	0.222	0.288				*PCSK9*	Body
cg27365701	12	89,744,150	1.44E-08	0.222	0.228				*DUSP6*	Body
cg22325764	8	1,959,442	0.002175	0.222	0.100					
cg15801007	6	131,383,376	8.75E-05	0.222	0.225				*EPB41L2*	5'UTR
cg04130163	4	42,401,214	0.000636	0.222	0.222				*SHISA3*	Body
cg04352305	17	80,654,922	6.97E-05	0.222	0.156				*RAB40B*	Body
cg17828255	X	40,435,744	2.4E-09	0.222	0.211					
cg11613003	2	202,901,288	5.41E-05	0.222	0.225				*FZD7*	1stExon
cg17547578	X	102,983,589	1.11E-05	0.222	0.234				*GLRA4*	TSS200
cg27478485	12	6,898,682	7.85E-07	0.222	0.206				*CD4*	5'UTR
cg00988346	1	27,729,801	6.33E-06	0.222	0.262					
cg09983301	2	69,404,831	0.000324	0.222	0.216				*ANTXR1*	Body
cg02909136	20	44,941,542	0.001881	0.222	0.148					
cg04964944	21	27,012,878	0.031718	0.222	0.227				*JAM2*	Body
cg17496661	5	3,326,343	2.7E-15	0.222	0.176					
cg20654074	15	41,223,179	1.1E-05	0.222	0.195				*DLL4*	Body
cg20858454	11	4,010,737	2.06E-11	0.222	0.221				*STIM1*	Body
cg24553673	1	200,005,453	0.005121	0.222	0.242				*NR5A2*	Body
cg08062338	3	181,428,669	2.47E-06	0.222	0.203				*SOX2OT*	Body
cg14355911	17	59,473,456	1.63E-07	0.222	0.215					
cg25401612	12	20,009,446	4.19E-07	0.222	0.233					
cg09619598	9	115,512,530	4.51E-06	0.221	0.206				*SNX30*	TSS1500
cg22675791	11	46,370,729	9.5E-09	0.221	0.197				*DGKZ*	Body
cg03853809	1	220,863,382	0.010651	0.221	0.107				*C1orf115*	TSS1500
cg24351901	6	29,692,092	1.24E-05	0.221	0.216				*HLA-F*	Body
cg25841265	4	6,660,482	2.14E-16	0.221	0.227					
cg02689072	4	802,844	5.34E-07	0.221	0.216				*CPLX1*	Body
cg03339668	12	120,241,957	5.28E-15	0.221	0.228				*CIT*	Body
cg04462652	2	133,429,088	1.51E-06	0.221	0.235				*LYPD1*	TSS1500

cg14327394	9	116,337,285	4.85E-15	0.221	0.197				*RGS3*	Body
cg15388804	14	36,993,079	5.16E-07	0.221	0.234					
cg18040878	12	54,409,229	0.016286	0.221	0.233				*HOXC4*	TSS1500
cg07337290	16	28,505,320	7.61E-08	0.221	0.217				*APOB48R*	TSS1500
cg21255939	X	9,858,802	2.09E-21	0.221	0.205				*SHROOM2*	Body
cg04678916	2	54,900,910	0.000157	0.221	0.210					
cg09410389	8	41,168,205	7.44E-11	0.221	0.193				*SFRP1*	TSS1500
cg13710662	7	150,896,489	9.77E-09	0.221	0.212					
cg14870271	17	76,976,010	0.00096	0.221	0.182	6.35E-23	0.162	Yes	*LGALS3BP*	1stExon
cg04415672	12	58,237,706	6.19E-13	0.221	0.222				*CTDSP2*	Body
cg14726488	6	137,815,572	0.016227	0.221	0.255				*OLIG3*	TSS200
cg17627654	11	70,508,410	1.93E-10	0.221	0.215				*SHANK2*	Body
cg22933847	11	68,780,136	2.54E-08	0.221	0.197	0.00257397	0.036		*MRGPRF*	5'UTR
cg24042578	1	2,066,981	7.02E-07	0.221	0.182				*PRKCZ*	5'UTR
cg02079034	6	150,589,148	0.009759	0.221	0.166					
cg07464206	11	15,960,015	4.46E-13	0.221	0.187					
cg11562411	6	35,206,618	6.84E-07	0.221	0.228				*SCUBE3*	Body
cg03558222	X	47,483,257	0.033021	0.221	0.067					
cg01550055	1	119,543,216	3.23E-05	0.221	0.228					
cg22185065	6	6,318,785	5.97E-05	0.221	0.227				*F13A1*	Body
cg16331358	10	1,147,675	0.00833	0.221	0.212				*WDR37*	Body
cg24076474	19	11,531,344	0.012838	0.221	0.288				*RGL3*	TSS1500
cg10172432	13	95,357,042	2.84E-06	0.221	0.204					
cg00093080	12	117,421,224	3.97E-12	0.221	0.224				*FBXW8*	Body
cg25138553	1	22,223,841	3.46E-09	0.221	0.199				*HSPG2*	Body
cg00644823	1	91,301,461	0.002037	0.221	0.233					
cg07628705	14	51,339,074	2.55E-05	0.221	0.207				*ABHD12B*	5'UTR
cg17476701	6	30,651,563	1.09E-05	0.221	0.217				*KIAA1949*	Body
cg05097643	16	3,018,470	0.020156	0.221	0.123				*PAQR4*	TSS1500
cg16866941	22	38,601,325	1.38E-07	0.221	0.251				*MAFF*	5'UTR
cg20713092	7	100,946,111	0.026448	0.221	0.228					
cg15720089	10	18,550,223	7.85E-07	0.221	0.222				*CACNB2*	Body
cg12806763	17	46,655,836	0.001107	0.221	0.262				*HOXB4*	TSS200
cg23477348	21	45,564,615	6.96E-06	0.221	0.233				*C21orf33*	Body

cg01446515	13	20,805,443	3.79E-06	0.221	0.203				*GJB6*	5'UTR
cg16120415	1	156,399,787	0.000614	0.221	0.304				*C1orf61*	TSS1500
cg00381755	16	30,572,815	0.003901	0.221	0.207					
cg09515921	1	221,057,558	1.54E-22	0.221	0.228				*HLX*	Body
cg02586730	11	65,639,869	1.98E-13	0.221	0.223	1.25E-19	0.169		*EFEMP2*	5'UTR
cg04253876	2	30,453,758	1.29E-05	0.221	0.206				*LBH*	TSS1500
cg20305024	15	40,575,730	1.34E-06	0.221	0.216					
cg06653004	6	50,785,325	0.002495	0.221	0.290				*TFAP2B*	TSS1500
cg25980637	1	16,163,967	2.72E-06	0.221	0.205				*FLJ37453*	Body
cg27419860	15	81,426,477	0.017744	0.221	0.132				*C15orf26*	TSS200
cg27589809	3	50,650,410	9.58E-12	0.221	0.208				*CISH*	TSS1500
cg08272286	15	33,486,937	0.028764	0.221	0.207					
cg00192220	16	48,570,215	2.82E-13	0.221	0.200					
cg00332937	5	72,740,460	0.000616	0.221	0.166					
cg15160686	1	24,562,825	3.42E-09	0.221	0.217					
cg04316624	2	177,036,809	5.28E-05	0.221	0.203				*HOXD3*	Body
cg11430259	10	123,355,748	2.05E-05	0.221	0.191				*FGFR2*	5'UTR
cg13444518	9	135,466,773	3.6E-06	0.221	0.221					
cg15397593	12	2,273,119	7.75E-09	0.221	0.222				*CACNA1C*	Body
cg20559634	3	45,609,400	4.07E-06	0.221	0.213					
cg24334438	X	13,395,432	0.000118	0.221	0.252					
cg04492228	10	8,101,513	0.001544	0.221	0.223				*GATA3*	Body
cg19750282	6	43,670,519	2.52E-09	0.221	0.212					
cg24251218	11	10,347,429	2.44E-06	0.221	0.222					
cg03936495	17	59,539,251	5.15E-05	0.221	0.199				*TBX4*	Body
cg06252276	11	34,852,392	2.05E-26	0.221	0.221					
cg07276534	3	193,534,580	0.001092	0.221	0.208					
cg07892413	2	129,076,032	1.44E-06	0.221	0.180				*HS6ST1*	1stExon
cg11713658	4	148,652,434	4.43E-05	0.221	0.222				*ARHGAP10*	TSS1500
cg14007090	20	60,915,009	2.89E-12	0.221	0.209				*LAMA5*	Body
cg04945350	1	228,605,010	0.000621	0.221	0.246				*TRIM17*	TSS1500
cg10914899	13	25,290,781	3.37E-06	0.221	0.225					
cg17666539	19	7,927,207	1.45E-09	0.221	0.214				*EVI5L*	Body
cg23655970	14	37,126,582	0.000167	0.221	0.232				*PAX9*	TSS200

cg26316946	6	101,846,967	1.29E-06	0.221	0.198	0.00581339	0.047		*GRIK2*	1stExon
cg27196745	12	15,475,319	0.000739	0.221	0.177	2.22E-26	0.189		*PTPRO*	TSS200
cg09940675	1	54,742,754	3.46E-19	0.221	0.198				*SSBP3*	Body
cg23017915	3	149,374,535	0.007485	0.221	0.149				*WWTR1*	Body
cg07699845	8	38,068,205	1.35E-08	0.221	0.224				*BAG4*	3'UTR
cg11251135	17	1,863,911	5.72E-12	0.221	0.211				*RTN4RL1*	Body
cg27620317	7	25,900,768	0.001607	0.221	0.174					
cg04231050	3	133,191,297	2.47E-06	0.221	0.177				*BFSP2*	Body
cg26845297	4	85,422,409	0.004786	0.221	0.204					
cg14149988	9	139,048,519	0.003347	0.221	0.204					
cg15203566	5	115,697,446	1.94E-10	0.221	0.227					
cg18886245	6	50,792,868	0.014001	0.221	0.224				*TFAP2B*	Body
cg18958126	13	100,547,367	0.033455	0.221	0.139				*CLYBL*	3'UTR
cg27416261	9	35,406,590	6.83E-05	0.221	0.153				*LOC158381*	TSS200
cg03204678	6	26,614,535	0.047017	0.221	0.184					
cg03475821	14	105,288,120	4.83E-08	0.221	0.195				*MGC23270*	Body
cg11512289	4	165,898,912	1.51E-05	0.221	0.199				*TRIM61*	TSS200
cg05036173	2	108,994,528	2.4E-05	0.221	0.180				*SULT1C4*	5'UTR
cg17409313	20	42,876,939	0.000822	0.221	0.172				*GDAP1L1*	Body
cg01142526	5	177,541,754	0.004891	0.221	0.240				*N4BP3*	5'UTR
cg14517390	15	78,463,330	2.48E-10	0.221	0.218				*ACSBG1*	3'UTR
cg18604823	19	56,687,364	0.002475	0.221	0.286				*GALP*	TSS200
cg21766667	17	48,287,888	8.16E-05	0.221	0.189					
cg02212009	11	12,820,860	0.004231	0.221	0.138				*TEAD1*	Body
cg08203127	16	331,727	0.009983	0.221	0.265				*ARHGDIG*	Body
cg12098750	12	99,548,757	2.19E-10	0.221	0.196				*ANKS1B*	TSS1500
cg13633270	18	55,095,555	0.000758	0.221	0.216					
cg23034757	5	140,787,864	0.001657	0.221	0.238				*PCDHGA4*	Body
cg06708634	6	100,906,895	0.000814	0.221	0.159				*SIM1*	Body
cg18586823	5	114,525,702	0.00012	0.221	0.204					
cg13655803	17	48,912,764	5.05E-08	0.221	0.207				*WFIKKN2*	1stExon
cg18454299	15	44,959,064	1.21E-06	0.221	0.230				*PATL2*	Body
cg21285198	1	1,360,970	2.99E-12	0.221	0.228				*TMEM88B*	TSS1500
cg02116942	2	69,519,143	4.39E-12	0.221	0.221					

cg05113410	2	102,327,511	2.19E-08	0.221	0.213				*MAP4K4*	Body
cg09074113	2	20,870,087	1.66E-17	0.221	0.214				*GDF7*	Body
cg15528028	1	33,828,944	4.37E-05	0.221	0.217				*PHC2*	Body
cg17119907	12	117,797,116	4.94E-07	0.221	0.210				*NOS1*	5'UTR
cg18707489	14	104,690,151	1.2E-06	0.221	0.212					
cg25260137	8	65,282,185	0.014756	0.221	0.270					
cg27102297	16	88,966,903	5.87E-24	0.221	0.211				*CBFA2T3*	Body
cg05325193	20	22,558,233	0.035544	0.221	0.175				*C20orf56*	Body
cg10785340	5	134,715,577	8.55E-11	0.221	0.213				*H2AFY*	Body
cg19522511	3	105,073,077	2.67E-07	0.221	0.228					
cg27349333	10	5,406,971	0.015502	0.221	0.184				*UCN3*	TSS200
cg02916272	11	1,769,443	0.003483	0.221	0.193				*HCCA2*	Body
cg16328007	1	41,119,988	0.007485	0.221	0.266				*RIMS3*	5'UTR
cg02023167	11	19,264,191	5.09E-07	0.221	0.214					
cg04259326	1	167,599,124	0.00046	0.221	0.177				*RCSD1*	TSS1500
cg05651511	17	78,754,090	1.94E-10	0.221	0.216				*RPTOR*	Body
cg11977605	4	1,076,247	4.27E-05	0.221	0.283				*RNF212*	Body
cg00544337	10	82,116,203	0.000162	0.221	0.166				*DYDC2*	TSS1500
cg00793935	2	234,847,769	0.026163	0.221	0.227				*TRPM8*	Body
cg14215711	10	22,894,054	1.33E-09	0.221	0.217				*PIP4K2A*	Body
cg18751141	7	27,138,173	0.018074	0.221	0.251					
cg14206056	9	110,797,033	1.42E-11	0.221	0.204					
cg16604801	17	2,718,310	1.69E-05	0.221	0.174				*RAP1GAP2*	Body
cg25296938	7	98,976,900	8.05E-06	0.221	0.221				*ARPC1B*	5'UTR
cg04299135	11	129,244,441	0.001247	0.221	0.239				*BARX2*	TSS1500
cg00054210	8	65,711,644	0.000798	0.221	0.107				*CYP7B1*	TSS1500
cg05441133	10	48,416,891	1.18E-05	0.221	0.226	2.25E-07	0.070		*GDF2*	TSS200
cg16026922	4	41,259,044	0.000104	0.221	0.212				*UCHL1*	Body
cg00175709	1	207,670,014	0.001077	0.221	0.258				*CR1*	Body
cg03444722	2	112,375,854	2.63E-11	0.221	0.227					
cg08198193	5	81,146,787	1.56E-05	0.221	0.226					
cg18222759	14	22,989,081	2.97E-12	0.221	0.227					
cg24127874	2	239,149,859	0.019499	0.221	0.259	1.16E-07	0.136		*HES6*	TSS1500
cg24421906	18	11,947,936	0.004665	0.221	0.245					

cg24602417	8	102,236,283	1.64E-14	0.221	0.215					
cg09112081	16	29,796,620	3.19E-06	0.221	0.229					
cg10508760	1	166,853,789	0.021322	0.221	0.182					
cg15583029	10	134,446,066	6.25E-12	0.221	0.216				*INPP5A*	Body
cg25228169	15	72,489,416	5.41E-15	0.221	0.199				*GRAMD2*	Body
cg25962699	3	170,332,444	4.6E-05	0.221	0.191					
cg02574464	16	57,940,653	1.75E-07	0.221	0.257				*CNGB1*	Body
cg03310937	3	8,648,591	1.93E-06	0.221	0.181					
cg24173049	17	6,939,795	0.012292	0.221	0.107	8.75E-21	0.161		*SLC16A13*	1stExon
cg26534508	1	236,306,311	0.033604	0.221	0.084	2.26E-16	0.170		*GPR137B*	1stExon
cg11964338	19	52,847,185	5.16E-20	0.221	0.220				*ZNF610*	5'UTR
cg12610312	12	109,162,409	1.16E-05	0.221	0.187					
cg23856413	6	106,429,659	3.19E-06	0.221	0.205					
cg00059737	1	12,400,434	8.93E-13	0.221	0.222				*VPS13D*	Body
cg00597801	4	109,092,918	0.003496	0.221	0.234				*LOC641518*	TSS1500
cg15296664	12	89,748,773	4.5E-12	0.221	0.215					
cg12659606	6	33,215,807	0.002147	0.221	0.219					
cg27177841	1	213,125,950	4.66E-09	0.221	0.242				*VASH2*	Body
cg01416891	10	94,820,892	3.22E-06	0.221	0.194				*CYP26C1*	TSS200
cg21093033	8	141,468,743	0.035457	0.221	0.170				*TRAPPC9*	TSS1500
cg09803764	7	107,572,753	7.61E-12	0.221	0.231				*LAMB1*	Body
cg03011535	20	42,544,794	7.17E-06	0.221	0.235				*TOX2*	5'UTR
cg01750507	18	158,229	0.013761	0.221	0.178				*USP14*	TSS1500
cg08110272	12	108,733,321	0.002815	0.221	0.230				*CMKLR1*	TSS1500
cg09887323	1	6,348,460	1.13E-06	0.221	0.201				*ACOT7*	Body
cg13042487	14	104,181,393	0.008552	0.221	0.120	1.21E-15	0.119		*ZFYVE21*	TSS1500
cg14114377	19	45,756,845	1.07E-19	0.221	0.220				*MARK4*	Body
cg14322298	6	10,585,683	1.79E-07	0.221	0.228				*GCNT2*	Body
cg05588228	5	145,132,641	7.71E-05	0.221	0.115					
cg10147974	12	1,759,064	7.73E-07	0.221	0.221					
cg15335334	17	73,642,547	0.009005	0.221	0.161				*RECQL5*	Body
cg22126440	7	120,628,658	0.000271	0.221	0.162				*C7orf58*	TSS1500
cg08033998	14	69,714,389	7.23E-13	0.221	0.227			Yes		
cg00469602	2	172,955,930	0.001492	0.221	0.229					

cg07671949	6	152,128,338	0.009134	0.221	0.202	0.00567154	0.060			*ESR1*	TSS1500
cg01040654	5	172,663,219	1.1E-07	0.221	0.201					*NKX2-5*	TSS1500
cg21874193	12	11,174,898	8.74E-11	0.221	0.250	5.06E-14	0.227			*TAS2R19*	1stExon
cg02737384	10	45,470,199	0.04895	0.221	0.085					*RASSF4*	Body
cg07106394	14	104,372,286	5.12E-09	0.221	0.216						
cg03059420	6	30,923,241	0.007661	0.221	0.253						
cg19376973	2	42,229,025	1.03E-09	0.221	0.197						
cg27390220	3	45,268,523	1.3E-07	0.221	0.224	1.38E-12	0.140			*TMEM158*	TSS1500
cg02239862	3	33,260,743	0.013595	0.221	0.155					*SUSD5*	TSS200
cg06117072	6	50,791,385	7.26E-05	0.221	0.257					*TFAP2B*	Body
cg10385101	7	707,728	0.030123	0.221	0.181					*PRKAR1B*	Body
cg26144567	1	29,446,085	4.05E-19	0.221	0.213					*EPB41*	3'UTR
cg26514099	6	33,397,710	0.00026	0.221	0.256					*SYNGAP1*	Body
cg03483626	1	111,218,276	3.51E-08	0.221	0.215	8.13E-11	0.174			*KCNA3*	TSS1500
cg08748170	5	54,518,667	0.000144	0.221	0.207						
cg13283635	20	61,450,653	8.64E-11	0.221	0.226					*COL9A3*	Body
cg13555278	1	26,348,484	1.07E-11	0.221	0.216					*EXTL1*	1stExon
cg03655371	5	134,827,355	0.003452	0.221	0.157						
cg04702940	11	66,084,599	0.007127	0.221	0.265					*CD248*	TSS200
cg09760908	2	45,028,712	0.02179	0.221	0.237						
cg13636189	9	102,587,074	0.000713	0.221	0.159					*NR4A3*	5'UTR
cg14199723	8	145,051,795	7.43E-08	0.221	0.209					*PLEC1*	TSS1500
cg18416881	7	134,143,823	0.020267	0.221	0.175	8.32E-15	0.197			*AKR1B1*	5'UTR
cg25957451	2	73,512,148	6.55E-06	0.221	0.215						
cg02281167	6	30,140,113	1.52E-06	0.221	0.202					*TRIM15*	Body
cg18780769	3	194,118,985	0.000124	0.221	0.170					*GP5*	Body
cg19334350	7	157,406,737	9.56E-06	0.221	0.224					*PTPRN2*	Body
cg08847856	2	106,184,698	2.48E-10	0.221	0.226						
cg14568830	10	23,463,243	0.004649	0.221	0.152						
cg00259249	1	35,246,726	3.31E-05	0.221	0.178					*GJB3*	TSS200
cg15677384	7	96,648,043	0.01142	0.221	0.279						
cg20660171	2	43,455,046	0.000728	0.221	0.135					*ZFP36L2*	TSS1500
cg23547017	2	177,030,171	3.18E-06	0.221	0.166					*HOXD3*	5'UTR
cg25479916	6	27,841,392	0.047067	0.221	0.276					*HIST1H4L*	TSS200

cg08058894	20	42,544,178	0.049591	0.220	0.191				*TOX2*	TSS1500
cg19239040	7	35,298,664	2.3E-05	0.220	0.224					
cg00727590	22	31,536,549	2.02E-05	0.220	0.242				*PLA2G3*	TSS200
cg15345741	14	103,674,333	5.85E-06	0.220	0.182					
cg00498289	2	27,719,787	1.69E-14	0.220	0.221				*GCKR*	1stExon
cg12581592	11	13,984,043	0.001525	0.220	0.191				*SPON1*	1stExon
cg17578322	3	55,546,118	2.74E-05	0.220	0.161				*ERC2*	3'UTR
cg20454400	17	46,720,386	1.17E-06	0.220	0.234					
cg11414821	6	30,652,771	4.04E-06	0.220	0.216				*KIAA1949*	Body
cg13302994	2	210,714,233	0.002145	0.220	0.235				*UNC80*	Body
cg24871887	9	98,111,365	2.07E-06	0.220	0.191					
cg27499820	13	21,296,301	0.023751	0.220	0.087				*IL17D*	3'UTR
cg04623265	1	224,363,494	9.38E-17	0.220	0.216					
cg05929675	7	28,452,098	0.00066	0.220	0.146				*CREB5*	5'UTR
cg15765502	7	20,816,521	7.18E-06	0.220	0.206					
cg20003368	6	44,026,475	0.021508	0.220	0.303					
cg26029221	1	12,653,721	2.06E-13	0.220	0.201				*DHRS3*	Body
cg01952953	12	108,168,562	0.000348	0.220	0.212				*ASCL4*	1stExon
cg07769790	15	83,317,281	3.11E-05	0.220	0.213				*CPEB1*	TSS1500
cg13407335	17	7,832,852	3.8E-12	0.220	0.209				*KCNAB3*	TSS200
cg01703329	17	3,859,977	5.92E-10	0.220	0.232				*ATP2A3*	Body
cg21145938	8	38,795,218	0.00075	0.220	0.210				*PLEKHA2*	Body
cg26830291	10	88,392,088	0.012467	0.220	0.212					
cg08461339	2	241,459,227	3.97E-08	0.220	0.199				*ANKMY1*	Body
cg00737840	11	125,036,420	0.011005	0.220	0.124				*PKNOX2*	5'UTR
cg07320017	X	138,774,347	0.023887	0.220	0.235				*MCF2*	5'UTR
cg02624298	8	144,108,018	2.2E-06	0.220	0.202					
cg04198091	5	139,283,351	0.001019	0.220	0.257				*NRG2*	Body
cg04582294	10	88,022,744	0.003527	0.220	0.140				*GRID1*	Body
cg04803994	11	1,592,587	0.00791	0.220	0.249				*HCCA2*	Body
cg15336196	18	42,261,241	0.026566	0.220	0.137				*SETBP1*	5'UTR
cg05237470	X	31,526,531	0.008816	0.220	0.224				*DMD*	Body
cg18692678	12	114,838,772	0.008436	0.220	0.187				*TBX5*	Body
cg00296038	3	11,624,533	6.41E-23	0.220	0.205				*VGLL4*	Body

cg06822690	1	82,040,009	1.73E-10	0.220	0.200					
cg16198692	5	115,152,835	0.044769	0.220	0.027				*CDO1*	TSS1500
cg27136740	2	172,965,837	3.84E-06	0.220	0.211				*DLX2*	Body
cg27614534	18	56,296,496	4.25E-17	0.220	0.203				*ALPK2*	TSS1500
cg27634724	20	43,729,861	5.23E-06	0.220	0.211				*KCNS1*	TSS200
cg18071865	7	55,224,895	9.49E-09	0.220	0.203				*EGFR*	Body
cg02421172	7	1,938,701	1.75E-09	0.220	0.222				*MAD1L1*	Body
cg07920930	7	158,381,884	7.32E-11	0.220	0.193				*PTPRN2*	TSS1500
cg20590797	17	48,253,122	8.28E-07	0.220	0.263				*SGCA*	3'UTR
cg22185100	20	20,344,529	0.006461	0.220	0.162					
cg04829830	14	105,339,549	2.61E-14	0.220	0.204				*KIAA0284*	5'UTR
cg05118960	3	50,359,978	2.54E-23	0.220	0.223				*HYAL2*	5'UTR
cg16824301	1	182,573,155	0.001484	0.220	0.249				*RGS16*	Body
cg17158913	1	10,764,886	0.000758	0.220	0.118				*CASZ1*	5'UTR
cg06546202	8	143,387,265	7.46E-12	0.220	0.204				*TSNARE1*	Body
cg12960126	4	187,686,256	5.93E-15	0.220	0.198					
cg21550372	14	100,908,908	3.07E-05	0.220	0.222				*WDR25*	Body
cg23118721	8	94,133,100	5.49E-08	0.220	0.215					
cg16662451	17	18,647,507	5.54E-10	0.220	0.223				*FBXW10*	5'UTR
cg23340017	10	102,895,133	9.21E-07	0.220	0.204				*TLX1*	Body
cg07493197	17	53,573,969	1.75E-07	0.220	0.237					
cg25391117	5	34,043,284	4.95E-12	0.220	0.197				*C1QTNF3*	1stExon
cg05597836	7	96,655,566	2.72E-08	0.220	0.197				*DLX5*	TSS1500
cg23526298	12	5,620,971	0.017838	0.220	0.218					
cg27422759	11	69,970,464	1.87E-07	0.220	0.209				*ANO1*	Body
cg05085500	6	19,692,172	0.023177	0.220	0.219					
cg14648774	20	61,900,546	7.32E-05	0.220	0.185					
cg03454225	6	168,225,472	2.93E-12	0.220	0.202				*C6orf124*	Body
cg09088576	1	36,947,785	9.82E-10	0.220	0.212				*CSF3R*	5'UTR
cg16387532	5	124,747,822	0.046499	0.220	0.096					
cg12571423	13	37,006,748	0.000623	0.220	0.200	9.15E-28	0.170		*CCNA1*	5'UTR
cg12755421	11	6,342,214	3.18E-05	0.220	0.202				*PRKCDBP*	TSS1500
cg17548735	6	41,020,969	1.79E-10	0.220	0.201				*APOBEC2*	5'UTR
cg22389949	6	152,003,038	1.07E-08	0.220	0.218					

cg10608948	17	6,478,486	6.64E-08	0.220	0.202					
cg03193847	4	169,637,496	2.93E-09	0.220	0.225				*PALLD*	Body
cg21985690	5	79,330,669	1.24E-06	0.220	0.224				*THBS4*	TSS1500
cg02356993	15	27,111,476	4.41E-08	0.220	0.199				*GABRA5*	TSS1500
cg19389293	18	13,641,872	6.33E-05	0.220	0.187				*C18orf1*	Body
cg26970042	11	111,815,419	1.03E-11	0.220	0.218				*DIXDC1*	Body
cg04227238	6	29,894,005	5.41E-18	0.220	0.191				*HCG4P6*	TSS1500
cg15204594	19	16,531,632	2.91E-11	0.220	0.223				*EPS15L1*	Body
cg06997226	13	111,452,040	1.63E-06	0.220	0.207					
cg14227460	19	4,518,743	0.037056	0.220	0.236			Yes	*PLIN4*	TSS1500
cg06882591	19	34,397,628	5E-07	0.220	0.198					
cg07556059	1	29,449,513	2.33E-08	0.220	0.226				*TMEM200B*	5'UTR
cg11375458	2	177,003,996	1.38E-07	0.220	0.185					
cg20986832	1	226,150,312	0.000812	0.220	0.242					
cg25300694	2	151,184,358	0.018152	0.220	0.152					
cg03856949	12	51,318,042	0.002141	0.220	0.184				*METTL7A*	TSS1500
cg05110828	3	61,570,099	8.73E-20	0.220	0.214				*PTPRG*	Body
cg09371281	9	8,858,574	0.017693	0.220	0.164				*PTPRD*	5'UTR
cg14385362	9	127,054,095	4.63E-07	0.220	0.203				*NEK6*	TSS1500
cg14414903	2	240,171,712	6.59E-08	0.220	0.219				*HDAC4*	Body
cg20618826	10	81,148,896	1.56E-14	0.220	0.192				*ZCCHC24*	Body
cg15775138	7	127,744,389	0.049122	0.220	0.199					
cg16138458	6	27,841,120	0.011531	0.220	0.228				*HIST1H4L*	1stExon
cg19693158	19	56,596,642	2.71E-07	0.220	0.196					
cg03819089	2	66,298,217	6.74E-10	0.220	0.223					
cg04144533	1	206,681,983	1.05E-08	0.220	0.212				*RASSF5*	Body
cg06060522	20	44,782,039	0.007854	0.220	0.251					
cg13075279	12	125,333,267	7.2E-17	0.220	0.194				*SCARB1*	Body
cg03455107	17	54,509,207	1.01E-06	0.220	0.197				*ANKFN1*	Body
cg21459340	20	2,781,324	0.047409	0.220	0.138				*CPXM1*	TSS200
cg23497383	2	239,140,182	0.000432	0.220	0.209				*LOC151174*	Body
cg13971030	11	35,366,721	2.64E-13	0.220	0.223				*SLC1A2*	Body
cg18254356	5	43,044,573	0.001005	0.220	0.245				*LOC153684*	Body
cg26288502	8	10,421,582	3.07E-07	0.220	0.255					

cg07095637	7	2,079,008	1.57E-19	0.220	0.180				*MAD1L1*	Body
cg13107768	1	119,542,229	0.012645	0.220	0.197					
cg25722029	19	13,907,696	1.95E-08	0.220	0.168				*ZSWIM4*	Body
cg03252313	7	148,117,075	8.7E-13	0.220	0.207				*CNTNAP2*	3'UTR
cg12636499	10	105,408,599	1.11E-08	0.220	0.202				*SH3PXD2A*	Body
cg13957043	4	100,174,038	4.36E-10	0.220	0.225					
cg11817961	1	39,025,469	0.023314	0.220	0.162					
cg07733851	5	142,781,498	0.004536	0.220	0.131				*NR3C1*	5'UTR
cg07812715	12	117,483,086	4.15E-10	0.220	0.168				*TESC*	Body
cg07281318	7	6,575,968	0.003885	0.220	0.255				*GRID2IP*	Body
cg10634136	2	202,901,470	7.7E-06	0.220	0.218				*FZD7*	1stExon
cg26127479	6	39,070,179	3.75E-08	0.220	0.213					
cg26978698	4	145,567,592	0.003059	0.220	0.121				*HHIP*	1stExon
cg05779675	16	50,637,092	5.55E-13	0.220	0.209				*NKD1*	Body
cg12024292	9	120,177,979	0.000177	0.220	0.187	2.07E-20	0.165		*ASTN2*	TSS1500
cg19611013	22	50,915,586	1.09E-10	0.220	0.208					
cg20669292	17	40,823,420	7.75E-06	0.220	0.192				*PLEKHH3*	Body
cg24392955	12	54,454,325	2.73E-12	0.220	0.192				*FLJ12825*	Body
cg02961101	16	48,533,155	1.7E-05	0.220	0.196					
cg17017317	1	4,645,438	0.021779	0.220	0.292					
cg10553204	2	20,871,195	1.61E-16	0.220	0.198				*GDF7*	3'UTR
cg20791593	2	242,751,796	1.73E-07	0.220	0.214	2.86E-11	0.090		*NEU4*	TSS1500
cg11518509	1	114,525,428	1.7E-33	0.220	0.210					
cg04370247	10	126,308,552	1.16E-10	0.220	0.197				*FAM53B*	3'UTR
cg08986840	8	38,409,865	2.32E-08	0.220	0.208					
cg21225548	9	100,864,335	0.000735	0.220	0.150				*TRIM14*	Body
cg15890075	17	18,011,923	3E-17	0.220	0.198				*MYO15A*	TSS200
cg18208602	18	10,488,229	0.002373	0.220	0.213				*APCDD1*	3'UTR
cg17438155	7	73,038,045	0.004171	0.220	0.227				*MLXIPL*	Body
cg10552446	2	66,809,923	0.000929	0.220	0.228					
cg15673955	2	173,787,028	2.45E-06	0.220	0.195				*RAPGEF4*	Body
cg24112713	14	21,494,093	2.67E-05	0.220	0.212				*NDRG2*	TSS1500
cg06132502	15	71,145,127	1.88E-09	0.220	0.200				*LARP6*	Body
cg12754421	11	105,480,790	0.003594	0.220	0.227				*GRIA4*	TSS200

cg00301795	1	200,195,818	0.020834	0.220	0.266					
cg10695490	19	49,659,299	8.26E-13	0.220	0.202				*HRC*	TSS1500
cg12165215	17	35,277,398	0.001249	0.220	0.164					
cg23559689	11	105,481,292	0.02223	0.220	0.258				*GRIA4*	5'UTR
cg05774978	1	146,649,842	0.010506	0.220	0.209				*PDIA3P*	Body
cg19871388	20	50,419,082	1.08E-06	0.220	0.186				*SALL4*	TSS200
cg12947833	11	111,783,499	6.44E-05	0.220	0.246				*HSPB2*	5'UTR
cg24422029	10	18,549,378	2.29E-12	0.220	0.202				*CACNB2*	Body
cg07245678	2	85,641,254	0.039889	0.220	0.324					
cg14774117	6	44,310,005	5.46E-10	0.220	0.208				*SPATS1*	TSS1500
cg24698655	6	28,557,774	0.000581	0.220	0.188					
cg13997645	11	15,136,475	0.024128	0.220	0.253				*INSC*	TSS200
cg09086087	16	68,000,763	8.83E-11	0.220	0.196				*SLC12A4*	1stExon
cg27405400	2	127,839,539	7.92E-06	0.220	0.214				*BIN1*	Body
cg01670677	11	71,955,322	0.027463	0.220	0.288				*PHOX2A*	TSS200
cg03665785	22	29,458,676	1.39E-07	0.220	0.223	6.39E-13	0.216		*C22orf31*	TSS1500
cg09287650	10	29,981,216	1.08E-19	0.220	0.224				*SVIL*	5'UTR
cg02361878	1	165,326,327	2.91E-10	0.220	0.193				*LMX1A*	TSS1500
cg08029978	6	28,784,801	0.04174	0.220	0.325					
cg21293464	11	66,885,276	0.000104	0.220	0.202				*KDM2A*	TSS1500
cg26312807	3	127,057,048	3.16E-06	0.220	0.192					
cg04789403	15	80,271,218	2.15E-07	0.220	0.236					
cg06204755	17	70,484,432	1.02E-05	0.220	0.152					
cg10585661	3	184,056,618	2.09E-09	0.220	0.186				*FAM131A*	5'UTR
cg16353318	16	81,564,161	3.17E-05	0.220	0.202				*CMIP*	Body
cg24849648	7	120,964,019	1.03E-06	0.220	0.167	1.08E-07	0.128		*WNT16*	TSS1500
cg02876293	7	157,456,001	6.94E-12	0.220	0.180				*PTPRN2*	Body
cg07409292	4	90,228,685	2.25E-05	0.220	0.147				*GPRIN3*	5'UTR
cg15869071	4	2,798,158	0.019866	0.220	0.085				*SH3BP2*	5'UTR
cg26341831	1	226,036,279	0.000463	0.220	0.161				*TMEM63A*	Body
cg01580681	4	174,450,016	0.002965	0.220	0.231	1.49E-08	0.133		*HAND2*	1stExon
cg06478457	7	29,604,764	0.015825	0.220	0.169				*PRR15*	5'UTR
cg07064711	8	117,950,335	0.026126	0.220	0.138				*C8orf85*	TSS200
cg14813083	11	18,477,280	0.000342	0.220	0.175	1.24E-08	0.128		*LDHAL6A*	TSS200

cg01299332	5	79,406,275	5.86E-08	0.220	0.229					
cg18182148	1	92,952,533	6.63E-06	0.220	0.190				*GFI1*	TSS1500
cg00905746	2	857,746	0.001908	0.220	0.212					
cg04368971	14	94,917,779	0.000442	0.220	0.213				*SERPINA11*	5'UTR
cg09543819	6	49,881,441	0.043144	0.220	0.187					
cg17510278	20	21,696,115	0.006037	0.220	0.260				*PAX1*	3'UTR
cg10862535	19	46,526,483	2.25E-07	0.220	0.190	1.64E-08	0.086		*PGLYRP1*	TSS200
cg17806162	1	2,472,133	1.34E-05	0.220	0.213					
cg20689476	4	6,385,854	0.007678	0.220	0.209				*PPP2R2C*	Body
cg03593823	1	240,690,404	1.99E-06	0.220	0.212				*GREM2*	5'UTR
cg14155193	9	129,148,742	1.21E-10	0.220	0.214				*FAM125B*	Body
cg03043665	1	3,615,762	3.09E-06	0.220	0.213				*TP73*	Body
cg03186851	17	44,896,748	0.004059	0.220	0.272				*WNT3*	TSS1500
cg07982740	20	44,410,114	2.08E-11	0.220	0.215				*WFDC3*	Body
cg10544251	1	156,215,727	0.02821	0.220	0.154				*PAQR6*	5'UTR
cg26220594	1	19,110,978	0.022196	0.220	0.218					
cg08594888	12	53,183,773	0.001717	0.220	0.205				*KRT3*	3'UTR
cg26588612	6	148,562,337	1.17E-17	0.220	0.226					
cg03274456	11	62,273,116	2.33E-10	0.220	0.213				*AHNAK*	Body
cg03532263	14	103,012,356	0.001691	0.220	0.182					
cg03700024	2	74,666,700	1.99E-06	0.220	0.221				*RTKN*	Body
cg16563187	16	85,730,606	5.77E-09	0.220	0.223					
cg26576398	10	18,550,135	3.69E-09	0.220	0.197				*CACNB2*	1stExon
cg01947583	4	8,310,999	9.03E-10	0.219	0.209					
cg22191981	6	41,472,382	0.002393	0.219	0.176					
cg26698460	19	58,716,004	1.38E-13	0.219	0.230				*ZNF274*	Body
cg13904874	9	89,411,000	1.89E-09	0.219	0.202					
cg24931346	1	22,979,546	5.47E-15	0.219	0.212				*C1QB*	TSS200
cg10918171	6	170,601,120	0.042172	0.219	0.157				*DLL1*	TSS1500
cg13933734	1	119,550,484	0.000929	0.219	0.199					
cg08966889	6	52,440,429	4.79E-10	0.219	0.205				*TRAM2*	Body
cg09547119	19	52,391,367	0.034482	0.219	0.208				*ZNF577*	TSS200
cg09833911	16	88,701,212	2.95E-07	0.219	0.195					
cg24063856	17	43,863,303	0.000199	0.219	0.128				*CRHR1*	Body

cg24680629	5	177,535,066	5.4E-09	0.219	0.229						
cg26275647	7	114,648,973	6.4E-05	0.219	0.181					*MDFIC*	Body
cg09706122	12	54,071,165	0.015127	0.219	0.184					*ATP5G2*	TSS1500
cg09887059	12	113,916,664	0.003382	0.219	0.221						
cg08958168	16	68,001,415	3.2E-11	0.219	0.190					*SLC12A4*	Body
cg11241663	2	66,810,341	0.014457	0.219	0.243						
cg14349977	4	187,219,430	0.029165	0.219	0.306						
cg14785392	5	61,180,442	1.18E-06	0.219	0.167						
cg18956714	13	97,646,803	0.011495	0.219	0.282					*OXGR1*	TSS200
cg01404873	13	50,701,050	3.23E-12	0.219	0.195					*DLEU2*	TSS1500
cg07848409	3	172,166,987	0.016458	0.219	0.296					*GHSR*	TSS1500
cg09719269	12	125,033,803	0.004326	0.219	0.186						
cg12103152	6	78,173,200	0.016409	0.219	0.201					*HTR1B*	TSS200
cg13785196	1	32,646,858	0.000142	0.219	0.192					*TXLNA*	Body
cg16867777	21	32,932,073	0.0012	0.219	0.145					*TIAM1*	TSS1500
cg22851200	7	100,465,833	4.7E-14	0.219	0.210					*TRIP6*	Body
cg27398401	1	56,976,548	3.55E-06	0.219	0.206					*PPAP2B*	Body
cg11129008	3	181,428,647	7.57E-06	0.219	0.205					*SOX2OT*	Body
cg11847636	19	50,015,523	7.21E-18	0.219	0.219					*FCGRT*	TSS200
cg18674643	4	154,713,748	0.026284	0.219	0.262						
cg19951244	5	172,672,766	0.000254	0.219	0.258						
cg11678896	4	568,514	0.03275	0.219	0.233						
cg24496666	1	78,511,979	0.000104	0.219	0.205	1.62E-13	0.184			*GIPC2*	1stExon
cg23098599	19	15,235,953	0.004018	0.219	0.165					*ILVBL*	5'UTR
cg21324456	20	50,248,076	2.39E-06	0.219	0.249					*ATP9A*	Body
cg09119967	20	9,494,838	1.36E-05	0.219	0.193	1.55E-07	0.087			*C20orf103*	TSS1500
cg20803293	1	110,254,709	2.12E-07	0.219	0.189					*GSTM5*	TSS200
cg08879365	12	106,066,082	0.002079	0.219	0.125						
cg02195680	16	28,519,566	5.4E-08	0.219	0.225					*IL27*	TSS1500
cg16200879	19	4,326,975	5.7E-06	0.219	0.232					*STAP2*	Body
cg10609310	8	38,722,935	0.000304	0.219	0.192						
cg03121281	7	96,627,253	0.024925	0.219	0.231					*DLX6AS*	Body
cg05142617	2	95,690,821	2.63E-05	0.219	0.218					*MAL*	TSS1500
cg24878115	19	18,545,062	0.005723	0.219	0.084					*SSBP4*	3'UTR

cg01613010	7	20,835,627	7.54E-09	0.219	0.214					
cg04811512	20	21,686,080	0.001162	0.219	0.189				*PAX1*	TSS1500
cg13878010	3	123,167,276	0.011289	0.219	0.253	3.93E-08	0.137		*ADCY5*	1stExon
cg05736768	8	105,479,248	0.025555	0.219	0.211				*DPYS*	1stExon
cg00971695	14	65,169,664	1.35E-08	0.219	0.182					
cg03740216	19	10,397,561	0.000613	0.219	0.229	6.57E-18	0.148		*ICAM4*	TSS200
cg04505845	6	170,553,549	1.58E-05	0.219	0.190					
cg14499058	8	141,129,321	0.000103	0.219	0.233				*TRAPPC9*	Body
cg06870213	6	55,444,335	2.98E-06	0.219	0.162				*HMGCLL1*	TSS1500
cg13307880	5	140,744,367	0.02289	0.219	0.225				*PCDHGA4*	Body
cg14021133	7	20,834,283	1.27E-06	0.219	0.208					
cg21269897	6	27,107,002	2.72E-05	0.219	0.210	5.33E-09	0.119		*HIST1H2BK*	3'UTR
cg00805880	1	203,045,044	0.011514	0.219	0.136				*PPFIA4*	Body
cg01106926	4	13,539,255	0.011693	0.219	0.298					
cg18361098	14	37,136,391	0.007556	0.219	0.154				*PAX9*	Body
cg03609488	12	12,044,761	2E-13	0.219	0.201				*ETV6*	3'UTR
cg12062282	19	49,524,228	0.011285	0.219	0.261					
cg12731773	11	846,055	9.38E-27	0.219	0.218				*TSPAN4*	5'UTR
cg19518104	8	70,981,345	4.01E-06	0.219	0.206				*PRDM14*	Body
cg26630171	19	3,459,270	2.84E-13	0.219	0.227				*NFIC*	Body
cg03319497	5	145,722,730	0.000891	0.219	0.216					
cg19244428	6	3,229,233	0.041584	0.219	0.282				*TUBB2B*	TSS1500
cg20564283	8	49,469,560	0.005978	0.219	0.218					
cg04751149	2	66,673,449	2.41E-05	0.219	0.215				*MEIS1*	Body
cg08009275	17	79,790,191	6.32E-30	0.219	0.206				*FAM195B*	5'UTR
cg20194973	22	50,524,676	2.44E-05	0.219	0.189				*MLC1*	TSS1500
cg01513063	17	75,470,567	2.22E-05	0.219	0.174				*SEPT9*	Body
cg02382400	20	3,218,487	1.47E-20	0.219	0.207				*SLC4A11*	TSS200
cg05141333	8	145,048,363	3.08E-21	0.219	0.211				*PLEC1*	Body
cg23338993	2	234,600,423	4.94E-09	0.219	0.190				*UGT1A10*	Body
cg03101021	7	126,891,319	0.008794	0.219	0.296				*GRM8*	5'UTR
cg04671932	7	79,082,898	0.005417	0.219	0.186				*MAGI2*	TSS200
cg05719612	10	18,629,545	0.005101	0.219	0.162				*CACNB2*	Body
cg12348202	7	158,381,023	6.46E-32	0.219	0.209				*PTPRN2*	TSS1500

cg12998503	4	184,177,628	1.24E-06	0.219	0.237				*WWC2*	Body
cg14503636	13	113,611,556	1.79E-17	0.219	0.215					
cg08764162	10	31,147,088	4.5E-12	0.219	0.227				*ZNF438*	Body
cg22044002	10	94,825,547	0.003611	0.219	0.092				*CYP26C1*	Body
cg07793033	16	85,256,423	5.23E-13	0.219	0.210					
cg11070059	7	96,626,399	0.035546	0.219	0.230				*DLX6AS*	Body
cg11382529	8	65,490,613	0.041189	0.219	0.165				*LOC401463*	TSS1500
cg14021168	17	8,242,177	5.94E-18	0.219	0.199				*ODF4*	TSS1500
cg23000806	8	48,854,968	0.014792	0.219	0.098				*PRKDC*	Body
cg20374595	13	100,627,956	0.000441	0.219	0.204					
cg12399687	15	66,949,579	2.1E-11	0.219	0.233					
cg15106134	3	181,428,466	3.27E-06	0.219	0.215				*SOX2OT*	Body
cg25560247	6	31,832,173	7.39E-09	0.219	0.188				*NEU1*	TSS1500
cg11479156	11	70,672,388	1.8E-14	0.219	0.193				*SHANK2*	Body
cg00271210	6	167,070,053	1.37E-07	0.219	0.160				*RPS6KA2*	Body
cg18697632	5	77,140,985	0.006703	0.219	0.234					
cg24324985	20	22,566,884	2E-05	0.219	0.211				*FOXA2*	TSS1500
cg03787603	7	101,768,610	4.83E-09	0.219	0.221				*CUX1*	Body
cg15989981	13	20,736,009	1.31E-05	0.219	0.197				*GJA3*	TSS1500
cg22970173	22	41,637,080	6.97E-08	0.219	0.198				*CHADL*	TSS200
cg10919664	6	28,945,322	3.31E-05	0.219	0.208					
cg12408601	6	27,841,665	0.044671	0.219	0.249				*HIST1H4L*	TSS1500
cg26461855	6	30,695,686	1.57E-13	0.219	0.210				*FLOT1*	3'UTR
cg07610192	2	131,130,423	0.038749	0.219	0.185				*PTPN18*	Body
cg05395187	12	124,913,717	6.05E-20	0.219	0.213				*NCOR2*	Body
cg15076318	6	32,847,788	8.15E-06	0.219	0.253				*PPP1R2P1*	Body
cg26710891	17	2,324,923	1.45E-06	0.219	0.242				*METT10D*	Body
cg04872675	12	108,983,234	0.006315	0.219	0.237					
cg10529613	14	25,287,305	0.006779	0.219	0.234				*STXBP6*	Body
cg07665260	20	42,544,086	0.026952	0.219	0.144				*TOX2*	TSS1500
cg16750777	16	50,583,441	1.43E-11	0.219	0.212				*NKD1*	Body
cg20927731	15	74,726,561	0.001551	0.219	0.133				*SEMA7A*	TSS1500
cg11071231	8	57,069,907	0.013398	0.219	0.120					
cg03425110	10	71,333,037	0.02002	0.219	0.248	0.0000412	0.089		*NEUROG3*	5'UTR

cg13866253	11	77,093,001	1.7E-15	0.219	0.220				*PAK1*	Body
cg15556591	4	1,027,967	1.65E-07	0.219	0.203					
cg23247185	3	14,530,356	6.29E-06	0.219	0.250				*SLC6A6*	3'UTR
cg13508175	10	88,296,364	0.000843	0.219	0.192					
cg27016494	7	96,651,586	8.67E-10	0.219	0.208	0.00106596	0.064		*DLX5*	Body
cg12930100	3	181,428,697	1.23E-06	0.219	0.202				*SOX2OT*	Body
cg21771463	3	44,626,400	0.007285	0.219	0.093				*ZNF660*	TSS200
cg01344171	6	43,612,647	1.17E-07	0.219	0.194	1.47E-15	0.173		*RSPH9*	TSS200
cg14188840	7	27,220,398	1.24E-10	0.219	0.202				*HOXA10*	TSS1500
cg17065262	17	27,506,097	0.033603	0.219	0.124				*MYO18A*	5'UTR
cg00407944	9	139,424,152	1.03E-12	0.219	0.196				*NOTCH1*	Body
cg08202144	8	1,004,177	0.001666	0.219	0.209					
cg27577993	7	151,454,795	6.87E-10	0.219	0.224				*PRKAG2*	Body
cg07047653	12	50,344,626	3.15E-22	0.219	0.204	0.00019757	0.044		*AQP2*	1stExon
cg10027723	1	51,888,574	4.63E-11	0.219	0.203				*EPS15*	TSS1500
cg12041056	22	27,053,247	0.04812	0.219	0.188				*MIAT*	TSS1500
cg08134091	6	11,213,750	4.07E-14	0.219	0.223				*NEDD9*	Body
cg16706389	10	1,568,812	0.000745	0.219	0.165				*ADARB2*	Body
cg18255353	4	154,712,422	0.040354	0.219	0.209					
cg24824371	7	158,075,968	5.73E-14	0.219	0.227				*PTPRN2*	Body
cg07608565	19	55,595,022	2.71E-11	0.219	0.205				*EPS8L1*	Body
cg24088496	11	96,071,506	1.51E-07	0.219	0.214				*MAML2*	Body
cg25824509	3	42,701,690	1.63E-05	0.219	0.256				*ZBTB47*	Body
cg26827533	8	22,458,122	3.32E-10	0.219	0.222				*C8orf58*	Body
cg01948850	3	11,651,536	1.84E-18	0.219	0.194				*VGLL4*	Body
cg06325209	1	162,602,217	1.52E-08	0.219	0.213				*DDR2*	TSS200
cg12929809	12	98,150,305	3.87E-06	0.219	0.227					
cg13560452	6	137,813,584	5.58E-06	0.219	0.213				*OLIG3*	1stExon
cg15223899	22	24,891,158	0.003054	0.219	0.246				*C22orf45*	TSS1500
cg16301004	10	106,082,537	0.003215	0.219	0.215				*ITPRIP*	5'UTR
cg21829328	22	24,188,134	4.74E-05	0.219	0.267					
cg06015218	6	146,350,434	0.004175	0.219	0.186	4.56E-20	0.140		*GRM1*	5'UTR
cg07838048	16	70,720,440	1.31E-11	0.219	0.196				*MTSS1L*	TSS1500
cg07272264	12	101,111,683	0.011881	0.219	0.256					

cg23379208	6	30,522,674	6.16E-13	0.219	0.203				*GNL1*	Body
cg17825384	1	31,380,853	0.00972	0.219	0.204				*SDC3*	Body
cg21085701	19	12,033,228	2.97E-10	0.219	0.207					
cg00736406	16	23,504,930	1.18E-08	0.219	0.222				*GGA2*	Body
cg20670946	12	122,502,170	6.22E-10	0.219	0.206					
cg23547157	12	133,199,323	2.99E-10	0.219	0.215					
cg14391586	20	62,681,296	0.024218	0.219	0.270				*SOX18*	TSS1500
cg00148862	2	1,286,394	0.000134	0.219	0.141				*SNTG2*	Body
cg02509058	5	2,753,746	0.00997	0.219	0.271				*C5orf38*	Body
cg08454507	17	78,755,406	9.41E-13	0.219	0.215				*RPTOR*	Body
cg13901675	9	109,460,023	6.06E-20	0.219	0.199					
cg19084629	12	117,407,879	2.41E-32	0.219	0.218				*FBXW8*	Body
cg02391509	3	27,766,382	0.002892	0.219	0.265					
cg07333191	4	13,526,769	0.017696	0.219	0.235					
cg25461801	13	100,630,316	0.000473	0.219	0.209					
cg10670430	3	196,755,688	4.74E-10	0.219	0.200				*MFI2*	Body
cg03834567	2	218,808,745	8.17E-19	0.219	0.213				*TNS1*	1stExon
cg16533767	2	23,736,552	0.000572	0.219	0.193				*KLHL29*	5'UTR
cg25534244	17	53,341,098	5.23E-09	0.219	0.186				*HLF*	TSS1500
cg00902823	17	71,162,349	0.013187	0.219	0.138				*SSTR2*	5'UTR
cg13726724	X	47,483,268	0.039925	0.219	0.064					
cg08145373	11	2,407,008	5.78E-15	0.219	0.202				*CD81*	Body
cg10179300	5	14,147,618	5.49E-06	0.219	0.158				*TRIO*	Body
cg11621464	19	41,120,268	5.38E-08	0.219	0.179				*LTBP4*	Body
cg24024056	10	102,415,556	0.038691	0.219	0.175					
cg02744113	2	73,147,030	0.000775	0.219	0.181				*EMX1*	Body
cg14482569	11	130,185,651	7.15E-10	0.219	0.213				*ZBTB44*	TSS1500
cg01408486	5	139,027,024	3.12E-08	0.219	0.183				*CXXC5*	TSS1500
cg06654537	13	100,641,317	0.000722	0.219	0.181					
cg15172053	8	105,342,214	5.85E-07	0.219	0.205					
cg18144593	2	71,116,411	0.010996	0.219	0.216					
cg00182758	17	43,047,971	0.001042	0.219	0.187					
cg00980060	1	14,849,511	8.77E-06	0.219	0.212			Yes		
cg16617366	18	46,064,816	0.000571	0.219	0.099				*KIAA0427*	TSS1500

cg23699274	12	40,618,284	0.001951	0.219	0.172				*LRRK2*	TSS1500
cg10016788	19	55,599,029	9.34E-07	0.219	0.211				*EPS8L1*	3'UTR
cg12140975	1	33,351,568	0.014846	0.219	0.237				*HPCA*	TSS1500
cg23621496	1	211,667,487	6.56E-34	0.219	0.211			Yes	*RD3*	TSS1500
cg05852760	7	23,508,224	0.040024	0.219	0.275				*IGF2BP3*	Body
cg22344631	2	177,012,133	7.03E-06	0.219	0.174					
cg21216477	12	54,440,773	5.62E-05	0.219	0.191				*HOXC4*	5'UTR
cg13850355	4	47,034,939	0.049851	0.219	0.241				*GABRB1*	Body
cg14093255	9	14,081,508	7.66E-09	0.219	0.188					
cg20553124	6	36,253,157	0.001843	0.219	0.216				*PNPLA1*	5'UTR
cg04787888	16	86,547,322	0.00201	0.219	0.215				*FOXF1*	3'UTR
cg05451094	17	26,557,385	4.85E-07	0.219	0.199					
cg20257866	8	134,203,235	0.000228	0.219	0.248				*WISP1*	TSS200
cg07685869	16	57,836,706	0.000469	0.218	0.192	1.2191E-05	0.081		*KIFC3*	TSS1500
cg10604168	15	65,360,370	0.035171	0.218	0.111	4.41E-16	0.140		*RASL12*	5'UTR
cg23987137	5	58,653,624	4.07E-08	0.218	0.222				*PDE4D*	Body
cg26140475	8	126,525,558	0.002119	0.218	0.205					
cg02516189	9	139,268,092	4.75E-11	0.218	0.216	3.94E-22	0.153		*CARD9*	1stExon
cg04158698	1	201,523,041	1.58E-09	0.218	0.213					
cg11393995	6	2,842,359	0.019276	0.218	0.195				*SERPINB1*	TSS1500
cg18628367	7	157,475,548	8.26E-06	0.218	0.200				*PTPRN2*	Body
cg01616178	8	41,755,140	0.037745	0.218	0.147				*ANK1*	TSS1500
cg21289397	8	41,510,988	0.029817	0.218	0.287				*ANK1*	3'UTR
cg21752517	11	74,951,359	1.74E-05	0.218	0.173					
cg24766308	5	147,162,432	0.036432	0.218	0.180				*JAKMIP2*	TSS200
cg03137071	8	49,496,369	8.46E-10	0.218	0.221					
cg13060157	1	113,286,610	0.016431	0.218	0.177					
cg13565138	9	91,614,156	9.82E-15	0.218	0.209				*S1PR3*	5'UTR
cg21042919	17	36,621,139	7.03E-06	0.218	0.223				*ARHGAP23*	Body
cg03160935	9	132,242,593	4.06E-07	0.218	0.226					
cg05501320	8	75,263,248	0.001588	0.218	0.148				*GDAP1*	Body
cg13490235	1	217,004,524	2.14E-06	0.218	0.181				*ESRRG*	5'UTR
cg19806642	5	178,422,140	0.036466	0.218	0.255				*GRM6*	TSS200
cg18088442	18	43,355,547	9.5E-05	0.218	0.192					

cg02657654	2	45,154,029	3.4E-06	0.218	0.224					
cg12461141	11	5,710,654	1.03E-07	0.218	0.229	0.00018243	0.099		*TRIM22*	TSS1500
cg17290213	11	65,344,355	4.14E-10	0.218	0.213				*EHBP1L1*	Body
cg23350294	8	57,359,992	0.001246	0.218	0.235				*PENK*	TSS1500
cg23740610	17	72,257,002	2.7E-11	0.218	0.222				*TTYH2*	3'UTR
cg02705759	4	7,283,005	0.002454	0.218	0.152				*SORCS2*	Body
cg26184501	17	48,139,723	4.58E-16	0.218	0.194				*ITGA3*	Body
cg03217729	16	11,222,890	7.43E-10	0.218	0.210				*CLEC16A*	Body
cg05755899	17	80,794,139	2.99E-05	0.218	0.190				*TBCD*	Body
cg12279419	6	33,943,662	2.09E-06	0.218	0.208					
cg21325822	3	154,497,591	0.000431	0.218	0.230					
cg09025327	1	9,309,004	7.08E-10	0.218	0.232				*H6PD*	Body
cg26519184	21	43,237,089	0.02272	0.218	0.288				*PRDM15*	Body
cg27297263	6	86,160,468	0.008582	0.218	0.249				*NT5E*	Body
cg18175809	3	62,355,525	0.023052	0.218	0.272				*FEZF2*	3'UTR
cg24402975	19	1,009,732	1.82E-21	0.218	0.192				*C19orf6*	3'UTR
cg09980058	19	18,898,325	0.000491	0.218	0.227				*COMP*	Body
cg16551732	4	169,799,087	0.028296	0.218	0.111				*PALLD*	Body
cg00857921	1	92,257,380	2.29E-13	0.218	0.220				*TGFBR3*	Body
cg11385338	7	63,250,817	0.000175	0.218	0.238					
cg12097883	17	62,774,939	0.008532	0.218	0.124				*LOC146880*	Body
cg17510121	6	35,182,910	8.2E-10	0.218	0.210				*SCUBE3*	Body
cg13869942	1	151,878,061	1.02E-08	0.218	0.198				*THEM4*	Body
cg21062347	11	62,783,543	5.5E-09	0.218	0.222	1.19E-08	0.092		*SLC22A8*	TSS1500
cg23864949	8	99,984,534	2.35E-05	0.218	0.232					
cg02108620	3	42,002,230	3.4E-10	0.218	0.227				*ULK4*	5'UTR
cg09665332	1	67,022,173	1.69E-06	0.218	0.211				*SGIP1*	Body
cg01020413	2	66,810,487	0.000172	0.218	0.220					
cg18164305	11	4,212,446	1.06E-06	0.218	0.195					
cg24598145	8	1,955,069	4.79E-05	0.218	0.215				*KBTBD11*	3'UTR
cg03653026	9	135,115,118	3.19E-05	0.218	0.192				*NTNG2*	Body
cg09735723	10	106,402,042	0.012645	0.218	0.163				*SORCS3*	Body
cg14542839	11	35,547,074	0.000104	0.218	0.179	4.79E-16	0.164		*PAMR1*	1stExon
cg00364016	6	10,113,648	2.73E-06	0.218	0.223					

cg12068280	19	46,804,528	4E-08	0.218	0.202				*HIF3A*	5'UTR
cg13137458	11	77,160,347	1.55E-05	0.218	0.171				*PAK1*	5'UTR
cg17717169	1	178,621,037	1.08E-05	0.218	0.200					
cg02227292	6	28,442,848	0.009898	0.218	0.228					
cg03561565	8	99,438,624	0.000503	0.218	0.153	5.08E-11	0.145		*KCNS2*	TSS1500
cg10236245	14	95,234,015	1.9E-06	0.218	0.214					
cg17966192	2	108,994,116	0.022007	0.218	0.093	2.73E-16	0.155		*SULT1C4*	TSS1500
cg19283806	18	66,389,420	0.033831	0.218	0.119				*CCDC102B*	5'UTR
cg02634341	1	179,711,583	0.040683	0.218	0.222				*FAM163A*	TSS1500
cg16601461	2	114,048,829	2.69E-10	0.218	0.195				*LOC440839*	Body
cg26393629	14	21,191,859	0.002364	0.218	0.168					
cg06901893	7	73,400,692	7.72E-11	0.218	0.221					
cg12137450	8	22,458,046	1.21E-08	0.218	0.212				*C8orf58*	Body
cg12893030	10	73,769,174	7.06E-10	0.218	0.197				*CHST3*	3'UTR
cg13536107	4	153,437,193	1.11E-20	0.218	0.202				*FBXW7*	5'UTR
cg09903430	1	44,172,605	0.043135	0.218	0.103	4.12E-11	0.099		*ST3GAL3*	TSS1500
cg15422685	3	62,356,774	0.002301	0.218	0.249				*FEZF2*	Body
cg01309213	16	331,821	0.01242	0.218	0.236				*ARHGDIG*	Body
cg07107824	10	95,633,260	4.31E-08	0.218	0.248					
cg06563450	5	55,737,211	0.01756	0.218	0.212					
cg07562821	15	43,810,131	0.000119	0.218	0.251				*MAP1A*	5'UTR
cg19412669	1	36,839,270	0.016189	0.218	0.170				*STK40*	5'UTR
cg09829636	14	102,976,856	0.028254	0.218	0.143				*ANKRD9*	TSS1500
cg17220055	1	42,248,998	5.06E-06	0.218	0.182				*HIVEP3*	5'UTR
cg10342908	15	43,415,239	0.007311	0.218	0.165					
cg10694507	3	134,076,315	2.89E-21	0.218	0.211				*AMOTL2*	3'UTR
cg22125433	8	143,386,979	1.8E-05	0.218	0.205				*TSNARE1*	Body
cg25557175	11	32,193,776	0.003442	0.218	0.251					
cg08061755	11	9,781,826	9.93E-10	0.218	0.203					
cg13821785	7	2,657,347	7.28E-13	0.218	0.218					
cg24990131	6	6,320,710	0.043014	0.218	0.164				*F13A1*	5'UTR
cg13520394	12	57,635,339	2.55E-05	0.218	0.175				*NDUFA4L2*	TSS1500
cg23402821	17	35,292,159	0.002605	0.218	0.195					
cg03343013	1	43,473,097	7.46E-05	0.218	0.177					

cg05496768	10	77,872,288	9.67E-12	0.218	0.190				*C10orf11*	Body
cg06958766	14	76,446,087	0.007189	0.218	0.320				*TGFB3*	Body
cg08495770	10	106,400,824	0.016329	0.218	0.087				*SORCS3*	TSS200
cg09926027	9	87,285,693	0.002954	0.218	0.222				*NTRK2*	Body
cg23104954	13	50,701,501	8.14E-05	0.218	0.202					
cg03696974	16	29,888,670	0.000124	0.218	0.201				*SEZ6L2*	Body
cg03778207	11	86,230,664	1.14E-09	0.218	0.221				*ME3*	Body
cg08529825	1	114,489,550	2.2E-05	0.218	0.215				*HIPK1*	Body
cg15095327	3	9,944,512	4.07E-06	0.218	0.223	4.69E-14	0.144		*IL17RE*	1stExon
cg18661379	10	104,393,081	0.000126	0.218	0.205				*SUFU*	3'UTR
cg01421548	16	25,705,355	0.000479	0.218	0.234				*HS3ST4*	Body
cg20286200	6	133,562,267	0.005276	0.218	0.273	9.48E-16	0.167		*EYA4*	TSS1500
cg01580934	7	104,909,815	0.021025	0.218	0.226				*SRPK2*	TSS1500
cg03840647	14	36,974,968	0.004835	0.218	0.248				*SFTA3*	Body
cg12222588	18	34,823,808	1.19E-06	0.218	0.228				*BRUNOL4*	3'UTR
cg12415060	15	26,950,274	1.41E-08	0.218	0.173				*GABRB3*	Body
cg14364903	3	48,631,834	7.02E-08	0.218	0.200				*COL7A1*	Body
cg27552679	15	74,425,757	0.004396	0.218	0.193				*ISLR2*	Body
cg06186450	1	154,942,910	0.000709	0.218	0.232				*SHC1*	5'UTR
cg13761284	9	130,708,393	5.67E-15	0.218	0.212				*FAM102A*	Body
cg14215082	8	9,055,850	2.03E-12	0.218	0.210					
cg17031967	6	25,297,487	3.8E-10	0.218	0.220				*LRRC16A*	Body
cg04752042	1	46,951,683	0.003711	0.218	0.181					
cg05962055	4	141,566,966	1.14E-14	0.218	0.217				*TBC1D9*	Body
cg20236750	10	95,516,908	1.01E-10	0.218	0.219				*LGI1*	TSS1500
cg02098752	5	176,024,005	0.020091	0.218	0.175				*GPRIN1*	Body
cg18741372	19	45,906,156	2.17E-06	0.218	0.189				*PPP1R13L*	5'UTR
cg25447873	15	63,548,963	4.23E-09	0.218	0.219				*RAB8B*	Body
cg27301674	8	885,217	5.52E-06	0.218	0.224					
cg01072639	20	5,891,781	1.09E-05	0.218	0.183				*CHGB*	TSS200
cg03357664	1	29,446,693	1.88E-07	0.218	0.234				*TMEM200B*	3'UTR
cg07155381	17	180,856	4.02E-12	0.218	0.193				*RPH3AL*	5'UTR
cg16824584	20	31,170,822	6.4E-06	0.218	0.181					
cg06897860	1	54,965,458	0.037024	0.218	0.284					

cg00566320	1	32,172,204	2.06E-06	0.218	0.258					
cg00880452	19	39,694,617	0.007453	0.218	0.176				*SYCN*	1stExon
cg00943382	7	156,786,185	1.51E-08	0.218	0.208					
cg10682560	11	31,845,148	0.015151	0.218	0.293					
cg15117372	6	35,992,219	7.94E-15	0.218	0.203				*SLC26A8*	5'UTR
cg16421871	13	113,634,978	2.07E-06	0.218	0.198				*MCF2L*	Body
cg24085885	19	18,879,446	8.2E-26	0.218	0.227				*CRTC1*	Body
cg26386624	12	54,366,859	0.000292	0.218	0.266				*HOXC11*	TSS200
cg12421834	6	168,714,673	3.75E-12	0.218	0.197				*DACT2*	Body
cg04137171	15	79,104,232	7.12E-07	0.218	0.146				*ADAMTS7*	TSS1500
cg04303330	5	131,992,430	1.66E-08	0.218	0.208				*IL13*	TSS1500
cg11381404	3	104,813,499	2.54E-06	0.218	0.212					
cg00213123	15	75,019,070	8.97E-10	0.218	0.203				*CYP1A1*	TSS1500
cg02895192	X	153,190,027	0.006909	0.218	0.227				*ARHGAP4*	Body
cg27295595	16	29,796,756	2.31E-07	0.218	0.226					
cg13733266	11	85,397,216	1.55E-06	0.218	0.175				*CCDC89*	1stExon
cg16312968	6	6,900,945	6.56E-10	0.218	0.202					
cg17840501	17	75,385,086	1.52E-07	0.218	0.205				*SEPT9*	5'UTR
cg20205704	17	2,213,196	1.49E-12	0.218	0.202				*SRR*	5'UTR
cg00436476	6	26,240,505	0.04066	0.218	0.264				*HIST1H4F*	TSS200
cg06196801	12	121,164,025	8.72E-05	0.218	0.196				*ACADS*	Body
cg23102014	15	70,574,295	5.69E-05	0.218	0.233					
cg00699219	4	174,422,442	0.000183	0.218	0.247					
cg07196577	1	109,850,486	1.92E-10	0.218	0.215				*MYBPHL*	TSS1500
cg14925328	2	207,913,718	3.01E-11	0.218	0.218					
cg17605814	11	44,585,659	2.19E-06	0.218	0.219				*CD82*	TSS1500
cg23953176	2	947,515	4.49E-15	0.218	0.206				*SNTG2*	Body
cg11960629	10	91,295,317	0.028464	0.218	0.253				*SLC16A12*	TSS200
cg04138103	5	38,796,759	2.3E-09	0.218	0.218					
cg13761321	9	97,768,771	0.022448	0.218	0.127				*C9orf3*	Body
cg14657725	8	99,959,549	2.73E-07	0.218	0.223				*OSR2*	5'UTR
cg14703061	11	120,435,915	0.023812	0.218	0.175					
cg16574134	10	102,588,315	0.04627	0.218	0.140				*PAX2*	3'UTR
cg06973615	2	175,208,761	0.00084	0.218	0.225					

cg10568624	9	100,619,991	0.013054	0.218	0.250					
cg15413523	5	133,451,625	7.52E-15	0.218	0.185				*TCF7*	5'UTR
cg07378309	3	121,312,164	6.78E-05	0.218	0.220				*FBXO40*	TSS200
cg14067761	2	23,840,739	6.72E-05	0.218	0.231				*KLHL29*	Body
cg15058645	2	175,528,343	1.35E-13	0.218	0.211				*WIPF1*	5'UTR
cg23142799	13	26,625,089	0.000967	0.218	0.095				*SHISA2*	1stExon
cg00835279	2	3,642,710	2.22E-06	0.218	0.171				*COLEC11*	1stExon
cg09467436	2	72,079,424	5.13E-17	0.218	0.197					
cg12169875	6	29,856,193	0.023781	0.218	0.177				*HLA-H*	Body
cg24575083	11	7,695,485	9.21E-06	0.218	0.187				*CYB5R2*	TSS1500
cg01154355	5	124,043,109	8.2E-05	0.218	0.229				*ZNF608*	Body
cg03695236	7	157,369,121	0.001295	0.218	0.146				*PTPRN2*	Body
cg11575295	7	158,250,911	2.37E-06	0.218	0.221				*PTPRN2*	Body
cg19122260	17	78,808,570	1.15E-11	0.218	0.212				*RPTOR*	Body
cg11583762	8	39,172,020	0.011076	0.218	0.232				*ADAM5P*	TSS200
cg18449021	5	107,004,816	5.37E-06	0.218	0.224				*EFNA5*	Body
cg18984103	14	104,163,025	2.29E-11	0.218	0.217				*KLC1*	Body
cg20793420	22	38,861,635	3.55E-06	0.218	0.242					
cg04478180	4	76,932,862	0.003387	0.218	0.233				*ART3*	5'UTR
cg05215272	17	6,899,095	1.64E-05	0.218	0.186				*ALOX12*	TSS1500
cg16711124	16	29,796,373	2.08E-06	0.218	0.214					
cg09541576	2	44,873,248	5.51E-08	0.218	0.229			Yes	*C2orf34*	Body
cg08443357	20	1,310,000	0.000272	0.218	0.206				*SDCBP2*	TSS200
cg00350199	11	67,418,213	1.8E-13	0.218	0.198				*ACY3*	TSS200
cg23698978	7	20,838,631	1.44E-05	0.218	0.164					
cg05121790	1	12,123,554	0.015257	0.218	0.130				*TNFRSF8*	5'UTR
cg02124022	4	54,974,886	5.26E-06	0.218	0.219					
cg00542992	5	141,595,654	0.015826	0.218	0.182					
cg11781986	14	24,808,787	7.42E-12	0.218	0.229				*RIPK3*	Body
cg26422458	1	79,472,452	7.51E-08	0.218	0.208				*ELTD1*	5'UTR
cg13910813	9	117,157,614	0.000185	0.218	0.240				*AKNA*	TSS1500
cg27090062	5	92,939,842	1.12E-09	0.218	0.211					
cg19951298	6	10,883,054	2.6E-07	0.218	0.195				*GCM2*	TSS1500
cg12426146	4	140,657,110	1.33E-06	0.218	0.205				*MAML3*	Body

cg03408271	4	13,537,647	0.010257	0.218	0.221					
cg11133524	8	145,104,106	0.003182	0.218	0.197					
cg08635097	13	44,833,857	4.35E-05	0.218	0.177					
cg14447193	9	87,433,864	1.81E-08	0.218	0.188				*NTRK2*	Body
cg16739178	16	85,470,674	0.003023	0.218	0.169					
cg24813038	4	85,404,026	9.57E-05	0.218	0.199					
cg11270393	2	96,991,017	0.022609	0.218	0.291				*ITPRIPL1*	TSS1500
cg04585669	3	11,599,640	1.07E-05	0.218	0.245				*VGLL4*	3'UTR
cg06361606	1	154,915,180	5.58E-08	0.218	0.219					
cg08826839	2	163,100,125	0.000594	0.218	0.175	3.17E-07	0.129		*FAP*	TSS200
cg15135803	1	12,579,742	5.15E-10	0.218	0.225					
cg00786685	2	218,842,585	1.61E-11	0.218	0.225					
cg05380019	7	19,146,555	0.00633	0.218	0.192					
cg10298052	10	57,391,055	8.44E-07	0.218	0.179					
cg15111638	11	16,631,993	1.08E-06	0.218	0.215					
cg18616175	20	39,321,473	0.000916	0.218	0.226					
cg19651694	11	71,955,164	0.003262	0.218	0.190				*PHOX2A*	1stExon
cg20852250	3	72,149,422	5.12E-07	0.218	0.203					
cg13207501	1	38,470,665	9.06E-05	0.218	0.178				*FHL3*	5'UTR
cg23601374	2	240,213,173	1.18E-10	0.218	0.207				*HDAC4*	Body
cg03677492	17	80,273,969	0.000964	0.217	0.214				*CD7*	Body
cg00491180	16	2,892,604	0.00057	0.217	0.224				*TMPRSS8*	Body
cg12352960	10	43,428,578	0.026322	0.217	0.357					
cg02945385	3	9,246,501	3.62E-20	0.217	0.209				*SRGAP3*	Body
cg12046758	17	59,474,922	0.033986	0.217	0.085					
cg13493563	9	132,260,096	3.67E-08	0.217	0.227					
cg10727673	3	136,537,267	9.25E-08	0.217	0.219				*TMEM22*	TSS1500
cg06825661	18	13,260,398	0.007262	0.217	0.218				*C18orf1*	5'UTR
cg06850005	10	35,926,564	6.08E-08	0.217	0.202					
cg21430685	8	29,387,008	9.69E-06	0.217	0.184					
cg14806083	6	169,002,120	6.76E-17	0.217	0.206				*SMOC2*	Body
cg20774846	8	105,479,420	0.000702	0.217	0.199	6.23E-21	0.159		*DPYS*	TSS200
cg12993163	3	157,821,407	0.048988	0.217	0.194				*SHOX2*	Body
cg07229688	18	59,564,213	0.000141	0.217	0.262					

cg20449379	8	968,983	1.33E-10	0.217	0.206					
cg00782828	1	54,940,382	0.000105	0.217	0.089					
cg02485922	16	30,379,391	5.97E-05	0.217	0.176				*TBC1D10B*	Body
cg05210266	8	65,282,507	0.006109	0.217	0.277					
cg13515064	13	113,611,440	6.28E-24	0.217	0.205					
cg17355191	9	136,039,576	0.000199	0.217	0.134				*GBGT1*	TSS1500
cg07866001	5	14,440,492	5.53E-09	0.217	0.222				*TRIO*	Body
cg14770102	11	58,984,446	0.038365	0.217	0.157					
cg18262201	10	6,187,854	2.87E-06	0.217	0.194				*PFKFB3*	Body
cg20238355	7	100,612,155	0.010952	0.217	0.299				*MUC12*	TSS1500
cg02102456	2	100,824,336	1.01E-20	0.217	0.200					
cg03024301	6	52,440,589	8.37E-11	0.217	0.206				*TRAM2*	Body
cg11105610	17	76,976,352	1.89E-09	0.217	0.190	1.48E-16	0.139		*LGALS3BP*	TSS1500
cg24634746	1	7,538,723	0.00073	0.217	0.250				*CAMTA1*	Body
cg23222745	6	27,791,912	0.001437	0.217	0.187				*HIST1H4J*	1stExon
cg15056128	17	38,821,487	0.010309	0.217	0.213				*KRT222*	TSS200
cg05231308	2	27,531,163	4.93E-09	0.217	0.196				*UCN*	TSS200
cg11549025	7	75,994,082	0.00011	0.217	0.232					
cg18127395	7	95,433,546	2.89E-06	0.217	0.197				*DYNC1I1*	5'UTR
cg05519187	17	35,303,387	0.001127	0.217	0.307					
cg09113474	6	1,601,369	9E-19	0.217	0.183					
cg10233454	12	57,529,389	6.67E-08	0.217	0.221				*LRP1*	Body
cg16276982	15	29,968,032	5.41E-10	0.217	0.194					
cg00073837	2	223,177,008	0.01728	0.217	0.224					
cg00146004	16	3,241,434	0.007985	0.217	0.184					
cg18556792	2	43,445,934	0.003007	0.217	0.203					
cg14501219	17	62,775,713	0.000727	0.217	0.131				*LOC146880*	Body
cg19795292	2	102,952,285	1.7E-10	0.217	0.227				*IL1RL1*	5'UTR
cg26258213	10	127,220,321	3.96E-05	0.217	0.215					
cg01700462	20	45,179,230	1.69E-09	0.217	0.199				*C20orf123*	TSS200
cg08579858	22	29,709,964	0.000386	0.217	0.226				*RASL10A*	Body
cg10256976	11	20,078,682	0.000101	0.217	0.193				*NAV2*	Body
cg14138540	15	93,571,988	0.000153	0.217	0.276					
cg15301794	11	31,822,243	2.64E-05	0.217	0.178				*PAX6*	Body

cg18212762	11	64,556,654	8.03E-12	0.217	0.218				*MAP4K2*	3'UTR
cg00921973	9	130,701,076	1.14E-08	0.217	0.232				*DPM2*	TSS1500
cg10767223	5	1,295,799	2.25E-07	0.217	0.201				*TERT*	TSS1500
cg00478479	19	40,904,851	1.83E-06	0.217	0.206				*PRX*	Body
cg25800500	2	97,505,275	1.92E-05	0.217	0.202				*ANKRD23*	3'UTR
cg19601035	12	48,399,140	9.65E-07	0.217	0.201	0.00000112	0.073		*COL2A1*	TSS1500
cg13284614	3	52,569,169	2.28E-05	0.217	0.184				*NT5DC2*	TSS200
cg13812927	9	89,517,863	2.61E-06	0.217	0.219					
cg16235891	7	158,243,546	1.15E-09	0.217	0.219				*PTPRN2*	Body
cg19262637	1	240,783,328	0.001911	0.217	0.168					
cg19594218	1	62,660,556	0.000816	0.217	0.187				*L1TD1*	1stExon
cg23525438	6	17,016,226	3.72E-06	0.217	0.241					
cg11847222	5	77,268,624	0.003817	0.217	0.205					
cg19236794	1	1,181,769	3.39E-10	0.217	0.217				*FAM132A*	Body
cg26927232	17	2,135,631	9.95E-15	0.217	0.219				*SMG6*	Body
cg27404023	2	162,283,994	0.039093	0.217	0.183					
cg25500196	17	40,464,780	3.08E-15	0.217	0.215					
cg10376827	10	88,730,324	0.000647	0.217	0.166				*AGAP11*	TSS200
cg12427162	1	168,198,488	9.06E-14	0.217	0.186				*SFT2D2*	Body
cg13017239	2	131,185,379	0.002504	0.217	0.247					
cg22942704	1	20,813,574	0.013389	0.217	0.100				*CAMK2N1*	TSS1500
cg13208704	17	36,761,029	8.27E-07	0.217	0.211				*SRCIN1*	Body
cg13583230	14	24,809,297	4.58E-05	0.217	0.200				*RIPK3*	TSS200
cg18210365	15	65,066,710	1.67E-11	0.217	0.215				*RBPMS2*	Body
cg22365906	12	99,548,546	4.85E-07	0.217	0.177				*ANKS1B*	TSS200
cg12829142	7	23,529,999	0.000139	0.217	0.178				*RPS2P32*	TSS200
cg02155398	2	45,160,490	6.27E-06	0.217	0.218					
cg20805367	17	6,918,952	6.79E-07	0.217	0.197				*C17orf49*	Body
cg00258597	8	23,567,607	6.62E-06	0.217	0.180					
cg04380519	17	61,778,366	1.63E-05	0.217	0.217				*LIMD2*	TSS1500
cg09267467	1	178,914,986	1.17E-05	0.217	0.205					
cg04651137	7	23,287,222	0.00093	0.217	0.218				*GPNMB*	Body
cg08422803	21	46,341,067	3.81E-08	0.217	0.217				*ITGB2*	TSS200
cg09357761	14	61,933,059	1.44E-13	0.217	0.214				*PRKCH*	Body

cg09924848	10	126,308,486	8.67E-11	0.217	0.206				*FAM53B*	3'UTR
cg04434896	8	23,562,003	6.92E-05	0.217	0.197				*NKX2-6*	Body
cg12076463	4	85,418,265	6.48E-10	0.217	0.218				*NKX6-1*	Body
cg13078671	12	6,662,193	3.3E-14	0.217	0.221				*IFFO1*	Body
cg16025118	12	114,273,678	2.19E-06	0.217	0.221				*RBM19*	Body
cg20219381	8	101,118,083	2.51E-06	0.217	0.222				*RGS22*	Body
cg21158087	17	58,216,415	0.005473	0.217	0.230					
cg07229001	15	70,897,224	0.015177	0.217	0.279					
cg07358855	8	99,960,843	4.38E-06	0.217	0.210				*OSR2*	5'UTR
cg20970369	1	111,744,108	1.03E-07	0.217	0.201				*DENND2D*	TSS1500
cg15422382	10	81,141,532	6.2E-14	0.217	0.193					
cg00019759	1	212,687,842	0.001399	0.217	0.235					
cg03714110	5	92,923,623	2.15E-05	0.217	0.185				*NR2F1*	Body
cg25464265	1	221,063,484	2.75E-05	0.217	0.187					
cg26813646	10	50,817,651	0.01685	0.217	0.258				*SLC18A3*	TSS1500
cg06563089	12	75,601,157	3.01E-05	0.217	0.242				*KCNC2*	Body
cg10571987	6	41,528,395	2.47E-06	0.217	0.209				*FOXP4*	5'UTR
cg11390073	12	29,302,515	0.003663	0.217	0.188					
cg21990700	12	7,260,776	0.004488	0.217	0.180				*LOC283314*	TSS200
cg07500325	21	47,062,264	0.001699	0.217	0.113					
cg10253847	6	28,227,085	4.69E-06	0.217	0.210				*NKAPL*	TSS200
cg20268039	7	25,900,167	0.001374	0.217	0.202					
cg26311734	4	94,755,520	7.21E-05	0.217	0.201					
cg08720517	5	138,729,919	0.002451	0.217	0.232				*LOC389333*	1stExon
cg13487918	3	38,067,350	0.000785	0.217	0.176				*PLCD1*	TSS1500
cg20884298	13	46,190,037	2.88E-05	0.217	0.242				*FAM194B*	TSS200
cg24361291	19	16,394,499	1.49E-05	0.217	0.206					
cg07740306	18	77,246,369	4.69E-12	0.217	0.215				*NFATC1*	Body
cg21201099	17	59,482,446	1.97E-05	0.217	0.226				*TBX2*	Body
cg22380508	11	63,381,144	0.018553	0.217	0.110				*PLA2G16*	Body
cg06410191	5	115,298,920	0.000477	0.217	0.244				*LVRN*	1stExon
cg13026370	18	8,367,098	0.000184	0.217	0.180				*LOC100192426*	TSS200
cg14632140	12	16,758,112	0.000338	0.217	0.166				*LMO3*	5'UTR
cg12243453	11	113,775,400	5.52E-10	0.217	0.206				*HTR3B*	TSS200

cg19611392	18	56,888,922	0.001062	0.217	0.267				*GRP*	Body
cg09807148	17	34,122,069	0.003525	0.217	0.174				*MMP28*	Body
cg02583525	2	26,624,612	0.041054	0.217	0.060				*C2orf39*	TSS200
cg07377994	17	55,533,142	5.45E-08	0.217	0.211			Yes	*MSI2*	Body
cg09533845	8	81,290,007	0.013525	0.217	0.143					
cg14826211	2	23,747,197	3.64E-10	0.217	0.202				*KLHL29*	5'UTR
cg03053788	8	65,498,340	0.00274	0.217	0.251			Yes		
cg12152002	14	57,279,496	0.002855	0.217	0.289				*OTX2OS1*	TSS1500
cg09335713	22	42,828,418	9.12E-15	0.217	0.201				*NFAM1*	TSS200
cg14205791	12	124,800,227	2.25E-14	0.217	0.207				*FAM101A*	3'UTR
cg04292993	7	73,894,730	0.038699	0.217	0.159				*GTF2IRD1*	5'UTR
cg20261167	4	88,896,942	7.95E-06	0.217	0.209	1.06E-12	0.218		*SPP1*	1stExon
cg03879594	16	85,207,569	1.5E-15	0.217	0.222					
cg06569139	8	101,117,949	5.42E-21	0.217	0.221				*RGS22*	Body
cg07105285	11	92,702,663	6.83E-10	0.217	0.200				*MTNR1B*	TSS200
cg10423771	10	50,604,499	0.018648	0.217	0.240					
cg19566658	7	100,466,241	4.34E-31	0.217	0.212				*TRIP6*	Body
cg19587237	1	54,797,076	0.008872	0.217	0.106				*SSBP3*	Body
cg01033642	16	81,564,192	0.00031	0.217	0.186				*CMIP*	Body
cg06733155	7	73,508,868	6.28E-06	0.217	0.206				*LIMK1*	Body
cg10534938	5	1,868,639	3.74E-05	0.217	0.215					
cg13736514	6	26,305,472	0.000224	0.217	0.223					
cg18554976	19	35,629,356	1.19E-07	0.217	0.181				*FXYD1*	TSS1500
cg19131647	17	27,397,005	0.001353	0.217	0.146					
cg07464674	17	6,358,363	1.16E-10	0.217	0.202				*PITPNM3*	3'UTR
cg12143499	12	27,615,389	0.006382	0.217	0.196					
cg03670393	4	81,128,690	1.06E-11	0.217	0.206					
cg04425920	10	50,604,518	0.03651	0.217	0.298					
cg03216043	19	10,928,639	8.6E-08	0.217	0.218				*DNM2*	Body
cg10722799	21	36,041,699	2.03E-05	0.217	0.210	3.48E-07	0.072		*CLIC6*	1stExon
cg13727629	1	2,232,469	3.39E-05	0.217	0.204				*SKI*	Body
cg14261840	3	62,360,417	0.034745	0.217	0.225				*FEZF2*	TSS1500
cg14447077	9	79,920,904	7.33E-09	0.217	0.205				*VPS13A*	Body
cg17430228	11	353,919	7.21E-11	0.217	0.205					

cg08550353	6	134,497,627	2.18E-11	0.217	0.233				*SGK1*	TSS1500
cg18173058	12	114,838,359	0.000148	0.217	0.259				*TBX5*	Body
cg05550420	11	6,668,214	2.11E-05	0.217	0.219				*DCHS1*	5'UTR
cg06993255	8	145,846,234	8.57E-06	0.217	0.200					
cg13644528	9	36,163,544	2.76E-08	0.217	0.218				*GLIPR2*	3'UTR
cg16691177	4	165,898,666	3.56E-06	0.217	0.210				*TRIM61*	5'UTR
cg20219329	8	1,994,478	0.026453	0.217	0.120				*MYOM2*	5'UTR
cg01301885	10	128,258,570	3.44E-07	0.217	0.202					
cg05945608	6	42,739,639	2.24E-06	0.217	0.246					
cg06226630	4	48,493,420	2.78E-05	0.217	0.218				*ZAR1*	Body
cg02165330	11	124,542,984	4.2E-17	0.217	0.217				*SIAE*	Body
cg05350268	6	73,330,112	0.006154	0.217	0.237				*KCNQ5*	TSS1500
cg27549878	19	38,886,554	1.8E-05	0.217	0.203				*SPRED3*	Body
cg00379648	12	106,979,451	0.023456	0.217	0.287				*RFX4*	Body
cg07704934	11	44,332,940	0.016118	0.217	0.268				*ALX4*	TSS1500
cg24310711	17	70,026,605	0.034086	0.217	0.297					
cg18925726	14	23,982,474	0.000822	0.217	0.207					
cg19365673	1	99,470,618	0.018567	0.217	0.279				*LPPR5*	TSS200
cg12571570	2	145,267,984	9.35E-08	0.217	0.186				*ZEB2*	Body
cg17388712	7	50,889,487	0.005829	0.217	0.187					
cg07249742	3	124,570,104	4.19E-13	0.217	0.239				*ITGB5*	Body
cg07731871	10	18,629,568	0.002696	0.217	0.168				*CACNB2*	Body
cg22344727	12	81,102,035	1.72E-09	0.217	0.196				*MYF6*	Body
cg05664581	3	181,428,242	1.4E-06	0.217	0.198				*SOX2OT*	Body
cg19351398	7	149,472,134	7.32E-11	0.217	0.201				*SSPO*	TSS1500
cg25371036	11	94,500,749	6.09E-10	0.217	0.185				*AMOTL1*	TSS1500
cg14851325	6	32,156,100	7.41E-23	0.217	0.217				*PBX2*	Body
cg20767912	3	158,486,217	0.005276	0.217	0.174					
cg11043815	12	103,358,817	4.61E-05	0.217	0.188					
cg12278467	5	87,971,573	0.011496	0.217	0.221				*LOC645323*	Body
cg18034295	22	42,475,135	4.61E-05	0.217	0.194				*C22orf32*	TSS1500
cg19944656	17	76,876,040	2.08E-06	0.217	0.210				*TIMP2*	Body
cg04146573	11	69,970,618	4.93E-07	0.217	0.205				*ANO1*	Body
cg05459609	16	4,689,950	7.12E-19	0.217	0.188				*MGRN1*	Body

cg27626620	6	167,171,727	0.002051	0.217	0.227				*RPS6KA2*	Body
cg08778851	17	29,888,936	0.000135	0.217	0.185					
cg11689625	4	154,386,689	0.029095	0.217	0.160				*KIAA0922*	TSS1500
cg03109101	10	119,305,095	8.9E-05	0.216	0.220				*EMX2*	Body
cg06531741	11	113,775,450	5.76E-17	0.216	0.201	9.81E-26	0.177		*HTR3B*	TSS200
cg19503963	17	2,085,005	3.78E-10	0.216	0.203				*SMG6*	Body
cg09834465	1	35,586,503	3.12E-05	0.216	0.217					
cg22105158	19	3,480,672	1.09E-05	0.216	0.210				*C19orf77*	TSS200
cg27193080	3	129,160,612	1.32E-11	0.216	0.238				*IFT122*	Body
cg10045881	1	111,770,291	6.61E-05	0.216	0.192	7.56E-14	0.157		*CHI3L2*	1stExon
cg12990116	6	34,433,392	0.007179	0.216	0.155				*PACSIN1*	TSS1500
cg15351214	14	71,277,192	1.45E-11	0.216	0.163				*MAP3K9*	TSS1500
cg04139449	6	45,631,407	1.33E-05	0.216	0.223					
cg08419873	13	27,296,010	0.017093	0.216	0.188					
cg16683060	13	99,974,673	1.44E-05	0.216	0.211				*UBAC2*	Body
cg11036041	4	41,365,307	0.01817	0.216	0.269				*LIMCH1*	Body
cg24827626	21	46,169,242	1.2E-14	0.216	0.190					
cg03276479	8	97,507,958	0.002736	0.216	0.184				*SDC2*	Body
cg05456713	19	2,513,356	2.52E-07	0.216	0.170				*GNG7*	3'UTR
cg10606834	17	59,532,197	5.8E-05	0.216	0.225					
cg13202751	5	1,446,443	2.47E-05	0.216	0.174	6.65E-26	0.204		*SLC6A3*	TSS1500
cg01271812	2	66,671,478	6.17E-05	0.216	0.208				*MEIS1*	Body
cg09368485	6	29,974,022	6.22E-07	0.216	0.204				*HLA-J*	Body
cg12369353	13	36,728,963	0.0014	0.216	0.188					
cg26143752	2	27,806,326	0.000698	0.216	0.219				*ZNF512*	Body
cg24901662	10	34,977,342	1.25E-11	0.216	0.185				*PARD3*	Body
cg27247697	2	87,018,054	0.000287	0.216	0.218				*CD8A*	5'UTR
cg07155422	1	12,114,402	1.91E-10	0.216	0.217					
cg11878182	8	102,217,722	2.53E-17	0.216	0.217				*ZNF706*	5'UTR
cg02551980	5	112,628,419	0.00033	0.216	0.236				*MCC*	Body
cg03377610	2	45,164,723	1.34E-05	0.216	0.210					
cg23400512	14	85,996,315	0.029219	0.216	0.242				*FLRT2*	TSS200
cg02577433	10	102,887,035	0.016375	0.216	0.257				*TLX1NB*	5'UTR
cg16797421	6	147,428,486	8.62E-05	0.216	0.269					

cg22241820	15	68,122,574	6.96E-06	0.216	0.209				*LBXCOR1*	Body
cg00721572	1	147,775,609	0.013184	0.216	0.267					
cg08204129	3	145,852,537	2.09E-14	0.216	0.194				*PLOD2*	Body
cg23352597	X	46,435,742	0.000317	0.216	0.189				*CHST7*	3'UTR
cg01581222	19	10,958,952	6.48E-08	0.216	0.192				*C19orf38*	TSS200
cg18735451	7	28,905,872	0.011208	0.216	0.139					
cg26505691	16	27,461,333	0.015938	0.216	0.191				*IL21R*	3'UTR
cg05471090	18	71,959,795	0.003456	0.216	0.152				*CYB5A*	TSS1500
cg13606889	11	121,335,457	4.38E-05	0.216	0.202				*SORL1*	Body
cg17159161	6	31,323,760	0.003572	0.216	0.142				*HLA-B*	Body
cg24159514	19	11,785,248	1.27E-10	0.216	0.207				*ZNF833*	Body
cg14871932	7	44,185,323	7.73E-09	0.216	0.218				*GCK*	Body
cg01575601	11	130,398,900	7.25E-06	0.216	0.221					
cg07111861	15	57,737,942	2.87E-14	0.216	0.197				*CGNL1*	Body
cg18180569	1	44,883,362	0.032892	0.216	0.212				*RNF220*	Body
cg18564989	1	226,411,057	0.012343	0.216	0.269				*MIXL1*	TSS1500
cg26751356	13	112,712,635	0.002454	0.216	0.253					
cg26825412	20	62,681,428	1.25E-13	0.216	0.206	9.45E-22	0.121		*SOX18*	TSS1500
cg23174616	5	145,713,698	0.000698	0.216	0.182					
cg10002668	7	107,665,708	1.42E-05	0.216	0.206				*LAMB4*	Body
cg11260097	7	121,940,647	0.000224	0.216	0.200					
cg17320136	5	10,567,905	6.02E-10	0.216	0.213				*ANKRD33B*	Body
cg22086566	7	3,989,097	2.57E-09	0.216	0.220				*SDK1*	Body
cg24161925	6	139,116,947	0.000607	0.216	0.227					
cg05360577	11	17,717,629	0.001727	0.216	0.200					
cg11537406	11	124,932,624	1.87E-12	0.216	0.201				*SLC37A2*	TSS1500
cg17298181	3	187,456,175	0.020429	0.216	0.271				*BCL6*	5'UTR
cg01970040	17	27,396,961	7.09E-12	0.216	0.206					
cg20755170	5	145,720,024	0.033226	0.216	0.089				*POU4F3*	3'UTR
cg12868067	12	128,752,246	0.038551	0.216	0.274			Yes	*TMEM132C*	Body
cg01874730	16	57,406,425	3.25E-12	0.216	0.215				*CX3CL1*	1stExon
cg13728308	14	34,532,891	1.75E-08	0.216	0.189					
cg04871131	7	94,954,202	5.06E-13	0.216	0.216				*PON1*	TSS1500
cg11839020	1	90,309,998	2.3E-10	0.216	0.211				*LRRC8D*	5'UTR

cg13334949	20	44,471,068	7.6E-07	0.216	0.199				*SNX21*	3'UTR
cg13748092	15	58,024,200	1.61E-16	0.216	0.212					
cg19453938	7	73,265,946	0.007494	0.216	0.255					
cg10044470	14	104,866,284	7.68E-06	0.216	0.163					
cg18433009	10	72,013,286	6.19E-12	0.216	0.199					
cg20319604	2	44,428,936	3.04E-09	0.216	0.207				*PPM1B*	Body
cg09063936	2	74,408,977	6.91E-05	0.216	0.210					
cg16158045	X	2,748,039	1.67E-08	0.216	0.217				*GYG2*	5'UTR
cg22445940	20	57,224,919	0.003271	0.216	0.196				*STX16*	TSS1500
cg22698028	5	400,494	0.009304	0.216	0.263				*AHRR*	Body
cg23662469	X	54,472,372	4.16E-06	0.216	0.219				*FGD1*	3'UTR
cg03625287	13	24,121,359	0.022726	0.216	0.191					
cg25583491	5	94,955,880	0.010503	0.216	0.146				*GPR150*	TSS200
cg02716792	16	81,480,551	0.01485	0.216	0.227				*CMIP*	Body
cg11139102	7	100,073,936	5.82E-06	0.216	0.230				*TSC22D4*	Body
cg25447894	22	41,957,443	1.78E-06	0.216	0.245				*CSDC2*	5'UTR
cg07797660	1	3,239,139	1.46E-13	0.216	0.178				*PRDM16*	Body
cg15812139	6	168,996,082	9.21E-08	0.216	0.211				*SMOC2*	Body
cg01285706	4	81,953,041	0.024364	0.216	0.161				*BMP3*	Body
cg16401270	1	32,404,469	0.010228	0.216	0.152				*PTP4A2*	TSS1500
cg21177426	15	37,386,586	0.001337	0.216	0.242				*MEIS2*	Body
cg26523005	3	42,947,267	0.001669	0.216	0.142	0.0000533	0.078		*ZNF662*	TSS1500
cg26926076	6	129,204,510	0.034558	0.216	0.234				*LAMA2*	Body
cg19862242	6	138,200,334	4.33E-10	0.216	0.225				*TNFAIP3*	Body
cg26034934	2	20,420,746	7.97E-06	0.216	0.168				*SDC1*	Body
cg09993814	20	36,154,222	2.02E-08	0.216	0.217				*BLCAP*	5'UTR
cg15110101	11	61,717,334	0.021328	0.216	0.206				*BEST1*	TSS200
cg08944961	12	111,537,195	8.21E-09	0.216	0.196				*CUX2*	Body
cg22604013	3	53,173,997	1.52E-05	0.216	0.202					
cg17147820	11	61,322,255	0.000344	0.216	0.228				*SYT7*	Body
cg25177692	X	129,193,893	3.17E-05	0.216	0.195					
cg13419390	2	131,512,651	0.003238	0.216	0.254				*FAM123C*	TSS1500
cg22305293	3	93,851,734	0.023654	0.216	0.179					
cg15782228	20	60,932,415	8.45E-09	0.216	0.219				*LAMA5*	Body

cg20293942	13	50,701,256	1.42E-06	0.216	0.193					
cg25090972	7	156,797,988	0.033556	0.216	0.243				*MNX1*	3'UTR
cg26069093	12	122,048,642	1.33E-05	0.216	0.218					
cg05896902	15	45,671,018	0.001248	0.216	0.184				*LOC145663*	Body
cg20794824	3	170,138,205	3.96E-07	0.216	0.189				*CLDN11*	Body
cg25741487	3	62,363,076	0.019623	0.216	0.205					
cg01074584	8	41,168,072	1.91E-05	0.216	0.195				*SFRP1*	TSS1500
cg09931783	12	123,469,669	1.44E-10	0.216	0.203				*PITPNM2*	3'UTR
cg19907796	2	71,134,694	0.000647	0.216	0.217				*VAX2*	Body
cg24834823	8	38,386,476	1.75E-12	0.216	0.205				*C8orf86*	TSS1500
cg14517133	6	42,664,420	6.46E-10	0.216	0.190				*PRPH2*	3'UTR
cg19907305	19	18,902,117	0.000788	0.216	0.172				*COMP*	TSS200
cg00935216	7	157,461,368	0.000995	0.216	0.156				*PTPRN2*	Body
cg09845489	7	157,667,853	6.88E-11	0.216	0.185				*PTPRN2*	Body
cg09866565	3	137,844,436	6.97E-07	0.216	0.228				*A4GNT*	Body
cg12009023	4	148,402,128	0.032503	0.216	0.175				*EDNRA*	Body
cg22782017	6	168,785,895	0.001075	0.216	0.185					
cg00123055	8	55,370,951	0.002235	0.216	0.276				*SOX17*	1stExon
cg09059267	2	220,223,082	1.17E-14	0.216	0.202					
cg15060599	3	13,974,467	0.015932	0.216	0.172					
cg16368146	6	29,943,425	6.68E-09	0.216	0.212				*HCG9*	Body
cg20864568	17	43,391,599	2.25E-06	0.216	0.188				*MAP3K14*	5'UTR
cg21808635	7	55,225,133	5.46E-19	0.216	0.197				*EGFR*	Body
cg06139749	7	135,480,516	0.00027	0.216	0.246					
cg23213887	5	127,872,767	7.82E-06	0.216	0.211				*FBN2*	Body
cg00666105	3	181,428,377	3.08E-06	0.216	0.199				*SOX2OT*	Body
cg12271981	13	88,326,752	0.017668	0.216	0.263				*SLITRK5*	5'UTR
cg18049638	6	28,733,695	0.04944	0.216	0.099					
cg27411712	12	30,976,016	0.000235	0.216	0.193					
cg08045599	Y	7,140,572	2.36E-08	0.216	0.227				*PRKY*	TSS1500
cg08423562	1	38,466,544	0.003007	0.216	0.260				*FHL3*	5'UTR
cg13931134	10	33,671,135	7.05E-07	0.216	0.218					
cg24880701	15	68,121,563	0.000265	0.216	0.180				*LBXCOR1*	Body
cg26512045	6	31,111,158	1.74E-16	0.216	0.213				*CCHCR1*	Body

cg27415425	8	95,084,766	2.85E-05	0.216	0.246					
cg01960748	1	24,522,592	6.23E-06	0.216	0.200					
cg08911048	5	139,168,029	1.17E-05	0.216	0.206			Yes		
cg11823496	8	23,553,798	8.53E-05	0.216	0.185					
cg13939055	1	244,094,869	1.4E-16	0.216	0.199					
cg18618429	3	122,043,865	4.89E-05	0.216	0.240				*CSTA*	TSS200
cg18694169	6	28,227,079	3.17E-06	0.216	0.208				*NKAPL*	TSS200
cg26372400	12	52,300,922	0.000888	0.216	0.219				*ACVRL1*	TSS1500
cg01864564	1	152,488,269	0.033966	0.216	0.197				*CRCT1*	3'UTR
cg13956167	2	23,792,747	9.8E-10	0.216	0.210				*KLHL29*	Body
cg01879039	1	52,456,656	3.31E-10	0.216	0.189				*RAB3B*	TSS1500
cg01989521	14	103,522,012	1.2E-12	0.216	0.198				*CDC42BPB*	Body
cg08164191	14	65,623,632	1.05E-07	0.216	0.205					
cg11700824	10	88,730,823	0.03138	0.216	0.196				*AGAP11*	5'UTR
cg01134643	10	5,406,948	0.015937	0.216	0.164				*UCN3*	TSS200
cg19778015	3	11,625,154	3.38E-17	0.216	0.214				*VGLL4*	Body
cg23991358	1	114,618,759	8.83E-07	0.216	0.176					
cg08949339	12	30,949,073	0.036628	0.216	0.141					
cg06573604	2	160,760,825	2.47E-05	0.216	0.212				*LY75*	Body
cg17544177	10	103,589,245	3.76E-12	0.216	0.193				*KCNIP2*	Body
cg08688659	11	35,249,965	2.05E-11	0.216	0.207				*CD44*	Body
cg01870826	7	5,111,875	0.045526	0.216	0.215	1.86E-09	0.183		*LOC389458*	Body
cg02139338	10	134,108,333	1.6E-08	0.216	0.223				*STK32C*	Body
cg04566826	19	34,289,872	2.83E-17	0.216	0.211				*KCTD15*	5'UTR
cg14644378	9	34,988,588	0.000972	0.216	0.236				*DNAJB5*	TSS1500
cg00698413	2	225,266,656	0.005081	0.216	0.228				*FAM124B*	5'UTR
cg21691166	18	43,355,681	3.11E-05	0.216	0.183					
cg01079860	2	219,828,633	0.010047	0.216	0.159					
cg03779078	7	45,554,419	0.02907	0.216	0.289					
cg07525682	11	63,729,080	0.000211	0.216	0.174					
cg14474777	9	95,889,381	2.49E-07	0.216	0.212				*NINJ1*	Body
cg15487620	1	160,084,833	7.33E-06	0.216	0.222				*ATP1A2*	TSS1500
cg26118943	5	53,816,563	5.95E-08	0.216	0.187				*SNX18*	1stExon
cg08114317	1	1,935,535	1.44E-05	0.216	0.199				*KIAA1751*	TSS1500

cg15665928	3	138,658,345	0.001113	0.216	0.273					
cg18555698	11	12,699,172	5.1E-11	0.216	0.214				*TEAD1*	5'UTR
cg19987356	17	40,823,930	9.1E-07	0.216	0.213				*PLEKHH3*	Body
cg07178969	11	59,323,439	0.007324	0.216	0.217					
cg08729178	15	93,633,249	0.000284	0.216	0.173				*RGMA*	TSS1500
cg25839439	1	19,764,480	1.47E-08	0.216	0.222				*CAPZB*	Body
cg12737497	18	56,530,420	3.07E-05	0.216	0.127				*ZNF532*	5'UTR
cg05377839	3	61,833,671	2.36E-08	0.216	0.227				*PTPRG*	Body
cg20353227	15	67,430,416	5.24E-13	0.216	0.205				*SMAD3*	Body
cg05389174	22	20,192,371	9.38E-13	0.216	0.228				*LOC150197*	TSS1500
cg13238479	13	36,049,673	0.013029	0.216	0.195				*MAB21L1*	1stExon
cg23136139	10	43,697,918	4.09E-26	0.216	0.214				*RASGEF1A*	Body
cg25313172	12	52,627,272	1.31E-05	0.216	0.206				*KRT7*	1stExon
cg04617948	10	129,535,138	0.000264	0.216	0.199				*FOXI2*	TSS1500
cg07053014	6	44,187,132	0.043077	0.216	0.143				*SLC29A1*	TSS200
cg26485937	5	140,802,432	0.000108	0.216	0.242				*PCDHGA4*	Body
cg04386144	6	43,970,475	0.046994	0.216	0.189				*C6orf223*	Body
cg03574306	16	85,470,662	0.001436	0.216	0.168					
cg07793808	12	122,019,006	0.022499	0.216	0.194				*KDM2B*	TSS200
cg09712756	6	125,276,550	2.17E-15	0.216	0.205				*STL*	Body
cg27624471	18	77,280,171	0.000828	0.216	0.203				*NFATC1*	Body
cg00997251	5	142,444,988	1.02E-07	0.216	0.210				*ARHGAP26*	Body
cg02919422	8	55,370,544	0.000994	0.216	0.253	1.26E-26	0.229		*SOX17*	5'UTR
cg06600725	12	48,690,915	0.004977	0.216	0.229					
cg08196968	19	38,746,749	0.010002	0.216	0.180				*PPP1R14A*	Body
cg10289074	14	24,682,916	0.000413	0.216	0.146				*CHMP4A*	1stExon
cg12428326	20	39,807,896	0.000271	0.216	0.220				*ZHX3*	3'UTR
cg13702222	3	152,017,240	0.002047	0.216	0.186				*MBNL1*	1stExon
cg24378227	6	1,396,771	5.85E-06	0.216	0.191					
cg24823222	10	30,247,300	5.88E-21	0.216	0.225					
cg26036018	1	65,641,680	0.000169	0.216	0.257				*AK3L1*	Body
cg26389053	11	67,815,280	9.22E-09	0.216	0.218				*TCIRG1*	Body
cg01166145	4	55,093,692	0.000148	0.216	0.208					
cg02812767	15	74,218,468	4.74E-12	0.216	0.195				*LOXL1*	TSS1500

cg15113766	1	155,290,418	3.05E-05	0.216	0.167				*RUSC1*	TSS1500
cg13656878	9	119,048,807	4.08E-06	0.216	0.196				*PAPPA*	Body
cg08405119	4	53,728,999	2.11E-07	0.216	0.209				*RASL11B*	Body
cg08621611	16	10,277,254	0.016248	0.216	0.258				*GRIN2A*	TSS1500
cg13974394	5	1,882,188	2.63E-07	0.216	0.235				*IRX4*	Body
cg21863888	6	31,127,173	8.77E-05	0.216	0.225				*TCF19*	5'UTR
cg02488338	5	63,798,214	6.49E-05	0.216	0.197					
cg11205173	4	1,775,077	8.26E-10	0.216	0.219					
cg13367647	1	155,213,714	0.003722	0.216	0.211				*GBA*	5'UTR
cg01211283	8	22,735,276	0.029711	0.216	0.036				*PEBP4*	Body
cg05820959	7	151,248,135	4.84E-10	0.216	0.198					
cg13304350	17	43,084,426	0.005274	0.216	0.292					
cg00184376	13	28,499,045	0.000902	0.215	0.157				*PDX1*	3'UTR
cg00624418	5	1,282,357	0.045418	0.215	0.062				*TERT*	Body
cg07084163	5	36,066,783	0.000114	0.215	0.193	1.46E-16	0.175		*UGT3A2*	Body
cg11960377	11	3,167,827	8.54E-28	0.215	0.216				*OSBPL5*	5'UTR
cg25775322	4	154,710,373	0.018515	0.215	0.285				*SFRP2*	TSS200
cg07042014	14	24,783,620	0.002814	0.215	0.203				*LTB4R*	5'UTR
cg07483989	9	33,751,159	0.000694	0.215	0.206				*PRSS3*	Body
cg25849196	2	175,198,280	0.013346	0.215	0.278					
cg12145349	11	63,712,934	7.96E-17	0.215	0.212				*NAA40*	Body
cg12896318	5	173,395,358	2.02E-12	0.215	0.209			Yes		
cg14088142	5	138,923,113	0.000309	0.215	0.186					
cg00886387	13	44,729,868	8.5E-12	0.215	0.208					
cg12473916	1	154,943,651	5.6E-06	0.215	0.249				*SHC1*	5'UTR
cg16353350	10	75,683,980	3.85E-08	0.215	0.209				*C10orf55*	TSS1500
cg25732028	17	46,619,313	0.000225	0.215	0.197					
cg26385222	7	150,497,077	0.00094	0.215	0.144	9.43E-20	0.162		*TMEM176B*	TSS1500
cg24644902	10	77,036,275	0.044631	0.215	0.250					
cg04085447	19	35,773,328	0.001887	0.215	0.257				*HAMP*	TSS200
cg05021589	6	6,588,931	4.07E-06	0.215	0.191				*LY86*	TSS200
cg05291966	16	52,742,008	3.99E-06	0.215	0.184					
cg00960263	5	15,501,678	0.042662	0.215	0.137				*FBXL7*	Body
cg00931874	13	113,274,535	3.17E-05	0.215	0.210					

cg05720733	16	84,829,007	7.01E-11	0.215	0.190					
cg18147233	1	970,294	2.03E-11	0.215	0.201				*AGRN*	Body
cg21762589	10	133,796,476	0.000327	0.215	0.169	1.02E-18	0.182		*BNIP3*	TSS1500
cg25651608	3	42,073,868	0.02413	0.215	0.063					
cg00790395	10	94,822,172	0.021563	0.215	0.161				*CYP26C1*	Body
cg00883311	2	62,932,096	0.008488	0.215	0.076				*EHBP1*	TSS1500
cg23683800	10	115,086,103	0.006148	0.215	0.210					
cg24727114	4	124,707,571	0.000171	0.215	0.179				*LOC285419*	Body
cg04127044	10	47,083,355	0.000519	0.215	0.094				*PPYR1*	TSS200
cg07135032	1	165,322,099	8.71E-06	0.215	0.179				*LMX1A*	Body
cg21708738	2	42,175,351	4.34E-05	0.215	0.244					
cg21916479	3	194,024,145	0.002381	0.215	0.207				*LOC100131551*	Body
cg23940612	2	218,898,788	0.000193	0.215	0.239					
cg26581714	4	52,943,247	0.003405	0.215	0.218				*SPATA18*	Body
cg12526091	12	58,245,042	4.33E-08	0.215	0.205					
cg19200515	6	45,609,499	0.000657	0.215	0.170					
cg23013473	17	77,881,799	1.21E-12	0.215	0.191					
cg15118606	1	14,954,053	0.007111	0.215	0.153				*KIAA1026*	Body
cg20976526	8	101,118,021	2.48E-06	0.215	0.221				*RGS22*	Body
cg22961747	17	28,973,565	0.000648	0.215	0.170					
cg02486145	2	38,334,178	4.15E-07	0.215	0.211					
cg02211519	11	2,407,267	2.87E-14	0.215	0.224				*CD81*	Body
cg13364230	1	35,258,779	0.000188	0.215	0.224				*GJA4*	5'UTR
cg17465423	12	54,784,180	5.68E-07	0.215	0.165				*ZNF385A*	Body
cg05349245	4	140,201,539	8.51E-09	0.215	0.213				*C4orf49*	TSS200
cg09093210	12	57,619,199	0.003363	0.215	0.157				*NXPH4*	Body
cg16680438	1	16,553,549	0.002229	0.215	0.211					
cg19065177	5	140,105,648	0.022155	0.215	0.193				*VTRNA1-3*	TSS200
cg23316253	10	8,076,277	0.001808	0.215	0.254					
cg23546474	2	223,163,033	2.29E-06	0.215	0.192				*PAX3*	Body
cg06111454	14	24,837,836	0.026162	0.215	0.274	1.08E-13	0.206		*NFATC4*	Body
cg18709306	2	106,431,541	1.98E-05	0.215	0.248				*NCK2*	5'UTR
cg21926782	3	49,459,909	1.55E-09	0.215	0.209				*AMT*	Body
cg22262757	15	86,632,956	4.7E-06	0.215	0.218					

cg22956023	2	96,675,729	0.001162	0.215	0.144				*LOC729234*	TSS1500
cg05527530	1	197,882,003	0.02914	0.215	0.254				*LHX9*	Body
cg27544190	21	33,785,434	0.000106	0.215	0.149	1.08E-26	0.138		*C21orf63*	Body
cg00615485	7	29,519,656	2.09E-07	0.215	0.211				*CHN2*	5'UTR
cg00965023	19	11,785,188	7.41E-06	0.215	0.162				*ZNF833*	Body
cg12157673	19	52,996,617	0.000953	0.215	0.189				*ZNF578*	5'UTR
cg19361800	2	208,014,177	5.83E-09	0.215	0.215				*KLF7*	Body
cg22805381	14	51,760,188	0.001279	0.215	0.238					
cg23875404	13	36,728,869	2.6E-07	0.215	0.175					
cg27050153	14	102,030,110	5.88E-05	0.215	0.189					
cg12335447	22	37,546,115	0.00011	0.215	0.177				*IL2RB*	TSS200
cg18877667	17	48,051,089	0.000155	0.215	0.185				*DLX4*	Body
cg02898293	20	25,061,762	4.77E-05	0.215	0.193				*VSX1*	Body
cg08078648	2	236,236,591	0.000565	0.215	0.209					
cg10270306	6	151,561,032	2.89E-07	0.215	0.210				*AKAP12*	TSS200
cg15822982	10	95,517,895	0.007146	0.215	0.226				*LGI1*	5'UTR
cg18229767	11	67,776,576	1.77E-07	0.215	0.232				*ALDH3B1*	TSS1500
cg14913644	19	11,530,065	0.003016	0.215	0.218				*RGL3*	TSS200
cg00849610	17	59,529,316	7.88E-05	0.215	0.206					
cg01767631	13	20,716,728	0.002899	0.215	0.189				*GJA3*	Body
cg14763104	11	2,406,384	3.06E-08	0.215	0.191				*CD81*	Body
cg04094225	6	24,930,971	4.99E-06	0.215	0.250					
cg18392644	1	1,951,466	0.000272	0.215	0.223				*GABRD*	Body
cg25636478	20	6,750,989	0.0008	0.215	0.158				*BMP2*	Body
cg00221382	16	54,322,308	2.24E-07	0.215	0.178					
cg08817983	4	3,391,423	2.3E-10	0.215	0.208				*RGS12*	Body
cg03703733	6	168,147,981	3.24E-23	0.215	0.203					
cg17884766	12	50,641,260	1.3E-05	0.215	0.252				*LIMA1*	Body
cg21686171	1	9,504,429	6.08E-13	0.215	0.199					
cg07402310	17	40,424,590	1.6E-07	0.215	0.232				*STAT5B*	5'UTR
cg13632655	7	158,266,097	1.54E-07	0.215	0.189				*PTPRN2*	Body
cg19119594	22	45,094,547	8.5E-08	0.215	0.198				*PRR5*	5'UTR
cg26678920	11	6,341,888	0.000705	0.215	0.205				*PRKCDBP*	TSS200
cg01627669	12	8,068,706	0.003696	0.215	0.148					

cg09435415	8	105,479,383	0.026648	0.215	0.193				*DPYS*	TSS200
cg18736431	2	240,253,264	1.49E-11	0.215	0.204				*HDAC4*	Body
cg08474859	4	85,414,016	0.000413	0.215	0.244					
cg16459349	11	6,341,717	0.00797	0.215	0.262				*PRKCDBP*	5'UTR
cg00094412	6	29,592,854	2.76E-07	0.215	0.223				*GABBR1*	Body
cg11345143	13	20,989,349	2.17E-13	0.215	0.195				*CRYL1*	Body
cg24100636	12	3,600,087	0.045004	0.215	0.197				*PRMT8*	TSS1500
cg03205238	2	128,440,053	8.71E-23	0.215	0.219				*LIMS2*	TSS1500
cg14129735	6	30,737,007	7.4E-05	0.215	0.205					
cg21275283	17	18,011,513	2.19E-06	0.215	0.239				*MYO15A*	TSS1500
cg13282837	14	96,180,648	0.01059	0.215	0.211	4.77E-13	0.158		*TCL1A*	TSS200
cg17738010	2	3,452,437	0.000551	0.215	0.196				*TTC15*	Body
cg26783160	8	140,871,183	3.74E-38	0.215	0.209				*TRAPPC9*	Body
cg05000748	21	36,399,146	2.48E-05	0.215	0.219				*RUNX1*	Body
cg20038996	4	41,883,107	0.004682	0.215	0.286					
cg21151432	2	25,142,229	0.03873	0.215	0.144				*ADCY3*	TSS200
cg02198895	13	110,759,424	1.04E-07	0.215	0.184					
cg17936572	3	35,706,114	0.004967	0.215	0.233				*ARPP-21*	5'UTR
cg22375853	17	58,928,702	1.56E-12	0.215	0.199				*BCAS3*	Body
cg25970832	18	28,621,651	0.031461	0.215	0.167				*DSC3*	Body
cg01441113	4	186,560,403	1.14E-05	0.215	0.186				*SORBS2*	Body
cg20440891	7	28,448,103	3.61E-09	0.215	0.171				*CREB5*	5'UTR
cg00590620	18	12,254,690	0.002906	0.215	0.225				*CIDEA*	Body
cg10640333	2	63,280,625	0.000118	0.215	0.209				*OTX1*	Body
cg04219544	17	38,860,042	0.005952	0.215	0.271				*KRT24*	TSS200
cg04939673	4	184,019,125	1E-05	0.215	0.199				*C4orf38*	Body
cg06995715	16	86,612,590	1.04E-05	0.215	0.167	2.15E-12	0.177		*FOXL1*	1stExon
cg19577671	19	42,782,524	1.16E-20	0.215	0.216					
cg21151355	1	35,246,607	0.000225	0.215	0.159				*GJB3*	TSS200
cg02260710	16	85,730,584	0.000266	0.215	0.221					
cg04843067	2	177,016,037	5.34E-07	0.215	0.180				*HOXD4*	TSS200
cg17178175	2	178,109,973	8.04E-06	0.215	0.214				*NFE2L2*	Body
cg17275781	14	103,740,085	0.003003	0.215	0.236					
cg22264436	17	41,836,423	1.67E-05	0.215	0.188	7.77E-20	0.178		*SOST*	TSS1500

cg25696974	6	33,396,405	1.09E-05	0.215	0.185				*SYNGAP1*	Body
cg06875552	2	177,320,218	1.7E-06	0.215	0.198					
cg10140454	17	17,626,019	2.77E-08	0.215	0.222				*RAI1*	5'UTR
cg02284014	6	41,674,099	0.009154	0.215	0.208				*TFEB*	5'UTR
cg22344841	2	20,421,656	0.001838	0.215	0.248				*SDC1*	Body
cg08570118	7	155,025,533	1.45E-06	0.215	0.180					
cg10406414	8	49,587,029	4.44E-10	0.215	0.202					
cg24938761	19	2,091,406	8E-10	0.215	0.199				*MOBKL2A*	5'UTR
cg02318567	16	31,548,908	0.026266	0.215	0.245					
cg02551743	2	66,673,428	1.23E-05	0.215	0.207				*MEIS1*	Body
cg06756919	14	37,135,898	0.002399	0.215	0.151				*PAX9*	Body
cg00871371	17	75,371,476	5.78E-05	0.215	0.188				*SEPT9*	5'UTR
cg03491584	19	17,958,961	0.00034	0.215	0.162				*JAK3*	TSS200
cg14832908	1	6,479,424	0.000409	0.215	0.134				*HES2*	Body
cg15798221	17	62,773,704	2.67E-10	0.215	0.198				*LOC146880*	Body
cg08527797	12	53,730,072	8.52E-06	0.215	0.214				*SP7*	TSS1500
cg13847070	5	137,071,523	0.042498	0.215	0.039	0.00026383	0.114		*KLHL3*	5'UTR
cg21475402	1	156,612,140	0.01261	0.215	0.173	0.00000414	0.114		*BCAN*	5'UTR
cg09332960	2	1,787,990	0.001532	0.215	0.187					
cg09595020	7	2,654,120	0.000309	0.215	0.173				*IQCE*	3'UTR
cg11149930	2	119,593,625	0.017	0.215	0.236					
cg02880444	17	29,860,851	1.93E-09	0.215	0.251				*RAB11FIP4*	3'UTR
cg16484162	11	8,133,258	2.03E-08	0.215	0.204				*RIC3*	Body
cg16528895	2	108,994,311	0.004385	0.215	0.120				*SULT1C4*	TSS200
cg18064256	19	45,905,621	2.01E-13	0.215	0.219				*PPP1R13L*	5'UTR
cg18470295	17	8,868,834	0.017146	0.215	0.067				*PIK3R5*	5'UTR
cg06697175	11	110,047,230	4.71E-05	0.215	0.272					
cg27310831	8	120,685,124	4.11E-09	0.215	0.202					
cg02299195	11	48,185,069	1.18E-40	0.215	0.216				*PTPRJ*	Body
cg00000321	8	41,167,802	1.73E-05	0.215	0.172				*SFRP1*	TSS1500
cg15357156	1	23,884,373	6.92E-07	0.215	0.215					
cg24717935	3	27,756,281	0.025921	0.215	0.150					
cg26820636	2	237,083,222	2.05E-05	0.215	0.208					
cg00201133	10	35,740,216	1.25E-08	0.215	0.201				*CCNY*	Body

cg01782097	1	846,637	1.57E-09	0.215	0.218					
cg18792381	5	179,781,547	1.19E-10	0.215	0.204				*GFPT2*	TSS1500
cg03707308	6	6,002,766	0.031597	0.215	0.274				*NRN1*	Body
cg06493386	8	72,987,797	0.000559	0.215	0.225	7E-25	0.195		*TRPA1*	1stExon
cg07598052	20	35,274,639	2.75E-06	0.215	0.222				*SLA2*	TSS200
cg11547613	8	38,308,427	3.07E-09	0.215	0.218				*FGFR1*	Body
cg23164938	6	152,128,366	0.028919	0.215	0.094				*ESR1*	TSS1500
cg21411812	8	67,875,646	1.28E-05	0.215	0.228					
cg15353061	14	51,562,486	0.001656	0.215	0.114				*TRIM9*	TSS200
cg01853949	16	66,537,403	2.02E-14	0.215	0.208					
cg16458153	6	33,160,192	1.41E-15	0.215	0.187				*COL11A2*	5'UTR
cg17159058	8	1,704,075	0.002406	0.215	0.185					
cg02304233	1	147,070,522	0.002866	0.215	0.140				*BCL9*	5'UTR
cg17447502	1	6,478,857	7.98E-05	0.215	0.169				*HES2*	3'UTR
cg01456368	3	52,351,859	0.00728	0.215	0.201				*DNAH1*	5'UTR
cg13696609	9	100,617,710	0.000564	0.215	0.193				*FOXE1*	3'UTR
cg15883603	4	186,622,408	1.78E-15	0.215	0.212				*SORBS2*	5'UTR
cg09703136	4	173,995,054	3.5E-08	0.215	0.213					
cg20719980	2	36,616,441	0.001898	0.215	0.164				*CRIM1*	Body
cg01701555	1	146,544,097	1.48E-05	0.215	0.204					
cg03469471	7	150,707,830	0.000305	0.215	0.232			Yes	*NOS3*	Body
cg11698035	17	73,105,356	2.54E-09	0.215	0.207				*ARMC7*	TSS1500
cg12782933	11	116,451,038	0.002009	0.215	0.204					
cg16498391	18	12,421,072	8.2E-10	0.215	0.189				*SLMO1*	Body
cg03020545	6	73,330,358	0.002715	0.215	0.246				*KCNQ5*	TSS1500
cg10208654	X	135,230,613	2.68E-05	0.215	0.216				*FHL1*	Body
cg24172675	14	105,830,248	1.14E-13	0.215	0.212				*PACS2*	Body
cg25170822	12	50,344,458	9.7E-19	0.215	0.203				*AQP2*	TSS200
cg04139223	5	528,962	0.03554	0.215	0.256					
cg21051580	7	4,998,415	4.04E-05	0.215	0.196				*MMD2*	Body
cg23037132	1	40,105,664	6.87E-05	0.215	0.220				*HEYL*	TSS1500
cg23281803	8	121,138,317	0.005266	0.215	0.236				*COL14A1*	5'UTR
cg12309360	5	177,412,636	3.07E-05	0.215	0.211					
cg16597406	17	8,130,173	0.001085	0.215	0.174				*C17orf68*	3'UTR

cg08681924	3	62,359,924	0.025832	0.215	0.198				*FEZF2*	TSS1500
cg25658884	6	88,054,637	0.000623	0.215	0.191				*C6orf163*	1stExon
cg12049550	1	65,035,261	1.3E-07	0.215	0.188				*CACHD1*	Body
cg22171829	7	95,225,520	0.033742	0.215	0.297	6.03E-09	0.163		*PDK4*	1stExon
cg24804144	15	101,203,595	6.02E-07	0.215	0.216					
cg25758690	17	21,414,701	0.001813	0.215	0.226					
cg04855216	5	118,609,467	6.78E-05	0.215	0.246				*TNFAIP8*	Body
cg05525649	6	32,156,269	3.04E-37	0.215	0.210				*PBX2*	Body
cg06925882	16	85,157,885	2.08E-18	0.215	0.206					
cg09624466	14	57,278,710	0.032947	0.215	0.285				*OTX2OS1*	TSS1500
cg13286614	7	96,655,501	3.88E-10	0.215	0.205				*DLX5*	TSS1500
cg15541170	2	238,224,257	0.000126	0.215	0.205					
cg19059839	6	33,085,577	7.27E-05	0.215	0.224				*HLA-DPB2*	Body
cg19251600	6	168,672,660	1.04E-11	0.215	0.213					
cg23622616	2	236,444,259	7.59E-06	0.215	0.198				*AGAP1*	Body
cg24185357	10	38,069,909	5.41E-05	0.215	0.212					
cg25037841	22	20,237,341	2.04E-06	0.215	0.207				*RTN4R*	Body
cg00768993	2	200,335,874	0.018647	0.215	0.178				*FLJ32063*	Body
cg10009497	7	1,095,416	1.45E-11	0.215	0.216				*C7orf50*	Body
cg15605858	15	29,395,932	0.000745	0.215	0.175				*APBA2*	Body
cg26431815	8	668,053	0.003388	0.215	0.194				*ERICH1*	Body
cg24199834	4	147,560,126	0.035965	0.215	0.195	2.29E-14	0.179		*POU4F2*	1stExon
cg00502254	1	12,201,600	4.43E-07	0.215	0.178				*TNFRSF8*	Body
cg22469388	17	48,912,860	4.42E-05	0.215	0.255				*WFIKKN2*	1stExon
cg02992546	18	14,748,439	0.011611	0.215	0.188				*ANKRD30B*	1stExon
cg04051565	2	171,211,176	2.34E-06	0.215	0.188				*MYO3B*	Body
cg27059389	8	10,855,370	0.001889	0.215	0.179				*XKR6*	Body
cg03387346	5	54,526,536	6.54E-05	0.215	0.218					
cg14679780	19	4,059,525	9.76E-11	0.215	0.232				*ZBTB7A*	5'UTR
cg04792330	20	21,001,266	0.022557	0.215	0.172					
cg07289335	5	158,937,447	4.27E-05	0.215	0.170					
cg13734833	11	68,779,817	5.53E-11	0.215	0.211				*MRGPRF*	5'UTR
cg06288788	16	65,158,052	0.003908	0.215	0.202					
cg17366225	4	11,398,983	1.4E-07	0.215	0.225					

cg02003272	13	50,702,719	1.57E-08	0.215	0.195					
cg19317715	17	40,996,578	1.79E-05	0.215	0.216	6.23E-15	0.179		*AOC2*	TSS200
cg11122255	10	24,995,583	0.000277	0.215	0.214				*ARHGAP21*	Body
cg00636508	16	70,624,430	1.35E-05	0.214	0.163					
cg20082877	18	19,926,966	0.000188	0.214	0.193					
cg18236079	17	13,505,590	0.035482	0.214	0.100	0.00208438	0.047		*HS3ST3A1*	TSS1500
cg12656424	2	18,923,186	4.4E-07	0.214	0.213					
cg19810715	17	55,483,844	0.000773	0.214	0.187				*MSI2*	Body
cg06284231	14	38,724,255	0.003533	0.214	0.264				*CLEC14A*	1stExon
cg27642554	6	146,755,622	0.028114	0.214	0.209				*GRM1*	3'UTR
cg13445593	6	30,228,076	0.003931	0.214	0.186				*HLA-L*	Body
cg02096793	4	683,148	3.43E-08	0.214	0.200				*MFSD7*	TSS200
cg05171197	2	240,230,850	2.11E-13	0.214	0.217				*HDAC4*	Body
cg09918751	15	100,517,450	2.65E-07	0.214	0.211				*ADAMTS17*	Body
cg18645081	1	221,051,758	4.38E-07	0.214	0.200				*HLX*	TSS1500
cg01357292	3	42,947,771	0.034234	0.214	0.145				*ZNF662*	5'UTR
cg20785336	13	28,064,505	3.07E-05	0.214	0.214					
cg10099827	16	85,551,514	5.65E-36	0.214	0.213					
cg23121457	15	80,543,983	0.001342	0.214	0.124					
cg14990808	6	28,493,651	4.81E-14	0.214	0.230				*GPX5*	TSS200
cg17278072	7	155,258,411	0.014117	0.214	0.273					
cg23894086	7	27,128,046	1.38E-06	0.214	0.222					
cg13858975	15	52,016,644	9.24E-07	0.214	0.220				*LYSMD2*	Body
cg16129132	11	3,182,119	2.7E-10	0.214	0.203				*OSBPL5*	5'UTR
cg20459495	2	159,779,958	0.005056	0.214	0.145					
cg12850036	6	114,177,329	0.013857	0.214	0.158				*MARCKS*	TSS1500
cg19951297	5	138,441,071	2.16E-11	0.214	0.190				*SIL1*	Body
cg23554497	12	103,359,836	0.020833	0.214	0.210					
cg09313931	6	149,803,444	8.1E-07	0.214	0.212				*ZC3H12D*	5'UTR
cg10180092	3	171,002,363	1.24E-05	0.214	0.207				*TNIK*	Body
cg18065195	2	202,817,042	0.0072	0.214	0.165					
cg00445405	2	63,281,844	1.34E-05	0.214	0.198				*OTX1*	Body
cg04914930	13	44,785,481	1.83E-09	0.214	0.222					
cg24039081	3	197,155,143	2.38E-05	0.214	0.191					

cg08480367	X	38,080,608	9.84E-06	0.214	0.212				*SRPX*	TSS1500
cg26159832	14	103,822,211	2.1E-06	0.214	0.162					
cg10540679	7	130,606,499	0.006182	0.214	0.218					
cg18515031	10	73,988,469	1.04E-11	0.214	0.237				*C10orf104*	Body
cg21082400	5	72,595,958	0.000305	0.214	0.203					
cg13591408	12	104,920,290	1.88E-06	0.214	0.193				*CHST11*	Body
cg24824178	5	141,263,222	0.03402	0.214	0.277					
cg08420415	22	38,477,802	0.002685	0.214	0.177				*SLC16A8*	Body
cg16597737	1	173,572,370	2.43E-12	0.214	0.197				*SLC9A11*	TSS200
cg14543998	20	3,220,719	0.000396	0.214	0.216					
cg01298678	9	126,772,537	0.004638	0.214	0.212				*LHX2*	TSS1500
cg04639241	8	129,031,355	2.15E-08	0.214	0.221				*PVT1*	Body
cg05732832	4	4,868,723	0.011522	0.214	0.240					
cg13284574	1	6,519,923	8.77E-05	0.214	0.210				*ESPN*	Body
cg25130385	9	135,646,027	2.54E-06	0.214	0.204				*C9orf98*	Body
cg13558774	4	1,196,025	4.95E-08	0.214	0.196				*LOC100130872*	Body
cg19815340	3	27,770,391	7.66E-06	0.214	0.158					
cg01541347	7	4,729,920	2.29E-11	0.214	0.200				*FOXK1*	Body
cg08433011	21	36,399,540	1.77E-05	0.214	0.224				*RUNX1*	Body
cg09128529	2	227,586,520	6.53E-11	0.214	0.197					
cg11298786	1	228,195,794	0.000468	0.214	0.222				*WNT3A*	Body
cg15856599	14	42,074,503	0.004961	0.214	0.269					
cg08655662	6	41,438,590	3.37E-05	0.214	0.211					
cg19821124	14	95,927,475	0.005754	0.214	0.196				*C14orf49*	Body
cg05355411	8	70,449,272	0.000266	0.214	0.209				*SULF1*	5'UTR
cg18642271	19	46,180,314	0.000844	0.214	0.172				*GIPR*	Body
cg01862363	17	8,928,028	5.33E-21	0.214	0.177				*NTN1*	Body
cg08466034	17	16,318,930	0.002944	0.214	0.242				*TRPV2*	1stExon
cg10933494	6	28,983,143	0.025325	0.214	0.135					
cg06145195	X	152,107,440	3.16E-07	0.214	0.194				*ZNF185*	Body
cg17587456	6	168,674,852	1.49E-07	0.214	0.193					
cg13584244	9	124,030,048	0.003647	0.214	0.084				*GSN*	TSS1500
cg27098470	22	42,828,411	2.02E-17	0.214	0.201				*NFAM1*	TSS200
cg25845597	6	27,841,122	0.038379	0.214	0.243				*HIST1H4L*	1stExon

cg06729034	15	51,339,830	1.64E-14	0.214	0.199					
cg27470066	17	59,485,779	4.69E-07	0.214	0.200				*TBX2*	Body
cg12226033	13	44,906,847	0.002608	0.214	0.237					
cg13272465	4	140,200,658	0.039161	0.214	0.219				*C4orf49*	Body
cg04132146	6	87,862,158	0.003949	0.214	0.150					
cg06691520	10	95,517,738	4.58E-05	0.214	0.212				*LGI1*	5'UTR
cg05942459	6	101,846,805	5.2E-07	0.214	0.198				*GRIK2*	TSS200
cg12057576	15	78,918,529	0.000148	0.214	0.214				*CHRNB4*	Body
cg22873661	14	88,788,597	0.044026	0.214	0.196				*KCNK10*	Body
cg22892607	10	8,102,583	0.01373	0.214	0.162				*GATA3*	Body
cg02571816	19	38,747,378	0.015584	0.214	0.237				*PPP1R14A*	TSS1500
cg12355681	13	99,404,153	0.001252	0.214	0.212				*SLC15A1*	Body
cg12857945	14	37,127,275	3.67E-06	0.214	0.217				*PAX9*	5'UTR
cg22482700	1	68,594,186	0.007153	0.214	0.257				*GPR177*	Body
cg16519587	6	26,614,649	0.002273	0.214	0.189					
cg15135762	2	66,805,086	0.041761	0.214	0.255					
cg11679455	10	8,100,761	7.03E-06	0.214	0.206				*GATA3*	Body
cg11445123	20	2,781,478	0.017534	0.214	0.155				*CPXM1*	TSS200
cg00073090	19	1,265,879	9.22E-09	0.214	0.206					
cg06487082	18	56,530,106	7.72E-06	0.214	0.134				*ZNF532*	1stExon
cg10997718	2	162,275,690	0.032014	0.214	0.217				*TBR1*	Body
cg16865965	2	85,811,662	1.86E-05	0.214	0.215				*VAMP5*	Body
cg24769879	19	46,503,907	0.002717	0.214	0.173				*CCDC61*	Body
cg00503298	1	47,263,713	0.000577	0.214	0.199				*CYP4B1*	TSS1500
cg01765545	5	81,045,418	0.004182	0.214	0.226				*SSBP2*	Body
cg00547425	6	31,829,960	7.5E-06	0.214	0.153				*NEU1*	Body
cg04094148	11	129,244,523	2.54E-06	0.214	0.221				*BARX2*	TSS1500
cg07260592	6	161,100,122	0.006523	0.214	0.215	1.08E-29	0.266			
cg11877773	14	71,660,200	4.98E-16	0.214	0.205					
cg13801381	12	4,384,022	0.017627	0.214	0.201	1.71E-18	0.152		*CCND2*	Body
cg00527825	15	74,218,418	4.59E-12	0.214	0.214				*LOXL1*	TSS1500
cg00765737	13	110,993,493	1.48E-05	0.214	0.208				*COL4A2*	Body
cg01391084	2	149,645,372	7.84E-05	0.214	0.247				*KIF5C*	Body
cg22120063	5	72,596,188	0.002894	0.214	0.243					

cg01530605	3	181,414,848	0.001585	0.214	0.250				*SOX2OT*	Body
cg03064067	12	85,306,916	0.017632	0.214	0.151	1.5E-19	0.164		*SLC6A15*	TSS1500
cg09868780	5	115,299,071	0.007218	0.214	0.249				*LVRN*	Body
cg12684209	6	393,110	0.018668	0.214	0.141				*IRF4*	5'UTR
cg16206090	18	20,916,335	4.72E-09	0.214	0.212				*C18orf45*	Body
cg00008488	5	175,199,915	0.018939	0.214	0.226					
cg12407867	4	1,167,369	2.76E-08	0.214	0.200				*LOC100130872-SPON2*	Body
cg15916160	16	54,972,094	0.022933	0.214	0.219					
cg04621521	7	99,332,765	3.51E-10	0.214	0.181				*CYP3A7*	5'UTR
cg10364630	19	36,132,567	0.004073	0.214	0.262				*ETV2*	TSS200
cg11058366	2	213,401,195	0.027512	0.214	0.099				*ERBB4*	Body
cg12810212	2	232,379,626	0.004591	0.214	0.109				*C2orf52*	TSS1500
cg22614327	1	17,750,198	4.05E-14	0.214	0.210				*RCC2*	Body
cg02792602	2	218,808,859	2.12E-15	0.214	0.212				*TNS1*	TSS200
cg11636504	2	99,439,883	1.38E-10	0.214	0.192				*C2orf55*	Body
cg24820936	8	101,270,745	0.000305	0.214	0.215				*RNF19A*	3'UTR
cg27179680	1	205,460,879	2.73E-08	0.214	0.209					
cg21469772	9	124,989,294	3.54E-05	0.214	0.208				*LHX6*	Body
cg01351032	16	10,971,191	3.54E-08	0.214	0.202	5.5E-30	0.166		*CIITA*	1stExon
cg03665078	5	118,689,961	1.19E-10	0.214	0.196				*TNFAIP8*	Body
cg08341874	17	4,689,655	0.001444	0.214	0.165	0.01485097	0.054		*VMO1*	1stExon
cg16957940	3	124,792,406	7.85E-13	0.214	0.187					
cg02903822	8	134,203,379	0.000189	0.214	0.270				*WISP1*	5'UTR
cg13826452	6	26,758,395	3.54E-05	0.214	0.224					
cg15765546	3	133,776,357	7E-08	0.214	0.211					
cg14009075	22	43,807,401	1.59E-06	0.214	0.189				*MPPED1*	TSS1500
cg26060817	5	14,406,676	7.33E-10	0.214	0.192				*TRIO*	Body
cg02819256	X	152,938,698	0.001558	0.214	0.236				*PNCK*	Body
cg04196298	17	1,480,710	0.014793	0.214	0.334				*SLC43A2*	Body
cg25590181	6	29,855,467	6.79E-07	0.214	0.226				*HLA-H*	Body
cg05789711	16	23,724,428	0.000107	0.214	0.251				*ERN2*	1stExon
cg11729679	2	111,877,065	4.99E-05	0.214	0.198				*BCL2L11*	TSS1500
cg14505585	22	17,082,770	0.047844	0.214	0.264				*psiTPTE22*	TSS200
cg00988247	3	169,378,758	3.25E-06	0.214	0.194				*MECOM*	Body

cg08548284	1	14,382,172	0.000982	0.214	0.228					
cg16245261	11	6,342,357	0.00161	0.214	0.197				*PRKCDBP*	TSS1500
cg19172487	22	44,394,856	5.37E-10	0.214	0.234				*PARVB*	TSS1500
cg00211625	3	169,554,913	4.57E-20	0.214	0.219				*LRRIQ4*	Body
cg03404742	16	58,314,575	0.000147	0.214	0.210				*KLKBL4*	Body
cg12728020	1	114,698,322	0.012529	0.214	0.230					
cg16399632	4	1,244,006	0.030015	0.214	0.123				*CTBP1*	TSS1500
cg02494945	17	59,535,723	3.38E-09	0.214	0.203				*TBX4*	Body
cg04102163	4	41,881,185	8.22E-06	0.214	0.199					
cg22532194	18	77,231,654	8.4E-05	0.214	0.240				*NFATC1*	Body
cg25884488	4	153,601,388	0.026342	0.214	0.195				*TMEM154*	TSS200
cg01293179	2	176,996,285	0.000755	0.214	0.172				*HOXD8*	Body
cg04439458	5	138,467,593	6.4E-10	0.214	0.184				*SIL1*	5'UTR
cg07047068	11	844,686	0.001754	0.214	0.147				*TSPAN4*	5'UTR
cg09804503	1	33,231,313	7.07E-20	0.214	0.197				*KIAA1522*	Body
cg12924654	7	138,348,384	5.72E-11	0.214	0.166				*SVOPL*	Body
cg03479715	14	62,584,513	0.032235	0.214	0.232				*FLJ43390*	Body
cg23696593	10	28,200,623	1.8E-09	0.214	0.213				*ARMC4*	Body
cg06254440	9	126,779,285	0.022169	0.214	0.222				*LHX2*	Body
cg09045262	1	19,600,817	2.36E-05	0.214	0.178				*AKR7L*	TSS1500
cg18449997	17	40,574,243	0.019654	0.214	0.293				*PTRF*	Body
cg10521767	1	237,116,052	0.024387	0.214	0.092					
cg13024368	11	12,030,088	0.003273	0.214	0.296				*DKK3*	1stExon
cg07680167	11	17,717,574	0.041968	0.214	0.253					
cg18085407	1	244,080,114	1.2E-14	0.214	0.195					
cg23359714	8	41,168,336	0.001235	0.214	0.196				*SFRP1*	TSS1500
cg09462240	4	1,409,497	0.001673	0.214	0.192					
cg16099023	6	58,148,810	3.32E-05	0.214	0.194					
cg20096204	3	169,378,675	7.1E-05	0.214	0.188				*MECOM*	Body
cg17711717	9	34,590,391	0.005664	0.214	0.175				*CNTFR*	TSS1500
cg00308159	22	28,198,591	6.33E-08	0.214	0.218				*MN1*	TSS1500
cg15975890	5	11,903,145	0.039119	0.214	0.156				*CTNND2*	Body
cg19692710	11	73,661,920	1.5E-05	0.214	0.231	1.77E-21	0.156		*DNAJB13*	1stExon
cg22480783	2	25,149,622	3.9E-05	0.214	0.190					

cg27314116	5	6,583,850	9.94E-08	0.214	0.198				*LOC255167*	Body
cg04242499	5	140,749,745	0.002943	0.214	0.227				*PCDHGA4*	Body
cg26047334	2	218,785,909	3.28E-06	0.214	0.230				*TNS1*	5'UTR
cg26537209	16	54,324,187	2.69E-05	0.214	0.160					
cg17328839	1	64,127,751	1.68E-05	0.214	0.193					
cg06456154	10	119,310,356	0.027909	0.214	0.273					
cg16309595	12	103,359,445	1.16E-05	0.214	0.196					
cg16311536	10	105,344,174	0.000798	0.214	0.211				*NEURL*	Body
cg20207470	17	37,366,501	4.93E-08	0.213	0.199					
cg25161512	7	95,026,066	0.000561	0.213	0.182				*PON3*	TSS1500
cg00610844	5	6,583,797	2.63E-05	0.213	0.193				*LOC255167*	Body
cg01587682	11	31,820,572	0.020624	0.213	0.222				*PAX6*	Body
cg03099208	7	8,474,544	0.001564	0.213	0.147				*NXPH1*	5'UTR
cg03310838	16	88,545,042	3.97E-09	0.213	0.208				*ZFPM1*	Body
cg06151464	2	179,278,855	0.042627	0.213	0.074				*MIR548N*	Body
cg19725553	7	43,797,898	0.025368	0.213	0.185				*BLVRA*	TSS1500
cg02888518	10	75,716,672	1.33E-06	0.213	0.181					
cg06183559	13	95,357,818	0.003347	0.213	0.220					
cg26502018	2	181,770,937	1.5E-12	0.213	0.189					
cg05071823	X	117,628,671	3.63E-09	0.213	0.207				*DOCK11*	TSS1500
cg10824810	16	54,967,714	0.021977	0.213	0.257				*IRX5*	Body
cg04370174	4	8,207,250	1.07E-10	0.213	0.208				*SH3TC1*	Body
cg21917728	6	41,471,064	1.02E-10	0.213	0.201					
cg11079129	5	172,669,916	0.019369	0.213	0.264					
cg17484733	2	85,362,030	0.00111	0.213	0.222				*TCF7L1*	Body
cg07237653	8	28,950,303	1.78E-17	0.213	0.183				*KIF13B*	Body
cg14783814	1	98,511,792	0.006949	0.213	0.248				*MIR137*	TSS200
cg19095568	15	41,062,113	1.84E-09	0.213	0.232				*DNAJC17*	Body
cg20493526	2	3,714,936	3.03E-05	0.213	0.216				*ALLC*	5'UTR
cg23907108	11	64,405,993	0.005465	0.213	0.126				*NRXN2*	Body
cg00787180	14	91,751,731	2.74E-05	0.213	0.196				*CCDC88C*	Body
cg04145287	1	26,552,102	0.004218	0.213	0.250					
cg12166018	15	72,564,196	0.013877	0.213	0.179				*PARP6*	TSS1500
cg25575628	10	7,709,639	0.001474	0.213	0.227				*ITIH5*	TSS1500

cg10631947	5	54,281,668	0.000351	0.213	0.228				*ESM1*	TSS1500
cg22473097	6	29,818,300	0.003097	0.213	0.189					
cg08801887	11	67,811,194	5.32E-11	0.213	0.226				*TCIRG1*	Body
cg11990630	13	100,641,978	8.82E-05	0.213	0.186					
cg23423191	5	171,524,525	7.02E-09	0.213	0.187				*STK10*	Body
cg10805039	17	750,241	0.017068	0.213	0.158				*NXN*	Body
cg25782375	6	108,442,906	0.000196	0.213	0.219					
cg12944183	14	104,073,931	0.008178	0.213	0.169					
cg00060374	1	1,355,235	1.4E-07	0.213	0.180				*LOC441869*	Body
cg01450807	13	73,715,706	1.27E-06	0.213	0.218					
cg14332524	14	65,770,225	2.08E-09	0.213	0.204			Yes		
cg05049448	7	2,321,355	2.12E-10	0.213	0.226				*SNX8*	Body
cg15590950	1	44,874,649	0.007532	0.213	0.256				*RNF220*	5'UTR
cg20402826	2	198,077,081	9.5E-13	0.213	0.202					
cg06693983	19	55,889,216	4.26E-05	0.213	0.195				*TMEM190*	Body
cg11481687	14	105,499,998	0.020543	0.213	0.258					
cg13476200	9	124,578,224	8.11E-11	0.213	0.203					
cg13967702	1	217,312,060	7.38E-11	0.213	0.198				*ESRRG*	TSS1500
cg24881289	17	40,822,424	7.04E-11	0.213	0.202				*PLEKHH3*	Body
cg01312762	22	42,222,192	7.27E-14	0.213	0.189				*CCDC134*	3'UTR
cg23654206	19	3,683,811	1.63E-05	0.213	0.168				*PIP5K1C*	Body
cg01109169	2	71,134,630	0.004814	0.213	0.201				*VAX2*	Body
cg11278311	17	4,544,507	7.33E-06	0.213	0.202				*ALOX15*	Body
cg23490300	2	41,461,406	4.47E-09	0.213	0.203					
cg04351088	2	225,183,651	0.018583	0.213	0.146					
cg14376836	9	94,606,638	2.25E-09	0.213	0.197				*ROR2*	Body
cg24427243	1	12,128,100	2.73E-06	0.213	0.197				*TNFRSF8*	Body
cg02469838	16	54,404,655	0.000305	0.213	0.198					
cg14046302	2	67,939,758	4.67E-07	0.213	0.188					
cg03014634	16	85,205,030	3.88E-11	0.213	0.226					
cg01569082	11	61,370,871	3.38E-13	0.213	0.193					
cg03693099	9	135,936,901	4.27E-07	0.213	0.204				*CEL*	TSS1500
cg10936879	10	74,397,277	0.000521	0.213	0.207					
cg07840446	4	169,690,674	3.37E-08	0.213	0.212				*PALLD*	Body

cg21205978	22	36,044,151	0.000768	0.213	0.155				*APOL6*	TSS1500
cg27287254	6	33,160,067	1.2E-05	0.213	0.183				*COL11A2*	5'UTR
cg00548824	2	3,452,407	0.000362	0.213	0.200				*TTC15*	Body
cg10143960	1	1,109,486	1.28E-06	0.213	0.200				*TTLL10*	5'UTR
cg12492496	19	18,271,859	3.42E-07	0.213	0.154				*PIK3R2*	Body
cg14432269	8	67,873,343	0.000124	0.213	0.250					
cg27428414	10	99,343,942	0.000214	0.213	0.256				*DHDPSL*	TSS200
cg04010915	12	110,547,053	0.010319	0.213	0.275					
cg11220161	12	117,320,316	3.34E-06	0.213	0.170				*HRK*	TSS1500
cg14865868	2	12,856,040	0.041484	0.213	0.091	2.11E-16	0.151		*TRIB2*	TSS1500
cg24115032	20	57,089,953	2.71E-05	0.213	0.197				*APCDD1L*	TSS200
cg12820681	7	155,166,290	0.019123	0.213	0.210					
cg15171839	5	92,924,603	5.71E-06	0.213	0.185				*NR2F1*	Body
cg21959618	2	100,498,600	5.04E-05	0.213	0.202				*AFF3*	Body
cg06828335	10	79,238,225	4.31E-06	0.213	0.186				*KCNMA1*	Body
cg09557991	1	12,227,927	3.45E-05	0.213	0.210				*TNFRSF1B*	Body
cg18631330	17	79,899,941	6.18E-13	0.213	0.202				*MYADML2*	5'UTR
cg00859478	14	60,978,094	0.031955	0.213	0.234				*SIX6*	3'UTR
cg05726002	12	115,341,178	4.33E-12	0.213	0.187					
cg14253517	5	140,810,726	2.55E-05	0.213	0.221				*PCDHGA4*	Body
cg16197925	7	121,944,282	0.002604	0.213	0.189				*FEZF1*	1stExon
cg26351776	7	98,972,156	0.022487	0.213	0.119				*ARPC1B*	TSS200
cg00234625	5	177,811,648	4.24E-05	0.213	0.215				*COL23A1*	Body
cg02311864	6	47,215,645	2.38E-13	0.213	0.211				*TNFRSF21*	Body
cg24690094	11	67,383,802	0.000521	0.213	0.274					
cg04682133	1	55,681,841	8.19E-08	0.213	0.179				*USP24*	TSS1500
cg09571082	6	14,549,941	1.45E-09	0.213	0.187					
cg23894219	1	1,356,565	8.66E-09	0.213	0.201				*LOC441869*	1stExon
cg26216760	12	258,365	2.13E-08	0.213	0.192			Yes	*IQSEC3*	Body
cg27051315	2	238,864,959	0.001852	0.213	0.219					
cg27615603	16	31,213,045	3.07E-05	0.213	0.200				*PYCARD*	Body
cg00614617	17	8,921,074	0.032481	0.213	0.318					
cg06392733	16	85,215,643	2.05E-16	0.213	0.204					
cg09550809	5	78,407,562	1.96E-07	0.213	0.212				*BHMT*	TSS200

cg05484548	16	29,086,428	0.000201	0.213	0.200				*RRN3P2*	Body
cg01518246	11	63,870,912	3.52E-11	0.213	0.212				*FLRT1*	TSS1500
cg06166767	8	41,167,848	9.35E-09	0.213	0.169	0.0126121	0.035		*SFRP1*	TSS1500
cg06675144	5	127,552,620	1.44E-07	0.213	0.199					
cg14458815	22	23,484,001	4.25E-15	0.213	0.196				*RTDR1*	5'UTR
cg21446511	2	179,316,481	0.016121	0.213	0.235				*PRKRA*	TSS1500
cg02470690	6	27,839,548	0.043184	0.213	0.202					
cg09093931	8	98,880,926	0.002847	0.213	0.221				*MATN2*	TSS1500
cg15215155	7	128,473,311	2.24E-05	0.213	0.241				*FLNC*	Body
cg27190385	6	10,421,865	2.96E-05	0.213	0.215					
cg16039869	11	125,260,943	0.02728	0.213	0.225				*PKNOX2*	Body
cg17039369	21	46,341,006	4.86E-05	0.213	0.209				*ITGB2*	TSS200
cg03045635	4	9,783,198	0.008444	0.213	0.292				*DRD5*	TSS200
cg06508062	21	38,063,872	0.020442	0.213	0.250					
cg24318076	17	18,091,662	3.23E-07	0.213	0.207				*ALKBH5*	Body
cg02370877	8	76,316,876	1.29E-06	0.213	0.202					
cg03266686	2	47,716,097	2.17E-13	0.213	0.177					
cg09907170	2	111,877,177	1.07E-05	0.213	0.209				*BCL2L11*	TSS1500
cg10249734	17	80,292,079	2.02E-14	0.213	0.184	1.05E-15	0.146		*SECTM1*	TSS200
cg16394290	1	36,788,416	3.69E-06	0.213	0.209				*FAM176B*	Body
cg00013451	8	23,711,589	0.008741	0.213	0.112				*STC1*	Body
cg00183886	4	53,524,296	9.42E-10	0.213	0.207				*USP46*	Body
cg02659568	1	116,221,763	3.64E-09	0.213	0.206				*VANGL1*	Body
cg03785728	20	17,674,195	0.005833	0.213	0.220				*BANF2*	TSS200
cg19713947	1	200,005,635	0.002149	0.213	0.278				*NR5A2*	Body
cg25600933	12	118,031,023	0.0001	0.213	0.216				*KSR2*	Body
cg26978172	6	31,696,223	4.56E-07	0.213	0.230				*DDAH2*	Body
cg19223299	6	100,057,160	0.043495	0.213	0.310				*PRDM13*	Body
cg02433564	6	37,673,335	0.036289	0.213	0.231					
cg03553278	2	177,055,168	6.19E-07	0.213	0.179				*HOXD1*	3'UTR
cg12108798	1	1,173,916	6.91E-20	0.213	0.199					
cg15254238	10	70,321,874	3.47E-05	0.213	0.206				*TET1*	5'UTR
cg24026619	14	59,027,109	4.33E-06	0.213	0.214					
cg08415853	5	87,979,005	5.73E-05	0.213	0.248				*LOC645323*	Body

cg20663364	11	120,110,627	0.016183	0.213	0.229				*POU2F3*	TSS1500
cg00759329	1	82,208,723	1.72E-06	0.213	0.154					
cg01373911	1	6,269,367	7.53E-06	0.213	0.196				*RNF207*	Body
cg02780919	11	9,026,308	7.35E-09	0.213	0.209				*NRIP3*	TSS1500
cg11950754	1	53,782,077	1.34E-17	0.213	0.200				*LRP8*	Body
cg14504259	9	970,882	0.006691	0.213	0.221					
cg27316828	10	104,436,518	0.001071	0.213	0.220				*ARL3*	3'UTR
cg04694035	7	96,647,440	0.001992	0.213	0.240					
cg13596235	7	99,229,650	0.001828	0.213	0.237				*ZNF498*	3'UTR
cg14900246	2	112,655,153	2.77E-14	0.213	0.217				*MERTK*	TSS1500
cg01764559	11	8,337,383	0.001986	0.213	0.174					
cg10581650	10	100,155,032	2.78E-13	0.213	0.195				*MIR1287*	Body
cg15641162	4	185,942,800	0.002784	0.213	0.215					
cg00658151	1	31,368,797	1.21E-06	0.213	0.208				*SDC3*	Body
cg14950829	13	53,419,617	0.008707	0.213	0.165				*PCDH8*	Body
cg26566898	11	117,069,891	1.97E-15	0.213	0.217				*TAGLN*	TSS200
cg05389024	2	170,366,131	3.26E-06	0.213	0.215	1.84E-07	0.126		*KBTBD10*	TSS200
cg21069965	6	25,230,306	3.89E-11	0.213	0.233					
cg14841828	14	99,729,088	0.028282	0.213	0.208				*BCL11B*	Body
cg18857062	6	43,276,478	0.002337	0.213	0.178				*CRIP3*	Body
cg08562004	17	41,008,835	2.76E-12	0.213	0.205				*AOC3*	3'UTR
cg10943921	3	129,279,429	1.93E-11	0.213	0.210				*PLXND1*	Body
cg20309640	1	178,027,432	2.55E-05	0.213	0.219					
cg05155784	12	110,316,989	0.000589	0.213	0.182				*GLTP*	Body
cg06275853	11	20,617,937	0.011406	0.213	0.197					
cg21320768	13	50,070,766	1.21E-05	0.213	0.189				*PHF11*	Body
cg22224597	1	41,444,871	0.001513	0.213	0.238				*CTPS*	TSS200
cg02804819	19	49,238,364	3.82E-07	0.213	0.227				*RASIP1*	Body
cg07135660	5	133,343,843	9.26E-10	0.213	0.201					
cg09076337	14	81,421,983	0.000412	0.213	0.272				*TSHR*	5'UTR
cg13782388	3	126,614,750	2.82E-13	0.213	0.199				*CHCHD6*	Body
cg14989308	19	17,357,472	0.000103	0.213	0.192				*NR2F6*	TSS1500
cg15418509	11	71,900,411	1.52E-11	0.213	0.220				*FOLR1*	TSS1500
cg23234149	1	3,615,561	2.4E-07	0.213	0.204				*TP73*	Body

cg25588852	2	216,877,276	0.002217	0.213	0.184				*MREG*	Body
cg11462315	2	43,455,048	0.012239	0.213	0.103				*ZFP36L2*	TSS1500
cg05825053	16	11,697,522	0.00273	0.213	0.175					
cg04018164	17	12,566,358	9.04E-05	0.213	0.222					
cg07192961	16	672,468	0.009426	0.213	0.198				*RAB40C*	Body
cg11245181	6	149,772,854	6.39E-14	0.213	0.200				*ZC3H12D*	Body
cg15661389	17	10,018,631	0.039624	0.213	0.238				*GAS7*	Body
cg26870771	16	67,197,640	3.31E-05	0.213	0.237				*HSF4*	5'UTR
cg10548163	15	101,228,329	4.25E-09	0.213	0.224					
cg18043267	10	135,170,752	8.82E-06	0.213	0.197				*C10orf125*	Body
cg18106366	14	38,590,628	2.33E-06	0.213	0.228					
cg24409808	10	64,957,287	0.000381	0.213	0.185				*JMJD1C*	Body
cg27385729	11	118,619,582	2.26E-16	0.213	0.204				*DDX6*	3'UTR
cg00290086	9	1,050,903	1.08E-08	0.213	0.199				*DMRT2*	5'UTR
cg00026909	1	58,089,001	0.00032	0.213	0.156				*DAB1*	5'UTR
cg02077216	5	57,753,951	0.001252	0.213	0.239				*PLK2*	Body
cg15010554	6	122,253,935	0.012807	0.213	0.224					
cg16190266	14	38,680,855	0.001139	0.213	0.178				*SSTR1*	3'UTR
cg20732304	13	113,752,657	2.55E-10	0.213	0.173				*MCF2L*	3'UTR
cg22930986	3	59,171,831	5.09E-09	0.213	0.241					
cg12136274	1	146,649,770	0.001401	0.213	0.178				*PDIA3P*	Body
cg18785787	1	164,750,253	2.34E-07	0.213	0.212				*PBX1*	Body
cg08599355	13	99,714,609	1.01E-09	0.213	0.212				*DOCK9*	Body
cg11219917	4	819,151	0.004976	0.213	0.157				*CPLX1*	5'UTR
cg15768901	19	41,120,329	3.7E-07	0.213	0.175				*LTBP4*	Body
cg16879806	19	20,277,919	0.005768	0.213	0.110				*ZNF486*	TSS200
cg17833902	1	54,143,168	2.73E-18	0.213	0.200				*GLIS1*	5'UTR
cg20071670	11	73,661,895	0.00091	0.213	0.271				*DNAJB13*	1stExon
cg13698196	12	52,257,893	0.022919	0.213	0.259					
cg01323777	17	7,832,943	6.54E-07	0.213	0.193				*KCNAB3*	TSS200
cg13347970	6	137,809,630	0.001571	0.213	0.255					
cg25536787	17	26,221,167	0.002428	0.213	0.238				*C17orf108*	TSS1500
cg03345805	2	191,208,171	0.002801	0.213	0.130				*INPP1*	TSS200
cg05418333	7	101,556,183	1.41E-08	0.213	0.190				*CUX1*	Body

cg05647733	12	106,474,322	2.7E-05	0.213	0.156				*NUAK1*	Body
cg05870586	2	240,196,769	1.33E-06	0.213	0.194				*HDAC4*	Body
cg10521480	1	182,721,424	0.000643	0.213	0.209					
cg16632159	10	7,466,434	1.53E-11	0.213	0.198					
cg26666835	5	170,742,118	0.001238	0.213	0.239					
cg09252806	10	131,669,630	0.04185	0.213	0.040				*EBF3*	Body
cg05040232	3	194,117,403	2.4E-05	0.213	0.194				*GP5*	Body
cg03420242	16	49,893,122	0.007416	0.213	0.267					
cg04854285	X	102,531,822	0.000939	0.213	0.235				*TCEAL5*	TSS200
cg08071700	16	81,579,244	4.45E-07	0.213	0.196				*CMIP*	Body
cg25602692	12	123,998,085	8.18E-07	0.213	0.213				*RILPL1*	Body
cg03137522	6	117,087,083	0.001152	0.213	0.153				*FAM162B*	TSS200
cg11100804	5	140,783,173	6.56E-06	0.213	0.146				*PCDHGA4*	Body
cg16456252	6	28,483,537	0.000184	0.213	0.228				*GPX6*	1stExon
cg22500555	3	159,756,996	0.026282	0.213	0.201					
cg24292612	8	6,735,472	0.000107	0.213	0.166	4.47E-23	0.169		*DEFB1*	5'UTR
cg13982505	2	233,642,094	1.49E-06	0.213	0.218	0.00024306	0.100		*GIGYF2*	Body
cg20275132	6	28,367,749	0.01949	0.213	0.212				*ZSCAN12*	TSS1500
cg03281139	19	3,451,135	4.26E-05	0.213	0.239				*NFIC*	Body
cg15919258	14	90,849,921	0.004347	0.213	0.169					
cg24387380	15	27,159,158	3.54E-12	0.213	0.161				*GABRA5*	Body
cg01166925	4	41,880,280	6.02E-11	0.213	0.204					
cg04551776	5	393,366	3.45E-06	0.213	0.193				*AHRR*	Body
cg13818276	22	32,437,865	3.16E-11	0.213	0.191				*SLC5A1*	TSS1500
cg01473837	12	54,409,321	0.041968	0.213	0.204				*HOXC4*	TSS1500
cg02900766	15	58,357,319	0.000285	0.213	0.185				*ALDH1A2*	Body
cg05371578	3	147,128,157	0.001257	0.213	0.205				*ZIC1*	1stExon
cg06023941	5	53,187,565	3.32E-16	0.213	0.191				*ARL15*	Body
cg22970003	7	157,406,032	8.51E-07	0.213	0.198				*PTPRN2*	Body
cg00280609	1	45,250,840	3.54E-05	0.213	0.220				*BEST4*	Body
cg27455017	7	150,037,988	0.000616	0.213	0.147				*RARRES2*	5'UTR
cg05760722	12	52,115,238	0.010774	0.213	0.243				*SCN8A*	Body
cg05826295	6	113,886,340	0.000765	0.213	0.206					
cg13394491	5	15,737,401	1.41E-06	0.213	0.217				*FBXL7*	Body

cg07650392	3	64,127,184	0.004271	0.212	0.230			Yes	*PRICKLE2*	Body
cg09644153	16	3,345,646	1.17E-07	0.212	0.185					
cg15855170	11	116,574,279	0.020666	0.212	0.194					
cg16967003	7	1,293,599	5.11E-06	0.212	0.215					
cg12425861	14	105,830,606	0.023872	0.212	0.102				*PACS2*	Body
cg19385628	3	6,904,640	0.021352	0.212	0.071				*GRM7*	Body
cg00194277	7	30,795,751	8.69E-07	0.212	0.221				*INMT*	3'UTR
cg17402060	12	81,108,011	6.32E-13	0.212	0.214					
cg07871202	3	42,306,580	0.014462	0.212	0.235				*CCK*	TSS200
cg13998093	17	44,896,877	0.000267	0.212	0.221				*WNT3*	TSS1500
cg22798707	9	23,822,666	0.031629	0.212	0.152				*ELAVL2*	TSS1500
cg26980529	2	109,882,934	3.49E-07	0.212	0.231				*SH3RF3*	Body
cg07140894	16	5,020,147	0.002602	0.212	0.241				*SEC14L5*	Body
cg07283407	X	133,679,994	3.38E-05	0.212	0.188				*MGC16121*	Body
cg15514896	1	229,074,115	2.9E-06	0.212	0.222					
cg17362052	4	107,955,762	0.039813	0.212	0.066				*DKK2*	Body
cg19030579	1	50,888,710	0.031831	0.212	0.286				*DMRTA2*	5'UTR
cg25674938	19	1,360,981	0.000131	0.212	0.236				*MUM1*	Body
cg26328335	12	50,354,840	2.05E-07	0.212	0.192				*AQP5*	TSS1500
cg27391627	16	86,704,130	1.77E-07	0.212	0.197					
cg04971779	6	44,190,729	1.77E-08	0.212	0.183				*SLC29A1*	5'UTR
cg13456470	9	98,266,766	1.34E-09	0.212	0.238				*PTCH1*	5'UTR
cg21747160	12	49,504,981	0.001475	0.212	0.199				*LMBR1L*	TSS1500
cg01087977	2	220,150,857	4.4E-09	0.212	0.220				*DNAJB2*	3'UTR
cg06177306	2	133,927,261	1.09E-08	0.212	0.205				*NCKAP5*	Body
cg13235787	12	117,100,516	1.06E-05	0.212	0.145			Yes		
cg20163033	4	2,627,194	4.81E-16	0.212	0.201	3.15E-10	0.133		*FAM193A*	1stExon
cg24536349	8	143,781,340	1.2E-10	0.212	0.209				*LY6K*	TSS200
cg25372296	1	98,510,328	0.00358	0.212	0.169					
cg11691093	22	36,727,373	0.002444	0.212	0.275				*MYH9*	Body
cg17371334	11	134,341,419	1.54E-09	0.212	0.201					
cg02312904	17	59,234,870	0.000425	0.212	0.216				*BCAS3*	Body
cg09122387	10	98,855,762	1.29E-07	0.212	0.238				*SLIT1*	Body
cg05262549	1	183,256,588	9.4E-06	0.212	0.242				*NMNAT2*	Body

cg00017826	2	30,644,955	3.17E-09	0.212	0.217					
cg13160874	2	242,549,431	0.002159	0.212	0.206				*THAP4*	Body
cg26834680	1	111,814,208	0.004066	0.212	0.241					
cg05389555	1	6,659,504	0.000502	0.212	0.212				*KLHL21*	Body
cg05891054	17	40,380,254	7.17E-09	0.212	0.218				*STAT5B*	Body
cg17844718	6	32,280,834	9.52E-10	0.212	0.194				*C6orf10*	Body
cg03369269	13	25,745,946	0.037795	0.212	0.073				*FAM123A*	TSS200
cg16535018	17	36,829,606	0.004838	0.212	0.259				*C17orf96*	1stExon
cg21650962	6	50,794,057	0.012841	0.212	0.254				*TFAP2B*	Body
cg23362669	2	106,776,913	0.000243	0.212	0.219				*UXS1*	Body
cg12744820	6	137,814,960	0.003631	0.212	0.202				*OLIG3*	1stExon
cg06973667	10	71,332,139	6.75E-07	0.212	0.189				*NEUROG3*	3'UTR
cg09415955	1	10,709,885	5.29E-16	0.212	0.225				*CASZ1*	Body
cg13834844	10	102,415,320	0.047365	0.212	0.235					
cg03185144	16	85,106,490	4.51E-15	0.212	0.199				*KIAA0513*	Body
cg03301200	8	99,954,557	5.45E-09	0.212	0.202					
cg12964697	7	2,140,231	1.32E-11	0.212	0.206				*MAD1L1*	Body
cg21516044	4	166,304,596	2.66E-13	0.212	0.209				*CPE*	Body
cg21906379	X	109,936,239	0.00102	0.212	0.217				*CHRDL1*	Body
cg22976218	2	182,544,321	0.04006	0.212	0.249				*NEUROD1*	5'UTR
cg11817899	10	108,923,111	0.03948	0.212	0.157				*SORCS1*	Body
cg22779972	12	3,207,241	5.27E-09	0.212	0.194				*TSPAN9*	5'UTR
cg11891925	10	13,091,949	0.00074	0.212	0.230					
cg18685879	3	141,012,746	1.47E-05	0.212	0.243				*ACPL2*	3'UTR
cg20184238	17	43,046,859	1.04E-07	0.212	0.202				*C1QL1*	TSS1500
cg09430445	3	42,918,546	1.26E-05	0.212	0.215				*CYP8B1*	TSS1500
cg15440688	14	95,237,637	5.54E-06	0.212	0.228				*GSC*	TSS1500
cg20860726	2	1,820,169	3.26E-11	0.212	0.178				*MYT1L*	Body
cg00250430	9	1,051,579	0.002732	0.212	0.163	8.52E-14	0.148		*DMRT2*	5'UTR
cg02331649	3	52,926,322	3.14E-06	0.212	0.209				*TMEM110*	Body
cg08231493	8	17,271,093	0.010213	0.212	0.180			Yes	*MTMR7*	TSS200
cg21174841	9	100,617,999	0.017259	0.212	0.245				*FOXE1*	3'UTR
cg05205813	10	18,549,413	2E-10	0.212	0.199				*CACNB2*	Body
cg07707031	17	79,504,142	2.88E-05	0.212	0.236				*FSCN2*	3'UTR

cg09254322	10	101,280,807	5.84E-05	0.212	0.202					
cg21854895	17	71,305,638	1.64E-08	0.212	0.213				*CDC42EP4*	5'UTR
cg21501064	X	118,827,889	8.63E-07	0.212	0.182	7.59E-20	0.128		*SEPT6*	TSS1500
cg08573435	7	1,398,116	0.005882	0.212	0.237					
cg21768287	X	150,068,356	0.001062	0.212	0.115				*CD99L2*	TSS1500
cg09080114	12	6,983,111	0.019928	0.212	0.214				*SPSB2*	TSS1500
cg09013655	1	184,005,063	1.6E-06	0.212	0.158				*GLT25D2*	Body
cg10493506	2	230,340,591	0.000311	0.212	0.139				*DNER*	Body
cg03103919	10	18,629,674	0.010412	0.212	0.165				*CACNB2*	Body
cg27114644	2	121,344,729	2.95E-57	0.212	0.193					
cg09951047	9	97,848,306	2.72E-05	0.212	0.182				*C9orf3*	3'UTR
cg04944853	3	139,252,106	1.5E-10	0.212	0.204				*RBP1*	Body
cg19738463	6	31,831,574	0.037068	0.212	0.198				*NEU1*	TSS1500
cg26444282	5	149,980,674	7.38E-06	0.212	0.218				*SYNPO*	1stExon
cg09137301	7	150,026,998	6.06E-14	0.212	0.207				*C7orf29*	5'UTR
cg26351479	X	125,686,936	0.035338	0.212	0.147				*DCAF12L1*	TSS200
cg07397616	22	38,035,109	2.21E-16	0.212	0.201				*SH3BP1*	TSS1500
cg16153168	5	68,114,337	2.35E-10	0.212	0.209					
cg04026492	9	139,024,611	0.003871	0.212	0.203					
cg01230325	9	96,108,319	1.33E-07	0.212	0.208				*C9orf129*	5'UTR
cg05860111	5	14,441,261	0.000647	0.212	0.173				*TRIO*	Body
cg13408344	15	31,631,240	0.010195	0.212	0.096				*KLF13*	Body
cg01464985	2	27,805,622	0.000477	0.212	0.181	2.02E-22	0.150		*ZNF512*	TSS1500
cg03979024	10	419,189	7.05E-08	0.212	0.222				*DIP2C*	Body
cg04679902	22	19,752,869	4.93E-08	0.212	0.192				*TBX1*	Body
cg11511795	4	10,463,749	0.001759	0.212	0.202					
cg03302008	2	96,012,735	0.000392	0.212	0.190				*KCNIP3*	TSS200
cg27200895	4	7,763,703	4.85E-06	0.212	0.214				*AFAP1*	3'UTR
cg00400832	7	96,650,323	7.44E-06	0.212	0.213				*DLX5*	Body
cg01540102	16	57,506,038	5.85E-17	0.212	0.212				*DOK4*	3'UTR
cg08498156	6	30,095,802	0.001109	0.212	0.315					
cg09678539	2	27,298,343	2.34E-06	0.212	0.201					
cg12141659	11	2,847,462	6.37E-05	0.212	0.182				*KCNQ1*	Body
cg13009111	11	71,350,975	0.025868	0.212	0.106					

cg05045981	7	129,418,470	6.37E-05	0.212	0.157					
cg09830455	11	70,886,197	1.37E-11	0.212	0.202				*SHANK2*	5'UTR
cg14254080	2	85,106,954	0.000372	0.212	0.213				*C2orf89*	Body
cg18624900	10	91,295,643	0.018709	0.212	0.218				*SLC16A12*	TSS1500
cg05128520	16	84,676,746	0.006441	0.212	0.280					
cg09785033	11	2,336,066	1.11E-10	0.212	0.178				*TSPAN32*	Body
cg04963697	16	3,570,555	1.27E-11	0.212	0.214				*CLUAP1*	Body
cg09266602	8	49,469,775	4.41E-05	0.212	0.214					
cg19419246	19	45,950,425	3.89E-12	0.212	0.202					
cg11118422	6	28,557,883	7.49E-07	0.212	0.211					
cg17588373	X	13,957,417	5.08E-08	0.212	0.180				*GPM6B*	TSS1500
cg24207176	4	89,079,587	0.018689	0.212	0.148	2.15E-16	0.159		*ABCG2*	1stExon
cg00747944	16	54,972,845	0.000205	0.212	0.205					
cg12404831	2	192,114,017	0.000308	0.212	0.212				*MYO1B*	5'UTR
cg19853936	6	19,692,304	0.000266	0.212	0.231					
cg13375589	17	4,487,125	0.040729	0.212	0.218				*SMTNL2*	TSS200
cg21493727	8	127,938,624	8.99E-05	0.212	0.215					
cg00481644	15	89,921,158	2.85E-05	0.212	0.178				*LOC254559*	TSS200
cg02730865	16	80,972,238	9.59E-12	0.212	0.213					
cg06703222	16	69,598,327	2.18E-06	0.212	0.193				*NFAT5*	TSS1500
cg12727256	22	24,838,292	8.76E-06	0.212	0.209				*C22orf45*	Body
cg00539581	5	66,477,808	0.00104	0.212	0.211					
cg02338778	2	23,840,763	2.35E-05	0.212	0.229				*KLHL29*	Body
cg12559197	5	76,654,783	4.82E-06	0.212	0.206				*PDE8B*	Body
cg17563034	4	5,021,268	4.58E-05	0.212	0.195				*CYTL1*	TSS200
cg00609135	6	26,614,014	0.000844	0.212	0.197					
cg07693270	3	186,856,928	0.005117	0.212	0.132	7.8E-24	0.165		*RPL39L*	5'UTR
cg11789804	1	219,958,132	1.54E-05	0.212	0.221					
cg14314744	6	100,897,445	0.009896	0.212	0.204				*SIM1*	Body
cg16045823	7	73,295,154	6.01E-14	0.212	0.193					
cg16845136	22	46,774,204	2.94E-07	0.212	0.209				*CELSR1*	Body
cg18188653	3	188,296,976	1.2E-06	0.212	0.184				*LPP*	Body
cg08507952	3	193,750,969	0.002833	0.212	0.250					
cg11126767	14	60,978,275	6.43E-06	0.212	0.195				*SIX6*	3'UTR

cg17071957	9	124,029,926	3.45E-05	0.212	0.106	2.59E-08	0.102		*GSN*	TSS1500
cg24983862	17	65,242,497	0.014261	0.212	0.144				*HELZ*	TSS1500
cg25262528	6	86,158,972	0.016112	0.212	0.130				*NT5E*	TSS1500
cg26157386	14	36,994,270	0.031349	0.212	0.190					
cg05600126	17	950,540	2.06E-10	0.212	0.213				*ABR*	Body
cg07186576	10	31,072,771	1.93E-05	0.212	0.151					
cg10667895	2	102,577,876	1.58E-11	0.212	0.207					
cg11412418	5	172,658,730	0.032048	0.212	0.316					
cg03085719	11	3,860,569	4.15E-07	0.212	0.191				*RHOG*	5'UTR
cg06854772	4	1,853,351	1.87E-23	0.212	0.221				*LETM1*	Body
cg15323638	7	131,229,865	1.95E-08	0.212	0.207				*PODXL*	Body
cg02046552	8	21,914,287	7.43E-09	0.212	0.194				*EPB49*	TSS1500
cg02787917	2	175,190,821	0.007236	0.212	0.131					
cg10857774	19	46,286,683	8.66E-10	0.212	0.219	4.79E-35	0.125		*DMPK*	TSS1500
cg18172877	5	1,878,672	9.17E-08	0.212	0.209				*IRX4*	Body
cg01960096	1	157,012,966	8.31E-09	0.212	0.195				*ARHGEF11*	Body
cg03846767	1	3,607,292	3.44E-05	0.212	0.204	6.42E-17	0.204		*TP73*	1stExon
cg04557197	17	77,764,992	0.003606	0.212	0.187					
cg19088141	1	113,056,151	0.000413	0.212	0.199				*WNT2B*	Body
cg26843711	5	140,753,449	0.003532	0.212	0.231				*PCDHGA4*	Body
cg16254756	1	154,943,676	3.2E-05	0.212	0.228				*SHC1*	5'UTR
cg19255477	1	151,810,586	0.000121	0.212	0.198				*LOC100132111*	TSS1500
cg27297315	2	23,704,185	4.57E-08	0.212	0.202				*KLHL29*	5'UTR
cg01460202	16	23,706,506	0.000236	0.212	0.236				*ERN2*	Body
cg19118951	21	35,575,070	6.56E-14	0.212	0.200					
cg00350296	11	66,084,841	4.43E-05	0.212	0.206	2.79E-10	0.125		*CD248*	TSS1500
cg06247406	6	101,846,791	6.09E-06	0.212	0.185				*GRIK2*	TSS200
cg11067786	2	74,780,467	5.5E-16	0.212	0.224				*LOXL3*	5'UTR
cg14029759	6	5,764,816	1.76E-10	0.212	0.196				*FARS2*	Body
cg16792363	16	14,499,735	0.002929	0.212	0.228					
cg20313873	10	101,436,820	3.36E-07	0.212	0.184				*ENTPD7*	Body
cg23206461	1	147,782,116	0.000842	0.212	0.252					
cg24683185	15	31,214,501	4.66E-16	0.212	0.209				*MTMR15*	Body
cg01378515	3	124,773,106	5.09E-09	0.212	0.205				*HEG1*	Body

cg26585452	13	50,702,914	1.39E-06	0.212	0.199					
cg02193762	11	36,399,139	0.005985	0.212	0.150				*PRR5L*	5'UTR
cg02805871	17	6,939,695	0.004682	0.212	0.094				*SLC16A13*	5'UTR
cg02860240	3	169,530,621	0.00858	0.212	0.186				*LRRC34*	TSS1500
cg04043571	9	127,265,989	0.000106	0.212	0.210				*NR5A1*	5'UTR
cg04654530	2	63,282,702	2.99E-05	0.212	0.209				*OTX1*	Body
cg15905200	17	29,013,706	1.95E-09	0.212	0.196					
cg24593138	1	19,687,988	1.57E-06	0.212	0.192				*CAPZB*	Body
cg26794871	20	44,934,712	0.000278	0.212	0.187					
cg01025774	5	42,944,457	2.16E-05	0.212	0.180					
cg02251393	2	159,651,559	1.03E-08	0.212	0.228				*DAPL1*	TSS1500
cg11268739	14	48,145,588	0.017408	0.212	0.265					
cg03531247	2	50,573,485	0.003044	0.212	0.227				*NRXN1*	Body
cg11582717	17	63,452,460	3.24E-09	0.212	0.183					
cg00661523	3	151,985,426	6.41E-08	0.212	0.206				*MBNL1*	TSS1500
cg00735023	11	58,830,355	0.000281	0.212	0.187					
cg06342195	17	36,734,475	6.01E-06	0.212	0.195				*SRCIN1*	Body
cg10142520	11	65,344,604	2.65E-11	0.212	0.205				*EHBP1L1*	Body
cg14372324	20	30,347,798	0.001348	0.212	0.161			Yes	*TPX2*	Body
cg08725840	11	89,224,774	0.022992	0.212	0.228				*NOX4*	5'UTR
cg09250933	2	114,035,619	4.88E-05	0.212	0.205				*PAX8*	Body
cg12604331	1	156,906,485	6.12E-08	0.212	0.224				*ARHGEF11*	Body
cg15760474	6	41,554,529	9.7E-08	0.212	0.210				*FOXP4*	Body
cg14304817	11	89,867,911	0.0004	0.212	0.250				*NAALAD2*	5'UTR
cg19565738	2	174,083,367	1.97E-13	0.212	0.216			Yes	*ZAK*	Body
cg20733761	21	47,295,215	0.000229	0.212	0.160				*PCBP3*	Body
cg21064451	22	38,071,534	0.000191	0.212	0.238				*LGALS1*	TSS200
cg22942200	6	151,560,992	6.55E-06	0.212	0.197				*AKAP12*	TSS200
cg07943780	8	41,502,612	0.000487	0.211	0.120					
cg08323277	2	20,248,420	1.68E-07	0.211	0.212				*LAPTM4A*	Body
cg18363035	13	20,805,385	1.53E-06	0.211	0.193				*GJB6*	5'UTR
cg18773937	2	113,594,611	0.003602	0.211	0.244				*IL1B*	TSS1500
cg21429615	6	16,180,388	0.014791	0.211	0.133					
cg22272056	11	33,065,566	0.000265	0.211	0.140				*TCP11L1*	Body

cg09473745	10	31,423,550	3.56E-06	0.211	0.210					
cg23702610	15	100,017,287	7.17E-08	0.211	0.193					
cg06791102	X	153,191,600	0.032871	0.211	0.219				*ARHGAP4*	1stExon
cg08985885	12	53,719,903	6.39E-08	0.211	0.249					
cg01557547	3	157,823,089	0.049516	0.211	0.252				*SHOX2*	Body
cg14846380	12	125,333,322	2.35E-40	0.211	0.191				*SCARB1*	Body
cg20610181	9	35,674,000	1.69E-11	0.211	0.207	1.07E-29	0.165		*CA9*	1stExon
cg24715245	4	41,258,794	0.001362	0.211	0.219	3.8E-34	0.272		*UCHL1*	TSS200
cg12395205	18	12,254,469	0.042676	0.211	0.186				*CIDEA*	Body
cg01588826	19	36,352,459	0.000291	0.211	0.200				*KIRREL2*	Body
cg15167044	6	100,039,413	0.007445	0.211	0.228					
cg20402783	12	54,381,013	7.39E-16	0.211	0.188				*HOXC10*	Body
cg02288964	1	967,561	3.12E-07	0.211	0.193				*AGRN*	Body
cg04342110	19	38,945,908	0.002358	0.211	0.231				*RYR1*	Body
cg15473329	19	39,862,918	2.87E-06	0.211	0.197				*SAMD4B*	Body
cg01064918	14	92,788,960	0.028639	0.211	0.197				*SLC24A4*	1stExon
cg13225166	17	37,761,216	0.018567	0.211	0.247				*NEUROD2*	3'UTR
cg19981243	3	114,478,256	2.34E-08	0.211	0.213				*ZBTB20*	TSS200
cg21639114	6	130,687,884	0.006158	0.211	0.105					
cg08727316	10	24,545,715	4.21E-09	0.211	0.210				*KIAA1217*	Body
cg11083848	14	76,371,681	3.37E-10	0.211	0.219				*TTLL5*	Body
cg26786382	11	61,277,328	2.6E-07	0.211	0.160				*LRRC10B*	1stExon
cg02860342	15	73,661,484	0.000402	0.211	0.193	1.84E-30	0.186		*HCN4*	1stExon
cg08452338	3	149,271,990	2.54E-13	0.211	0.215				*WWTR1*	Body
cg00579036	6	25,138,551	0.047025	0.211	0.111				*CMAH*	TSS1500
cg00182273	16	66,876,847	3.14E-10	0.211	0.219				*CA7*	TSS1500
cg19343464	11	105,481,509	0.025553	0.211	0.215	2.09E-15	0.173		*GRIA4*	5'UTR
cg25907215	22	24,178,181	1.72E-05	0.211	0.222				*DERL3*	3'UTR
cg26485844	5	77,256,916	1.25E-08	0.211	0.205					
cg00934355	9	1,049,244	0.021567	0.211	0.191				*DMRT2*	TSS1500
cg22449337	1	40,943,913	0.001172	0.211	0.156				*ZNF642*	5'UTR
cg07924363	X	133,680,812	0.047775	0.211	0.192				*MGC16121*	TSS200
cg10457539	4	85,418,584	2.6E-05	0.211	0.231				*NKX6-1*	Body
cg13134650	1	203,156,764	2.17E-08	0.211	0.214				*CHI3L1*	TSS1500

cg02867514	17	34,207,341	9.92E-06	0.211	0.185				*CCL5*	1stExon
cg07815836	19	58,715,351	2.63E-08	0.211	0.226				*ZNF274*	Body
cg07324245	17	75,445,905	2.17E-07	0.211	0.141				*SEPT9*	Body
cg14542583	19	38,886,667	1.7E-07	0.211	0.189				*SPRED3*	Body
cg26824709	5	159,740,383	0.000799	0.211	0.114				*CCNJL*	TSS1500
cg03625415	5	172,662,741	3.04E-05	0.211	0.206				*NKX2-5*	TSS1500
cg05219621	X	15,683,403	2.49E-05	0.211	0.193				*TMEM27*	TSS1500
cg14015441	8	105,479,318	0.022853	0.211	0.206				*DPYS*	TSS200
cg07222604	6	30,418,872	0.041646	0.211	0.318					
cg01179851	18	76,746,274	0.005205	0.211	0.257				*SALL3*	Body
cg01204927	6	28,505,394	1.9E-06	0.211	0.204					
cg01504784	19	6,414,019	6.29E-47	0.211	0.211				*KHSRP*	3'UTR
cg24557048	1	2,063,799	2.65E-06	0.211	0.210				*PRKCZ*	5'UTR
cg26700716	2	183,730,940	5.01E-07	0.211	0.191				*FRZB*	1stExon
cg27100236	8	11,561,458	0.004283	0.211	0.207				*GATA4*	TSS1500
cg13361910	1	232,750,286	3.96E-12	0.211	0.198					
cg22752052	19	34,397,682	0.000157	0.211	0.154					
cg26389380	3	11,330,567	1.17E-06	0.211	0.223				*ATG7*	5'UTR
cg18011517	2	42,175,341	0.015445	0.211	0.238					
cg01262290	7	142,748,458	3.08E-10	0.211	0.211				*OR6V1*	TSS1500
cg06891775	10	80,704,982	3.02E-09	0.211	0.192				*LOC283050*	Body
cg15419378	17	79,435,967	3.25E-10	0.211	0.200					
cg26004203	1	217,310,918	4.5E-06	0.211	0.201			Yes	*ESRRG*	5'UTR
cg04329455	9	137,215,364	1.71E-08	0.211	0.201					
cg06951677	1	221,061,837	0.003492	0.211	0.131					
cg05600717	13	52,378,744	0.000882	0.211	0.202	4.93E-10	0.118		*DHRS12*	TSS1500
cg11108890	2	85,811,471	8.45E-05	0.211	0.209	1.14E-14	0.151		*VAMP5*	TSS200
cg11717507	12	114,838,825	0.032835	0.211	0.262				*TBX5*	Body
cg03607951	1	79,085,586	0.011634	0.211	0.171				*IFI44L*	TSS1500
cg10433128	16	70,720,314	2.02E-09	0.211	0.192				*MTSS1L*	TSS1500
cg12021814	2	172,973,195	0.000227	0.211	0.198					
cg26116447	6	21,666,030	0.041182	0.211	0.273				*FLJ22536*	TSS1500
cg00360794	15	41,803,850	0.002514	0.211	0.194				*LTK*	Body
cg05886811	6	17,280,740	0.000101	0.211	0.203				*RBM24*	TSS1500

cg13845561	9	71,631,514	9.41E-08	0.211	0.209					
cg19414706	8	76,319,215	2.38E-05	0.211	0.215					
cg21214293	5	145,713,882	0.010497	0.211	0.216					
cg00808969	11	77,898,785	1.72E-10	0.211	0.191				*USP35*	TSS1500
cg04605987	1	200,007,403	1.67E-06	0.211	0.193				*NR5A2*	Body
cg14416523	2	162,273,928	0.019386	0.211	0.232				*TBR1*	Body
cg21266502	4	155,663,225	0.001793	0.211	0.242					
cg15137566	4	13,544,301	0.003755	0.211	0.218				*NKX3-2*	Body
cg08102508	3	13,115,168	0.000332	0.211	0.221				*IQSEC1*	TSS1500
cg22517925	12	117,535,242	0.005898	0.211	0.124				*TESC*	Body
cg22900266	2	65,089,000	6.84E-13	0.211	0.200					
cg06613515	15	77,287,656	1.68E-14	0.211	0.201				*PSTPIP1*	5'UTR
cg06891458	15	41,951,943	0.001368	0.211	0.229				*MGA*	TSS1500
cg08132940	7	1,081,526	7.17E-25	0.211	0.198				*C7orf50*	Body
cg06372962	1	173,638,690	0.006912	0.211	0.134				*ANKRD45*	5'UTR
cg18485215	14	100,625,110	0.004128	0.211	0.196				*DEGS2*	Body
cg23346945	5	125,671,946	0.00326	0.211	0.186					
cg13280041	6	138,866,865	7.01E-11	0.211	0.219				*NHSL1*	Body
cg26942124	5	124,080,075	0.014354	0.211	0.235				*ZNF608*	1stExon
cg05295388	2	118,594,304	1.87E-09	0.211	0.212					
cg06133110	7	30,028,307	0.000956	0.211	0.113				*SCRN1*	5'UTR
cg10369363	12	52,404,422	5.3E-10	0.211	0.204				*GRASP*	Body
cg12017745	11	65,352,327	0.000993	0.211	0.248				*EHBP1L1*	Body
cg23739746	13	114,905,867	0.037055	0.211	0.200					
cg24348479	1	155,007,323	7.64E-08	0.211	0.207				*DCST1*	Body
cg27013947	X	21,776,178	3.92E-11	0.211	0.213				*SMPX*	1stExon
cg00414384	11	68,518,048	2.43E-10	0.211	0.192				*MTL5*	Body
cg07368507	5	137,774,431	5.74E-06	0.211	0.142				*REEP2*	TSS1500
cg14253596	13	110,132,672	0.031764	0.211	0.166			Yes		
cg24111955	7	64,734,226	0.018368	0.211	0.237					
cg03140487	X	40,027,582	5.55E-09	0.211	0.187				*BCOR*	5'UTR
cg24719487	1	78,512,129	0.000107	0.211	0.182				*GIPC2*	Body
cg00808935	16	85,215,140	0.012784	0.211	0.102					
cg06804778	1	147,790,658	1.15E-06	0.211	0.230					

cg21163429	10	44,788,812	0.009201	0.211	0.139					
cg15110917	7	7,959,339	0.012654	0.211	0.251					
cg18025275	8	22,498,768	2.09E-12	0.211	0.192				*BIN3*	Body
cg26053832	16	54,971,600	0.004047	0.211	0.214					
cg21921384	4	57,976,556	0.04811	0.211	0.217				*IGFBP7*	TSS200
cg00629427	13	95,357,638	1.1E-05	0.211	0.201					
cg09372750	14	23,823,420	9.99E-09	0.211	0.202				*SLC22A17*	TSS1500
cg15601228	1	21,996,376	0.009435	0.211	0.164				*RAP1GAP*	TSS1500
cg04834770	10	127,936,505	1.62E-05	0.211	0.180				*ADAM12*	Body
cg13248581	22	17,680,545	1.18E-06	0.211	0.191				*CECR1*	TSS200
cg07328796	2	227,389,311	1.53E-11	0.211	0.184					
cg05634630	12	99,251,017	7.86E-08	0.211	0.204				*ANKS1B*	Body
cg07136133	11	36,422,377	6.73E-06	0.211	0.159				*PRR5L*	5'UTR
cg07962338	19	53,540,902	0.044464	0.211	0.157					
cg20662858	2	235,166,931	0.029797	0.211	0.190					
cg21822146	14	77,961,897	7.82E-12	0.211	0.230				*ISM2*	Body
cg23238834	6	31,126,879	0.001225	0.211	0.182				*CCHCR1*	TSS1500
cg00158789	17	59,534,683	8.41E-06	0.211	0.212				*TBX4*	Body
cg04885832	5	176,560,265	0.033873	0.211	0.265				*NSD1*	TSS1500
cg26081162	6	49,518,449	0.010382	0.211	0.143	1.96E-08	0.078		*C6orf141*	5'UTR
cg03575666	8	41,168,186	1.35E-07	0.211	0.200				*SFRP1*	TSS1500
cg10992465	1	9,504,049	2.11E-09	0.211	0.196					
cg20359994	6	27,599,141	0.00094	0.211	0.193					
cg04983821	5	133,407,209	0.000104	0.211	0.196					
cg10502150	17	8,242,124	3.36E-13	0.211	0.194				*ODF4*	TSS1500
cg02774705	2	121,741,143	1.31E-07	0.211	0.214				*GLI2*	Body
cg03515656	16	1,600,304	5.11E-06	0.211	0.247				*IFT140*	Body
cg05460318	1	232,941,530	0.027524	0.211	0.168				*KIAA1383*	1stExon
cg13340335	11	133,907,726	0.043468	0.211	0.130				*LOC100128239*	Body
cg19708659	19	8,407,443	2.08E-10	0.211	0.198				*KANK3*	5'UTR
cg21626848	17	39,969,267	0.041833	0.211	0.089				*SC65*	TSS1500
cg22824738	12	54,765,988	1.37E-08	0.211	0.174				*ZNF385A*	Body
cg24880736	7	156,870,822	1.1E-07	0.211	0.244					
cg12209693	7	73,894,884	0.007213	0.211	0.173				*GTF2IRD1*	5'UTR

cg16598244	1	225,671,862	3.1E-07	0.211	0.226					
cg00835745	15	79,722,776	5.39E-07	0.211	0.200					
cg09195389	13	20,767,968	0.007383	0.211	0.162				*GJB2*	TSS1500
cg06819546	3	62,360,674	0.006459	0.211	0.146				*FEZF2*	TSS1500
cg08594681	8	27,468,684	7.9E-08	0.211	0.198				*CLU*	1stExon
cg04661929	17	75,320,035	1.27E-09	0.211	0.185				*SEPT9*	5'UTR
cg07493499	17	911,887	1.04E-13	0.211	0.213				*ABR*	Body
cg14264372	4	6,248,114	0.002887	0.211	0.203					
cg13155295	6	31,239,653	0.011175	0.211	0.299				*HLA-C*	Body
cg07725183	6	32,190,556	1.86E-05	0.211	0.205				*NOTCH4*	Body
cg13110334	22	19,003,848	1.95E-18	0.211	0.198				*DGCR9*	TSS1500
cg09064252	2	71,693,090	1.32E-07	0.211	0.172				*DYSF*	TSS1500
cg10379890	6	391,208	0.049268	0.211	0.066				*IRF4*	TSS1500
cg05560435	5	172,671,526	0.021939	0.211	0.213					
cg14210287	1	161,695,659	0.023176	0.211	0.176				*FCRLB*	Body
cg15844871	2	20,068,384	0.046926	0.211	0.092					
cg06535156	6	155,538,055	6.42E-05	0.211	0.161				*TIAM2*	Body
cg10095801	21	33,785,988	3.4E-08	0.211	0.200				*C21orf63*	Body
cg09232358	14	23,015,657	3.61E-06	0.211	0.208					
cg16066354	22	39,417,451	2.62E-05	0.211	0.197				*APOBEC3D*	1stExon
cg23687466	11	504,937	0.007675	0.211	0.182				*RNH1*	5'UTR
cg00841760	7	158,362,966	4.56E-09	0.211	0.217				*PTPRN2*	Body
cg09209257	14	78,907,020	3.12E-05	0.211	0.205				*NRXN3*	5'UTR
cg10324998	1	92,338,262	0.000692	0.211	0.242				*TGFBR3*	5'UTR
cg13671582	12	122,519,674	0.035025	0.211	0.180				*MLXIP*	Body
cg06329345	4	121,844,259	0.023065	0.211	0.043				*PRDM5*	TSS1500
cg11144753	14	103,740,065	0.000967	0.211	0.244					
cg20611680	1	16,554,171	1.19E-07	0.211	0.210					
cg05894970	3	119,041,204	0.048293	0.211	0.057				*CDGAP*	Body
cg24085232	5	151,195,433	1.23E-07	0.211	0.222					
cg14268200	17	21,094,779	4.25E-15	0.211	0.212				*DHRS7B*	3'UTR
cg23188430	6	125,850,052	0.000597	0.211	0.236					
cg03041109	4	109,057,004	5.95E-10	0.211	0.185				*LEF1*	Body
cg07956003	7	95,433,460	1.66E-20	0.211	0.193				*DYNC1I1*	5'UTR

cg16607065	1	3,607,222	0.006758	0.211	0.197	1.36E-14	0.179		*TP73*	Body
cg21751684	15	89,920,887	0.000191	0.211	0.192				*LOC254559*	TSS1500
cg00657094	1	1,174,190	2.29E-13	0.211	0.198					
cg08017928	7	156,801,818	0.028982	0.211	0.253				*MNX1*	1stExon
cg27088150	3	62,353,894	0.010293	0.211	0.234					
cg07376535	18	905,450	0.014627	0.211	0.193	4.73E-08	0.134		*ADCYAP1*	Body
cg08526991	2	5,831,394	2.16E-05	0.211	0.193				*SOX11*	TSS1500
cg13582816	4	85,404,099	0.001941	0.211	0.231					
cg14373403	6	169,351,425	3.1E-05	0.211	0.182					
cg15393221	19	40,919,485	1.65E-06	0.211	0.199				*PRX*	TSS1500
cg21865249	1	208,144,007	6.14E-12	0.211	0.187					
cg25463779	12	124,773,236	2.29E-09	0.211	0.195				*FAM101A*	TSS1500
cg11884933	7	2,774,414	3.3E-11	0.211	0.197				*GNA12*	Body
cg15619377	10	80,270,722	5.7E-06	0.211	0.178					
cg19940312	4	20,257,590	2.51E-05	0.211	0.209				*SLIT2*	Body
cg10308654	17	59,479,306	5.46E-06	0.211	0.187				*TBX2*	Body
cg14962509	1	36,039,655	1.11E-16	0.211	0.209				*TFAP2E*	Body
cg02892577	10	121,181,759	0.000168	0.211	0.236				*GRK5*	Body
cg06080874	18	46,501,625	0.005382	0.211	0.130					
cg06527318	1	25,349,006	1.75E-06	0.211	0.213					
cg09516959	7	30,721,478	4.63E-06	0.211	0.243				*CRHR2*	Body
cg18743287	19	55,575,837	5.59E-09	0.211	0.222				*RDH13*	TSS1500
cg16061498	18	55,095,886	0.002553	0.211	0.202					
cg06154531	1	846,507	9.32E-10	0.211	0.198					
cg21340170	6	5,084,517	0.007645	0.211	0.153				*PPP1R3G*	TSS1500
cg13607218	2	71,118,823	0.001821	0.211	0.274					
cg27310710	12	124,941,781	1.11E-06	0.211	0.225				*NCOR2*	Body
cg04897892	16	81,564,314	1.04E-05	0.211	0.195				*CMIP*	Body
cg10792302	12	54,376,019	8.55E-24	0.211	0.189					
cg16306900	1	182,026,435	2.36E-14	0.211	0.210				*ZNF648*	Body
cg18316621	6	144,283,348	0.001067	0.211	0.197				*PLAGL1*	5'UTR
cg16452651	21	35,016,873	6.8E-10	0.211	0.206				*ITSN1*	5'UTR
cg18637459	12	108,991,991	0.002661	0.211	0.283				*TMEM119*	TSS200
cg03192551	17	42,403,152	1.74E-05	0.210	0.169	8.47E-32	0.206		*SLC25A39*	TSS1500

cg08696638	16	31,160,393	1.36E-17	0.210	0.228				*PRSS36*	Body
cg09990169	2	241,835,740	1.26E-09	0.210	0.194				*C2orf54*	TSS200
cg11726288	1	11,902,283	7.88E-05	0.210	0.173				*CLCN6*	3'UTR
cg10420310	16	14,397,576	0.000109	0.210	0.215				*MIR193B*	TSS1500
cg11072645	7	157,754,746	0.00764	0.210	0.127				*PTPRN2*	Body
cg12634591	2	177,028,606	0.013393	0.210	0.208				*HOXD3*	TSS200
cg19442915	19	45,827,305	0.001268	0.210	0.166				*CKM*	TSS1500
cg23034373	21	37,915,354	8.47E-11	0.210	0.210				*CLDN14*	5'UTR
cg27038472	19	19,102,129	3.08E-14	0.210	0.201				*SFRS14*	3'UTR
cg07772660	1	17,085,863	1.27E-05	0.210	0.210				*MST1P9*	Body
cg15174396	6	29,796,363	0.00123	0.210	0.244				*HLA-G*	Body
cg22332157	X	12,995,746	0.008066	0.210	0.239					
cg00692173	1	53,217,358	4.71E-17	0.210	0.224				*ZYG11B*	Body
cg02807859	13	28,350,407	0.017625	0.210	0.244					
cg15618978	3	160,167,990	0.000878	0.210	0.198				*TRIM59*	TSS1500
cg16973715	3	64,254,112	7.89E-09	0.210	0.201					
cg17852588	6	76,059,756	0.001662	0.210	0.175				*FILIP1*	Body
cg07862554	3	154,797,083	0.014171	0.210	0.062				*MME*	TSS1500
cg08197287	4	87,952,173	0.000657	0.210	0.185				*AFF1*	Body
cg09491962	10	98,900,771	5.47E-14	0.210	0.198				*SLIT1*	Body
cg04511728	6	137,817,846	0.033536	0.210	0.246					
cg18152887	2	920,593	2.51E-05	0.210	0.168					
cg25632299	5	60,733,133	3.5E-10	0.210	0.198				*ZSWIM6*	Body
cg20347882	7	155,166,554	0.018886	0.210	0.199					
cg23990012	15	95,870,409	0.024048	0.210	0.169					
cg03465652	2	121,625,735	1.66E-06	0.210	0.195				*GLI2*	Body
cg03320492	1	45,187,810	2.19E-11	0.210	0.225				*C1orf228*	Body
cg04569165	X	153,688,677	5.16E-08	0.210	0.199				*PLXNA3*	Body
cg05348776	14	95,693,784	0.004717	0.210	0.133				*CLMN*	Body
cg06131666	X	18,373,515	1.49E-08	0.210	0.188				*SCML2*	TSS1500
cg15305361	8	1,405,138	1.5E-06	0.210	0.195					
cg00563678	4	141,968,563	0.000493	0.210	0.194				*RNF150*	Body
cg01501312	1	6,480,594	6.42E-05	0.210	0.184				*HES2*	TSS1500
cg19537511	17	8,213,813	9.35E-08	0.210	0.211	1.81E-14	0.115		*ARHGEF15*	5'UTR

cg22402467	12	7,168,056	2.18E-07	0.210	0.220				*C1S*	1stExon
cg20633941	6	169,351,479	0.000517	0.210	0.173					
cg16265906	5	115,152,326	0.006212	0.210	0.099				*CDO1*	1stExon
cg06625934	14	95,238,988	0.004492	0.210	0.239					
cg16906765	15	75,475,038	1.18E-06	0.210	0.140					
cg18158438	11	64,322,994	0.00272	0.210	0.230				*SLC22A11*	TSS200
cg10380643	1	1,356,764	5.01E-09	0.210	0.158				*LOC441869*	TSS200
cg23323669	6	31,126,866	0.011991	0.210	0.196				*CCHCR1*	TSS1500
cg20656587	17	44,895,317	3.28E-06	0.210	0.191				*WNT3*	Body
cg23176316	2	20,784,406	1.66E-13	0.210	0.198					
cg21865533	19	16,394,774	0.000862	0.210	0.173					
cg00595237	8	11,560,763	0.002674	0.210	0.212				*GATA4*	TSS1500
cg06961233	22	18,531,385	9.12E-07	0.210	0.187					
cg26242687	6	41,471,014	4.37E-06	0.210	0.224					
cg08438435	15	29,408,145	6.21E-07	0.210	0.193				*APBA2*	Body
cg17368808	20	9,496,892	2.01E-05	0.210	0.240				*C20orf103*	Body
cg09071155	7	27,280,914	3.7E-05	0.210	0.182				*EVX1*	TSS1500
cg11276093	10	95,123,774	2.39E-08	0.210	0.191				*MYOF*	Body
cg25510591	17	11,212,090	3.81E-11	0.210	0.187				*SHISA6*	Body
cg19319588	10	46,010,025	0.031411	0.210	0.119				*MARCH8*	Body
cg23623622	6	30,139,880	0.009846	0.210	0.213				*TRIM15*	Body
cg02672737	15	72,476,759	0.008169	0.210	0.165				*GRAMD2*	Body
cg06540458	1	226,951,747	0.000249	0.210	0.177					
cg11067829	13	23,500,153	0.00075	0.210	0.190					
cg11640208	17	42,857,157	0.033295	0.210	0.255				*ADAM11*	3'UTR
cg00895132	3	13,318,410	4.71E-20	0.210	0.215					
cg08738280	1	168,197,198	0.000001	0.210	0.201				*SFT2D2*	Body
cg15022739	18	34,823,045	2.18E-16	0.210	0.205				*BRUNOL4*	3'UTR
cg21714557	13	113,776,873	0.000139	0.210	0.246				*F10*	TSS1500
cg08467371	2	233,180,415	1.37E-08	0.210	0.194				*DIS3L2*	Body
cg15022039	5	139,081,063	8.26E-06	0.210	0.191					
cg16199747	6	147,235,033	1.07E-05	0.210	0.199					
cg02747319	2	242,480,276	9.4E-05	0.210	0.219					
cg14288326	7	77,042,644	4E-09	0.210	0.202				*PION*	Body

cg18941641	10	78,392,320	0.006235	0.210	0.214					
cg26505974	11	1,975,791	3.04E-17	0.210	0.183				*MRPL23*	Body
cg05707458	1	205,425,241	0.020594	0.210	0.188					
cg08004073	3	169,377,725	0.000127	0.210	0.244				*MECOM*	Body
cg10014308	2	73,518,221	0.01429	0.210	0.150				*EGR4*	3'UTR
cg16706349	10	85,836,590	0.025999	0.210	0.179					
cg23690166	17	46,711,017	0.002734	0.210	0.165				*MIR196A1*	TSS1500
cg24812442	2	237,079,690	0.011223	0.210	0.183					
cg16554164	1	32,410,678	7.45E-07	0.210	0.199					
cg21767952	2	220,379,260	8.56E-05	0.210	0.185				*ACCN4*	1stExon
cg02419321	12	54,811,762	5.28E-07	0.210	0.203				*ITGA5*	Body
cg04899656	15	62,718,755	3.9E-09	0.210	0.197					
cg26131286	6	29,521,783	0.0008	0.210	0.201					
cg10037049	12	48,276,633	1.43E-08	0.210	0.184				*VDR*	5'UTR
cg17520027	1	200,010,708	2.12E-13	0.210	0.203				*NR5A2*	Body
cg20276750	3	52,279,555	0.010179	0.210	0.169				*PPM1M*	TSS1500
cg25011252	8	61,777,859	5.6E-07	0.210	0.190				*CHD7*	Body
cg26469608	12	113,913,695	8.44E-07	0.210	0.235					
cg01153385	12	115,874,926	7.78E-10	0.210	0.220					
cg16248756	7	127,795,594	4.57E-05	0.210	0.222					
cg26140911	6	33,399,151	9.32E-15	0.210	0.203				*SYNGAP1*	Body
cg11035304	10	112,096,355	4.92E-09	0.210	0.200					
cg11610614	17	75,877,811	0.000202	0.210	0.218				*FLJ45079*	Body
cg00421173	4	140,603,682	1.53E-11	0.210	0.210			Yes	*MGST2*	Body
cg01777812	4	90,117,697	3.36E-13	0.210	0.179					
cg01978558	8	93,114,437	0.048674	0.210	0.252					
cg09096031	2	198,540,569	0.001801	0.210	0.205	1.4E-21	0.170		*RFTN2*	1stExon
cg24057218	14	68,660,680	4.69E-11	0.210	0.212				*RAD51L1*	Body
cg18778986	X	154,842,064	0.037131	0.210	0.212				*TMLHE*	5'UTR
cg03957481	22	50,986,962	0.000124	0.210	0.211				*KLHDC7B*	1stExon
cg07784042	2	67,893,948	0.003495	0.210	0.163					
cg08798307	7	141,190,187	3.58E-11	0.210	0.209					
cg13818316	21	43,185,803	0.001384	0.210	0.204				*RIPK4*	Body
cg15171154	3	30,722,557	3.49E-08	0.210	0.233				*TGFBR2*	Body

cg17579753	17	34,079,998	2.79E-06	0.210	0.206				*GAS2L2*	TSS200
cg25863503	6	27,799,295	0.001583	0.210	0.170				*HIST1H4K*	1stExon
cg00297721	20	55,200,767	4.42E-06	0.210	0.200					
cg02144647	22	45,597,019	6.74E-08	0.210	0.167				*C22orf9*	Body
cg23548163	19	46,807,119	0.000379	0.210	0.221				*HIF3A*	5'UTR
cg03225623	8	141,294,240	5.37E-17	0.210	0.193				*TRAPPC9*	Body
cg06962867	20	8,110,080	9.9E-10	0.210	0.204					
cg12006284	11	32,449,638	0.027919	0.210	0.245	7.12E-15	0.201		*WT1*	Body
cg27380715	17	39,802,082	3.16E-07	0.210	0.174					
cg00792751	11	44,979,029	5.23E-09	0.210	0.212					
cg08876943	5	1,865,843	0.000149	0.210	0.125					
cg13324103	10	29,948,428	1.19E-07	0.210	0.178				*SVIL*	5'UTR
cg18105675	2	3,642,400	2.99E-07	0.210	0.206	6.24E-08	0.077		*COLEC11*	TSS1500
cg01044025	3	151,613,372	3E-09	0.210	0.205					
cg10521450	10	105,439,280	4.89E-12	0.210	0.190				*SH3PXD2A*	Body
cg12199406	12	6,184,176	8.58E-06	0.210	0.208				*VWF*	Body
cg13520770	11	3,117,181	0.000276	0.210	0.208				*OSBPL5*	Body
cg18217235	6	50,817,157	0.016476	0.210	0.271					
cg26889552	5	179,032,532	2E-05	0.210	0.208				*RUFY1*	Body
cg05212464	5	154,071,076	4.1E-07	0.210	0.193					
cg14162361	9	82,449,859	0.000111	0.210	0.184					
cg15593298	3	142,681,996	0.000257	0.210	0.210				*PAQR9*	1stExon
cg16359657	1	41,119,931	1.91E-05	0.210	0.228				*RIMS3*	5'UTR
cg19474888	6	125,790,562	0.032072	0.210	0.240					
cg22985134	X	106,163,661	1.9E-10	0.210	0.199				*CLDN2*	5'UTR
cg01904243	14	74,251,254	1.5E-06	0.210	0.205				*C14orf43*	5'UTR
cg07977202	11	66,311,348	2.54E-19	0.210	0.203				*ZDHHC24*	Body
cg10068300	18	11,752,215	0.008677	0.210	0.181				*GNAL*	5'UTR
cg13940929	15	40,829,048	1.01E-12	0.210	0.200					
cg15619439	11	102,700,944	4.58E-06	0.210	0.183					
cg02495823	5	72,599,143	0.006545	0.210	0.242					
cg03642635	11	133,788,233	0.047921	0.210	0.269				*IGSF9B*	3'UTR
cg03958883	11	73,020,729	0.001501	0.210	0.090				*ARHGEF17*	1stExon
cg05053735	16	937,312	4.42E-09	0.210	0.199				*LMF1*	Body

cg13754259	10	101,294,337	0.002618	0.210	0.253				*NKX2-3*	Body
cg18808904	20	11,898,851	0.01737	0.210	0.072				*BTBD3*	1stExon
cg23345697	7	520,489	7.74E-09	0.210	0.161					
cg02297616	1	40,255,140	4.62E-05	0.210	0.134				*BMP8B*	TSS1500
cg07267600	12	2,750,053	0.000643	0.210	0.224				*CACNA1C*	Body
cg04339790	16	10,675,656	5.06E-08	0.210	0.205				*EMP2*	TSS1500
cg23448978	7	51,209,365	0.003979	0.210	0.145				*COBL*	Body
cg24757778	10	60,616,392	6.2E-05	0.210	0.191					
cg25258843	8	10,587,305	0.002843	0.210	0.101				*SOX7*	Body
cg25695992	2	38,057,966	7.45E-09	0.210	0.225					
cg27550721	15	79,381,747	0.003492	0.210	0.232				*RASGRF1*	Body
cg04850286	10	81,895,943	0.004282	0.210	0.207				*PLAC9*	Body
cg09417849	16	331,892	0.015848	0.210	0.235				*ARHGDIG*	Body
cg05422883	2	43,072,932	7.16E-08	0.210	0.214					
cg11273176	2	26,947,126	3.71E-27	0.210	0.207				*KCNK3*	Body
cg13967908	9	117,159,457	9.64E-09	0.210	0.223					
cg18311360	19	34,311,562	0.010061	0.210	0.215					
cg05091135	13	51,103,373	0.007397	0.210	0.250					
cg00690609	10	72,218,900	0.002355	0.210	0.192					
cg15944060	5	180,248,018	0.000244	0.210	0.216					
cg25460753	6	10,384,975	0.000111	0.210	0.212					
cg01480180	7	90,896,329	0.017991	0.210	0.272				*FZD1*	1stExon
cg02216246	11	128,564,756	0.017525	0.210	0.172				*FLI1*	Body
cg19375810	3	42,787,927	3.49E-07	0.210	0.233				*CCDC13*	Body
cg13471424	1	149,223,500	0.000158	0.210	0.236					
cg19419874	1	48,462,278	0.014979	0.210	0.085					
cg26990680	10	50,816,058	0.015211	0.210	0.264				*CHAT*	TSS1500
cg16902385	18	55,019,669	0.003407	0.210	0.225				*ST8SIA3*	TSS200
cg23032674	6	70,992,911	0.001522	0.210	0.194				*COL9A1*	Body
cg19424531	8	65,711,522	0.008253	0.210	0.109				*CYP7B1*	TSS200
cg08141862	3	177,458,423	1.75E-09	0.210	0.183					
cg10481065	20	37,357,371	0.015486	0.210	0.206			Yes	*SLC32A1*	3'UTR
cg12069540	10	82,173,570	3.45E-06	0.210	0.192				*C10orf58*	TSS200
cg12542656	2	43,269,469	7.82E-06	0.210	0.222					

cg13547889	9	139,433,088	0.008891	0.210	0.125				*NOTCH1*	Body
cg14623306	9	84,280,537	2.05E-09	0.210	0.183				*TLE1*	Body
cg26811372	5	140,772,182	0.009688	0.210	0.262				*PCDHGA4*	Body
cg20248516	2	119,602,901	0.020821	0.210	0.258				*EN1*	Body
cg24004007	15	63,889,250	1.07E-08	0.210	0.203				*FBXL22*	TSS1500
cg02081019	5	92,930,813	1.53E-06	0.210	0.208					
cg22261669	13	98,959,799	1.41E-08	0.210	0.162				*FARP1*	Body
cg23578346	2	3,750,973	5.63E-05	0.210	0.224					
cg15934368	2	240,044,021	1.31E-07	0.210	0.217				*HDAC4*	Body
cg20656294	17	76,962,736	8.64E-11	0.210	0.189					
cg02564259	16	67,978,429	3.93E-11	0.210	0.186				*SLC12A4*	3'UTR
cg24686957	6	5,214,950	0.000124	0.210	0.174				*LYRM4*	Body
cg16105870	2	131,451,075	1.44E-07	0.210	0.215					
cg25117523	17	76,719,640	8.2E-05	0.210	0.244				*CYTH1*	Body
cg05146922	2	10,634,174	1.26E-05	0.210	0.207					
cg06963205	6	73,092,820	1.67E-12	0.210	0.202				*RIMS1*	Body
cg14181576	1	27,961,563	4.83E-07	0.210	0.219				*FGR*	5'UTR
cg15584486	8	100,667,796	8.59E-09	0.210	0.194				*VPS13B*	Body
cg18855104	2	45,181,829	0.00015	0.210	0.218					
cg22501608	15	78,449,129	1.33E-08	0.210	0.205				*IDH3A*	Body
cg25040466	20	61,636,172	0.000128	0.210	0.201					
cg05933037	16	81,223,254	9.18E-15	0.210	0.202				*PKD1L2*	Body
cg12477370	6	45,391,852	0.005661	0.210	0.162				*RUNX2*	Body
cg02716490	2	242,986,091	8.67E-07	0.210	0.165					
cg09746494	4	147,568,715	0.004128	0.210	0.234					
cg11796455	6	10,883,895	0.000958	0.210	0.230					
cg21837207	1	119,869,897	0.034346	0.210	0.165					
cg07298347	2	153,264,085	0.000592	0.210	0.202				*FMNL2*	Body
cg09174411	12	107,792,027	7.1E-12	0.210	0.194				*BTBD11*	Body
cg14579118	7	43,797,547	4.57E-10	0.210	0.189				*BLVRA*	TSS1500
cg15634877	2	220,299,643	1.34E-06	0.210	0.211				*SPEG*	TSS200
cg19035526	2	217,107,125	0.000147	0.210	0.180					
cg04293175	11	14,282,775	4.47E-12	0.210	0.217				*SPON1*	Body
cg04436994	1	221,055,665	7.48E-16	0.210	0.214				*HLX*	Body

cg04819580	7	150,635,526	0.029074	0.210	0.217					
cg15263021	11	61,273,282	1.28E-10	0.210	0.207					
cg17552357	17	76,932,332	2.53E-11	0.210	0.218					
cg11587191	1	3,382,237	1.58E-07	0.210	0.215				*ARHGEF16*	Body
cg15453278	11	67,134,607	8.33E-09	0.210	0.210				*CLCF1*	Body
cg06801857	1	230,367,682	1.11E-05	0.210	0.216				*GALNT2*	Body
cg23625823	4	183,698,078	0.000666	0.210	0.162				*ODZ3*	Body
cg06049171	15	69,126,265	1.78E-09	0.210	0.202				*MIR548H4*	Body
cg09730801	17	48,048,875	1.52E-10	0.210	0.192				*DLX4*	Body
cg11664818	21	37,852,665	2.01E-14	0.210	0.188				*CLDN14*	5'UTR
cg24881973	4	6,660,945	0.001448	0.210	0.182					
cg26938013	14	69,015,199	5.16E-09	0.210	0.213				*RAD51L1*	Body
cg00272971	10	102,419,564	0.024191	0.210	0.198					
cg00539300	16	2,895,093	6.19E-07	0.210	0.190					
cg11165960	15	62,876,940	5.33E-13	0.210	0.210					
cg13098071	1	156,370,496	2.95E-05	0.210	0.226					
cg04337096	5	146,257,347	0.000585	0.210	0.232				*PPP2R2B*	Body
cg03540028	21	43,183,977	2.12E-11	0.210	0.190				*RIPK4*	Body
cg27480580	19	12,835,134	1.14E-06	0.209	0.202				*TNPO2*	TSS1500
cg08903441	17	80,304,981	0.000832	0.209	0.177					
cg10602757	2	114,256,781	0.000105	0.209	0.233				*FOXD4L1*	1stExon
cg23213230	13	50,570,091	0.00613	0.209	0.123				*TRIM13*	TSS1500
cg00928753	X	36,976,515	2.97E-05	0.209	0.220					
cg12456799	10	106,441,036	0.00406	0.209	0.254				*SORCS3*	Body
cg19688584	6	167,529,678	1.28E-06	0.209	0.157				*CCR6*	5'UTR
cg03883502	6	5,086,610	3.53E-07	0.209	0.178				*PPP1R3G*	1stExon
cg05996160	2	16,124,662	8.44E-10	0.209	0.196					
cg19871588	5	151,098,316	9.04E-15	0.209	0.206			Yes		
cg22917372	12	122,186,536	8.17E-09	0.209	0.212				*TMEM120B*	Body
cg05437823	6	50,813,676	0.028917	0.209	0.296				*TFAP2B*	3'UTR
cg23395177	14	77,498,304	1.51E-15	0.209	0.214			Yes		
cg23599385	2	106,637,299	8.69E-09	0.209	0.169					
cg04037470	22	42,196,210	0.000789	0.209	0.150				*CCDC134*	TSS1500
cg17824540	2	161,260,031	3.02E-06	0.209	0.174				*RBMS1*	Body

cg10269548	4	53,728,923	8.38E-09	0.209	0.220				*RASL11B*	Body
cg12544191	5	172,671,754	0.000281	0.209	0.198					
cg27022584	17	79,071,378	0.006366	0.209	0.246				*BAIAP2*	Body
cg04847275	14	95,693,828	0.025299	0.209	0.125				*CLMN*	Body
cg06992324	2	122,322,947	1.78E-11	0.209	0.232				*CLASP1*	Body
cg09307985	19	36,643,070	1.46E-08	0.209	0.223				*COX7A1*	Body
cg09450352	7	157,495,481	1.63E-08	0.209	0.196				*PTPRN2*	Body
cg12107072	6	36,826,690	1.93E-07	0.209	0.192				*PPIL1*	Body
cg13902024	7	131,960,464	0.001586	0.209	0.227				*PLXNA4*	Body
cg25400746	9	131,965,635	0.002878	0.209	0.119					
cg15025248	2	191,278,648	8.03E-05	0.209	0.197				*MFSD6*	5'UTR
cg20489909	2	242,711,046	8.3E-14	0.209	0.190					
cg10726357	9	115,653,543	3.51E-11	0.209	0.198	1.11E-19	0.147		*SLC46A2*	TSS1500
cg14606256	12	54,132,568	1.24E-05	0.209	0.196					
cg20438687	17	4,761,644	0.026451	0.209	0.119				*MINK1*	Body
cg23026995	X	128,872,940	9.58E-07	0.209	0.192	1.38E-19	0.229		*XPNPEP2*	TSS200
cg08006672	16	85,427,738	5E-29	0.209	0.200					
cg12533496	17	26,859,931	1.54E-13	0.209	0.213				*FOXN1*	Body
cg00875272	1	47,695,321	0.02977	0.209	0.231	3.72E-11	0.175		*TAL1*	5'UTR
cg13757263	3	53,807,593	0.000329	0.209	0.262				*CACNA1D*	Body
cg19600750	14	57,274,677	0.008413	0.209	0.192				*OTX2*	5'UTR
cg02064724	14	74,179,953	0.014132	0.209	0.126				*PNMA1*	1stExon
cg04212150	18	56,531,132	6.43E-05	0.209	0.114				*ZNF532*	5'UTR
cg09042448	8	23,260,443	4.3E-11	0.209	0.188				*LOXL2*	5'UTR
cg11142489	4	74,810,142	0.006114	0.209	0.187					
cg13604166	3	133,811,326	3.16E-17	0.209	0.201					
cg25102417	1	115,632,693	0.016927	0.209	0.079				*TSPAN2*	TSS1500
cg07076816	7	38,351,154	0.001252	0.209	0.107					
cg07126235	6	43,192,225	5.64E-05	0.209	0.209				*CUL9*	3'UTR
cg09287629	2	203,036,208	5.17E-06	0.209	0.214					
cg14608384	5	54,516,879	6.32E-05	0.209	0.201					
cg22724998	15	60,288,082	0.009275	0.209	0.160					
cg27279674	12	3,049,965	8.13E-15	0.209	0.206				*TULP3*	3'UTR
cg02664349	18	11,149,841	0.009564	0.209	0.145				*FAM38B*	TSS1500

cg06255601	13	51,086,835	0.041963	0.209	0.104					
cg07501955	6	161,395,110	1.15E-08	0.209	0.206					
cg24324815	10	21,783,445	0.000299	0.209	0.194				*C10orf114*	3'UTR
cg27109600	2	200,332,025	1.76E-05	0.209	0.196				*FLJ32063*	TSS1500
cg14260073	6	137,244,681	6.22E-05	0.209	0.202				*SLC35D3*	Body
cg07318609	10	128,585,354	0.000228	0.209	0.234					
cg27376024	11	63,273,565	3.39E-08	0.209	0.188				*LGALS12*	5'UTR
cg26094554	17	36,719,654	3.62E-05	0.209	0.203				*SRCIN1*	Body
cg10234477	12	6,585,594	0.0003	0.209	0.191					
cg15202353	1	162,831,613	5.71E-11	0.209	0.205				*C1orf110*	5'UTR
cg19159365	1	67,218,505	0.000155	0.209	0.181				*TCTEX1D1*	5'UTR
cg22041190	11	125,037,348	0.006213	0.209	0.113				*PKNOX2*	5'UTR
cg22362636	4	2,758,447	0.002833	0.209	0.133	2.05E-40	0.181		*TNIP2*	TSS1500
cg05114606	17	21,274,193	4.11E-06	0.209	0.236					
cg22891600	2	104,018,319	0.02823	0.209	0.267					
cg15082028	1	19,043,507	0.040332	0.209	0.248				*PAX7*	Body
cg23151014	4	2,418,311	9.45E-07	0.209	0.195				*ZFYVE28*	Body
cg01876338	2	54,087,008	0.000541	0.209	0.126				*GPR75*	5'UTR
cg08618173	16	81,709,749	1.95E-17	0.209	0.192				*CMIP*	Body
cg09820479	X	128,810,342	9.8E-08	0.209	0.215					
cg14080888	9	130,712,990	9.12E-11	0.209	0.193				*FAM102A*	5'UTR
cg07284994	16	66,982,673	1.03E-06	0.209	0.191					
cg01896298	X	69,643,036	0.00304	0.209	0.186				*GDPD2*	1stExon
cg06960461	12	132,950,559	0.0349	0.209	0.224					
cg08712669	1	151,585,705	0.000617	0.209	0.118				*SNX27*	Body
cg10506882	3	35,681,627	0.01536	0.209	0.153					
cg14880894	4	78,697,859	1.91E-09	0.209	0.208				*CNOT6L*	Body
cg20635409	12	103,359,273	0.002018	0.209	0.232					
cg04552206	10	102,896,546	0.000447	0.209	0.166				*TLX1*	Body
cg04651781	4	1,398,798	0.005732	0.209	0.217					
cg08081323	11	57,232,407	2.59E-08	0.209	0.199				*RTN4RL2*	Body
cg23144473	7	38,340,103	0.000137	0.209	0.212					
cg18680626	4	6,052,527	0.003367	0.209	0.170				*JAKMIP1*	Body
cg21863499	1	27,113,640	0.000786	0.209	0.143				*PIGV*	TSS1500

cg00109703	20	39,320,005	0.000174	0.209	0.209					
cg12584458	6	28,447,107	0.00447	0.209	0.211					
cg13565575	12	48,397,568	0.009153	0.209	0.109				*COL2A1*	Body
cg21325715	17	80,297,128	0.000228	0.209	0.270					
cg26931862	12	54,349,169	0.000283	0.209	0.213				*HOXC12*	1stExon
cg01377200	4	184,938,316	0.000136	0.209	0.212				*STOX2*	Body
cg01857151	5	145,304,100	2.46E-11	0.209	0.201					
cg23194346	X	49,033,828	1.33E-15	0.209	0.205				*PRICKLE3*	Body
cg24549659	4	187,219,643	1.49E-08	0.209	0.184					
cg06520675	10	102,996,310	0.022113	0.209	0.269				*FLJ41350*	Body
cg11287077	5	158,590,712	4.21E-21	0.209	0.192				*RNF145*	Body
cg13364881	1	197,880,513	0.005272	0.209	0.247				*LHX9*	TSS1500
cg04247603	6	5,994,743	0.025149	0.209	0.197					
cg07333792	14	26,674,164	0.005306	0.209	0.234					
cg24642523	14	65,009,265	3.71E-05	0.209	0.183	1.16E-09	0.106		*HSPA2*	1stExon
cg02452944	5	140,810,109	0.011403	0.209	0.255				*PCDHGA4*	Body
cg13709982	9	136,567,739	0.000455	0.209	0.213				*SARDH*	Body
cg18267489	20	23,067,159	0.000559	0.209	0.212				*CD93*	TSS200
cg17503456	3	138,666,054	0.049297	0.209	0.231	0.0000256	0.092		*FOXL2*	TSS200
cg02702693	12	116,808,845	1.82E-18	0.209	0.209					
cg13382769	7	79,084,078	0.013914	0.209	0.203				*MAGI2*	TSS1500
cg21767759	17	48,154,356	7.36E-14	0.209	0.209				*ITGA3*	Body
cg00713400	7	22,233,206	1.99E-09	0.209	0.194				*RAPGEF5*	Body
cg09792970	12	111,374,169	0.016963	0.209	0.191					
cg11267546	7	27,167,828	4.11E-09	0.209	0.229				*HOXA3*	TSS1500
cg12078229	19	19,245,687	1.3E-05	0.209	0.199				*TMEM161A*	Body
cg18028999	15	81,426,610	0.009392	0.209	0.176				*C15orf26*	TSS200
cg00328900	5	82,769,097	0.017575	0.209	0.087				*VCAN*	5'UTR
cg03235661	20	60,525,775	9.88E-05	0.209	0.182					
cg04661629	1	26,687,174	0.022757	0.209	0.274					
cg14568105	19	37,282,931	0.017417	0.209	0.119					
cg16823083	1	3,567,412	3.92E-05	0.209	0.210				*WDR8*	TSS1500
cg25600472	5	140,857,647	9.69E-10	0.209	0.186				*PCDHGA4*	Body
cg02721394	3	195,914,017	0.000165	0.209	0.181					

cg20925383	13	50,702,410	1.73E-15	0.209	0.194					
cg02043791	8	38,322,629	6.55E-08	0.209	0.200				*FGFR1*	5'UTR
cg11302945	12	115,133,159	2.48E-11	0.209	0.178					
cg14667799	17	17,253,104	1.57E-20	0.209	0.203					
cg14963928	1	40,392,088	2.82E-11	0.209	0.210					
cg20640281	17	53,341,592	1.82E-05	0.209	0.198				*HLF*	TSS1500
cg21175326	14	59,074,518	0.003541	0.209	0.206					
cg03591690	3	166,884,766	0.001279	0.209	0.202					
cg17552714	13	100,607,949	0.000449	0.209	0.213					
cg26853787	2	202,904,337	1.97E-05	0.209	0.221					
cg10418755	11	27,015,519	0.047732	0.209	0.274				*FIBIN*	TSS200
cg21663666	2	232,220,549	7.74E-07	0.209	0.192					
cg15807143	2	3,452,624	2.59E-06	0.209	0.201				*TTC15*	Body
cg21065196	15	89,913,423	0.025711	0.209	0.197					
cg26248586	14	95,693,981	4.94E-05	0.209	0.146				*CLMN*	Body
cg24522654	12	19,535,154	0.016315	0.209	0.201					
cg00910882	15	65,648,312	1.98E-05	0.209	0.121				*IGDCC3*	Body
cg01357370	1	156,818,961	0.002948	0.209	0.209				*NTRK1*	Body
cg05010967	6	44,310,640	0.003162	0.209	0.213	5.28E-22	0.197		*SPATS1*	5'UTR
cg07631407	6	43,243,961	1.66E-05	0.209	0.218				*TTBK1*	Body
cg08473330	2	63,282,883	3.56E-05	0.209	0.195				*OTX1*	Body
cg16593333	12	96,514,873	4.91E-07	0.209	0.184					
cg21077578	7	1,022,970	1.57E-10	0.209	0.194				*CYP2W1*	1stExon
cg24495008	15	90,198,882	0.020517	0.209	0.110				*KIF7*	TSS1500
cg07393369	7	550,640	2.82E-12	0.209	0.173				*PDGFA*	Body
cg07972762	22	33,196,384	0.038095	0.209	0.283			Yes	*SYN3*	Body
cg22139212	15	68,062,715	6.3E-07	0.209	0.190				*MAP2K5*	Body
cg25882366	17	46,622,037	0.000238	0.209	0.217	2.08E-21	0.208		*HOXB2*	1stExon
cg02633924	17	75,315,563	0.035377	0.209	0.215				*SEPT9*	5'UTR
cg08122232	17	74,100,132	6.04E-08	0.209	0.184				*EXOC7*	TSS1500
cg26727435	22	50,453,437	0.00702	0.209	0.135					
cg08647652	17	18,538,582	5.78E-07	0.209	0.210					
cg02728721	20	9,487,112	1.52E-10	0.209	0.176					
cg07658590	21	46,963,587	1.89E-06	0.209	0.209	3.16E-13	0.077		*SLC19A1*	TSS1500

cg23052861	14	69,448,387	5.5E-13	0.209	0.190					
cg24902920	6	10,416,373	0.019505	0.209	0.134				*TFAP2A*	Body
cg00426659	12	99,548,329	3E-05	0.209	0.176				*ANKS1B*	1stExon
cg08327744	8	33,423,298	1.11E-06	0.209	0.196				*RNF122*	Body
cg11998932	7	3,901,843	0.000118	0.209	0.228				*SDK1*	Body
cg16996144	1	67,217,950	0.000621	0.209	0.210				*TCTEX1D1*	TSS200
cg14930075	8	140,715,018	0.013744	0.209	0.190				*KCNK9*	1stExon
cg13755873	17	70,113,187	0.000816	0.209	0.219					
cg17612681	2	66,298,197	2.43E-07	0.209	0.219					
cg18541254	13	112,709,550	0.000151	0.209	0.162					
cg18709570	12	8,172,018	0.041824	0.209	0.167					
cg02628360	17	79,315,269	0.002289	0.209	0.238					
cg06612196	6	6,737,390	0.000857	0.209	0.218					
cg10553596	2	242,751,847	0.000937	0.209	0.236				*NEU4*	TSS200
cg11427314	7	157,516,593	1.74E-13	0.209	0.206				*PTPRN2*	Body
cg22093640	15	29,033,886	0.000282	0.209	0.211					
cg22242539	17	1,665,220	4.01E-08	0.209	0.205	4.91E-34	0.136		*SERPINF1*	TSS200
cg24311933	1	180,138,771	3.28E-17	0.209	0.205				*QSOX1*	Body
cg26662049	15	89,903,672	0.013527	0.209	0.148					
cg19696622	X	20,285,908	0.017438	0.209	0.106	4.46E-11	0.057		*RPS6KA3*	TSS1500
cg17860158	1	205,012,569	0.008842	0.209	0.286	9.55E-14	0.131		*CNTN2*	5'UTR
cg00701706	17	72,931,632	3.13E-09	0.209	0.203				*OTOP3*	TSS1500
cg02065790	12	27,674,284	3.09E-10	0.209	0.208					
cg04032071	12	54,376,241	3.33E-06	0.209	0.161					
cg04457792	10	102,473,207	0.008113	0.209	0.233					
cg12453631	3	157,812,226	3.98E-06	0.209	0.219					
cg22560214	4	13,531,213	1.5E-06	0.209	0.189					
cg05479627	16	869,560	0.00129	0.209	0.229					
cg14041452	14	38,071,968	1.05E-06	0.209	0.222					
cg18916055	17	74,562,048	0.01661	0.209	0.211				*ST6GALNAC2*	3'UTR
cg19317960	4	54,452,606	6.21E-28	0.209	0.203				*LNX1*	5'UTR
cg21140843	17	1,398,405	5.1E-13	0.209	0.193				*INPP5K*	3'UTR
cg20987924	15	60,296,981	0.008583	0.209	0.120				*FOXB1*	5'UTR
cg11639708	6	20,209,127	2.18E-05	0.209	0.224				*MBOAT1*	Body

cg20674701	8	10,591,081	0.000153	0.209	0.225					
cg03160135	6	117,586,918	0.000136	0.209	0.158	9.42E-19	0.181		*VGLL2*	1stExon
cg24115264	13	43,149,043	0.006089	0.209	0.217				*TNFSF11*	Body
cg00929496	6	72,060,594	7.42E-11	0.209	0.221					
cg02909789	14	68,386,026	1.05E-19	0.209	0.237				*RAD51L1*	Body
cg04600055	8	102,092,805	0.026491	0.209	0.215					
cg26681975	6	36,238,123	0.011936	0.209	0.226				*PNPLA1*	5'UTR
cg24396631	X	119,134,082	0.001083	0.209	0.159					
cg03129014	13	20,736,156	1.1E-06	0.209	0.188				*GJA3*	TSS1500
cg07780095	12	85,674,296	0.044943	0.209	0.249				*ALX1*	Body
cg10123662	1	17,865,713	2.34E-06	0.209	0.202				*ARHGEF10L*	TSS1500
cg25934064	3	52,569,026	1.19E-09	0.209	0.174				*NT5DC2*	5'UTR
cg18664514	2	219,851,181	2.58E-06	0.209	0.211				*FEV*	TSS1500
cg26643967	19	36,485,282	0.001256	0.209	0.206				*SDHAF1*	TSS1500
cg27109106	5	141,294,040	0.026103	0.209	0.127					
cg00694520	2	223,916,687	0.025511	0.209	0.196				*KCNE4*	TSS200
cg02266399	7	143,582,146	1.15E-10	0.209	0.221				*FAM115A*	5'UTR
cg04155718	3	52,519,802	1.59E-16	0.209	0.190				*NISCH*	Body
cg15195412	16	57,406,955	4.97E-06	0.209	0.181	3.18E-36	0.190		*CX3CL1*	Body
cg22539744	3	64,476,370	1.95E-11	0.209	0.188					
cg07020001	12	122,019,031	0.018035	0.209	0.193				*KDM2B*	TSS200
cg07906193	10	126,599,966	1.31E-10	0.209	0.205					
cg10420161	10	102,899,983	9.9E-07	0.209	0.190					
cg24407096	6	169,653,618	0.03261	0.209	0.311				*THBS2*	5'UTR
cg25075776	6	30,848,828	3.31E-09	0.209	0.195					
cg18593081	1	181,286,640	0.016355	0.209	0.162					
cg20545410	10	33,232,548	1.13E-10	0.209	0.195				*ITGB1*	5'UTR
cg22758916	15	42,303,292	5.75E-07	0.209	0.200				*PLA2G4E*	TSS1500
cg24599107	1	161,049,443	2.5E-21	0.209	0.191				*PVRL4*	Body
cg02100373	5	134,367,090	0.000115	0.209	0.241				*PITX1*	Body
cg14375890	6	13,355,672	1.03E-13	0.209	0.182					
cg07128124	21	44,070,430	1.16E-06	0.209	0.227					
cg23129478	18	44,337,922	0.049937	0.209	0.060	4.48E-10	0.132		*ST8SIA5*	TSS1500
cg01837846	2	23,755,343	4.52E-08	0.209	0.195				*KLHL29*	5'UTR

cg02892286	1	214,153,377	0.04692	0.209	0.287					
cg09579094	2	174,949,048	1.46E-10	0.209	0.173				*OLA1*	Body
cg09632271	4	68,441,222	0.03237	0.209	0.233				*STAP1*	Body
cg07273992	11	68,925,191	3.66E-11	0.209	0.214					
cg10742801	10	135,161,047	0.011243	0.209	0.190	5.67E-21	0.197		*PRAP1*	Body
cg01957088	8	122,651,486	0.016257	0.209	0.254				*HAS2*	5'UTR
cg10104252	12	3,563,899	0.006959	0.209	0.214					
cg12055395	7	96,642,605	0.010952	0.209	0.228				*DLX6AS*	Body
cg21347353	22	25,678,439	0.049787	0.209	0.117					
cg21913528	1	185,283,071	1.24E-06	0.209	0.217				*IVNS1ABP*	5'UTR
cg08031967	17	853,971	2.8E-08	0.208	0.210				*NXN*	Body
cg08478116	15	32,933,919	2.17E-09	0.208	0.161			Yes	*SCG5*	1stExon
cg18396357	2	50,201,413	0.035502	0.208	0.231				*NRXN1*	Body
cg23151649	7	151,393,469	3.13E-05	0.208	0.180				*PRKAG2*	Body
cg00588517	6	31,895,464	3.7E-09	0.208	0.199				*C2*	1stExon
cg11726792	4	113,432,701	1.19E-06	0.208	0.198					
cg16484473	10	50,649,723	2.12E-05	0.208	0.184					
cg03249020	7	143,060,108	5.07E-06	0.208	0.173				*FAM131B*	TSS1500
cg04361266	16	20,910,376	2.18E-23	0.208	0.233				*LYRM1*	TSS1500
cg22119466	2	9,331,371	1.01E-05	0.208	0.224					
cg01020263	16	50,732,073	0.001484	0.208	0.164				*NOD2*	Body
cg25995980	10	46,993,515	7.83E-07	0.208	0.167				*GPRIN2*	TSS200
cg14865129	6	3,229,530	0.012535	0.208	0.280					
cg03497399	3	33,260,934	9.75E-05	0.208	0.215				*SUSD5*	TSS1500
cg14099718	9	36,879,449	9.16E-09	0.208	0.193				*PAX5*	Body
cg16706749	15	74,222,816	1.31E-09	0.208	0.198				*LOXL1*	Body
cg22826333	4	48,987,791	1.85E-05	0.208	0.196				*CWH43*	TSS1500
cg25180075	2	129,076,367	0.002557	0.208	0.209				*HS6ST1*	TSS200
cg01393945	5	10,457,034	2.2E-39	0.208	0.209				*ROPN1L*	Body
cg16185299	5	3,326,644	0.004494	0.208	0.236					
cg16549043	1	156,050,126	2.98E-09	0.208	0.221				*MEX3A*	Body
cg23706836	8	6,407,997	9.3E-09	0.208	0.161				*ANGPT2*	Body
cg23710218	8	72,756,656	0.000133	0.208	0.234	3.95E-29	0.211		*MSC*	1stExon
cg01650904	20	37,354,889	0.000593	0.208	0.248				*SLC32A1*	Body

cg06437417	11	111,895,279	0.002583	0.208	0.161				*DLAT*	TSS1500
cg07983363	15	101,991,886	3.08E-07	0.208	0.205				*PCSK6*	Body
cg15900221	1	3,382,434	3.49E-13	0.208	0.191				*ARHGEF16*	Body
cg17018946	18	5,894,665	0.02193	0.208	0.257					
cg19697981	6	108,487,820	0.010154	0.208	0.307	3.39E-17	0.178		*NR2E1*	5'UTR
cg08156066	20	21,695,334	0.015494	0.208	0.302				*PAX1*	Body
cg09873409	7	132,095,782	1.08E-13	0.208	0.200				*PLXNA4*	Body
cg00509670	14	37,130,313	0.000142	0.208	0.208	0.0406722	0.038		*PAX9*	5'UTR
cg04091555	4	79,417,584	9.57E-16	0.208	0.193				*FRAS1*	Body
cg23975840	12	117,042,895	0.004728	0.208	0.215					
cg05766251	17	37,024,760	1.05E-05	0.208	0.193				*LASP1*	TSS1500
cg19603836	10	30,317,688	1.56E-09	0.208	0.211				*KIAA1462*	Body
cg03792042	8	145,045,235	1.27E-06	0.208	0.186				*PLEC1*	Body
cg12927277	2	238,509,162	1.05E-07	0.208	0.228					
cg20442078	2	27,531,360	4.82E-09	0.208	0.220				*UCN*	TSS1500
cg23497016	2	66,666,684	0.034088	0.208	0.233				*MEIS1*	Body
cg02109484	2	74,875,253	6.76E-05	0.208	0.182				*C2orf65*	TSS200
cg15840039	X	66,788,098	4.52E-09	0.208	0.209				*AR*	TSS1500
cg16927242	2	66,664,667	0.016511	0.208	0.246				*MEIS1*	Body
cg05143345	14	23,447,408	1.43E-06	0.208	0.196				*JUB*	Body
cg08832220	3	55,516,184	5.61E-05	0.208	0.179				*WNT5A*	Body
cg10018167	14	64,663,683	2.9E-08	0.208	0.203				*SYNE2*	Body
cg12186317	5	1,183,173	3.95E-06	0.208	0.221					
cg16357388	4	5,892,083	0.018974	0.208	0.214				*CRMP1*	Body
cg26266842	1	99,470,801	0.025519	0.208	0.257				*LPPR5*	TSS1500
cg15012054	12	13,253,742	0.000202	0.208	0.191				*GSG1*	Body
cg17403397	2	145,282,262	1.11E-05	0.208	0.200					
cg25889711	10	102,473,609	0.006903	0.208	0.186					
cg14643892	12	64,784,070	0.00027	0.208	0.226				*C12orf56*	Body
cg13918518	17	59,488,240	2.26E-05	0.208	0.190				*C17orf82*	TSS1500
cg14515812	1	21,978,863	3.06E-07	0.208	0.204				*RAP1GAP*	5'UTR
cg18438591	1	205,473,341	8.92E-16	0.208	0.203				*CDK18*	TSS1500
cg18838941	10	15,376,939	1.18E-18	0.208	0.212				*FAM171A1*	Body
cg21117808	1	9,887,936	3.71E-13	0.208	0.195					

cg26455386	17	16,999,983	2.93E-11	0.208	0.204				*MPRIP*	Body
cg20943039	15	65,186,445	0.000119	0.208	0.182					
cg07586272	11	94,134,580	0.014769	0.208	0.157				*GPR83*	1stExon
cg09155774	9	96,723,033	0.000808	0.208	0.210					
cg16934685	7	143,060,037	0.016453	0.208	0.145				*FAM131B*	TSS1500
cg15999165	5	102,201,421	0.000593	0.208	0.213				*PAM*	TSS200
cg01152019	2	177,015,044	0.012647	0.208	0.178				*HOXD4*	TSS1500
cg14985891	1	116,270,626	4.83E-11	0.208	0.192				*CASQ2*	Body
cg16169475	6	14,407,532	0.000503	0.208	0.226					
cg16268769	1	156,890,782	5.38E-06	0.208	0.154				*C1orf92*	Body
cg18303896	11	75,922,637	0.000254	0.208	0.200					
cg07867517	16	75,520,165	0.000169	0.208	0.189				*CHST6*	5'UTR
cg18289581	14	23,830,974	0.0004	0.208	0.241				*EFS*	Body
cg19206146	6	28,661,759	7.05E-05	0.208	0.219					
cg01142776	2	228,582,816	0.022166	0.208	0.165				*SLC19A3*	TSS200
cg12036303	6	30,652,907	0.000104	0.208	0.209				*KIAA1949*	Body
cg18592365	1	79,472,536	1.75E-06	0.208	0.204				*ELTD1*	TSS200
cg22842797	17	48,912,621	1.03E-06	0.208	0.196				*WFIKKN2*	1stExon
cg24414325	12	56,414,442	5.5E-09	0.208	0.196				*IKZF4*	TSS1500
cg26334209	10	13,726,634	0.002863	0.208	0.196				*FRMD4A*	Body
cg03215181	4	122,873,487	0.011298	0.208	0.188				*TRPC3*	TSS1500
cg14415160	1	47,010,105	0.003655	0.208	0.260					
cg15894581	15	63,889,406	4.43E-12	0.208	0.190				*FBXL22*	TSS200
cg18647268	1	39,735,142	9.93E-09	0.208	0.202				*MACF1*	Body
cg00145079	15	68,112,888	0.000314	0.208	0.241					
cg02708705	17	74,264,300	4.62E-08	0.208	0.220				*FAM100B*	Body
cg03512098	11	2,730,129	1.53E-11	0.208	0.193				*KCNQ1*	Body
cg23287005	8	141,588,165	2.22E-08	0.208	0.222				*EIF2C2*	Body
cg02019444	21	35,016,787	2.07E-11	0.208	0.182				*ITSN1*	5'UTR
cg09333471	7	24,796,355	0.009606	0.208	0.126				*DFNA5*	5'UTR
cg09838217	5	179,770,299	7.02E-06	0.208	0.195				*GFPT2*	Body
cg03996195	18	55,095,229	0.002023	0.208	0.261					
cg17453840	15	83,317,526	1.08E-07	0.208	0.240				*CPEB1*	TSS1500
cg21241889	9	123,690,658	1.57E-06	0.208	0.148				*TRAF1*	TSS1500

cg00990769	8	98,956,478	1.83E-09	0.208	0.205				*MATN2*	Body
cg07461072	16	28,073,940	2.69E-05	0.208	0.202				*GSG1L*	Body
cg08166272	2	227,663,151	0.017692	0.208	0.188				*IRS1*	1stExon
cg13597013	17	78,922,527	2.49E-15	0.208	0.187				*RPTOR*	Body
cg22804000	7	24,797,691	4.4E-08	0.208	0.178				*DFNA5*	TSS1500
cg25947489	8	95,246,814	0.031444	0.208	0.308					
cg02245378	2	223,161,771	0.000907	0.208	0.157	3.81E-17	0.178		*PAX3*	Body
cg16272030	7	138,868,118	3.42E-10	0.208	0.217				*TTC26*	Body
cg24729839	1	225,955,869	2.52E-09	0.208	0.192					
cg02042623	12	104,227,548	1.03E-14	0.208	0.205				*NT5DC3*	Body
cg14011845	17	59,533,635	3.67E-10	0.208	0.183				*TBX4*	TSS200
cg11599830	12	49,504,967	9.29E-05	0.208	0.210				*LMBR1L*	TSS1500
cg19250799	1	47,910,456	0.008427	0.208	0.214					
cg21988119	19	17,958,937	9.89E-05	0.208	0.145				*JAK3*	TSS200
cg13935437	1	19,600,673	4.09E-05	0.208	0.182				*AKR7L*	TSS200
cg15055817	3	186,079,142	0.000854	0.208	0.166				*DGKG*	5'UTR
cg18927901	6	36,238,043	4.08E-06	0.208	0.180				*PNPLA1*	5'UTR
cg03305955	12	54,417,033	1.99E-09	0.208	0.169				*HOXC4*	5'UTR
cg05477823	17	59,481,085	6.58E-06	0.208	0.199				*TBX2*	Body
cg09731073	16	29,262,594	0.001941	0.208	0.136					
cg24749089	2	153,322,823	1.43E-05	0.208	0.197				*FMNL2*	Body
cg02406098	3	32,911,355	1.66E-17	0.208	0.205				*TRIM71*	Body
cg05670898	16	66,460,448	0.000303	0.208	0.185				*BEAN*	TSS1500
cg20200460	10	130,165,501	0.000165	0.208	0.186					
cg02026535	19	7,580,217	1.49E-09	0.208	0.188				*ZNF358*	TSS1500
cg14543255	17	8,905,775	0.039018	0.208	0.145					
cg23351327	7	151,433,178	1.17E-08	0.208	0.161				*PRKAG2*	Body
cg00541683	1	32,041,933	4.24E-05	0.208	0.167				*TINAGL1*	TSS200
cg01978213	4	41,866,309	3.86E-08	0.208	0.210					
cg23621418	10	75,670,653	0.024675	0.208	0.131				*PLAU*	TSS1500
cg18231690	15	85,294,586	2.98E-12	0.208	0.221				*ZNF592*	5'UTR
cg25375764	6	24,494,665	0.000872	0.208	0.136	4.25E-21	0.133		*ALDH5A1*	TSS1500
cg05130115	16	11,327,053	2.69E-06	0.208	0.222					
cg08203788	7	25,900,279	2.32E-06	0.208	0.185					

cg08244301	19	17,610,751	7.34E-06	0.208	0.221				*SLC27A1*	Body
cg00607755	2	42,274,082	6.61E-06	0.208	0.155				*PKDCC*	TSS1500
cg06237151	10	46,123,153	1.38E-05	0.208	0.231				*ANUBL1*	Body
cg06936231	4	176,626,819	0.000464	0.208	0.238				*GPM6A*	Body
cg22907295	11	84,659,234	3.49E-05	0.208	0.108				*DLG2*	Body
cg13305415	5	141,697,464	3.28E-07	0.208	0.208				*SPRY4*	Body
cg22117805	11	36,422,455	2.98E-08	0.208	0.171				*PRR5L*	5'UTR
cg06488655	15	96,903,665	1.65E-07	0.208	0.211					
cg07441278	3	48,256,968	3.68E-09	0.208	0.205					
cg12461113	X	13,587,550	0.000574	0.208	0.249				*EGFL6*	TSS200
cg18763247	2	12,907,913	0.001618	0.208	0.129					
cg17139085	1	36,787,356	1.25E-07	0.208	0.206					
cg23409774	5	175,300,141	0.008681	0.208	0.188				*CPLX2*	5'UTR
cg01427957	1	151,689,979	0.030223	0.208	0.193				*TNRC4*	TSS1500
cg18345386	3	62,363,466	0.001764	0.208	0.201					
cg18460175	19	36,132,561	0.001769	0.208	0.253				*ETV2*	TSS200
cg25309292	13	113,613,574	0.000542	0.208	0.140					
cg26434983	15	89,952,904	1.98E-06	0.208	0.183					
cg23700513	6	29,856,305	0.002425	0.208	0.213				*HLA-H*	Body
cg04340651	15	93,617,080	1.64E-08	0.208	0.173				*RGMA*	1stExon
cg09091185	16	936,993	1.39E-09	0.208	0.181				*LMF1*	Body
cg22487391	1	95,437,072	2.98E-14	0.208	0.217					
cg23501051	11	19,736,912	4.91E-06	0.208	0.182				*LOC100126784*	TSS1500
cg27223805	11	2,889,616	1.05E-13	0.208	0.213					
cg18724565	7	5,633,068	0.000506	0.208	0.210				*FSCN1*	1stExon
cg01753788	8	145,024,929	2.46E-06	0.208	0.229				*PLEC1*	5'UTR
cg26006951	5	1,295,988	8.51E-06	0.208	0.173				*TERT*	TSS1500
cg04573398	11	86,086,005	6.54E-09	0.208	0.203				*CCDC81*	1stExon
cg04999691	7	150,027,050	2.68E-12	0.208	0.240	4.02E-22	0.105		*C7orf29*	5'UTR
cg07804276	2	219,856,889	0.000447	0.208	0.206				*CRYBA2*	Body
cg12615761	18	8,608,907	0.03563	0.208	0.232				*RAB12*	TSS1500
cg18690385	1	47,078,966	0.000146	0.208	0.227				*MOBKL2C*	Body
cg26156167	1	161,582,651	0.01089	0.208	0.097					
cg27392559	17	27,410,759	1.97E-07	0.208	0.195				*MYO18A*	Body

cg06893273	17	46,604,774	3.63E-06	0.208	0.212					
cg10838925	3	138,666,318	0.001728	0.208	0.186				*FOXL2*	TSS1500
cg11869862	15	37,399,798	0.00012	0.208	0.225					
cg01965636	14	90,237,942	0.004417	0.208	0.239					
cg06536333	6	41,339,977	0.002853	0.208	0.178					
cg11551879	12	111,620,258	3.81E-12	0.208	0.188				*CUX2*	Body
cg10476773	5	177,412,688	2.64E-06	0.208	0.199					
cg11812015	2	85,482,129	2.51E-13	0.208	0.197				*TCF7L1*	Body
cg26391794	13	49,323,899	0.002518	0.208	0.176					
cg13689996	17	71,810,497	0.001198	0.208	0.256				*C17orf54*	Body
cg14624731	2	121,436,004	1.49E-10	0.208	0.239					
cg03893150	1	6,526,325	9.73E-05	0.208	0.208				*TNFRSF25*	TSS200
cg04387597	17	72,443,179	3.81E-10	0.208	0.194				*GPRC5C*	3'UTR
cg22778120	22	45,094,531	7.57E-07	0.208	0.192				*PRR5*	5'UTR
cg26899598	15	91,429,088	7.48E-06	0.208	0.214				*FES*	Body
cg16590319	1	109,805,584	4.49E-06	0.208	0.236				*CELSR2*	Body
cg22875872	1	44,031,756	5.76E-06	0.208	0.205				*PTPRF*	Body
cg25550823	2	190,192,866	0.01974	0.208	0.266					
cg06781608	7	157,361,040	4.43E-09	0.208	0.196				*PTPRN2*	Body
cg22142254	15	29,228,305	4.9E-17	0.208	0.202				*APBA2*	5'UTR
cg23150604	2	85,732,147	2.85E-09	0.208	0.206					
cg26554592	10	108,924,398	0.004808	0.208	0.210				*SORCS1*	TSS200
cg21087137	12	75,728,469	0.009991	0.208	0.149	2.78E-23	0.202		*GLIPR1L1*	5'UTR
cg15600969	X	119,868,737	9.24E-15	0.208	0.206					
cg24342961	2	97,351,094	4.93E-10	0.208	0.190				*FER1L5*	Body
cg27425327	12	102,270,723	0.001244	0.208	0.141				*DRAM1*	TSS1500
cg05242915	19	1,263,080	2.6E-14	0.208	0.203					
cg09937500	2	208,175,271	6.61E-09	0.208	0.204					
cg01018477	5	172,658,226	6.96E-06	0.208	0.189					
cg04543233	20	21,689,105	0.006848	0.208	0.226				*PAX1*	Body
cg23072823	3	14,445,421	0.001065	0.208	0.177				*SLC6A6*	5'UTR
cg00679520	12	63,133,578	1.42E-07	0.208	0.207				*PPM1H*	Body
cg08829024	16	2,879,393	2.86E-12	0.208	0.196				*ZG16B*	TSS1500
cg09954698	14	74,705,733	0.000616	0.208	0.225				*VSX2*	TSS1500

cg23991636	7	23,507,657	0.000194	0.208	0.205				*IGF2BP3*	Body
cg01602730	1	153,671,011	0.000165	0.208	0.221					
cg14025556	1	200,011,411	2.35E-06	0.208	0.231				*NR5A2*	Body
cg17054386	2	219,855,054	3.2E-08	0.208	0.178				*CRYBA2*	Body
cg03280245	11	57,242,445	5.36E-07	0.208	0.188				*RTN4RL2*	Body
cg09050832	8	1,365,175	0.019131	0.208	0.203					
cg10930308	6	30,039,476	0.003945	0.208	0.216				*RNF39*	Body
cg14613470	5	137,071,799	1.51E-05	0.208	0.096				*KLHL3*	TSS200
cg00741624	14	93,897,389	0.004316	0.208	0.167				*KIAA1409*	5'UTR
cg01853561	21	38,120,466	0.002128	0.208	0.122				*SIM2*	3'UTR
cg16248515	3	156,840,234	7.03E-07	0.208	0.202					
cg18082788	6	149,806,339	7.73E-08	0.208	0.201				*ZC3H12D*	TSS200
cg19716643	14	52,735,026	0.023384	0.208	0.138				*PTGDR*	1stExon
cg24332002	1	22,228,582	2.63E-12	0.208	0.203				*HSPG2*	Body
cg09502809	1	32,248,172	1.64E-12	0.208	0.208					
cg15377283	8	55,371,369	0.006227	0.208	0.200				*SOX17*	Body
cg15416279	5	3,606,795	0.007825	0.208	0.329					
cg04101194	4	25,656,865	0.005503	0.208	0.172				*SLC34A2*	TSS1500
cg15120525	3	188,108,233	0.000357	0.208	0.205				*LPP*	5'UTR
cg19954218	X	102,983,556	4.69E-05	0.208	0.173				*GLRA4*	TSS200
cg08241465	16	57,924,256	9.78E-13	0.208	0.191				*CNGB1*	Body
cg08771171	5	138,211,084	1.01E-05	0.208	0.192				*CTNNA1*	Body
cg14485643	9	134,484,581	0.029163	0.208	0.284				*RAPGEF1*	Body
cg19509663	4	7,863,560	1.52E-11	0.208	0.191				*AFAP1*	Body
cg02689489	10	31,423,315	3.14E-05	0.208	0.220					
cg14110969	12	27,236,884	1.75E-11	0.208	0.214				*C12orf71*	TSS1500
cg21838979	2	106,681,945	6.44E-05	0.208	0.183				*C2orf40*	TSS200
cg01776278	7	149,472,049	4.4E-15	0.208	0.202				*SSPO*	TSS1500
cg08834922	17	41,003,236	1.06E-06	0.208	0.241				*AOC3*	1stExon
cg11118413	3	128,292,699	9.25E-06	0.208	0.222				*C3orf27*	5'UTR
cg24027397	3	181,437,712	0.027784	0.208	0.286				*SOX2OT*	Body
cg20321801	11	2,404,384	1.77E-31	0.208	0.219				*CD81*	Body
cg00590029	12	114,838,618	0.001234	0.208	0.225				*TBX5*	Body
cg20287434	17	79,958,592	1.12E-05	0.208	0.209				*ASPSCR1*	Body

cg11574184	8	145,755,264	0.01494	0.208	0.286				*MGC70857*	TSS1500
cg16874347	2	86,450,216	1.08E-08	0.208	0.197				*REEP1*	Body
cg20338426	11	86,188,827	1.62E-08	0.208	0.200				*ME3*	Body
cg26486175	X	18,658,942	3.76E-06	0.208	0.184				*CDKL5*	Body
cg03761894	X	71,527,796	0.026272	0.207	0.099				*CITED1*	TSS1500
cg09115473	5	14,440,991	9.63E-05	0.207	0.182				*TRIO*	Body
cg10741308	10	97,175,272	1.89E-08	0.207	0.213				*SORBS1*	Body
cg27642470	6	117,802,710	0.000469	0.207	0.204				*DCBLD1*	TSS1500
cg10454937	9	96,715,047	0.021483	0.207	0.299				*BARX1*	Body
cg04479757	22	26,951,319	0.004705	0.207	0.222				*MIR548J*	TSS200
cg04597449	5	94,955,575	0.006696	0.207	0.160	0.0000208	0.096		*GPR150*	TSS1500
cg17770096	12	121,078,022	2.74E-05	0.207	0.176				*CABP1*	TSS1500
cg19711579	2	182,545,562	0.007694	0.207	0.118				*NEUROD1*	TSS200
cg22708902	8	23,100,981	1.26E-09	0.207	0.184				*CHMP7*	TSS200
cg02316596	11	120,434,979	0.030534	0.207	0.141					
cg06971092	17	35,291,286	0.000316	0.207	0.206					
cg22377389	13	20,805,679	3.23E-05	0.207	0.166	0.0000206	0.051		*GJB6*	5'UTR
cg26891237	6	36,966,223	0.008821	0.207	0.194			Yes		
cg01943874	20	44,746,944	0.000182	0.207	0.255				*CD40*	1stExon
cg12298688	2	172,946,691	6E-05	0.207	0.165					
cg14862395	20	44,879,700	0.01829	0.207	0.223				*CDH22*	1stExon
cg02851047	16	2,132,723	7.86E-07	0.207	0.200				*TSC2*	Body
cg16081096	3	12,980,202	5.01E-10	0.207	0.198				*IQSEC1*	Body
cg19839325	19	18,761,132	2.04E-07	0.207	0.223				*KLHL26*	Body
cg00156230	7	45,073,692	0.021935	0.207	0.258				*CCM2*	Body
cg02068015	22	17,848,581	1.77E-13	0.207	0.202					
cg04939026	7	25,898,446	0.001689	0.207	0.199					
cg21206949	14	94,595,582	0.002476	0.207	0.277				*IFI27L2*	Body
cg02389040	6	31,650,786	2.68E-09	0.207	0.208					
cg08827001	11	111,384,048	0.001991	0.207	0.225				*BTG4*	TSS1500
cg24788090	8	74,332,815	0.000551	0.207	0.165				*STAU2*	3'UTR
cg03034070	2	190,044,841	0.000889	0.207	0.218				*COL5A2*	TSS1500
cg17436460	10	17,028,480	1.01E-08	0.207	0.202				*CUBN*	Body
cg24416190	4	186,007,379	9.05E-10	0.207	0.185					

cg04287375	10	102,490,920	0.000281	0.207	0.122					
cg11065015	20	327,170	9.23E-07	0.207	0.123				*NRSN2*	TSS200
cg14524936	11	20,627,597	0.021419	0.207	0.224				*SLC6A5*	Body
cg18447972	17	7,036,934	0.001388	0.207	0.203					
cg23361659	2	28,914,775	5.03E-10	0.207	0.197					
cg06970290	11	64,951,536	5.05E-08	0.207	0.193				*CAPN1*	Body
cg07638450	7	101,796,416	5.83E-05	0.207	0.183				*CUX1*	Body
cg26973488	16	67,450,606	0.032451	0.207	0.144				*ZDHHC1*	TSS1500
cg12108730	16	69,141,258	0.03135	0.207	0.221				*HAS3*	5'UTR
cg20050826	12	52,995,295	2.48E-05	0.207	0.161	1.41E-19	0.199		*KRT72*	1stExon
cg23072008	19	38,916,738	7.12E-07	0.207	0.219				*RASGRP4*	1stExon
cg09234582	6	33,048,286	0.035356	0.207	-0.001				*HLA-DPB1*	Body
cg15288618	3	139,257,775	5.88E-05	0.207	0.205				*RBP1*	Body
cg01295034	16	48,576,092	1.92E-15	0.207	0.189				*N4BP1*	3'UTR
cg06888460	11	18,477,303	1.99E-05	0.207	0.164				*LDHAL6A*	TSS200
cg07377446	19	39,754,498	0.01104	0.207	0.277					
cg11525285	14	74,706,336	0.008504	0.207	0.241	9.74E-18	0.207		*VSX2*	1stExon
cg17263206	5	154,071,247	1.81E-06	0.207	0.194					
cg18342462	11	111,385,659	0.00222	0.207	0.153				*C11orf88*	1stExon
cg20400838	7	21,208,923	4.01E-07	0.207	0.217					
cg26306869	1	153,522,973	0.000113	0.207	0.238				*S100A3*	TSS1500
cg25935014	12	103,889,042	1.05E-05	0.207	0.193				*C12orf42*	5'UTR
cg11363230	6	29,796,351	0.022068	0.207	0.251				*HLA-G*	Body
cg16660891	16	84,828,913	3.61E-15	0.207	0.184					
cg22623649	8	99,179,021	6.46E-15	0.207	0.217					
cg08167110	4	152,543,520	1.33E-08	0.207	0.190				*FAM160A1*	Body
cg10236603	4	85,246,175	7.43E-08	0.207	0.181					
cg24041917	1	119,535,986	0.000543	0.207	0.231					
cg12927641	6	109,611,667	1.25E-10	0.207	0.193					
cg23890008	11	20,229,545	0.028251	0.207	0.192					
cg02485328	5	158,522,603	0.007971	0.207	0.183				*EBF1*	Body
cg06759629	4	25,090,198	0.012089	0.207	0.231					
cg08736225	2	18,654,908	4.59E-08	0.207	0.186					
cg20650914	13	44,627,134	3.17E-10	0.207	0.163					

cg24588381	13	28,551,145	7.71E-07	0.207	0.193					
cg14141919	2	10,572,524	0.0119	0.207	0.182					
cg17785786	4	166,414,666	0.002782	0.207	0.278				*CPE*	Body
cg00043510	1	235,814,134	0.049293	0.207	0.127				*GNG4*	TSS1500
cg03857664	5	171,864,134	7.24E-09	0.207	0.213				*SH3PXD2B*	Body
cg26907472	16	69,006,828	9.2E-12	0.207	0.199				*TMCO7*	Body
cg02603022	X	39,547,825	0.003596	0.207	0.132					
cg06246094	15	42,174,085	0.000132	0.207	0.195				*SPTBN5*	Body
cg14548259	12	116,946,214	0.031976	0.207	0.189					
cg06616729	8	55,379,248	0.044026	0.207	0.274					
cg10975001	1	33,627,155	1.1E-14	0.207	0.211				*TRIM62*	Body
cg14750757	3	127,506,604	2.12E-09	0.207	0.205				*MGLL*	Body
cg15473084	5	50,265,600	0.007227	0.207	0.198					
cg24674368	14	97,879,637	0.000774	0.207	0.254					
cg07241090	12	124,864,594	4.5E-11	0.207	0.210				*NCOR2*	Body
cg08679180	11	73,034,459	7.84E-07	0.207	0.205				*ARHGEF17*	Body
cg07494691	22	44,395,110	7.2E-16	0.207	0.209				*PARVB*	TSS200
cg19796532	12	50,426,901	3.34E-06	0.207	0.206					
cg23820828	8	99,959,602	3.08E-07	0.207	0.197				*OSR2*	5'UTR
cg23729107	17	6,679,264	0.037836	0.207	0.300				*FBXO39*	TSS1500
cg06324785	22	25,960,219	3.38E-05	0.207	0.197				*ADRBK2*	TSS1500
cg09955705	8	30,253,570	0.001137	0.207	0.223				*RBPMS*	Body
cg14996263	12	7,046,277	6.95E-07	0.207	0.171				*ATN1*	Body
cg20533899	11	76,381,105	0.000005	0.207	0.208				*LRRC32*	5'UTR
cg22478317	2	110,271,930	0.001161	0.207	0.191					
cg24375215	17	72,462,559	2.51E-08	0.207	0.186				*CD300A*	5'UTR
cg00568910	1	43,429,380	1.16E-08	0.207	0.201					
cg01236063	11	16,632,088	3.67E-07	0.207	0.192					
cg18526607	5	54,519,307	5.09E-05	0.207	0.190					
cg00039326	1	36,615,845	4.11E-17	0.207	0.202				*TRAPPC3*	TSS1500
cg08178072	2	45,164,495	1.33E-06	0.207	0.205					
cg08182171	11	130,291,156	2.5E-09	0.207	0.179				*ADAMTS8*	Body
cg13883479	9	574,312	7.15E-15	0.207	0.197				*KANK1*	5'UTR
cg23007325	8	10,924,633	9.57E-08	0.207	0.195				*XKR6*	Body

cg26868345	3	127,912,642	7.54E-15	0.207	0.199				*EEFSEC*	Body
cg09973986	12	58,135,973	0.008862	0.207	0.258				*AGAP2*	TSS200
cg20282303	1	236,924,392	9.81E-11	0.207	0.216				*ACTN2*	Body
cg18852692	11	58,830,191	4.29E-08	0.207	0.200					
cg23330450	6	31,548,285	1.96E-06	0.207	0.196					
cg25036527	22	36,123,779	8.4E-05	0.207	0.225				*APOL5*	Body
cg07606495	19	13,993,318	3.86E-11	0.207	0.196				*C19orf57*	3'UTR
cg13492337	8	52,722,140	2.33E-09	0.207	0.192				*PXDNL*	TSS200
cg05674437	3	196,694,153	2.02E-12	0.207	0.179				*PIGZ*	5'UTR
cg16787652	6	29,759,955	0.011726	0.207	0.254				*HCG4*	Body
cg00407245	19	55,598,874	6.88E-05	0.207	0.192				*EPS8L1*	Body
cg00603097	18	72,125,359	0.002257	0.207	0.180				*FAM69C*	TSS1500
cg03877174	17	4,923,126	2.69E-08	0.207	0.206				*KIF1C*	Body
cg09229893	14	54,423,811	0.002047	0.207	0.136				*BMP4*	TSS1500
cg25679766	6	28,558,113	2.91E-06	0.207	0.206					
cg11935147	1	145,075,831	5.78E-06	0.207	0.151				*PDE4DIP*	1stExon
cg08477574	19	2,219,369	2.91E-08	0.207	0.188				*DOT1L*	Body
cg13445230	9	89,422,493	5.87E-07	0.207	0.197					
cg01758314	17	6,797,034	0.00011	0.207	0.219				*ALOX12P2*	Body
cg25016544	5	72,511,412	0.019065	0.207	0.146					
cg02013527	5	88,426,579	0.00128	0.207	0.140					
cg11715858	7	1,267,228	0.001786	0.207	0.228					
cg21547371	3	52,869,521	6.12E-09	0.207	0.216				*MUSTN1*	TSS1500
cg24706115	12	110,252,831	1.99E-05	0.207	0.188				*TRPV4*	5'UTR
cg20732198	10	35,894,310	5.11E-05	0.207	0.211				*GJD4*	TSS200
cg24335310	4	85,284,492	0.001642	0.207	0.208					
cg05155319	4	109,355,164	0.009374	0.207	0.152					
cg07405021	5	115,152,019	0.007929	0.207	0.097				*CDO1*	1stExon
cg17701159	8	18,705,777	6.6E-11	0.207	0.191				*PSD3*	Body
cg21037008	6	32,119,939	0.012025	0.207	0.216				*PRRT1*	TSS1500
cg23340218	3	23,653,462	5.83E-08	0.207	0.226					
cg23558002	13	28,553,501	0.047323	0.207	0.265				*PRHOXNB*	Body
cg23893332	14	94,576,048	0.010313	0.207	0.237				*IFI27*	TSS1500
cg11299854	5	132,083,184	0.000878	0.207	0.192				*CCNI2*	1stExon

cg01217071	2	201,450,323	0.01038	0.207	0.188				*AOX1*	TSS1500
cg00920572	12	54,349,349	0.00244	0.207	0.234				*HOXC12*	Body
cg19988181	10	73,478,985	9.33E-21	0.207	0.209				*C10orf105*	5'UTR
cg24399122	6	70,992,907	0.001383	0.207	0.218				*COL9A1*	Body
cg24834436	1	54,941,132	8.88E-07	0.207	0.184					
cg25526039	6	107,813,291	0.002004	0.207	0.107				*SOBP*	Body
cg05160181	1	151,694,281	0.005347	0.207	0.219				*C1orf230*	Body
cg02287693	22	46,276,479	7.92E-07	0.207	0.193					
cg02642565	16	4,579,402	3.09E-12	0.207	0.202				*C16orf5*	5'UTR
cg07052737	2	26,224,428	5.68E-12	0.207	0.211					
cg04071779	7	30,517,627	0.039206	0.207	0.104				*NOD1*	5'UTR
cg25478114	6	19,328,477	7.85E-12	0.207	0.171					
cg03427330	13	111,465,028	0.000176	0.207	0.160					
cg21773142	7	54,612,709	0.037545	0.207	0.181				*VSTM2A*	Body
cg04430911	1	36,914,349	1.22E-06	0.207	0.197				*OSCP1*	Body
cg06779248	7	95,458,266	3.55E-05	0.207	0.185				*DYNC1I1*	Body
cg11148581	12	123,998,115	5.31E-11	0.207	0.193				*RILPL1*	Body
cg17545182	1	247,694,041	0.000888	0.207	0.197				*LOC148824*	Body
cg20964304	11	125,040,416	1.01E-07	0.207	0.182				*PKNOX2*	5'UTR
cg22281697	7	96,642,037	0.029428	0.207	0.308				*DLX6AS*	Body
cg04902302	2	175,594,739	0.003844	0.207	0.163					
cg14041855	8	1,068,250	0.000934	0.207	0.226					
cg24027477	8	53,883,763	5.64E-14	0.207	0.204					
cg27172287	22	46,974,976	0.004574	0.207	0.177					
cg09644157	12	124,896,212	1.21E-09	0.207	0.187				*NCOR2*	Body
cg00937742	2	133,429,299	4.26E-05	0.207	0.218				*LYPD1*	TSS1500
cg01641620	22	50,609,803	0.007762	0.207	0.088				*PANX2*	Body
cg02047051	3	12,792,283	0.000496	0.207	0.161				*TMEM40*	5'UTR
cg02193611	10	23,982,779	0.038251	0.207	0.253				*KIAA1217*	TSS1500
cg02922776	1	212,420,632	2.49E-07	0.207	0.222					
cg04404694	14	37,236,997	0.047331	0.207	0.147				*SLC25A21*	Body
cg08890067	17	77,478,927	0.005576	0.207	0.214				*HRNBP3*	TSS1500
cg13465852	17	27,942,115	0.00206	0.207	0.194				*CORO6*	3'UTR
cg23252784	X	44,203,803	5.28E-07	0.207	0.184				*EFHC2*	TSS1500

cg09025393	1	18,438,784	0.000971	0.207	0.165				*IGSF21*	Body
cg04169243	14	76,200,552	1.94E-19	0.207	0.205				*TTLL5*	Body
cg08453458	4	187,491,428	0.030822	0.207	0.114					
cg08576476	16	84,231,032	8.28E-06	0.207	0.184					
cg10606029	5	37,952,598	8.36E-07	0.207	0.210					
cg11967111	4	24,105,842	0.002925	0.207	0.214					
cg18767735	1	54,200,420	0.001994	0.207	0.190				*GLIS1*	TSS1500
cg24132991	4	186,560,360	5.86E-06	0.207	0.178				*SORBS2*	Body
cg24406619	1	53,611,984	1.43E-09	0.207	0.177					
cg24710480	6	29,717,136	0.036762	0.207	0.171				*LOC285830*	TSS1500
cg27074174	6	101,847,318	3.46E-05	0.207	0.192				*GRIK2*	Body
cg03651478	1	159,893,671	0.01792	0.207	0.221				*TAGLN2*	5'UTR
cg00851389	16	56,669,681	6.41E-07	0.207	0.190					
cg01838004	9	100,070,007	0.003494	0.207	0.185				*KIAA1529*	Body
cg27256309	19	49,220,485	8.56E-08	0.207	0.172	5.66E-18	0.158		*MAMSTR*	TSS1500
cg07207367	16	67,978,216	1.98E-09	0.207	0.185				*SLC12A4*	3'UTR
cg14312114	17	80,571,580	0.000337	0.207	0.186					
cg01007828	16	62,070,306	0.008253	0.207	0.117				*CDH8*	TSS1500
cg08151370	3	43,073,152	6.2E-14	0.207	0.209				*FAM198A*	Body
cg04522045	2	172,958,324	0.001311	0.207	0.204					
cg25210835	1	110,254,828	3.32E-06	0.207	0.207				*GSTM5*	TSS200
cg25378181	17	79,391,490	3.54E-07	0.207	0.210				*BAHCC1*	Body
cg26839356	15	99,603,018	7.59E-08	0.207	0.158					
cg05256204	20	281,440	5.31E-08	0.207	0.176					
cg08996805	3	11,010,256	2.77E-06	0.207	0.216					
cg10521706	2	19,556,586	3.4E-06	0.207	0.201			Yes	*OSR1*	5'UTR
cg12627726	1	41,290,409	3.08E-11	0.207	0.197				*KCNQ4*	Body
cg13891181	4	41,869,086	0.01585	0.207	0.225					
cg14893857	14	102,554,969	2.74E-06	0.207	0.197				*HSP90AA1*	TSS1500
cg22233890	11	36,477,229	9.66E-12	0.207	0.205				*PRR5L*	Body
cg02897366	1	201,425,990	1.9E-06	0.207	0.198					
cg05325182	19	13,915,751	3.81E-20	0.207	0.195				*ZSWIM4*	Body
cg18435832	15	45,671,155	4E-05	0.207	0.192				*LOC145663*	Body
cg23672278	1	36,787,678	9.82E-11	0.207	0.202				*FAM176B*	3'UTR

cg00179196	16	117,934	7.24E-61	0.207	0.218				*RHBDF1*	5'UTR
cg08335662	14	19,614,556	2.52E-09	0.207	0.166					
cg16714755	1	149,719,243	0.001024	0.207	0.193					
cg24064055	8	145,104,176	0.005608	0.207	0.181					
cg24420717	17	42,426,506	2.03E-10	0.207	0.211				*GRN*	5'UTR
cg27111150	10	97,183,248	4.58E-16	0.207	0.209				*SORBS1*	Body
cg01527777	11	71,956,145	5.91E-06	0.207	0.182				*PHOX2A*	TSS1500
cg01974478	7	4,779,312	3.93E-16	0.207	0.203				*FOXK1*	Body
cg11800620	1	217,311,993	4.53E-06	0.207	0.190				*ESRRG*	TSS1500
cg14646893	6	143,592,425	3.24E-10	0.207	0.237				*AIG1*	Body
cg17097923	17	6,679,541	0.004636	0.207	0.150				*FBXO39*	TSS200
cg18593374	10	126,302,546	1.38E-07	0.207	0.176				*LHPP*	3'UTR
cg24354624	19	40,732,728	0.004526	0.207	0.111				*CNTD2*	TSS200
cg24701772	1	51,888,961	1.13E-13	0.207	0.211				*EPS15*	TSS1500
cg27252766	2	42,229,092	5.23E-08	0.207	0.179					
cg04606076	13	80,934,105	0.00757	0.207	0.232					
cg13704086	2	45,177,125	0.013501	0.207	0.129					
cg25335190	6	27,791,899	0.002284	0.207	0.168				*HIST1H4J*	TSS200
cg00366103	7	96,645,019	0.002012	0.207	0.088					
cg01148781	8	99,963,021	3.43E-08	0.207	0.200				*OSR2*	Body
cg11368643	5	140,625,059	0.026436	0.207	0.235	2.14E-21	0.243		*PCDHB15*	TSS200
cg04907257	5	7,395,318	2.22E-08	0.207	0.198	2.76E-09	0.094		*ADCY2*	TSS1500
cg22108360	8	124,704,218	2.56E-08	0.207	0.196				*ANXA13*	Body
cg01080902	19	44,039,870	5.51E-05	0.207	0.187				*ZNF575*	3'UTR
cg01176947	6	10,385,279	6.18E-05	0.207	0.186					
cg04011461	16	89,638,389	2.07E-11	0.207	0.169					
cg13010373	6	86,113,013	1.37E-05	0.207	0.170					
cg24368383	1	1,565,856	8.18E-05	0.207	0.216				*MIB2*	Body
cg21433231	17	34,820,712	0.030778	0.207	0.251					
cg09565310	5	112,541,553	0.029872	0.207	0.245				*MCC*	Body
cg19358493	10	119,300,942	0.000558	0.207	0.170	6.71E-20	0.204		*EMX2*	TSS1500
cg08613577	17	20,687,698	0.000299	0.207	0.189					
cg10333402	7	100,845,647	0.002693	0.207	0.141				*MOGAT3*	TSS1500
cg16047117	22	42,470,063	0.000331	0.207	0.187				*FAM109B*	TSS200

cg19293162	19	2,513,302	4.02E-12	0.206	0.187				*GNG7*	3'UTR
cg26360616	4	128,544,667	0.010447	0.206	0.178					
cg26915924	16	8,807,043	7.35E-13	0.206	0.208				*ABAT*	5'UTR
cg12877723	8	57,358,312	1.04E-05	0.206	0.195				*PENK*	Body
cg03889542	3	44,039,578	0.007895	0.206	0.185					
cg16818395	3	128,274,084	4.7E-05	0.206	0.203					
cg14186963	1	113,051,777	0.00287	0.206	0.199				*WNT2B*	Body
cg03398156	2	220,154,779	9.18E-15	0.206	0.202				*PTPRN*	3'UTR
cg14681511	5	140,805,681	0.003157	0.206	0.239				*PCDHGA4*	Body
cg22229828	1	245,874,647	6.32E-11	0.206	0.192					
cg06342072	13	50,985,639	6.95E-10	0.206	0.196					
cg08899442	5	157,398,818	0.004726	0.206	0.232					
cg10574006	5	123,965,051	8.68E-06	0.206	0.186					
cg14967066	11	314,341	0.008785	0.206	0.075				*IFITM1*	1stExon
cg17176016	6	108,441,207	7.01E-05	0.206	0.197					
cg19956155	5	176,511,416	0.005384	0.206	0.175					
cg23482132	2	153,415,027	1.26E-08	0.206	0.187				*FMNL2*	Body
cg02166842	7	55,179,471	1.75E-12	0.206	0.187				*EGFR*	Body
cg26211349	17	77,404,555	4E-05	0.206	0.167				*HRNBP3*	5'UTR
cg27353308	10	133,796,559	0.002355	0.206	0.187				*BNIP3*	TSS1500
cg27443071	4	190,939,090	0.022381	0.206	0.275					
cg01530283	7	5,085,252	0.011057	0.206	0.213				*RBAK*	TSS1500
cg06300254	10	50,822,752	0.019476	0.206	0.215				*CHAT*	Body
cg18059012	15	44,969,129	0.000346	0.206	0.156				*PATL2*	TSS200
cg19156231	12	52,115,539	6.5E-06	0.206	0.203				*SCN8A*	Body
cg23684410	11	116,897,558	1.1E-06	0.206	0.202				*SIK3*	Body
cg05623526	11	2,548,319	3.08E-07	0.206	0.159				*KCNQ1*	Body
cg11160572	4	169,706,422	6.86E-17	0.206	0.204				*PALLD*	Body
cg01563071	3	131,647,680	2.03E-05	0.206	0.192				*CPNE4*	5'UTR
cg09654247	2	200,524,002	0.001603	0.206	0.212					
cg15448076	12	4,380,872	0.010834	0.206	0.222					
cg16404106	11	86,085,623	1.06E-05	0.206	0.189	3.51E-08	0.115		*CCDC81*	TSS200
cg19918734	11	86,343,931	5.4E-05	0.206	0.197				*ME3*	Body
cg20287790	1	53,359,982	9.14E-11	0.206	0.203				*ZYG11A*	3'UTR

cg00122765	17	29,457,918	1.42E-06	0.206	0.165				*NF1*	Body
cg02754470	15	59,157,857	2.88E-09	0.206	0.191					
cg03455555	15	85,356,531	2.73E-07	0.206	0.204					
cg07428004	7	127,912,372	6.27E-07	0.206	0.219					
cg20567768	8	22,082,066	6.32E-06	0.206	0.213				*PHYHIP*	Body
cg00377915	10	70,631,035	4.61E-05	0.206	0.125				*STOX1*	5'UTR
cg05127924	17	18,647,524	1.29E-09	0.206	0.196	8.1E-12	0.116		*FBXW10*	5'UTR
cg05622577	10	33,710,900	8.04E-09	0.206	0.191					
cg09510752	5	134,526,173	3.61E-11	0.206	0.207					
cg19764489	11	2,730,616	1.15E-08	0.206	0.159				*KCNQ1*	Body
cg08810842	8	22,548,546	0.000149	0.206	0.201				*EGR3*	Body
cg02872373	7	129,418,058	2.3E-06	0.206	0.198					
cg10180297	16	22,827,393	8.06E-10	0.206	0.189				*HS3ST2*	Body
cg15146515	19	49,250,561	2.3E-05	0.206	0.184				*IZUMO1*	TSS1500
cg24463290	10	114,922,641	1.34E-08	0.206	0.211				*TCF7L2*	Body
cg10261191	11	125,364,744	2.25E-05	0.206	0.202	2.48E-15	0.162		*FEZ1*	5'UTR
cg10513330	2	28,829,939	7.94E-09	0.206	0.216				*PLB1*	Body
cg12438547	13	100,642,971	6.86E-05	0.206	0.185					
cg18735956	6	34,113,908	0.000612	0.206	0.227					
cg21563049	22	18,386,798	3.03E-08	0.206	0.179			Yes	*MICAL3*	Body
cg00393837	2	105,470,050	0.012825	0.206	0.205					
cg26077454	14	95,240,500	0.003361	0.206	0.218					
cg02816297	12	56,224,411	0.040566	0.206	0.117				*DNAJC14*	TSS1500
cg11463505	5	1,841,594	0.000741	0.206	0.135					
cg00049664	16	66,613,334	3.62E-05	0.206	0.195				*CMTM2*	TSS200
cg02584537	10	131,569,018	2.03E-15	0.206	0.187					
cg05279738	3	141,560,204	0.005139	0.206	0.205					
cg20180247	6	10,884,140	0.001222	0.206	0.220					
cg26648103	11	66,791,718	1.33E-06	0.206	0.182				*SYT12*	5'UTR
cg14914254	6	29,796,370	0.0002	0.206	0.231				*HLA-G*	Body
cg20029201	11	118,781,813	0.012148	0.206	0.231	5.75E-19	0.167		*BCL9L*	TSS1500
cg20248093	17	77,722,496	4.2E-05	0.206	0.204					
cg24713770	1	20,649,959	1.13E-30	0.206	0.197				*VWA5B1*	Body
cg14644871	7	26,192,756	0.015504	0.206	0.073				*NFE2L3*	Body

cg19715111	2	224,904,683	0.002145	0.206	0.129				*SERPINE2*	TSS1500
cg00116092	1	217,313,044	0.000192	0.206	0.190					
cg04768697	19	15,122,135	0.001261	0.206	0.192				*CCDC105*	1stExon
cg11077681	11	10,715,188	3.62E-05	0.206	0.169				*MRVI1*	1stExon
cg11828163	7	5,531,009	3.71E-18	0.206	0.201				*FBXL18*	Body
cg16072884	10	30,317,625	3.06E-08	0.206	0.206				*KIAA1462*	Body
cg17406359	21	45,773,189	8.76E-06	0.206	0.239				*TRPM2*	TSS1500
cg01414464	5	113,695,858	1.32E-06	0.206	0.229					
cg01579841	6	166,400,494	0.009584	0.206	0.120				*C6orf176*	Body
cg04270489	11	85,827,505	0.000178	0.206	0.194					
cg20411935	1	228,653,095	0.009968	0.206	0.291					
cg22462240	17	76,962,663	9.2E-12	0.206	0.199					
cg27504292	12	124,779,532	0.017807	0.206	0.145				*FAM101A*	5'UTR
cg10036013	7	4,778,839	2.98E-11	0.206	0.210				*FOXK1*	Body
cg12912690	2	239,197,457	2.27E-06	0.206	0.191				*PER2*	TSS1500
cg17953300	11	65,418,265	2.59E-05	0.206	0.156				*SIPA1*	3'UTR
cg01906015	8	63,161,579	0.039458	0.206	0.118				*NKAIN3*	1stExon
cg13398027	18	46,447,840	8.48E-10	0.206	0.212				*SMAD7*	Body
cg18864124	2	227,288,863	0.01631	0.206	0.193					
cg20288617	4	22,239,907	0.001242	0.206	0.167			Yes		
cg13366470	15	81,666,512	8.05E-11	0.206	0.202				*TMC3*	TSS200
cg14157578	9	110,764,967	2.46E-07	0.206	0.205					
cg04064452	2	242,486,462	0.015278	0.206	0.171					
cg00260012	2	31,031,702	9.84E-18	0.206	0.191				*CAPN13*	TSS1500
cg02269978	6	32,847,585	0.003646	0.206	0.255				*PPP1R2P1*	Body
cg14663965	6	108,495,385	5.36E-05	0.206	0.213				*NR2E1*	Body
cg25556330	7	27,279,575	3.32E-07	0.206	0.200					
cg26128383	6	31,239,158	0.011868	0.206	0.183				*HLA-C*	Body
cg15786205	6	108,495,578	7.44E-05	0.206	0.207				*NR2E1*	Body
cg20536364	5	55,790,102	2.1E-10	0.206	0.185					
cg02194717	11	70,415,188	8.27E-08	0.206	0.172				*SHANK2*	Body
cg22700686	1	153,538,764	7.02E-07	0.206	0.201				*S100A2*	TSS1500
cg06339657	5	76,505,932	0.006854	0.206	0.120	7.1E-13	0.174		*PDE8B*	TSS1500
cg08454563	22	31,040,187	5.3E-06	0.206	0.204				*SLC35E4*	Body

cg08794544	11	45,825,518	0.000478	0.206	0.137				*SLC35C1*	TSS200
cg10148591	19	7,197,021	0.000174	0.206	0.231				*INSR*	Body
cg01621716	2	11,636,147	1.01E-05	0.206	0.179					
cg03111114	5	149,886,185	3.16E-09	0.206	0.190				*NDST1*	TSS1500
cg07337793	2	43,038,584	0.016873	0.206	0.117					
cg03173722	4	71,587,640	3.71E-05	0.206	0.217	0.0000256	0.102		*RUFY3*	Body
cg03401297	19	41,025,959	9.74E-06	0.206	0.160				*SPTBN4*	Body
cg07835443	16	89,734,986	7.48E-06	0.206	0.158				*C16orf55*	Body
cg01701634	19	51,876,119	5.58E-06	0.206	0.189				*NKG7*	TSS200
cg26282505	15	90,171,693	1.91E-12	0.206	0.209				*KIF7*	Body
cg00351781	17	17,709,007	1.64E-08	0.206	0.190				*RAI1*	Body
cg00734629	7	128,471,146	2.11E-07	0.206	0.191				*FLNC*	Body
cg04010586	1	36,788,436	2.19E-05	0.206	0.221				*FAM176B*	Body
cg07606234	14	24,551,147	0.004894	0.206	0.091				*NRL*	Body
cg09403249	12	45,444,203	4.41E-05	0.206	0.188				*DBX2*	Body
cg23418510	10	25,240,660	0.000951	0.206	0.215				*PRTFDC1*	Body
cg03117611	12	125,223,679	3.92E-10	0.206	0.185					
cg06637618	1	203,595,048	5.25E-06	0.206	0.170				*ATP2B4*	TSS1500
cg10065736	12	117,440,120	2.04E-13	0.206	0.175				*FBXW8*	Body
cg19260084	12	103,349,055	0.004395	0.206	0.188					
cg27109912	17	38,707,719	0.021028	0.206	0.242					
cg10942097	2	176,947,435	0.00561	0.206	0.209				*EVX2*	Body
cg11380483	2	127,933,992	0.003324	0.206	0.195					
cg13304825	17	79,297,548	0.000373	0.206	0.219				*TMEM105*	5'UTR
cg15270350	17	76,215,886	9.36E-07	0.206	0.167				*BIRC5*	Body
cg26577252	15	99,212,332	0.01784	0.206	0.213				*IGF1R*	Body
cg06126335	7	73,497,616	1.9E-06	0.206	0.195				*LIMK1*	TSS1500
cg07105272	3	186,490,785	1.11E-05	0.206	0.207					
cg09294347	2	1,286,397	0.000163	0.206	0.130				*SNTG2*	Body
cg16158779	11	113,258,455	0.01859	0.206	0.141				*ANKK1*	TSS200
cg23208513	8	101,118,377	0.004346	0.206	0.225				*RGS22*	TSS200
cg24399395	21	34,392,203	0.040657	0.206	0.176					
cg00194126	2	157,186,312	0.000556	0.206	0.193				*NR4A2*	Body
cg00139681	7	106,125,168	6.1E-05	0.206	0.213					

cg07548372	14	24,848,202	3.72E-05	0.206	0.200			Yes	*NFATC4*	3'UTR
cg22306749	X	101,967,085	0.037937	0.206	0.177				*GPRASP2*	TSS1500
cg23045073	1	179,545,458	0.00022	0.206	0.146				*NPHS2*	TSS1500
cg00151271	4	7,872,993	3.5E-07	0.206	0.171				*AFAP1*	Body
cg08153621	19	53,561,441	1.9E-05	0.206	0.197					
cg10443421	16	56,644,991	1.25E-07	0.206	0.211					
cg13858742	1	204,621,504	7.58E-09	0.206	0.184				*LRRN2*	5'UTR
cg25851789	1	217,309,794	8.01E-07	0.206	0.211				*ESRRG*	5'UTR
cg00745835	10	80,778,842	1.15E-10	0.206	0.193				*LOC283050*	Body
cg06027057	14	90,849,959	0.00353	0.206	0.173					
cg08439439	6	149,190,883	2.45E-12	0.206	0.212				*UST*	Body
cg08755224	20	21,001,116	2.47E-09	0.206	0.205					
cg16444607	2	173,061,858	0.000968	0.206	0.173			Yes		
cg09511126	10	100,993,597	0.001291	0.206	0.209				*HPSE2*	Body
cg12559119	17	63,590,575	1.01E-07	0.206	0.196					
cg22886323	15	100,782,544	3.54E-12	0.206	0.210				*ADAMTS17*	Body
cg26127114	11	12,419,483	1.97E-08	0.206	0.193				*PARVA*	Body
cg12041266	11	75,050,147	8.3E-15	0.206	0.205			Yes	*ARRB1*	Body
cg04109661	X	133,680,249	3.27E-09	0.206	0.188				*MGC16121*	Body
cg23877720	7	49,815,117	0.00024	0.206	0.302				*VWC2*	Body
cg25196088	1	7,703,835	1.29E-08	0.206	0.194				*CAMTA1*	Body
cg16603596	12	8,855,125	5.54E-08	0.206	0.209				*RIMKLB*	5'UTR
cg07005055	1	113,052,167	0.004793	0.206	0.225				*WNT2B*	Body
cg13085980	12	94,544,425	0.006043	0.206	0.247				*PLXNC1*	Body
cg00831931	10	114,823,094	1.76E-20	0.206	0.186				*TCF7L2*	Body
cg02912316	1	32,527,924	8.35E-15	0.206	0.202					
cg05124918	12	52,473,462	3.16E-13	0.206	0.185					
cg22341104	1	92,952,261	0.031398	0.206	0.143	8.7E-20	0.162		*GFI1*	TSS1500
cg24126768	17	53,645,286	0.006946	0.206	0.196					
cg25563256	17	7,341,641	0.016631	0.206	0.217				*FGF11*	TSS1500
cg08309529	7	156,797,833	0.027864	0.206	0.203				*MNX1*	3'UTR
cg09193791	11	117,740,722	0.001048	0.206	0.183				*FXYD6*	5'UTR
cg16626322	17	78,439,556	1.08E-05	0.206	0.170					
cg18745629	3	57,163,527	5.22E-14	0.206	0.196				*IL17RD*	Body

cg14245228	9	135,619,345	4.91E-10	0.206	0.191				*C9orf98*	Body
cg16742873	4	13,544,632	2.98E-10	0.206	0.187				*NKX3-2*	Body
cg22728904	7	100,433,983	0.03651	0.206	0.151					
cg24371574	1	230,428,167	9.06E-12	0.206	0.202					
cg25320816	13	114,099,871	7.53E-08	0.206	0.194				*ADPRHL1*	5'UTR
cg25353281	2	219,155,378	0.007689	0.206	0.269				*PNKD*	Body
cg01795122	19	48,828,859	0.017948	0.206	0.184	5.72E-15	0.141		*EMP3*	1stExon
cg00816770	4	144,621,089	0.000163	0.206	0.183				*FREM3*	1stExon
cg13917662	13	100,548,513	9.64E-07	0.206	0.186				*CLYBL*	3'UTR
cg21443659	2	217,745,721	8.08E-07	0.206	0.190					
cg03564201	3	42,948,169	2.36E-06	0.206	0.179				*ZNF662*	5'UTR
cg05951817	17	28,562,142	5.67E-06	0.206	0.206				*SLC6A4*	5'UTR
cg13654588	10	120,354,081	0.013848	0.206	0.126				*PRLHR*	Body
cg14425869	9	98,343,728	6.4E-10	0.206	0.208					
cg21279316	15	64,915,229	3.97E-05	0.206	0.175				*ZNF609*	Body
cg15523463	5	150,804,815	1.06E-14	0.206	0.198					
cg23678594	1	1,712,021	1.48E-06	0.206	0.204					
cg00111335	8	593,194	6.78E-13	0.206	0.168					
cg12747860	6	3,229,027	0.007554	0.206	0.279				*TUBB2B*	TSS1500
cg14468280	20	47,862,976	2.14E-11	0.206	0.220				*ZNFX1*	3'UTR
cg16077226	11	62,156,701	8.64E-13	0.206	0.188				*ASRGL1*	Body
cg17338816	7	2,607,424	0.00014	0.206	0.146				*IQCE*	Body
cg01032119	5	10,857,295	0.025881	0.206	0.174					
cg05324789	10	134,602,475	0.000842	0.206	0.142					
cg06159269	1	8,767,347	0.004339	0.206	0.199				*RERE*	5'UTR
cg07770854	19	4,607,057	0.016173	0.206	0.151					
cg10552473	2	206,525,681	2.27E-07	0.206	0.217					
cg18601426	7	158,381,220	2.63E-05	0.206	0.193	2.9E-22	0.139		*PTPRN2*	TSS1500
cg04656222	17	7,690,529	3.76E-05	0.206	0.221				*DNAH2*	Body
cg08328750	17	39,675,355	0.003874	0.206	0.139				*KRT15*	TSS200
cg13071508	1	24,373,210	0.000129	0.206	0.204					
cg19803052	7	19,813,244	0.002579	0.206	0.154				*TMEM196*	TSS1500
cg21210090	4	15,704,429	0.010308	0.206	0.198				*BST1*	TSS200
cg21893358	11	120,434,880	0.025389	0.206	0.150					

cg22354782	1	115,881,132	0.000305	0.206	0.168				*NGF*	TSS1500
cg00157572	14	60,977,961	0.023144	0.206	0.183				*SIX6*	Body
cg02211449	10	110,671,609	0.018336	0.206	0.257					
cg06629734	6	39,017,116	2.28E-05	0.206	0.211				*GLP1R*	Body
cg12854186	6	32,805,398	3.49E-09	0.206	0.166				*TAP2*	Body
cg17111846	1	51,567,810	3.35E-08	0.206	0.203				*C1orf185*	TSS200
cg20253872	1	110,166,571	2.54E-09	0.206	0.193				*AMPD2*	Body
cg21923770	17	1,183,159	1.67E-05	0.206	0.254				*TUSC5*	1stExon
cg22854985	2	174,082,101	9.35E-05	0.206	0.146				*ZAK*	Body
cg05349542	10	88,127,572	0.000923	0.206	0.172				*GRID1*	TSS1500
cg11329482	12	91,493,337	2.05E-05	0.206	0.206					
cg13053653	2	177,037,631	0.000588	0.206	0.174				*HOXD3*	3'UTR
cg13906792	15	75,199,810	7.51E-07	0.206	0.188				*C15orf17*	TSS1500
cg14063488	4	154,709,878	0.042869	0.206	0.288				*SFRP2*	1stExon
cg17198308	12	6,665,108	0.000363	0.206	0.193				*IFFO1*	1stExon
cg03316237	19	45,738,857	0.019302	0.206	0.144				*EXOC3L2*	TSS1500
cg04153489	8	41,655,983	2.22E-05	0.206	0.160				*ANK1*	TSS1500
cg05106770	4	128,545,193	0.001158	0.206	0.216					
cg17261676	6	26,016,808	1.35E-07	0.206	0.227					
cg17786894	2	65,131,556	4.88E-08	0.206	0.205					
cg02275784	11	85,242,554	0.000107	0.206	0.151				*DLG2*	Body
cg03337093	9	135,463,171	3.8E-05	0.206	0.217				*BARHL1*	Body
cg16886175	7	114,562,063	0.011282	0.206	0.163				*MDFIC*	TSS200
cg24211976	15	60,285,744	0.000333	0.206	0.216					
cg03434029	1	45,274,048	0.00275	0.206	0.255				*TCTEX1D4*	TSS1500
cg11353647	7	158,243,730	8.16E-11	0.206	0.202				*PTPRN2*	Body
cg13814747	15	31,373,178	6.74E-06	0.206	0.179				*TRPM1*	5'UTR
cg20051292	8	101,118,512	0.006133	0.206	0.287				*RGS22*	TSS200
cg04333575	11	6,340,890	0.000414	0.206	0.232				*PRKCDBP*	Body
cg15986413	2	233,352,776	2.01E-08	0.206	0.149				*ECEL1*	TSS1500
cg06615940	1	110,211,023	1.06E-18	0.206	0.198				*GSTM2*	Body
cg11324538	19	47,778,804	7.09E-14	0.206	0.173				*PRR24*	3'UTR
cg14934253	6	108,436,768	0.035354	0.206	0.279					
cg24826644	6	30,457,240	2.82E-06	0.206	0.204				*HLA-E*	TSS200

cg03607638	3	193,788,482	0.013971	0.206	0.118					
cg04961101	20	37,303,484	0.004322	0.206	0.167					
cg06335918	11	62,610,003	0.002453	0.206	0.184					
cg14838970	11	7,533,817	1.73E-05	0.206	0.151				*PPFIBP2*	TSS1500
cg02809707	2	175,191,069	0.016718	0.206	0.219					
cg19842809	14	37,125,997	0.000825	0.206	0.220				*PAX9*	TSS1500
cg20372666	10	71,149,910	3.53E-07	0.206	0.192				*HK1*	Body
cg21644489	15	63,312,170	3.17E-08	0.206	0.192					
cg06706550	22	45,125,480	8.62E-08	0.206	0.208				*PRR5*	Body
cg18939596	14	95,330,880	1.44E-05	0.206	0.201					
cg05053440	1	227,748,442	3.69E-06	0.206	0.195					
cg21116457	18	43,304,007	1.47E-05	0.206	0.235				*SLC14A1*	TSS200
cg04470072	8	87,082,014	0.001695	0.206	0.221				*PSKH2*	TSS200
cg14839558	1	1,874,488	2.15E-18	0.206	0.191					
cg15036894	5	16,539,535	1.02E-06	0.206	0.207				*FAM134B*	Body
cg22537334	15	70,354,691	2.65E-08	0.206	0.190				*TLE3*	Body
cg23674882	2	74,780,272	1.7E-09	0.206	0.211	5.8887E-05	0.061		*LOXL3*	5'UTR
cg01613249	2	54,727,293	1.47E-07	0.206	0.220				*SPTBN1*	5'UTR
cg05630111	1	150,948,030	2.36E-07	0.206	0.199				*LASS2*	TSS1500
cg18827332	12	103,344,506	0.001932	0.206	0.229					
cg03852144	5	95,159,639	0.001919	0.206	0.175	0.0000967	0.066		*GLRX*	TSS1500
cg07277549	7	30,509,141	0.002517	0.206	0.234				*NOD1*	5'UTR
cg02917321	22	38,215,204	0.036348	0.205	0.092					
cg14822490	10	124,909,889	2.34E-05	0.205	0.181				*HMX2*	3'UTR
cg15564098	7	27,281,346	1.37E-14	0.205	0.197				*EVX1*	TSS1500
cg15612257	2	65,085,598	1.11E-16	0.205	0.202					
cg17102205	2	208,091,961	4.12E-10	0.205	0.180					
cg26606589	7	64,403,082	1.39E-06	0.205	0.143					
cg08939394	1	17,614,144	1.16E-08	0.205	0.192					
cg24621972	2	223,160,175	0.000605	0.205	0.207				*PAX3*	Body
cg21184369	8	38,831,148	0.040912	0.205	0.100				*HTRA4*	TSS1500
cg22863744	12	124,942,176	5.05E-05	0.205	0.195			Yes	*NCOR2*	Body
cg17551891	7	1,960,795	8.25E-15	0.205	0.181				*MAD1L1*	Body
cg19096825	13	112,711,941	0.010365	0.205	0.239					

cg21244086	15	45,479,462	5.36E-17	0.205	0.198				*SHF*	Body
cg02573472	5	76,940,356	6.86E-08	0.205	0.209					
cg10040116	17	13,332,667	1.78E-07	0.205	0.218					
cg10058920	20	1,974,738	0.012395	0.205	0.190				*PDYN*	TSS200
cg16799111	8	10,589,359	9.89E-05	0.205	0.187				*SOX7*	TSS1500
cg01039990	X	136,510,831	0.018997	0.205	0.189					
cg05726661	17	7,348,438	2.12E-23	0.205	0.174				*CHRNB1*	1stExon
cg09854734	16	66,613,273	2.66E-05	0.205	0.182				*CMTM2*	TSS200
cg11637968	4	159,732,036	0.002628	0.205	0.150				*FNIP2*	Body
cg00157359	11	66,233,183	0.000306	0.205	0.219				*PELI3*	TSS1500
cg00678709	3	14,584,745	6.88E-09	0.205	0.199				*GRIP2*	TSS1500
cg10690003	7	96,622,311	2.93E-08	0.205	0.199				*DLX6AS*	Body
cg15392413	12	78,603,664	4.98E-05	0.205	0.191				*NAV3*	Body
cg21929564	5	42,757,171	5.89E-09	0.205	0.204				*CCDC152*	5'UTR
cg07466560	7	5,712,269	7.42E-14	0.205	0.204				*RNF216*	Body
cg07944051	15	69,068,216	6.59E-10	0.205	0.196					
cg11095383	5	140,174,887	0.000688	0.205	0.194				*PCDHA1*	Body
cg16064237	12	132,654,984	0.006541	0.205	0.200					
cg17356036	2	240,213,057	0.005423	0.205	0.233				*HDAC4*	Body
cg21570493	1	16,157,388	1.23E-12	0.205	0.202					
cg03468671	14	39,737,849	4.77E-05	0.205	0.204				*CTAGE5*	Body
cg03960066	1	150,255,503	0.011257	0.205	0.214				*C1orf51*	1stExon
cg11005692	4	1,595,294	3.67E-06	0.205	0.160					
cg18202521	1	19,600,702	1.1E-05	0.205	0.184				*AKR7L*	TSS200
cg18639125	2	11,051,728	0.00041	0.205	0.103				*KCNF1*	TSS1500
cg22516643	1	107,682,302	0.006448	0.205	0.218				*NTNG1*	TSS1500
cg07250771	12	70,289,007	1.06E-19	0.205	0.203					
cg10861541	2	210,444,201	2.92E-06	0.205	0.219				*MAP2*	TSS1500
cg13697735	14	65,172,103	1.82E-06	0.205	0.259				*PLEKHG3*	5'UTR
cg24162603	3	127,945,858	1.2E-08	0.205	0.225				*EEFSEC*	Body
cg26277026	12	22,486,029	4.17E-05	0.205	0.183				*ST8SIA1*	Body
cg27037937	15	93,876,630	4.58E-05	0.205	0.221					
cg20245757	17	46,663,759	0.045256	0.205	0.234					
cg10189962	2	175,192,878	5.34E-08	0.205	0.221					

cg13528854	4	15,704,795	0.008001	0.205	0.221				*BST1*	1stExon
cg16530881	17	38,708,399	1.05E-10	0.205	0.180					
cg22531018	7	142,981,779	2.07E-07	0.205	0.141				*TMEM139*	TSS1500
cg26094599	2	219,748,780	0.006709	0.205	0.168				*WNT10A*	Body
cg02710534	5	141,225,619	7.79E-09	0.205	0.213					
cg07044282	1	178,840,137	5.64E-10	0.205	0.208				*ANGPTL1*	1stExon
cg09581226	5	140,857,474	1.31E-08	0.205	0.177				*PCDHGA4*	Body
cg09884423	12	104,697,220	0.003509	0.205	0.128				*TXNRD1*	5'UTR
cg15174623	7	35,494,345	6.46E-07	0.205	0.197					
cg26503653	5	169,064,024	0.009739	0.205	0.215				*DOCK2*	TSS1500
cg26672959	3	65,387,308	1.97E-08	0.205	0.196				*MAGI1*	Body
cg14375111	3	14,165,186	2.01E-08	0.205	0.164				*TMEM43*	TSS1500
cg23745864	18	77,806,288	8.11E-22	0.205	0.202				*C18orf22*	3'UTR
cg01891252	1	50,881,219	0.00063	0.205	0.194					
cg02049629	6	28,662,065	0.003864	0.205	0.231					
cg22653915	8	52,722,023	2.83E-08	0.205	0.172				*PXDNL*	TSS200
cg24074477	22	17,956,455	1.47E-10	0.205	0.189				*CECR2*	TSS200
cg27034935	5	153,873,021	0.007659	0.205	0.148					
cg09643186	14	65,409,452	1.86E-05	0.205	0.185	4.89E-28	0.226		*GPX2*	1stExon
cg18774195	3	170,303,830	0.006193	0.205	0.226				*SLC7A14*	5'UTR
cg01523763	X	129,068,084	0.000294	0.205	0.258					
cg08119553	15	93,616,966	3.36E-05	0.205	0.168				*RGMA*	1stExon
cg19258062	7	152,133,423	0.013849	0.205	0.254				*FABP5L3*	TSS1500
cg01505870	2	85,107,190	0.001708	0.205	0.229				*C2orf89*	Body
cg04865290	3	52,927,548	8.28E-09	0.205	0.199				*TMEM110*	Body
cg07048519	17	15,522,876	4.97E-10	0.205	0.211				*CDRT1*	1stExon
cg08557649	15	76,639,713	0.012574	0.205	0.176					
cg15409133	12	132,900,072	0.012876	0.205	0.248				*GALNT9*	Body
cg24348495	20	62,693,971	1.87E-12	0.205	0.204				*TCEA2*	TSS200
cg10782668	13	28,496,082	0.00724	0.205	0.243				*PDX1*	Body
cg18817990	4	13,543,119	8.33E-06	0.205	0.191				*NKX3-2*	3'UTR
cg16430166	1	232,941,468	0.017969	0.205	0.178				*KIAA1383*	1stExon
cg24146331	10	102,590,225	0.00128	0.205	0.224					
cg00317020	10	60,933,578	1.24E-05	0.205	0.150					

cg03901660	1	221,050,459	8.76E-09	0.205	0.198					
cg07787634	1	241,715,378	2.15E-11	0.205	0.182				*KMO*	Body
cg18124852	17	43,506,540	3.92E-10	0.205	0.180					
cg21886425	14	29,228,476	0.02578	0.205	0.291					
cg22461324	7	155,252,944	0.031334	0.205	0.276				*EN2*	Body
cg09582200	11	117,748,236	2.83E-05	0.205	0.179				*FXYD6*	TSS1500
cg20954179	4	175,138,488	0.002775	0.205	0.237					
cg24090911	5	400,732	1.91E-10	0.205	0.225				*AHRR*	Body
cg02959637	11	64,513,289	0.020198	0.205	0.129				*RASGRP2*	TSS1500
cg08087268	14	93,406,082	1.08E-06	0.205	0.199				*ITPK1*	3'UTR
cg02772106	X	152,992,265	0.02288	0.205	0.244				*ABCD1*	Body
cg01923252	6	108,436,849	0.01113	0.205	0.302					
cg02768162	12	108,992,148	9.62E-05	0.205	0.248				*TMEM119*	TSS1500
cg11572548	1	22,401,641	0.00192	0.205	0.197				*CDC42*	5'UTR
cg10950111	1	228,337,379	1.28E-07	0.205	0.229				*GJC2*	TSS200
cg18989909	2	121,344,551	9.12E-09	0.205	0.211					
cg21522159	4	7,252,415	0.019004	0.205	0.178				*SORCS2*	Body
cg23402305	2	178,178,939	2.44E-05	0.205	0.214				*LOC100130691*	Body
cg15202102	11	6,341,867	0.024791	0.205	0.254				*PRKCDBP*	TSS200
cg09036837	1	25,392,224	2.32E-08	0.205	0.197					
cg12311936	2	144,994,885	3.41E-07	0.205	0.226				*GTDC1*	5'UTR
cg27606341	5	39,219,632	0.000561	0.205	0.163	8.21E-16	0.132		*FYB*	1stExon
cg01861825	11	62,693,818	2.59E-05	0.205	0.185					
cg07274253	5	10,330,984	2.59E-08	0.205	0.214					
cg08679971	19	38,281,047	7.3E-08	0.205	0.180					
cg17147317	1	207,669,324	1.44E-05	0.205	0.172				*CR1*	TSS200
cg18146226	3	187,443,267	0.011047	0.205	0.146			Yes	*BCL6*	Body
cg02179700	10	3,336,162	3.93E-07	0.205	0.195					
cg16849609	2	119,916,662	0.049741	0.205	0.205				*C1QL2*	TSS200
cg24296900	12	49,657,537	4.04E-15	0.205	0.213				*TUBA1C*	TSS1500
cg00983948	6	158,937,485	5.56E-10	0.205	0.199					
cg01270736	16	3,097,736	4.84E-05	0.205	0.206				*MMP25*	Body
cg01529552	14	95,687,948	1.02E-12	0.205	0.204				*CLMN*	Body
cg15600488	8	17,271,119	0.016824	0.205	0.198				*MTMR7*	TSS200

cg17753347	1	197,883,190	4.5E-07	0.205	0.199				*LHX9*	Body
cg03292040	2	132,286,665	1.01E-08	0.205	0.209				*CCDC74A*	Body
cg12966875	20	43,883,746	0.004537	0.205	0.170	1.63E-18	0.214		*SLPI*	TSS1500
cg03562360	2	121,670,282	1.43E-23	0.205	0.185				*GLI2*	Body
cg04488758	12	95,945,366	1.87E-11	0.205	0.180	5.43E-09	0.075		*USP44*	TSS200
cg18228116	10	133,995,345	2.55E-07	0.205	0.192				*JAKMIP3*	3'UTR
cg20661138	8	145,925,314	0.046775	0.205	0.109					
cg26174326	6	26,016,674	1.15E-11	0.205	0.202					
cg27079118	7	128,809,298	0.000501	0.205	0.165					
cg01244006	12	6,485,916	4.21E-05	0.205	0.193				*SCNN1A*	Body
cg03536657	15	69,099,834	6.3E-12	0.205	0.195				*C15orf28*	TSS1500
cg04265797	14	38,679,952	5.72E-05	0.205	0.161				*SSTR1*	3'UTR
cg12444166	4	8,359,458	2.07E-05	0.205	0.207					
cg21204588	15	50,316,779	3.69E-09	0.205	0.197				*ATP8B4*	Body
cg21565937	2	709,250	2.98E-05	0.205	0.217					
cg01626112	12	76,653,220	2.19E-07	0.205	0.238					
cg10512769	1	211,675,356	1.24E-05	0.205	0.192					
cg10705379	10	99,080,932	0.00035	0.205	0.161				*FRAT1*	1stExon
cg12799029	17	984,184	2.11E-05	0.205	0.220				*ABR*	Body
cg18221021	11	84,432,312	0.001178	0.205	0.310				*DLG2*	Body
cg20948956	2	33,317,174	0.000618	0.205	0.261				*LTBP1*	Body
cg13969239	6	106,439,962	0.043697	0.205	0.102					
cg18208842	2	287,395	9.55E-06	0.205	0.208				*FAM150B*	Body
cg09258553	2	241,564,827	0.000412	0.205	0.157					
cg13177747	8	109,095,951	0.006741	0.205	0.193				*RSPO2*	TSS200
cg10275315	15	83,954,246	3.04E-10	0.205	0.208				*BNC1*	TSS1500
cg12751521	12	109,711,293	2.06E-12	0.205	0.216					
cg21647257	19	36,523,945	6.81E-05	0.205	0.184				*CLIP3*	TSS200
cg05526364	3	129,205,070	7.24E-11	0.205	0.204				*IFT122*	Body
cg12901910	1	35,586,453	1.59E-07	0.205	0.217					
cg24503003	12	110,682,837	9.88E-07	0.205	0.194					
cg04036182	15	45,458,818	1.49E-11	0.205	0.189					
cg10397082	2	1,609,832	1.97E-07	0.205	0.196					
cg14411266	2	40,679,582	0.017719	0.205	0.099				*SLC8A1*	5'UTR

cg02927252	1	14,926,373	1.79E-06	0.205	0.208				*KIAA1026*	Body
cg10729196	3	149,797,095	1.99E-09	0.205	0.208					
cg14476852	8	8,506,370	8.41E-07	0.205	0.171					
cg20433906	17	62,076,318	0.000173	0.205	0.226				*C17orf72*	Body
cg21739754	2	185,462,847	0.001055	0.205	0.086				*ZNF804A*	TSS1500
cg05686445	7	127,636,396	2.94E-14	0.205	0.207				*C7orf54*	TSS1500
cg09015246	16	10,971,105	7.17E-06	0.205	0.219				*CIITA*	1stExon
cg09492451	16	54,967,389	5.26E-05	0.205	0.243				*IRX5*	Body
cg19889856	7	134,856,654	2.24E-07	0.205	0.218				*C7orf49*	TSS1500
cg01173432	3	71,622,662	4.87E-08	0.205	0.185				*FOXP1*	5'UTR
cg02578942	3	174,558,606	2.86E-05	0.205	0.237					
cg25596289	17	10,741,383	2.78E-09	0.205	0.222				*PIRT*	1stExon
cg13581155	5	135,415,781	0.001531	0.205	0.152					
cg18685455	6	135,796,690	1.21E-05	0.205	0.177				*AHI1*	Body
cg19402876	3	71,119,415	7.18E-06	0.205	0.176				*FOXP1*	Body
cg21544633	11	12,494,428	3.16E-08	0.205	0.185				*PARVA*	Body
cg21859594	12	4,872,801	0.015545	0.205	0.244				*GALNT8*	Body
cg04105250	2	171,679,114	2.08E-05	0.205	0.222				*GAD1*	Body
cg09295799	6	5,994,235	0.009777	0.205	0.183					
cg15261665	3	46,506,374	0.001438	0.205	0.166	1.57E-26	0.239		*LTF*	5'UTR
cg23424125	8	1,494,546	5.15E-05	0.205	0.158				*DLGAP2*	5'UTR
cg04983903	6	29,974,065	2.83E-09	0.205	0.177				*HLA-J*	Body
cg19567594	11	10,715,175	3.55E-05	0.205	0.168				*MRVI1*	1stExon
cg23082635	3	193,991,194	1.82E-07	0.205	0.202					
cg04080517	1	120,905,322	1.49E-06	0.205	0.161				*HIST2H2BA*	TSS1500
cg20022223	17	80,260,410	1.46E-09	0.205	0.198					
cg24884444	8	49,572,044	3.38E-05	0.205	0.219					
cg00232402	18	77,385,290	2.55E-05	0.205	0.177					
cg00336320	6	36,253,099	0.004205	0.205	0.183				*PNPLA1*	5'UTR
cg04144521	2	191,276,183	1.14E-12	0.205	0.189				*MFSD6*	5'UTR
cg06740950	3	171,878,318	7.5E-11	0.205	0.197				*FNDC3B*	Body
cg08306303	11	119,567,761	3.21E-06	0.205	0.193				*PVRL1*	Body
cg03833604	12	54,378,500	0.010919	0.205	0.162				*HOXC10*	TSS1500
cg06741367	2	128,453,108	0.00913	0.205	0.173					

cg20956815	3	184,320,006	3.3E-05	0.205	0.192					
cg02235249	2	171,570,003	0.000544	0.205	0.157				*LOC440925*	Body
cg12744061	1	115,444,733	9.84E-08	0.205	0.168				*SYCP1*	Body
cg17860090	1	209,605,104	1.43E-05	0.205	0.138				*LOC642587*	Body
cg11022926	7	43,652,643	6.48E-06	0.205	0.209				*STK17A*	Body
cg14692377	17	28,562,685	0.001704	0.205	0.147				*SLC6A4*	1stExon
cg05431842	4	30,724,915	5.78E-07	0.205	0.211				*PCDH7*	1stExon
cg11707219	8	21,905,756	1.82E-08	0.205	0.169				*FGF17*	Body
cg13871843	1	9,887,984	4.35E-13	0.205	0.183					
cg20161179	4	4,863,282	0.000396	0.205	0.211	2.57E-17	0.198		*MSX1*	Body
cg26333564	14	105,147,781	0.000208	0.205	0.181					
cg04738888	17	73,584,111	1.82E-06	0.205	0.201				*MYO15B*	TSS200
cg16142313	8	28,950,249	2.18E-12	0.205	0.183				*KIF13B*	Body
cg19555331	4	147,561,900	0.035185	0.205	0.248				*POU4F2*	Body
cg20073553	4	13,545,291	9.23E-05	0.205	0.209	1.91E-09	0.133		*NKX3-2*	Body
cg22787186	11	324,634	2.79E-16	0.205	0.188					
cg09437162	9	138,555,074	0.007302	0.205	0.161				*LCN9*	TSS200
cg10092957	19	39,687,079	0.002973	0.205	0.291	1.73E-13	0.095		*NCCRP1*	TSS1500
cg17375248	2	66,811,157	0.011175	0.205	0.203					
cg19273694	2	24,233,923	1.98E-11	0.205	0.208				*MFSD2B*	Body
cg20138067	1	203,273,571	5.52E-10	0.205	0.180				*BTG2*	TSS1500
cg22008026	4	155,413,392	0.000212	0.205	0.216				*DCHS2*	TSS1500
cg02923485	1	223,959,609	9.66E-05	0.205	0.195				*CAPN2*	Body
cg18117669	15	57,510,284	5.78E-08	0.205	0.190				*TCF12*	TSS1500
cg06570224	3	157,812,475	4.55E-05	0.205	0.177					
cg14706428	4	6,113,389	0.00312	0.205	0.211				*JAKMIP1*	Body
cg00188032	5	96,141,721	1.16E-05	0.205	0.196				*ERAP1*	5'UTR
cg10953225	1	149,326,531	0.000249	0.205	0.227					
cg13458005	10	27,702,309	3.99E-05	0.205	0.224				*PTCHD3*	1stExon
cg13529733	2	1,609,194	8.85E-06	0.205	0.207					
cg13757081	7	26,480,747	0.000374	0.205	0.145				*LOC441204*	Body
cg18530324	18	46,064,992	0.001124	0.205	0.067	1.4E-23	0.201		*KIAA0427*	TSS1500
cg21756647	11	75,024,611	5.26E-08	0.205	0.211				*ARRB1*	Body
cg11996817	17	8,230,768	6.24E-06	0.205	0.180					

cg13994241	17	46,667,725	4.76E-12	0.205	0.190				*LOC404266*	TSS200
cg21440587	6	31,583,458	0.000108	0.205	0.234	1.86E-16	0.080		*AIF1*	TSS1500
cg23781334	4	1,772,062	1.34E-23	0.205	0.200					
cg25310081	12	56,959,642	0.000432	0.205	0.179				*RBMS2*	Body
cg00277591	17	4,079,652	1.71E-08	0.205	0.186				*ANKFY1*	Body
cg02455706	16	84,918,851	0.000129	0.205	0.190				*CRISPLD2*	Body
cg08288561	16	66,462,307	3.33E-05	0.205	0.187				*BEAN*	5'UTR
cg10090792	1	38,198,142	1.86E-10	0.205	0.205				*EPHA10*	Body
cg14066905	11	124,953,090	2.13E-08	0.205	0.200				*SLC37A2*	Body
cg25345178	8	72,459,908	0.018264	0.205	0.245					
cg03327325	10	43,846,574	0.048025	0.205	0.129			Yes		
cg09900242	16	68,676,451	7.99E-08	0.205	0.188					
cg14973892	13	46,944,143	9.19E-05	0.205	0.212				*C13orf18*	Body
cg22366375	4	25,033,140	0.002085	0.205	0.173				*LGI2*	TSS1500
cg19319393	1	6,321,737	0.00542	0.205	0.144				*GPR153*	TSS1500
cg23337631	12	52,994,933	3.17E-09	0.205	0.221				*KRT72*	1stExon
cg00124009	1	201,439,578	2.37E-23	0.205	0.197				*PHLDA3*	TSS1500
cg12110437	8	144,098,888	0.00023	0.205	0.208				*LY6E*	TSS1500
cg20068535	6	151,560,981	2.2E-06	0.205	0.204				*AKAP12*	TSS200
cg04171065	14	42,075,760	0.018606	0.205	0.286				*LRFN5*	TSS1500
cg20517697	14	74,208,225	0.000206	0.205	0.189				*C14orf43*	5'UTR
cg16502826	1	970,237	4E-09	0.205	0.193				*AGRN*	Body
cg22219379	3	140,541,355	0.000122	0.205	0.189					
cg24946736	19	7,542,181	8.68E-21	0.205	0.200				*PEX11G*	Body
cg01583753	2	39,470,725	0.044901	0.205	0.089					
cg06982745	10	72,454,006	0.00827	0.205	0.133				*ADAMTS14*	Body
cg15299388	4	48,491,941	0.000427	0.205	0.184				*ZAR1*	TSS1500
cg16555909	1	23,721,285	0.00278	0.205	0.230				*TCEA3*	Body
cg07243366	12	19,729,703	1.09E-08	0.205	0.221					
cg13780614	15	49,607,868	8.27E-14	0.205	0.207				*GALK2*	Body
cg13059719	1	179,712,027	0.022737	0.205	0.175				*FAM163A*	TSS1500
cg15498274	19	47,742,558	1.39E-19	0.205	0.173					
cg19107504	10	88,467,518	3.81E-05	0.205	0.224				*LDB3*	Body
cg09762021	1	153,762,224	0.002154	0.205	0.172					

cg21092790	8	117,950,244	0.009941	0.205	0.197				*C8orf85*	TSS1500
cg25465406	17	7,906,098	0.039796	0.205	0.221	4.17E-13	0.161		*GUCY2D*	5'UTR
cg00625783	17	43,222,106	0.025432	0.205	0.122					
cg05740045	8	65,499,926	0.000195	0.205	0.173					
cg17149655	15	61,521,910	0.001993	0.205	0.179				*RORA*	TSS1500
cg05300248	18	24,537,063	3.36E-06	0.205	0.181				*CHST9*	Body
cg14652095	6	26,018,185	5.85E-05	0.205	0.173	3.13E-21	0.249		*HIST1H1A*	TSS200
cg17696160	14	37,136,192	0.008184	0.205	0.127				*PAX9*	Body
cg05643188	1	197,873,430	4.14E-06	0.204	0.200				*C1orf53*	Body
cg07739851	17	72,231,448	0.002613	0.204	0.212				*TTYH2*	Body
cg23177714	8	142,877,740	3.64E-06	0.204	0.212					
cg02122367	5	1,841,622	0.000587	0.204	0.129					
cg03958979	6	108,486,387	2.27E-07	0.204	0.188	3.39E-17	0.205		*NR2E1*	TSS1500
cg20169062	5	53,751,411	5.56E-09	0.204	0.194	0.0000412	0.099		*HSPB3*	TSS200
cg20735282	11	69,481,734	2.61E-06	0.204	0.179				*ORAOV1*	3'UTR
cg26278454	20	982,623	0.000467	0.204	0.175				*RSPO4*	Body
cg00093099	18	2,849,071	0.001243	0.204	0.221				*EMILIN2*	Body
cg15641024	11	57,232,331	1.42E-06	0.204	0.240				*RTN4RL2*	Body
cg07249684	8	689,392	0.021089	0.204	0.249					
cg14768245	11	14,926,780	0.012094	0.204	0.235					
cg19962424	11	35,110,045	0.00886	0.204	0.268					
cg03733207	20	36,932,553	6.22E-05	0.204	0.189				*BPI*	1stExon
cg08133931	17	48,636,626	2.89E-05	0.204	0.217					
cg13058457	15	38,422,160	0.003547	0.204	0.160					
cg24379332	19	10,627,706	0.000645	0.204	0.227				*S1PR5*	5'UTR
cg06465011	16	84,860,871	9.17E-07	0.204	0.189				*CRISPLD2*	5'UTR
cg23180841	2	28,897,312	0.001582	0.204	0.211					
cg20559633	8	142,734,242	1.91E-05	0.204	0.161					
cg01325935	X	67,905,522	1.18E-11	0.204	0.187				*STARD8*	5'UTR
cg13130319	14	77,964,542	1.89E-05	0.204	0.198				*ISM2*	Body
cg13928759	10	95,517,382	3.73E-08	0.204	0.203				*LGI1*	TSS200
cg18074297	21	36,041,612	0.000349	0.204	0.191				*CLIC6*	TSS200
cg22459630	15	68,121,028	0.020474	0.204	0.241				*LBXCOR1*	Body
cg09975397	11	74,384,522	4.22E-10	0.204	0.201					

cg22489957	6	28,921,805	0.042966	0.204	0.142					
cg21380341	12	94,543,978	0.03494	0.204	0.204				*PLXNC1*	Body
cg00463746	1	64,434,320	0.007605	0.204	0.200				*ROR1*	Body
cg17549878	17	18,280,849	1.82E-07	0.204	0.174				*EVPLL*	TSS1500
cg20991420	2	173,061,715	8.92E-06	0.204	0.148					
cg09556952	21	38,119,946	0.012658	0.204	0.191				*SIM2*	Body
cg16362949	17	1,390,671	0.00047	0.204	0.105				*MYO1C*	5'UTR
cg24910943	10	11,206,320	0.000629	0.204	0.210				*CUGBP2*	Body
cg04655481	9	125,796,809	3.73E-18	0.204	0.195	2.27E-18	0.141		*GPR21*	TSS200
cg10853431	10	11,574,300	0.000102	0.204	0.212				*USP6NL*	Body
cg14534144	13	114,150,007	6.95E-07	0.204	0.199				*TMCO3*	Body
cg17783086	17	62,775,188	0.003807	0.204	0.112				*LOC146880*	Body
cg04076774	12	107,954,581	4.74E-07	0.204	0.196				*BTBD11*	Body
cg07116163	2	170,589,499	1.13E-05	0.204	0.193				*KLHL23*	5'UTR
cg11337388	1	44,446,092	2.84E-09	0.204	0.204				*B4GALT2*	5'UTR
cg25772221	1	1,079,622	6.34E-08	0.204	0.155					
cg26266099	1	33,391,638	2.98E-05	0.204	0.198					
cg00721947	8	143,755,447	1.85E-09	0.204	0.193					
cg01883046	2	174,890,420	1.25E-09	0.204	0.180					
cg03769939	1	25,254,129	1.2E-10	0.204	0.198				*RUNX3*	Body
cg11762968	13	95,354,190	1.04E-05	0.204	0.180					
cg13629753	1	89,591,719	0.009351	0.204	0.326	0.00000377	0.090		*GBP2*	1stExon
cg12579212	3	46,448,868	3.29E-06	0.204	0.196				*CCRL2*	5'UTR
cg20892260	8	23,564,025	0.00015	0.204	0.189				*NKX2-6*	TSS200
cg09948958	14	94,443,055	0.010434	0.204	0.230					
cg12068124	19	1,238,538	8.41E-07	0.204	0.243				*C19orf26*	TSS1500
cg21794767	7	120,657,213	5.81E-08	0.204	0.214				*C7orf58*	Body
cg08247587	14	38,091,400	0.000126	0.204	0.199					
cg13983182	9	130,853,333	9.94E-15	0.204	0.192				*SLC25A25*	Body
cg14334014	17	64,226,953	3.06E-13	0.204	0.210				*APOH*	TSS1500
cg11018723	1	25,256,939	0.033992	0.204	0.238				*RUNX3*	Body
cg09566302	16	30,964,627	7.12E-10	0.204	0.217				*ORAI3*	Body
cg12530864	4	188,953,092	0.006242	0.204	0.208					
cg12680205	10	80,303,482	6.61E-06	0.204	0.165					

cg19567891	15	89,921,083	8.59E-07	0.204	0.173				*LOC254559*	TSS200
cg25988321	14	95,238,750	0.000554	0.204	0.203					
cg06215457	13	46,190,028	0.001477	0.204	0.231				*FAM194B*	TSS200
cg19893473	3	177,778,301	1.25E-09	0.204	0.206					
cg14266251	9	135,462,291	0.032498	0.204	0.198				*BARHL1*	Body
cg06520300	2	16,081,824	0.001924	0.204	0.145				*MYCN*	5'UTR
cg06783423	3	42,538,831	6.69E-06	0.204	0.206					
cg21097788	14	93,502,930	4.27E-10	0.204	0.195				*ITPK1*	Body
cg02642822	17	46,666,926	0.003227	0.204	0.191				*LOC404266*	TSS1500
cg03466525	10	50,650,021	5.98E-08	0.204	0.181					
cg08457898	19	15,343,786	0.002922	0.204	0.221				*EPHX3*	5'UTR
cg11810743	6	168,714,693	2.86E-06	0.204	0.170				*DACT2*	Body
cg23998478	2	121,269,292	6.33E-06	0.204	0.199					
cg24335116	12	123,469,507	3.89E-06	0.204	0.248				*PITPNM2*	3'UTR
cg24834659	19	22,805,803	0.031825	0.204	0.225					
cg24574963	7	149,120,622	1.57E-15	0.204	0.197					
cg06842071	2	233,499,460	2.3E-05	0.204	0.184				*EFHD1*	Body
cg14883961	8	127,846,077	1.49E-06	0.204	0.184					
cg00214171	17	73,642,991	4.07E-09	0.204	0.200				*RECQL5*	Body
cg03463523	16	73,099,917	0.000301	0.204	0.197					
cg07770222	8	144,120,106	0.015197	0.204	0.119	0.00993538	0.041		*C8orf31*	TSS1500
cg08388455	17	37,081,875	7.33E-09	0.204	0.194					
cg26262576	13	20,420,028	2.32E-05	0.204	0.229				*ZMYM5*	Body
cg15338967	13	49,175,326	5.61E-06	0.204	0.234					
cg17007374	15	71,417,947	2.01E-09	0.204	0.210					
cg17926940	14	97,685,060	0.036194	0.204	0.273					
cg23473955	17	27,893,407	2.01E-09	0.204	0.189				*ABHD15*	1stExon
cg24156261	3	99,721,850	2.29E-19	0.204	0.193				*C3orf26*	Body
cg08777316	5	158,531,511	0.002488	0.204	0.181					
cg10576245	1	233,249,267	0.000311	0.204	0.200				*PCNXL2*	Body
cg17480077	1	10,895,517	3.05E-09	0.204	0.188					
cg25010500	5	140,619,586	0.001613	0.204	0.263				*PCDHB19P*	TSS200
cg26064794	1	204,435,434	2.1E-19	0.204	0.199				*PIK3C2B*	Body
cg05239811	6	31,734,147	6.72E-05	0.204	0.212				*C6orf27*	Body

cg08224569	8	142,369,705	7.93E-11	0.204	0.190				*GPR20*	5'UTR
cg24940576	11	125,904,314	7.43E-06	0.204	0.232				*CDON*	5'UTR
cg04650789	15	96,864,196	2.66E-06	0.204	0.214					
cg18290981	11	63,789,520	3.64E-14	0.204	0.188				*MACROD1*	Body
cg04734712	17	1,811,474	0.001084	0.204	0.150					
cg26251325	5	54,518,745	0.021909	0.204	0.191					
cg03828874	5	3,605,954	0.001637	0.204	0.267					
cg24937877	1	11,444,066	0.000414	0.204	0.213					
cg27241845	2	233,250,370	0.001937	0.204	0.173					
cg06587257	12	50,452,135	2.38E-05	0.204	0.176				*ACCN2*	5'UTR
cg16468389	17	46,831,742	1.61E-07	0.204	0.198					
cg25560775	15	65,707,468	1.67E-05	0.204	0.198				*IGDCC4*	Body
cg26393630	14	30,399,576	0.007199	0.204	0.208					
cg00517787	4	154,713,235	0.001694	0.204	0.209					
cg09430957	19	51,419,804	5.02E-10	0.204	0.192					
cg05655534	2	62,798,448	0.004735	0.204	0.222					
cg08288016	4	187,590,536	8.08E-09	0.204	0.191				*FAT1*	Body
cg01300684	4	141,230,118	2.78E-11	0.204	0.197				*SCOC*	5'UTR
cg09146183	22	38,610,376	5.27E-05	0.204	0.204				*MAFF*	Body
cg14880184	18	19,758,221	1.31E-17	0.204	0.197				*GATA6*	Body
cg19584871	2	11,810,508	0.001305	0.204	0.206				*NTSR2*	TSS200
cg05138949	3	57,195,542	0.002366	0.204	0.225				*IL17RD*	Body
cg20381115	15	45,671,148	7.45E-05	0.204	0.201				*LOC145663*	Body
cg27517681	1	119,528,848	0.033147	0.204	0.201				*TBX15*	5'UTR
cg13827582	15	99,971,213	2.44E-06	0.204	0.182					
cg22600043	8	109,095,991	0.001039	0.204	0.237				*RSPO2*	TSS200
cg07103201	17	20,171,047	7.59E-07	0.204	0.199				*CYTSB*	Body
cg17518215	2	172,973,241	5.04E-06	0.204	0.178					
cg07013148	8	146,233,339	6.55E-17	0.204	0.204					
cg08823954	11	73,359,662	1.36E-05	0.204	0.168				*PLEKHB1*	5'UTR
cg14848289	11	117,732,291	0.001831	0.204	0.179				*FXYD6*	5'UTR
cg15880760	17	48,546,290	0.000253	0.204	0.174				*CHAD*	TSS200
cg03434509	8	82,543,424	1.69E-06	0.204	0.197					
cg03847705	10	115,860,959	0.045174	0.204	0.008					

cg00696323	3	124,520,019	2.51E-11	0.204	0.187				*ITGB5*	Body
cg06962787	17	15,522,897	2.48E-12	0.204	0.205				*CDRT1*	1stExon
cg09078293	16	17,510,730	3.12E-07	0.204	0.187				*XYLT1*	Body
cg17843665	13	24,351,673	0.000191	0.204	0.226				*MIPEP*	Body
cg20341836	6	29,894,228	0.003552	0.204	0.163				*HCG4P6*	TSS1500
cg02659920	11	726,335	0.009782	0.204	0.134				*EPS8L2*	Body
cg02990302	16	58,155,189	0.000558	0.204	0.222				*C16orf80*	Body
cg21578330	4	87,829,966	1.64E-07	0.204	0.190					
cg12210914	1	228,075,694	0.000105	0.204	0.201					
cg13976134	4	77,733,007	3.25E-05	0.204	0.221					
cg15709989	3	185,912,227	0.000106	0.204	0.163				*DGKG*	Body
cg15895121	5	141,625,843	0.002878	0.204	0.240					
cg25799109	3	57,102,900	0.00094	0.204	0.166				*ARHGEF3*	5'UTR
cg11346237	7	130,019,736	7.17E-09	0.204	0.201				*CPA1*	TSS1500
cg02286761	19	14,315,694	1.08E-16	0.204	0.212				*LPHN1*	5'UTR
cg10854092	10	133,985,931	0.000979	0.204	0.209				*JAKMIP3*	3'UTR
cg02531277	7	101,558,597	8.55E-30	0.204	0.199				*CUX1*	Body
cg23361265	17	15,917,660	1.2E-07	0.204	0.241				*TTC19*	Body
cg22157027	2	10,638,113	8.31E-05	0.204	0.178					
cg25635500	22	29,712,139	7.91E-09	0.204	0.210	6.58E-15	0.112		*RASL10A*	TSS1500
cg04523603	1	3,622,085	7.91E-09	0.204	0.223				*TP73*	Body
cg21609154	13	113,624,991	8.54E-12	0.204	0.182				*MCF2L*	Body
cg05824809	16	85,427,847	1.68E-14	0.204	0.190					
cg13634560	13	38,173,852	0.01051	0.204	0.216				*POSTN*	TSS1500
cg14762973	2	187,714,068	0.004378	0.204	0.178				*ZSWIM2*	TSS200
cg04593696	16	84,076,900	5.18E-06	0.204	0.176				*SLC38A8*	TSS1500
cg10198294	10	28,616,726	2.5E-06	0.204	0.200					
cg11964308	12	54,535,018	0.000236	0.204	0.208					
cg16058493	1	91,189,167	0.001567	0.204	0.233					
cg13980454	14	36,985,905	0.000792	0.204	0.228				*NKX2-1*	3'UTR
cg13824500	10	114,394,236	1.51E-18	0.204	0.189				*VTI1A*	Body
cg25657713	6	100,038,708	7.42E-07	0.204	0.212					
cg08710739	12	111,470,672	2.52E-13	0.204	0.205				*CUX2*	TSS1500
cg21903646	6	28,447,115	0.003896	0.204	0.184					

cg26413900	17	43,985,654	9.01E-06	0.204	0.173				*MAPT*	5'UTR
cg03714531	6	30,923,590	0.011291	0.204	0.309					
cg17614974	3	44,039,919	0.007514	0.204	0.147					
cg21121843	4	3,203,982	3.36E-09	0.204	0.172				*HTT*	Body
cg03300078	11	1,769,522	0.007431	0.204	0.174				*HCCA2*	Body
cg04304333	17	59,474,038	0.000309	0.204	0.175					
cg06616081	15	77,336,938	4.57E-05	0.204	0.205				*TSPAN3*	3'UTR
cg15511120	11	6,598,119	5.14E-09	0.204	0.190					
cg27651243	7	156,799,148	0.004599	0.204	0.216				*MNX1*	Body
cg03551963	5	39,502,320	3.62E-07	0.204	0.171					
cg22525581	12	54,439,112	3.31E-13	0.204	0.190				*HOXC4*	5'UTR
cg05152781	1	41,780,807	2.72E-05	0.204	0.207			Yes		
cg05201970	5	45,696,206	5.62E-11	0.204	0.204				*HCN1*	5'UTR
cg17924813	4	15,704,844	0.004007	0.204	0.204				*BST1*	1stExon
cg23149064	2	130,970,868	4.43E-10	0.204	0.183					
cg24649332	20	45,280,359	3.4E-10	0.204	0.203				*SLC13A3*	5'UTR
cg03128890	8	122,558,224	1.35E-05	0.204	0.181					
cg05573844	12	54,354,590	0.038593	0.204	0.125					
cg14950321	19	4,530,233	7.12E-09	0.204	0.212				*PLIN5*	Body
cg15081886	1	1,356,902	3E-08	0.204	0.202				*LOC441869*	TSS1500
cg18400079	4	7,813,911	1.32E-08	0.204	0.210			Yes	*AFAP1*	Body
cg01062113	11	93,865,443	3.27E-25	0.204	0.189				*PANX1*	Body
cg02525435	16	88,977,768	2.59E-07	0.204	0.215				*CBFA2T3*	5'UTR
cg22743761	2	162,273,648	0.015497	0.204	0.249				*TBR1*	Body
cg02304584	6	31,650,790	7.72E-08	0.204	0.202					
cg25869196	2	102,952,419	7.89E-08	0.204	0.210				*IL1RL1*	5'UTR
cg07460804	16	14,163,963	4.15E-07	0.204	0.182				*MKL2*	TSS1500
cg10485752	1	26,021,655	1.41E-17	0.204	0.201				*MAN1C1*	Body
cg01536979	8	117,840,383	1.23E-08	0.204	0.209					
cg04066316	4	189,433,040	0.001659	0.204	0.252					
cg08626653	7	100,082,322	4.78E-18	0.204	0.197	3.21E-07	0.059		*C7orf51*	5'UTR
cg21701379	12	31,079,694	0.018749	0.204	0.113				*TSPAN11*	TSS200
cg21841632	3	66,853,898	0.000787	0.204	0.223			Yes		
cg00044871	13	25,945,811	0.0041	0.204	0.086				*ATP8A2*	TSS1500

cg02423064	17	32,484,008	0.000315	0.204	0.239				*ACCN1*	TSS200
cg05783135	11	78,092,939	4.24E-11	0.204	0.182				*GAB2*	Body
cg13635299	6	42,409,704	2.09E-08	0.204	0.203				*TRERF1*	5'UTR
cg20383637	2	2,965,744	2.5E-05	0.204	0.165					
cg06865612	2	946,999	1.25E-06	0.204	0.217				*SNTG2*	Body
cg03997176	6	29,796,066	0.030616	0.204	0.263				*HLA-G*	Body
cg04565337	14	38,071,768	2.53E-07	0.204	0.203					
cg13332142	3	27,575,278	4.29E-07	0.204	0.185					
cg01245155	2	205,919,517	3.42E-11	0.203	0.201				*PARD3B*	Body
cg02219949	17	45,927,392	2E-05	0.203	0.156				*SP6*	5'UTR
cg04522003	17	40,913,702	8.61E-20	0.203	0.225				*LOC100190938*	TSS1500
cg16096311	1	151,693,261	0.008864	0.203	0.162				*C1orf230*	TSS1500
cg04074004	6	31,696,462	1.98E-07	0.203	0.222				*DDAH2*	Body
cg10708189	1	58,898,793	8.26E-05	0.203	0.142					
cg11735997	11	30,039,113	0.024391	0.203	0.225				*KCNA4*	TSS1500
cg12384499	15	89,949,617	0.017406	0.203	0.284					
cg15752885	7	158,255,389	6.07E-08	0.203	0.195				*PTPRN2*	Body
cg26328633	5	135,416,394	3.03E-05	0.203	0.175				*MIR886*	TSS200
cg00697703	7	25,018,855	1.76E-05	0.203	0.162				*OSBPL3*	5'UTR
cg04694437	5	88,185,051	0.002316	0.203	0.163				*MEF2C*	5'UTR
cg05554161	9	127,540,080	4.87E-10	0.203	0.181				*OLFML2A*	Body
cg17658113	11	1,413,282	0.000955	0.203	0.183				*BRSK2*	Body
cg04837832	10	101,290,958	0.010642	0.203	0.168					
cg13845094	12	81,108,034	1.72E-08	0.203	0.201					
cg08632388	16	88,700,818	0.001045	0.203	0.191					
cg12149609	20	42,816,600	1.87E-05	0.203	0.213				*JPH2*	TSS1500
cg00306909	2	175,194,079	0.000201	0.203	0.201					
cg03295107	7	151,236,292	1.87E-08	0.203	0.210					
cg06359394	2	187,559,328	0.017555	0.203	0.071				*FAM171B*	Body
cg11348249	6	151,560,831	9.56E-06	0.203	0.207				*AKAP12*	TSS1500
cg25224048	5	38,467,248	3.63E-09	0.203	0.220					
cg25687642	2	210,871	6.27E-05	0.203	0.217					
cg16809460	1	50,885,369	0.038419	0.203	0.324				*DMRTA2*	Body
cg12626957	3	187,770,324	3.18E-07	0.203	0.194					

cg13842240	4	13,539,213	3.33E-06	0.203	0.187					
cg16610917	17	40,912,707	5.93E-11	0.203	0.196				*LOC100190938*	Body
cg17002328	14	91,751,773	3.95E-12	0.203	0.208				*CCDC88C*	Body
cg18261050	1	119,551,319	0.034616	0.203	0.276					
cg20811456	7	20,816,971	1.28E-06	0.203	0.180					
cg24114556	17	44,897,431	1.41E-06	0.203	0.187				*WNT3*	TSS1500
cg09313734	7	155,327,245	1.05E-06	0.203	0.181				*CNPY1*	TSS1500
cg13348530	4	7,283,184	0.000698	0.203	0.187				*SORCS2*	Body
cg21116900	12	100,750,760	0.000169	0.203	0.226				*SLC17A8*	TSS200
cg19035107	6	26,745,662	0.027153	0.203	0.182					
cg21412456	1	26,608,782	0.033698	0.203	0.237				*UBXN11*	3'UTR
cg02615468	3	58,563,618	0.000139	0.203	0.224				*FAM107A*	TSS200
cg19249811	10	29,747,315	2.18E-05	0.203	0.201				*SVIL*	Body
cg26978064	17	70,461,791	4.35E-05	0.203	0.122					
cg00385761	1	228,131,966	5.45E-12	0.203	0.188				*WNT9A*	Body
cg09307564	14	36,983,737	0.00307	0.203	0.211				*SFTA3*	TSS1500
cg14632941	4	42,156,182	0.000153	0.203	0.207				*BEND4*	TSS1500
cg22763680	17	33,815,432	0.006146	0.203	0.242				*SLFN12L*	Body
cg07359379	1	27,445,362	3.17E-09	0.203	0.211				*SLC9A1*	Body
cg12157761	15	71,005,778	1.02E-09	0.203	0.201				*UACA*	Body
cg15259233	6	170,712,708	6.57E-09	0.203	0.232				*FAM120B*	Body
cg18633230	7	151,425,104	4.74E-13	0.203	0.198				*PRKAG2*	Body
cg27344315	15	100,560,801	1.01E-06	0.203	0.190				*ADAMTS17*	Body
cg02138331	20	32,893,975	8.09E-09	0.203	0.207				*AHCY*	5'UTR
cg02430039	17	47,967,818	7.32E-06	0.203	0.204					
cg07829379	17	35,294,198	0.039921	0.203	0.177				*LHX1*	TSS1500
cg09047734	16	85,202,191	8.83E-06	0.203	0.185					
cg04568799	3	197,840,782	0.000558	0.203	0.195					
cg13071569	12	107,296,946	3.73E-05	0.203	0.187					
cg11385476	6	138,653,150	2.42E-09	0.203	0.218				*KIAA1244*	Body
cg24897126	1	6,474,383	5.8E-08	0.203	0.191					
cg08822075	7	26,193,607	0.000391	0.203	0.135				*NFE2L3*	Body
cg15531930	7	108,097,606	0.002639	0.203	0.172				*NRCAM*	TSS1500
cg02800334	8	124,750,855	2.09E-11	0.203	0.201				*ANXA13*	TSS1500

cg04601228	7	70,096,029	2.14E-11	0.203	0.191				*AUTS2*	Body
cg07145664	6	2,579,591	5.08E-13	0.203	0.178					
cg08531042	5	145,717,661	0.026873	0.203	0.262				*POU4F3*	TSS1500
cg22025233	7	1,022,904	3.88E-09	0.203	0.167	3.97E-23	0.145		*CYP2W1*	1stExon
cg22867729	12	121,622,315	9.67E-05	0.203	0.164				*P2RX7*	Body
cg23855989	12	50,355,821	3.55E-06	0.203	0.184	4.73E-18	0.121		*AQP5*	1stExon
cg26703880	11	78,764,003	2.76E-08	0.203	0.214				*ODZ4*	Body
cg27027242	17	59,485,894	1.31E-06	0.203	0.186				*TBX2*	3'UTR
cg06148812	22	19,746,368	1.64E-05	0.203	0.204				*TBX1*	5'UTR
cg06319475	8	145,105,829	2.05E-05	0.203	0.203					
cg19913626	8	49,533,357	2.01E-14	0.203	0.186					
cg23105419	2	43,496,213	3.14E-08	0.203	0.196			Yes	*THADA*	Body
cg04540882	17	27,703,877	5.27E-08	0.203	0.194					
cg13382072	19	1,851,882	1.11E-05	0.203	0.187					
cg19948701	17	75,471,193	2.66E-09	0.203	0.202				*SEPT9*	Body
cg03846408	11	1,769,390	0.023098	0.203	0.177				*HCCA2*	Body
cg23281972	2	223,610,984	6.79E-10	0.203	0.205					
cg02347652	12	122,332,665	1.37E-14	0.203	0.186				*PSMD9*	Body
cg07301952	18	158,011	1.25E-05	0.203	0.154			Yes	*USP14*	TSS1500
cg19363499	8	70,983,530	0.01035	0.203	0.248				*PRDM14*	1stExon
cg22274745	2	182,451,537	0.000492	0.203	0.180				*CERKL*	Body
cg11775521	11	74,178,795	6.62E-09	0.203	0.197				*KCNE3*	TSS200
cg20407792	6	32,044,203	2.59E-21	0.203	0.219				*TNXB*	Body
cg01052699	4	171,030,995	0.003958	0.203	0.226					
cg01193554	1	41,846,683	6.54E-05	0.203	0.153					
cg06760238	10	134,400,036	3.81E-07	0.203	0.189				*INPP5A*	Body
cg21695024	8	65,498,708	0.040127	0.203	0.274					
cg06248480	7	107,642,453	0.030912	0.203	0.211				*LAMB1*	Body
cg01513392	20	21,488,198	0.000233	0.203	0.167					
cg06579738	11	111,165,311	9.39E-11	0.203	0.200				*C11orf92*	3'UTR
cg22773334	10	131,569,486	8.13E-05	0.203	0.194					
cg24535475	12	7,167,900	9.21E-07	0.203	0.214				*C1S*	TSS200
cg01883635	2	45,033,155	0.044155	0.203	0.137					
cg05143012	7	10,974,404	0.000478	0.203	0.192				*NDUFA4*	Body

cg10831607	12	115,134,374	0.000117	0.203	0.188					
cg21556281	7	27,154,720	1.56E-05	0.203	0.170				*HOXA3*	5'UTR
cg16655791	6	106,429,610	8.73E-07	0.203	0.228					
cg19159759	13	20,989,818	3.49E-10	0.203	0.183				*CRYL1*	Body
cg14297340	9	72,871,343	1.61E-05	0.203	0.214					
cg14948757	16	56,669,726	0.000896	0.203	0.187					
cg27502457	2	87,018,585	1.27E-05	0.203	0.157				*CD8A*	5'UTR
cg14918927	12	105,477,694	4.89E-05	0.203	0.163				*ALDH1L2*	Body
cg04946274	2	127,963,067	1.08E-06	0.203	0.169				*CYP27C1*	5'UTR
cg10507482	2	175,198,039	0.027609	0.203	0.223					
cg14832904	2	54,087,028	3.27E-05	0.203	0.120	2.89E-11	0.161		*GPR75*	5'UTR
cg23927970	11	504,933	0.005384	0.203	0.169				*RNH1*	5'UTR
cg10857167	12	126,346,276	0.028407	0.203	0.091					
cg24265969	3	55,654,481	8.3E-05	0.203	0.122				*ERC2*	3'UTR
cg03232933	21	18,985,641	0.002751	0.203	0.149				*BTG3*	TSS1500
cg04149978	1	10,764,896	8.19E-05	0.203	0.148				*CASZ1*	5'UTR
cg16416987	1	155,177,561	1.12E-12	0.203	0.177				*THBS3*	Body
cg23719124	1	110,254,919	3.42E-05	0.203	0.212	8.66E-13	0.102		*GSTM5*	1stExon
cg13509147	19	4,153,603	0.000771	0.203	0.205	3.16E-08	0.091		*CREB3L3*	TSS200
cg15270687	6	90,143,634	0.030227	0.203	0.169				*ANKRD6*	5'UTR
cg06520812	19	46,000,742	0.011313	0.203	0.137				*RTN2*	TSS1500
cg27170458	16	89,988,252	0.008258	0.203	0.193				*TUBB3*	TSS1500
cg04018702	4	56,047,829	0.001286	0.203	0.248					
cg07689260	7	157,381,098	1.07E-14	0.203	0.188				*PTPRN2*	Body
cg04552418	1	31,958,245	0.008632	0.203	0.182					
cg05496514	5	87,985,513	0.031108	0.203	0.199					
cg06785429	3	182,699,414	6.39E-09	0.203	0.179	2.41E-21	0.183		*DCUN1D1*	TSS1500
cg09533168	5	175,560,568	0.004333	0.203	0.173					
cg14336674	17	41,995,064	0.002001	0.203	0.223				*C17orf88*	Body
cg07257441	17	33,814,596	0.002014	0.203	0.165				*SLFN12L*	Body
cg10572354	X	99,194,929	0.012455	0.203	0.095				*LOC442459*	TSS200
cg17575906	5	111,090,585	3.17E-10	0.203	0.206				*C5orf13*	Body
cg26339788	X	153,745,603	6.89E-06	0.203	0.231				*FAM3A*	TSS1500
cg05897316	3	40,657,497	3.74E-07	0.203	0.198					

cg09991306	2	241,975,140	1.3E-07	0.203	0.176				*SNED1*	Body
cg22650271	22	39,760,165	1E-09	0.203	0.187				*SYNGR1*	Body
cg26021007	17	45,283,040	0.000171	0.203	0.213					
cg27294268	14	36,983,518	0.00071	0.203	0.278				*SFTA3*	TSS1500
cg08022436	2	155,555,414	0.002527	0.203	0.208				*KCNJ3*	1stExon
cg08856033	2	121,280,078	4.24E-08	0.203	0.186					
cg09007447	1	114,545,478	1.15E-07	0.203	0.169					
cg15810815	6	114,663,139	0.034733	0.203	0.210					
cg19689322	1	41,284,639	0.008349	0.203	0.200				*KCNQ4*	Body
cg25486618	11	78,136,642	6.97E-07	0.203	0.190					
cg07103722	6	31,370,532	5.72E-07	0.203	0.183				*MICA*	TSS1500
cg15316660	21	38,119,920	0.005015	0.203	0.136				*SIM2*	Body
cg22738642	12	43,137,977	6.54E-07	0.203	0.163					
cg27304020	6	28,956,327	7.1E-05	0.203	0.193					
cg00606900	22	50,175,250	1.14E-05	0.203	0.195				*BRD1*	Body
cg26811159	11	89,223,981	0.003524	0.203	0.229				*NOX4*	5'UTR
cg01396530	X	12,974,262	2.03E-06	0.203	0.206					
cg15383276	22	29,709,282	7.82E-06	0.203	0.189				*RASL10A*	3'UTR
cg17766305	10	90,147,030	0.007511	0.203	0.199				*RNLS*	Body
cg21046358	5	41,708,556	0.001944	0.203	0.173					
cg08542715	17	34,217,960	2.31E-07	0.203	0.163					
cg17186750	8	38,529,336	8.03E-06	0.203	0.201					
cg01965477	17	35,285,295	0.000856	0.203	0.248					
cg02268569	16	1,032,713	8.79E-06	0.203	0.177				*SOX8*	Body
cg04747941	15	70,877,925	0.03469	0.203	0.130					
cg06635722	4	13,532,622	0.013973	0.203	0.267					
cg21581504	2	68,545,960	8.14E-06	0.203	0.192				*CNRIP1*	Body
cg23531257	15	45,422,553	3.48E-07	0.203	0.184				*DUOX1*	5'UTR
cg25318296	13	100,547,785	0.000583	0.203	0.170				*CLYBL*	3'UTR
cg09506504	6	106,439,790	0.009054	0.203	0.178					
cg17035539	1	229,544,326	3.52E-13	0.203	0.198					
cg18825458	4	55,095,446	0.002748	0.203	0.258				*PDGFRA*	1stExon
cg07191152	1	25,061,987	6.86E-11	0.203	0.198					
cg12211091	19	2,512,999	1.84E-07	0.203	0.181				*GNG7*	3'UTR

cg13719885	3	127,795,394	1.5E-06	0.203	0.214					
cg21741220	11	19,367,175	0.031261	0.203	0.089					
cg18168924	Y	7,177,888	6.77E-08	0.203	0.193				*PRKY*	Body
cg18277979	9	79,630,127	0.025207	0.203	0.186					
cg23681213	14	74,180,391	0.003552	0.203	0.087	2.45E-32	0.190		*PNMA1*	1stExon
cg03150515	8	25,836,888	0.001922	0.203	0.156				*EBF2*	Body
cg08798295	7	98,972,169	0.018283	0.203	0.108				*ARPC1B*	TSS200
cg22463553	5	140,788,504	0.00101	0.203	0.216				*PCDHGA4*	Body
cg17733447	17	45,929,399	7.35E-08	0.203	0.199				*SP6*	5'UTR
cg22517356	1	1,361,216	4.23E-07	0.203	0.259				*TMEM88B*	TSS1500
cg02286819	1	219,985,565	0.001213	0.203	0.200					
cg00027114	7	42,740,486	4.87E-06	0.203	0.226					
cg00538212	7	158,751,591	0.026768	0.203	0.074					
cg01647314	1	23,280,066	6.61E-05	0.203	0.201					
cg08739433	1	113,051,925	1.97E-07	0.203	0.178				*WNT2B*	Body
cg13396607	6	102,055,059	0.033188	0.203	0.135				*GRIK2*	Body
cg23369670	14	104,171,944	4.88E-06	0.203	0.174				*XRCC3*	Body
cg24475171	9	132,598,886	0.004618	0.203	0.151	0.00000049	0.114		*C9orf78*	TSS1500
cg01495509	5	169,816,378	0.00194	0.203	0.186	1.11E-12	0.150		*KCNIP1*	Body
cg09219093	3	184,099,259	1.09E-08	0.203	0.218				*CHRD*	Body
cg16848712	12	46,767,747	0.000594	0.203	0.202				*SLC38A2*	TSS1500
cg03266453	2	119,606,055	0.001692	0.203	0.234	0.00289955	0.068		*EN1*	TSS1500
cg04732324	11	68,564,292	9.41E-10	0.203	0.194				*CPT1A*	Body
cg07278047	12	99,090,758	7.19E-13	0.203	0.191				*APAF1*	Body
cg07984286	16	54,317,760	0.016104	0.203	0.207				*IRX3*	Body
cg17694351	7	27,252,737	6.1E-05	0.203	0.176					
cg23260993	2	56,149,464	8.66E-08	0.203	0.209				*EFEMP1*	Body
cg07917609	5	33,938,225	0.00027	0.203	0.175				*RXFP3*	1stExon
cg16583987	1	44,509,575	3.23E-05	0.203	0.176					
cg18016365	4	47,033,634	0.045577	0.203	0.277	1.44E-21	0.176		*GABRB1*	1stExon
cg18875453	22	50,517,795	2.37E-08	0.203	0.193				*MLC1*	Body
cg24811864	18	55,019,711	0.007735	0.203	0.175				*ST8SIA3*	TSS200
cg09544050	8	143,580,965	0.013314	0.203	0.240				*BAI1*	Body
cg26440261	7	229,594	9.27E-07	0.203	0.224				*FAM20C*	Body

cg08420923	16	89,436,447	5.09E-09	0.203	0.191				*ANKRD11*	5'UTR
cg14367229	13	28,367,741	0.001043	0.203	0.164				*GSX1*	Body
cg23836163	2	157,393,600	3.32E-05	0.203	0.173				*GPD2*	Body
cg25413843	2	71,126,691	1.53E-06	0.203	0.182				*VAX2*	TSS1500
cg15856028	10	29,892,129	0.012207	0.203	0.123				*SVIL*	5'UTR
cg27477277	21	45,811,432	0.007897	0.203	0.257				*TRPM2*	Body
cg18153322	1	43,534,145	0.008746	0.203	0.086					
cg00721170	4	74,864,239	0.0481	0.203	0.047				*CXCL5*	1stExon
cg00838423	1	146,552,329	7.31E-05	0.203	0.201					
cg06780726	13	111,978,350	0.000618	0.203	0.167				*C13orf16*	Body
cg09858862	1	23,887,514	2.73E-14	0.203	0.211				*ID3*	TSS1500
cg00821731	2	219,744,626	7.78E-10	0.203	0.210				*WNT10A*	TSS1500
cg15790214	6	26,522,325	0.012755	0.203	0.115				*HCG11*	Body
cg16362786	21	45,177,726	0.00082	0.203	0.237				*PDXK*	3'UTR
cg18567041	19	2,654,246	4.26E-06	0.203	0.215				*GNG7*	5'UTR
cg03473518	13	20,805,656	1.9E-05	0.203	0.169	0.00103223	0.038		*GJB6*	5'UTR
cg12913879	2	85,362,480	0.000145	0.203	0.198				*TCF7L1*	Body
cg24860371	2	36,779,825	3.9E-21	0.203	0.185				*FEZ2*	3'UTR
cg25133376	7	73,392,981	1.16E-06	0.203	0.199					
cg13636583	9	86,756,031	0.014	0.203	0.133					
cg14807565	10	94,179,972	9.57E-08	0.203	0.195					
cg19453488	14	95,330,984	9.74E-05	0.203	0.205					
cg26425313	10	102,981,446	6.62E-06	0.203	0.182					
cg13099429	14	70,827,109	0.010209	0.203	0.245				*COX16*	TSS1500
cg20048045	10	73,770,523	1.94E-09	0.203	0.209				*CHST3*	3'UTR
cg06821199	4	779,230	3.56E-07	0.202	0.194				*CPLX1*	3'UTR
cg08446038	5	52,327,649	0.01133	0.202	0.284				*ITGA2*	Body
cg08961893	16	88,966,466	1.07E-15	0.202	0.198				*CBFA2T3*	Body
cg13765939	22	27,053,250	0.029817	0.202	0.220				*MIAT*	TSS1500
cg09857513	7	120,969,044	4.92E-05	0.202	0.170				*WNT16*	TSS200
cg11057205	8	134,588,243	7.43E-07	0.202	0.216					
cg20473453	X	15,683,007	0.000196	0.202	0.163	2.5E-11	0.176		*TMEM27*	5'UTR
cg23024326	6	36,761,843	1.6E-06	0.202	0.172				*CPNE5*	Body
cg24999727	4	88,243,818	8.75E-13	0.202	0.202	0.0000262	0.096		*HSD17B13*	1stExon

cg07868721	8	117,950,348	0.021567	0.202	0.139				*C8orf85*	TSS200
cg11285284	6	30,709,012	4.02E-11	0.202	0.162				*FLOT1*	Body
cg13329217	11	63,975,481	3.19E-05	0.202	0.210				*FERMT3*	Body
cg15989892	4	41,063,747	3.76E-05	0.202	0.211				*APBB2*	5'UTR
cg11607648	3	116,936,427	1.31E-08	0.202	0.219					
cg14843341	6	42,072,683	1.94E-07	0.202	0.190				*C6orf132*	Body
cg24384120	10	7,594,027	6.72E-11	0.202	0.202					
cg09264782	10	78,004,239	2.17E-09	0.202	0.183				*C10orf11*	Body
cg10327428	8	39,172,022	0.009421	0.202	0.215				*ADAM5P*	TSS200
cg13867326	X	13,821,440	6.76E-10	0.202	0.191				*GPM6B*	Body
cg16291048	1	153,507,498	1.16E-07	0.202	0.217				*S100A6*	Body
cg22745397	4	42,122,161	1.7E-08	0.202	0.197				*BEND4*	Body
cg10805850	13	98,794,470	0.002031	0.202	0.099				*FARP1*	TSS1500
cg11098259	15	58,430,391	3.84E-08	0.202	0.193	4.85E-15	0.182		*AQP9*	TSS200
cg27627803	17	20,744,682	0.000309	0.202	0.165					
cg17055736	11	113,743,320	3.06E-09	0.202	0.167				*USP28*	Body
cg03295417	16	14,397,892	7.13E-06	0.202	0.223				*MIR193B*	Body
cg08005660	12	47,226,005	0.02943	0.202	0.154					
cg09125999	11	117,534,685	0.001813	0.202	0.164				*DSCAML1*	Body
cg12363903	5	134,526,269	3.56E-15	0.202	0.202					
cg14637649	17	81,057,526	2.56E-05	0.202	0.132					
cg14678084	7	127,627,251	1.27E-14	0.202	0.202				*SND1*	Body
cg18128976	15	40,355,992	6.7E-12	0.202	0.175					
cg20168849	3	188,995,030	9.2E-08	0.202	0.203				*TPRG1*	Body
cg06471596	7	101,579,936	0.011802	0.202	0.166				*CUX1*	Body
cg07166795	1	44,351,061	1.67E-13	0.202	0.197				*ST3GAL3*	Body
cg23841186	12	53,492,662	5.01E-07	0.202	0.205	3.31E-08	0.112		*IGFBP6*	Body
cg26736812	17	48,042,353	0.011804	0.202	0.272					
cg05045335	1	8,457,417	0.002159	0.202	0.138				*RERE*	5'UTR
cg07914856	17	77,788,484	2.64E-07	0.202	0.210					
cg09916651	12	111,620,313	1.6E-08	0.202	0.201				*CUX2*	Body
cg19192626	17	38,708,638	3.1E-06	0.202	0.202					
cg02595832	10	129,535,786	0.031041	0.202	0.210				*FOXI2*	1stExon
cg01069159	5	161,275,865	0.014649	0.202	0.229				*GABRA1*	5'UTR

cg03330485	8	21,647,596	5.08E-05	0.202	0.197				*GFRA2*	TSS1500
cg01931222	12	54,623,795	4.81E-10	0.202	0.187					
cg08829841	10	129,535,348	1.53E-06	0.202	0.215				*FOXI2*	TSS200
cg23657809	1	43,847,821	1.7E-07	0.202	0.186					
cg00030047	1	6,268,790	1.1E-06	0.202	0.181				*RNF207*	Body
cg00766220	5	72,598,865	0.001845	0.202	0.143					
cg17801864	20	45,179,345	1.38E-08	0.202	0.178				*C20orf123*	TSS200
cg26254045	19	38,886,658	5.03E-10	0.202	0.189				*SPRED3*	Body
cg13286281	22	21,386,999	0.042827	0.202	0.060				*SLC7A4*	TSS200
cg18892485	7	129,417,053	1.03E-07	0.202	0.191					
cg20421446	6	5,994,588	0.008824	0.202	0.194					
cg00451194	16	69,141,250	0.032139	0.202	0.219				*HAS3*	5'UTR
cg05597624	1	44,874,524	0.019236	0.202	0.194				*RNF220*	5'UTR
cg08168580	1	27,729,992	8.3E-09	0.202	0.188					
cg16616918	17	1,686,627	0.010647	0.202	0.123				*SMYD4*	Body
cg19389953	7	121,939,827	0.003345	0.202	0.182					
cg01064725	1	204,461,714	9.47E-10	0.202	0.176					
cg01881182	16	8,806,531	9.25E-06	0.202	0.232				*ABAT*	5'UTR
cg09586183	10	102,890,514	0.001641	0.202	0.203				*TLX1*	TSS1500
cg05026884	X	40,032,180	0.000186	0.202	0.173				*BCOR*	5'UTR
cg14640149	2	216,546,226	0.000653	0.202	0.234					
cg01335980	16	27,733,133	3.41E-08	0.202	0.199				*KIAA0556*	Body
cg04743876	7	130,013,617	0.028702	0.202	0.143					
cg05110133	20	3,220,565	0.011037	0.202	0.173					
cg10655471	19	41,025,339	9.77E-05	0.202	0.155				*SPTBN4*	Body
cg15193228	22	30,855,332	8.05E-05	0.202	0.161				*SEC14L3*	3'UTR
cg19639898	1	147,826,639	4.31E-05	0.202	0.174					
cg24998096	5	115,912,261	0.004538	0.202	0.132					
cg06821999	14	95,238,156	1.55E-05	0.202	0.186					
cg08285151	7	18,534,872	0.009335	0.202	0.200				*HDAC9*	TSS1500
cg08972954	5	156,680,241	0.000318	0.202	0.178				*ITK*	3'UTR
cg16987552	1	231,015,123	1.15E-07	0.202	0.222					
cg23766360	12	6,193,688	2.27E-11	0.202	0.203				*VWF*	Body
cg10227024	17	11,234,662	0.000153	0.202	0.238				*SHISA6*	Body

cg13166642	10	134,645,995	0.042181	0.202	0.171					
cg14560430	3	32,863,175	0.001691	0.202	0.183				*TRIM71*	Body
cg15380508	5	166,874,870	0.011361	0.202	0.210				*ODZ2*	Body
cg13194425	17	7,341,936	0.0014	0.202	0.189				*FGF11*	TSS1500
cg16273943	8	38,175,684	2.15E-09	0.202	0.181				*WHSC1L1*	Body
cg21078322	6	134,491,163	8.29E-16	0.202	0.203				*SGK1*	3'UTR
cg22061628	2	172,966,180	0.00036	0.202	0.176				*DLX2*	Body
cg03100044	7	29,519,450	1.7E-07	0.202	0.190				*CHN2*	TSS200
cg08143343	12	85,307,152	0.039812	0.202	0.251				*SLC6A15*	TSS1500
cg09916157	8	23,584,760	0.003625	0.202	0.156					
cg12415479	3	129,311,698	3.54E-08	0.202	0.213				*PLXND1*	Body
cg04141948	7	1,023,156	1.24E-08	0.202	0.183				*CYP2W1*	Body
cg07938743	2	63,283,939	6.96E-05	0.202	0.207				*OTX1*	3'UTR
cg16772514	17	43,046,872	5.44E-10	0.202	0.170	0.03906922	0.026		*C1QL1*	TSS1500
cg17177536	10	19,778,008	8.23E-07	0.202	0.198					
cg08327269	2	182,545,555	0.036652	0.202	0.158				*NEUROD1*	TSS200
cg02288165	20	3,687,992	3.84E-07	0.202	0.243	2.34E-12	0.144	Yes	*SIGLEC1*	TSS1500
cg27136994	15	81,426,534	0.010476	0.202	0.108				*C15orf26*	TSS200
cg00719224	X	133,681,567	0.00604	0.202	0.239				*MIR424*	TSS1500
cg08898055	5	179,597,395	2.19E-07	0.202	0.196				*RASGEF1C*	5'UTR
cg20178764	5	137,609,835	0.000187	0.202	0.193	0.00000732	0.071		*GFRA3*	Body
cg21342728	22	41,075,673	0.000225	0.202	0.213	1.85E-14	0.179		*MCHR1*	1stExon
cg05214460	10	111,766,841	0.000305	0.202	0.169				*ADD3*	5'UTR
cg06895640	11	93,277,191	0.000807	0.202	0.131				*C11orf75*	TSS1500
cg20372821	17	40,337,892	6.06E-08	0.202	0.216				*HCRT*	TSS1500
cg26500142	6	137,811,589	4.41E-06	0.202	0.197					
cg27180671	17	65,527,566	0.016068	0.202	0.131				*PITPNC1*	Body
cg03129884	2	95,692,727	2.36E-07	0.202	0.212				*MAL*	Body
cg06016427	4	71,862,187	2.64E-10	0.202	0.199				*DCK*	Body
cg11264878	10	47,004,362	0.000386	0.202	0.140					
cg27353899	3	195,488,922	0.00011	0.202	0.197				*MUC4*	Body
cg00018198	2	65,297,247	3.5E-12	0.202	0.206				*CEP68*	Body
cg06765217	14	38,091,644	8.36E-05	0.202	0.207					
cg11602901	3	62,354,878	0.013175	0.202	0.311					

cg16320249	17	46,719,761	0.000218	0.202	0.196					
cg26573518	17	33,700,745	0.009022	0.202	0.246				*SLFN11*	TSS200
cg01734772	7	65,723,664	1.84E-06	0.202	0.195				*TPST1*	Body
cg11915218	17	40,335,849	0.000552	0.202	0.190					
cg24943627	18	35,147,826	0.034017	0.202	0.147					
cg05516489	12	54,359,761	0.003875	0.202	0.287				*HOTAIR*	Body
cg18443893	5	192,103	1.95E-08	0.202	0.191				*LRRC14B*	1stExon
cg04088697	7	96,627,050	0.029408	0.202	0.215				*DLX6AS*	Body
cg05886620	7	1,266,180	0.003393	0.202	0.221					
cg22900476	2	173,647,945	0.007404	0.202	0.092				*RAPGEF4*	Body
cg09255732	1	32,171,050	0.008832	0.202	0.187				*COL16A1*	TSS1500
cg14370448	16	30,103,978	4.61E-05	0.202	0.196	0.0000498	0.068		*YPEL3*	3'UTR
cg02151983	3	194,756,646	2.26E-06	0.202	0.184					
cg09567915	6	29,717,260	0.013015	0.202	0.148				*LOC285830*	TSS1500
cg12418043	5	141,900,671	1.31E-10	0.202	0.209					
cg24459147	21	38,068,581	0.0105	0.202	0.217					
cg02271621	11	1,592,675	0.048117	0.202	0.255	3.35E-13	0.137		*HCCA2*	Body
cg07412254	2	121,223,534	9.98E-09	0.202	0.197	2.75E-17	0.120		*LOC84931*	Body
cg07609788	20	39,995,652	0.00087	0.202	0.147				*EMILIN3*	TSS200
cg13963053	6	170,540,050	1.75E-09	0.202	0.188					
cg20631351	19	711,001	7.47E-12	0.202	0.197				*PALM*	Body
cg14247008	2	242,786,046	0.002113	0.202	0.230					
cg15798862	7	76,129,360	0.000262	0.202	0.179				*DTX2*	Body
cg02215171	4	89,379,156	1.6E-08	0.202	0.172				*HERC5*	Body
cg02221520	4	38,859,706	0.00361	0.202	0.260				*TLR6*	TSS1500
cg10914815	13	99,148,667	1.74E-07	0.202	0.182				*STK24*	Body
cg18597411	7	148,394,542	3.19E-08	0.202	0.186				*CUL1*	TSS1500
cg18749015	6	45,631,363	6.14E-05	0.202	0.174					
cg00078318	2	5,837,429	0.010589	0.202	0.210				*SOX11*	3'UTR
cg13588388	9	138,981,584	2.01E-05	0.202	0.219				*NACC2*	5'UTR
cg16222581	7	926,431	1.14E-10	0.202	0.198				*C7orf20*	Body
cg19794163	X	152,157,977	0.000163	0.202	0.207				*PNMA5*	3'UTR
cg23145794	20	20,433,062	0.003603	0.202	0.211				*RALGAPA2*	Body
cg00692610	17	34,996,066	0.00026	0.202	0.216					

cg04508804	3	45,077,765	0.048513	0.202	0.259					
cg01803759	1	27,831,295	7.17E-14	0.202	0.198					
cg18169095	1	201,353,512	3.13E-08	0.202	0.190				*LAD1*	Body
cg18943588	10	7,517,833	2.18E-08	0.202	0.181					
cg26336935	17	39,769,213	0.000223	0.202	0.207				*KRT16*	TSS200
cg02442640	17	75,451,779	5.03E-12	0.202	0.179			Yes	*SEPT9*	Body
cg09807817	14	50,490,901	7.4E-18	0.202	0.222					
cg04515200	5	135,415,762	0.001635	0.202	0.141					
cg05620980	7	562,844	0.005741	0.202	0.232					
cg09538651	11	70,001,896	0.001285	0.202	0.205				*ANO1*	Body
cg13966710	1	113,392,781	0.008154	0.202	0.180	6.94E-12	0.120			
cg17081976	2	24,347,010	0.000603	0.202	0.132				*LOC375190*	5'UTR
cg05421688	1	179,711,859	0.038155	0.202	0.175	9.2E-11	0.138		*FAM163A*	TSS1500
cg06851753	X	153,745,734	5.07E-09	0.202	0.226				*FAM3A*	TSS1500
cg15665400	2	133,428,652	0.001479	0.202	0.220				*LYPD1*	TSS200
cg18148179	3	181,428,298	8.68E-06	0.202	0.177				*SOX2OT*	Body
cg09888283	1	2,211,524	3.32E-06	0.202	0.219				*SKI*	Body
cg18520238	11	36,422,557	0.000174	0.202	0.160				*PRR5L*	5'UTR
cg21307484	22	37,546,220	2.29E-05	0.202	0.162				*IL2RB*	TSS1500
cg12210255	7	72,838,671	4.75E-05	0.202	0.185					
cg23508813	3	137,488,094	0.011223	0.202	0.207					
cg21466107	7	42,136,966	9.09E-13	0.202	0.190				*GLI3*	Body
cg22450406	2	235,422,165	4.05E-15	0.202	0.182					
cg00743269	17	60,747,654	1.68E-06	0.202	0.215				*MRC2*	Body
cg06650255	5	139,078,529	2.73E-07	0.202	0.205					
cg19963856	17	33,759,573	0.049138	0.202	0.170				*SLFN12*	TSS200
cg00021242	15	29,773,425	2.11E-09	0.202	0.181				*FAM189A1*	Body
cg07761358	X	152,991,647	1.18E-12	0.202	0.215				*BCAP31*	TSS1500
cg14831622	17	11,148,761	0.003129	0.202	0.141				*SHISA6*	Body
cg17885794	1	176,521,193	0.002507	0.202	0.167				*PAPPA2*	5'UTR
cg21013395	17	73,131,789	2.07E-07	0.202	0.193				*HN1*	3'UTR
cg26985149	17	40,464,935	2.16E-13	0.202	0.177					
cg04366300	11	125,157,962	5.17E-11	0.202	0.186				*PKNOX2*	5'UTR
cg00808555	17	37,323,734	1.18E-08	0.202	0.205				*ARL5C*	TSS1500

cg02035605	12	1,725,867	5.8E-10	0.202	0.184				*WNT5B*	TSS1500
cg02709032	19	33,699,683	6.84E-07	0.202	0.196				*SLC7A10*	3'UTR
cg10475970	5	140,772,512	0.000127	0.202	0.177				*PCDHGA4*	Body
cg26298967	6	108,444,791	5.97E-05	0.202	0.198					
cg05447343	17	19,648,925	3.3E-05	0.202	0.184				*ALDH3A1*	5'UTR
cg17826956	1	77,230,619	1.89E-05	0.202	0.142					
cg18177731	1	19,249,376	5.69E-13	0.202	0.194				*IFFO2*	Body
cg23028772	1	162,601,485	2.6E-07	0.202	0.197				*DDR2*	TSS1500
cg00240732	7	70,923,396	0.00016	0.202	0.118				*WBSCR17*	Body
cg02019774	3	138,658,470	0.043051	0.202	0.268					
cg07291445	7	39,015,928	0.016605	0.202	0.312					
cg09884076	5	72,595,434	0.009732	0.202	0.185					
cg10159951	11	35,441,881	4.46E-05	0.202	0.199				*SLC1A2*	TSS1500
cg21149983	20	61,038,949	1.94E-11	0.202	0.205				*GATA5*	3'UTR
cg00738934	8	78,996,279	0.017897	0.202	0.178					
cg07737503	19	1,132,895	7.32E-07	0.202	0.182				*SBNO2*	Body
cg09975867	1	18,958,324	0.001829	0.202	0.241				*PAX7*	Body
cg26154714	1	27,318,618	1.45E-05	0.202	0.203					
cg01884549	12	116,997,308	2.46E-07	0.202	0.163				*MAP1LC3B2*	5'UTR
cg10380328	4	188,953,126	0.000152	0.202	0.193					
cg01230067	6	31,239,063	0.000579	0.202	0.183				*HLA-C*	Body
cg13121428	14	76,446,161	1.47E-09	0.202	0.197				*TGFB3*	Body
cg02795515	17	37,381,273	0.01719	0.202	0.294				*STAC2*	Body
cg13223682	2	174,889,929	1.48E-06	0.202	0.190					
cg17244980	10	49,664,616	2.64E-05	0.202	0.192				*ARHGAP22*	Body
cg24792289	5	134,825,895	7.13E-06	0.202	0.189					
cg08115618	8	134,667,555	2.68E-08	0.202	0.165					
cg17129576	17	27,508,027	4.44E-18	0.202	0.200				*MYO18A*	TSS1500
cg10504000	11	2,402,391	8.2E-09	0.202	0.208				*CD81*	Body
cg20824939	1	205,255,082	3.59E-06	0.202	0.185					
cg22630733	11	334,833	3.84E-15	0.202	0.195					
cg01720455	2	176,948,343	0.002136	0.202	0.189				*EVX2*	1stExon
cg05951084	12	54,343,681	0.024087	0.202	0.105					
cg08619378	7	45,616,358	1.92E-10	0.202	0.188				*ADCY1*	Body

cg11877061	2	71,133,502	5.13E-08	0.202	0.196				*VAX2*	Body
cg26189139	8	22,560,972	0.000722	0.202	0.165					
cg08750510	5	32,537,617	2.29E-05	0.202	0.178					
cg16581347	6	114,662,739	3.79E-05	0.202	0.212					
cg16896911	19	33,895,690	8.13E-06	0.202	0.226				*PEPD*	Body
cg17995050	7	55,073,248	5.25E-10	0.202	0.172					
cg02295872	1	228,131,977	1.5E-12	0.202	0.203				*WNT9A*	Body
cg21410991	5	50,678,451	0.015312	0.202	0.146	3.96E-07	0.131		*ISL1*	TSS1500
cg09310092	19	35,529,201	2.61E-07	0.202	0.201				*SCN1B*	Body
cg15177855	11	70,517,411	0.000359	0.202	0.185				*SHANK2*	Body
cg04678315	12	125,212,010	1.62E-05	0.202	0.174					
cg14389122	15	74,945,851	0.002275	0.202	0.150				*EDC3*	Body
cg19867250	1	225,648,715	1.6E-08	0.202	0.222					
cg22674789	10	81,740,565	3.89E-06	0.202	0.162					
cg25344239	1	68,326,786	7.93E-08	0.202	0.174					
cg02854229	14	101,962,994	9.29E-09	0.202	0.209					
cg03126058	1	3,282,757	0.001059	0.202	0.225				*PRDM16*	Body
cg09102864	11	68,780,892	0.021823	0.202	0.266				*MRGPRF*	TSS200
cg12074541	2	1,286,641	0.000567	0.202	0.133				*SNTG2*	Body
cg21579710	7	69,238,693	7.12E-09	0.202	0.203				*AUTS2*	Body
cg25500444	11	86,086,101	0.000267	0.202	0.241	4.33E-08	0.148		*CCDC81*	1stExon
cg19676285	4	7,967,275	0.001745	0.202	0.153				*ABLIM2*	3'UTR
cg26822097	22	17,082,581	0.026764	0.202	0.273				*psiTPTE22*	TSS1500
cg02927682	1	54,844,424	1.51E-10	0.202	0.192				*SSBP3*	Body
cg10436410	7	114,561,988	4.94E-11	0.202	0.191				*MDFIC*	TSS1500
cg26837043	19	8,407,675	3.3E-10	0.202	0.165				*KANK3*	5'UTR
cg15744134	15	32,933,835	1.5E-11	0.202	0.154				*SCG5*	TSS200
cg16731811	20	21,085,004	0.001141	0.202	0.199					
cg06679694	5	72,510,954	0.021722	0.202	0.217					
cg11853788	1	179,561,347	8.17E-07	0.202	0.195				*TDRD5*	5'UTR
cg12924402	2	218,898,511	0.02295	0.202	0.266			Yes		
cg15964018	1	19,490,180	7.24E-07	0.202	0.204				*UBR4*	Body
cg04773827	20	55,201,655	0.000348	0.201	0.184					
cg16206813	5	3,122,162	0.001531	0.201	0.214					

cg26130581	17	42,449,613	0.003887	0.201	0.253				*ITGA2B*	3'UTR
cg01218206	11	116,933,977	2.7E-05	0.201	0.187				*SIK3*	Body
cg04100337	17	71,336,777	2.33E-06	0.201	0.197				*SDK2*	Body
cg17894318	3	181,441,384	6.84E-06	0.201	0.182				*SOX2OT*	Body
cg19980151	5	92,914,290	1.09E-05	0.201	0.181				*FLJ42709*	Body
cg21101465	13	28,493,404	0.004858	0.201	0.252				*PDX1*	TSS1500
cg13008315	19	42,785,474	0.00014	0.201	0.173					
cg02636004	3	119,747,339	5.79E-18	0.201	0.185				*GSK3B*	Body
cg04045544	16	27,298,091	4.2E-08	0.201	0.211					
cg07115767	7	19,146,255	0.001426	0.201	0.199					
cg22070406	18	55,019,708	0.002186	0.201	0.162				*ST8SIA3*	TSS200
cg22958315	2	39,295,686	1.35E-05	0.201	0.223				*SOS1*	Body
cg23723105	1	68,150,193	0.005815	0.201	0.126				*GADD45A*	TSS1500
cg00643651	1	245,848,375	5.42E-16	0.201	0.209				*KIF26B*	Body
cg12011977	10	122,215,735	0.002289	0.201	0.094				*PPAPDC1A*	TSS1500
cg16438686	11	75,968,191	0.003632	0.201	0.193					
cg16945312	6	1,393,028	0.03812	0.201	0.258				*FOXF2*	Body
cg25340688	5	135,416,398	0.000156	0.201	0.173				*MIR886*	TSS200
cg26424888	11	74,037,163	6.41E-12	0.201	0.187					
cg21920775	1	114,696,641	0.02391	0.201	0.184				*SYT6*	TSS200
cg04189328	14	24,505,653	4.74E-16	0.201	0.204				*DHRS4L1*	TSS200
cg24358395	4	139,580,260	3.77E-08	0.201	0.200					
cg05490591	17	55,648,137	3.48E-07	0.201	0.199				*MSI2*	Body
cg05841929	15	59,730,852	0.004153	0.201	0.131				*FAM81A*	5'UTR
cg07672051	5	149,887,787	4.5E-12	0.201	0.183				*NDST1*	5'UTR
cg12718582	8	1,312,875	1.57E-07	0.201	0.209					
cg07313334	1	144,533,861	0.000118	0.201	0.242					
cg18492126	21	22,369,802	0.001535	0.201	0.181				*NCAM2*	TSS1500
cg21786808	1	84,227,630	5.32E-09	0.201	0.182					
cg01712452	20	56,736,072	5.81E-11	0.201	0.192				*C20orf85*	3'UTR
cg09351315	17	77,775,823	6.75E-07	0.201	0.214					
cg09403666	10	23,461,493	0.000598	0.201	0.218					
cg11081272	11	78,130,236	1.48E-05	0.201	0.231				*GAB2*	TSS1500
cg14378057	6	42,788,563	2.46E-11	0.201	0.205	0.00023513	0.050		*KIAA0240*	TSS1500

cg20403938	12	54,377,457	5E-07	0.201	0.156				*HOXC10*	TSS1500
cg27302675	6	90,385,873	1.16E-07	0.201	0.223				*MDN1*	Body
cg06572160	19	50,831,901	0.003828	0.201	0.188	1.67E-08	0.087		*KCNC3*	1stExon
cg03182688	17	33,825,300	0.000368	0.201	0.203				*SLFN12L*	Body
cg09178261	1	16,482,818	0.00043	0.201	0.159				*EPHA2*	TSS1500
cg18022893	1	44,951,225	1.4E-06	0.201	0.184				*RNF220*	Body
cg19103219	10	101,287,059	0.022605	0.201	0.152					
cg23181580	11	22,454,301	0.000389	0.201	0.192					
cg03536711	1	221,509,067	0.041675	0.201	0.127				*LOC400804*	Body
cg08472583	7	65,580,288	0.001094	0.201	0.180	3.84E-12	0.139		*CRCP*	Body
cg13522244	7	116,164,762	0.028948	0.201	0.160				*CAV1*	TSS200
cg16315376	1	202,679,762	0.01233	0.201	0.173				*SYT2*	TSS1500
cg14190522	9	124,344,221	0.00233	0.201	0.198				*DAB2IP*	Body
cg05957749	1	39,875,787	0.000704	0.201	0.189				*KIAA0754*	5'UTR
cg09850980	5	36,067,127	0.000173	0.201	0.224				*UGT3A2*	TSS200
cg14793287	12	48,578,573	0.000122	0.201	0.197				*C12orf68*	3'UTR
cg07355514	16	23,420,385	4.57E-07	0.201	0.228				*COG7*	Body
cg12528056	11	60,619,955	2.71E-24	0.201	0.179				*GPR44*	3'UTR
cg13835436	11	69,809,807	5.3E-12	0.201	0.197					
cg16398581	1	59,986,591	4.69E-09	0.201	0.188				*FGGY*	Body
cg23563927	10	134,755,679	0.006342	0.201	0.247				*C10orf93*	Body
cg25218351	2	27,505,446	4.39E-09	0.201	0.187	2.49E-07	0.047	Yes	*TRIM54*	TSS200
cg06770532	7	42,214,276	7.57E-06	0.201	0.160				*GLI3*	Body
cg07083818	19	18,385,672	0.015511	0.201	0.267				*KIAA1683*	TSS1500
cg13358623	X	15,402,593	1.61E-11	0.201	0.200				*FIGF*	TSS200
cg20434856	5	16,713,588	9.66E-07	0.201	0.187				*MYO10*	Body
cg26651148	7	157,867,300	7.71E-08	0.201	0.188				*PTPRN2*	Body
cg22283925	4	188,918,218	0.002353	0.201	0.188				*ZFP42*	5'UTR
cg25006077	3	152,176,018	2.97E-09	0.201	0.210				*MBNL1*	Body
cg25312372	2	129,018,482	1.96E-07	0.201	0.161					
cg01446393	3	58,563,609	2.17E-05	0.201	0.239	0.0000607	0.095		*FAM107A*	TSS200
cg04359840	16	17,563,300	1.61E-09	0.201	0.194				*XYLT1*	Body
cg11560654	13	27,317,117	9.09E-08	0.201	0.199					
cg01959435	22	39,759,957	1.56E-09	0.201	0.222				*SYNGR1*	TSS1500

cg02856124	3	112,736,847	8.1E-09	0.201	0.201				*C3orf17*	Body
cg14254767	9	71,819,910	1.28E-07	0.201	0.219				*TJP2*	TSS200
cg22180580	6	138,867,046	3.53E-07	0.201	0.191				*NHSL1*	Body
cg25899829	11	75,046,147	2.01E-16	0.201	0.213				*ARRB1*	Body
cg00024086	7	156,814,491	0.011927	0.201	0.208					
cg04590434	16	66,369,967	6.53E-08	0.201	0.201					
cg01640606	14	61,119,891	0.000788	0.201	0.216					
cg03157182	12	132,655,797	0.000165	0.201	0.184					
cg11944757	10	31,422,942	0.022142	0.201	0.242					
cg00807586	3	38,080,790	0.04096	0.201	0.120	0.00055949	0.076		*DLEC1*	1stExon
cg08703055	1	2,232,481	7.05E-05	0.201	0.204				*SKI*	Body
cg26544742	6	27,533,825	0.030233	0.201	0.209					
cg04468543	5	77,268,331	0.003247	0.201	0.177					
cg11656547	9	72,658,747	0.005187	0.201	0.187	7.6E-18	0.160		*MAMDC2*	1stExon
cg08379987	13	31,507,139	5.8E-05	0.201	0.214				*C13orf26*	Body
cg08462030	6	167,033,966	3.96E-07	0.201	0.203				*RPS6KA2*	Body
cg09921610	4	41,259,866	0.024411	0.201	0.194				*UCHL1*	Body
cg21839338	16	10,912,331	0.013432	0.201	0.242				*FAM18A*	Body
cg04986504	11	12,031,266	0.001759	0.201	0.118				*DKK3*	TSS1500
cg20505457	14	69,388,953	0.001228	0.201	0.196				*ACTN1*	Body
cg26862316	19	40,792,613	3.1E-11	0.201	0.197				*AKT2*	TSS1500
cg02172312	16	62,069,337	0.017638	0.201	0.235				*CDH8*	5'UTR
cg14065857	2	33,436,054	4.91E-05	0.201	0.221				*LTBP1*	Body
cg18894552	14	53,478,112	3.3E-08	0.201	0.199					
cg24724689	2	11,087,224	8.53E-07	0.201	0.189					
cg09700701	11	65,816,819	0.000184	0.201	0.134				*GAL3ST3*	TSS200
cg23572944	11	60,720,130	3.41E-06	0.201	0.200				*SLC15A3*	TSS1500
cg01845111	20	31,446,967	8.55E-09	0.201	0.207				*EFCAB8*	5'UTR
cg20482760	11	78,725,125	1.94E-13	0.201	0.182				*ODZ4*	Body
cg20606255	20	36,839,442	0.002081	0.201	0.251			Yes	*KIAA1755*	3'UTR
cg09499787	10	118,900,492	0.016092	0.201	0.214					
cg24109116	7	99,725,975	3.39E-07	0.201	0.155				*MBLAC1*	3'UTR
cg01102046	17	19,801,249	5.12E-12	0.201	0.207					
cg05364567	17	5,019,761	0.020223	0.201	0.238				*ZNF232*	5'UTR

cg07142797	10	50,605,613	0.040552	0.201	0.264					
cg07546139	5	92,907,553	0.003061	0.201	0.086				*FLJ42709*	TSS1500
cg23251746	14	92,047,329	0.002082	0.201	0.197				*CATSPERB*	Body
cg24430580	4	111,544,235	0.010717	0.201	0.123				*PITX2*	1stExon
cg27212234	19	36,048,757	0.033811	0.201	0.165				*ATP4A*	Body
cg00336800	19	1,450,386	0.034606	0.201	0.168				*APC2*	5'UTR
cg11576274	1	146,552,552	2.97E-07	0.201	0.200					
cg00292701	4	10,463,590	0.005472	0.201	0.192					
cg06410746	8	63,161,676	0.002031	0.201	0.110				*NKAIN3*	1stExon
cg15996459	17	184,106	0.004566	0.201	0.122				*RPH3AL*	5'UTR
cg23195547	11	8,740,646	5.05E-10	0.201	0.189				*ST5*	Body
cg13264840	7	142,494,213	0.000246	0.201	0.221					
cg20379007	12	53,271,818	1.32E-05	0.201	0.188					
cg20449659	11	32,421,752	0.010089	0.201	0.212				*WT1*	Body
cg15757019	4	149,756,665	4.57E-08	0.201	0.191					
cg00478326	15	78,978,136	7.81E-05	0.201	0.184					
cg01390950	6	34,117,273	3.47E-07	0.201	0.194					
cg11072570	13	24,734,180	2.01E-05	0.201	0.163				*SPATA13*	TSS1500
cg19448837	18	48,085,671	0.000427	0.201	0.183				*MAPK4*	TSS1500
cg24733637	19	19,050,451	1.26E-05	0.201	0.169				*HOMER3*	5'UTR
cg05337019	1	16,062,361	1.24E-11	0.201	0.201				*SLC25A34*	TSS1500
cg09423312	7	1,163,549	7.33E-08	0.201	0.200				*C7orf50*	Body
cg03475509	4	85,402,143	0.013932	0.201	0.212					
cg05749351	1	205,897,809	0.000445	0.201	0.178				*SLC26A9*	Body
cg08857729	1	67,217,826	0.001829	0.201	0.191				*TCTEX1D1*	TSS1500
cg10782206	14	76,445,988	1.74E-11	0.201	0.225				*TGFB3*	Body
cg12784175	10	103,589,988	6.06E-06	0.201	0.187				*KCNIP2*	Body
cg03683166	13	113,611,729	5.3E-09	0.201	0.190					
cg05180182	10	3,666,755	3.26E-05	0.201	0.186					
cg07933656	17	20,445,757	0.042147	0.201	0.317					
cg26707664	18	60,278,353	1.11E-05	0.201	0.163					
cg00588853	16	29,624,485	0.002092	0.201	0.127				*SLC7A5P1*	Body
cg03474687	14	104,179,160	0.000474	0.201	0.161				*XRCC3*	5'UTR
cg06683487	16	936,480	3.77E-13	0.201	0.196				*LMF1*	Body

cg09642857	12	133,026,813	1.99E-16	0.201	0.191					
cg06767187	7	2,640,127	2.05E-11	0.201	0.197				*IQCE*	Body
cg08271153	14	69,726,221	0.001379	0.201	0.146				*GALNTL1*	TSS1500
cg18471993	6	100,917,416	2.06E-06	0.201	0.206					
cg27504195	3	113,342,400	1.18E-14	0.201	0.193				*SIDT1*	Body
cg08493294	2	25,500,416	0.003304	0.201	0.229				*DNMT3A*	Body
cg09451215	6	82,459,960	0.00164	0.201	0.246				*FAM46A*	Body
cg04093225	1	166,917,158	0.000833	0.201	0.156				*ILDR2*	Body
cg10521023	1	245,411,583	0.039998	0.201	0.175				*KIF26B*	Body
cg17662774	15	89,582,414	1.24E-09	0.201	0.207					
cg22540489	X	99,930,094	2.38E-10	0.201	0.193				*SYTL4*	3'UTR
cg03806095	8	37,655,840	0.01886	0.201	0.237				*GPR124*	Body
cg03485172	13	95,655,579	0.012539	0.201	0.070					
cg04745805	17	40,427,474	0.004708	0.201	0.158	0.00867966	0.033		*STAT5B*	5'UTR
cg27547291	2	201,451,026	3.18E-06	0.201	0.186				*AOX1*	Body
cg04494873	5	72,677,287	0.034378	0.201	0.182					
cg11794956	5	92,443,321	6.32E-08	0.201	0.184					
cg01856887	6	36,812,436	1.99E-09	0.201	0.212					
cg04834436	16	55,362,754	2.72E-05	0.201	0.200				*IRX6*	Body
cg16668359	2	45,159,434	1.47E-06	0.201	0.197					
cg21823119	1	209,601,066	0.003612	0.201	0.087				*LOC642587*	TSS1500
cg02136452	16	49,872,492	9.21E-05	0.201	0.166					
cg11282156	3	63,428,840	0.00311	0.201	0.144				*SYNPR*	5'UTR
cg17219326	3	44,517,846	0.015124	0.201	0.239				*ZNF445*	5'UTR
cg25938806	2	177,028,162	0.007604	0.201	0.225				*HOXD3*	TSS1500
cg01113339	7	1,660,895	0.009416	0.201	0.253					
cg02230811	14	56,967,892	0.000448	0.201	0.194					
cg08254359	12	54,398,518	0.003925	0.201	0.225					
cg25383479	6	108,491,702	0.014767	0.201	0.244				*NR2E1*	Body
cg00615241	19	839,689	0.001943	0.201	0.256				*PRTN3*	TSS1500
cg14283328	11	66,823,587	9.82E-07	0.201	0.158				*RHOD*	TSS1500
cg23418968	2	9,816,010	4.66E-07	0.201	0.205					
cg21867733	12	124,985,584	9.25E-06	0.201	0.217				*NCOR2*	5'UTR
cg06135285	17	19,675,785	0.00454	0.201	0.153				*ULK2*	3'UTR

cg17692403	20	42,964,959	1.28E-06	0.201	0.204	5.63E-25	0.175		*R3HDML*	TSS1500
cg09884480	11	1,967,966	3.85E-10	0.201	0.199				*MRPL23*	TSS1500
cg10205038	14	93,052,501	7.87E-06	0.201	0.177				*RIN3*	Body
cg11599864	6	38,145,464	2.67E-18	0.201	0.187				*BTBD9*	Body
cg13860928	1	209,915,875	2.6E-11	0.201	0.187					
cg05281894	1	13,910,698	0.049988	0.201	0.386				*PDPN*	TSS1500
cg05708114	9	34,624,587	0.00021	0.201	0.231				*ARID3C*	Body
cg08045932	20	61,659,980	0.047051	0.201	0.220					
cg16623710	22	45,073,049	1.19E-05	0.201	0.222				*PRR5*	5'UTR
cg18282388	11	129,716,470	0.003314	0.201	0.123				*TMEM45B*	5'UTR
cg22761176	2	173,539,542	0.000832	0.201	0.141					
cg07077013	2	177,025,198	0.001602	0.201	0.164					
cg10625931	12	57,632,417	0.000147	0.201	0.209				*NDUFA4L2*	5'UTR
cg02268171	13	24,477,610	0.00039	0.201	0.163					
cg06704518	8	11,565,530	0.026352	0.201	0.253				*GATA4*	5'UTR
cg22920300	3	181,441,364	0.001383	0.201	0.201				*SOX2OT*	Body
cg02805028	16	27,899,239	0.000137	0.201	0.167	1.51E-14	0.124		*GSG1L*	Body
cg19247475	5	140,789,745	5.47E-06	0.201	0.212				*PCDHGA4*	Body
cg21667069	11	67,778,641	1.13E-10	0.201	0.198				*ALDH3B1*	5'UTR
cg17200920	7	127,808,798	0.039011	0.201	0.192					
cg01511557	1	50,514,319	2.62E-05	0.201	0.197				*ELAVL4*	Body
cg05746227	5	54,516,830	0.00145	0.201	0.196					
cg02721065	8	120,932,635	2.67E-10	0.201	0.167				*DEPDC6*	Body
cg08523865	2	124,782,253	0.008709	0.201	0.168				*CNTNAP5*	TSS1500
cg09768257	8	11,628,346	2.16E-07	0.201	0.187				*NEIL2*	5'UTR
cg10425245	5	168,454,709	0.006954	0.201	0.128				*SLIT3*	Body
cg23511157	7	123,241,752	0.002396	0.201	0.210					
cg23719713	6	10,586,167	1.17E-11	0.201	0.192				*GCNT2*	Body
cg00346985	1	46,940,516	9.51E-08	0.201	0.208					
cg08008352	16	27,322,356	3.91E-07	0.201	0.183					
cg12623364	4	109,038,130	6.85E-08	0.201	0.187				*LEF1*	Body
cg14394264	11	46,121,221	2.61E-06	0.201	0.210				*PHF21A*	5'UTR
cg16922753	8	1,113,291	5.18E-06	0.201	0.203					
cg25410785	5	3,597,760	0.005182	0.201	0.224				*IRX1*	Body

cg26177041	4	114,389,632	4.47E-07	0.201	0.188				*CAMK2D*	Body
cg02139871	2	127,783,370	0.040513	0.201	0.158					
cg03201044	20	55,203,291	0.000286	0.201	0.166				*TFAP2C*	TSS1500
cg07547765	17	46,685,448	4.88E-08	0.201	0.178				*HOXB7*	Body
cg08891110	15	96,952,425	0.007229	0.201	0.193					
cg22216643	17	74,704,158	1.98E-12	0.201	0.219				*MXRA7*	Body
cg25596082	10	43,626,561	0.000428	0.201	0.208					
cg08027484	6	10,556,523	0.021909	0.201	0.148				*GCNT2*	5'UTR
cg02416510	8	54,791,763	5.51E-05	0.201	0.197				*RGS20*	Body
cg20138186	10	102,642,655	2.46E-09	0.201	0.199					
cg10053507	1	15,307,522	2.45E-08	0.201	0.191				*KIAA1026*	Body
cg10989634	1	111,813,745	4.11E-07	0.201	0.187					
cg05759150	10	124,906,565	0.000347	0.201	0.099				*HMX2*	TSS1500
cg12414301	5	137,224,213	0.041236	0.201	0.127				*PKD2L2*	TSS1500
cg13372879	2	172,948,073	0.029881	0.201	0.135					
cg15721145	20	61,867,205	0.013029	0.201	0.103				*BIRC7*	TSS200
cg15895690	14	60,982,635	4.84E-05	0.201	0.154					
cg03638905	5	72,595,625	0.000167	0.201	0.161					
cg10431747	1	34,642,383	0.003921	0.201	0.142				*C1orf94*	5'UTR
cg05913684	19	710,906	6.01E-22	0.201	0.217				*PALM*	Body
cg14334338	14	75,115,531	0.007659	0.201	0.166					
cg02923300	X	68,759,322	9.88E-14	0.201	0.194					
cg13420848	1	197,888,894	0.004593	0.201	0.258				*LHX9*	Body
cg18318006	10	97,169,147	0.000233	0.201	0.231				*SORBS1*	Body
cg24683041	14	100,413,076	0.000623	0.201	0.190					
cg06428163	13	96,204,856	0.027554	0.201	0.238				*CLDN10*	TSS200
cg07314530	7	162,691	2.3E-10	0.201	0.201					
cg09439204	12	54,090,464	0.000238	0.201	0.122					
cg19644991	5	122,482,336	3.98E-14	0.201	0.169				*PRDM6*	Body
cg21837192	12	54,398,765	0.029438	0.201	0.162					
cg01266287	16	66,959,833	1.28E-08	0.201	0.196				*RRAD*	TSS1500
cg03199745	12	54,454,445	1.7E-10	0.201	0.185				*FLJ12825*	Body
cg15462879	X	133,930,054	0.000117	0.201	0.206				*FAM122B*	Body
cg20491406	1	201,693,818	3.51E-12	0.201	0.196				*NAV1*	Body

cg20841748	17	40,838,078	1.43E-05	0.201	0.211				*CNTNAP1*	Body
cg08753468	7	71,911,541	9.35E-08	0.200	0.190					
cg11539695	6	32,287,314	9.08E-10	0.200	0.182				*C6orf10*	Body
cg14526039	3	49,210,221	3.13E-12	0.200	0.199				*KLHDC8B*	Body
cg21476940	1	160,854,954	0.00367	0.200	0.152	1.91E-13	0.145		*ITLN1*	5'UTR
cg23521507	14	52,519,112	0.010591	0.200	0.206				*NID2*	Body
cg26363759	1	9,377,984	7.67E-25	0.200	0.206				*SPSB1*	5'UTR
cg00509207	12	31,078,979	7.14E-11	0.200	0.200				*TSPAN11*	TSS1500
cg02571636	4	188,953,906	0.003759	0.200	0.230					
cg16324409	4	3,416,370	2.55E-18	0.200	0.196				*RGS12*	Body
cg27346356	19	19,648,914	0.049018	0.200	0.007				*CILP2*	TSS200
cg02539682	12	54,132,444	0.013791	0.200	0.206					
cg09214412	15	93,614,970	1.74E-09	0.200	0.189				*RGMA*	Body
cg15505412	5	3,596,996	0.032389	0.200	0.257				*IRX1*	Body
cg06991732	8	94,934,036	0.035467	0.200	0.246				*PDP1*	5'UTR
cg09578028	2	176,987,411	0.001326	0.200	0.174				*HOXD9*	TSS200
cg17573301	5	72,732,265	0.015127	0.200	0.187					
cg23373640	14	65,696,480	0.000942	0.200	0.188					
cg23689615	15	74,228,839	0.007839	0.200	0.168				*LOXL1*	Body
cg14519952	12	124,491,432	0.000592	0.200	0.179				*ZNF664*	5'UTR
cg15230985	17	78,753,887	3.02E-13	0.200	0.186				*RPTOR*	Body
cg16921727	7	51,539,678	0.002801	0.200	0.183					
cg26817546	5	135,538,704	5.44E-09	0.200	0.194					
cg26869615	11	20,044,639	0.000614	0.200	0.145				*NAV2*	Body
cg27287745	14	61,104,967	0.001736	0.200	0.159					
cg04875821	15	68,253,897	8.35E-11	0.200	0.191					
cg05543030	2	45,005,364	1.72E-07	0.200	0.192					
cg07264048	6	10,556,326	0.018	0.200	0.131				*GCNT2*	5'UTR
cg09258689	12	132,853,954	0.000246	0.200	0.243				*GALNT9*	Body
cg24864203	5	61,612,144	1.88E-13	0.200	0.197				*KIF2A*	Body
cg08841257	8	10,191,002	5.24E-09	0.200	0.164				*MSRA*	Body
cg16747109	4	111,544,463	0.037687	0.200	0.155				*PITX2*	TSS1500
cg20271511	22	17,566,989	0.000185	0.200	0.189				*IL17RA*	Body
cg00512872	7	6,268,584	8.39E-07	0.200	0.181				*CYTH3*	Body

cg01462856	13	108,922,745	1.54E-05	0.200	0.164				*TNFSF13B*	Body
cg07600193	5	1,228,443	0.014423	0.200	0.154				*SLC6A18*	Body
cg11554566	21	47,398,596	0.015767	0.200	0.226					
cg26805263	13	24,212,312	0.000613	0.200	0.182				*TNFRSF19*	Body
cg01478204	11	44,339,330	0.005927	0.200	0.276					
cg03642555	5	34,502,788	2.79E-07	0.200	0.228					
cg14376467	4	86,851,411	0.00056	0.200	0.211				*ARHGAP24*	Body
cg15201175	5	115,889,873	2.51E-07	0.200	0.201			Yes	*SEMA6A*	5'UTR
cg24871089	17	184,131	0.022929	0.200	0.102				*RPH3AL*	5'UTR
cg01593673	17	46,648,014	0.000309	0.200	0.206				*HOXB3*	5'UTR
cg13584882	10	74,836,656	0.010374	0.200	0.210				*P4HA1*	5'UTR
cg13655341	17	79,859,826	0.010816	0.200	0.142				*NPB*	TSS1500
cg05356496	7	107,300,998	0.000199	0.200	0.154				*SLC26A4*	TSS200
cg26924825	16	67,977,865	2.86E-10	0.200	0.201	7.75E-11	0.112		*SLC12A4*	3'UTR
cg02086801	5	76,928,406	0.001566	0.200	0.181				*OTP*	Body
cg24394819	17	78,725,049	7.95E-05	0.200	0.188				*RPTOR*	Body
cg00631329	6	26,305,371	0.005329	0.200	0.239					
cg07709606	2	17,699,243	4.73E-06	0.200	0.218				*RAD51AP2*	1stExon
cg17472111	11	75,287,721	1.25E-07	0.200	0.155					
cg06594404	4	380,396	0.001819	0.200	0.230					
cg12261451	13	22,243,030	5.95E-06	0.200	0.187					
cg16127759	1	110,613,596	2.68E-06	0.200	0.175				*ALX3*	TSS1500
cg00561634	16	54,085,398	5.42E-07	0.200	0.192				*FTO*	Body
cg16263367	19	53,935,096	9.29E-07	0.200	0.209				*ZNF761*	TSS200
cg04959790	11	47,278,977	0.002126	0.200	0.140				*NR1H3*	5'UTR
cg19801921	1	63,784,864	0.035692	0.200	0.266					
cg24067150	6	30,956,397	0.001496	0.200	0.209				*MUC21*	3'UTR
cg25507767	7	138,483,143	2.21E-05	0.200	0.175				*TMEM213*	Body
cg12588880	2	8,597,159	0.024588	0.200	0.196					
cg01803928	13	50,701,619	0.001693	0.200	0.177					
cg02489908	17	6,946,086	0.000126	0.200	0.204				*SLC16A11*	Body
cg04714041	19	35,068,710	0.015316	0.200	0.226				*LOC643719*	TSS200
cg09119776	10	104,387,263	1.72E-06	0.200	0.210				*SUFU*	Body
cg19570154	2	5,513,337	0.013069	0.200	0.196					

cg02241397	1	47,698,081	0.015585	0.200	0.270					
cg03711485	10	101,296,036	0.0007	0.200	0.180				*NKX2-3*	3'UTR
cg16609021	7	157,754,648	0.021393	0.200	0.127				*PTPRN2*	Body
cg16847760	8	28,912,915	2.06E-07	0.200	0.200					
cg08084806	6	28,574,524	0.011328	0.200	0.285					
cg03946955	1	156,096,301	0.00154	0.200	0.250				*LMNA*	Body
cg07721547	22	42,475,119	8.62E-07	0.200	0.178				*C22orf32*	TSS1500
cg11991058	2	168,150,602	0.028567	0.200	0.203					
cg16087826	5	131,654,574	5E-07	0.200	0.210				*SLC22A4*	Body
cg17075808	2	225,774,214	0.000809	0.200	0.143				*DOCK10*	Body
cg02331883	5	140,798,095	0.006128	0.200	0.168				*PCDHGA4*	Body
cg03742238	4	41,748,676	0.027904	0.200	0.267				*PHOX2B*	Body
cg25361506	1	201,426,290	5.8E-09	0.200	0.179					
cg05134015	14	36,973,365	0.012343	0.200	0.240				*SFTA3*	Body
cg17851496	22	46,368,649	2.5E-05	0.200	0.189				*WNT7B*	Body
cg20890210	8	73,450,181	0.001879	0.200	0.201	1.2E-26	0.207		*KCNB2*	5'UTR
cg24129390	13	33,780,205	0.006069	0.200	0.149				*STARD13*	TSS200
cg07962934	8	144,809,621	2.49E-07	0.200	0.167				*FAM83H*	Body
cg09412707	4	26,085,653	0.015767	0.200	0.273					
cg01799681	6	11,043,368	2.33E-05	0.200	0.199				*ELOVL2*	Body
cg18888464	14	76,597,657	0.020463	0.200	0.186					
cg26083180	6	99,272,089	0.042354	0.200	0.270					
cg00025643	1	37,904,694	1.65E-08	0.200	0.181					
cg05268769	14	75,733,932	0.021883	0.200	0.231					
cg08846079	1	110,420,044	1.28E-06	0.200	0.185					
cg10646968	10	111,728,983	1.29E-15	0.200	0.183					
cg18449739	12	113,495,701	5.84E-08	0.200	0.201				*DTX1*	1stExon
cg05572456	2	172,961,504	0.013665	0.200	0.116					
cg07480640	3	64,211,328	1.06E-05	0.200	0.187				*PRICKLE2*	TSS200
cg08637438	17	70,113,856	0.015886	0.200	0.223					
cg20907051	1	47,999,516	1.88E-06	0.200	0.180					
cg11491247	1	155,035,530	0.009097	0.200	0.115				*EFNA4*	TSS1500
cg11510131	12	120,679,618	1.91E-10	0.200	0.201				*PXN*	5'UTR
cg12150256	3	50,359,849	7.24E-08	0.200	0.212				*HYAL2*	5'UTR

cg05928806	7	127,991,614	7.86E-05	0.200	0.203				*PRRT4*	Body
cg18181703	17	76,354,621	1.98E-07	0.200	0.219				*SOCS3*	Body
cg03465513	13	28,550,046	1.03E-05	0.200	0.159					
cg05461270	6	28,574,608	0.030618	0.200	0.213					
cg21982455	5	2,757,976	0.014275	0.200	0.210					
cg04729004	7	128,550,932	2.73E-19	0.200	0.190				*KCP*	TSS200
cg22770911	10	8,101,307	8.67E-08	0.200	0.172				*GATA3*	Body
cg15394142	12	66,275,988	0.04514	0.200	0.264				*HMGA2*	Body
cg02627991	7	74,032,045	0.048997	0.200	0.169					
cg08757574	X	70,273,027	4.08E-09	0.200	0.217					
cg19725377	14	69,535,979	8.31E-08	0.200	0.208				*DCAF5*	Body
cg05813673	4	53,862,521	3.47E-07	0.200	0.198				*SCFD2*	Body
cg18499083	14	91,637,035	0.000825	0.200	0.237				*C14orf159*	Body
cg21723861	17	39,686,628	7.98E-06	0.200	0.160					
cg24382070	8	22,013,694	4.19E-08	0.200	0.179				*LGI3*	Body
cg24841318	17	27,038,686	0.007586	0.200	0.192				*PROCA1*	5'UTR
cg26262055	4	25,090,618	0.001678	0.200	0.160					
cg09764807	10	43,820,676	4E-09	0.200	0.200					
cg12972064	3	171,887,393	9.69E-05	0.200	0.246				*FNDC3B*	Body
cg13942922	2	238,383,665	0.000383	0.200	0.153					
cg09950256	2	64,836,449	0.000224	0.200	0.219					
cg14377922	2	23,840,537	3.27E-05	0.200	0.191				*KLHL29*	Body
cg25109019	3	181,441,345	1.56E-05	0.200	0.190				*SOX2OT*	Body
cg15289427	6	24,911,001	0.007506	0.200	0.144				*FAM65B*	5'UTR
cg16811108	17	7,905,840	5.4E-06	0.200	0.191				*GUCY2D*	TSS200
cg23601468	22	33,200,307	0.002582	0.200	0.229				*SYN3*	Body
cg25656762	5	60,927,728	2.26E-07	0.200	0.174					
cg27314002	3	46,506,519	0.001432	0.200	0.166				*LTF*	TSS200
cg11515095	11	132,949,437	0.014619	-0.200	-0.133				*OPCML*	Body
cg18220087	11	134,783,448	0.002179	-0.200	-0.136					
cg04558386	6	13,815,568	0.000299	-0.200	-0.257				*CCDC90A*	TSS1500
cg05261272	6	137,494,924	0.000184	-0.200	-0.167				*IL22RA2*	TSS200
cg14199090	1	65,990,564	0.001862	-0.200	-0.228				*LEPR*	5'UTR
cg20153240	1	2,016,789	2.71E-07	-0.200	-0.189				*PRKCZ*	Body

cg21760363	3	167,835,467	0.002459	-0.200	-0.143					
cg09418852	15	46,636,037	0.002418	-0.200	-0.228					
cg05611565	10	130,829,629	0.000299	-0.200	-0.167					
cg15777553	12	34,493,996	0.002457	-0.200	-0.223					
cg16292181	6	72,254,462	0.003076	-0.200	-0.116					
cg20156165	1	118,322,537	0.027641	-0.200	-0.195					
cg24156149	2	197,033,988	0.003658	-0.200	-0.195				*STK17B*	5'UTR
cg00816406	1	240,286,626	1.13E-05	-0.200	-0.199				*FMN2*	Body
cg03994184	7	1,714,969	0.006378	-0.200	-0.147					
cg12505085	10	75,913,585	7.46E-06	-0.200	-0.187				*ADK*	Body
cg22942908	1	110,578,259	1.05E-05	-0.200	-0.153				*FAM40A*	Body
cg23943272	17	45,851,891	0.004817	-0.200	-0.139					
cg04518304	14	76,600,760	0.004312	-0.200	-0.158					
cg25741597	3	147,722,622	3.95E-08	-0.200	-0.205					
cg02958014	12	127,206,602	0.019358	-0.200	-0.216					
cg03033543	20	31,870,884	0.000669	-0.200	-0.188				*C20orf114*	TSS200
cg08973950	7	1,083,309	2.8E-06	-0.200	-0.199				*C7orf50*	Body
cg18092977	14	101,190,167	0.023146	-0.200	-0.147					
cg18280251	7	48,237,515	0.029214	-0.200	-0.141				*ABCA13*	Body
cg05068636	15	44,887,664	4.27E-06	-0.200	-0.175				*SPG11*	Body
cg13921444	4	186,733,024	5.02E-09	-0.200	-0.199				*SORBS2*	5'UTR
cg16010911	17	72,581,014	0.000715	-0.200	-0.249				*C17orf77*	TSS200
cg16309727	8	117,963,055	2.74E-06	-0.200	-0.209					
cg23561148	18	33,688,575	1.28E-06	-0.200	-0.180				*SLC39A6*	3'UTR
cg13635710	13	56,216,872	0.003766	-0.200	-0.133					
cg27377632	19	677,895	8.61E-08	-0.200	-0.171				*FSTL3*	Body
cg00615010	X	103,809,062	0.019388	-0.200	-0.183					
cg02148324	7	150,925,493	3.39E-12	-0.200	-0.175				*ABCF2*	TSS1500
cg03540912	16	71,792,711	0.001669	-0.200	-0.175				*SNORD71*	TSS1500
cg19787650	8	130,998,171	4.04E-06	-0.200	-0.220					
cg10236631	14	36,899,948	0.032257	-0.200	-0.059					
cg20206277	21	43,098,901	6.05E-06	-0.200	-0.196				*NCRNA00111*	TSS1500
cg03878567	6	70,952,001	5.64E-06	-0.200	-0.197				*COL9A1*	Body
cg10948284	8	143,882,645	0.027279	-0.200	-0.080					

cg27075917	17	38,596,324	6.64E-06	-0.200	-0.190					
cg21474062	3	141,087,363	9.7E-07	-0.200	-0.180				*ZBTB38*	5'UTR
cg00092443	20	46,578,370	0.011825	-0.200	-0.168					
cg04231636	6	18,186,411	9.36E-08	-0.200	-0.184				*KDM1B*	Body
cg10887810	1	119,442,124	1.97E-07	-0.200	-0.188				*TBX15*	Body
cg20287514	1	25,869,837	0.000303	-0.200	-0.167				*LDLRAP1*	TSS1500
cg11830876	17	78,006,238	0.000483	-0.200	-0.168				*TBC1D16*	5'UTR
cg15646123	6	20,954,499	0.00051	-0.200	-0.152				*CDKAL1*	Body
cg04225760	14	96,152,464	0.028178	-0.200	-0.048				*TCL1B*	TSS1500
cg23091067	X	118,104,340	0.003457	-0.200	-0.158					
cg17738861	5	32,233,621	4.76E-06	-0.200	-0.174				*MTMR12*	Body
cg18789624	7	30,516,742	3.47E-05	-0.200	-0.210				*NOD1*	5'UTR
cg09857577	7	50,136,102	7.74E-05	-0.200	-0.176				*C7orf72*	1stExon
cg05733285	20	44,168,184	0.000734	-0.200	-0.229				*WFDC6*	TSS200
cg06970472	4	40,910,981	3.29E-07	-0.200	-0.139				*APBB2*	Body
cg08217234	19	4,042,688	3.22E-08	-0.200	-0.193					
cg16016034	1	205,579,533	3.73E-08	-0.200	-0.203					
cg15959080	3	13,939,366	0.029894	-0.200	-0.097					
cg03503295	5	13,944,491	0.000526	-0.200	-0.207	0.0105252	-0.059		*DNAH5*	1stExon
cg03741458	2	200,468,445	0.020822	-0.200	-0.189					
cg04431935	16	1,413,516	1.44E-06	-0.200	-0.202				*UNKL*	3'UTR
cg07652628	12	53,343,514	1.75E-07	-0.200	-0.198				*KRT18*	Body
cg02468966	20	56,287,198	0.005115	-0.201	-0.218				*PMEPA1*	TSS1500
cg03840511	15	64,649,139	0.001242	-0.201	-0.203				*CSNK1G1*	TSS1500
cg12113251	12	58,176,230	0.003784	-0.201	-0.130				*FAM119B*	3'UTR
cg24720672	15	57,599,126	8.26E-10	-0.201	-0.215				*LOC283663*	Body
cg08719289	1	41,328,378	3.83E-13	-0.201	-0.198				*CITED4*	TSS1500
cg11042505	2	15,950,244	0.00045	-0.201	-0.175					
cg15013761	17	25,982,249	1.61E-05	-0.201	-0.128					
cg21691717	1	153,321,907	0.009216	-0.201	-0.193				*PGLYRP4*	TSS1500
cg13578134	15	75,081,572	9.84E-05	-0.201	-0.227				*CSK*	5'UTR
cg01635303	12	34,511,504	0.007773	-0.201	-0.203					
cg22603037	1	152,087,267	0.000576	-0.201	-0.167				*TCHH*	TSS1500
cg03109316	3	113,957,903	0.038008	-0.201	-0.179	1.7E-08	-0.146		*ZNF80*	TSS1500

cg11936341	6	1,604,140	2.74E-05	-0.201	-0.198					
cg02938964	17	17,120,279	0.011392	-0.201	-0.108				*FLCN*	Body
cg04522792	8	122,543,204	9.32E-06	-0.201	-0.207					
cg03726785	20	1,532,543	0.049867	-0.201	-0.120				*SIRPD*	Body
cg19741359	2	73,429,176	0.037805	-0.201	-0.168				*NOTO*	TSS1500
cg04019148	13	56,226,793	0.002035	-0.201	-0.154					
cg06777202	13	65,532,878	0.040705	-0.201	-0.154					
cg22738289	10	42,859,004	0.041339	-0.201	-0.167				*LOC441666*	Body
cg22405653	21	32,128,800	0.006858	-0.201	-0.168				*KRTAP21-1*	TSS1500
cg02657245	16	5,976,578	0.019504	-0.201	-0.128					
cg07478467	8	91,712,588	0.005339	-0.201	-0.219					
cg16899306	6	32,732,585	0.022882	-0.201	-0.113	0.01193822	-0.090	Yes	*HLA-DQB2*	TSS1500
cg05224524	4	176,924,985	0.000681	-0.201	-0.140				*GPM6A*	TSS1500
cg18146398	3	46,251,215	4.51E-07	-0.201	-0.182				*CCR1*	TSS1500
cg02120470	1	67,267,129	7.28E-05	-0.201	-0.187				*INSL5*	TSS200
cg06631531	4	5,382,335	0.03841	-0.201	-0.088				*STK32B*	Body
cg10262744	3	12,599,968	7.65E-06	-0.201	-0.212				*MKRN2*	Body
cg16148956	8	54,140,771	8.02E-06	-0.201	-0.223				*OPRK1*	3'UTR
cg24586528	13	58,299,073	0.000621	-0.201	-0.125				*PCDH17*	Body
cg04239798	1	159,236,068	0.016047	-0.201	-0.127					
cg06817539	4	166,131,638	0.000467	-0.201	-0.187				*KLHL2*	Body
cg17334970	6	33,218,094	1.2E-07	-0.201	-0.207				*VPS52*	3'UTR
cg04010817	16	62,273,362	0.032705	-0.201	-0.056					
cg07813628	2	88,587,857	0.000505	-0.201	-0.138					
cg20490031	18	61,143,926	0.003268	-0.201	-0.168				*SERPINB5*	TSS1500
cg00500626	1	5,544,470	0.035045	-0.201	-0.082					
cg04023526	11	76,371,932	5.56E-08	-0.201	-0.176				*LRRC32*	Body
cg08572278	4	62,467,955	0.040257	-0.201	-0.105				*LPHN3*	Body
cg21013206	11	113,347,208	0.004148	-0.201	-0.208				*DRD2*	TSS1500
cg05587926	11	122,387,899	0.012507	-0.201	-0.200					
cg20479063	4	10,016,908	1.42E-07	-0.201	-0.194				*SLC2A9*	Body
cg01477861	4	77,609,841	0.000995	-0.201	-0.229				*SHROOM3*	Body
cg23822644	7	27,777,487	0.002944	-0.201	-0.219					
cg25191332	7	112,062,419	0.000185	-0.201	-0.173				*IFRD1*	TSS1500

cg10115203	1	7,121,245	0.029512	-0.201	-0.332				*CAMTA1*	Body
cg24532610	12	48,961,603	0.043969	-0.201	-0.119				*LALBA*	3'UTR
cg00331032	22	38,339,972	0.001813	-0.201	-0.279				*C22orf23*	3'UTR
cg02981092	3	57,543,499	1.57E-09	-0.201	-0.201				*PDE12*	Body
cg17658568	11	122,033,987	0.018087	-0.201	-0.168				*LOC399959*	Body
cg18405691	11	55,322,753	0.018364	-0.201	-0.206				*OR4C15*	1stExon
cg25143359	1	245,533,949	0.03397	-0.201	-0.107				*KIF26B*	Body
cg21770322	7	97,807,741	9.89E-05	-0.201	-0.190				*LMTK2*	Body
cg04572540	6	30,908,739	0.008472	-0.201	-0.059				*DPCR1*	TSS200
cg11201447	8	128,808,063	1.62E-05	-0.201	-0.185				*MIR1204*	TSS200
cg16204420	5	44,332,757	1.01E-09	-0.201	-0.194				*FGF10*	Body
cg09241295	3	192,560,263	0.015668	-0.201	-0.153				*C3orf59*	Body
cg14978994	1	1,978,115	1.14E-11	-0.201	-0.166					
cg15997518	1	81,814,874	1.07E-08	-0.201	-0.195					
cg19403586	8	80,938,785	5.1E-06	-0.201	-0.161				*MRPS28*	Body
cg27365103	20	1,578,550	0.01027	-0.201	-0.169				*SIRPB1*	Body
cg04511463	11	43,589,582	0.037474	-0.201	-0.125					
cg04577162	7	73,667,397	0.008933	-0.201	-0.089				*RFC2*	Body
cg03609034	7	101,680,927	0.002077	-0.201	-0.164				*CUX1*	Body
cg04267605	4	52,859,928	0.003029	-0.201	-0.233				*LRRC66*	3'UTR
cg11580026	3	44,730,244	1.26E-08	-0.201	-0.193					
cg11441533	12	64,295,363	0.00358	-0.201	-0.119				*SRGAP1*	Body
cg25814149	6	169,420,648	7.9E-05	-0.201	-0.126					
cg19928703	13	30,143,971	7.23E-06	-0.201	-0.194				*SLC7A1*	5'UTR
cg20079335	X	151,883,293	0.007296	-0.201	-0.148				*MAGEA2*	5'UTR
cg05088605	17	79,987,813	0.006409	-0.201	-0.131				*LRRC45*	Body
cg14643415	3	167,293,056	0.048801	-0.201	-0.130				*WDR49*	Body
cg23764008	11	61,344,823	0.00208	-0.201	-0.164				*SYT7*	Body
cg14327359	2	47,419,263	5.21E-11	-0.201	-0.196					
cg02082650	X	25,022,003	4.23E-08	-0.201	-0.192				*ARX*	3'UTR
cg02490626	2	234,653,891	0.023613	-0.201	-0.201				*UGT1A10*	Body
cg03531326	19	39,443,344	2.56E-09	-0.201	-0.202				*FBXO17*	5'UTR
cg05683808	7	157,133,186	2.74E-07	-0.201	-0.190				*DNAJB6*	5'UTR
cg08250055	6	32,052,736	0.033472	-0.201	-0.043				*TNXB*	Body

cg14255981	12	105,414,154	4.95E-05	-0.201	-0.211				*ALDH1L2*	Body
cg18566313	4	186,733,428	9.18E-10	-0.201	-0.197				*SORBS2*	TSS200
cg08844169	3	119,180,915	0.001125	-0.201	-0.168				*TMEM39A*	Body
cg18552413	1	159,174,610	0.001926	-0.201	-0.215	7.78E-23	-0.161		*DARC*	TSS1500
cg20667778	15	53,943,590	0.033348	-0.201	-0.146				*WDR72*	Body
cg20668718	5	146,991,927	0.000145	-0.201	-0.239				*JAKMIP2*	Body
cg01919999	5	180,668,568	2.37E-08	-0.201	-0.171				*GNB2L1*	Body
cg06884352	8	122,808,923	1.13E-05	-0.201	-0.228					
cg19313311	7	74,058,031	7.1E-12	-0.201	-0.223					
cg24711070	11	118,842,126	0.022482	-0.201	-0.064				*FOXR1*	TSS1500
cg11506843	8	7,153,933	0.005946	-0.201	-0.222				*FAM90A20*	Body
cg13353152	1	158,465,963	7.01E-07	-0.201	-0.208					
cg10071848	14	74,770,110	0.000002	-0.201	-0.172				*ABCD4*	TSS1500
cg16471362	20	44,170,643	0.043162	-0.201	-0.081				*SPINLW1*	3'UTR
cg14972228	19	38,570,495	0.003283	-0.201	-0.217				*SIPA1L3*	5'UTR
cg00033703	15	63,566,255	3.53E-05	-0.201	-0.207					
cg18115481	14	106,173,722	0.013894	-0.201	-0.108					
cg11358713	7	41,188,092	7.86E-05	-0.201	-0.171					
cg21211039	22	45,555,611	7.14E-09	-0.201	-0.184					
cg22636631	5	79,334,767	5.1E-07	-0.201	-0.173				*THBS4*	Body
cg13425422	2	167,168,604	0.035504	-0.201	-0.103				*SCN9A*	5'UTR
cg21977507	17	12,145,856	3.77E-08	-0.201	-0.194					
cg14619949	5	76,116,169	0.016368	-0.201	-0.199				*F2RL1*	Body
cg21188409	3	180,466,040	2.12E-08	-0.201	-0.189					
cg18195238	8	64,681,840	0.035999	-0.201	-0.141					
cg22008551	19	46,452,989	5.17E-07	-0.201	-0.180				*NOVA2*	Body
cg00230837	5	9,714,909	0.032557	-0.201	-0.105				*LOC285692*	Body
cg02297967	8	57,473,034	0.00093	-0.201	-0.228					
cg09514545	19	54,200,652	3.08E-08	-0.201	-0.185				*MIR525*	TSS200
cg03964208	10	132,461,229	0.015167	-0.201	-0.093					
cg08791424	2	208,795,653	6.95E-06	-0.201	-0.199				*PLEKHM3*	Body
cg12132396	14	26,676,915	0.026779	-0.201	-0.095					
cg20131968	17	61,850,012	7.74E-11	-0.202	-0.194	0.0000896	-0.061		*CCDC47*	5'UTR
cg24032710	14	104,770,459	0.01468	-0.202	-0.147					

cg27605977	5	152,309,662	0.027726	-0.202	-0.190					
cg14640670	5	139,494,937	6.05E-17	-0.202	-0.192				*PURA*	3'UTR
cg13470969	1	152,490,870	0.038581	-0.202	-0.216					
cg19373760	1	247,679,988	1.7E-07	-0.202	-0.183					
cg24464789	1	43,751,497	2.91E-05	-0.202	-0.209				*C1orf210*	TSS1500
cg17346176	6	119,239,700	2.02E-05	-0.202	-0.182				*MCM9*	Body
cg23873288	6	35,705,851	2.84E-08	-0.202	-0.194				*C6orf81*	Body
cg27468419	8	57,309,109	0.041773	-0.202	-0.305				*SDR16C6*	TSS1500
cg00554948	12	51,631,858	5.85E-05	-0.202	-0.183				*DAZAP2*	TSS1500
cg07347148	1	167,786,802	0.001432	-0.202	-0.254				*ADCY10*	Body
cg14620221	11	124,310,939	0.022407	-0.202	-0.164	1.61E-07	-0.109		*OR8B8*	1stExon
cg16509592	2	106,120,415	0.001796	-0.202	-0.135					
cg17095929	11	64,883,911	7.28E-09	-0.202	-0.192				*ZNHIT2*	1stExon
cg17806482	7	107,608,007	2.7E-05	-0.202	-0.192				*LAMB1*	Body
cg13110574	21	46,493,169	0.00226	-0.202	-0.166				*ADARB1*	TSS1500
cg18532070	7	41,940,672	0.021164	-0.202	-0.153					
cg25987408	5	154,318,104	4.7E-06	-0.202	-0.191				*GEMIN5*	TSS1500
cg14059959	13	111,144,447	0.002265	-0.202	-0.141				*COL4A2*	Body
cg11179610	15	95,588,018	0.001963	-0.202	-0.139					
cg07648740	7	154,864,630	0.00141	-0.202	-0.206				*HTR5A*	Body
cg05415308	15	71,406,783	9.45E-08	-0.202	-0.203				*CT62*	5'UTR
cg05941106	11	123,248,842	0.022931	-0.202	-0.153					
cg08422172	16	11,886,668	1.86E-07	-0.202	-0.196					
cg10610428	12	5,884,295	0.031153	-0.202	-0.198				*ANO2*	Body
cg16900910	15	72,789,182	3.49E-05	-0.202	-0.187				*ARIH1*	Body
cg13049624	3	1,050,278	1.2E-06	-0.202	-0.182					
cg15472885	3	71,056,608	2.09E-06	-0.202	-0.227				*FOXP1*	Body
cg15167841	11	71,787,492	6.78E-05	-0.202	-0.193				*NUMA1*	5'UTR
cg22203607	5	180,536,151	0.028897	-0.202	-0.071					
cg12751948	10	133,794,454	4.53E-09	-0.202	-0.181				*BNIP3*	Body
cg20621674	13	56,188,634	2.2E-05	-0.202	-0.165					
cg26339974	1	153,230,907	0.019507	-0.202	-0.168				*LOR*	TSS1500
cg18397975	5	33,997,484	0.019733	-0.202	-0.170				*AMACR*	Body
cg10517650	3	113,235,015	4.93E-06	-0.202	-0.173				*CCDC52*	TSS1500

cg23067085	3	71,180,032	8.13E-45	-0.202	-0.194				*FOXP1*	Body
cg21070527	7	63,558,196	0.000352	-0.202	-0.139					
cg23024102	13	45,149,706	9.05E-07	-0.202	-0.211				*TSC22D1*	1stExon
cg06275134	X	140,335,783	0.012669	-0.202	-0.181				*SPANXC*	Body
cg26773183	X	108,616,697	0.022068	-0.202	-0.279				*GUCY2F*	3'UTR
cg02265105	5	83,268,490	1.27E-06	-0.202	-0.196				*EDIL3*	Body
cg07029777	2	1,658,233	2.91E-05	-0.202	-0.169				*PXDN*	Body
cg09044174	20	60,308,212	0.002231	-0.202	-0.124				*CDH4*	Body
cg20570016	1	156,261,298	4.77E-08	-0.202	-0.192				*TMEM79*	Body
cg05729745	22	49,937,644	0.000182	-0.202	-0.155					
cg05854698	10	134,910,850	0.037441	-0.202	-0.155				*GPR123*	Body
cg14565752	19	47,955,003	0.031318	-0.202	-0.185				*SLC8A2*	Body
cg15625631	11	123,814,972	0.000303	-0.202	-0.153				*OR6T1*	TSS1500
cg22838882	3	138,952,118	1.52E-05	-0.202	-0.247				*PISRT1*	Body
cg05343159	10	129,347,529	0.009123	-0.202	-0.165				*NPS*	TSS200
cg11239749	6	32,781,307	0.03113	-0.202	-0.128				*HLA-DOB*	Body
cg02552189	2	223,891,284	0.000127	-0.202	-0.201					
cg11988476	21	46,368,090	0.043965	-0.202	-0.124				*C21orf70*	Body
cg19959621	22	37,179,912	0.010824	-0.202	-0.149					
cg11770920	2	218,465,695	0.037225	-0.202	-0.144				*DIRC3*	Body
cg24443446	10	65,647,572	4.33E-05	-0.202	-0.188					
cg03065888	8	145,148,823	1.33E-06	-0.202	-0.148				*CYC1*	TSS1500
cg02822963	3	149,470,953	1.69E-09	-0.202	-0.224				*COMMD2*	TSS1500
cg14074970	18	19,932,132	0.04098	-0.202	-0.181					
cg18719016	10	125,558,960	0.004293	-0.202	-0.128				*CPXM2*	Body
cg12705541	X	75,245,844	0.04504	-0.202	-0.186					
cg25214823	13	41,626,718	0.000542	-0.202	-0.210					
cg00217306	3	141,681,567	2.18E-09	-0.202	-0.172				*TFDP2*	Body
cg03262887	20	29,978,114	3.49E-16	-0.202	-0.197				*DEFB119*	Body
cg14286140	9	93,279,614	5.16E-20	-0.202	-0.188					
cg07440648	4	23,236,618	0.008247	-0.202	-0.115					
cg09206878	15	25,165,585	0.001689	-0.202	-0.174				*SNRPN*	5'UTR
cg07431888	16	8,813,274	1.97E-06	-0.202	-0.196				*ABAT*	5'UTR
cg25850629	1	33,210,830	4.04E-05	-0.202	-0.185				*KIAA1522*	Body

cg04813697	10	22,920,025	2.03E-05	-0.202	-0.182				*PIP4K2A*	Body
cg01325188	16	88,050,684	0.000331	-0.202	-0.206				*BANP*	Body
cg13984173	15	40,674,438	1.73E-07	-0.202	-0.191				*C15orf23*	TSS1500
cg17839216	5	18,504,434	0.005897	-0.202	-0.149					
cg00356981	13	112,804,728	0.008105	-0.202	-0.229					
cg02186141	17	80,864,562	0.012765	-0.202	-0.171				*TBCD*	Body
cg04441453	5	41,902,083	0.001088	-0.202	-0.178					
cg20975733	15	87,155,056	4.94E-06	-0.202	-0.198				*AGBL1*	Body
cg03228407	17	5,392,363	0.000963	-0.202	-0.163				*MIS12*	Body
cg03812562	4	190,597,513	0.004022	-0.202	-0.161					
cg24411946	10	52,645,408	0.000565	-0.202	-0.196				*A1CF*	5'UTR
cg15507486	19	46,627,896	0.039569	-0.202	-0.160				*IGFL3*	1stExon
cg23931819	1	1,245,076	5.54E-08	-0.202	-0.148				*PUSL1*	Body
cg06696292	8	23,536,779	0.003755	-0.202	-0.171				*NKX3-1*	3'UTR
cg22568403	5	169,758,055	2.83E-06	-0.202	-0.180				*LOC257358*	TSS1500
cg19478478	6	123,957,927	0.003362	-0.202	-0.137	0.00000109	-0.137		*TRDN*	1stExon
cg22104371	7	1,787,371	0.001435	-0.202	-0.163				*ELFN1*	3'UTR
cg25009896	17	21,904,695	0.003968	-0.202	-0.154				*FLJ36000*	Body
cg20249071	11	122,684,258	0.026414	-0.202	-0.094				*UBASH3B*	3'UTR
cg21765032	19	3,293,547	0.00354	-0.202	-0.185				*BRUNOL5*	3'UTR
cg04900872	2	31,803,536	5.69E-10	-0.202	-0.212				*SRD5A2*	Body
cg12798194	17	43,462,791	0.006633	-0.202	-0.109					
cg23844115	6	10,434,871	6.07E-05	-0.202	-0.153				*C6orf218*	Body
cg16463044	17	3,956,989	0.004313	-0.202	-0.185				*ZZEF1*	Body
cg11881735	12	30,945,390	0.001399	-0.202	-0.152					
cg16627193	7	157,801,404	0.002579	-0.202	-0.239				*PTPRN2*	Body
cg23565607	17	80,395,310	0.000157	-0.202	-0.147				*HEXDC*	Body
cg26076724	6	15,090,163	3.09E-06	-0.202	-0.183					
cg05512061	6	100,042,195	0.001855	-0.202	-0.120					
cg08096854	17	48,617,637	0.002503	-0.202	-0.178				*EPN3*	Body
cg21613620	13	97,927,242	1.19E-05	-0.202	-0.154				*MBNL2*	5'UTR
cg16387254	1	231,472,287	0.000795	-0.203	-0.183				*C1orf124*	TSS1500
cg01736133	4	120,605,519	4.14E-05	-0.203	-0.196					
cg02909176	13	113,426,343	0.000212	-0.203	-0.187				*ATP11A*	Body

cg16180848	11	3,612,651	0.000333	-0.203	-0.147					
cg05073620	3	57,530,799	9.9E-06	-0.203	-0.145				*DNAH12*	TSS1500
cg12491169	11	40,137,955	0.045096	-0.203	-0.149				*LRRC4C*	5'UTR
cg24141885	5	12,883,715	0.017334	-0.203	-0.152					
cg05307141	1	170,132,227	0.008258	-0.203	-0.135				*METTL11B*	Body
cg19524824	12	50,023,313	0.000238	-0.203	-0.167				*PRPF40B*	5'UTR
cg26390526	1	152,299,078	0.044117	-0.203	-0.243	0.00025886	-0.117		*FLG*	TSS1500
cg26442740	12	55,613,318	0.04717	-0.203	-0.147				*OR10A7*	TSS1500
cg27019059	3	133,852,523	2.83E-05	-0.203	-0.202					
cg03848127	5	100,126,093	0.003494	-0.203	-0.189					
cg10968815	20	31,595,346	0.001438	-0.203	-0.229	4.95E-13	-0.111		*BPIL1*	TSS200
cg15075709	11	1,217,335	0.024126	-0.203	-0.045					
cg04611649	2	152,681,240	1.05E-07	-0.203	-0.197				*ARL5A*	Body
cg18574911	17	80,355,371	0.005813	-0.203	-0.270				*C17orf101*	Body
cg15627145	10	61,802,440	1.29E-06	-0.203	-0.213				*ANK3*	3'UTR
cg21052687	11	55,561,545	0.019835	-0.203	-0.197				*OR5D14*	TSS1500
cg01938476	12	7,076,853	6.72E-06	-0.203	-0.215				*SCARNA12*	TSS200
cg07385257	8	35,803,167	0.003785	-0.203	-0.200					
cg05119467	1	1,765,440	3.84E-08	-0.203	-0.185				*GNB1*	5'UTR
cg13054975	1	205,603,464	0.024536	-0.203	-0.079				*ELK4*	TSS1500
cg18289710	11	103,811,641	0.001142	-0.203	-0.213				*PDGFD*	Body
cg24640735	12	10,065,986	0.011247	-0.203	-0.110				*CLEC2A*	3'UTR
cg02638909	8	30,888,801	0.00086	-0.203	-0.201				*PURG*	Body
cg13784443	2	80,807,289	0.001771	-0.203	-0.232			Yes	*CTNNA2*	Body
cg17603763	11	94,799,719	3.01E-14	-0.203	-0.202				*SFRS2B*	TSS1500
cg18480432	13	63,637,003	0.002231	-0.203	-0.194					
cg27509347	1	28,385,657	1.01E-07	-0.203	-0.209				*EYA3*	5'UTR
cg15803671	18	3,457,981	9.94E-05	-0.203	-0.232				*TGIF1*	3'UTR
cg22956205	13	43,321,660	4.89E-06	-0.203	-0.203					
cg24299913	19	42,408,316	0.000534	-0.203	-0.180				*ARHGEF1*	Body
cg09075787	2	69,965,513	0.001136	-0.203	-0.196					
cg27504460	2	188,169,789	0.004585	-0.203	-0.136					
cg16462006	14	95,155,784	0.016277	-0.203	-0.266					
cg00054741	19	54,201,247	0.026545	-0.203	-0.171				*MIR523*	TSS1500

cg23384017	4	52,808,725	0.023392	-0.203	-0.153					
cg15646919	18	77,608,799	0.000563	-0.203	-0.239					
cg13846509	8	64,376,550	0.004726	-0.203	-0.177					
cg25122699	5	173,314,531	1.92E-06	-0.203	-0.181				*CPEB4*	TSS1500
cg10705800	1	41,328,420	9.68E-16	-0.203	-0.195	0.00000508	-0.046		*CITED4*	TSS1500
cg27180868	1	161,008,750	3.54E-11	-0.203	-0.195				*TSTD1*	5'UTR
cg07465609	6	66,205,227	0.016418	-0.203	-0.171	0.00000222	-0.140		*EYS*	Body
cg11395306	11	98,939,366	0.009599	-0.203	-0.134				*CNTN5*	5'UTR
cg00045910	10	23,466,070	0.003442	-0.203	-0.187					
cg01229658	15	70,924,395	2.2E-05	-0.203	-0.188					
cg08273233	6	87,724,979	0.000324	-0.203	-0.149				*HTR1E*	5'UTR
cg24328944	13	96,256,596	3.93E-08	-0.203	-0.200				*DZIP1*	Body
cg12032655	19	43,856,746	1.31E-06	-0.203	-0.183				*CD177*	TSS1500
cg23210521	4	95,680,414	3.14E-06	-0.203	-0.197				*BMPR1B*	5'UTR
cg03698145	15	98,064,729	0.024341	-0.203	-0.163					
cg09159821	5	146,937,183	0.000961	-0.203	-0.145					
cg20897616	15	26,874,515	1.77E-06	-0.203	-0.163				*GABRB3*	Body
cg24540281	2	105,912,735	6.8E-05	-0.203	-0.198				*TGFBRAP1*	Body
cg14703102	17	2,975,585	0.006617	-0.203	-0.121					
cg25184787	20	3,898,644	0.013334	-0.203	-0.162				*PANK2*	Body
cg16890121	13	28,198,579	0.000342	-0.203	-0.143				*POLR1D*	Body
cg03769908	7	79,631,950	0.012845	-0.203	-0.218					
cg07516307	6	33,165,658	0.000158	-0.203	-0.181				*RXRB*	Body
cg20901464	13	112,763,220	0.015486	-0.203	-0.076					
cg13797779	14	99,180,409	0.03029	-0.203	-0.136				*C14orf177*	5'UTR
cg20316758	13	106,797,152	6.94E-06	-0.203	-0.202					
cg14412833	9	128,021,077	5.87E-05	-0.203	-0.194					
cg10449561	5	25,909,633	0.013761	-0.203	-0.120					
cg13480465	4	16,795,757	0.000303	-0.203	-0.214				*LDB2*	Body
cg07999668	12	8,765,336	0.04336	-0.203	-0.125				*AICDA*	Body
cg26218823	X	146,318,566	0.038419	-0.203	-0.158				*MIR508*	TSS200
cg05170856	16	32,255,825	0.000257	-0.203	-0.190					
cg01212562	21	46,868,092	0.003234	-0.203	-0.211				*COL18A1*	Body
cg10506618	3	42,246,902	0.00526	-0.203	-0.199				*TRAK1*	Body

cg15570432	20	30,460,881	0.037034	-0.203	-0.105				*TTLL9*	Body
cg18827756	15	42,130,735	0.000202	-0.203	-0.195				*JMJD7-PLA2G4B*	Body
cg00107916	5	3,188,066	0.01659	-0.203	-0.248					
cg19894085	12	66,633,274	6.44E-05	-0.203	-0.154			Yes	*IRAK3*	Body
cg04012844	22	49,020,400	0.002832	-0.203	-0.214				*FAM19A5*	Body
cg04830757	5	87,880,065	7.91E-05	-0.203	-0.203				*LOC645323*	Body
cg10946681	12	127,633,722	0.005981	-0.203	-0.149					
cg17259265	11	125,753,373	0.00531	-0.203	-0.133	0.00000355	-0.083		*HYLS1*	TSS200
cg19205850	3	128,446,370	1.55E-11	-0.203	-0.216				*RAB7A*	5'UTR
cg22109208	1	172,414,443	0.001495	-0.203	-0.150				*C1orf105*	Body
cg23154497	10	134,919,573	0.01076	-0.203	-0.132				*GPR123*	Body
cg07033253	7	75,908,694	8.7E-06	-0.203	-0.209				*SRRM3*	Body
cg08626382	11	62,108,591	1.11E-06	-0.203	-0.191				*ASRGL1*	Body
cg01889083	3	36,803,565	0.045742	-0.203	-0.128					
cg08426130	15	95,872,552	0.000765	-0.203	-0.144					
cg15382258	7	48,495,072	0.03838	-0.203	-0.131				*ABCA13*	Body
cg21304163	19	35,607,221	3.25E-05	-0.203	-0.193				*FXYD3*	5'UTR
cg21723559	20	44,048,174	5.7E-08	-0.203	-0.215				*PIGT*	Body
cg15446073	11	62,339,136	2.12E-05	-0.203	-0.134				*EEF1G*	Body
cg10543582	2	199,202,432	0.002694	-0.203	-0.155					
cg21029155	13	20,069,043	0.001897	-0.203	-0.156				*TPTE2*	Body
cg07338464	5	148,244,123	1.28E-08	-0.203	-0.179					
cg07585334	16	8,617,571	4.75E-05	-0.203	-0.193					
cg20992439	15	91,429,916	0.001513	-0.203	-0.154				*FES*	Body
cg22812604	8	140,417,841	3.46E-05	-0.203	-0.176					
cg21643429	15	28,216,043	0.008991	-0.203	-0.212				*OCA2*	Body
cg03592833	3	194,129,373	0.002773	-0.203	-0.193				*ATP13A3*	Body
cg07805642	6	132,890,602	0.035968	-0.203	-0.144				*TAAR6*	TSS1500
cg24158157	10	124,581,726	0.000456	-0.203	-0.147					
cg08204867	16	10,208,426	0.000116	-0.204	-0.223				*GRIN2A*	Body
cg19870706	1	75,469,109	3.73E-05	-0.204	-0.187					
cg26492744	X	68,046,595	4.58E-10	-0.204	-0.190					
cg11185753	8	105,988,283	0.006952	-0.204	-0.235					
cg16450097	8	101,348,467	0.008978	-0.204	-0.198					

cg14005657	Y	9,365,108	0.024625	-0.204	-0.127				*TSPY3*	TSS1500
cg25904183	4	165,877,875	2.25E-05	-0.204	-0.220				*C4orf39*	TSS1500
cg00920269	7	45,431,741	0.007949	-0.204	-0.110					
cg08312198	2	235,961,352	0.022945	-0.204	-0.106				*SH3BP4*	Body
cg14104148	11	126,203,133	0.000274	-0.204	-0.098				*DCPS*	Body
cg26353859	12	60,081,776	0.028264	-0.204	-0.096				*SLC16A7*	TSS1500
cg17453252	13	110,964,116	0.017717	-0.204	-0.096				*COL4A2*	Body
cg03240663	19	52,150,547	0.033388	-0.204	-0.183				*SIGLEC14*	TSS1500
cg09874415	1	35,989,973	0.001245	-0.204	-0.176				*KIAA0319L*	Body
cg06868132	5	178,987,429	0.013095	-0.204	-0.267				*RUFY1*	Body
cg18456942	20	29,956,330	0.001862	-0.204	-0.214				*DEFB118*	TSS200
cg23198334	10	76,179,907	5.45E-05	-0.204	-0.199				*ADK*	Body
cg21409829	1	2,850,859	0.020642	-0.204	-0.084					
cg25153233	20	37,854,269	0.014223	-0.204	-0.134				*LOC339568*	TSS1500
cg26806260	15	62,356,512	1.23E-06	-0.204	-0.214					
cg01171429	16	69,939,015	0.003016	-0.204	-0.156				*WWP2*	Body
cg00532449	1	2,375,420	0.009285	-0.204	-0.249					
cg17536071	4	71,383,999	1.6E-06	-0.204	-0.213				*AMTN*	TSS1500
cg20792256	19	14,992,047	3.8E-05	-0.204	-0.142				*OR7A17*	1stExon
cg26872482	6	32,051,108	4.24E-09	-0.204	-0.211				*TNXB*	Body
cg07744216	19	7,682,838	0.004932	-0.204	-0.146				*KIAA1543*	Body
cg25077371	10	10,346,139	0.01929	-0.204	-0.184					
cg04735660	1	176,999,916	0.000363	-0.204	-0.182				*MIR488*	TSS1500
cg18086520	4	186,741,362	0.019784	-0.204	-0.161				*SORBS2*	5'UTR
cg19580003	13	19,236,676	0.033346	-0.204	-0.115					
cg14020914	19	53,798,641	0.000621	-0.204	-0.224					
cg16298202	4	122,680,946	0.007599	-0.204	-0.126				*TMEM155*	3'UTR
cg24369466	6	31,843,146	0.000275	-0.204	-0.221				*SLC44A4*	Body
cg27500983	20	34,700,201	9.87E-06	-0.204	-0.226				*EPB41L1*	TSS200
cg05604487	17	77,386,277	0.002661	-0.204	-0.168				*HRNBP3*	5'UTR
cg00865390	8	91,713,322	0.010995	-0.204	-0.242					
cg27666123	5	126,988,029	0.002641	-0.204	-0.133				*CTXN3*	5'UTR
cg25463409	4	46,848,264	0.000311	-0.204	-0.122				*COX7B2*	5'UTR
cg25458175	19	59,025,570	3.87E-06	-0.204	-0.172				*ZBTB45*	Body

cg12943259	8	89,603,364	0.043268	-0.204	-0.115					
cg08543028	12	31,902,503	2.6E-07	-0.204	-0.198			Yes		
cg23180171	12	113,055,704	2.87E-05	-0.204	-0.189					
cg01963464	2	228,339,337	2.45E-06	-0.204	-0.178			Yes	*AGFG1*	Body
cg14430151	4	8,440,600	0.000721	-0.204	-0.184				*ACOX3*	5'UTR
cg08510178	6	165,062,070	0.003936	-0.204	-0.149					
cg14680360	17	39,598,430	0.000115	-0.204	-0.119				*KRT38*	TSS1500
cg07529428	12	15,717,516	6.75E-08	-0.204	-0.210				*PTPRO*	Body
cg11780053	19	7,268,027	0.020203	-0.204	-0.208				*INSR*	Body
cg26621014	X	88,001,695	0.039735	-0.204	-0.100				*CPXCR1*	TSS1500
cg05569767	18	76,638,494	0.027054	-0.204	-0.089					
cg13705774	1	152,670,764	0.019297	-0.204	-0.195				*LCE2A*	TSS200
cg18448898	2	128,788,611	0.000212	-0.204	-0.245					
cg21038123	16	733,280	4.31E-11	-0.204	-0.202				*JMJD8*	Body
cg16773563	7	44,614,475	0.025772	-0.204	-0.202				*DDX56*	TSS1500
cg23865597	18	46,987,786	0.038269	-0.204	-0.225				*DYM*	TSS1500
cg00180631	X	149,736,640	0.005701	-0.204	-0.216	0.00511158	-0.055		*MTM1*	TSS1500
cg22436195	4	154,951,775	0.04749	-0.204	-0.127			Yes		
cg02570643	5	77,531,473	0.027467	-0.204	-0.114				*AP3B1*	Body
cg02573176	8	82,607,505	0.000129	-0.204	-0.214				*SLC10A5*	TSS1500
cg04629433	11	124,191,389	0.021935	-0.204	-0.123				*OR8D2*	TSS1500
cg08510253	11	31,904,940	0.03331	-0.204	-0.178					
cg26130533	4	1,202,700	8E-08	-0.204	-0.176				*LOC100130872-SPON2*	TSS200
cg10835286	2	66,657,913	5.61E-07	-0.204	-0.222					
cg14613361	1	109,751,302	1.31E-05	-0.204	-0.169					
cg00461311	12	109,460,299	2.08E-06	-0.204	-0.199				*SVOP*	TSS1500
cg21340845	2	142,890,700	1.32E-05	-0.204	-0.218				*LRP1B*	TSS1500
cg19655510	20	62,374,329	0.001057	-0.204	-0.216				*SLC2A4RG*	Body
cg21765730	6	155,777,175	0.000695	-0.204	-0.163				*NOX3*	TSS200
cg25812683	10	6,691,515	0.002108	-0.204	-0.206					
cg02671458	5	178,801,812	0.020075	-0.204	-0.103					
cg25939242	4	9,371,392	0.01866	-0.204	-0.111					
cg20506745	2	2,581,285	1.08E-09	-0.204	-0.189					
cg14626907	6	33,873,691	0.001121	-0.204	-0.144					

cg20658635	15	26,877,820	1.26E-06	-0.204	-0.199				*GABRB3*	Body
cg08632095	5	172,639,423	0.001838	-0.204	-0.117					
cg13590475	6	133,137,827	6.37E-06	-0.204	-0.163				*RPS12*	Body
cg05932024	2	42,086,889	2.35E-06	-0.204	-0.186					
cg21848981	4	190,597,409	0.001849	-0.204	-0.179					
cg22106847	5	118,584,299	0.000208	-0.204	-0.213				*DMXL1*	3'UTR
cg23673761	1	196,865,686	0.01635	-0.204	-0.195				*CFHR4*	Body
cg08702011	16	86,934,677	0.0448	-0.204	-0.084			Yes		
cg23200873	3	167,191,837	0.017203	-0.204	-0.143				*SERPINI2*	TSS200
cg01661742	4	142,141,192	0.043567	-0.204	-0.191				*ZNF330*	TSS1500
cg00571870	6	109,349,597	5.84E-06	-0.204	-0.216				*SESN1*	Body
cg14300812	9	134,818,827	0.00771	-0.204	-0.226				*MED27*	Body
cg26560419	6	72,290,918	0.037952	-0.204	-0.237					
cg04672506	6	33,117,384	1.6E-05	-0.204	-0.184					
cg05361594	13	68,220,805	0.014891	-0.204	-0.226					
cg05991092	10	134,731,965	0.000224	-0.204	-0.206					
cg10735319	14	65,121,324	2.22E-11	-0.204	-0.205					
cg14591439	5	121,190,799	0.043238	-0.204	-0.092					
cg02335700	12	110,282,809	0.001054	-0.204	-0.141					
cg11356398	10	133,783,182	0.000389	-0.204	-0.196				*BNIP3*	Body
cg16993682	1	158,259,268	0.000572	-0.204	-0.218				*CD1C*	TSS1500
cg25614726	1	10,490,058	2.64E-17	-0.204	-0.198				*APITD1*	TSS200
cg00859147	19	8,841,460	0.026293	-0.204	-0.164				*OR2Z1*	1stExon
cg12275410	10	135,123,239	0.000148	-0.204	-0.197				*ZNF511*	Body
cg17920195	4	129,149,383	8.06E-10	-0.204	-0.189					
cg07412043	2	137,748,366	0.013384	-0.204	-0.163				*THSD7B*	TSS200
cg17339327	6	31,939,025	4.01E-06	-0.204	-0.214				*STK19*	TSS1500
cg15287443	3	147,121,229	2.7E-07	-0.204	-0.184				*ZIC4*	Body
cg19333980	20	5,094,433	0.000876	-0.204	-0.204				*C20orf30*	TSS1500
cg00976619	3	156,876,725	1.52E-08	-0.204	-0.221				*CCNL1*	Body
cg02309868	19	35,741,001	8.24E-08	-0.204	-0.173				*LSR*	Body
cg15602359	22	50,144,431	0.007972	-0.204	-0.117					
cg20743708	11	4,726,105	0.002807	-0.204	-0.171					
cg25882240	10	134,868,158	0.013143	-0.204	-0.101					

cg00817501	4	138,440,719	4.8E-09	-0.204	-0.186				*PCDH18*	3'UTR
cg27152299	11	71,238,225	5.7E-05	-0.204	-0.170				*KRTAP5-7*	TSS200
cg01752041	8	63,055,444	0.048327	-0.204	-0.193					
cg08881938	3	132,599,319	0.012854	-0.204	-0.240					
cg02470923	13	22,392,755	0.028183	-0.204	-0.137					
cg27455211	16	86,734,223	0.003135	-0.204	-0.134					
cg04362839	14	105,688,421	0.013268	-0.204	-0.164				*BRF1*	Body
cg12331160	2	135,290,945	0.001409	-0.204	-0.123				*TMEM163*	Body
cg18450130	14	105,031,193	0.011855	-0.204	-0.121					
cg01157146	21	42,877,638	1.44E-14	-0.204	-0.201				*TMPRSS2*	Body
cg02361306	19	35,940,461	4.9E-08	-0.204	-0.189				*FFAR2*	TSS200
cg25486039	6	41,715,166	9.41E-06	-0.204	-0.174				*PGC*	TSS200
cg26979537	19	48,016,860	0.00074	-0.204	-0.190				*NAPA*	Body
cg05334655	14	23,890,294	0.00038	-0.204	-0.184				*MYH7*	Body
cg09048404	2	91,937,754	0.012148	-0.204	-0.157					
cg19549898	14	68,000,922	3.35E-07	-0.204	-0.234				*PLEKHH1*	5'UTR
cg10167508	20	46,988,400	0.024685	-0.204	-0.159				*LOC284749*	TSS1500
cg07682515	10	134,838,014	3.25E-05	-0.205	-0.163					
cg18339075	18	76,555,521	0.029976	-0.205	-0.063					
cg18477674	10	133,793,527	0.010134	-0.205	-0.201				*BNIP3*	Body
cg21538450	1	205,313,833	1.01E-06	-0.205	-0.240				*KLHDC8A*	5'UTR
cg26476852	7	27,204,728	0.001621	-0.205	-0.129				*HOXA9*	1stExon
cg25249713	4	177,045,356	0.000351	-0.205	-0.195				*WDR17*	Body
cg11675165	8	117,853,304	4.78E-05	-0.205	-0.164					
cg02321821	17	79,804,422	0.029738	-0.205	-0.182				*P4HB*	Body
cg07778145	16	17,178,083	0.014956	-0.205	-0.089					
cg03519907	5	154,092,426	0.000121	-0.205	-0.192				*LARP1*	TSS200
cg06961205	6	6,623,143	1.16E-05	-0.205	-0.138				*LOC285780*	TSS200
cg15771735	6	57,614,321	0.007917	-0.205	-0.252					
cg04961042	14	30,218,600	0.01058	-0.205	-0.253				*PRKD1*	Body
cg23138161	8	27,337,259	0.000691	-0.205	-0.189				*CHRNA2*	TSS1500
cg01743846	9	127,176,827	1.21E-05	-0.205	-0.193				*PSMB7*	Body
cg24656517	4	40,844,307	2.93E-05	-0.205	-0.229				*APBB2*	Body
cg19667460	15	60,863,379	0.000169	-0.205	-0.220				*RORA*	Body

cg22706106	1	153,607,701	6.5E-10	-0.205	-0.191				*C1orf77*	5'UTR
cg14971327	5	4,230,502	0.037134	-0.205	-0.120					
cg22062667	2	187,081,534	0.009625	-0.205	-0.241					
cg24732563	1	204,349,887	1.5E-05	-0.205	-0.180					
cg15252768	10	133,273,850	0.035042	-0.205	-0.066					
cg05060976	18	21,741,492	0.000319	-0.205	-0.183				*CABYR*	3'UTR
cg22413399	11	55,028,287	0.004356	-0.205	-0.134				*TRIM48*	TSS1500
cg00989002	16	52,225,569	2.41E-05	-0.205	-0.171					
cg01871498	16	4,855,523	0.000138	-0.205	-0.172			Yes	*GLYR1*	Body
cg07882687	X	49,460,608	0.031061	-0.205	-0.105				*PAGE1*	TSS200
cg21347412	19	4,339,217	0.000142	-0.205	-0.167				*STAP2*	TSS1500
cg02562419	6	159,049,626	0.003393	-0.205	-0.239				*TMEM181*	Body
cg27599015	20	50,175,489	0.036012	-0.205	-0.200				*NFATC2*	Body
cg05229723	12	104,526,470	2.32E-06	-0.205	-0.238				*NFYB*	Body
cg13540981	6	32,011,506	0.000841	-0.205	-0.112				*TNXB*	Body
cg24573321	6	69,726,000	0.001454	-0.205	-0.218				*BAI3*	Body
cg00558645	1	218,711,575	8.2E-06	-0.205	-0.238					
cg07863354	17	80,832,611	5.53E-05	-0.205	-0.215				*TBCD*	Body
cg23167506	7	1,303,302	2.73E-07	-0.205	-0.208					
cg12617456	1	85,240,821	1.1E-05	-0.205	-0.184					
cg23511851	13	108,087,589	0.001333	-0.205	-0.143				*FAM155A*	Body
cg02216731	16	801,936	3.08E-06	-0.205	-0.201					
cg08695113	10	74,842,425	0.001574	-0.205	-0.171				*P4HA1*	5'UTR
cg09324663	6	41,621,232	2.83E-18	-0.205	-0.207				*MDFI*	Body
cg12025690	13	107,991,871	0.030867	-0.205	-0.114				*FAM155A*	Body
cg12413346	15	43,425,285	5.83E-08	-0.205	-0.219				*TMEM62*	TSS1500
cg01597784	2	89,986,470	5.94E-05	-0.205	-0.185					
cg10911747	4	15,171,497	0.003591	-0.205	-0.127					
cg00163727	6	98,989,775	0.033928	-0.205	-0.127					
cg00778084	1	173,681,595	0.01725	-0.205	-0.099					
cg03815116	4	44,654,206	0.004712	-0.205	-0.177				*YIPF7*	TSS1500
cg13990848	9	93,883,110	9.02E-07	-0.205	-0.205					
cg20168964	12	130,333,393	0.001464	-0.205	-0.222				*TMEM132D*	Body
cg15444358	14	102,051,071	0.041775	-0.205	-0.168					

cg23604959	X	153,283,694	0.000167	-0.205	-0.164				*IRAK1*	Body
cg03654169	8	11,710,911	1.71E-05	-0.205	-0.168				*CTSB*	Body
cg24531714	8	67,475,459	0.000921	-0.205	-0.181				*MYBL1*	3'UTR
cg02039214	15	88,521,695	0.01039	-0.205	-0.160				*NTRK3*	Body
cg15983538	1	156,123,411	7.9E-05	-0.205	-0.205	7.62E-07	-0.081		*SEMA4A*	5'UTR
cg25605731	19	13,054,434	0.005867	-0.205	-0.164				*CALR*	Body
cg27501612	16	56,977,363	0.000727	-0.205	-0.200				*HERPUD1*	3'UTR
cg15405588	13	33,854,227	0.002999	-0.205	-0.169				*STARD13*	Body
cg06079062	3	108,672,412	0.005318	-0.205	-0.158				*GUCA1C*	1stExon
cg23225508	17	62,500,377	8.37E-11	-0.205	-0.182				*DDX5*	Body
cg16249827	19	11,153,168	1.15E-06	-0.205	-0.201				*SMARCA4*	Body
cg00915830	12	34,448,653	0.006073	-0.205	-0.140					
cg09940861	14	101,201,649	0.024954	-0.205	-0.122					
cg23920120	11	122,239,845	0.002101	-0.205	-0.188					
cg18695670	14	61,123,667	3.02E-13	-0.205	-0.175					
cg25503323	7	36,567,098	0.004614	-0.205	-0.148				*AOAH*	Body
cg15379170	2	233,476,236	1.46E-09	-0.205	-0.172				*EFHD1*	Body
cg16564358	16	85,912,452	0.028864	-0.205	-0.111					
cg00861695	13	24,736,455	0.00011	-0.205	-0.177				*SPATA13*	5'UTR
cg06183336	13	112,196,813	0.009635	-0.205	-0.082					
cg23201841	1	205,602,113	2.18E-09	-0.205	-0.203				*ELK4*	TSS200
cg24897320	1	110,042,528	9.47E-08	-0.205	-0.177				*CYB561D1*	3'UTR
cg15214183	11	67,232,269	1.56E-06	-0.205	-0.178				*TMEM134*	Body
cg13933535	X	96,795,331	6.96E-10	-0.205	-0.207				*DIAPH2*	Body
cg10351740	7	38,470,121	0.027991	-0.205	-0.208				*AMPH*	Body
cg20318358	12	108,167,448	3.2E-05	-0.205	-0.200				*ASCL4*	TSS1500
cg11108702	11	67,432,681	0.047632	-0.205	-0.102				*ALDH3B2*	Body
cg21226735	9	92,807,111	0.000205	-0.205	-0.229					
cg02707593	5	54,823,369	0.001048	-0.205	-0.204			Yes	*PPAP2A*	Body
cg03533858	1	2,324,396	1.24E-07	-0.205	-0.213	1.2E-16	-0.223		*MORN1*	TSS1500
cg03670994	5	6,182,647	0.002211	-0.205	-0.186					
cg09086416	6	50,428,999	0.002233	-0.205	-0.192					
cg06778853	1	6,672,620	8.13E-15	-0.205	-0.209	0.00000887	-0.062		*PHF13*	TSS1500
cg22848704	1	48,648,439	0.000432	-0.205	-0.191				*SKINTL*	TSS1500

cg23494863	8	38,009,476	1.05E-06	-0.205	-0.194				*STAR*	TSS1500
cg24467337	20	31,262,314	0.000919	-0.205	-0.179				*LOC284805*	TSS1500
cg05168215	12	18,433,545	0.019246	-0.205	-0.149				*PIK3C2G*	5'UTR
cg13260377	6	31,592,983	0.000562	-0.205	-0.201				*BAT2*	Body
cg14848461	3	74,060,982	2.83E-07	-0.205	-0.188					
cg17477990	1	144,937,317	9.72E-06	-0.205	-0.208				*PDE4DIP*	Body
cg20157097	10	6,569,802	0.010777	-0.205	-0.133				*PRKCQ*	5'UTR
cg10690657	21	15,215,654	0.003676	-0.205	-0.151				*C21orf15*	Body
cg06002305	5	178,591,479	0.036383	-0.205	-0.120				*ADAMTS2*	Body
cg19618279	17	40,715,228	3.28E-14	-0.205	-0.193				*COASY*	1stExon
cg02861926	19	56,348,742	1.7E-06	-0.205	-0.216				*NLRP11*	TSS1500
cg21003380	1	156,261,207	1.17E-07	-0.205	-0.200				*TMEM79*	Body
cg24872815	2	65,623,541	8.62E-10	-0.205	-0.209				*SPRED2*	Body
cg25755428	19	13,875,111	0.03685	-0.205	-0.172				*MRI1*	TSS1500
cg10946404	8	59,575,772	6.57E-06	-0.205	-0.217					
cg15531099	1	152,770,502	2.04E-05	-0.205	-0.180				*LCE1D*	Body
cg08382332	2	58,728,740	0.000953	-0.205	-0.148					
cg14377739	9	139,699,037	5.79E-06	-0.205	-0.208				*KIAA1984*	Body
cg08614769	6	1,679,393	4.2E-07	-0.205	-0.201				*GMDS*	Body
cg12443030	1	75,565,024	0.002073	-0.205	-0.177					
cg08478672	8	129,374,295	1.07E-08	-0.205	-0.189					
cg16050028	17	6,068,454	0.038533	-0.205	-0.087					
cg20519577	12	132,329,360	0.002584	-0.205	-0.077				*MMP17*	Body
cg07596401	10	126,150,086	1.27E-07	-0.205	-0.188				*LHPP*	TSS1500
cg08198470	17	1,801,637	1.8E-05	-0.205	-0.227				*RPA1*	3'UTR
cg19927661	6	3,150,267	0.003174	-0.205	-0.156				*BPHL*	Body
cg00793946	2	46,163,986	4.1E-06	-0.205	-0.170				*PRKCE*	Body
cg02018326	19	51,670,251	0.011346	-0.205	-0.175				*SIGLECP3*	TSS1500
cg12667125	1	36,181,447	9.15E-17	-0.205	-0.187				*C1orf216*	Body
cg17421561	7	31,726,639	0.034605	-0.205	-0.087				*C7orf16*	TSS200
cg04984818	14	104,195,301	3.07E-05	-0.205	-0.205				*ZFYVE21*	Body
cg12136883	4	120,613,606	0.025043	-0.205	-0.176					
cg18010085	6	1,297,024	7.93E-06	-0.205	-0.191					
cg26738530	15	71,581,059	5.91E-05	-0.205	-0.162				*THSD4*	Body

cg02034204	1	8,394,077	0.005437	-0.205	-0.119					*SLC45A1*	Body
cg11481675	8	32,504,965	0.002067	-0.205	-0.188					*NRG1*	Body
cg17294330	17	77,089,070	0.007163	-0.205	-0.092					*HRNBP3*	Body
cg18898325	22	42,394,382	0.003997	-0.205	-0.235					*WBP2NL*	TSS1500
cg27534624	12	7,072,727	0.000555	-0.205	-0.190					*MIR141*	TSS1500
cg23082003	13	21,713,913	2.1E-05	-0.205	-0.190					*SAP18*	TSS1500
cg26751513	16	1,542,910	0.000498	-0.205	-0.186					*TELO2*	TSS1500
cg01502919	6	124,122,318	0.000195	-0.205	-0.195						
cg16580616	1	235,256,519	0.044793	-0.205	-0.176						
cg23557769	22	16,871,766	0.003151	-0.205	-0.160						
cg09631495	8	4,617,087	0.011384	-0.205	-0.186					*CSMD1*	Body
cg11154040	4	3,795,063	0.044281	-0.205	-0.122						
cg21996360	1	215,940,809	0.0303	-0.205	-0.118					*USH2A*	Body
cg00607609	4	119,962,922	1.25E-07	-0.205	-0.171					*SYNPO2*	Body
cg05082563	7	137,775,882	0.002207	-0.205	-0.094					*AKR1D1*	Body
cg19788754	17	80,847,662	0.00011	-0.205	-0.203					*TBCD*	Body
cg24324837	19	49,891,574	0.023378	-0.205	-0.265					*CCDC155*	1stExon
cg02973354	14	70,316,670	0.011478	-0.205	-0.081						
cg27445072	16	82,921,787	0.016333	-0.205	-0.198					*CDH13*	Body
cg27431625	2	107,679,531	3.42E-05	-0.206	-0.145						
cg15795186	4	58,291,839	0.024291	-0.206	-0.120						
cg04687551	12	129,187,286	0.030402	-0.206	-0.170					*TMEM132C*	Body
cg12976310	X	113,865,129	0.017398	-0.206	-0.260					*SNORA35*	TSS200
cg19611822	15	96,101,553	1.25E-05	-0.206	-0.222						
cg00159764	20	35,059,674	0.001789	-0.206	-0.153					*DLGAP4*	5'UTR
cg04121368	10	122,316,088	0.038563	-0.206	-0.216					*PPAPDC1A*	Body
cg16444826	2	30,441,244	3.52E-05	-0.206	-0.204						
cg05070250	11	36,620,199	0.004925	-0.206	-0.106					*C11orf74*	5'UTR
cg11152263	7	569,642	0.010644	-0.206	-0.163						
cg26995656	14	88,121,644	0.016378	-0.206	-0.139						
cg02281821	10	129,692,341	1.12E-09	-0.206	-0.215					*CLRN3*	TSS1500
cg01566170	1	223,899,746	0.000347	-0.206	-0.147					*CAPN2*	TSS1500
cg23010693	14	103,911,341	5.54E-06	-0.206	-0.182					*MARK3*	Body
cg04741728	12	133,003,907	0.013067	-0.206	-0.228						

cg09328012	22	46,481,677	2.19E-07	-0.206	-0.179				*LOC400931*	TSS200
cg16924061	18	71,955,528	7.74E-05	-0.206	-0.182				*CYB5A*	Body
cg26813137	5	151,784,326	0.025026	-0.206	-0.239				*NMUR2*	1stExon
cg11793422	19	15,239,169	1.36E-06	-0.206	-0.203					
cg17723122	1	205,641,542	3.11E-12	-0.206	-0.198				*SLC45A3*	5'UTR
cg14883135	2	43,051,175	0.000386	-0.206	-0.178			Yes		
cg13377341	6	41,715,281	0.000553	-0.206	-0.202				*PGC*	TSS200
cg14467464	17	7,960,406	0.000262	-0.206	-0.178					
cg18140889	1	153,177,577	0.000371	-0.206	-0.198				*LELP1*	3'UTR
cg01126560	9	139,886,304	9.22E-07	-0.206	-0.205	1.19E-12	-0.103		*C9orf142*	TSS1500
cg16863332	10	64,566,678	9.05E-07	-0.206	-0.217				*ADO*	3'UTR
cg23781075	20	170,581	0.000127	-0.206	-0.155				*DEFB128*	TSS1500
cg09365529	1	75,140,289	0.013312	-0.206	-0.101				*C1orf173*	TSS1500
cg26652266	10	90,932,152	0.029146	-0.206	-0.111					
cg00961325	7	41,547,329	1.25E-07	-0.206	-0.198					
cg11365072	5	154,392,274	0.000277	-0.206	-0.185				*KIF4B*	TSS1500
cg10449558	17	70,552,917	0.000197	-0.206	-0.137					
cg00225006	15	83,477,872	6.13E-06	-0.206	-0.221				*WHAMM*	TSS200
cg05418395	2	58,689,455	0.040814	-0.206	-0.101					
cg26175729	7	150,726,136	3.95E-12	-0.206	-0.211				*ABCB8*	Body
cg26898869	8	56,364,964	0.017806	-0.206	-0.169				*XKR4*	Body
cg14016200	19	10,399,157	0.005532	-0.206	-0.173				*ICAM5*	TSS1500
cg02475600	12	34,503,594	0.013755	-0.206	-0.159					
cg08008692	1	159,961,591	0.000336	-0.206	-0.185					
cg15746620	19	14,785,849	5.1E-10	-0.206	-0.195	1.5E-13	-0.173		*EMR3*	TSS200
cg19284751	2	119,699,789	0.003808	-0.206	-0.220				*MARCO*	1stExon
cg03197032	15	36,708,407	0.007228	-0.206	-0.307					
cg14381543	9	93,798,720	0.003684	-0.206	-0.227					
cg19190519	11	123,175,740	0.015824	-0.206	-0.126					
cg05582397	12	8,674,684	0.001916	-0.206	-0.166				*CLEC4D*	3'UTR
cg15896624	4	164,246,900	0.018899	-0.206	-0.138				*NPY1R*	Body
cg02344361	10	134,806,481	2.76E-05	-0.206	-0.195					
cg20424036	8	32,777,053	0.006367	-0.206	-0.123					
cg02103822	1	147,246,329	0.004602	-0.206	-0.132				*GJA5*	TSS1500

cg10153214	16	71,571,146	3.01E-05	-0.206	-0.143				*CHST4*	Body
cg10803722	21	46,713,166	0.000532	-0.206	-0.131				*LOC642852*	Body
cg15669228	9	21,409,505	0.048344	-0.206	-0.139	3.15E-07	-0.155		*IFNA8*	1stExon
cg22288927	4	144,482,688	2.2E-06	-0.206	-0.211					
cg00126657	X	47,967,250	0.004131	-0.206	-0.144	6.75E-12	-0.138		*SSX6*	TSS200
cg12043732	20	61,284,223	1E-19	-0.206	-0.180				*SLCO4A1*	5'UTR
cg20140940	1	241,390,013	0.024876	-0.206	-0.239				*RGS7*	Body
cg26561254	15	89,164,044	5.56E-09	-0.206	-0.199	4.74E-09	-0.075		*AEN*	TSS1500
cg11371706	10	117,817,578	0.048447	-0.206	-0.123				*GFRA1*	3'UTR
cg19270309	17	77,712,853	0.000457	-0.206	-0.181				*ENPP7*	3'UTR
cg07696099	8	133,573,809	0.000138	-0.206	-0.208				*HPYR1*	TSS200
cg02608124	11	18,193,422	0.002653	-0.206	-0.132				*MRGPRX4*	TSS1500
cg06853230	7	57,832,834	0.010283	-0.206	-0.185					
cg12030523	5	126,984,765	1.08E-06	-0.206	-0.202				*CTXN3*	1stExon
cg18890615	13	112,161,629	0.009452	-0.206	-0.164			Yes		
cg20025677	6	44,217,736	8.33E-05	-0.206	-0.166				*HSP90AB1*	Body
cg00231085	5	1,773,166	0.001474	-0.206	-0.168					
cg13441891	16	85,648,370	1.53E-05	-0.206	-0.194				*KIAA0182*	5'UTR
cg05008296	14	68,163,792	4.24E-06	-0.206	-0.197				*RDH11*	TSS1500
cg19511425	X	13,749,859	5.48E-06	-0.206	-0.195				*TRAPPC2*	5'UTR
cg02835977	1	37,782,729	0.027638	-0.206	-0.130					
cg02658528	13	113,689,139	7.02E-06	-0.206	-0.165				*MCF2L*	Body
cg03538079	7	34,874,196	0.008301	-0.206	-0.175				*AAA1*	TSS1500
cg05553829	8	57,307,649	0.000264	-0.206	-0.209				*SDR16C6*	1stExon
cg24459209	11	57,148,215	7.4E-05	-0.206	-0.244	0.00807386	-0.065		*PRG3*	5'UTR
cg07664312	12	18,433,550	0.014345	-0.206	-0.164				*PIK3C2G*	5'UTR
cg23949925	11	68,147,880	4.79E-07	-0.206	-0.167				*LRP5*	Body
cg22416002	22	39,713,062	2.11E-09	-0.206	-0.182				*RPL3*	Body
cg04479876	5	38,479,736	0.003226	-0.206	-0.187				*LIFR*	3'UTR
cg20868817	1	160,677,398	0.000785	-0.206	-0.188				*CD48*	Body
cg21691065	1	2,804,835	0.00603	-0.206	-0.139					
cg20619751	13	114,092,131	8.35E-08	-0.206	-0.181				*ADPRHL1*	Body
cg02022322	12	109,576,193	0.022782	-0.206	-0.113				*ACACB*	TSS1500
cg21293933	20	42,539,946	1.25E-05	-0.206	-0.192					

cg21730821	X	100,717,965	0.019894	-0.206	-0.222				*ARMCX4*	Body
cg25073998	2	47,492,481	0.001275	-0.206	-0.192					
cg26131315	4	140,787,316	1.36E-07	-0.206	-0.201				*MAML3*	Body
cg23108559	14	55,491,009	0.021905	-0.206	-0.216				*WDHD1*	5'UTR
cg07897871	1	201,979,846	9.47E-05	-0.206	-0.204				*ELF3*	5'UTR
cg06321300	1	104,239,696	0.031932	-0.206	-0.097				*AMY1B*	TSS1500
cg26277069	10	19,352,926	0.002507	-0.206	-0.124					
cg12300018	8	63,903,574	0.048008	-0.206	-0.157				*NKAIN3*	3'UTR
cg12611448	3	185,255,217	5.77E-10	-0.206	-0.203				*LIPH*	Body
cg19045344	X	84,364,350	0.030174	-0.206	-0.107				*SATL1*	TSS1500
cg00111778	2	3,833,127	5.99E-08	-0.206	-0.213					
cg07625619	16	69,050,940	3.1E-05	-0.206	-0.196				*TMCO7*	Body
cg10987788	11	34,535,377	0.009252	-0.206	-0.207				*ELF5*	TSS200
cg21568141	6	169,235,962	9.93E-05	-0.206	-0.101					
cg18884447	14	105,018,912	0.003779	-0.206	-0.080					
cg05924537	3	169,588,423	0.014169	-0.206	-0.297				*LRRC31*	TSS1500
cg23735602	3	178,276,672	2.97E-05	-0.206	-0.222				*KCNMB2*	5'UTR
cg01241450	1	3,110,867	0.043481	-0.206	-0.049				*PRDM16*	Body
cg04456892	11	6,898,245	0.038728	-0.206	-0.234				*OR10A4*	1stExon
cg07612300	2	242,033,426	1.59E-05	-0.206	-0.200				*MTERFD2*	Body
cg03574946	4	65,723,509	0.016598	-0.206	-0.143					
cg22512041	2	22,504,250	0.001193	-0.206	-0.133					
cg22759185	19	1,492,038	7.7E-06	-0.206	-0.230	4.88E-23	-0.158		*REEP6*	Body
cg06758484	10	109,671,761	0.019569	-0.207	-0.160					
cg07340894	8	8,238,774	0.002987	-0.207	-0.204				*PRAGMIN*	Body
cg00095629	6	168,811,070	0.002028	-0.207	-0.179					
cg14852603	6	164,585,948	0.026176	-0.207	-0.197					
cg18934443	7	149,569,776	1.94E-06	-0.207	-0.220				*ATP6V0E2*	TSS1500
cg20900312	14	77,670,584	7.05E-06	-0.207	-0.221				*TMEM63C*	5'UTR
cg16974699	22	20,858,742	1.23E-07	-0.207	-0.167					
cg16928869	20	60,397,766	0.023876	-0.207	-0.135			Yes	*CDH4*	Body
cg24750674	17	79,097,243	0.029145	-0.207	-0.168				*AATK*	Body
cg05134325	13	52,423,122	2.07E-05	-0.207	-0.194					
cg27118080	6	159,618,364	0.000623	-0.207	-0.222				*FNDC1*	Body

cg02760280	1	21,993,555	0.001451	-0.207	-0.210				*RAP1GAP*	5'UTR
cg07804658	5	100,108,624	0.00276	-0.207	-0.222					
cg17815049	1	246,728,663	1.6E-08	-0.207	-0.176				*CNST*	TSS1500
cg06852395	17	7,478,131	2.09E-09	-0.207	-0.177				*EIF4A1*	Body
cg13621396	4	26,861,261	0.000132	-0.207	-0.195				*STIM2*	TSS1500
cg10391102	1	3,206,316	0.008536	-0.207	-0.104				*PRDM16*	Body
cg27312242	15	50,116,359	1.21E-05	-0.207	-0.219					
cg15042473	X	103,294,786	0.012849	-0.207	-0.149				*H2BFM*	1stExon
cg23995738	15	102,308,058	1.04E-05	-0.207	-0.142					
cg06497051	1	156,261,200	1.59E-08	-0.207	-0.183				*TMEM79*	Body
cg24494262	4	155,339,844	0.000332	-0.207	-0.106				*DCHS2*	Body
cg24950067	21	43,406,941	8.59E-05	-0.207	-0.150				*ZNF295*	3'UTR
cg13616190	8	88,041,484	0.022324	-0.207	-0.177				*CNBD1*	Body
cg27312652	21	38,073,517	5.63E-06	-0.207	-0.201				*SIM2*	Body
cg13765621	1	158,149,228	2.79E-05	-0.207	-0.172	1.17E-15	-0.179		*CD1D*	TSS1500
cg12069151	6	30,580,546	9.92E-05	-0.207	-0.176				*PPP1R10*	5'UTR
cg09473585	3	62,305,519	0.000145	-0.207	-0.208	0.00014063	-0.104		*C3orf14*	5'UTR
cg06572974	15	93,442,219	0.002355	-0.207	-0.196	8.1E-08	-0.134		*CHD2*	TSS1500
cg23817132	13	56,790,593	0.007435	-0.207	-0.141					
cg27139836	2	219,868,065	0.039299	-0.207	-0.212				*CCDC108*	Body
cg07282879	11	49,843,431	1.6E-05	-0.207	-0.194					
cg02441982	17	76,183,641	4.1E-05	-0.207	-0.183				*AFMID*	Body
cg04587424	2	776,719	1.15E-12	-0.207	-0.198					
cg03527802	16	29,819,931	7.52E-21	-0.207	-0.197				*MAZ*	Body
cg04117590	3	139,346,788	2.17E-06	-0.207	-0.184				*NMNAT3*	5'UTR
cg06526622	2	196,458,613	0.01735	-0.207	-0.182					
cg11841867	18	11,153,912	1.37E-06	-0.207	-0.201					
cg15335683	11	66,465,299	0.001356	-0.207	-0.186			Yes	*SPTBN2*	Body
cg23709855	14	21,927,732	0.000445	-0.207	-0.169				*RAB2B*	Body
cg03901431	16	85,517,684	6.76E-06	-0.207	-0.140					
cg19539385	7	6,095,195	1.41E-05	-0.207	-0.212				*EIF2AK1*	Body
cg25002350	15	94,771,562	3.59E-06	-0.207	-0.196					
cg03310594	7	22,704,316	0.001229	-0.207	-0.156					
cg11323828	15	41,230,578	3.7E-09	-0.207	-0.186				*DLL4*	3'UTR

cg15714227	5	2,225,482	0.003351	-0.207	-0.255						
cg03815683	16	61,897,489	0.044424	-0.207	-0.100					*CDH8*	Body
cg13692426	9	79,625,269	6.03E-06	-0.207	-0.191						
cg02662170	10	133,508,385	0.035142	-0.207	-0.102						
cg08448341	6	134,324,873	2.92E-05	-0.207	-0.223					*SLC2A12*	Body
cg21902964	14	101,198,397	0.019463	-0.207	-0.103					*DLK1*	Body
cg04094548	1	39,301,897	0.00761	-0.207	-0.123						
cg14492992	15	53,101,548	0.014897	-0.207	-0.209						
cg24849831	17	17,389,971	0.003312	-0.207	-0.122					*MED9*	Body
cg06036459	18	75,884,536	0.035927	-0.207	-0.091						
cg12911556	7	26,403,780	8.41E-06	-0.207	-0.208					*SNX10*	Body
cg15778089	3	35,721,037	0.049972	-0.207	-0.169					*ARPP-21*	5'UTR
cg02050028	7	43,156,037	0.00855	-0.207	-0.139					*HECW1*	5'UTR
cg00862016	3	13,859,125	0.026007	-0.207	-0.164						
cg01449973	16	87,027,662	0.046494	-0.207	-0.084						
cg01799015	19	707,791	1.23E-08	-0.207	-0.200					*PALM*	TSS1500
cg04170298	15	86,805,563	9.87E-05	-0.207	-0.196					*AGBL1*	Body
cg06538345	19	49,993,186	2.04E-06	-0.207	-0.206					*RPL13AP5*	Body
cg25963412	4	8,412,369	0.003491	-0.207	-0.174					*ACOX3*	Body
cg20128319	2	97,174,815	1.21E-05	-0.207	-0.154					*NEURL3*	TSS1500
cg27156515	11	9,879,023	7.36E-06	-0.207	-0.207					*SBF2*	Body
cg11186224	6	132,859,485	0.003204	-0.207	-0.136					*TAAR9*	1stExon
cg26654827	7	100,738,414	0.000268	-0.207	-0.128						
cg17869851	1	230,976,414	0.001145	-0.207	-0.192					*C1orf198*	Body
cg00574819	4	128,981,252	2.25E-06	-0.207	-0.209					*LARP1B*	TSS1500
cg00150837	3	52,721,831	3.68E-08	-0.207	-0.207					*GNL3*	Body
cg00871207	14	89,080,250	0.000114	-0.207	-0.195						
cg18758405	10	107,451,306	3.27E-06	-0.207	-0.192						
cg03514770	8	19,313,167	0.000745	-0.207	-0.207					*CSGALNACT1*	Body
cg18416585	4	62,756,100	0.041607	-0.207	-0.156					*LPHN3*	Body
cg12273506	10	70,627,857	0.011551	-0.207	-0.160					*STOX1*	5'UTR
cg14933439	16	49,407,794	0.015736	-0.207	-0.209					*C16orf78*	TSS200
cg14711592	1	157,839,587	0.008858	-0.207	-0.172						
cg00592494	X	75,249,222	0.022481	-0.207	-0.175						

cg12200188	7	111,496,282	0.0013	-0.207	-0.200				*DOCK4*	Body
cg01115380	6	1,552,904	0.001051	-0.207	-0.102					
cg11253879	1	3,664,522	2.6E-07	-0.207	-0.199				*KIAA0495*	TSS1500
cg17488785	X	140,009,093	0.016547	-0.207	-0.227				*MIR320D2*	TSS1500
cg19386998	6	28,351,829	2.04E-05	-0.207	-0.126				*ZSCAN12*	Body
cg03021100	3	31,542,063	0.007092	-0.207	-0.149					
cg08269733	16	62,057,179	0.001535	-0.207	-0.116				*CDH8*	5'UTR
cg27280332	12	77,378,563	0.002857	-0.207	-0.217					
cg25564800	3	122,234,134	4.63E-10	-0.207	-0.219	6.63E-15	-0.070		*KPNA1*	TSS1500
cg06818582	15	67,286,903	0.002416	-0.207	-0.174					
cg11583392	7	103,087,458	2.32E-08	-0.207	-0.209				*SLC26A5*	TSS1500
cg18560366	3	109,057,564	0.006342	-0.207	-0.155				*DPPA4*	TSS1500
cg12349422	3	52,485,216	0.001787	-0.207	-0.106				*TNNC1*	3'UTR
cg14398214	17	56,493,260	0.000328	-0.207	-0.211				*RNF43*	5'UTR
cg03331924	1	208,038,413	3.03E-05	-0.207	-0.193					
cg12423553	21	47,541,643	0.028808	-0.207	-0.105				*COL6A2*	Body
cg16604658	12	64,847,188	3.22E-06	-0.207	-0.184				*TBK1*	5'UTR
cg04927725	8	30,670,732	2.83E-05	-0.207	-0.185				*PPP2CB*	TSS1500
cg14481339	11	128,737,319	8.36E-05	-0.207	-0.211	2.34E-08	-0.097		*KCNJ1*	TSS200
cg21439896	1	36,108,233	0.002992	-0.207	-0.162				*PSMB2*	TSS1500
cg04578317	14	105,189,737	0.000001	-0.207	-0.226				*ADSSL1*	TSS1500
cg01150351	1	4,220,528	0.002907	-0.207	-0.110					
cg02096790	16	89,920,010	0.028631	-0.207	-0.120				*SPIRE2*	Body
cg07516742	8	55,383,951	0.018437	-0.207	-0.140					
cg02391410	16	59,790,082	0.012452	-0.207	-0.148				*LOC644649*	TSS1500
cg20239081	12	121,687,822	0.000362	-0.207	-0.139				*CAMKK2*	Body
cg02072170	3	194,406,190	0.000104	-0.207	-0.139				*FAM43A*	TSS1500
cg03457781	3	20,418,856	0.009872	-0.207	-0.196			Yes		
cg16596250	7	53,099,739	0.026371	-0.207	-0.129					
cg24288715	6	163,745,629	1.11E-05	-0.207	-0.219				*LOC285796*	TSS200
cg27056759	14	105,373,391	0.007892	-0.207	-0.140					
cg01790523	2	167,229,656	0.012227	-0.207	-0.148				*SCN9A*	5'UTR
cg13423469	10	116,856,408	4.75E-05	-0.207	-0.210				*ATRNL1*	Body
cg04329382	22	46,480,891	3.11E-07	-0.207	-0.188				*LOC400931*	TSS1500

cg11252555	19	49,993,865	0.032695	-0.207	-0.120				*RPL13AP5*	Body
cg25960090	5	127,277,370	0.00018	-0.207	-0.202					
cg17007389	7	55,874,624	0.044719	-0.207	-0.099				*SEPT14*	Body
cg21771528	5	18,745,861	0.01711	-0.207	-0.175					
cg12920333	14	71,805,172	1.6E-07	-0.207	-0.224					
cg18146074	2	232,242,955	0.000499	-0.207	-0.183					
cg16459890	11	104,916,331	0.044421	-0.208	-0.118				*CASP1*	Body
cg05982301	5	121,751,763	0.000111	-0.208	-0.172				*SNCAIP*	Body
cg15163151	8	100,906,373	0.001071	-0.208	-0.225				*COX6C*	TSS1500
cg15328241	2	80,547,585	0.001478	-0.208	-0.247				*CTNNA2*	Body
cg18087953	11	68,964,113	0.000531	-0.208	-0.121					
cg06276810	6	153,224,221	0.003312	-0.208	-0.136					
cg22608619	1	10,703,231	0.001396	-0.208	-0.154				*CASZ1*	Body
cg02339243	19	8,810,253	0.011717	-0.208	-0.150				*ACTL9*	TSS1500
cg01386157	6	29,403,982	0.011111	-0.208	-0.190					
cg15618525	5	180,022,909	5.26E-05	-0.208	-0.196					
cg04010040	4	93,646,144	0.000558	-0.208	-0.162				*GRID2*	Body
cg08187034	2	38,970,936	2.76E-08	-0.208	-0.200				*SFRS7*	3'UTR
cg09109133	16	82,688,759	0.012268	-0.208	-0.178				*CDH13*	Body
cg08048699	11	57,296,305	7.22E-08	-0.208	-0.206				*TIMM10*	Body
cg15082843	7	130,371,296	0.000212	-0.208	-0.143				*TSGA13*	5'UTR
cg04568121	13	102,072,766	0.005918	-0.208	-0.158					
cg16471612	5	127,294,693	0.000102	-0.208	-0.200					
cg04286438	8	144,898,672	5.85E-10	-0.208	-0.172				*PUF60*	3'UTR
cg17934470	5	49,959,703	0.000223	-0.208	-0.165					
cg26753302	18	61,143,993	0.000693	-0.208	-0.178				*SERPINB5*	TSS200
cg27508281	16	87,027,322	0.040179	-0.208	-0.072					
cg04334496	6	133,953,605	0.012413	-0.208	-0.174					
cg12072611	14	22,433,693	0.008749	-0.208	-0.188					
cg12391048	16	2,770,336	0.000289	-0.208	-0.243	4.49E-09	-0.110		*PRSS27*	1stExon
cg10082354	11	131,672,204	0.003547	-0.208	-0.192				*NTM*	Body
cg14733867	8	144,884,056	0.01201	-0.208	-0.180				*SCRIB*	Body
cg15008587	4	122,927,073	0.013806	-0.208	-0.184					
cg01469547	11	7,847,155	0.016088	-0.208	-0.165	0.00000644	-0.129	Yes	*OR5P3*	1stExon

cg04397593	16	55,880,243	0.009969	-0.208	-0.136			Yes	*CES7*	3'UTR
cg21610297	12	53,857,464	0.000951	-0.208	-0.198				*PCBP2*	Body
cg22619784	6	32,793,355	6.65E-07	-0.208	-0.206				*TAP2*	3'UTR
cg27295288	14	58,616,337	0.015198	-0.208	-0.217				*C14orf37*	5'UTR
cg12792367	7	100,238,750	0.008784	-0.208	-0.125	5.31E-10	-0.094		*TFR2*	Body
cg19107435	3	191,588,392	0.046203	-0.208	-0.089					
cg21322022	2	115,421,931	0.021156	-0.208	-0.132				*DPP10*	Body
cg03813951	X	52,684,130	0.002612	-0.208	-0.165				*SSX7*	TSS200
cg02244933	16	71,324,066	1.85E-08	-0.208	-0.246				*FTSJD1*	TSS1500
cg23988024	19	594,272	0.000628	-0.208	-0.146				*HCN2*	Body
cg08385211	10	50,118,141	0.038375	-0.208	-0.106				*LRRC18*	3'UTR
cg25342642	1	2,743,491	0.001394	-0.208	-0.189					
cg05836475	X	73,163,537	6.65E-09	-0.208	-0.207				*LOC554203*	TSS1500
cg10708400	6	30,130,449	7.55E-08	-0.208	-0.185				*TRIM15*	TSS1500
cg14924160	13	41,496,235	0.036017	-0.208	-0.278				*SUGT1L1*	TSS1500
cg19359983	13	103,346,900	0.031936	-0.208	-0.084				*C13orf39*	TSS200
cg22901249	8	5,762,990	0.042962	-0.208	-0.137					
cg27058221	16	14,766,421	4.94E-07	-0.208	-0.221				*PLA2G10*	3'UTR
cg09684245	1	57,263,497	0.000965	-0.208	-0.177				*C1orf168*	Body
cg20695587	4	68,995,759	2.2E-05	-0.208	-0.195	0.00235185	-0.050		*TMPRSS11F*	TSS200
cg01311955	11	132,965,250	0.018294	-0.208	-0.103				*OPCML*	Body
cg02016764	4	38,805,732	3.28E-10	-0.208	-0.197				*TLR1*	5'UTR
cg05696436	16	1,373,786	2.11E-07	-0.208	-0.214				*UBE2I*	Body
cg08195632	16	51,059,377	0.020342	-0.208	-0.187					
cg10067062	21	44,086,302	4.5E-19	-0.208	-0.198				*PDE9A*	Body
cg05881682	3	95,402,607	3.34E-05	-0.208	-0.185					
cg12950492	4	187,000,846	0.001758	-0.208	-0.223				*TLR3*	Body
cg15613991	7	5,277,080	2.02E-05	-0.208	-0.165					
cg26532664	1	2,739,523	0.004402	-0.208	-0.159					
cg02074956	16	4,730,657	4.73E-07	-0.208	-0.170				*MGRN1*	Body
cg04790169	11	1,673,436	0.032161	-0.208	-0.156				*HCCA2*	Body
cg23589587	1	75,558,249	3.02E-06	-0.208	-0.204					
cg06768993	4	8,443,412	8.99E-05	-0.208	-0.197				*ACOX3*	TSS1500
cg05078091	11	129,984,345	0.003228	-0.208	-0.218				*APLP2*	Body

cg15001372	7	44,229,892	7.69E-07	-0.208	-0.166				*GCK*	TSS1500
cg17868307	7	73,597,649	2.88E-05	-0.208	-0.229				*EIF4H*	Body
cg00651359	7	132,219,281	0.019654	-0.208	-0.130				*PLXNA4*	5'UTR
cg24466229	8	32,618,341	0.010695	-0.208	-0.188				*NRG1*	Body
cg05119686	10	31,967,157	7.39E-06	-0.208	-0.198					
cg07007062	5	38,417,095	4.58E-06	-0.208	-0.208				*EGFLAM*	Body
cg16031846	8	33,628,265	3.13E-05	-0.208	-0.189					
cg27275374	7	47,539,765	1.22E-07	-0.208	-0.198				*TNS3*	5'UTR
cg02929780	11	59,282,555	0.002756	-0.208	-0.172				*OR4D9*	1stExon
cg18229092	2	196,228,235	1.09E-05	-0.208	-0.188					
cg06746365	7	151,468,796	3.27E-05	-0.208	-0.109				*PRKAG2*	Body
cg12050175	10	44,754,714	2.92E-07	-0.208	-0.204			Yes		
cg00750606	1	20,899,121	1.64E-05	-0.208	-0.176					
cg12074915	7	6,882,112	0.001509	-0.208	-0.223					
cg25485734	10	30,981,289	0.005575	-0.208	-0.142					
cg07672631	10	38,445,710	0.031808	-0.208	-0.145					
cg23290556	10	81,707,922	0.000477	-0.208	-0.157				*SFTPD*	5'UTR
cg02853387	11	117,484,904	0.005976	-0.208	-0.108				*DSCAML1*	Body
cg21150843	4	153,641,409	0.001634	-0.208	-0.212					
cg07483086	2	150,444,834	0.000698	-0.208	-0.212				*MMADHC*	TSS1500
cg23161359	X	21,960,400	3.61E-09	-0.208	-0.164				*SMS*	Body
cg07220366	8	10,405,379	0.001967	-0.208	-0.201					
cg04946506	14	104,646,441	0.03085	-0.208	-0.077				*KIF26A*	3'UTR
cg23768940	12	108,585,952	9.28E-07	-0.208	-0.220				*WSCD2*	5'UTR
cg26522708	1	10,839,450	8.05E-06	-0.208	-0.210				*CASZ1*	5'UTR
cg27418851	10	54,531,653	0.00028	-0.208	-0.182				*MBL2*	TSS200
cg18444589	14	101,521,608	0.029342	-0.208	-0.177				*MIR485*	TSS200
cg19165216	11	811,661	2.45E-11	-0.208	-0.198				*RPLP2*	Body
cg07153978	5	35,773,878	0.000222	-0.208	-0.203				*SPEF2*	Body
cg03097190	22	32,854,361	0.001653	-0.208	-0.164				*BPIL2*	TSS1500
cg13496244	7	4,925,830	0.028275	-0.208	-0.076					
cg00326648	2	61,246,248	6.43E-08	-0.208	-0.173				*PEX13*	Body
cg06111374	12	14,109,584	0.033678	-0.208	-0.206				*GRIN2B*	5'UTR
cg17987059	13	28,752,067	7.48E-05	-0.208	-0.242				*PAN3*	Body

cg02467735	8	143,999,729	0.038642	-0.208	-0.106				*CYP11B2*	TSS1500
cg07592388	3	191,178,814	0.000438	-0.208	-0.128				*PYDC2*	TSS200
cg20837735	18	61,144,177	0.00117	-0.208	-0.186				*SERPINB5*	1stExon
cg16563470	17	40,119,847	2.31E-06	-0.208	-0.200				*CNP*	Body
cg09044294	2	50,234,533	0.002781	-0.208	-0.146				*NRXN1*	Body
cg08607280	19	58,693,421	3.86E-09	-0.208	-0.203				*ZNF274*	TSS1500
cg24333364	12	128,869,782	0.025649	-0.208	-0.151				*TMEM132C*	Body
cg22461018	4	174,538,011	0.008483	-0.208	-0.188	1.01E-07	-0.087		*MORF4*	TSS1500
cg22789515	1	233,432,915	0.000335	-0.208	-0.123				*PCNXL2*	TSS1500
cg02130289	17	80,732,581	4.51E-06	-0.208	-0.192				*TBCD*	Body
cg07142325	1	119,684,001	0.004569	-0.208	-0.137				*WARS2*	TSS1500
cg07990681	16	86,666,077	0.035216	-0.208	-0.129					
cg05305046	5	40,841,315	0.001689	-0.208	-0.177				*CARD6*	TSS200
cg09315586	13	104,168,550	0.005214	-0.208	-0.216					
cg22115706	7	130,125,719	0.006043	-0.208	-0.183				*MEST*	TSS1500
cg02547263	20	32,450,501	5.09E-09	-0.208	-0.199					
cg18674828	12	132,839,436	0.003794	-0.208	-0.112				*GALNT9*	Body
cg20724862	5	84,943,476	0.010662	-0.208	-0.167					
cg16958148	7	49,060,407	5.09E-09	-0.208	-0.222					
cg24393844	12	68,115,110	3.55E-09	-0.208	-0.204					
cg05477331	11	133,716,207	0.040088	-0.208	-0.104				*SPATA19*	TSS1500
cg14308375	4	111,545,119	0.018402	-0.208	-0.132				*PITX2*	TSS1500
cg19970883	15	35,082,573	8.03E-05	-0.208	-0.218				*ACTC1*	3'UTR
cg06917998	14	73,269,031	0.008655	-0.208	-0.226				*DPF3*	Body
cg08099626	17	3,213,032	0.013833	-0.208	-0.159				*OR3A4*	TSS1500
cg06829157	1	93,430,542	2.01E-05	-0.208	-0.177					
cg15528411	18	5,545,169	0.026678	-0.208	-0.243				*EPB41L3*	TSS1500
cg26114457	1	157,895,649	0.001325	-0.208	-0.172					
cg26390400	5	10,556,455	0.000179	-0.208	-0.178					
cg14434922	9	991,047	0.014651	-0.208	-0.143				*DMRT3*	3'UTR
cg00238543	20	56,246,759	0.009835	-0.209	-0.214				*PMEPA1*	5'UTR
cg01449756	4	128,125,758	0.017726	-0.209	-0.140					
cg17987968	5	152,869,882	2.62E-05	-0.209	-0.222				*GRIA1*	TSS1500
cg25546004	11	114,645,223	0.000315	-0.209	-0.168					

cg27027375	4	6,695,319	0.001366	-0.209	-0.221				*S100P*	TSS1500
cg01996004	5	180,338,435	0.006516	-0.209	-0.213				*BTNL8*	5'UTR
cg04844534	11	13,073,904	4.53E-07	-0.209	-0.202					
cg13777287	6	154,830,168	1.95E-11	-0.209	-0.203				*CNKSR3*	Body
cg09601514	2	119,698,352	0.001419	-0.209	-0.233				*MARCO*	TSS1500
cg11728145	2	1,658,190	0.001455	-0.209	-0.187				*PXDN*	Body
cg27304415	3	77,064,282	0.01534	-0.209	-0.169					
cg20932426	1	2,415,221	3.44E-05	-0.209	-0.159				*PLCH2*	Body
cg20312418	10	10,403,467	0.047648	-0.209	-0.181					
cg23395692	12	23,119,040	0.039673	-0.209	-0.142					
cg08234372	16	86,094,189	0.030005	-0.209	-0.076					
cg09076299	10	9,920,000	7.38E-05	-0.209	-0.196					
cg02848961	1	202,091,865	7.77E-05	-0.209	-0.141				*GPR37L1*	TSS200
cg08988797	8	102,505,513	1.86E-13	-0.209	-0.199				*GRHL2*	Body
cg21375452	12	9,997,326	0.011114	-0.209	-0.138				*KLRF1*	3'UTR
cg24057107	7	117,970,353	1.71E-05	-0.209	-0.204					
cg21149595	14	93,813,495	0.013555	-0.209	-0.100				*KIAA1409*	5'UTR
cg23244761	6	161,796,850	0.001368	-0.209	-0.207				*PARK2*	Body
cg00673646	11	59,950,525	7.38E-08	-0.209	-0.211				*MS4A6A*	1stExon
cg09673807	6	33,575,719	5.31E-09	-0.209	-0.180					
cg18639495	10	70,584,876	6.11E-05	-0.209	-0.169					
cg12687069	11	5,474,001	3.16E-05	-0.209	-0.168				*OR51I2*	TSS1500
cg22593395	3	21,038,502	0.042045	-0.209	-0.219					
cg26315457	2	48,759,821	9.68E-11	-0.209	-0.205					
cg09805403	16	12,010,698	6.58E-07	-0.209	-0.224				*GSPT1*	TSS1500
cg13460378	2	88,788,866	0.032169	-0.209	-0.058					
cg16178855	20	56,269,979	0.000261	-0.209	-0.250				*PMEPA1*	5'UTR
cg13282691	13	112,555,987	0.018841	-0.209	-0.048					
cg18787420	20	30,309,466	8.21E-10	-0.209	-0.218				*BCL2L1*	Body
cg13704497	6	31,590,622	8.35E-06	-0.209	-0.204				*SNORA38*	TSS1500
cg02936263	17	32,596,886	0.032583	-0.209	-0.087				*CCL7*	TSS1500
cg03625515	16	18,157,108	0.035706	-0.209	-0.153					
cg05164406	16	29,830,908	0.000946	-0.209	-0.195				*C16orf53*	Body
cg19676238	1	2,342,094	0.00031	-0.209	-0.229				*PEX10*	Body

cg04049225	16	85,672,175	0.002026	-0.209	-0.152				*KIAA0182*	5'UTR
cg12529432	8	23,538,610	0.009245	-0.209	-0.169				*NKX3-1*	3'UTR
cg19844110	4	9,533,426	9.73E-05	-0.209	-0.197					
cg25532292	6	30,415,782	0.035806	-0.209	-0.134					
cg01719179	16	29,296,693	3.32E-08	-0.209	-0.197					
cg06593447	7	7,832,563	0.006713	-0.209	-0.133					
cg15912390	3	194,826,514	0.015855	-0.209	-0.241				*C3orf21*	Body
cg16594910	11	132,107,419	0.045389	-0.209	-0.129				*NTM*	Body
cg00157656	8	644,663	0.000491	-0.209	-0.148				*ERICH1*	Body
cg18447077	X	77,912,605	0.026828	-0.209	-0.189				*ZCCHC5*	Body
cg16029623	14	58,663,626	0.000114	-0.209	-0.185					
cg05149537	22	45,709,699	0.000144	-0.209	-0.195				*FAM118A*	5'UTR
cg04165004	16	17,299,953	2.2E-08	-0.209	-0.237				*XYLT1*	Body
cg05892146	17	3,337,184	0.01157	-0.209	-0.179				*OR1E2*	TSS200
cg01556466	7	31,146,260	0.032944	-0.209	-0.160				*ADCYAP1R1*	Body
cg02119955	13	102,104,573	0.017159	-0.209	-0.083				*ITGBL1*	TSS1500
cg14210985	12	28,115,804	1.82E-06	-0.209	-0.221				*PTHLH*	Body
cg05058809	16	834,201	5.37E-08	-0.209	-0.146				*MSLNL*	TSS1500
cg05357338	2	364,326	1.32E-05	-0.209	-0.144					
cg06427176	5	178,854,265	0.038701	-0.209	-0.157					
cg11802013	11	69,469,175	2.16E-05	-0.209	-0.204				*CCND1*	3'UTR
cg25471639	4	26,063,077	0.00767	-0.209	-0.123					
cg21245207	9	136,837,406	4.98E-05	-0.209	-0.223				*VAV2*	Body
cg00434535	7	9,022,554	0.041566	-0.209	-0.146					
cg11428427	X	151,563,983	0.035742	-0.209	-0.098				*MIR105-2*	TSS1500
cg15906260	3	186,505,240	4.27E-13	-0.209	-0.206				*SNORA4*	TSS200
cg21534317	2	115,602,681	0.004013	-0.209	-0.167				*DPP10*	Body
cg01777913	12	7,077,090	9.75E-08	-0.209	-0.214				*PHB2*	Body
cg22446491	15	98,961,376	0.031486	-0.209	-0.101					
cg26495526	16	49,407,968	0.048036	-0.209	-0.184				*C16orf78*	1stExon
cg22791045	11	133,403,793	0.014736	-0.209	-0.155				*OPCML*	TSS1500
cg02755847	6	29,232,651	9.98E-06	-0.209	-0.218					
cg17589883	11	1,260,199	0.000123	-0.209	-0.197				*MUC5B*	Body
cg03128219	10	50,366,272	0.000974	-0.209	-0.197				*C10orf128*	Body

cg10057126	4	77,819,792	0.004673	-0.209	-0.178				*ANKRD56*	TSS1500
cg22693863	19	14,118,191	2.29E-06	-0.209	-0.239				*RFX1*	TSS1500
cg23173806	6	30,299,968	7.52E-05	-0.209	-0.210				*TRIM39*	Body
cg05973132	2	240,451,894	0.006527	-0.209	-0.155					
cg07161395	16	51,390,410	0.039726	-0.209	-0.172					
cg10988075	12	115,751,106	0.01926	-0.209	-0.265					
cg20911707	8	3,855,415	0.016887	-0.209	-0.120				*CSMD1*	Body
cg15348679	21	45,626,491	8.9E-09	-0.209	-0.205					
cg24633312	7	43,300,742	0.008876	-0.209	-0.170				*HECW1*	Body
cg09174601	17	13,442,632	0.022869	-0.209	-0.104				*HS3ST3A1*	Body
cg20995127	2	135,808,369	0.00173	-0.209	-0.178				*RAB3GAP1*	TSS1500
cg26029547	6	31,939,085	2.9E-08	-0.209	-0.214				*STK19*	TSS1500
cg08818297	5	44,911,232	0.020018	-0.209	-0.151					
cg06572812	15	27,110,587	0.005624	-0.209	-0.143				*GABRA5*	TSS1500
cg23870181	6	32,713,044	0.008351	-0.209	-0.115			Yes	*HLA-DQA2*	Body
cg26327040	12	131,561,080	0.04375	-0.209	-0.143				*GPR133*	Body
cg01880929	2	36,614,588	1.79E-06	-0.209	-0.209				*CRIM1*	Body
cg08996459	10	125,617,230	0.020815	-0.209	-0.143				*CPXM2*	Body
cg02769535	2	56,574,420	0.010702	-0.209	-0.206				*CCDC85A*	Body
cg02937080	19	23,183,750	0.000146	-0.209	-0.207					
cg17288892	5	178,956,874	7.17E-06	-0.209	-0.217					
cg23066564	22	46,481,734	2.09E-07	-0.209	-0.187				*LOC400931*	TSS200
cg15923139	4	186,801,896	1.57E-05	-0.209	-0.200				*SORBS2*	5'UTR
cg19961980	7	55,063,854	1.12E-09	-0.209	-0.219					
cg26226546	7	103,088,199	2.38E-07	-0.209	-0.189					
cg20015986	4	62,072,253	0.035466	-0.209	-0.180					
cg08238568	1	241,474,238	0.01103	-0.209	-0.235				*RGS7*	Body
cg10358939	3	192,973,502	0.016549	-0.209	-0.137				*HRASLS*	Body
cg10437722	17	35,087,542	0.000127	-0.209	-0.204					
cg23976981	13	22,560,659	0.045028	-0.209	-0.203					
cg08899593	12	132,892,613	0.015751	-0.209	-0.096				*GALNT9*	Body
cg17206001	11	118,219,601	0.004058	-0.209	-0.071				*CD3G*	Body
cg15298173	15	29,903,900	1.62E-05	-0.209	-0.138					
cg02992596	4	155,702,220	0.002978	-0.209	-0.130	3.96E-12	-0.145		*RBM46*	TSS1500

cg20592707	1	152,086,623	0.000157	-0.209	-0.206				*TCHH*	TSS200
cg21176026	4	156,592,010	0.026245	-0.209	-0.338				*GUCY1A3*	5'UTR
cg02232377	7	86,297,084	0.002367	-0.209	-0.177				*GRM3*	5'UTR
cg14746118	X	135,569,630	0.039104	-0.209	-0.158				*BRS3*	TSS1500
cg15695194	11	35,179,802	3.05E-05	-0.209	-0.168				*CD44*	Body
cg01490663	16	87,091,301	0.028276	-0.209	-0.137					
cg16285854	11	85,469,247	6.9E-05	-0.209	-0.194				*SYTL2*	TSS1500
cg02743342	5	126,984,564	3.97E-06	-0.209	-0.224				*CTXN3*	TSS200
cg17667294	19	6,333,761	1.46E-05	-0.209	-0.232				*ACER1*	TSS200
cg12752325	11	2,137,875	0.000132	-0.209	-0.156					
cg22599740	13	76,215,076	1.92E-06	-0.209	-0.184				*LMO7*	Body
cg02363960	7	79,080,535	7.5E-07	-0.209	-0.223				*MAGI2*	Body
cg25994871	7	107,967,614	2.14E-05	-0.210	-0.184				*NRCAM*	5'UTR
cg02876129	14	75,394,075	0.000384	-0.210	-0.235					
cg00179940	12	126,978,053	0.00078	-0.210	-0.242					
cg17316078	12	127,221,700	0.043299	-0.210	-0.151					
cg19628619	1	155,104,050	4.09E-06	-0.210	-0.203				*EFNA1*	Body
cg09640621	10	129,346,391	0.005102	-0.210	-0.137				*NPS*	TSS1500
cg16586227	16	24,359,682	0.038701	-0.210	-0.155				*CACNG3*	Body
cg13986969	9	73,984,868	3.88E-05	-0.210	-0.182					
cg22063369	2	101,504,017	0.007471	-0.210	-0.273				*NPAS2*	5'UTR
cg04603355	19	35,264,987	0.003774	-0.210	-0.168				*ZNF599*	TSS1500
cg09982883	17	33,704,726	0.000105	-0.210	-0.149					
cg13049471	17	74,641,167	0.000222	-0.210	-0.199				*ST6GALNAC1*	TSS1500
cg02050232	16	29,296,614	1.36E-19	-0.210	-0.194					
cg14199629	9	84,202,866	0.001356	-0.210	-0.187				*TLE1*	Body
cg01066189	4	158,493,216	0.024407	-0.210	-0.157				*LOC340017*	TSS1500
cg07956012	1	156,542,701	4.14E-06	-0.210	-0.175				*IQGAP3*	TSS1500
cg20852364	3	30,301,024	0.012151	-0.210	-0.135					
cg07303330	6	132,945,515	0.033467	-0.210	-0.112				*TAAR2*	TSS200
cg07548668	3	151,388,160	0.012247	-0.210	-0.145					
cg03415001	4	181,480,012	0.048527	-0.210	-0.136					
cg05801374	1	24,283,657	2.49E-05	-0.210	-0.208					
cg26422784	11	62,063,821	0.002938	-0.210	-0.173				*SCGB1D4*	3'UTR

cg01829342	14	52,218,954	4.56E-05	-0.210	-0.232					
cg03541338	10	121,301,343	0.036647	-0.210	-0.267				*RGS10*	Body
cg01223204	22	22,863,655	4.79E-07	-0.210	-0.244				*ZNF280B*	TSS200
cg03468967	3	19,673,982	0.002825	-0.210	-0.188					
cg19095714	11	2,645,098	0.002223	-0.210	-0.229				*KCNQ1*	Body
cg03429348	2	233,750,589	1.96E-05	-0.210	-0.210				*NGEF*	Body
cg09588186	3	118,479,934	0.00109	-0.210	-0.239					
cg11237207	1	158,911,377	0.000916	-0.210	-0.155				*PYHIN1*	Body
cg14653988	5	167,171,284	3.56E-06	-0.210	-0.231				*ODZ2*	Body
cg07207043	6	7,051,497	0.013716	-0.210	-0.192					
cg17404981	4	1,240,264	1.01E-08	-0.210	-0.196				*CTBP1*	Body
cg13437554	19	2,272,716	2.36E-17	-0.210	-0.205				*OAZ1*	Body
cg04536957	3	184,487,758	0.003421	-0.210	-0.145					
cg07099640	16	88,292,973	7.76E-16	-0.210	-0.187					
cg18622538	12	34,523,825	0.000132	-0.210	-0.217					
cg20181079	11	59,270,409	0.025746	-0.210	-0.199				*OR4D11*	TSS1500
cg01156081	17	3,214,298	0.023119	-0.210	-0.086				*OR3A4*	Body
cg12752420	1	43,751,363	5.84E-07	-0.210	-0.213				*C1orf210*	TSS200
cg15549435	7	14,192,484	7.2E-05	-0.210	-0.197				*DGKB*	Body
cg23235622	20	34,039,349	0.000285	-0.210	-0.237					
cg24481441	1	29,509,511	1.9E-06	-0.210	-0.204				*SFRS4*	TSS1500
cg05221326	12	126,172,639	0.01417	-0.210	-0.221					
cg11809107	2	3,826,782	5.61E-06	-0.210	-0.241					
cg13917002	9	72,260,688	1.03E-05	-0.210	-0.200				*APBA1*	5'UTR
cg26978976	8	63,164,796	0.042583	-0.210	-0.171				*NKAIN3*	Body
cg15886294	12	131,767,291	0.000404	-0.210	-0.137					
cg12213811	12	122,251,152	4.7E-07	-0.210	-0.200				*SETD1B*	Body
cg20797528	6	14,218,193	0.000472	-0.210	-0.239					
cg11902093	11	1,661,972	0.000822	-0.210	-0.074				*HCCA2*	Body
cg24310126	10	124,514,711	0.00522	-0.210	-0.107				*FLJ46361*	TSS1500
cg08670181	16	48,846,942	0.017331	-0.210	-0.169					
cg14329066	8	73,885,161	0.017726	-0.210	-0.173					
cg10877315	6	141,335,831	0.000294	-0.210	-0.148					
cg17516997	15	53,100,108	1.49E-05	-0.210	-0.179					

cg22039357	1	184,388,675	6.92E-08	-0.210	-0.206				*C1orf21*	5'UTR
cg18893311	14	101,201,611	0.035445	-0.210	-0.135					
cg20993386	17	65,408,367	4.64E-07	-0.210	-0.202				*PITPNC1*	Body
cg17900015	17	38,957,507	0.000774	-0.210	-0.165				*KRT28*	TSS1500
cg14221048	9	89,735,272	0.003285	-0.210	-0.146					
cg16580289	6	90,025,307	2.52E-06	-0.210	-0.207				*GABRR2*	TSS1500
cg16725552	14	26,338,282	0.040171	-0.210	-0.065					
cg12937413	7	158,820,418	0.000372	-0.210	-0.233					
cg23262094	1	209,422,036	5.39E-18	-0.210	-0.202					
cg05231542	2	219,902,901	6.33E-06	-0.210	-0.192				*CCDC108*	Body
cg25014026	8	73,735,989	3.47E-05	-0.210	-0.173				*KCNB2*	Body
cg15717377	2	28,971,087	6E-05	-0.210	-0.203					
cg08500171	6	31,590,674	1.22E-05	-0.210	-0.208				*BAT2*	Body
cg13983692	19	3,808,649	3.12E-07	-0.210	-0.206				*ZFR2*	Body
cg23415373	6	91,086,362	0.000839	-0.210	-0.149					
cg05741638	16	90,084,774	4.64E-06	-0.210	-0.178				*DBNDD1*	Body
cg13043454	2	48,980,605	0.019601	-0.210	-0.173				*LHCGR*	Body
cg10022966	12	34,518,957	0.002073	-0.210	-0.157					
cg16985023	10	133,195,834	0.008901	-0.210	-0.125					
cg18966140	10	15,898,849	0.001993	-0.210	-0.188				*FAM188A*	Body
cg03309455	8	88,845,118	0.024364	-0.210	-0.163					
cg11274778	20	31,781,232	0.000106	-0.210	-0.203				*BASE*	TSS200
cg05383657	12	34,757,822	0.000227	-0.210	-0.215					
cg08918845	20	31,595,432	6.21E-05	-0.210	-0.229				*BPIL1*	5'UTR
cg10000148	14	101,447,095	0.005658	-0.210	-0.204				*SNORD114-21*	TSS1500
cg20668096	4	151,500,192	3.09E-06	-0.210	-0.208				*LRBA*	Body
cg12755471	12	93,096,780	7.05E-06	-0.210	-0.220				*C12orf74*	TSS200
cg00327332	20	23,498,993	0.012235	-0.210	-0.159				*CSTT*	TSS1500
cg04565216	3	171,728,208	0.002063	-0.210	-0.237					
cg07895124	16	57,599,076	0.045088	-0.210	-0.073				*GPR114*	Body
cg10546603	11	49,003,527	0.007183	-0.210	-0.145					
cg14520947	1	225,942,842	4.2E-06	-0.210	-0.235					
cg15983182	7	73,474,232	4.82E-06	-0.210	-0.209				*ELN*	Body
cg19939969	1	209,801,351	3.98E-14	-0.210	-0.202				*LAMB3*	Body

cg23048656	14	91,227,147	0.000286	-0.210	-0.131				*TTC7B*	Body
cg17256192	5	31,323,297	0.046972	-0.210	-0.150				*CDH6*	Body
cg19794481	12	7,073,240	0.000227	-0.210	-0.201				*MIR141*	TSS200
cg04334235	14	106,188,793	0.000321	-0.210	-0.217					
cg10432933	13	19,965,341	0.016625	-0.210	-0.158					
cg26111327	3	195,164,931	0.001992	-0.210	-0.146				*ACAP2*	TSS1500
cg02076020	1	201,979,938	1.47E-05	-0.210	-0.205				*ELF3*	5'UTR
cg16434510	8	129,800,022	0.04088	-0.210	-0.229					
cg01206347	7	49,811,765	0.028539	-0.210	-0.229				*VWC2*	TSS1500
cg18515872	14	72,943,461	1.65E-10	-0.210	-0.211				*RGS6*	Body
cg21165909	2	211,419,938	0.003034	-0.210	-0.192	0.00000304	-0.129		*CPS1*	Body
cg10250660	14	23,876,824	9.92E-06	-0.210	-0.190				*MYH6*	5'UTR
cg13027851	2	102,433,793	5.78E-09	-0.210	-0.171				*MAP4K4*	Body
cg01378062	3	88,961,910	0.040436	-0.210	-0.159					
cg20707786	17	79,058,303	7.9E-13	-0.210	-0.173				*BAIAP2*	Body
cg22077313	4	6,691,093	9.17E-15	-0.210	-0.208					
cg23717593	1	86,617,731	0.005955	-0.210	-0.171				*COL24A1*	Body
cg20884362	21	46,358,702	5.82E-06	-0.210	-0.201	0.01827691	-0.040		*C21orf67*	Body
cg01612003	4	15,355,722	0.032738	-0.210	-0.210				*C1QTNF7*	Body
cg26743132	13	111,107,905	3.44E-07	-0.210	-0.165				*COL4A2*	Body
cg01344243	16	9,855,280	0.007398	-0.210	-0.172				*GRIN2A*	3'UTR
cg08645400	16	49,307,062	0.033606	-0.210	-0.141					
cg18695263	3	196,387,505	0.000646	-0.210	-0.189				*LRRC33*	Body
cg19169535	X	71,699,559	9.74E-09	-0.210	-0.205				*HDAC8*	Body
cg22137572	5	19,915,602	0.000597	-0.210	-0.231				*CDH18*	5'UTR
cg25747783	7	105,603,622	1.68E-06	-0.210	-0.205				*FLJ23834*	TSS200
cg27635859	2	92,263,464	0.011368	-0.210	-0.165					
cg13201362	1	215,567,839	0.009812	-0.210	-0.150					
cg26138073	13	22,979,750	1.05E-05	-0.210	-0.186					
cg08015107	11	8,618,950	2.47E-09	-0.210	-0.199					
cg08623256	5	3,858,275	0.005902	-0.210	-0.112					
cg27095035	17	48,623,891	0.007174	-0.210	-0.197				*SPATA20*	TSS1500
cg26622537	11	101,458,233	0.023582	-0.211	-0.195					
cg07614747	8	2,418,520	0.000163	-0.211	-0.231					

cg14072219	1	242,162,802	0.007593	-0.211	-0.155				*MAP1LC3C*	TSS1500
cg25282652	12	128,364,739	0.011698	-0.211	-0.187					
cg08924619	13	111,160,399	2E-05	-0.211	-0.204				*COL4A2*	Body
cg13745946	14	104,205,299	0.046464	-0.211	-0.106				*PPP1R13B*	Body
cg05954120	1	156,254,757	2.56E-11	-0.211	-0.204				*TMEM79*	Body
cg04826831	16	87,392,001	0.022853	-0.211	-0.060				*FBXO31*	Body
cg16720379	11	33,506,184	0.033004	-0.211	-0.086					
cg05279643	11	62,445,555	3.33E-07	-0.211	-0.190				*UBXN1*	Body
cg10038490	10	124,515,334	0.006392	-0.211	-0.134				*FLJ46361*	TSS1500
cg01861131	16	4,845,708	0.011803	-0.211	-0.186				*LOC440335*	Body
cg01897191	5	167,097,366	0.013045	-0.211	-0.161				*ODZ2*	Body
cg05306183	8	11,144,705	4.41E-06	-0.211	-0.202				*MTMR9*	Body
cg15165950	5	83,720,892	0.030621	-0.211	-0.152					
cg18008487	7	95,157,228	4.12E-08	-0.211	-0.206				*ASB4*	Body
cg24247132	7	64,458,642	2.3E-05	-0.211	-0.187					
cg10759651	1	163,144,950	4.78E-05	-0.211	-0.180				*RGS5*	Body
cg14360405	11	87,912,414	4.32E-05	-0.211	-0.182					
cg15682266	6	134,155,787	0.004772	-0.211	-0.149				*MGC34034*	Body
cg25481160	3	71,111,489	7.16E-12	-0.211	-0.205				*FOXP1*	Body
cg12624721	8	143,511,575	0.005595	-0.211	-0.120					
cg07044276	1	246,859,889	1.38E-06	-0.211	-0.182					
cg10763891	7	93,300,578	0.016279	-0.211	-0.166					
cg22682297	6	57,972,721	0.043623	-0.211	-0.135					
cg02134660	6	54,710,399	0.037287	-0.211	-0.109				*FAM83B*	TSS1500
cg03049917	15	88,752,391	0.014466	-0.211	-0.180				*NTRK3*	Body
cg03146196	20	30,007,925	0.003513	-0.211	-0.140				*DEFB122*	TSS1500
cg08319974	6	164,506,861	0.031101	-0.211	-0.152					
cg08583240	2	102,460,597	0.000491	-0.211	-0.198				*MAP4K4*	Body
cg24167473	3	113,899,263	0.000177	-0.211	-0.193				*DRD3*	TSS1500
cg05686467	11	124,789,005	0.00024	-0.211	-0.205				*HEPN1*	TSS200
cg07007122	8	89,270,978	0.040992	-0.211	-0.174				*MMP16*	Body
cg07014174	21	32,253,760	0.036704	-0.211	-0.144	1.61E-08	-0.184		*KRTAP11-1*	1stExon
cg11420780	11	71,320,513	0.017243	-0.211	-0.108					
cg27569034	1	152,957,018	0.00205	-0.211	-0.143				*SPRR1A*	5'UTR

cg07800498	8	2,244,943	0.002952	-0.211	-0.186					
cg21546821	2	10,975,271	2.58E-07	-0.211	-0.216					
cg00780520	8	128,808,554	0.004629	-0.211	-0.109				*PVT1*	Body
cg14753070	19	14,163,842	0.019633	-0.211	-0.188				*IL27RA*	3'UTR
cg06393098	19	46,235,602	1.6E-06	-0.211	-0.180				*FBXO46*	TSS1500
cg10077899	2	25,818,974	0.001219	-0.211	-0.229				*DTNB*	Body
cg09763373	16	596,899	0.008515	-0.211	-0.111				*SOLH*	Body
cg07912020	2	2,713,385	0.014094	-0.211	-0.174					
cg10520226	1	2,811,554	6.64E-05	-0.211	-0.153					
cg03238075	17	21,113,363	0.00069	-0.211	-0.202				*TMEM11*	Body
cg00716660	1	224,543,513	1.83E-06	-0.211	-0.190				*CNIH4*	TSS1500
cg01476885	17	21,908,498	0.015447	-0.211	-0.165				*FLJ36000*	Body
cg00564163	7	87,935,979	0.0299	-0.211	-0.244	0.00441312	-0.041		*STEAP4*	5'UTR
cg17085280	18	31,018,457	0.014206	-0.211	-0.147				*C18orf34*	5'UTR
cg04921771	1	40,349,687	0.004097	-0.211	-0.141				*TRIT1*	TSS1500
cg10423328	X	84,364,131	0.032414	-0.211	-0.182				*SATL1*	TSS200
cg00947896	16	10,349,228	0.011317	-0.211	-0.104					
cg18027831	1	227,083,489	0.000265	-0.211	-0.240				*PSEN2*	3'UTR
cg03062717	19	35,940,483	2.24E-07	-0.211	-0.202				*FFAR2*	TSS200
cg04317927	11	55,418,816	0.000203	-0.211	-0.240				*OR4S2*	1stExon
cg04348305	20	25,230,959	0.000268	-0.211	-0.188				*PYGB*	Body
cg08212545	16	31,584,046	7.91E-06	-0.211	-0.169					
cg14776195	20	29,956,028	0.019089	-0.211	-0.153				*DEFB118*	TSS1500
cg26973989	4	190,949,036	0.033966	-0.211	-0.124				*FRG2*	TSS1500
cg04050782	4	47,707,064	0.003697	-0.211	-0.157				*CORIN*	Body
cg04036366	17	79,976,900	0.004262	-0.211	-0.181				*STRA13*	3'UTR
cg14518227	1	196,312,876	3.89E-08	-0.211	-0.200				*KCNT2*	Body
cg17004025	19	840,795	1.63E-08	-0.211	-0.192				*PRTN3*	TSS200
cg18884942	1	161,679,921	0.002946	-0.211	-0.169				*FCRLA*	Body
cg07651080	3	77,145,918	0.005642	-0.211	-0.158				*ROBO2*	TSS1500
cg04062040	2	103,343,421	3.71E-05	-0.211	-0.205				*MFSD9*	Body
cg25598203	10	53,870,395	0.02067	-0.211	-0.066				*PRKG1*	Body
cg21212505	7	117,228,958	1.57E-07	-0.211	-0.203				*CFTR*	Body
cg22029284	1	3,077,356	0.000593	-0.211	-0.111				*PRDM16*	Body

cg26088269	11	131,983,984	0.003612	-0.211	-0.177					*NTM*	Body
cg05070372	16	87,027,489	0.002407	-0.211	-0.127						
cg06639544	19	14,940,319	0.014488	-0.211	-0.108					*OR7A5*	TSS1500
cg12017221	12	22,968,563	0.016522	-0.211	-0.145						
cg12400457	1	115,919,812	9.8E-07	-0.211	-0.207						
cg02283204	6	40,570,601	0.000144	-0.211	-0.177						
cg00576736	1	20,030,383	0.000124	-0.211	-0.208					*TMCO4*	Body
cg07844563	1	28,423,139	7.31E-06	-0.211	-0.202					*LOC653566*	TSS200
cg14877963	3	108,854,657	0.011651	-0.211	-0.142						
cg20832320	3	49,712,339	0.000124	-0.211	-0.147					*APEH*	Body
cg01514353	22	46,259,643	4.25E-10	-0.211	-0.175						
cg06198390	1	152,648,726	0.018305	-0.211	-0.142					*LCE2C*	Body
cg24328844	12	26,122,271	5.57E-05	-0.211	-0.219					*RASSF8*	5'UTR
cg24870391	17	32,612,675	4.19E-06	-0.211	-0.209	2.24E-16	-0.136			*CCL11*	TSS200
cg01921323	7	153,881,192	9.75E-05	-0.211	-0.223					*DPP6*	Body
cg18185987	14	88,477,518	6.13E-08	-0.211	-0.199					*GPR65*	Body
cg25233308	11	66,192,121	0.002298	-0.211	-0.122					*NPAS4*	Body
cg03034101	4	166,631,112	0.00024	-0.211	-0.213						
cg12034871	14	92,525,005	1.25E-05	-0.211	-0.243					*ATXN3*	Body
cg12593764	15	83,332,873	0.014619	-0.211	-0.105					*AP3B2*	Body
cg05911990	7	112,063,449	1.51E-09	-0.211	-0.204					*IFRD1*	1stExon
cg00357546	10	135,032,154	0.044736	-0.211	-0.062					*KNDC1*	Body
cg12569880	18	11,151,764	0.004014	-0.211	-0.156						
cg25292309	2	58,989,427	1.12E-05	-0.211	-0.180						
cg03039990	14	101,317,622	0.000995	-0.211	-0.183					*MIR770*	TSS1500
cg09215582	4	71,575,507	0.000202	-0.211	-0.212					*RUFY3*	Body
cg00473045	8	120,779,899	0.000132	-0.211	-0.224					*TAF2*	Body
cg21838013	11	122,708,207	0.02434	-0.211	-0.160					*CRTAM*	TSS1500
cg01633359	5	33,439,359	0.000435	-0.211	-0.216						
cg04208853	5	5,890,802	0.020768	-0.211	-0.180						
cg22688815	8	9,775,751	0.005522	-0.211	-0.200						
cg05778559	3	16,646,375	0.022837	-0.211	-0.099					*DAZL*	Body
cg14255655	2	137,891,808	0.011972	-0.211	-0.160					*THSD7B*	Body
cg00018866	10	50,343,286	0.019658	-0.211	-0.082					*FAM170B*	TSS1500

cg00210127	16	12,352,233	0.015634	-0.211	-0.177				*SNX29*	Body
cg00494183	13	21,645,350	9.03E-07	-0.211	-0.180					
cg24336547	20	1,539,702	0.005438	-0.211	-0.210				*SIRPD*	TSS1500
cg26808478	1	6,760,926	2.39E-06	-0.211	-0.249				*DNAJC11*	Body
cg21822905	1	2,341,337	0.026441	-0.211	-0.293				*PEX10*	Body
cg05025046	2	119,013,708	0.00184	-0.211	-0.209					
cg10358212	16	81,069,005	0.000462	-0.212	-0.193				*ATMIN*	TSS1500
cg27078562	4	175,838,768	0.003254	-0.212	-0.149				*ADAM29*	TSS1500
cg18991321	7	100,144,475	0.000149	-0.212	-0.162				*AGFG2*	Body
cg22553301	19	3,839,231	0.000448	-0.212	-0.176				*ZFR2*	Body
cg05886537	11	132,936,428	0.047638	-0.212	-0.126			Yes	*OPCML*	Body
cg08168844	16	29,830,973	4.62E-05	-0.212	-0.183				*C16orf53*	Body
cg18936502	7	35,187,811	6.57E-08	-0.212	-0.213					
cg19861312	8	143,214,422	0.003005	-0.212	-0.121					
cg02309655	19	2,588,629	0.000367	-0.212	-0.238				*GNG7*	5'UTR
cg20905413	3	36,375,125	0.012039	-0.212	-0.161					
cg04767756	16	10,652,706	5.06E-13	-0.212	-0.204				*EMP2*	5'UTR
cg05885484	5	122,847,316	2.97E-05	-0.212	-0.199				*CSNK1G3*	TSS1500
cg18926420	15	27,035,113	9.73E-05	-0.212	-0.159					
cg15191887	5	664,666	0.031107	-0.212	-0.152				*TPPP*	3'UTR
cg22427279	1	116,384,534	0.000765	-0.212	-0.198	0.0000175	-0.096	Yes	*NHLH2*	TSS1500
cg27356115	15	53,422,311	4.34E-11	-0.212	-0.197					
cg24414363	22	42,336,273	3.02E-06	-0.212	-0.189				*CENPM*	TSS200
cg10309454	7	158,775,112	1.08E-05	-0.212	-0.183					
cg09305680	8	117,778,069	7.45E-06	-0.212	-0.195				*UTP23*	TSS1500
cg08166767	1	152,671,348	0.000668	-0.212	-0.196				*LCE2A*	5'UTR
cg23313644	14	82,296,090	0.002272	-0.212	-0.207					
cg04703950	10	104,211,424	4.31E-06	-0.212	-0.194				*C10orf95*	TSS200
cg16548605	1	169,453,550	1.78E-06	-0.212	-0.128				*SLC19A2*	Body
cg00949424	5	4,144,830	0.03354	-0.212	-0.098					
cg18614510	22	47,081,903	2.01E-05	-0.212	-0.152				*CERK*	3'UTR
cg24533864	22	22,307,684	3.43E-05	-0.212	-0.140				*PPM1F*	TSS1500
cg03952578	15	61,509,038	6.72E-05	-0.212	-0.206				*RORA*	Body
cg10531104	6	169,235,025	7.29E-07	-0.212	-0.184					

cg08005208	1	163,395,211	0.009801	-0.212	-0.150					
cg23008136	2	55,743,936	0.001209	-0.212	-0.180					
cg18022607	3	61,924,802	7.46E-05	-0.212	-0.223				*PTPRG*	Body
cg10999670	16	86,870,144	0.013208	-0.212	-0.105					
cg15314588	7	129,470,730	0.000364	-0.212	-0.204					
cg23611710	12	132,152,645	0.024938	-0.212	-0.058					
cg24183678	4	16,919,935	0.001873	-0.212	-0.128					
cg26010756	6	7,731,069	0.003632	-0.212	-0.201				*BMP6*	Body
cg20695203	13	112,335,939	0.006449	-0.212	-0.089					
cg02777447	6	47,452,990	1.98E-06	-0.212	-0.235				*CD2AP*	Body
cg02343275	1	212,589,072	1.79E-05	-0.212	-0.181				*TMEM206*	TSS1500
cg11061946	22	43,407,460	0.045422	-0.212	-0.147					
cg14813258	11	27,935,243	0.034554	-0.212	-0.149					
cg19290738	19	2,152,184	3.58E-13	-0.212	-0.209				*AP3D1*	TSS1500
cg26274995	8	11,302,983	1.74E-05	-0.212	-0.177				*FAM167A*	5'UTR
cg02491003	3	57,581,393	7.47E-09	-0.212	-0.195				*ARF4*	Body
cg03401963	2	125,755,746	0.005509	-0.212	-0.200					
cg11042746	11	57,167,405	0.003072	-0.212	-0.175					
cg00758374	3	111,804,343	0.001885	-0.212	-0.164				*C3orf52*	TSS1500
cg00115458	6	33,141,305	0.007225	-0.212	-0.088				*COL11A2*	Body
cg10443159	7	7,839,258	1.48E-05	-0.212	-0.189					
cg27019122	12	34,538,786	0.000257	-0.212	-0.207					
cg06235145	4	17,034,835	0.000547	-0.212	-0.168					
cg26555733	16	23,846,279	0.00904	-0.212	-0.178				*PRKCB*	TSS1500
cg00602655	10	35,979,870	0.000283	-0.212	-0.214					
cg01662869	16	4,730,410	3.71E-08	-0.212	-0.211				*MGRN1*	Body
cg27069616	7	49,076,868	0.026068	-0.212	-0.211					
cg17851657	7	99,680,296	7.1E-07	-0.212	-0.222				*ZNF3*	TSS1500
cg26321840	8	49,241,685	0.001832	-0.212	-0.159					
cg20188621	10	105,127,192	4.5E-10	-0.212	-0.208				*TAF5*	TSS1500
cg27631593	1	153,065,613	0.023324	-0.212	-0.183				*SPRR2E*	3'UTR
cg00527514	11	132,002,516	0.030882	-0.212	-0.222				*NTM*	Body
cg02723533	11	69,468,991	0.001633	-0.212	-0.202	0.00013485	-0.063		*CCND1*	3'UTR
cg07506081	17	77,952,290	0.021179	-0.212	-0.205				*TBC1D16*	Body

cg10983206	10	7,564,401	0.000683	-0.212	-0.208					
cg01374885	X	146,319,567	2.09E-06	-0.212	-0.210				*MIR508*	TSS1500
cg09312149	5	114,516,420	7.73E-11	-0.212	-0.191	1.33E-07	-0.079		*TRIM36*	TSS200
cg13900542	1	74,888,995	0.03579	-0.212	-0.123				*TNNI3K*	Body
cg01717194	13	39,543,257	0.030009	-0.212	-0.158				*STOML3*	Body
cg21850934	11	90,451,108	0.017245	-0.212	-0.143					
cg20363199	10	108,335,668	0.001573	-0.212	-0.207				*SORCS1*	3'UTR
cg02279503	1	30,374,065	0.001592	-0.212	-0.121					
cg10954765	4	3,412,750	5.57E-06	-0.212	-0.223				*RGS12*	Body
cg19250790	19	2,614,086	1.41E-08	-0.212	-0.187				*GNG7*	5'UTR
cg02263260	16	82,658,496	0.024339	-0.212	-0.213					
cg05201300	5	172,443,740	5.18E-09	-0.212	-0.207				*ATP6V0E1*	Body
cg15983005	17	39,506,938	0.000917	-0.212	-0.120	1.91E-09	-0.135		*KRT33A*	1stExon
cg22608265	1	3,351,106	0.025048	-0.212	-0.191				*PRDM16*	3'UTR
cg16057408	14	101,196,998	0.011087	-0.212	-0.164				*DLK1*	Body
cg12315311	19	54,568,443	0.00372	-0.212	-0.244				*VSTM1*	TSS1500
cg05979170	3	131,974,369	1.95E-10	-0.212	-0.220					
cg19533977	17	57,719,682	2.01E-07	-0.212	-0.206				*CLTC*	Body
cg23211310	10	50,120,250	0.042714	-0.212	-0.123				*LRRC18*	Body
cg02487491	8	4,183,726	0.019127	-0.212	-0.196				*CSMD1*	Body
cg15081844	3	133,117,569	0.002825	-0.212	-0.173				*BFSP2*	TSS1500
cg19015997	7	12,611,607	0.038376	-0.212	-0.173				*SCIN*	Body
cg06294766	4	154,680,314	0.040854	-0.212	-0.188				*RNF175*	Body
cg07918509	17	62,083,407	8.3E-06	-0.212	-0.212				*ICAM2*	Body
cg16325421	20	60,470,081	0.038712	-0.212	-0.103				*CDH4*	Body
cg18115132	1	107,819,805	0.022095	-0.212	-0.124				*NTNG1*	Body
cg06484232	11	5,905,350	7.45E-05	-0.212	-0.198				*OR52E4*	TSS200
cg03171710	8	133,140,125	0.036106	-0.212	-0.164					
cg07224291	6	133,777,802	0.003885	-0.212	-0.219				*EYA4*	Body
cg19228683	11	91,963,266	0.000265	-0.212	-0.160					
cg02709052	11	86,748,071	1.29E-05	-0.212	-0.189				*TMEM135*	TSS1500
cg15649671	1	157,895,227	0.04046	-0.212	-0.109					
cg17108958	1	221,129,545	0.006109	-0.212	-0.238					
cg23822732	2	2,692,854	0.013652	-0.212	-0.191					

cg09334699	2	2,193,511	0.002252	-0.212	-0.127				*MYT1L*	5'UTR
cg00670721	3	186,504,202	5.37E-10	-0.212	-0.200				*SNORA63*	TSS1500
cg23228019	20	46,579,870	0.00067	-0.212	-0.147					
cg07561132	20	18,794,975	0.001503	-0.212	-0.164				*C20orf79*	1stExon
cg11865119	7	130,125,932	0.000799	-0.212	-0.240				*MEST*	TSS200
cg11870021	17	21,913,158	0.014049	-0.212	-0.185					
cg08343600	10	43,894,095	9.83E-06	-0.212	-0.205				*HNRNPF*	5'UTR
cg11064170	2	178,080,435	6.12E-05	-0.212	-0.158				*HNRNPA3*	Body
cg17233896	12	127,227,116	0.03184	-0.212	-0.182					
cg26620747	16	27,523,214	0.000238	-0.212	-0.228				*GTF3C1*	Body
cg07960922	7	83,461,505	3.06E-06	-0.212	-0.174					
cg15209376	13	102,104,976	0.001985	-0.212	-0.149				*ITGBL1*	1stExon
cg23898213	X	62,979,075	0.005829	-0.212	-0.160					
cg01080552	X	2,986,093	1.13E-06	-0.212	-0.207				*ARSF*	5'UTR
cg07884251	15	89,398,614	0.008336	-0.212	-0.107				*ACAN*	Body
cg10013324	16	6,335,194	0.002746	-0.212	-0.220				*A2BP1*	5'UTR
cg14577716	3	95,402,225	0.005904	-0.212	-0.122					
cg09614301	16	1,090,228	0.026424	-0.212	-0.113					
cg10158151	17	51,900,032	0.015271	-0.213	-0.150				*KIF2B*	TSS1500
cg21408061	1	155,989,796	4.07E-09	-0.213	-0.193				*SSR2*	Body
cg08155584	3	82,314,701	0.020429	-0.213	-0.155					
cg11629955	3	126,679,199	0.000274	-0.213	-0.241				*CHCHD6*	3'UTR
cg18890249	10	51,361,773	4.15E-05	-0.213	-0.176				*LOC728407*	Body
cg23066675	3	118,676,357	0.000128	-0.213	-0.211				*IGSF11*	Body
cg17372047	19	22,807,010	0.006109	-0.213	-0.249					
cg20545694	8	48,910,778	1.37E-05	-0.213	-0.151					
cg00415389	7	138,162,679	3.34E-05	-0.213	-0.192			Yes	*TRIM24*	Body
cg17218342	21	43,918,422	6.09E-09	-0.213	-0.219				*SLC37A1*	TSS1500
cg03888764	7	57,207,547	4.63E-07	-0.213	-0.196				*ZNF479*	5'UTR
cg15769756	13	107,489,008	0.00177	-0.213	-0.162					
cg24320225	8	2,238,305	0.000209	-0.213	-0.202					
cg09211256	12	38,552,636	0.000194	-0.213	-0.181					
cg10625488	2	203,733,187	3.86E-05	-0.213	-0.245				*ICA1L*	5'UTR
cg15389025	12	49,627,934	9.23E-05	-0.213	-0.166					

cg21263710	18	49,865,488	0.000222	-0.213	-0.159				*DCC*	TSS1500
cg00045762	5	167,181,762	0.039079	-0.213	-0.179				*ODZ2*	Body
cg14833337	1	157,490,546	0.024985	-0.213	-0.163				*FCRL5*	Body
cg23311800	11	34,535,396	5.74E-05	-0.213	-0.210				*ELF5*	TSS200
cg25765192	1	13,184,954	0.019021	-0.213	-0.116				*LOC440563*	TSS1500
cg18984002	17	36,876,514	0.007438	-0.213	-0.144				*MLLT6*	Body
cg21926883	2	100,939,477	1.37E-06	-0.213	-0.210				*LONRF2*	TSS1500
cg27473997	4	9,355,351	0.001361	-0.213	-0.137				*USP17*	TSS200
cg24707219	6	31,847,009	2.86E-12	-0.213	-0.216				*SLC44A4*	TSS200
cg14851608	1	22,777,266	8.97E-06	-0.213	-0.180				*ZBTB40*	TSS1500
cg18649198	7	151,477,667	5.84E-06	-0.213	-0.230				*PRKAG2*	Body
cg03617867	6	155,053,338	8.33E-05	-0.213	-0.211				*RBM16*	TSS1500
cg23304784	8	2,179,815	0.014891	-0.213	-0.071					
cg09814128	4	178,243,783	0.002704	-0.213	-0.199				*NEIL3*	Body
cg03142975	9	125,026,202	7.43E-20	-0.213	-0.221				*MRRF*	TSS1500
cg04846432	3	186,965,021	0.014357	-0.213	-0.195				*MASP1*	3'UTR
cg15380358	20	61,937,737	0.003989	-0.213	-0.185				*COL20A1*	Body
cg22537167	12	178,510	8.85E-11	-0.213	-0.201				*IQSEC3*	Body
cg27236146	3	191,851,266	0.048943	-0.213	-0.161					
cg08084390	11	55,044,082	0.007723	-0.213	-0.169					
cg19442493	17	77,375,519	1.69E-08	-0.213	-0.191				*HRNBP3*	5'UTR
cg26950369	2	167,816,565	0.015176	-0.213	-0.190				*XIRP2*	Body
cg23350812	2	236,415,734	0.010477	-0.213	-0.194				*AGAP1*	Body
cg21813377	1	4,192,817	0.004105	-0.213	-0.120					
cg07551952	7	12,613,522	0.00114	-0.213	-0.196				*SCIN*	Body
cg02291365	13	25,317,267	0.001861	-0.213	-0.120					
cg03729603	16	24,269,804	0.029017	-0.213	-0.133				*CACNG3*	Body
cg04994795	1	8,404,050	3.93E-06	-0.213	-0.176				*SLC45A1*	Body
cg15541062	11	5,531,199	0.00716	-0.213	-0.234				*UBQLN3*	TSS200
cg18009093	5	177,853,574	4.59E-05	-0.213	-0.208				*COL23A1*	Body
cg21663916	8	122,863,171	0.00067	-0.213	-0.174					
cg09571345	10	26,931,926	2.66E-10	-0.213	-0.203				*LOC731789*	TSS200
cg14414873	9	112,970,463	7.68E-06	-0.213	-0.206				*C9orf152*	TSS200
cg19050940	13	76,445,108	6.92E-12	-0.213	-0.198					

cg19785773	8	142,398,412	9.02E-14	-0.213	-0.214						
cg12744694	8	89,300,240	0.03773	-0.213	-0.181					*MMP16*	Body
cg16659026	19	54,617,827	0.015973	-0.213	-0.225					*PRPF31*	TSS1500
cg19556854	6	32,945,549	8.62E-06	-0.213	-0.249					*BRD2*	Body
cg26063643	22	16,998,752	0.004393	-0.213	-0.126						
cg00253971	19	4,038,851	3.36E-11	-0.213	-0.233						
cg04595200	12	34,486,507	0.000431	-0.213	-0.207						
cg20810675	4	171,604,188	0.047953	-0.213	-0.208						
cg21611093	3	134,512,956	0.000632	-0.213	-0.196					*EPHB1*	TSS1500
cg19300923	1	14,026,137	2.17E-07	-0.213	-0.240					*PRDM2*	TSS1500
cg18377376	11	131,707,728	0.035692	-0.213	-0.282					*NTM*	Body
cg11188440	11	45,667,273	0.001067	-0.213	-0.153						
cg00767201	19	22,141,063	7.75E-06	-0.213	-0.210						
cg02686091	19	43,673,501	0.000832	-0.213	-0.155					*PSG5*	3'UTR
cg09479341	17	40,119,957	7.96E-07	-0.213	-0.226					*CNP*	Body
cg10044209	14	88,122,084	0.00248	-0.213	-0.137						
cg10079131	11	688,397	0.026634	-0.213	-0.126					*DEAF1*	Body
cg03473127	19	51,411,782	6.12E-10	-0.213	-0.217					*KLK4*	Body
cg18191092	8	64,331,042	9.55E-06	-0.213	-0.209						
cg05195318	5	156,722,426	3.65E-05	-0.213	-0.198				Yes	*CYFIP2*	Body
cg05693204	5	1,860,135	6.41E-05	-0.213	-0.162						
cg02480320	5	2,135,054	0.000184	-0.213	-0.222						
cg09043941	10	6,297,748	0.001079	-0.213	-0.207						
cg04646186	15	27,215,757	0.000106	-0.213	-0.153					*GABRG3*	TSS1500
cg01939535	5	55,010,789	1.27E-07	-0.213	-0.194						
cg03410269	12	7,077,969	1.36E-05	-0.213	-0.199					*PHB2*	Body
cg10755723	3	192,576,964	0.026112	-0.213	-0.125					*C3orf59*	Body
cg26875832	7	54,346,209	0.000796	-0.213	-0.218						
cg08145178	X	103,294,452	0.015486	-0.213	-0.169					*MIR1256*	Body
cg06143663	16	11,242,115	9.53E-06	-0.213	-0.199					*CLEC16A*	Body
cg07467958	4	190,597,742	0.004215	-0.213	-0.185						
cg00249974	1	156,254,149	3.42E-05	-0.213	-0.206					*TMEM79*	1stExon
cg25553522	4	71,388,589	3.06E-05	-0.213	-0.202					*AMTN*	Body
cg15579926	2	207,511,246	0.005258	-0.213	-0.099					*LOC200726*	Body

cg24174959	7	34,528,600	0.029261	-0.213	-0.212					
cg05122213	13	114,062,198	1.07E-08	-0.213	-0.178					
cg17753169	8	41,523,177	0.003066	-0.213	-0.109			Yes	*ANK1*	TSS1500
cg17367832	2	241,395,288	0.000724	-0.213	-0.204				*MIR149*	TSS200
cg26839288	10	44,287,132	0.02767	-0.213	-0.169				*HNRNPA3P1*	TSS1500
cg19747968	11	131,940,559	4.05E-05	-0.213	-0.228				*NTM*	Body
cg07859753	13	96,300,257	0.000349	-0.213	-0.210					
cg15363455	7	1,650,412	0.016711	-0.213	-0.065					
cg19647474	6	159,736,729	0.000968	-0.213	-0.169					
cg08363001	10	10,945,277	0.021796	-0.213	-0.207					
cg19837298	7	5,348,995	0.000269	-0.213	-0.147				*TNRC18*	Body
cg27303733	11	8,007,457	1.3E-05	-0.213	-0.173				*EIF3F*	TSS1500
cg15629868	1	8,319,945	1.25E-07	-0.213	-0.208					
cg02416536	12	98,577,192	0.042676	-0.213	-0.320					
cg08316234	17	51,899,386	0.001124	-0.213	-0.140				*KIF2B*	TSS1500
cg01678292	8	41,522,873	5.25E-08	-0.213	-0.205				*ANK1*	TSS200
cg16907101	13	112,747,087	0.046913	-0.213	-0.113					
cg19765351	6	49,986,643	0.033905	-0.213	-0.169				*DEFB110*	Body
cg25593631	1	9,687,880	0.000595	-0.213	-0.131					
cg02815102	20	60,388,903	0.014581	-0.213	-0.160			Yes	*CDH4*	Body
cg03526920	14	77,291,706	0.001653	-0.213	-0.157				*C14orf166B*	TSS1500
cg23029159	14	101,524,635	0.026116	-0.214	-0.226				*MIR154*	TSS1500
cg15916628	19	56,092,834	5.75E-07	-0.214	-0.203	1.94E-09	-0.079		*ZNF579*	TSS1500
cg02711212	15	69,854,024	2.32E-05	-0.214	-0.213				*LOC145837*	TSS200
cg20575856	10	50,428,882	0.047057	-0.214	-0.086					
cg25628429	11	50,005,457	0.011468	-0.214	-0.187				*OR4C12*	TSS1500
cg26899012	5	86,042,549	0.000326	-0.214	-0.197					
cg01004918	1	2,731,370	3.63E-06	-0.214	-0.170					
cg13376438	7	108,168,711	0.043082	-0.214	-0.067					
cg20546259	2	49,271,470	0.01537	-0.214	-0.242				*FSHR*	Body
cg25247520	8	128,808,017	2.94E-05	-0.214	-0.189				*MIR1204*	TSS200
cg07353266	11	120,387,832	0.000218	-0.214	-0.264					
cg09040942	7	158,060,003	0.000222	-0.214	-0.214				*PTPRN2*	Body
cg11267879	19	41,619,952	0.045821	-0.214	-0.117				*CYP2F1*	TSS1500

cg00162104	4	2,926,894	6.56E-05	-0.214	-0.240				*ADD1*	Body
cg00522231	2	9,549,277	6.73E-05	-0.214	-0.214				*ITGB1BP1*	Body
cg19277516	1	94,235,220	0.00404	-0.214	-0.157					
cg20466895	7	83,319,354	9.62E-05	-0.214	-0.198					
cg17136696	1	2,823,861	0.031441	-0.214	-0.094					
cg03138282	13	39,187,482	0.009814	-0.214	-0.158					
cg16706613	12	52,423,671	2.28E-06	-0.214	-0.218					
cg19201723	20	7,921,280	0.00078	-0.214	-0.194				*HAO1*	TSS200
cg17035844	1	171,640,211	0.000333	-0.214	-0.217					
cg14449317	7	62,198,343	0.015678	-0.214	-0.135					
cg03265969	7	54,617,631	0.02796	-0.214	-0.200				*VSTM2A*	Body
cg04365699	3	177,320,997	2.65E-05	-0.214	-0.205					
cg08319312	12	34,500,811	0.038351	-0.214	-0.097					
cg14541582	5	601,475	0.040123	-0.214	-0.205			Yes		
cg17034342	12	132,870,388	7.81E-05	-0.214	-0.121				*GALNT9*	Body
cg04164640	2	162,137,680	9.5E-14	-0.214	-0.168					
cg04765207	X	151,883,250	0.026732	-0.214	-0.160				*MAGEA2*	5'UTR
cg08523756	11	41,165,407	0.008612	-0.214	-0.168					
cg11834658	1	21,611,224	3.7E-07	-0.214	-0.211				*ECE1*	Body
cg11343506	11	34,535,568	0.048272	-0.214	-0.273				*ELF5*	TSS1500
cg12758147	6	30,103,696	0.008132	-0.214	-0.210				*TRIM40*	TSS1500
cg21767657	8	47,751,623	0.000938	-0.214	-0.216				*BEYLA*	TSS1500
cg26329274	5	156,607,853	0.00792	-0.214	-0.169				*ITK*	TSS200
cg03503758	19	2,614,104	1.19E-07	-0.214	-0.181			Yes	*GNG7*	5'UTR
cg04854917	10	131,378,330	7.77E-09	-0.214	-0.203				*MGMT*	Body
cg05878332	11	109,302,849	8.31E-05	-0.214	-0.187					
cg16933248	12	132,932,245	0.026847	-0.214	-0.104					
cg18720533	10	134,833,372	0.014776	-0.214	-0.096					
cg26389913	12	97,631,769	0.000276	-0.214	-0.280					
cg02398007	12	67,616,546	0.02788	-0.214	-0.109					
cg07869023	20	17,212,376	0.046058	-0.214	-0.235				*PCSK2*	Body
cg22611523	10	10,630,114	0.028851	-0.214	-0.234					
cg07294114	3	63,047,382	4.54E-12	-0.214	-0.221					
cg11806305	5	139,782,703	8.04E-19	-0.214	-0.190				*ANKHD1*	Body

cg02689785	10	105,004,949	3.12E-05	-0.214	-0.163				*LOC729020*	TSS1500
cg04655834	15	25,031,239	1.86E-08	-0.214	-0.193					
cg16732341	4	112,130,812	0.019928	-0.214	-0.174					
cg19304088	4	111,547,790	0.012765	-0.214	-0.149				*PITX2*	Body
cg09170397	1	181,480,632	0.01492	-0.214	-0.245				*CACNA1E*	Body
cg21649629	8	66,933,425	0.000154	-0.214	-0.232				*DNAJC5B*	TSS1500
cg20985521	1	55,168,830	0.000233	-0.214	-0.153				*C1orf175*	Body
cg05030450	5	167,480,853	0.032664	-0.214	-0.169				*ODZ2*	Body
cg22455963	X	132,095,750	7.1E-05	-0.214	-0.249				*HS6ST2*	TSS1500
cg10015500	7	56,241,697	0.004067	-0.214	-0.193					
cg16697850	7	141,946,928	2.82E-06	-0.214	-0.235				*MOXD2*	TSS200
cg26080567	21	37,536,137	1.6E-06	-0.214	-0.204				*DOPEY2*	TSS1500
cg26137103	4	40,193,114	0.000173	-0.214	-0.201					
cg24522489	20	30,553,236	0.024611	-0.214	-0.083					
cg02876221	4	134,545,513	0.000184	-0.214	-0.204					
cg22831029	7	83,325,268	0.000382	-0.214	-0.254					
cg00397871	7	31,692,686	0.02328	-0.214	-0.120				*CCDC129*	3'UTR
cg11241549	6	56,114,591	0.002111	-0.214	-0.158					
cg09905416	11	59,861,219	0.000676	-0.214	-0.214				*MS4A2*	Body
cg10257070	10	125,399,468	0.021762	-0.214	-0.199					
cg19153300	11	134,794,980	0.029114	-0.214	-0.116					
cg05810129	10	14,597,004	0.001049	-0.214	-0.151				*FAM107B*	Body
cg11025037	14	25,104,483	7.96E-08	-0.214	-0.207				*GZMB*	TSS1500
cg08439276	2	68,937,224	0.000168	-0.214	-0.188					
cg16747973	4	155,533,784	0.007751	-0.214	-0.152				*FGG*	5'UTR
cg19911566	17	28,164,614	8.37E-05	-0.214	-0.179				*SSH2*	Body
cg21588838	3	190,152,481	0.003473	-0.214	-0.175				*TMEM207*	Body
cg18030265	6	32,975,083	0.000913	-0.214	-0.163				*HLA-DOA*	Body
cg08271622	20	37,053,969	2.83E-12	-0.214	-0.181				*LOC388796*	Body
cg27026405	4	4,224,879	1.31E-06	-0.214	-0.183				*OTOP1*	Body
cg19350375	10	98,624,529	9.95E-05	-0.214	-0.219				*LCOR*	5'UTR
cg24311643	1	159,748,210	0.000396	-0.214	-0.118					
cg03419014	21	38,362,742	4.37E-15	-0.214	-0.224				*HLCS*	TSS1500
cg04067806	10	50,974,397	0.003122	-0.214	-0.153					

cg07012823	6	155,777,070	1.24E-05	-0.214	-0.196				*NOX3*	TSS200
cg21615663	16	19,727,911	4.77E-06	-0.214	-0.230	1.74E-08	-0.090		*IQCK*	5'UTR
cg05816993	11	59,709,403	0.014432	-0.214	-0.201					
cg06109939	11	33,637,874	0.027977	-0.214	-0.148				*C11orf41*	Body
cg00305947	18	13,822,384	9.78E-06	-0.214	-0.195					
cg00111826	5	7,983,865	0.016954	-0.214	-0.156					
cg03467728	1	46,220,388	0.005191	-0.214	-0.148					
cg03877014	4	142,197,646	5.72E-10	-0.214	-0.219					
cg10300464	11	15,012,924	0.008239	-0.214	-0.125					
cg06752698	7	98,366,427	0.008258	-0.214	-0.147					
cg07568203	11	5,365,377	0.02387	-0.214	-0.170				*OR51B5*	TSS1500
cg09650495	7	32,463,798	0.000174	-0.214	-0.166					
cg13691599	9	74,863,413	0.014641	-0.214	-0.173				*GDA*	3'UTR
cg18289557	13	113,426,705	1.91E-05	-0.214	-0.185				*ATP11A*	Body
cg03688324	13	22,251,017	0.021763	-0.214	-0.188				*FGF9*	Body
cg22540067	2	125,305,001	0.008501	-0.214	-0.257				*CNTNAP5*	Body
cg25853612	10	19,540,759	0.000513	-0.214	-0.197					
cg18571451	4	16,507,312	0.019185	-0.214	-0.209				*LDB2*	Body
cg23336810	21	43,936,497	0.000144	-0.214	-0.199				*SLC37A1*	5'UTR
cg25666210	12	71,148,346	1.08E-09	-0.214	-0.207				*PTPRR*	1stExon
cg08738346	5	152,352,697	0.039437	-0.214	-0.228					
cg18807895	5	110,427,112	0.000415	-0.214	-0.170				*WDR36*	TSS1500
cg26422762	22	46,481,785	1.96E-06	-0.214	-0.198				*LOC400931*	TSS200
cg04212127	11	78,809,824	2.48E-05	-0.214	-0.167				*ODZ4*	5'UTR
cg01902355	4	49,520,738	0.001766	-0.215	-0.160					
cg05089225	4	7,395,027	0.000239	-0.215	-0.153				*SORCS2*	Body
cg07333608	2	240,445,555	0.000748	-0.215	-0.156					
cg09765297	16	83,029,415	0.000286	-0.215	-0.173				*CDH13*	Body
cg07443717	3	129,383,357	0.00606	-0.215	-0.252				*TMCC1*	Body
cg08651590	2	65,543,410	7.28E-09	-0.215	-0.206				*SPRED2*	Body
cg02305353	12	113,307,748	0.03024	-0.215	-0.164				*RPH3A*	Body
cg23024484	3	35,780,851	0.029736	-0.215	-0.148				*ARPP-21*	Body
cg00009553	16	61,854,084	0.018973	-0.215	-0.145				*CDH8*	Body
cg03123091	1	27,080,598	9.94E-06	-0.215	-0.218				*ARID1A*	Body

cg07641643	12	123,234,584	1.14E-10	-0.215	-0.211					
cg05269432	3	118,663,470	0.016364	-0.215	-0.198				*IGSF11*	Body
cg10559407	4	147,287,029	1.86E-05	-0.215	-0.189				*SLC10A7*	Body
cg27580689	10	115,939,651	3.43E-11	-0.215	-0.212				*TDRD1*	5'UTR
cg02634861	1	10,757,954	0.000231	-0.215	-0.219				*CASZ1*	5'UTR
cg19303187	2	172,968,881	4.55E-06	-0.215	-0.204				*DLX2*	TSS1500
cg05123257	16	88,163,879	0.002264	-0.215	-0.145					
cg02088996	7	41,817,771	0.024007	-0.215	-0.186				*LOC285954*	Body
cg10899768	8	102,506,635	5.81E-12	-0.215	-0.211				*GRHL2*	Body
cg04293948	15	96,158,220	0.001152	-0.215	-0.153					
cg23766996	7	86,272,023	0.03093	-0.215	-0.208				*GRM3*	TSS1500
cg08908482	13	80,377,426	0.039945	-0.215	-0.083					
cg00114478	8	73,658,110	0.000222	-0.215	-0.240				*KCNB2*	Body
cg09029902	20	52,199,594	1.36E-13	-0.215	-0.228				*ZNF217*	5'UTR
cg14721506	20	61,758,358	0.000782	-0.215	-0.172					
cg09009466	10	11,483,241	0.001952	-0.215	-0.170					
cg10315366	10	134,911,047	0.000566	-0.215	-0.113				*GPR123*	Body
cg17497052	11	125,763,722	6.35E-05	-0.215	-0.177				*PUS3*	Body
cg09473613	1	24,152,604	0.002112	-0.215	-0.212				*HMGCL*	TSS1500
cg01751800	4	77,820,391	0.000205	-0.215	-0.191				*ANKRD56*	TSS1500
cg07356534	1	158,008,055	0.008708	-0.215	-0.140				*KIRREL*	Body
cg26318682	2	53,261,081	0.026708	-0.215	-0.144					
cg20962500	1	149,174,971	0.041763	-0.215	-0.117					
cg09021430	5	549,028	0.001071	-0.215	-0.140					
cg07857792	19	18,060,683	0.024278	-0.215	-0.278				*KCNN1*	TSS1500
cg10677017	11	132,184,930	0.026906	-0.215	-0.235				*NTM*	Body
cg26772104	17	51,501,596	0.020231	-0.215	-0.132					
cg10283182	16	77,998,415	0.004	-0.215	-0.178				*VAT1L*	Body
cg08470031	11	2,423,043	1.16E-09	-0.215	-0.189				*TSSC4*	TSS1500
cg15590780	1	216,596,639	0.025834	-0.215	-0.274	0.00060695	-0.095		*USH2A*	1stExon
cg10028983	11	68,174,077	1.09E-07	-0.215	-0.221				*LRP5*	Body
cg25494605	5	173,472,097	0.0161	-0.215	-0.211				*HMP19*	TSS1500
cg22671986	4	62,799,523	0.002234	-0.215	-0.179				*LPHN3*	Body
cg01407447	13	77,090,294	0.005961	-0.215	-0.161					

cg09617441	6	36,287,380	3.63E-08	-0.215	-0.212				*C6orf222*	Body
cg00208218	2	71,251,453	0.000162	-0.215	-0.215				*OR7E91P*	Body
cg24205414	2	127,560,448	0.011607	-0.215	-0.157					
cg05648964	12	8,833,355	0.007668	-0.215	-0.138					
cg07179329	16	83,171,992	0.00161	-0.215	-0.210				*CDH13*	Body
cg21762655	3	30,554,481	1.73E-10	-0.215	-0.210					
cg21015470	11	132,621,619	0.048194	-0.215	-0.268				*OPCML*	Body
cg08845685	2	8,167,037	0.002548	-0.215	-0.138					
cg22866825	5	125,936,419	1.87E-05	-0.215	-0.168				*PHAX*	TSS1500
cg07458500	19	2,796,382	0.00012	-0.215	-0.199				*THOP1*	Body
cg09455823	2	201,606,912	3.74E-11	-0.215	-0.199					
cg09834770	5	78,536,281	0.009355	-0.215	-0.210				*JMY*	Body
cg18252424	3	13,862,475	0.024631	-0.215	-0.127				*WNT7A*	Body
cg24837219	3	132,772,692	0.001455	-0.215	-0.235				*TMEM108*	5'UTR
cg04119852	3	182,840,603	0.000166	-0.215	-0.142				*LAMP3*	3'UTR
cg05612673	12	72,836,959	0.027404	-0.215	-0.112				*TRHDE*	Body
cg04446870	17	64,440,273	0.007403	-0.215	-0.259				*PRKCA*	Body
cg21854228	11	122,192,427	0.000513	-0.215	-0.192					
cg00081465	18	77,383,609	0.001252	-0.215	-0.129					
cg07824497	16	86,930,033	0.032761	-0.215	-0.110					
cg09228327	12	24,908,780	0.039645	-0.215	-0.231					
cg15086439	1	236,563,070	0.001063	-0.215	-0.187				*EDARADD*	Body
cg09581935	10	114,591,707	4.31E-05	-0.215	-0.234					
cg07473976	21	39,292,764	1.67E-06	-0.215	-0.178					
cg15813550	20	62,221,886	0.013628	-0.215	-0.223				*GMEB2*	Body
cg14696820	1	152,800,168	0.003642	-0.215	-0.216	3.69E-09	-0.095		*LCE1A*	1stExon
cg03794214	22	42,912,017	0.000102	-0.215	-0.182				*RRP7A*	Body
cg04208435	1	192,116,518	0.034358	-0.215	-0.127					
cg04314261	12	10,163,030	2.67E-05	-0.215	-0.207				*CLEC12B*	TSS200
cg15799605	17	30,948,425	6.73E-10	-0.215	-0.211				*MYO1D*	Body
cg10008571	17	76,987,812	0.000209	-0.215	-0.200				*CANT1*	3'UTR
cg19232395	11	8,550,221	0.000976	-0.215	-0.187				*STK33*	5'UTR
cg23891487	12	93,969,700	0.017569	-0.215	-0.071				*SOCS2*	3'UTR
cg08001580	8	6,637,992	5.32E-06	-0.215	-0.205					

cg12309668	4	186,732,837	3.95E-09	-0.215	-0.212				*SORBS2*	5'UTR
cg15039415	17	45,690,396	4.22E-11	-0.215	-0.219				*NPEPPS*	Body
cg16740364	19	34,718,795	2.8E-06	-0.215	-0.213				*LSM14A*	3'UTR
cg08787698	2	9,172,228	2.79E-06	-0.215	-0.191					
cg02617560	8	32,785,757	0.000291	-0.215	-0.179					
cg03632782	16	82,781,365	0.001154	-0.215	-0.219				*CDH13*	Body
cg20016599	11	104,508,637	0.005886	-0.215	-0.096					
cg11366886	20	11,666,805	0.01127	-0.215	-0.166					
cg05082920	8	91,108,188	0.009735	-0.215	-0.208					
cg12068366	19	4,548,354	1.66E-05	-0.215	-0.187				*SEMA6B*	Body
cg22786657	10	11,381,854	0.019403	-0.215	-0.143					
cg19091779	12	13,200,089	0.003148	-0.215	-0.301				*KIAA1467*	Body
cg13576330	5	92,943,267	3.41E-05	-0.215	-0.232					
cg02609724	5	139,506,447	0.000651	-0.215	-0.214				*C5orf53*	1stExon
cg10947001	4	139,593,196	0.002237	-0.215	-0.197					
cg03192231	3	35,786,480	0.005971	-0.216	-0.157				*ARPP-21*	Body
cg02540593	11	103,908,576	0.009103	-0.216	-0.190				*PDGFD*	Body
cg04900186	7	50,347,487	0.027869	-0.216	-0.143			Yes	*IKZF1*	5'UTR
cg19243842	1	3,058,521	0.003984	-0.216	-0.162				*PRDM16*	Body
cg07047815	11	1,909,656	0.004047	-0.216	-0.148				*LSP1*	3'UTR
cg16219879	16	32,937,057	1.03E-05	-0.216	-0.192					
cg17373345	8	66,946,188	1.39E-09	-0.216	-0.210				*DNAJC5B*	5'UTR
cg17000480	10	134,756,994	1.69E-05	-0.216	-0.198				*C10orf93*	TSS1500
cg10841338	X	150,342,651	3.17E-08	-0.216	-0.168					
cg05000772	17	48,194,500	3.12E-06	-0.216	-0.221				*SAMD14*	Body
cg04350120	8	93,004,284	0.022129	-0.216	-0.272				*RUNX1T1*	Body
cg08197072	16	1,316,081	0.012041	-0.216	-0.065					
cg27408897	18	67,136,810	0.028039	-0.216	-0.101				*DOK6*	Body
cg16377738	17	76,141,068	3.37E-06	-0.216	-0.220				*C17orf99*	TSS1500
cg27361847	5	2,865,179	0.049864	-0.216	-0.069					
cg05330604	15	91,837,300	1.96E-05	-0.216	-0.259				*SV2B*	3'UTR
cg00618087	17	16,282,382	0.000493	-0.216	-0.250					
cg09787089	1	54,739,176	7.54E-05	-0.216	-0.190				*SSBP3*	Body
cg14834881	4	92,034,737	3.93E-07	-0.216	-0.175				*FAM190A*	Body

cg16710099	1	3,162,173	2E-05	-0.216	-0.165				*PRDM16*	Body
cg11058951	7	92,855,729	0.005716	-0.216	-0.213				*HEPACAM2*	1stExon
cg07656641	22	50,018,876	0.029682	-0.216	-0.117				*C22orf34*	Body
cg06969622	17	79,022,798	0.000612	-0.216	-0.151				*BAIAP2*	Body
cg22235258	11	34,675,402	1.03E-05	-0.216	-0.217				*EHF*	Body
cg26856658	12	128,899,884	0.01012	-0.216	-0.159				*TMEM132C*	Body
cg25080924	4	176,339,367	0.000249	-0.216	-0.169					
cg26518932	5	169,720,975	4.77E-06	-0.216	-0.149				*LCP2*	Body
cg25029529	2	208,795,859	0.000133	-0.216	-0.230				*PLEKHM3*	Body
cg26242755	6	6,624,147	6.68E-11	-0.216	-0.183				*LOC285780*	TSS1500
cg10115148	1	2,872,908	0.025813	-0.216	-0.111					
cg02328713	1	2,704,354	0.015833	-0.216	-0.091					
cg02487061	1	85,721,250	0.000256	-0.216	-0.230				*C1orf52*	Body
cg20135307	7	127,256,705	0.020352	-0.216	-0.118				*PAX4*	TSS1500
cg01553182	6	29,342,533	0.003582	-0.216	-0.162				*OR12D3*	1stExon
cg18009704	17	3,117,981	0.031026	-0.216	-0.120				*OR1A1*	TSS1500
cg22073121	5	41,045,396	0.040831	-0.216	-0.166				*HEATR7B2*	Body
cg09241714	7	50,366,850	0.014023	-0.216	-0.211				*IKZF1*	Body
cg18413237	7	41,742,795	0.006252	-0.216	-0.215				*LOC285954*	Body
cg10773972	15	81,035,670	4.09E-05	-0.216	-0.234				*FAM108C1*	Body
cg27533472	11	59,856,225	0.000194	-0.216	-0.197				*MS4A2*	1stExon
cg04957903	7	38,880,852	0.006512	-0.216	-0.099				*VPS41*	Body
cg09154034	4	172,846,599	0.004737	-0.216	-0.170				*GALNTL6*	Body
cg26236655	2	200,051,434	0.034832	-0.216	-0.203					
cg06832258	8	34,533,034	0.04142	-0.216	-0.087					
cg23484230	10	133,218,314	0.045917	-0.216	-0.106					
cg24938372	6	1,603,115	8.15E-07	-0.216	-0.232					
cg25429446	4	113,839,045	5.06E-07	-0.216	-0.200				*ANK2*	Body
cg02855850	1	152,882,420	0.023545	-0.216	-0.168				*IVL*	Body
cg23290674	8	112,906,823	0.001583	-0.216	-0.187					
cg08908643	5	12,794,310	0.010096	-0.216	-0.176					
cg14494812	17	48,941,039	1.29E-11	-0.216	-0.203				*TOB1*	1stExon
cg07199257	15	78,640,536	1.9E-06	-0.216	-0.206				*CRABP1*	3'UTR
cg18472159	5	3,188,172	0.016702	-0.216	-0.272					

cg20162599	11	63,990,138	0.020738	-0.216	-0.207	0.00176914	-0.073		*FERMT3*	Body
cg23268879	7	5,359,951	0.000132	-0.216	-0.182				*TNRC18*	Body
cg23615488	1	66,869,943	2.66E-12	-0.216	-0.207					
cg15306060	22	39,712,956	2.81E-11	-0.216	-0.211				*RPL3*	Body
cg01387036	17	32,690,255	1.67E-08	-0.216	-0.180				*CCL1*	TSS200
cg25013044	3	193,096,009	0.036144	-0.216	-0.111				*ATP13A5*	Body
cg00232160	9	129,468,157	1.72E-06	-0.216	-0.187					
cg24645149	1	3,159,083	0.01217	-0.216	-0.172				*PRDM16*	Body
cg02492873	2	25,193,821	0.000791	-0.216	-0.228				*DNAJC27*	Body
cg03970643	4	23,505,221	0.012026	-0.216	-0.106					
cg10481072	7	92,459,517	0.000225	-0.216	-0.206				*CDK6*	Body
cg04415689	2	242,128,250	1.36E-07	-0.216	-0.173	3.05E-25	-0.187		*ANO7*	Body
cg07945618	15	99,926,398	8.79E-08	-0.216	-0.186				*LRRC28*	3'UTR
cg08691775	8	134,310,658	2.1E-11	-0.216	-0.206				*NDRG1*	TSS1500
cg09218757	15	66,944,475	0.020563	-0.216	-0.140					
cg18067134	3	71,084,634	7.15E-06	-0.216	-0.224				*FOXP1*	Body
cg18740800	11	27,914,934	0.03544	-0.216	-0.142	0.00000111	-0.158			
cg15811371	19	9,093,198	0.001164	-0.216	-0.188				*MUC16*	TSS1500
cg09538031	5	41,072,796	0.020701	-0.216	-0.167				*HEATR7B2*	TSS1500
cg24784129	15	57,215,558	0.000133	-0.216	-0.207				*TCF12*	Body
cg12981595	17	39,254,427	1.45E-05	-0.216	-0.140				*KRTAP4-8*	TSS200
cg09829888	12	32,758,168	7.71E-06	-0.216	-0.202				*FGD4*	Body
cg18029167	8	110,693,289	9.2E-06	-0.216	-0.228				*GOLSYN*	5'UTR
cg19371121	1	175,050,479	0.015299	-0.216	-0.169				*TNN*	Body
cg20694666	13	21,343,614	3.19E-08	-0.216	-0.191			Yes	*N6AMT2*	5'UTR
cg02876191	6	54,328,858	0.005706	-0.216	-0.198					
cg05440289	1	152,881,119	0.006038	-0.216	-0.133	0.00035855	-0.114		*IVL*	5'UTR
cg06980460	11	49,230,548	7.25E-05	-0.216	-0.188	6.03E-11	-0.213		*FOLH1*	TSS1500
cg00434573	19	2,041,960	7.21E-08	-0.216	-0.198				*MKNK2*	Body
cg23856293	20	62,795,341	0.014589	-0.216	-0.230				*MYT1*	TSS1500
cg26606947	10	123,781,602	8.36E-07	-0.216	-0.202				*TACC2*	Body
cg07111988	1	24,971,764	7.01E-06	-0.216	-0.215				*SRRM1*	Body
cg21549695	5	74,333,288	1.31E-05	-0.216	-0.202					
cg03684845	16	4,606,754	1.07E-12	-0.216	-0.229			Yes	*LOC342346*	1stExon

cg10893667	5	111,313,992	0.014789	-0.216	-0.240				*C5orf13*	TSS1500
cg27015047	10	124,320,805	7.71E-06	-0.216	-0.207	0.00000112	-0.121		*DMBT1*	Body
cg09180566	13	24,736,095	6.36E-19	-0.216	-0.196				*SPATA13*	5'UTR
cg10785153	13	48,011,758	2.49E-05	-0.217	-0.226					
cg24139898	19	35,531,817	5.82E-05	-0.217	-0.200				*HPN*	5'UTR
cg11020894	3	186,659,238	1.55E-12	-0.217	-0.210				*ST6GAL1*	5'UTR
cg17345276	2	27,349,220	1.56E-06	-0.217	-0.211			Yes	*ABHD1*	Body
cg22575436	5	31,323,515	0.010827	-0.217	-0.151				*CDH6*	3'UTR
cg26794227	5	169,483,131	0.03247	-0.217	-0.189				*DOCK2*	Body
cg16734451	8	32,430,022	0.000451	-0.217	-0.155				*NRG1*	Body
cg17572903	20	47,713,223	0.004764	-0.217	-0.252				*CSE1L*	3'UTR
cg11737879	6	143,779,064	0.000115	-0.217	-0.230				*PEX3*	Body
cg12665414	5	173,318,755	2.37E-07	-0.217	-0.207				*CPEB4*	Body
cg01373819	15	91,209,329	4.63E-07	-0.217	-0.199					
cg03768338	8	76,823,133	0.000387	-0.217	-0.235					
cg27287305	4	100,576,985	0.005781	-0.217	-0.201					
cg06154311	20	61,002,657	5.96E-06	-0.217	-0.218				*C20orf151*	TSS200
cg13804467	9	28,907,222	0.000801	-0.217	-0.165					
cg19942083	12	7,070,562	6.41E-11	-0.217	-0.197					
cg24369233	2	127,713,082	0.000219	-0.217	-0.223					
cg14019464	20	377,350	0.006759	-0.217	-0.103				*TRIB3*	3'UTR
cg03785456	2	134,255,543	0.001756	-0.217	-0.292				*NCKAP5*	Body
cg00406188	1	152,647,216	0.044542	-0.217	-0.093	0.00186677	-0.101		*LCE2C*	TSS1500
cg20597842	12	7,818,585	0.01677	-0.217	-0.136				*APOBEC1*	TSS200
cg21186966	4	5,401,430	7.79E-14	-0.217	-0.201				*STK32B*	Body
cg06625721	12	33,205,814	0.003438	-0.217	-0.186					
cg08925606	20	31,595,509	6.22E-10	-0.217	-0.219				*BPIL1*	5'UTR
cg18319612	1	203,257,946	1.72E-06	-0.217	-0.216					
cg00215851	4	46,427,528	0.00098	-0.217	-0.176					
cg21334510	5	1,093,580	7.74E-05	-0.217	-0.174				*SLC12A7*	Body
cg25767154	8	145,594,413	1.67E-05	-0.217	-0.261					
cg05086987	21	46,931,501	0.001929	-0.217	-0.152				*COL18A1*	Body
cg09807227	17	80,201,957	3.9E-05	-0.217	-0.202					
cg22934449	14	104,199,773	0.000166	-0.217	-0.198				*ZFYVE21*	3'UTR

cg18051316	6	31,925,660	5.81E-08	-0.217	-0.215				*RDBP*	Body
cg23112423	7	10,516,354	0.013969	-0.217	-0.172					
cg16262284	3	182,987,901	0.003505	-0.217	-0.216				*MCF2L2*	Body
cg16854200	12	5,603,261	0.002209	-0.217	-0.171				*NTF3*	TSS200
cg00246486	3	112,642,487	0.00084	-0.217	-0.163				*CD200R1*	3'UTR
cg09820557	17	8,249,349	3.91E-05	-0.217	-0.203				*ODF4*	3'UTR
cg16819051	2	229,173,235	2.67E-05	-0.217	-0.223					
cg16179938	18	77,277,583	0.026643	-0.217	-0.219				*NFATC1*	Body
cg21484315	2	2,712,669	1.98E-06	-0.217	-0.170					
cg15814750	15	53,880,678	0.02689	-0.217	-0.164				*WDR72*	Body
cg02960488	3	178,863,507	8.36E-07	-0.217	-0.206					
cg05626536	8	144,801,088	1.68E-06	-0.217	-0.217			Yes	*MAPK15*	Body
cg22726414	8	3,316,819	0.038817	-0.217	-0.105				*CSMD1*	Body
cg14898232	2	201,124,405	5.79E-06	-0.217	-0.214					
cg09140665	11	7,960,899	0.00324	-0.217	-0.204				*OR10A3*	1stExon
cg17913347	2	98,357,103	0.000555	-0.217	-0.155					
cg24212047	11	121,973,805	0.040416	-0.217	-0.159				*LOC399959*	Body
cg09577324	11	6,890,860	0.032948	-0.217	-0.222				*OR10A2*	TSS200
cg13364075	7	157,320,369	0.001398	-0.217	-0.150					
cg17721480	4	187,619,391	0.000604	-0.217	-0.227				*FAT1*	Body
cg14898127	15	81,587,493	0.000597	-0.217	-0.241				*IL16*	Body
cg21296602	11	93,476,060	0.002938	-0.217	-0.110				*TAF1D*	TSS1500
cg22692725	3	52,722,452	0.000155	-0.217	-0.218				*GNL3*	Body
cg25577367	11	123,676,979	0.010775	-0.217	-0.199				*OR6M1*	1stExon
cg13712197	12	124,844,874	2.29E-07	-0.217	-0.190				*NCOR2*	Body
cg24947212	10	127,999,713	6.99E-07	-0.217	-0.200				*ADAM12*	Body
cg17887364	8	37,891,957	0.0016	-0.217	-0.199			Yes	*EIF4EBP1*	Body
cg02154084	5	146,184,364	0.015783	-0.217	-0.272				*PPP2R2B*	Body
cg24614200	13	19,967,206	0.000341	-0.217	-0.166					
cg18065599	11	47,968,995	1.15E-09	-0.217	-0.217					
cg18640098	5	9,888,660	0.027329	-0.217	-0.156				*LOC285692*	Body
cg23719663	X	71,093,852	0.016801	-0.217	-0.202					
cg01229998	11	60,049,447	0.0018	-0.217	-0.211				*MS4A4A*	Body
cg04839975	3	193,656,618	0.000462	-0.217	-0.195					

cg08394602	19	22,123,885	0.000773	-0.217	-0.206					
cg23990124	7	63,639,860	0.049155	-0.217	-0.124					
cg06576783	6	53,572,893	3.45E-06	-0.217	-0.167					
cg21565960	11	61,793,285	8.58E-05	-0.217	-0.230					
cg06500134	2	11,822,560	6.37E-05	-0.217	-0.173					
cg15461017	12	34,519,837	6.56E-09	-0.217	-0.209					
cg14283447	21	43,735,412	1.93E-06	-0.217	-0.187				*TFF3*	1stExon
cg18485530	13	59,547,037	0.000393	-0.217	-0.204					
cg17667625	8	134,224,711	0.003007	-0.217	-0.247				*WISP1*	Body
cg20533982	7	158,533,927	0.002921	-0.217	-0.197				*ESYT2*	Body
cg08315399	14	76,298,938	1.37E-06	-0.217	-0.194				*TTLL5*	Body
cg09174555	1	153,364,020	1.97E-06	-0.217	-0.186				*S100A8*	TSS1500
cg06389098	16	71,424,252	2.23E-05	-0.217	-0.205				*CALB2*	Body
cg09828010	5	173,533,377	0.011382	-0.217	-0.249				*HMP19*	Body
cg25144149	10	133,317,423	0.019622	-0.217	-0.104					
cg24645447	1	243,426,679	0.0008	-0.217	-0.199				*SDCCAG8*	Body
cg27582081	3	119,294,816	1.17E-08	-0.217	-0.200					
cg26317899	17	35,527,041	0.00015	-0.217	-0.190			Yes	*ACACA*	Body
cg06417570	16	31,828,859	0.011531	-0.217	-0.215					
cg06848199	16	77,983,066	0.000635	-0.217	-0.145				*VAT1L*	Body
cg16169950	5	165,412	0.000255	-0.217	-0.297				*PLEKHG4B*	Body
cg08447373	5	90,478,943	0.034311	-0.217	-0.089					
cg09194202	11	103,910,096	0.004429	-0.217	-0.184				*PDGFD*	Body
cg12080079	1	19,007,925	0.004368	-0.217	-0.108				*PAX7*	Body
cg14313576	3	134,339,988	0.006141	-0.217	-0.259				*KY*	Body
cg17444747	5	177,915,909	1.13E-05	-0.217	-0.234				*COL23A1*	Body
cg17036744	3	56,717,720	0.002494	-0.217	-0.245				*C3orf63*	TSS1500
cg26075408	2	203,006,406	0.00024	-0.217	-0.137					
cg20642115	10	134,316,586	0.021765	-0.217	-0.041					
cg25072766	11	3,073,488	2.2E-09	-0.217	-0.243				*CARS*	Body
cg20530843	3	9,976,241	3.31E-08	-0.217	-0.195				*CRELD1*	Body
cg01841050	17	39,539,863	0.010127	-0.217	-0.115			Yes	*KRT34*	TSS1500
cg13812570	X	144,327,920	0.002918	-0.217	-0.203				*SPANXN1*	TSS1500
cg22203867	5	171,621,698	0.001356	-0.217	-0.194				*EFCAB9*	Body

cg09667753	11	50,239,550	5.87E-08	-0.217	-0.204				*LOC441601*	Body
cg14814536	11	22,648,107	0.000104	-0.217	-0.248				*FANCF*	TSS1500
cg17120792	11	55,705,284	0.002284	-0.217	-0.166				*OR5I1*	TSS1500
cg03673094	11	76,391,253	0.003444	-0.217	-0.149				*GUCY2E*	Body
cg25122369	3	168,801,790	0.000495	-0.217	-0.131				*MECOM*	3'UTR
cg05462446	16	77,574,072	0.003033	-0.217	-0.163					
cg18512205	15	40,183,559	5.34E-12	-0.217	-0.219				*GPR176*	Body
cg19540371	5	13,945,611	0.000529	-0.217	-0.204				*DNAH5*	TSS1500
cg22150051	20	23,499,697	0.045203	-0.217	-0.159				*CSTT*	TSS200
cg08596618	1	24,275,885	0.003951	-0.217	-0.124					
cg15718811	8	120,427,810	3.95E-05	-0.217	-0.219				*NOV*	TSS1500
cg18502477	11	57,946,199	0.000904	-0.217	-0.153				*OR9Q1*	5'UTR
cg20469625	8	106,582,085	0.034932	-0.217	-0.228				*ZFPM2*	Body
cg19103704	19	40,440,963	9.82E-07	-0.217	-0.183	1.58E-12	-0.120		*FCGBP*	TSS1500
cg22836134	14	75,647,041	0.000481	-0.217	-0.191					
cg01264963	11	123,885,967	5.62E-11	-0.217	-0.213				*OR10G4*	TSS1500
cg01454540	10	134,782,773	1.65E-05	-0.217	-0.173					
cg04231701	4	1,032,855	8.8E-09	-0.217	-0.204					
cg18268948	7	2,113,694	3.45E-07	-0.217	-0.201				*MAD1L1*	Body
cg25719521	2	192,742,700	0.000251	-0.217	-0.143					
cg12115882	11	133,449,267	0.026205	-0.217	-0.156					
cg26275841	14	103,040,826	0.000523	-0.217	-0.173					
cg13430173	9	112,914,149	1.03E-10	-0.217	-0.200				*AKAP2*	Body
cg25362714	22	32,804,978	0.00829	-0.217	-0.177				*C22orf28*	Body
cg14660328	11	79,878,290	4.46E-10	-0.217	-0.228					
cg04988729	11	606,583	1.73E-11	-0.217	-0.202				*PHRF1*	Body
cg22792461	10	97,850,322	0.005879	-0.217	-0.234					
cg26977737	21	22,175,522	0.007091	-0.217	-0.176				*C21orf131*	TSS200
cg01102471	2	58,584,770	0.003098	-0.217	-0.201			Yes		
cg09300089	16	89,539,230	2.38E-08	-0.217	-0.200				*ANKRD11*	5'UTR
cg14856914	10	86,018,582	0.001045	-0.217	-0.176				*RGR*	3'UTR
cg26306530	13	60,053,952	0.028354	-0.217	-0.207					
cg04287755	1	2,732,366	0.000722	-0.218	-0.191					
cg10976311	5	152,591,026	9.1E-05	-0.218	-0.207					

cg26552067	12	131,747,296	0.018247	-0.218	-0.140					
cg08327428	10	109,641,026	0.000142	-0.218	-0.208					
cg15821546	6	31,843,465	4.44E-11	-0.218	-0.213				*SLC44A4*	Body
cg25837904	4	154,167,187	9.4E-08	-0.218	-0.204				*TRIM2*	Body
cg10369242	12	52,625,486	1.78E-05	-0.218	-0.221				*KRT7*	TSS1500
cg23629187	8	125,576,109	3.86E-07	-0.218	-0.219				*MTSS1*	Body
cg27188860	8	33,801,282	1.5E-05	-0.218	-0.167					
cg06083483	17	32,646,006	5.81E-07	-0.218	-0.203				*CCL8*	TSS200
cg07588439	2	198,571,554	5.8E-07	-0.218	-0.199				*MARS2*	1stExon
cg11373895	2	78,545,567	0.028813	-0.218	-0.201					
cg21177502	8	50,968,578	0.000343	-0.218	-0.207				*SNTG1*	5'UTR
cg00050133	4	178,167,583	0.001613	-0.218	-0.150					
cg00213668	7	98,028,207	1.42E-09	-0.218	-0.225				*BAIAP2L1*	Body
cg01589580	18	77,440,800	8.22E-08	-0.218	-0.218	2.8E-12	-0.079		*CTDP1*	Body
cg05594286	16	86,840,467	0.002276	-0.218	-0.252					
cg10640284	20	13,971,811	1.69E-05	-0.218	-0.211				*SEL1L2*	TSS1500
cg13640423	3	120,412,084	0.00135	-0.218	-0.255				*RABL3*	Body
cg06127719	18	11,150,784	0.000608	-0.218	-0.201					
cg10720545	19	45,504,295	5.6E-06	-0.218	-0.171				*RELB*	TSS1500
cg17761538	8	145,753,477	0.01725	-0.218	-0.171				*MGC70857*	Body
cg18162059	4	5,405,706	0.014446	-0.218	-0.151				*STK32B*	Body
cg07700925	10	129,540,267	2.35E-05	-0.218	-0.159					
cg13085403	1	232,901,128	6.45E-08	-0.218	-0.193					
cg21166999	6	117,746,793	2.28E-06	-0.218	-0.191	0.00000855	-0.099		*ROS1*	1stExon
cg00641679	8	94,230,311	0.006769	-0.218	-0.201					
cg04960007	17	5,820,294	0.001928	-0.218	-0.156					
cg05331214	2	167,343,774	0.045076	-0.218	-0.150	4.65E-16	-0.168		*SCN7A*	TSS1500
cg09904358	10	24,446,911	0.000192	-0.218	-0.219				*KIAA1217*	5'UTR
cg15015426	1	159,506,559	4.28E-11	-0.218	-0.215				*OR10J5*	TSS1500
cg22127773	17	7,754,785	0.01941	-0.218	-0.108				*KDM6B*	Body
cg02870485	17	14,891,458	0.013491	-0.218	-0.098			Yes		
cg04133673	2	142,890,490	0.000731	-0.218	-0.194				*LRP1B*	TSS1500
cg08688636	7	31,935,390	0.001897	-0.218	-0.152				*PDE1C*	Body
cg16759787	14	85,667,733	0.007617	-0.218	-0.182					

cg15285975	3	146,498,234	5.08E-06	-0.218	-0.236					
cg19621065	15	92,459,857	0.000239	-0.218	-0.177				*SLCO3A1*	Body
cg22635088	5	2,096,922	1.02E-05	-0.218	-0.168					
cg10023915	11	9,772,285	1.21E-10	-0.218	-0.210				*SWAP70*	3'UTR
cg24359868	21	30,261,524	0.023929	-0.218	-0.143					
cg17980364	11	86,748,241	1.28E-06	-0.218	-0.217				*TMEM135*	TSS1500
cg06528591	12	131,978,899	0.005467	-0.218	-0.161					
cg14074184	10	133,837,230	5.35E-15	-0.218	-0.207					
cg20624429	11	122,471,331	0.027265	-0.218	-0.115			Yes		
cg23575688	11	119,486,443	9.45E-07	-0.218	-0.176					
cg17759816	19	1,313,033	1.64E-05	-0.218	-0.180					
cg06966245	1	30,043,002	0.036698	-0.218	-0.127					
cg13676215	7	17,337,976	8.97E-12	-0.218	-0.225				*AHR*	TSS1500
cg03782437	11	71,295,908	0.000301	-0.218	-0.141					
cg16663980	1	158,809,049	0.033607	-0.218	-0.272				*MNDA*	5'UTR
cg00169419	4	111,530,900	0.041309	-0.218	-0.100					
cg01243226	X	15,807,818	0.000111	-0.218	-0.200				*ZRSR2*	TSS1500
cg01571491	17	72,440,050	0.000976	-0.218	-0.121				*GPRC5C*	Body
cg02537838	20	61,002,595	7.88E-05	-0.218	-0.231	1.11E-07	-0.075		*C20orf151*	TSS200
cg08038219	20	62,031,924	0.000605	-0.218	-0.204					
cg16660394	16	19,089,709	4.55E-09	-0.218	-0.219				*COQ7*	3'UTR
cg21292473	1	181,683,239	0.009077	-0.218	-0.272				*CACNA1E*	Body
cg12496211	12	2,193,060	6.67E-15	-0.218	-0.203				*CACNA1C*	Body
cg17633170	12	131,217,151	0.014349	-0.218	-0.149					
cg21295827	1	178,708,719	0.038989	-0.218	-0.064				*RALGPS2*	5'UTR
cg23216731	8	130,832,237	0.000247	-0.218	-0.127					
cg24629972	8	43,147,439	0.027718	-0.218	-0.190				*POTEA*	TSS200
cg09815642	16	59,602,460	0.001362	-0.218	-0.182					
cg12599765	4	112,729,702	0.000685	-0.218	-0.163					
cg15846556	8	13,735,387	1.03E-07	-0.218	-0.203					
cg20169766	1	66,747,598	8.04E-09	-0.218	-0.212				*PDE4B*	Body
cg02307277	6	150,690,125	1.28E-10	-0.218	-0.197				*IYD*	5'UTR
cg20743662	3	38,589,496	0.022668	-0.218	-0.143					
cg03583746	6	1,604,013	2.03E-05	-0.218	-0.196					

cg02936593	11	45,734,486	5.74E-05	-0.218	-0.231					
cg07100532	18	713,085	0.000286	-0.218	-0.254				*ENOSF1*	TSS1500
cg00606396	5	19,531,574	2.13E-05	-0.218	-0.230				*CDH18*	Body
cg02442572	7	1,303,351	6.92E-05	-0.218	-0.219					
cg15441416	19	54,972,047	0.007154	-0.218	-0.176				*LENG8*	Body
cg21680327	1	36,108,225	0.002713	-0.218	-0.182				*PSMB2*	TSS1500
cg00334941	3	146,964,907	8.51E-07	-0.218	-0.251					
cg01323371	3	179,667,823	0.018093	-0.218	-0.158			Yes	*PEX5L*	Body
cg24786174	18	74,118,243	1.59E-05	-0.218	-0.200				*ZNF516*	Body
cg24984506	3	135,086,818	0.016937	-0.218	-0.266					
cg06252455	12	38,558,124	8.9E-05	-0.218	-0.193					
cg06919312	12	64,474,195	0.000494	-0.218	-0.177				*SRGAP1*	Body
cg07234199	20	37,059,825	0.006788	-0.218	-0.147				*LOC388796*	Body
cg13724820	2	44,104,732	0.000465	-0.218	-0.190				*ABCG8*	Body
cg27041655	5	87,733,473	0.000586	-0.218	-0.172					
cg05376362	6	74,826,447	0.003861	-0.218	-0.269					
cg25232786	3	161,138,842	2.76E-05	-0.218	-0.228					
cg27168033	19	3,983,219	4.7E-10	-0.218	-0.197				*EEF2*	Body
cg05193162	12	101,604,787	0.00112	-0.218	-0.168				*SLC5A8*	TSS1500
cg06855422	4	94,566,151	0.005573	-0.218	-0.175			Yes	*GRID2*	Body
cg10002131	19	53,794,492	0.002812	-0.218	-0.154				*BIRC8*	5'UTR
cg19614643	2	19,499,118	0.000245	-0.218	-0.252					
cg27244482	5	149,669,488	9.98E-06	-0.218	-0.227	0.00000131	-0.123		*CAMK2A*	TSS200
cg03943115	1	153,014,367	1.88E-05	-0.218	-0.187				*SPRR2D*	TSS1500
cg07695603	X	150,399,990	0.027313	-0.218	-0.137					
cg11039314	7	16,921,699	0.026825	-0.218	-0.235				*AGR3*	TSS200
cg09627060	10	134,875,916	0.002275	-0.218	-0.209					
cg03084648	8	23,429,119	0.000171	-0.218	-0.200				*SLC25A37*	Body
cg05402622	16	86,666,183	0.024773	-0.218	-0.125					
cg16710042	2	165,760,725	1.17E-07	-0.218	-0.187				*SLC38A11*	Body
cg10651502	4	46,738,197	0.03919	-0.218	-0.102				*COX7B2*	5'UTR
cg16237191	12	55,945,613	0.002757	-0.218	-0.180				*OR6C4*	1stExon
cg00534479	5	168,196,464	0.002873	-0.218	-0.135				*SLIT3*	Body
cg04548096	6	133,691,357	0.020662	-0.218	-0.100				*EYA4*	Body

cg04876407	2	134,781,671	0.013027	-0.218	-0.146					
cg17512522	20	6,195,391	0.041318	-0.218	-0.109					
cg26247795	21	14,407,237	0.018847	-0.218	-0.189			Yes		
cg27570873	10	135,029,717	0.006371	-0.218	-0.099				*KNDC1*	Body
cg19829001	17	74,640,078	4.33E-05	-0.218	-0.203				*ST6GALNAC1*	TSS200
cg00381745	7	99,096,450	0.000286	-0.218	-0.251				*ZNF394*	Body
cg01282725	12	1,645,963	1.05E-07	-0.218	-0.203					
cg14086922	22	18,268,239	2.01E-06	-0.218	-0.223					
cg09554339	19	43,442,693	0.013845	-0.218	-0.248				*PSG7*	TSS1500
cg04181079	11	104,896,587	0.000626	-0.218	-0.173				*CASP1*	3'UTR
cg05320945	19	14,911,751	0.004291	-0.218	-0.132				*OR7C1*	TSS1500
cg10019467	4	40,632,788	2.01E-05	-0.218	-0.224				*RBM47*	TSS1500
cg17736285	17	44,347,643	2.21E-06	-0.218	-0.210					
cg03023152	8	102,505,556	3.54E-15	-0.218	-0.221				*GRHL2*	Body
cg10488012	5	3,306,368	0.010983	-0.218	-0.127					
cg12904891	1	155,901,756	5.07E-05	-0.218	-0.156				*KIAA0907*	Body
cg05012620	17	47,886,433	1.72E-09	-0.218	-0.242				*MYST2*	Body
cg10623221	3	173,119,212	0.002953	-0.218	-0.225				*NLGN1*	5'UTR
cg01181150	7	154,407,878	0.035227	-0.218	-0.124				*DPP6*	Body
cg17081914	2	134,326,366	2.46E-09	-0.218	-0.233				*NCKAP5*	TSS1500
cg05304729	1	158,800,024	0.029665	-0.218	-0.294				*MNDA*	TSS1500
cg22069749	20	62,111,366	3.67E-20	-0.218	-0.225					
cg03424602	12	121,687,747	0.003688	-0.218	-0.166				*CAMKK2*	Body
cg21443274	8	106,782,921	0.000117	-0.219	-0.231				*ZFPM2*	Body
cg22287731	11	55,066,025	0.006719	-0.219	-0.146					
cg11150520	1	157,660,319	0.03205	-0.219	-0.134				*FCRL3*	Body
cg26192689	2	206,730,773	1.9E-12	-0.219	-0.215					
cg00425678	13	81,226,644	4.5E-07	-0.219	-0.237			Yes		
cg08279693	1	152,657,950	0.038416	-0.219	-0.095				*LCE2B*	TSS1500
cg14966325	1	153,029,005	0.019235	-0.219	-0.177				*SPRR2A*	Body
cg16404898	5	161,111,187	0.008629	-0.219	-0.220				*GABRA6*	TSS1500
cg24687276	15	89,163,928	5.67E-06	-0.219	-0.218				*AEN*	TSS1500
cg23883265	2	133,039,779	0.00017	-0.219	-0.157					
cg01130157	7	157,111,309	1.46E-08	-0.219	-0.193					

cg13066693	14	76,878,042	5.37E-05	-0.219	-0.200				*ESRRB*	5'UTR
cg08467103	2	65,593,933	2.94E-22	-0.219	-0.201				*SPRED2*	TSS200
cg09736361	2	242,833,228	0.000135	-0.219	-0.200					
cg18177920	11	5,956,787	0.007697	-0.219	-0.182					
cg25054821	12	119,216,236	0.005797	-0.219	-0.204					
cg03904711	3	118,865,255	1.34E-06	-0.219	-0.183				*C3orf30*	1stExon
cg12387464	11	123,775,935	3.35E-05	-0.219	-0.200				*OR8D4*	TSS1500
cg27304952	12	52,077,539	1.21E-07	-0.219	-0.201				*SCN8A*	Body
cg23517941	20	62,052,997	3.91E-05	-0.219	-0.129				*KCNQ2*	Body
cg11479568	8	41,522,951	1.21E-06	-0.219	-0.208				*ANK1*	TSS200
cg16663645	3	116,639,591	0.000135	-0.219	-0.225					
cg17277939	12	70,093,008	0.000106	-0.219	-0.217				*BEST3*	1stExon
cg19235974	12	1,063,197	0.004135	-0.219	-0.155					
cg19071976	6	32,026,656	0.002311	-0.219	-0.173				*TNXB*	Body
cg21214566	9	138,549,731	0.014058	-0.219	-0.135					
cg13374304	7	63,016,648	0.001434	-0.219	-0.173					
cg10200451	8	130,353,282	0.043995	-0.219	-0.210					
cg17631914	4	49,514,820	5.34E-25	-0.219	-0.196					
cg24655087	15	26,416,943	0.000161	-0.219	-0.209					
cg21542643	7	99,425,614	4.2E-10	-0.219	-0.209				*CYP3A43*	TSS200
cg24670445	11	57,519,282	1.04E-10	-0.219	-0.208				*BTBD18*	TSS200
cg26347851	20	61,754,869	0.003121	-0.219	-0.125					
cg19518426	5	2,866,779	0.01216	-0.219	-0.119					
cg14668152	10	131,357,256	0.000233	-0.219	-0.215				*MGMT*	Body
cg18384588	22	46,463,747	1.35E-08	-0.219	-0.194					
cg19223521	2	119,436,490	0.011222	-0.219	-0.225					
cg23846443	6	155,777,949	0.004414	-0.219	-0.178				*NOX3*	TSS1500
cg13588599	9	131,102,140	1.65E-07	-0.219	-0.195				*SLC27A4*	TSS1500
cg24375470	2	68,066,948	1.06E-12	-0.219	-0.211					
cg14301118	20	47,003,643	0.041597	-0.219	-0.162					
cg22036057	1	104,097,670	3.52E-05	-0.219	-0.198				*AMY2B*	5'UTR
cg04742334	20	37,850,248	0.004117	-0.219	-0.176				*LOC339568*	Body
cg22839180	5	175,117,697	0.044842	-0.219	-0.126					
cg22912214	8	145,675,857	7.8E-05	-0.219	-0.200				*CYHR1*	Body

cg04234597	5	3,851,581	2.29E-11	-0.219	-0.227					
cg00032490	12	131,402,349	0.008919	-0.219	-0.066					
cg01179769	13	45,742,810	1.76E-07	-0.219	-0.261				*GTF2F2*	Body
cg18587499	14	94,121,146	3.16E-09	-0.219	-0.184				*KIAA1409*	Body
cg11526413	15	90,223,712	1.19E-09	-0.219	-0.230				*PLIN1*	TSS1500
cg01169521	14	19,746,900	1.29E-09	-0.219	-0.192					
cg04515258	17	78,825,412	0.038805	-0.219	-0.180				*RPTOR*	Body
cg07183970	12	34,457,254	0.036777	-0.219	-0.132					
cg21093807	3	56,717,625	1.81E-05	-0.219	-0.240				*C3orf63*	TSS1500
cg02315870	7	26,236,721	0.000118	-0.219	-0.192				*HNRNPA2B1*	Body
cg02639667	5	154,092,321	1.18E-05	-0.219	-0.208				*LARP1*	TSS200
cg11850659	6	164,254,856	0.011104	-0.219	-0.181					
cg22618219	6	133,036,651	0.012288	-0.219	-0.181				*VNN1*	TSS1500
cg24580655	1	159,145,638	0.000276	-0.219	-0.157				*CADM3*	Body
cg23296792	5	147,777,756	0.001236	-0.219	-0.203			Yes	*FBXO38*	Body
cg07450749	14	43,123,081	2.6E-05	-0.219	-0.250					
cg13016237	7	154,681,513	0.044385	-0.219	-0.163				*DPP6*	Body
cg24702147	12	7,071,814	0.000337	-0.219	-0.215				*MIR141*	TSS1500
cg23714454	11	125,756,760	0.012658	-0.219	-0.275				*HYLS1*	TSS200
cg09668599	2	44,231,045	0.000795	-0.219	-0.158					
cg22022671	5	83,253,023	0.006326	-0.219	-0.157				*EDIL3*	Body
cg03448548	16	33,604,228	5.83E-05	-0.219	-0.192					
cg05433222	7	94,948,020	0.000347	-0.219	-0.212				*PON1*	Body
cg10711528	3	189,022,661	7.49E-05	-0.219	-0.188				*TPRG1*	Body
cg21045575	13	59,169,584	5.86E-05	-0.219	-0.203					
cg10399850	1	1,089,668	6.41E-05	-0.219	-0.196					
cg15998773	1	15,987,116	0.001313	-0.219	-0.197				*RSC1A1*	1stExon
cg20995977	1	4,856,996	0.049115	-0.219	-0.082					
cg15307285	11	22,648,074	0.000168	-0.219	-0.237				*FANCF*	TSS1500
cg23990303	8	130,760,646	0.033056	-0.219	-0.159				*GSDMC*	3'UTR
cg00585076	6	168,376,325	0.021274	-0.219	-0.142					
cg14004552	6	32,167,840	4.87E-05	-0.219	-0.143				*NOTCH4*	Body
cg20353653	15	85,143,219	3.59E-08	-0.219	-0.218				*ZSCAN2*	TSS1500
cg20067780	17	78,008,520	1.13E-05	-0.219	-0.184				*TBC1D16*	5'UTR

cg02586198	16	29,820,119	7.36E-27	-0.219	-0.209				*MAZ*	Body
cg26012050	20	17,292,250	0.03552	-0.219	-0.146				*PCSK2*	Body
cg02683929	13	39,566,053	0.004712	-0.219	-0.185				*STOML3*	TSS1500
cg10529845	16	49,522,337	0.028461	-0.219	-0.160					
cg01394884	4	186,961,192	1.02E-12	-0.219	-0.209					
cg04092699	2	76,975,220	0.000621	-0.219	-0.194				*LRRTM4*	3'UTR
cg06587955	Y	9,368,165	0.000278	-0.219	-0.175				*TSPY4*	3'UTR
cg12210543	19	12,175,107	2.31E-11	-0.219	-0.208				*ZNF844*	TSS1500
cg02326301	1	228,650,406	2.86E-06	-0.219	-0.227					
cg01519063	8	102,505,293	2.27E-08	-0.219	-0.219				*GRHL2*	Body
cg14266927	14	75,987,682	0.000428	-0.219	-0.229				*BATF*	TSS1500
cg18805469	1	152,944,810	0.032558	-0.219	-0.158				*SPRR4*	3'UTR
cg22867156	6	28,555,664	8.83E-22	-0.219	-0.206				*SCAND3*	TSS1500
cg14728856	X	47,052,534	5.91E-10	-0.219	-0.199				*UBA1*	5'UTR
cg24082174	3	186,503,643	4.07E-09	-0.219	-0.225				*SNORA63*	TSS1500
cg15143024	7	91,199,642	5.07E-07	-0.219	-0.199					
cg01879083	5	10,306,031	0.000844	-0.219	-0.291				*CMBL*	5'UTR
cg26906737	1	212,541,551	1.44E-06	-0.219	-0.177				*TMEM206*	Body
cg02901136	1	153,348,305	0.043923	-0.219	-0.141				*S100A12*	TSS1500
cg19397885	7	12,444,115	1.38E-06	-0.219	-0.223				*VWDE*	TSS1500
cg24052731	12	34,529,645	0.005605	-0.219	-0.160					
cg10961758	5	83,222,828	1.48E-06	-0.219	-0.220					
cg25533594	11	101,450,197	0.001187	-0.219	-0.224				*TRPC6*	Body
cg02077256	17	15,133,981	3.07E-05	-0.220	-0.229				*PMP22*	3'UTR
cg05680111	6	32,047,122	0.013371	-0.220	-0.127				*TNXB*	Body
cg07511317	14	104,159,997	0.001265	-0.220	-0.229				*KLC1*	Body
cg10094886	1	204,301,278	0.021292	-0.220	-0.139				*PLEKHA6*	5'UTR
cg18440774	4	69,212,380	0.000299	-0.220	-0.170				*YTHDC1*	Body
cg22378678	3	167,185,752	1.57E-05	-0.220	-0.205			Yes	*SERPINI2*	Body
cg17002719	7	12,444,013	0.025311	-0.220	-0.198			Yes	*VWDE*	TSS200
cg08096702	15	31,358,323	0.007581	-0.220	-0.221				*MIR211*	TSS1500
cg15556873	2	78,263,008	0.009136	-0.220	-0.208					
cg10462897	16	86,457,593	0.038735	-0.220	-0.129					
cg14014964	9	94,443,692	0.034137	-0.220	-0.125					

cg19755714	4	140,842,902	1.3E-05	-0.220	-0.218				*MAML3*	Body
cg00697290	3	22,410,416	1.26E-06	-0.220	-0.223					
cg00069003	5	140,164,106	0.000293	-0.220	-0.243					
cg14248084	Y	14,076,009	0.019966	-0.220	-0.154					
cg16818361	3	118,792,317	0.030789	-0.220	-0.150				*IGSF11*	Body
cg01282269	21	41,515,306	7.21E-05	-0.220	-0.169				*DSCAM*	Body
cg17540450	13	35,167,000	0.003493	-0.220	-0.168					
cg12677267	8	36,602,729	0.003518	-0.220	-0.130					
cg06765219	8	644,572	8.21E-07	-0.220	-0.207				*ERICH1*	Body
cg03207987	11	56,000,287	0.016259	-0.220	-0.101				*OR5T2*	1stExon
cg20972294	11	133,005,919	0.048421	-0.220	-0.143				*OPCML*	Body
cg08805386	8	37,797,731	0.00252	-0.220	-0.152				*GOT1L1*	TSS200
cg11684549	17	79,122,243	0.033091	-0.220	-0.099				*AATK*	Body
cg15654892	8	60,834,013	0.00129	-0.220	-0.177					
cg24903668	5	142,941,044	9.22E-06	-0.220	-0.207					
cg07572927	14	21,155,679	1.22E-05	-0.220	-0.214				*RNASE4*	TSS1500
cg19737334	8	46,951,755	0.009044	-0.220	-0.145					
cg08120226	3	195,531,633	1.44E-08	-0.220	-0.221				*MUC4*	Body
cg09697696	5	139,488,623	0.006429	-0.220	-0.145					
cg19878326	6	30,078,632	0.010396	-0.220	-0.202				*TRIM31*	Body
cg26175846	6	32,972,492	0.003746	-0.220	-0.124				*HLA-DOA*	3'UTR
cg00214856	11	2,242,500	0.036846	-0.220	-0.191					
cg09905047	16	33,570,115	0.001365	-0.220	-0.147					
cg24850334	1	218,879,437	1.02E-10	-0.220	-0.237					
cg14646039	5	152,729,842	0.000126	-0.220	-0.199					
cg15202552	1	157,671,759	8.41E-08	-0.220	-0.230				*FCRL3*	TSS1500
cg23083074	4	89,202,819	3.32E-06	-0.220	-0.214				*PPM1K*	5'UTR
cg08371224	5	170,066,539	0.002848	-0.220	-0.154				*KCNIP1*	Body
cg26150300	6	134,141,433	1.82E-08	-0.220	-0.221				*MGC34034*	TSS1500
cg08097973	11	71,793,051	3.16E-05	-0.220	-0.193				*LRTOMT*	5'UTR
cg25636441	11	88,059,527	0.007786	-0.220	-0.266				*CTSC*	3'UTR
cg18513357	5	135,696,688	0.008673	-0.220	-0.215				*TRPC7*	Body
cg24072224	19	43,410,455	3.42E-07	-0.220	-0.207				*PSG6*	Body
cg13541257	9	44,873,530	0.001905	-0.220	-0.222					

cg15870576	5	178,545,115	0.001652	-0.220	-0.232				*ADAMTS2*	Body
cg01927373	4	82,407,884	0.000135	-0.220	-0.206					
cg04186249	22	37,006,716	0.009883	-0.220	-0.128				*CACNG2*	Body
cg09354518	16	61,951,081	0.01822	-0.220	-0.146				*CDH8*	Body
cg13181695	2	31,749,899	2.08E-06	-0.220	-0.211				*SRD5A2*	3'UTR
cg23260578	14	20,978,830	0.021235	-0.220	-0.190				*RNASE10*	1stExon
cg14201634	11	111,831,876	0.001328	-0.220	-0.176				*DIXDC1*	Body
cg10137253	14	101,507,590	0.000596	-0.220	-0.248				*MIR300*	TSS200
cg16777493	16	84,483,628	3.85E-06	-0.220	-0.208				*ATP2C2*	Body
cg09756014	16	89,152,366	0.042403	-0.220	-0.088					
cg11619879	5	121,022,314	0.001554	-0.220	-0.138					
cg16405876	5	78,317,871	3.98E-07	-0.220	-0.194				*DMGDH*	Body
cg21997672	7	123,221,351	2.86E-10	-0.220	-0.202					
cg02512902	17	25,799,447	4.32E-05	-0.220	-0.203				*KSR1*	5'UTR
cg01828798	Y	8,571,457	0.026667	-0.220	-0.145				*TTTY19*	TSS1500
cg15896356	18	72,168,601	0.000925	-0.220	-0.162				*CNDP2*	Body
cg23504924	5	13,945,371	2.14E-06	-0.220	-0.221				*DNAH5*	TSS1500
cg18940763	22	29,197,846	4.3E-07	-0.220	-0.235	3.88E-10	-0.175		*XBP1*	TSS1500
cg21631443	15	100,049,225	0.003185	-0.220	-0.139					
cg23493497	11	131,992,673	0.01521	-0.220	-0.154				*NTM*	Body
cg20084079	11	55,135,656	0.005801	-0.220	-0.166				*OR4A15*	1stExon
cg00012148	6	54,172,997	0.049738	-0.220	-0.063				*TINAG*	TSS1500
cg07151236	20	59,543,878	0.006943	-0.220	-0.147					
cg23003315	6	30,070,109	1.95E-06	-0.220	-0.217					
cg25840824	20	56,269,575	3.41E-07	-0.220	-0.242				*PMEPA1*	5'UTR
cg03180819	2	166,061,599	0.001019	-0.220	-0.172				*SCN3A*	TSS1500
cg03271150	19	46,387,760	0.00169	-0.220	-0.184				*IRF2BP1*	1stExon
cg06878009	16	77,874,011	1.92E-07	-0.220	-0.224				*VAT1L*	Body
cg07624310	15	26,235,362	0.012469	-0.220	-0.123					
cg15690511	11	50,004,210	1.89E-08	-0.220	-0.227				*OR4C12*	TSS200
cg06411879	10	21,456,039	1.4E-08	-0.220	-0.220				*NEBL*	Body
cg18617860	20	10,202,522	1.98E-07	-0.220	-0.190				*SNAP25*	5'UTR
cg03072740	12	56,030,437	0.041859	-0.220	-0.126				*OR10P1*	TSS1500
cg05696931	1	227,175,867	1.83E-06	-0.220	-0.226					

cg13959062	8	2,363,737	0.001865	-0.220	-0.180					
cg22222261	11	22,219,092	0.006329	-0.220	-0.288				*ANO5*	Body
cg25032793	18	76,125,627	0.047409	-0.220	-0.122					
cg09418000	7	39,056,400	1.62E-05	-0.220	-0.231				*POU6F2*	Body
cg13556767	9	8,234,760	0.000339	-0.220	-0.199					
cg04165933	16	49,833,847	0.010847	-0.220	-0.119				*ZNF423*	Body
cg14200127	1	97,604,182	1.56E-08	-0.220	-0.234				*DPYD*	Body
cg16237565	15	101,922,942	2.55E-07	-0.220	-0.201				*PCSK6*	Body
cg02384419	7	104,568,180	0.000224	-0.220	-0.231				*LOC723809*	TSS1500
cg23855121	4	38,784,938	0.000293	-0.220	-0.174				*TLR10*	TSS1500
cg10821841	13	112,200,892	0.046069	-0.220	-0.065					
cg11073813	6	109,029,018	0.000195	-0.220	-0.238					
cg20564865	6	31,096,861	2.44E-05	-0.220	-0.227				*PSORS1C1*	5'UTR
cg19214658	13	112,892,641	0.022619	-0.221	-0.133					
cg01276031	14	88,737,828	0.009989	-0.221	-0.155				*KCNK10*	TSS1500
cg10996368	11	131,940,246	0.001645	-0.221	-0.201				*NTM*	Body
cg12811617	14	105,446,698	2.24E-14	-0.221	-0.190					
cg11182199	6	150,161,689	4.15E-06	-0.221	-0.198				*LRP11*	Body
cg06954520	18	68,095,624	2.23E-05	-0.221	-0.202					
cg13721907	14	48,148,429	2.12E-07	-0.221	-0.208					
cg20318217	2	147,172,982	0.01406	-0.221	-0.192					
cg07646665	4	12,052,569	0.007965	-0.221	-0.260					
cg03274669	13	74,805,233	5.76E-05	-0.221	-0.189					
cg12990035	3	98,690,540	0.015481	-0.221	-0.243					
cg14840487	1	220,962,870	0.009166	-0.221	-0.097				*MOSC1*	Body
cg27292264	3	128,079,636	2.51E-06	-0.221	-0.268				*MIR1280*	TSS1500
cg02094827	10	99,496,515	8.11E-09	-0.221	-0.197				*ZFYVE27*	TSS1500
cg04107005	13	113,029,350	0.03299	-0.221	-0.131				*C13orf28*	TSS1500
cg08981648	2	233,750,457	2.02E-05	-0.221	-0.210				*NGEF*	Body
cg18181492	1	2,340,084	0.001811	-0.221	-0.259				*PEX10*	Body
cg11819637	3	184,095,505	0.008901	-0.221	-0.197	2.31E-11	-0.185		*THPO*	5'UTR
cg20481640	1	100,928,376	1.68E-09	-0.221	-0.219				*CDC14A*	Body
cg20503535	14	91,755,616	1.85E-05	-0.221	-0.220				*CCDC88C*	Body
cg24756966	6	43,478,692	0.005495	-0.221	-0.179				*C6orf154*	TSS1500

cg26548729	2	127,401,321	0.004952	-0.221	-0.162					
cg20325454	2	74,078,577	6.5E-10	-0.221	-0.228				*STAMBP*	Body
cg01272393	2	103,239,393	0.000201	-0.221	-0.153				*SLC9A2*	Body
cg01727408	16	85,609,071	0.000313	-0.221	-0.213					
cg13097800	14	47,104,140	0.010611	-0.221	-0.264					
cg01554539	7	157,413,902	0.013527	-0.221	-0.215				*PTPRN2*	Body
cg15082394	6	33,264,525	0.000891	-0.221	-0.178				*RGL2*	Body
cg10131232	20	1,610,358	0.007647	-0.221	-0.131				*SIRPG*	3'UTR
cg22542632	4	59,362,677	0.034925	-0.221	-0.099					
cg01478628	11	20,614,302	0.000204	-0.221	-0.206					
cg21181391	8	88,886,411	0.035256	-0.221	-0.111				*DCAF4L2*	TSS200
cg22860195	2	364,258	6.66E-06	-0.221	-0.155					
cg00148513	1	241,584,653	0.042599	-0.221	-0.105					
cg13934236	22	43,507,609	1.05E-08	-0.221	-0.217				*BIK*	5'UTR
cg02490589	1	158,944,784	0.019229	-0.221	-0.177				*PYHIN1*	Body
cg08464505	13	113,425,982	3.13E-05	-0.221	-0.202				*ATP11A*	Body
cg08658634	1	184,319,916	1.74E-05	-0.221	-0.191					
cg23567627	14	104,093,551	1.76E-05	-0.221	-0.218					
cg02304751	4	99,583,945	6.1E-06	-0.221	-0.230					
cg08891123	15	59,576,097	3.56E-06	-0.221	-0.219				*MYO1E*	Body
cg00397764	10	115,939,717	1.03E-10	-0.221	-0.214				*TDRD1*	5'UTR
cg23050227	8	112,241,152	0.017461	-0.221	-0.221					
cg19521693	19	54,468,577	9.38E-08	-0.221	-0.186				*CACNG8*	Body
cg23132327	11	80,167,599	1.01E-07	-0.221	-0.214					
cg01787460	5	4,868,532	2.84E-07	-0.221	-0.200					
cg27021368	6	155,639,080	0.006033	-0.221	-0.195					
cg00927226	7	115,608,801	0.00411	-0.221	-0.248				*TFEC*	Body
cg01661235	16	23,988,974	0.036596	-0.221	-0.163				*PRKCB*	Body
cg26124968	7	44,801,571	0.000922	-0.221	-0.183				*ZMIZ2*	Body
cg15776919	4	181,463,296	0.000153	-0.221	-0.190					
cg11394361	17	67,570,862	0.041909	-0.221	-0.129					
cg03784113	13	112,602,492	0.007764	-0.221	-0.144					
cg05017309	6	154,361,687	0.014552	-0.221	-0.109				*OPRM1*	Body
cg18068140	6	18,123,164	9.03E-06	-0.221	-0.228				*NHLRC1*	TSS1500

cg07568233	16	7,382,520	0.009016	-0.221	-0.125				*A2BP1*	Body
cg09493966	16	51,165,636	0.030054	-0.221	-0.173					
cg10489672	1	117,296,494	0.000977	-0.221	-0.229				*CD2*	TSS1500
cg19282411	6	48,842,056	0.004365	-0.221	-0.223					
cg05919105	1	77,533,592	5.33E-07	-0.221	-0.217					
cg02681040	11	3,240,201	0.000967	-0.221	-0.143				*MRGPRG*	TSS200
cg24681654	7	31,555,516	6.77E-11	-0.221	-0.232				*CCDC129*	TSS1500
cg25042465	8	50,825,702	0.006298	-0.221	-0.156				*SNTG1*	5'UTR
cg03329572	1	157,523,253	0.016204	-0.221	-0.166				*FCRL5*	TSS1500
cg19770955	7	107,203,448	0.000307	-0.221	-0.180	2.19E-17	-0.135		*COG5*	Body
cg07760622	10	117,754,806	0.029421	-0.221	-0.214					
cg09546889	2	22,918,193	0.011463	-0.221	-0.131					
cg13150785	15	41,138,160	0.000138	-0.221	-0.224				*SPINT1*	Body
cg23457518	10	134,810,240	0.001845	-0.221	-0.129					
cg25551622	17	65,052,432	0.000159	-0.221	-0.226				*CACNG1*	3'UTR
cg13235825	11	107,574,836	0.001875	-0.221	-0.198					
cg23140050	14	97,673,803	0.037145	-0.221	-0.153					
cg12196692	5	41,071,405	0.045565	-0.221	-0.170				*HEATR7B2*	1stExon
cg18537027	5	64,601,816	0.000575	-0.221	-0.190				*ADAMTS6*	Body
cg24418182	7	97,841,447	0.035835	-0.221	-0.086				*BHLHA15*	TSS200
cg01515534	19	42,415,665	0.001457	-0.221	-0.154					
cg03787088	11	34,847,603	2.81E-05	-0.221	-0.190					
cg23367089	2	220,169,805	0.027935	-0.221	-0.180				*PTPRN*	Body
cg01651570	14	104,194,141	0.007774	-0.221	-0.210				*ZFYVE21*	Body
cg14043752	2	64,770,117	1.37E-05	-0.221	-0.224				*AFTPH*	5'UTR
cg08934318	1	44,583,886	5.19E-08	-0.221	-0.200				*KLF17*	TSS1500
cg17004733	1	55,682,652	6.38E-07	-0.221	-0.211					
cg02456804	1	166,039,858	0.004427	-0.221	-0.256				*FAM78B*	Body
cg11906285	5	23,315,719	0.019984	-0.221	-0.184					
cg01416657	1	205,392,241	1.52E-10	-0.221	-0.211				*LEMD1*	TSS1500
cg13392680	8	72,935,034	0.000483	-0.221	-0.243				*TRPA1*	3'UTR
cg15230460	8	11,989,691	0.006259	-0.221	-0.139				*FAM66D*	Body
cg10700114	4	189,956,937	0.027672	-0.221	-0.121					
cg16456980	11	70,714,131	0.004887	-0.221	-0.146				*SHANK2*	Body

cg04242070	5	1,749,101	0.007674	-0.221	-0.144					
cg23232188	3	121,556,543	0.000307	-0.221	-0.149				*EAF2*	Body
cg00473929	19	35,417,259	1.09E-10	-0.221	-0.198				*ZNF30*	TSS1500
cg01751245	2	65,593,761	2.68E-09	-0.221	-0.203				*SPRED2*	5'UTR
cg23840044	11	22,696,087	2.69E-08	-0.221	-0.210				*GAS2*	5'UTR
cg09509365	1	3,006,755	0.038219	-0.221	-0.045				*PRDM16*	Body
cg12512173	13	111,936,059	0.012269	-0.221	-0.225				*ARHGEF7*	Body
cg19086905	15	102,342,791	0.003827	-0.221	-0.166					
cg13580382	9	140,081,032	1.87E-06	-0.222	-0.205				*ANAPC2*	Body
cg27435799	X	3,030,635	0.023511	-0.222	-0.178				*ARSF*	3'UTR
cg17822978	15	72,073,485	2.83E-06	-0.222	-0.230				*THSD4*	3'UTR
cg06502755	2	84,517,950	0.04643	-0.222	-0.148				*LOC388965*	Body
cg15458801	1	209,254,540	0.029001	-0.222	-0.179					
cg13531735	3	42,923,116	0.013241	-0.222	-0.132					
cg03926846	4	184,678,574	0.000504	-0.222	-0.234					
cg18555746	11	18,725,854	0.005669	-0.222	-0.129				*IGSF22*	3'UTR
cg26961103	X	139,865,953	0.011648	-0.222	-0.213				*CDR1*	1stExon
cg10704680	11	2,064,747	0.000546	-0.222	-0.175					
cg11708358	6	150,183,516	1.73E-08	-0.222	-0.216				*LRP11*	Body
cg00347938	11	69,469,113	2.25E-05	-0.222	-0.218				*CCND1*	3'UTR
cg01534527	3	38,003,247	6.89E-11	-0.222	-0.213				*CTDSPL*	Body
cg16240319	10	127,590,074	2.7E-06	-0.222	-0.176				*FANK1*	Body
cg24013105	8	137,955,493	0.030771	-0.222	-0.162					
cg18870085	4	187,985,253	0.003474	-0.222	-0.189					
cg19931596	2	172,645,256	1.23E-05	-0.222	-0.203				*SLC25A12*	Body
cg09596722	2	232,525,772	0.032762	-0.222	-0.203					
cg25968149	3	174,255,312	0.00081	-0.222	-0.119					
cg06844719	16	9,326,393	0.046351	-0.222	-0.176					
cg00880562	3	153,838,126	1.21E-07	-0.222	-0.223				*SGEF*	TSS1500
cg02880679	17	49,295,615	7.48E-07	-0.222	-0.268				*MBTD1*	Body
cg02968890	2	3,281,036	0.02678	-0.222	-0.275				*TSSC1*	Body
cg12530994	10	5,136,782	0.001153	-0.222	-0.207				*AKR1C3*	Body
cg02055988	22	31,671,702	0.000331	-0.222	-0.216				*LIMK2*	Body
cg09374462	4	49,314,051	0.029185	-0.222	-0.117					

cg18827501	2	167,338,333	0.006771	-0.222	-0.124				*SCN7A*	5'UTR
cg00523067	6	154,440,289	0.002245	-0.222	-0.180				*OPRM1*	3'UTR
cg00918005	2	79,253,210	0.00221	-0.222	-0.186	0.0000313	-0.107		*REG3G*	5'UTR
cg19800856	14	104,645,554	0.011271	-0.222	-0.175				*KIF26A*	Body
cg19849728	4	111,536,767	0.034613	-0.222	-0.118					
cg00095431	2	67,775,781	8.21E-08	-0.222	-0.238					
cg10127415	X	26,210,523	0.013894	-0.222	-0.267	2.35E-13	-0.214		*MAGEB6*	TSS200
cg18722615	11	40,359,642	0.001634	-0.222	-0.181					
cg06599514	16	86,328,711	0.008436	-0.222	-0.166					
cg14229404	9	107,894,426	0.000336	-0.222	-0.146					
cg15823330	1	3,207,539	0.040139	-0.222	-0.048				*PRDM16*	Body
cg01170129	2	110,448,269	7.06E-08	-0.222	-0.202					
cg12555810	12	119,187,794	0.006973	-0.222	-0.149					
cg26393791	2	16,180,066	0.000835	-0.222	-0.256					
cg11018432	12	124,832,427	0.018331	-0.222	-0.124				*NCOR2*	Body
cg21143109	15	93,851,183	0.019886	-0.222	-0.104					
cg23895722	11	128,776,112	2.17E-09	-0.222	-0.214				*C11orf45*	TSS1500
cg24717078	11	126,744,469	0.048612	-0.222	-0.234				*KIRREL3*	Body
cg26223968	14	101,204,240	0.025153	-0.222	-0.174			Yes		
cg10931853	12	34,548,115	9.55E-06	-0.222	-0.215					
cg03880506	12	132,638,107	0.007745	-0.222	-0.152					
cg17959416	19	22,987,826	0.005036	-0.222	-0.164					
cg23338408	14	31,289,523	0.000909	-0.222	-0.154					
cg00920668	1	207,991,122	0.037199	-0.222	-0.086				*LOC148696*	TSS1500
cg09535475	2	42,444,827	0.000637	-0.222	-0.252				*EML4*	Body
cg01886855	21	43,786,997	1.04E-06	-0.222	-0.215				*TFF1*	TSS1500
cg03898365	11	6,897,491	0.004629	-0.222	-0.252	0.00000167	-0.131		*OR10A4*	TSS1500
cg04514206	20	4,576,647	3.99E-05	-0.222	-0.202					
cg12690127	12	7,055,683	4.25E-29	-0.222	-0.222				*PTPN6*	TSS200
cg26208159	11	48,266,856	0.002192	-0.222	-0.165				*OR4X2*	1stExon
cg20849957	1	2,820,995	0.035135	-0.222	-0.070					
cg00789143	14	98,013,058	0.02153	-0.222	-0.150					
cg02153208	15	57,589,160	9.5E-07	-0.222	-0.205					
cg03374976	1	205,630,722	1.33E-08	-0.222	-0.177				*SLC45A3*	Body

cg02966841	2	1,926,366	0.008761	-0.222	-0.178				*MYT1L*	Body
cg08408740	5	52,558,413	0.000363	-0.222	-0.174					
cg18715189	19	57,631,405	0.036768	-0.222	-0.093				*USP29*	TSS200
cg02179478	3	35,785,377	0.026353	-0.222	-0.187				*MIR128-2*	TSS1500
cg09144520	10	134,842,984	1.48E-08	-0.222	-0.195					
cg09873772	16	4,896,197	1.36E-16	-0.222	-0.219				*GLYR1*	Body
cg18384794	5	148,509,515	1.19E-06	-0.222	-0.198					
cg05544940	6	33,170,339	0.000172	-0.222	-0.253				*SLC39A7*	Body
cg10087556	7	116,232,819	1.08E-05	-0.222	-0.244					
cg20299572	11	1,750,763	0.020317	-0.222	-0.187				*HCCA2*	Body
cg07056967	11	68,176,220	1.34E-05	-0.222	-0.217				*LRP5*	Body
cg19227053	20	60,116,795	0.014542	-0.222	-0.130				*CDH4*	Body
cg25326319	7	1,733,132	5.8E-10	-0.222	-0.215					
cg12380772	9	34,374,990	0.0017	-0.222	-0.201				*KIAA1161*	5'UTR
cg15855924	13	111,565,111	4.44E-05	-0.222	-0.218				*ANKRD10*	Body
cg14244402	9	130,681,102	2.99E-06	-0.222	-0.203					
cg17769229	10	83,639,690	0.003213	-0.222	-0.154				*NRG3*	Body
cg09251492	1	3,701,070	0.002855	-0.222	-0.127				*LRRC47*	Body
cg17276450	1	23,044,353	0.019977	-0.222	-0.259				*EPHB2*	Body
cg22972187	4	155,512,766	0.00041	-0.222	-0.160				*FGA*	TSS1500
cg09851241	3	186,731,798	0.024456	-0.222	-0.185				*ST6GAL1*	5'UTR
cg11407198	19	5,047,591	3.84E-07	-0.222	-0.197				*KDM4B*	Body
cg16857852	X	115,567,695	3.14E-09	-0.222	-0.244				*SLC6A14*	TSS200
cg04063339	8	56,545,297	0.028729	-0.222	-0.168					
cg21805791	7	1,581,258	1.01E-05	-0.222	-0.147				*MAFK*	3'UTR
cg24432768	1	158,149,974	0.004819	-0.222	-0.276				*CD1D*	5'UTR
cg02878382	14	104,665,702	1.48E-05	-0.222	-0.186					
cg15078211	7	42,265,517	9.54E-08	-0.222	-0.234				*GLI3*	5'UTR
cg17983632	6	1,621,930	6.03E-05	-0.222	-0.186					
cg02505681	19	39,615,285	1.52E-05	-0.222	-0.146				*PAK4*	TSS1500
cg16432350	12	52,585,695	6.63E-06	-0.222	-0.191				*KRT80*	1stExon
cg19923622	8	2,793,577	0.020854	-0.222	-0.170				*CSMD1*	3'UTR
cg05937687	8	70,101,609	1.95E-05	-0.222	-0.259					
cg12079266	18	55,924,005	0.015651	-0.222	-0.156				*NEDD4L*	5'UTR

cg15018704	19	1,908,132	2.98E-06	-0.222	-0.234				*ADAT3*	5'UTR
cg09186101	17	80,197,062	4.15E-07	-0.222	-0.238				*SLC16A3*	3'UTR
cg24365121	11	1,860,114	0.012077	-0.222	-0.143				*TNNI2*	TSS1500
cg23242649	20	34,196,465	0.000606	-0.222	-0.188				*FER1L4*	TSS1500
cg25370836	6	32,175,001	0.001085	-0.222	-0.124				*NOTCH4*	Body
cg21094613	11	78,900,687	8.28E-05	-0.222	-0.223				*ODZ4*	5'UTR
cg12078857	13	79,845,094	0.000241	-0.222	-0.249					
cg08898349	10	116,697,070	0.003697	-0.222	-0.181				*TRUB1*	TSS1500
cg12339328	20	18,491,484	0.004209	-0.223	-0.104				*SEC23B*	Body
cg07737566	7	50,847,263	0.010679	-0.223	-0.250				*GRB10*	5'UTR
cg14780990	15	93,181,182	2.1E-06	-0.223	-0.196				*FAM174B*	Body
cg16746369	16	51,192,174	0.011552	-0.223	-0.162					
cg05627696	4	16,933,239	0.001886	-0.223	-0.172					
cg07668671	12	113,305,756	1.07E-06	-0.223	-0.216				*RPH3A*	Body
cg11168687	4	185,726,782	0.000109	-0.223	-0.205				*ACSL1*	5'UTR
cg00237825	20	29,844,575	0.006781	-0.223	-0.186				*DEFB115*	TSS1500
cg05713044	5	16,671,496	0.000246	-0.223	-0.228				*MYO10*	Body
cg06747288	1	31,247,617	0.000481	-0.223	-0.136					
cg12929160	13	77,030,645	0.041212	-0.223	-0.209					
cg20789620	19	4,304,334	2.08E-07	-0.223	-0.234	6.14E-21	-0.151		*FSD1*	TSS1500
cg24897291	6	136,910,199	1.89E-05	-0.223	-0.189				*MAP3K5*	Body
cg03132551	7	55,411,178	0.000329	-0.223	-0.165					
cg12426196	1	161,008,826	2.37E-28	-0.223	-0.231				*TSTD1*	TSS200
cg21505231	6	30,984,668	8.32E-10	-0.223	-0.166					
cg07622201	7	49,929,836	0.019096	-0.223	-0.186				*VWC2*	Body
cg08650961	10	104,748,594	5.07E-06	-0.223	-0.199				*CNNM2*	Body
cg10909192	14	45,579,847	0.000137	-0.223	-0.224				*PRPF39*	Body
cg17079788	7	57,878,893	0.004605	-0.223	-0.191					
cg25093503	5	174,163,341	0.000832	-0.223	-0.157					
cg05538118	16	32,493,059	0.000848	-0.223	-0.231			Yes		
cg20983531	7	21,986,672	6.44E-08	-0.223	-0.233				*CDCA7L*	TSS1500
cg10694598	7	95,957,175	1.28E-08	-0.223	-0.210					
cg18414381	11	34,642,885	6.86E-09	-0.223	-0.213	0.00886177	-0.056		*EHF*	5'UTR
cg00300969	20	31,366,486	0.000228	-0.223	-0.161				*DNMT3B*	5'UTR

cg06029905	11	2,155,521	0.005594	-0.223	-0.123				*INS-IGF2*	Body
cg17275991	19	58,003,312	0.00401	-0.223	-0.093				*ZNF419*	Body
cg23260554	1	2,934,497	0.005756	-0.223	-0.099					
cg07371757	20	45,437,086	0.003569	-0.223	-0.161					
cg22971594	14	23,442,770	5.87E-06	-0.223	-0.163				*JUB*	Body
cg04498801	4	2,932,447	5.97E-07	-0.223	-0.232				*MFSD10*	3'UTR
cg02489219	2	197,676,272	1.11E-08	-0.223	-0.204				*C2orf66*	TSS1500
cg09492832	17	32,966,466	0.003605	-0.223	-0.130					
cg20250343	2	92,160,107	0.000846	-0.223	-0.181					
cg15076179	2	113,804,820	2.11E-10	-0.223	-0.217				*IL1F8*	5'UTR
cg15732053	X	47,662,641	3.45E-11	-0.223	-0.219					
cg08675521	11	18,716,666	7.16E-07	-0.223	-0.200					
cg21536086	1	154,166,546	0.000131	-0.223	-0.160				*MIR190B*	TSS1500
cg09136052	7	95,546,508	4.28E-05	-0.223	-0.270				*DYNC1I1*	Body
cg02449975	1	165,571,280	0.000123	-0.223	-0.256					
cg06362052	12	37,940,985	0.000208	-0.223	-0.216					
cg15781873	6	156,077,514	0.000991	-0.223	-0.175					
cg23117999	14	96,508,302	0.002305	-0.223	-0.141				*C14orf132*	Body
cg09163412	10	133,644,714	0.022205	-0.223	-0.090					
cg19949697	4	133,902,873	0.017771	-0.223	-0.181					
cg26080545	10	15,174,924	0.000397	-0.223	-0.234				*NMT2*	Body
cg01024458	1	8,703,816	1.17E-07	-0.223	-0.217				*RERE*	Body
cg01338296	7	129,556,606	7.06E-05	-0.223	-0.206				*UBE2H*	Body
cg02141358	8	57,228,615	0.004741	-0.223	-0.185				*SDR16C5*	Body
cg19121056	3	62,864,393	1.26E-06	-0.223	-0.179					
cg21312929	8	72,111,329	0.007577	-0.223	-0.202				*EYA1*	3'UTR
cg00492070	3	107,810,716	5.69E-06	-0.223	-0.236				*CD47*	TSS1500
cg01466011	3	156,837,590	4.07E-06	-0.223	-0.231					
cg22702857	12	4,269,985	0.022802	-0.223	-0.126					
cg09438085	X	52,087,706	0.006306	-0.223	-0.212					
cg27383651	6	28,888,809	1.34E-05	-0.223	-0.172				*TRIM27*	Body
cg01240823	15	92,667,819	0.029901	-0.223	-0.137				*SLCO3A1*	Body
cg01432087	11	67,242,162	1.15E-08	-0.223	-0.210					
cg04596544	5	33,997,325	0.002891	-0.223	-0.151				*AMACR*	Body

cg16134254	13	39,181,323	0.036827	-0.223	-0.170					
cg00320453	11	123,756,564	0.023582	-0.223	-0.126				*TMEM225*	TSS1500
cg24530116	10	81,904,331	1.03E-13	-0.223	-0.181				*PLAC9*	Body
cg01800148	1	9,130,886	2.56E-05	-0.223	-0.214				*SLC2A5*	TSS1500
cg21899558	7	661,634	5.97E-05	-0.223	-0.186				*PRKAR1B*	Body
cg15446520	4	120,930,379	0.011637	-0.223	-0.173					
cg20469680	7	135,458,419	9.54E-06	-0.223	-0.216					
cg21664614	13	111,277,611	0.023343	-0.223	-0.139				*CARKD*	Body
cg26343358	6	31,275,666	0.032177	-0.223	-0.246					
cg14321918	3	168,526,492	0.019575	-0.223	-0.113				*C3orf50*	Body
cg26622634	10	117,886,935	8.25E-05	-0.223	-0.208				*GFRA1*	Body
cg16636682	16	33,781,365	0.003471	-0.223	-0.196					
cg05155809	7	62,562,718	0.004198	-0.223	-0.182					
cg11387248	19	43,269,877	0.028676	-0.223	-0.268				*PSG8*	TSS200
cg16284238	6	87,196,595	0.000819	-0.223	-0.282					
cg04239863	16	87,093,670	0.039505	-0.223	-0.125					
cg01067983	6	2,961,157	2.5E-07	-0.223	-0.188				*SERPINB6*	5'UTR
cg03607573	11	93,471,889	4.78E-09	-0.223	-0.192				*TAF1D*	Body
cg18674460	6	32,169,650	0.000264	-0.223	-0.141				*NOTCH4*	Body
cg00526273	20	59,864,749	0.011886	-0.223	-0.115				*CDH4*	Body
cg04428071	17	80,225,377	0.03259	-0.223	-0.044				*CSNK1D*	Body
cg26427827	1	10,713,944	0.004765	-0.223	-0.149				*CASZ1*	Body
cg15195541	8	4,188,646	0.026831	-0.223	-0.132				*CSMD1*	Body
cg07959747	10	82,050,672	9.41E-08	-0.223	-0.208				*MAT1A*	TSS1500
cg08293623	11	40,803,410	0.000375	-0.223	-0.216					
cg12710637	1	2,918,373	0.026532	-0.223	-0.096					
cg27039467	16	1,730,561	0.049567	-0.223	-0.250				*HN1L*	Body
cg13665853	9	34,689,585	4.32E-05	-0.223	-0.208				*CCL19*	3'UTR
cg07151443	6	32,920,962	8.11E-06	-0.223	-0.216				*HLA-DMA*	TSS200
cg15557758	1	13,828,828	0.001474	-0.223	-0.088					
cg20793897	2	200,025,325	0.037083	-0.223	-0.165					
cg09418673	11	76,372,128	0.000159	-0.223	-0.214				*LRRC32*	Body
cg10581876	1	205,645,246	0.003305	-0.223	-0.161				*SLC45A3*	5'UTR
cg26034799	9	5,185,804	2.81E-05	-0.223	-0.181				*INSL6*	TSS200

cg05453893	13	56,169,684	0.035618	-0.223	-0.149					
cg25195987	7	31,125,925	0.042031	-0.223	-0.118				*ADCYAP1R1*	Body
cg08697797	2	234,668,834	1.01E-07	-0.223	-0.183				*UGT1A10*	Body
cg23075771	1	64,188,741	0.003823	-0.223	-0.141					
cg05265703	3	137,787,902	1.58E-07	-0.223	-0.205				*DZIP1L*	Body
cg18414021	14	100,069,535	2.3E-05	-0.223	-0.147				*CCDC85C*	1stExon
cg18080370	3	62,305,486	3.63E-05	-0.224	-0.223				*C3orf14*	1stExon
cg22882121	6	31,842,909	2.49E-08	-0.224	-0.240				*SLC44A4*	Body
cg19011552	X	146,364,725	9.01E-08	-0.224	-0.214				*MIR514-2*	TSS1500
cg00180447	3	126,260,260	2.99E-06	-0.224	-0.253				*CHST13*	Body
cg02745398	4	9,445,158	0.004765	-0.224	-0.179				*DEFB131*	TSS1500
cg00326902	6	117,747,649	0.00015	-0.224	-0.223				*ROS1*	TSS1500
cg22522402	X	152,402,356	0.003452	-0.224	-0.178					
cg03868978	21	42,537,294	1.47E-10	-0.224	-0.212					
cg16799191	3	112,395,154	0.002405	-0.224	-0.212					
cg24563501	11	67,442,408	3.06E-09	-0.224	-0.222				*ALDH3B2*	TSS1500
cg00583861	1	32,014,363	0.010347	-0.224	-0.152					
cg22665383	11	55,339,961	0.025438	-0.224	-0.216				*OR4C16*	1stExon
cg24882334	12	24,902,649	0.010786	-0.224	-0.261					
cg16587725	11	41,139,791	0.021908	-0.224	-0.217					
cg17570276	7	6,099,250	0.042918	-0.224	-0.344				*EIF2AK1*	TSS1500
cg16166230	17	32,480,663	1.93E-08	-0.224	-0.208				*ACCN1*	Body
cg05505103	11	109,785,847	0.007107	-0.224	-0.183					
cg03035538	1	75,139,958	0.046494	-0.224	-0.117				*C1orf173*	TSS1500
cg12473257	20	56,239,669	1.03E-05	-0.224	-0.244				*PMEPA1*	5'UTR
cg13560914	1	9,565,530	6.9E-07	-0.224	-0.215					
cg16225663	6	32,026,797	0.013957	-0.224	-0.166				*TNXB*	Body
cg13371910	6	69,990,372	0.033465	-0.224	-0.245				*BAI3*	Body
cg15527308	18	54,813,067	0.000772	-0.224	-0.167					
cg16587174	6	31,778,842	0.004884	-0.224	-0.161				*HSPA1L*	Body
cg07045803	4	160,125,667	3.43E-08	-0.224	-0.204					
cg22712883	13	31,579,374	0.038546	-0.224	-0.065					
cg13094583	17	21,831,674	0.018951	-0.224	-0.132					
cg19415202	11	133,706,356	0.005008	-0.224	-0.128					

cg16727072	4	8,646,957	0.007753	-0.224	-0.093					
cg26601187	X	110,653,460	0.006897	-0.224	-0.170				*DCX*	Body
cg27561786	1	8,525,010	5.38E-07	-0.224	-0.208				*RERE*	Body
cg24373608	3	103,645,997	0.007413	-0.224	-0.177					
cg10408178	14	72,219,272	0.029222	-0.224	-0.097					
cg16802159	16	86,457,719	0.000403	-0.224	-0.177					
cg04948906	11	124,413,229	0.001454	-0.224	-0.235				*OR8B12*	1stExon
cg15128334	3	185,215,532	2.01E-11	-0.224	-0.204				*TMEM41A*	Body
cg21359747	15	101,420,636	4.69E-08	-0.224	-0.231	0.00153659	-0.081		*ALDH1A3*	Body
cg18512262	11	6,049,729	0.017863	-0.224	-0.235				*OR56A1*	TSS1500
cg12011596	19	22,442,461	0.001341	-0.224	-0.173					
cg05085729	3	109,213,358	1.76E-06	-0.224	-0.255				*FLJ25363*	3'UTR
cg13805793	10	54,515,952	0.012831	-0.224	-0.233					
cg22578425	7	115,456,904	0.019797	-0.224	-0.249					
cg13197551	20	60,709,957	7.34E-06	-0.224	-0.222				*LSM14B*	3'UTR
cg15496763	6	32,711,102	0.030378	-0.224	-0.098				*HLA-DQA2*	Body
cg20989454	11	613,478	3.68E-08	-0.224	-0.240				*IRF7*	Body
cg24069653	5	169,408,301	1.09E-07	-0.224	-0.198				*FAM196B*	TSS1500
cg10095968	21	45,847,903	0.000492	-0.224	-0.146				*TRPM2*	Body
cg10910685	4	46,911,519	0.038824	-0.224	-0.103				*COX7B2*	TSS1500
cg17057663	5	4,863,806	0.039355	-0.224	-0.107					
cg21871338	14	87,262,857	0.005193	-0.224	-0.183					
cg12603671	1	71,418,388	0.000408	-0.224	-0.154				*PTGER3*	Body
cg24181605	15	62,458,847	5.46E-05	-0.224	-0.220				*C2CD4B*	TSS1500
cg26666538	2	115,021,229	0.015579	-0.224	-0.272					
cg03579446	8	126,216,633	4.03E-09	-0.224	-0.223				*NSMCE2*	Body
cg14022090	11	34,642,303	1.47E-05	-0.224	-0.220				*EHF*	TSS1500
cg16372051	6	31,794,302	8.36E-17	-0.224	-0.225				*HSPA1B*	TSS1500
cg26639085	19	54,972,720	2.1E-06	-0.224	-0.190				*LENG8*	3'UTR
cg22630918	10	102,748,988	0.045657	-0.224	-0.154				*C10orf2*	1stExon
cg20611039	2	168,121,101	0.000382	-0.224	-0.191					
cg22186380	6	132,940,093	0.026456	-0.224	-0.131				*TAAR2*	Body
cg00968640	1	185,834,094	0.000862	-0.224	-0.156				*HMCN1*	Body
cg20141817	1	153,124,825	0.024692	-0.224	-0.174				*SPRR2G*	TSS1500

cg02980055	8	47,866,183	0.000595	-0.224	-0.192					
cg07283011	3	133,970,158	5.41E-10	-0.224	-0.235				*RYK*	TSS1500
cg08321867	13	112,857,019	0.02201	-0.224	-0.067					
cg14386668	17	3,337,334	0.003552	-0.224	-0.122				*OR1E2*	TSS200
cg26450574	6	32,944,067	1.16E-13	-0.224	-0.230				*BRD2*	Body
cg01534808	11	128,578,726	0.001036	-0.224	-0.122				*FLI1*	Body
cg08081485	2	242,617,771	1.38E-06	-0.224	-0.229				*DTYMK*	Body
cg12843489	17	35,061,545	1.91E-06	-0.224	-0.178					
cg15948795	4	7,968,678	0.006643	-0.224	-0.240				*ABLIM2*	3'UTR
cg13472610	21	40,174,791	4.79E-05	-0.224	-0.194					
cg17797815	10	119,806,972	9.62E-07	-0.224	-0.261				*CASC2*	Body
cg21861953	13	29,158,391	0.048355	-0.224	-0.123					
cg24802813	7	38,389,991	9.21E-05	-0.224	-0.146					
cg05406749	11	123,495,671	0.010382	-0.225	-0.208					
cg05431947	11	12,621,248	0.005932	-0.225	-0.118					
cg12583394	5	101,827,323	0.030224	-0.225	-0.083				*SLCO6A1*	Body
cg16113347	14	93,899,367	4.76E-05	-0.225	-0.197				*KIAA1409*	5'UTR
cg25621991	5	142,942,670	1.05E-08	-0.225	-0.167					
cg25123362	10	133,793,734	3.96E-05	-0.225	-0.209				*BNIP3*	Body
cg05384663	1	157,671,386	0.007358	-0.225	-0.191				*FCRL3*	TSS1500
cg18066206	22	46,459,890	2.96E-06	-0.225	-0.225					
cg00061185	12	50,596,347	0.000181	-0.225	-0.229				*LIMA1*	Body
cg10186254	14	80,267,501	3.11E-06	-0.225	-0.173				*NRXN3*	Body
cg00146928	12	9,269,671	0.024613	-0.225	-0.099				*A2M*	TSS1500
cg12810264	10	43,329,092	0.010316	-0.225	-0.200				*BMS1*	3'UTR
cg16986661	2	65,866,869	3.55E-12	-0.225	-0.224					
cg20260619	14	54,449,398	0.021339	-0.225	-0.357					
cg27259633	16	19,534,852	0.007339	-0.225	-0.169				*GDE1*	TSS1500
cg14771419	7	142,012,988	0.035397	-0.225	-0.290					
cg04627863	2	234,741,670	0.011675	-0.225	-0.239					
cg19616783	10	1,063,172	3.55E-05	-0.225	-0.242				*GTPBP4*	3'UTR
cg05667197	7	142,828,903	0.000853	-0.225	-0.162				*PIP*	TSS1500
cg09643350	6	66,498,032	0.031804	-0.225	-0.196				*MCART3P*	Body
cg19389370	11	10,161,484	0.013176	-0.225	-0.126				*SBF2*	Body

cg14321066	3	168,115,836	0.003481	-0.225	-0.130				*C3orf50*	Body
cg26283480	3	129,252,584	3.21E-05	-0.225	-0.170				*RHO*	3'UTR
cg14654811	1	158,550,705	0.034897	-0.225	-0.118				*OR10X1*	TSS1500
cg09277566	22	50,017,428	0.007976	-0.225	-0.137				*C22orf34*	Body
cg24467326	10	9,646,929	0.018028	-0.225	-0.193					
cg03160217	2	117,814,299	0.016594	-0.225	-0.177					
cg06219695	16	434,256	3.64E-08	-0.225	-0.205				*LOC100134368*	Body
cg15825510	7	97,882,260	0.000112	-0.225	-0.187				*TECPR1*	TSS1500
cg23360101	8	858,591	0.000101	-0.225	-0.189					
cg24924502	17	79,109,777	0.029554	-0.225	-0.106				*AATK*	Body
cg13847226	4	7,193,237	0.001917	-0.225	-0.159				*SORCS2*	TSS1500
cg16714933	12	34,529,386	1.27E-07	-0.225	-0.242					
cg08484337	6	31,590,563	1.27E-06	-0.225	-0.219				*SNORA38*	TSS1500
cg16955318	11	124,056,323	0.01878	-0.225	-0.171					
cg20998200	11	22,370,099	1.01E-05	-0.225	-0.231			Yes	*SLC17A6*	Body
cg05078644	13	101,910,857	0.025584	-0.225	-0.148				*NALCN*	Body
cg15642035	8	139,165,052	0.01026	-0.225	-0.181	0.00135878	-0.107		*FAM135B*	Body
cg18186343	14	101,317,620	0.001625	-0.225	-0.190				*MIR770*	TSS1500
cg14286514	9	32,525,315	3.44E-05	-0.225	-0.165				*DDX58*	Body
cg26277556	14	54,507,223	0.014357	-0.225	-0.312			Yes		
cg01960885	18	24,765,781	5.55E-07	-0.225	-0.190				*CHST9*	TSS1500
cg00861010	1	41,966,113	4.75E-05	-0.225	-0.224					
cg01889461	7	34,606,530	0.008663	-0.225	-0.158					
cg16916688	6	34,101,441	0.001434	-0.225	-0.160				*GRM4*	1stExon
cg22366943	6	124,882,293	0.005946	-0.225	-0.178				*NKAIN2*	Body
cg08014182	6	33,241,594	1.27E-09	-0.225	-0.188				*RPS18*	Body
cg15910070	11	101,681,939	0.020679	-0.225	-0.222					
cg21173150	12	53,321,588	0.000266	-0.225	-0.143					
cg26540441	2	236,120,905	0.000423	-0.225	-0.184					
cg02981942	16	54,574,605	3.5E-07	-0.225	-0.190					
cg04366343	4	16,819,676	0.002654	-0.225	-0.149				*LDB2*	Body
cg10567744	10	130,829,670	6.29E-07	-0.225	-0.193					
cg11440666	11	75,115,402	0.000523	-0.225	-0.243				*RPS3*	Body
cg26703245	11	131,460,647	0.032574	-0.225	-0.141				*NTM*	Body

cg03400410	1	198,745,486	0.00142	-0.225	-0.126					
cg16223358	10	86,372,858	1.58E-08	-0.225	-0.218					
cg17331569	1	160,788,135	0.002134	-0.225	-0.270				*LY9*	Body
cg10467926	7	57,090,465	0.015451	-0.225	-0.176					
cg10808936	1	159,173,216	0.013966	-0.225	-0.151				*DARC*	TSS1500
cg00038857	17	32,645,753	0.000231	-0.225	-0.213				*CCL8*	TSS1500
cg15756839	6	57,972,349	0.049416	-0.225	-0.115					
cg11172483	11	78,900,896	0.00022	-0.225	-0.225				*ODZ4*	5'UTR
cg11232156	1	158,035,207	0.003398	-0.225	-0.161				*KIRREL*	Body
cg13399773	20	34,700,112	6.16E-06	-0.225	-0.232				*EPB41L1*	TSS1500
cg17184255	1	12,304,762	1.95E-05	-0.225	-0.216				*VPS13D*	Body
cg22724854	11	55,737,072	0.005532	-0.225	-0.214				*OR10AG1*	TSS1500
cg05431942	11	6,866,629	0.012983	-0.225	-0.226				*OR10A5*	TSS1500
cg06361127	1	182,024,689	0.000365	-0.225	-0.214				*ZNF648*	3'UTR
cg11842367	1	78,703,580	3.69E-07	-0.225	-0.191				*MGC27382*	Body
cg14401837	7	34,697,493	0.036862	-0.225	-0.191	3.48E-10	-0.174		*NPSR1*	TSS1500
cg06994787	16	11,834,960	1.28E-06	-0.225	-0.281				*TXNDC11*	Body
cg09933323	1	214,832,208	0.00568	-0.225	-0.251				*CENPF*	Body
cg21715189	2	38,304,802	0.000319	-0.225	-0.240				*CYP1B1*	TSS1500
cg09799307	17	80,046,218	0.009542	-0.225	-0.124				*FASN*	Body
cg15139163	3	11,408,424	3.83E-07	-0.225	-0.193				*ATG7*	Body
cg11620557	X	39,963,545	0.004658	-0.225	-0.284				*BCOR*	5'UTR
cg00616000	7	6,521,599	1.77E-09	-0.225	-0.221				*KDELR2*	Body
cg20146904	1	192,216,653	0.006682	-0.225	-0.140					
cg06772202	7	75,678,527	2.72E-08	-0.225	-0.226				*MDH2*	Body
cg26746309	10	101,943,528	2.65E-07	-0.225	-0.184				*ERLIN1*	Body
cg01039849	5	110,816,428	4.69E-06	-0.225	-0.208				*CAMK4*	Body
cg02464901	2	188,851,274	7.99E-06	-0.225	-0.216					
cg18482892	3	56,833,426	1.45E-17	-0.225	-0.217				*ARHGEF3*	Body
cg03975156	11	7,961,516	0.002066	-0.225	-0.178				*OR10A3*	TSS1500
cg09385306	1	85,724,328	2.23E-06	-0.225	-0.147				*C1orf52*	Body
cg00190034	8	38,584,636	0.009911	-0.225	-0.147				*TACC1*	TSS1500
cg01094891	19	31,048,117	0.005141	-0.225	-0.165				*ZNF536*	3'UTR
cg14024893	9	139,943,146	0.011895	-0.225	-0.124				*ENTPD2*	Body

cg02073887	15	96,531,851	2.5E-07	-0.225	-0.233					
cg12663426	1	155,115,785	0.028012	-0.225	-0.188					
cg16384355	7	157,320,516	0.031206	-0.225	-0.075					
cg16401608	2	220,523,857	0.030072	-0.225	-0.207					
cg09424271	X	147,586,050	0.003993	-0.225	-0.203				*AFF2*	Body
cg14343414	6	31,025,967	0.010204	-0.225	-0.143				*HCG22*	Body
cg05670717	21	46,368,052	5.62E-06	-0.225	-0.193				*C21orf70*	Body
cg07592513	19	49,994,239	9.73E-07	-0.225	-0.188				*SNORD35A*	TSS200
cg23793965	8	91,841,347	4E-05	-0.225	-0.256				*NECAB1*	Body
cg11288686	12	29,735,807	4.98E-10	-0.225	-0.212				*TMTC1*	Body
cg22479532	4	62,450,110	0.009904	-0.225	-0.151				*LPHN3*	Body
cg10956860	5	32,175,561	3.12E-12	-0.225	-0.252				*GOLPH3*	TSS1500
cg24630825	4	176,909,979	1.45E-06	-0.225	-0.240				*GPM6A*	5'UTR
cg23323297	19	51,195,418	0.002723	-0.225	-0.225				*SHANK1*	Body
cg21968796	21	46,358,532	1.95E-05	-0.225	-0.219	0.01592102	-0.064		*C21orf67*	Body
cg23406509	17	31,479,452	0.003361	-0.225	-0.152				*ACCN1*	Body
cg11214047	7	92,761,722	4.48E-05	-0.225	-0.182				*SAMD9L*	Body
cg04084173	1	48,458,049	0.014275	-0.225	-0.119					
cg00170459	4	136,509,310	0.000708	-0.226	-0.154					
cg06817269	15	59,909,035	0.005155	-0.226	-0.274	1.22E-09	-0.157		*GCNT3*	5'UTR
cg10717610	2	394,242	0.00045	-0.226	-0.261					
cg18640514	7	53,664,246	0.002657	-0.226	-0.199					
cg05209306	19	39,899,376	2.6E-09	-0.226	-0.190				*ZFP36*	3'UTR
cg07211251	5	72,989,417	6.36E-06	-0.226	-0.197				*RGNEF*	Body
cg04955791	6	152,959,047	0.006799	-0.226	-0.196				*SYNE1*	TSS1500
cg24884543	18	77,139,072	0.039279	-0.226	-0.155					
cg00532399	5	171,209,553	0.00226	-0.226	-0.156					
cg05285429	3	77,122,541	0.000006	-0.226	-0.214				*ROBO2*	Body
cg25920535	3	196,233,831	0.005474	-0.226	-0.143				*C3orf43*	3'UTR
cg02772823	X	115,567,582	4.5E-09	-0.226	-0.239				*SLC6A14*	TSS200
cg02678638	16	85,671,115	0.001211	-0.226	-0.218				*KIAA0182*	5'UTR
cg06872010	8	12,236,258	0.00311	-0.226	-0.161				*FAM66A*	Body
cg24423088	21	32,185,365	0.049407	-0.226	-0.081	0.00771579	-0.081		*KRTAP8-1*	1stExon
cg08247866	1	3,096,529	0.011231	-0.226	-0.111				*PRDM16*	Body

cg14347463	8	129,886,819	0.000618	-0.226	-0.202						
cg22164531	1	192,146,717	0.032905	-0.226	-0.126					*RGS18*	Body
cg23244540	2	127,532,388	0.012865	-0.226	-0.143						
cg01617018	16	30,116,135	0.002026	-0.226	-0.270					*GDPD3*	3'UTR
cg02571560	6	32,037,587	0.000993	-0.226	-0.158					*TNXB*	Body
cg23527409	19	44,956,065	0.017107	-0.226	-0.168						
cg17128024	8	2,196,833	0.003461	-0.226	-0.161						
cg23823833	3	159,075,576	0.000234	-0.226	-0.159					*SCHIP1*	Body
cg26403580	10	120,936,730	7.56E-07	-0.226	-0.221					*PRDX3*	Body
cg00043591	7	156,874,860	0.001387	-0.226	-0.166						
cg08486180	2	56,461,845	0.026819	-0.226	-0.259					*CCDC85A*	Body
cg05139761	1	4,842,529	6.97E-05	-0.226	-0.175					*AJAP1*	3'UTR
cg17385247	10	115,718,469	4.08E-11	-0.226	-0.222						
cg23276912	1	35,326,118	2.21E-12	-0.226	-0.229					*C1orf212*	TSS1500
cg11198968	10	83,676,596	0.025507	-0.226	-0.128					*NRG3*	Body
cg12114804	14	70,236,481	0.020526	-0.226	-0.192					*SFRS5*	Body
cg20738274	1	207,201,020	6.76E-12	-0.226	-0.229					*C1orf116*	5'UTR
cg06759874	4	186,960,904	4.12E-13	-0.226	-0.226						
cg13513349	4	175,851,061	0.014385	-0.226	-0.134					*ADAM29*	5'UTR
cg01763947	15	92,459,894	0.020005	-0.226	-0.189					*SLCO3A1*	Body
cg05988964	8	140,629,559	0.013133	-0.226	-0.131					*KCNK9*	3'UTR
cg10821813	10	129,562,528	0.030006	-0.226	-0.184						
cg09242541	1	10,490,025	1.48E-12	-0.226	-0.208	4E-21	-0.148			*APITD1*	TSS200
cg10138313	22	44,964,253	2.29E-06	-0.226	-0.172					*NCRNA00207*	TSS1500
cg14284630	11	56,018,532	2.75E-05	-0.226	-0.223					*OR5T3*	TSS1500
cg03992092	X	139,169,879	0.000281	-0.226	-0.221						
cg15023686	18	76,675,402	3.83E-06	-0.226	-0.175						
cg19532959	12	65,518,055	0.001688	-0.226	-0.191						
cg09059945	7	130,126,018	0.000619	-0.226	-0.239	0.01226872	-0.043			*MEST*	TSS200
cg02805690	1	155,895,456	0.006556	-0.226	-0.178					*KIAA0907*	Body
cg05638689	11	65,314,454	1.8E-17	-0.226	-0.248					*LTBP3*	Body
cg27094323	7	1,216,898	0.000454	-0.226	-0.149						
cg26680644	1	24,920,997	4.3E-06	-0.226	-0.218					*C1orf130*	5'UTR
cg01431057	1	153,362,927	0.000194	-0.226	-0.195					*S100A8*	Body

cg16960593	6	161,087,573	0.01057	-0.226	-0.220				*LPA*	TSS200
cg24995014	22	49,934,662	0.044846	-0.226	-0.070					
cg16393935	11	128,263,817	5.77E-09	-0.226	-0.219					
cg04530015	2	215,796,436	3.35E-07	-0.226	-0.215				*ABCA12*	3'UTR
cg06254768	4	114,064,176	3.24E-05	-0.226	-0.168				*ANK2*	Body
cg01397065	19	1,908,328	7.15E-05	-0.226	-0.238				*ADAT3*	5'UTR
cg03090436	6	71,121,859	0.002426	-0.226	-0.257				*FAM135A*	TSS1500
cg00111333	1	198,828,249	0.023005	-0.226	-0.094				*MIR181B1*	TSS200
cg02303399	1	153,175,248	0.001617	-0.226	-0.176				*LELP1*	TSS1500
cg14219111	18	13,826,671	0.000107	-0.226	-0.226				*MC5R*	1stExon
cg14321343	22	29,838,869	2.21E-06	-0.226	-0.159				*RFPL1S*	TSS1500
cg10089626	1	45,969,600	0.04438	-0.226	-0.122				*MMACHC*	Body
cg18183817	3	13,665,008	0.029048	-0.226	-0.268				*FBLN2*	Body
cg04522625	3	64,751,651	2.3E-08	-0.226	-0.225				*MIR548A2*	Body
cg10734581	14	101,520,078	0.004337	-0.226	-0.225				*MIR134*	TSS1500
cg18218190	1	201,982,372	0.008714	-0.226	-0.306				*ELF3*	Body
cg26626829	2	181,386,053	6.54E-11	-0.226	-0.213					
cg07008784	1	205,498,551	4.03E-10	-0.226	-0.205				*CDK18*	Body
cg09478995	11	57,264,045	3.29E-06	-0.226	-0.166				*SLC43A1*	Body
cg11010561	4	155,630,610	0.000702	-0.226	-0.150					
cg26605760	17	61,597,601	4.38E-09	-0.226	-0.219					
cg03330223	16	86,371,363	0.00253	-0.226	-0.197				*LOC732275*	Body
cg12277653	22	50,028,456	0.001506	-0.226	-0.141				*C22orf34*	Body
cg26486069	X	48,434,354	0.000117	-0.226	-0.228				*RBM3*	Body
cg00678892	2	53,543,334	0.008561	-0.226	-0.202					
cg08877257	16	29,820,592	1.6E-09	-0.226	-0.202				*MAZ*	Body
cg15029475	5	3,028,202	0.018592	-0.226	-0.076					
cg20035127	8	80,819,477	8.61E-05	-0.226	-0.246					
cg20314918	2	166,151,447	0.005082	-0.226	-0.164				*SCN2A*	5'UTR
cg09450260	7	155,193,250	0.00173	-0.226	-0.152					
cg21554588	5	138,720,378	0.001993	-0.226	-0.119				*SLC23A1*	TSS1500
cg00032643	1	3,700,680	0.003765	-0.226	-0.154				*LRRC47*	Body
cg17503259	17	32,966,081	0.000262	-0.226	-0.210				*TMEM132E*	3'UTR
cg00757453	X	17,986,297	2.08E-05	-0.226	-0.218					

cg10290988	11	1,673,766	0.035273	-0.226	-0.167				*HCCA2*	Body
cg17163527	1	3,447,348	1.73E-32	-0.226	-0.210				*MEGF6*	Body
cg00252594	14	102,974,737	7.77E-08	-0.226	-0.195				*ANKRD9*	5'UTR
cg02449336	7	143,805,356	1.08E-05	-0.226	-0.228				*OR2A2*	TSS1500
cg16784198	7	157,236,093	0.002298	-0.226	-0.147					
cg04850842	14	27,023,180	0.009747	-0.226	-0.109				*NOVA1*	Body
cg15766408	4	16,850,240	0.035392	-0.226	-0.186				*LDB2*	Body
cg23227851	3	42,633,273	1.83E-06	-0.226	-0.253				*SS18L2*	Body
cg05046083	7	111,425,428	0.000383	-0.226	-0.226				*DOCK4*	Body
cg10815253	2	116,925,524	0.002113	-0.226	-0.230			Yes		
cg02915215	8	134,467,165	3.07E-05	-0.226	-0.199				*ST3GAL1*	3'UTR
cg09412619	16	11,323,792	9.07E-05	-0.226	-0.249					
cg13978352	6	30,908,706	3.11E-05	-0.226	-0.154				*DPCR1*	TSS200
cg18099058	11	55,737,045	0.000242	-0.226	-0.236				*OR10AG1*	TSS1500
cg05859533	16	57,727,230	0.003034	-0.227	-0.223				*CCDC135*	TSS1500
cg11384084	12	132,045,365	0.027505	-0.227	-0.168					
cg18498565	10	3,173,604	0.007195	-0.227	-0.174				*PFKP*	Body
cg24351427	1	185,670,109	0.043348	-0.227	-0.126					
cg05385805	7	143,106,577	3.05E-05	-0.227	-0.196				*EPHA1*	TSS1500
cg20256009	1	153,363,020	8.64E-08	-0.227	-0.203				*S100A8*	5'UTR
cg03802982	16	65,089,255	0.000449	-0.227	-0.189				*CDH11*	5'UTR
cg15909393	2	364,075	0.026572	-0.227	-0.111					
cg18454510	6	40,363,184	0.000258	-0.227	-0.250				*LRFN2*	Body
cg24229750	11	5,531,283	0.025157	-0.227	-0.235				*UBQLN3*	TSS200
cg04508984	22	43,536,864	7.44E-05	-0.227	-0.211			Yes	*MCAT*	Body
cg09985615	4	58,291,304	0.005961	-0.227	-0.143					
cg16639860	4	20,885,019	5.28E-08	-0.227	-0.200				*KCNIP4*	Body
cg27092605	7	93,357,562	0.000259	-0.227	-0.224					
cg01079019	16	21,566,602	1.71E-08	-0.227	-0.171					
cg08084967	2	137,747,719	0.008419	-0.227	-0.185				*THSD7B*	TSS1500
cg01766699	2	196,602,679	7.91E-07	-0.227	-0.201				*DNAH7*	Body
cg23075139	3	115,524,031	0.0047	-0.227	-0.208				*LSAMP*	3'UTR
cg07874983	16	62,065,904	0.001218	-0.227	-0.154				*CDH8*	5'UTR
cg10666909	6	32,820,249	8.87E-07	-0.227	-0.249			Yes	*TAP1*	Body

cg15666275	19	57,274,897	0.031408	-0.227	-0.233					
cg22730864	1	3,161,448	6.92E-08	-0.227	-0.222				*PRDM16*	Body
cg03102729	X	120,181,504	0.006976	-0.227	-0.192				*GLUD2*	5'UTR
cg07774589	18	73,625,808	0.015301	-0.227	-0.129					
cg12848417	10	108,576,571	1.97E-07	-0.227	-0.216				*SORCS1*	Body
cg21143441	17	8,124,011	2.63E-10	-0.227	-0.228				*C17orf44*	Body
cg24562669	7	97,807,699	1.13E-06	-0.227	-0.235				*LMTK2*	Body
cg08070235	11	118,381,379	3.97E-07	-0.227	-0.205				*MLL*	Body
cg01882991	6	6,677,756	2.22E-11	-0.227	-0.186					
cg10627429	10	115,511,135	5.76E-07	-0.227	-0.251				*C10orf81*	TSS200
cg16821267	X	27,840,014	0.039027	-0.227	-0.166				*MAGEB10*	Body
cg00098750	16	23,727,847	0.007547	-0.227	-0.183					
cg27130075	13	110,935,325	0.029322	-0.227	-0.141				*COL4A1*	Body
cg01405985	19	36,345,675	1.52E-10	-0.227	-0.218					
cg15357118	2	128,927,972	1.02E-05	-0.227	-0.231				*UGGT1*	Body
cg10222734	10	52,612,019	0.004026	-0.227	-0.158				*A1CF*	5'UTR
cg13601993	9	127,534,760	4.15E-06	-0.227	-0.185				*NR6A1*	TSS1500
cg11410649	1	24,122,853	6.01E-05	-0.227	-0.201				*GALE*	Body
cg16987735	1	152,759,772	0.007366	-0.227	-0.227				*LCE1E*	5'UTR
cg24695977	1	171,640,387	5.75E-07	-0.227	-0.233					
cg27450167	2	135,272,843	3.38E-05	-0.227	-0.165				*TMEM163*	Body
cg06314702	11	104,501,066	0.005756	-0.227	-0.069					
cg14894216	11	65,794,352	0.001178	-0.227	-0.196				*CATSPER1*	TSS1500
cg14710459	10	15,906,284	4.35E-07	-0.227	-0.181					
cg03673470	1	238,055,429	6.21E-05	-0.227	-0.159	0.00035016	-0.068		*ZP4*	TSS1500
cg24881471	17	73,996,870	0.000179	-0.227	-0.132				*CDK3*	TSS200
cg25720825	1	2,849,682	5.72E-07	-0.227	-0.220					
cg20395040	13	23,310,225	0.046079	-0.227	-0.167					
cg10806586	14	45,366,116	2.97E-07	-0.227	-0.224				*C14orf28*	TSS1500
cg12297501	6	28,364,357	3.99E-07	-0.227	-0.270				*ZSCAN12*	Body
cg14240993	12	132,929,352	0.011388	-0.227	-0.104					
cg03078733	17	14,822,587	9.13E-06	-0.227	-0.201					
cg05763609	3	100,535,443	0.043099	-0.227	-0.120				*ABI3BP*	Body
cg14406134	9	117,014,922	3.74E-05	-0.227	-0.167				*COL27A1*	Body

cg02750034	15	84,320,596	9.87E-06	-0.227	-0.169					
cg04031344	10	130,829,645	6.16E-06	-0.227	-0.203					
cg25890048	11	55,703,443	0.021677	-0.227	-0.220	0.0000647	-0.117		*OR5I1*	1stExon
cg01021196	14	38,340,164	1.25E-05	-0.227	-0.227					
cg08342919	7	100,894,787	0.005626	-0.227	-0.212					
cg23240355	17	10,276,428	0.024294	-0.227	-0.117				*MYH13*	TSS200
cg26102295	7	1,423,622	0.036935	-0.227	-0.175					
cg09058554	1	9,600,721	8.87E-06	-0.227	-0.215				*SLC25A33*	Body
cg02486096	19	57,373,324	6.76E-09	-0.227	-0.223					
cg11384744	7	16,794,623	1.64E-07	-0.227	-0.236				*TSPAN13*	Body
cg17372758	10	73,976,798	0.000373	-0.227	-0.179				*ASCC1*	TSS1500
cg18899018	2	119,006,923	0.004638	-0.227	-0.222					
cg27232401	6	127,977,312	4.16E-05	-0.227	-0.219					
cg03399574	13	19,759,420	0.006031	-0.227	-0.141					
cg08704660	13	76,052,395	0.002307	-0.227	-0.214				*TBC1D4*	Body
cg08151470	1	10,092,299	2.27E-06	-0.227	-0.247	5.49E-10	-0.127		*UBE4B*	TSS1500
cg25649784	8	115,565,512	2.41E-05	-0.227	-0.248					
cg02674804	19	1,492,043	3.44E-05	-0.227	-0.256	1.87E-28	-0.231		*REEP6*	Body
cg16212298	19	51,051,954	2.27E-05	-0.227	-0.221				*LRRC4B*	Body
cg01565083	17	80,042,523	0.015521	-0.227	-0.092				*FASN*	Body
cg11574665	2	242,580,471	0.0067	-0.227	-0.125				*ATG4B*	Body
cg03696603	16	69,111,974	4.22E-06	-0.227	-0.248				*TMCO7*	Body
cg06741568	12	129,028,641	0.029642	-0.227	-0.130				*TMEM132C*	Body
cg24157349	22	47,081,751	9.7E-08	-0.227	-0.211				*CERK*	3'UTR
cg20097929	10	83,464,779	0.019054	-0.227	-0.121					
cg11643733	12	82,148,913	0.006855	-0.227	-0.136				*PPFIA2*	5'UTR
cg15552238	19	14,785,749	0.001792	-0.227	-0.201	6.13E-08	-0.106		*EMR3*	TSS200
cg23165226	6	31,511,287	3.99E-10	-0.227	-0.231				*BAT1*	TSS1500
cg24102479	1	149,259,342	0.002086	-0.227	-0.236					
cg04158612	16	1,157,349	0.048528	-0.227	-0.164					
cg18369680	20	41,984,014	0.039995	-0.227	-0.109					
cg24949632	4	47,032,678	0.002458	-0.227	-0.192				*GABRB1*	TSS1500
cg01560773	21	47,428,846	0.014922	-0.228	-0.143					
cg06596221	12	131,416,672	0.02599	-0.228	-0.096					

cg19241878	17	48,846,189	0.000144	-0.228	-0.184				*C17orf73*	TSS1500
cg05997059	7	116,411,780	2.79E-07	-0.228	-0.210				*MET*	Body
cg21537187	7	135,662,562	2.88E-07	-0.228	-0.198				*MTPN*	TSS1500
cg26014580	7	2,113,781	2.37E-07	-0.228	-0.221				*MAD1L1*	Body
cg17150898	12	1,702,116	1.22E-19	-0.228	-0.206				*FBXL14*	1stExon
cg07450292	3	76,419,070	0.01468	-0.228	-0.166					
cg00279755	11	103,584,918	0.017014	-0.228	-0.131					
cg13758913	18	24,283,684	1.78E-06	-0.228	-0.175				*LOC728606*	TSS200
cg06967998	11	132,527,438	0.049715	-0.228	-0.261				*OPCML*	Body
cg24082526	19	49,993,125	5.7E-17	-0.228	-0.224				*RPL13AP5*	Body
cg10626169	7	48,319,696	0.004213	-0.228	-0.128				*ABCA13*	Body
cg22349442	17	39,507,113	0.001363	-0.228	-0.160				*KRT33A*	TSS200
cg23090936	12	43,687,522	0.001252	-0.228	-0.180					
cg26516680	1	118,540,478	2.99E-07	-0.228	-0.224				*SPAG17*	Body
cg03764965	2	117,971,251	0.003631	-0.228	-0.256					
cg21571135	4	88,038,713	1.58E-06	-0.228	-0.234				*AFF1*	Body
cg24878051	1	3,305,115	0.000117	-0.228	-0.176				*PRDM16*	Body
cg03623568	16	6,915,990	0.016251	-0.228	-0.161				*A2BP1*	5'UTR
cg02075221	19	51,376,639	1.38E-11	-0.228	-0.218				*KLK2*	TSS200
cg26874764	7	63,667,212	0.001559	-0.228	-0.198				*ZNF735*	TSS1500
cg11345210	13	60,052,169	0.006894	-0.228	-0.162					
cg13860360	13	33,846,010	0.000814	-0.228	-0.268				*STARD13*	Body
cg01244043	20	1,448,437	0.000334	-0.228	-0.240				*NSFL1C*	TSS200
cg12819873	11	57,157,632	0.001882	-0.228	-0.250				*PRG2*	5'UTR
cg03513858	6	54,763,001	0.013054	-0.228	-0.183				*FAM83B*	Body
cg13932473	1	1,282,742	0.000191	-0.228	-0.172				*DVL1*	Body
cg17897699	8	137,806,993	0.004092	-0.228	-0.123					
cg01232206	16	33,044,270	0.001101	-0.228	-0.191					
cg03820608	11	131,547,319	0.022408	-0.228	-0.195				*NTM*	Body
cg08089153	11	1,956,292	1.3E-06	-0.228	-0.176				*TNNT3*	Body
cg12101256	1	196,547,408	0.002497	-0.228	-0.151				*KCNT2*	Body
cg03114584	4	1,534,311	0.003819	-0.228	-0.137					
cg11849327	10	76,682,695	0.008974	-0.228	-0.164				*MYST4*	Body
cg23648516	13	78,467,552	0.000101	-0.228	-0.248					

cg21592463	5	85,756,447	0.00488	-0.228	-0.140					
cg09467433	3	171,283,606	0.000351	-0.228	-0.271					
cg03842933	4	155,244,316	0.007744	-0.228	-0.151				*DCHS2*	Body
cg16931076	12	1,702,001	5.12E-30	-0.228	-0.205				*FBXL14*	Body
cg10076902	11	1,858,135	6.61E-08	-0.228	-0.230				*SYT8*	Body
cg11746464	2	137,748,833	5.36E-05	-0.228	-0.207				*THSD7B*	Body
cg15035476	19	46,457,107	0.018044	-0.228	-0.127				*NOVA2*	Body
cg04209766	5	114,512,608	1.82E-06	-0.228	-0.207				*TRIM36*	Body
cg16327839	11	133,715,264	0.019381	-0.228	-0.189				*SPATA19*	1stExon
cg20340146	6	160,172,821	4.5E-07	-0.228	-0.212				*WTAP*	Body
cg01080424	7	6,520,004	0.001123	-0.228	-0.190				*KDELR2*	Body
cg14931669	6	66,497,586	0.025743	-0.228	-0.204				*MCART3P*	TSS200
cg21156912	17	80,847,546	8.43E-05	-0.228	-0.220				*TBCD*	Body
cg04490596	8	32,452,181	0.002914	-0.228	-0.137				*NRG1*	Body
cg10143323	11	127,332,027	0.03029	-0.228	-0.231					
cg20255560	8	36,347,870	0.000368	-0.228	-0.190					
cg20910408	7	44,801,589	0.002171	-0.228	-0.181				*ZMIZ2*	Body
cg02493544	11	125,361,336	6.41E-05	-0.228	-0.217				*FEZ1*	5'UTR
cg17378966	5	172,196,746	7.71E-06	-0.228	-0.223				*DUSP1*	Body
cg21156756	14	32,866,773	1.36E-05	-0.228	-0.216				*AKAP6*	5'UTR
cg20845494	13	55,576,092	0.009599	-0.228	-0.213					
cg04926380	11	44,213,376	0.000327	-0.228	-0.300				*EXT2*	Body
cg02892650	16	67,380,234	5.15E-08	-0.228	-0.205				*LRRC36*	Body
cg09326204	3	121,953,249	2.02E-05	-0.228	-0.212				*CASR*	5'UTR
cg27046117	1	5,125,428	0.012896	-0.228	-0.098					
cg26930408	10	50,340,100	0.048527	-0.228	-0.186				*FAM170B*	Body
cg02967056	3	111,853,315	3.31E-06	-0.228	-0.239				*GCET2*	TSS1500
cg01951086	4	111,551,642	0.001509	-0.228	-0.269				*PITX2*	Body
cg03542322	20	21,227,215	0.000187	-0.228	-0.181				*PLK1S1*	3'UTR
cg09199250	11	2,191,147	8.3E-14	-0.228	-0.218				*TH*	Body
cg14887116	11	124,293,907	0.027913	-0.228	-0.233				*OR8B4*	1stExon
cg00143527	15	81,292,171	8.38E-12	-0.228	-0.231				*MESDC1*	TSS1500
cg24524694	10	52,829,575	0.002962	-0.228	-0.186				*PRKG1*	Body
cg05778080	17	21,832,243	0.000574	-0.228	-0.189					

cg05527267	17	1,472,819	9.31E-06	-0.228	-0.184					
cg23845250	12	132,944,590	6.24E-07	-0.228	-0.190					
cg24161057	1	161,008,811	4.92E-13	-0.228	-0.234				*TSTD1*	TSS200
cg02420480	7	41,919,892	0.044685	-0.228	-0.167					
cg25428846	4	141,107,931	3.37E-05	-0.228	-0.184					
cg17519477	10	83,848,597	0.001214	-0.229	-0.170				*NRG3*	Body
cg06711333	1	2,750,406	0.000597	-0.229	-0.214					
cg14795163	1	74,620,779	0.000504	-0.229	-0.155				*LRRIQ3*	Body
cg04824189	7	80,142,634	4.62E-07	-0.229	-0.235				*GNAT3*	TSS1500
cg02487712	10	134,733,396	0.008217	-0.229	-0.094					
cg06324909	16	33,574,151	0.000567	-0.229	-0.211					
cg06755296	15	98,199,925	0.000374	-0.229	-0.197					
cg25351550	12	118,227,792	1.05E-06	-0.229	-0.194				*KSR2*	Body
cg06120359	8	127,105,723	0.021594	-0.229	-0.221					
cg19582128	13	39,265,218	0.008274	-0.229	-0.217				*FREM2*	1stExon
cg06842181	11	2,066,779	0.000738	-0.229	-0.205					
cg09192940	16	318,694	0.001395	-0.229	-0.231				*RGS11*	3'UTR
cg08678000	8	89,047,821	0.000384	-0.229	-0.211					
cg14656774	8	71,182,802	3.3E-05	-0.229	-0.193				*NCOA2*	5'UTR
cg16619721	3	160,942,782	0.000112	-0.229	-0.235				*NMD3*	Body
cg20987429	8	91,553,142	0.028184	-0.229	-0.169					
cg06152845	7	1,216,568	0.005391	-0.229	-0.206					
cg10665263	5	107,883,502	0.006842	-0.229	-0.249					
cg10891959	13	81,432,486	0.015063	-0.229	-0.123					
cg26259818	1	181,632,621	0.039598	-0.229	-0.282				*CACNA1E*	Body
cg10143823	14	28,192,481	0.014204	-0.229	-0.127					
cg04291014	11	131,506,437	0.048056	-0.229	-0.127				*NTM*	Body
cg06995828	2	84,160,861	0.004007	-0.229	-0.230					
cg19178742	6	30,070,099	2.11E-07	-0.229	-0.233					
cg06084534	1	158,725,300	0.029934	-0.229	-0.208				*OR6K6*	1stExon
cg26817755	6	19,812,885	2.9E-08	-0.229	-0.211					
cg14961476	1	9,130,738	5.97E-06	-0.229	-0.211				*SLC2A5*	TSS1500
cg06580966	16	7,684,063	5.91E-08	-0.229	-0.229				*A2BP1*	Body
cg19052042	10	44,321,465	0.048212	-0.229	-0.138					

cg01620540	13	47,472,064	0.023467	-0.229	-0.191				*HTR2A*	TSS1500
cg24151308	X	52,826,694	0.015233	-0.229	-0.186				*SPANXN5*	TSS1500
cg18110578	10	50,533,993	0.046994	-0.229	-0.089				*C10orf71*	Body
cg22520372	4	164,271,492	0.010255	-0.229	-0.151				*NPY5R*	Body
cg26008650	1	203,708,794	0.031359	-0.229	-0.117				*ATP2B4*	3'UTR
cg19681610	1	162,148,501	1.59E-11	-0.229	-0.237				*NOS1AP*	Body
cg24949579	11	6,024,966	0.023363	-0.229	-0.168				*OR56A4*	TSS1500
cg24682512	7	86,277,679	0.003381	-0.229	-0.245				*GRM3*	5'UTR
cg04552811	3	149,708,132	6.64E-05	-0.229	-0.192					
cg23858537	15	72,039,838	1.31E-05	-0.229	-0.222				*THSD4*	Body
cg24166018	10	128,021,421	0.004135	-0.229	-0.236				*ADAM12*	Body
cg23172080	5	172,168,177	2.88E-08	-0.229	-0.201					
cg15209053	10	87,426,906	0.006525	-0.229	-0.216				*GRID1*	Body
cg16105569	12	27,627,468	0.01033	-0.229	-0.152				*C12orf70*	Body
cg02379784	5	471,326	3.67E-05	-0.229	-0.222				*LOC25845*	Body
cg14080521	11	27,761,859	0.036323	-0.229	-0.119					
cg15623687	7	112,001,736	2.66E-05	-0.229	-0.192					
cg16989719	2	238,392,110	1.07E-06	-0.229	-0.176					
cg19858323	X	151,283,154	0.000637	-0.229	-0.159				*MAGEA5*	3'UTR
cg20119532	20	749,581	1.12E-13	-0.229	-0.207				*C20orf54*	TSS1500
cg26578726	14	56,289,611	1.27E-05	-0.229	-0.173					
cg03532253	12	56,399,235	9.61E-05	-0.229	-0.149				*SUOX*	3'UTR
cg26782825	8	110,989,116	0.002705	-0.229	-0.232					
cg04519327	15	41,149,780	1.05E-05	-0.229	-0.237				*SPINT1*	3'UTR
cg25350065	8	145,978,427	3.67E-08	-0.229	-0.260				*ZNF251*	Body
cg10447095	16	60,678,710	0.0003	-0.229	-0.199					
cg24561305	13	29,149,132	3.61E-05	-0.229	-0.198					
cg03020635	14	82,526,476	0.000745	-0.229	-0.250					
cg22457769	8	144,822,917	1.34E-06	-0.229	-0.247					
cg23619890	1	5,896,050	0.030176	-0.229	-0.074					
cg17145652	8	42,750,650	1.34E-09	-0.229	-0.203				*RNF170*	5'UTR
cg25982965	6	44,253,910	0.016969	-0.229	-0.135				*TCTE1*	Body
cg03587117	11	100,227,279	0.001197	-0.229	-0.165				*CNTN5*	3'UTR
cg10285618	2	47,604,745	2.86E-10	-0.229	-0.223				*MIR559*	TSS200

cg24977541	3	170,305,197	0.001112	-0.229	-0.214				*SLC7A14*	TSS1500
cg13696545	3	55,784,854	7.58E-07	-0.229	-0.224				*ERC2*	Body
cg03541437	1	210,485,267	0.01179	-0.229	-0.148					
cg02925904	16	32,165,196	0.004667	-0.229	-0.135				*HERC2P4*	TSS1500
cg04944300	1	17,022,524	0.001	-0.229	-0.186				*ESPNP*	Body
cg13451937	17	67,710,309	3.5E-06	-0.229	-0.223					
cg24395386	1	3,328,593	0.012424	-0.229	-0.203				*PRDM16*	Body
cg07788537	1	2,799,876	0.001274	-0.229	-0.161					
cg09228949	10	50,340,173	0.003382	-0.229	-0.145				*FAM170B*	Body
cg22272888	8	27,850,772	1.18E-05	-0.229	-0.160				*SCARA5*	TSS1500
cg26155152	19	2,943,999	6.66E-06	-0.229	-0.253				*ZNF77*	Body
cg05064936	17	45,764,416	2.88E-05	-0.229	-0.157					
cg15246805	6	133,584,189	4.74E-05	-0.229	-0.213				*EYA4*	5'UTR
cg06079495	10	119,599,940	0.017439	-0.229	-0.149					
cg08667569	5	147,240,402	6.65E-08	-0.229	-0.203					
cg03251918	4	156,298,325	0.000315	-0.229	-0.238				*MAP9*	TSS1500
cg13905199	9	103,789,186	1.14E-05	-0.229	-0.221					
cg01717047	7	62,765,320	0.006456	-0.229	-0.123				*LOC643955*	TSS1500
cg06300107	X	63,616,425	5.01E-06	-0.229	-0.200				*MTMR8*	TSS1500
cg07424912	16	58,718,915	3.42E-06	-0.229	-0.218				*SLC38A7*	TSS1500
cg03661929	3	116,503,715	0.005278	-0.229	-0.199					
cg09208942	14	52,794,647	4.54E-10	-0.229	-0.258				*PTGER2*	3'UTR
cg20258486	7	158,800,888	7.46E-05	-0.229	-0.193				*LOC154822*	TSS200
cg22619910	1	55,096,622	0.000671	-0.229	-0.160				*ACOT11*	Body
cg02945019	1	160,550,488	1.57E-06	-0.229	-0.193	2.19E-07	-0.139		*CD84*	TSS1500
cg04167811	8	65,152,945	0.036252	-0.229	-0.144					
cg05215304	2	101,402,664	3.81E-11	-0.229	-0.224					
cg04745064	6	29,416,095	0.031504	-0.229	-0.184					
cg25112284	1	31,246,011	0.001413	-0.229	-0.196					
cg12261055	7	41,860,801	0.007237	-0.229	-0.157					
cg15158924	4	21,710,948	2.49E-06	-0.229	-0.198				*KCNIP4*	5'UTR
cg02854396	12	72,696,687	0.000766	-0.229	-0.170				*TRHDE*	Body
cg11333681	1	149,232,963	0.00387	-0.229	-0.150					
cg26663590	16	28,959,310	2.29E-09	-0.229	-0.240					

cg05303690	8	32,284,962	0.000178	-0.229	-0.138					*NRG1*	Body
cg03640051	4	42,657,689	3.42E-10	-0.229	-0.238					*ATP8A1*	Body
cg04297995	19	52,023,213	0.000441	-0.229	-0.199					*SIGLEC6*	3'UTR
cg05762612	18	76,260,568	0.010603	-0.229	-0.123						
cg08199529	11	104,894,514	0.003514	-0.229	-0.127					*CASP5*	TSS1500
cg01174441	18	52,262,555	3.16E-05	-0.230	-0.209					*C18orf26*	Body
cg02186027	4	189,580,798	3.42E-05	-0.230	-0.227						
cg06501157	2	52,856,495	0.036889	-0.230	-0.080						
cg17001753	6	44,254,089	0.026776	-0.230	-0.137					*TCTE1*	Body
cg03324851	7	100,274,414	2.45E-12	-0.230	-0.201					*GNB2*	Body
cg06372145	20	29,979,846	0.012407	-0.230	-0.177					*DEFB119*	TSS1500
cg15071463	1	116,854,912	5.83E-06	-0.230	-0.234						
cg18391442	2	56,318,614	0.015957	-0.230	-0.120						
cg05825085	16	86,379,872	0.000658	-0.230	-0.207					*LOC732275*	TSS1500
cg21823425	11	123,757,331	0.000379	-0.230	-0.224					*TMEM225*	TSS1500
cg16447311	6	1,604,134	2.07E-07	-0.230	-0.220						
cg19448144	6	32,169,707	0.001997	-0.230	-0.105					*NOTCH4*	Body
cg02283042	16	83,200,382	1.88E-06	-0.230	-0.208					*CDH13*	Body
cg19840494	14	89,606,221	0.006312	-0.230	-0.286						
cg22135279	11	124,055,251	0.006451	-0.230	-0.204						
cg14353278	2	21,229,231	0.041878	-0.230	-0.154					*APOB*	Body
cg15247483	3	111,853,297	0.001317	-0.230	-0.187					*GCET2*	TSS1500
cg21825792	12	5,015,078	0.02902	-0.230	-0.144						
cg17291924	X	140,756,646	0.003051	-0.230	-0.203						
cg00851261	19	1,526,651	0.005074	-0.230	-0.109				Yes	*PLK5P*	Body
cg04060317	12	5,603,248	0.00079	-0.230	-0.177					*NTF3*	TSS200
cg21328389	8	109,145,172	0.021181	-0.230	-0.258						
cg23575349	11	88,800,388	0.005741	-0.230	-0.143						
cg26119806	2	18,061,572	4.55E-10	-0.230	-0.243					*KCNS3*	5'UTR
cg24928023	21	45,395,775	0.00229	-0.230	-0.143					*AGPAT3*	Body
cg08508335	13	19,692,412	0.000846	-0.230	-0.161					*DKFZp686A1627*	Body
cg04388863	16	48,269,349	0.000203	-0.230	-0.194					*ABCC11*	TSS1500
cg14948379	2	105,657,348	0.000192	-0.230	-0.233					*MRPS9*	Body
cg18446069	13	111,644,279	0.016793	-0.230	-0.115						

cg11341142	12	72,152,267	7.82E-08	-0.230	-0.214				*RAB21*	Body
cg16792071	10	134,842,795	0.0026	-0.230	-0.182					
cg02133070	4	104,637,628	2.2E-05	-0.230	-0.154				*TACR3*	Body
cg08400475	1	158,390,842	0.034874	-0.230	-0.162				*OR10K2*	TSS200
cg21643939	3	172,467,326	4.37E-06	-0.230	-0.211					
cg06902828	8	92,647,788	0.001078	-0.230	-0.190					
cg15659974	X	115,567,527	1.74E-07	-0.230	-0.250			Yes	*SLC6A14*	TSS1500
cg19263469	7	63,655,394	4.11E-11	-0.230	-0.204					
cg21802682	11	69,682,593	2.46E-05	-0.230	-0.257					
cg00382930	16	87,100,229	0.009016	-0.230	-0.092					
cg00436435	6	30,119,889	2.9E-07	-0.230	-0.197				*TRIM10*	3'UTR
cg03762767	X	27,884,673	0.025558	-0.230	-0.117					
cg22026150	8	50,653,870	0.012886	-0.230	-0.148					
cg01699693	11	124,181,447	0.019723	-0.230	-0.220				*OR8D1*	TSS1500
cg02292206	7	149,569,715	0.001022	-0.230	-0.205				*ATP6V0E2*	TSS1500
cg19459470	11	131,199,383	0.000292	-0.230	-0.210					
cg24868292	1	84,876,625	3.19E-07	-0.230	-0.228				*DNASE2B*	5'UTR
cg02642585	21	32,202,284	0.014912	-0.230	-0.162				*KRTAP7-1*	TSS1500
cg03895540	11	5,841,482	6.74E-05	-0.230	-0.189				*OR52N2*	TSS200
cg14386870	16	2,013,110	2.84E-11	-0.230	-0.251				*SNORA10*	TSS1500
cg06837325	5	97,445,281	0.002366	-0.230	-0.225					
cg08272996	16	64,128,218	0.002893	-0.230	-0.206					
cg10767420	2	2,581,557	0.000814	-0.230	-0.244					
cg06514727	1	198,711,467	0.041399	-0.230	-0.113				*PTPRC*	Body
cg00629688	11	105,479,843	1.39E-05	-0.230	-0.189				*GRIA4*	TSS1500
cg11976616	5	41,213,172	0.003426	-0.230	-0.141	8.04E-09	-0.126		*C6*	5'UTR
cg25711239	17	73,996,656	3.27E-05	-0.230	-0.160				*CDK3*	TSS1500
cg07851738	8	131,896,788	1.08E-06	-0.230	-0.242				*ADCY8*	Body
cg09979311	16	1,730,579	0.012537	-0.230	-0.273				*HN1L*	Body
cg14284187	9	116,355,097	1.85E-08	-0.230	-0.227				*RGS3*	Body
cg16802855	16	86,371,130	0.035522	-0.230	-0.161				*LOC732275*	Body
cg06194602	17	61,997,056	0.005556	-0.230	-0.143				*GH1*	TSS1500
cg19480470	2	559,042	2.09E-08	-0.230	-0.229					
cg19951409	18	74,175,071	0.031742	-0.230	-0.183				*ZNF516*	TSS200

cg22543892	7	85,157,964	0.00214	-0.230	-0.177					
cg03287339	16	711,135	6E-16	-0.230	-0.227				*WDR90*	Body
cg06789677	10	108,525,625	0.016687	-0.230	-0.166				*SORCS1*	Body
cg13700912	7	1,968,348	1.9E-07	-0.230	-0.236				*MAD1L1*	Body
cg24080902	18	73,631,749	0.013311	-0.230	-0.172					
cg06217320	11	132,112,645	0.018706	-0.230	-0.159				*NTM*	Body
cg23120936	6	44,251,344	0.004111	-0.230	-0.173				*TCTE1*	Body
cg23634451	3	100,713,646	0.021539	-0.230	-0.149				*ABI3BP*	TSS1500
cg14445205	8	72,565,468	0.020333	-0.230	-0.167					
cg25312122	1	160,184,732	1.24E-06	-0.230	-0.230				*PEA15*	3'UTR
cg14318001	14	70,236,385	0.005774	-0.230	-0.195				*SFRS5*	Body
cg17596359	13	114,844,124	3.67E-09	-0.230	-0.214				*RASA3*	Body
cg01883966	6	31,938,886	1.31E-06	-0.230	-0.217	2.26E-24	-0.192		*STK19*	TSS200
cg00988175	11	59,481,031	0.014311	-0.230	-0.201				*OR10V1*	1stExon
cg12576411	13	22,694,106	0.019161	-0.230	-0.252					
cg17979595	5	175,995,850	0.030351	-0.230	-0.101				*PCDH24*	Body
cg06702891	12	132,872,081	0.009046	-0.230	-0.106				*GALNT9*	Body
cg03641797	7	5,396,436	0.026095	-0.230	-0.261				*TNRC18*	Body
cg03722396	4	111,537,907	0.047889	-0.230	-0.129					
cg08242576	1	153,177,188	0.006751	-0.230	-0.165				*LELP1*	Body
cg02576228	16	7,702,102	0.017466	-0.231	-0.123				*A2BP1*	Body
cg04846348	6	25,024,044	0.003168	-0.231	-0.203					
cg26757472	16	86,387,131	0.022194	-0.231	-0.145					
cg02197192	8	64,968,883	0.010247	-0.231	-0.172					
cg02708121	4	16,670,376	0.016757	-0.231	-0.098				*LDB2*	Body
cg14171986	11	4,629,749	0.000182	-0.231	-0.236				*TRIM68*	TSS1500
cg07837520	16	19,871,730	1.96E-05	-0.231	-0.250				*GPRC5B*	3'UTR
cg00781378	3	173,999,704	9.02E-14	-0.231	-0.241				*NLGN1*	3'UTR
cg09233156	15	96,531,769	2.39E-05	-0.231	-0.250					
cg11583211	1	152,769,246	0.000353	-0.231	-0.199				*LCE1D*	5'UTR
cg13753460	14	101,520,989	0.000825	-0.231	-0.247				*MIR134*	TSS200
cg15079124	7	142,829,213	0.001995	-0.231	-0.152				*PIP*	1stExon
cg05868547	2	115,425,220	0.001803	-0.231	-0.243				*DPP10*	Body
cg01002636	21	15,588,120	0.001275	-0.231	-0.139				*RBM11*	TSS1500

cg19765196	3	105,299,405	2.66E-08	-0.231	-0.212					
cg27258486	12	130,420,637	0.012227	-0.231	-0.160					
cg21792737	6	155,776,999	1.01E-11	-0.231	-0.217	0.01216615	-0.054		*NOX3*	1stExon
cg18324583	5	142,975,083	2.48E-11	-0.231	-0.235					
cg24377285	19	38,943,724	2.48E-05	-0.231	-0.152				*RYR1*	Body
cg12386752	1	143,907,550	1.36E-11	-0.231	-0.226				*FAM72D*	Body
cg04214938	2	119,496,171	0.014731	-0.231	-0.124					
cg12373951	3	133,503,437	0.000384	-0.231	-0.238					
cg10680621	5	139,069,449	7.94E-13	-0.231	-0.215					
cg27073431	2	47,404,636	4.81E-09	-0.231	-0.207				*CALM2*	TSS1500
cg03718411	5	31,280,802	0.001962	-0.231	-0.250				*CDH6*	Body
cg09437783	2	88,747,976	0.003579	-0.231	-0.155				*FOXI3*	Body
cg14884160	7	89,840,396	3.45E-24	-0.231	-0.216				*STEAP2*	TSS1500
cg11731805	19	35,637,957	0.000479	-0.231	-0.166				*FXYD7*	Body
cg00163541	1	238,811,442	8.36E-05	-0.231	-0.215					
cg25946540	20	30,028,418	0.024046	-0.231	-0.181				*DEFB123*	1stExon
cg00543684	13	113,652,980	0.004804	-0.231	-0.182				*MCF2L*	Body
cg27220660	16	29,820,429	3.07E-17	-0.231	-0.225				*MAZ*	Body
cg06582250	11	133,445,802	0.003614	-0.231	-0.153					
cg19866594	8	73,848,457	0.010936	-0.231	-0.185				*KCNB2*	Body
cg21462633	2	239,437,806	0.003616	-0.231	-0.283					
cg09329930	2	223,135,786	4.7E-07	-0.231	-0.249				*PAX3*	Body
cg12727680	3	75,959,386	0.014835	-0.231	-0.179					
cg11966284	1	2,774,346	8.65E-05	-0.231	-0.192			Yes		
cg14319135	7	63,667,452	0.006425	-0.231	-0.167				*ZNF735*	TSS200
cg20844619	4	152,875,860	0.001992	-0.231	-0.157					
cg02840936	1	20,492,386	0.014062	-0.231	-0.331				*PLA2G2C*	Body
cg01456609	16	25,700,781	9.11E-13	-0.231	-0.224					
cg06833778	1	241,464,644	0.001317	-0.231	-0.235				*RGS7*	Body
cg17129188	8	134,307,597	3.43E-06	-0.231	-0.233				*NDRG1*	5'UTR
cg03328485	16	62,267,020	0.008653	-0.231	-0.127					
cg21367256	20	62,795,658	0.024885	-0.231	-0.195				*MYT1*	TSS200
cg22914987	12	4,054,555	0.018461	-0.231	-0.144					
cg16642695	8	67,343,633	8.73E-09	-0.231	-0.225				*ADHFE1*	TSS1500

cg25502462	X	48,620,142	0.001021	-0.231	-0.271				*GLOD5*	TSS200
cg01092932	1	198,828,060	0.047491	-0.231	-0.106				*MIR181B1*	Body
cg02167456	X	147,800,736	0.007936	-0.231	-0.218				*AFF2*	Body
cg24382712	10	119,802,739	1.02E-05	-0.231	-0.197				*RAB11FIP2*	Body
cg10422455	11	19,083,542	0.00183	-0.231	-0.158				*MRGPRX2*	TSS1500
cg21098892	19	534,887	2.86E-05	-0.231	-0.136				*CDC34*	Body
cg19576556	17	80,024,177	3.9E-05	-0.231	-0.200				*DUS1L*	TSS1500
cg05142677	16	88,601,446	6.78E-09	-0.231	-0.198				*ZFPM1*	3'UTR
cg19864808	6	31,938,773	3.93E-10	-0.231	-0.233				*STK19*	TSS200
cg10021288	2	128,175,891	0.000551	-0.231	-0.186				*PROC*	TSS200
cg21803927	8	14,412,919	4.5E-06	-0.231	-0.230				*SGCZ*	Body
cg23496571	20	30,644,728	4.28E-05	-0.231	-0.187				*HCK*	Body
cg00007076	8	67,342,600	2.64E-15	-0.231	-0.224				*RRS1*	1stExon
cg02703563	10	91,852,861	7.13E-05	-0.231	-0.280					
cg21688152	17	80,926,101	0.006154	-0.231	-0.276				*B3GNTL1*	Body
cg00345366	11	18,128,177	2.88E-08	-0.231	-0.194				*SAAL1*	TSS1500
cg02215357	13	53,191,046	0.000108	-0.231	-0.227				*HNRNPA1L2*	TSS1500
cg15403616	14	105,658,685	0.00444	-0.231	-0.126					
cg11838237	5	1,752,864	0.009648	-0.231	-0.130					
cg18247124	11	122,943,141	6.06E-06	-0.231	-0.228				*ASAM*	3'UTR
cg22602592	20	61,956,679	0.000985	-0.231	-0.136				*COL20A1*	Body
cg11937849	3	76,292,114	0.0307	-0.231	-0.100					
cg25237113	2	80,551,812	0.000347	-0.231	-0.285				*CTNNA2*	Body
cg05999628	17	32,612,684	7.34E-07	-0.231	-0.227				*CCL11*	TSS200
cg16204066	6	33,638,922	0.0193	-0.231	-0.136				*ITPR3*	Body
cg24009250	14	98,017,621	0.000594	-0.231	-0.181					
cg06560282	11	103,830,847	0.000561	-0.231	-0.149				*PDGFD*	Body
cg14567374	9	140,084,348	1.42E-10	-0.231	-0.218				*ANAPC2*	TSS1500
cg14943002	11	47,256,035	6.64E-10	-0.231	-0.213				*DDB2*	Body
cg00432659	18	11,151,283	0.003251	-0.231	-0.164					
cg09165554	16	33,935,224	0.003965	-0.231	-0.186					
cg12377641	10	11,868,179	0.040293	-0.231	-0.131				*C10orf47*	5'UTR
cg13917968	5	151,504,425	0.019968	-0.231	-0.250					
cg00921682	19	42,081,255	0.000482	-0.231	-0.188				*CEACAM21*	TSS1500

cg04811706	16	55,686,715	0.028238	-0.231	-0.082					
cg26017484	1	3,322,309	0.00069	-0.231	-0.220				*PRDM16*	Body
cg14500932	9	126,534,337	3.97E-08	-0.231	-0.190				*DENND1A*	Body
cg17679980	11	22,696,306	2.07E-08	-0.231	-0.236				*GAS2*	5'UTR
cg14788655	11	40,224,146	0.029425	-0.231	-0.202				*LRRC4C*	5'UTR
cg25331127	10	133,608,464	0.011629	-0.231	-0.152					
cg24669741	1	186,265,423	4.92E-06	-0.231	-0.221				*PRG4*	1stExon
cg12156167	8	133,760,274	0.032005	-0.231	-0.168				*TMEM71*	Body
cg24556382	4	174,173,455	0.001577	-0.231	-0.204				*GALNT7*	Body
cg02546833	15	53,372,675	0.006583	-0.231	-0.149					
cg20901032	13	37,680,691	7.2E-05	-0.231	-0.207				*CSNK1A1L*	TSS1500
cg17576736	13	112,908,450	0.005711	-0.231	-0.154					
cg23462788	3	38,941,491	0.012136	-0.231	-0.181				*SCN11A*	Body
cg14286486	10	105,125,108	1.03E-05	-0.231	-0.240					
cg03581262	2	106,192,732	1.77E-17	-0.231	-0.212					
cg14006798	20	30,222,136	0.000409	-0.231	-0.105					
cg07891457	11	67,442,075	1.76E-06	-0.231	-0.223				*ALDH3B2*	1stExon
cg03037761	11	86,663,341	4.1E-10	-0.231	-0.179				*FZD4*	Body
cg07236204	1	199,372,786	0.004304	-0.231	-0.230					
cg09513758	1	110,526,368	1.8E-08	-0.231	-0.204				*AHCYL1*	TSS1500
cg15499275	1	9,780,620	2.67E-05	-0.231	-0.173				*PIK3CD*	Body
cg00334056	6	33,755,658	3.66E-09	-0.232	-0.225				*LEMD2*	Body
cg15484808	6	33,242,542	1.78E-10	-0.232	-0.236				*RPS18*	Body
cg02583247	12	4,555,311	0.018427	-0.232	-0.111				*FGF6*	TSS1500
cg17186132	15	90,703,444	2.49E-09	-0.232	-0.238					
cg01571916	11	44,881,716	0.000403	-0.232	-0.159				*TSPAN18*	5'UTR
cg12930930	11	5,372,503	0.008944	-0.232	-0.236				*OR51B6*	TSS1500
cg20562823	4	63,872,386	0.000509	-0.232	-0.195					
cg17976205	20	61,002,857	1.27E-06	-0.232	-0.241				*C20orf151*	TSS1500
cg02630628	19	50,116,703	4.14E-07	-0.232	-0.202				*PRR12*	Body
cg22377480	3	35,934,217	0.004299	-0.232	-0.152					
cg22792177	1	1,912,606	0.017019	-0.232	-0.150				*KIAA1751*	Body
cg05936516	5	114,507,066	1.49E-10	-0.232	-0.245				*TRIM36*	Body
cg05149258	5	13,944,584	1.16E-05	-0.232	-0.235				*DNAH5*	1stExon

cg11026867	12	6,473,643	4.3E-05	-0.232	-0.247				*SCNN1A*	Body
cg06612122	16	59,788,951	0.018474	-0.232	-0.197				*LOC644649*	Body
cg12629586	10	50,122,337	0.04818	-0.232	-0.120				*WDFY4*	Body
cg06867285	7	79,642,961	0.00609	-0.232	-0.208					
cg07875692	12	64,173,139	2.11E-10	-0.232	-0.207				*TMEM5*	TSS1500
cg04232098	3	112,607,776	0.020121	-0.232	-0.149					
cg16900102	7	98,283,412	0.011912	-0.232	-0.183					
cg04969878	12	72,079,240	1.84E-08	-0.232	-0.225	5.41E-10	-0.092		*TMEM19*	TSS1500
cg14993663	8	63,055,928	0.044861	-0.232	-0.188					
cg22356268	19	58,693,616	7.22E-05	-0.232	-0.228				*ZNF274*	TSS1500
cg06706813	3	194,826,411	0.010549	-0.232	-0.258				*C3orf21*	Body
cg13460934	1	3,003,690	0.02155	-0.232	-0.077				*PRDM16*	Body
cg25434272	10	117,697,078	2.15E-06	-0.232	-0.196				*ATRNL1*	Body
cg23676880	19	23,082,052	0.034838	-0.232	-0.159			Yes		
cg26388177	11	1,889,547	0.013894	-0.232	-0.099				*LSP1*	TSS1500
cg03498091	14	28,399,440	0.006203	-0.232	-0.162					
cg10923855	12	34,488,830	1.53E-05	-0.232	-0.229					
cg11760500	11	128,385,880	1.4E-12	-0.232	-0.248				*ETS1*	Body
cg01306183	11	63,057,478	0.000124	-0.232	-0.228				*SLC22A10*	5'UTR
cg02723558	11	27,536,073	5.82E-05	-0.232	-0.211				*BDNFOS*	Body
cg08355317	X	100,642,128	0.001749	-0.232	-0.159				*BTK*	TSS1500
cg16009120	12	8,686,707	0.013	-0.232	-0.190				*CLEC4E*	3'UTR
cg17742051	19	13,321,231	0.001953	-0.232	-0.090				*CACNA1A*	Body
cg08825075	16	3,934,709	0.000348	-0.232	-0.149					
cg19749001	2	11,681,405	0.005323	-0.232	-0.141				*GREB1*	TSS1500
cg21017775	11	34,535,481	0.038994	-0.232	-0.275				*ELF5*	TSS200
cg14425054	9	136,860,187	2.9E-13	-0.232	-0.220					
cg14642773	18	75,888,474	0.004575	-0.232	-0.131					
cg11331191	X	63,446,644	0.004266	-0.232	-0.221				*ASB12*	TSS1500
cg16933762	3	77,147,109	0.003941	-0.232	-0.216				*ROBO2*	TSS200
cg02066184	7	16,841,504	0.006748	-0.232	-0.281				*AGR2*	5'UTR
cg03948902	1	170,669,166	0.021502	-0.232	-0.150				*PRRX1*	Body
cg11408632	1	4,856,487	0.025812	-0.232	-0.124					
cg22825220	15	54,582,445	0.001782	-0.232	-0.260				*UNC13C*	Body

cg12942155	3	35,721,284	0.019297	-0.232	-0.150				*ARPP-21*	5'UTR
cg15397577	22	39,910,060	4.64E-05	-0.232	-0.190				*SMCR7L*	Body
cg07426101	11	59,841,011	0.001608	-0.232	-0.169					
cg24016995	10	129,868,149	7.05E-07	-0.232	-0.169				*PTPRE*	Body
cg15853771	2	52,656,979	0.000149	-0.232	-0.239					
cg16215361	11	26,594,620	1.1E-05	-0.232	-0.221	0.00061542	-0.063		*MUC15*	TSS1500
cg17393458	5	176,982,009	4.71E-11	-0.232	-0.215				*FAM193B*	TSS1500
cg25071869	8	21,867,345	0.001504	-0.232	-0.149					
cg05233614	12	131,142,966	0.004946	-0.232	-0.173					
cg17079556	7	57,252,146	0.000404	-0.232	-0.175					
cg18461347	7	78,947,094	3.09E-06	-0.232	-0.240				*MAGI2*	Body
cg06182261	20	5,451,661	0.001637	-0.232	-0.178				*LOC643406*	TSS200
cg13824815	11	124,527,240	1.29E-06	-0.232	-0.180				*SIAE*	Body
cg14251216	13	76,445,065	5.54E-06	-0.232	-0.169					
cg22262095	19	8,669,981	0.001146	-0.232	-0.130				*ADAMTS10*	Body
cg23613317	5	154,092,109	9.08E-06	-0.232	-0.222				*LARP1*	TSS1500
cg15638170	20	44,261,252	0.015554	-0.232	-0.165				*WFDC9*	TSS1500
cg01992512	21	46,493,156	0.006103	-0.232	-0.155				*ADARB1*	TSS1500
cg16921285	13	104,003,206	0.046685	-0.232	-0.177					
cg18758233	13	102,374,627	0.002733	-0.232	-0.134				*FGF14*	3'UTR
cg16451027	5	112,042,859	0.0001	-0.232	-0.268				*APC*	TSS1500
cg03548035	11	86,663,407	7.78E-09	-0.232	-0.188				*FZD4*	Body
cg16132208	13	76,536,939	0.00187	-0.232	-0.228					
cg03621504	12	116,571,240	2.75E-10	-0.232	-0.223				*MED13L*	Body
cg07787771	10	104,341,826	1.47E-05	-0.232	-0.224				*SUFU*	Body
cg21911343	3	46,672,436	0.000203	-0.232	-0.214					
cg20319457	2	20,133,404	4.06E-06	-0.232	-0.245				*WDR35*	Body
cg09641127	2	10,193,895	0.009492	-0.232	-0.182				*KLF11*	3'UTR
cg18196709	1	170,646,916	0.038691	-0.232	-0.139				*PRRX1*	Body
cg18430645	7	63,491,149	0.00012	-0.232	-0.184					
cg19178642	15	89,657,390	5.87E-05	-0.232	-0.194				*ABHD2*	5'UTR
cg25943719	15	101,421,391	1.92E-10	-0.232	-0.233				*ALDH1A3*	Body
cg25049844	21	46,933,374	9.75E-10	-0.232	-0.231				*COL18A1*	3'UTR
cg23110514	1	152,538,888	0.00194	-0.232	-0.182	3.62E-09	-0.176		*LCE3E*	5'UTR

cg00190738	5	112,042,650	0.000649	-0.232	-0.285					*APC*	TSS1500
cg07912766	18	45,458,698	7.35E-06	-0.232	-0.222					*SMAD2*	TSS1500
cg14187242	9	117,673,469	2.42E-07	-0.232	-0.205					*TNFSF8*	Body
cg13985485	1	239,550,283	5.77E-09	-0.232	-0.232						
cg21041579	14	95,907,997	0.001208	-0.232	-0.155					*C14orf49*	Body
cg07184316	1	157,669,111	0.002696	-0.232	-0.241					*FCRL3*	Body
cg02106180	16	845,767	2.29E-05	-0.232	-0.247					*CHTF18*	Body
cg01731341	12	4,554,295	0.01266	-0.232	-0.119	1.03E-08	-0.121			*FGF6*	Body
cg01303032	10	25,940,372	0.005687	-0.232	-0.224						
cg12756893	4	24,232,894	0.007891	-0.232	-0.182						
cg14975238	7	97,841,591	0.011425	-0.232	-0.094	2.18E-18	-0.125			*BHLHA15*	1stExon
cg18794475	8	55,533,829	0.012219	-0.232	-0.105					*RP1*	Body
cg04394529	11	2,067,849	0.018106	-0.232	-0.118						
cg09374437	19	52,769,790	3.56E-06	-0.232	-0.191						
cg24403338	20	31,366,437	0.000883	-0.232	-0.169					*DNMT3B*	5'UTR
cg01279413	20	62,030,494	2.14E-10	-0.232	-0.231						
cg10938393	5	32,171,158	2.08E-08	-0.233	-0.223					*GOLPH3*	Body
cg14492302	17	21,911,362	0.038336	-0.233	-0.117					*FLJ36000*	Body
cg23770008	12	34,453,757	0.046464	-0.233	-0.128						
cg05089968	6	166,722,773	1.19E-07	-0.233	-0.209	1.11E-08	-0.139			*PRR18*	TSS1500
cg25580656	14	104,197,160	1.46E-05	-0.233	-0.206					*ZFYVE21*	Body
cg01928231	6	3,502,698	1.87E-05	-0.233	-0.201						
cg21027035	7	152,591,949	0.012775	-0.233	-0.129						
cg25569300	14	97,538,227	0.017276	-0.233	-0.185						
cg14863944	13	22,740,131	0.036151	-0.233	-0.209						
cg08325307	7	31,557,142	0.027087	-0.233	-0.111					*CCDC129*	1stExon
cg24253136	11	34,535,140	0.02431	-0.233	-0.259					*ELF5*	5'UTR
cg24681125	2	154,725,150	0.002118	-0.233	-0.155						
cg02260630	16	75,491,894	9.05E-08	-0.233	-0.218					*TMEM170A*	Body
cg17929238	4	111,545,628	7.33E-05	-0.233	-0.113					*PITX2*	TSS1500
cg17819250	8	36,380,237	4.13E-06	-0.233	-0.244						
cg17908564	3	89,294,826	0.002469	-0.233	-0.197					*EPHA3*	Body
cg19224278	15	101,419,017	0.004902	-0.233	-0.174	6.91E-12	-0.197			*ALDH1A3*	TSS1500
cg03976856	1	2,764,669	1.23E-12	-0.233	-0.213						

cg07241084	7	47,493,388	0.004042	-0.233	-0.201					*TNS3*	5'UTR
cg01518591	6	29,408,229	0.008312	-0.233	-0.220					*OR10C1*	1stExon
cg09947274	2	27,439,915	2.44E-05	-0.233	-0.200	1.07E-08	-0.078			*C2orf28*	3'UTR
cg20742415	1	170,114,481	0.026056	-0.233	-0.070					*METTL11B*	TSS1500
cg06050193	16	87,101,721	0.027569	-0.233	-0.127						
cg08077807	14	62,001,072	0.002749	-0.233	-0.139					*PRKCH*	Body
cg03310930	10	109,677,537	0.046069	-0.233	-0.176						
cg07856280	8	48,038,289	0.006376	-0.233	-0.198						
cg23903244	11	2,812,987	0.007749	-0.233	-0.275					*KCNQ1*	Body
cg03312565	1	4,577,365	0.008858	-0.233	-0.179						
cg11915309	10	50,123,344	0.026026	-0.233	-0.093					*LRRC18*	TSS1500
cg15698945	6	132,858,597	0.005289	-0.233	-0.162					*TAAR9*	TSS1500
cg04774930	16	23,768,982	0.034538	-0.233	-0.182					*CHP2*	3'UTR
cg15230164	8	89,747,827	0.005331	-0.233	-0.162						
cg05463326	8	133,118,481	0.038335	-0.233	-0.199					*HHLA1*	TSS1500
cg01747862	4	174,457,371	1.53E-07	-0.233	-0.239					*NBLA00301*	Body
cg18537464	18	76,345,144	0.028202	-0.233	-0.126						
cg27020161	10	108,318,417	1.34E-07	-0.233	-0.212						
cg16002378	14	105,036,533	0.01236	-0.233	-0.141						
cg02490034	7	130,125,836	7.7E-05	-0.233	-0.243					*MEST*	TSS1500
cg11999580	8	118,383,430	0.001218	-0.233	-0.178						
cg21901160	13	48,046,005	0.004116	-0.233	-0.195						
cg00491207	18	76,270,362	0.000282	-0.233	-0.137						
cg05046020	1	158,148,523	0.012992	-0.233	-0.241					*CD1D*	TSS1500
cg08134681	2	88,742,592	0.022687	-0.233	-0.129						
cg04291383	1	12,787,380	0.009201	-0.233	-0.193					*AADACL3*	3'UTR
cg07973026	4	103,553,119	0.00958	-0.233	-0.218					*MANBA*	3'UTR
cg18123581	8	90,772,386	1.25E-07	-0.233	-0.253					*RIPK2*	Body
cg26800525	13	113,411,519	7.78E-05	-0.233	-0.238					*ATP11A*	Body
cg14216734	1	158,817,808	0.000285	-0.233	-0.274					*MNDA*	Body
cg15444735	13	56,140,478	0.001987	-0.233	-0.158						
cg13635184	7	148,903,126	1.93E-06	-0.233	-0.234					*ZNF282*	Body
cg22793142	1	207,995,752	1.2E-07	-0.233	-0.211					*LOC148696*	Body
cg25762854	8	80,048,524	0.008216	-0.233	-0.182						

cg09869595	1	48,703,725	1.88E-09	-0.233	-0.230				*SLC5A9*	Body
cg11759354	6	32,946,541	0.000586	-0.233	-0.303				*BRD2*	Body
cg09250365	11	92,080,444	1.45E-12	-0.233	-0.238					
cg15623444	4	103,867,628	0.003565	-0.233	-0.219				*NHEDC1*	Body
cg17546376	3	42,172,097	8.95E-06	-0.233	-0.228				*TRAK1*	Body
cg09599971	16	7,615,516	0.00134	-0.233	-0.174				*A2BP1*	Body
cg06580419	2	215,276,156	8.01E-08	-0.233	-0.207				*VWC2L*	TSS1500
cg17073708	8	55,374,489	0.006985	-0.233	-0.169					
cg21196708	8	118,357,170	0.002264	-0.233	-0.205					
cg26370066	19	7,685,601	0.009432	-0.233	-0.130				*XAB2*	Body
cg04604142	17	48,796,515	0.000898	-0.233	-0.204				*LUC7L3*	TSS1500
cg06995408	2	48,977,089	9.73E-06	-0.233	-0.218				*LHCGR*	Body
cg12598937	10	10,987,228	0.048757	-0.233	-0.142				*LOC254312*	Body
cg19404207	7	99,587,136	1.67E-07	-0.233	-0.224					
cg03660010	4	48,342,263	2.73E-08	-0.233	-0.243				*SLAIN2*	TSS1500
cg23069297	2	242,833,648	9.46E-05	-0.233	-0.227					
cg11645465	8	55,243,181	0.006059	-0.233	-0.156					
cg15489693	4	10,498,037	1.34E-11	-0.233	-0.220				*CLNK*	Body
cg27652350	15	101,420,989	8.08E-11	-0.233	-0.229	1E-12	-0.168		*ALDH1A3*	Body
cg10883352	3	142,895,375	9.08E-05	-0.233	-0.178	0.0002182	-0.089			
cg03886699	1	212,698,918	0.026348	-0.233	-0.041					
cg03539903	20	53,088,317	0.004241	-0.233	-0.170					
cg09455887	16	11,629,183	0.010775	-0.233	-0.185					
cg24595152	3	43,733,831	7.1E-08	-0.233	-0.192				*ABHD5*	Body
cg06646682	1	1,273,442	0.020351	-0.233	-0.220				*DVL1*	Body
cg10237442	22	31,475,122	0.000334	-0.233	-0.225					
cg08412936	6	31,097,051	0.000113	-0.233	-0.197				*PSORS1C1*	5'UTR
cg15698171	20	62,795,464	0.007371	-0.233	-0.217				*MYT1*	TSS1500
cg19112204	11	35,425,551	6.57E-07	-0.233	-0.225				*SLC1A2*	Body
cg14219115	9	115,707,123	0.006065	-0.233	-0.230					
cg13561518	14	22,336,334	0.008295	-0.233	-0.244					
cg18329797	4	173,585,926	0.029975	-0.233	-0.175				*GALNTL6*	Body
cg23319289	14	82,292,196	0.000168	-0.233	-0.222					
cg04049033	17	1,553,661	0.001262	-0.233	-0.158	3.61E-12	-0.102		*RILP*	TSS1500

cg04840339	6	123,957,958	0.024657	-0.233	-0.148				*TRDN*	TSS200
cg21181162	8	72,650,106	0.006435	-0.234	-0.265					
cg12496800	10	72,648,987	1.7E-07	-0.234	-0.231				*PCBD1*	TSS1500
cg16149943	21	46,932,082	9.78E-05	-0.234	-0.212				*COL18A1*	Body
cg00731958	7	48,081,031	0.042751	-0.234	-0.164				*C7orf57*	Body
cg00096106	5	100,176,809	3.81E-05	-0.234	-0.242				*ST8SIA4*	Body
cg18071162	5	25,909,453	0.000792	-0.234	-0.159					
cg04170440	6	154,410,350	9.87E-06	-0.234	-0.174				*OPRM1*	Body
cg24839529	4	185,668,393	3.24E-09	-0.234	-0.242					
cg08853572	16	1,843,915	0.000156	-0.234	-0.197				*IGFALS*	TSS200
cg20127899	8	32,579,253	2.55E-07	-0.234	-0.198				*NRG1*	Body
cg21268144	12	34,531,722	4.07E-09	-0.234	-0.216					
cg08886154	7	127,256,676	8.17E-05	-0.234	-0.174	3.06E-16	-0.171		*PAX4*	TSS1500
cg22338152	19	57,642,143	0.004191	-0.234	-0.240				*USP29*	Body
cg05933815	16	22,012,743	1.59E-05	-0.234	-0.195				*C16orf65*	TSS1500
cg06582512	12	47,629,311	2.25E-11	-0.234	-0.209				*FAM113B*	Body
cg02109704	5	1,721,452	2.37E-05	-0.234	-0.197					
cg13488501	1	46,805,584	0.000643	-0.234	-0.302				*NSUN4*	TSS1500
cg26891645	1	12,908,204	0.02163	-0.234	-0.142				*HNRNPCL1*	5'UTR
cg15238224	6	30,081,408	0.000503	-0.234	-0.180	2.95E-13	-0.179		*TRIM31*	TSS1500
cg15697852	5	140,098,576	0.035359	-0.234	-0.187				*VTRNA1-2*	Body
cg01845764	11	127,112,911	8.72E-05	-0.234	-0.244					
cg09288312	10	134,830,961	0.0056	-0.234	-0.127					
cg23620049	3	185,270,558	2.71E-10	-0.234	-0.210				*LIPH*	TSS200
cg21166380	13	39,565,478	0.001991	-0.234	-0.195				*STOML3*	TSS1500
cg22183373	4	24,806,777	6.62E-05	-0.234	-0.224					
cg10664754	9	139,939,662	9.63E-10	-0.234	-0.240				*NPDC1*	Body
cg03996020	7	157,180,124	8.04E-05	-0.234	-0.152				*DNAJB6*	Body
cg22255095	20	60,472,331	0.00186	-0.234	-0.142				*CDH4*	Body
cg05658922	16	86,743,763	0.023032	-0.234	-0.098					
cg08415557	19	9,226,505	0.002469	-0.234	-0.123				*OR7G1*	TSS200
cg15486846	15	24,509,693	1.92E-05	-0.234	-0.224					
cg25839267	3	77,091,645	0.009783	-0.234	-0.204				*ROBO2*	Body
cg09221035	1	3,059,517	0.006144	-0.234	-0.067				*PRDM16*	Body

cg26562691	16	23,850,404	0.018549	-0.234	-0.240				*PRKCB*	Body
cg18826076	7	48,108,684	0.016263	-0.234	-0.179					
cg05647431	2	241,971,306	0.001434	-0.234	-0.230				*SNED1*	Body
cg10622126	X	147,598,391	0.032382	-0.234	-0.151				*AFF2*	Body
cg03848588	9	32,525,008	4.73E-06	-0.234	-0.219				*DDX58*	Body
cg00554260	2	59,494,314	0.001128	-0.234	-0.176					
cg03854077	4	189,580,596	0.004323	-0.234	-0.185					
cg17001868	7	63,247,871	0.008943	-0.234	-0.189					
cg09319695	6	13,976,724	7.21E-07	-0.234	-0.193				*RNF182*	5'UTR
cg11789371	14	102,551,385	7.25E-05	-0.234	-0.165				*HSP90AA1*	Body
cg06174454	3	50,371,818	2.12E-07	-0.234	-0.224				*RASSF1*	Body
cg20082618	18	19,318,496	2.07E-06	-0.234	-0.203					
cg00337703	10	115,938,729	6.09E-14	-0.234	-0.248				*TDRD1*	TSS1500
cg07459248	17	77,194,008	0.006153	-0.234	-0.142				*HRNBP3*	5'UTR
cg04790749	17	34,123,272	1.13E-09	-0.234	-0.224				*MMP28*	TSS1500
cg07342258	5	2,866,851	0.011405	-0.234	-0.120					
cg19417526	11	131,895,599	0.019015	-0.234	-0.291				*NTM*	Body
cg06641537	7	142,020,422	0.013765	-0.234	-0.271					
cg00394718	8	120,684,921	0.000177	-0.234	-0.212					
cg16559630	7	56,301,065	0.000158	-0.234	-0.220					
cg14072016	10	384,652	0.032028	-0.234	-0.207				*DIP2C*	Body
cg12437821	12	114,852,027	0.001075	-0.234	-0.193					
cg06223834	16	4,103,161	0.003974	-0.234	-0.297				*ADCY9*	Body
cg15061827	1	4,015,423	7.85E-05	-0.234	-0.263					
cg20207276	8	23,428,718	3.52E-07	-0.234	-0.208				*SLC25A37*	Body
cg17070005	8	13,395,492	0.000988	-0.234	-0.199					
cg12914733	11	955,328	1.62E-05	-0.234	-0.266				*AP2A2*	Body
cg20594465	13	58,246,705	0.00186	-0.234	-0.179				*PCDH17*	Body
cg02578929	2	61,414,329	1.57E-05	-0.234	-0.205				*AHSA2*	3'UTR
cg05074133	4	16,686,903	0.010957	-0.234	-0.231				*LDB2*	Body
cg14227762	14	106,053,344	0.001388	-0.234	-0.107					
cg04880751	1	241,695,164	6.83E-08	-0.234	-0.229				*KMO*	TSS1500
cg07283630	15	48,667,436	0.017373	-0.234	-0.201					
cg23326129	8	46,951,404	0.013245	-0.234	-0.189					

cg05230854	8	8,162,989	4.05E-05	-0.234	-0.267					
cg22916646	5	162,672,583	0.0345	-0.234	-0.108					
cg03367622	7	144,967,385	0.003551	-0.234	-0.231					
cg04574507	1	158,301,038	0.032951	-0.234	-0.151	2.91E-15	-0.188		*CD1B*	Body
cg08470264	16	86,580,384	2.89E-06	-0.234	-0.207				*MTHFSD*	Body
cg11853970	1	986,037	6.46E-05	-0.234	-0.192				*AGRN*	Body
cg27480913	11	6,008,246	0.002096	-0.234	-0.235				*OR52L1*	TSS200
cg01233025	11	55,637,839	1.65E-07	-0.234	-0.227					
cg20723705	11	122,118,676	0.009935	-0.234	-0.156					
cg18221016	1	185,934,251	4.83E-08	-0.234	-0.239				*HMCN1*	Body
cg20624941	12	125,811,144	0.006155	-0.234	-0.256				*TMEM132B*	TSS200
cg04105230	15	101,938,681	2.72E-07	-0.234	-0.226				*PCSK6*	Body
cg13697223	4	173,710,219	0.035232	-0.234	-0.125				*GALNTL6*	Body
cg23246095	2	162,931,638	4.44E-08	-0.234	-0.211				*DPP4*	TSS1500
cg23316648	4	17,096,664	0.001341	-0.234	-0.210					
cg05331856	13	40,516,584	5.88E-08	-0.235	-0.254					
cg16521643	16	32,452,502	0.029216	-0.235	-0.140					
cg13496790	3	66,455,738	0.015048	-0.235	-0.255				*LRIG1*	Body
cg03124880	12	108,617,533	0.024569	-0.235	-0.153			Yes	*WSCD2*	Body
cg21966319	4	169,543,126	1.04E-05	-0.235	-0.195				*PALLD*	Body
cg05038881	1	10,490,043	1.36E-14	-0.235	-0.223				*APITD1*	TSS200
cg13439703	9	20,566,875	0.00015	-0.235	-0.208				*MLLT3*	Body
cg20387172	8	145,689,754	1.02E-07	-0.235	-0.198				*CYHR1*	Body
cg10149533	4	154,064,784	7.48E-08	-0.235	-0.236					
cg12612406	6	30,103,459	0.000849	-0.235	-0.150				*TRIM40*	TSS1500
cg10842913	12	125,755,229	0.018463	-0.235	-0.253					
cg15061682	13	102,850,387	0.021268	-0.235	-0.166				*FGF14*	Body
cg26919735	1	152,647,793	0.018754	-0.235	-0.160				*LCE2C*	1stExon
cg03344806	7	18,067,282	0.038075	-0.235	-0.113				*PRPS1L1*	1stExon
cg03461296	20	60,637,948	1.52E-05	-0.235	-0.299				*TAF4*	Body
cg07280272	15	41,230,536	1.77E-39	-0.235	-0.227				*DLL4*	3'UTR
cg05565809	6	31,740,787	1.32E-07	-0.235	-0.197				*C6orf27*	Body
cg07369399	16	50,772,296	0.001408	-0.235	-0.107					
cg08449144	11	1,857,919	7.72E-06	-0.235	-0.196				*SYT8*	Body

cg12968800	2	108,565,893	0.002137	-0.235	-0.141						
cg01022345	14	101,317,819	0.000138	-0.235	-0.127					*MIR770*	TSS1500
cg23982574	12	132,174,431	0.000278	-0.235	-0.176						
cg24853313	X	145,078,200	0.014596	-0.235	-0.160					*MIR892A*	Body
cg27562311	12	8,766,941	0.038589	-0.235	-0.131					*AICDA*	TSS1500
cg14469928	2	125,733,618	0.000601	-0.235	-0.238						
cg26909217	16	72,974,588	1.62E-05	-0.235	-0.225				Yes	*ZFHX3*	Body
cg05855347	7	129,773,086	0.00048	-0.235	-0.156					*KLHDC10*	3'UTR
cg25786780	19	12,957,979	1.58E-07	-0.235	-0.183					*MAST1*	Body
cg01231009	8	130,641,185	0.011552	-0.235	-0.231						
cg00517080	11	134,098,583	3.35E-05	-0.235	-0.145					*VPS26B*	Body
cg15810474	Y	9,306,450	1.97E-06	-0.235	-0.202					*TSPY1*	Body
cg04608582	1	113,798,121	1.56E-06	-0.235	-0.229						
cg07791803	8	34,343,944	0.002859	-0.235	-0.152						
cg05210558	11	131,410,879	0.005503	-0.235	-0.205					*NTM*	Body
cg25753473	6	170,532,835	1.01E-10	-0.235	-0.222						
cg17667592	11	123,496,000	0.010244	-0.235	-0.174						
cg22103831	13	58,256,880	0.001458	-0.235	-0.157				Yes	*PCDH17*	Body
cg18574499	21	40,669,669	2.14E-06	-0.235	-0.261					*BRWD1*	Body
cg10465202	12	8,766,467	0.014932	-0.235	-0.160					*AICDA*	TSS1500
cg01974330	1	196,177,626	0.02785	-0.235	-0.161						
cg03594972	5	3,659,656	0.018568	-0.235	-0.127						
cg08228600	11	120,911,604	5.02E-07	-0.235	-0.262					*TBCEL*	5'UTR
cg18176054	12	93,839,011	2.1E-08	-0.235	-0.236						
cg24703336	12	5,257,426	0.010224	-0.235	-0.160						
cg07891120	4	171,663,610	0.031316	-0.235	-0.207						
cg11538307	5	40,984,654	0.033564	-0.235	-0.213						
cg08085487	X	145,110,794	0.003531	-0.235	-0.132					*MIR891A*	TSS1500
cg16842280	17	10,422,189	5.2E-10	-0.235	-0.213					*MYH1*	TSS1500
cg09796270	17	17,721,594	0.035758	-0.235	-0.245					*SREBF1*	Body
cg05545891	11	56,113,768	0.036757	-0.235	-0.124					*OR8K1*	1stExon
cg17895446	4	173,573,053	0.036832	-0.235	-0.210					*GALNTL6*	Body
cg07436896	13	84,512,421	2.01E-05	-0.235	-0.218						
cg23219332	X	134,971,334	0.000109	-0.235	-0.169					*CT45A6*	TSS200

cg02842704	5	151,305,746	1.29E-06	-0.235	-0.232				*GLRA1*	TSS1500
cg04290171	1	207,924,482	1.4E-06	-0.235	-0.225				*CD46*	TSS1500
cg11868247	7	7,962,810	6.3E-09	-0.235	-0.218					
cg24055855	17	1,535,293	2.8E-05	-0.235	-0.227					
cg24107750	10	6,569,373	0.007057	-0.235	-0.155				*PRKCQ*	5'UTR
cg11813658	14	87,370,892	0.003455	-0.235	-0.211					
cg01015123	3	161,214,690	0.02189	-0.235	-0.191				*OTOL1*	1stExon
cg03267167	19	29,587,012	0.023587	-0.235	-0.140					
cg14573759	18	73,966,755	0.002495	-0.235	-0.202					
cg20566970	3	137,321,245	0.008284	-0.235	-0.314					
cg00347056	8	3,254,000	0.000304	-0.235	-0.186				*CSMD1*	Body
cg13097102	1	172,886,544	0.003649	-0.235	-0.279					
cg21899520	18	71,957,896	0.000183	-0.235	-0.175				*CYB5A*	Body
cg05424333	16	86,718,577	0.006002	-0.235	-0.209					
cg18674899	6	83,904,319	3.83E-05	-0.235	-0.279				*PGM3*	TSS1500
cg24150385	3	66,543,263	1.3E-06	-0.235	-0.224				*LRIG1*	Body
cg05677630	22	22,020,012	4.05E-07	-0.235	-0.200				*PPIL2*	TSS1500
cg12033297	1	176,998,801	0.000522	-0.235	-0.222				*MIR488*	TSS1500
cg18116418	1	152,553,244	5.47E-10	-0.235	-0.223			Yes	*LCE3D*	TSS1500
cg03752741	15	84,168,459	2.31E-12	-0.235	-0.229				*SH3GL3*	Body
cg11012557	14	70,512,583	1.54E-05	-0.235	-0.200				*SLC8A3*	3'UTR
cg07810733	12	66,324,092	2.57E-06	-0.235	-0.247				*HMGA2*	Body
cg13555050	4	111,985,640	0.000433	-0.235	-0.169					
cg15961219	14	53,960,406	2.16E-07	-0.235	-0.263					
cg18599533	19	58,627,297	0.01152	-0.235	-0.181				*ZSCAN18*	Body
cg22862319	1	3,289,994	1.74E-05	-0.235	-0.200				*PRDM16*	Body
cg16836250	11	87,224,821	0.000793	-0.235	-0.239					
cg25912428	10	125,427,770	0.000617	-0.235	-0.198				*GPR26*	Body
cg14181111	1	168,773,313	9.87E-06	-0.235	-0.233					
cg15304679	7	53,104,001	0.013574	-0.235	-0.149				*POM121L12*	1stExon
cg24456981	10	26,930,863	0.005324	-0.235	-0.224				*LOC731789*	TSS1500
cg02539662	12	93,560,520	0.000532	-0.236	-0.284					
cg24764810	6	31,025,949	0.000795	-0.236	-0.144				*HCG22*	Body
cg13412154	12	127,941,600	7.01E-06	-0.236	-0.187					

cg22868951	4	129,767,588	0.001626	-0.236	-0.204				*PHF17*	Body
cg25765936	8	58,130,041	0.01488	-0.236	-0.178					
cg20703895	1	157,407,802	0.040933	-0.236	-0.206					
cg24338513	1	210,072,223	0.00042	-0.236	-0.173					
cg24536691	6	33,238,768	5.14E-05	-0.236	-0.229				*VPS52*	Body
cg03861532	20	56,275,607	0.01916	-0.236	-0.274				*PMEPA1*	5'UTR
cg24826784	6	131,952,764	6.92E-06	-0.236	-0.208					
cg25562958	14	101,490,676	0.023591	-0.236	-0.222				*MIR1197*	TSS1500
cg18577239	12	53,955,664	2.4E-07	-0.236	-0.247				*ATF7*	Body
cg06700060	12	65,859,647	0.007552	-0.236	-0.226				*MSRB3*	3'UTR
cg04136817	17	32,369,686	0.026043	-0.236	-0.133				*ACCN1*	Body
cg11970733	12	129,469,367	0.00255	-0.236	-0.166				*GLT1D1*	3'UTR
cg16691898	1	156,255,600	0.011358	-0.236	-0.258				*TMEM79*	Body
cg03137700	2	32,905,314	3.16E-09	-0.236	-0.260				*TTC27*	Body
cg03379894	5	141,131,868	5.11E-07	-0.236	-0.201					
cg09295202	14	21,535,394	9.39E-05	-0.236	-0.176					
cg20681783	5	8,839,868	0.000141	-0.236	-0.221					
cg00874605	16	14,569,598	1.11E-08	-0.236	-0.243				*PARN*	Body
cg24380053	3	13,028,632	8.86E-13	-0.236	-0.240				*IQSEC1*	Body
cg02224329	2	185,740,590	0.003677	-0.236	-0.275				*ZNF804A*	Body
cg15073065	12	34,530,380	4.73E-05	-0.236	-0.190					
cg20267897	20	62,433,369	0.010724	-0.236	-0.149				*ZBTB46*	5'UTR
cg07711387	16	51,529,443	0.01015	-0.236	-0.181					
cg21166092	5	1,763,818	2.65E-10	-0.236	-0.219					
cg18311633	2	155,309,539	0.000193	-0.236	-0.138				*GALNT13*	3'UTR
cg16506887	11	6,867,337	0.019913	-0.236	-0.271				*OR10A5*	1stExon
cg18348318	4	7,736,837	0.001234	-0.236	-0.148				*SORCS2*	Body
cg20070090	1	153,363,489	9.14E-07	-0.236	-0.249	0.00207367	-0.043		*S100A8*	5'UTR
cg21656251	2	240,031,700	4.57E-12	-0.236	-0.218				*HDAC4*	Body
cg00239171	4	136,198,747	0.001715	-0.236	-0.228					
cg05918292	14	47,834,520	0.000179	-0.236	-0.244				*MDGA2*	Body
cg13711538	13	77,283,725	0.043262	-0.236	-0.196					
cg06738239	1	2,789,089	0.007589	-0.236	-0.151					
cg16855440	19	45,842,723	1.49E-06	-0.236	-0.212				*KLC3*	TSS1500

cg07629125	13	58,299,015	0.004429	-0.236	-0.159				*PCDH17*	Body
cg12799790	10	54,071,134	0.002673	-0.236	-0.192					
cg27100471	11	794,834	1.3E-08	-0.236	-0.234				*SLC25A22*	Body
cg16911872	7	152,591,175	5.33E-05	-0.236	-0.169					
cg08746033	4	175,897,327	0.042627	-0.236	-0.123				*ADAM29*	Body
cg17185705	8	89,123,876	0.005815	-0.236	-0.139				*MMP16*	Body
cg13550670	14	70,547,432	0.001555	-0.236	-0.235				*SLC8A3*	Body
cg27270968	16	48,846,874	0.02024	-0.236	-0.230			Yes		
cg20207414	12	55,423,208	0.000189	-0.236	-0.174				*NEUROD4*	3'UTR
cg00431835	16	86,796,158	0.007486	-0.236	-0.180					
cg10838380	10	1,156,113	0.04261	-0.236	-0.236				*WDR37*	Body
cg19315730	4	49,320,210	0.023685	-0.236	-0.121					
cg14571619	3	100,537,801	0.001211	-0.236	-0.159				*ABI3BP*	Body
cg15711987	10	99,393,886	1.91E-05	-0.236	-0.193				*MORN4*	1stExon
cg03015672	10	32,216,066	0.001937	-0.236	-0.263				*ARHGAP12*	5'UTR
cg19274618	12	50,240,958	7.56E-06	-0.236	-0.208					
cg22258892	17	77,042,315	1.78E-10	-0.236	-0.211				*C1QTNF1*	5'UTR
cg04071644	X	149,613,662	0.001214	-0.236	-0.282				*MAMLD1*	TSS200
cg07340651	11	94,038,819	0.009867	-0.236	-0.261				*FOLR4*	1stExon
cg09325375	11	22,450,715	0.003225	-0.236	-0.222					
cg26151163	2	239,079,168	2.38E-07	-0.236	-0.208				*ILKAP*	3'UTR
cg00364325	15	89,342,191	0.005501	-0.236	-0.145					
cg10269560	16	88,133,238	1.45E-12	-0.236	-0.205					
cg03043911	X	86,771,551	0.005698	-0.236	-0.206				*KLHL4*	TSS1500
cg05862007	2	48,950,153	0.030114	-0.236	-0.162				*GTF2A1L*	Body
cg25925578	8	8,388,047	0.001636	-0.236	-0.283					
cg09555879	19	42,569,956	0.013233	-0.236	-0.153	0.0000017	-0.100		*GRIK5*	1stExon
cg09640686	16	89,496,981	1.39E-06	-0.236	-0.210				*ANKRD11*	5'UTR
cg22226342	21	31,693,907	5.02E-07	-0.236	-0.222				*KRTAP26-1*	TSS1500
cg23521262	10	114,323,333	1.23E-08	-0.236	-0.228				*VTI1A*	Body
cg26901096	10	10,994,189	0.027425	-0.236	-0.200				*LOC254312*	TSS200
cg06634886	5	5,033,763	0.022391	-0.236	-0.184				*LOC340094*	TSS1500
cg23638725	7	48,965,301	0.00133	-0.236	-0.205				*CDC14C*	Body
cg01027010	16	31,576,339	0.000412	-0.236	-0.199					

cg17936359	3	168,524,260	0.003231	-0.236	-0.175				*C3orf50*	Body
cg21831485	7	31,594,014	2.26E-07	-0.236	-0.238				*CCDC129*	Body
cg23377607	17	39,728,362	0.006813	-0.236	-0.125				*KRT9*	TSS200
cg05776787	3	38,835,276	0.045865	-0.236	-0.138				*SCN10A*	1stExon
cg25015038	14	89,604,062	5.75E-16	-0.236	-0.249					
cg08019151	3	38,836,991	0.002836	-0.236	-0.140				*SCN10A*	TSS1500
cg12298697	6	32,850,425	1.32E-10	-0.236	-0.236					
cg18296784	11	116,147,929	0.000435	-0.236	-0.166					
cg02661623	7	105,603,743	3.53E-09	-0.236	-0.234				*FLJ23834*	1stExon
cg23452220	4	16,846,297	5.44E-06	-0.236	-0.243				*LDB2*	Body
cg06647930	22	45,135,965	0.000266	-0.236	-0.201				*PRR5-ARHGAP8*	Body
cg15734955	17	79,977,477	0.04274	-0.236	-0.091				*STRA13*	Body
cg27325772	Y	9,178,472	0.000116	-0.236	-0.184					
cg00174201	7	10,819,370	0.010537	-0.236	-0.138					
cg00242951	3	9,596,482	0.000657	-0.236	-0.203				*LHFPL4*	TSS1500
cg01538165	8	71,240,128	4.18E-09	-0.236	-0.241				*NCOA2*	5'UTR
cg13837822	10	26,931,731	0.01811	-0.236	-0.157				*LOC731789*	TSS1500
cg12989041	6	159,049,761	0.006931	-0.236	-0.280				*TMEM181*	Body
cg01713348	16	1,480,790	1.21E-06	-0.236	-0.251				*C16orf91*	TSS1500
cg13519373	2	219,030,898	0.009858	-0.237	-0.182				*CXCR1*	5'UTR
cg16746901	11	2,428,431	0.0173	-0.237	-0.146				*TRPM5*	Body
cg26674487	3	9,590,728	0.009947	-0.237	-0.175				*LHFPL4*	Body
cg26144569	2	38,304,804	0.000133	-0.237	-0.241				*CYP1B1*	TSS1500
cg26254050	5	25,193,483	0.011959	-0.237	-0.196					
cg05834895	3	69,172,203	4.29E-11	-0.237	-0.237				*LMOD3*	TSS1500
cg07213415	8	144,254,866	1.13E-06	-0.237	-0.241					
cg09158638	16	62,309,996	0.014757	-0.237	-0.145					
cg16626760	6	41,621,322	7.55E-11	-0.237	-0.213				*MDFI*	3'UTR
cg19026320	21	31,588,354	4.29E-06	-0.237	-0.227				*CLDN8*	TSS200
cg26446133	18	72,167,187	3.71E-06	-0.237	-0.170				*CNDP2*	5'UTR
cg08849514	5	106,530,218	0.035804	-0.237	-0.072					
cg18839416	1	12,805,849	5.87E-10	-0.237	-0.240				*C1orf158*	TSS1500
cg04281426	5	69,345,099	3.85E-08	-0.237	-0.255				*SMN2*	TSS1500
cg09469812	1	2,899,497	0.000686	-0.237	-0.161					

cg17162911	8	13,209,750	0.029697	-0.237	-0.237				*DLC1*	Body
cg03963963	10	44,434,410	0.008269	-0.237	-0.229					
cg22164298	4	114,276,834	0.021462	-0.237	-0.099				*ANK2*	Body
cg27475652	8	24,774,727	0.004112	-0.237	-0.160				*NEFM*	Body
cg03319695	18	59,156,627	0.001508	-0.237	-0.170				*CDH20*	TSS1500
cg12575429	17	77,029,518	0.001396	-0.237	-0.112				*C1QTNF1*	5'UTR
cg20333727	11	1,889,893	0.00028	-0.237	-0.194				*LSP1*	TSS200
cg13137032	4	5,904,897	0.025425	-0.237	-0.122					
cg16298813	1	2,878,455	0.023708	-0.237	-0.101					
cg07616471	3	46,413,711	2.88E-09	-0.237	-0.224				*CCR5*	5'UTR
cg11001769	14	61,748,440	2.2E-05	-0.237	-0.246				*TMEM30B*	1stExon
cg13123025	4	190,661,960	0.018812	-0.237	-0.153					
cg05829696	8	140,488,084	0.005901	-0.237	-0.139					
cg00653455	3	53,914,075	0.004519	-0.237	-0.263				*ACTR8*	Body
cg24087039	4	62,788,690	3.61E-05	-0.237	-0.170				*LPHN3*	Body
cg27410383	16	29,179,329	0.002596	-0.237	-0.156					
cg11465466	17	39,549,192	0.031556	-0.237	-0.084					
cg14838801	2	234,385,359	0.000548	-0.237	-0.157				*USP40*	3'UTR
cg01793704	7	130,126,024	9.36E-06	-0.237	-0.252				*MEST*	TSS200
cg01794405	1	198,869,002	0.013302	-0.237	-0.113					
cg23313963	6	32,023,923	0.033085	-0.237	-0.093				*TNXB*	Body
cg00233633	7	29,153,560	4.9E-05	-0.237	-0.230				*CPVL*	Body
cg19886365	8	56,159,797	0.029417	-0.237	-0.223				*XKR4*	Body
cg16219583	5	135,702,333	0.000235	-0.237	-0.198			Yes	*TRPC7*	TSS1500
cg06332269	19	1,978,692	0.006109	-0.237	-0.213				*CSNK1G2*	Body
cg25528806	11	124,190,035	5.71E-05	-0.237	-0.234				*OR8D2*	1stExon
cg05465955	17	40,715,236	1.6E-13	-0.237	-0.222				*COASY*	1stExon
cg12641024	17	40,070,691	1.96E-08	-0.237	-0.235				*ACLY*	5'UTR
cg06455190	13	19,451,923	0.039667	-0.237	-0.143					
cg21201572	7	16,844,606	0.007282	-0.237	-0.312				*AGR2*	5'UTR
cg01246444	2	161,775,589	2.66E-11	-0.237	-0.222					
cg17130745	1	81,797,258	8.22E-13	-0.237	-0.254					
cg20784899	7	32,341,352	0.022176	-0.237	-0.111					
cg02008627	16	6,318,633	0.010271	-0.237	-0.188				*A2BP1*	5'UTR

cg07060505	12	116,756,136	6.21E-08	-0.237	-0.245						
cg08500510	10	6,216,350	0.000898	-0.237	-0.183					*PFKFB3*	Body
cg09529437	16	24,136,792	1.24E-06	-0.237	-0.231					*PRKCB*	Body
cg25793597	10	9,450,986	5.69E-05	-0.237	-0.244						
cg00175487	14	101,490,186	0.014672	-0.237	-0.215					*MIR380*	TSS1500
cg12611013	13	103,047,760	3.15E-06	-0.237	-0.185					*FGF14*	Body
cg20899213	3	142,546,545	0.00769	-0.237	-0.106					*PCOLCE2*	Body
cg07581462	4	178,038,657	7.87E-05	-0.237	-0.214						
cg25002129	11	131,965,815	0.013038	-0.237	-0.207					*NTM*	Body
cg22514075	1	99,357,073	0.00023	-0.237	-0.284					*LPPR5*	3'UTR
cg07725167	11	66,185,078	0.004438	-0.237	-0.230						
cg26640927	4	120,726,319	4.37E-05	-0.237	-0.241						
cg04066021	16	86,293,287	2.49E-05	-0.237	-0.231						
cg06076518	8	130,307,191	0.011788	-0.237	-0.212						
cg13379558	19	14,797,552	0.001033	-0.237	-0.178						
cg23885889	10	19,400,365	4.67E-05	-0.237	-0.201						
cg00444360	11	88,270,612	0.003391	-0.237	-0.156					*GRM5*	Body
cg00013660	9	140,068,770	0.001065	-0.237	-0.154						
cg08478046	19	42,093,086	5.96E-05	-0.237	-0.217					*CEACAM21*	3'UTR
cg22215836	17	81,026,716	0.00027	-0.237	-0.152						
cg26745038	16	86,371,833	0.002343	-0.237	-0.199					*LOC732275*	Body
cg09075515	8	56,192,721	0.002761	-0.237	-0.248					*XKR4*	Body
cg01046833	11	51,515,596	0.000627	-0.237	-0.216				Yes	*OR4C46*	1stExon
cg11460714	7	63,463,421	5.4E-05	-0.237	-0.187						
cg03770410	20	57,766,332	0.002656	-0.237	-0.165					*ZNF831*	1stExon
cg25712758	19	8,271,179	0.000101	-0.237	-0.193						
cg18087023	1	153,014,956	0.001745	-0.237	-0.210					*SPRR2D*	TSS1500
cg27645294	17	21,795,257	0.026625	-0.237	-0.105						
cg10012711	6	28,553,927	0.016527	-0.237	-0.147					*SCAND3*	Body
cg10132287	10	131,584,334	4.37E-05	-0.237	-0.205						
cg16199460	5	5,087,391	1.89E-08	-0.237	-0.215						
cg22319016	11	86,663,418	1.14E-09	-0.237	-0.210					*FZD4*	Body
cg04692506	11	47,401,027	1.43E-10	-0.237	-0.240					*SPI1*	TSS1500
cg04357225	14	98,153,758	0.014715	-0.237	-0.168						

cg08913410	7	30,726,859	5.42E-09	-0.237	-0.214					
cg00736299	16	4,730,465	6.47E-09	-0.237	-0.204			Yes	*MGRN1*	Body
cg22433447	7	39,288,391	0.00018	-0.237	-0.210				*POU6F2*	Body
cg08245989	12	9,280,923	0.006728	-0.238	-0.170					
cg26311677	3	168,142,949	0.003622	-0.238	-0.181				*C3orf50*	Body
cg07989466	12	64,797,213	6.41E-08	-0.238	-0.241				*XPOT*	TSS1500
cg16464328	17	42,346,923	3.01E-08	-0.238	-0.232				*SLC4A1*	TSS1500
cg19821682	1	4,849,063	0.006543	-0.238	-0.156					
cg24327361	8	4,583,386	0.004795	-0.238	-0.222				*CSMD1*	Body
cg13897627	16	49,378,497	0.011009	-0.238	-0.161	0.0000027	-0.110			
cg21605238	13	39,264,965	0.009231	-0.238	-0.238				*FREM2*	1stExon
cg24803687	11	5,462,916	0.001817	-0.238	-0.217				*OR51I1*	TSS200
cg24995678	2	235,936,394	5.08E-07	-0.238	-0.216				*SH3BP4*	5'UTR
cg18125814	11	49,455,214	0.000451	-0.238	-0.188					
cg18186478	8	23,423,960	1.93E-05	-0.238	-0.199				*SLC25A37*	Body
cg16112050	18	713,200	0.000508	-0.238	-0.281	1.57E-15	-0.140		*ENOSF1*	TSS1500
cg21871038	6	50,697,940	0.000307	-0.238	-0.227			Yes	*TFAP2D*	Body
cg19964491	6	32,016,236	0.03476	-0.238	-0.050				*TNXB*	Body
cg19656820	11	34,535,085	0.015307	-0.238	-0.243				*ELF5*	5'UTR
cg22852840	6	47,749,714	5.04E-05	-0.238	-0.231				*OPN5*	TSS200
cg06859106	4	17,582,677	0.006927	-0.238	-0.220			Yes	*LAP3*	Body
cg06515543	19	5,823,791	0.003853	-0.238	-0.176				*NRTN*	TSS200
cg02413104	X	150,395,248	9.21E-06	-0.238	-0.240					
cg14346895	9	37,576,819	4.58E-09	-0.238	-0.231				*FBXO10*	TSS1500
cg00014104	22	22,220,367	1.81E-12	-0.238	-0.238				*MAPK1*	Body
cg01165661	3	49,157,146	2.04E-05	-0.238	-0.216				*USP19*	5'UTR
cg19437852	19	56,195,465	2.25E-05	-0.238	-0.237				*EPN1*	Body
cg03462901	15	93,613,453	1.07E-05	-0.238	-0.253				*RGMA*	Body
cg27338353	22	46,471,129	2.51E-16	-0.238	-0.218					
cg09561365	22	45,676,755	1.82E-05	-0.238	-0.226					
cg09043962	17	79,694,615	1.36E-05	-0.238	-0.246					
cg26152051	2	219,994,830	4.27E-08	-0.238	-0.226				*NHEJ1*	Body
cg27247689	8	73,445,394	1.55E-05	-0.238	-0.241					
cg06465306	X	84,503,836	8.3E-06	-0.238	-0.185				*ZNF711*	Body

cg14139814	7	83,378,795	0.03441	-0.238	-0.191					
cg03452625	3	11,537,620	0.041838	-0.238	-0.223				*ATG7*	Body
cg15975283	1	173,571,345	0.012857	-0.238	-0.270				*SLC9A11*	5'UTR
cg00134787	17	10,419,499	0.006621	-0.238	-0.189	0.0000704	-0.140		*MYH1*	Body
cg00090787	15	66,795,345	8.69E-09	-0.238	-0.235				*RPL4*	Body
cg03284670	16	55,909,732	0.00632	-0.238	-0.142				*CES7*	TSS1500
cg16847374	X	152,772,343	1.22E-05	-0.238	-0.205				*BGN*	Body
cg17770910	8	143,851,427	0.03899	-0.238	-0.156				*LYNX1*	TSS200
cg03579179	7	12,444,095	1.73E-06	-0.238	-0.244				*VWDE*	TSS1500
cg06627043	1	147,838,662	1.65E-08	-0.238	-0.201					
cg27296413	1	159,037,843	0.008148	-0.238	-0.267				*AIM2*	Body
cg01518790	20	9,823,348	0.005597	-0.238	-0.137					
cg06617217	12	56,708,578	1.86E-07	-0.238	-0.242				*CNPY2*	Body
cg14128411	2	1,926,907	0.01011	-0.238	-0.283				*MYT1L*	Body
cg24935655	3	193,097,750	0.049865	-0.238	-0.092				*ATP13A5*	TSS1500
cg25799589	4	146,800,380	1.9E-12	-0.238	-0.240				*ZNF827*	Body
cg21997401	4	12,225,077	0.008658	-0.238	-0.258					
cg01761453	X	140,983,037	0.019217	-0.238	-0.157				*MAGEC3*	Body
cg08026039	16	1,486,384	0.014766	-0.238	-0.111				*CCDC154*	Body
cg14909213	12	8,694,468	0.008138	-0.238	-0.145				*CLEC4E*	TSS1500
cg13050115	3	73,503,670	2.98E-07	-0.238	-0.249				*PDZRN3*	Body
cg02282041	16	82,908,556	0.0025	-0.238	-0.227				*CDH13*	Body
cg01916400	5	155,548,458	3.3E-13	-0.238	-0.226					
cg03228766	7	57,878,945	0.00082	-0.238	-0.206					
cg05749728	10	126,826,902	6.59E-05	-0.238	-0.195				*CTBP2*	5'UTR
cg13631222	3	147,654,565	0.001866	-0.238	-0.190			Yes		
cg14624463	3	153,947,356	0.006939	-0.238	-0.115				*SGEF*	Body
cg11472422	17	80,926,014	0.000538	-0.238	-0.259				*B3GNTL1*	Body
cg07826516	5	3,106,230	0.029288	-0.238	-0.116					
cg19740476	12	130,750,328	0.035615	-0.238	-0.122					
cg26014066	X	66,763,094	9.08E-07	-0.238	-0.218				*AR*	TSS1500
cg19029127	4	37,604,497	4.92E-11	-0.238	-0.196				*RELL1*	3'UTR
cg21684179	1	110,576,933	0.002549	-0.238	-0.165				*FAM40A*	TSS1500
cg03582419	4	177,804,777	0.018822	-0.238	-0.136					

cg08833126	4	180,086,254	1.18E-05	-0.238	-0.252					
cg14030940	14	101,906,274	0.004198	-0.238	-0.182					
cg26354750	10	38,499,434	9.63E-06	-0.238	-0.213				*LOC100129055*	Body
cg14126408	10	44,705,342	0.004455	-0.238	-0.258					
cg19809669	6	151,346,268	0.002597	-0.238	-0.190				*MTHFD1L*	Body
cg13290059	2	104,781,890	0.018423	-0.238	-0.234					
cg05784226	2	91,923,833	0.034593	-0.238	-0.104					
cg07151197	13	87,098,885	0.000195	-0.238	-0.188					
cg14150803	1	8,035,305	3.99E-10	-0.238	-0.229				*PARK7*	Body
cg18161211	4	177,716,461	0.000451	-0.238	-0.241					
cg22614624	2	242,618,417	2.5E-06	-0.238	-0.228				*DTYMK*	Body
cg10652558	7	57,208,277	0.000108	-0.238	-0.208				*ZNF479*	TSS1500
cg11534519	5	171,534,678	5.93E-05	-0.238	-0.163				*STK10*	Body
cg12492670	11	50,231,261	0.006253	-0.238	-0.156					
cg00133403	16	57,212,731	4.83E-06	-0.238	-0.194				*FAM192A*	5'UTR
cg07795402	1	2,938,954	0.03026	-0.238	-0.068				*ACTRT2*	1stExon
cg24197116	X	68,110,972	8.35E-07	-0.238	-0.251					
cg09884146	2	65,593,908	1.38E-22	-0.239	-0.229				*SPRED2*	5'UTR
cg18748085	5	22,372,872	0.003299	-0.239	-0.191				*CDH12*	5'UTR
cg09450467	22	39,711,262	0.049495	-0.239	-0.144				*SNORD83B*	TSS1500
cg12905592	12	50,091,449	1.16E-08	-0.239	-0.223				*FMNL3*	Body
cg25133753	20	44,299,726	0.009633	-0.239	-0.214				*WFDC11*	TSS1500
cg11958666	8	5,019,242	0.007572	-0.239	-0.180					
cg09261791	14	52,347,968	8.12E-06	-0.239	-0.247				*GNG2*	5'UTR
cg09775238	16	86,580,410	4.26E-05	-0.239	-0.214				*MTHFSD*	Body
cg00268869	11	91,884,576	0.001655	-0.239	-0.252					
cg00863469	19	49,116,423	0.000311	-0.239	-0.247				*FAM83E*	1stExon
cg16383871	6	31,888,543	0.004673	-0.239	-0.204					
cg19880077	12	131,975,791	0.049193	-0.239	-0.194					
cg24174145	12	52,585,892	4.52E-09	-0.239	-0.207				*KRT80*	TSS200
cg10575986	7	154,003,309	0.020352	-0.239	-0.159				*DPP6*	Body
cg03804732	12	14,136,365	0.023412	-0.239	-0.233					
cg18548400	3	182,506,874	8.29E-05	-0.239	-0.244					
cg20978858	22	41,653,226	5.04E-07	-0.239	-0.207				*RANGAP1*	Body

cg19911494	5	9,169,758	0.00094	-0.239	-0.174					*SEMA5A*	Body
cg20949198	6	134,317,925	0.000101	-0.239	-0.248					*SLC2A12*	Body
cg12610078	3	82,547,324	0.007098	-0.239	-0.182						
cg12169568	14	89,708,532	1.62E-05	-0.239	-0.177					*FOXN3*	Body
cg23257105	11	45,671,015	0.004265	-0.239	-0.093					*CHST1*	3'UTR
cg20615517	17	3,213,559	0.013671	-0.239	-0.105					*OR3A4*	Body
cg25986727	11	71,723,428	0.001297	-0.239	-0.180					*NUMA1*	Body
cg27289628	19	29,458,302	0.031032	-0.239	-0.140					*LOC148145*	Body
cg06059043	16	559,311	9.11E-11	-0.239	-0.248					*RAB11FIP3*	Body
cg20521198	4	27,102,907	4.02E-07	-0.239	-0.248						
cg17450075	12	130,411,204	0.002066	-0.239	-0.195						
cg11576988	6	26,544,615	8.1E-12	-0.239	-0.220					*HMGN4*	5'UTR
cg11592631	2	119,470,715	0.000371	-0.239	-0.233						
cg12163365	8	140,465,582	0.014208	-0.239	-0.157						
cg15050328	3	124,448,651	4.33E-06	-0.239	-0.253					*UMPS*	TSS1500
cg27258696	11	20,987,359	0.002098	-0.239	-0.256					*NELL1*	Body
cg01997606	5	151,308,141	0.000183	-0.239	-0.235						
cg03954343	12	15,822,734	6.03E-06	-0.239	-0.255					*EPS8*	Body
cg26561570	1	161,602,002	0.002117	-0.239	-0.212					*FCGR3B*	TSS1500
cg03447413	16	86,912,782	0.00162	-0.239	-0.144						
[truncated: 383,662 more chars]
