# Supplementary material for: Identification of New Differentially Methylated Genes That Have Potential Functional Consequences in Prostate Cancer
Source: PLoS One. 2012 Oct 31;7(10):e48455. doi: 10.1371/journal.pone.0048455 (PMC3485209; doi:10.1371/journal.pone.0048455)
Supplement: Table S4 — Comparison of differentially methylated genes between genome-wide profiles and 67 known methylated genes in PCa. (PDF) [file pone.0048455.s011.pdf]

Table S4. Comparison of differentially methylated genes between genome-wide profiles and 67 known methylated genes in PCa.

	HM450	N-NGS[1]	HM27
No. of indentified genes*	2874	2318	363
No. of common genes	32	20	15

* The number of promoter hypermethylated genes in each data set.

[1] Cancer-specific methylation only (Group 1 and 2 only)
