# Supplementary material for: Identification of New Differentially Methylated Genes That Have Potential Functional Consequences in Prostate Cancer
Source: PLoS One. 2012 Oct 31;7(10):e48455. doi: 10.1371/journal.pone.0048455 (PMC3485209; doi:10.1371/journal.pone.0048455)
Supplement: Table S5 — Differentially methylated genes by combination of DNA methylation and gene expression data. (PDF) [file pone.0048455.s012.pdf]

Table S5. Differentially methylated genes by combination of DNA methylation and gene expression data.

Gene Symbol	Gene expression[1]		HM450		HM27[1]		M-NGS[1]	Known methylated gene
	FDR *P*	ΔLog Intensity[2]	No. of DMC[3]	$\Delta\beta$[2]	FDR *P*	$\Delta\beta$[2]		
AOX1	1.5E-08	-1.07	11	0.52	2.8E-23	0.33	cDMR	
GSTP1	1.1E-09	-1.05	3	0.48	3.6E-28	0.31		Yes
CAV1	4.0E-09	-1.04	8	0.43	9.5E-26	0.24		Yes
FABP3	1.5E-08	-0.76	5	0.41				
TIMP2	5.0E-06	-0.73	3	0.57			cDMR	
GSTM2	2.4E-06	-0.69	6	0.44	1.8E-19	0.23	cDMR	
ANXA2	5.2E-07	-0.69	7	0.50			cDMR	
EFEMP1	7.6E-07	-0.68	5	0.47	1.2E-27	0.28	cDMR	
ANXA6	3.2E-07	-0.66	5	0.52	1.3E-30	0.29	cDMR	
ACSF2	7.8E-10	-0.64	3	0.49	4.0E-33	0.29	cDMR	
ACSS3	2.8E-07	-0.60	13	0.40	3.7E-24	0.21		
TMLHE	1.4E-08	-0.60	8	0.42	2.6E-27	0.24	cDMR	
C5orf4	7.7E-10	-0.60	5	0.48	7.1E-26	0.24		
WFDC2	3.7E-08	-0.60	7	0.48	1.0E-27	0.22	cDMR	
CRABP2	6.0E-07	-0.59	3	0.40			cDMR	
HLF	1.4E-06	-0.57	4	0.40			cDMR	
GPRC5B	1.2E-07	-0.56	2	0.53			cDMR	
GLI3	1.2E-07	-0.56	4	0.53				
EYA4	6.5E-07	-0.55	16	0.43	9.5E-16	0.17	cDMR	
PRICKLE2	3.2E-08	-0.55	3	0.41	1.0E-07	0.10		
FIBIN	2.4E-03	-0.52	6	0.44	3.5E-33	0.31		
COL4A6	1.1E-09	-0.50	2	0.57				
ITIH5	7.6E-08	-0.48	4	0.42			cDMR	
ZNF154	2.5E-08	-0.48	9	0.40	2.9E-21	0.33	cDMR	
PRKCB	5.9E-05	-0.47	4	0.49	5.9E-15	0.22		
LAYN	3.1E-07	-0.47	9	0.44			cDMR	
NUAK1	5.9E-07	-0.46	8	0.59	8.4E-31	0.28	cDMR	
RARB	1.4E-06	-0.45	18	0.60	1.6E-27	0.33		Yes
RAPGEFL1	3.2E-07	-0.45	7	0.42				
MARVELD1	1.5E-08	-0.45	4	0.48				
GPRASP1	2.7E-09	-0.44	10	0.43	8.5E-29	0.20		
NNT	6.0E-07	-0.44	4	0.53			cDMR	
PFKP	1.0E-05	-0.44	4	0.50				
CD200	3.0E-04	-0.43	4	0.40	6.7E-12	0.15		
COL4A5	9.1E-10	-0.43	2	0.43				
FBXO30	1.5E-06	-0.43	4	0.50				
IFITM1	2.3E-04	-0.42	3	0.41				
CLIP4	4.1E-05	-0.41	9	0.57	1.2E-32	0.28		
STK33	2.3E-06	-0.41	4	0.41				
MMP2	4.5E-03	-0.41	4	0.42	3.5E-24	0.20	cDMR	
RNF152	5.9E-07	-0.40	8	0.49	9.3E-25	0.28	cDMR	
FHL2	5.6E-06	-0.39	7	0.40	2.3E-25	0.22	cDMR	

APOBEC3G	1.5E-08	-0.38	2	0.42				
SPARC	2.0E-02	-0.38	6	0.47	6.1E-22	0.22	cDMR	
SLC16A5	2.2E-07	-0.37	9	0.44	7.4E-37	0.27		
FGFR1	1.0E-05	-0.37	3	0.49				
MFAP4	2.3E-02	-0.36	7	0.42	7.7E-16	0.12		
FBLN5	2.8E-02	-0.36	3	0.50	2.5E-22	0.14		
ADD3	1.7E-02	-0.35	3	0.48			cDMR	
BMP7	2.3E-04	-0.35	6	0.42				
RAI14	1.9E-03	-0.33	2	0.45				
CYP27A1	6.3E-03	-0.33	4	0.58			cDMR	
SERPINB1	1.7E-03	-0.33	9	0.47	4.6E-21	0.26	cDMR	
ZNF660	2.8E-07	-0.32	7	0.42	2.9E-11	0.21	cDMR	
BCAR3	2.0E-08	-0.32	2	0.41	4.6E-27	0.21		
EFS	1.5E-07	-0.32	11	0.50	8.1E-33	0.23		
PODN	1.1E-02	-0.32	4	0.43	7.2E-23	0.26	cDMR	
LDB1	1.4E-05	-0.32	5	0.52				
C2orf43	2.0E-03	-0.32	8	0.43	7.7E-22	0.19		
FBXO17	2.5E-08	-0.32	6	0.44				
TRIP6	4.5E-06	-0.30	3	0.56				
SPRY4	3.6E-02	-0.30	5	0.50			cDMR	
VIM	2.3E-02	-0.30	11	0.42			cDMR	
PRKY	6.1E-06	-0.29	3	0.57			cDMR	
PLEKHH2	4.2E-05	-0.28	3	0.43				
KLF8	5.2E-07	-0.28	12	0.52	8.2E-24	0.28		
PDGFRA	2.3E-02	-0.27	5	0.42			cDMR	
DDX26B	1.5E-06	-0.27	3	0.43				
PRIMA1	5.6E-04	-0.25	3	0.45	9.1E-21	0.28		Yes
HOPX	6.6E-04	-0.25	7	0.50			cDMR	
C16orf5	5.2E-04	-0.24	4	0.41			cDMR	
HAAO	6.2E-05	-0.24	10	0.45	5.8E-19	0.31	cDMR	
IQCA1	2.8E-06	-0.24	2	0.41			cDMR	
C2orf88	2.6E-04	-0.23	10	0.52				
PROM1	4.6E-02	-0.23	9	0.46				
MACF1	3.3E-03	-0.23	7	0.50	2.3E-23	0.17		
EVC2	2.2E-06	-0.22	7	0.46			cDMR	
AKR1B1	8.6E-07	-0.22	10	0.40	1.8E-15	0.26	cDMR	
LOC644538	6.6E-04	-0.21	5	0.46				
ZNF655	4.6E-03	-0.21	14	0.46	8.5E-19	0.13	cDMR	
TMEM106A	1.3E-05	-0.21	8	0.54	6.4E-25	0.17	cDMR	
N4BP2L1	8.5E-05	-0.19	2	0.42			cDMR	
SCGB3A1	2.7E-03	-0.19	7	0.54	1.3E-25	0.20		Yes
DSE	9.2E-03	-0.19	4	0.43	2.8E-23	0.24		
PLD3	1.2E-03	-0.19	3	0.49				
APC	1.9E-02	-0.19	15	0.43	5.3E-10	0.26	cDMR	Yes
CHST3	4.7E-03	-0.19	4	0.60				
HOXA7	5.1E-04	-0.18	9	0.54	5.5E-16	0.18		
ELF4	9.0E-05	-0.18	6	0.52	1.6E-31	0.34		

CBX5	2.5E-02	-0.18	5	0.43			cDMR	
NFATC3	2.6E-03	-0.18	3	0.48	3.8E-41	0.24	cDMR	
ALDH1L1	1.2E-02	-0.17	10	0.52				
FAIM2	2.6E-02	-0.17	6	0.44	1.3E-21	0.25	cDMR	
LRRC17	4.9E-03	-0.17	6	0.43	1.8E-23	0.27		
CYBA	4.3E-02	-0.17	13	0.56	2.8E-33	0.36		
DHRS4L2	5.8E-05	-0.16	8	0.52	9.6E-25	0.14	cDMR	
MMGT1	1.3E-02	-0.16	2	0.42	8.9E-31	0.21		
RAI1	1.1E-04	-0.16	7	0.46				
ATXN7L1	2.6E-05	-0.16	2	0.42			cDMR	
FLOT1	2.0E-03	-0.16	7	0.47			cDMR	
CUL1	1.9E-02	-0.16	6	0.40	1.0E-07	0.08		
TSPAN5	2.7E-02	-0.15	2	0.45				
DGKZ	7.2E-04	-0.15	2	0.43			cDMR	
SOSTDC1	1.9E-02	-0.14	6	0.44	3.0E-10	0.17		
PYCARD	2.1E-02	-0.14	9	0.41	1.3E-23	0.20	cDMR	Yes
PPT2	3.6E-03	-0.14	25	0.47	1.2E-28	0.30		
KCNH2	3.9E-02	-0.13	5	0.57				
RND2	2.4E-02	-0.13	2	0.56	4.7E-28	0.38	cDMR	
USP44	3.3E-03	-0.12	4	0.46	5.4E-09	0.07	cDMR	
SHROOM4	2.6E-03	-0.12	7	0.45			cDMR	
CORIN	2.7E-02	-0.12	5	0.40	2.0E-12	0.17	cDMR	
MAN1C1	8.3E-03	-0.12	4	0.47	4.7E-24	0.21		
PLEKHA6	2.2E-02	-0.11	9	0.42	5.7E-38	0.24		
MCF2	1.5E-02	-0.10	6	0.43	3.8E-20	0.22		
C1orf114	4.7E-02	-0.09	9	0.52	3.9E-24	0.25	cDMR	
MC5R	3.9E-02	0.11	3	-0.46				
KIAA0182	1.2E-02	0.14	10	-0.43				
LOC100130872	4.1E-03	0.35	5	-0.50				
ALDH1A3	6.0E-07	0.61	8	-0.45	1.2E-20	-0.20		
CLDN8	2.4E-07	0.81	4	-0.43	1.9E-18	-0.29		
SPON2	3.1E-04	0.93	5	-0.50				
GDF15	6.0E-07	1.06	2	-0.46			cDMR	

1. HM27 [18], M-NGS [16], Expression [29],Known methylated gene [10]
2. Difference between PCa and normal (Tumor - Normal). $\Delta\beta$ (methylation difference) indicated the biggest difference one among multiple DMCs.
3. Only located in the proximal promoter.
