# Supplementary material for: Identification of New Differentially Methylated Genes That Have Potential Functional Consequences in Prostate Cancer
Source: PLoS One. 2012 Oct 31;7(10):e48455. doi: 10.1371/journal.pone.0048455 (PMC3485209; doi:10.1371/journal.pone.0048455)
Supplement: Table S7 — Results of bisulfied sequencing in the SPON2 promoter region. (PDF) [file pone.0048455.s014.pdf]

Table S7. Results of bisulfied sequencing in the *SPON2* promoter region.

Sample	Phenotype	Pair	No. of tested clones	Race*	CpG_1	CpG_2	CpG_3	CpG_4	CpG_5	CpG_6	CpG_7	CpG_8	CpG_9	CpG_10	CpG_11	CpG_12	CpG_13	CpG_14	CpG_15	CpG_16	CpG_17	CpG_18	CpG_19	CpG_20	CpG_21	CpG_22	CpG_23	CpG_24	CpG_25	CpG_26	CpG_27
G10-001	Tumor	Yes	11	EA	1.00	0.55	0.73	0.55	0.91	0.82	1.00	0.82	0.91	0.64	0.82	0.46	0.64	0.36	0.64	0.55	0.50	0.46	0.55	0.36	0.46	0.36	0.46	0.46	0.46	0.27	0.18
G10-001	Normal	Yes	11	EA	0.73	0.73	0.82	0.91	0.90	0.91	1.00	1.00	1.00	0.91	0.82	0.91	0.73	0.55	0.91	1.00	0.91	0.91	1.00	1.00	1.00	1.00	1.00	1.00	0.91	0.91	0.91
G10-002	Tumor	Yes	12	.	0.50	0.42	0.42	0.50	0.42	0.50	0.67	0.50	0.58	0.58	0.58	0.58	0.50	0.50	0.58	0.58	0.58	0.58	0.58	0.58	0.67	0.58	0.58	0.58	0.67	0.58	0.42
G10-002	Normal	Yes	12	.	0.75	0.67	0.75	0.83	1.00	1.00	0.92	0.83	0.92	0.73	1.00	0.75	0.42	0.92	0.92	0.92	0.92	0.75	0.83	1.00	1.00	1.00	1.00	1.00	1.00	0.33	0.83
G6-002	Tumor	Yes	15	EA	0.93	0.80	0.73	0.80	0.67	0.87	0.73	0.80	0.80	0.73	0.80	0.73	0.67	0.73	0.80	1.00	0.60	0.73	0.80	0.80	0.87	0.47	1.00	0.50	0.53	0.40	0.33
G6-002	Normal	Yes	17	EA	0.53	0.47	0.41	0.41	0.53	0.75	0.65	0.77	0.47	0.47	0.59	0.71	0.71	0.77	0.77	0.88	0.47	0.65	0.88	0.71	0.53	0.71	0.59	0.65	0.82	0.59	0.29
G6-003	Tumor	Yes	11	EA	0.46	0.55	0.36	0.46	0.64	0.46	0.73	0.55	0.73	0.46	0.46	0.36	0.46	0.73	0.64	0.70	0.46	0.73	0.73	0.73	0.55	0.55	0.73	0.64	0.64	0.55	0.27
G6-003	Normal	Yes	12	EA	0.50	0.25	0.25	0.50	0.58	0.67	0.67	0.67	0.83	0.67	0.58	0.58	0.83	0.67	0.92	0.83	0.83	0.67	0.83	1.00	0.75	0.67	0.75	0.75	0.83	0.58	0.67
G6-013	Tumor	Yes	12	EA	0.58	0.25	0.08	0.58	0.25	0.58	0.58	0.58	0.58	0.58	0.58	0.58	0.50	0.55	0.50	0.64	0.42	0.58	0.58	0.50	0.42	0.25	0.50	0.33	0.42	0.58	0.42
G6-013	Normal	Yes	12	EA	0.33	0.33	0.17	0.67	0.75	0.75	0.75	0.75	0.67	0.33	0.58	0.67	0.58	0.83	0.92	0.92	0.92	0.75	0.92	0.92	0.75	0.75	0.83	0.92	0.75	0.75	0.67
G6-015	Tumor	Yes	10	EA	0.40	0.30	0.20	0.30	0.40	0.30	0.40	0.40	0.20	0.30	0.50	0.40	0.10	0.20	0.20	0.50	0.40	0.50	0.40	0.40	0.40	0.44	0.40	0.40	0.40	0.50	0.00
G6-015	Normal	Yes	10	EA	0.80	0.80	0.40	0.60	0.60	0.50	0.80	0.80	0.50	0.80	0.70	0.60	0.60	0.80	0.90	0.90	0.90	0.90	0.90	0.90	0.90	0.90	0.90	0.90	0.90	0.80	0.80
G6-016	Tumor	Yes	10	EA	0.80	0.80	0.60	0.70	0.70	0.70	0.70	0.70	0.70	0.90	0.80	0.40	0.60	0.40	0.50	0.67	0.70	0.70	0.70	0.80	0.30	0.89	0.80	0.50	0.70	0.50	0.70
G6-016	Normal	Yes	12	EA	0.50	0.42	0.83	0.42	0.67	0.92	1.00	1.00	0.92	0.58	0.58	0.08	0.50	0.58	0.67	0.75	0.67	0.58	0.75	0.83	0.25	0.58	1.00	0.83	0.67	0.42	0.00
G6-017	Tumor	Yes	12	AA	0.25	0.33	0.42	0.33	0.50	0.42	0.67	0.42	0.42	0.33	0.25	0.17	0.42	0.33	0.17	0.70	0.42	0.42	0.42	0.42	0.42	0.17	0.33	0.25	0.25	0.08	0.00
G6-017	Normal	Yes	10	AA	0.30	0.40	0.40	0.80	0.60	0.80	0.90	0.70	0.90	0.80	0.80	0.80	0.80	0.80	0.80	0.80	0.80	0.80	0.80	0.80	1.00	0.90	0.80	0.80	0.80	0.90	0.60
G6-018	Tumor	Yes	10	EA	0.50	0.50	0.30	0.40	0.50	0.50	0.60	0.60	0.40	0.60	0.40	0.60	0.50	0.50	0.67	0.60	0.40	0.50	0.20	0.50	0.50	0.10	0.60	0.50	0.20	0.20	0.00
G6-018	Normal	Yes	10	EA	1.00	0.70	0.50	0.80	1.00	1.00	1.00	1.00	1.00	1.00	1.00	1.00	0.40	0.40	0.90	1.00	0.70	0.40	0.80	0.50	0.80	0.80	1.00	0.80	0.90	0.60	0.70
G6-019	Tumor	Yes	11	EA	0.73	0.64	0.55	0.64	0.64	0.64	0.64	0.36	0.64	0.64	0.64	0.64	0.27	0.18	0.27	0.64	0.27	0.27	0.64	0.82	0.60	0.64	0.73	0.64	0.27	0.64	0.73
G6-019	Normal	Yes	12	EA	0.50	0.50	0.25	0.50	0.50	0.58	0.50	0.50	0.50	0.50	0.75	0.58	0.58	0.50	0.50	0.75	0.58	0.75	0.67	0.75	0.75	0.75	0.75	0.75	0.75	0.67	0.75
G6-020	Tumor	Yes	11	EA	0.64	0.64	0.55	0.46	0.55	0.73	0.64	0.73	0.73	0.73	0.46	0.73	1.00	0.73	0.73	0.78	0.64	0.64	0.55	0.73	0.64	0.64	0.73	0.73	0.64	0.64	0.55
G6-020	Normal	Yes	14	EA	0.43	0.29	0.29	0.50	0.57	0.57	0.93	0.50	0.64	0.57	0.64	0.21	0.36	0.93	0.93	1.00	0.92	0.50	0.86	1.00	0.86	0.86	1.00	0.79	0.71	0.86	0.64
G7-002	Tumor	Yes	13	EA	0.31	0.00	0.39	0.39	0.31	0.46	0.46	0.39	0.39	0.46	0.31	0.23	0.15	0.23	0.31	0.40	0.15	0.39	0.23	0.31	0.15	0.23	0.23	0.39	0.39	0.23	0.08
G7-002	Normal	Yes	12	EA	0.58	0.58	1.00	0.58	1.00	1.00	1.00	1.00	1.00	1.00	0.58	1.00	1.00	1.00	1.00	1.00	1.00	1.00	0.50	1.00	0.50	1.00	1.00	1.00	1.00	0.50	0.00
G7-013	Tumor	Yes	12	EA	0.67	0.58	0.33	0.33	0.50	0.42	0.67	0.67	0.67	0.67	0.67	0.33	0.42	0.25	0.58	0.67	0.42	0.50	0.58	0.50	0.58	0.42	0.82	0.58	0.42	0.33	0.25
G7-013	Normal	Yes	11	EA	0.64	0.64	0.64	0.64	0.73	0.73	0.73	0.82	0.73	0.73	0.82	0.73	0.55	0.73	0.73	0.73	0.64	0.55	0.73	0.64	0.64	0.64	0.64	0.64	0.64	0.36	0.36
G7-015	Tumor	Yes	11	EA	0.82	0.73	0.18	0.73	0.73	0.91	0.82	0.91	0.91	1.00	0.55	0.64	0.55	0.18	0.36	0.55	0.36	0.55	0.36	0.73	0.36	0.27	0.46	0.27	0.46	0.36	0.18
G7-015	Normal	Yes	15	EA	0.60	0.53	0.53	0.53	0.80	0.60	0.67	0.53	0.67	0.80	0.87	0.80	0.33	0.60	1.00	0.73	0.79	0.87	1.00	0.87	0.73	1.00	1.00	0.93	0.47	0.27	0.20
G7-016	Tumor	Yes	11	EA	0.27	0.55	0.27	0.27	0.27	0.36	0.55	0.46	0.55	0.55	0.46	0.46	0.18	0.46	0.55	0.60	0.55	0.46	0.46	0.55	0.55	0.46	0.46	0.46	0.18	0.36	0.46
G7-016	Normal	Yes	12	EA	0.75	0.50	0.33	0.33	0.83	0.92	0.75	0.83	0.75	0.92	0.83	0.83	0.83	0.83	0.83	0.91	0.83	0.83	0.83	0.83	0.83	0.83	0.92	0.83	0.83	0.83	0.83
G7-017	Tumor	Yes	12	EA	0.50	0.50	0.33	0.67	0.50	0.42	0.33	0.50	0.58	0.58	0.50	0.42	0.17	0.67	0.75	0.83	0.58	0.58	0.67	0.58	0.50	0.42	0.75	0.42	0.50	0.33	0.33
G7-017	Normal	Yes	12	EA	0.58	0.42	0.33	0.50	0.50	0.42	0.33	0.50	0.50	0.67	0.42	0.42	0.50	0.25	0.25	0.64	0.50	0.50	0.42	0.50	0.42	0.25	0.42	0.33	0.50	0.00	0.17
G7-018	Tumor	Yes	11	EA	1.00	0.91	0.46	0.18	0.82	0.82	1.00	0.82	0.73	0.64	1.00	0.36	0.64	0.27	0.64	0.60	0.55	0.64	0.55	0.64	0.82	0.55	0.82	0.64	0.46	0.36	0.27
G7-018	Normal	Yes	11	EA	0.64	0.55	0.46	0.64	0.46	0.46	0.64	0.55	0.64	0.64	0.64	0.46	0.55	0.55	0.91	0.64	0.91	0.91	0.82	0.91	0.64	0.82	0.91	0.82	0.91	0.64	0.82
G7-020	Tumor	Yes	15	EA	0.40	0.40	0.27	0.40	0.47	0.47	0.47	0.33	0.47	0.53	0.47	0.33	0.33	0.47	0.33	0.47	0.47	0.47	0.40	0.60	0.40	0.27	0.40	0.29	0.47	0.40	0.27
G7-020	Normal	Yes	11	EA	0.73	0.73	0.64	0.73	0.91	0.46	0.73	0.82	0.73	0.73	0.73	0.73	0.73	0.73	0.73	0.64	0.64	0.64	0.91	0.73	0.73	0.73	0.73	0.70	0.73	0.73	0.73
G7-021	Tumor	Yes	18	EA	0.39	0.33	0.39	0.56	0.44	0.28	0.61	0.61	0.50	0.50	0.28	0.33	0.44	0.44	0.44	0.65	0.44	0.44	0.39	0.33	0.56	0.56	0.67	0.44	0.50	0.44	0.39
G7-021	Normal	Yes	11	EA	0.64	0.36	0.55	0.46	0.55	0.46	0.55	0.55	0.55	0.55	0.73	0.55	0.55	0.91	0.91	0.82	0.82	0.82	0.91	0.91	0.82	0.82	0.91	0.82	0.82	0.55	0.55
G7-022	Tumor	Yes	11	EA	0.46	0.36	0.18	0.18	0.18	0.09	0.55	0.46	0.36	0.36	0.46	0.46	0.36	0.46	0.46	0.46	0.27	0.46	0.64	0.64	0.27	0.27	0.55	0.36	0.36	0.18	0.27
G7-022	Normal	Yes	10	EA	0.60	0.20	0.50	0.60	0.14	0.80	0.80	0.90	0.70	0.70	0.70	0.40	0.40	0.20	0.70	0.70	0.70	0.50	0.80	0.70	0.40	0.80	0.80	0.50	0.30	0.20	0.00
G7-023	Tumor	Yes	12	EA	0.67	0.67	0.42	0.33	0.50	0.75	0.67	0.58	0.67	0.67	0.67	0.50	0.50	0.42	0.75	0.50	0.42	0.33	0.42	0.58	0.50	0.50	0.67	0.33	0.58	0.42	0.25
G7-023	Normal	Yes	11	EA	0.64	0.91	0.46	0.73	0.73	0.73	0.73	0.73	0.91	0.91	0.73	0.91	0.91	0.91	0.91	0.82	0.73	0.91	0.73	0.73	0.73	0.73	0.91	0.91	0.91	0.91	0.73
G7-026	Tumor	Yes	11	EA	0.55	0.46	0.36	0.27	0.55	0.46	0.55	0.64	0.64	0.27	0.55	0.46	0.46	0.27	0.46	0.80	0.55	0.46	0.36	0.55	0.55	0.36	0.82	0.36	0.36	0.36	0.64
G7-026	Normal	Yes	18	EA	0.50	0.33	0.33	0.39	0.33	0.67	0.78	0.78	0.78	0.67	0.72	0.67	0.33	0.39	0.77	0.78	0.44	0.50	0.67	0.72	0.39	0.39	0.61	0.44	0.50	0.17	0.11
G7-028	Tumor	Yes	12	AA	0.83	0.92	0.50	0.67	0.75	0.83	0.75	0.83	0.75	0.83	0.67	0.75	0.58	0.58	0.92	0.73	0.73	0.58	0.58	0.58	0.67	0.75	0.75	0.75	0.67	0.50	0.17
G7-028	Normal	Yes	14	AA	0.64	0.50	0.43	0.36	0.29	0.50	0.50	0.50	0.50	0.57	0.43	0.36	0.43	0.50	0.50	0.64	0.36	0.50	0.50	0.50	0.50	0.43	0.77	0.50	0.36	0.36	0.43
G7-029	Normal	No	12	Other	0.58	0.67	0.58	0.92	0.50	0.92	0.67	0.83	0.67	0.67	0.67	0.75	1.00	0.83	0.92	1.00	0.67	0.83	0.92	0.92	0.92	0.92	0.92	0.83	0.67	0.92	0.92
G7-030	Tumor	Yes	12	EA	0.25	0.17	0.17	0.25	0.25	0.42	0.33	0.33	0.25	0.25	0.25	0.17	0.17	0.17	0.33	0.36	0.33	0.33	0.42	0.42	0.17	0.25	0.25	0.33	0.33	0.42	0.33
G7-030	Normal	Yes	15	EA	0.40	0.07	0.40	0.33	0.47	0.60	0.33	0.60	0.60	0.60	0.40	0.47	0.40	0.80	0.67	0.36	0.60	0.87	0.73	0.67	0.67	0.67	0.67	0.47	0.67	0.53	0.33
G7-032	Tumor	Yes	12	EA	0.58	0.58	0.42	0.33	0.50	0.58	0.67	0.67	0.75	0.58	0.67	0.50	0.83	0.33	0.50	0.75	0.67	0.42	0.75	0.58	0.58	0.73	0.90	0.67	0.67	0.33	0.33
G7-032	Normal	Yes	11	EA	0.64	0.64	0.64	0.82	0.73	0.64	0.73	0.82	0.60	0.46	0.73	0.91	0.82	0.64	0.55	1.00	0.36	0.46	0.73	0.73	0.46	0.91	0.91	0.64	0.64	0.18	0.09
G7-033	Tumor	Yes	11	EA	1.00	0.91	0.91	0.91	0.82	0.64	0.91	0.91	0.91	0.91	0.91	0.91	0.09	0.46	0.36	0.91	0.91	0.91	0.82	0.82	0.73	0.46	0.91	0.73	0.73	0.36	0.00
G7-033	Normal	Yes	15	EA	0.73	0.53	0.67	0.67	0.53	0.73	0.73	0.73	0.73	0.73	0.60	0.73	0.80	0.80	0.73	0.93	0.73	0.67	0.67	0.67	0.69	0.67	0.67	0.64	0.87	0.60	0.60
G7-048	Tumor	Yes	10	EA	0.70	0.70	0.56	0.60	0.90	0.80	0.90	0.80	0.80	0.80	0.70	0.70	0.60	0.60	0.90	0.90	0.90	0.80	0.80	0.80	1.00	0.40	0.90	0.70	0.90	0.40	0.00
G7-048	Normal	Yes	15	EA	0.33	0.33	0.33	0.27	0.40	0.40	0.53	0.40	0.47	0.53	0.53	0.67	0.47	0.47	0.67	0.93	0.47	0.47	0.60	0.67	0.46	0.53	0.67	0.60	0.53	0.20	0.07
G7-049	Tumor	Yes	12	EA	0.92	0.92	0.33	0.75	0.83	0.83	0.83	0.75	0.75	0.75	0.58	0.33	0.33	0.25	0.42	0.40	0.25	0.46	0.33	0.33	0.42	0.33	0.58	0.25	0.25	0.17	0.25
G7-049	Normal	Yes	16	EA	0.63	0.44	0.38	0.56	0.56	0.56	0.69	0.56	0.56	0.56	0.50	0.50	0.25	0.63	0.75	0.69	0.56	0.56	0.69	0.44	0.56	0.25	0.63	0.31	0.50	0.44	0.00
G7-050	Tumor	Yes	10	EA	0.40	0.10	0.20	0.20	0.20	0.20	0.50	0.20	0.20	0.20	0.20	0.20	0.20	0.00	0.00	0.20	0.20	0.20	0.20	0.20	0.20	0.20	0.30	0.20	0.20	0.00	0.00
G7-050	Normal	Yes	12	EA	0.33	0.33	0.25	0.25	0.83	0.83	0.83	0.83	0.92	0.83	0.83	0.42	0.42	0.08	0.42	0.58	0.83	0.91	0.92	0.50	0.46	0.27	0.58	0.25	0.42	0.17	0.17
G7-052	Tumor	Yes	12	AA	0.50	0.42	0.50	0.42	0.58	0.75	0.75	0.58	0.67	0.50	0.42	0.42	0.42	0.25	0.50	0.50	0.50	0.25	0.50	0.33	0.25	0.33	0.58	0.33	0.50	0.25	0.33
G7-052	Normal	Yes	12	AA	0.67	0.58	0.42	0.58	0.67	0.67	0.92	0.67	0.67	0.58	0.75	0.67	0.83	0.67	0.67	0.67	0.67	0.58	0.67	0.67	0.67	0.67	0.67	0.67	0.67	0.42	0.25
G7-054	Tumor	Yes	12	EA	0.67	0.67	0.50	0.67	0.83	0.75	0.67	0.67	0.67	0.50	0.75	0.50	0.75	0.42	0.75	0.67	0.75	0.75	0.83	0.73	0.50	0.58	0.83	0.42	0.58	0.50	0.33
G7-054	Normal	Yes	11	EA	1.00	0.82	0.73	0.82	0.64	0.91	0.82	0.82	0.82	0.64	0.82	0.73	0.55	0.27	0.73	0.27	0.27	0.55	0.55	0.82	0.18	0.27	0.55	0.18	0.46	0.18	0.09
G7-055	Tumor	Yes	12	EA	0.83	0.67	0.42	0.50	0.75	0.75	0.92	0.83	0.75	0.75	0.67	0.75	0.25	0.25	0.42	0.50	0.33	0.58	0.50	0.58	0.25	0.33	0.50	0.17	0.33	0.33	0.08
G7-055	Normal	Yes	12	EA	0.58	0.58	0.67	0.58	0.75	0.67	0.75	0.75	0.67	0.75	0.67	0.50	0.67	0.67	0.67	0.92	0.92	0.75	0.92	0.83	0.92	0.83	0.92	0.83	0.92	0.75	0.58
G7-056	Tumor	Yes	11	.	0.36	0.36	0.36	0.36	0.36	0.64	0.46	0.64	0.64	0.36	0.46	0.82	0.00	0.00	0.36	0.60	0.46	0.46	0.46	0.46	0.36	0.46	0.55	0.46	0.46	0.46	0.46
G7-056	Normal	Yes	12	.	1.00	1.00	0.17	0.92	0.33	1.00	1.00	0.92	1.00	1.00	1.00	0.42	0.08	0.25	0.92	0.92	0.42	1.00	1.00	0.83	1.00	1.00	1.00	1.00	0.75	0.33	0.92
G7-057	Tumor	Yes	12	EA	0.42	0.42	0.42	0.42	0.42	0.42	0.50	0.42	0.42	0.42	0.42	0.42	0.42	0.42	0.42	0.58	0.25	0.50	0.42	0.58	0.58	0.50	0.58	0.42	0.50	0.25	0.50
G7-057	Normal	Yes	12	EA	0.75	0.75	0.67	0.67	1.00	0.92	1.00	0.75	0.92	0.67	0.92	0.92	0.67	0.75	0.92	1.00	0.92	0.83	1.00	1.00	0.92	1.00	1.00	1.00	1.00	0.92	0.58
G7-058	Tumor	Yes	11	.	0.18	0.00	0.09	0.09	0.00	0.09	0.09	0.09	0.09	0.09	0.09	0.09	0.00	0.18	0.09	0.13	0.09	0.09	0.27	0.09	0.09	0.09	0.18	0.09	0.09	0.18	0.18
G7-058	Normal	Yes	12	.	0.33	0.33	0.08	0.33	0.33	0.33	0.58	0.50	0.92	0.67	0.50	0.83	0.58	0.75	1.00	1.00	0.92	0.83	1.00	1.00	1.00	0.92	1.00	1.00	1.00	0.75	1.00
G8-002	Tumor	Yes	10	EA	0.70	0.70	0.30	0.60	0.60	0.70	0.90	0.90	0.80	0.70	0.70	0.70	0.70	0.60	0.50	0.70	0.30	0.60	0.40	0.40	0.40	0.50	0.44	0.63	0.40	0.20	0.20
G8-002	Normal	Yes	12	EA	0.50	0.42	0.17	0.42	0.50	0.50	0.58	0.58	0.33	0.25	0.33	0.42	0.67	0.58	0.67	0.80	0.58	0.33	0.50	0.75	0.50	0.50	0.83	0.67	0.58	0.50	0.25
G8-003	Tumor	Yes	11	EA	0.73	0.55	0.46	0.09	0.73	0.73	0.46	0.73	0.82	0.73	0.64	0.55	0.82	0.73	0.82	0.80	0.82	0.73	0.82	0.82	0.73	0.82	0.91	0.82	0.82	0.82	0.73
G8-003	Normal	Yes	10	EA	0.60	0.50	0.50	0.40	0.70	0.80	0.80	0.50	0.50	0.50	0.40	0.50	0.50	0.80	1.00	1.00	1.00	0.80	0.80	0.70	0.80	0.70	0.90	0.50	0.70	0.40	0.40
G8-004	Tumor	Yes	12	EA	0.17	0.00	0.00	0.08	0.08	0.08	0.17	0.17	0.25	0.08	0.25	0.17	0.17	0.17	0.25	0.08	0.08	0.08	0.17	0.17	0.08	0.17	0.17	0.17	0.17	0.25	0.17
G8-004	Normal	Yes	11	EA	0.82	0.73	0.82	0.82	0.73	0.73	0.82	0.91	0.82	0.82	0.82	0.64	0.91	0.91	0.91	0.91	1.00	0.91	0.91	0.90	0.91	0.91	0.91	0.91	0.91	0.55	0.91
G8-012	Tumor	Yes	12	EA	0.25	0.42	0.17	0.42	0.17	0.33	0.50	0.33	0.33	0.42	0.50	0.25	0.42	0.67	0.50	0.58	0.67	0.67	0.67	0.67	0.50	0.33	0.83	0.42	0.25	0.08	0.08
G8-012	Normal	Yes	11	EA	0.55	0.46	0.36	0.46	0.46	0.55	0.64	0.82	0.73	0.64	0.55	0.64	0.64	0.55	0.73	0.73	0.73	0.73	0.82	0.64	0.55	0.64	0.80	0.64	0.64	0.64	0.46
G8-013	Tumor	Yes	11	EA	0.73	0.64	0.55	0.64	0.91	0.73	0.91	0.82	0.55	0.73	0.55	0.64	0.55	0.46	0.64	0.91	0.46	0.36	0.64	0.91	0.36	0.78	0.73	0.46	0.55	0.27	0.09
G8-013	Normal	Yes	11	EA	0.27	0.36	0.36	0.46	0.73	0.73	0.73	1.00	0.91	0.64	0.82	0.64	0.91	0.82	0.91	1.00	1.00	0.91	1.00	0.73	1.00	1.00	1.00	0.73	1.00	0.73	0.91
G9-001	Tumor	Yes	11	EA	0.27	0.27	0.18	0.09	0.46	0.46	0.55	0.55	0.55	0.09	0.46	0.18	0.09	0.46	0.36	0.36	0.36	0.36	0.46	0.50	0.46	0.36	0.36	0.36	0.36	0.36	0.36
G9-001	Normal	Yes	12	EA	0.67	0.58	0.25	0.58	0.50	0.50	0.58	0.75	0.58	0.58	0.58	0.58	0.50	0.67	0.50	0.67	0.50	0.55	0.64	0.58	0.58	0.50	0.64	0.50	0.58	0.58	0.42
G9-002	Tumor	Yes	12	EA	0.58	0.50	0.50	0.42	0.58	0.58	0.50	0.50	0.42	0.42	0.25	0.75	0.67	0.42	0.50	0.50	0.58	0.42	0.58	0.58	0.64	0.42	0.58	0.50	0.50	0.58	0.42
G9-002	Normal	Yes	11	EA	0.64	0.73	0.46	0.73	0.73	0.82	0.82	0.82	0.82	0.91	0.82	0.73	0.55	0.82	1.00	1.00	0.82	0.91	1.00	1.00	1.00	1.00	0.91	1.00	0.91	0.64	0.82
G9-003	Tumor	Yes	11	EA	0.46	0.46	0.27	0.64	0.82	0.82	0.91	0.82	0.82	1.00	1.00	0.55	0.18	0.64	0.64	0.82	0.73	0.73	0.73	0.82	0.60	0.73	0.73	0.55	0.64	0.55	0.46
G9-003	Normal	Yes	12	EA	0.75	0.75	0.50	0.67	0.83	0.83	0.83	0.92	0.92	0.92	0.75	0.75	0.50	0.58	0.75	0.92	0.42	0.50	0.92	0.83	0.58	0.50	0.92	0.73	0.50	0.33	0.17
G9-004	Tumor	Yes	15	EA	0.33	0.33	0.20	0.40	0.73	0.73	0.73	0.73	0.73	0.73	0.73	0.73	0.67	0.40	0.47	0.73	0.67	0.60	0.79	0.67	0.53	0.60	0.60	0.53	0.60	0.67	0.60
G9-004	Normal	Yes	10	EA	0.80	0.60	0.80	0.60	0.60	0.70	0.50	0.70	0.80	0.60	0.40	0.70	0.50	0.60	0.50	0.90	0.60	0.90	0.90	0.70	0.60	0.60	0.70	0.60	0.50	0.50	0.50
G9-005	Tumor	Yes	12	AA	0.18	0.42	0.25	0.42	0.42	0.25	0.46	0.42	0.33	0.25	0.42	0.42	0.25	0.42	0.42	0.42	0.50	0.42	0.42	0.42	0.42	0.42	0.42	0.25	0.42	0.42	0.42
G9-005	Normal	Yes	11	AA	0.91	0.73	0.27	0.46	0.64	0.91	0.91	0.82	0.91	0.64	0.55	0.64	0.46	0.46	0.36	0.60	0.46	0.67	0.55	0.73	0.36	0.55	0.64	0.46	0.64	0.73	0.18
G9-007	Tumor	Yes	12	EA	0.50	0.42	0.08	0.42	0.50	0.58	0.67	0.33	0.42	0.42	0.50	0.42	0.58	0.50	0.75	0.75	0.67	0.83	0.92	0.83	0.83	0.50	0.83	0.75	0.75	0.17	0.33
G9-007	Normal	Yes	10	EA	0.80	0.80	0.89	0.70	0.80	0.90	0.80	0.70	0.90	0.90	0.80	0.90	0.60	0.60	1.00	0.90	0.60	0.60	1.00	1.00	0.40	0.80	0.90	0.70	0.80	0.70	0.50
G9-008	Tumor	Yes	12	EA	0.42	0.33	0.33	0.42	0.50	0.42	0.33	0.33	0.58	0.42	0.42	0.42	0.50	0.50	0.50	0.58	0.58	0.55	0.58	0.67	0.50	0.64	0.58	0.58	0.67	0.58	0.58
G9-008	Normal	Yes	11	EA	0.64	0.55	0.46	0.55	0.36	0.64	0.64	0.64	0.36	0.36	0.82	0.55	0.46	0.46	0.46	0.50	0.46	0.27	0.46	0.46	0.46	0.27	0.46	0.46	0.27	0.64	0.09
G9-009	Tumor	Yes	15	EA	0.73	0.60	0.40	0.47	0.40	0.40	0.73	0.67	0.60	0.67	0.80	0.73	0.13	0.53	0.60	0.62	0.40	0.33	0.40	0.40	0.40	0.40	0.53	0.40	0.33	0.33	0.27
G9-009	Normal	Yes	11	EA	0.27	0.46	0.46	0.64	0.64	0.55	0.78	0.73	0.82	0.73	0.46	0.64	0.36	0.46	0.82	0.60	0.55	0.73	0.73	0.91	0.64	0.73	0.91	0.55	0.55	0.36	0.27
G9-010	Tumor	Yes	10	EA	0.30	0.30	0.40	0.40	0.40	0.50	0.40	0.50	0.30	0.30	0.40	0.40	0.30	0.30	0.50	0.56	0.40	0.40	0.40	0.50	0.60	0.50	0.40	0.50	0.60	0.20	0.00
G9-010	Normal	Yes	12	EA	0.67	0.75	0.58	0.58	0.50	0.67	0.75	0.50	0.67	0.67	0.75	0.75	0.83	0.75	0.83	1.00	1.00	0.92	1.00	1.00	1.00	1.00	1.00	1.00	0.92	0.67	0.75
G9-011	Tumor	Yes	12	EA	0.25	0.33	0.25	0.33	0.58	0.67	0.83	0.42	0.50	0.50	0.67	0.46	0.58	0.92	0.83	0.92	0.58	0.67	0.83	0.83	0.75	0.50	0.92	0.67	0.67	0.58	0.50
G9-011	Normal	Yes	15	EA	0.73	0.27	0.40	0.47	0.73	0.67	0.67	0.73	0.67	0.60	0.60	0.60	0.73	0.80	0.80	1.00	0.87	0.67	1.00	0.80	0.87	0.87	1.00	0.93	1.00	0.87	0.60
G9-012	Tumor	Yes	17	EA	0.53	0.35	0.35	0.41	0.47	0.53	0.59	0.47	0.29	0.35	0.35	0.29	0.41	0.29	0.41	0.56	0.24	0.47	0.47	0.59	0.29	0.29	0.47	0.29	0.47	0.29	0.24
G9-012	Normal	Yes	10	EA	0.30	0.30	0.33	0.40	0.40	0.40	0.40	0.30	0.20	0.40	0.50	0.50	0.40	0.60	0.80	0.78	0.50	0.70	0.70	0.50	0.78	0.70	0.80	0.60	0.60	0.50	0.50

G9-013	Tumor	Yes	12	EA	0.83	0.83	0.50	0.75	0.75	0.83	0.83	0.75	0.75	0.75	0.92	0.75	0.80	0.92	0.92	0.92	0.92	0.92	1.00	0.75	0.83	0.92	0.92	0.83	0.92	0.58	0.50
G9-013	Normal	Yes	10	EA	0.30	0.00	0.30	0.30	0.20	0.20	0.40	0.33	0.40	0.30	0.30	0.40	0.40	0.50	0.50	0.56	0.10	0.50	0.50	0.40	0.50	0.40	0.50	0.50	0.50	0.60	0.50
G9-015	Tumor	Yes	10	EA	0.40	0.60	0.00	0.20	0.40	0.40	0.70	0.70	0.50	0.40	0.60	0.40	0.20	0.00	0.30	0.20	0.10	0.22	0.20	0.10	0.10	0.10	0.22	0.10	0.00	0.00	0.00
G9-015	Normal	Yes	11	EA	0.27	0.09	0.18	0.36	0.46	0.64	0.46	0.55	0.55	0.36	0.64	0.46	0.55	0.55	0.55	0.91	0.80	0.73	0.91	0.91	0.64	0.64	0.82	0.55	0.73	0.55	0.55
GN-001	Tumor	Yes	12	EA	0.58	0.58	0.42	0.50	0.50	0.58	0.58	0.58	0.58	0.58	0.58	0.58	0.50	0.50	0.58	0.58	0.58	0.58	0.58	0.64	0.50	0.50	0.58	0.58	0.58	0.58	0.50
GN-001	Normal	Yes	11	EA	0.09	0.00	0.00	0.64	0.64	0.64	1.00	0.73	0.09	0.64	0.73	0.64	1.00	1.00	1.00	0.73	1.00	1.00	1.00	1.00	1.00	1.00	0.73	0.36	0.36	0.00	1.00

*EA: European American, AA: African American.
