# Supplementary material for: Identification of New Differentially Methylated Genes That Have Potential Functional Consequences in Prostate Cancer
Source: PLoS One. 2012 Oct 31;7(10):e48455. doi: 10.1371/journal.pone.0048455 (PMC3485209; doi:10.1371/journal.pone.0048455)
Supplement: Table S8 — List of Gleason score-associated DMCs in PCa. (PDF) [file pone.0048455.s015.pdf]

Table S8. List of Gleason score-associated DMCs in PCa.

Taeget_ID	chr	locus	FDR *P*	Δβ*	Gene Symbol	Genomic region
cg14117320	11	17,032,483	9.5E-30	0.38	*PLEKHA7*	Body
cg09491897	17	78,935,269	1.1E-18	-0.31	*RPTOR*	Body
cg07964113	17	78,935,289	1.4E-08	-0.24	*RPTOR*	Body
cg07217350	5	162,934,022	2.7E-07	0.21	*MAT2B*	Body
cg13690280	9	112,562,314	9.4E-05	0.22	*PALM2-AKAP2*	Body
cg05315334	13	113,656,212	3.0E-04	-0.27	*MCF2L*	Body
cg22329423	20	21,490,572	3.0E-04	0.25		
cg06233593	16	337,645	5.7E-04	-0.22	*AXIN1*	3'UTR
cg03362815	16	50,407,774	8.6E-04	0.22		
cg06868132	5	178,987,429	1.9E-03	-0.21	*RUFY1*	Body
cg14794741	1	7,408,911	1.9E-03	0.20	*CAMTA1*	Body
cg10772532	14	81,405,846	2.1E-03	-0.36	*C14orf145*	1stExon
cg01135780	5	158,527,707	2.4E-03	-0.26	*EBF1*	TSS1500
cg03177600	3	164,911,744	3.2E-03	0.21	*SLITRK3*	5'UTR
cg20497212	7	36,672,687	5.2E-03	0.24	*AOAH*	Body
cg13619408	1	109,756,118	5.3E-03	-0.21	*SARS*	TSS1500
cg20980653	6	33,143,760	6.2E-03	0.21	*COL11A2*	Body
cg03871124	7	1,643,681	6.5E-03	0.23		
cg22264898	18	3,297,176	6.7E-03	-0.33		
cg04929022	X	135,617,792	6.9E-03	0.20	*VGLL1*	5'UTR
cg07639198	10	5,004,250	7.2E-03	0.23	*AKR1C1*	TSS1500
cg00292312	1	7,318,138	1.0E-02	0.21	*CAMTA1*	Body
cg05922453	16	50,425,414	1.0E-02	0.22		
cg21094405	13	113,656,117	1.6E-02	-0.25	*MCF2L*	Body
cg07259110	17	62,098,222	1.8E-02	0.20	*ICAM2*	TSS1500
cg12691631	4	23,891,835	1.9E-02	0.25	*PPARGC1A*	TSS200
cg10649525	7	87,974,756	2.7E-02	0.20		
cg05534186	5	54,530,279	2.8E-02	-0.20	*CCNO*	TSS1500
cg22541755	2	10,183,094	2.9E-02	-0.22	*KLF11*	TSS1500
cg26920627	1	7,319,248	2.9E-02	0.20	*CAMTA1*	Body
cg14964336	4	1,523,275	3.0E-02	-0.25		
cg24729983	5	54,530,114	3.0E-02	-0.21	*CCNO*	TSS1500
cg06346388	4	13,539,513	4.1E-02	0.22		
cg27027827	8	55,383,182	4.1E-02	0.26		
cg11668844	13	113,655,622	4.2E-02	-0.23	*MCF2L*	Body
cg19975916	12	123,215,308	4.2E-02	-0.22	*GPR81*	TSS200
cg03673737	7	87,974,733	4.3E-02	0.22		
cg04300684	4	1,523,326	4.6E-02	-0.23		
cg03075487	10	125,257,350	4.7E-02	0.21		
cg22366088	4	154,713,789	4.7E-02	0.23		

*Methylation difference between higher Gleason and lower Gleason PCa (higher - lower).
